# Impact of Shades and Thickness on the Polymerization of Low-Viscosity Bulk-Fill Composites in Pediatric Restorations: An In Vitro Study

**DOI:** 10.3390/dj13080352

**Published:** 2025-08-01

**Authors:** Gennaro Musella, Stefania Cantore, Maria Eleonora Bizzoca, Mario Dioguardi, Rossella Intini, Lorenzo Lo Muzio, Federico Moramarco, Francesco Pettini, Andrea Ballini

**Affiliations:** 1 Department of Clinical and Experimental Medicine, University of Foggia, 71122 Foggia, Italy; gennaro.musella@unifg.it (G.M.); mario.dioguardi@unifg.it (M.D.); lorenzo.lomuzio@unifg.it (L.L.M.); 2 Department of Mechanics, Mathematics and Management, Polytechnic University of Bari, 70125 Bari, Italy; s.cantore3@phd.poliba.it; 3 Department of Precision Medicine in Medical, Surgical, and Critical Care Areas, University of Palermo, 90127 Palermo, Italy; 4 Department of Dentistry, Faculty of Dentistry, Universitat Internacional de Catalunya, 08017 Barcelona, Spain; rintini@uic.es; 5 Department of Interdisciplinary Medicine, University of Bari, 70124 Bari, Italy; fede.moramarco@hotmail.it (F.M.); francesco.pettini@uniba.it (F.P.); 6 Department of Life Science, Health and Health Professions, Link Campus University, 00165 Rome, Italy; a.ballini@unilink.it

**Keywords:** bulk-fill composite, degree of conversion, polymerization, pediatric dentistry, shade and thickness

## Abstract

**Background/Objectives:** This study aimed to investigate the influence of shade and thickness on the polymerization of SDR^®^ flow+, a low-viscosity bulk-fill composite, by assessing its degree of conversion (DC). **Methods:** An in vitro study was conducted using SDR^®^ flow+ composite resin. Specimens were prepared at two thicknesses (2 mm and 4 mm) and four shades (Universal, A1, A2, A3). Polymerization was performed using a high-intensity LED curing unit. The DC was assessed using Fourier-transform infrared spectroscopy (ATR-FTIR). **Results:** Both shade and thickness significantly influenced DC. Thicker specimens (4 mm) exhibited reduced polymerization compared to thinner specimens (2 mm). Darker shades, particularly A3, demonstrated the lowest DC values due to their higher chroma, which limits light penetration. In contrast, the Universal shade achieved higher DC values, even at increased depths, likely due to its greater translucency. **Conclusions**: Shade and thickness play a critical role in the polymerization of bulk-fill composites. Ensuring adequate polymerization is essential for the longevity of pediatric restorations. Further in vivo research is needed to confirm these findings and assess their clinical implications.

## 1. Introduction

The use of composite resins in pediatric dentistry has significantly increased over the past decades due to their ability to provide both aesthetic and functional restoration [1]. Pediatric patients present unique challenges during dental procedures, including limited compliance, smaller oral cavities, and a heightened need for efficient and effective treatments to minimize chair time [2]. Consequently, selecting restorative materials that simplify restorative techniques while maintaining high clinical standards is critical. In pediatric patients, the primary restorative needs involve the management of carious lesions in young permanent teeth, where rubber dam isolation is feasible, including anterior teeth. In primary teeth, Glass Ionomer Cements (GICs), High-Viscosity GIC (HVGIC), and Resin-Modified Glass Ionomer Cements (RMGICs) are recommended for occlusal (Class I) restorations [3]. For more extensive cavities, including Class II, alternative restorative strategies should be selected based on the clinical situation and patient compliance.

Risk factors for caries in pediatric patients include frequent consumption of sugary foods and beverages, insufficient fluoride exposure, poor oral hygiene practices, and socio-economic barriers to accessing regular dental care [4]. Additionally, children with high caries risk may develop extensive lesions even in deciduous teeth, further complicating restorative treatment [5]. Although conservative approaches to deep carious lesions are increasingly adopted today [6,7], the use of restorative materials that enable efficient and long-lasting restorations is essential to preserve tooth structure and functionality [8]. Beyond the technical challenges, the psychological component plays a critical role in pediatric dental care. Children may associate dental treatments with discomfort or fear, which can significantly influence their behavior during procedures. Long and complex treatments often increase anxiety, making it harder for clinicians to manage children effectively [8]. This reinforces the need for restorative materials that enable efficient and less invasive procedures, reducing chair times and maintaining high clinical outcomes. Bulk-fill resin composites are particularly suitable for this purpose, allowing for quicker restorations with fewer steps, which can positively impact the overall treatment experience for young patients. Their main advantages in pediatric dentistry include reduced chair time, simplified application techniques (e.g., single-increment filling), lower polymerization shrinkage stress, and enhanced depth of cure, which is critical in cavities with limited access [9,10]. These properties are particularly beneficial when managing children with limited compliance and tolerance for lengthy procedures. Furthermore, the anatomical particularities of primary molars, such as thinner enamel and dentin layers, relatively large pulp chambers, and prominent cervical bulges, require restorative materials that can adapt well to cavity morphology while minimizing the risk of pulp exposure and post-operative sensitivity [11,12].

The flowable nature and optimized translucency of modern bulk-fill composites facilitate adaptation to these complex anatomical features, supporting a more predictable and effective restorative outcome.

Nevertheless, the degree of conversion (DC) remains a critical factor in both anterior and posterior restorations, as it not only affects the mechanical properties and longevity of restorations but also influences the final appearance of the material. Insufficient polymerization can lead to color instability, surface roughness, and reduced translucency, potentially compromising the restoration’s aesthetic integration with natural dentition [13]. Generally, several factors influence DC, including material thickness, shade, and light-curing protocols. Increased thickness reduces the penetration of curing light, leading to incomplete polymerization in deeper layers, while darker or more opaque shades further limit light transmission, resulting in lower DC values [14]. These variables are particularly significant in pediatric dentistry, where carious lesions are often extensive, and restorations must primarily ensure durability and functional integrity while considering biological compatibility [1]. For anterior restorations, selecting shades that match the natural tooth color while maintaining adequate DC is essential to achieve a seamless and natural appearance [15]. Manufacturers have developed bulk-fill composites with enhanced optical properties and improved curing capabilities to address these challenges [9]. While these restorative materials show promising results in general dentistry, their specific application in pediatric patients requires further investigation [16]. Pediatric cavities often involve unique anatomical and clinical conditions, such as high-C-factor configurations in posterior teeth and aesthetic demands in anterior regions [1]. Furthermore, the translucency and color stability of restorative materials over time are critical factors for ensuring long-term success in restorations for young patients who are still growing and developing [17]. This study aims to evaluate the impact of thickness and shade on the DC of a low-viscosity bulk-fill composite.

By addressing these factors, the study seeks to determine the suitability of these materials for pediatric restorations, emphasizing their potential to enhance clinical outcomes while simplifying procedures and improving patient comfort. The null hypothesis of this study was that neither the thickness nor the shade of the bulk-fill composite would significantly influence its degree of conversion.

## 2. Materials and Methods

**Figure 1:**
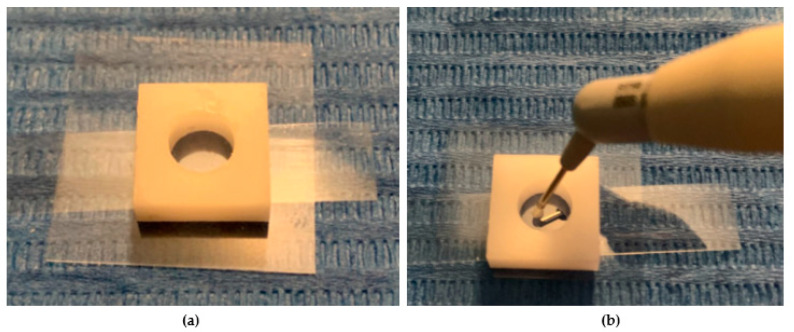
(**a**,**b**) The image shows the preparation of specimens for measuring the degree of conversion. A total of four samples were prepared for each tested composite, with measurements taken at two different depths.

**Figure 2:**
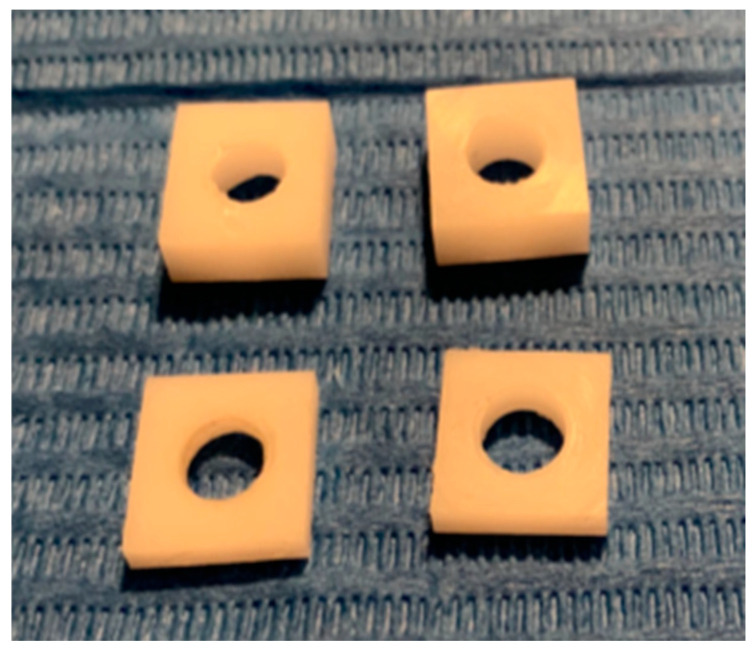
The image shows a cylindrical polyvinyl mold placed on a microscope slide, with a transparent Mylar strip positioned between the glass and the composite material to prevent direct contact. The composite resin is inserted into the mold using a single-increment technique, ensuring uniform filling.

In this experimental study, four shades of a low-viscosity self-adapting bulk-fill material, SDR^®^flow+ (Dentsply-Caulk, Milford, DE, USA), were tested: Universal (U), A1, A2, and A3 (Table 1). The study followed the CRIS Guidelines (Checklist for Reporting In-Vitro Studies). To minimize variability and operator-dependent bias, all specimen preparations were performed by the same operator (F.M.). Specimens were prepared using cylindrical polyvinyl molds with a diameter of 6 mm and different heights of 2 mm and 4 mm (Figure 1a,b and Figure 2). The upper surface was covered with a Mylar strip and lightly pressed with a glass slide to remove excess material and obtain a smooth, flat surface. The Mylar strip prevented the formation of an oxygen-inhibited layer.

### 2.1. Polymerization Process

**Figure 3:**
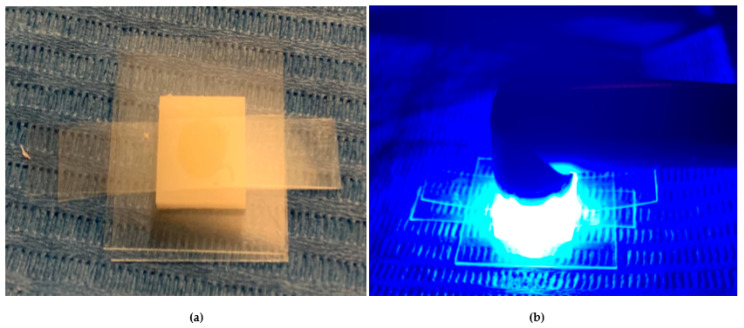
(**a**). The image depicts a cylindrical mold positioned on a microscope slide. A transparent Mylar strip is placed between the glass and the composite material to prevent direct contact. The mold is filled with the composite resin, and the upper surface is covered by the Mylar strip. (**b**). After removing the glass slide, the polymerization process is carried out, with the curing tip placed directly in contact with the Mylar strip on the upper surface of the specimen. The setup ensures uniform curing while minimizing oxygen inhibition.

**Figure 4:**
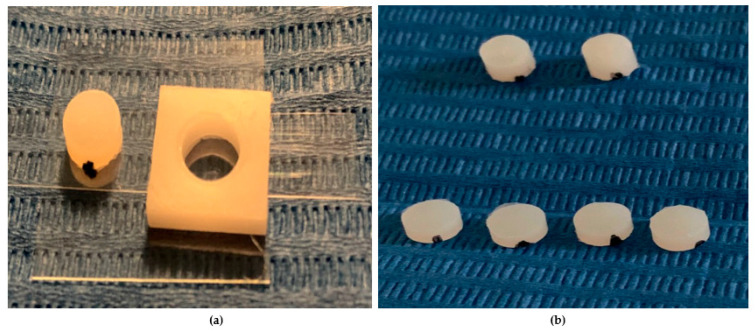
(**a**,**b**) The image shows a cylindrical composite specimen with a smooth, polymerized upper surface. On the lower surface, a small mark made with a permanent marker is visible, used to distinguish the bottom side from the top.

After removing the glass slide, polymerization was performed with the curing tip in contact with the Mylar strip covering the upper surface of each specimen (Figure 3a,b). Once polymerization was obtained, the lower surface was marked with a notch made with a permanent marker (Figure 4a,b). A latest-generation LED light unit (SmartLite Pro^®^; Dentsply Sirona Inc., New York, NY, USA) was used, which is compatible with the absorption spectrum of SDR^®^flow+. The unit had an output of 1200 mW/cm^2^ and a 10 mm diameter active tip, used according to the manufacturer’s recommendations (Table 2). Every four polymerization cycles, the light intensity was checked with a radiometer (C10 Light Meter, Premium Plus, Conservation Support Systems, Santa Barbara, CA, USA), consistently measuring approximately 1200 mW/cm^2^. All cylindrical specimens were stored dry and away from light for 24 h.

### 2.2. Grouping and Study Variables

After the storage period, samples (n = 36) were divided into groups (n = 9) based on their thickness and shade. The DC, a key chemical property of resinous materials, was assessed. This characteristic is defined by the amount of C=C terminal double bonds that transform into C-C single bonds when monomers join during polymerization. Despite proper polymerization protocols, 100% conversion is not achieved due to monomer properties and intrinsic polymerization limitations, which trap reactive molecules in the solidified matrix during the final phases.

### 2.3. Fourier-Transform Infrared Spectroscopy Analysis

**Figure 5:**
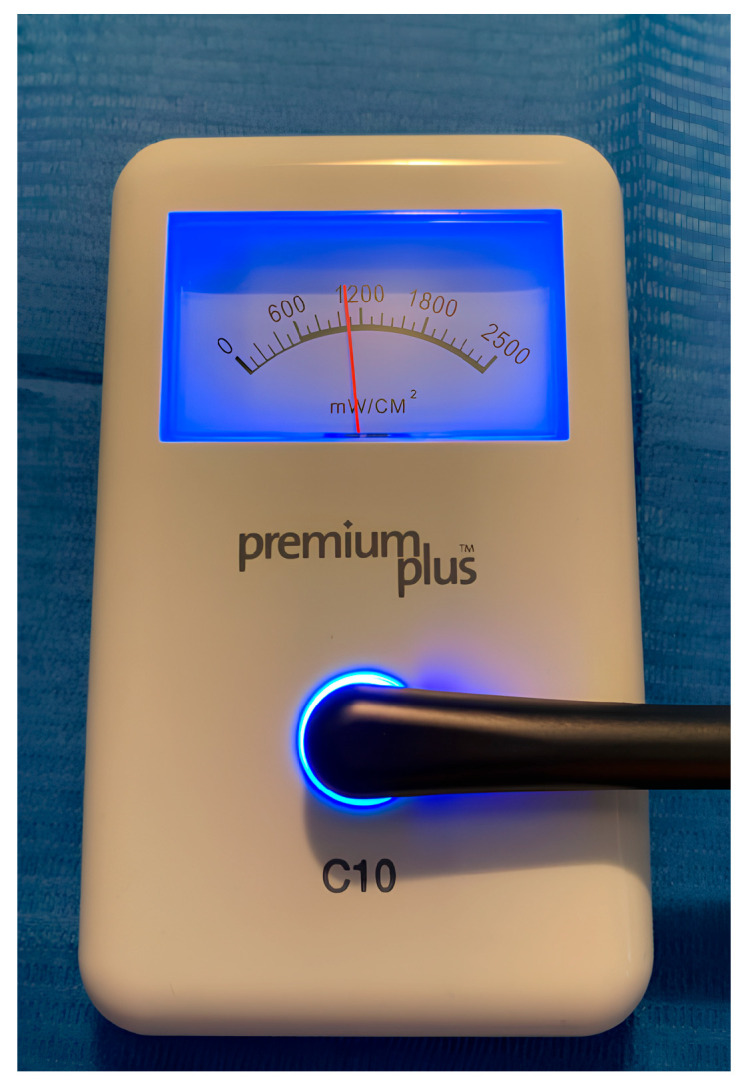
The measurement of each sample is carried out with a spectroscope infrared Spectrum Two (PerkinElmer, Waltham, MA, USA) which exploits the medium-infrared range (~4000–400 cm^−1^) and attenuated total reflection (ATR) technology.

Infrared spectroscopy is the most commonly used method to measure the DC, with different techniques available. This study used Attenuated Total Reflectance Fourier-Transform Infrared (ATR-FTIR) spectroscopy, with spectra acquired using a Spectrum Two Spectrophotometer (Perkin Elmer, Waltham, MA, USA) (Figure 5). The spectra were recorded in the range of 4000–400 cm^−1^, with a resolution of 4 cm^−1^, using a 0.25 cm^−1^ acquisition interval, and acquiring 16 scans per sample. FTIR spectra are characterized by absorption peaks for each functional group (C=O, C=C, C=N, O-H, etc.), though peak positioning is influenced by the molecular structure they belong to. ATR-FTIR specifically analyzes the most superficial layer of the tested material, making it suitable for this study. The spectroscopic investigation was carried out on the upper and lower surfaces of each sample. The spectrometry data are provided as Supplementary Materials (File S1: ATR-FTIR Raw Data Supporting DC Evaluation.).

### 2.4. Calculation of the DC

To determine the DC of dental composites, the intensity of the spectra from un-cured and cured samples was compared. Specifically, the absorption peaks of the aliphatic C=C functional groups (1630–1640 cm^−1^, corresponding to the C=C term in the formula) were analyzed. The quantitative difference in C=C aliphatic groups, which convert into C-C aliphatic groups during polymerization, was evaluated. To minimize measurement errors, an internal reference peak was introduced using the C-C aromatic functional group (1583–1610 cm^−1^, corresponding to the C-C term in the formula), which remains stable during the curing process.

$$DC\;\%=\left(\;1-\frac{\frac{C=C}{\;C-C}\;CURED}{\frac{C=C}{\;C-C}\;UNCURED}\right)\times 100$$

The DC was measured on both the top and bottom surfaces of each sample to assess differences in polymerization efficiency at different depths.

### 2.5. Statistical Analysis

The statistical analysis was performed on normally distributed data. To evaluate the effects of color and depth on the DC, the Welch *t*-test, One-Way ANOVA, and Tukey HSD test were used. A significance level of 95% (*p* = 0.05) was adopted for all statistical tests.

## 3. Results

**Figure 6:**
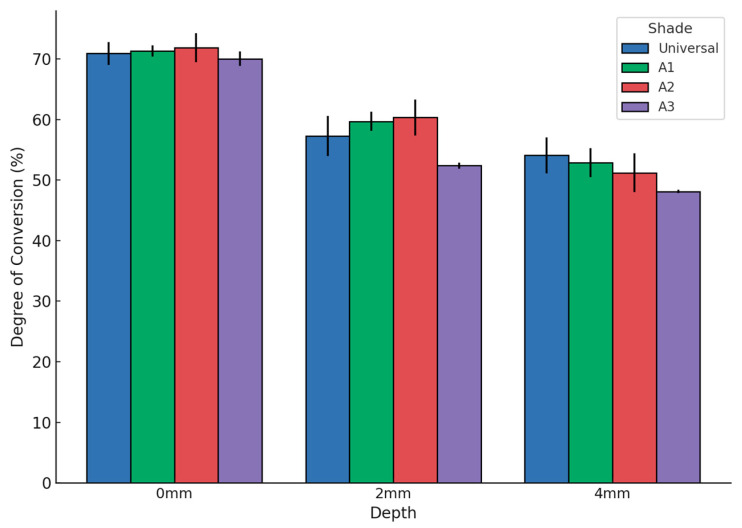
Degree of conversion (DC, %) of the bulk-fill composite for different shades (Universal, A1, A2, A3) and specimen thicknesses (0 mm, 2 mm, 4 mm). Error bars represent standard deviations. The DC decreased with increasing thickness, and darker shades exhibited lower DC values.

**Figure 7:**
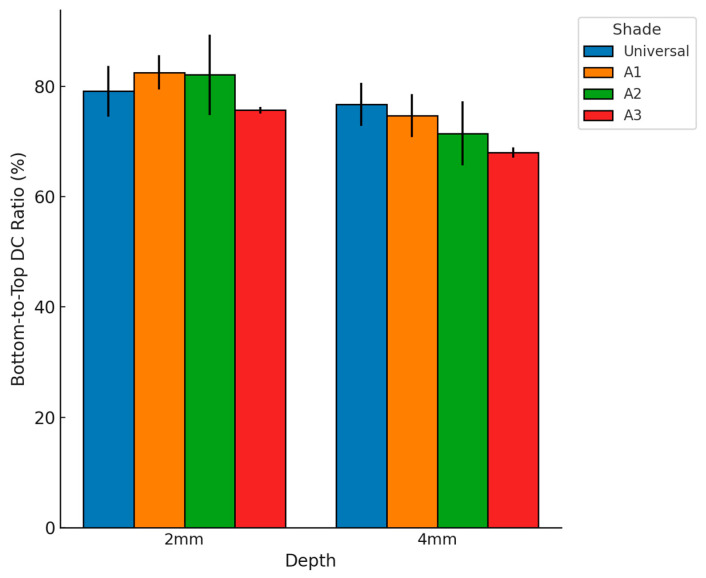
Bottom-to-top DC ratio (%) of the bulk-fill composite for different shades (Universal, A1, A2, A3) and specimen thicknesses (2 mm, 4 mm). Error bars represent standard deviations. Darker shades show greater reductions in polymerization efficiency with increasing thickness.

The normally distributed data collected from the degree of conversion (DC) measurements are presented as mean ± standard deviation in the Supplementary Materials (File S2: Degree of Conversion Data Tables) and graphically illustrated in Figure 6 and Figure 7. Polymerization was performed following the manufacturer’s instructions. To evaluate the DC of the 0 mm surfaces for each shade, statistical analysis included samples polymerized for 20 and 40 s. The DC values ranged from 71.86% (A2, 0 mm) to 48.12% (A3, 4 mm).

### 3.1. Effect of Depth on DC (One-Way ANOVA and Tukey HSD)

One-Way ANOVA (*p*-value < 0.05) conducted on the Universal (U) samples showed significant differences (*p*-value = 0.00001) between the depth groups (0 mm, 2 mm, and 4 mm). Further analysis using the Tukey HSD test revealed that the only comparable group was between 2 mm and 4 mm (*p*-value = 0.29).

The same One-Way ANOVA was performed for the other shades (A1, A2, and A3) at different depths, revealing a significant variation in DC (*p*-value < 0.05) across all clusters.

### 3.2. Effect of Shade on DC (Welch’s t-Test Analysis)

The effect of shade on DC was assessed using Welch’s *t*-tests, comparing different groups at the same depth. At 0 mm thickness, DC values were similar across shades, with no significant differences observed (Universal: 70.91% ± 1.9; A1: 71.30% ± 0.9; A2: 71.86% ± 2.4; A3: 70.01% ± 1.2).

At 2 mm thickness, statistically significant differences were found. A2 (60.32% ± 3.0) and A1 (59.68% ± 1.6) exhibited higher DC values compared to A3 (52.40% ± 0.5) (*p* < 0.05). Universal (57.28% ± 3.3) showed intermediate values.

At 4 mm thickness, A3 again showed the lowest DC (48.12% ± 0.3), significantly different from A2 (51.20% ± 3.2), A1 (52.90% ± 2.4), and Universal (54.07% ± 3.0) (*p* < 0.05).

Overall, darker shades (particularly A3) exhibited significantly lower DC values at increasing depths, as shown in Figure 6.

### 3.3. Bottom-to-Top DC Ratio and Depth Comparison

The bottom-to-top DC ratio values are presented in Figure 7. One-Way ANOVA and Welch’s *t*-test (*p*-value < 0.05) were applied to evaluate differences.

At 2 mm thickness, bottom-to-top DC ratios were comparable among shades. Universal showed a ratio of 79.10% ± 4.6, A1 was 82.51% ± 3.1, A2 reached 82.06% ± 7.3, and A3 exhibited 75.65% ± 0.6. No statistically significant differences were observed at this depth (*p* > 0.05). At 4 mm thickness, significant differences were detected (*p* < 0.05). Universal recorded a ratio of 76.68% ± 3.9, A1 was 74.63% ± 3.9, A2 was 71.46% ± 5.8, and A3 presented the lowest value at 68.00% ± 0.9, which was significantly lower compared to the other shades. Overall, the Universal shade maintained relatively stable bottom-to-top DC ratios across depths, whereas darker shades, particularly A3, exhibited a more pronounced reduction in polymerization efficiency with increasing thickness.

## 4. Discussion

The selection of restorative materials in pediatric dentistry requires a balance between efficiency, durability, and clinical feasibility. Ensuring adequate polymerization is crucial, particularly in younger patients, where restorative success depends on both material properties and the ability to achieve optimal isolation during the procedure.

### 4.1. Factors Influencing Polymerization in Pediatric Dentistry

The setting process of composite resins has a major effect on their mechanical and biological properties [18]. Filler loading, shape and size, resin matrix composition, photoinitiator concentration, DC, and the polymerization conditions are all factors influencing composite polymerization. It seems that bulk-fill resin composite manufacturers have followed different strategies to increase the depth of cure in these resin composites, integrating bioactive properties and self-curing/photo-curing mechanisms to enhance clinical performance [19,20]. These improvements not only optimize polymerization but also promote a more biologically favorable environment for long-term restorations. This may explain the high DC observed at 4 mm for bulk-fill materials compared to conventional composites.

In the current study, the DC of a newly introduced bulk-fill flowable resin was compared at different depths and shades. The results rejected the null hypothesis, showing that both variables influenced polymerization, with depth having the greatest impact on DC.

The external factors involving the light-curing unit can be considered constant in this study. However, it is important to note that the curing time was 20 s for the Universal shade and 40 s for the A1, A2, and A3 shades, as reported in Table 2 and according to the manufacturer’s instructions [21]. This should be considered when comparing the degree of conversion results among groups.

The DC at 0 mm showed no significant difference between groups (One-Way ANOVA; *p* < 0.05), indicating that the DC achieved at the upper surface of the samples, in direct contact with light, is closer to the maximum. This result acknowledges the impossibility of achieving total conversion of reactive monomers into composite resin [22].

The obtained DC values ranged from 74.95% to a low of 44.75%, which aligns with previous studies [23]. However, the literature shows variability in results, likely attributable to sample preparation conditions, polymerization protocols, spectrophotometer measurement procedures, and material properties [24].

Certainly, the distance between the lamp tip and the material surface influences polymerization. A reduction in irradiation power due to increased sample depth, coupled with light attenuation by the composite material, significantly affects curing efficiency [25].

### 4.2. Depth and Shade Effects on Degree of Conversion

The DC of the Universal shade was comparable between the 2 mm and 4 mm surfaces, likely due to its greater translucency. Interestingly, the cure time for these two groups was identical. Comparisons of the Universal shade at different depths revealed statistically significant differences for deeper layers. For thicker increments exceeding 4 mm, shrinkage stress should also be carefully considered.

The shade groups A1, A2, and A3 showed statistically significant variations (One-Way ANOVA and Tukey HSD; *p* < 0.05) in DC for each analyzed surface, even with doubled polymerization times for the 4 mm groups. These findings confirm that depth remains a critical variable.

The color and translucency characteristics of the composite material are equally influenced by the organic matrix and the inorganic component, as well as by thickness. As thickness increases, light penetration diminishes, leading to color changes and reduced polymerization efficiency [22]. This is especially critical in pediatric anterior restorations, where aesthetic outcomes are directly tied to the restoration’s translucency and shade stability, influencing a child’s confidence and social interactions.

### 4.3. Clinical Implications and Future Perspectives

Current trends in pediatric restorative dentistry emphasize the use of bioactive, bulk-fill, and auto-cure/photo-cure materials, particularly for posterior teeth, including young permanent molars [19,26]. These materials have been developed to address common clinical challenges, such as humidity control, polymerization shrinkage, and patient compliance. Their ability to provide reliable polymerization, enhance remineralization, and reduce the need for extensive cavity preparation supports a minimally invasive, ultraconservative approach to pediatric restorations [27]. Bulk-fill resin composites are particularly advantageous due to their translucency, which allows deeper polymerization, ensuring adequate curing even in restorations with increased thickness [10]. Additionally, advancements in bioactive bulk-fill materials offer not only improved curing efficiency but also potential benefits such as ion release, which can aid in maintaining enamel integrity and supporting the surrounding dental tissues [28]. Therefore, the incorporation of bioactive materials in pediatric restorations may further enhance longevity by promoting remineralization and reducing the risk of secondary caries, which is a crucial factor in young patients with high caries risk [29].

However, their use should be primarily directed towards restorations in young permanent teeth, particularly in cases where rubber dam isolation is feasible, ensuring optimal polymerization and longevity [30]. In primary teeth, hybrid and bulk-fill composite resins are recommended for both occlusal (Class I) and occluso-proximal (Class II) restorations of primary carious molars, provided that an appropriate curing protocol is followed to ensure complete polymerization and clinical efficacy [3].

The comparison between shades at equivalent depths showed significant differences (Welch’s *t*-test, *p*-value < 0.05). Notably, the A3 shade consistently exhibited lower DC values, likely due to its higher chroma, lower translucency, and higher light absorption. However, its use in pediatric restorations is extremely rare, as primary and early permanent teeth predominantly present lighter shades, such as A1, A2 and B2 [31]. While findings related to the A3 shade are relevant for dental material research, they have minimal clinical relevance in pediatric dentistry. Nonetheless, its inclusion in this study was intentional, as it reflects real-world situations frequently encountered in public or social dentistry. In such contexts, particularly when treating underserved populations, clinicians often have access to a limited range of shades and may rely on a single available option for all restorations, regardless of patient age or case specifics. Therefore, including the A3 shade helps simulate these practical constraints and enhances the generalizability of the findings to broader clinical scenarios.

This study has several limitations that should be addressed in future research. Firstly, the investigation was conducted under in vitro conditions, which do not fully replicate the clinical environment of pediatric dentistry. Factors such as saliva, oral temperature, and variations in cavity morphology were not considered, potentially influencing the DC in real-world settings. Furthermore, shade comparisons are limited by the differing curing times applied, as recommended by the manufacturer for each shade. While this introduces a constraint in terms of direct comparability, we chose to follow the manufacturer’s instructions to simulate realistic clinical conditions as closely as possible. The aim was to evaluate the materials under optimal and clinically relevant curing protocols, despite the resulting variation in exposure time [32]. Additionally, the study focused on a single bulk-fill composite, limiting the generalizability of the findings to other materials available on the market. The psychological component of pediatric dental procedures, while acknowledged, was not directly studied; hence, the benefits of reduced procedural time on patient compliance remain theoretical. Moreover, the evaluation of the DC relied solely on spectroscopic methods, without correlating the results to clinical parameters such as restoration durability, wear resistance, or secondary caries incidence. Further in vivo studies are needed to validate the clinical applicability of these findings in pediatric patients. Finally, an important aspect that was not assessed in the present study is the potential release of residual monomers from bulk-fill resin composites. Several in vitro studies have demonstrated that common dental methacrylates such as TEGDMA, Bis-GMA, and UDMA can have cytotoxic effects on various human cells, including neutrophils and gingival fibroblasts, and may induce genotoxicity [33,34,35,36]. Additionally, the elution of monomers from different bulk-fill composites has been documented [37], highlighting the importance of optimizing polymerization protocols and further evaluating the biological safety of these materials. Future research should include a detailed investigation of residual monomer release and its potential effects on pulpal and periapical tissues, particularly in pediatric patients where dentin thickness is reduced.

## 5. Conclusions

This study confirms that both thickness and shade significantly influence the DC of low-viscosity bulk-fill composites. In pediatric dentistry, where rapid procedures and material reliability are paramount, clinicians must consider these variables carefully to optimize outcomes. Moreover, achieving an adequate DC is essential to ensure the mechanical stability and biocompatibility of restorations. While these materials show promising properties, their use in primary teeth should be limited to selected cases with minimal cavity extension and adequate patient compliance. Future investigations should also assess the clinical performance of these materials specifically in young permanent dentition, also because a more comprehensive understanding of material behavior in pediatric patients will support safer and more effective restorative strategies. In addition, further studies should address the potential release of residual monomers and their biological effects on pulpal and surrounding tissues. It will also be important for future studies to standardize curing time across shades or, alternatively, evaluate multiple curing regimens to better isolate the influence of shade and thickness on polymerization outcomes.

## Figures and Tables

**Table 1:** Material used in this study and its composition.

**Composite (Type)**	SDR^®^Flow+ (Low-viscosity bulk-fill)
**Manufacturer**	Dentsply Sirona
**Organic component**	EBPADMA TEGDMA Modified urethane dimethacrylate resin Butylated hydroxyl toluene (BHT)
**Filler**	Particles of inorganic filler range from 20 nm to 10 μm, total filler 47.3% by volume. Barium-alumino-fluoro-borosilicate glass Strontium alumino-fluoro-silicate glass.
**Photoactive component**	Camphorquinone (CQ) photoinitiator Photoaccelerator UV stabilizer Fluorescing agent
**Shade component**	Iron oxide pigments Titanium dioxide

**Table 2:** Curing protocol.

Shade	2 mm	4 mm
**Universal**	20 s	20 s
**A1 A2 A3**	20 s	40 s

## Data Availability

Data are reported in the manuscript.

## Supplementary Materials

The following supporting information can be downloaded at https://www.mdpi.com/article/10.3390/dj13080352/s1. File S1: ATR-FTIR Raw Data Supporting DC Evaluation; File S2: Degree of Conversion Data Tables.
