# Supplementary material for: Impact of Shades and Thickness on the Polymerization of Low-Viscosity Bulk-Fill Composites in Pediatric Restorations: An In Vitro Study
Source: Dent J (Basel). 2025 Aug 1;13(8):352. doi: 10.3390/dj13080352 (PMC12385521; doi:10.3390/dj13080352)
Supplement: Supplementary file 1 [file dentistry-3684601-supplementary.pdf › dentistry-3684601-supplementary.pdf]

A1_2mm_a

PE IR SUBTECH SPECTRUM ASCII PEDS 4.00

-1

A1_2mm_a_1_1_1.ASC

21/07/23

16:11:46.00

21/12/06

15:42:15.00

ir

Sample 017 By ir Date venerd, luglio 23 2021

400.000000

#HDR

-1

-1

#GR

cm-1

A

1.0

0.0

4000.000000

-0.250000

14401

8

0.026952

0.000541

#DATA

4000.000000 0.000695

3999.750000	0.000701
3999.500000	0.000708
3999.250000	0.000714
3999.000000	0.000721
3998.750000	0.000728
3998.500000	0.000733
3998.250000	0.000733
3998.000000	0.000729
3997.750000	0.000723
3997.500000	0.000715
3997.250000	0.000710
3997.000000	0.000707
3996.750000	0.000711
3996.500000	0.000720
3996.250000	0.000735
3996.000000	0.000752
3995.750000	0.000771
3995.500000	0.000788
3995.250000	0.000800
3995.000000	0.000807
3994.750000	0.000808
3994.500000	0.000805
3994.250000	0.000800
3994.000000	0.000794
3993.750000	0.000788
3993.500000	0.000785

3993.250000	0.000783
3993.000000	0.000782
3992.750000	0.000780
3992.500000	0.000779
3992.250000	0.000776
3992.000000	0.000773
3991.750000	0.000771
3991.500000	0.000769
3991.250000	0.000770
3991.000000	0.000773
3990.750000	0.000778
3990.500000	0.000783
3990.250000	0.000788
3990.000000	0.000791
3989.750000	0.000793
3989.500000	0.000793
3989.250000	0.000791
3989.000000	0.000790
3988.750000	0.000789
3988.500000	0.000790
3988.250000	0.000793
3988.000000	0.000798
3987.750000	0.000805
3987.500000	0.000814
3987.250000	0.000823
3987.000000	0.000831

3986.750000	0.000839
3986.500000	0.000843
3986.250000	0.000844
3986.000000	0.000841
3985.750000	0.000834
3985.500000	0.000824
3985.250000	0.000811
3985.000000	0.000797
3984.750000	0.000784
3984.500000	0.000774
3984.250000	0.000768
3984.000000	0.000768
3983.750000	0.000773
3983.500000	0.000781
3983.250000	0.000793
3983.000000	0.000805
3982.750000	0.000816
3982.500000	0.000824
3982.250000	0.000829
3982.000000	0.000829
3981.750000	0.000827
3981.500000	0.000823
3981.250000	0.000818
3981.000000	0.000815
3980.750000	0.000813
3980.500000	0.000814

3980.250000	0.000817
3980.000000	0.000821
3979.750000	0.000823
3979.500000	0.000824
3979.250000	0.000823
3979.000000	0.000820
3978.750000	0.000818
3978.500000	0.000817
3978.250000	0.000820
3978.000000	0.000827
3977.750000	0.000839
3977.500000	0.000852
3977.250000	0.000866
3977.000000	0.000877
3976.750000	0.000883
3976.500000	0.000884
3976.250000	0.000880
3976.000000	0.000873
3975.750000	0.000864
3975.500000	0.000856
3975.250000	0.000851
3975.000000	0.000848
3974.750000	0.000849
3974.500000	0.000853
3974.250000	0.000859
3974.000000	0.000868

3973.750000	0.000878
3973.500000	0.000888
3973.250000	0.000899
3973.000000	0.000909
3972.750000	0.000918
3972.500000	0.000923
3972.250000	0.000925
3972.000000	0.000925
3971.750000	0.000922
3971.500000	0.000918
3971.250000	0.000913
3971.000000	0.000909
3970.750000	0.000906
3970.500000	0.000905
3970.250000	0.000905
3970.000000	0.000906
3969.750000	0.000908
3969.500000	0.000910
3969.250000	0.000911
3969.000000	0.000912
3968.750000	0.000912
3968.500000	0.000910
3968.250000	0.000906
3968.000000	0.000901
3967.750000	0.000895
3967.500000	0.000889

3967.250000	0.000884
3967.000000	0.000879
3966.750000	0.000876
3966.500000	0.000875
3966.250000	0.000876
3966.000000	0.000879
3965.750000	0.000885
3965.500000	0.000893
3965.250000	0.000903
3965.000000	0.000914
3964.750000	0.000924
3964.500000	0.000932
3964.250000	0.000935
3964.000000	0.000935
3963.750000	0.000929
3963.500000	0.000921
3963.250000	0.000912
3963.000000	0.000905
3962.750000	0.000902
3962.500000	0.000906
3962.250000	0.000916
3962.000000	0.000929
3961.750000	0.000944
3961.500000	0.000958
3961.250000	0.000967
3961.000000	0.000972

3960.750000	0.000972
3960.500000	0.000970
3960.250000	0.000967
3960.000000	0.000966
3959.750000	0.000967
3959.500000	0.000970
3959.250000	0.000976
3959.000000	0.000981
3958.750000	0.000984
3958.500000	0.000985
3958.250000	0.000984
3958.000000	0.000980
3957.750000	0.000976
3957.500000	0.000973
3957.250000	0.000971
3957.000000	0.000971
3956.750000	0.000971
3956.500000	0.000971
3956.250000	0.000968
3956.000000	0.000962
3955.750000	0.000953
3955.500000	0.000943
3955.250000	0.000932
3955.000000	0.000923
3954.750000	0.000919
3954.500000	0.000920

3954.250000	0.000928
3954.000000	0.000941
3953.750000	0.000956
3953.500000	0.000970
3953.250000	0.000980
3953.000000	0.000984
3952.750000	0.000981
3952.500000	0.000972
3952.250000	0.000959
3952.000000	0.000947
3951.750000	0.000939
3951.500000	0.000938
3951.250000	0.000946
3951.000000	0.000963
3950.750000	0.000986
3950.500000	0.001011
3950.250000	0.001034
3950.000000	0.001052
3949.750000	0.001063
3949.500000	0.001069
3949.250000	0.001073
3949.000000	0.001079
3948.750000	0.001087
3948.500000	0.001100
3948.250000	0.001114
3948.000000	0.001127

3947.750000	0.001135
3947.500000	0.001134
3947.250000	0.001126
3947.000000	0.001111
3946.750000	0.001094
3946.500000	0.001080
3946.250000	0.001071
3946.000000	0.001069
3945.750000	0.001074
3945.500000	0.001081
3945.250000	0.001086
3945.000000	0.001087
3944.750000	0.001080
3944.500000	0.001066
3944.250000	0.001047
3944.000000	0.001028
3943.750000	0.001015
3943.500000	0.001009
3943.250000	0.001012
3943.000000	0.001022
3942.750000	0.001032
3942.500000	0.001040
3942.250000	0.001042
3942.000000	0.001041
3941.750000	0.001038
3941.500000	0.001038

3941.250000	0.001042
3941.000000	0.001053
3940.750000	0.001068
3940.500000	0.001088
3940.250000	0.001107
3940.000000	0.001125
3939.750000	0.001139
3939.500000	0.001148
3939.250000	0.001152
3939.000000	0.001151
3938.750000	0.001148
3938.500000	0.001143
3938.250000	0.001136
3938.000000	0.001129
3937.750000	0.001121
3937.500000	0.001114
3937.250000	0.001107
3937.000000	0.001101
3936.750000	0.001097
3936.500000	0.001095
3936.250000	0.001095
3936.000000	0.001099
3935.750000	0.001106
3935.500000	0.001116
3935.250000	0.001126
3935.000000	0.001133

3934.750000	0.001136
3934.500000	0.001133
3934.250000	0.001124
3934.000000	0.001111
3933.750000	0.001097
3933.500000	0.001088
3933.250000	0.001085
3933.000000	0.001091
3932.750000	0.001101
3932.500000	0.001113
3932.250000	0.001121
3932.000000	0.001122
3931.750000	0.001118
3931.500000	0.001113
3931.250000	0.001109
3931.000000	0.001112
3930.750000	0.001124
3930.500000	0.001142
3930.250000	0.001164
3930.000000	0.001186
3929.750000	0.001203
3929.500000	0.001213
3929.250000	0.001216
3929.000000	0.001214
3928.750000	0.001211
3928.500000	0.001208

3928.250000	0.001208
3928.000000	0.001209
3927.750000	0.001210
3927.500000	0.001208
3927.250000	0.001201
3927.000000	0.001188
3926.750000	0.001171
3926.500000	0.001152
3926.250000	0.001134
3926.000000	0.001124
3925.750000	0.001122
3925.500000	0.001130
3925.250000	0.001146
3925.000000	0.001166
3924.750000	0.001185
3924.500000	0.001199
3924.250000	0.001209
3924.000000	0.001214
3923.750000	0.001216
3923.500000	0.001218
3923.250000	0.001220
3923.000000	0.001222
3922.750000	0.001224
3922.500000	0.001225
3922.250000	0.001223
3922.000000	0.001219

3921.750000	0.001216
3921.500000	0.001214
3921.250000	0.001218
3921.000000	0.001228
3920.750000	0.001242
3920.500000	0.001255
3920.250000	0.001260
3920.000000	0.001252
3919.750000	0.001229
3919.500000	0.001195
3919.250000	0.001156
3919.000000	0.001122
3918.750000	0.001099
3918.500000	0.001093
3918.250000	0.001106
3918.000000	0.001136
3917.750000	0.001177
3917.500000	0.001220
3917.250000	0.001252
3917.000000	0.001268
3916.750000	0.001263
3916.500000	0.001242
3916.250000	0.001215
3916.000000	0.001189
3915.750000	0.001173
3915.500000	0.001169

3915.250000	0.001179
3915.000000	0.001200
3914.750000	0.001227
3914.500000	0.001257
3914.250000	0.001285
3914.000000	0.001309
3913.750000	0.001325
3913.500000	0.001332
3913.250000	0.001332
3913.000000	0.001324
3912.750000	0.001311
3912.500000	0.001296
3912.250000	0.001281
3912.000000	0.001267
3911.750000	0.001256
3911.500000	0.001249
3911.250000	0.001245
3911.000000	0.001242
3910.750000	0.001241
3910.500000	0.001239
3910.250000	0.001238
3910.000000	0.001237
3909.750000	0.001238
3909.500000	0.001240
3909.250000	0.001244
3909.000000	0.001249

3908.750000	0.001253
3908.500000	0.001255
3908.250000	0.001255
3908.000000	0.001252
3907.750000	0.001246
3907.500000	0.001240
3907.250000	0.001231
3907.000000	0.001218
3906.750000	0.001200
3906.500000	0.001175
3906.250000	0.001144
3906.000000	0.001110
3905.750000	0.001079
3905.500000	0.001059
3905.250000	0.001054
3905.000000	0.001064
3904.750000	0.001084
3904.500000	0.001102
3904.250000	0.001107
3904.000000	0.001095
3903.750000	0.001070
3903.500000	0.001043
3903.250000	0.001026
3903.000000	0.001031
3902.750000	0.001061
3902.500000	0.001110

3902.250000	0.001166
3902.000000	0.001209
3901.750000	0.001227
3901.500000	0.001215
3901.250000	0.001180
3901.000000	0.001139
3900.750000	0.001107
3900.500000	0.001101
3900.250000	0.001129
3900.000000	0.001190
3899.750000	0.001272
3899.500000	0.001353
3899.250000	0.001409
3899.000000	0.001425
3898.750000	0.001400
3898.500000	0.001348
3898.250000	0.001290
3898.000000	0.001244
3897.750000	0.001222
3897.500000	0.001227
3897.250000	0.001255
3897.000000	0.001296
3896.750000	0.001340
3896.500000	0.001377
3896.250000	0.001401
3896.000000	0.001410

3895.750000	0.001407
3895.500000	0.001396
3895.250000	0.001383
3895.000000	0.001371
3894.750000	0.001361
3894.500000	0.001353
3894.250000	0.001342
3894.000000	0.001324
3893.750000	0.001297
3893.500000	0.001262
3893.250000	0.001221
3893.000000	0.001180
3892.750000	0.001146
3892.500000	0.001130
3892.250000	0.001136
3892.000000	0.001164
3891.750000	0.001204
3891.500000	0.001239
3891.250000	0.001253
3891.000000	0.001241
3890.750000	0.001208
3890.500000	0.001171
3890.250000	0.001147
3890.000000	0.001148
3889.750000	0.001177
3889.500000	0.001228

3889.250000	0.001290
3889.000000	0.001349
3888.750000	0.001391
3888.500000	0.001408
3888.250000	0.001393
3888.000000	0.001350
3887.750000	0.001282
3887.500000	0.001202
3887.250000	0.001126
3887.000000	0.001070
3886.750000	0.001050
3886.500000	0.001074
3886.250000	0.001133
3886.000000	0.001203
3885.750000	0.001255
3885.500000	0.001271
3885.250000	0.001253
3885.000000	0.001218
3884.750000	0.001190
3884.500000	0.001184
3884.250000	0.001207
3884.000000	0.001255
3883.750000	0.001318
3883.500000	0.001383
3883.250000	0.001437
3883.000000	0.001468

3882.750000	0.001473
3882.500000	0.001453
3882.250000	0.001414
3882.000000	0.001366
3881.750000	0.001321
3881.500000	0.001291
3881.250000	0.001282
3881.000000	0.001296
3880.750000	0.001320
3880.500000	0.001334
3880.250000	0.001323
3880.000000	0.001281
3879.750000	0.001221
3879.500000	0.001162
3879.250000	0.001123
3879.000000	0.001115
3878.750000	0.001141
3878.500000	0.001196
3878.250000	0.001269
3878.000000	0.001349
3877.750000	0.001424
3877.500000	0.001483
3877.250000	0.001523
3877.000000	0.001541
3876.750000	0.001539
3876.500000	0.001523

3876.250000	0.001497
3876.000000	0.001467
3875.750000	0.001438
3875.500000	0.001415
3875.250000	0.001401
3875.000000	0.001399
3874.750000	0.001404
3874.500000	0.001405
3874.250000	0.001392
3874.000000	0.001358
3873.750000	0.001305
3873.500000	0.001246
3873.250000	0.001196
3873.000000	0.001167
3872.750000	0.001163
3872.500000	0.001179
3872.250000	0.001208
3872.000000	0.001238
3871.750000	0.001261
3871.500000	0.001274
3871.250000	0.001279
3871.000000	0.001288
3870.750000	0.001314
3870.500000	0.001363
3870.250000	0.001426
3870.000000	0.001477

3869.750000	0.001489
3869.500000	0.001452
3869.250000	0.001377
3869.000000	0.001293
3868.750000	0.001228
3868.500000	0.001202
3868.250000	0.001218
3868.000000	0.001268
3867.750000	0.001335
3867.500000	0.001402
3867.250000	0.001456
3867.000000	0.001485
3866.750000	0.001489
3866.500000	0.001472
3866.250000	0.001447
3866.000000	0.001427
3865.750000	0.001423
3865.500000	0.001436
3865.250000	0.001452
3865.000000	0.001454
3864.750000	0.001427
3864.500000	0.001373
3864.250000	0.001305
3864.000000	0.001244
3863.750000	0.001206
3863.500000	0.001201

3863.250000	0.001231
3863.000000	0.001293
3862.750000	0.001379
3862.500000	0.001474
3862.250000	0.001561
3862.000000	0.001624
3861.750000	0.001652
3861.500000	0.001644
3861.250000	0.001609
3861.000000	0.001564
3860.750000	0.001524
3860.500000	0.001499
3860.250000	0.001496
3860.000000	0.001511
3859.750000	0.001540
3859.500000	0.001574
3859.250000	0.001606
3859.000000	0.001631
3858.750000	0.001647
3858.500000	0.001657
3858.250000	0.001661
3858.000000	0.001663
3857.750000	0.001659
3857.500000	0.001646
3857.250000	0.001616
3857.000000	0.001564

3856.750000	0.001486
3856.500000	0.001385
3856.250000	0.001262
3856.000000	0.001123
3855.750000	0.000974
3855.500000	0.000824
3855.250000	0.000692
3855.000000	0.000601
3854.750000	0.000574
3854.500000	0.000611
3854.250000	0.000684
3854.000000	0.000747
3853.750000	0.000765
3853.500000	0.000741
3853.250000	0.000715
3853.000000	0.000729
3852.750000	0.000803
3852.500000	0.000928
3852.250000	0.001077
3852.000000	0.001222
3851.750000	0.001344
3851.500000	0.001437
3851.250000	0.001502
3851.000000	0.001549
3850.750000	0.001586
3850.500000	0.001622

3850.250000	0.001659
3850.000000	0.001697
3849.750000	0.001733
3849.500000	0.001763
3849.250000	0.001782
3849.000000	0.001787
3848.750000	0.001779
3848.500000	0.001760
3848.250000	0.001736
3848.000000	0.001710
3847.750000	0.001687
3847.500000	0.001668
3847.250000	0.001652
3847.000000	0.001638
3846.750000	0.001623
3846.500000	0.001605
3846.250000	0.001583
3846.000000	0.001556
3845.750000	0.001527
3845.500000	0.001500
3845.250000	0.001481
3845.000000	0.001475
3844.750000	0.001487
3844.500000	0.001513
3844.250000	0.001545
3844.000000	0.001568

3843.750000	0.001568
3843.500000	0.001541
3843.250000	0.001488
3843.000000	0.001422
3842.750000	0.001356
3842.500000	0.001302
3842.250000	0.001270
3842.000000	0.001265
3841.750000	0.001286
3841.500000	0.001329
3841.250000	0.001384
3841.000000	0.001435
3840.750000	0.001467
3840.500000	0.001470
3840.250000	0.001442
3840.000000	0.001392
3839.750000	0.001333
3839.500000	0.001278
3839.250000	0.001241
3839.000000	0.001236
3838.750000	0.001269
3838.500000	0.001339
3838.250000	0.001424
3838.000000	0.001492
3837.750000	0.001513
3837.500000	0.001478

3837.250000	0.001405
3837.000000	0.001324
3836.750000	0.001264
3836.500000	0.001242
3836.250000	0.001261
3836.000000	0.001318
3835.750000	0.001398
3835.500000	0.001485
3835.250000	0.001558
3835.000000	0.001597
3834.750000	0.001592
3834.500000	0.001548
3834.250000	0.001481
3834.000000	0.001411
3833.750000	0.001357
3833.500000	0.001328
3833.250000	0.001327
3833.000000	0.001352
3832.750000	0.001399
3832.500000	0.001462
3832.250000	0.001528
3832.000000	0.001581
3831.750000	0.001605
3831.500000	0.001593
3831.250000	0.001549
3831.000000	0.001485

3830.750000	0.001422
3830.500000	0.001374
3830.250000	0.001351
3830.000000	0.001355
3829.750000	0.001380
3829.500000	0.001418
3829.250000	0.001463
3829.000000	0.001506
3828.750000	0.001542
3828.500000	0.001572
3828.250000	0.001595
3828.000000	0.001614
3827.750000	0.001628
3827.500000	0.001638
3827.250000	0.001638
3827.000000	0.001626
3826.750000	0.001600
3826.500000	0.001562
3826.250000	0.001519
3826.000000	0.001481
3825.750000	0.001456
3825.500000	0.001450
3825.250000	0.001463
3825.000000	0.001490
3824.750000	0.001522
3824.500000	0.001549

3824.250000	0.001560
3824.000000	0.001549
3823.750000	0.001512
3823.500000	0.001449
3823.250000	0.001362
3823.000000	0.001258
3822.750000	0.001145
3822.500000	0.001039
3822.250000	0.000958
3822.000000	0.000925
3821.750000	0.000949
3821.500000	0.001021
3821.250000	0.001110
3821.000000	0.001180
3820.750000	0.001210
3820.500000	0.001205
3820.250000	0.001188
3820.000000	0.001181
3819.750000	0.001197
3819.500000	0.001238
3819.250000	0.001298
3819.000000	0.001363
3818.750000	0.001420
3818.500000	0.001457
3818.250000	0.001466
3818.000000	0.001443

3817.750000	0.001394
3817.500000	0.001329
3817.250000	0.001270
3817.000000	0.001239
3816.750000	0.001253
3816.500000	0.001309
3816.250000	0.001379
3816.000000	0.001427
3815.750000	0.001431
3815.500000	0.001394
3815.250000	0.001343
3815.000000	0.001307
3814.750000	0.001304
3814.500000	0.001335
3814.250000	0.001393
3814.000000	0.001464
3813.750000	0.001536
3813.500000	0.001600
3813.250000	0.001650
3813.000000	0.001681
3812.750000	0.001694
3812.500000	0.001694
3812.250000	0.001685
3812.000000	0.001671
3811.750000	0.001657
3811.500000	0.001644

3811.250000	0.001631
3811.000000	0.001618
3810.750000	0.001606
3810.500000	0.001593
3810.250000	0.001577
3810.000000	0.001558
3809.750000	0.001530
3809.500000	0.001491
3809.250000	0.001440
3809.000000	0.001379
3808.750000	0.001313
3808.500000	0.001250
3808.250000	0.001202
3808.000000	0.001181
3807.750000	0.001196
3807.500000	0.001240
3807.250000	0.001293
3807.000000	0.001327
3806.750000	0.001325
3806.500000	0.001289
3806.250000	0.001238
3806.000000	0.001198
3805.750000	0.001190
3805.500000	0.001218
3805.250000	0.001277
3805.000000	0.001354

3804.750000	0.001432
3804.500000	0.001498
3804.250000	0.001539
3804.000000	0.001548
3803.750000	0.001520
3803.500000	0.001459
3803.250000	0.001370
3803.000000	0.001268
3802.750000	0.001169
3802.500000	0.001096
3802.250000	0.001070
3802.000000	0.001103
3801.750000	0.001182
3801.500000	0.001271
3801.250000	0.001334
3801.000000	0.001352
3800.750000	0.001333
3800.500000	0.001303
3800.250000	0.001284
3800.000000	0.001290
3799.750000	0.001322
3799.500000	0.001372
3799.250000	0.001429
3799.000000	0.001482
3798.750000	0.001523
3798.500000	0.001545

3798.250000	0.001548
3798.000000	0.001535
3797.750000	0.001514
3797.500000	0.001493
3797.250000	0.001481
3797.000000	0.001477
3796.750000	0.001477
3796.500000	0.001473
3796.250000	0.001460
3796.000000	0.001438
3795.750000	0.001413
3795.500000	0.001392
3795.250000	0.001382
3795.000000	0.001388
3794.750000	0.001409
3794.500000	0.001439
3794.250000	0.001476
3794.000000	0.001511
3793.750000	0.001541
3793.500000	0.001561
3793.250000	0.001570
3793.000000	0.001569
3792.750000	0.001561
3792.500000	0.001549
3792.250000	0.001537
3792.000000	0.001528

3791.750000	0.001521
3791.500000	0.001518
3791.250000	0.001516
3791.000000	0.001515
3790.750000	0.001514
3790.500000	0.001513
3790.250000	0.001511
3790.000000	0.001509
3789.750000	0.001509
3789.500000	0.001509
3789.250000	0.001510
3789.000000	0.001510
3788.750000	0.001507
3788.500000	0.001502
3788.250000	0.001492
3788.000000	0.001480
3787.750000	0.001466
3787.500000	0.001453
3787.250000	0.001443
3787.000000	0.001438
3786.750000	0.001439
3786.500000	0.001447
3786.250000	0.001462
3786.000000	0.001483
3785.750000	0.001506
3785.500000	0.001530

3785.250000	0.001549
3785.000000	0.001562
3784.750000	0.001565
3784.500000	0.001558
3784.250000	0.001542
3784.000000	0.001523
3783.750000	0.001503
3783.500000	0.001487
3783.250000	0.001479
3783.000000	0.001478
3782.750000	0.001484
3782.500000	0.001492
3782.250000	0.001498
3782.000000	0.001499
3781.750000	0.001492
3781.500000	0.001475
3781.250000	0.001452
3781.000000	0.001425
3780.750000	0.001401
3780.500000	0.001383
3780.250000	0.001376
3780.000000	0.001377
3779.750000	0.001380
3779.500000	0.001379
3779.250000	0.001367
3779.000000	0.001347

3778.750000	0.001327
3778.500000	0.001316
3778.250000	0.001323
3778.000000	0.001348
3777.750000	0.001388
3777.500000	0.001436
3777.250000	0.001483
3777.000000	0.001522
3776.750000	0.001548
3776.500000	0.001559
3776.250000	0.001556
3776.000000	0.001545
3775.750000	0.001529
3775.500000	0.001514
3775.250000	0.001501
3775.000000	0.001491
3774.750000	0.001486
3774.500000	0.001482
3774.250000	0.001481
3774.000000	0.001480
3773.750000	0.001479
3773.500000	0.001477
3773.250000	0.001472
3773.000000	0.001462
3772.750000	0.001444
3772.500000	0.001417

3772.250000	0.001380
3772.000000	0.001336
3771.750000	0.001288
3771.500000	0.001243
3771.250000	0.001211
3771.000000	0.001200
3770.750000	0.001216
3770.500000	0.001257
3770.250000	0.001311
3770.000000	0.001358
3769.750000	0.001382
3769.500000	0.001376
3769.250000	0.001347
3769.000000	0.001311
3768.750000	0.001285
3768.500000	0.001276
3768.250000	0.001289
3768.000000	0.001319
3767.750000	0.001359
3767.500000	0.001402
3767.250000	0.001443
3767.000000	0.001479
3766.750000	0.001510
3766.500000	0.001535
3766.250000	0.001552
3766.000000	0.001559

3765.750000	0.001551
3765.500000	0.001526
3765.250000	0.001490
3765.000000	0.001450
3764.750000	0.001417
3764.500000	0.001399
3764.250000	0.001400
3764.000000	0.001420
3763.750000	0.001451
3763.500000	0.001486
3763.250000	0.001516
3763.000000	0.001536
3762.750000	0.001539
3762.500000	0.001526
3762.250000	0.001499
3762.000000	0.001459
3761.750000	0.001412
3761.500000	0.001363
3761.250000	0.001316
3761.000000	0.001278
3760.750000	0.001255
3760.500000	0.001254
3760.250000	0.001275
3760.000000	0.001307
3759.750000	0.001337
3759.500000	0.001351

3759.250000	0.001345
3759.000000	0.001323
3758.750000	0.001296
3758.500000	0.001279
3758.250000	0.001279
3758.000000	0.001301
3757.750000	0.001346
3757.500000	0.001410
3757.250000	0.001484
3757.000000	0.001555
3756.750000	0.001608
3756.500000	0.001635
3756.250000	0.001632
3756.000000	0.001605
3755.750000	0.001566
3755.500000	0.001524
3755.250000	0.001484
3755.000000	0.001448
3754.750000	0.001409
3754.500000	0.001363
3754.250000	0.001303
3754.000000	0.001226
3753.750000	0.001137
3753.500000	0.001045
3753.250000	0.000971
3753.000000	0.000937

3752.750000	0.000952
3752.500000	0.001005
3752.250000	0.001063
3752.000000	0.001094
3751.750000	0.001085
3751.500000	0.001048
3751.250000	0.001005
3751.000000	0.000978
3750.750000	0.000980
3750.500000	0.001024
3750.250000	0.001116
3750.000000	0.001256
3749.750000	0.001416
3749.500000	0.001552
3749.250000	0.001625
3749.000000	0.001624
3748.750000	0.001574
3748.500000	0.001514
3748.250000	0.001475
3748.000000	0.001467
3747.750000	0.001481
3747.500000	0.001501
3747.250000	0.001506
3747.000000	0.001480
3746.750000	0.001414
3746.500000	0.001303

3746.250000	0.001148
3746.000000	0.000959
3745.750000	0.000763
3745.500000	0.000613
3745.250000	0.000570
3745.000000	0.000659
3744.750000	0.000826
3744.500000	0.000965
3744.250000	0.000986
3744.000000	0.000894
3743.750000	0.000768
3743.500000	0.000696
3743.250000	0.000719
3743.000000	0.000828
3742.750000	0.000992
3742.500000	0.001178
3742.250000	0.001366
3742.000000	0.001536
3741.750000	0.001673
3741.500000	0.001763
3741.250000	0.001801
3741.000000	0.001790
3740.750000	0.001744
3740.500000	0.001679
3740.250000	0.001608
3740.000000	0.001542

3739.750000	0.001486
3739.500000	0.001439
3739.250000	0.001401
3739.000000	0.001369
3738.750000	0.001340
3738.500000	0.001312
3738.250000	0.001285
3738.000000	0.001261
3737.750000	0.001242
3737.500000	0.001231
3737.250000	0.001227
3737.000000	0.001228
3736.750000	0.001232
3736.500000	0.001237
3736.250000	0.001247
3736.000000	0.001267
3735.750000	0.001302
3735.500000	0.001347
3735.250000	0.001387
3735.000000	0.001407
3734.750000	0.001396
3734.500000	0.001359
3734.250000	0.001307
3734.000000	0.001254
3733.750000	0.001213
3733.500000	0.001196

3733.250000	0.001208
3733.000000	0.001254
3732.750000	0.001327
3732.500000	0.001407
3732.250000	0.001469
3732.000000	0.001492
3731.750000	0.001471
3731.500000	0.001419
3731.250000	0.001359
3731.000000	0.001316
3730.750000	0.001300
3730.500000	0.001312
3730.250000	0.001344
3730.000000	0.001385
3729.750000	0.001422
3729.500000	0.001448
3729.250000	0.001456
3729.000000	0.001447
3728.750000	0.001423
3728.500000	0.001388
3728.250000	0.001350
3728.000000	0.001314
3727.750000	0.001284
3727.500000	0.001262
3727.250000	0.001248
3727.000000	0.001241

3726.750000	0.001241
3726.500000	0.001244
3726.250000	0.001251
3726.000000	0.001262
3725.750000	0.001276
3725.500000	0.001293
3725.250000	0.001310
3725.000000	0.001327
3724.750000	0.001339
3724.500000	0.001348
3724.250000	0.001351
3724.000000	0.001352
3723.750000	0.001353
3723.500000	0.001358
3723.250000	0.001372
3723.000000	0.001394
3722.750000	0.001421
3722.500000	0.001443
3722.250000	0.001450
3722.000000	0.001438
3721.750000	0.001409
3721.500000	0.001370
3721.250000	0.001333
3721.000000	0.001308
3720.750000	0.001299
3720.500000	0.001308

3720.250000	0.001332
3720.000000	0.001368
3719.750000	0.001410
3719.500000	0.001451
3719.250000	0.001483
3719.000000	0.001503
3718.750000	0.001510
3718.500000	0.001507
3718.250000	0.001498
3718.000000	0.001490
3717.750000	0.001485
3717.500000	0.001486
3717.250000	0.001491
3717.000000	0.001499
3716.750000	0.001506
3716.500000	0.001511
3716.250000	0.001514
3716.000000	0.001512
3715.750000	0.001506
3715.500000	0.001495
3715.250000	0.001476
3715.000000	0.001446
3714.750000	0.001404
3714.500000	0.001349
3714.250000	0.001284
3714.000000	0.001212

3713.750000	0.001142
3713.500000	0.001083
3713.250000	0.001050
3713.000000	0.001055
3712.750000	0.001099
3712.500000	0.001166
3712.250000	0.001225
3712.000000	0.001249
3711.750000	0.001228
3711.500000	0.001174
3711.250000	0.001116
3711.000000	0.001080
3710.750000	0.001082
3710.500000	0.001127
3710.250000	0.001211
3710.000000	0.001316
3709.750000	0.001418
3709.500000	0.001493
3709.250000	0.001523
3709.000000	0.001508
3708.750000	0.001460
3708.500000	0.001402
3708.250000	0.001355
3708.000000	0.001330
3707.750000	0.001332
3707.500000	0.001355

3707.250000	0.001391
3707.000000	0.001430
3706.750000	0.001465
3706.500000	0.001491
3706.250000	0.001507
3706.000000	0.001513
3705.750000	0.001510
3705.500000	0.001498
3705.250000	0.001479
3705.000000	0.001452
3704.750000	0.001418
3704.500000	0.001379
3704.250000	0.001335
3704.000000	0.001291
3703.750000	0.001250
3703.500000	0.001215
3703.250000	0.001193
3703.000000	0.001189
3702.750000	0.001206
3702.500000	0.001242
3702.250000	0.001289
3702.000000	0.001333
3701.750000	0.001358
3701.500000	0.001359
3701.250000	0.001340
3701.000000	0.001314

3700.750000	0.001295
3700.500000	0.001294
3700.250000	0.001317
3700.000000	0.001360
3699.750000	0.001415
3699.500000	0.001470
3699.250000	0.001516
3699.000000	0.001545
3698.750000	0.001555
3698.500000	0.001548
3698.250000	0.001530
3698.000000	0.001509
3697.750000	0.001492
3697.500000	0.001481
3697.250000	0.001477
3697.000000	0.001480
3696.750000	0.001486
3696.500000	0.001492
3696.250000	0.001496
3696.000000	0.001496
3695.750000	0.001492
3695.500000	0.001484
3695.250000	0.001475
3695.000000	0.001467
3694.750000	0.001460
3694.500000	0.001454

3694.250000	0.001447
3694.000000	0.001438
3693.750000	0.001422
3693.500000	0.001399
3693.250000	0.001367
3693.000000	0.001330
3692.750000	0.001290
3692.500000	0.001254
3692.250000	0.001229
3692.000000	0.001219
3691.750000	0.001223
3691.500000	0.001231
3691.250000	0.001228
3691.000000	0.001200
3690.750000	0.001146
3690.500000	0.001073
3690.250000	0.000996
3690.000000	0.000933
3689.750000	0.000902
3689.500000	0.000922
3689.250000	0.001006
3689.000000	0.001150
3688.750000	0.001322
3688.500000	0.001473
3688.250000	0.001561
3688.000000	0.001577

3687.750000	0.001543
3687.500000	0.001497
3687.250000	0.001471
3687.000000	0.001479
3686.750000	0.001519
3686.500000	0.001581
3686.250000	0.001648
3686.000000	0.001706
3685.750000	0.001746
3685.500000	0.001763
3685.250000	0.001758
3685.000000	0.001735
3684.750000	0.001702
3684.500000	0.001667
3684.250000	0.001635
3684.000000	0.001609
3683.750000	0.001591
3683.500000	0.001580
3683.250000	0.001575
3683.000000	0.001575
3682.750000	0.001576
3682.500000	0.001577
3682.250000	0.001576
3682.000000	0.001571
3681.750000	0.001563
3681.500000	0.001553

3681.250000	0.001543
3681.000000	0.001536
3680.750000	0.001532
3680.500000	0.001534
3680.250000	0.001542
3680.000000	0.001554
3679.750000	0.001569
3679.500000	0.001582
3679.250000	0.001590
3679.000000	0.001588
3678.750000	0.001576
3678.500000	0.001555
3678.250000	0.001524
3678.000000	0.001481
3677.750000	0.001424
3677.500000	0.001350
3677.250000	0.001259
3677.000000	0.001152
3676.750000	0.001042
3676.500000	0.000949
3676.250000	0.000909
3676.000000	0.000953
3675.750000	0.001075
3675.500000	0.001232
3675.250000	0.001361
3675.000000	0.001430

3674.750000	0.001444
3674.500000	0.001438
3674.250000	0.001443
3674.000000	0.001474
3673.750000	0.001529
3673.500000	0.001600
3673.250000	0.001671
3673.000000	0.001730
3672.750000	0.001762
3672.500000	0.001763
3672.250000	0.001732
3672.000000	0.001681
3671.750000	0.001622
3671.500000	0.001571
3671.250000	0.001536
3671.000000	0.001510
3670.750000	0.001484
3670.500000	0.001448
3670.250000	0.001405
3670.000000	0.001370
3669.750000	0.001363
3669.500000	0.001395
3669.250000	0.001465
3669.000000	0.001557
3668.750000	0.001651
3668.500000	0.001729

3668.250000	0.001781
3668.000000	0.001809
3667.750000	0.001819
3667.500000	0.001822
3667.250000	0.001826
3667.000000	0.001837
3666.750000	0.001856
3666.500000	0.001880
3666.250000	0.001903
3666.000000	0.001922
3665.750000	0.001933
3665.500000	0.001935
3665.250000	0.001932
3665.000000	0.001926
3664.750000	0.001921
3664.500000	0.001918
3664.250000	0.001918
3664.000000	0.001921
3663.750000	0.001925
3663.500000	0.001928
3663.250000	0.001930
3663.000000	0.001929
3662.750000	0.001926
3662.500000	0.001922
3662.250000	0.001916
3662.000000	0.001911

3661.750000	0.001906
3661.500000	0.001901
3661.250000	0.001896
3661.000000	0.001890
3660.750000	0.001884
3660.500000	0.001880
3660.250000	0.001878
3660.000000	0.001880
3659.750000	0.001884
3659.500000	0.001890
3659.250000	0.001893
3659.000000	0.001890
3658.750000	0.001878
3658.500000	0.001854
3658.250000	0.001819
3658.000000	0.001777
3657.750000	0.001735
3657.500000	0.001702
3657.250000	0.001692
3657.000000	0.001711
3656.750000	0.001758
3656.500000	0.001818
3656.250000	0.001871
3656.000000	0.001902
3655.750000	0.001910
3655.500000	0.001907

3655.250000	0.001908
3655.000000	0.001924
3654.750000	0.001955
3654.500000	0.001997
3654.250000	0.002037
3654.000000	0.002065
3653.750000	0.002071
3653.500000	0.002051
3653.250000	0.002005
3653.000000	0.001940
3652.750000	0.001864
3652.500000	0.001788
3652.250000	0.001722
3652.000000	0.001670
3651.750000	0.001636
3651.500000	0.001618
3651.250000	0.001611
3651.000000	0.001612
3650.750000	0.001617
3650.500000	0.001626
3650.250000	0.001639
3650.000000	0.001656
3649.750000	0.001678
3649.500000	0.001704
3649.250000	0.001734
3649.000000	0.001771

3648.750000	0.001817
3648.500000	0.001876
3648.250000	0.001953
3648.000000	0.002046
3647.750000	0.002150
3647.500000	0.002248
3647.250000	0.002325
3647.000000	0.002368
3646.750000	0.002375
3646.500000	0.002357
3646.250000	0.002327
3646.000000	0.002302
3645.750000	0.002292
3645.500000	0.002299
3645.250000	0.002321
3645.000000	0.002352
3644.750000	0.002384
3644.500000	0.002410
3644.250000	0.002427
3644.000000	0.002435
3643.750000	0.002435
3643.500000	0.002431
3643.250000	0.002428
3643.000000	0.002428
3642.750000	0.002431
3642.500000	0.002435

3642.250000	0.002438
3642.000000	0.002438
3641.750000	0.002432
3641.500000	0.002421
3641.250000	0.002407
3641.000000	0.002392
3640.750000	0.002378
3640.500000	0.002366
3640.250000	0.002356
3640.000000	0.002349
3639.750000	0.002344
3639.500000	0.002343
3639.250000	0.002345
3639.000000	0.002352
3638.750000	0.002363
3638.500000	0.002378
3638.250000	0.002393
3638.000000	0.002405
3637.750000	0.002409
3637.500000	0.002405
3637.250000	0.002393
3637.000000	0.002376
3636.750000	0.002355
3636.500000	0.002336
3636.250000	0.002320
3636.000000	0.002308

3635.750000	0.002301
3635.500000	0.002299
3635.250000	0.002303
3635.000000	0.002314
3634.750000	0.002332
3634.500000	0.002357
3634.250000	0.002387
3634.000000	0.002415
3633.750000	0.002437
3633.500000	0.002450
3633.250000	0.002454
3633.000000	0.002453
3632.750000	0.002450
3632.500000	0.002445
3632.250000	0.002437
3632.000000	0.002422
3631.750000	0.002393
3631.500000	0.002345
3631.250000	0.002275
3631.000000	0.002181
3630.750000	0.002068
3630.500000	0.001948
3630.250000	0.001840
3630.000000	0.001770
3629.750000	0.001762
3629.500000	0.001827

3629.250000	0.001946
3629.000000	0.002080
3628.750000	0.002189
3628.500000	0.002259
3628.250000	0.002301
3628.000000	0.002338
3627.750000	0.002390
3627.500000	0.002465
3627.250000	0.002560
3627.000000	0.002664
3626.750000	0.002765
3626.500000	0.002851
3626.250000	0.002911
3626.000000	0.002941
3625.750000	0.002944
3625.500000	0.002924
3625.250000	0.002891
3625.000000	0.002852
3624.750000	0.002815
3624.500000	0.002782
3624.250000	0.002756
3624.000000	0.002734
3623.750000	0.002717
3623.500000	0.002703
3623.250000	0.002690
3623.000000	0.002676

3622.750000	0.002660
3622.500000	0.002642
3622.250000	0.002620
3622.000000	0.002596
3621.750000	0.002569
3621.500000	0.002542
3621.250000	0.002521
3621.000000	0.002508
3620.750000	0.002509
3620.500000	0.002525
3620.250000	0.002553
3620.000000	0.002587
3619.750000	0.002619
3619.500000	0.002644
3619.250000	0.002663
3619.000000	0.002682
3618.750000	0.002710
3618.500000	0.002754
3618.250000	0.002812
3618.000000	0.002877
3617.750000	0.002934
3617.500000	0.002973
3617.250000	0.002989
3617.000000	0.002984
3616.750000	0.002967
3616.500000	0.002948

3616.250000	0.002939
3616.000000	0.002944
3615.750000	0.002961
3615.500000	0.002982
3615.250000	0.002992
3615.000000	0.002980
3614.750000	0.002941
3614.500000	0.002878
3614.250000	0.002806
3614.000000	0.002745
3613.750000	0.002716
3613.500000	0.002734
3613.250000	0.002799
3613.000000	0.002898
3612.750000	0.003003
3612.500000	0.003086
3612.250000	0.003127
3612.000000	0.003121
3611.750000	0.003076
3611.500000	0.003010
3611.250000	0.002937
3611.000000	0.002874
3610.750000	0.002831
3610.500000	0.002817
3610.250000	0.002835
3610.000000	0.002882

3609.750000	0.002944
3609.500000	0.003006
3609.250000	0.003051
3609.000000	0.003072
3608.750000	0.003072
3608.500000	0.003061
3608.250000	0.003055
3608.000000	0.003062
3607.750000	0.003088
3607.500000	0.003128
3607.250000	0.003172
3607.000000	0.003208
3606.750000	0.003226
3606.500000	0.003224
3606.250000	0.003207
3606.000000	0.003185
3605.750000	0.003166
3605.500000	0.003159
3605.250000	0.003163
3605.000000	0.003177
3604.750000	0.003195
3604.500000	0.003213
3604.250000	0.003226
3604.000000	0.003232
3603.750000	0.003231
3603.500000	0.003222

3603.250000	0.003205
3603.000000	0.003183
3602.750000	0.003156
3602.500000	0.003128
3602.250000	0.003103
3602.000000	0.003086
3601.750000	0.003077
3601.500000	0.003078
3601.250000	0.003087
3601.000000	0.003099
3600.750000	0.003111
3600.500000	0.003122
3600.250000	0.003131
3600.000000	0.003141
3599.750000	0.003155
3599.500000	0.003176
3599.250000	0.003204
3599.000000	0.003236
3598.750000	0.003267
3598.500000	0.003289
3598.250000	0.003299
3598.000000	0.003294
3597.750000	0.003274
3597.500000	0.003244
3597.250000	0.003210
3597.000000	0.003179

3596.750000	0.003156
3596.500000	0.003145
3596.250000	0.003146
3596.000000	0.003155
3595.750000	0.003165
3595.500000	0.003168
3595.250000	0.003162
3595.000000	0.003146
3594.750000	0.003129
3594.500000	0.003119
3594.250000	0.003126
3594.000000	0.003155
3593.750000	0.003207
3593.500000	0.003272
3593.250000	0.003339
3593.000000	0.003394
3592.750000	0.003428
3592.500000	0.003437
3592.250000	0.003425
3592.000000	0.003399
3591.750000	0.003365
3591.500000	0.003329
3591.250000	0.003293
3591.000000	0.003256
3590.750000	0.003216
3590.500000	0.003173

3590.250000	0.003127
3590.000000	0.003084
3589.750000	0.003048
3589.500000	0.003025
3589.250000	0.003016
3589.000000	0.003017
3588.750000	0.003022
3588.500000	0.003025
3588.250000	0.003026
3588.000000	0.003033
3587.750000	0.003056
3587.500000	0.003104
3587.250000	0.003180
3587.000000	0.003272
3586.750000	0.003366
3586.500000	0.003444
3586.250000	0.003493
3586.000000	0.003513
3585.750000	0.003511
3585.500000	0.003499
3585.250000	0.003488
3585.000000	0.003485
3584.750000	0.003492
3584.500000	0.003509
3584.250000	0.003529
3584.000000	0.003548

3583.750000	0.003561
3583.500000	0.003565
3583.250000	0.003558
3583.000000	0.003542
3582.750000	0.003519
3582.500000	0.003491
3582.250000	0.003463
3582.000000	0.003438
3581.750000	0.003420
3581.500000	0.003411
3581.250000	0.003411
3581.000000	0.003419
3580.750000	0.003432
3580.500000	0.003444
3580.250000	0.003453
3580.000000	0.003457
3579.750000	0.003454
3579.500000	0.003448
3579.250000	0.003442
3579.000000	0.003438
3578.750000	0.003441
3578.500000	0.003450
3578.250000	0.003467
3578.000000	0.003488
3577.750000	0.003511
3577.500000	0.003532

3577.250000	0.003548
3577.000000	0.003555
3576.750000	0.003556
3576.500000	0.003551
3576.250000	0.003544
3576.000000	0.003539
3575.750000	0.003538
3575.500000	0.003544
3575.250000	0.003554
3575.000000	0.003567
3574.750000	0.003577
3574.500000	0.003584
3574.250000	0.003584
3574.000000	0.003577
3573.750000	0.003567
3573.500000	0.003555
3573.250000	0.003544
3573.000000	0.003536
3572.750000	0.003531
3572.500000	0.003531
3572.250000	0.003535
3572.000000	0.003541
3571.750000	0.003549
3571.500000	0.003555
3571.250000	0.003556
3571.000000	0.003550

3570.750000	0.003533
3570.500000	0.003505
3570.250000	0.003468
3570.000000	0.003424
3569.750000	0.003381
3569.500000	0.003343
3569.250000	0.003318
3569.000000	0.003309
3568.750000	0.003317
3568.500000	0.003338
3568.250000	0.003368
3568.000000	0.003402
3567.750000	0.003441
3567.500000	0.003488
3567.250000	0.003549
3567.000000	0.003625
3566.750000	0.003713
3566.500000	0.003802
3566.250000	0.003879
3566.000000	0.003933
3565.750000	0.003960
3565.500000	0.003963
3565.250000	0.003952
3565.000000	0.003936
3564.750000	0.003924
3564.500000	0.003920

3564.250000	0.003924
3564.000000	0.003932
3563.750000	0.003937
3563.500000	0.003934
3563.250000	0.003919
3563.000000	0.003893
3562.750000	0.003858
3562.500000	0.003820
3562.250000	0.003783
3562.000000	0.003752
3561.750000	0.003730
3561.500000	0.003718
3561.250000	0.003716
3561.000000	0.003724
3560.750000	0.003737
3560.500000	0.003751
3560.250000	0.003764
3560.000000	0.003772
3559.750000	0.003776
3559.500000	0.003775
3559.250000	0.003774
3559.000000	0.003776
3558.750000	0.003782
3558.500000	0.003794
3558.250000	0.003810
3558.000000	0.003827

3557.750000	0.003842
3557.500000	0.003852
3557.250000	0.003855
3557.000000	0.003850
3556.750000	0.003837
3556.500000	0.003819
3556.250000	0.003799
3556.000000	0.003778
3555.750000	0.003760
3555.500000	0.003745
3555.250000	0.003734
3555.000000	0.003728
3554.750000	0.003726
3554.500000	0.003728
3554.250000	0.003734
3554.000000	0.003743
3553.750000	0.003755
3553.500000	0.003771
3553.250000	0.003791
3553.000000	0.003814
3552.750000	0.003841
3552.500000	0.003869
3552.250000	0.003895
3552.000000	0.003917
3551.750000	0.003931
3551.500000	0.003938

3551.250000	0.003939
3551.000000	0.003935
3550.750000	0.003928
3550.500000	0.003922
3550.250000	0.003915
3550.000000	0.003907
3549.750000	0.003898
3549.500000	0.003885
3549.250000	0.003869
3549.000000	0.003852
3548.750000	0.003836
3548.500000	0.003826
3548.250000	0.003824
3548.000000	0.003830
3547.750000	0.003843
3547.500000	0.003858
3547.250000	0.003872
3547.000000	0.003883
3546.750000	0.003891
3546.500000	0.003900
3546.250000	0.003913
3546.000000	0.003934
3545.750000	0.003963
3545.500000	0.004000
3545.250000	0.004038
3545.000000	0.004072

3544.750000	0.004097
3544.500000	0.004112
3544.250000	0.004118
3544.000000	0.004117
3543.750000	0.004113
3543.500000	0.004109
3543.250000	0.004107
3543.000000	0.004106
3542.750000	0.004105
3542.500000	0.004102
3542.250000	0.004097
3542.000000	0.004090
3541.750000	0.004083
3541.500000	0.004076
3541.250000	0.004071
3541.000000	0.004067
3540.750000	0.004065
3540.500000	0.004063
3540.250000	0.004062
3540.000000	0.004061
3539.750000	0.004058
3539.500000	0.004056
3539.250000	0.004052
3539.000000	0.004046
3538.750000	0.004039
3538.500000	0.004029

3538.250000	0.004019
3538.000000	0.004009
3537.750000	0.004002
3537.500000	0.004001
3537.250000	0.004007
3537.000000	0.004021
3536.750000	0.004042
3536.500000	0.004066
3536.250000	0.004090
3536.000000	0.004112
3535.750000	0.004128
3535.500000	0.004140
3535.250000	0.004148
3535.000000	0.004154
3534.750000	0.004160
3534.500000	0.004166
3534.250000	0.004172
3534.000000	0.004176
3533.750000	0.004177
3533.500000	0.004174
3533.250000	0.004166
3533.000000	0.004156
3532.750000	0.004147
3532.500000	0.004140
3532.250000	0.004139
3532.000000	0.004144

3531.750000	0.004152
3531.500000	0.004161
3531.250000	0.004167
3531.000000	0.004166
3530.750000	0.004158
3530.500000	0.004144
3530.250000	0.004127
3530.000000	0.004112
3529.750000	0.004103
3529.500000	0.004102
3529.250000	0.004111
3529.000000	0.004126
3528.750000	0.004145
3528.500000	0.004164
3528.250000	0.004181
3528.000000	0.004196
3527.750000	0.004210
3527.500000	0.004223
3527.250000	0.004238
3527.000000	0.004256
3526.750000	0.004276
3526.500000	0.004297
3526.250000	0.004316
3526.000000	0.004331
3525.750000	0.004341
3525.500000	0.004345

3525.250000	0.004343
3525.000000	0.004336
3524.750000	0.004329
3524.500000	0.004323
3524.250000	0.004321
3524.000000	0.004325
3523.750000	0.004335
3523.500000	0.004350
3523.250000	0.004367
3523.000000	0.004383
3522.750000	0.004396
3522.500000	0.004404
3522.250000	0.004407
3522.000000	0.004405
3521.750000	0.004400
3521.500000	0.004394
3521.250000	0.004386
3521.000000	0.004378
3520.750000	0.004370
3520.500000	0.004363
3520.250000	0.004357
3520.000000	0.004352
3519.750000	0.004348
3519.500000	0.004346
3519.250000	0.004347
3519.000000	0.004350

3518.750000	0.004355
3518.500000	0.004363
3518.250000	0.004372
3518.000000	0.004382
3517.750000	0.004391
3517.500000	0.004398
3517.250000	0.004402
3517.000000	0.004402
3516.750000	0.004398
3516.500000	0.004392
3516.250000	0.004385
3516.000000	0.004378
3515.750000	0.004373
3515.500000	0.004371
3515.250000	0.004370
3515.000000	0.004370
3514.750000	0.004368
3514.500000	0.004365
3514.250000	0.004359
3514.000000	0.004350
3513.750000	0.004340
3513.500000	0.004333
3513.250000	0.004329
3513.000000	0.004332
3512.750000	0.004340
3512.500000	0.004353

3512.250000	0.004367
3512.000000	0.004377
3511.750000	0.004382
3511.500000	0.004381
3511.250000	0.004374
3511.000000	0.004365
3510.750000	0.004360
3510.500000	0.004365
3510.250000	0.004382
3510.000000	0.004413
3509.750000	0.004455
3509.500000	0.004500
3509.250000	0.004543
3509.000000	0.004576
3508.750000	0.004597
3508.500000	0.004605
3508.250000	0.004602
3508.000000	0.004591
3507.750000	0.004576
3507.500000	0.004561
3507.250000	0.004547
3507.000000	0.004537
3506.750000	0.004529
3506.500000	0.004522
3506.250000	0.004516
3506.000000	0.004508

3505.750000	0.004498
3505.500000	0.004484
3505.250000	0.004466
3505.000000	0.004446
3504.750000	0.004427
3504.500000	0.004411
3504.250000	0.004403
3504.000000	0.004406
3503.750000	0.004421
3503.500000	0.004447
3503.250000	0.004482
3503.000000	0.004522
3502.750000	0.004562
3502.500000	0.004599
3502.250000	0.004630
3502.000000	0.004653
3501.750000	0.004669
3501.500000	0.004677
3501.250000	0.004678
3501.000000	0.004674
3500.750000	0.004667
3500.500000	0.004658
3500.250000	0.004650
3500.000000	0.004643
3499.750000	0.004639
3499.500000	0.004635

3499.250000	0.004632
3499.000000	0.004626
3498.750000	0.004618
3498.500000	0.004608
3498.250000	0.004597
3498.000000	0.004588
3497.750000	0.004583
3497.500000	0.004585
3497.250000	0.004592
3497.000000	0.004604
3496.750000	0.004616
3496.500000	0.004628
3496.250000	0.004637
3496.000000	0.004641
3495.750000	0.004643
3495.500000	0.004643
3495.250000	0.004643
3495.000000	0.004641
3494.750000	0.004639
3494.500000	0.004636
3494.250000	0.004630
3494.000000	0.004623
3493.750000	0.004616
3493.500000	0.004609
3493.250000	0.004604
3493.000000	0.004602

3492.750000	0.004605
3492.500000	0.004612
3492.250000	0.004624
3492.000000	0.004641
3491.750000	0.004662
3491.500000	0.004685
3491.250000	0.004709
3491.000000	0.004729
3490.750000	0.004744
3490.500000	0.004750
3490.250000	0.004748
3490.000000	0.004737
3489.750000	0.004721
3489.500000	0.004703
3489.250000	0.004687
3489.000000	0.004676
3488.750000	0.004671
3488.500000	0.004671
3488.250000	0.004675
3488.000000	0.004680
3487.750000	0.004685
3487.500000	0.004688
3487.250000	0.004692
3487.000000	0.004697
3486.750000	0.004704
3486.500000	0.004713

3486.250000	0.004725
3486.000000	0.004737
3485.750000	0.004750
3485.500000	0.004762
3485.250000	0.004772
3485.000000	0.004779
3484.750000	0.004782
3484.500000	0.004782
3484.250000	0.004778
3484.000000	0.004771
3483.750000	0.004763
3483.500000	0.004757
3483.250000	0.004754
3483.000000	0.004757
3482.750000	0.004766
3482.500000	0.004780
3482.250000	0.004797
3482.000000	0.004813
3481.750000	0.004826
3481.500000	0.004834
3481.250000	0.004837
3481.000000	0.004835
3480.750000	0.004832
3480.500000	0.004829
3480.250000	0.004830
3480.000000	0.004834

3479.750000	0.004842
3479.500000	0.004855
3479.250000	0.004869
3479.000000	0.004884
3478.750000	0.004898
3478.500000	0.004909
3478.250000	0.004916
3478.000000	0.004918
3477.750000	0.004915
3477.500000	0.004908
3477.250000	0.004901
3477.000000	0.004894
3476.750000	0.004890
3476.500000	0.004892
3476.250000	0.004900
3476.000000	0.004912
3475.750000	0.004927
3475.500000	0.004943
3475.250000	0.004958
3475.000000	0.004969
3474.750000	0.004977
3474.500000	0.004983
3474.250000	0.004988
3474.000000	0.004993
3473.750000	0.004999
3473.500000	0.005005

3473.250000	0.005012
3473.000000	0.005018
3472.750000	0.005022
3472.500000	0.005023
3472.250000	0.005023
3472.000000	0.005021
3471.750000	0.005019
3471.500000	0.005019
3471.250000	0.005020
3471.000000	0.005023
3470.750000	0.005027
3470.500000	0.005033
3470.250000	0.005040
3470.000000	0.005047
3469.750000	0.005054
3469.500000	0.005060
3469.250000	0.005066
3469.000000	0.005073
3468.750000	0.005079
3468.500000	0.005086
3468.250000	0.005093
3468.000000	0.005102
3467.750000	0.005112
3467.500000	0.005125
3467.250000	0.005140
3467.000000	0.005159

3466.750000	0.005182
3466.500000	0.005206
3466.250000	0.005230
3466.000000	0.005252
3465.750000	0.005271
3465.500000	0.005285
3465.250000	0.005293
3465.000000	0.005297
3464.750000	0.005298
3464.500000	0.005297
3464.250000	0.005298
3464.000000	0.005302
3463.750000	0.005309
3463.500000	0.005319
3463.250000	0.005331
3463.000000	0.005344
3462.750000	0.005357
3462.500000	0.005369
3462.250000	0.005378
3462.000000	0.005386
3461.750000	0.005393
3461.500000	0.005400
3461.250000	0.005408
3461.000000	0.005417
3460.750000	0.005427
3460.500000	0.005438

3460.250000	0.005450
3460.000000	0.005462
3459.750000	0.005473
3459.500000	0.005482
3459.250000	0.005489
3459.000000	0.005494
3458.750000	0.005497
3458.500000	0.005500
3458.250000	0.005501
3458.000000	0.005502
3457.750000	0.005502
3457.500000	0.005501
3457.250000	0.005500
3457.000000	0.005498
3456.750000	0.005497
3456.500000	0.005498
3456.250000	0.005504
3456.000000	0.005515
3455.750000	0.005531
3455.500000	0.005552
3455.250000	0.005577
3455.000000	0.005603
3454.750000	0.005626
3454.500000	0.005647
3454.250000	0.005661
3454.000000	0.005669

3453.750000	0.005672
3453.500000	0.005670
3453.250000	0.005667
3453.000000	0.005665
3452.750000	0.005667
3452.500000	0.005675
3452.250000	0.005689
3452.000000	0.005707
3451.750000	0.005727
3451.500000	0.005745
3451.250000	0.005759
3451.000000	0.005765
3450.750000	0.005764
3450.500000	0.005756
3450.250000	0.005743
3450.000000	0.005727
3449.750000	0.005711
3449.500000	0.005697
3449.250000	0.005686
3449.000000	0.005679
3448.750000	0.005679
3448.500000	0.005687
3448.250000	0.005705
3448.000000	0.005733
3447.750000	0.005769
3447.500000	0.005809

3447.250000	0.005849
3447.000000	0.005884
3446.750000	0.005913
3446.500000	0.005935
3446.250000	0.005952
3446.000000	0.005968
3445.750000	0.005984
3445.500000	0.006001
3445.250000	0.006017
3445.000000	0.006030
3444.750000	0.006037
3444.500000	0.006035
3444.250000	0.006025
3444.000000	0.006011
3443.750000	0.005996
3443.500000	0.005985
3443.250000	0.005980
3443.000000	0.005984
3442.750000	0.005993
3442.500000	0.006007
3442.250000	0.006021
3442.000000	0.006034
3441.750000	0.006046
3441.500000	0.006055
3441.250000	0.006064
3441.000000	0.006072

3440.750000	0.006081
3440.500000	0.006090
3440.250000	0.006100
3440.000000	0.006109
3439.750000	0.006117
3439.500000	0.006125
3439.250000	0.006134
3439.000000	0.006142
3438.750000	0.006152
3438.500000	0.006162
3438.250000	0.006171
3438.000000	0.006178
3437.750000	0.006182
3437.500000	0.006182
3437.250000	0.006179
3437.000000	0.006174
3436.750000	0.006167
3436.500000	0.006160
3436.250000	0.006154
3436.000000	0.006150
3435.750000	0.006148
3435.500000	0.006147
3435.250000	0.006150
3435.000000	0.006155
3434.750000	0.006165
3434.500000	0.006180

3434.250000	0.006199
3434.000000	0.006223
3433.750000	0.006249
3433.500000	0.006275
3433.250000	0.006299
3433.000000	0.006319
3432.750000	0.006335
3432.500000	0.006346
3432.250000	0.006355
3432.000000	0.006365
3431.750000	0.006375
3431.500000	0.006389
3431.250000	0.006405
3431.000000	0.006422
3430.750000	0.006439
3430.500000	0.006454
3430.250000	0.006468
3430.000000	0.006480
3429.750000	0.006492
3429.500000	0.006504
3429.250000	0.006516
3429.000000	0.006529
3428.750000	0.006541
3428.500000	0.006554
3428.250000	0.006568
3428.000000	0.006583

3427.750000	0.006601
3427.500000	0.006622
3427.250000	0.006646
3427.000000	0.006672
3426.750000	0.006698
3426.500000	0.006722
3426.250000	0.006742
3426.000000	0.006759
3425.750000	0.006772
3425.500000	0.006784
3425.250000	0.006796
3425.000000	0.006808
3424.750000	0.006821
3424.500000	0.006833
3424.250000	0.006843
3424.000000	0.006849
3423.750000	0.006851
3423.500000	0.006850
3423.250000	0.006845
3423.000000	0.006840
3422.750000	0.006834
3422.500000	0.006831
3422.250000	0.006830
3422.000000	0.006833
3421.750000	0.006841
3421.500000	0.006853

3421.250000	0.006873
3421.000000	0.006900
3420.750000	0.006934
3420.500000	0.006975
3420.250000	0.007019
3420.000000	0.007064
3419.750000	0.007106
3419.500000	0.007141
3419.250000	0.007168
3419.000000	0.007186
3418.750000	0.007195
3418.500000	0.007197
3418.250000	0.007195
3418.000000	0.007192
3417.750000	0.007190
3417.500000	0.007192
3417.250000	0.007197
3417.000000	0.007207
3416.750000	0.007221
3416.500000	0.007236
3416.250000	0.007254
3416.000000	0.007272
3415.750000	0.007289
3415.500000	0.007304
3415.250000	0.007317
3415.000000	0.007325

3414.750000	0.007329
3414.500000	0.007329
3414.250000	0.007327
3414.000000	0.007325
3413.750000	0.007326
3413.500000	0.007332
3413.250000	0.007345
3413.000000	0.007365
3412.750000	0.007391
3412.500000	0.007421
3412.250000	0.007453
3412.000000	0.007485
3411.750000	0.007514
3411.500000	0.007538
3411.250000	0.007558
3411.000000	0.007572
3410.750000	0.007583
3410.500000	0.007591
3410.250000	0.007599
3410.000000	0.007609
3409.750000	0.007621
3409.500000	0.007637
3409.250000	0.007657
3409.000000	0.007680
3408.750000	0.007706
3408.500000	0.007733

3408.250000	0.007761
3408.000000	0.007791
3407.750000	0.007822
3407.500000	0.007854
3407.250000	0.007885
3407.000000	0.007913
3406.750000	0.007936
3406.500000	0.007953
3406.250000	0.007962
3406.000000	0.007964
3405.750000	0.007963
3405.500000	0.007961
3405.250000	0.007961
3405.000000	0.007965
3404.750000	0.007972
3404.500000	0.007983
3404.250000	0.007993
3404.000000	0.008002
3403.750000	0.008009
3403.500000	0.008015
3403.250000	0.008021
3403.000000	0.008029
3402.750000	0.008042
3402.500000	0.008059
3402.250000	0.008080
3402.000000	0.008105

3401.750000	0.008131
3401.500000	0.008158
3401.250000	0.008184
3401.000000	0.008209
3400.750000	0.008232
3400.500000	0.008254
3400.250000	0.008273
3400.000000	0.008291
3399.750000	0.008307
3399.500000	0.008323
3399.250000	0.008340
3399.000000	0.008358
3398.750000	0.008381
3398.500000	0.008407
3398.250000	0.008436
3398.000000	0.008469
3397.750000	0.008502
3397.500000	0.008533
3397.250000	0.008562
3397.000000	0.008586
3396.750000	0.008604
3396.500000	0.008617
3396.250000	0.008626
3396.000000	0.008631
3395.750000	0.008632
3395.500000	0.008631

3395.250000	0.008629
3395.000000	0.008628
3394.750000	0.008627
3394.500000	0.008629
3394.250000	0.008636
3394.000000	0.008648
3393.750000	0.008664
3393.500000	0.008683
3393.250000	0.008704
3393.000000	0.008724
3392.750000	0.008741
3392.500000	0.008753
3392.250000	0.008762
3392.000000	0.008769
3391.750000	0.008777
3391.500000	0.008788
3391.250000	0.008802
3391.000000	0.008819
3390.750000	0.008837
3390.500000	0.008855
3390.250000	0.008871
3390.000000	0.008884
3389.750000	0.008894
3389.500000	0.008902
3389.250000	0.008910
3389.000000	0.008919

3388.750000	0.008930
3388.500000	0.008941
3388.250000	0.008952
3388.000000	0.008963
3387.750000	0.008973
3387.500000	0.008984
3387.250000	0.008994
3387.000000	0.009005
3386.750000	0.009017
3386.500000	0.009029
3386.250000	0.009041
3386.000000	0.009050
3385.750000	0.009057
3385.500000	0.009062
3385.250000	0.009066
3385.000000	0.009069
3384.750000	0.009072
3384.500000	0.009075
3384.250000	0.009079
3384.000000	0.009082
3383.750000	0.009084
3383.500000	0.009085
3383.250000	0.009083
3383.000000	0.009080
3382.750000	0.009076
3382.500000	0.009072

3382.250000	0.009067
3382.000000	0.009065
3381.750000	0.009064
3381.500000	0.009066
3381.250000	0.009071
3381.000000	0.009078
3380.750000	0.009084
3380.500000	0.009088
3380.250000	0.009089
3380.000000	0.009086
3379.750000	0.009081
3379.500000	0.009073
3379.250000	0.009066
3379.000000	0.009061
3378.750000	0.009059
3378.500000	0.009060
3378.250000	0.009063
3378.000000	0.009066
3377.750000	0.009068
3377.500000	0.009070
3377.250000	0.009072
3377.000000	0.009074
3376.750000	0.009076
3376.500000	0.009079
3376.250000	0.009083
3376.000000	0.009087

3375.750000	0.009091
3375.500000	0.009097
3375.250000	0.009105
3375.000000	0.009116
3374.750000	0.009130
3374.500000	0.009146
3374.250000	0.009160
3374.000000	0.009170
3373.750000	0.009173
3373.500000	0.009166
3373.250000	0.009150
3373.000000	0.009127
3372.750000	0.009101
3372.500000	0.009073
3372.250000	0.009048
3372.000000	0.009027
3371.750000	0.009009
3371.500000	0.008994
3371.250000	0.008981
3371.000000	0.008970
3370.750000	0.008961
3370.500000	0.008953
3370.250000	0.008948
3370.000000	0.008947
3369.750000	0.008948
3369.500000	0.008953

3369.250000	0.008960
3369.000000	0.008968
3368.750000	0.008976
3368.500000	0.008983
3368.250000	0.008986
3368.000000	0.008986
3367.750000	0.008981
3367.500000	0.008972
3367.250000	0.008960
3367.000000	0.008947
3366.750000	0.008935
3366.500000	0.008926
3366.250000	0.008921
3366.000000	0.008920
3365.750000	0.008925
3365.500000	0.008934
3365.250000	0.008944
3365.000000	0.008954
3364.750000	0.008962
3364.500000	0.008966
3364.250000	0.008966
3364.000000	0.008961
3363.750000	0.008952
3363.500000	0.008942
3363.250000	0.008931
3363.000000	0.008922

3362.750000	0.008915
3362.500000	0.008912
3362.250000	0.008911
3362.000000	0.008913
3361.750000	0.008915
3361.500000	0.008917
3361.250000	0.008918
3361.000000	0.008916
3360.750000	0.008911
3360.500000	0.008903
3360.250000	0.008892
3360.000000	0.008879
3359.750000	0.008866
3359.500000	0.008855
3359.250000	0.008847
3359.000000	0.008844
3358.750000	0.008846
3358.500000	0.008853
3358.250000	0.008863
3358.000000	0.008874
3357.750000	0.008884
3357.500000	0.008890
3357.250000	0.008891
3357.000000	0.008887
3356.750000	0.008876
3356.500000	0.008860

3356.250000	0.008839
3356.000000	0.008816
3355.750000	0.008793
3355.500000	0.008771
3355.250000	0.008754
3355.000000	0.008741
3354.750000	0.008733
3354.500000	0.008727
3354.250000	0.008722
3354.000000	0.008714
3353.750000	0.008701
3353.500000	0.008683
3353.250000	0.008661
3353.000000	0.008638
3352.750000	0.008617
3352.500000	0.008600
3352.250000	0.008590
3352.000000	0.008586
3351.750000	0.008587
3351.500000	0.008590
3351.250000	0.008594
3351.000000	0.008595
3350.750000	0.008594
3350.500000	0.008591
3350.250000	0.008587
3350.000000	0.008583

3349.750000	0.008582
3349.500000	0.008582
3349.250000	0.008584
3349.000000	0.008588
3348.750000	0.008593
3348.500000	0.008598
3348.250000	0.008604
3348.000000	0.008610
3347.750000	0.008615
3347.500000	0.008619
3347.250000	0.008621
3347.000000	0.008620
3346.750000	0.008615
3346.500000	0.008605
3346.250000	0.008593
3346.000000	0.008579
3345.750000	0.008565
3345.500000	0.008553
3345.250000	0.008544
3345.000000	0.008540
3344.750000	0.008538
3344.500000	0.008539
3344.250000	0.008539
3344.000000	0.008538
3343.750000	0.008533
3343.500000	0.008524

3343.250000	0.008513
3343.000000	0.008500
3342.750000	0.008487
3342.500000	0.008476
3342.250000	0.008467
3342.000000	0.008461
3341.750000	0.008458
3341.500000	0.008456
3341.250000	0.008455
3341.000000	0.008456
3340.750000	0.008459
3340.500000	0.008463
3340.250000	0.008469
3340.000000	0.008474
3339.750000	0.008477
3339.500000	0.008476
3339.250000	0.008470
3339.000000	0.008460
3338.750000	0.008445
3338.500000	0.008429
3338.250000	0.008414
3338.000000	0.008402
3337.750000	0.008393
3337.500000	0.008388
3337.250000	0.008383
3337.000000	0.008377

3336.750000	0.008367
3336.500000	0.008356
3336.250000	0.008342
3336.000000	0.008331
3335.750000	0.008323
3335.500000	0.008320
3335.250000	0.008323
3335.000000	0.008328
3334.750000	0.008334
3334.500000	0.008335
3334.250000	0.008329
3334.000000	0.008315
3333.750000	0.008295
3333.500000	0.008269
3333.250000	0.008242
3333.000000	0.008215
3332.750000	0.008191
3332.500000	0.008172
3332.250000	0.008157
3332.000000	0.008146
3331.750000	0.008140
3331.500000	0.008137
3331.250000	0.008136
3331.000000	0.008137
3330.750000	0.008138
3330.500000	0.008139

3330.250000	0.008138
3330.000000	0.008134
3329.750000	0.008127
3329.500000	0.008116
3329.250000	0.008102
3329.000000	0.008086
3328.750000	0.008069
3328.500000	0.008053
3328.250000	0.008038
3328.000000	0.008026
3327.750000	0.008015
3327.500000	0.008005
3327.250000	0.007996
3327.000000	0.007987
3326.750000	0.007976
3326.500000	0.007965
3326.250000	0.007953
3326.000000	0.007942
3325.750000	0.007933
3325.500000	0.007928
3325.250000	0.007926
3325.000000	0.007926
3324.750000	0.007929
3324.500000	0.007930
3324.250000	0.007931
3324.000000	0.007928

3323.750000	0.007922
3323.500000	0.007914
3323.250000	0.007903
3323.000000	0.007891
3322.750000	0.007877
3322.500000	0.007860
3322.250000	0.007840
3322.000000	0.007816
3321.750000	0.007789
3321.500000	0.007760
3321.250000	0.007733
3321.000000	0.007711
3320.750000	0.007695
3320.500000	0.007685
3320.250000	0.007683
3320.000000	0.007683
3319.750000	0.007685
3319.500000	0.007684
3319.250000	0.007678
3319.000000	0.007667
3318.750000	0.007651
3318.500000	0.007633
3318.250000	0.007613
3318.000000	0.007595
3317.750000	0.007578
3317.500000	0.007565

3317.250000	0.007554
3317.000000	0.007545
3316.750000	0.007537
3316.500000	0.007529
3316.250000	0.007520
3316.000000	0.007509
3315.750000	0.007497
3315.500000	0.007484
3315.250000	0.007470
3315.000000	0.007457
3314.750000	0.007445
3314.500000	0.007434
3314.250000	0.007423
3314.000000	0.007414
3313.750000	0.007405
3313.500000	0.007396
3313.250000	0.007388
3313.000000	0.007380
3312.750000	0.007371
3312.500000	0.007359
3312.250000	0.007345
3312.000000	0.007326
3311.750000	0.007302
3311.500000	0.007273
3311.250000	0.007242
3311.000000	0.007211

3310.750000	0.007182
3310.500000	0.007159
3310.250000	0.007143
3310.000000	0.007134
3309.750000	0.007131
3309.500000	0.007131
3309.250000	0.007131
3309.000000	0.007130
3308.750000	0.007125
3308.500000	0.007116
3308.250000	0.007104
3308.000000	0.007089
3307.750000	0.007074
3307.500000	0.007058
3307.250000	0.007041
3307.000000	0.007025
3306.750000	0.007007
3306.500000	0.006989
3306.250000	0.006970
3306.000000	0.006951
3305.750000	0.006932
3305.500000	0.006916
3305.250000	0.006901
3305.000000	0.006889
3304.750000	0.006879
3304.500000	0.006870

3304.250000	0.006862
3304.000000	0.006855
3303.750000	0.006849
3303.500000	0.006844
3303.250000	0.006840
3303.000000	0.006837
3302.750000	0.006834
3302.500000	0.006829
3302.250000	0.006821
3302.000000	0.006809
3301.750000	0.006793
3301.500000	0.006773
3301.250000	0.006751
3301.000000	0.006728
3300.750000	0.006706
3300.500000	0.006685
3300.250000	0.006666
3300.000000	0.006650
3299.750000	0.006634
3299.500000	0.006619
3299.250000	0.006606
3299.000000	0.006593
3298.750000	0.006582
3298.500000	0.006573
3298.250000	0.006566
3298.000000	0.006561

3297.750000	0.006556
3297.500000	0.006550
3297.250000	0.006543
3297.000000	0.006533
3296.750000	0.006520
3296.500000	0.006505
3296.250000	0.006489
3296.000000	0.006472
3295.750000	0.006457
3295.500000	0.006444
3295.250000	0.006435
3295.000000	0.006428
3294.750000	0.006424
3294.500000	0.006420
3294.250000	0.006415
3294.000000	0.006407
3293.750000	0.006392
3293.500000	0.006370
3293.250000	0.006341
3293.000000	0.006306
3292.750000	0.006269
3292.500000	0.006234
3292.250000	0.006203
3292.000000	0.006182
3291.750000	0.006170
3291.500000	0.006168

3291.250000	0.006172
3291.000000	0.006181
3290.750000	0.006190
3290.500000	0.006196
3290.250000	0.006198
3290.000000	0.006195
3289.750000	0.006189
3289.500000	0.006180
3289.250000	0.006171
3289.000000	0.006162
3288.750000	0.006155
3288.500000	0.006148
3288.250000	0.006140
3288.000000	0.006131
3287.750000	0.006120
3287.500000	0.006108
3287.250000	0.006095
3287.000000	0.006084
3286.750000	0.006075
3286.500000	0.006069
3286.250000	0.006066
3286.000000	0.006066
3285.750000	0.006066
3285.500000	0.006064
3285.250000	0.006057
3285.000000	0.006045

3284.750000	0.006027
3284.500000	0.006003
3284.250000	0.005976
3284.000000	0.005948
3283.750000	0.005923
3283.500000	0.005904
3283.250000	0.005891
3283.000000	0.005884
3282.750000	0.005882
3282.500000	0.005881
3282.250000	0.005879
3282.000000	0.005872
3281.750000	0.005860
3281.500000	0.005843
3281.250000	0.005825
3281.000000	0.005807
3280.750000	0.005792
3280.500000	0.005781
3280.250000	0.005775
3280.000000	0.005773
3279.750000	0.005773
3279.500000	0.005774
3279.250000	0.005775
3279.000000	0.005775
3278.750000	0.005774
3278.500000	0.005770

3278.250000	0.005763
3278.000000	0.005752
3277.750000	0.005735
3277.500000	0.005712
3277.250000	0.005685
3277.000000	0.005655
3276.750000	0.005624
3276.500000	0.005595
3276.250000	0.005569
3276.000000	0.005547
3275.750000	0.005529
3275.500000	0.005516
3275.250000	0.005506
3275.000000	0.005500
3274.750000	0.005497
3274.500000	0.005496
3274.250000	0.005495
3274.000000	0.005494
3273.750000	0.005491
3273.500000	0.005485
3273.250000	0.005475
3273.000000	0.005461
3272.750000	0.005445
3272.500000	0.005428
3272.250000	0.005410
3272.000000	0.005393

3271.750000	0.005378
3271.500000	0.005366
3271.250000	0.005358
3271.000000	0.005355
3270.750000	0.005357
3270.500000	0.005365
3270.250000	0.005378
3270.000000	0.005393
3269.750000	0.005407
3269.500000	0.005417
3269.250000	0.005420
3269.000000	0.005415
3268.750000	0.005403
3268.500000	0.005384
3268.250000	0.005361
3268.000000	0.005336
3267.750000	0.005312
3267.500000	0.005291
3267.250000	0.005273
3267.000000	0.005260
3266.750000	0.005251
3266.500000	0.005247
3266.250000	0.005245
3266.000000	0.005245
3265.750000	0.005245
3265.500000	0.005243

3265.250000	0.005237
3265.000000	0.005228
3264.750000	0.005217
3264.500000	0.005205
3264.250000	0.005193
3264.000000	0.005182
3263.750000	0.005171
3263.500000	0.005162
3263.250000	0.005153
3263.000000	0.005143
3262.750000	0.005132
3262.500000	0.005122
3262.250000	0.005113
3262.000000	0.005106
3261.750000	0.005100
3261.500000	0.005098
3261.250000	0.005098
3261.000000	0.005100
3260.750000	0.005105
3260.500000	0.005110
3260.250000	0.005116
3260.000000	0.005119
3259.750000	0.005119
3259.500000	0.005113
3259.250000	0.005101
3259.000000	0.005083

3258.750000	0.005061
3258.500000	0.005038
3258.250000	0.005017
3258.000000	0.005000
3257.750000	0.004988
3257.500000	0.004980
3257.250000	0.004975
3257.000000	0.004970
3256.750000	0.004962
3256.500000	0.004953
3256.250000	0.004942
3256.000000	0.004933
3255.750000	0.004926
3255.500000	0.004922
3255.250000	0.004923
3255.000000	0.004926
3254.750000	0.004929
3254.500000	0.004930
3254.250000	0.004928
3254.000000	0.004923
3253.750000	0.004915
3253.500000	0.004906
3253.250000	0.004897
3253.000000	0.004889
3252.750000	0.004883
3252.500000	0.004879

3252.250000	0.004877
3252.000000	0.004877
3251.750000	0.004878
3251.500000	0.004880
3251.250000	0.004880
3251.000000	0.004880
3250.750000	0.004877
3250.500000	0.004872
3250.250000	0.004863
3250.000000	0.004851
3249.750000	0.004837
3249.500000	0.004821
3249.250000	0.004804
3249.000000	0.004786
3248.750000	0.004768
3248.500000	0.004749
3248.250000	0.004731
3248.000000	0.004714
3247.750000	0.004700
3247.500000	0.004689
3247.250000	0.004683
3247.000000	0.004679
3246.750000	0.004679
3246.500000	0.004680
3246.250000	0.004683
3246.000000	0.004685

3245.750000	0.004687
3245.500000	0.004688
3245.250000	0.004688
3245.000000	0.004688
3244.750000	0.004686
3244.500000	0.004683
3244.250000	0.004679
3244.000000	0.004675
3243.750000	0.004671
3243.500000	0.004668
3243.250000	0.004666
3243.000000	0.004665
3242.750000	0.004665
3242.500000	0.004665
3242.250000	0.004665
3242.000000	0.004665
3241.750000	0.004664
3241.500000	0.004662
3241.250000	0.004660
3241.000000	0.004656
3240.750000	0.004651
3240.500000	0.004644
3240.250000	0.004637
3240.000000	0.004627
3239.750000	0.004617
3239.500000	0.004606

3239.250000	0.004594
3239.000000	0.004581
3238.750000	0.004567
3238.500000	0.004552
3238.250000	0.004536
3238.000000	0.004519
3237.750000	0.004503
3237.500000	0.004490
3237.250000	0.004479
3237.000000	0.004473
3236.750000	0.004471
3236.500000	0.004473
3236.250000	0.004476
3236.000000	0.004478
3235.750000	0.004478
3235.500000	0.004474
3235.250000	0.004467
3235.000000	0.004458
3234.750000	0.004450
3234.500000	0.004443
3234.250000	0.004441
3234.000000	0.004444
3233.750000	0.004452
3233.500000	0.004463
3233.250000	0.004475
3233.000000	0.004486

3232.750000	0.004493
3232.500000	0.004495
3232.250000	0.004492
3232.000000	0.004486
3231.750000	0.004477
3231.500000	0.004468
3231.250000	0.004461
3231.000000	0.004456
3230.750000	0.004454
3230.500000	0.004456
3230.250000	0.004459
3230.000000	0.004463
3229.750000	0.004466
3229.500000	0.004467
3229.250000	0.004465
3229.000000	0.004459
3228.750000	0.004449
3228.500000	0.004436
3228.250000	0.004421
3228.000000	0.004405
3227.750000	0.004390
3227.500000	0.004375
3227.250000	0.004363
3227.000000	0.004353
3226.750000	0.004346
3226.500000	0.004342

3226.250000	0.004341
3226.000000	0.004342
3225.750000	0.004345
3225.500000	0.004349
3225.250000	0.004354
3225.000000	0.004358
3224.750000	0.004360
3224.500000	0.004361
3224.250000	0.004359
3224.000000	0.004355
3223.750000	0.004350
3223.500000	0.004344
3223.250000	0.004339
3223.000000	0.004335
3222.750000	0.004330
3222.500000	0.004325
3222.250000	0.004319
3222.000000	0.004310
3221.750000	0.004299
3221.500000	0.004286
3221.250000	0.004274
3221.000000	0.004263
3220.750000	0.004256
3220.500000	0.004254
3220.250000	0.004257
3220.000000	0.004265

3219.750000	0.004275
3219.500000	0.004287
3219.250000	0.004296
3219.000000	0.004301
3218.750000	0.004300
3218.500000	0.004293
3218.250000	0.004282
3218.000000	0.004269
3217.750000	0.004254
3217.500000	0.004241
3217.250000	0.004230
3217.000000	0.004220
3216.750000	0.004212
3216.500000	0.004205
3216.250000	0.004199
3216.000000	0.004194
3215.750000	0.004192
3215.500000	0.004194
3215.250000	0.004201
3215.000000	0.004212
3214.750000	0.004225
3214.500000	0.004238
3214.250000	0.004249
3214.000000	0.004254
3213.750000	0.004255
3213.500000	0.004250

3213.250000	0.004243
3213.000000	0.004235
3212.750000	0.004228
3212.500000	0.004220
3212.250000	0.004211
3212.000000	0.004201
3211.750000	0.004189
3211.500000	0.004175
3211.250000	0.004161
3211.000000	0.004149
3210.750000	0.004140
3210.500000	0.004135
3210.250000	0.004136
3210.000000	0.004140
3209.750000	0.004146
3209.500000	0.004154
3209.250000	0.004161
3209.000000	0.004168
3208.750000	0.004174
3208.500000	0.004178
3208.250000	0.004180
3208.000000	0.004179
3207.750000	0.004176
3207.500000	0.004171
3207.250000	0.004166
3207.000000	0.004162

3206.750000	0.004160
3206.500000	0.004161
3206.250000	0.004164
3206.000000	0.004167
3205.750000	0.004170
3205.500000	0.004171
3205.250000	0.004169
3205.000000	0.004165
3204.750000	0.004161
3204.500000	0.004157
3204.250000	0.004154
3204.000000	0.004153
3203.750000	0.004154
3203.500000	0.004156
3203.250000	0.004158
3203.000000	0.004158
3202.750000	0.004158
3202.500000	0.004155
3202.250000	0.004152
3202.000000	0.004147
3201.750000	0.004142
3201.500000	0.004137
3201.250000	0.004134
3201.000000	0.004133
3200.750000	0.004133
3200.500000	0.004135

3200.250000	0.004135
3200.000000	0.004133
3199.750000	0.004128
3199.500000	0.004119
3199.250000	0.004106
3199.000000	0.004091
3198.750000	0.004076
3198.500000	0.004062
3198.250000	0.004050
3198.000000	0.004039
3197.750000	0.004029
3197.500000	0.004020
3197.250000	0.004012
3197.000000	0.004005
3196.750000	0.004001
3196.500000	0.004002
3196.250000	0.004007
3196.000000	0.004016
3195.750000	0.004026
3195.500000	0.004037
3195.250000	0.004046
3195.000000	0.004052
3194.750000	0.004055
3194.500000	0.004054
3194.250000	0.004052
3194.000000	0.004049

3193.750000	0.004045
3193.500000	0.004039
3193.250000	0.004033
3193.000000	0.004027
3192.750000	0.004022
3192.500000	0.004019
3192.250000	0.004018
3192.000000	0.004020
3191.750000	0.004024
3191.500000	0.004028
3191.250000	0.004031
3191.000000	0.004031
3190.750000	0.004029
3190.500000	0.004025
3190.250000	0.004022
3190.000000	0.004019
3189.750000	0.004017
3189.500000	0.004015
3189.250000	0.004011
3189.000000	0.004004
3188.750000	0.003993
3188.500000	0.003978
3188.250000	0.003962
3188.000000	0.003946
3187.750000	0.003932
3187.500000	0.003921

3187.250000	0.003914
3187.000000	0.003911
3186.750000	0.003910
3186.500000	0.003911
3186.250000	0.003912
3186.000000	0.003914
3185.750000	0.003915
3185.500000	0.003916
3185.250000	0.003915
3185.000000	0.003912
3184.750000	0.003909
3184.500000	0.003904
3184.250000	0.003900
3184.000000	0.003897
3183.750000	0.003895
3183.500000	0.003895
3183.250000	0.003895
3183.000000	0.003897
3182.750000	0.003897
3182.500000	0.003897
3182.250000	0.003896
3182.000000	0.003896
3181.750000	0.003895
3181.500000	0.003896
3181.250000	0.003897
3181.000000	0.003899

3180.750000	0.003901
3180.500000	0.003902
3180.250000	0.003903
3180.000000	0.003902
3179.750000	0.003902
3179.500000	0.003902
3179.250000	0.003903
3179.000000	0.003907
3178.750000	0.003912
3178.500000	0.003919
3178.250000	0.003925
3178.000000	0.003931
3177.750000	0.003933
3177.500000	0.003932
3177.250000	0.003925
3177.000000	0.003914
3176.750000	0.003900
3176.500000	0.003883
3176.250000	0.003866
3176.000000	0.003851
3175.750000	0.003839
3175.500000	0.003833
3175.250000	0.003832
3175.000000	0.003835
3174.750000	0.003842
3174.500000	0.003849

3174.250000	0.003855
3174.000000	0.003857
3173.750000	0.003855
3173.500000	0.003847
3173.250000	0.003834
3173.000000	0.003820
3172.750000	0.003804
3172.500000	0.003790
3172.250000	0.003780
3172.000000	0.003773
3171.750000	0.003769
3171.500000	0.003769
3171.250000	0.003769
3171.000000	0.003770
3170.750000	0.003771
3170.500000	0.003770
3170.250000	0.003769
3170.000000	0.003767
3169.750000	0.003764
3169.500000	0.003761
3169.250000	0.003757
3169.000000	0.003753
3168.750000	0.003747
3168.500000	0.003742
3168.250000	0.003735
3168.000000	0.003729

3167.750000	0.003722
3167.500000	0.003715
3167.250000	0.003707
3167.000000	0.003701
3166.750000	0.003694
3166.500000	0.003689
3166.250000	0.003686
3166.000000	0.003684
3165.750000	0.003685
3165.500000	0.003687
3165.250000	0.003689
3165.000000	0.003691
3164.750000	0.003691
3164.500000	0.003690
3164.250000	0.003686
3164.000000	0.003679
3163.750000	0.003671
3163.500000	0.003660
3163.250000	0.003650
3163.000000	0.003640
3162.750000	0.003632
3162.500000	0.003628
3162.250000	0.003627
3162.000000	0.003631
3161.750000	0.003637
3161.500000	0.003644

3161.250000	0.003651
3161.000000	0.003657
3160.750000	0.003660
3160.500000	0.003661
3160.250000	0.003660
3160.000000	0.003657
3159.750000	0.003652
3159.500000	0.003645
3159.250000	0.003637
3159.000000	0.003625
3158.750000	0.003611
3158.500000	0.003595
3158.250000	0.003581
3158.000000	0.003569
3157.750000	0.003562
3157.500000	0.003561
3157.250000	0.003566
3157.000000	0.003574
3156.750000	0.003583
3156.500000	0.003590
3156.250000	0.003593
3156.000000	0.003592
3155.750000	0.003587
3155.500000	0.003579
3155.250000	0.003569
3155.000000	0.003560

3154.750000	0.003553
3154.500000	0.003548
3154.250000	0.003546
3154.000000	0.003545
3153.750000	0.003546
3153.500000	0.003549
3153.250000	0.003553
3153.000000	0.003558
3152.750000	0.003564
3152.500000	0.003570
3152.250000	0.003576
3152.000000	0.003580
3151.750000	0.003580
3151.500000	0.003577
3151.250000	0.003568
3151.000000	0.003556
3150.750000	0.003541
3150.500000	0.003524
3150.250000	0.003510
3150.000000	0.003499
3149.750000	0.003494
3149.500000	0.003494
3149.250000	0.003499
3149.000000	0.003506
3148.750000	0.003515
3148.500000	0.003521

3148.250000	0.003524
3148.000000	0.003521
3147.750000	0.003513
3147.500000	0.003501
3147.250000	0.003487
3147.000000	0.003475
3146.750000	0.003466
3146.500000	0.003462
3146.250000	0.003461
3146.000000	0.003465
3145.750000	0.003469
3145.500000	0.003472
3145.250000	0.003471
3145.000000	0.003467
3144.750000	0.003460
3144.500000	0.003450
3144.250000	0.003439
3144.000000	0.003430
3143.750000	0.003422
3143.500000	0.003417
3143.250000	0.003415
3143.000000	0.003415
3142.750000	0.003416
3142.500000	0.003420
3142.250000	0.003425
3142.000000	0.003432

3141.750000	0.003439
3141.500000	0.003446
3141.250000	0.003451
3141.000000	0.003454
3140.750000	0.003454
3140.500000	0.003450
3140.250000	0.003444
3140.000000	0.003436
3139.750000	0.003428
3139.500000	0.003422
3139.250000	0.003417
3139.000000	0.003414
3138.750000	0.003414
3138.500000	0.003415
3138.250000	0.003417
3138.000000	0.003419
3137.750000	0.003421
3137.500000	0.003421
3137.250000	0.003419
3137.000000	0.003415
3136.750000	0.003410
3136.500000	0.003404
3136.250000	0.003399
3136.000000	0.003398
3135.750000	0.003401
3135.500000	0.003408

3135.250000	0.003420
3135.000000	0.003434
3134.750000	0.003447
3134.500000	0.003457
3134.250000	0.003461
3134.000000	0.003459
3133.750000	0.003452
3133.500000	0.003440
3133.250000	0.003427
3133.000000	0.003416
3132.750000	0.003408
3132.500000	0.003404
3132.250000	0.003403
3132.000000	0.003403
3131.750000	0.003403
3131.500000	0.003400
3131.250000	0.003393
3131.000000	0.003382
3130.750000	0.003369
3130.500000	0.003357
3130.250000	0.003347
3130.000000	0.003343
3129.750000	0.003345
3129.500000	0.003351
3129.250000	0.003361
3129.000000	0.003372

3128.750000	0.003380
3128.500000	0.003383
3128.250000	0.003382
3128.000000	0.003377
3127.750000	0.003370
3127.500000	0.003363
3127.250000	0.003359
3127.000000	0.003360
3126.750000	0.003364
3126.500000	0.003371
3126.250000	0.003379
3126.000000	0.003388
3125.750000	0.003394
3125.500000	0.003399
3125.250000	0.003401
3125.000000	0.003402
3124.750000	0.003402
3124.500000	0.003402
3124.250000	0.003402
3124.000000	0.003402
3123.750000	0.003401
3123.500000	0.003399
3123.250000	0.003396
3123.000000	0.003390
3122.750000	0.003383
3122.500000	0.003375

3122.250000	0.003366
3122.000000	0.003357
3121.750000	0.003349
3121.500000	0.003343
3121.250000	0.003338
3121.000000	0.003336
3120.750000	0.003335
3120.500000	0.003335
3120.250000	0.003336
3120.000000	0.003337
3119.750000	0.003338
3119.500000	0.003338
3119.250000	0.003337
3119.000000	0.003336
3118.750000	0.003335
3118.500000	0.003335
3118.250000	0.003336
3118.000000	0.003339
3117.750000	0.003342
3117.500000	0.003347
3117.250000	0.003351
3117.000000	0.003355
3116.750000	0.003358
3116.500000	0.003360
3116.250000	0.003362
3116.000000	0.003365

3115.750000	0.003369
3115.500000	0.003376
3115.250000	0.003385
3115.000000	0.003397
3114.750000	0.003410
3114.500000	0.003423
3114.250000	0.003435
3114.000000	0.003445
3113.750000	0.003452
3113.500000	0.003453
3113.250000	0.003451
3113.000000	0.003445
3112.750000	0.003436
3112.500000	0.003428
3112.250000	0.003423
3112.000000	0.003420
3111.750000	0.003423
3111.500000	0.003430
3111.250000	0.003441
3111.000000	0.003454
3110.750000	0.003466
3110.500000	0.003475
3110.250000	0.003480
3110.000000	0.003480
3109.750000	0.003474
3109.500000	0.003466

3109.250000	0.003455
3109.000000	0.003445
3108.750000	0.003438
3108.500000	0.003437
3108.250000	0.003442
3108.000000	0.003453
3107.750000	0.003469
3107.500000	0.003487
3107.250000	0.003505
3107.000000	0.003520
3106.750000	0.003530
3106.500000	0.003536
3106.250000	0.003536
3106.000000	0.003533
3105.750000	0.003527
3105.500000	0.003522
3105.250000	0.003516
3105.000000	0.003512
3104.750000	0.003509
3104.500000	0.003507
3104.250000	0.003505
3104.000000	0.003502
3103.750000	0.003500
3103.500000	0.003497
3103.250000	0.003495
3103.000000	0.003493

3102.750000	0.003493
3102.500000	0.003495
3102.250000	0.003499
3102.000000	0.003504
3101.750000	0.003510
3101.500000	0.003514
3101.250000	0.003517
3101.000000	0.003518
3100.750000	0.003519
3100.500000	0.003519
3100.250000	0.003523
3100.000000	0.003529
3099.750000	0.003539
3099.500000	0.003552
3099.250000	0.003564
3099.000000	0.003575
3098.750000	0.003580
3098.500000	0.003579
3098.250000	0.003571
3098.000000	0.003559
3097.750000	0.003544
3097.500000	0.003529
3097.250000	0.003516
3097.000000	0.003509
3096.750000	0.003506
3096.500000	0.003510

3096.250000	0.003518
3096.000000	0.003529
3095.750000	0.003541
3095.500000	0.003553
3095.250000	0.003561
3095.000000	0.003566
3094.750000	0.003566
3094.500000	0.003563
3094.250000	0.003558
3094.000000	0.003554
3093.750000	0.003552
3093.500000	0.003553
3093.250000	0.003557
3093.000000	0.003564
3092.750000	0.003571
3092.500000	0.003574
3092.250000	0.003573
3092.000000	0.003567
3091.750000	0.003555
3091.500000	0.003540
3091.250000	0.003524
3091.000000	0.003511
3090.750000	0.003503
3090.500000	0.003502
3090.250000	0.003508
3090.000000	0.003520

3089.750000	0.003536
3089.500000	0.003553
3089.250000	0.003571
3089.000000	0.003587
3088.750000	0.003599
3088.500000	0.003606
3088.250000	0.003610
3088.000000	0.003609
3087.750000	0.003604
3087.500000	0.003596
3087.250000	0.003586
3087.000000	0.003574
3086.750000	0.003562
3086.500000	0.003552
3086.250000	0.003544
3086.000000	0.003539
3085.750000	0.003539
3085.500000	0.003542
3085.250000	0.003550
3085.000000	0.003560
3084.750000	0.003570
3084.500000	0.003580
3084.250000	0.003589
3084.000000	0.003595
3083.750000	0.003600
3083.500000	0.003603

3083.250000	0.003606
3083.000000	0.003609
3082.750000	0.003613
3082.500000	0.003619
3082.250000	0.003625
3082.000000	0.003631
3081.750000	0.003636
3081.500000	0.003639
3081.250000	0.003640
3081.000000	0.003638
3080.750000	0.003634
3080.500000	0.003628
3080.250000	0.003624
3080.000000	0.003621
3079.750000	0.003622
3079.500000	0.003627
3079.250000	0.003636
3079.000000	0.003647
3078.750000	0.003659
3078.500000	0.003669
3078.250000	0.003676
3078.000000	0.003680
3077.750000	0.003682
3077.500000	0.003683
3077.250000	0.003686
3077.000000	0.003690

3076.750000	0.003698
3076.500000	0.003707
3076.250000	0.003718
3076.000000	0.003727
3075.750000	0.003736
3075.500000	0.003742
3075.250000	0.003746
3075.000000	0.003749
3074.750000	0.003752
3074.500000	0.003755
3074.250000	0.003758
3074.000000	0.003763
3073.750000	0.003768
3073.500000	0.003774
3073.250000	0.003780
3073.000000	0.003786
3072.750000	0.003791
3072.500000	0.003795
3072.250000	0.003798
3072.000000	0.003798
3071.750000	0.003798
3071.500000	0.003796
3071.250000	0.003794
3071.000000	0.003792
3070.750000	0.003791
3070.500000	0.003791

3070.250000	0.003789
3070.000000	0.003787
3069.750000	0.003783
3069.500000	0.003777
3069.250000	0.003771
3069.000000	0.003766
3068.750000	0.003763
3068.500000	0.003763
3068.250000	0.003767
3068.000000	0.003773
3067.750000	0.003779
3067.500000	0.003784
3067.250000	0.003786
3067.000000	0.003784
3066.750000	0.003780
3066.500000	0.003776
3066.250000	0.003774
3066.000000	0.003777
3065.750000	0.003787
3065.500000	0.003802
3065.250000	0.003820
3065.000000	0.003838
3064.750000	0.003851
3064.500000	0.003859
3064.250000	0.003859
3064.000000	0.003854

3063.750000	0.003847
3063.500000	0.003841
3063.250000	0.003839
3063.000000	0.003842
3062.750000	0.003850
3062.500000	0.003860
3062.250000	0.003872
3062.000000	0.003881
3061.750000	0.003886
3061.500000	0.003886
3061.250000	0.003884
3061.000000	0.003879
3060.750000	0.003874
3060.500000	0.003872
3060.250000	0.003871
3060.000000	0.003874
3059.750000	0.003878
3059.500000	0.003883
3059.250000	0.003888
3059.000000	0.003892
3058.750000	0.003894
3058.500000	0.003894
3058.250000	0.003892
3058.000000	0.003889
3057.750000	0.003884
3057.500000	0.003880

3057.250000	0.003875
3057.000000	0.003871
3056.750000	0.003866
3056.500000	0.003861
3056.250000	0.003855
3056.000000	0.003847
3055.750000	0.003839
3055.500000	0.003829
3055.250000	0.003821
3055.000000	0.003814
3054.750000	0.003811
3054.500000	0.003812
3054.250000	0.003817
3054.000000	0.003824
3053.750000	0.003831
3053.500000	0.003838
3053.250000	0.003843
3053.000000	0.003845
3052.750000	0.003844
3052.500000	0.003841
3052.250000	0.003836
3052.000000	0.003831
3051.750000	0.003824
3051.500000	0.003818
3051.250000	0.003812
3051.000000	0.003806

3050.750000	0.003803
3050.500000	0.003804
3050.250000	0.003808
3050.000000	0.003817
3049.750000	0.003830
3049.500000	0.003845
3049.250000	0.003860
3049.000000	0.003873
3048.750000	0.003881
3048.500000	0.003883
3048.250000	0.003879
3048.000000	0.003871
3047.750000	0.003859
3047.500000	0.003848
3047.250000	0.003838
3047.000000	0.003830
3046.750000	0.003827
3046.500000	0.003827
3046.250000	0.003831
3046.000000	0.003836
3045.750000	0.003843
3045.500000	0.003849
3045.250000	0.003853
3045.000000	0.003856
3044.750000	0.003857
3044.500000	0.003856

3044.250000	0.003854
3044.000000	0.003854
3043.750000	0.003855
3043.500000	0.003859
3043.250000	0.003867
3043.000000	0.003878
3042.750000	0.003892
3042.500000	0.003908
3042.250000	0.003923
3042.000000	0.003935
3041.750000	0.003943
3041.500000	0.003945
3041.250000	0.003943
3041.000000	0.003938
3040.750000	0.003930
3040.500000	0.003924
3040.250000	0.003923
3040.000000	0.003926
3039.750000	0.003936
3039.500000	0.003952
3039.250000	0.003970
3039.000000	0.003989
3038.750000	0.004006
3038.500000	0.004018
3038.250000	0.004025
3038.000000	0.004026

3037.750000	0.004024
3037.500000	0.004019
3037.250000	0.004013
3037.000000	0.004007
3036.750000	0.004003
3036.500000	0.003999
3036.250000	0.003996
3036.000000	0.003995
3035.750000	0.003995
3035.500000	0.003999
3035.250000	0.004005
3035.000000	0.004015
3034.750000	0.004027
3034.500000	0.004041
3034.250000	0.004056
3034.000000	0.004071
3033.750000	0.004086
3033.500000	0.004101
3033.250000	0.004115
3033.000000	0.004130
3032.750000	0.004145
3032.500000	0.004160
3032.250000	0.004176
3032.000000	0.004191
3031.750000	0.004204
3031.500000	0.004214

3031.250000	0.004223
3031.000000	0.004229
3030.750000	0.004233
3030.500000	0.004237
3030.250000	0.004241
3030.000000	0.004247
3029.750000	0.004253
3029.500000	0.004260
3029.250000	0.004268
3029.000000	0.004275
3028.750000	0.004282
3028.500000	0.004289
3028.250000	0.004295
3028.000000	0.004300
3027.750000	0.004304
3027.500000	0.004308
3027.250000	0.004310
3027.000000	0.004312
3026.750000	0.004312
3026.500000	0.004314
3026.250000	0.004316
3026.000000	0.004321
3025.750000	0.004329
3025.500000	0.004340
3025.250000	0.004353
3025.000000	0.004367

3024.750000	0.004380
3024.500000	0.004392
3024.250000	0.004401
3024.000000	0.004407
3023.750000	0.004413
3023.500000	0.004418
3023.250000	0.004424
3023.000000	0.004433
3022.750000	0.004445
3022.500000	0.004460
3022.250000	0.004478
3022.000000	0.004498
3021.750000	0.004519
3021.500000	0.004539
3021.250000	0.004557
3021.000000	0.004572
3020.750000	0.004583
3020.500000	0.004590
3020.250000	0.004594
3020.000000	0.004597
3019.750000	0.004602
3019.500000	0.004608
3019.250000	0.004619
3019.000000	0.004634
3018.750000	0.004652
3018.500000	0.004674

3018.250000	0.004699
3018.000000	0.004725
3017.750000	0.004752
3017.500000	0.004780
3017.250000	0.004808
3017.000000	0.004835
3016.750000	0.004860
3016.500000	0.004883
3016.250000	0.004902
3016.000000	0.004919
3015.750000	0.004932
3015.500000	0.004943
3015.250000	0.004952
3015.000000	0.004961
3014.750000	0.004972
3014.500000	0.004984
3014.250000	0.004999
3014.000000	0.005018
3013.750000	0.005041
3013.500000	0.005068
3013.250000	0.005100
3013.000000	0.005134
3012.750000	0.005169
3012.500000	0.005203
3012.250000	0.005235
3012.000000	0.005263

3011.750000	0.005289
3011.500000	0.005312
3011.250000	0.005335
3011.000000	0.005358
3010.750000	0.005384
3010.500000	0.005413
3010.250000	0.005444
3010.000000	0.005477
3009.750000	0.005508
3009.500000	0.005536
3009.250000	0.005560
3009.000000	0.005578
3008.750000	0.005591
3008.500000	0.005601
3008.250000	0.005612
3008.000000	0.005626
3007.750000	0.005644
3007.500000	0.005668
3007.250000	0.005698
3007.000000	0.005731
3006.750000	0.005767
3006.500000	0.005802
3006.250000	0.005835
3006.000000	0.005867
3005.750000	0.005896
3005.500000	0.005923

3005.250000	0.005947
3005.000000	0.005970
3004.750000	0.005990
3004.500000	0.006009
3004.250000	0.006027
3004.000000	0.006045
3003.750000	0.006064
3003.500000	0.006085
3003.250000	0.006109
3003.000000	0.006134
3002.750000	0.006160
3002.500000	0.006186
3002.250000	0.006210
3002.000000	0.006235
3001.750000	0.006258
3001.500000	0.006283
3001.250000	0.006308
3001.000000	0.006333
3000.750000	0.006359
3000.500000	0.006383
3000.250000	0.006407
3000.000000	0.006429
2999.750000	0.006450
2999.500000	0.006472
2999.250000	0.006495
2999.000000	0.006521

2998.750000	0.006548
2998.500000	0.006577
2998.250000	0.006606
2998.000000	0.006635
2997.750000	0.006663
2997.500000	0.006692
2997.250000	0.006723
2997.000000	0.006755
2996.750000	0.006789
2996.500000	0.006824
2996.250000	0.006858
2996.000000	0.006890
2995.750000	0.006919
2995.500000	0.006944
2995.250000	0.006966
2995.000000	0.006986
2994.750000	0.007006
2994.500000	0.007026
2994.250000	0.007048
2994.000000	0.007072
2993.750000	0.007098
2993.500000	0.007126
2993.250000	0.007156
2993.000000	0.007186
2992.750000	0.007217
2992.500000	0.007249

2992.250000	0.007281
2992.000000	0.007314
2991.750000	0.007348
2991.500000	0.007382
2991.250000	0.007418
2991.000000	0.007453
2990.750000	0.007488
2990.500000	0.007521
2990.250000	0.007551
2990.000000	0.007578
2989.750000	0.007602
2989.500000	0.007625
2989.250000	0.007647
2989.000000	0.007671
2988.750000	0.007697
2988.500000	0.007726
2988.250000	0.007757
2988.000000	0.007789
2987.750000	0.007821
2987.500000	0.007852
2987.250000	0.007881
2987.000000	0.007908
2986.750000	0.007934
2986.500000	0.007960
2986.250000	0.007985
2986.000000	0.008011

2985.750000	0.008039
2985.500000	0.008068
2985.250000	0.008098
2985.000000	0.008130
2984.750000	0.008162
2984.500000	0.008193
2984.250000	0.008223
2984.000000	0.008250
2983.750000	0.008274
2983.500000	0.008296
2983.250000	0.008315
2983.000000	0.008333
2982.750000	0.008350
2982.500000	0.008366
2982.250000	0.008380
2982.000000	0.008392
2981.750000	0.008402
2981.500000	0.008411
2981.250000	0.008418
2981.000000	0.008427
2980.750000	0.008438
2980.500000	0.008452
2980.250000	0.008470
2980.000000	0.008491
2979.750000	0.008515
2979.500000	0.008541

2979.250000	0.008566
2979.000000	0.008590
2978.750000	0.008613
2978.500000	0.008636
2978.250000	0.008659
2978.000000	0.008682
2977.750000	0.008706
2977.500000	0.008732
2977.250000	0.008760
2977.000000	0.008788
2976.750000	0.008816
2976.500000	0.008844
2976.250000	0.008871
2976.000000	0.008899
2975.750000	0.008928
2975.500000	0.008961
2975.250000	0.008999
2975.000000	0.009045
2974.750000	0.009100
2974.500000	0.009162
2974.250000	0.009229
2974.000000	0.009300
2973.750000	0.009372
2973.500000	0.009442
2973.250000	0.009509
2973.000000	0.009574

2972.750000	0.009640
2972.500000	0.009708
2972.250000	0.009783
2972.000000	0.009866
2971.750000	0.009957
2971.500000	0.010054
2971.250000	0.010152
2971.000000	0.010248
2970.750000	0.010335
2970.500000	0.010410
2970.250000	0.010469
2970.000000	0.010512
2969.750000	0.010539
2969.500000	0.010554
2969.250000	0.010558
2969.000000	0.010555
2968.750000	0.010549
2968.500000	0.010542
2968.250000	0.010539
2968.000000	0.010540
2967.750000	0.010548
2967.500000	0.010564
2967.250000	0.010587
2967.000000	0.010616
2966.750000	0.010650
2966.500000	0.010687

2966.250000	0.010727
2966.000000	0.010769
2965.750000	0.010814
2965.500000	0.010862
2965.250000	0.010914
2965.000000	0.010969
2964.750000	0.011026
2964.500000	0.011082
2964.250000	0.011135
2964.000000	0.011183
2963.750000	0.011228
2963.500000	0.011270
2963.250000	0.011311
2963.000000	0.011353
2962.750000	0.011396
2962.500000	0.011442
2962.250000	0.011489
2962.000000	0.011536
2961.750000	0.011582
2961.500000	0.011625
2961.250000	0.011667
2961.000000	0.011709
2960.750000	0.011751
2960.500000	0.011795
2960.250000	0.011841
2960.000000	0.011887

2959.750000	0.011934
2959.500000	0.011980
2959.250000	0.012025
2959.000000	0.012067
2958.750000	0.012109
2958.500000	0.012149
2958.250000	0.012188
2958.000000	0.012227
2957.750000	0.012264
2957.500000	0.012299
2957.250000	0.012333
2957.000000	0.012365
2956.750000	0.012398
2956.500000	0.012430
2956.250000	0.012463
2956.000000	0.012497
2955.750000	0.012529
2955.500000	0.012561
2955.250000	0.012589
2955.000000	0.012614
2954.750000	0.012635
2954.500000	0.012653
2954.250000	0.012670
2954.000000	0.012687
2953.750000	0.012706
2953.500000	0.012728

2953.250000	0.012754
2953.000000	0.012782
2952.750000	0.012812
2952.500000	0.012842
2952.250000	0.012872
2952.000000	0.012901
2951.750000	0.012928
2951.500000	0.012954
2951.250000	0.012979
2951.000000	0.013005
2950.750000	0.013032
2950.500000	0.013060
2950.250000	0.013091
2950.000000	0.013125
2949.750000	0.013160
2949.500000	0.013197
2949.250000	0.013236
2949.000000	0.013275
2948.750000	0.013316
2948.500000	0.013358
2948.250000	0.013402
2948.000000	0.013447
2947.750000	0.013494
2947.500000	0.013543
2947.250000	0.013595
2947.000000	0.013649

2946.750000	0.013705
2946.500000	0.013763
2946.250000	0.013823
2946.000000	0.013885
2945.750000	0.013948
2945.500000	0.014012
2945.250000	0.014078
2945.000000	0.014145
2944.750000	0.014212
2944.500000	0.014279
2944.250000	0.014346
2944.000000	0.014413
2943.750000	0.014481
2943.500000	0.014550
2943.250000	0.014622
2943.000000	0.014698
2942.750000	0.014777
2942.500000	0.014861
2942.250000	0.014948
2942.000000	0.015037
2941.750000	0.015125
2941.500000	0.015212
2941.250000	0.015296
2941.000000	0.015378
2940.750000	0.015458
2940.500000	0.015538

2940.250000	0.015621
2940.000000	0.015706
2939.750000	0.015795
2939.500000	0.015888
2939.250000	0.015983
2939.000000	0.016079
2938.750000	0.016174
2938.500000	0.016267
2938.250000	0.016356
2938.000000	0.016442
2937.750000	0.016526
2937.500000	0.016608
2937.250000	0.016690
2937.000000	0.016773
2936.750000	0.016857
2936.500000	0.016942
2936.250000	0.017029
2936.000000	0.017116
2935.750000	0.017204
2935.500000	0.017292
2935.250000	0.017379
2935.000000	0.017465
2934.750000	0.017550
2934.500000	0.017634
2934.250000	0.017717
2934.000000	0.017800

2933.750000	0.017883
2933.500000	0.017968
2933.250000	0.018054
2933.000000	0.018141
2932.750000	0.018227
2932.500000	0.018310
2932.250000	0.018390
2932.000000	0.018465
2931.750000	0.018535
2931.500000	0.018601
2931.250000	0.018664
2931.000000	0.018726
2930.750000	0.018788
2930.500000	0.018853
2930.250000	0.018919
2930.000000	0.018988
2929.750000	0.019059
2929.500000	0.019132
2929.250000	0.019204
2929.000000	0.019276
2928.750000	0.019345
2928.500000	0.019411
2928.250000	0.019472
2928.000000	0.019529
2927.750000	0.019582
2927.500000	0.019630

2927.250000	0.019673
2927.000000	0.019710
2926.750000	0.019742
2926.500000	0.019767
2926.250000	0.019784
2926.000000	0.019794
2925.750000	0.019798
2925.500000	0.019798
2925.250000	0.019795
2925.000000	0.019790
2924.750000	0.019784
2924.500000	0.019777
2924.250000	0.019765
2924.000000	0.019750
2923.750000	0.019728
2923.500000	0.019699
2923.250000	0.019662
2923.000000	0.019619
2922.750000	0.019569
2922.500000	0.019512
2922.250000	0.019449
2922.000000	0.019379
2921.750000	0.019302
2921.500000	0.019218
2921.250000	0.019128
2921.000000	0.019032

2920.750000	0.018931
2920.500000	0.018826
2920.250000	0.018718
2920.000000	0.018606
2919.750000	0.018490
2919.500000	0.018370
2919.250000	0.018246
2919.000000	0.018118
2918.750000	0.017983
2918.500000	0.017843
2918.250000	0.017695
2918.000000	0.017542
2917.750000	0.017382
2917.500000	0.017218
2917.250000	0.017052
2917.000000	0.016886
2916.750000	0.016723
2916.500000	0.016564
2916.250000	0.016411
2916.000000	0.016261
2915.750000	0.016115
2915.500000	0.015970
2915.250000	0.015824
2915.000000	0.015678
2914.750000	0.015529
2914.500000	0.015380

2914.250000	0.015231
2914.000000	0.015085
2913.750000	0.014940
2913.500000	0.014798
2913.250000	0.014659
2913.000000	0.014521
2912.750000	0.014386
2912.500000	0.014251
2912.250000	0.014120
2912.000000	0.013992
2911.750000	0.013868
2911.500000	0.013749
2911.250000	0.013634
2911.000000	0.013523
2910.750000	0.013415
2910.500000	0.013308
2910.250000	0.013203
2910.000000	0.013100
2909.750000	0.013001
2909.500000	0.012905
2909.250000	0.012814
2909.000000	0.012729
2908.750000	0.012648
2908.500000	0.012571
2908.250000	0.012496
2908.000000	0.012423

2907.750000	0.012349
2907.500000	0.012274
2907.250000	0.012197
2907.000000	0.012119
2906.750000	0.012040
2906.500000	0.011961
2906.250000	0.011883
2906.000000	0.011808
2905.750000	0.011737
2905.500000	0.011670
2905.250000	0.011606
2905.000000	0.011544
2904.750000	0.011483
2904.500000	0.011422
2904.250000	0.011359
2904.000000	0.011296
2903.750000	0.011235
2903.500000	0.011175
2903.250000	0.011120
2903.000000	0.011068
2902.750000	0.011019
2902.500000	0.010973
2902.250000	0.010928
2902.000000	0.010884
2901.750000	0.010840
2901.500000	0.010797

2901.250000	0.010755
2901.000000	0.010713
2900.750000	0.010670
2900.500000	0.010626
2900.250000	0.010580
2900.000000	0.010531
2899.750000	0.010480
2899.500000	0.010429
2899.250000	0.010378
2899.000000	0.010330
2898.750000	0.010285
2898.500000	0.010243
2898.250000	0.010204
2898.000000	0.010167
2897.750000	0.010132
2897.500000	0.010096
2897.250000	0.010061
2897.000000	0.010024
2896.750000	0.009986
2896.500000	0.009947
2896.250000	0.009905
2896.000000	0.009863
2895.750000	0.009821
2895.500000	0.009781
2895.250000	0.009744
2895.000000	0.009710

2894.750000	0.009680
2894.500000	0.009653
2894.250000	0.009626
2894.000000	0.009599
2893.750000	0.009569
2893.500000	0.009536
2893.250000	0.009501
2893.000000	0.009463
2892.750000	0.009425
2892.500000	0.009387
2892.250000	0.009350
2892.000000	0.009316
2891.750000	0.009285
2891.500000	0.009256
2891.250000	0.009229
2891.000000	0.009203
2890.750000	0.009176
2890.500000	0.009147
2890.250000	0.009116
2890.000000	0.009083
2889.750000	0.009048
2889.500000	0.009015
2889.250000	0.008983
2889.000000	0.008957
2888.750000	0.008934
2888.500000	0.008915

2888.250000	0.008898
2888.000000	0.008880
2887.750000	0.008860
2887.500000	0.008836
2887.250000	0.008810
2887.000000	0.008782
2886.750000	0.008754
2886.500000	0.008728
2886.250000	0.008706
2886.000000	0.008686
2885.750000	0.008670
2885.500000	0.008657
2885.250000	0.008644
2885.000000	0.008633
2884.750000	0.008623
2884.500000	0.008612
2884.250000	0.008601
2884.000000	0.008590
2883.750000	0.008579
2883.500000	0.008567
2883.250000	0.008555
2883.000000	0.008544
2882.750000	0.008534
2882.500000	0.008526
2882.250000	0.008520
2882.000000	0.008515

2881.750000	0.008512
2881.500000	0.008510
2881.250000	0.008506
2881.000000	0.008502
2880.750000	0.008496
2880.500000	0.008489
2880.250000	0.008482
2880.000000	0.008476
2879.750000	0.008472
2879.500000	0.008472
2879.250000	0.008475
2879.000000	0.008480
2878.750000	0.008489
2878.500000	0.008498
2878.250000	0.008508
2878.000000	0.008518
2877.750000	0.008529
2877.500000	0.008540
2877.250000	0.008552
2877.000000	0.008566
2876.750000	0.008582
2876.500000	0.008600
2876.250000	0.008620
2876.000000	0.008643
2875.750000	0.008667
2875.500000	0.008691

2875.250000	0.008715
2875.000000	0.008738
2874.750000	0.008759
2874.500000	0.008777
2874.250000	0.008791
2874.000000	0.008803
2873.750000	0.008812
2873.500000	0.008822
2873.250000	0.008833
2873.000000	0.008846
2872.750000	0.008864
2872.500000	0.008885
2872.250000	0.008909
2872.000000	0.008935
2871.750000	0.008962
2871.500000	0.008989
2871.250000	0.009014
2871.000000	0.009036
2870.750000	0.009056
2870.500000	0.009072
2870.250000	0.009084
2870.000000	0.009092
2869.750000	0.009097
2869.500000	0.009101
2869.250000	0.009105
2869.000000	0.009112

2868.750000	0.009122
2868.500000	0.009136
2868.250000	0.009153
2868.000000	0.009172
2867.750000	0.009191
2867.500000	0.009209
2867.250000	0.009226
2867.000000	0.009242
2866.750000	0.009259
2866.500000	0.009278
2866.250000	0.009302
2866.000000	0.009330
2865.750000	0.009364
2865.500000	0.009402
2865.250000	0.009443
2865.000000	0.009486
2864.750000	0.009530
2864.500000	0.009574
2864.250000	0.009617
2864.000000	0.009662
2863.750000	0.009707
2863.500000	0.009755
2863.250000	0.009804
2863.000000	0.009856
2862.750000	0.009910
2862.500000	0.009966

2862.250000	0.010024
2862.000000	0.010083
2861.750000	0.010145
2861.500000	0.010211
2861.250000	0.010281
2861.000000	0.010355
2860.750000	0.010432
2860.500000	0.010511
2860.250000	0.010589
2860.000000	0.010666
2859.750000	0.010739
2859.500000	0.010811
2859.250000	0.010880
2859.000000	0.010950
2858.750000	0.011020
2858.500000	0.011092
2858.250000	0.011163
2858.000000	0.011234
2857.750000	0.011302
2857.500000	0.011365
2857.250000	0.011423
2857.000000	0.011476
2856.750000	0.011525
2856.500000	0.011571
2856.250000	0.011615
2856.000000	0.011656

2855.750000	0.011694
2855.500000	0.011728
2855.250000	0.011756
2855.000000	0.011778
2854.750000	0.011792
2854.500000	0.011798
2854.250000	0.011798
2854.000000	0.011792
2853.750000	0.011779
2853.500000	0.011761
2853.250000	0.011738
2853.000000	0.011708
2852.750000	0.011673
2852.500000	0.011631
2852.250000	0.011582
2852.000000	0.011526
2851.750000	0.011463
2851.500000	0.011391
2851.250000	0.011310
2851.000000	0.011219
2850.750000	0.011117
2850.500000	0.011005
2850.250000	0.010881
2850.000000	0.010747
2849.750000	0.010605
2849.500000	0.010455

2849.250000	0.010299
2849.000000	0.010140
2848.750000	0.009979
2848.500000	0.009817
2848.250000	0.009655
2848.000000	0.009493
2847.750000	0.009332
2847.500000	0.009171
2847.250000	0.009010
2847.000000	0.008849
2846.750000	0.008688
2846.500000	0.008528
2846.250000	0.008367
2846.000000	0.008208
2845.750000	0.008049
2845.500000	0.007892
2845.250000	0.007737
2845.000000	0.007584
2844.750000	0.007435
2844.500000	0.007290
2844.250000	0.007149
2844.000000	0.007014
2843.750000	0.006884
2843.500000	0.006760
2843.250000	0.006641
2843.000000	0.006527

2842.750000	0.006417
2842.500000	0.006310
2842.250000	0.006206
2842.000000	0.006106
2841.750000	0.006008
2841.500000	0.005913
2841.250000	0.005822
2841.000000	0.005735
2840.750000	0.005651
2840.500000	0.005571
2840.250000	0.005493
2840.000000	0.005418
2839.750000	0.005346
2839.500000	0.005277
2839.250000	0.005212
2839.000000	0.005150
2838.750000	0.005092
2838.500000	0.005038
2838.250000	0.004987
2838.000000	0.004940
2837.750000	0.004895
2837.500000	0.004853
2837.250000	0.004812
2837.000000	0.004774
2836.750000	0.004737
2836.500000	0.004701

2836.250000	0.004667
2836.000000	0.004634
2835.750000	0.004601
2835.500000	0.004568
2835.250000	0.004535
2835.000000	0.004500
2834.750000	0.004464
2834.500000	0.004427
2834.250000	0.004391
2834.000000	0.004355
2833.750000	0.004322
2833.500000	0.004291
2833.250000	0.004265
2833.000000	0.004242
2832.750000	0.004222
2832.500000	0.004206
2832.250000	0.004191
2832.000000	0.004176
2831.750000	0.004160
2831.500000	0.004143
2831.250000	0.004124
2831.000000	0.004103
2830.750000	0.004081
2830.500000	0.004059
2830.250000	0.004038
2830.000000	0.004020

2829.750000	0.004003
2829.500000	0.003988
2829.250000	0.003974
2829.000000	0.003959
2828.750000	0.003943
2828.500000	0.003925
2828.250000	0.003906
2828.000000	0.003886
2827.750000	0.003867
2827.500000	0.003848
2827.250000	0.003831
2827.000000	0.003814
2826.750000	0.003798
2826.500000	0.003780
2826.250000	0.003760
2826.000000	0.003739
2825.750000	0.003718
2825.500000	0.003698
2825.250000	0.003680
2825.000000	0.003665
2824.750000	0.003653
2824.500000	0.003644
2824.250000	0.003635
2824.000000	0.003628
2823.750000	0.003620
2823.500000	0.003610

2823.250000	0.003599
2823.000000	0.003587
2822.750000	0.003573
2822.500000	0.003560
2822.250000	0.003546
2822.000000	0.003533
2821.750000	0.003521
2821.500000	0.003510
2821.250000	0.003498
2821.000000	0.003485
2820.750000	0.003470
2820.500000	0.003454
2820.250000	0.003436
2820.000000	0.003417
2819.750000	0.003400
2819.500000	0.003385
2819.250000	0.003374
2819.000000	0.003365
2818.750000	0.003360
2818.500000	0.003356
2818.250000	0.003352
2818.000000	0.003349
2817.750000	0.003346
2817.500000	0.003342
2817.250000	0.003338
2817.000000	0.003334

2816.750000	0.003328
2816.500000	0.003320
2816.250000	0.003311
2816.000000	0.003300
2815.750000	0.003288
2815.500000	0.003277
2815.250000	0.003268
2815.000000	0.003260
2814.750000	0.003254
2814.500000	0.003247
2814.250000	0.003239
2814.000000	0.003229
2813.750000	0.003217
2813.500000	0.003204
2813.250000	0.003192
2813.000000	0.003181
2812.750000	0.003173
2812.500000	0.003167
2812.250000	0.003162
2812.000000	0.003156
2811.750000	0.003148
2811.500000	0.003139
2811.250000	0.003129
2811.000000	0.003119
2810.750000	0.003111
2810.500000	0.003104

2810.250000	0.003098
2810.000000	0.003092
2809.750000	0.003087
2809.500000	0.003080
2809.250000	0.003074
2809.000000	0.003069
2808.750000	0.003065
2808.500000	0.003063
2808.250000	0.003061
2808.000000	0.003059
2807.750000	0.003056
2807.500000	0.003050
2807.250000	0.003042
2807.000000	0.003033
2806.750000	0.003023
2806.500000	0.003015
2806.250000	0.003008
2806.000000	0.003003
2805.750000	0.002997
2805.500000	0.002992
2805.250000	0.002986
2805.000000	0.002978
2804.750000	0.002971
2804.500000	0.002964
2804.250000	0.002959
2804.000000	0.002956

2803.750000	0.002954
2803.500000	0.002954
2803.250000	0.002954
2803.000000	0.002954
2802.750000	0.002953
2802.500000	0.002951
2802.250000	0.002947
2802.000000	0.002941
2801.750000	0.002933
2801.500000	0.002923
2801.250000	0.002912
2801.000000	0.002900
2800.750000	0.002887
2800.500000	0.002876
2800.250000	0.002866
2800.000000	0.002858
2799.750000	0.002854
2799.500000	0.002853
2799.250000	0.002856
2799.000000	0.002861
2798.750000	0.002869
2798.500000	0.002877
2798.250000	0.002884
2798.000000	0.002888
2797.750000	0.002890
2797.500000	0.002888

2797.250000	0.002883
2797.000000	0.002876
2796.750000	0.002867
2796.500000	0.002858
2796.250000	0.002850
2796.000000	0.002841
2795.750000	0.002833
2795.500000	0.002825
2795.250000	0.002817
2795.000000	0.002810
2794.750000	0.002803
2794.500000	0.002797
2794.250000	0.002792
2794.000000	0.002788
2793.750000	0.002785
2793.500000	0.002783
2793.250000	0.002782
2793.000000	0.002782
2792.750000	0.002783
2792.500000	0.002785
2792.250000	0.002786
2792.000000	0.002786
2791.750000	0.002783
2791.500000	0.002779
2791.250000	0.002771
2791.000000	0.002761

2790.750000	0.002750
2790.500000	0.002739
2790.250000	0.002729
2790.000000	0.002722
2789.750000	0.002716
2789.500000	0.002713
2789.250000	0.002712
2789.000000	0.002712
2788.750000	0.002713
2788.500000	0.002715
2788.250000	0.002717
2788.000000	0.002718
2787.750000	0.002720
2787.500000	0.002721
2787.250000	0.002722
2787.000000	0.002721
2786.750000	0.002719
2786.500000	0.002715
2786.250000	0.002711
2786.000000	0.002706
2785.750000	0.002702
2785.500000	0.002698
2785.250000	0.002695
2785.000000	0.002694
2784.750000	0.002693
2784.500000	0.002692

2784.250000	0.002691
2784.000000	0.002690
2783.750000	0.002688
2783.500000	0.002687
2783.250000	0.002685
2783.000000	0.002684
2782.750000	0.002681
2782.500000	0.002678
2782.250000	0.002673
2782.000000	0.002667
2781.750000	0.002660
2781.500000	0.002654
2781.250000	0.002648
2781.000000	0.002644
2780.750000	0.002641
2780.500000	0.002640
2780.250000	0.002640
2780.000000	0.002639
2779.750000	0.002636
2779.500000	0.002633
2779.250000	0.002628
2779.000000	0.002624
2778.750000	0.002619
2778.500000	0.002616
2778.250000	0.002612
2778.000000	0.002608

2777.750000	0.002604
2777.500000	0.002598
2777.250000	0.002591
2777.000000	0.002585
2776.750000	0.002580
2776.500000	0.002578
2776.250000	0.002578
2776.000000	0.002581
2775.750000	0.002585
2775.500000	0.002589
2775.250000	0.002593
2775.000000	0.002594
2774.750000	0.002593
2774.500000	0.002591
2774.250000	0.002588
2774.000000	0.002587
2773.750000	0.002586
2773.500000	0.002586
2773.250000	0.002586
2773.000000	0.002585
2772.750000	0.002582
2772.500000	0.002575
2772.250000	0.002565
2772.000000	0.002554
2771.750000	0.002543
2771.500000	0.002535

2771.250000	0.002533
2771.000000	0.002536
2770.750000	0.002546
2770.500000	0.002559
2770.250000	0.002572
2770.000000	0.002584
2769.750000	0.002591
2769.500000	0.002592
2769.250000	0.002588
2769.000000	0.002579
2768.750000	0.002568
2768.500000	0.002556
2768.250000	0.002545
2768.000000	0.002536
2767.750000	0.002529
2767.500000	0.002525
2767.250000	0.002521
2767.000000	0.002519
2766.750000	0.002519
2766.500000	0.002520
2766.250000	0.002524
2766.000000	0.002530
2765.750000	0.002537
2765.500000	0.002546
2765.250000	0.002554
2765.000000	0.002560

2764.750000	0.002563
2764.500000	0.002562
2764.250000	0.002558
2764.000000	0.002551
2763.750000	0.002542
2763.500000	0.002532
2763.250000	0.002524
2763.000000	0.002518
2762.750000	0.002514
2762.500000	0.002514
2762.250000	0.002515
2762.000000	0.002518
2761.750000	0.002522
2761.500000	0.002525
2761.250000	0.002527
2761.000000	0.002527
2760.750000	0.002527
2760.500000	0.002526
2760.250000	0.002524
2760.000000	0.002522
2759.750000	0.002520
2759.500000	0.002518
2759.250000	0.002516
2759.000000	0.002513
2758.750000	0.002510
2758.500000	0.002506

2758.250000	0.002501
2758.000000	0.002495
2757.750000	0.002489
2757.500000	0.002483
2757.250000	0.002478
2757.000000	0.002475
2756.750000	0.002474
2756.500000	0.002476
2756.250000	0.002479
2756.000000	0.002482
2755.750000	0.002484
2755.500000	0.002484
2755.250000	0.002483
2755.000000	0.002481
2754.750000	0.002478
2754.500000	0.002476
2754.250000	0.002475
2754.000000	0.002475
2753.750000	0.002476
2753.500000	0.002478
2753.250000	0.002480
2753.000000	0.002482
2752.750000	0.002482
2752.500000	0.002481
2752.250000	0.002478
2752.000000	0.002473

2751.750000	0.002467
2751.500000	0.002458
2751.250000	0.002449
2751.000000	0.002440
2750.750000	0.002432
2750.500000	0.002425
2750.250000	0.002420
2750.000000	0.002415
2749.750000	0.002412
2749.500000	0.002409
2749.250000	0.002406
2749.000000	0.002404
2748.750000	0.002403
2748.500000	0.002403
2748.250000	0.002404
2748.000000	0.002407
2747.750000	0.002412
2747.500000	0.002418
2747.250000	0.002425
2747.000000	0.002432
2746.750000	0.002439
2746.500000	0.002446
2746.250000	0.002452
2746.000000	0.002456
2745.750000	0.002458
2745.500000	0.002459

2745.250000	0.002457
2745.000000	0.002455
2744.750000	0.002452
2744.500000	0.002448
2744.250000	0.002445
2744.000000	0.002442
2743.750000	0.002439
2743.500000	0.002437
2743.250000	0.002433
2743.000000	0.002430
2742.750000	0.002427
2742.500000	0.002424
2742.250000	0.002422
2742.000000	0.002421
2741.750000	0.002421
2741.500000	0.002422
2741.250000	0.002422
2741.000000	0.002422
2740.750000	0.002420
2740.500000	0.002417
2740.250000	0.002413
2740.000000	0.002407
2739.750000	0.002401
2739.500000	0.002394
2739.250000	0.002386
2739.000000	0.002379

2738.750000	0.002372
2738.500000	0.002365
2738.250000	0.002361
2738.000000	0.002358
2737.750000	0.002359
2737.500000	0.002364
2737.250000	0.002372
2737.000000	0.002383
2736.750000	0.002396
2736.500000	0.002408
2736.250000	0.002417
2736.000000	0.002422
2735.750000	0.002421
2735.500000	0.002416
2735.250000	0.002406
2735.000000	0.002396
2734.750000	0.002386
2734.500000	0.002379
2734.250000	0.002375
2734.000000	0.002373
2733.750000	0.002374
2733.500000	0.002375
2733.250000	0.002375
2733.000000	0.002375
2732.750000	0.002376
2732.500000	0.002376

2732.250000	0.002378
2732.000000	0.002382
2731.750000	0.002386
2731.500000	0.002389
2731.250000	0.002391
2731.000000	0.002389
2730.750000	0.002384
2730.500000	0.002376
2730.250000	0.002366
2730.000000	0.002355
2729.750000	0.002345
2729.500000	0.002337
2729.250000	0.002332
2729.000000	0.002329
2728.750000	0.002328
2728.500000	0.002327
2728.250000	0.002325
2728.000000	0.002322
2727.750000	0.002316
2727.500000	0.002308
2727.250000	0.002300
2727.000000	0.002291
2726.750000	0.002284
2726.500000	0.002280
2726.250000	0.002280
2726.000000	0.002284

2725.750000	0.002292
2725.500000	0.002303
2725.250000	0.002314
2725.000000	0.002324
2724.750000	0.002331
2724.500000	0.002335
2724.250000	0.002334
2724.000000	0.002329
2723.750000	0.002320
2723.500000	0.002309
2723.250000	0.002298
2723.000000	0.002287
2722.750000	0.002278
2722.500000	0.002272
2722.250000	0.002269
2722.000000	0.002268
2721.750000	0.002270
2721.500000	0.002272
2721.250000	0.002275
2721.000000	0.002278
2720.750000	0.002279
2720.500000	0.002280
2720.250000	0.002279
2720.000000	0.002277
2719.750000	0.002275
2719.500000	0.002271

2719.250000	0.002268
2719.000000	0.002264
2718.750000	0.002260
2718.500000	0.002257
2718.250000	0.002254
2718.000000	0.002252
2717.750000	0.002250
2717.500000	0.002248
2717.250000	0.002246
2717.000000	0.002245
2716.750000	0.002244
2716.500000	0.002245
2716.250000	0.002246
2716.000000	0.002249
2715.750000	0.002253
2715.500000	0.002256
2715.250000	0.002258
2715.000000	0.002258
2714.750000	0.002256
2714.500000	0.002252
2714.250000	0.002247
2714.000000	0.002242
2713.750000	0.002239
2713.500000	0.002238
2713.250000	0.002241
2713.000000	0.002245

2712.750000	0.002250
2712.500000	0.002255
2712.250000	0.002257
2712.000000	0.002257
2711.750000	0.002254
2711.500000	0.002248
2711.250000	0.002239
2711.000000	0.002228
2710.750000	0.002217
2710.500000	0.002206
2710.250000	0.002197
2710.000000	0.002189
2709.750000	0.002185
2709.500000	0.002182
2709.250000	0.002183
2709.000000	0.002185
2708.750000	0.002189
2708.500000	0.002193
2708.250000	0.002198
2708.000000	0.002202
2707.750000	0.002205
2707.500000	0.002207
2707.250000	0.002207
2707.000000	0.002206
2706.750000	0.002204
2706.500000	0.002201

2706.250000	0.002196
2706.000000	0.002189
2705.750000	0.002182
2705.500000	0.002174
2705.250000	0.002166
2705.000000	0.002161
2704.750000	0.002158
2704.500000	0.002160
2704.250000	0.002165
2704.000000	0.002173
2703.750000	0.002184
2703.500000	0.002195
2703.250000	0.002204
2703.000000	0.002211
2702.750000	0.002214
2702.500000	0.002213
2702.250000	0.002208
2702.000000	0.002201
2701.750000	0.002192
2701.500000	0.002182
2701.250000	0.002174
2701.000000	0.002166
2700.750000	0.002161
2700.500000	0.002157
2700.250000	0.002156
2700.000000	0.002156

2699.750000	0.002157
2699.500000	0.002158
2699.250000	0.002158
2699.000000	0.002158
2698.750000	0.002158
2698.500000	0.002158
2698.250000	0.002158
2698.000000	0.002159
2697.750000	0.002160
2697.500000	0.002162
2697.250000	0.002163
2697.000000	0.002164
2696.750000	0.002165
2696.500000	0.002166
2696.250000	0.002168
2696.000000	0.002170
2695.750000	0.002172
2695.500000	0.002172
2695.250000	0.002170
2695.000000	0.002166
2694.750000	0.002159
2694.500000	0.002152
2694.250000	0.002146
2694.000000	0.002142
2693.750000	0.002143
2693.500000	0.002147

2693.250000	0.002156
2693.000000	0.002165
2692.750000	0.002174
2692.500000	0.002180
2692.250000	0.002181
2692.000000	0.002178
2691.750000	0.002171
2691.500000	0.002163
2691.250000	0.002155
2691.000000	0.002149
2690.750000	0.002147
2690.500000	0.002148
2690.250000	0.002153
2690.000000	0.002159
2689.750000	0.002166
2689.500000	0.002173
2689.250000	0.002177
2689.000000	0.002180
2688.750000	0.002180
2688.500000	0.002178
2688.250000	0.002175
2688.000000	0.002169
2687.750000	0.002162
2687.500000	0.002154
2687.250000	0.002145
2687.000000	0.002136

2686.750000	0.002129
2686.500000	0.002124
2686.250000	0.002121
2686.000000	0.002121
2685.750000	0.002123
2685.500000	0.002127
2685.250000	0.002130
2685.000000	0.002132
2684.750000	0.002131
2684.500000	0.002129
2684.250000	0.002124
2684.000000	0.002120
2683.750000	0.002115
2683.500000	0.002113
2683.250000	0.002113
2683.000000	0.002114
2682.750000	0.002116
2682.500000	0.002117
2682.250000	0.002116
2682.000000	0.002113
2681.750000	0.002108
2681.500000	0.002101
2681.250000	0.002094
2681.000000	0.002088
2680.750000	0.002083
2680.500000	0.002080

2680.250000	0.002077
2680.000000	0.002076
2679.750000	0.002075
2679.500000	0.002074
2679.250000	0.002073
2679.000000	0.002073
2678.750000	0.002074
2678.500000	0.002075
2678.250000	0.002076
2678.000000	0.002077
2677.750000	0.002077
2677.500000	0.002076
2677.250000	0.002074
2677.000000	0.002072
2676.750000	0.002068
2676.500000	0.002064
2676.250000	0.002059
2676.000000	0.002054
2675.750000	0.002049
2675.500000	0.002045
2675.250000	0.002041
2675.000000	0.002038
2674.750000	0.002037
2674.500000	0.002037
2674.250000	0.002039
2674.000000	0.002042

2673.750000	0.002045
2673.500000	0.002048
2673.250000	0.002050
2673.000000	0.002052
2672.750000	0.002053
2672.500000	0.002054
2672.250000	0.002054
2672.000000	0.002055
2671.750000	0.002056
2671.500000	0.002056
2671.250000	0.002057
2671.000000	0.002058
2670.750000	0.002058
2670.500000	0.002057
2670.250000	0.002056
2670.000000	0.002054
2669.750000	0.002053
2669.500000	0.002052
2669.250000	0.002052
2669.000000	0.002053
2668.750000	0.002057
2668.500000	0.002061
2668.250000	0.002066
2668.000000	0.002070
2667.750000	0.002073
2667.500000	0.002075

2667.250000	0.002075
2667.000000	0.002075
2666.750000	0.002074
2666.500000	0.002074
2666.250000	0.002074
2666.000000	0.002075
2665.750000	0.002077
2665.500000	0.002079
2665.250000	0.002083
2665.000000	0.002087
2664.750000	0.002092
2664.500000	0.002097
2664.250000	0.002104
2664.000000	0.002110
2663.750000	0.002115
2663.500000	0.002119
2663.250000	0.002121
2663.000000	0.002122
2662.750000	0.002121
2662.500000	0.002120
2662.250000	0.002120
2662.000000	0.002120
2661.750000	0.002122
2661.500000	0.002124
2661.250000	0.002127
2661.000000	0.002131

2660.750000	0.002134
2660.500000	0.002136
2660.250000	0.002138
2660.000000	0.002138
2659.750000	0.002137
2659.500000	0.002135
2659.250000	0.002131
2659.000000	0.002127
2658.750000	0.002121
2658.500000	0.002115
2658.250000	0.002110
2658.000000	0.002106
2657.750000	0.002104
2657.500000	0.002105
2657.250000	0.002108
2657.000000	0.002112
2656.750000	0.002118
2656.500000	0.002123
2656.250000	0.002127
2656.000000	0.002130
2655.750000	0.002130
2655.500000	0.002129
2655.250000	0.002126
2655.000000	0.002123
2654.750000	0.002120
2654.500000	0.002118

2654.250000	0.002117
2654.000000	0.002117
2653.750000	0.002120
2653.500000	0.002123
2653.250000	0.002128
2653.000000	0.002134
2652.750000	0.002140
2652.500000	0.002145
2652.250000	0.002150
2652.000000	0.002153
2651.750000	0.002154
2651.500000	0.002154
2651.250000	0.002153
2651.000000	0.002151
2650.750000	0.002149
2650.500000	0.002148
2650.250000	0.002148
2650.000000	0.002147
2649.750000	0.002146
2649.500000	0.002144
2649.250000	0.002138
2649.000000	0.002130
2648.750000	0.002119
2648.500000	0.002106
2648.250000	0.002093
2648.000000	0.002081

2647.750000	0.002072
2647.500000	0.002066
2647.250000	0.002063
2647.000000	0.002064
2646.750000	0.002066
2646.500000	0.002068
2646.250000	0.002070
2646.000000	0.002071
2645.750000	0.002071
2645.500000	0.002069
2645.250000	0.002067
2645.000000	0.002064
2644.750000	0.002062
2644.500000	0.002059
2644.250000	0.002056
2644.000000	0.002054
2643.750000	0.002052
2643.500000	0.002050
2643.250000	0.002048
2643.000000	0.002046
2642.750000	0.002044
2642.500000	0.002041
2642.250000	0.002036
2642.000000	0.002031
2641.750000	0.002025
2641.500000	0.002020

2641.250000	0.002017
2641.000000	0.002016
2640.750000	0.002018
2640.500000	0.002021
2640.250000	0.002026
2640.000000	0.002029
2639.750000	0.002031
2639.500000	0.002029
2639.250000	0.002025
2639.000000	0.002018
2638.750000	0.002010
2638.500000	0.002001
2638.250000	0.001993
2638.000000	0.001986
2637.750000	0.001982
2637.500000	0.001979
2637.250000	0.001978
2637.000000	0.001980
2636.750000	0.001984
2636.500000	0.001989
2636.250000	0.001996
2636.000000	0.002002
2635.750000	0.002007
2635.500000	0.002010
2635.250000	0.002010
2635.000000	0.002006

2634.750000	0.002000
2634.500000	0.001992
2634.250000	0.001982
2634.000000	0.001973
2633.750000	0.001967
2633.500000	0.001963
2633.250000	0.001964
2633.000000	0.001968
2632.750000	0.001976
2632.500000	0.001984
2632.250000	0.001992
2632.000000	0.001997
2631.750000	0.001999
2631.500000	0.001997
2631.250000	0.001992
2631.000000	0.001983
2630.750000	0.001973
2630.500000	0.001964
2630.250000	0.001956
2630.000000	0.001950
2629.750000	0.001948
2629.500000	0.001949
2629.250000	0.001953
2629.000000	0.001959
2628.750000	0.001963
2628.500000	0.001966

2628.250000	0.001964
2628.000000	0.001958
2627.750000	0.001949
2627.500000	0.001936
2627.250000	0.001924
2627.000000	0.001913
2626.750000	0.001905
2626.500000	0.001901
2626.250000	0.001901
2626.000000	0.001902
2625.750000	0.001905
2625.500000	0.001907
2625.250000	0.001909
2625.000000	0.001910
2624.750000	0.001911
2624.500000	0.001912
2624.250000	0.001913
2624.000000	0.001914
2623.750000	0.001914
2623.500000	0.001914
2623.250000	0.001913
2623.000000	0.001913
2622.750000	0.001915
2622.500000	0.001917
2622.250000	0.001921
2622.000000	0.001924

2621.750000	0.001927
2621.500000	0.001927
2621.250000	0.001925
2621.000000	0.001920
2620.750000	0.001915
2620.500000	0.001911
2620.250000	0.001907
2620.000000	0.001904
2619.750000	0.001902
2619.500000	0.001899
2619.250000	0.001896
2619.000000	0.001891
2618.750000	0.001886
2618.500000	0.001881
2618.250000	0.001877
2618.000000	0.001875
2617.750000	0.001874
2617.500000	0.001874
2617.250000	0.001875
2617.000000	0.001875
2616.750000	0.001876
2616.500000	0.001875
2616.250000	0.001874
2616.000000	0.001873
2615.750000	0.001872
2615.500000	0.001872

2615.250000	0.001872
2615.000000	0.001873
2614.750000	0.001873
2614.500000	0.001873
2614.250000	0.001872
2614.000000	0.001870
2613.750000	0.001868
2613.500000	0.001866
2613.250000	0.001864
2613.000000	0.001864
2612.750000	0.001866
2612.500000	0.001871
2612.250000	0.001878
2612.000000	0.001886
2611.750000	0.001895
2611.500000	0.001904
2611.250000	0.001910
2611.000000	0.001914
2610.750000	0.001914
2610.500000	0.001909
2610.250000	0.001901
2610.000000	0.001888
2609.750000	0.001872
2609.500000	0.001853
2609.250000	0.001834
2609.000000	0.001816

2608.750000	0.001802
2608.500000	0.001793
2608.250000	0.001789
2608.000000	0.001791
2607.750000	0.001797
2607.500000	0.001806
2607.250000	0.001816
2607.000000	0.001825
2606.750000	0.001830
2606.500000	0.001832
2606.250000	0.001829
2606.000000	0.001822
2605.750000	0.001810
2605.500000	0.001797
2605.250000	0.001782
2605.000000	0.001769
2604.750000	0.001758
2604.500000	0.001752
2604.250000	0.001750
2604.000000	0.001752
2603.750000	0.001755
2603.500000	0.001759
2603.250000	0.001761
2603.000000	0.001761
2602.750000	0.001760
2602.500000	0.001758

2602.250000	0.001756
2602.000000	0.001755
2601.750000	0.001755
2601.500000	0.001754
2601.250000	0.001753
2601.000000	0.001750
2600.750000	0.001745
2600.500000	0.001738
2600.250000	0.001733
2600.000000	0.001729
2599.750000	0.001730
2599.500000	0.001735
2599.250000	0.001743
2599.000000	0.001753
2598.750000	0.001763
2598.500000	0.001771
2598.250000	0.001776
2598.000000	0.001778
2597.750000	0.001778
2597.500000	0.001775
2597.250000	0.001772
2597.000000	0.001769
2596.750000	0.001766
2596.500000	0.001765
2596.250000	0.001765
2596.000000	0.001766

2595.750000	0.001768
2595.500000	0.001770
2595.250000	0.001771
2595.000000	0.001772
2594.750000	0.001771
2594.500000	0.001769
2594.250000	0.001766
2594.000000	0.001764
2593.750000	0.001762
2593.500000	0.001762
2593.250000	0.001763
2593.000000	0.001765
2592.750000	0.001766
2592.500000	0.001766
2592.250000	0.001764
2592.000000	0.001761
2591.750000	0.001759
2591.500000	0.001758
2591.250000	0.001760
2591.000000	0.001766
2590.750000	0.001775
2590.500000	0.001787
2590.250000	0.001798
2590.000000	0.001806
2589.750000	0.001810
2589.500000	0.001810

2589.250000	0.001805
2589.000000	0.001797
2588.750000	0.001789
2588.500000	0.001782
2588.250000	0.001777
2588.000000	0.001774
2587.750000	0.001774
2587.500000	0.001776
2587.250000	0.001779
2587.000000	0.001782
2586.750000	0.001785
2586.500000	0.001786
2586.250000	0.001784
2586.000000	0.001778
2585.750000	0.001768
2585.500000	0.001754
2585.250000	0.001737
2585.000000	0.001719
2584.750000	0.001704
2584.500000	0.001693
2584.250000	0.001689
2584.000000	0.001691
2583.750000	0.001700
2583.500000	0.001714
2583.250000	0.001731
2583.000000	0.001747

2582.750000	0.001762
2582.500000	0.001773
2582.250000	0.001779
2582.000000	0.001778
2581.750000	0.001773
2581.500000	0.001763
2581.250000	0.001752
2581.000000	0.001740
2580.750000	0.001732
2580.500000	0.001728
2580.250000	0.001729
2580.000000	0.001734
2579.750000	0.001741
2579.500000	0.001747
2579.250000	0.001750
2579.000000	0.001748
2578.750000	0.001742
2578.500000	0.001731
2578.250000	0.001719
2578.000000	0.001708
2577.750000	0.001698
2577.500000	0.001689
2577.250000	0.001682
2577.000000	0.001676
2576.750000	0.001669
2576.500000	0.001663

2576.250000	0.001659
2576.000000	0.001658
2575.750000	0.001662
2575.500000	0.001672
2575.250000	0.001687
2575.000000	0.001704
2574.750000	0.001722
2574.500000	0.001738
2574.250000	0.001750
2574.000000	0.001757
2573.750000	0.001761
2573.500000	0.001762
2573.250000	0.001760
2573.000000	0.001756
2572.750000	0.001749
2572.500000	0.001740
2572.250000	0.001727
2572.000000	0.001712
2571.750000	0.001695
2571.500000	0.001679
2571.250000	0.001666
2571.000000	0.001658
2570.750000	0.001656
2570.500000	0.001659
2570.250000	0.001666
2570.000000	0.001677

2569.750000	0.001690
2569.500000	0.001703
2569.250000	0.001716
2569.000000	0.001727
2568.750000	0.001735
2568.500000	0.001739
2568.250000	0.001738
2568.000000	0.001731
2567.750000	0.001720
2567.500000	0.001704
2567.250000	0.001688
2567.000000	0.001673
2566.750000	0.001663
2566.500000	0.001658
2566.250000	0.001659
2566.000000	0.001665
2565.750000	0.001673
2565.500000	0.001681
2565.250000	0.001689
2565.000000	0.001695
2564.750000	0.001701
2564.500000	0.001706
2564.250000	0.001711
2564.000000	0.001717
2563.750000	0.001723
2563.500000	0.001727

2563.250000	0.001728
2563.000000	0.001723
2562.750000	0.001713
2562.500000	0.001699
2562.250000	0.001683
2562.000000	0.001668
2561.750000	0.001656
2561.500000	0.001650
2561.250000	0.001650
2561.000000	0.001654
2560.750000	0.001661
2560.500000	0.001666
2560.250000	0.001668
2560.000000	0.001666
2559.750000	0.001659
2559.500000	0.001651
2559.250000	0.001643
2559.000000	0.001639
2558.750000	0.001640
2558.500000	0.001646
2558.250000	0.001657
2558.000000	0.001671
2557.750000	0.001684
2557.500000	0.001694
2557.250000	0.001700
2557.000000	0.001702

2556.750000	0.001700
2556.500000	0.001695
2556.250000	0.001690
2556.000000	0.001684
2555.750000	0.001678
2555.500000	0.001673
2555.250000	0.001667
2555.000000	0.001660
2554.750000	0.001653
2554.500000	0.001645
2554.250000	0.001638
2554.000000	0.001631
2553.750000	0.001626
2553.500000	0.001622
2553.250000	0.001619
2553.000000	0.001618
2552.750000	0.001617
2552.500000	0.001618
2552.250000	0.001622
2552.000000	0.001629
2551.750000	0.001639
2551.500000	0.001652
2551.250000	0.001667
2551.000000	0.001681
2550.750000	0.001693
2550.500000	0.001700

2550.250000	0.001702
2550.000000	0.001700
2549.750000	0.001694
2549.500000	0.001686
2549.250000	0.001678
2549.000000	0.001671
2548.750000	0.001665
2548.500000	0.001661
2548.250000	0.001659
2548.000000	0.001657
2547.750000	0.001657
2547.500000	0.001658
2547.250000	0.001661
2547.000000	0.001665
2546.750000	0.001669
2546.500000	0.001671
2546.250000	0.001669
2546.000000	0.001662
2545.750000	0.001651
2545.500000	0.001637
2545.250000	0.001620
2545.000000	0.001604
2544.750000	0.001589
2544.500000	0.001576
2544.250000	0.001566
2544.000000	0.001558

2543.750000	0.001552
2543.500000	0.001546
2543.250000	0.001542
2543.000000	0.001539
2542.750000	0.001536
2542.500000	0.001534
2542.250000	0.001533
2542.000000	0.001532
2541.750000	0.001532
2541.500000	0.001531
2541.250000	0.001531
2541.000000	0.001532
2540.750000	0.001535
2540.500000	0.001537
2540.250000	0.001540
2540.000000	0.001543
2539.750000	0.001544
2539.500000	0.001544
2539.250000	0.001543
2539.000000	0.001541
2538.750000	0.001539
2538.500000	0.001539
2538.250000	0.001541
2538.000000	0.001545
2537.750000	0.001554
2537.500000	0.001566

2537.250000	0.001582
2537.000000	0.001600
2536.750000	0.001618
2536.500000	0.001633
2536.250000	0.001643
2536.000000	0.001646
2535.750000	0.001642
2535.500000	0.001634
2535.250000	0.001624
2535.000000	0.001614
2534.750000	0.001607
2534.500000	0.001603
2534.250000	0.001600
2534.000000	0.001597
2533.750000	0.001591
2533.500000	0.001582
2533.250000	0.001572
2533.000000	0.001562
2532.750000	0.001555
2532.500000	0.001554
2532.250000	0.001559
2532.000000	0.001570
2531.750000	0.001583
2531.500000	0.001596
2531.250000	0.001606
2531.000000	0.001610

2530.750000	0.001609
2530.500000	0.001603
2530.250000	0.001594
2530.000000	0.001585
2529.750000	0.001577
2529.500000	0.001573
2529.250000	0.001574
2529.000000	0.001580
2528.750000	0.001590
2528.500000	0.001602
2528.250000	0.001614
2528.000000	0.001621
2527.750000	0.001622
2527.500000	0.001615
2527.250000	0.001600
2527.000000	0.001582
2526.750000	0.001564
2526.500000	0.001551
2526.250000	0.001547
2526.000000	0.001552
2525.750000	0.001566
2525.500000	0.001585
2525.250000	0.001606
2525.000000	0.001624
2524.750000	0.001635
2524.500000	0.001639

2524.250000	0.001635
2524.000000	0.001626
2523.750000	0.001613
2523.500000	0.001600
2523.250000	0.001589
2523.000000	0.001581
2522.750000	0.001578
2522.500000	0.001578
2522.250000	0.001581
2522.000000	0.001583
2521.750000	0.001583
2521.500000	0.001580
2521.250000	0.001575
2521.000000	0.001568
2520.750000	0.001561
2520.500000	0.001556
2520.250000	0.001553
2520.000000	0.001554
2519.750000	0.001555
2519.500000	0.001557
2519.250000	0.001556
2519.000000	0.001552
2518.750000	0.001545
2518.500000	0.001537
2518.250000	0.001527
2518.000000	0.001520

2517.750000	0.001515
2517.500000	0.001513
2517.250000	0.001515
2517.000000	0.001520
2516.750000	0.001528
2516.500000	0.001537
2516.250000	0.001548
2516.000000	0.001560
2515.750000	0.001573
2515.500000	0.001585
2515.250000	0.001596
2515.000000	0.001604
2514.750000	0.001608
2514.500000	0.001607
2514.250000	0.001602
2514.000000	0.001592
2513.750000	0.001580
2513.500000	0.001568
2513.250000	0.001557
2513.000000	0.001551
2512.750000	0.001550
2512.500000	0.001554
2512.250000	0.001560
2512.000000	0.001567
2511.750000	0.001569
2511.500000	0.001566

2511.250000	0.001554
2511.000000	0.001536
2510.750000	0.001513
2510.500000	0.001490
2510.250000	0.001472
2510.000000	0.001463
2509.750000	0.001465
2509.500000	0.001478
2509.250000	0.001499
2509.000000	0.001525
2508.750000	0.001551
2508.500000	0.001572
2508.250000	0.001584
2508.000000	0.001586
2507.750000	0.001577
2507.500000	0.001562
2507.250000	0.001542
2507.000000	0.001521
2506.750000	0.001504
2506.500000	0.001491
2506.250000	0.001485
2506.000000	0.001483
2505.750000	0.001485
2505.500000	0.001489
2505.250000	0.001494
2505.000000	0.001499

2504.750000	0.001505
2504.500000	0.001512
2504.250000	0.001520
2504.000000	0.001529
2503.750000	0.001539
2503.500000	0.001550
2503.250000	0.001560
2503.000000	0.001568
2502.750000	0.001575
2502.500000	0.001579
2502.250000	0.001579
2502.000000	0.001577
2501.750000	0.001572
2501.500000	0.001564
2501.250000	0.001555
2501.000000	0.001547
2500.750000	0.001540
2500.500000	0.001536
2500.250000	0.001536
2500.000000	0.001539
2499.750000	0.001545
2499.500000	0.001552
2499.250000	0.001558
2499.000000	0.001562
2498.750000	0.001563
2498.500000	0.001561

2498.250000	0.001555
2498.000000	0.001548
2497.750000	0.001541
2497.500000	0.001535
2497.250000	0.001532
2497.000000	0.001533
2496.750000	0.001538
2496.500000	0.001548
2496.250000	0.001561
2496.000000	0.001574
2495.750000	0.001587
2495.500000	0.001597
2495.250000	0.001602
2495.000000	0.001603
2494.750000	0.001600
2494.500000	0.001594
2494.250000	0.001586
2494.000000	0.001577
2493.750000	0.001568
2493.500000	0.001558
2493.250000	0.001549
2493.000000	0.001540
2492.750000	0.001533
2492.500000	0.001529
2492.250000	0.001529
2492.000000	0.001532

2491.750000	0.001538
2491.500000	0.001546
2491.250000	0.001555
2491.000000	0.001563
2490.750000	0.001569
2490.500000	0.001573
2490.250000	0.001574
2490.000000	0.001574
2489.750000	0.001573
2489.500000	0.001569
2489.250000	0.001564
2489.000000	0.001557
2488.750000	0.001548
2488.500000	0.001539
2488.250000	0.001529
2488.000000	0.001521
2487.750000	0.001516
2487.500000	0.001512
2487.250000	0.001511
2487.000000	0.001512
2486.750000	0.001515
2486.500000	0.001520
2486.250000	0.001526
2486.000000	0.001534
2485.750000	0.001543
2485.500000	0.001552

2485.250000	0.001561
2485.000000	0.001567
2484.750000	0.001571
2484.500000	0.001571
2484.250000	0.001569
2484.000000	0.001565
2483.750000	0.001561
2483.500000	0.001556
2483.250000	0.001551
2483.000000	0.001545
2482.750000	0.001537
2482.500000	0.001524
2482.250000	0.001508
2482.000000	0.001488
2481.750000	0.001469
2481.500000	0.001453
2481.250000	0.001443
2481.000000	0.001441
2480.750000	0.001449
2480.500000	0.001464
2480.250000	0.001484
2480.000000	0.001506
2479.750000	0.001526
2479.500000	0.001541
2479.250000	0.001552
2479.000000	0.001558

2478.750000	0.001560
2478.500000	0.001560
2478.250000	0.001559
2478.000000	0.001558
2477.750000	0.001557
2477.500000	0.001556
2477.250000	0.001555
2477.000000	0.001552
2476.750000	0.001549
2476.500000	0.001544
2476.250000	0.001537
2476.000000	0.001530
2475.750000	0.001523
2475.500000	0.001517
2475.250000	0.001514
2475.000000	0.001515
2474.750000	0.001519
2474.500000	0.001525
2474.250000	0.001531
2474.000000	0.001536
2473.750000	0.001538
2473.500000	0.001535
2473.250000	0.001527
2473.000000	0.001516
2472.750000	0.001502
2472.500000	0.001488

2472.250000	0.001475
2472.000000	0.001464
2471.750000	0.001457
2471.500000	0.001453
2471.250000	0.001453
2471.000000	0.001456
2470.750000	0.001461
2470.500000	0.001468
2470.250000	0.001477
2470.000000	0.001486
2469.750000	0.001494
2469.500000	0.001500
2469.250000	0.001504
2469.000000	0.001505
2468.750000	0.001503
2468.500000	0.001499
2468.250000	0.001495
2468.000000	0.001491
2467.750000	0.001489
2467.500000	0.001488
2467.250000	0.001489
2467.000000	0.001492
2466.750000	0.001495
2466.500000	0.001497
2466.250000	0.001499
2466.000000	0.001499

2465.750000	0.001496
2465.500000	0.001492
2465.250000	0.001485
2465.000000	0.001478
2464.750000	0.001469
2464.500000	0.001461
2464.250000	0.001456
2464.000000	0.001454
2463.750000	0.001458
2463.500000	0.001467
2463.250000	0.001483
2463.000000	0.001502
2462.750000	0.001523
2462.500000	0.001542
2462.250000	0.001557
2462.000000	0.001565
2461.750000	0.001567
2461.500000	0.001562
2461.250000	0.001552
2461.000000	0.001541
2460.750000	0.001529
2460.500000	0.001519
2460.250000	0.001510
2460.000000	0.001503
2459.750000	0.001496
2459.500000	0.001491

2459.250000	0.001488
2459.000000	0.001487
2458.750000	0.001489
2458.500000	0.001494
2458.250000	0.001501
2458.000000	0.001510
2457.750000	0.001519
2457.500000	0.001527
2457.250000	0.001535
2457.000000	0.001544
2456.750000	0.001555
2456.500000	0.001568
2456.250000	0.001583
2456.000000	0.001597
2455.750000	0.001609
2455.500000	0.001616
2455.250000	0.001618
2455.000000	0.001613
2454.750000	0.001606
2454.500000	0.001597
2454.250000	0.001590
2454.000000	0.001586
2453.750000	0.001586
2453.500000	0.001590
2453.250000	0.001594
2453.000000	0.001597

2452.750000	0.001597
2452.500000	0.001591
2452.250000	0.001581
2452.000000	0.001565
2451.750000	0.001545
2451.500000	0.001524
2451.250000	0.001502
2451.000000	0.001483
2450.750000	0.001470
2450.500000	0.001462
2450.250000	0.001462
2450.000000	0.001467
2449.750000	0.001477
2449.500000	0.001488
2449.250000	0.001500
2449.000000	0.001511
2448.750000	0.001520
2448.500000	0.001528
2448.250000	0.001535
2448.000000	0.001540
2447.750000	0.001544
2447.500000	0.001546
2447.250000	0.001546
2447.000000	0.001542
2446.750000	0.001535
2446.500000	0.001526

2446.250000	0.001516
2446.000000	0.001504
2445.750000	0.001491
2445.500000	0.001478
2445.250000	0.001464
2445.000000	0.001448
2444.750000	0.001433
2444.500000	0.001418
2444.250000	0.001405
2444.000000	0.001395
2443.750000	0.001390
2443.500000	0.001389
2443.250000	0.001391
2443.000000	0.001397
2442.750000	0.001404
2442.500000	0.001412
2442.250000	0.001419
2442.000000	0.001424
2441.750000	0.001428
2441.500000	0.001429
2441.250000	0.001430
2441.000000	0.001430
2440.750000	0.001431
2440.500000	0.001434
2440.250000	0.001441
2440.000000	0.001451

2439.750000	0.001463
2439.500000	0.001477
2439.250000	0.001489
2439.000000	0.001498
2438.750000	0.001504
2438.500000	0.001507
2438.250000	0.001506
2438.000000	0.001505
2437.750000	0.001505
2437.500000	0.001505
2437.250000	0.001507
2437.000000	0.001510
2436.750000	0.001513
2436.500000	0.001516
2436.250000	0.001518
2436.000000	0.001519
2435.750000	0.001521
2435.500000	0.001524
2435.250000	0.001529
2435.000000	0.001534
2434.750000	0.001540
2434.500000	0.001544
2434.250000	0.001544
2434.000000	0.001539
2433.750000	0.001529
2433.500000	0.001515

2433.250000	0.001497
2433.000000	0.001478
2432.750000	0.001459
2432.500000	0.001445
2432.250000	0.001435
2432.000000	0.001432
2431.750000	0.001435
2431.500000	0.001444
2431.250000	0.001458
2431.000000	0.001472
2430.750000	0.001486
2430.500000	0.001495
2430.250000	0.001497
2430.000000	0.001492
2429.750000	0.001480
2429.500000	0.001464
2429.250000	0.001445
2429.000000	0.001428
2428.750000	0.001414
2428.500000	0.001404
2428.250000	0.001400
2428.000000	0.001401
2427.750000	0.001405
2427.500000	0.001414
2427.250000	0.001425
2427.000000	0.001437

2426.750000	0.001449
2426.500000	0.001458
2426.250000	0.001461
2426.000000	0.001458
2425.750000	0.001446
2425.500000	0.001429
2425.250000	0.001409
2425.000000	0.001390
2424.750000	0.001375
2424.500000	0.001369
2424.250000	0.001370
2424.000000	0.001377
2423.750000	0.001387
2423.500000	0.001396
2423.250000	0.001401
2423.000000	0.001400
2422.750000	0.001393
2422.500000	0.001384
2422.250000	0.001372
2422.000000	0.001362
2421.750000	0.001353
2421.500000	0.001348
2421.250000	0.001347
2421.000000	0.001350
2420.750000	0.001356
2420.500000	0.001364

2420.250000	0.001372
2420.000000	0.001380
2419.750000	0.001386
2419.500000	0.001387
2419.250000	0.001385
2419.000000	0.001379
2418.750000	0.001372
2418.500000	0.001365
2418.250000	0.001359
2418.000000	0.001355
2417.750000	0.001354
2417.500000	0.001353
2417.250000	0.001354
2417.000000	0.001357
2416.750000	0.001362
2416.500000	0.001370
2416.250000	0.001382
2416.000000	0.001397
2415.750000	0.001415
2415.500000	0.001431
2415.250000	0.001444
2415.000000	0.001451
2414.750000	0.001453
2414.500000	0.001449
2414.250000	0.001443
2414.000000	0.001435

2413.750000	0.001427
2413.500000	0.001421
2413.250000	0.001415
2413.000000	0.001410
2412.750000	0.001404
2412.500000	0.001397
2412.250000	0.001390
2412.000000	0.001382
2411.750000	0.001376
2411.500000	0.001371
2411.250000	0.001369
2411.000000	0.001369
2410.750000	0.001372
2410.500000	0.001378
2410.250000	0.001385
2410.000000	0.001393
2409.750000	0.001400
2409.500000	0.001405
2409.250000	0.001407
2409.000000	0.001405
2408.750000	0.001398
2408.500000	0.001387
2408.250000	0.001372
2408.000000	0.001356
2407.750000	0.001340
2407.500000	0.001324

2407.250000	0.001311
2407.000000	0.001301
2406.750000	0.001294
2406.500000	0.001290
2406.250000	0.001289
2406.000000	0.001292
2405.750000	0.001297
2405.500000	0.001306
2405.250000	0.001316
2405.000000	0.001328
2404.750000	0.001341
2404.500000	0.001352
2404.250000	0.001362
2404.000000	0.001369
2403.750000	0.001373
2403.500000	0.001373
2403.250000	0.001369
2403.000000	0.001363
2402.750000	0.001356
2402.500000	0.001348
2402.250000	0.001341
2402.000000	0.001334
2401.750000	0.001328
2401.500000	0.001323
2401.250000	0.001320
2401.000000	0.001318

2400.750000	0.001319
2400.500000	0.001322
2400.250000	0.001328
2400.000000	0.001335
2399.750000	0.001342
2399.500000	0.001347
2399.250000	0.001349
2399.000000	0.001348
2398.750000	0.001346
2398.500000	0.001342
2398.250000	0.001340
2398.000000	0.001338
2397.750000	0.001338
2397.500000	0.001338
2397.250000	0.001338
2397.000000	0.001335
2396.750000	0.001329
2396.500000	0.001323
2396.250000	0.001316
2396.000000	0.001310
2395.750000	0.001309
2395.500000	0.001311
2395.250000	0.001317
2395.000000	0.001326
2394.750000	0.001335
2394.500000	0.001343

2394.250000	0.001349
2394.000000	0.001354
2393.750000	0.001357
2393.500000	0.001360
2393.250000	0.001362
2393.000000	0.001363
2392.750000	0.001362
2392.500000	0.001357
2392.250000	0.001347
2392.000000	0.001333
2391.750000	0.001315
2391.500000	0.001297
2391.250000	0.001281
2391.000000	0.001270
2390.750000	0.001265
2390.500000	0.001267
2390.250000	0.001275
2390.000000	0.001285
2389.750000	0.001296
2389.500000	0.001304
2389.250000	0.001309
2389.000000	0.001310
2388.750000	0.001308
2388.500000	0.001305
2388.250000	0.001301
2388.000000	0.001296

2387.750000	0.001292
2387.500000	0.001287
2387.250000	0.001280
2387.000000	0.001273
2386.750000	0.001264
2386.500000	0.001253
2386.250000	0.001242
2386.000000	0.001231
2385.750000	0.001220
2385.500000	0.001212
2385.250000	0.001206
2385.000000	0.001204
2384.750000	0.001204
2384.500000	0.001207
2384.250000	0.001211
2384.000000	0.001214
2383.750000	0.001214
2383.500000	0.001210
2383.250000	0.001204
2383.000000	0.001196
2382.750000	0.001189
2382.500000	0.001186
2382.250000	0.001187
2382.000000	0.001192
2381.750000	0.001198
2381.500000	0.001204

2381.250000	0.001205
2381.000000	0.001199
2380.750000	0.001186
2380.500000	0.001167
2380.250000	0.001146
2380.000000	0.001126
2379.750000	0.001109
2379.500000	0.001097
2379.250000	0.001090
2379.000000	0.001087
2378.750000	0.001088
2378.500000	0.001090
2378.250000	0.001095
2378.000000	0.001100
2377.750000	0.001108
2377.500000	0.001116
2377.250000	0.001126
2377.000000	0.001136
2376.750000	0.001145
2376.500000	0.001151
2376.250000	0.001153
2376.000000	0.001150
2375.750000	0.001141
2375.500000	0.001126
2375.250000	0.001106
2375.000000	0.001084

2374.750000	0.001062
2374.500000	0.001044
2374.250000	0.001031
2374.000000	0.001025
2373.750000	0.001026
2373.500000	0.001032
2373.250000	0.001037
2373.000000	0.001040
2372.750000	0.001037
2372.500000	0.001029
2372.250000	0.001017
2372.000000	0.001002
2371.750000	0.000988
2371.500000	0.000979
2371.250000	0.000976
2371.000000	0.000979
2370.750000	0.000988
2370.500000	0.001002
2370.250000	0.001019
2370.000000	0.001040
2369.750000	0.001062
2369.500000	0.001081
2369.250000	0.001097
2369.000000	0.001107
2368.750000	0.001112
2368.500000	0.001111

2368.250000	0.001106
2368.000000	0.001101
2367.750000	0.001098
2367.500000	0.001101
2367.250000	0.001109
2367.000000	0.001120
2366.750000	0.001129
2366.500000	0.001131
2366.250000	0.001124
2366.000000	0.001109
2365.750000	0.001087
2365.500000	0.001060
2365.250000	0.001034
2365.000000	0.001015
2364.750000	0.001006
2364.500000	0.001007
2364.250000	0.001011
2364.000000	0.001014
2363.750000	0.001013
2363.500000	0.001007
2363.250000	0.000997
2363.000000	0.000985
2362.750000	0.000978
2362.500000	0.000983
2362.250000	0.001005
2362.000000	0.001043

2361.750000	0.001092
2361.500000	0.001144
2361.250000	0.001191
2361.000000	0.001226
2360.750000	0.001243
2360.500000	0.001243
2360.250000	0.001229
2360.000000	0.001206
2359.750000	0.001180
2359.500000	0.001156
2359.250000	0.001139
2359.000000	0.001129
2358.750000	0.001124
2358.500000	0.001120
2358.250000	0.001111
2358.000000	0.001098
2357.750000	0.001084
2357.500000	0.001070
2357.250000	0.001060
2357.000000	0.001056
2356.750000	0.001060
2356.500000	0.001073
2356.250000	0.001098
2356.000000	0.001130
2355.750000	0.001162
2355.500000	0.001187

2355.250000	0.001203
2355.000000	0.001213
2354.750000	0.001218
2354.500000	0.001222
2354.250000	0.001225
2354.000000	0.001226
2353.750000	0.001228
2353.500000	0.001231
2353.250000	0.001235
2353.000000	0.001242
2352.750000	0.001251
2352.500000	0.001262
2352.250000	0.001277
2352.000000	0.001294
2351.750000	0.001312
2351.500000	0.001327
2351.250000	0.001339
2351.000000	0.001345
2350.750000	0.001345
2350.500000	0.001337
2350.250000	0.001323
2350.000000	0.001302
2349.750000	0.001279
2349.500000	0.001259
2349.250000	0.001245
2349.000000	0.001237

2348.750000	0.001233
2348.500000	0.001229
2348.250000	0.001224
2348.000000	0.001219
2347.750000	0.001215
2347.500000	0.001211
2347.250000	0.001208
2347.000000	0.001206
2346.750000	0.001206
2346.500000	0.001209
2346.250000	0.001217
2346.000000	0.001231
2345.750000	0.001247
2345.500000	0.001260
2345.250000	0.001267
2345.000000	0.001268
2344.750000	0.001265
2344.500000	0.001261
2344.250000	0.001259
2344.000000	0.001259
2343.750000	0.001261
2343.500000	0.001263
2343.250000	0.001266
2343.000000	0.001269
2342.750000	0.001277
2342.500000	0.001290

2342.250000	0.001305
2342.000000	0.001319
2341.750000	0.001327
2341.500000	0.001327
2341.250000	0.001319
2341.000000	0.001307
2340.750000	0.001295
2340.500000	0.001283
2340.250000	0.001274
2340.000000	0.001267
2339.750000	0.001264
2339.500000	0.001269
2339.250000	0.001281
2339.000000	0.001300
2338.750000	0.001322
2338.500000	0.001341
2338.250000	0.001355
2338.000000	0.001365
2337.750000	0.001372
2337.500000	0.001376
2337.250000	0.001377
2337.000000	0.001372
2336.750000	0.001356
2336.500000	0.001330
2336.250000	0.001299
2336.000000	0.001271

2335.750000	0.001254
2335.500000	0.001252
2335.250000	0.001262
2335.000000	0.001281
2334.750000	0.001301
2334.500000	0.001319
2334.250000	0.001333
2334.000000	0.001342
2333.750000	0.001347
2333.500000	0.001348
2333.250000	0.001343
2333.000000	0.001334
2332.750000	0.001322
2332.500000	0.001310
2332.250000	0.001303
2332.000000	0.001302
2331.750000	0.001311
2331.500000	0.001329
2331.250000	0.001352
2331.000000	0.001377
2330.750000	0.001398
2330.500000	0.001414
2330.250000	0.001424
2330.000000	0.001430
2329.750000	0.001433
2329.500000	0.001436

2329.250000	0.001438
2329.000000	0.001435
2328.750000	0.001428
2328.500000	0.001418
2328.250000	0.001409
2328.000000	0.001406
2327.750000	0.001415
2327.500000	0.001436
2327.250000	0.001471
2327.000000	0.001514
2326.750000	0.001564
2326.500000	0.001620
2326.250000	0.001681
2326.000000	0.001745
2325.750000	0.001808
2325.500000	0.001863
2325.250000	0.001903
2325.000000	0.001923
2324.750000	0.001924
2324.500000	0.001910
2324.250000	0.001890
2324.000000	0.001869
2323.750000	0.001850
2323.500000	0.001830
2323.250000	0.001807
2323.000000	0.001780

2322.750000	0.001751
2322.500000	0.001723
2322.250000	0.001701
2322.000000	0.001686
2321.750000	0.001677
2321.500000	0.001669
2321.250000	0.001660
2321.000000	0.001647
2320.750000	0.001633
2320.500000	0.001624
2320.250000	0.001621
2320.000000	0.001624
2319.750000	0.001630
2319.500000	0.001632
2319.250000	0.001625
2319.000000	0.001610
2318.750000	0.001589
2318.500000	0.001571
2318.250000	0.001559
2318.000000	0.001556
2317.750000	0.001560
2317.500000	0.001564
2317.250000	0.001567
2317.000000	0.001567
2316.750000	0.001566
2316.500000	0.001568

2316.250000	0.001574
2316.000000	0.001583
2315.750000	0.001589
2315.500000	0.001587
2315.250000	0.001573
2315.000000	0.001551
2314.750000	0.001527
2314.500000	0.001510
2314.250000	0.001504
2314.000000	0.001512
2313.750000	0.001528
2313.500000	0.001547
2313.250000	0.001562
2313.000000	0.001568
2312.750000	0.001568
2312.500000	0.001563
2312.250000	0.001557
2312.000000	0.001550
2311.750000	0.001541
2311.500000	0.001524
2311.250000	0.001501
2311.000000	0.001472
2310.750000	0.001443
2310.500000	0.001422
2310.250000	0.001413
2310.000000	0.001418

2309.750000	0.001431
2309.500000	0.001448
2309.250000	0.001460
2309.000000	0.001464
2308.750000	0.001461
2308.500000	0.001456
2308.250000	0.001453
2308.000000	0.001457
2307.750000	0.001466
2307.500000	0.001479
2307.250000	0.001491
2307.000000	0.001498
2306.750000	0.001498
2306.500000	0.001494
2306.250000	0.001489
2306.000000	0.001486
2305.750000	0.001485
2305.500000	0.001486
2305.250000	0.001487
2305.000000	0.001485
2304.750000	0.001481
2304.500000	0.001476
2304.250000	0.001473
2304.000000	0.001476
2303.750000	0.001484
2303.500000	0.001498

2303.250000	0.001514
2303.000000	0.001527
2302.750000	0.001533
2302.500000	0.001532
2302.250000	0.001524
2302.000000	0.001513
2301.750000	0.001501
2301.500000	0.001491
2301.250000	0.001484
2301.000000	0.001480
2300.750000	0.001477
2300.500000	0.001475
2300.250000	0.001473
2300.000000	0.001473
2299.750000	0.001475
2299.500000	0.001481
2299.250000	0.001490
2299.000000	0.001500
2298.750000	0.001509
2298.500000	0.001514
2298.250000	0.001516
2298.000000	0.001516
2297.750000	0.001516
2297.500000	0.001522
2297.250000	0.001534
2297.000000	0.001553

2296.750000	0.001577
2296.500000	0.001601
2296.250000	0.001621
2296.000000	0.001634
2295.750000	0.001639
2295.500000	0.001638
2295.250000	0.001632
2295.000000	0.001625
2294.750000	0.001619
2294.500000	0.001615
2294.250000	0.001612
2294.000000	0.001612
2293.750000	0.001616
2293.500000	0.001624
2293.250000	0.001638
2293.000000	0.001659
2292.750000	0.001683
2292.500000	0.001709
2292.250000	0.001732
2292.000000	0.001749
2291.750000	0.001759
2291.500000	0.001761
2291.250000	0.001758
2291.000000	0.001754
2290.750000	0.001751
2290.500000	0.001752

2290.250000	0.001755
2290.000000	0.001761
2289.750000	0.001766
2289.500000	0.001770
2289.250000	0.001773
2289.000000	0.001774
2288.750000	0.001775
2288.500000	0.001775
2288.250000	0.001775
2288.000000	0.001775
2287.750000	0.001775
2287.500000	0.001775
2287.250000	0.001775
2287.000000	0.001776
2286.750000	0.001779
2286.500000	0.001784
2286.250000	0.001791
2286.000000	0.001799
2285.750000	0.001809
2285.500000	0.001818
2285.250000	0.001827
2285.000000	0.001834
2284.750000	0.001838
2284.500000	0.001837
2284.250000	0.001829
2284.000000	0.001813

2283.750000	0.001790
2283.500000	0.001762
2283.250000	0.001733
2283.000000	0.001707
2282.750000	0.001687
2282.500000	0.001676
2282.250000	0.001674
2282.000000	0.001679
2281.750000	0.001689
2281.500000	0.001701
2281.250000	0.001711
2281.000000	0.001718
2280.750000	0.001722
2280.500000	0.001722
2280.250000	0.001718
2280.000000	0.001711
2279.750000	0.001703
2279.500000	0.001693
2279.250000	0.001684
2279.000000	0.001677
2278.750000	0.001674
2278.500000	0.001674
2278.250000	0.001680
2278.000000	0.001689
2277.750000	0.001701
2277.500000	0.001715

2277.250000	0.001731
2277.000000	0.001745
2276.750000	0.001758
2276.500000	0.001766
2276.250000	0.001769
2276.000000	0.001764
2275.750000	0.001753
2275.500000	0.001736
2275.250000	0.001715
2275.000000	0.001693
2274.750000	0.001674
2274.500000	0.001658
2274.250000	0.001645
2274.000000	0.001637
2273.750000	0.001631
2273.500000	0.001626
2273.250000	0.001622
2273.000000	0.001618
2272.750000	0.001616
2272.500000	0.001616
2272.250000	0.001620
2272.000000	0.001629
2271.750000	0.001642
2271.500000	0.001659
2271.250000	0.001677
2271.000000	0.001694

2270.750000	0.001705
2270.500000	0.001709
2270.250000	0.001703
2270.000000	0.001687
2269.750000	0.001664
2269.500000	0.001638
2269.250000	0.001613
2269.000000	0.001592
2268.750000	0.001580
2268.500000	0.001576
2268.250000	0.001581
2268.000000	0.001591
2267.750000	0.001605
2267.500000	0.001622
2267.250000	0.001640
2267.000000	0.001660
2266.750000	0.001680
2266.500000	0.001701
2266.250000	0.001718
2266.000000	0.001729
2265.750000	0.001732
2265.500000	0.001727
2265.250000	0.001715
2265.000000	0.001699
2264.750000	0.001683
2264.500000	0.001668

2264.250000	0.001655
2264.000000	0.001645
2263.750000	0.001634
2263.500000	0.001621
2263.250000	0.001605
2263.000000	0.001587
2262.750000	0.001567
2262.500000	0.001549
2262.250000	0.001536
2262.000000	0.001529
2261.750000	0.001529
2261.500000	0.001536
2261.250000	0.001551
2261.000000	0.001571
2260.750000	0.001595
2260.500000	0.001621
2260.250000	0.001646
2260.000000	0.001668
2259.750000	0.001685
2259.500000	0.001695
2259.250000	0.001700
2259.000000	0.001703
2258.750000	0.001704
2258.500000	0.001707
2258.250000	0.001712
2258.000000	0.001716

2257.750000	0.001717
2257.500000	0.001711
2257.250000	0.001697
2257.000000	0.001676
2256.750000	0.001650
2256.500000	0.001622
2256.250000	0.001598
2256.000000	0.001579
2255.750000	0.001567
2255.500000	0.001561
2255.250000	0.001558
2255.000000	0.001555
2254.750000	0.001551
2254.500000	0.001544
2254.250000	0.001532
2254.000000	0.001518
2253.750000	0.001504
2253.500000	0.001493
2253.250000	0.001487
2253.000000	0.001489
2252.750000	0.001500
2252.500000	0.001518
2252.250000	0.001541
2252.000000	0.001564
2251.750000	0.001584
2251.500000	0.001597

2251.250000	0.001601
2251.000000	0.001599
2250.750000	0.001592
2250.500000	0.001584
2250.250000	0.001579
2250.000000	0.001578
2249.750000	0.001583
2249.500000	0.001593
2249.250000	0.001606
2249.000000	0.001621
2248.750000	0.001634
2248.500000	0.001643
2248.250000	0.001648
2248.000000	0.001647
2247.750000	0.001640
2247.500000	0.001628
2247.250000	0.001614
2247.000000	0.001600
2246.750000	0.001590
2246.500000	0.001585
2246.250000	0.001586
2246.000000	0.001595
2245.750000	0.001608
2245.500000	0.001623
2245.250000	0.001637
2245.000000	0.001647

2244.750000	0.001653
2244.500000	0.001653
2244.250000	0.001649
2244.000000	0.001641
2243.750000	0.001632
2243.500000	0.001622
2243.250000	0.001613
2243.000000	0.001607
2242.750000	0.001606
2242.500000	0.001609
2242.250000	0.001617
2242.000000	0.001632
2241.750000	0.001650
2241.500000	0.001669
2241.250000	0.001686
2241.000000	0.001697
2240.750000	0.001700
2240.500000	0.001693
2240.250000	0.001678
2240.000000	0.001658
2239.750000	0.001637
2239.500000	0.001620
2239.250000	0.001609
2239.000000	0.001604
2238.750000	0.001603
2238.500000	0.001604

2238.250000	0.001603
2238.000000	0.001599
2237.750000	0.001593
2237.500000	0.001585
2237.250000	0.001579
2237.000000	0.001574
2236.750000	0.001573
2236.500000	0.001575
2236.250000	0.001578
2236.000000	0.001583
2235.750000	0.001588
2235.500000	0.001594
2235.250000	0.001602
2235.000000	0.001610
2234.750000	0.001620
2234.500000	0.001629
2234.250000	0.001637
2234.000000	0.001644
2233.750000	0.001651
2233.500000	0.001658
2233.250000	0.001667
2233.000000	0.001677
2232.750000	0.001686
2232.500000	0.001692
2232.250000	0.001692
2232.000000	0.001682

2231.750000	0.001664
2231.500000	0.001640
2231.250000	0.001613
2231.000000	0.001589
2230.750000	0.001571
2230.500000	0.001561
2230.250000	0.001559
2230.000000	0.001562
2229.750000	0.001570
2229.500000	0.001581
2229.250000	0.001593
2229.000000	0.001606
2228.750000	0.001620
2228.500000	0.001633
2228.250000	0.001642
2228.000000	0.001647
2227.750000	0.001646
2227.500000	0.001640
2227.250000	0.001631
2227.000000	0.001622
2226.750000	0.001616
2226.500000	0.001612
2226.250000	0.001612
2226.000000	0.001613
2225.750000	0.001614
2225.500000	0.001613

2225.250000	0.001611
2225.000000	0.001606
2224.750000	0.001601
2224.500000	0.001597
2224.250000	0.001593
2224.000000	0.001591
2223.750000	0.001591
2223.500000	0.001594
2223.250000	0.001600
2223.000000	0.001611
2222.750000	0.001626
2222.500000	0.001643
2222.250000	0.001658
2222.000000	0.001669
2221.750000	0.001671
2221.500000	0.001663
2221.250000	0.001646
2221.000000	0.001623
2220.750000	0.001599
2220.500000	0.001579
2220.250000	0.001567
2220.000000	0.001565
2219.750000	0.001574
2219.500000	0.001590
2219.250000	0.001613
2219.000000	0.001637

2218.750000	0.001660
2218.500000	0.001677
2218.250000	0.001686
2218.000000	0.001686
2217.750000	0.001676
2217.500000	0.001658
2217.250000	0.001637
2217.000000	0.001615
2216.750000	0.001596
2216.500000	0.001582
2216.250000	0.001574
2216.000000	0.001572
2215.750000	0.001577
2215.500000	0.001588
2215.250000	0.001606
2215.000000	0.001630
2214.750000	0.001660
2214.500000	0.001692
2214.250000	0.001723
2214.000000	0.001746
2213.750000	0.001759
2213.500000	0.001762
2213.250000	0.001756
2213.000000	0.001748
2212.750000	0.001743
2212.500000	0.001745

2212.250000	0.001757
2212.000000	0.001775
2211.750000	0.001792
2211.500000	0.001801
2211.250000	0.001796
2211.000000	0.001772
2210.750000	0.001731
2210.500000	0.001678
2210.250000	0.001622
2210.000000	0.001569
2209.750000	0.001528
2209.500000	0.001500
2209.250000	0.001486
2209.000000	0.001483
2208.750000	0.001486
2208.500000	0.001490
2208.250000	0.001495
2208.000000	0.001499
2207.750000	0.001505
2207.500000	0.001517
2207.250000	0.001539
2207.000000	0.001571
2206.750000	0.001612
2206.500000	0.001658
2206.250000	0.001700
2206.000000	0.001731

2205.750000	0.001746
2205.500000	0.001741
2205.250000	0.001715
2205.000000	0.001674
2204.750000	0.001624
2204.500000	0.001576
2204.250000	0.001539
2204.000000	0.001520
2203.750000	0.001525
2203.500000	0.001555
2203.250000	0.001605
2203.000000	0.001668
2202.750000	0.001734
2202.500000	0.001792
2202.250000	0.001831
2202.000000	0.001847
2201.750000	0.001840
2201.500000	0.001812
2201.250000	0.001773
2201.000000	0.001731
2200.750000	0.001695
2200.500000	0.001670
2200.250000	0.001659
2200.000000	0.001662
2199.750000	0.001674
2199.500000	0.001692

2199.250000	0.001710
2199.000000	0.001723
2198.750000	0.001728
2198.500000	0.001723
2198.250000	0.001707
2198.000000	0.001684
2197.750000	0.001657
2197.500000	0.001633
2197.250000	0.001616
2197.000000	0.001609
2196.750000	0.001611
2196.500000	0.001618
2196.250000	0.001627
2196.000000	0.001633
2195.750000	0.001636
2195.500000	0.001641
2195.250000	0.001651
2195.000000	0.001672
2194.750000	0.001705
2194.500000	0.001745
2194.250000	0.001784
2194.000000	0.001812
2193.750000	0.001820
2193.500000	0.001806
2193.250000	0.001775
2193.000000	0.001736

2192.750000	0.001701
2192.500000	0.001682
2192.250000	0.001683
2192.000000	0.001704
2191.750000	0.001736
2191.500000	0.001769
2191.250000	0.001796
2191.000000	0.001811
2190.750000	0.001816
2190.500000	0.001815
2190.250000	0.001817
2190.000000	0.001827
2189.750000	0.001849
2189.500000	0.001881
2189.250000	0.001919
2189.000000	0.001955
2188.750000	0.001980
2188.500000	0.001988
2188.250000	0.001978
2188.000000	0.001953
2187.750000	0.001918
2187.500000	0.001883
2187.250000	0.001857
2187.000000	0.001845
2186.750000	0.001850
2186.500000	0.001869

2186.250000	0.001896
2186.000000	0.001927
2185.750000	0.001953
2185.500000	0.001972
2185.250000	0.001984
2185.000000	0.001988
2184.750000	0.001987
2184.500000	0.001982
2184.250000	0.001973
2184.000000	0.001960
2183.750000	0.001943
2183.500000	0.001922
2183.250000	0.001897
2183.000000	0.001868
2182.750000	0.001836
2182.500000	0.001800
2182.250000	0.001758
2182.000000	0.001712
2181.750000	0.001661
2181.500000	0.001611
2181.250000	0.001567
2181.000000	0.001535
2180.750000	0.001519
2180.500000	0.001520
2180.250000	0.001538
2180.000000	0.001568

2179.750000	0.001606
2179.500000	0.001649
2179.250000	0.001695
2179.000000	0.001742
2178.750000	0.001789
2178.500000	0.001832
2178.250000	0.001866
2178.000000	0.001886
2177.750000	0.001889
2177.500000	0.001873
2177.250000	0.001842
2177.000000	0.001804
2176.750000	0.001765
2176.500000	0.001731
2176.250000	0.001705
2176.000000	0.001683
2175.750000	0.001665
2175.500000	0.001646
2175.250000	0.001624
2175.000000	0.001601
2174.750000	0.001580
2174.500000	0.001564
2174.250000	0.001554
2174.000000	0.001551
2173.750000	0.001556
2173.500000	0.001565

2173.250000	0.001579
2173.000000	0.001597
2172.750000	0.001619
2172.500000	0.001642
2172.250000	0.001666
2172.000000	0.001686
2171.750000	0.001701
2171.500000	0.001711
2171.250000	0.001715
2171.000000	0.001719
2170.750000	0.001725
2170.500000	0.001738
2170.250000	0.001759
2170.000000	0.001789
2169.750000	0.001827
2169.500000	0.001872
2169.250000	0.001921
2169.000000	0.001975
2168.750000	0.002030
2168.500000	0.002084
2168.250000	0.002132
2168.000000	0.002170
2167.750000	0.002194
2167.500000	0.002204
2167.250000	0.002202
2167.000000	0.002194

2166.750000	0.002185
2166.500000	0.002180
2166.250000	0.002181
2166.000000	0.002185
2165.750000	0.002189
2165.500000	0.002190
2165.250000	0.002184
2165.000000	0.002174
2164.750000	0.002164
2164.500000	0.002159
2164.250000	0.002164
2164.000000	0.002181
2163.750000	0.002211
2163.500000	0.002254
2163.250000	0.002309
2163.000000	0.002377
2162.750000	0.002457
2162.500000	0.002546
2162.250000	0.002640
2162.000000	0.002730
2161.750000	0.002805
2161.500000	0.002853
2161.250000	0.002862
2161.000000	0.002823
2160.750000	0.002726
2160.500000	0.002571

2160.250000	0.002357
2160.000000	0.002094
2159.750000	0.001796
2159.500000	0.001486
2159.250000	0.001189
2159.000000	0.000932
2158.750000	0.000736
2158.500000	0.000611
2158.250000	0.000554
2158.000000	0.000553
2157.750000	0.000588
2157.500000	0.000641
2157.250000	0.000695
2157.000000	0.000743
2156.750000	0.000782
2156.500000	0.000813
2156.250000	0.000837
2156.000000	0.000852
2155.750000	0.000858
2155.500000	0.000853
2155.250000	0.000839
2155.000000	0.000821
2154.750000	0.000809
2154.500000	0.000810
2154.250000	0.000832
2154.000000	0.000876

2153.750000	0.000937
2153.500000	0.001006
2153.250000	0.001073
2153.000000	0.001126
2152.750000	0.001158
2152.500000	0.001166
2152.250000	0.001154
2152.000000	0.001126
2151.750000	0.001095
2151.500000	0.001070
2151.250000	0.001060
2151.000000	0.001071
2150.750000	0.001104
2150.500000	0.001154
2150.250000	0.001211
2150.000000	0.001266
2149.750000	0.001307
2149.500000	0.001328
2149.250000	0.001329
2149.000000	0.001312
2148.750000	0.001284
2148.500000	0.001253
2148.250000	0.001222
2148.000000	0.001194
2147.750000	0.001169
2147.500000	0.001145

2147.250000	0.001122
2147.000000	0.001100
2146.750000	0.001081
2146.500000	0.001070
2146.250000	0.001066
2146.000000	0.001069
2145.750000	0.001077
2145.500000	0.001085
2145.250000	0.001089
2145.000000	0.001088
2144.750000	0.001084
2144.500000	0.001080
2144.250000	0.001079
2144.000000	0.001084
2143.750000	0.001094
2143.500000	0.001107
2143.250000	0.001119
2143.000000	0.001127
2142.750000	0.001127
2142.500000	0.001119
2142.250000	0.001103
2142.000000	0.001084
2141.750000	0.001065
2141.500000	0.001049
2141.250000	0.001039
2141.000000	0.001037

2140.750000	0.001044
2140.500000	0.001058
2140.250000	0.001078
2140.000000	0.001101
2139.750000	0.001125
2139.500000	0.001148
2139.250000	0.001167
2139.000000	0.001183
2138.750000	0.001195
2138.500000	0.001205
2138.250000	0.001211
2138.000000	0.001215
2137.750000	0.001214
2137.500000	0.001208
2137.250000	0.001194
2137.000000	0.001173
2136.750000	0.001143
2136.500000	0.001108
2136.250000	0.001070
2136.000000	0.001032
2135.750000	0.000997
2135.500000	0.000966
2135.250000	0.000941
2135.000000	0.000919
2134.750000	0.000897
2134.500000	0.000875

2134.250000	0.000850
2134.000000	0.000823
2133.750000	0.000797
2133.500000	0.000776
2133.250000	0.000762
2133.000000	0.000758
2132.750000	0.000764
2132.500000	0.000780
2132.250000	0.000802
2132.000000	0.000827
2131.750000	0.000852
2131.500000	0.000875
2131.250000	0.000896
2131.000000	0.000914
2130.750000	0.000931
2130.500000	0.000948
2130.250000	0.000964
2130.000000	0.000980
2129.750000	0.000997
2129.500000	0.001016
2129.250000	0.001037
2129.000000	0.001062
2128.750000	0.001088
2128.500000	0.001112
2128.250000	0.001133
2128.000000	0.001145

2127.750000	0.001148
2127.500000	0.001140
2127.250000	0.001124
2127.000000	0.001106
2126.750000	0.001089
2126.500000	0.001077
2126.250000	0.001074
2126.000000	0.001078
2125.750000	0.001088
2125.500000	0.001102
2125.250000	0.001117
2125.000000	0.001131
2124.750000	0.001144
2124.500000	0.001155
2124.250000	0.001163
2124.000000	0.001169
2123.750000	0.001174
2123.500000	0.001179
2123.250000	0.001184
2123.000000	0.001190
2122.750000	0.001199
2122.500000	0.001209
2122.250000	0.001220
2122.000000	0.001230
2121.750000	0.001238
2121.500000	0.001242

2121.250000	0.001243
2121.000000	0.001241
2120.750000	0.001237
2120.500000	0.001233
2120.250000	0.001229
2120.000000	0.001226
2119.750000	0.001227
2119.500000	0.001230
2119.250000	0.001237
2119.000000	0.001248
2118.750000	0.001262
2118.500000	0.001278
2118.250000	0.001295
2118.000000	0.001310
2117.750000	0.001323
2117.500000	0.001336
2117.250000	0.001349
2117.000000	0.001363
2116.750000	0.001379
2116.500000	0.001396
2116.250000	0.001410
2116.000000	0.001420
2115.750000	0.001422
2115.500000	0.001417
2115.250000	0.001406
2115.000000	0.001394

2114.750000	0.001386
2114.500000	0.001385
2114.250000	0.001394
2114.000000	0.001410
2113.750000	0.001432
2113.500000	0.001453
2113.250000	0.001470
2113.000000	0.001479
2112.750000	0.001479
2112.500000	0.001470
2112.250000	0.001454
2112.000000	0.001434
2111.750000	0.001413
2111.500000	0.001394
2111.250000	0.001381
2111.000000	0.001376
2110.750000	0.001378
2110.500000	0.001386
2110.250000	0.001397
2110.000000	0.001409
2109.750000	0.001418
2109.500000	0.001423
2109.250000	0.001425
2109.000000	0.001425
2108.750000	0.001425
2108.500000	0.001425

2108.250000	0.001423
2108.000000	0.001419
2107.750000	0.001410
2107.500000	0.001395
2107.250000	0.001378
2107.000000	0.001362
2106.750000	0.001350
2106.500000	0.001347
2106.250000	0.001353
2106.000000	0.001366
2105.750000	0.001381
2105.500000	0.001393
2105.250000	0.001399
2105.000000	0.001396
2104.750000	0.001386
2104.500000	0.001371
2104.250000	0.001355
2104.000000	0.001341
2103.750000	0.001330
2103.500000	0.001323
2103.250000	0.001319
2103.000000	0.001317
2102.750000	0.001315
2102.500000	0.001312
2102.250000	0.001309
2102.000000	0.001307

2101.750000	0.001305
2101.500000	0.001305
2101.250000	0.001308
2101.000000	0.001315
2100.750000	0.001324
2100.500000	0.001334
2100.250000	0.001342
2100.000000	0.001346
2099.750000	0.001345
2099.500000	0.001340
2099.250000	0.001334
2099.000000	0.001330
2098.750000	0.001333
2098.500000	0.001343
2098.250000	0.001359
2098.000000	0.001377
2097.750000	0.001392
2097.500000	0.001400
2097.250000	0.001397
2097.000000	0.001382
2096.750000	0.001357
2096.500000	0.001326
2096.250000	0.001294
2096.000000	0.001264
2095.750000	0.001240
2095.500000	0.001222

2095.250000	0.001212
2095.000000	0.001209
2094.750000	0.001212
2094.500000	0.001216
2094.250000	0.001221
2094.000000	0.001224
2093.750000	0.001222
2093.500000	0.001216
2093.250000	0.001208
2093.000000	0.001199
2092.750000	0.001191
2092.500000	0.001188
2092.250000	0.001188
2092.000000	0.001193
2091.750000	0.001199
2091.500000	0.001207
2091.250000	0.001214
2091.000000	0.001221
2090.750000	0.001228
2090.500000	0.001235
2090.250000	0.001242
2090.000000	0.001251
2089.750000	0.001259
2089.500000	0.001268
2089.250000	0.001277
2089.000000	0.001287

2088.750000	0.001297
2088.500000	0.001306
2088.250000	0.001313
2088.000000	0.001316
2087.750000	0.001316
2087.500000	0.001313
2087.250000	0.001310
2087.000000	0.001311
2086.750000	0.001319
2086.500000	0.001336
2086.250000	0.001361
2086.000000	0.001390
2085.750000	0.001420
2085.500000	0.001445
2085.250000	0.001461
2085.000000	0.001466
2084.750000	0.001461
2084.500000	0.001448
2084.250000	0.001430
2084.000000	0.001410
2083.750000	0.001392
2083.500000	0.001379
2083.250000	0.001371
2083.000000	0.001370
2082.750000	0.001374
2082.500000	0.001380

2082.250000	0.001385
2082.000000	0.001385
2081.750000	0.001378
2081.500000	0.001365
2081.250000	0.001348
2081.000000	0.001332
2080.750000	0.001321
2080.500000	0.001320
2080.250000	0.001328
2080.000000	0.001344
2079.750000	0.001363
2079.500000	0.001379
2079.250000	0.001388
2079.000000	0.001388
2078.750000	0.001378
2078.500000	0.001362
2078.250000	0.001342
2078.000000	0.001324
2077.750000	0.001310
2077.500000	0.001302
2077.250000	0.001302
2077.000000	0.001308
2076.750000	0.001321
2076.500000	0.001337
2076.250000	0.001353
2076.000000	0.001368

2075.750000	0.001381
2075.500000	0.001391
2075.250000	0.001401
2075.000000	0.001413
2074.750000	0.001429
2074.500000	0.001449
2074.250000	0.001471
2074.000000	0.001490
2073.750000	0.001501
2073.500000	0.001502
2073.250000	0.001492
2073.000000	0.001474
2072.750000	0.001452
2072.500000	0.001431
2072.250000	0.001415
2072.000000	0.001405
2071.750000	0.001402
2071.500000	0.001403
2071.250000	0.001407
2071.000000	0.001412
2070.750000	0.001418
2070.500000	0.001423
2070.250000	0.001424
2070.000000	0.001419
2069.750000	0.001406
2069.500000	0.001386

2069.250000	0.001361
2069.000000	0.001338
2068.750000	0.001323
2068.500000	0.001320
2068.250000	0.001330
2068.000000	0.001348
2067.750000	0.001368
2067.500000	0.001381
2067.250000	0.001382
2067.000000	0.001370
2066.750000	0.001348
2066.500000	0.001323
2066.250000	0.001301
2066.000000	0.001288
2065.750000	0.001283
2065.500000	0.001285
2065.250000	0.001290
2065.000000	0.001293
2064.750000	0.001293
2064.500000	0.001288
2064.250000	0.001282
2064.000000	0.001274
2063.750000	0.001269
2063.500000	0.001265
2063.250000	0.001263
2063.000000	0.001263

2062.750000	0.001264
2062.500000	0.001266
2062.250000	0.001268
2062.000000	0.001268
2061.750000	0.001266
2061.500000	0.001262
2061.250000	0.001257
2061.000000	0.001255
2060.750000	0.001258
2060.500000	0.001271
2060.250000	0.001293
2060.000000	0.001322
2059.750000	0.001355
2059.500000	0.001386
2059.250000	0.001409
2059.000000	0.001424
2058.750000	0.001429
2058.500000	0.001427
2058.250000	0.001420
2058.000000	0.001411
2057.750000	0.001400
2057.500000	0.001389
2057.250000	0.001380
2057.000000	0.001375
2056.750000	0.001376
2056.500000	0.001383

2056.250000	0.001394
2056.000000	0.001405
2055.750000	0.001410
2055.500000	0.001405
2055.250000	0.001386
2055.000000	0.001354
2054.750000	0.001316
2054.500000	0.001280
2054.250000	0.001256
2054.000000	0.001251
2053.750000	0.001269
2053.500000	0.001311
2053.250000	0.001375
2053.000000	0.001457
2052.750000	0.001553
2052.500000	0.001656
2052.250000	0.001759
2052.000000	0.001855
2051.750000	0.001934
2051.500000	0.001988
2051.250000	0.002010
2051.000000	0.001998
2050.750000	0.001954
2050.500000	0.001883
2050.250000	0.001795
2050.000000	0.001698

2049.750000	0.001601
2049.500000	0.001513
2049.250000	0.001437
2049.000000	0.001380
2048.750000	0.001343
2048.500000	0.001328
2048.250000	0.001334
2048.000000	0.001355
2047.750000	0.001385
2047.500000	0.001414
2047.250000	0.001432
2047.000000	0.001435
2046.750000	0.001420
2046.500000	0.001393
2046.250000	0.001359
2046.000000	0.001328
2045.750000	0.001307
2045.500000	0.001302
2045.250000	0.001313
2045.000000	0.001339
2044.750000	0.001375
2044.500000	0.001414
2044.250000	0.001448
2044.000000	0.001469
2043.750000	0.001474
2043.500000	0.001460

2043.250000	0.001430
2043.000000	0.001390
2042.750000	0.001347
2042.500000	0.001311
2042.250000	0.001287
2042.000000	0.001281
2041.750000	0.001293
2041.500000	0.001320
2041.250000	0.001356
2041.000000	0.001394
2040.750000	0.001426
2040.500000	0.001449
2040.250000	0.001458
2040.000000	0.001453
2039.750000	0.001438
2039.500000	0.001419
2039.250000	0.001403
2039.000000	0.001399
2038.750000	0.001412
2038.500000	0.001444
2038.250000	0.001492
2038.000000	0.001551
2037.750000	0.001610
2037.500000	0.001658
2037.250000	0.001690
2037.000000	0.001700

2036.750000	0.001688
2036.500000	0.001658
2036.250000	0.001612
2036.000000	0.001556
2035.750000	0.001492
2035.500000	0.001427
2035.250000	0.001366
2035.000000	0.001314
2034.750000	0.001277
2034.500000	0.001259
2034.250000	0.001261
2034.000000	0.001280
2033.750000	0.001313
2033.500000	0.001356
2033.250000	0.001403
2033.000000	0.001453
2032.750000	0.001500
2032.500000	0.001542
2032.250000	0.001570
2032.000000	0.001581
2031.750000	0.001569
2031.500000	0.001535
2031.250000	0.001485
2031.000000	0.001433
2030.750000	0.001390
2030.500000	0.001370

2030.250000	0.001376
2030.000000	0.001407
2029.750000	0.001450
2029.500000	0.001494
2029.250000	0.001524
2029.000000	0.001534
2028.750000	0.001522
2028.500000	0.001494
2028.250000	0.001457
2028.000000	0.001419
2027.750000	0.001387
2027.500000	0.001363
2027.250000	0.001348
2027.000000	0.001340
2026.750000	0.001339
2026.500000	0.001342
2026.250000	0.001347
2026.000000	0.001350
2025.750000	0.001350
2025.500000	0.001344
2025.250000	0.001333
2025.000000	0.001318
2024.750000	0.001303
2024.500000	0.001288
2024.250000	0.001274
2024.000000	0.001259

2023.750000	0.001241
2023.500000	0.001221
2023.250000	0.001201
2023.000000	0.001185
2022.750000	0.001179
2022.500000	0.001183
2022.250000	0.001195
2022.000000	0.001207
2021.750000	0.001210
2021.500000	0.001194
2021.250000	0.001159
2021.000000	0.001110
2020.750000	0.001060
2020.500000	0.001023
2020.250000	0.001008
2020.000000	0.001016
2019.750000	0.001040
2019.500000	0.001070
2019.250000	0.001094
2019.000000	0.001109
2018.750000	0.001117
2018.500000	0.001128
2018.250000	0.001149
2018.000000	0.001182
2017.750000	0.001223
2017.500000	0.001260

2017.250000	0.001277
2017.000000	0.001267
2016.750000	0.001229
2016.500000	0.001173
2016.250000	0.001115
2016.000000	0.001072
2015.750000	0.001054
2015.500000	0.001062
2015.250000	0.001091
2015.000000	0.001129
2014.750000	0.001166
2014.500000	0.001194
2014.250000	0.001209
2014.000000	0.001211
2013.750000	0.001203
2013.500000	0.001189
2013.250000	0.001174
2013.000000	0.001162
2012.750000	0.001160
2012.500000	0.001173
2012.250000	0.001205
2012.000000	0.001255
2011.750000	0.001317
2011.500000	0.001380
2011.250000	0.001431
2011.000000	0.001459

2010.750000	0.001456
2010.500000	0.001420
2010.250000	0.001357
2010.000000	0.001278
2009.750000	0.001195
2009.500000	0.001122
2009.250000	0.001066
2009.000000	0.001033
2008.750000	0.001022
2008.500000	0.001026
2008.250000	0.001037
2008.000000	0.001045
2007.750000	0.001041
2007.500000	0.001024
2007.250000	0.000996
2007.000000	0.000963
2006.750000	0.000936
2006.500000	0.000921
2006.250000	0.000919
2006.000000	0.000930
2005.750000	0.000945
2005.500000	0.000958
2005.250000	0.000963
2005.000000	0.000961
2004.750000	0.000953
2004.500000	0.000944

2004.250000	0.000936
2004.000000	0.000930
2003.750000	0.000923
2003.500000	0.000908
2003.250000	0.000884
2003.000000	0.000849
2002.750000	0.000808
2002.500000	0.000768
2002.250000	0.000739
2002.000000	0.000724
2001.750000	0.000727
2001.500000	0.000744
2001.250000	0.000771
2001.000000	0.000804
2000.750000	0.000840
2000.500000	0.000879
2000.250000	0.000922
2000.000000	0.000968
1999.750000	0.001014
1999.500000	0.001058
1999.250000	0.001092
1999.000000	0.001112
1998.750000	0.001117
1998.500000	0.001109
1998.250000	0.001095
1998.000000	0.001083

1997.750000	0.001082
1997.500000	0.001094
1997.250000	0.001120
1997.000000	0.001155
1996.750000	0.001192
1996.500000	0.001223
1996.250000	0.001242
1996.000000	0.001250
1995.750000	0.001247
1995.500000	0.001240
1995.250000	0.001235
1995.000000	0.001234
1994.750000	0.001240
1994.500000	0.001249
1994.250000	0.001260
1994.000000	0.001268
1993.750000	0.001271
1993.500000	0.001265
1993.250000	0.001251
1993.000000	0.001227
1992.750000	0.001194
1992.500000	0.001152
1992.250000	0.001102
1992.000000	0.001046
1991.750000	0.000988
1991.500000	0.000936

1991.250000	0.000902
1991.000000	0.000898
1990.750000	0.000931
1990.500000	0.001000
1990.250000	0.001096
1990.000000	0.001206
1989.750000	0.001310
1989.500000	0.001388
1989.250000	0.001427
1989.000000	0.001419
1988.750000	0.001369
1988.500000	0.001289
1988.250000	0.001198
1988.000000	0.001116
1987.750000	0.001060
1987.500000	0.001037
1987.250000	0.001046
1987.000000	0.001082
1986.750000	0.001136
1986.500000	0.001196
1986.250000	0.001256
1986.000000	0.001307
1985.750000	0.001349
1985.500000	0.001380
1985.250000	0.001400
1985.000000	0.001411

1984.750000	0.001415
1984.500000	0.001415
1984.250000	0.001412
1984.000000	0.001409
1983.750000	0.001407
1983.500000	0.001406
1983.250000	0.001409
1983.000000	0.001418
1982.750000	0.001435
1982.500000	0.001460
1982.250000	0.001497
1982.000000	0.001542
1981.750000	0.001593
1981.500000	0.001646
1981.250000	0.001699
1981.000000	0.001751
1980.750000	0.001799
1980.500000	0.001844
1980.250000	0.001883
1980.000000	0.001908
1979.750000	0.001910
1979.500000	0.001880
1979.250000	0.001811
1979.000000	0.001704
1978.750000	0.001568
1978.500000	0.001418

1978.250000	0.001275
1978.000000	0.001154
1977.750000	0.001068
1977.500000	0.001017
1977.250000	0.000997
1977.000000	0.001001
1976.750000	0.001020
1976.500000	0.001048
1976.250000	0.001081
1976.000000	0.001114
1975.750000	0.001142
1975.500000	0.001158
1975.250000	0.001156
1975.000000	0.001130
1974.750000	0.001082
1974.500000	0.001017
1974.250000	0.000947
1974.000000	0.000885
1973.750000	0.000845
1973.500000	0.000833
1973.250000	0.000852
1973.000000	0.000897
1972.750000	0.000956
1972.500000	0.001017
1972.250000	0.001067
1972.000000	0.001095

1971.750000	0.001094
1971.500000	0.001067
1971.250000	0.001021
1971.000000	0.000968
1970.750000	0.000920
1970.500000	0.000886
1970.250000	0.000874
1970.000000	0.000884
1969.750000	0.000911
1969.500000	0.000947
1969.250000	0.000985
1969.000000	0.001017
1968.750000	0.001038
1968.500000	0.001044
1968.250000	0.001034
1968.000000	0.001008
1967.750000	0.000967
1967.500000	0.000915
1967.250000	0.000858
1967.000000	0.000805
1966.750000	0.000768
1966.500000	0.000753
1966.250000	0.000767
1966.000000	0.000806
1965.750000	0.000865
1965.500000	0.000932

1965.250000	0.000997
1965.000000	0.001054
1964.750000	0.001098
1964.500000	0.001128
1964.250000	0.001146
1964.000000	0.001156
1963.750000	0.001160
1963.500000	0.001157
1963.250000	0.001149
1963.000000	0.001137
1962.750000	0.001121
1962.500000	0.001103
1962.250000	0.001085
1962.000000	0.001066
1961.750000	0.001046
1961.500000	0.001023
1961.250000	0.000996
1961.000000	0.000965
1960.750000	0.000930
1960.500000	0.000894
1960.250000	0.000857
1960.000000	0.000821
1959.750000	0.000786
1959.500000	0.000753
1959.250000	0.000721
1959.000000	0.000692

1958.750000	0.000669
1958.500000	0.000656
1958.250000	0.000657
1958.000000	0.000675
1957.750000	0.000708
1957.500000	0.000752
1957.250000	0.000804
1957.000000	0.000855
1956.750000	0.000903
1956.500000	0.000944
1956.250000	0.000978
1956.000000	0.001005
1955.750000	0.001026
1955.500000	0.001042
1955.250000	0.001053
1955.000000	0.001059
1954.750000	0.001059
1954.500000	0.001054
1954.250000	0.001045
1954.000000	0.001032
1953.750000	0.001015
1953.500000	0.000996
1953.250000	0.000976
1953.000000	0.000958
1952.750000	0.000943
1952.500000	0.000935

1952.250000	0.000933
1952.000000	0.000935
1951.750000	0.000939
1951.500000	0.000941
1951.250000	0.000936
1951.000000	0.000922
1950.750000	0.000899
1950.500000	0.000871
1950.250000	0.000842
1950.000000	0.000816
1949.750000	0.000797
1949.500000	0.000785
1949.250000	0.000782
1949.000000	0.000786
1948.750000	0.000797
1948.500000	0.000814
1948.250000	0.000835
1948.000000	0.000860
1947.750000	0.000885
1947.500000	0.000904
1947.250000	0.000914
1947.000000	0.000912
1946.750000	0.000894
1946.500000	0.000859
1946.250000	0.000818
1946.000000	0.000794

1945.750000	0.000797
1945.500000	0.000815
1945.250000	0.000828
1945.000000	0.000836
1944.750000	0.000846
1944.500000	0.000859
1944.250000	0.000868
1944.000000	0.000869
1943.750000	0.000866
1943.500000	0.000865
1943.250000	0.000865
1943.000000	0.000872
1942.750000	0.000886
1942.500000	0.000907
1942.250000	0.000930
1942.000000	0.000950
1941.750000	0.000963
1941.500000	0.000969
1941.250000	0.000968
1941.000000	0.000963
1940.750000	0.000956
1940.500000	0.000950
1940.250000	0.000947
1940.000000	0.000946
1939.750000	0.000947
1939.500000	0.000952

1939.250000	0.000963
1939.000000	0.000980
1938.750000	0.000997
1938.500000	0.001012
1938.250000	0.001018
1938.000000	0.001014
1937.750000	0.000998
1937.500000	0.000971
1937.250000	0.000936
1937.000000	0.000898
1936.750000	0.000861
1936.500000	0.000830
1936.250000	0.000808
1936.000000	0.000796
1935.750000	0.000791
1935.500000	0.000790
1935.250000	0.000791
1935.000000	0.000792
1934.750000	0.000793
1934.500000	0.000796
1934.250000	0.000801
1934.000000	0.000809
1933.750000	0.000816
1933.500000	0.000821
1933.250000	0.000823
1933.000000	0.000822

1932.750000	0.000819
1932.500000	0.000819
1932.250000	0.000824
1932.000000	0.000836
1931.750000	0.000856
1931.500000	0.000881
1931.250000	0.000907
1931.000000	0.000931
1930.750000	0.000948
1930.500000	0.000957
1930.250000	0.000957
1930.000000	0.000948
1929.750000	0.000934
1929.500000	0.000918
1929.250000	0.000902
1929.000000	0.000890
1928.750000	0.000883
1928.500000	0.000881
1928.250000	0.000882
1928.000000	0.000883
1927.750000	0.000882
1927.500000	0.000876
1927.250000	0.000867
1927.000000	0.000859
1926.750000	0.000856
1926.500000	0.000863

1926.250000	0.000880
1926.000000	0.000905
1925.750000	0.000936
1925.500000	0.000967
1925.250000	0.000995
1925.000000	0.001017
1924.750000	0.001034
1924.500000	0.001043
1924.250000	0.001041
1924.000000	0.001024
1923.750000	0.000988
1923.500000	0.000935
1923.250000	0.000866
1923.000000	0.000787
1922.750000	0.000715
1922.500000	0.000670
1922.250000	0.000650
1922.000000	0.000646
1921.750000	0.000672
1921.500000	0.000761
1921.250000	0.000896
1921.000000	0.000998
1920.750000	0.001013
1920.500000	0.000970
1920.250000	0.000938
1920.000000	0.000938

1919.750000	0.000947
1919.500000	0.000953
1919.250000	0.000968
1919.000000	0.000996
1918.750000	0.001028
1918.500000	0.001058
1918.250000	0.001087
1918.000000	0.001112
1917.750000	0.001128
1917.500000	0.001132
1917.250000	0.001126
1917.000000	0.001111
1916.750000	0.001092
1916.500000	0.001072
1916.250000	0.001053
1916.000000	0.001036
1915.750000	0.001018
1915.500000	0.001000
1915.250000	0.000978
1915.000000	0.000953
1914.750000	0.000925
1914.500000	0.000898
1914.250000	0.000874
1914.000000	0.000859
1913.750000	0.000853
1913.500000	0.000854

1913.250000	0.000860
1913.000000	0.000870
1912.750000	0.000884
1912.500000	0.000897
1912.250000	0.000905
1912.000000	0.000911
1911.750000	0.000918
1911.500000	0.000926
1911.250000	0.000933
1911.000000	0.000935
1910.750000	0.000927
1910.500000	0.000910
1910.250000	0.000883
1910.000000	0.000852
1909.750000	0.000825
1909.500000	0.000808
1909.250000	0.000805
1909.000000	0.000815
1908.750000	0.000833
1908.500000	0.000851
1908.250000	0.000865
1908.000000	0.000870
1907.750000	0.000870
1907.500000	0.000870
1907.250000	0.000876
1907.000000	0.000893

1906.750000	0.000921
1906.500000	0.000957
1906.250000	0.000993
1906.000000	0.001019
1905.750000	0.001028
1905.500000	0.001018
1905.250000	0.000991
1905.000000	0.000953
1904.750000	0.000913
1904.500000	0.000881
1904.250000	0.000861
1904.000000	0.000858
1903.750000	0.000869
1903.500000	0.000890
1903.250000	0.000915
1903.000000	0.000937
1902.750000	0.000952
1902.500000	0.000958
1902.250000	0.000953
1902.000000	0.000941
1901.750000	0.000926
1901.500000	0.000912
1901.250000	0.000903
1901.000000	0.000900
1900.750000	0.000904
1900.500000	0.000915

1900.250000	0.000931
1900.000000	0.000949
1899.750000	0.000963
1899.500000	0.000972
1899.250000	0.000972
1899.000000	0.000965
1898.750000	0.000953
1898.500000	0.000939
1898.250000	0.000926
1898.000000	0.000918
1897.750000	0.000916
1897.500000	0.000922
1897.250000	0.000937
1897.000000	0.000958
1896.750000	0.000984
1896.500000	0.001012
1896.250000	0.001034
1896.000000	0.001040
1895.750000	0.001032
1895.500000	0.001016
1895.250000	0.000987
1895.000000	0.000936
1894.750000	0.000892
1894.500000	0.000909
1894.250000	0.000990
1894.000000	0.001068

1893.750000	0.001081
1893.500000	0.001043
1893.250000	0.001008
1893.000000	0.000992
1892.750000	0.000980
1892.500000	0.000963
1892.250000	0.000954
1892.000000	0.000958
1891.750000	0.000966
1891.500000	0.000974
1891.250000	0.000981
1891.000000	0.000988
1890.750000	0.000993
1890.500000	0.000995
1890.250000	0.000999
1890.000000	0.001006
1889.750000	0.001019
1889.500000	0.001036
1889.250000	0.001054
1889.000000	0.001069
1888.750000	0.001077
1888.500000	0.001078
1888.250000	0.001071
1888.000000	0.001059
1887.750000	0.001045
1887.500000	0.001031

1887.250000	0.001019
1887.000000	0.001011
1886.750000	0.001006
1886.500000	0.001004
1886.250000	0.001002
1886.000000	0.001000
1885.750000	0.000997
1885.500000	0.000993
1885.250000	0.000988
1885.000000	0.000983
1884.750000	0.000979
1884.500000	0.000977
1884.250000	0.000977
1884.000000	0.000979
1883.750000	0.000982
1883.500000	0.000983
1883.250000	0.000984
1883.000000	0.000984
1882.750000	0.000983
1882.500000	0.000981
1882.250000	0.000978
1882.000000	0.000976
1881.750000	0.000976
1881.500000	0.000977
1881.250000	0.000977
1881.000000	0.000976

1880.750000	0.000976
1880.500000	0.000977
1880.250000	0.000978
1880.000000	0.000978
1879.750000	0.000975
1879.500000	0.000970
1879.250000	0.000960
1879.000000	0.000948
1878.750000	0.000934
1878.500000	0.000922
1878.250000	0.000914
1878.000000	0.000909
1877.750000	0.000908
1877.500000	0.000908
1877.250000	0.000910
1877.000000	0.000913
1876.750000	0.000916
1876.500000	0.000921
1876.250000	0.000930
1876.000000	0.000942
1875.750000	0.000957
1875.500000	0.000972
1875.250000	0.000986
1875.000000	0.000995
1874.750000	0.000999
1874.500000	0.000998

1874.250000	0.000993
1874.000000	0.000983
1873.750000	0.000969
1873.500000	0.000955
1873.250000	0.000944
1873.000000	0.000940
1872.750000	0.000943
1872.500000	0.000950
1872.250000	0.000958
1872.000000	0.000965
1871.750000	0.000974
1871.500000	0.000992
1871.250000	0.001022
1871.000000	0.001067
1870.750000	0.001124
1870.500000	0.001186
1870.250000	0.001239
1870.000000	0.001270
1869.750000	0.001266
1869.500000	0.001221
1869.250000	0.001135
1869.000000	0.001022
1868.750000	0.000902
1868.500000	0.000797
1868.250000	0.000725
1868.000000	0.000690

1867.750000	0.000687
1867.500000	0.000703
1867.250000	0.000727
1867.000000	0.000749
1866.750000	0.000765
1866.500000	0.000777
1866.250000	0.000787
1866.000000	0.000800
1865.750000	0.000817
1865.500000	0.000839
1865.250000	0.000864
1865.000000	0.000890
1864.750000	0.000913
1864.500000	0.000931
1864.250000	0.000944
1864.000000	0.000950
1863.750000	0.000949
1863.500000	0.000944
1863.250000	0.000941
1863.000000	0.000943
1862.750000	0.000952
1862.500000	0.000963
1862.250000	0.000972
1862.000000	0.000975
1861.750000	0.000969
1861.500000	0.000955

1861.250000	0.000938
1861.000000	0.000921
1860.750000	0.000905
1860.500000	0.000895
1860.250000	0.000891
1860.000000	0.000891
1859.750000	0.000893
1859.500000	0.000895
1859.250000	0.000894
1859.000000	0.000891
1858.750000	0.000885
1858.500000	0.000880
1858.250000	0.000876
1858.000000	0.000876
1857.750000	0.000880
1857.500000	0.000886
1857.250000	0.000893
1857.000000	0.000900
1856.750000	0.000905
1856.500000	0.000909
1856.250000	0.000910
1856.000000	0.000910
1855.750000	0.000910
1855.500000	0.000910
1855.250000	0.000910
1855.000000	0.000911

1854.750000	0.000912
1854.500000	0.000914
1854.250000	0.000916
1854.000000	0.000918
1853.750000	0.000919
1853.500000	0.000918
1853.250000	0.000916
1853.000000	0.000914
1852.750000	0.000911
1852.500000	0.000910
1852.250000	0.000911
1852.000000	0.000913
1851.750000	0.000916
1851.500000	0.000919
1851.250000	0.000919
1851.000000	0.000916
1850.750000	0.000907
1850.500000	0.000894
1850.250000	0.000881
1850.000000	0.000874
1849.750000	0.000874
1849.500000	0.000882
1849.250000	0.000900
1849.000000	0.000926
1848.750000	0.000942
1848.500000	0.000929

1848.250000	0.000902
1848.000000	0.000906
1847.750000	0.000957
1847.500000	0.001016
1847.250000	0.001036
1847.000000	0.001022
1846.750000	0.001005
1846.500000	0.000996
1846.250000	0.000984
1846.000000	0.000966
1845.750000	0.000951
1845.500000	0.000946
1845.250000	0.000952
1845.000000	0.000976
1844.750000	0.001024
1844.500000	0.001099
1844.250000	0.001185
1844.000000	0.001261
1843.750000	0.001310
1843.500000	0.001325
1843.250000	0.001311
1843.000000	0.001278
1842.750000	0.001234
1842.500000	0.001186
1842.250000	0.001139
1842.000000	0.001096

1841.750000	0.001059
1841.500000	0.001027
1841.250000	0.001001
1841.000000	0.000980
1840.750000	0.000963
1840.500000	0.000951
1840.250000	0.000943
1840.000000	0.000938
1839.750000	0.000937
1839.500000	0.000938
1839.250000	0.000939
1839.000000	0.000941
1838.750000	0.000941
1838.500000	0.000940
1838.250000	0.000940
1838.000000	0.000944
1837.750000	0.000948
1837.500000	0.000946
1837.250000	0.000946
1837.000000	0.000958
1836.750000	0.000962
1836.500000	0.000922
1836.250000	0.000841
1836.000000	0.000778
1835.750000	0.000781
1835.500000	0.000829

1835.250000	0.000873
1835.000000	0.000896
1834.750000	0.000913
1834.500000	0.000930
1834.250000	0.000939
1834.000000	0.000937
1833.750000	0.000933
1833.500000	0.000929
1833.250000	0.000920
1833.000000	0.000908
1832.750000	0.000901
1832.500000	0.000905
1832.250000	0.000930
1832.000000	0.000978
1831.750000	0.001051
1831.500000	0.001141
1831.250000	0.001234
1831.000000	0.001309
1830.750000	0.001349
1830.500000	0.001339
1830.250000	0.001271
1830.000000	0.001144
1829.750000	0.000976
1829.500000	0.000797
1829.250000	0.000647
1829.000000	0.000558

1828.750000	0.000541
1828.500000	0.000582
1828.250000	0.000657
1828.000000	0.000742
1827.750000	0.000821
1827.500000	0.000892
1827.250000	0.000958
1827.000000	0.001023
1826.750000	0.001090
1826.500000	0.001154
1826.250000	0.001204
1826.000000	0.001225
1825.750000	0.001205
1825.500000	0.001139
1825.250000	0.001034
1825.000000	0.000910
1824.750000	0.000798
1824.500000	0.000726
1824.250000	0.000709
1824.000000	0.000743
1823.750000	0.000810
1823.500000	0.000882
1823.250000	0.000939
1823.000000	0.000971
1822.750000	0.000978
1822.500000	0.000970

1822.250000	0.000957
1822.000000	0.000949
1821.750000	0.000949
1821.500000	0.000958
1821.250000	0.000971
1821.000000	0.000984
1820.750000	0.000992
1820.500000	0.000995
1820.250000	0.000994
1820.000000	0.000992
1819.750000	0.000990
1819.500000	0.000987
1819.250000	0.000984
1819.000000	0.000983
1818.750000	0.000980
1818.500000	0.000967
1818.250000	0.000945
1818.000000	0.000924
1817.750000	0.000912
1817.500000	0.000909
1817.250000	0.000906
1817.000000	0.000902
1816.750000	0.000900
1816.500000	0.000901
1816.250000	0.000904
1816.000000	0.000910

1815.750000	0.000920
1815.500000	0.000932
1815.250000	0.000947
1815.000000	0.000963
1814.750000	0.000977
1814.500000	0.000990
1814.250000	0.001001
1814.000000	0.001013
1813.750000	0.001026
1813.500000	0.001037
1813.250000	0.001040
1813.000000	0.001035
1812.750000	0.001027
1812.500000	0.001021
1812.250000	0.001021
1812.000000	0.001028
1811.750000	0.001038
1811.500000	0.001045
1811.250000	0.001041
1811.000000	0.001021
1810.750000	0.000983
1810.500000	0.000934
1810.250000	0.000889
1810.000000	0.000861
1809.750000	0.000860
1809.500000	0.000886

1809.250000	0.000933
1809.000000	0.000988
1808.750000	0.001035
1808.500000	0.001056
1808.250000	0.001058
1808.000000	0.001065
1807.750000	0.001089
1807.500000	0.001115
1807.250000	0.001123
1807.000000	0.001115
1806.750000	0.001106
1806.500000	0.001102
1806.250000	0.001098
1806.000000	0.001094
1805.750000	0.001094
1805.500000	0.001101
1805.250000	0.001112
1805.000000	0.001123
1804.750000	0.001135
1804.500000	0.001146
1804.250000	0.001155
1804.000000	0.001161
1803.750000	0.001165
1803.500000	0.001169
1803.250000	0.001176
1803.000000	0.001186

1802.750000	0.001201
1802.500000	0.001222
1802.250000	0.001245
1802.000000	0.001267
1801.750000	0.001285
1801.500000	0.001295
1801.250000	0.001297
1801.000000	0.001293
1800.750000	0.001287
1800.500000	0.001284
1800.250000	0.001288
1800.000000	0.001301
1799.750000	0.001321
1799.500000	0.001343
1799.250000	0.001359
1799.000000	0.001365
1798.750000	0.001358
1798.500000	0.001343
1798.250000	0.001322
1798.000000	0.001300
1797.750000	0.001283
1797.500000	0.001271
1797.250000	0.001269
1797.000000	0.001275
1796.750000	0.001290
1796.500000	0.001311

1796.250000	0.001337
1796.000000	0.001364
1795.750000	0.001387
1795.500000	0.001402
1795.250000	0.001408
1795.000000	0.001404
1794.750000	0.001391
1794.500000	0.001372
1794.250000	0.001351
1794.000000	0.001332
1793.750000	0.001321
1793.500000	0.001321
1793.250000	0.001340
1793.000000	0.001380
1792.750000	0.001439
1792.500000	0.001506
1792.250000	0.001568
1792.000000	0.001616
1791.750000	0.001648
1791.500000	0.001669
1791.250000	0.001674
1791.000000	0.001660
1790.750000	0.001644
1790.500000	0.001647
1790.250000	0.001632
1790.000000	0.001527

1789.750000	0.001347
1789.500000	0.001212
1789.250000	0.001213
1789.000000	0.001299
1788.750000	0.001364
1788.500000	0.001378
1788.250000	0.001384
1788.000000	0.001400
1787.750000	0.001409
1787.500000	0.001409
1787.250000	0.001417
1787.000000	0.001439
1786.750000	0.001470
1786.500000	0.001505
1786.250000	0.001547
1786.000000	0.001590
1785.750000	0.001621
1785.500000	0.001627
1785.250000	0.001600
1785.000000	0.001544
1784.750000	0.001471
1784.500000	0.001402
1784.250000	0.001356
1784.000000	0.001343
1783.750000	0.001363
1783.500000	0.001404

1783.250000	0.001455
1783.000000	0.001504
1782.750000	0.001545
1782.500000	0.001580
1782.250000	0.001610
1782.000000	0.001636
1781.750000	0.001654
1781.500000	0.001659
1781.250000	0.001645
1781.000000	0.001608
1780.750000	0.001552
1780.500000	0.001489
1780.250000	0.001435
1780.000000	0.001404
1779.750000	0.001404
1779.500000	0.001431
1779.250000	0.001475
1779.000000	0.001522
1778.750000	0.001563
1778.500000	0.001593
1778.250000	0.001613
1778.000000	0.001629
1777.750000	0.001650
1777.500000	0.001680
1777.250000	0.001716
1777.000000	0.001756

1776.750000	0.001792
1776.500000	0.001820
1776.250000	0.001832
1776.000000	0.001822
1775.750000	0.001790
1775.500000	0.001745
1775.250000	0.001708
1775.000000	0.001704
1774.750000	0.001753
1774.500000	0.001863
1774.250000	0.002021
1774.000000	0.002200
1773.750000	0.002363
1773.500000	0.002477
1773.250000	0.002517
1773.000000	0.002475
1772.750000	0.002359
1772.500000	0.002195
1772.250000	0.002023
1772.000000	0.001881
1771.750000	0.001797
1771.500000	0.001774
1771.250000	0.001796
1771.000000	0.001843
1770.750000	0.001905
1770.500000	0.001980

1770.250000	0.002072
1770.000000	0.002188
1769.750000	0.002323
1769.500000	0.002465
1769.250000	0.002594
1769.000000	0.002686
1768.750000	0.002723
1768.500000	0.002696
1768.250000	0.002613
1768.000000	0.002498
1767.750000	0.002386
1767.500000	0.002314
1767.250000	0.002303
1767.000000	0.002354
1766.750000	0.002448
1766.500000	0.002560
1766.250000	0.002669
1766.000000	0.002761
1765.750000	0.002833
1765.500000	0.002887
1765.250000	0.002931
1765.000000	0.002970
1764.750000	0.003007
1764.500000	0.003044
1764.250000	0.003085
1764.000000	0.003135

1763.750000	0.003200
1763.500000	0.003285
1763.250000	0.003389
1763.000000	0.003501
1762.750000	0.003606
1762.500000	0.003684
1762.250000	0.003723
1762.000000	0.003717
1761.750000	0.003669
1761.500000	0.003593
1761.250000	0.003520
1761.000000	0.003483
1760.750000	0.003508
1760.500000	0.003591
1760.250000	0.003708
1760.000000	0.003832
1759.750000	0.003939
1759.500000	0.004020
1759.250000	0.004076
1759.000000	0.004119
1758.750000	0.004165
1758.500000	0.004227
1758.250000	0.004310
1758.000000	0.004410
1757.750000	0.004514
1757.500000	0.004609

1757.250000	0.004681
1757.000000	0.004726
1756.750000	0.004748
1756.500000	0.004761
1756.250000	0.004783
1756.000000	0.004829
1755.750000	0.004909
1755.500000	0.005020
1755.250000	0.005150
1755.000000	0.005287
1754.750000	0.005415
1754.500000	0.005527
1754.250000	0.005620
1754.000000	0.005696
1753.750000	0.005765
1753.500000	0.005837
1753.250000	0.005921
1753.000000	0.006023
1752.750000	0.006140
1752.500000	0.006263
1752.250000	0.006384
1752.000000	0.006497
1751.750000	0.006596
1751.500000	0.006670
1751.250000	0.006709
1751.000000	0.006718

1750.750000	0.006722
1750.500000	0.006761
1750.250000	0.006865
1750.000000	0.007028
1749.750000	0.007220
1749.500000	0.007406
1749.250000	0.007562
1749.000000	0.007680
1748.750000	0.007769
1748.500000	0.007844
1748.250000	0.007926
1748.000000	0.008030
1747.750000	0.008161
1747.500000	0.008313
1747.250000	0.008475
1747.000000	0.008638
1746.750000	0.008794
1746.500000	0.008943
1746.250000	0.009084
1746.000000	0.009220
1745.750000	0.009350
1745.500000	0.009474
1745.250000	0.009597
1745.000000	0.009720
1744.750000	0.009847
1744.500000	0.009979

1744.250000	0.010120
1744.000000	0.010275
1743.750000	0.010451
1743.500000	0.010645
1743.250000	0.010848
1743.000000	0.011045
1742.750000	0.011223
1742.500000	0.011383
1742.250000	0.011531
1742.000000	0.011675
1741.750000	0.011821
1741.500000	0.011970
1741.250000	0.012123
1741.000000	0.012282
1740.750000	0.012459
1740.500000	0.012678
1740.250000	0.012959
1740.000000	0.013301
1739.750000	0.013665
1739.500000	0.013992
1739.250000	0.014244
1739.000000	0.014428
1738.750000	0.014578
1738.500000	0.014727
1738.250000	0.014883
1738.000000	0.015037

1737.750000	0.015173
1737.500000	0.015284
1737.250000	0.015371
1737.000000	0.015439
1736.750000	0.015498
1736.500000	0.015557
1736.250000	0.015611
1736.000000	0.015650
1735.750000	0.015670
1735.500000	0.015670
1735.250000	0.015641
1735.000000	0.015606
1734.750000	0.015690
1734.500000	0.016077
1734.250000	0.016830
1734.000000	0.017773
1733.750000	0.018594
1733.500000	0.019080
1733.250000	0.019238
1733.000000	0.019223
1732.750000	0.019179
1732.500000	0.019162
1732.250000	0.019162
1732.000000	0.019165
1731.750000	0.019175
1731.500000	0.019197

1731.250000	0.019230
1731.000000	0.019291
1730.750000	0.019411
1730.500000	0.019623
1730.250000	0.019931
1730.000000	0.020306
1729.750000	0.020698
1729.500000	0.021054
1729.250000	0.021344
1729.000000	0.021564
1728.750000	0.021729
1728.500000	0.021861
1728.250000	0.021977
1728.000000	0.022085
1727.750000	0.022192
1727.500000	0.022304
1727.250000	0.022426
1727.000000	0.022562
1726.750000	0.022707
1726.500000	0.022856
1726.250000	0.023003
1726.000000	0.023142
1725.750000	0.023268
1725.500000	0.023379
1725.250000	0.023478
1725.000000	0.023568

1724.750000	0.023657
1724.500000	0.023754
1724.250000	0.023873
1724.000000	0.024024
1723.750000	0.024208
1723.500000	0.024417
1723.250000	0.024628
1723.000000	0.024821
1722.750000	0.024981
1722.500000	0.025109
1722.250000	0.025208
1722.000000	0.025282
1721.750000	0.025330
1721.500000	0.025353
1721.250000	0.025346
1721.000000	0.025303
1720.750000	0.025220
1720.500000	0.025099
1720.250000	0.024939
1720.000000	0.024742
1719.750000	0.024513
1719.500000	0.024264
1719.250000	0.024007
1719.000000	0.023773
1718.750000	0.023641
1718.500000	0.023738

1718.250000	0.024181
1718.000000	0.024959
1717.750000	0.025866
1717.500000	0.026594
1717.250000	0.026946
1717.000000	0.026952
1716.750000	0.026782
1716.500000	0.026576
1716.250000	0.026381
1716.000000	0.026206
1715.750000	0.026069
1715.500000	0.025998
1715.250000	0.026017
1715.000000	0.026128
1714.750000	0.026296
1714.500000	0.026462
1714.250000	0.026570
1714.000000	0.026600
1713.750000	0.026563
1713.500000	0.026481
1713.250000	0.026367
1713.000000	0.026223
1712.750000	0.026052
1712.500000	0.025860
1712.250000	0.025654
1712.000000	0.025441

1711.750000	0.025230
1711.500000	0.025036
1711.250000	0.024876
1711.000000	0.024764
1710.750000	0.024700
1710.500000	0.024679
1710.250000	0.024683
1710.000000	0.024693
1709.750000	0.024687
1709.500000	0.024649
1709.250000	0.024571
1709.000000	0.024451
1708.750000	0.024289
1708.500000	0.024087
1708.250000	0.023854
1708.000000	0.023603
1707.750000	0.023355
1707.500000	0.023133
1707.250000	0.022969
1707.000000	0.022888
1706.750000	0.022898
1706.500000	0.022976
1706.250000	0.023077
1706.000000	0.023158
1705.750000	0.023195
1705.500000	0.023194

1705.250000	0.023178
1705.000000	0.023171
1704.750000	0.023181
1704.500000	0.023193
1704.250000	0.023182
1704.000000	0.023126
1703.750000	0.023007
1703.500000	0.022818
1703.250000	0.022575
1703.000000	0.022298
1702.750000	0.022004
1702.500000	0.021700
1702.250000	0.021362
1702.000000	0.020968
1701.750000	0.020542
1701.500000	0.020092
1701.250000	0.019540
1701.000000	0.018927
1700.750000	0.018732
1700.500000	0.019557
1700.250000	0.021283
1700.000000	0.022872
1699.750000	0.023375
1699.500000	0.022883
1699.250000	0.022150
1699.000000	0.021627

1698.750000	0.021244
1698.500000	0.020852
1698.250000	0.020461
1698.000000	0.020097
1697.750000	0.019728
1697.500000	0.019327
1697.250000	0.018900
1697.000000	0.018454
1696.750000	0.018027
1696.500000	0.017723
1696.250000	0.017686
1696.000000	0.017974
1695.750000	0.018429
1695.500000	0.018749
1695.250000	0.018739
1695.000000	0.018477
1694.750000	0.018173
1694.500000	0.017937
1694.250000	0.017743
1694.000000	0.017534
1693.750000	0.017300
1693.500000	0.017044
1693.250000	0.016767
1693.000000	0.016474
1692.750000	0.016181
1692.500000	0.015902

1692.250000	0.015640
1692.000000	0.015393
1691.750000	0.015154
1691.500000	0.014920
1691.250000	0.014688
1691.000000	0.014461
1690.750000	0.014236
1690.500000	0.014014
1690.250000	0.013796
1690.000000	0.013585
1689.750000	0.013382
1689.500000	0.013187
1689.250000	0.012998
1689.000000	0.012815
1688.750000	0.012636
1688.500000	0.012458
1688.250000	0.012279
1688.000000	0.012097
1687.750000	0.011911
1687.500000	0.011722
1687.250000	0.011539
1687.000000	0.011370
1686.750000	0.011222
1686.500000	0.011084
1686.250000	0.010933
1686.000000	0.010743

1685.750000	0.010496
1685.500000	0.010179
1685.250000	0.009775
1685.000000	0.009319
1684.750000	0.008960
1684.500000	0.008872
1684.250000	0.009065
1684.000000	0.009332
1683.750000	0.009461
1683.500000	0.009437
1683.250000	0.009380
1683.000000	0.009362
1682.750000	0.009354
1682.500000	0.009317
1682.250000	0.009250
1682.000000	0.009168
1681.750000	0.009077
1681.500000	0.008974
1681.250000	0.008857
1681.000000	0.008727
1680.750000	0.008590
1680.500000	0.008457
1680.250000	0.008336
1680.000000	0.008235
1679.750000	0.008155
1679.500000	0.008095

1679.250000	0.008051
1679.000000	0.008020
1678.750000	0.007994
1678.500000	0.007962
1678.250000	0.007915
1678.000000	0.007852
1677.750000	0.007776
1677.500000	0.007698
1677.250000	0.007627
1677.000000	0.007573
1676.750000	0.007536
1676.500000	0.007507
1676.250000	0.007475
1676.000000	0.007441
1675.750000	0.007419
1675.500000	0.007414
1675.250000	0.007401
1675.000000	0.007336
1674.750000	0.007204
1674.500000	0.007046
1674.250000	0.006917
1674.000000	0.006839
1673.750000	0.006796
1673.500000	0.006762
1673.250000	0.006724
1673.000000	0.006675

1672.750000	0.006609
1672.500000	0.006525
1672.250000	0.006425
1672.000000	0.006316
1671.750000	0.006204
1671.500000	0.006098
1671.250000	0.006009
1671.000000	0.005951
1670.750000	0.005931
1670.500000	0.005947
1670.250000	0.005979
1670.000000	0.006001
1669.750000	0.005995
1669.500000	0.005946
1669.250000	0.005847
1669.000000	0.005697
1668.750000	0.005514
1668.500000	0.005337
1668.250000	0.005207
1668.000000	0.005142
1667.750000	0.005126
1667.500000	0.005131
1667.250000	0.005132
1667.000000	0.005117
1666.750000	0.005088
1666.500000	0.005050

1666.250000	0.005010
1666.000000	0.004966
1665.750000	0.004919
1665.500000	0.004868
1665.250000	0.004816
1665.000000	0.004769
1664.750000	0.004735
1664.500000	0.004718
1664.250000	0.004719
1664.000000	0.004727
1663.750000	0.004731
1663.500000	0.004720
1663.250000	0.004687
1663.000000	0.004631
1662.750000	0.004548
1662.500000	0.004443
1662.250000	0.004331
1662.000000	0.004236
1661.750000	0.004177
1661.500000	0.004155
1661.250000	0.004157
1661.000000	0.004168
1660.750000	0.004172
1660.500000	0.004164
1660.250000	0.004146
1660.000000	0.004120

1659.750000	0.004094
1659.500000	0.004071
1659.250000	0.004052
1659.000000	0.004032
1658.750000	0.004010
1658.500000	0.003986
1658.250000	0.003963
1658.000000	0.003940
1657.750000	0.003916
1657.500000	0.003889
1657.250000	0.003861
1657.000000	0.003836
1656.750000	0.003819
1656.500000	0.003813
1656.250000	0.003825
1656.000000	0.003855
1655.750000	0.003904
1655.500000	0.003963
1655.250000	0.004012
1655.000000	0.004036
1654.750000	0.004040
1654.500000	0.004028
1654.250000	0.003962
1654.000000	0.003809
1653.750000	0.003641
1653.500000	0.003593

1653.250000	0.003674
1653.000000	0.003697
1652.750000	0.003483
1652.500000	0.003083
1652.250000	0.002712
1652.000000	0.002531
1651.750000	0.002551
1651.500000	0.002703
1651.250000	0.002918
1651.000000	0.003147
1650.750000	0.003352
1650.500000	0.003510
1650.250000	0.003616
1650.000000	0.003680
1649.750000	0.003724
1649.500000	0.003762
1649.250000	0.003798
1649.000000	0.003822
1648.750000	0.003829
1648.500000	0.003815
1648.250000	0.003786
1648.000000	0.003750
1647.750000	0.003723
1647.500000	0.003722
1647.250000	0.003758
1647.000000	0.003827

1646.750000	0.003908
1646.500000	0.003976
1646.250000	0.003998
1646.000000	0.003960
1645.750000	0.003869
1645.500000	0.003765
1645.250000	0.003695
1645.000000	0.003690
1644.750000	0.003752
1644.500000	0.003857
1644.250000	0.003975
1644.000000	0.004081
1643.750000	0.004160
1643.500000	0.004209
1643.250000	0.004234
1643.000000	0.004245
1642.750000	0.004254
1642.500000	0.004269
1642.250000	0.004294
1642.000000	0.004325
1641.750000	0.004358
1641.500000	0.004392
1641.250000	0.004427
1641.000000	0.004465
1640.750000	0.004502
1640.500000	0.004534

1640.250000	0.004555
1640.000000	0.004568
1639.750000	0.004579
1639.500000	0.004596
1639.250000	0.004623
1639.000000	0.004656
1638.750000	0.004688
1638.500000	0.004709
1638.250000	0.004712
1638.000000	0.004696
1637.750000	0.004666
1637.500000	0.004637
1637.250000	0.004627
1637.000000	0.004649
1636.750000	0.004693
1636.500000	0.004739
1636.250000	0.004773
1636.000000	0.004797
1635.750000	0.004802
1635.500000	0.004757
1635.250000	0.004629
1635.000000	0.004430
1634.750000	0.004225
1634.500000	0.004095
1634.250000	0.004074
1634.000000	0.004132

1633.750000	0.004209
1633.500000	0.004262
1633.250000	0.004282
1633.000000	0.004273
1632.750000	0.004241
1632.500000	0.004198
1632.250000	0.004153
1632.000000	0.004113
1631.750000	0.004080
1631.500000	0.004051
1631.250000	0.004023
1631.000000	0.003996
1630.750000	0.003966
1630.500000	0.003934
1630.250000	0.003903
1630.000000	0.003875
1629.750000	0.003855
1629.500000	0.003844
1629.250000	0.003841
1629.000000	0.003838
1628.750000	0.003827
1628.500000	0.003800
1628.250000	0.003750
1628.000000	0.003676
1627.750000	0.003586
1627.500000	0.003496

1627.250000	0.003421
1627.000000	0.003374
1626.750000	0.003353
1626.500000	0.003350
1626.250000	0.003355
1626.000000	0.003358
1625.750000	0.003359
1625.500000	0.003361
1625.250000	0.003371
1625.000000	0.003391
1624.750000	0.003417
1624.500000	0.003436
1624.250000	0.003439
1624.000000	0.003413
1623.750000	0.003352
1623.500000	0.003253
1623.250000	0.003125
1623.000000	0.002989
1622.750000	0.002876
1622.500000	0.002809
1622.250000	0.002792
1622.000000	0.002814
1621.750000	0.002853
1621.500000	0.002888
1621.250000	0.002910
1621.000000	0.002914

1620.750000	0.002906
1620.500000	0.002893
1620.250000	0.002882
1620.000000	0.002872
1619.750000	0.002863
1619.500000	0.002853
1619.250000	0.002847
1619.000000	0.002851
1618.750000	0.002875
1618.500000	0.002926
1618.250000	0.002997
1618.000000	0.003072
1617.750000	0.003126
1617.500000	0.003141
1617.250000	0.003114
1617.000000	0.003048
1616.750000	0.002934
1616.500000	0.002768
1616.250000	0.002577
1616.000000	0.002425
1615.750000	0.002376
1615.500000	0.002441
1615.250000	0.002581
1615.000000	0.002741
1614.750000	0.002882
1614.500000	0.002987

1614.250000	0.003052
1614.000000	0.003088
1613.750000	0.003108
1613.500000	0.003127
1613.250000	0.003153
1613.000000	0.003186
1612.750000	0.003221
1612.500000	0.003254
1612.250000	0.003285
1612.000000	0.003316
1611.750000	0.003350
1611.500000	0.003391
1611.250000	0.003436
1611.000000	0.003483
1610.750000	0.003530
1610.500000	0.003573
1610.250000	0.003611
1610.000000	0.003645
1609.750000	0.003674
1609.500000	0.003702
1609.250000	0.003734
1609.000000	0.003772
1608.750000	0.003821
1608.500000	0.003879
1608.250000	0.003945
1608.000000	0.004014

1607.750000	0.004082
1607.500000	0.004146
1607.250000	0.004207
1607.000000	0.004269
1606.750000	0.004335
1606.500000	0.004410
1606.250000	0.004493
1606.000000	0.004585
1605.750000	0.004682
1605.500000	0.004781
1605.250000	0.004876
1605.000000	0.004966
1604.750000	0.005051
1604.500000	0.005131
1604.250000	0.005206
1604.000000	0.005277
1603.750000	0.005343
1603.500000	0.005405
1603.250000	0.005461
1603.000000	0.005511
1602.750000	0.005554
1602.500000	0.005590
1602.250000	0.005617
1602.000000	0.005636
1601.750000	0.005646
1601.500000	0.005645

1601.250000	0.005631
1601.000000	0.005605
1600.750000	0.005566
1600.500000	0.005517
1600.250000	0.005460
1600.000000	0.005395
1599.750000	0.005325
1599.500000	0.005248
1599.250000	0.005164
1599.000000	0.005073
1598.750000	0.004974
1598.500000	0.004868
1598.250000	0.004758
1598.000000	0.004645
1597.750000	0.004530
1597.500000	0.004413
1597.250000	0.004294
1597.000000	0.004173
1596.750000	0.004052
1596.500000	0.003932
1596.250000	0.003817
1596.000000	0.003706
1595.750000	0.003602
1595.500000	0.003504
1595.250000	0.003408
1595.000000	0.003313

1594.750000	0.003219
1594.500000	0.003124
1594.250000	0.003033
1594.000000	0.002949
1593.750000	0.002876
1593.500000	0.002815
1593.250000	0.002767
1593.000000	0.002727
1592.750000	0.002691
1592.500000	0.002655
1592.250000	0.002616
1592.000000	0.002573
1591.750000	0.002529
1591.500000	0.002486
1591.250000	0.002446
1591.000000	0.002411
1590.750000	0.002380
1590.500000	0.002350
1590.250000	0.002321
1590.000000	0.002291
1589.750000	0.002262
1589.500000	0.002235
1589.250000	0.002213
1589.000000	0.002198
1588.750000	0.002188
1588.500000	0.002184

1588.250000	0.002184
1588.000000	0.002186
1587.750000	0.002187
1587.500000	0.002187
1587.250000	0.002186
1587.000000	0.002184
1586.750000	0.002182
1586.500000	0.002182
1586.250000	0.002183
1586.000000	0.002186
1585.750000	0.002190
1585.500000	0.002194
1585.250000	0.002199
1585.000000	0.002204
1584.750000	0.002209
1584.500000	0.002215
1584.250000	0.002220
1584.000000	0.002224
1583.750000	0.002228
1583.500000	0.002233
1583.250000	0.002238
1583.000000	0.002246
1582.750000	0.002256
1582.500000	0.002269
1582.250000	0.002282
1582.000000	0.002296

1581.750000	0.002310
1581.500000	0.002323
1581.250000	0.002336
1581.000000	0.002346
1580.750000	0.002353
1580.500000	0.002356
1580.250000	0.002358
1580.000000	0.002364
1579.750000	0.002375
1579.500000	0.002388
1579.250000	0.002400
1579.000000	0.002410
1578.750000	0.002422
1578.500000	0.002440
1578.250000	0.002472
1578.000000	0.002523
1577.750000	0.002586
1577.500000	0.002648
1577.250000	0.002685
1577.000000	0.002677
1576.750000	0.002620
1576.500000	0.002516
1576.250000	0.002365
1576.000000	0.002167
1575.750000	0.001955
1575.500000	0.001793

1575.250000	0.001741
1575.000000	0.001807
1574.750000	0.001949
1574.500000	0.002108
1574.250000	0.002243
1574.000000	0.002335
1573.750000	0.002384
1573.500000	0.002401
1573.250000	0.002399
1573.000000	0.002392
1572.750000	0.002387
1572.500000	0.002387
1572.250000	0.002390
1572.000000	0.002399
1571.750000	0.002419
1571.500000	0.002454
1571.250000	0.002508
1571.000000	0.002573
1570.750000	0.002633
1570.500000	0.002669
1570.250000	0.002672
1570.000000	0.002640
1569.750000	0.002577
1569.500000	0.002481
1569.250000	0.002367
1569.000000	0.002262

1568.750000	0.002200
1568.500000	0.002199
1568.250000	0.002248
1568.000000	0.002321
1567.750000	0.002393
1567.500000	0.002448
1567.250000	0.002484
1567.000000	0.002508
1566.750000	0.002526
1566.500000	0.002547
1566.250000	0.002571
1566.000000	0.002598
1565.750000	0.002621
1565.500000	0.002637
1565.250000	0.002642
1565.000000	0.002636
1564.750000	0.002622
1564.500000	0.002607
1564.250000	0.002601
1564.000000	0.002613
1563.750000	0.002647
1563.500000	0.002694
1563.250000	0.002744
1563.000000	0.002785
1562.750000	0.002818
1562.500000	0.002850

1562.250000	0.002894
1562.000000	0.002956
1561.750000	0.003035
1561.500000	0.003122
1561.250000	0.003202
1561.000000	0.003257
1560.750000	0.003266
1560.500000	0.003218
1560.250000	0.003109
1560.000000	0.002960
1559.750000	0.002818
1559.500000	0.002748
1559.250000	0.002806
1559.000000	0.002994
1558.750000	0.003238
1558.500000	0.003428
1558.250000	0.003491
1558.000000	0.003438
1557.750000	0.003346
1557.500000	0.003288
1557.250000	0.003297
1557.000000	0.003365
1556.750000	0.003468
1556.500000	0.003585
1556.250000	0.003705
1556.000000	0.003817

1555.750000	0.003911
1555.500000	0.003980
1555.250000	0.004025
1555.000000	0.004055
1554.750000	0.004079
1554.500000	0.004104
1554.250000	0.004125
1554.000000	0.004137
1553.750000	0.004142
1553.500000	0.004161
1553.250000	0.004209
1553.000000	0.004285
1552.750000	0.004372
1552.500000	0.004452
1552.250000	0.004519
1552.000000	0.004568
1551.750000	0.004602
1551.500000	0.004626
1551.250000	0.004645
1551.000000	0.004660
1550.750000	0.004676
1550.500000	0.004700
1550.250000	0.004735
1550.000000	0.004782
1549.750000	0.004834
1549.500000	0.004890

1549.250000	0.004954
1549.000000	0.005032
1548.750000	0.005123
1548.500000	0.005219
1548.250000	0.005307
1548.000000	0.005377
1547.750000	0.005426
1547.500000	0.005461
1547.250000	0.005491
1547.000000	0.005524
1546.750000	0.005561
1546.500000	0.005603
1546.250000	0.005644
1546.000000	0.005685
1545.750000	0.005731
1545.500000	0.005787
1545.250000	0.005848
1545.000000	0.005899
1544.750000	0.005930
1544.500000	0.005950
1544.250000	0.005978
1544.000000	0.006031
1543.750000	0.006110
1543.500000	0.006201
1543.250000	0.006287
1543.000000	0.006359

1542.750000	0.006420
1542.500000	0.006482
1542.250000	0.006551
1542.000000	0.006628
1541.750000	0.006699
1541.500000	0.006759
1541.250000	0.006806
1541.000000	0.006842
1540.750000	0.006851
1540.500000	0.006821
1540.250000	0.006778
1540.000000	0.006796
1539.750000	0.006927
1539.500000	0.007139
1539.250000	0.007325
1539.000000	0.007397
1538.750000	0.007356
1538.500000	0.007287
1538.250000	0.007273
1538.000000	0.007336
1537.750000	0.007439
1537.500000	0.007546
1537.250000	0.007641
1537.000000	0.007714
1536.750000	0.007758
1536.500000	0.007767

1536.250000	0.007751
1536.000000	0.007725
1535.750000	0.007704
1535.500000	0.007694
1535.250000	0.007697
1535.000000	0.007705
1534.750000	0.007710
1534.500000	0.007713
1534.250000	0.007737
1534.000000	0.007815
1533.750000	0.007951
1533.500000	0.008112
1533.250000	0.008245
1533.000000	0.008323
1532.750000	0.008357
1532.500000	0.008374
1532.250000	0.008390
1532.000000	0.008405
1531.750000	0.008412
1531.500000	0.008406
1531.250000	0.008392
1531.000000	0.008376
1530.750000	0.008362
1530.500000	0.008356
1530.250000	0.008354
1530.000000	0.008352

1529.750000	0.008345
1529.500000	0.008329
1529.250000	0.008308
1529.000000	0.008288
1528.750000	0.008271
1528.500000	0.008264
1528.250000	0.008267
1528.000000	0.008280
1527.750000	0.008297
1527.500000	0.008316
1527.250000	0.008332
1527.000000	0.008347
1526.750000	0.008363
1526.500000	0.008381
1526.250000	0.008401
1526.000000	0.008424
1525.750000	0.008451
1525.500000	0.008474
1525.250000	0.008483
1525.000000	0.008471
1524.750000	0.008441
1524.500000	0.008407
1524.250000	0.008382
1524.000000	0.008370
1523.750000	0.008362
1523.500000	0.008345

1523.250000	0.008309
1523.000000	0.008253
1522.750000	0.008186
1522.500000	0.008118
1522.250000	0.008057
1522.000000	0.008019
1521.750000	0.008032
1521.500000	0.008117
1521.250000	0.008260
1521.000000	0.008401
1520.750000	0.008480
1520.500000	0.008481
1520.250000	0.008439
1520.000000	0.008402
1519.750000	0.008395
1519.500000	0.008412
1519.250000	0.008434
1519.000000	0.008445
1518.750000	0.008436
1518.500000	0.008402
1518.250000	0.008337
1518.000000	0.008251
1517.750000	0.008179
1517.500000	0.008171
1517.250000	0.008250
1517.000000	0.008394

1516.750000	0.008549
1516.500000	0.008676
1516.250000	0.008769
1516.000000	0.008842
1515.750000	0.008911
1515.500000	0.008979
1515.250000	0.009041
1515.000000	0.009094
1514.750000	0.009138
1514.500000	0.009174
1514.250000	0.009204
1514.000000	0.009233
1513.750000	0.009264
1513.500000	0.009298
1513.250000	0.009336
1513.000000	0.009380
1512.750000	0.009437
1512.500000	0.009509
1512.250000	0.009588
1512.000000	0.009655
1511.750000	0.009697
1511.500000	0.009712
1511.250000	0.009704
1511.000000	0.009680
1510.750000	0.009639
1510.500000	0.009582

1510.250000	0.009507
1510.000000	0.009416
1509.750000	0.009315
1509.500000	0.009214
1509.250000	0.009121
1509.000000	0.009040
1508.750000	0.008961
1508.500000	0.008874
1508.250000	0.008766
1508.000000	0.008628
1507.750000	0.008456
1507.500000	0.008266
1507.250000	0.008112
1507.000000	0.008057
1506.750000	0.008108
1506.500000	0.008196
1506.250000	0.008216
1506.000000	0.008115
1505.750000	0.007915
1505.500000	0.007682
1505.250000	0.007466
1505.000000	0.007290
1504.750000	0.007149
1504.500000	0.007038
1504.250000	0.006951
1504.000000	0.006878

1503.750000	0.006803
1503.500000	0.006711
1503.250000	0.006597
1503.000000	0.006463
1502.750000	0.006315
1502.500000	0.006162
1502.250000	0.006012
1502.000000	0.005871
1501.750000	0.005739
1501.500000	0.005616
1501.250000	0.005502
1501.000000	0.005403
1500.750000	0.005322
1500.500000	0.005263
1500.250000	0.005218
1500.000000	0.005178
1499.750000	0.005128
1499.500000	0.005061
1499.250000	0.004971
1499.000000	0.004862
1498.750000	0.004741
1498.500000	0.004625
1498.250000	0.004535
1498.000000	0.004486
1497.750000	0.004475
1497.500000	0.004486

1497.250000	0.004490
1497.000000	0.004464
1496.750000	0.004402
1496.500000	0.004308
1496.250000	0.004194
1496.000000	0.004073
1495.750000	0.003963
1495.500000	0.003887
1495.250000	0.003860
1495.000000	0.003882
1494.750000	0.003931
1494.500000	0.003983
1494.250000	0.004019
1494.000000	0.004031
1493.750000	0.004022
1493.500000	0.004001
1493.250000	0.003977
1493.000000	0.003958
1492.750000	0.003953
1492.500000	0.003961
1492.250000	0.003977
1492.000000	0.003994
1491.750000	0.004005
1491.500000	0.004007
1491.250000	0.004001
1491.000000	0.003990

1490.750000	0.003981
1490.500000	0.003979
1490.250000	0.003978
1490.000000	0.003968
1489.750000	0.003944
1489.500000	0.003915
1489.250000	0.003899
1489.000000	0.003912
1488.750000	0.003955
1488.500000	0.004017
1488.250000	0.004084
1488.000000	0.004142
1487.750000	0.004185
1487.500000	0.004209
1487.250000	0.004214
1487.000000	0.004204
1486.750000	0.004188
1486.500000	0.004174
1486.250000	0.004171
1486.000000	0.004181
1485.750000	0.004200
1485.500000	0.004221
1485.250000	0.004241
1485.000000	0.004259
1484.750000	0.004274
1484.500000	0.004290

1484.250000	0.004305
1484.000000	0.004319
1483.750000	0.004333
1483.500000	0.004346
1483.250000	0.004357
1483.000000	0.004366
1482.750000	0.004376
1482.500000	0.004385
1482.250000	0.004393
1482.000000	0.004399
1481.750000	0.004405
1481.500000	0.004412
1481.250000	0.004425
1481.000000	0.004445
1480.750000	0.004472
1480.500000	0.004504
1480.250000	0.004541
1480.000000	0.004579
1479.750000	0.004618
1479.500000	0.004652
1479.250000	0.004680
1479.000000	0.004700
1478.750000	0.004717
1478.500000	0.004733
1478.250000	0.004752
1478.000000	0.004774

1477.750000	0.004800
1477.500000	0.004828
1477.250000	0.004858
1477.000000	0.004892
1476.750000	0.004929
1476.500000	0.004970
1476.250000	0.005007
1476.000000	0.005035
1475.750000	0.005059
1475.500000	0.005091
1475.250000	0.005140
1475.000000	0.005206
1474.750000	0.005273
1474.500000	0.005323
1474.250000	0.005346
1474.000000	0.005345
1473.750000	0.005333
1473.500000	0.005332
1473.250000	0.005366
1473.000000	0.005452
1472.750000	0.005594
1472.500000	0.005770
1472.250000	0.005944
1472.000000	0.006081
1471.750000	0.006168
1471.500000	0.006218

1471.250000	0.006258
1471.000000	0.006306
1470.750000	0.006370
1470.500000	0.006445
1470.250000	0.006524
1470.000000	0.006599
1469.750000	0.006667
1469.500000	0.006725
1469.250000	0.006773
1469.000000	0.006818
1468.750000	0.006866
1468.500000	0.006919
1468.250000	0.006974
1468.000000	0.007026
1467.750000	0.007069
1467.500000	0.007103
1467.250000	0.007130
1467.000000	0.007155
1466.750000	0.007179
1466.500000	0.007206
1466.250000	0.007235
1466.000000	0.007265
1465.750000	0.007305
1465.500000	0.007367
1465.250000	0.007463
1465.000000	0.007585

1464.750000	0.007708
1464.500000	0.007800
1464.250000	0.007851
1464.000000	0.007871
1463.750000	0.007882
1463.500000	0.007897
1463.250000	0.007916
1463.000000	0.007931
1462.750000	0.007940
1462.500000	0.007942
1462.250000	0.007943
1462.000000	0.007945
1461.750000	0.007951
1461.500000	0.007956
1461.250000	0.007958
1461.000000	0.007954
1460.750000	0.007944
1460.500000	0.007929
1460.250000	0.007912
1460.000000	0.007892
1459.750000	0.007872
1459.500000	0.007852
1459.250000	0.007838
1459.000000	0.007834
1458.750000	0.007848
1458.500000	0.007884

1458.250000	0.007938
1458.000000	0.008004
1457.750000	0.008087
1457.500000	0.008211
1457.250000	0.008395
1457.000000	0.008620
1456.750000	0.008826
1456.500000	0.008946
1456.250000	0.008958
1456.000000	0.008906
1455.750000	0.008850
1455.500000	0.008826
1455.250000	0.008829
1455.000000	0.008837
1454.750000	0.008837
1454.500000	0.008824
1454.250000	0.008799
1454.000000	0.008765
1453.750000	0.008728
1453.500000	0.008694
1453.250000	0.008667
1453.000000	0.008645
1452.750000	0.008628
1452.500000	0.008613
1452.250000	0.008602
1452.000000	0.008599

1451.750000	0.008602
1451.500000	0.008608
1451.250000	0.008613
1451.000000	0.008611
1450.750000	0.008603
1450.500000	0.008586
1450.250000	0.008562
1450.000000	0.008531
1449.750000	0.008493
1449.500000	0.008452
1449.250000	0.008411
1449.000000	0.008373
1448.750000	0.008344
1448.500000	0.008333
1448.250000	0.008342
1448.000000	0.008364
1447.750000	0.008387
1447.500000	0.008398
1447.250000	0.008393
1447.000000	0.008375
1446.750000	0.008352
1446.500000	0.008327
1446.250000	0.008298
1446.000000	0.008266
1445.750000	0.008230
1445.500000	0.008193

1445.250000	0.008155
1445.000000	0.008120
1444.750000	0.008088
1444.500000	0.008061
1444.250000	0.008038
1444.000000	0.008017
1443.750000	0.007998
1443.500000	0.007980
1443.250000	0.007963
1443.000000	0.007946
1442.750000	0.007929
1442.500000	0.007911
1442.250000	0.007891
1442.000000	0.007870
1441.750000	0.007850
1441.500000	0.007830
1441.250000	0.007809
1441.000000	0.007787
1440.750000	0.007766
1440.500000	0.007749
1440.250000	0.007733
1440.000000	0.007715
1439.750000	0.007691
1439.500000	0.007659
1439.250000	0.007622
1439.000000	0.007582

1438.750000	0.007546
1438.500000	0.007518
1438.250000	0.007500
1438.000000	0.007483
1437.750000	0.007455
1437.500000	0.007417
1437.250000	0.007397
1437.000000	0.007425
1436.750000	0.007498
1436.500000	0.007563
1436.250000	0.007565
1436.000000	0.007501
1435.750000	0.007409
1435.500000	0.007331
1435.250000	0.007281
1435.000000	0.007245
1434.750000	0.007206
1434.500000	0.007155
1434.250000	0.007094
1434.000000	0.007028
1433.750000	0.006962
1433.500000	0.006901
1433.250000	0.006848
1433.000000	0.006801
1432.750000	0.006757
1432.500000	0.006714

1432.250000	0.006670
1432.000000	0.006629
1431.750000	0.006592
1431.500000	0.006557
1431.250000	0.006522
1431.000000	0.006483
1430.750000	0.006442
1430.500000	0.006403
1430.250000	0.006369
1430.000000	0.006341
1429.750000	0.006316
1429.500000	0.006292
1429.250000	0.006270
1429.000000	0.006251
1428.750000	0.006236
1428.500000	0.006224
1428.250000	0.006213
1428.000000	0.006201
1427.750000	0.006185
1427.500000	0.006168
1427.250000	0.006148
1427.000000	0.006128
1426.750000	0.006109
1426.500000	0.006091
1426.250000	0.006074
1426.000000	0.006057

1425.750000	0.006039
1425.500000	0.006020
1425.250000	0.005999
1425.000000	0.005978
1424.750000	0.005961
1424.500000	0.005956
1424.250000	0.005967
1424.000000	0.005993
1423.750000	0.006031
1423.500000	0.006072
1423.250000	0.006113
1423.000000	0.006152
1422.750000	0.006187
1422.500000	0.006215
1422.250000	0.006233
1422.000000	0.006239
1421.750000	0.006235
1421.500000	0.006226
1421.250000	0.006220
1421.000000	0.006219
1420.750000	0.006219
1420.500000	0.006213
1420.250000	0.006196
1420.000000	0.006182
1419.750000	0.006195
1419.500000	0.006246

1419.250000	0.006321
1419.000000	0.006383
1418.750000	0.006409
1418.500000	0.006411
1418.250000	0.006415
1418.000000	0.006436
1417.750000	0.006465
1417.500000	0.006488
1417.250000	0.006497
1417.000000	0.006496
1416.750000	0.006492
1416.500000	0.006490
1416.250000	0.006493
1416.000000	0.006504
1415.750000	0.006519
1415.500000	0.006536
1415.250000	0.006548
1415.000000	0.006555
1414.750000	0.006558
1414.500000	0.006562
1414.250000	0.006567
1414.000000	0.006573
1413.750000	0.006579
1413.500000	0.006583
1413.250000	0.006586
1413.000000	0.006587

1412.750000	0.006586
1412.500000	0.006586
1412.250000	0.006588
1412.000000	0.006595
1411.750000	0.006607
1411.500000	0.006626
1411.250000	0.006650
1411.000000	0.006679
1410.750000	0.006709
1410.500000	0.006737
1410.250000	0.006762
1410.000000	0.006786
1409.750000	0.006807
1409.500000	0.006825
1409.250000	0.006841
1409.000000	0.006855
1408.750000	0.006869
1408.500000	0.006884
1408.250000	0.006898
1408.000000	0.006911
1407.750000	0.006923
1407.500000	0.006931
1407.250000	0.006936
1407.000000	0.006936
1406.750000	0.006934
1406.500000	0.006929

1406.250000	0.006923
1406.000000	0.006919
1405.750000	0.006920
1405.500000	0.006931
1405.250000	0.006957
1405.000000	0.006993
1404.750000	0.007033
1404.500000	0.007070
1404.250000	0.007100
1404.000000	0.007122
1403.750000	0.007137
1403.500000	0.007145
1403.250000	0.007147
1403.000000	0.007144
1402.750000	0.007137
1402.500000	0.007126
1402.250000	0.007113
1402.000000	0.007096
1401.750000	0.007077
1401.500000	0.007056
1401.250000	0.007036
1401.000000	0.007016
1400.750000	0.006997
1400.500000	0.006977
1400.250000	0.006957
1400.000000	0.006938

1399.750000	0.006928
1399.500000	0.006933
1399.250000	0.006952
1399.000000	0.006978
1398.750000	0.007002
1398.500000	0.007015
1398.250000	0.007016
1398.000000	0.007007
1397.750000	0.006991
1397.500000	0.006970
1397.250000	0.006944
1397.000000	0.006912
1396.750000	0.006878
1396.500000	0.006843
1396.250000	0.006813
1396.000000	0.006791
1395.750000	0.006782
1395.500000	0.006791
1395.250000	0.006826
1395.000000	0.006887
1394.750000	0.006965
1394.500000	0.007044
1394.250000	0.007107
1394.000000	0.007146
1393.750000	0.007165
1393.500000	0.007172

1393.250000	0.007176
1393.000000	0.007180
1392.750000	0.007186
1392.500000	0.007191
1392.250000	0.007193
1392.000000	0.007192
1391.750000	0.007192
1391.500000	0.007195
1391.250000	0.007207
1391.000000	0.007228
1390.750000	0.007253
1390.500000	0.007280
1390.250000	0.007304
1390.000000	0.007325
1389.750000	0.007343
1389.500000	0.007361
1389.250000	0.007379
1389.000000	0.007396
1388.750000	0.007410
1388.500000	0.007423
1388.250000	0.007440
1388.000000	0.007472
1387.750000	0.007524
1387.500000	0.007596
1387.250000	0.007678
1387.000000	0.007758

1386.750000	0.007826
1386.500000	0.007882
1386.250000	0.007931
1386.000000	0.007978
1385.750000	0.008022
1385.500000	0.008062
1385.250000	0.008098
1385.000000	0.008132
1384.750000	0.008165
1384.500000	0.008200
1384.250000	0.008237
1384.000000	0.008276
1383.750000	0.008316
1383.500000	0.008355
1383.250000	0.008392
1383.000000	0.008426
1382.750000	0.008458
1382.500000	0.008490
1382.250000	0.008522
1382.000000	0.008553
1381.750000	0.008584
1381.500000	0.008614
1381.250000	0.008642
1381.000000	0.008666
1380.750000	0.008684
1380.500000	0.008697

1380.250000	0.008704
1380.000000	0.008708
1379.750000	0.008709
1379.500000	0.008707
1379.250000	0.008704
1379.000000	0.008700
1378.750000	0.008696
1378.500000	0.008691
1378.250000	0.008684
1378.000000	0.008675
1377.750000	0.008664
1377.500000	0.008651
1377.250000	0.008635
1377.000000	0.008613
1376.750000	0.008584
1376.500000	0.008546
1376.250000	0.008499
1376.000000	0.008445
1375.750000	0.008385
1375.500000	0.008324
1375.250000	0.008268
1375.000000	0.008219
1374.750000	0.008180
1374.500000	0.008152
1374.250000	0.008134
1374.000000	0.008122

1373.750000	0.008111
1373.500000	0.008097
1373.250000	0.008079
1373.000000	0.008058
1372.750000	0.008034
1372.500000	0.008010
1372.250000	0.007986
1372.000000	0.007964
1371.750000	0.007942
1371.500000	0.007920
1371.250000	0.007897
1371.000000	0.007873
1370.750000	0.007846
1370.500000	0.007818
1370.250000	0.007789
1370.000000	0.007760
1369.750000	0.007732
1369.500000	0.007705
1369.250000	0.007681
1369.000000	0.007661
1368.750000	0.007645
1368.500000	0.007633
1368.250000	0.007625
1368.000000	0.007622
1367.750000	0.007625
1367.500000	0.007636

1367.250000	0.007657
1367.000000	0.007686
1366.750000	0.007721
1366.500000	0.007761
1366.250000	0.007799
1366.000000	0.007829
1365.750000	0.007846
1365.500000	0.007843
1365.250000	0.007818
1365.000000	0.007774
1364.750000	0.007712
1364.500000	0.007636
1364.250000	0.007549
1364.000000	0.007458
1363.750000	0.007371
1363.500000	0.007299
1363.250000	0.007246
1363.000000	0.007214
1362.750000	0.007197
1362.500000	0.007190
1362.250000	0.007188
1362.000000	0.007187
1361.750000	0.007182
1361.500000	0.007169
1361.250000	0.007145
1361.000000	0.007108

1360.750000	0.007062
1360.500000	0.007009
1360.250000	0.006956
1360.000000	0.006906
1359.750000	0.006858
1359.500000	0.006814
1359.250000	0.006770
1359.000000	0.006727
1358.750000	0.006684
1358.500000	0.006644
1358.250000	0.006608
1358.000000	0.006578
1357.750000	0.006554
1357.500000	0.006535
1357.250000	0.006520
1357.000000	0.006506
1356.750000	0.006492
1356.500000	0.006478
1356.250000	0.006462
1356.000000	0.006445
1355.750000	0.006429
1355.500000	0.006413
1355.250000	0.006399
1355.000000	0.006390
1354.750000	0.006383
1354.500000	0.006379

1354.250000	0.006375
1354.000000	0.006370
1353.750000	0.006364
1353.500000	0.006358
1353.250000	0.006351
1353.000000	0.006343
1352.750000	0.006333
1352.500000	0.006322
1352.250000	0.006308
1352.000000	0.006291
1351.750000	0.006272
1351.500000	0.006251
1351.250000	0.006230
1351.000000	0.006208
1350.750000	0.006185
1350.500000	0.006161
1350.250000	0.006136
1350.000000	0.006110
1349.750000	0.006083
1349.500000	0.006057
1349.250000	0.006031
1349.000000	0.006007
1348.750000	0.005983
1348.500000	0.005958
1348.250000	0.005932
1348.000000	0.005905

1347.750000	0.005876
1347.500000	0.005846
1347.250000	0.005816
1347.000000	0.005787
1346.750000	0.005757
1346.500000	0.005726
1346.250000	0.005695
1346.000000	0.005664
1345.750000	0.005634
1345.500000	0.005606
1345.250000	0.005579
1345.000000	0.005554
1344.750000	0.005530
1344.500000	0.005505
1344.250000	0.005479
1344.000000	0.005451
1343.750000	0.005423
1343.500000	0.005395
1343.250000	0.005368
1343.000000	0.005341
1342.750000	0.005316
1342.500000	0.005292
1342.250000	0.005269
1342.000000	0.005246
1341.750000	0.005221
1341.500000	0.005192

1341.250000	0.005159
1341.000000	0.005122
1340.750000	0.005083
1340.500000	0.005044
1340.250000	0.005010
1340.000000	0.004982
1339.750000	0.004962
1339.500000	0.004950
1339.250000	0.004947
1339.000000	0.004949
1338.750000	0.004956
1338.500000	0.004964
1338.250000	0.004974
1338.000000	0.004983
1337.750000	0.004991
1337.500000	0.004998
1337.250000	0.005004
1337.000000	0.005009
1336.750000	0.005013
1336.500000	0.005018
1336.250000	0.005024
1336.000000	0.005033
1335.750000	0.005045
1335.500000	0.005060
1335.250000	0.005077
1335.000000	0.005093

1334.750000	0.005108
1334.500000	0.005120
1334.250000	0.005131
1334.000000	0.005140
1333.750000	0.005150
1333.500000	0.005161
1333.250000	0.005173
1333.000000	0.005187
1332.750000	0.005202
1332.500000	0.005217
1332.250000	0.005232
1332.000000	0.005248
1331.750000	0.005264
1331.500000	0.005281
1331.250000	0.005299
1331.000000	0.005319
1330.750000	0.005340
1330.500000	0.005361
1330.250000	0.005383
1330.000000	0.005404
1329.750000	0.005425
1329.500000	0.005444
1329.250000	0.005463
1329.000000	0.005480
1328.750000	0.005498
1328.500000	0.005514

1328.250000	0.005530
1328.000000	0.005546
1327.750000	0.005562
1327.500000	0.005579
1327.250000	0.005597
1327.000000	0.005616
1326.750000	0.005636
1326.500000	0.005658
1326.250000	0.005679
1326.000000	0.005698
1325.750000	0.005717
1325.500000	0.005734
1325.250000	0.005751
1325.000000	0.005768
1324.750000	0.005786
1324.500000	0.005805
1324.250000	0.005826
1324.000000	0.005847
1323.750000	0.005869
1323.500000	0.005889
1323.250000	0.005909
1323.000000	0.005926
1322.750000	0.005941
1322.500000	0.005953
1322.250000	0.005962
1322.000000	0.005969

1321.750000	0.005973
1321.500000	0.005973
1321.250000	0.005971
1321.000000	0.005966
1320.750000	0.005961
1320.500000	0.005954
1320.250000	0.005947
1320.000000	0.005942
1319.750000	0.005939
1319.500000	0.005939
1319.250000	0.005942
1319.000000	0.005947
1318.750000	0.005954
1318.500000	0.005959
1318.250000	0.005962
1318.000000	0.005962
1317.750000	0.005958
1317.500000	0.005952
1317.250000	0.005944
1317.000000	0.005936
1316.750000	0.005929
1316.500000	0.005922
1316.250000	0.005916
1316.000000	0.005910
1315.750000	0.005904
1315.500000	0.005896

1315.250000	0.005887
1315.000000	0.005876
1314.750000	0.005864
1314.500000	0.005852
1314.250000	0.005841
1314.000000	0.005832
1313.750000	0.005825
1313.500000	0.005821
1313.250000	0.005820
1313.000000	0.005820
1312.750000	0.005821
1312.500000	0.005822
1312.250000	0.005822
1312.000000	0.005820
1311.750000	0.005816
1311.500000	0.005813
1311.250000	0.005810
1311.000000	0.005808
1310.750000	0.005806
1310.500000	0.005805
1310.250000	0.005803
1310.000000	0.005798
1309.750000	0.005793
1309.500000	0.005787
1309.250000	0.005782
1309.000000	0.005780

1308.750000	0.005782
1308.500000	0.005789
1308.250000	0.005800
1308.000000	0.005813
1307.750000	0.005828
1307.500000	0.005844
1307.250000	0.005861
1307.000000	0.005880
1306.750000	0.005901
1306.500000	0.005923
1306.250000	0.005946
1306.000000	0.005970
1305.750000	0.005994
1305.500000	0.006019
1305.250000	0.006045
1305.000000	0.006074
1304.750000	0.006105
1304.500000	0.006138
1304.250000	0.006173
1304.000000	0.006208
1303.750000	0.006243
1303.500000	0.006278
1303.250000	0.006313
1303.000000	0.006349
1302.750000	0.006386
1302.500000	0.006424

1302.250000	0.006463
1302.000000	0.006503
1301.750000	0.006544
1301.500000	0.006585
1301.250000	0.006628
1301.000000	0.006672
1300.750000	0.006715
1300.500000	0.006759
1300.250000	0.006801
1300.000000	0.006841
1299.750000	0.006880
1299.500000	0.006919
1299.250000	0.006956
1299.000000	0.006994
1298.750000	0.007031
1298.500000	0.007066
1298.250000	0.007099
1298.000000	0.007129
1297.750000	0.007155
1297.500000	0.007179
1297.250000	0.007201
1297.000000	0.007222
1296.750000	0.007242
1296.500000	0.007260
1296.250000	0.007275
1296.000000	0.007285

1295.750000	0.007289
1295.500000	0.007289
1295.250000	0.007283
1295.000000	0.007274
1294.750000	0.007261
1294.500000	0.007245
1294.250000	0.007226
1294.000000	0.007203
1293.750000	0.007177
1293.500000	0.007147
1293.250000	0.007114
1293.000000	0.007078
1292.750000	0.007039
1292.500000	0.006999
1292.250000	0.006957
1292.000000	0.006912
1291.750000	0.006867
1291.500000	0.006821
1291.250000	0.006776
1291.000000	0.006732
1290.750000	0.006690
1290.500000	0.006650
1290.250000	0.006611
1290.000000	0.006573
1289.750000	0.006534
1289.500000	0.006494

1289.250000	0.006454
1289.000000	0.006413
1288.750000	0.006372
1288.500000	0.006332
1288.250000	0.006295
1288.000000	0.006260
1287.750000	0.006228
1287.500000	0.006200
1287.250000	0.006175
1287.000000	0.006153
1286.750000	0.006133
1286.500000	0.006115
1286.250000	0.006096
1286.000000	0.006078
1285.750000	0.006059
1285.500000	0.006042
1285.250000	0.006026
1285.000000	0.006013
1284.750000	0.006003
1284.500000	0.005996
1284.250000	0.005990
1284.000000	0.005986
1283.750000	0.005984
1283.500000	0.005983
1283.250000	0.005984
1283.000000	0.005988

1282.750000	0.005997
1282.500000	0.006009
1282.250000	0.006024
1282.000000	0.006041
1281.750000	0.006061
1281.500000	0.006081
1281.250000	0.006102
1281.000000	0.006124
1280.750000	0.006147
1280.500000	0.006172
1280.250000	0.006197
1280.000000	0.006223
1279.750000	0.006250
1279.500000	0.006278
1279.250000	0.006307
1279.000000	0.006338
1278.750000	0.006371
1278.500000	0.006404
1278.250000	0.006438
1278.000000	0.006470
1277.750000	0.006502
1277.500000	0.006534
1277.250000	0.006566
1277.000000	0.006601
1276.750000	0.006638
1276.500000	0.006678

1276.250000	0.006721
1276.000000	0.006766
1275.750000	0.006812
1275.500000	0.006859
1275.250000	0.006905
1275.000000	0.006952
1274.750000	0.006998
1274.500000	0.007043
1274.250000	0.007088
1274.000000	0.007133
1273.750000	0.007177
1273.500000	0.007219
1273.250000	0.007261
1273.000000	0.007302
1272.750000	0.007342
1272.500000	0.007380
1272.250000	0.007418
1272.000000	0.007455
1271.750000	0.007493
1271.500000	0.007531
1271.250000	0.007570
1271.000000	0.007610
1270.750000	0.007652
1270.500000	0.007694
1270.250000	0.007738
1270.000000	0.007780

1269.750000	0.007823
1269.500000	0.007864
1269.250000	0.007904
1269.000000	0.007944
1268.750000	0.007983
1268.500000	0.008021
1268.250000	0.008058
1268.000000	0.008095
1267.750000	0.008132
1267.500000	0.008169
1267.250000	0.008207
1267.000000	0.008246
1266.750000	0.008287
1266.500000	0.008330
1266.250000	0.008374
1266.000000	0.008420
1265.750000	0.008466
1265.500000	0.008513
1265.250000	0.008560
1265.000000	0.008607
1264.750000	0.008654
1264.500000	0.008700
1264.250000	0.008746
1264.000000	0.008792
1263.750000	0.008839
1263.500000	0.008887

1263.250000	0.008935
1263.000000	0.008984
1262.750000	0.009033
1262.500000	0.009082
1262.250000	0.009131
1262.000000	0.009180
1261.750000	0.009229
1261.500000	0.009280
1261.250000	0.009333
1261.000000	0.009389
1260.750000	0.009449
1260.500000	0.009511
1260.250000	0.009574
1260.000000	0.009638
1259.750000	0.009700
1259.500000	0.009761
1259.250000	0.009819
1259.000000	0.009875
1258.750000	0.009929
1258.500000	0.009981
1258.250000	0.010031
1258.000000	0.010081
1257.750000	0.010132
1257.500000	0.010184
1257.250000	0.010237
1257.000000	0.010293

1256.750000	0.010351
1256.500000	0.010410
1256.250000	0.010471
1256.000000	0.010532
1255.750000	0.010591
1255.500000	0.010650
1255.250000	0.010707
1255.000000	0.010761
1254.750000	0.010813
1254.500000	0.010863
1254.250000	0.010911
1254.000000	0.010958
1253.750000	0.011003
1253.500000	0.011048
1253.250000	0.011092
1253.000000	0.011137
1252.750000	0.011181
1252.500000	0.011226
1252.250000	0.011270
1252.000000	0.011313
1251.750000	0.011355
1251.500000	0.011395
1251.250000	0.011434
1251.000000	0.011471
1250.750000	0.011506
1250.500000	0.011538

1250.250000	0.011569
1250.000000	0.011599
1249.750000	0.011626
1249.500000	0.011651
1249.250000	0.011675
1249.000000	0.011697
1248.750000	0.011718
1248.500000	0.011737
1248.250000	0.011755
1248.000000	0.011772
1247.750000	0.011788
1247.500000	0.011803
1247.250000	0.011815
1247.000000	0.011826
1246.750000	0.011835
1246.500000	0.011842
1246.250000	0.011848
1246.000000	0.011855
1245.750000	0.011863
1245.500000	0.011872
1245.250000	0.011884
1245.000000	0.011897
1244.750000	0.011910
1244.500000	0.011921
1244.250000	0.011930
1244.000000	0.011938

1243.750000	0.011943
1243.500000	0.011948
1243.250000	0.011954
1243.000000	0.011960
1242.750000	0.011967
1242.500000	0.011975
1242.250000	0.011984
1242.000000	0.011993
1241.750000	0.012002
1241.500000	0.012012
1241.250000	0.012022
1241.000000	0.012034
1240.750000	0.012047
1240.500000	0.012060
1240.250000	0.012075
1240.000000	0.012090
1239.750000	0.012106
1239.500000	0.012123
1239.250000	0.012139
1239.000000	0.012155
1238.750000	0.012170
1238.500000	0.012184
1238.250000	0.012196
1238.000000	0.012207
1237.750000	0.012217
1237.500000	0.012229

1237.250000	0.012242
1237.000000	0.012257
1236.750000	0.012274
1236.500000	0.012293
1236.250000	0.012311
1236.000000	0.012328
1235.750000	0.012343
1235.500000	0.012357
1235.250000	0.012368
1235.000000	0.012377
1234.750000	0.012383
1234.500000	0.012388
1234.250000	0.012391
1234.000000	0.012391
1233.750000	0.012389
1233.500000	0.012385
1233.250000	0.012379
1233.000000	0.012371
1232.750000	0.012360
1232.500000	0.012347
1232.250000	0.012333
1232.000000	0.012316
1231.750000	0.012298
1231.500000	0.012279
1231.250000	0.012258
1231.000000	0.012236

1230.750000	0.012212
1230.500000	0.012186
1230.250000	0.012157
1230.000000	0.012124
1229.750000	0.012088
1229.500000	0.012048
1229.250000	0.012006
1229.000000	0.011960
1228.750000	0.011912
1228.500000	0.011861
1228.250000	0.011808
1228.000000	0.011753
1227.750000	0.011697
1227.500000	0.011641
1227.250000	0.011586
1227.000000	0.011531
1226.750000	0.011477
1226.500000	0.011422
1226.250000	0.011366
1226.000000	0.011308
1225.750000	0.011247
1225.500000	0.011183
1225.250000	0.011118
1225.000000	0.011052
1224.750000	0.010986
1224.500000	0.010920

1224.250000	0.010856
1224.000000	0.010793
1223.750000	0.010730
1223.500000	0.010668
1223.250000	0.010607
1223.000000	0.010546
1222.750000	0.010486
1222.500000	0.010427
1222.250000	0.010371
1222.000000	0.010320
1221.750000	0.010275
1221.500000	0.010238
1221.250000	0.010213
1221.000000	0.010201
1220.750000	0.010203
1220.500000	0.010217
1220.250000	0.010242
1220.000000	0.010277
1219.750000	0.010318
1219.500000	0.010363
1219.250000	0.010411
1219.000000	0.010459
1218.750000	0.010506
1218.500000	0.010550
1218.250000	0.010589
1218.000000	0.010620

1217.750000	0.010640
1217.500000	0.010648
1217.250000	0.010642
1217.000000	0.010623
1216.750000	0.010593
1216.500000	0.010551
1216.250000	0.010502
1216.000000	0.010447
1215.750000	0.010388
1215.500000	0.010329
1215.250000	0.010269
1215.000000	0.010211
1214.750000	0.010156
1214.500000	0.010103
1214.250000	0.010053
1214.000000	0.010007
1213.750000	0.009965
1213.500000	0.009928
1213.250000	0.009897
1213.000000	0.009873
1212.750000	0.009855
1212.500000	0.009845
1212.250000	0.009840
1212.000000	0.009839
1211.750000	0.009840
1211.500000	0.009841

1211.250000	0.009841
1211.000000	0.009840
1210.750000	0.009838
1210.500000	0.009837
1210.250000	0.009839
1210.000000	0.009843
1209.750000	0.009852
1209.500000	0.009863
1209.250000	0.009878
1209.000000	0.009894
1208.750000	0.009911
1208.500000	0.009928
1208.250000	0.009946
1208.000000	0.009964
1207.750000	0.009983
1207.500000	0.010004
1207.250000	0.010026
1207.000000	0.010049
1206.750000	0.010073
1206.500000	0.010096
1206.250000	0.010119
1206.000000	0.010140
1205.750000	0.010161
1205.500000	0.010180
1205.250000	0.010198
1205.000000	0.010216

1204.750000	0.010233
1204.500000	0.010249
1204.250000	0.010265
1204.000000	0.010281
1203.750000	0.010297
1203.500000	0.010315
1203.250000	0.010335
1203.000000	0.010357
1202.750000	0.010382
1202.500000	0.010407
1202.250000	0.010432
1202.000000	0.010456
1201.750000	0.010479
1201.500000	0.010500
1201.250000	0.010519
1201.000000	0.010536
1200.750000	0.010551
1200.500000	0.010564
1200.250000	0.010575
1200.000000	0.010583
1199.750000	0.010591
1199.500000	0.010598
1199.250000	0.010606
1199.000000	0.010616
1198.750000	0.010630
1198.500000	0.010647

1198.250000	0.010667
1198.000000	0.010688
1197.750000	0.010710
1197.500000	0.010732
1197.250000	0.010752
1197.000000	0.010772
1196.750000	0.010791
1196.500000	0.010810
1196.250000	0.010830
1196.000000	0.010850
1195.750000	0.010871
1195.500000	0.010893
1195.250000	0.010915
1195.000000	0.010937
1194.750000	0.010960
1194.500000	0.010984
1194.250000	0.011009
1194.000000	0.011034
1193.750000	0.011060
1193.500000	0.011085
1193.250000	0.011110
1193.000000	0.011136
1192.750000	0.011163
1192.500000	0.011192
1192.250000	0.011223
1192.000000	0.011257

1191.750000	0.011293
1191.500000	0.011331
1191.250000	0.011369
1191.000000	0.011407
1190.750000	0.011446
1190.500000	0.011485
1190.250000	0.011524
1190.000000	0.011566
1189.750000	0.011608
1189.500000	0.011650
1189.250000	0.011693
1189.000000	0.011735
1188.750000	0.011777
1188.500000	0.011818
1188.250000	0.011861
1188.000000	0.011907
1187.750000	0.011956
1187.500000	0.012009
1187.250000	0.012064
1187.000000	0.012122
1186.750000	0.012180
1186.500000	0.012238
1186.250000	0.012294
1186.000000	0.012350
1185.750000	0.012405
1185.500000	0.012459

1185.250000	0.012514
1185.000000	0.012569
1184.750000	0.012623
1184.500000	0.012678
1184.250000	0.012732
1184.000000	0.012786
1183.750000	0.012839
1183.500000	0.012891
1183.250000	0.012942
1183.000000	0.012993
1182.750000	0.013041
1182.500000	0.013088
1182.250000	0.013134
1182.000000	0.013178
1181.750000	0.013222
1181.500000	0.013266
1181.250000	0.013310
1181.000000	0.013355
1180.750000	0.013401
1180.500000	0.013447
1180.250000	0.013493
1180.000000	0.013541
1179.750000	0.013590
1179.500000	0.013640
1179.250000	0.013690
1179.000000	0.013741

1178.750000	0.013792
1178.500000	0.013842
1178.250000	0.013891
1178.000000	0.013939
1177.750000	0.013986
1177.500000	0.014032
1177.250000	0.014077
1177.000000	0.014121
1176.750000	0.014165
1176.500000	0.014208
1176.250000	0.014252
1176.000000	0.014298
1175.750000	0.014346
1175.500000	0.014399
1175.250000	0.014456
1175.000000	0.014518
1174.750000	0.014582
1174.500000	0.014648
1174.250000	0.014715
1174.000000	0.014780
1173.750000	0.014845
1173.500000	0.014908
1173.250000	0.014970
1173.000000	0.015031
1172.750000	0.015092
1172.500000	0.015153

1172.250000	0.015215
1172.000000	0.015277
1171.750000	0.015341
1171.500000	0.015407
1171.250000	0.015474
1171.000000	0.015544
1170.750000	0.015614
1170.500000	0.015686
1170.250000	0.015758
1170.000000	0.015831
1169.750000	0.015903
1169.500000	0.015974
1169.250000	0.016043
1169.000000	0.016111
1168.750000	0.016176
1168.500000	0.016239
1168.250000	0.016300
1168.000000	0.016358
1167.750000	0.016415
1167.500000	0.016469
1167.250000	0.016520
1167.000000	0.016569
1166.750000	0.016614
1166.500000	0.016654
1166.250000	0.016692
1166.000000	0.016726

1165.750000	0.016759
1165.500000	0.016790
1165.250000	0.016821
1165.000000	0.016850
1164.750000	0.016877
1164.500000	0.016901
1164.250000	0.016921
1164.000000	0.016935
1163.750000	0.016945
1163.500000	0.016950
1163.250000	0.016952
1163.000000	0.016951
1162.750000	0.016949
1162.500000	0.016947
1162.250000	0.016947
1162.000000	0.016949
1161.750000	0.016954
1161.500000	0.016960
1161.250000	0.016967
1161.000000	0.016973
1160.750000	0.016977
1160.500000	0.016979
1160.250000	0.016979
1160.000000	0.016979
1159.750000	0.016979
1159.500000	0.016981

1159.250000	0.016986
1159.000000	0.016995
1158.750000	0.017008
1158.500000	0.017023
1158.250000	0.017041
1158.000000	0.017060
1157.750000	0.017080
1157.500000	0.017101
1157.250000	0.017122
1157.000000	0.017142
1156.750000	0.017162
1156.500000	0.017181
1156.250000	0.017200
1156.000000	0.017219
1155.750000	0.017238
1155.500000	0.017259
1155.250000	0.017281
1155.000000	0.017303
1154.750000	0.017326
1154.500000	0.017349
1154.250000	0.017373
1154.000000	0.017397
1153.750000	0.017422
1153.500000	0.017449
1153.250000	0.017476
1153.000000	0.017504

1152.750000	0.017533
1152.500000	0.017562
1152.250000	0.017590
1152.000000	0.017619
1151.750000	0.017647
1151.500000	0.017674
1151.250000	0.017701
1151.000000	0.017725
1150.750000	0.017747
1150.500000	0.017766
1150.250000	0.017783
1150.000000	0.017797
1149.750000	0.017810
1149.500000	0.017822
1149.250000	0.017835
1149.000000	0.017850
1148.750000	0.017864
1148.500000	0.017880
1148.250000	0.017896
1148.000000	0.017911
1147.750000	0.017925
1147.500000	0.017939
1147.250000	0.017952
1147.000000	0.017965
1146.750000	0.017977
1146.500000	0.017989

1146.250000	0.018000
1146.000000	0.018011
1145.750000	0.018021
1145.500000	0.018029
1145.250000	0.018035
1145.000000	0.018038
1144.750000	0.018039
1144.500000	0.018039
1144.250000	0.018039
1144.000000	0.018040
1143.750000	0.018044
1143.500000	0.018050
1143.250000	0.018059
1143.000000	0.018068
1142.750000	0.018077
1142.500000	0.018084
1142.250000	0.018089
1142.000000	0.018092
1141.750000	0.018093
1141.500000	0.018095
1141.250000	0.018096
1141.000000	0.018098
1140.750000	0.018099
1140.500000	0.018101
1140.250000	0.018102
1140.000000	0.018102

1139.750000	0.018100
1139.500000	0.018096
1139.250000	0.018090
1139.000000	0.018083
1138.750000	0.018073
1138.500000	0.018063
1138.250000	0.018053
1138.000000	0.018045
1137.750000	0.018040
1137.500000	0.018039
1137.250000	0.018039
1137.000000	0.018042
1136.750000	0.018045
1136.500000	0.018048
1136.250000	0.018050
1136.000000	0.018052
1135.750000	0.018054
1135.500000	0.018055
1135.250000	0.018056
1135.000000	0.018057
1134.750000	0.018057
1134.500000	0.018058
1134.250000	0.018060
1134.000000	0.018063
1133.750000	0.018070
1133.500000	0.018080

1133.250000	0.018092
1133.000000	0.018104
1132.750000	0.018116
1132.500000	0.018125
1132.250000	0.018131
1132.000000	0.018136
1131.750000	0.018139
1131.500000	0.018143
1131.250000	0.018146
1131.000000	0.018150
1130.750000	0.018153
1130.500000	0.018154
1130.250000	0.018155
1130.000000	0.018156
1129.750000	0.018157
1129.500000	0.018161
1129.250000	0.018168
1129.000000	0.018177
1128.750000	0.018189
1128.500000	0.018202
1128.250000	0.018214
1128.000000	0.018226
1127.750000	0.018239
1127.500000	0.018252
1127.250000	0.018267
1127.000000	0.018284

1126.750000	0.018304
1126.500000	0.018326
1126.250000	0.018348
1126.000000	0.018370
1125.750000	0.018393
1125.500000	0.018415
1125.250000	0.018437
1125.000000	0.018461
1124.750000	0.018486
1124.500000	0.018514
1124.250000	0.018544
1124.000000	0.018575
1123.750000	0.018608
1123.500000	0.018639
1123.250000	0.018669
1123.000000	0.018695
1122.750000	0.018717
1122.500000	0.018735
1122.250000	0.018751
1122.000000	0.018767
1121.750000	0.018783
1121.500000	0.018802
1121.250000	0.018823
1121.000000	0.018849
1120.750000	0.018876
1120.500000	0.018906

1120.250000	0.018936
1120.000000	0.018967
1119.750000	0.018998
1119.500000	0.019027
1119.250000	0.019055
1119.000000	0.019081
1118.750000	0.019104
1118.500000	0.019125
1118.250000	0.019144
1118.000000	0.019161
1117.750000	0.019179
1117.500000	0.019198
1117.250000	0.019218
1117.000000	0.019240
1116.750000	0.019264
1116.500000	0.019288
1116.250000	0.019314
1116.000000	0.019339
1115.750000	0.019365
1115.500000	0.019389
1115.250000	0.019410
1115.000000	0.019429
1114.750000	0.019445
1114.500000	0.019458
1114.250000	0.019470
1114.000000	0.019483

1113.750000	0.019498
1113.500000	0.019516
1113.250000	0.019536
1113.000000	0.019558
1112.750000	0.019579
1112.500000	0.019599
1112.250000	0.019617
1112.000000	0.019632
1111.750000	0.019647
1111.500000	0.019661
1111.250000	0.019674
1111.000000	0.019686
1110.750000	0.019697
1110.500000	0.019705
1110.250000	0.019713
1110.000000	0.019722
1109.750000	0.019733
1109.500000	0.019748
1109.250000	0.019768
1109.000000	0.019790
1108.750000	0.019814
1108.500000	0.019835
1108.250000	0.019851
1108.000000	0.019862
1107.750000	0.019867
1107.500000	0.019868

1107.250000	0.019867
1107.000000	0.019867
1106.750000	0.019868
1106.500000	0.019871
1106.250000	0.019876
1106.000000	0.019883
1105.750000	0.019892
1105.500000	0.019900
1105.250000	0.019908
1105.000000	0.019915
1104.750000	0.019921
1104.500000	0.019925
1104.250000	0.019930
1104.000000	0.019935
1103.750000	0.019940
1103.500000	0.019947
1103.250000	0.019956
1103.000000	0.019965
1102.750000	0.019975
1102.500000	0.019983
1102.250000	0.019991
1102.000000	0.019997
1101.750000	0.020004
1101.500000	0.020010
1101.250000	0.020017
1101.000000	0.020025

1100.750000	0.020034
1100.500000	0.020043
1100.250000	0.020053
1100.000000	0.020063
1099.750000	0.020074
1099.500000	0.020086
1099.250000	0.020096
1099.000000	0.020104
1098.750000	0.020110
1098.500000	0.020111
1098.250000	0.020110
1098.000000	0.020107
1097.750000	0.020103
1097.500000	0.020101
1097.250000	0.020101
1097.000000	0.020105
1096.750000	0.020114
1096.500000	0.020126
1096.250000	0.020142
1096.000000	0.020160
1095.750000	0.020180
1095.500000	0.020200
1095.250000	0.020218
1095.000000	0.020232
1094.750000	0.020242
1094.500000	0.020247

1094.250000	0.020248
1094.000000	0.020248
1093.750000	0.020248
1093.500000	0.020252
1093.250000	0.020261
1093.000000	0.020274
1092.750000	0.020290
1092.500000	0.020308
1092.250000	0.020325
1092.000000	0.020341
1091.750000	0.020356
1091.500000	0.020369
1091.250000	0.020381
1091.000000	0.020392
1090.750000	0.020401
1090.500000	0.020409
1090.250000	0.020414
1090.000000	0.020418
1089.750000	0.020422
1089.500000	0.020426
1089.250000	0.020431
1089.000000	0.020436
1088.750000	0.020442
1088.500000	0.020448
1088.250000	0.020453
1088.000000	0.020458

1087.750000	0.020463
1087.500000	0.020470
1087.250000	0.020480
1087.000000	0.020494
1086.750000	0.020510
1086.500000	0.020529
1086.250000	0.020549
1086.000000	0.020570
1085.750000	0.020591
1085.500000	0.020611
1085.250000	0.020631
1085.000000	0.020648
1084.750000	0.020663
1084.500000	0.020674
1084.250000	0.020682
1084.000000	0.020687
1083.750000	0.020692
1083.500000	0.020699
1083.250000	0.020710
1083.000000	0.020726
1082.750000	0.020747
1082.500000	0.020773
1082.250000	0.020800
1082.000000	0.020827
1081.750000	0.020851
1081.500000	0.020871

1081.250000	0.020885
1081.000000	0.020895
1080.750000	0.020901
1080.500000	0.020906
1080.250000	0.020912
1080.000000	0.020921
1079.750000	0.020934
1079.500000	0.020951
1079.250000	0.020971
1079.000000	0.020993
1078.750000	0.021013
1078.500000	0.021031
1078.250000	0.021046
1078.000000	0.021058
1077.750000	0.021070
1077.500000	0.021081
1077.250000	0.021092
1077.000000	0.021105
1076.750000	0.021117
1076.500000	0.021129
1076.250000	0.021141
1076.000000	0.021152
1075.750000	0.021165
1075.500000	0.021180
1075.250000	0.021198
1075.000000	0.021218

1074.750000	0.021240
1074.500000	0.021262
1074.250000	0.021281
1074.000000	0.021297
1073.750000	0.021310
1073.500000	0.021318
1073.250000	0.021323
1073.000000	0.021326
1072.750000	0.021327
1072.500000	0.021329
1072.250000	0.021330
1072.000000	0.021332
1071.750000	0.021337
1071.500000	0.021343
1071.250000	0.021352
1071.000000	0.021363
1070.750000	0.021375
1070.500000	0.021387
1070.250000	0.021399
1070.000000	0.021411
1069.750000	0.021423
1069.500000	0.021435
1069.250000	0.021448
1069.000000	0.021461
1068.750000	0.021475
1068.500000	0.021487

1068.250000	0.021498
1068.000000	0.021508
1067.750000	0.021518
1067.500000	0.021528
1067.250000	0.021541
1067.000000	0.021555
1066.750000	0.021570
1066.500000	0.021586
1066.250000	0.021601
1066.000000	0.021613
1065.750000	0.021624
1065.500000	0.021632
1065.250000	0.021639
1065.000000	0.021646
1064.750000	0.021652
1064.500000	0.021658
1064.250000	0.021664
1064.000000	0.021668
1063.750000	0.021672
1063.500000	0.021673
1063.250000	0.021672
1063.000000	0.021670
1062.750000	0.021667
1062.500000	0.021664
1062.250000	0.021662
1062.000000	0.021664

1061.750000	0.021668
1061.500000	0.021676
1061.250000	0.021687
1061.000000	0.021699
1060.750000	0.021711
1060.500000	0.021722
1060.250000	0.021730
1060.000000	0.021736
1059.750000	0.021739
1059.500000	0.021740
1059.250000	0.021740
1059.000000	0.021739
1058.750000	0.021738
1058.500000	0.021737
1058.250000	0.021736
1058.000000	0.021736
1057.750000	0.021737
1057.500000	0.021740
1057.250000	0.021743
1057.000000	0.021747
1056.750000	0.021753
1056.500000	0.021758
1056.250000	0.021764
1056.000000	0.021771
1055.750000	0.021777
1055.500000	0.021782

1055.250000	0.021786
1055.000000	0.021788
1054.750000	0.021785
1054.500000	0.021779
1054.250000	0.021769
1054.000000	0.021756
1053.750000	0.021742
1053.500000	0.021730
1053.250000	0.021722
1053.000000	0.021717
1052.750000	0.021717
1052.500000	0.021721
1052.250000	0.021727
1052.000000	0.021735
1051.750000	0.021743
1051.500000	0.021751
1051.250000	0.021758
1051.000000	0.021763
1050.750000	0.021767
1050.500000	0.021772
1050.250000	0.021778
1050.000000	0.021788
1049.750000	0.021801
1049.500000	0.021818
1049.250000	0.021839
1049.000000	0.021861

1048.750000	0.021882
1048.500000	0.021901
1048.250000	0.021915
1048.000000	0.021924
1047.750000	0.021927
1047.500000	0.021926
1047.250000	0.021922
1047.000000	0.021915
1046.750000	0.021907
1046.500000	0.021900
1046.250000	0.021894
1046.000000	0.021890
1045.750000	0.021888
1045.500000	0.021887
1045.250000	0.021888
1045.000000	0.021888
1044.750000	0.021887
1044.500000	0.021884
1044.250000	0.021880
1044.000000	0.021877
1043.750000	0.021873
1043.500000	0.021871
1043.250000	0.021871
1043.000000	0.021872
1042.750000	0.021874
1042.500000	0.021876

1042.250000	0.021879
1042.000000	0.021882
1041.750000	0.021886
1041.500000	0.021893
1041.250000	0.021903
1041.000000	0.021914
1040.750000	0.021926
1040.500000	0.021937
1040.250000	0.021944
1040.000000	0.021948
1039.750000	0.021948
1039.500000	0.021945
1039.250000	0.021940
1039.000000	0.021935
1038.750000	0.021931
1038.500000	0.021929
1038.250000	0.021930
1038.000000	0.021932
1037.750000	0.021936
1037.500000	0.021940
1037.250000	0.021942
1037.000000	0.021942
1036.750000	0.021940
1036.500000	0.021935
1036.250000	0.021930
1036.000000	0.021924

1035.750000	0.021921
1035.500000	0.021921
1035.250000	0.021926
1035.000000	0.021933
1034.750000	0.021943
1034.500000	0.021954
1034.250000	0.021964
1034.000000	0.021971
1033.750000	0.021975
1033.500000	0.021977
1033.250000	0.021974
1033.000000	0.021969
1032.750000	0.021961
1032.500000	0.021951
1032.250000	0.021941
1032.000000	0.021932
1031.750000	0.021924
1031.500000	0.021918
1031.250000	0.021915
1031.000000	0.021914
1030.750000	0.021914
1030.500000	0.021915
1030.250000	0.021916
1030.000000	0.021916
1029.750000	0.021915
1029.500000	0.021914

1029.250000	0.021912
1029.000000	0.021909
1028.750000	0.021905
1028.500000	0.021900
1028.250000	0.021895
1028.000000	0.021889
1027.750000	0.021883
1027.500000	0.021877
1027.250000	0.021870
1027.000000	0.021864
1026.750000	0.021858
1026.500000	0.021852
1026.250000	0.021847
1026.000000	0.021841
1025.750000	0.021835
1025.500000	0.021829
1025.250000	0.021820
1025.000000	0.021810
1024.750000	0.021797
1024.500000	0.021784
1024.250000	0.021769
1024.000000	0.021756
1023.750000	0.021745
1023.500000	0.021736
1023.250000	0.021728
1023.000000	0.021721

1022.750000	0.021714
1022.500000	0.021706
1022.250000	0.021697
1022.000000	0.021690
1021.750000	0.021684
1021.500000	0.021681
1021.250000	0.021681
1021.000000	0.021683
1020.750000	0.021684
1020.500000	0.021683
1020.250000	0.021678
1020.000000	0.021669
1019.750000	0.021657
1019.500000	0.021644
1019.250000	0.021630
1019.000000	0.021618
1018.750000	0.021608
1018.500000	0.021601
1018.250000	0.021596
1018.000000	0.021594
1017.750000	0.021594
1017.500000	0.021597
1017.250000	0.021600
1017.000000	0.021604
1016.750000	0.021606
1016.500000	0.021607

1016.250000	0.021605
1016.000000	0.021602
1015.750000	0.021597
1015.500000	0.021592
1015.250000	0.021588
1015.000000	0.021584
1014.750000	0.021582
1014.500000	0.021579
1014.250000	0.021576
1014.000000	0.021572
1013.750000	0.021567
1013.500000	0.021562
1013.250000	0.021558
1013.000000	0.021555
1012.750000	0.021554
1012.500000	0.021553
1012.250000	0.021554
1012.000000	0.021554
1011.750000	0.021553
1011.500000	0.021551
1011.250000	0.021547
1011.000000	0.021542
1010.750000	0.021534
1010.500000	0.021526
1010.250000	0.021517
1010.000000	0.021506

1009.750000	0.021495
1009.500000	0.021482
1009.250000	0.021468
1009.000000	0.021452
1008.750000	0.021435
1008.500000	0.021416
1008.250000	0.021396
1008.000000	0.021375
1007.750000	0.021355
1007.500000	0.021335
1007.250000	0.021317
1007.000000	0.021299
1006.750000	0.021283
1006.500000	0.021267
1006.250000	0.021253
1006.000000	0.021238
1005.750000	0.021224
1005.500000	0.021210
1005.250000	0.021195
1005.000000	0.021179
1004.750000	0.021160
1004.500000	0.021139
1004.250000	0.021116
1004.000000	0.021093
1003.750000	0.021069
1003.500000	0.021047

1003.250000	0.021025
1003.000000	0.021004
1002.750000	0.020984
1002.500000	0.020964
1002.250000	0.020943
1002.000000	0.020922
1001.750000	0.020900
1001.500000	0.020878
1001.250000	0.020856
1001.000000	0.020835
1000.750000	0.020814
1000.500000	0.020794
1000.250000	0.020776
1000.000000	0.020758
999.750000	0.020741
999.500000	0.020724
999.250000	0.020706
999.000000	0.020687
998.750000	0.020666
998.500000	0.020644
998.250000	0.020619
998.000000	0.020594
997.750000	0.020568
997.500000	0.020543
997.250000	0.020519
997.000000	0.020497

996.750000	0.020475
996.500000	0.020455
996.250000	0.020437
996.000000	0.020419
995.750000	0.020403
995.500000	0.020388
995.250000	0.020374
995.000000	0.020361
994.750000	0.020347
994.500000	0.020334
994.250000	0.020319
994.000000	0.020304
993.750000	0.020288
993.500000	0.020271
993.250000	0.020252
993.000000	0.020231
992.750000	0.020208
992.500000	0.020184
992.250000	0.020160
992.000000	0.020137
991.750000	0.020116
991.500000	0.020098
991.250000	0.020084
991.000000	0.020072
990.750000	0.020064
990.500000	0.020057

990.250000	0.020052
990.000000	0.020049
989.750000	0.020047
989.500000	0.020046
989.250000	0.020045
989.000000	0.020044
988.750000	0.020041
988.500000	0.020037
988.250000	0.020030
988.000000	0.020022
987.750000	0.020012
987.500000	0.020001
987.250000	0.019989
987.000000	0.019977
986.750000	0.019965
986.500000	0.019955
986.250000	0.019948
986.000000	0.019944
985.750000	0.019944
985.500000	0.019947
985.250000	0.019950
985.000000	0.019953
984.750000	0.019952
984.500000	0.019947
984.250000	0.019937
984.000000	0.019923

983.750000	0.019906
983.500000	0.019887
983.250000	0.019866
983.000000	0.019844
982.750000	0.019819
982.500000	0.019791
982.250000	0.019761
982.000000	0.019729
981.750000	0.019697
981.500000	0.019666
981.250000	0.019638
981.000000	0.019613
980.750000	0.019593
980.500000	0.019575
980.250000	0.019560
980.000000	0.019546
979.750000	0.019532
979.500000	0.019516
979.250000	0.019497
979.000000	0.019473
978.750000	0.019445
978.500000	0.019412
978.250000	0.019376
978.000000	0.019339
977.750000	0.019302
977.500000	0.019269

977.250000	0.019238
977.000000	0.019212
976.750000	0.019187
976.500000	0.019164
976.250000	0.019141
976.000000	0.019115
975.750000	0.019088
975.500000	0.019060
975.250000	0.019029
975.000000	0.018998
974.750000	0.018966
974.500000	0.018936
974.250000	0.018908
974.000000	0.018883
973.750000	0.018861
973.500000	0.018842
973.250000	0.018826
973.000000	0.018811
972.750000	0.018794
972.500000	0.018776
972.250000	0.018754
972.000000	0.018730
971.750000	0.018706
971.500000	0.018681
971.250000	0.018658
971.000000	0.018636

970.750000	0.018616
970.500000	0.018597
970.250000	0.018576
970.000000	0.018555
969.750000	0.018532
969.500000	0.018509
969.250000	0.018485
969.000000	0.018462
968.750000	0.018441
968.500000	0.018421
968.250000	0.018403
968.000000	0.018388
967.750000	0.018374
967.500000	0.018362
967.250000	0.018351
967.000000	0.018340
966.750000	0.018327
966.500000	0.018312
966.250000	0.018295
966.000000	0.018274
965.750000	0.018250
965.500000	0.018224
965.250000	0.018197
965.000000	0.018170
964.750000	0.018145
964.500000	0.018122

964.250000	0.018102
964.000000	0.018085
963.750000	0.018070
963.500000	0.018056
963.250000	0.018042
963.000000	0.018027
962.750000	0.018010
962.500000	0.017990
962.250000	0.017968
962.000000	0.017943
961.750000	0.017917
961.500000	0.017891
961.250000	0.017866
961.000000	0.017841
960.750000	0.017818
960.500000	0.017797
960.250000	0.017777
960.000000	0.017760
959.750000	0.017744
959.500000	0.017728
959.250000	0.017711
959.000000	0.017692
958.750000	0.017672
958.500000	0.017649
958.250000	0.017625
958.000000	0.017601

957.750000	0.017579
957.500000	0.017560
957.250000	0.017544
957.000000	0.017529
956.750000	0.017515
956.500000	0.017500
956.250000	0.017484
956.000000	0.017465
955.750000	0.017443
955.500000	0.017421
955.250000	0.017397
955.000000	0.017374
954.750000	0.017351
954.500000	0.017329
954.250000	0.017309
954.000000	0.017291
953.750000	0.017274
953.500000	0.017258
953.250000	0.017243
953.000000	0.017227
952.750000	0.017210
952.500000	0.017191
952.250000	0.017171
952.000000	0.017150
951.750000	0.017128
951.500000	0.017106

951.250000	0.017085
951.000000	0.017065
950.750000	0.017046
950.500000	0.017027
950.250000	0.017010
950.000000	0.016992
949.750000	0.016976
949.500000	0.016962
949.250000	0.016948
949.000000	0.016936
948.750000	0.016923
948.500000	0.016911
948.250000	0.016899
948.000000	0.016887
947.750000	0.016874
947.500000	0.016861
947.250000	0.016848
947.000000	0.016835
946.750000	0.016821
946.500000	0.016806
946.250000	0.016790
946.000000	0.016775
945.750000	0.016760
945.500000	0.016748
945.250000	0.016736
945.000000	0.016726

944.750000	0.016716
944.500000	0.016705
944.250000	0.016691
944.000000	0.016675
943.750000	0.016655
943.500000	0.016634
943.250000	0.016612
943.000000	0.016589
942.750000	0.016566
942.500000	0.016544
942.250000	0.016521
942.000000	0.016498
941.750000	0.016472
941.500000	0.016446
941.250000	0.016418
941.000000	0.016389
940.750000	0.016362
940.500000	0.016338
940.250000	0.016316
940.000000	0.016298
939.750000	0.016283
939.500000	0.016269
939.250000	0.016255
939.000000	0.016239
938.750000	0.016221
938.500000	0.016200

938.250000	0.016178
938.000000	0.016155
937.750000	0.016132
937.500000	0.016109
937.250000	0.016085
937.000000	0.016061
936.750000	0.016034
936.500000	0.016006
936.250000	0.015976
936.000000	0.015945
935.750000	0.015913
935.500000	0.015883
935.250000	0.015852
935.000000	0.015821
934.750000	0.015791
934.500000	0.015760
934.250000	0.015730
934.000000	0.015701
933.750000	0.015674
933.500000	0.015649
933.250000	0.015625
933.000000	0.015602
932.750000	0.015578
932.500000	0.015551
932.250000	0.015522
932.000000	0.015492

931.750000	0.015461
931.500000	0.015431
931.250000	0.015402
931.000000	0.015376
930.750000	0.015352
930.500000	0.015329
930.250000	0.015308
930.000000	0.015286
929.750000	0.015264
929.500000	0.015240
929.250000	0.015214
929.000000	0.015185
928.750000	0.015152
928.500000	0.015118
928.250000	0.015082
928.000000	0.015047
927.750000	0.015014
927.500000	0.014984
927.250000	0.014957
927.000000	0.014933
926.750000	0.014910
926.500000	0.014887
926.250000	0.014865
926.000000	0.014844
925.750000	0.014824
925.500000	0.014807

925.250000	0.014792
925.000000	0.014778
924.750000	0.014763
924.500000	0.014747
924.250000	0.014729
924.000000	0.014707
923.750000	0.014683
923.500000	0.014657
923.250000	0.014630
923.000000	0.014602
922.750000	0.014572
922.500000	0.014540
922.250000	0.014507
922.000000	0.014472
921.750000	0.014437
921.500000	0.014404
921.250000	0.014373
921.000000	0.014345
920.750000	0.014320
920.500000	0.014296
920.250000	0.014273
920.000000	0.014250
919.750000	0.014225
919.500000	0.014200
919.250000	0.014174
919.000000	0.014147

918.750000	0.014121
918.500000	0.014094
918.250000	0.014066
918.000000	0.014038
917.750000	0.014009
917.500000	0.013978
917.250000	0.013946
917.000000	0.013913
916.750000	0.013879
916.500000	0.013844
916.250000	0.013808
916.000000	0.013772
915.750000	0.013736
915.500000	0.013698
915.250000	0.013660
915.000000	0.013619
914.750000	0.013575
914.500000	0.013529
914.250000	0.013482
914.000000	0.013434
913.750000	0.013388
913.500000	0.013346
913.250000	0.013307
913.000000	0.013271
912.750000	0.013237
912.500000	0.013202

912.250000	0.013166
912.000000	0.013126
911.750000	0.013083
911.500000	0.013037
911.250000	0.012990
911.000000	0.012942
910.750000	0.012893
910.500000	0.012844
910.250000	0.012795
910.000000	0.012747
909.750000	0.012701
909.500000	0.012657
909.250000	0.012618
909.000000	0.012581
908.750000	0.012548
908.500000	0.012516
908.250000	0.012481
908.000000	0.012444
907.750000	0.012402
907.500000	0.012357
907.250000	0.012309
907.000000	0.012261
906.750000	0.012215
906.500000	0.012172
906.250000	0.012132
906.000000	0.012093

905.750000	0.012054
905.500000	0.012014
905.250000	0.011971
905.000000	0.011926
904.750000	0.011880
904.500000	0.011834
904.250000	0.011790
904.000000	0.011749
903.750000	0.011710
903.500000	0.011672
903.250000	0.011636
903.000000	0.011599
902.750000	0.011561
902.500000	0.011523
902.250000	0.011485
902.000000	0.011448
901.750000	0.011411
901.500000	0.011375
901.250000	0.011339
901.000000	0.011303
900.750000	0.011266
900.500000	0.011226
900.250000	0.011186
900.000000	0.011143
899.750000	0.011101
899.500000	0.011058

899.250000	0.011016
899.000000	0.010975
898.750000	0.010935
898.500000	0.010897
898.250000	0.010859
898.000000	0.010822
897.750000	0.010786
897.500000	0.010751
897.250000	0.010716
897.000000	0.010681
896.750000	0.010645
896.500000	0.010609
896.250000	0.010571
896.000000	0.010533
895.750000	0.010494
895.500000	0.010455
895.250000	0.010416
895.000000	0.010376
894.750000	0.010336
894.500000	0.010296
894.250000	0.010256
894.000000	0.010217
893.750000	0.010178
893.500000	0.010139
893.250000	0.010101
893.000000	0.010063

892.750000	0.010025
892.500000	0.009989
892.250000	0.009954
892.000000	0.009921
891.750000	0.009890
891.500000	0.009860
891.250000	0.009831
891.000000	0.009800
890.750000	0.009768
890.500000	0.009733
890.250000	0.009696
890.000000	0.009659
889.750000	0.009621
889.500000	0.009585
889.250000	0.009550
889.000000	0.009518
888.750000	0.009488
888.500000	0.009460
888.250000	0.009432
888.000000	0.009406
887.750000	0.009380
887.500000	0.009352
887.250000	0.009323
887.000000	0.009292
886.750000	0.009257
886.500000	0.009220

886.250000	0.009182
886.000000	0.009143
885.750000	0.009105
885.500000	0.009070
885.250000	0.009036
885.000000	0.009003
884.750000	0.008971
884.500000	0.008939
884.250000	0.008906
884.000000	0.008874
883.750000	0.008844
883.500000	0.008816
883.250000	0.008790
883.000000	0.008764
882.750000	0.008738
882.500000	0.008710
882.250000	0.008679
882.000000	0.008645
881.750000	0.008610
881.500000	0.008575
881.250000	0.008542
881.000000	0.008512
880.750000	0.008484
880.500000	0.008457
880.250000	0.008431
880.000000	0.008405

879.750000	0.008378
879.500000	0.008351
879.250000	0.008324
879.000000	0.008296
878.750000	0.008269
878.500000	0.008240
878.250000	0.008210
878.000000	0.008179
877.750000	0.008146
877.500000	0.008112
877.250000	0.008078
877.000000	0.008044
876.750000	0.008011
876.500000	0.007980
876.250000	0.007951
876.000000	0.007925
875.750000	0.007900
875.500000	0.007877
875.250000	0.007854
875.000000	0.007831
874.750000	0.007807
874.500000	0.007780
874.250000	0.007750
874.000000	0.007718
873.750000	0.007686
873.500000	0.007654

873.250000	0.007623
873.000000	0.007593
872.750000	0.007565
872.500000	0.007536
872.250000	0.007506
872.000000	0.007474
871.750000	0.007440
871.500000	0.007406
871.250000	0.007372
871.000000	0.007340
870.750000	0.007310
870.500000	0.007283
870.250000	0.007260
870.000000	0.007239
869.750000	0.007220
869.500000	0.007204
869.250000	0.007188
869.000000	0.007173
868.750000	0.007157
868.500000	0.007139
868.250000	0.007119
868.000000	0.007097
867.750000	0.007072
867.500000	0.007047
867.250000	0.007020
867.000000	0.006994

866.750000	0.006967
866.500000	0.006941
866.250000	0.006916
866.000000	0.006891
865.750000	0.006867
865.500000	0.006844
865.250000	0.006824
865.000000	0.006807
864.750000	0.006792
864.500000	0.006778
864.250000	0.006764
864.000000	0.006750
863.750000	0.006734
863.500000	0.006715
863.250000	0.006695
863.000000	0.006672
862.750000	0.006649
862.500000	0.006626
862.250000	0.006604
862.000000	0.006582
861.750000	0.006562
861.500000	0.006542
861.250000	0.006521
861.000000	0.006501
860.750000	0.006479
860.500000	0.006457

860.250000	0.006435
860.000000	0.006414
859.750000	0.006394
859.500000	0.006377
859.250000	0.006361
859.000000	0.006347
858.750000	0.006334
858.500000	0.006321
858.250000	0.006307
858.000000	0.006292
857.750000	0.006277
857.500000	0.006263
857.250000	0.006249
857.000000	0.006236
856.750000	0.006224
856.500000	0.006213
856.250000	0.006201
856.000000	0.006190
855.750000	0.006179
855.500000	0.006169
855.250000	0.006160
855.000000	0.006152
854.750000	0.006145
854.500000	0.006139
854.250000	0.006131
854.000000	0.006121

853.750000	0.006108
853.500000	0.006093
853.250000	0.006074
853.000000	0.006055
852.750000	0.006036
852.500000	0.006019
852.250000	0.006005
852.000000	0.005995
851.750000	0.005989
851.500000	0.005986
851.250000	0.005985
851.000000	0.005984
850.750000	0.005981
850.500000	0.005975
850.250000	0.005967
850.000000	0.005958
849.750000	0.005948
849.500000	0.005940
849.250000	0.005934
849.000000	0.005930
848.750000	0.005929
848.500000	0.005929
848.250000	0.005929
848.000000	0.005928
847.750000	0.005927
847.500000	0.005926

847.250000	0.005926
847.000000	0.005928
846.750000	0.005932
846.500000	0.005937
846.250000	0.005944
846.000000	0.005953
845.750000	0.005963
845.500000	0.005975
845.250000	0.005989
845.000000	0.006006
844.750000	0.006022
844.500000	0.006039
844.250000	0.006053
844.000000	0.006066
843.750000	0.006076
843.500000	0.006085
843.250000	0.006094
843.000000	0.006104
842.750000	0.006116
842.500000	0.006130
842.250000	0.006145
842.000000	0.006161
841.750000	0.006177
841.500000	0.006193
841.250000	0.006209
841.000000	0.006225

840.750000	0.006240
840.500000	0.006254
840.250000	0.006267
840.000000	0.006277
839.750000	0.006285
839.500000	0.006291
839.250000	0.006297
839.000000	0.006302
838.750000	0.006307
838.500000	0.006311
838.250000	0.006313
838.000000	0.006314
837.750000	0.006313
837.500000	0.006311
837.250000	0.006309
837.000000	0.006309
836.750000	0.006312
836.500000	0.006317
836.250000	0.006324
836.000000	0.006331
835.750000	0.006336
835.500000	0.006339
835.250000	0.006339
835.000000	0.006338
834.750000	0.006337
834.500000	0.006336

834.250000	0.006335
834.000000	0.006336
833.750000	0.006336
833.500000	0.006336
833.250000	0.006335
833.000000	0.006334
832.750000	0.006332
832.500000	0.006330
832.250000	0.006328
832.000000	0.006325
831.750000	0.006321
831.500000	0.006315
831.250000	0.006308
831.000000	0.006299
830.750000	0.006288
830.500000	0.006274
830.250000	0.006258
830.000000	0.006240
829.750000	0.006219
829.500000	0.006195
829.250000	0.006170
829.000000	0.006144
828.750000	0.006118
828.500000	0.006093
828.250000	0.006069
828.000000	0.006046

827.750000	0.006024
827.500000	0.006003
827.250000	0.005981
827.000000	0.005958
826.750000	0.005934
826.500000	0.005908
826.250000	0.005882
826.000000	0.005856
825.750000	0.005832
825.500000	0.005811
825.250000	0.005794
825.000000	0.005779
824.750000	0.005766
824.500000	0.005755
824.250000	0.005744
824.000000	0.005733
823.750000	0.005722
823.500000	0.005711
823.250000	0.005703
823.000000	0.005698
822.750000	0.005696
822.500000	0.005697
822.250000	0.005701
822.000000	0.005707
821.750000	0.005714
821.500000	0.005724

821.250000	0.005736
821.000000	0.005751
820.750000	0.005768
820.500000	0.005787
820.250000	0.005806
820.000000	0.005826
819.750000	0.005844
819.500000	0.005863
819.250000	0.005883
819.000000	0.005905
818.750000	0.005930
818.500000	0.005958
818.250000	0.005987
818.000000	0.006018
817.750000	0.006048
817.500000	0.006075
817.250000	0.006102
817.000000	0.006126
816.750000	0.006151
816.500000	0.006177
816.250000	0.006203
816.000000	0.006229
815.750000	0.006255
815.500000	0.006277
815.250000	0.006297
815.000000	0.006312

814.750000	0.006325
814.500000	0.006334
814.250000	0.006340
814.000000	0.006345
813.750000	0.006347
813.500000	0.006347
813.250000	0.006344
813.000000	0.006339
812.750000	0.006332
812.500000	0.006323
812.250000	0.006313
812.000000	0.006303
811.750000	0.006290
811.500000	0.006276
811.250000	0.006260
811.000000	0.006240
810.750000	0.006218
810.500000	0.006193
810.250000	0.006166
810.000000	0.006138
809.750000	0.006108
809.500000	0.006076
809.250000	0.006044
809.000000	0.006011
808.750000	0.005979
808.500000	0.005947

808.250000	0.005918
808.000000	0.005892
807.750000	0.005869
807.500000	0.005850
807.250000	0.005834
807.000000	0.005820
806.750000	0.005806
806.500000	0.005792
806.250000	0.005777
806.000000	0.005760
805.750000	0.005742
805.500000	0.005723
805.250000	0.005703
805.000000	0.005681
804.750000	0.005659
804.500000	0.005637
804.250000	0.005614
804.000000	0.005592
803.750000	0.005571
803.500000	0.005554
803.250000	0.005539
803.000000	0.005527
802.750000	0.005519
802.500000	0.005511
802.250000	0.005503
802.000000	0.005495

801.750000	0.005484
801.500000	0.005471
801.250000	0.005456
801.000000	0.005440
800.750000	0.005424
800.500000	0.005407
800.250000	0.005390
800.000000	0.005374
799.750000	0.005357
799.500000	0.005340
799.250000	0.005322
799.000000	0.005305
798.750000	0.005288
798.500000	0.005271
798.250000	0.005256
798.000000	0.005243
797.750000	0.005231
797.500000	0.005220
797.250000	0.005208
797.000000	0.005196
796.750000	0.005181
796.500000	0.005164
796.250000	0.005144
796.000000	0.005121
795.750000	0.005097
795.500000	0.005073

795.250000	0.005050
795.000000	0.005030
794.750000	0.005012
794.500000	0.004998
794.250000	0.004987
794.000000	0.004978
793.750000	0.004970
793.500000	0.004962
793.250000	0.004953
793.000000	0.004941
792.750000	0.004928
792.500000	0.004911
792.250000	0.004892
792.000000	0.004872
791.750000	0.004852
791.500000	0.004832
791.250000	0.004814
791.000000	0.004799
790.750000	0.004786
790.500000	0.004774
790.250000	0.004764
790.000000	0.004753
789.750000	0.004742
789.500000	0.004732
789.250000	0.004722
789.000000	0.004715

788.750000	0.004709
788.500000	0.004706
788.250000	0.004705
788.000000	0.004704
787.750000	0.004702
787.500000	0.004698
787.250000	0.004690
787.000000	0.004680
786.750000	0.004667
786.500000	0.004654
786.250000	0.004642
786.000000	0.004632
785.750000	0.004627
785.500000	0.004626
785.250000	0.004631
785.000000	0.004642
784.750000	0.004658
784.500000	0.004679
784.250000	0.004704
784.000000	0.004731
783.750000	0.004757
783.500000	0.004782
783.250000	0.004805
783.000000	0.004825
782.750000	0.004844
782.500000	0.004863

782.250000	0.004883
782.000000	0.004906
781.750000	0.004933
781.500000	0.004964
781.250000	0.004999
781.000000	0.005037
780.750000	0.005075
780.500000	0.005112
780.250000	0.005147
780.000000	0.005178
779.750000	0.005205
779.500000	0.005228
779.250000	0.005247
779.000000	0.005263
778.750000	0.005276
778.500000	0.005288
778.250000	0.005300
778.000000	0.005312
777.750000	0.005325
777.500000	0.005339
777.250000	0.005354
777.000000	0.005368
776.750000	0.005381
776.500000	0.005392
776.250000	0.005402
776.000000	0.005411

775.750000	0.005419
775.500000	0.005426
775.250000	0.005431
775.000000	0.005435
774.750000	0.005436
774.500000	0.005433
774.250000	0.005426
774.000000	0.005414
773.750000	0.005398
773.500000	0.005379
773.250000	0.005356
773.000000	0.005330
772.750000	0.005301
772.500000	0.005269
772.250000	0.005233
772.000000	0.005193
771.750000	0.005150
771.500000	0.005105
771.250000	0.005058
771.000000	0.005011
770.750000	0.004966
770.500000	0.004924
770.250000	0.004884
770.000000	0.004848
769.750000	0.004815
769.500000	0.004785

769.250000	0.004757
769.000000	0.004731
768.750000	0.004707
768.500000	0.004684
768.250000	0.004662
768.000000	0.004641
767.750000	0.004620
767.500000	0.004599
767.250000	0.004578
767.000000	0.004557
766.750000	0.004537
766.500000	0.004517
766.250000	0.004500
766.000000	0.004485
765.750000	0.004473
765.500000	0.004465
765.250000	0.004460
765.000000	0.004458
764.750000	0.004459
764.500000	0.004460
764.250000	0.004461
764.000000	0.004462
763.750000	0.004463
763.500000	0.004465
763.250000	0.004466
763.000000	0.004469

762.750000	0.004473
762.500000	0.004476
762.250000	0.004477
762.000000	0.004476
761.750000	0.004469
761.500000	0.004457
761.250000	0.004440
761.000000	0.004418
760.750000	0.004392
760.500000	0.004364
760.250000	0.004334
760.000000	0.004304
759.750000	0.004273
759.500000	0.004242
759.250000	0.004212
759.000000	0.004181
758.750000	0.004152
758.500000	0.004123
758.250000	0.004096
758.000000	0.004070
757.750000	0.004046
757.500000	0.004024
757.250000	0.004001
757.000000	0.003979
756.750000	0.003957
756.500000	0.003934

756.250000	0.003911
756.000000	0.003888
755.750000	0.003866
755.500000	0.003846
755.250000	0.003827
755.000000	0.003811
754.750000	0.003799
754.500000	0.003790
754.250000	0.003786
754.000000	0.003787
753.750000	0.003791
753.500000	0.003799
753.250000	0.003808
753.000000	0.003817
752.750000	0.003823
752.500000	0.003824
752.250000	0.003820
752.000000	0.003812
751.750000	0.003802
751.500000	0.003793
751.250000	0.003786
751.000000	0.003784
750.750000	0.003785
750.500000	0.003788
750.250000	0.003792
750.000000	0.003794

749.750000	0.003793
749.500000	0.003790
749.250000	0.003786
749.000000	0.003782
748.750000	0.003779
748.500000	0.003780
748.250000	0.003785
748.000000	0.003791
747.750000	0.003798
747.500000	0.003804
747.250000	0.003808
747.000000	0.003808
746.750000	0.003806
746.500000	0.003801
746.250000	0.003793
746.000000	0.003782
745.750000	0.003769
745.500000	0.003752
745.250000	0.003732
745.000000	0.003709
744.750000	0.003684
744.500000	0.003657
744.250000	0.003629
744.000000	0.003601
743.750000	0.003574
743.500000	0.003549

743.250000	0.003525
743.000000	0.003502
742.750000	0.003481
742.500000	0.003461
742.250000	0.003441
742.000000	0.003421
741.750000	0.003402
741.500000	0.003382
741.250000	0.003363
741.000000	0.003345
740.750000	0.003327
740.500000	0.003311
740.250000	0.003297
740.000000	0.003285
739.750000	0.003275
739.500000	0.003269
739.250000	0.003264
739.000000	0.003260
738.750000	0.003256
738.500000	0.003251
738.250000	0.003244
738.000000	0.003235
737.750000	0.003224
737.500000	0.003214
737.250000	0.003206
737.000000	0.003201

736.750000	0.003201
736.500000	0.003204
736.250000	0.003209
736.000000	0.003214
735.750000	0.003217
735.500000	0.003217
735.250000	0.003214
735.000000	0.003207
734.750000	0.003198
734.500000	0.003189
734.250000	0.003181
734.000000	0.003175
733.750000	0.003172
733.500000	0.003171
733.250000	0.003172
733.000000	0.003175
732.750000	0.003178
732.500000	0.003182
732.250000	0.003185
732.000000	0.003189
731.750000	0.003192
731.500000	0.003194
731.250000	0.003195
731.000000	0.003194
730.750000	0.003192
730.500000	0.003189

730.250000	0.003184
730.000000	0.003180
729.750000	0.003176
729.500000	0.003174
729.250000	0.003174
729.000000	0.003175
728.750000	0.003177
728.500000	0.003176
728.250000	0.003173
728.000000	0.003166
727.750000	0.003154
727.500000	0.003138
727.250000	0.003121
727.000000	0.003103
726.750000	0.003087
726.500000	0.003074
726.250000	0.003065
726.000000	0.003060
725.750000	0.003059
725.500000	0.003059
725.250000	0.003061
725.000000	0.003062
724.750000	0.003063
724.500000	0.003063
724.250000	0.003062
724.000000	0.003059

723.750000	0.003057
723.500000	0.003054
723.250000	0.003051
723.000000	0.003046
722.750000	0.003040
722.500000	0.003030
722.250000	0.003016
722.000000	0.002998
721.750000	0.002978
721.500000	0.002957
721.250000	0.002939
721.000000	0.002925
720.750000	0.002917
720.500000	0.002913
720.250000	0.002913
720.000000	0.002913
719.750000	0.002909
719.500000	0.002901
719.250000	0.002887
719.000000	0.002868
718.750000	0.002847
718.500000	0.002826
718.250000	0.002806
718.000000	0.002791
717.750000	0.002780
717.500000	0.002773

717.250000	0.002769
717.000000	0.002765
716.750000	0.002761
716.500000	0.002755
716.250000	0.002747
716.000000	0.002736
715.750000	0.002725
715.500000	0.002714
715.250000	0.002706
715.000000	0.002702
714.750000	0.002701
714.500000	0.002704
714.250000	0.002708
714.000000	0.002713
713.750000	0.002716
713.500000	0.002716
713.250000	0.002712
713.000000	0.002705
712.750000	0.002696
712.500000	0.002685
712.250000	0.002674
712.000000	0.002665
711.750000	0.002656
711.500000	0.002650
711.250000	0.002647
711.000000	0.002646

710.750000	0.002647
710.500000	0.002648
710.250000	0.002649
710.000000	0.002648
709.750000	0.002645
709.500000	0.002639
709.250000	0.002631
709.000000	0.002623
708.750000	0.002614
708.500000	0.002607
708.250000	0.002603
708.000000	0.002602
707.750000	0.002604
707.500000	0.002610
707.250000	0.002618
707.000000	0.002629
706.750000	0.002640
706.500000	0.002650
706.250000	0.002655
706.000000	0.002654
705.750000	0.002647
705.500000	0.002635
705.250000	0.002619
705.000000	0.002604
704.750000	0.002592
704.500000	0.002586

704.250000	0.002585
704.000000	0.002590
703.750000	0.002598
703.500000	0.002606
703.250000	0.002614
703.000000	0.002620
702.750000	0.002625
702.500000	0.002629
702.250000	0.002633
702.000000	0.002637
701.750000	0.002640
701.500000	0.002641
701.250000	0.002640
701.000000	0.002636
700.750000	0.002629
700.500000	0.002621
700.250000	0.002614
700.000000	0.002609
699.750000	0.002607
699.500000	0.002608
699.250000	0.002611
699.000000	0.002615
698.750000	0.002618
698.500000	0.002620
698.250000	0.002621
698.000000	0.002620

697.750000	0.002618
697.500000	0.002616
697.250000	0.002615
697.000000	0.002613
696.750000	0.002610
696.500000	0.002606
696.250000	0.002601
696.000000	0.002593
695.750000	0.002585
695.500000	0.002576
695.250000	0.002568
695.000000	0.002561
694.750000	0.002556
694.500000	0.002553
694.250000	0.002552
694.000000	0.002552
693.750000	0.002552
693.500000	0.002551
693.250000	0.002549
693.000000	0.002546
692.750000	0.002541
692.500000	0.002534
692.250000	0.002526
692.000000	0.002515
691.750000	0.002502
691.500000	0.002487

691.250000	0.002470
691.000000	0.002453
690.750000	0.002435
690.500000	0.002419
690.250000	0.002405
690.000000	0.002393
689.750000	0.002384
689.500000	0.002378
689.250000	0.002375
689.000000	0.002373
688.750000	0.002372
688.500000	0.002370
688.250000	0.002366
688.000000	0.002360
687.750000	0.002350
687.500000	0.002340
687.250000	0.002330
687.000000	0.002322
686.750000	0.002317
686.500000	0.002318
686.250000	0.002322
686.000000	0.002328
685.750000	0.002335
685.500000	0.002340
685.250000	0.002341
685.000000	0.002339

684.750000	0.002334
684.500000	0.002326
684.250000	0.002318
684.000000	0.002310
683.750000	0.002304
683.500000	0.002299
683.250000	0.002295
683.000000	0.002293
682.750000	0.002291
682.500000	0.002288
682.250000	0.002284
682.000000	0.002278
681.750000	0.002269
681.500000	0.002259
681.250000	0.002248
681.000000	0.002237
680.750000	0.002227
680.500000	0.002220
680.250000	0.002216
680.000000	0.002214
679.750000	0.002216
679.500000	0.002219
679.250000	0.002225
679.000000	0.002231
678.750000	0.002238
678.500000	0.002244

678.250000	0.002247
678.000000	0.002246
677.750000	0.002242
677.500000	0.002236
677.250000	0.002228
677.000000	0.002222
676.750000	0.002217
676.500000	0.002216
676.250000	0.002217
676.000000	0.002220
675.750000	0.002223
675.500000	0.002225
675.250000	0.002227
675.000000	0.002228
674.750000	0.002229
674.500000	0.002229
674.250000	0.002227
674.000000	0.002224
673.750000	0.002219
673.500000	0.002213
673.250000	0.002205
673.000000	0.002199
672.750000	0.002193
672.500000	0.002189
672.250000	0.002183
672.000000	0.002177

671.750000	0.002168
671.500000	0.002155
671.250000	0.002136
671.000000	0.002110
670.750000	0.002081
670.500000	0.002047
670.250000	0.002005
670.000000	0.001950
669.750000	0.001888
669.500000	0.001822
669.250000	0.001747
669.000000	0.001664
668.750000	0.001609
668.500000	0.001650
668.250000	0.001835
668.000000	0.002129
667.750000	0.002426
667.500000	0.002612
667.250000	0.002649
667.000000	0.002581
666.750000	0.002481
666.500000	0.002388
666.250000	0.002308
666.000000	0.002236
665.750000	0.002174
665.500000	0.002126

665.250000	0.002090
665.000000	0.002061
664.750000	0.002037
664.500000	0.002019
664.250000	0.002007
664.000000	0.002001
663.750000	0.001999
663.500000	0.002002
663.250000	0.002009
663.000000	0.002018
662.750000	0.002026
662.500000	0.002031
662.250000	0.002031
662.000000	0.002027
661.750000	0.002018
661.500000	0.002005
661.250000	0.001989
661.000000	0.001973
660.750000	0.001958
660.500000	0.001946
660.250000	0.001938
660.000000	0.001935
659.750000	0.001936
659.500000	0.001941
659.250000	0.001946
659.000000	0.001951

658.750000	0.001954
658.500000	0.001952
658.250000	0.001948
658.000000	0.001939
657.750000	0.001927
657.500000	0.001913
657.250000	0.001897
657.000000	0.001880
656.750000	0.001865
656.500000	0.001853
656.250000	0.001846
656.000000	0.001843
655.750000	0.001845
655.500000	0.001850
655.250000	0.001857
655.000000	0.001862
654.750000	0.001865
654.500000	0.001863
654.250000	0.001859
654.000000	0.001851
653.750000	0.001841
653.500000	0.001830
653.250000	0.001819
653.000000	0.001809
652.750000	0.001800
652.500000	0.001791

652.250000	0.001784
652.000000	0.001778
651.750000	0.001771
651.500000	0.001765
651.250000	0.001758
651.000000	0.001750
650.750000	0.001741
650.500000	0.001732
650.250000	0.001722
650.000000	0.001713
649.750000	0.001704
649.500000	0.001695
649.250000	0.001686
649.000000	0.001677
648.750000	0.001669
648.500000	0.001661
648.250000	0.001653
648.000000	0.001647
647.750000	0.001642
647.500000	0.001638
647.250000	0.001634
647.000000	0.001630
646.750000	0.001625
646.500000	0.001620
646.250000	0.001613
646.000000	0.001606

645.750000	0.001597
645.500000	0.001587
645.250000	0.001575
645.000000	0.001562
644.750000	0.001549
644.500000	0.001535
644.250000	0.001523
644.000000	0.001513
643.750000	0.001504
643.500000	0.001499
643.250000	0.001494
643.000000	0.001491
642.750000	0.001486
642.500000	0.001481
642.250000	0.001473
642.000000	0.001461
641.750000	0.001447
641.500000	0.001428
641.250000	0.001407
641.000000	0.001386
640.750000	0.001366
640.500000	0.001350
640.250000	0.001341
640.000000	0.001340
639.750000	0.001348
639.500000	0.001363

639.250000	0.001384
639.000000	0.001407
638.750000	0.001429
638.500000	0.001448
638.250000	0.001459
638.000000	0.001462
637.750000	0.001455
637.500000	0.001438
637.250000	0.001414
637.000000	0.001385
636.750000	0.001355
636.500000	0.001330
636.250000	0.001313
636.000000	0.001306
635.750000	0.001308
635.500000	0.001319
635.250000	0.001333
635.000000	0.001348
634.750000	0.001360
634.500000	0.001366
634.250000	0.001368
634.000000	0.001366
633.750000	0.001362
633.500000	0.001357
633.250000	0.001352
633.000000	0.001348

632.750000	0.001346
632.500000	0.001344
632.250000	0.001344
632.000000	0.001344
631.750000	0.001344
631.500000	0.001344
631.250000	0.001341
631.000000	0.001335
630.750000	0.001325
630.500000	0.001310
630.250000	0.001294
630.000000	0.001277
629.750000	0.001262
629.500000	0.001250
629.250000	0.001242
629.000000	0.001237
628.750000	0.001235
628.500000	0.001233
628.250000	0.001231
628.000000	0.001226
627.750000	0.001220
627.500000	0.001214
627.250000	0.001208
627.000000	0.001203
626.750000	0.001201
626.500000	0.001203

626.250000	0.001206
626.000000	0.001210
625.750000	0.001215
625.500000	0.001218
625.250000	0.001220
625.000000	0.001220
624.750000	0.001219
624.500000	0.001218
624.250000	0.001216
624.000000	0.001215
623.750000	0.001214
623.500000	0.001215
623.250000	0.001216
623.000000	0.001219
622.750000	0.001223
622.500000	0.001229
622.250000	0.001236
622.000000	0.001243
621.750000	0.001249
621.500000	0.001251
621.250000	0.001250
621.000000	0.001243
620.750000	0.001233
620.500000	0.001223
620.250000	0.001214
620.000000	0.001210

619.750000	0.001212
619.500000	0.001222
619.250000	0.001237
619.000000	0.001255
618.750000	0.001275
618.500000	0.001293
618.250000	0.001307
618.000000	0.001316
617.750000	0.001318
617.500000	0.001315
617.250000	0.001306
617.000000	0.001292
616.750000	0.001277
616.500000	0.001262
616.250000	0.001250
616.000000	0.001243
615.750000	0.001242
615.500000	0.001247
615.250000	0.001255
615.000000	0.001264
614.750000	0.001272
614.500000	0.001277
614.250000	0.001279
614.000000	0.001277
613.750000	0.001274
613.500000	0.001270

613.250000	0.001266
613.000000	0.001261
612.750000	0.001256
612.500000	0.001250
612.250000	0.001243
612.000000	0.001235
611.750000	0.001228
611.500000	0.001221
611.250000	0.001215
611.000000	0.001209
610.750000	0.001204
610.500000	0.001198
610.250000	0.001193
610.000000	0.001190
609.750000	0.001188
609.500000	0.001191
609.250000	0.001198
609.000000	0.001208
608.750000	0.001220
608.500000	0.001232
608.250000	0.001243
608.000000	0.001250
607.750000	0.001255
607.500000	0.001256
607.250000	0.001256
607.000000	0.001255

606.750000	0.001255
606.500000	0.001256
606.250000	0.001259
606.000000	0.001264
605.750000	0.001269
605.500000	0.001275
605.250000	0.001280
605.000000	0.001283
604.750000	0.001283
604.500000	0.001280
604.250000	0.001273
604.000000	0.001265
603.750000	0.001256
603.500000	0.001250
603.250000	0.001247
603.000000	0.001248
602.750000	0.001251
602.500000	0.001256
602.250000	0.001261
602.000000	0.001265
601.750000	0.001266
601.500000	0.001265
601.250000	0.001263
601.000000	0.001262
600.750000	0.001263
600.500000	0.001268

600.250000	0.001275
600.000000	0.001285
599.750000	0.001296
599.500000	0.001306
599.250000	0.001314
599.000000	0.001320
598.750000	0.001323
598.500000	0.001324
598.250000	0.001324
598.000000	0.001324
597.750000	0.001323
597.500000	0.001322
597.250000	0.001320
597.000000	0.001318
596.750000	0.001315
596.500000	0.001312
596.250000	0.001309
596.000000	0.001308
595.750000	0.001309
595.500000	0.001312
595.250000	0.001316
595.000000	0.001321
594.750000	0.001325
594.500000	0.001327
594.250000	0.001327
594.000000	0.001324

593.750000	0.001318
593.500000	0.001312
593.250000	0.001307
593.000000	0.001303
592.750000	0.001302
592.500000	0.001304
592.250000	0.001310
592.000000	0.001316
591.750000	0.001321
591.500000	0.001322
591.250000	0.001318
591.000000	0.001308
590.750000	0.001297
590.500000	0.001285
590.250000	0.001275
590.000000	0.001271
589.750000	0.001272
589.500000	0.001277
589.250000	0.001285
589.000000	0.001292
588.750000	0.001296
588.500000	0.001296
588.250000	0.001292
588.000000	0.001287
587.750000	0.001281
587.500000	0.001278

587.250000	0.001277
587.000000	0.001279
586.750000	0.001282
586.500000	0.001283
586.250000	0.001281
586.000000	0.001276
585.750000	0.001269
585.500000	0.001260
585.250000	0.001253
585.000000	0.001248
584.750000	0.001248
584.500000	0.001253
584.250000	0.001262
584.000000	0.001274
583.750000	0.001286
583.500000	0.001298
583.250000	0.001307
583.000000	0.001311
582.750000	0.001310
582.500000	0.001303
582.250000	0.001291
582.000000	0.001275
581.750000	0.001258
581.500000	0.001242
581.250000	0.001231
581.000000	0.001226

580.750000	0.001228
580.500000	0.001236
580.250000	0.001248
580.000000	0.001263
579.750000	0.001278
579.500000	0.001291
579.250000	0.001302
579.000000	0.001310
578.750000	0.001316
578.500000	0.001320
578.250000	0.001320
578.000000	0.001317
577.750000	0.001309
577.500000	0.001297
577.250000	0.001283
577.000000	0.001269
576.750000	0.001258
576.500000	0.001254
576.250000	0.001258
576.000000	0.001270
575.750000	0.001287
575.500000	0.001306
575.250000	0.001323
575.000000	0.001333
574.750000	0.001335
574.500000	0.001330

574.250000	0.001318
574.000000	0.001302
573.750000	0.001285
573.500000	0.001269
573.250000	0.001257
573.000000	0.001249
572.750000	0.001246
572.500000	0.001248
572.250000	0.001255
572.000000	0.001265
571.750000	0.001275
571.500000	0.001284
571.250000	0.001289
571.000000	0.001288
570.750000	0.001280
570.500000	0.001267
570.250000	0.001250
570.000000	0.001233
569.750000	0.001219
569.500000	0.001210
569.250000	0.001207
569.000000	0.001210
568.750000	0.001217
568.500000	0.001227
568.250000	0.001237
568.000000	0.001245

567.750000	0.001250
567.500000	0.001252
567.250000	0.001248
567.000000	0.001240
566.750000	0.001228
566.500000	0.001212
566.250000	0.001194
566.000000	0.001178
565.750000	0.001166
565.500000	0.001159
565.250000	0.001159
565.000000	0.001164
564.750000	0.001173
564.500000	0.001182
564.250000	0.001190
564.000000	0.001194
563.750000	0.001194
563.500000	0.001192
563.250000	0.001188
563.000000	0.001184
562.750000	0.001181
562.500000	0.001177
562.250000	0.001173
562.000000	0.001167
561.750000	0.001159
561.500000	0.001150

561.250000	0.001142
561.000000	0.001137
560.750000	0.001137
560.500000	0.001142
560.250000	0.001152
560.000000	0.001164
559.750000	0.001177
559.500000	0.001188
559.250000	0.001194
559.000000	0.001194
558.750000	0.001189
558.500000	0.001180
558.250000	0.001167
558.000000	0.001155
557.750000	0.001146
557.500000	0.001141
557.250000	0.001143
557.000000	0.001151
556.750000	0.001163
556.500000	0.001176
556.250000	0.001186
556.000000	0.001190
555.750000	0.001188
555.500000	0.001180
555.250000	0.001169
555.000000	0.001159

554.750000	0.001152
554.500000	0.001150
554.250000	0.001154
554.000000	0.001160
553.750000	0.001167
553.500000	0.001170
553.250000	0.001168
553.000000	0.001160
552.750000	0.001147
552.500000	0.001129
552.250000	0.001110
552.000000	0.001092
551.750000	0.001074
551.500000	0.001059
551.250000	0.001045
551.000000	0.001032
550.750000	0.001020
550.500000	0.001009
550.250000	0.001000
550.000000	0.000993
549.750000	0.000988
549.500000	0.000986
549.250000	0.000986
549.000000	0.000988
548.750000	0.000991
548.500000	0.000995

548.250000	0.000997
548.000000	0.000996
547.750000	0.000993
547.500000	0.000986
547.250000	0.000976
547.000000	0.000963
546.750000	0.000950
546.500000	0.000937
546.250000	0.000926
546.000000	0.000917
545.750000	0.000911
545.500000	0.000909
545.250000	0.000910
545.000000	0.000913
544.750000	0.000918
544.500000	0.000922
544.250000	0.000926
544.000000	0.000926
543.750000	0.000923
543.500000	0.000916
543.250000	0.000906
543.000000	0.000895
542.750000	0.000886
542.500000	0.000881
542.250000	0.000883
542.000000	0.000890

541.750000	0.000901
541.500000	0.000914
541.250000	0.000925
541.000000	0.000932
540.750000	0.000932
540.500000	0.000925
540.250000	0.000914
540.000000	0.000899
539.750000	0.000883
539.500000	0.000868
539.250000	0.000854
539.000000	0.000841
538.750000	0.000828
538.500000	0.000814
538.250000	0.000799
538.000000	0.000784
537.750000	0.000768
537.500000	0.000754
537.250000	0.000743
537.000000	0.000736
536.750000	0.000733
536.500000	0.000734
536.250000	0.000739
536.000000	0.000745
535.750000	0.000751
535.500000	0.000755

535.250000	0.000758
535.000000	0.000759
534.750000	0.000758
534.500000	0.000757
534.250000	0.000754
534.000000	0.000750
533.750000	0.000745
533.500000	0.000739
533.250000	0.000731
533.000000	0.000724
532.750000	0.000716
532.500000	0.000709
532.250000	0.000703
532.000000	0.000696
531.750000	0.000691
531.500000	0.000686
531.250000	0.000685
531.000000	0.000689
530.750000	0.000700
530.500000	0.000720
530.250000	0.000747
530.000000	0.000778
529.750000	0.000811
529.500000	0.000839
529.250000	0.000859
529.000000	0.000869

528.750000	0.000868
528.500000	0.000858
528.250000	0.000841
528.000000	0.000820
527.750000	0.000796
527.500000	0.000771
527.250000	0.000745
527.000000	0.000722
526.750000	0.000704
526.500000	0.000696
526.250000	0.000700
526.000000	0.000718
525.750000	0.000746
525.500000	0.000776
525.250000	0.000801
525.000000	0.000816
524.750000	0.000820
524.500000	0.000814
524.250000	0.000802
524.000000	0.000788
523.750000	0.000777
523.500000	0.000771
523.250000	0.000769
523.000000	0.000772
522.750000	0.000774
522.500000	0.000774

522.250000	0.000769
522.000000	0.000759
521.750000	0.000744
521.500000	0.000726
521.250000	0.000710
521.000000	0.000697
520.750000	0.000691
520.500000	0.000691
520.250000	0.000698
520.000000	0.000709
519.750000	0.000721
519.500000	0.000729
519.250000	0.000729
519.000000	0.000721
518.750000	0.000705
518.500000	0.000685
518.250000	0.000667
518.000000	0.000657
517.750000	0.000660
517.500000	0.000679
517.250000	0.000712
517.000000	0.000754
516.750000	0.000797
516.500000	0.000831
516.250000	0.000853
516.000000	0.000860

515.750000	0.000856
515.500000	0.000845
515.250000	0.000831
515.000000	0.000817
514.750000	0.000807
514.500000	0.000802
514.250000	0.000803
514.000000	0.000809
513.750000	0.000818
513.500000	0.000829
513.250000	0.000838
513.000000	0.000845
512.750000	0.000848
512.500000	0.000846
512.250000	0.000843
512.000000	0.000838
511.750000	0.000836
511.500000	0.000837
511.250000	0.000843
511.000000	0.000855
510.750000	0.000870
510.500000	0.000888
510.250000	0.000905
510.000000	0.000920
509.750000	0.000929
509.500000	0.000933

509.250000	0.000930
509.000000	0.000921
508.750000	0.000906
508.500000	0.000889
508.250000	0.000871
508.000000	0.000858
507.750000	0.000853
507.500000	0.000859
507.250000	0.000877
507.000000	0.000904
506.750000	0.000934
506.500000	0.000960
506.250000	0.000977
506.000000	0.000984
505.750000	0.000983
505.500000	0.000976
505.250000	0.000968
505.000000	0.000961
504.750000	0.000957
504.500000	0.000954
504.250000	0.000951
504.000000	0.000946
503.750000	0.000938
503.500000	0.000929
503.250000	0.000924
503.000000	0.000929

502.750000	0.000950
502.500000	0.000989
502.250000	0.001041
502.000000	0.001097
501.750000	0.001144
501.500000	0.001174
501.250000	0.001188
501.000000	0.001188
500.750000	0.001181
500.500000	0.001172
500.250000	0.001165
500.000000	0.001161
499.750000	0.001160
499.500000	0.001160
499.250000	0.001160
499.000000	0.001160
498.750000	0.001159
498.500000	0.001158
498.250000	0.001161
498.000000	0.001169
497.750000	0.001183
497.500000	0.001204
497.250000	0.001231
497.000000	0.001262
496.750000	0.001295
496.500000	0.001330

496.250000	0.001364
496.000000	0.001395
495.750000	0.001424
495.500000	0.001449
495.250000	0.001472
495.000000	0.001491
494.750000	0.001509
494.500000	0.001525
494.250000	0.001540
494.000000	0.001555
493.750000	0.001568
493.500000	0.001579
493.250000	0.001589
493.000000	0.001598
492.750000	0.001605
492.500000	0.001612
492.250000	0.001619
492.000000	0.001627
491.750000	0.001636
491.500000	0.001647
491.250000	0.001663
491.000000	0.001684
490.750000	0.001712
490.500000	0.001746
490.250000	0.001784
490.000000	0.001824

489.750000	0.001862
489.500000	0.001895
489.250000	0.001920
489.000000	0.001937
488.750000	0.001948
488.500000	0.001956
488.250000	0.001963
488.000000	0.001973
487.750000	0.001986
487.500000	0.002002
487.250000	0.002021
487.000000	0.002040
486.750000	0.002058
486.500000	0.002074
486.250000	0.002086
486.000000	0.002094
485.750000	0.002099
485.500000	0.002100
485.250000	0.002100
485.000000	0.002101
484.750000	0.002108
484.500000	0.002124
484.250000	0.002152
484.000000	0.002193
483.750000	0.002244
483.500000	0.002301

483.250000	0.002357
483.000000	0.002407
482.750000	0.002450
482.500000	0.002483
482.250000	0.002508
482.000000	0.002526
481.750000	0.002538
481.500000	0.002544
481.250000	0.002546
481.000000	0.002545
480.750000	0.002545
480.500000	0.002548
480.250000	0.002557
480.000000	0.002574
479.750000	0.002599
479.500000	0.002630
479.250000	0.002665
479.000000	0.002700
478.750000	0.002732
478.500000	0.002758
478.250000	0.002778
478.000000	0.002791
477.750000	0.002799
477.500000	0.002805
477.250000	0.002811
477.000000	0.002821

476.750000	0.002837
476.500000	0.002861
476.250000	0.002891
476.000000	0.002925
475.750000	0.002957
475.500000	0.002982
475.250000	0.002997
475.000000	0.002999
474.750000	0.002989
474.500000	0.002969
474.250000	0.002943
474.000000	0.002914
473.750000	0.002888
473.500000	0.002870
473.250000	0.002868
473.000000	0.002891
472.750000	0.002946
472.500000	0.003028
472.250000	0.003124
472.000000	0.003217
471.750000	0.003291
471.500000	0.003339
471.250000	0.003361
471.000000	0.003362
470.750000	0.003347
470.500000	0.003323

470.250000	0.003296
470.000000	0.003271
469.750000	0.003254
469.500000	0.003248
469.250000	0.003254
469.000000	0.003269
468.750000	0.003293
468.500000	0.003323
468.250000	0.003355
468.000000	0.003388
467.750000	0.003422
467.500000	0.003457
467.250000	0.003492
467.000000	0.003530
466.750000	0.003567
466.500000	0.003602
466.250000	0.003632
466.000000	0.003652
465.750000	0.003662
465.500000	0.003661
465.250000	0.003651
465.000000	0.003637
464.750000	0.003623
464.500000	0.003611
464.250000	0.003604
464.000000	0.003600

463.750000	0.003596
463.500000	0.003588
463.250000	0.003575
463.000000	0.003556
462.750000	0.003533
462.500000	0.003509
462.250000	0.003491
462.000000	0.003483
461.750000	0.003492
461.500000	0.003518
461.250000	0.003561
461.000000	0.003612
460.750000	0.003665
460.500000	0.003710
460.250000	0.003739
460.000000	0.003748
459.750000	0.003737
459.500000	0.003709
459.250000	0.003671
459.000000	0.003629
458.750000	0.003595
458.500000	0.003582
458.250000	0.003603
458.000000	0.003663
457.750000	0.003750
457.500000	0.003833

457.250000	0.003888
457.000000	0.003907
456.750000	0.003898
456.500000	0.003876
456.250000	0.003851
456.000000	0.003825
455.750000	0.003798
455.500000	0.003767
455.250000	0.003733
455.000000	0.003696
454.750000	0.003662
454.500000	0.003636
454.250000	0.003625
454.000000	0.003632
453.750000	0.003660
453.500000	0.003703
453.250000	0.003755
453.000000	0.003804
452.750000	0.003840
452.500000	0.003859
452.250000	0.003861
452.000000	0.003848
451.750000	0.003828
451.500000	0.003803
451.250000	0.003776
451.000000	0.003750

450.750000	0.003726
450.500000	0.003704
450.250000	0.003686
450.000000	0.003671
449.750000	0.003656
449.500000	0.003639
449.250000	0.003615
449.000000	0.003582
448.750000	0.003539
448.500000	0.003490
448.250000	0.003441
448.000000	0.003397
447.750000	0.003367
447.500000	0.003355
447.250000	0.003365
447.000000	0.003396
446.750000	0.003441
446.500000	0.003492
446.250000	0.003540
446.000000	0.003581
445.750000	0.003612
445.500000	0.003635
445.250000	0.003650
445.000000	0.003660
444.750000	0.003666
444.500000	0.003667

444.250000	0.003664
444.000000	0.003655
443.750000	0.003637
443.500000	0.003612
443.250000	0.003580
443.000000	0.003545
442.750000	0.003513
442.500000	0.003489
442.250000	0.003477
442.000000	0.003476
441.750000	0.003484
441.500000	0.003495
441.250000	0.003500
441.000000	0.003497
440.750000	0.003482
440.500000	0.003457
440.250000	0.003424
440.000000	0.003386
439.750000	0.003347
439.500000	0.003310
439.250000	0.003277
439.000000	0.003250
438.750000	0.003227
438.500000	0.003210
438.250000	0.003195
438.000000	0.003181

437.750000	0.003166
437.500000	0.003149
437.250000	0.003129
437.000000	0.003110
436.750000	0.003094
436.500000	0.003088
436.250000	0.003094
436.000000	0.003116
435.750000	0.003149
435.500000	0.003188
435.250000	0.003227
435.000000	0.003261
434.750000	0.003285
434.500000	0.003297
434.250000	0.003299
434.000000	0.003290
433.750000	0.003270
433.500000	0.003241
433.250000	0.003203
433.000000	0.003159
432.750000	0.003113
432.500000	0.003068
432.250000	0.003032
432.000000	0.003007
431.750000	0.002998
431.500000	0.003002

431.250000	0.003014
431.000000	0.003028
430.750000	0.003038
430.500000	0.003040
430.250000	0.003036
430.000000	0.003028
429.750000	0.003020
429.500000	0.003016
429.250000	0.003017
429.000000	0.003022
428.750000	0.003028
428.500000	0.003032
428.250000	0.003030
428.000000	0.003018
427.750000	0.002994
427.500000	0.002956
427.250000	0.002906
427.000000	0.002847
426.750000	0.002785
426.500000	0.002726
426.250000	0.002678
426.000000	0.002647
425.750000	0.002634
425.500000	0.002634
425.250000	0.002640
425.000000	0.002641

424.750000	0.002632
424.500000	0.002611
424.250000	0.002583
424.000000	0.002557
423.750000	0.002544
423.500000	0.002554
423.250000	0.002592
423.000000	0.002651
422.750000	0.002714
422.500000	0.002764
422.250000	0.002786
422.000000	0.002776
421.750000	0.002740
421.500000	0.002687
421.250000	0.002617
421.000000	0.002532
420.750000	0.002432
420.500000	0.002316
420.250000	0.002185
420.000000	0.002044
419.750000	0.001920
419.500000	0.001867
419.250000	0.001942
419.000000	0.002163
418.750000	0.002468
418.500000	0.002744

418.250000	0.002902
418.000000	0.002922
417.750000	0.002848
417.500000	0.002726
417.250000	0.002582
417.000000	0.002428
416.750000	0.002276
416.500000	0.002137
416.250000	0.002016
416.000000	0.001919
415.750000	0.001848
415.500000	0.001806
415.250000	0.001789
415.000000	0.001792
414.750000	0.001806
414.500000	0.001824
414.250000	0.001838
414.000000	0.001846
413.750000	0.001847
413.500000	0.001839
413.250000	0.001825
413.000000	0.001806
412.750000	0.001786
412.500000	0.001767
412.250000	0.001751
412.000000	0.001741

411.750000	0.001731
411.500000	0.001717
411.250000	0.001690
411.000000	0.001646
410.750000	0.001584
410.500000	0.001510
410.250000	0.001440
410.000000	0.001388
409.750000	0.001367
409.500000	0.001379
409.250000	0.001417
409.000000	0.001462
408.750000	0.001493
408.500000	0.001490
408.250000	0.001444
408.000000	0.001364
407.750000	0.001267
407.500000	0.001182
407.250000	0.001132
407.000000	0.001135
406.750000	0.001190
406.500000	0.001285
406.250000	0.001395
406.000000	0.001499
405.750000	0.001575
405.500000	0.001615

405.250000	0.001615
405.000000	0.001580
404.750000	0.001517
404.500000	0.001434
404.250000	0.001333
404.000000	0.001217
403.750000	0.001089
403.500000	0.000954
403.250000	0.000823
403.000000	0.000709
402.750000	0.000622
402.500000	0.000570
402.250000	0.000555
402.000000	0.000572
401.750000	0.000611
401.500000	0.000662
401.250000	0.000715
401.000000	0.000763
400.750000	0.000793
400.500000	0.000796
400.250000	0.000774
400.000000	0.000738

A1_2mm_adown

PE IR SUBTECH SPECTRUM ASCII PEDS 4.00

-1

A1_2mm_a_s_1_1_1.ASC

21/07/23

16:08:45.00

21/12/06

15:42:15.00

ir

Sample 016 By ir Date venerd, luglio 23 2021

400.000000

#HDR

-1

-1

#GR

cm-1

A

1.0

0.0

4000.000000

-0.250000

14401

8

0.021676

-0.000252

#DATA

4000.000000 0.000488

3999.750000	0.000493
3999.500000	0.000498
3999.250000	0.000503
3999.000000	0.000509
3998.750000	0.000514
3998.500000	0.000518
3998.250000	0.000520
3998.000000	0.000520
3997.750000	0.000518
3997.500000	0.000514
3997.250000	0.000509
3997.000000	0.000504
3996.750000	0.000499
3996.500000	0.000495
3996.250000	0.000493
3996.000000	0.000493
3995.750000	0.000495
3995.500000	0.000499
3995.250000	0.000504
3995.000000	0.000508
3994.750000	0.000510
3994.500000	0.000511
3994.250000	0.000511
3994.000000	0.000509
3993.750000	0.000508
3993.500000	0.000508

3993.250000	0.000510
3993.000000	0.000512
3992.750000	0.000515
3992.500000	0.000517
3992.250000	0.000520
3992.000000	0.000523
3991.750000	0.000527
3991.500000	0.000534
3991.250000	0.000542
3991.000000	0.000552
3990.750000	0.000561
3990.500000	0.000568
3990.250000	0.000570
3990.000000	0.000567
3989.750000	0.000560
3989.500000	0.000548
3989.250000	0.000535
3989.000000	0.000523
3988.750000	0.000514
3988.500000	0.000510
3988.250000	0.000510
3988.000000	0.000515
3987.750000	0.000524
3987.500000	0.000534
3987.250000	0.000545
3987.000000	0.000556

3986.750000	0.000564
3986.500000	0.000570
3986.250000	0.000572
3986.000000	0.000570
3985.750000	0.000565
3985.500000	0.000556
3985.250000	0.000545
3985.000000	0.000533
3984.750000	0.000521
3984.500000	0.000511
3984.250000	0.000505
3984.000000	0.000502
3983.750000	0.000503
3983.500000	0.000507
3983.250000	0.000515
3983.000000	0.000524
3982.750000	0.000534
3982.500000	0.000543
3982.250000	0.000552
3982.000000	0.000558
3981.750000	0.000562
3981.500000	0.000564
3981.250000	0.000563
3981.000000	0.000559
3980.750000	0.000553
3980.500000	0.000547

3980.250000	0.000540
3980.000000	0.000534
3979.750000	0.000530
3979.500000	0.000528
3979.250000	0.000528
3979.000000	0.000531
3978.750000	0.000536
3978.500000	0.000543
3978.250000	0.000553
3978.000000	0.000563
3977.750000	0.000572
3977.500000	0.000579
3977.250000	0.000583
3977.000000	0.000583
3976.750000	0.000578
3976.500000	0.000570
3976.250000	0.000561
3976.000000	0.000552
3975.750000	0.000545
3975.500000	0.000542
3975.250000	0.000542
3975.000000	0.000545
3974.750000	0.000548
3974.500000	0.000551
3974.250000	0.000552
3974.000000	0.000553

3973.750000	0.000555
3973.500000	0.000558
3973.250000	0.000565
3973.000000	0.000576
3972.750000	0.000588
3972.500000	0.000602
3972.250000	0.000614
3972.000000	0.000622
3971.750000	0.000626
3971.500000	0.000625
3971.250000	0.000621
3971.000000	0.000615
3970.750000	0.000610
3970.500000	0.000606
3970.250000	0.000606
3970.000000	0.000608
3969.750000	0.000613
3969.500000	0.000618
3969.250000	0.000624
3969.000000	0.000628
3968.750000	0.000629
3968.500000	0.000627
3968.250000	0.000622
3968.000000	0.000615
3967.750000	0.000606
3967.500000	0.000595

3967.250000	0.000584
3967.000000	0.000574
3966.750000	0.000565
3966.500000	0.000558
3966.250000	0.000554
3966.000000	0.000554
3965.750000	0.000557
3965.500000	0.000564
3965.250000	0.000575
3965.000000	0.000589
3964.750000	0.000605
3964.500000	0.000620
3964.250000	0.000634
3964.000000	0.000644
3963.750000	0.000650
3963.500000	0.000651
3963.250000	0.000648
3963.000000	0.000644
3962.750000	0.000638
3962.500000	0.000633
3962.250000	0.000630
3962.000000	0.000628
3961.750000	0.000626
3961.500000	0.000625
3961.250000	0.000623
3961.000000	0.000621

3960.750000	0.000618
3960.500000	0.000614
3960.250000	0.000611
3960.000000	0.000608
3959.750000	0.000607
3959.500000	0.000607
3959.250000	0.000607
3959.000000	0.000608
3958.750000	0.000609
3958.500000	0.000610
3958.250000	0.000610
3958.000000	0.000611
3957.750000	0.000612
3957.500000	0.000614
3957.250000	0.000617
3957.000000	0.000621
3956.750000	0.000624
3956.500000	0.000625
3956.250000	0.000621
3956.000000	0.000612
3955.750000	0.000599
3955.500000	0.000583
3955.250000	0.000566
3955.000000	0.000552
3954.750000	0.000545
3954.500000	0.000545

3954.250000	0.000553
3954.000000	0.000566
3953.750000	0.000581
3953.500000	0.000593
3953.250000	0.000600
3953.000000	0.000599
3952.750000	0.000592
3952.500000	0.000579
3952.250000	0.000565
3952.000000	0.000555
3951.750000	0.000551
3951.500000	0.000556
3951.250000	0.000570
3951.000000	0.000589
3950.750000	0.000611
3950.500000	0.000631
3950.250000	0.000645
3950.000000	0.000651
3949.750000	0.000649
3949.500000	0.000642
3949.250000	0.000634
3949.000000	0.000629
3948.750000	0.000631
3948.500000	0.000639
3948.250000	0.000654
3948.000000	0.000671

3947.750000	0.000687
3947.500000	0.000697
3947.250000	0.000699
3947.000000	0.000695
3946.750000	0.000686
3946.500000	0.000677
3946.250000	0.000670
3946.000000	0.000669
3945.750000	0.000671
3945.500000	0.000676
3945.250000	0.000680
3945.000000	0.000680
3944.750000	0.000673
3944.500000	0.000659
3944.250000	0.000640
3944.000000	0.000620
3943.750000	0.000602
3943.500000	0.000591
3943.250000	0.000588
3943.000000	0.000591
3942.750000	0.000598
3942.500000	0.000604
3942.250000	0.000609
3942.000000	0.000612
3941.750000	0.000617
3941.500000	0.000625

3941.250000	0.000638
3941.000000	0.000657
3940.750000	0.000680
3940.500000	0.000703
3940.250000	0.000725
3940.000000	0.000740
3939.750000	0.000748
3939.500000	0.000747
3939.250000	0.000739
3939.000000	0.000726
3938.750000	0.000711
3938.500000	0.000698
3938.250000	0.000687
3938.000000	0.000681
3937.750000	0.000679
3937.500000	0.000681
3937.250000	0.000683
3937.000000	0.000685
3936.750000	0.000686
3936.500000	0.000684
3936.250000	0.000680
3936.000000	0.000675
3935.750000	0.000669
3935.500000	0.000663
3935.250000	0.000659
3935.000000	0.000654

3934.750000	0.000650
3934.500000	0.000646
3934.250000	0.000641
3934.000000	0.000637
3933.750000	0.000636
3933.500000	0.000638
3933.250000	0.000644
3933.000000	0.000653
3932.750000	0.000664
3932.500000	0.000673
3932.250000	0.000677
3932.000000	0.000675
3931.750000	0.000668
3931.500000	0.000660
3931.250000	0.000654
3931.000000	0.000653
3930.750000	0.000659
3930.500000	0.000671
3930.250000	0.000687
3930.000000	0.000704
3929.750000	0.000719
3929.500000	0.000729
3929.250000	0.000735
3929.000000	0.000735
3928.750000	0.000733
3928.500000	0.000729

3928.250000	0.000726
3928.000000	0.000723
3927.750000	0.000720
3927.500000	0.000715
3927.250000	0.000708
3927.000000	0.000698
3926.750000	0.000685
3926.500000	0.000671
3926.250000	0.000656
3926.000000	0.000645
3925.750000	0.000640
3925.500000	0.000642
3925.250000	0.000651
3925.000000	0.000665
3924.750000	0.000681
3924.500000	0.000697
3924.250000	0.000711
3924.000000	0.000721
3923.750000	0.000728
3923.500000	0.000733
3923.250000	0.000734
3923.000000	0.000732
3922.750000	0.000728
3922.500000	0.000721
3922.250000	0.000712
3922.000000	0.000703

3921.750000	0.000696
3921.500000	0.000693
3921.250000	0.000696
3921.000000	0.000706
3920.750000	0.000719
3920.500000	0.000730
3920.250000	0.000732
3920.000000	0.000722
3919.750000	0.000697
3919.500000	0.000662
3919.250000	0.000621
3919.000000	0.000584
3918.750000	0.000557
3918.500000	0.000547
3918.250000	0.000556
3918.000000	0.000584
3917.750000	0.000626
3917.500000	0.000673
3917.250000	0.000714
3917.000000	0.000739
3916.750000	0.000745
3916.500000	0.000735
3916.250000	0.000716
3916.000000	0.000697
3915.750000	0.000686
3915.500000	0.000686

3915.250000	0.000698
3915.000000	0.000719
3914.750000	0.000746
3914.500000	0.000774
3914.250000	0.000797
3914.000000	0.000814
3913.750000	0.000823
3913.500000	0.000824
3913.250000	0.000818
3913.000000	0.000807
3912.750000	0.000794
3912.500000	0.000780
3912.250000	0.000767
3912.000000	0.000756
3911.750000	0.000748
3911.500000	0.000743
3911.250000	0.000741
3911.000000	0.000740
3910.750000	0.000741
3910.500000	0.000741
3910.250000	0.000741
3910.000000	0.000739
3909.750000	0.000735
3909.500000	0.000730
3909.250000	0.000724
3909.000000	0.000718

3908.750000	0.000711
3908.500000	0.000704
3908.250000	0.000699
3908.000000	0.000695
3907.750000	0.000692
3907.500000	0.000689
3907.250000	0.000684
3907.000000	0.000673
3906.750000	0.000655
3906.500000	0.000627
3906.250000	0.000590
3906.000000	0.000547
3905.750000	0.000507
3905.500000	0.000476
3905.250000	0.000462
3905.000000	0.000466
3904.750000	0.000481
3904.500000	0.000499
3904.250000	0.000511
3904.000000	0.000510
3903.750000	0.000500
3903.500000	0.000490
3903.250000	0.000489
3903.000000	0.000507
3902.750000	0.000547
3902.500000	0.000601

3902.250000	0.000658
3902.000000	0.000702
3901.750000	0.000719
3901.500000	0.000707
3901.250000	0.000672
3901.000000	0.000627
3900.750000	0.000588
3900.500000	0.000569
3900.250000	0.000577
3900.000000	0.000613
3899.750000	0.000670
3899.500000	0.000728
3899.250000	0.000770
3899.000000	0.000783
3898.750000	0.000768
3898.500000	0.000734
3898.250000	0.000697
3898.000000	0.000671
3897.750000	0.000665
3897.500000	0.000678
3897.250000	0.000706
3897.000000	0.000739
3896.750000	0.000772
3896.500000	0.000796
3896.250000	0.000810
3896.000000	0.000814

3895.750000	0.000813
3895.500000	0.000809
3895.250000	0.000808
3895.000000	0.000810
3894.750000	0.000813
3894.500000	0.000814
3894.250000	0.000808
3894.000000	0.000790
3893.750000	0.000759
3893.500000	0.000716
3893.250000	0.000664
3893.000000	0.000610
3892.750000	0.000562
3892.500000	0.000529
3892.250000	0.000517
3892.000000	0.000529
3891.750000	0.000555
3891.500000	0.000584
3891.250000	0.000602
3891.000000	0.000603
3890.750000	0.000591
3890.500000	0.000579
3890.250000	0.000578
3890.000000	0.000597
3889.750000	0.000635
3889.500000	0.000687

3889.250000	0.000742
3889.000000	0.000790
3888.750000	0.000819
3888.500000	0.000823
3888.250000	0.000799
3888.000000	0.000747
3887.750000	0.000676
3887.500000	0.000594
3887.250000	0.000516
3887.000000	0.000458
3886.750000	0.000433
3886.500000	0.000449
3886.250000	0.000497
3886.000000	0.000558
3885.750000	0.000606
3885.500000	0.000626
3885.250000	0.000618
3885.000000	0.000595
3884.750000	0.000573
3884.500000	0.000568
3884.250000	0.000583
3884.000000	0.000617
3883.750000	0.000662
3883.500000	0.000708
3883.250000	0.000744
3883.000000	0.000764

3882.750000	0.000763
3882.500000	0.000744
3882.250000	0.000711
3882.000000	0.000675
3881.750000	0.000644
3881.500000	0.000630
3881.250000	0.000638
3881.000000	0.000667
3880.750000	0.000704
3880.500000	0.000732
3880.250000	0.000736
3880.000000	0.000711
3879.750000	0.000668
3879.500000	0.000621
3879.250000	0.000587
3879.000000	0.000576
3878.750000	0.000590
3878.500000	0.000627
3878.250000	0.000678
3878.000000	0.000735
3877.750000	0.000789
3877.500000	0.000833
3877.250000	0.000862
3877.000000	0.000874
3876.750000	0.000871
3876.500000	0.000855

3876.250000	0.000831
3876.000000	0.000804
3875.750000	0.000777
3875.500000	0.000756
3875.250000	0.000747
3875.000000	0.000752
3874.750000	0.000766
3874.500000	0.000781
3874.250000	0.000782
3874.000000	0.000762
3873.750000	0.000722
3873.500000	0.000670
3873.250000	0.000621
3873.000000	0.000585
3872.750000	0.000568
3872.500000	0.000567
3872.250000	0.000576
3872.000000	0.000586
3871.750000	0.000589
3871.500000	0.000583
3871.250000	0.000569
3871.000000	0.000560
3870.750000	0.000568
3870.500000	0.000602
3870.250000	0.000657
3870.000000	0.000710

3869.750000	0.000737
3869.500000	0.000726
3869.250000	0.000685
3869.000000	0.000635
3868.750000	0.000600
3868.500000	0.000594
3868.250000	0.000619
3868.000000	0.000667
3867.750000	0.000725
3867.500000	0.000779
3867.250000	0.000815
3867.000000	0.000826
3866.750000	0.000808
3866.500000	0.000768
3866.250000	0.000718
3866.000000	0.000673
3865.750000	0.000648
3865.500000	0.000646
3865.250000	0.000660
3865.000000	0.000674
3864.750000	0.000675
3864.500000	0.000658
3864.250000	0.000631
3864.000000	0.000605
3863.750000	0.000592
3863.500000	0.000597

3863.250000	0.000623
3863.000000	0.000669
3862.750000	0.000730
3862.500000	0.000799
3862.250000	0.000865
3862.000000	0.000916
3861.750000	0.000943
3861.500000	0.000944
3861.250000	0.000926
3861.000000	0.000898
3860.750000	0.000872
3860.500000	0.000855
3860.250000	0.000849
3860.000000	0.000853
3859.750000	0.000864
3859.500000	0.000874
3859.250000	0.000881
3859.000000	0.000883
3858.750000	0.000880
3858.500000	0.000874
3858.250000	0.000870
3858.000000	0.000870
3857.750000	0.000869
3857.500000	0.000864
3857.250000	0.000845
3857.000000	0.000804

3856.750000	0.000739
3856.500000	0.000647
3856.250000	0.000530
3856.000000	0.000392
3855.750000	0.000238
3855.500000	0.000076
3855.250000	-0.000075
3855.000000	-0.000191
3854.750000	-0.000252
3854.500000	-0.000250
3854.250000	-0.000206
3854.000000	-0.000154
3853.750000	-0.000122
3853.500000	-0.000111
3853.250000	-0.000099
3853.000000	-0.000059
3852.750000	0.000024
3852.500000	0.000141
3852.250000	0.000275
3852.000000	0.000405
3851.750000	0.000519
3851.500000	0.000611
3851.250000	0.000684
3851.000000	0.000745
3850.750000	0.000801
3850.500000	0.000856

3850.250000	0.000911
3850.000000	0.000964
3849.750000	0.001011
3849.500000	0.001046
3849.250000	0.001067
3849.000000	0.001072
3848.750000	0.001064
3848.500000	0.001047
3848.250000	0.001026
3848.000000	0.001005
3847.750000	0.000985
3847.500000	0.000967
3847.250000	0.000950
3847.000000	0.000931
3846.750000	0.000910
3846.500000	0.000885
3846.250000	0.000858
3846.000000	0.000829
3845.750000	0.000800
3845.500000	0.000776
3845.250000	0.000760
3845.000000	0.000756
3844.750000	0.000767
3844.500000	0.000791
3844.250000	0.000819
3844.000000	0.000841

3843.750000	0.000847
3843.500000	0.000831
3843.250000	0.000797
3843.000000	0.000751
3842.750000	0.000706
3842.500000	0.000670
3842.250000	0.000650
3842.000000	0.000650
3841.750000	0.000670
3841.500000	0.000705
3841.250000	0.000747
3841.000000	0.000784
3840.750000	0.000802
3840.500000	0.000793
3840.250000	0.000758
3840.000000	0.000702
3839.750000	0.000637
3839.500000	0.000575
3839.250000	0.000529
3839.000000	0.000513
3838.750000	0.000532
3838.500000	0.000584
3838.250000	0.000648
3838.000000	0.000693
3837.750000	0.000696
3837.500000	0.000653

3837.250000	0.000581
3837.000000	0.000507
3836.750000	0.000458
3836.500000	0.000447
3836.250000	0.000482
3836.000000	0.000557
3835.750000	0.000660
3835.500000	0.000772
3835.250000	0.000869
3835.000000	0.000930
3834.750000	0.000945
3834.500000	0.000920
3834.250000	0.000870
3834.000000	0.000812
3833.750000	0.000764
3833.500000	0.000734
3833.250000	0.000725
3833.000000	0.000739
3832.750000	0.000772
3832.500000	0.000820
3832.250000	0.000873
3832.000000	0.000919
3831.750000	0.000942
3831.500000	0.000936
3831.250000	0.000902
3831.000000	0.000852

3830.750000	0.000799
3830.500000	0.000758
3830.250000	0.000736
3830.000000	0.000736
3829.750000	0.000754
3829.500000	0.000785
3829.250000	0.000823
3829.000000	0.000861
3828.750000	0.000895
3828.500000	0.000924
3828.250000	0.000947
3828.000000	0.000966
3827.750000	0.000984
3827.500000	0.000997
3827.250000	0.001005
3827.000000	0.001003
3826.750000	0.000989
3826.500000	0.000963
3826.250000	0.000932
3826.000000	0.000901
3825.750000	0.000877
3825.500000	0.000866
3825.250000	0.000868
3825.000000	0.000879
3824.750000	0.000894
3824.500000	0.000903

3824.250000	0.000902
3824.000000	0.000884
3823.750000	0.000848
3823.500000	0.000792
3823.250000	0.000720
3823.000000	0.000637
3822.750000	0.000548
3822.500000	0.000467
3822.250000	0.000407
3822.000000	0.000386
3821.750000	0.000412
3821.500000	0.000474
3821.250000	0.000550
3821.000000	0.000609
3820.750000	0.000638
3820.500000	0.000639
3820.250000	0.000631
3820.000000	0.000632
3819.750000	0.000653
3819.500000	0.000693
3819.250000	0.000746
3819.000000	0.000800
3818.750000	0.000845
3818.500000	0.000870
3818.250000	0.000871
3818.000000	0.000844

3817.750000	0.000795
3817.500000	0.000735
3817.250000	0.000680
3817.000000	0.000652
3816.750000	0.000664
3816.500000	0.000710
3816.250000	0.000767
3816.000000	0.000805
3815.750000	0.000805
3815.500000	0.000774
3815.250000	0.000733
3815.000000	0.000708
3814.750000	0.000713
3814.500000	0.000750
3814.250000	0.000812
3814.000000	0.000888
3813.750000	0.000965
3813.500000	0.001033
3813.250000	0.001085
3813.000000	0.001116
3812.750000	0.001128
3812.500000	0.001122
3812.250000	0.001106
3812.000000	0.001085
3811.750000	0.001063
3811.500000	0.001043

3811.250000	0.001028
3811.000000	0.001017
3810.750000	0.001012
3810.500000	0.001009
3810.250000	0.001005
3810.000000	0.000996
3809.750000	0.000977
3809.500000	0.000944
3809.250000	0.000896
3809.000000	0.000835
3808.750000	0.000767
3808.500000	0.000700
3808.250000	0.000647
3808.000000	0.000621
3807.750000	0.000632
3807.500000	0.000676
3807.250000	0.000734
3807.000000	0.000783
3806.750000	0.000804
3806.500000	0.000795
3806.250000	0.000770
3806.000000	0.000749
3805.750000	0.000745
3805.500000	0.000764
3805.250000	0.000803
3805.000000	0.000851

3804.750000	0.000899
3804.500000	0.000937
3804.250000	0.000957
3804.000000	0.000952
3803.750000	0.000922
3803.500000	0.000867
3803.250000	0.000791
3803.000000	0.000705
3802.750000	0.000622
3802.500000	0.000560
3802.250000	0.000539
3802.000000	0.000567
3801.750000	0.000633
3801.500000	0.000708
3801.250000	0.000761
3801.000000	0.000778
3800.750000	0.000765
3800.500000	0.000744
3800.250000	0.000734
3800.000000	0.000745
3799.750000	0.000777
3799.500000	0.000824
3799.250000	0.000877
3799.000000	0.000927
3798.750000	0.000965
3798.500000	0.000986

3798.250000	0.000990
3798.000000	0.000983
3797.750000	0.000970
3797.500000	0.000962
3797.250000	0.000964
3797.000000	0.000977
3796.750000	0.000995
3796.500000	0.001008
3796.250000	0.001012
3796.000000	0.001003
3795.750000	0.000987
3795.500000	0.000971
3795.250000	0.000960
3795.000000	0.000958
3794.750000	0.000968
3794.500000	0.000986
3794.250000	0.001011
3794.000000	0.001037
3793.750000	0.001062
3793.500000	0.001080
3793.250000	0.001090
3793.000000	0.001092
3792.750000	0.001087
3792.500000	0.001078
3792.250000	0.001067
3792.000000	0.001057

3791.750000	0.001048
3791.500000	0.001040
3791.250000	0.001032
3791.000000	0.001023
3790.750000	0.001012
3790.500000	0.001001
3790.250000	0.000991
3790.000000	0.000983
3789.750000	0.000980
3789.500000	0.000981
3789.250000	0.000986
3789.000000	0.000993
3788.750000	0.001001
3788.500000	0.001005
3788.250000	0.001006
3788.000000	0.001002
3787.750000	0.000994
3787.500000	0.000983
3787.250000	0.000972
3787.000000	0.000964
3786.750000	0.000961
3786.500000	0.000964
3786.250000	0.000974
3786.000000	0.000992
3785.750000	0.001017
3785.500000	0.001045

3785.250000	0.001074
3785.000000	0.001099
3784.750000	0.001115
3784.500000	0.001121
3784.250000	0.001117
3784.000000	0.001103
3783.750000	0.001084
3783.500000	0.001064
3783.250000	0.001046
3783.000000	0.001033
3782.750000	0.001023
3782.500000	0.001017
3782.250000	0.001012
3782.000000	0.001005
3781.750000	0.000995
3781.500000	0.000983
3781.250000	0.000969
3781.000000	0.000956
3780.750000	0.000948
3780.500000	0.000949
3780.250000	0.000957
3780.000000	0.000971
3779.750000	0.000984
3779.500000	0.000988
3779.250000	0.000982
3779.000000	0.000967

3778.750000	0.000951
3778.500000	0.000943
3778.250000	0.000950
3778.000000	0.000974
3777.750000	0.001010
3777.500000	0.001052
3777.250000	0.001092
3777.000000	0.001123
3776.750000	0.001140
3776.500000	0.001144
3776.250000	0.001135
3776.000000	0.001120
3775.750000	0.001103
3775.500000	0.001088
3775.250000	0.001079
3775.000000	0.001075
3774.750000	0.001077
3774.500000	0.001083
3774.250000	0.001090
3774.000000	0.001098
3773.750000	0.001105
3773.500000	0.001110
3773.250000	0.001110
3773.000000	0.001103
3772.750000	0.001088
3772.500000	0.001064

3772.250000	0.001029
3772.000000	0.000987
3771.750000	0.000940
3771.500000	0.000894
3771.250000	0.000857
3771.000000	0.000838
3770.750000	0.000842
3770.500000	0.000869
3770.250000	0.000910
3770.000000	0.000949
3769.750000	0.000972
3769.500000	0.000973
3769.250000	0.000956
3769.000000	0.000933
3768.750000	0.000916
3768.500000	0.000911
3768.250000	0.000922
3768.000000	0.000944
3767.750000	0.000974
3767.500000	0.001008
3767.250000	0.001043
3767.000000	0.001078
3766.750000	0.001112
3766.500000	0.001145
3766.250000	0.001174
3766.000000	0.001191

3765.750000	0.001193
3765.500000	0.001177
3765.250000	0.001146
3765.000000	0.001109
3764.750000	0.001075
3764.500000	0.001054
3764.250000	0.001048
3764.000000	0.001060
3763.750000	0.001083
3763.500000	0.001111
3763.250000	0.001136
3763.000000	0.001152
3762.750000	0.001154
3762.500000	0.001141
3762.250000	0.001113
3762.000000	0.001074
3761.750000	0.001029
3761.500000	0.000981
3761.250000	0.000939
3761.000000	0.000909
3760.750000	0.000899
3760.500000	0.000913
3760.250000	0.000948
3760.000000	0.000991
3759.750000	0.001025
3759.500000	0.001037

3759.250000	0.001024
3759.000000	0.000993
3758.750000	0.000959
3758.500000	0.000935
3758.250000	0.000934
3758.000000	0.000960
3757.750000	0.001015
3757.500000	0.001091
3757.250000	0.001175
3757.000000	0.001250
3756.750000	0.001302
3756.500000	0.001321
3756.250000	0.001308
3756.000000	0.001270
3755.750000	0.001219
3755.500000	0.001166
3755.250000	0.001116
3755.000000	0.001073
3754.750000	0.001032
3754.500000	0.000987
3754.250000	0.000933
3754.000000	0.000867
3753.750000	0.000791
3753.500000	0.000712
3753.250000	0.000647
3753.000000	0.000614

3752.750000	0.000618
3752.500000	0.000650
3752.250000	0.000684
3752.000000	0.000694
3751.750000	0.000671
3751.500000	0.000625
3751.250000	0.000577
3751.000000	0.000543
3750.750000	0.000536
3750.500000	0.000566
3750.250000	0.000640
3750.000000	0.000758
3749.750000	0.000895
3749.500000	0.001014
3749.250000	0.001085
3749.000000	0.001100
3748.750000	0.001079
3748.500000	0.001051
3748.250000	0.001039
3748.000000	0.001047
3747.750000	0.001070
3747.500000	0.001091
3747.250000	0.001096
3747.000000	0.001070
3746.750000	0.001007
3746.500000	0.000904

3746.250000	0.000763
3746.000000	0.000595
3745.750000	0.000430
3745.500000	0.000315
3745.250000	0.000298
3745.000000	0.000390
3744.750000	0.000537
3744.500000	0.000644
3744.250000	0.000645
3744.000000	0.000551
3743.750000	0.000433
3743.500000	0.000365
3743.250000	0.000379
3743.000000	0.000468
3742.750000	0.000610
3742.500000	0.000778
3742.250000	0.000951
3742.000000	0.001111
3741.750000	0.001242
3741.500000	0.001331
3741.250000	0.001371
3741.000000	0.001368
3740.750000	0.001333
3740.500000	0.001280
3740.250000	0.001219
3740.000000	0.001161

3739.750000	0.001109
3739.500000	0.001066
3739.250000	0.001032
3739.000000	0.001004
3738.750000	0.000980
3738.500000	0.000958
3738.250000	0.000934
3738.000000	0.000911
3737.750000	0.000887
3737.500000	0.000864
3737.250000	0.000844
3737.000000	0.000826
3736.750000	0.000810
3736.500000	0.000798
3736.250000	0.000798
3736.000000	0.000815
3735.750000	0.000854
3735.500000	0.000904
3735.250000	0.000950
3735.000000	0.000973
3734.750000	0.000965
3734.500000	0.000930
3734.250000	0.000881
3734.000000	0.000831
3733.750000	0.000795
3733.500000	0.000782

3733.250000	0.000802
3733.000000	0.000857
3732.750000	0.000939
3732.500000	0.001028
3732.250000	0.001099
3732.000000	0.001135
3731.750000	0.001131
3731.500000	0.001100
3731.250000	0.001062
3731.000000	0.001035
3730.750000	0.001030
3730.500000	0.001047
3730.250000	0.001077
3730.000000	0.001111
3729.750000	0.001138
3729.500000	0.001150
3729.250000	0.001143
3729.000000	0.001120
3728.750000	0.001083
3728.500000	0.001042
3728.250000	0.001003
3728.000000	0.000971
3727.750000	0.000952
3727.500000	0.000943
3727.250000	0.000944
3727.000000	0.000949

3726.750000	0.000954
3726.500000	0.000956
3726.250000	0.000957
3726.000000	0.000957
3725.750000	0.000958
3725.500000	0.000963
3725.250000	0.000972
3725.000000	0.000982
3724.750000	0.000993
3724.500000	0.001001
3724.250000	0.001005
3724.000000	0.001005
3723.750000	0.001004
3723.500000	0.001006
3723.250000	0.001015
3723.000000	0.001034
3722.750000	0.001061
3722.500000	0.001087
3722.250000	0.001104
3722.000000	0.001103
3721.750000	0.001086
3721.500000	0.001059
3721.250000	0.001028
3721.000000	0.001003
3720.750000	0.000989
3720.500000	0.000987

3720.250000	0.000999
3720.000000	0.001022
3719.750000	0.001054
3719.500000	0.001089
3719.250000	0.001120
3719.000000	0.001143
3718.750000	0.001155
3718.500000	0.001159
3718.250000	0.001156
3718.000000	0.001151
3717.750000	0.001147
3717.500000	0.001144
3717.250000	0.001143
3717.000000	0.001142
3716.750000	0.001140
3716.500000	0.001135
3716.250000	0.001128
3716.000000	0.001120
3715.750000	0.001110
3715.500000	0.001098
3715.250000	0.001081
3715.000000	0.001057
3714.750000	0.001022
3714.500000	0.000975
3714.250000	0.000915
3714.000000	0.000845

3713.750000	0.000772
3713.500000	0.000706
3713.250000	0.000663
3713.000000	0.000656
3712.750000	0.000688
3712.500000	0.000746
3712.250000	0.000801
3712.000000	0.000828
3711.750000	0.000817
3711.500000	0.000777
3711.250000	0.000732
3711.000000	0.000704
3710.750000	0.000710
3710.500000	0.000755
3710.250000	0.000835
3710.000000	0.000937
3709.750000	0.001039
3709.500000	0.001120
3709.250000	0.001163
3709.000000	0.001166
3708.750000	0.001142
3708.500000	0.001107
3708.250000	0.001076
3708.000000	0.001061
3707.750000	0.001063
3707.500000	0.001080

3707.250000	0.001107
3707.000000	0.001138
3706.750000	0.001168
3706.500000	0.001195
3706.250000	0.001217
3706.000000	0.001231
3705.750000	0.001235
3705.500000	0.001228
3705.250000	0.001209
3705.000000	0.001179
3704.750000	0.001141
3704.500000	0.001097
3704.250000	0.001050
3704.000000	0.001005
3703.750000	0.000962
3703.500000	0.000926
3703.250000	0.000899
3703.000000	0.000888
3702.750000	0.000896
3702.500000	0.000923
3702.250000	0.000965
3702.000000	0.001009
3701.750000	0.001042
3701.500000	0.001057
3701.250000	0.001055
3701.000000	0.001044

3700.750000	0.001036
3700.500000	0.001040
3700.250000	0.001060
3700.000000	0.001095
3699.750000	0.001137
3699.500000	0.001178
3699.250000	0.001209
3699.000000	0.001226
3698.750000	0.001226
3698.500000	0.001214
3698.250000	0.001197
3698.000000	0.001181
3697.750000	0.001171
3697.500000	0.001170
3697.250000	0.001176
3697.000000	0.001184
3696.750000	0.001190
3696.500000	0.001189
3696.250000	0.001180
3696.000000	0.001163
3695.750000	0.001141
3695.500000	0.001118
3695.250000	0.001099
3695.000000	0.001087
3694.750000	0.001083
3694.500000	0.001087

3694.250000	0.001094
3694.000000	0.001099
3693.750000	0.001098
3693.500000	0.001086
3693.250000	0.001063
3693.000000	0.001029
3692.750000	0.000989
3692.500000	0.000949
3692.250000	0.000917
3692.000000	0.000898
3691.750000	0.000894
3691.500000	0.000896
3691.250000	0.000890
3691.000000	0.000864
3690.750000	0.000815
3690.500000	0.000746
3690.250000	0.000670
3690.000000	0.000600
3689.750000	0.000556
3689.500000	0.000556
3689.250000	0.000614
3689.000000	0.000730
3688.750000	0.000875
3688.500000	0.001007
3688.250000	0.001089
3688.000000	0.001112

3687.750000	0.001094
3687.500000	0.001067
3687.250000	0.001054
3687.000000	0.001067
3686.750000	0.001106
3686.500000	0.001160
3686.250000	0.001218
3686.000000	0.001270
3685.750000	0.001307
3685.500000	0.001326
3685.250000	0.001329
3685.000000	0.001320
3684.750000	0.001305
3684.500000	0.001289
3684.250000	0.001276
3684.000000	0.001270
3683.750000	0.001270
3683.500000	0.001274
3683.250000	0.001282
3683.000000	0.001289
3682.750000	0.001294
3682.500000	0.001295
3682.250000	0.001289
3682.000000	0.001279
3681.750000	0.001265
3681.500000	0.001250

3681.250000	0.001235
3681.000000	0.001220
3680.750000	0.001206
3680.500000	0.001194
3680.250000	0.001186
3680.000000	0.001182
3679.750000	0.001182
3679.500000	0.001184
3679.250000	0.001183
3679.000000	0.001177
3678.750000	0.001163
3678.500000	0.001139
3678.250000	0.001104
3678.000000	0.001057
3677.750000	0.000997
3677.500000	0.000924
3677.250000	0.000839
3677.000000	0.000747
3676.750000	0.000659
3676.500000	0.000596
3676.250000	0.000584
3676.000000	0.000645
3675.750000	0.000766
3675.500000	0.000905
3675.250000	0.001011
3675.000000	0.001057

3674.750000	0.001057
3674.500000	0.001042
3674.250000	0.001041
3674.000000	0.001065
3673.750000	0.001115
3673.500000	0.001178
3673.250000	0.001243
3673.000000	0.001296
3672.750000	0.001325
3672.500000	0.001323
3672.250000	0.001293
3672.000000	0.001241
3671.750000	0.001180
3671.500000	0.001125
3671.250000	0.001083
3671.000000	0.001052
3670.750000	0.001025
3670.500000	0.000995
3670.250000	0.000965
3670.000000	0.000946
3669.750000	0.000950
3669.500000	0.000986
3669.250000	0.001050
3669.000000	0.001133
3668.750000	0.001219
3668.500000	0.001295

3668.250000	0.001352
3668.000000	0.001391
3667.750000	0.001414
3667.500000	0.001428
3667.250000	0.001440
3667.000000	0.001451
3666.750000	0.001462
3666.500000	0.001474
3666.250000	0.001483
3666.000000	0.001490
3665.750000	0.001493
3665.500000	0.001496
3665.250000	0.001499
3665.000000	0.001505
3664.750000	0.001514
3664.500000	0.001526
3664.250000	0.001537
3664.000000	0.001546
3663.750000	0.001549
3663.500000	0.001546
3663.250000	0.001538
3663.000000	0.001525
3662.750000	0.001511
3662.500000	0.001497
3662.250000	0.001486
3662.000000	0.001480

3661.750000	0.001477
3661.500000	0.001477
3661.250000	0.001480
3661.000000	0.001482
3660.750000	0.001482
3660.500000	0.001480
3660.250000	0.001476
3660.000000	0.001469
3659.750000	0.001461
3659.500000	0.001452
3659.250000	0.001442
3659.000000	0.001430
3658.750000	0.001414
3658.500000	0.001394
3658.250000	0.001369
3658.000000	0.001342
3657.750000	0.001316
3657.500000	0.001300
3657.250000	0.001301
3657.000000	0.001325
3656.750000	0.001369
3656.500000	0.001421
3656.250000	0.001464
3656.000000	0.001489
3655.750000	0.001494
3655.500000	0.001490

3655.250000	0.001488
3655.000000	0.001499
3654.750000	0.001522
3654.500000	0.001552
3654.250000	0.001582
3654.000000	0.001603
3653.750000	0.001607
3653.500000	0.001592
3653.250000	0.001559
3653.000000	0.001512
3652.750000	0.001457
3652.500000	0.001401
3652.250000	0.001350
3652.000000	0.001307
3651.750000	0.001271
3651.500000	0.001241
3651.250000	0.001214
3651.000000	0.001191
3650.750000	0.001174
3650.500000	0.001164
3650.250000	0.001165
3650.000000	0.001177
3649.750000	0.001198
3649.500000	0.001224
3649.250000	0.001253
3649.000000	0.001285

3648.750000	0.001321
3648.500000	0.001364
3648.250000	0.001416
3648.000000	0.001480
3647.750000	0.001551
3647.500000	0.001619
3647.250000	0.001674
3647.000000	0.001705
3646.750000	0.001714
3646.500000	0.001706
3646.250000	0.001693
3646.000000	0.001687
3645.750000	0.001693
3645.500000	0.001711
3645.250000	0.001739
3645.000000	0.001771
3644.750000	0.001800
3644.500000	0.001823
3644.250000	0.001839
3644.000000	0.001846
3643.750000	0.001849
3643.500000	0.001850
3643.250000	0.001851
3643.000000	0.001853
3642.750000	0.001857
3642.500000	0.001861

3642.250000	0.001864
3642.000000	0.001864
3641.750000	0.001862
3641.500000	0.001857
3641.250000	0.001852
3641.000000	0.001846
3640.750000	0.001840
3640.500000	0.001834
3640.250000	0.001827
3640.000000	0.001820
3639.750000	0.001812
3639.500000	0.001806
3639.250000	0.001803
3639.000000	0.001805
3638.750000	0.001814
3638.500000	0.001829
3638.250000	0.001849
3638.000000	0.001869
3637.750000	0.001886
3637.500000	0.001897
3637.250000	0.001901
3637.000000	0.001896
3636.750000	0.001886
3636.500000	0.001871
3636.250000	0.001855
3636.000000	0.001839

3635.750000	0.001827
3635.500000	0.001820
3635.250000	0.001820
3635.000000	0.001827
3634.750000	0.001843
3634.500000	0.001866
3634.250000	0.001891
3634.000000	0.001915
3633.750000	0.001932
3633.500000	0.001942
3633.250000	0.001944
3633.000000	0.001942
3632.750000	0.001937
3632.500000	0.001931
3632.250000	0.001920
3632.000000	0.001902
3631.750000	0.001870
3631.500000	0.001820
3631.250000	0.001749
3631.000000	0.001660
3630.750000	0.001557
3630.500000	0.001451
3630.250000	0.001360
3630.000000	0.001304
3629.750000	0.001300
3629.500000	0.001348

3629.250000	0.001429
3629.000000	0.001510
3628.750000	0.001566
3628.500000	0.001590
3628.250000	0.001601
3628.000000	0.001622
3627.750000	0.001669
3627.500000	0.001747
3627.250000	0.001849
3627.000000	0.001962
3626.750000	0.002072
3626.500000	0.002165
3626.250000	0.002234
3626.000000	0.002274
3625.750000	0.002289
3625.500000	0.002284
3625.250000	0.002266
3625.000000	0.002241
3624.750000	0.002215
3624.500000	0.002190
3624.250000	0.002167
3624.000000	0.002147
3623.750000	0.002128
3623.500000	0.002111
3623.250000	0.002095
3623.000000	0.002079

3622.750000	0.002064
3622.500000	0.002047
3622.250000	0.002029
3622.000000	0.002008
3621.750000	0.001986
3621.500000	0.001961
3621.250000	0.001937
3621.000000	0.001917
3620.750000	0.001903
3620.500000	0.001897
3620.250000	0.001900
3620.000000	0.001909
3619.750000	0.001920
3619.500000	0.001930
3619.250000	0.001943
3619.000000	0.001961
3618.750000	0.001990
3618.500000	0.002032
3618.250000	0.002085
3618.000000	0.002141
3617.750000	0.002189
3617.500000	0.002223
3617.250000	0.002240
3617.000000	0.002243
3616.750000	0.002238
3616.500000	0.002236

3616.250000	0.002241
3616.000000	0.002258
3615.750000	0.002285
3615.500000	0.002312
3615.250000	0.002330
3615.000000	0.002330
3614.750000	0.002306
3614.500000	0.002262
3614.250000	0.002208
3614.000000	0.002158
3613.750000	0.002128
3613.500000	0.002129
3613.250000	0.002162
3613.000000	0.002217
3612.750000	0.002278
3612.500000	0.002327
3612.250000	0.002350
3612.000000	0.002347
3611.750000	0.002323
3611.500000	0.002287
3611.250000	0.002250
3611.000000	0.002218
3610.750000	0.002196
3610.500000	0.002185
3610.250000	0.002188
3610.000000	0.002202

3609.750000	0.002224
3609.500000	0.002244
3609.250000	0.002257
3609.000000	0.002259
3608.750000	0.002254
3608.500000	0.002250
3608.250000	0.002255
3608.000000	0.002276
3607.750000	0.002313
3607.500000	0.002361
3607.250000	0.002413
3607.000000	0.002458
3606.750000	0.002489
3606.500000	0.002505
3606.250000	0.002508
3606.000000	0.002505
3605.750000	0.002502
3605.500000	0.002502
3605.250000	0.002507
3605.000000	0.002513
3604.750000	0.002518
3604.500000	0.002519
3604.250000	0.002515
3604.000000	0.002505
3603.750000	0.002491
3603.500000	0.002473

3603.250000	0.002452
3603.000000	0.002429
3602.750000	0.002405
3602.500000	0.002382
3602.250000	0.002363
3602.000000	0.002349
3601.750000	0.002342
3601.500000	0.002343
3601.250000	0.002352
3601.000000	0.002367
3600.750000	0.002386
3600.500000	0.002405
3600.250000	0.002422
3600.000000	0.002438
3599.750000	0.002452
3599.500000	0.002464
3599.250000	0.002474
3599.000000	0.002483
3598.750000	0.002488
3598.500000	0.002489
3598.250000	0.002485
3598.000000	0.002477
3597.750000	0.002467
3597.500000	0.002458
3597.250000	0.002451
3597.000000	0.002448

3596.750000	0.002450
3596.500000	0.002455
3596.250000	0.002462
3596.000000	0.002467
3595.750000	0.002467
3595.500000	0.002459
3595.250000	0.002443
3595.000000	0.002423
3594.750000	0.002403
3594.500000	0.002391
3594.250000	0.002394
3594.000000	0.002414
3593.750000	0.002451
3593.500000	0.002498
3593.250000	0.002546
3593.000000	0.002585
3592.750000	0.002608
3592.500000	0.002613
3592.250000	0.002602
3592.000000	0.002581
3591.750000	0.002554
3591.500000	0.002527
3591.250000	0.002502
3591.000000	0.002480
3590.750000	0.002459
3590.500000	0.002436

3590.250000	0.002411
3590.000000	0.002386
3589.750000	0.002364
3589.500000	0.002347
3589.250000	0.002337
3589.000000	0.002334
3588.750000	0.002333
3588.500000	0.002330
3588.250000	0.002325
3588.000000	0.002321
3587.750000	0.002323
3587.500000	0.002336
3587.250000	0.002363
3587.000000	0.002400
3586.750000	0.002440
3586.500000	0.002476
3586.250000	0.002503
3586.000000	0.002522
3585.750000	0.002535
3585.500000	0.002547
3585.250000	0.002564
3585.000000	0.002585
3584.750000	0.002610
3584.500000	0.002637
3584.250000	0.002661
3584.000000	0.002681

3583.750000	0.002695
3583.500000	0.002700
3583.250000	0.002697
3583.000000	0.002687
3582.750000	0.002672
3582.500000	0.002653
3582.250000	0.002635
3582.000000	0.002619
3581.750000	0.002609
3581.500000	0.002605
3581.250000	0.002608
3581.000000	0.002615
3580.750000	0.002626
3580.500000	0.002636
3580.250000	0.002643
3580.000000	0.002648
3579.750000	0.002648
3579.500000	0.002647
3579.250000	0.002645
3579.000000	0.002645
3578.750000	0.002648
3578.500000	0.002656
3578.250000	0.002666
3578.000000	0.002679
3577.750000	0.002691
3577.500000	0.002701

3577.250000	0.002707
3577.000000	0.002709
3576.750000	0.002708
3576.500000	0.002705
3576.250000	0.002704
3576.000000	0.002705
3575.750000	0.002711
3575.500000	0.002719
3575.250000	0.002729
3575.000000	0.002738
3574.750000	0.002743
3574.500000	0.002743
3574.250000	0.002737
3574.000000	0.002726
3573.750000	0.002713
3573.500000	0.002699
3573.250000	0.002687
3573.000000	0.002679
3572.750000	0.002675
3572.500000	0.002674
3572.250000	0.002676
3572.000000	0.002680
3571.750000	0.002683
3571.500000	0.002685
3571.250000	0.002684
3571.000000	0.002680

3570.750000	0.002672
3570.500000	0.002660
3570.250000	0.002644
3570.000000	0.002625
3569.750000	0.002605
3569.500000	0.002587
3569.250000	0.002573
3569.000000	0.002565
3568.750000	0.002565
3568.500000	0.002572
3568.250000	0.002585
3568.000000	0.002601
3567.750000	0.002621
3567.500000	0.002647
3567.250000	0.002681
3567.000000	0.002724
3566.750000	0.002773
3566.500000	0.002822
3566.250000	0.002863
3566.000000	0.002890
3565.750000	0.002902
3565.500000	0.002900
3565.250000	0.002893
3565.000000	0.002887
3564.750000	0.002889
3564.500000	0.002901

3564.250000	0.002921
3564.000000	0.002945
3563.750000	0.002966
3563.500000	0.002978
3563.250000	0.002976
3563.000000	0.002961
3562.750000	0.002934
3562.500000	0.002902
3562.250000	0.002870
3562.000000	0.002844
3561.750000	0.002825
3561.500000	0.002816
3561.250000	0.002815
3561.000000	0.002818
3560.750000	0.002824
3560.500000	0.002828
3560.250000	0.002828
3560.000000	0.002825
3559.750000	0.002819
3559.500000	0.002813
3559.250000	0.002810
3559.000000	0.002812
3558.750000	0.002821
3558.500000	0.002837
3558.250000	0.002857
3558.000000	0.002879

3557.750000	0.002900
3557.500000	0.002916
3557.250000	0.002926
3557.000000	0.002930
3556.750000	0.002928
3556.500000	0.002921
3556.250000	0.002911
3556.000000	0.002900
3555.750000	0.002889
3555.500000	0.002878
3555.250000	0.002868
3555.000000	0.002860
3554.750000	0.002855
3554.500000	0.002852
3554.250000	0.002852
3554.000000	0.002857
3553.750000	0.002865
3553.500000	0.002877
3553.250000	0.002891
3553.000000	0.002905
3552.750000	0.002918
3552.500000	0.002927
3552.250000	0.002932
3552.000000	0.002932
3551.750000	0.002928
3551.500000	0.002921

3551.250000	0.002913
3551.000000	0.002908
3550.750000	0.002906
3550.500000	0.002907
3550.250000	0.002913
3550.000000	0.002920
3549.750000	0.002926
3549.500000	0.002931
3549.250000	0.002931
3549.000000	0.002928
3548.750000	0.002923
3548.500000	0.002916
3548.250000	0.002910
3548.000000	0.002906
3547.750000	0.002904
3547.500000	0.002904
3547.250000	0.002904
3547.000000	0.002905
3546.750000	0.002907
3546.500000	0.002912
3546.250000	0.002921
3546.000000	0.002935
3545.750000	0.002953
3545.500000	0.002973
3545.250000	0.002992
3545.000000	0.003007

3544.750000	0.003017
3544.500000	0.003022
3544.250000	0.003024
3544.000000	0.003025
3543.750000	0.003028
3543.500000	0.003033
3543.250000	0.003039
3543.000000	0.003046
3542.750000	0.003051
3542.500000	0.003053
3542.250000	0.003052
3542.000000	0.003048
3541.750000	0.003042
3541.500000	0.003036
3541.250000	0.003029
3541.000000	0.003024
3540.750000	0.003020
3540.500000	0.003018
3540.250000	0.003019
3540.000000	0.003021
3539.750000	0.003027
3539.500000	0.003034
3539.250000	0.003042
3539.000000	0.003050
3538.750000	0.003056
3538.500000	0.003059

3538.250000	0.003058
3538.000000	0.003056
3537.750000	0.003052
3537.500000	0.003049
3537.250000	0.003049
3537.000000	0.003051
3536.750000	0.003055
3536.500000	0.003062
3536.250000	0.003069
3536.000000	0.003078
3535.750000	0.003086
3535.500000	0.003095
3535.250000	0.003105
3535.000000	0.003117
3534.750000	0.003130
3534.500000	0.003143
3534.250000	0.003154
3534.000000	0.003161
3533.750000	0.003164
3533.500000	0.003161
3533.250000	0.003154
3533.000000	0.003144
3532.750000	0.003135
3532.500000	0.003128
3532.250000	0.003125
3532.000000	0.003126

3531.750000	0.003131
3531.500000	0.003136
3531.250000	0.003140
3531.000000	0.003139
3530.750000	0.003133
3530.500000	0.003122
3530.250000	0.003110
3530.000000	0.003100
3529.750000	0.003093
3529.500000	0.003094
3529.250000	0.003101
3529.000000	0.003113
3528.750000	0.003127
3528.500000	0.003140
3528.250000	0.003149
3528.000000	0.003154
3527.750000	0.003156
3527.500000	0.003156
3527.250000	0.003159
3527.000000	0.003164
3526.750000	0.003173
3526.500000	0.003185
3526.250000	0.003197
3526.000000	0.003207
3525.750000	0.003213
3525.500000	0.003216

3525.250000	0.003216
3525.000000	0.003214
3524.750000	0.003213
3524.500000	0.003214
3524.250000	0.003217
3524.000000	0.003222
3523.750000	0.003228
3523.500000	0.003235
3523.250000	0.003241
3523.000000	0.003247
3522.750000	0.003253
3522.500000	0.003259
3522.250000	0.003266
3522.000000	0.003272
3521.750000	0.003279
3521.500000	0.003284
3521.250000	0.003286
3521.000000	0.003286
3520.750000	0.003284
3520.500000	0.003280
3520.250000	0.003275
3520.000000	0.003269
3519.750000	0.003263
3519.500000	0.003256
3519.250000	0.003248
3519.000000	0.003240

3518.750000	0.003230
3518.500000	0.003222
3518.250000	0.003216
3518.000000	0.003214
3517.750000	0.003217
3517.500000	0.003223
3517.250000	0.003233
3517.000000	0.003244
3516.750000	0.003254
3516.500000	0.003262
3516.250000	0.003267
3516.000000	0.003267
3515.750000	0.003264
3515.500000	0.003259
3515.250000	0.003251
3515.000000	0.003243
3514.750000	0.003235
3514.500000	0.003227
3514.250000	0.003220
3514.000000	0.003214
3513.750000	0.003210
3513.500000	0.003209
3513.250000	0.003211
3513.000000	0.003216
3512.750000	0.003226
3512.500000	0.003237

3512.250000	0.003249
3512.000000	0.003259
3511.750000	0.003264
3511.500000	0.003264
3511.250000	0.003258
3511.000000	0.003250
3510.750000	0.003242
3510.500000	0.003239
3510.250000	0.003243
3510.000000	0.003256
3509.750000	0.003277
3509.500000	0.003301
3509.250000	0.003326
3509.000000	0.003347
3508.750000	0.003362
3508.500000	0.003370
3508.250000	0.003374
3508.000000	0.003374
3507.750000	0.003373
3507.500000	0.003371
3507.250000	0.003369
3507.000000	0.003366
3506.750000	0.003362
3506.500000	0.003356
3506.250000	0.003347
3506.000000	0.003336

3505.750000	0.003324
3505.500000	0.003311
3505.250000	0.003299
3505.000000	0.003289
3504.750000	0.003281
3504.500000	0.003277
3504.250000	0.003277
3504.000000	0.003282
3503.750000	0.003292
3503.500000	0.003307
3503.250000	0.003326
3503.000000	0.003347
3502.750000	0.003368
3502.500000	0.003388
3502.250000	0.003405
3502.000000	0.003417
3501.750000	0.003425
3501.500000	0.003429
3501.250000	0.003430
3501.000000	0.003430
3500.750000	0.003429
3500.500000	0.003431
3500.250000	0.003433
3500.000000	0.003438
3499.750000	0.003443
3499.500000	0.003446

3499.250000	0.003447
3499.000000	0.003443
3498.750000	0.003435
3498.500000	0.003423
3498.250000	0.003410
3498.000000	0.003395
3497.750000	0.003383
3497.500000	0.003374
3497.250000	0.003369
3497.000000	0.003368
3496.750000	0.003371
3496.500000	0.003376
3496.250000	0.003383
3496.000000	0.003389
3495.750000	0.003394
3495.500000	0.003397
3495.250000	0.003398
3495.000000	0.003398
3494.750000	0.003395
3494.500000	0.003391
3494.250000	0.003385
3494.000000	0.003379
3493.750000	0.003372
3493.500000	0.003364
3493.250000	0.003358
3493.000000	0.003353

3492.750000	0.003352
3492.500000	0.003354
3492.250000	0.003362
3492.000000	0.003376
3491.750000	0.003396
3491.500000	0.003419
3491.250000	0.003444
3491.000000	0.003468
3490.750000	0.003486
3490.500000	0.003496
3490.250000	0.003498
3490.000000	0.003492
3489.750000	0.003480
3489.500000	0.003465
3489.250000	0.003450
3489.000000	0.003439
3488.750000	0.003431
3488.500000	0.003426
3488.250000	0.003423
3488.000000	0.003420
3487.750000	0.003416
3487.500000	0.003411
3487.250000	0.003407
3487.000000	0.003405
3486.750000	0.003408
3486.500000	0.003416

3486.250000	0.003429
3486.000000	0.003444
3485.750000	0.003459
3485.500000	0.003472
3485.250000	0.003481
3485.000000	0.003486
3484.750000	0.003486
3484.500000	0.003483
3484.250000	0.003477
3484.000000	0.003470
3483.750000	0.003461
3483.500000	0.003452
3483.250000	0.003445
3483.000000	0.003441
3482.750000	0.003441
3482.500000	0.003446
3482.250000	0.003456
3482.000000	0.003469
3481.750000	0.003482
3481.500000	0.003494
3481.250000	0.003504
3481.000000	0.003511
3480.750000	0.003517
3480.500000	0.003523
3480.250000	0.003529
3480.000000	0.003538

3479.750000	0.003549
3479.500000	0.003561
3479.250000	0.003571
3479.000000	0.003579
3478.750000	0.003584
3478.500000	0.003584
3478.250000	0.003581
3478.000000	0.003576
3477.750000	0.003570
3477.500000	0.003565
3477.250000	0.003561
3477.000000	0.003561
3476.750000	0.003564
3476.500000	0.003571
3476.250000	0.003579
3476.000000	0.003589
3475.750000	0.003597
3475.500000	0.003602
3475.250000	0.003604
3475.000000	0.003602
3474.750000	0.003598
3474.500000	0.003595
3474.250000	0.003595
3474.000000	0.003600
3473.750000	0.003610
3473.500000	0.003623

3473.250000	0.003639
3473.000000	0.003654
3472.750000	0.003665
3472.500000	0.003672
3472.250000	0.003673
3472.000000	0.003670
3471.750000	0.003666
3471.500000	0.003660
3471.250000	0.003656
3471.000000	0.003654
3470.750000	0.003654
3470.500000	0.003656
3470.250000	0.003657
3470.000000	0.003659
3469.750000	0.003659
3469.500000	0.003659
3469.250000	0.003658
3469.000000	0.003658
3468.750000	0.003659
3468.500000	0.003661
3468.250000	0.003664
3468.000000	0.003669
3467.750000	0.003677
3467.500000	0.003686
3467.250000	0.003699
3467.000000	0.003715

3466.750000	0.003734
3466.500000	0.003754
3466.250000	0.003775
3466.000000	0.003794
3465.750000	0.003809
3465.500000	0.003818
3465.250000	0.003822
3465.000000	0.003821
3464.750000	0.003819
3464.500000	0.003817
3464.250000	0.003818
3464.000000	0.003823
3463.750000	0.003831
3463.500000	0.003843
3463.250000	0.003856
3463.000000	0.003869
3462.750000	0.003881
3462.500000	0.003892
3462.250000	0.003901
3462.000000	0.003908
3461.750000	0.003914
3461.500000	0.003918
3461.250000	0.003921
3461.000000	0.003923
3460.750000	0.003924
3460.500000	0.003927

3460.250000	0.003930
3460.000000	0.003935
3459.750000	0.003941
3459.500000	0.003948
3459.250000	0.003954
3459.000000	0.003961
3458.750000	0.003968
3458.500000	0.003975
3458.250000	0.003984
3458.000000	0.003992
3457.750000	0.004001
3457.500000	0.004009
3457.250000	0.004013
3457.000000	0.004015
3456.750000	0.004015
3456.500000	0.004014
3456.250000	0.004017
3456.000000	0.004025
3455.750000	0.004039
3455.500000	0.004059
3455.250000	0.004084
3455.000000	0.004109
3454.750000	0.004133
3454.500000	0.004152
3454.250000	0.004165
3454.000000	0.004172

3453.750000	0.004175
3453.500000	0.004174
3453.250000	0.004172
3453.000000	0.004170
3452.750000	0.004171
3452.500000	0.004174
3452.250000	0.004180
3452.000000	0.004189
3451.750000	0.004199
3451.500000	0.004208
3451.250000	0.004215
3451.000000	0.004219
3450.750000	0.004218
3450.500000	0.004213
3450.250000	0.004206
3450.000000	0.004199
3449.750000	0.004194
3449.500000	0.004192
3449.250000	0.004194
3449.000000	0.004202
3448.750000	0.004214
3448.500000	0.004230
3448.250000	0.004249
3448.000000	0.004270
3447.750000	0.004291
3447.500000	0.004312

3447.250000	0.004329
3447.000000	0.004344
3446.750000	0.004357
3446.500000	0.004369
3446.250000	0.004384
3446.000000	0.004401
3445.750000	0.004422
3445.500000	0.004444
3445.250000	0.004465
3445.000000	0.004483
3444.750000	0.004493
3444.500000	0.004496
3444.250000	0.004491
3444.000000	0.004481
3443.750000	0.004469
3443.500000	0.004459
3443.250000	0.004451
3443.000000	0.004448
3442.750000	0.004449
3442.500000	0.004453
3442.250000	0.004459
3442.000000	0.004464
3441.750000	0.004471
3441.500000	0.004478
3441.250000	0.004487
3441.000000	0.004498

3440.750000	0.004511
3440.500000	0.004527
3440.250000	0.004544
3440.000000	0.004561
3439.750000	0.004576
3439.500000	0.004589
3439.250000	0.004599
3439.000000	0.004607
3438.750000	0.004613
3438.500000	0.004617
3438.250000	0.004621
3438.000000	0.004624
3437.750000	0.004627
3437.500000	0.004630
3437.250000	0.004632
3437.000000	0.004632
3436.750000	0.004632
3436.500000	0.004630
3436.250000	0.004628
3436.000000	0.004626
3435.750000	0.004625
3435.500000	0.004626
3435.250000	0.004630
3435.000000	0.004639
3434.750000	0.004652
3434.500000	0.004668

3434.250000	0.004688
3434.000000	0.004710
3433.750000	0.004730
3433.500000	0.004748
3433.250000	0.004762
3433.000000	0.004770
3432.750000	0.004774
3432.500000	0.004776
3432.250000	0.004778
3432.000000	0.004783
3431.750000	0.004792
3431.500000	0.004807
3431.250000	0.004826
3431.000000	0.004848
3430.750000	0.004870
3430.500000	0.004892
3430.250000	0.004911
3430.000000	0.004926
3429.750000	0.004939
3429.500000	0.004949
3429.250000	0.004958
3429.000000	0.004967
3428.750000	0.004978
3428.500000	0.004991
3428.250000	0.005008
3428.000000	0.005028

3427.750000	0.005051
3427.500000	0.005075
3427.250000	0.005099
3427.000000	0.005122
3426.750000	0.005142
3426.500000	0.005159
3426.250000	0.005172
3426.000000	0.005184
3425.750000	0.005195
3425.500000	0.005206
3425.250000	0.005219
3425.000000	0.005232
3424.750000	0.005246
3424.500000	0.005258
3424.250000	0.005267
3424.000000	0.005273
3423.750000	0.005275
3423.500000	0.005274
3423.250000	0.005272
3423.000000	0.005270
3422.750000	0.005269
3422.500000	0.005271
3422.250000	0.005275
3422.000000	0.005281
3421.750000	0.005289
3421.500000	0.005300

3421.250000	0.005314
3421.000000	0.005332
3420.750000	0.005356
3420.500000	0.005385
3420.250000	0.005418
3420.000000	0.005454
3419.750000	0.005489
3419.500000	0.005521
3419.250000	0.005547
3419.000000	0.005566
3418.750000	0.005579
3418.500000	0.005588
3418.250000	0.005596
3418.000000	0.005604
3417.750000	0.005613
3417.500000	0.005622
3417.250000	0.005632
3417.000000	0.005640
3416.750000	0.005648
3416.500000	0.005655
3416.250000	0.005663
3416.000000	0.005674
3415.750000	0.005687
3415.500000	0.005701
3415.250000	0.005716
3415.000000	0.005729

3414.750000	0.005739
3414.500000	0.005745
3414.250000	0.005749
3414.000000	0.005752
3413.750000	0.005759
3413.500000	0.005769
3413.250000	0.005786
3413.000000	0.005808
3412.750000	0.005833
3412.500000	0.005860
3412.250000	0.005887
3412.000000	0.005911
3411.750000	0.005933
3411.500000	0.005951
3411.250000	0.005966
3411.000000	0.005980
3410.750000	0.005992
3410.500000	0.006006
3410.250000	0.006023
3410.000000	0.006043
3409.750000	0.006067
3409.500000	0.006094
3409.250000	0.006121
3409.000000	0.006146
3408.750000	0.006168
3408.500000	0.006186

3408.250000	0.006201
3408.000000	0.006214
3407.750000	0.006227
3407.500000	0.006242
3407.250000	0.006260
3407.000000	0.006278
3406.750000	0.006296
3406.500000	0.006312
3406.250000	0.006325
3406.000000	0.006335
3405.750000	0.006344
3405.500000	0.006352
3405.250000	0.006361
3405.000000	0.006372
3404.750000	0.006382
3404.500000	0.006393
3404.250000	0.006402
3404.000000	0.006411
3403.750000	0.006419
3403.500000	0.006429
3403.250000	0.006441
3403.000000	0.006456
3402.750000	0.006473
3402.500000	0.006491
3402.250000	0.006510
3402.000000	0.006529

3401.750000	0.006547
3401.500000	0.006565
3401.250000	0.006584
3401.000000	0.006603
3400.750000	0.006623
3400.500000	0.006642
3400.250000	0.006660
3400.000000	0.006678
3399.750000	0.006695
3399.500000	0.006712
3399.250000	0.006731
3399.000000	0.006754
3398.750000	0.006781
3398.500000	0.006812
3398.250000	0.006846
3398.000000	0.006881
3397.750000	0.006915
3397.500000	0.006946
3397.250000	0.006972
3397.000000	0.006992
3396.750000	0.007006
3396.500000	0.007013
3396.250000	0.007015
3396.000000	0.007015
3395.750000	0.007013
3395.500000	0.007011

3395.250000	0.007012
3395.000000	0.007016
3394.750000	0.007022
3394.500000	0.007032
3394.250000	0.007043
3394.000000	0.007055
3393.750000	0.007067
3393.500000	0.007078
3393.250000	0.007087
3393.000000	0.007094
3392.750000	0.007099
3392.500000	0.007103
3392.250000	0.007107
3392.000000	0.007114
3391.750000	0.007126
3391.500000	0.007144
3391.250000	0.007168
3391.000000	0.007196
3390.750000	0.007225
3390.500000	0.007250
3390.250000	0.007268
3390.000000	0.007278
3389.750000	0.007281
3389.500000	0.007279
3389.250000	0.007275
3389.000000	0.007273

3388.750000	0.007275
3388.500000	0.007281
3388.250000	0.007292
3388.000000	0.007304
3387.750000	0.007316
3387.500000	0.007329
3387.250000	0.007340
3387.000000	0.007352
3386.750000	0.007364
3386.500000	0.007376
3386.250000	0.007387
3386.000000	0.007397
3385.750000	0.007403
3385.500000	0.007406
3385.250000	0.007406
3385.000000	0.007404
3384.750000	0.007401
3384.500000	0.007399
3384.250000	0.007398
3384.000000	0.007400
3383.750000	0.007402
3383.500000	0.007403
3383.250000	0.007404
3383.000000	0.007402
3382.750000	0.007398
3382.500000	0.007392

3382.250000	0.007386
3382.000000	0.007382
3381.750000	0.007379
3381.500000	0.007379
3381.250000	0.007381
3381.000000	0.007384
3380.750000	0.007388
3380.500000	0.007390
3380.250000	0.007390
3380.000000	0.007388
3379.750000	0.007383
3379.500000	0.007377
3379.250000	0.007371
3379.000000	0.007366
3378.750000	0.007363
3378.500000	0.007361
3378.250000	0.007361
3378.000000	0.007361
3377.750000	0.007360
3377.500000	0.007357
3377.250000	0.007353
3377.000000	0.007348
3376.750000	0.007341
3376.500000	0.007335
3376.250000	0.007330
3376.000000	0.007326

3375.750000	0.007326
3375.500000	0.007329
3375.250000	0.007338
3375.000000	0.007353
3374.750000	0.007372
3374.500000	0.007393
3374.250000	0.007414
3374.000000	0.007429
3373.750000	0.007437
3373.500000	0.007436
3373.250000	0.007425
3373.000000	0.007406
3372.750000	0.007383
3372.500000	0.007359
3372.250000	0.007337
3372.000000	0.007319
3371.750000	0.007304
3371.500000	0.007292
3371.250000	0.007282
3371.000000	0.007273
3370.750000	0.007266
3370.500000	0.007259
3370.250000	0.007254
3370.000000	0.007250
3369.750000	0.007247
3369.500000	0.007244

3369.250000	0.007241
3369.000000	0.007238
3368.750000	0.007236
3368.500000	0.007234
3368.250000	0.007234
3368.000000	0.007234
3367.750000	0.007234
3367.500000	0.007232
3367.250000	0.007227
3367.000000	0.007218
3366.750000	0.007205
3366.500000	0.007191
3366.250000	0.007179
3366.000000	0.007170
3365.750000	0.007168
3365.500000	0.007172
3365.250000	0.007181
3365.000000	0.007192
3364.750000	0.007202
3364.500000	0.007210
3364.250000	0.007213
3364.000000	0.007214
3363.750000	0.007212
3363.500000	0.007209
3363.250000	0.007207
3363.000000	0.007207

3362.750000	0.007208
3362.500000	0.007209
3362.250000	0.007210
3362.000000	0.007208
3361.750000	0.007204
3361.500000	0.007197
3361.250000	0.007186
3361.000000	0.007172
3360.750000	0.007155
3360.500000	0.007136
3360.250000	0.007117
3360.000000	0.007099
3359.750000	0.007083
3359.500000	0.007072
3359.250000	0.007066
3359.000000	0.007066
3358.750000	0.007071
3358.500000	0.007080
3358.250000	0.007091
3358.000000	0.007101
3357.750000	0.007109
3357.500000	0.007113
3357.250000	0.007111
3357.000000	0.007104
3356.750000	0.007091
3356.500000	0.007074

3356.250000	0.007053
3356.000000	0.007030
3355.750000	0.007008
3355.500000	0.006988
3355.250000	0.006973
3355.000000	0.006962
3354.750000	0.006957
3354.500000	0.006954
3354.250000	0.006953
3354.000000	0.006951
3353.750000	0.006945
3353.500000	0.006935
3353.250000	0.006922
3353.000000	0.006906
3352.750000	0.006890
3352.500000	0.006875
3352.250000	0.006863
3352.000000	0.006855
3351.750000	0.006850
3351.500000	0.006847
3351.250000	0.006845
3351.000000	0.006841
3350.750000	0.006837
3350.500000	0.006831
3350.250000	0.006825
3350.000000	0.006820

3349.750000	0.006817
3349.500000	0.006817
3349.250000	0.006821
3349.000000	0.006829
3348.750000	0.006840
3348.500000	0.006852
3348.250000	0.006863
3348.000000	0.006872
3347.750000	0.006878
3347.500000	0.006879
3347.250000	0.006877
3347.000000	0.006870
3346.750000	0.006862
3346.500000	0.006852
3346.250000	0.006841
3346.000000	0.006831
3345.750000	0.006823
3345.500000	0.006816
3345.250000	0.006812
3345.000000	0.006809
3344.750000	0.006809
3344.500000	0.006809
3344.250000	0.006808
3344.000000	0.006805
3343.750000	0.006800
3343.500000	0.006791

3343.250000	0.006779
3343.000000	0.006765
3342.750000	0.006750
3342.500000	0.006736
3342.250000	0.006724
3342.000000	0.006715
3341.750000	0.006709
3341.500000	0.006707
3341.250000	0.006707
3341.000000	0.006710
3340.750000	0.006713
3340.500000	0.006717
3340.250000	0.006718
3340.000000	0.006717
3339.750000	0.006711
3339.500000	0.006700
3339.250000	0.006685
3339.000000	0.006667
3338.750000	0.006647
3338.500000	0.006630
3338.250000	0.006617
3338.000000	0.006609
3337.750000	0.006608
3337.500000	0.006613
3337.250000	0.006620
3337.000000	0.006628

3336.750000	0.006634
3336.500000	0.006637
3336.250000	0.006637
3336.000000	0.006634
3335.750000	0.006630
3335.500000	0.006627
3335.250000	0.006625
3335.000000	0.006622
3334.750000	0.006618
3334.500000	0.006611
3334.250000	0.006599
3334.000000	0.006582
3333.750000	0.006561
3333.500000	0.006538
3333.250000	0.006515
3333.000000	0.006495
3332.750000	0.006479
3332.500000	0.006466
3332.250000	0.006456
3332.000000	0.006448
3331.750000	0.006440
3331.500000	0.006431
3331.250000	0.006421
3331.000000	0.006412
3330.750000	0.006402
3330.500000	0.006394

3330.250000	0.006388
3330.000000	0.006384
3329.750000	0.006380
3329.500000	0.006377
3329.250000	0.006374
3329.000000	0.006369
3328.750000	0.006363
3328.500000	0.006357
3328.250000	0.006349
3328.000000	0.006341
3327.750000	0.006333
3327.500000	0.006324
3327.250000	0.006316
3327.000000	0.006308
3326.750000	0.006299
3326.500000	0.006291
3326.250000	0.006284
3326.000000	0.006278
3325.750000	0.006275
3325.500000	0.006274
3325.250000	0.006275
3325.000000	0.006277
3324.750000	0.006278
3324.500000	0.006276
3324.250000	0.006270
3324.000000	0.006258

3323.750000	0.006242
3323.500000	0.006222
3323.250000	0.006201
3323.000000	0.006180
3322.750000	0.006161
3322.500000	0.006145
3322.250000	0.006130
3322.000000	0.006115
3321.750000	0.006099
3321.500000	0.006082
3321.250000	0.006063
3321.000000	0.006044
3320.750000	0.006026
3320.500000	0.006011
3320.250000	0.005998
3320.000000	0.005988
3319.750000	0.005978
3319.500000	0.005969
3319.250000	0.005960
3319.000000	0.005952
3318.750000	0.005944
3318.500000	0.005938
3318.250000	0.005935
3318.000000	0.005933
3317.750000	0.005933
3317.500000	0.005932

3317.250000	0.005929
3317.000000	0.005923
3316.750000	0.005914
3316.500000	0.005901
3316.250000	0.005886
3316.000000	0.005871
3315.750000	0.005855
3315.500000	0.005840
3315.250000	0.005826
3315.000000	0.005814
3314.750000	0.005802
3314.500000	0.005792
3314.250000	0.005783
3314.000000	0.005775
3313.750000	0.005769
3313.500000	0.005764
3313.250000	0.005758
3313.000000	0.005751
3312.750000	0.005743
3312.500000	0.005730
3312.250000	0.005714
3312.000000	0.005695
3311.750000	0.005672
3311.500000	0.005648
3311.250000	0.005625
3311.000000	0.005604

3310.750000	0.005587
3310.500000	0.005574
3310.250000	0.005565
3310.000000	0.005558
3309.750000	0.005552
3309.500000	0.005546
3309.250000	0.005538
3309.000000	0.005529
3308.750000	0.005519
3308.500000	0.005508
3308.250000	0.005497
3308.000000	0.005487
3307.750000	0.005476
3307.500000	0.005466
3307.250000	0.005455
3307.000000	0.005444
3306.750000	0.005433
3306.500000	0.005421
3306.250000	0.005410
3306.000000	0.005399
3305.750000	0.005387
3305.500000	0.005375
3305.250000	0.005361
3305.000000	0.005345
3304.750000	0.005328
3304.500000	0.005310

3304.250000	0.005293
3304.000000	0.005277
3303.750000	0.005264
3303.500000	0.005255
3303.250000	0.005250
3303.000000	0.005247
3302.750000	0.005246
3302.500000	0.005242
3302.250000	0.005235
3302.000000	0.005223
3301.750000	0.005206
3301.500000	0.005185
3301.250000	0.005162
3301.000000	0.005141
3300.750000	0.005124
3300.500000	0.005112
3300.250000	0.005106
3300.000000	0.005104
3299.750000	0.005103
3299.500000	0.005101
3299.250000	0.005096
3299.000000	0.005089
3298.750000	0.005079
3298.500000	0.005067
3298.250000	0.005056
3298.000000	0.005045

3297.750000	0.005034
3297.500000	0.005024
3297.250000	0.005013
3297.000000	0.005000
3296.750000	0.004986
3296.500000	0.004971
3296.250000	0.004956
3296.000000	0.004941
3295.750000	0.004927
3295.500000	0.004915
3295.250000	0.004905
3295.000000	0.004897
3294.750000	0.004890
3294.500000	0.004885
3294.250000	0.004879
3294.000000	0.004871
3293.750000	0.004858
3293.500000	0.004841
3293.250000	0.004818
3293.000000	0.004790
3292.750000	0.004759
3292.500000	0.004731
3292.250000	0.004708
3292.000000	0.004693
3291.750000	0.004687
3291.500000	0.004689

3291.250000	0.004697
3291.000000	0.004707
3290.750000	0.004714
3290.500000	0.004715
3290.250000	0.004711
3290.000000	0.004702
3289.750000	0.004688
3289.500000	0.004674
3289.250000	0.004660
3289.000000	0.004649
3288.750000	0.004640
3288.500000	0.004633
3288.250000	0.004627
3288.000000	0.004622
3287.750000	0.004615
3287.500000	0.004607
3287.250000	0.004598
3287.000000	0.004590
3286.750000	0.004582
3286.500000	0.004575
3286.250000	0.004570
3286.000000	0.004566
3285.750000	0.004564
3285.500000	0.004561
3285.250000	0.004558
3285.000000	0.004552

3284.750000	0.004543
3284.500000	0.004530
3284.250000	0.004514
3284.000000	0.004497
3283.750000	0.004479
3283.500000	0.004461
3283.250000	0.004446
3283.000000	0.004433
3282.750000	0.004423
3282.500000	0.004414
3282.250000	0.004407
3282.000000	0.004399
3281.750000	0.004392
3281.500000	0.004385
3281.250000	0.004378
3281.000000	0.004372
3280.750000	0.004368
3280.500000	0.004364
3280.250000	0.004360
3280.000000	0.004357
3279.750000	0.004354
3279.500000	0.004351
3279.250000	0.004347
3279.000000	0.004344
3278.750000	0.004340
3278.500000	0.004337

3278.250000	0.004331
3278.000000	0.004322
3277.750000	0.004308
3277.500000	0.004289
3277.250000	0.004266
3277.000000	0.004238
3276.750000	0.004209
3276.500000	0.004180
3276.250000	0.004152
3276.000000	0.004128
3275.750000	0.004109
3275.500000	0.004093
3275.250000	0.004083
3275.000000	0.004077
3274.750000	0.004075
3274.500000	0.004076
3274.250000	0.004078
3274.000000	0.004078
3273.750000	0.004075
3273.500000	0.004067
3273.250000	0.004056
3273.000000	0.004041
3272.750000	0.004025
3272.500000	0.004010
3272.250000	0.003997
3272.000000	0.003988

3271.750000	0.003982
3271.500000	0.003980
3271.250000	0.003980
3271.000000	0.003982
3270.750000	0.003986
3270.500000	0.003990
3270.250000	0.003995
3270.000000	0.004000
3269.750000	0.004003
3269.500000	0.004004
3269.250000	0.004000
3269.000000	0.003994
3268.750000	0.003984
3268.500000	0.003973
3268.250000	0.003961
3268.000000	0.003950
3267.750000	0.003940
3267.500000	0.003930
3267.250000	0.003920
3267.000000	0.003910
3266.750000	0.003900
3266.500000	0.003890
3266.250000	0.003881
3266.000000	0.003872
3265.750000	0.003864
3265.500000	0.003856

3265.250000	0.003848
3265.000000	0.003840
3264.750000	0.003833
3264.500000	0.003826
3264.250000	0.003822
3264.000000	0.003819
3263.750000	0.003817
3263.500000	0.003815
3263.250000	0.003811
3263.000000	0.003805
3262.750000	0.003795
3262.500000	0.003783
3262.250000	0.003769
3262.000000	0.003755
3261.750000	0.003743
3261.500000	0.003736
3261.250000	0.003733
3261.000000	0.003737
3260.750000	0.003745
3260.500000	0.003757
3260.250000	0.003770
3260.000000	0.003781
3259.750000	0.003787
3259.500000	0.003785
3259.250000	0.003776
3259.000000	0.003761

3258.750000	0.003742
3258.500000	0.003722
3258.250000	0.003704
3258.000000	0.003690
3257.750000	0.003680
3257.500000	0.003674
3257.250000	0.003667
3257.000000	0.003660
3256.750000	0.003650
3256.500000	0.003639
3256.250000	0.003627
3256.000000	0.003616
3255.750000	0.003609
3255.500000	0.003607
3255.250000	0.003610
3255.000000	0.003617
3254.750000	0.003625
3254.500000	0.003633
3254.250000	0.003638
3254.000000	0.003639
3253.750000	0.003636
3253.500000	0.003628
3253.250000	0.003617
3253.000000	0.003605
3252.750000	0.003594
3252.500000	0.003584

3252.250000	0.003579
3252.000000	0.003577
3251.750000	0.003580
3251.500000	0.003585
3251.250000	0.003590
3251.000000	0.003594
3250.750000	0.003595
3250.500000	0.003592
3250.250000	0.003584
3250.000000	0.003573
3249.750000	0.003560
3249.500000	0.003547
3249.250000	0.003534
3249.000000	0.003522
3248.750000	0.003510
3248.500000	0.003499
3248.250000	0.003488
3248.000000	0.003478
3247.750000	0.003467
3247.500000	0.003458
3247.250000	0.003451
3247.000000	0.003447
3246.750000	0.003444
3246.500000	0.003444
3246.250000	0.003445
3246.000000	0.003446

3245.750000	0.003447
3245.500000	0.003447
3245.250000	0.003445
3245.000000	0.003442
3244.750000	0.003439
3244.500000	0.003434
3244.250000	0.003429
3244.000000	0.003423
3243.750000	0.003418
3243.500000	0.003413
3243.250000	0.003410
3243.000000	0.003410
3242.750000	0.003412
3242.500000	0.003416
3242.250000	0.003423
3242.000000	0.003430
3241.750000	0.003437
3241.500000	0.003440
3241.250000	0.003441
3241.000000	0.003438
3240.750000	0.003433
3240.500000	0.003426
3240.250000	0.003420
3240.000000	0.003415
3239.750000	0.003411
3239.500000	0.003407

3239.250000	0.003404
3239.000000	0.003400
3238.750000	0.003393
3238.500000	0.003384
3238.250000	0.003371
3238.000000	0.003357
3237.750000	0.003342
3237.500000	0.003326
3237.250000	0.003311
3237.000000	0.003300
3236.750000	0.003291
3236.500000	0.003287
3236.250000	0.003286
3236.000000	0.003288
3235.750000	0.003291
3235.500000	0.003294
3235.250000	0.003294
3235.000000	0.003292
3234.750000	0.003288
3234.500000	0.003283
3234.250000	0.003278
3234.000000	0.003274
3233.750000	0.003272
3233.500000	0.003270
3233.250000	0.003269
3233.000000	0.003268

3232.750000	0.003265
3232.500000	0.003263
3232.250000	0.003260
3232.000000	0.003259
3231.750000	0.003259
3231.500000	0.003260
3231.250000	0.003261
3231.000000	0.003263
3230.750000	0.003263
3230.500000	0.003263
3230.250000	0.003263
3230.000000	0.003263
3229.750000	0.003264
3229.500000	0.003267
3229.250000	0.003271
3229.000000	0.003274
3228.750000	0.003275
3228.500000	0.003272
3228.250000	0.003265
3228.000000	0.003254
3227.750000	0.003240
3227.500000	0.003224
3227.250000	0.003208
3227.000000	0.003195
3226.750000	0.003185
3226.500000	0.003178

3226.250000	0.003174
3226.000000	0.003173
3225.750000	0.003174
3225.500000	0.003177
3225.250000	0.003181
3225.000000	0.003187
3224.750000	0.003193
3224.500000	0.003199
3224.250000	0.003204
3224.000000	0.003206
3223.750000	0.003205
3223.500000	0.003201
3223.250000	0.003194
3223.000000	0.003185
3222.750000	0.003175
3222.500000	0.003165
3222.250000	0.003154
3222.000000	0.003143
3221.750000	0.003132
3221.500000	0.003121
3221.250000	0.003111
3221.000000	0.003104
3220.750000	0.003102
3220.500000	0.003106
3220.250000	0.003116
3220.000000	0.003131

3219.750000	0.003149
3219.500000	0.003167
3219.250000	0.003180
3219.000000	0.003187
3218.750000	0.003188
3218.500000	0.003181
3218.250000	0.003170
3218.000000	0.003156
3217.750000	0.003141
3217.500000	0.003126
3217.250000	0.003113
3217.000000	0.003101
3216.750000	0.003089
3216.500000	0.003078
3216.250000	0.003068
3216.000000	0.003060
3215.750000	0.003054
3215.500000	0.003051
3215.250000	0.003053
3215.000000	0.003059
3214.750000	0.003069
3214.500000	0.003080
3214.250000	0.003091
3214.000000	0.003101
3213.750000	0.003109
3213.500000	0.003115

3213.250000	0.003118
3213.000000	0.003118
3212.750000	0.003115
3212.500000	0.003110
3212.250000	0.003101
3212.000000	0.003091
3211.750000	0.003078
3211.500000	0.003066
3211.250000	0.003057
3211.000000	0.003051
3210.750000	0.003049
3210.500000	0.003052
3210.250000	0.003058
3210.000000	0.003066
3209.750000	0.003073
3209.500000	0.003079
3209.250000	0.003081
3209.000000	0.003082
3208.750000	0.003080
3208.500000	0.003077
3208.250000	0.003073
3208.000000	0.003070
3207.750000	0.003067
3207.500000	0.003066
3207.250000	0.003067
3207.000000	0.003070

3206.750000	0.003075
3206.500000	0.003081
3206.250000	0.003087
3206.000000	0.003090
3205.750000	0.003091
3205.500000	0.003087
3205.250000	0.003081
3205.000000	0.003074
3204.750000	0.003067
3204.500000	0.003063
3204.250000	0.003061
3204.000000	0.003060
3203.750000	0.003059
3203.500000	0.003058
3203.250000	0.003054
3203.000000	0.003048
3202.750000	0.003042
3202.500000	0.003037
3202.250000	0.003035
3202.000000	0.003037
3201.750000	0.003042
3201.500000	0.003050
3201.250000	0.003058
3201.000000	0.003067
3200.750000	0.003075
3200.500000	0.003080

3200.250000	0.003083
3200.000000	0.003081
3199.750000	0.003075
3199.500000	0.003065
3199.250000	0.003052
3199.000000	0.003039
3198.750000	0.003027
3198.500000	0.003018
3198.250000	0.003014
3198.000000	0.003014
3197.750000	0.003016
3197.500000	0.003017
3197.250000	0.003017
3197.000000	0.003015
3196.750000	0.003012
3196.500000	0.003010
3196.250000	0.003011
3196.000000	0.003014
3195.750000	0.003020
3195.500000	0.003028
3195.250000	0.003035
3195.000000	0.003041
3194.750000	0.003043
3194.500000	0.003044
3194.250000	0.003043
3194.000000	0.003041

3193.750000	0.003039
3193.500000	0.003037
3193.250000	0.003034
3193.000000	0.003030
3192.750000	0.003026
3192.500000	0.003021
3192.250000	0.003017
3192.000000	0.003015
3191.750000	0.003013
3191.500000	0.003012
3191.250000	0.003011
3191.000000	0.003010
3190.750000	0.003009
3190.500000	0.003008
3190.250000	0.003009
3190.000000	0.003011
3189.750000	0.003015
3189.500000	0.003017
3189.250000	0.003017
3189.000000	0.003013
3188.750000	0.003004
3188.500000	0.002990
3188.250000	0.002974
3188.000000	0.002957
3187.750000	0.002943
3187.500000	0.002933

3187.250000	0.002928
3187.000000	0.002927
3186.750000	0.002929
3186.500000	0.002935
3186.250000	0.002942
3186.000000	0.002950
3185.750000	0.002956
3185.500000	0.002960
3185.250000	0.002961
3185.000000	0.002959
3184.750000	0.002954
3184.500000	0.002945
3184.250000	0.002935
3184.000000	0.002925
3183.750000	0.002915
3183.500000	0.002906
3183.250000	0.002900
3183.000000	0.002895
3182.750000	0.002894
3182.500000	0.002897
3182.250000	0.002902
3182.000000	0.002911
3181.750000	0.002921
3181.500000	0.002930
3181.250000	0.002936
3181.000000	0.002939

3180.750000	0.002938
3180.500000	0.002933
3180.250000	0.002928
3180.000000	0.002924
3179.750000	0.002922
3179.500000	0.002923
3179.250000	0.002927
3179.000000	0.002933
3178.750000	0.002939
3178.500000	0.002946
3178.250000	0.002952
3178.000000	0.002955
3177.750000	0.002956
3177.500000	0.002954
3177.250000	0.002949
3177.000000	0.002939
3176.750000	0.002928
3176.500000	0.002915
3176.250000	0.002905
3176.000000	0.002898
3175.750000	0.002896
3175.500000	0.002900
3175.250000	0.002908
3175.000000	0.002918
3174.750000	0.002929
3174.500000	0.002937

3174.250000	0.002940
3174.000000	0.002939
3173.750000	0.002931
3173.500000	0.002917
3173.250000	0.002899
3173.000000	0.002877
3172.750000	0.002853
3172.500000	0.002830
3172.250000	0.002810
3172.000000	0.002793
3171.750000	0.002782
3171.500000	0.002775
3171.250000	0.002772
3171.000000	0.002772
3170.750000	0.002774
3170.500000	0.002778
3170.250000	0.002782
3170.000000	0.002788
3169.750000	0.002795
3169.500000	0.002802
3169.250000	0.002806
3169.000000	0.002808
3168.750000	0.002805
3168.500000	0.002797
3168.250000	0.002785
3168.000000	0.002770

3167.750000	0.002755
3167.500000	0.002743
3167.250000	0.002734
3167.000000	0.002730
3166.750000	0.002730
3166.500000	0.002732
3166.250000	0.002736
3166.000000	0.002739
3165.750000	0.002741
3165.500000	0.002740
3165.250000	0.002736
3165.000000	0.002731
3164.750000	0.002724
3164.500000	0.002718
3164.250000	0.002713
3164.000000	0.002709
3163.750000	0.002707
3163.500000	0.002706
3163.250000	0.002704
3163.000000	0.002702
3162.750000	0.002700
3162.500000	0.002697
3162.250000	0.002695
3162.000000	0.002694
3161.750000	0.002695
3161.500000	0.002697

3161.250000	0.002701
3161.000000	0.002704
3160.750000	0.002706
3160.500000	0.002707
3160.250000	0.002706
3160.000000	0.002704
3159.750000	0.002700
3159.500000	0.002693
3159.250000	0.002685
3159.000000	0.002674
3158.750000	0.002661
3158.500000	0.002647
3158.250000	0.002634
3158.000000	0.002622
3157.750000	0.002615
3157.500000	0.002613
3157.250000	0.002615
3157.000000	0.002620
3156.750000	0.002627
3156.500000	0.002633
3156.250000	0.002636
3156.000000	0.002635
3155.750000	0.002631
3155.500000	0.002623
3155.250000	0.002613
3155.000000	0.002602

3154.750000	0.002592
3154.500000	0.002584
3154.250000	0.002579
3154.000000	0.002577
3153.750000	0.002577
3153.500000	0.002580
3153.250000	0.002583
3153.000000	0.002587
3152.750000	0.002593
3152.500000	0.002599
3152.250000	0.002607
3152.000000	0.002616
3151.750000	0.002625
3151.500000	0.002632
3151.250000	0.002636
3151.000000	0.002634
3150.750000	0.002627
3150.500000	0.002615
3150.250000	0.002601
3150.000000	0.002587
3149.750000	0.002577
3149.500000	0.002573
3149.250000	0.002574
3149.000000	0.002579
3148.750000	0.002586
3148.500000	0.002593

3148.250000	0.002596
3148.000000	0.002594
3147.750000	0.002588
3147.500000	0.002577
3147.250000	0.002564
3147.000000	0.002551
3146.750000	0.002539
3146.500000	0.002530
3146.250000	0.002524
3146.000000	0.002520
3145.750000	0.002519
3145.500000	0.002517
3145.250000	0.002515
3145.000000	0.002512
3144.750000	0.002507
3144.500000	0.002503
3144.250000	0.002498
3144.000000	0.002495
3143.750000	0.002494
3143.500000	0.002494
3143.250000	0.002495
3143.000000	0.002496
3142.750000	0.002499
3142.500000	0.002502
3142.250000	0.002507
3142.000000	0.002514

3141.750000	0.002522
3141.500000	0.002529
3141.250000	0.002535
3141.000000	0.002537
3140.750000	0.002535
3140.500000	0.002528
3140.250000	0.002518
3140.000000	0.002507
3139.750000	0.002496
3139.500000	0.002489
3139.250000	0.002483
3139.000000	0.002481
3138.750000	0.002481
3138.500000	0.002483
3138.250000	0.002486
3138.000000	0.002489
3137.750000	0.002492
3137.500000	0.002494
3137.250000	0.002494
3137.000000	0.002491
3136.750000	0.002485
3136.500000	0.002478
3136.250000	0.002470
3136.000000	0.002464
3135.750000	0.002463
3135.500000	0.002468

3135.250000	0.002477
3135.000000	0.002489
3134.750000	0.002501
3134.500000	0.002510
3134.250000	0.002514
3134.000000	0.002513
3133.750000	0.002507
3133.500000	0.002498
3133.250000	0.002489
3133.000000	0.002482
3132.750000	0.002478
3132.500000	0.002476
3132.250000	0.002477
3132.000000	0.002478
3131.750000	0.002479
3131.500000	0.002477
3131.250000	0.002472
3131.000000	0.002464
3130.750000	0.002454
3130.500000	0.002444
3130.250000	0.002436
3130.000000	0.002433
3129.750000	0.002436
3129.500000	0.002445
3129.250000	0.002458
3129.000000	0.002472

3128.750000	0.002484
3128.500000	0.002490
3128.250000	0.002490
3128.000000	0.002485
3127.750000	0.002475
3127.500000	0.002464
3127.250000	0.002455
3127.000000	0.002449
3126.750000	0.002447
3126.500000	0.002448
3126.250000	0.002451
3126.000000	0.002455
3125.750000	0.002457
3125.500000	0.002457
3125.250000	0.002457
3125.000000	0.002455
3124.750000	0.002453
3124.500000	0.002450
3124.250000	0.002447
3124.000000	0.002444
3123.750000	0.002440
3123.500000	0.002437
3123.250000	0.002433
3123.000000	0.002431
3122.750000	0.002429
3122.500000	0.002429

3122.250000	0.002430
3122.000000	0.002430
3121.750000	0.002430
3121.500000	0.002427
3121.250000	0.002423
3121.000000	0.002418
3120.750000	0.002413
3120.500000	0.002408
3120.250000	0.002406
3120.000000	0.002406
3119.750000	0.002407
3119.500000	0.002409
3119.250000	0.002409
3119.000000	0.002408
3118.750000	0.002405
3118.500000	0.002402
3118.250000	0.002400
3118.000000	0.002402
3117.750000	0.002407
3117.500000	0.002417
3117.250000	0.002428
3117.000000	0.002440
3116.750000	0.002448
3116.500000	0.002452
3116.250000	0.002451
3116.000000	0.002447

3115.750000	0.002442
3115.500000	0.002439
3115.250000	0.002440
3115.000000	0.002446
3114.750000	0.002458
3114.500000	0.002474
3114.250000	0.002490
3114.000000	0.002504
3113.750000	0.002514
3113.500000	0.002517
3113.250000	0.002512
3113.000000	0.002502
3112.750000	0.002487
3112.500000	0.002471
3112.250000	0.002457
3112.000000	0.002449
3111.750000	0.002448
3111.500000	0.002455
3111.250000	0.002469
3111.000000	0.002487
3110.750000	0.002506
3110.500000	0.002522
3110.250000	0.002534
3110.000000	0.002540
3109.750000	0.002540
3109.500000	0.002536

3109.250000	0.002529
3109.000000	0.002522
3108.750000	0.002517
3108.500000	0.002514
3108.250000	0.002516
3108.000000	0.002521
3107.750000	0.002528
3107.500000	0.002537
3107.250000	0.002546
3107.000000	0.002554
3106.750000	0.002561
3106.500000	0.002566
3106.250000	0.002570
3106.000000	0.002572
3105.750000	0.002573
3105.500000	0.002573
3105.250000	0.002572
3105.000000	0.002570
3104.750000	0.002568
3104.500000	0.002566
3104.250000	0.002564
3104.000000	0.002561
3103.750000	0.002559
3103.500000	0.002557
3103.250000	0.002555
3103.000000	0.002554

3102.750000	0.002553
3102.500000	0.002552
3102.250000	0.002551
3102.000000	0.002550
3101.750000	0.002547
3101.500000	0.002541
3101.250000	0.002533
3101.000000	0.002524
3100.750000	0.002514
3100.500000	0.002506
3100.250000	0.002502
3100.000000	0.002502
3099.750000	0.002508
3099.500000	0.002518
3099.250000	0.002530
3099.000000	0.002541
3098.750000	0.002550
3098.500000	0.002554
3098.250000	0.002554
3098.000000	0.002550
3097.750000	0.002544
3097.500000	0.002537
3097.250000	0.002532
3097.000000	0.002528
3096.750000	0.002527
3096.500000	0.002527

3096.250000	0.002530
3096.000000	0.002533
3095.750000	0.002538
3095.500000	0.002543
3095.250000	0.002547
3095.000000	0.002552
3094.750000	0.002555
3094.500000	0.002559
3094.250000	0.002562
3094.000000	0.002565
3093.750000	0.002568
3093.500000	0.002572
3093.250000	0.002574
3093.000000	0.002574
3092.750000	0.002571
3092.500000	0.002565
3092.250000	0.002555
3092.000000	0.002542
3091.750000	0.002527
3091.500000	0.002511
3091.250000	0.002496
3091.000000	0.002484
3090.750000	0.002475
3090.500000	0.002470
3090.250000	0.002471
3090.000000	0.002477

3089.750000	0.002488
3089.500000	0.002503
3089.250000	0.002519
3089.000000	0.002536
3088.750000	0.002551
3088.500000	0.002563
3088.250000	0.002570
3088.000000	0.002573
3087.750000	0.002571
3087.500000	0.002565
3087.250000	0.002554
3087.000000	0.002541
3086.750000	0.002525
3086.500000	0.002510
3086.250000	0.002496
3086.000000	0.002487
3085.750000	0.002482
3085.500000	0.002484
3085.250000	0.002491
3085.000000	0.002503
3084.750000	0.002516
3084.500000	0.002529
3084.250000	0.002540
3084.000000	0.002549
3083.750000	0.002556
3083.500000	0.002561

3083.250000	0.002566
3083.000000	0.002572
3082.750000	0.002580
3082.500000	0.002589
3082.250000	0.002598
3082.000000	0.002605
3081.750000	0.002608
3081.500000	0.002607
3081.250000	0.002602
3081.000000	0.002592
3080.750000	0.002580
3080.500000	0.002569
3080.250000	0.002561
3080.000000	0.002558
3079.750000	0.002562
3079.500000	0.002573
3079.250000	0.002589
3079.000000	0.002607
3078.750000	0.002625
3078.500000	0.002639
3078.250000	0.002648
3078.000000	0.002651
3077.750000	0.002649
3077.500000	0.002643
3077.250000	0.002638
3077.000000	0.002634

3076.750000	0.002635
3076.500000	0.002640
3076.250000	0.002649
3076.000000	0.002661
3075.750000	0.002673
3075.500000	0.002684
3075.250000	0.002691
3075.000000	0.002693
3074.750000	0.002690
3074.500000	0.002685
3074.250000	0.002678
3074.000000	0.002673
3073.750000	0.002671
3073.500000	0.002673
3073.250000	0.002680
3073.000000	0.002691
3072.750000	0.002703
3072.500000	0.002715
3072.250000	0.002725
3072.000000	0.002731
3071.750000	0.002733
3071.500000	0.002731
3071.250000	0.002725
3071.000000	0.002718
3070.750000	0.002710
3070.500000	0.002703

3070.250000	0.002698
3070.000000	0.002695
3069.750000	0.002693
3069.500000	0.002693
3069.250000	0.002692
3069.000000	0.002692
3068.750000	0.002691
3068.500000	0.002690
3068.250000	0.002688
3068.000000	0.002685
3067.750000	0.002681
3067.500000	0.002675
3067.250000	0.002668
3067.000000	0.002661
3066.750000	0.002654
3066.500000	0.002650
3066.250000	0.002651
3066.000000	0.002659
3065.750000	0.002674
3065.500000	0.002695
3065.250000	0.002718
3065.000000	0.002739
3064.750000	0.002756
3064.500000	0.002765
3064.250000	0.002765
3064.000000	0.002760

3063.750000	0.002751
3063.500000	0.002742
3063.250000	0.002737
3063.000000	0.002736
3062.750000	0.002740
3062.500000	0.002747
3062.250000	0.002755
3062.000000	0.002761
3061.750000	0.002764
3061.500000	0.002763
3061.250000	0.002759
3061.000000	0.002752
3060.750000	0.002743
3060.500000	0.002736
3060.250000	0.002730
3060.000000	0.002728
3059.750000	0.002730
3059.500000	0.002735
3059.250000	0.002743
3059.000000	0.002752
3058.750000	0.002760
3058.500000	0.002767
3058.250000	0.002770
3058.000000	0.002771
3057.750000	0.002770
3057.500000	0.002767

3057.250000	0.002763
3057.000000	0.002758
3056.750000	0.002751
3056.500000	0.002742
3056.250000	0.002730
3056.000000	0.002717
3055.750000	0.002703
3055.500000	0.002691
3055.250000	0.002682
3055.000000	0.002678
3054.750000	0.002680
3054.500000	0.002686
3054.250000	0.002694
3054.000000	0.002703
3053.750000	0.002711
3053.500000	0.002715
3053.250000	0.002716
3053.000000	0.002714
3052.750000	0.002709
3052.500000	0.002703
3052.250000	0.002697
3052.000000	0.002691
3051.750000	0.002684
3051.500000	0.002677
3051.250000	0.002670
3051.000000	0.002663

3050.750000	0.002657
3050.500000	0.002653
3050.250000	0.002654
3050.000000	0.002658
3049.750000	0.002668
3049.500000	0.002681
3049.250000	0.002696
3049.000000	0.002709
3048.750000	0.002720
3048.500000	0.002726
3048.250000	0.002728
3048.000000	0.002726
3047.750000	0.002721
3047.500000	0.002717
3047.250000	0.002712
3047.000000	0.002708
3046.750000	0.002705
3046.500000	0.002702
3046.250000	0.002699
3046.000000	0.002695
3045.750000	0.002692
3045.500000	0.002688
3045.250000	0.002685
3045.000000	0.002683
3044.750000	0.002682
3044.500000	0.002682

3044.250000	0.002683
3044.000000	0.002684
3043.750000	0.002687
3043.500000	0.002692
3043.250000	0.002699
3043.000000	0.002708
3042.750000	0.002719
3042.500000	0.002730
3042.250000	0.002741
3042.000000	0.002747
3041.750000	0.002749
3041.500000	0.002744
3041.250000	0.002735
3041.000000	0.002722
3040.750000	0.002709
3040.500000	0.002699
3040.250000	0.002694
3040.000000	0.002696
3039.750000	0.002706
3039.500000	0.002721
3039.250000	0.002740
3039.000000	0.002757
3038.750000	0.002771
3038.500000	0.002780
3038.250000	0.002782
3038.000000	0.002779

3037.750000	0.002770
3037.500000	0.002760
3037.250000	0.002748
3037.000000	0.002738
3036.750000	0.002731
3036.500000	0.002728
3036.250000	0.002729
3036.000000	0.002733
3035.750000	0.002741
3035.500000	0.002750
3035.250000	0.002760
3035.000000	0.002768
3034.750000	0.002775
3034.500000	0.002779
3034.250000	0.002782
3034.000000	0.002784
3033.750000	0.002786
3033.500000	0.002789
3033.250000	0.002795
3033.000000	0.002803
3032.750000	0.002814
3032.500000	0.002827
3032.250000	0.002841
3032.000000	0.002853
3031.750000	0.002864
3031.500000	0.002872

3031.250000	0.002876
3031.000000	0.002876
3030.750000	0.002873
3030.500000	0.002868
3030.250000	0.002863
3030.000000	0.002860
3029.750000	0.002859
3029.500000	0.002861
3029.250000	0.002865
3029.000000	0.002871
3028.750000	0.002878
3028.500000	0.002885
3028.250000	0.002891
3028.000000	0.002896
3027.750000	0.002900
3027.500000	0.002902
3027.250000	0.002902
3027.000000	0.002899
3026.750000	0.002895
3026.500000	0.002890
3026.250000	0.002885
3026.000000	0.002881
3025.750000	0.002880
3025.500000	0.002883
3025.250000	0.002890
3025.000000	0.002900

3024.750000	0.002911
3024.500000	0.002923
3024.250000	0.002934
3024.000000	0.002945
3023.750000	0.002955
3023.500000	0.002965
3023.250000	0.002976
3023.000000	0.002986
3022.750000	0.002998
3022.500000	0.003010
3022.250000	0.003022
3022.000000	0.003035
3021.750000	0.003048
3021.500000	0.003061
3021.250000	0.003073
3021.000000	0.003083
3020.750000	0.003091
3020.500000	0.003096
3020.250000	0.003099
3020.000000	0.003101
3019.750000	0.003104
3019.500000	0.003108
3019.250000	0.003115
3019.000000	0.003125
3018.750000	0.003136
3018.500000	0.003148

3018.250000	0.003160
3018.000000	0.003172
3017.750000	0.003184
3017.500000	0.003197
3017.250000	0.003211
3017.000000	0.003226
3016.750000	0.003241
3016.500000	0.003254
3016.250000	0.003265
3016.000000	0.003272
3015.750000	0.003276
3015.500000	0.003276
3015.250000	0.003276
3015.000000	0.003277
3014.750000	0.003284
3014.500000	0.003296
3014.250000	0.003316
3014.000000	0.003342
3013.750000	0.003373
3013.500000	0.003406
3013.250000	0.003439
3013.000000	0.003469
3012.750000	0.003494
3012.500000	0.003513
3012.250000	0.003527
3012.000000	0.003536

3011.750000	0.003544
3011.500000	0.003552
3011.250000	0.003562
3011.000000	0.003576
3010.750000	0.003595
3010.500000	0.003620
3010.250000	0.003647
3010.000000	0.003677
3009.750000	0.003706
3009.500000	0.003732
3009.250000	0.003753
3009.000000	0.003769
3008.750000	0.003781
3008.500000	0.003789
3008.250000	0.003795
3008.000000	0.003803
3007.750000	0.003813
3007.500000	0.003827
3007.250000	0.003845
3007.000000	0.003866
3006.750000	0.003889
3006.500000	0.003914
3006.250000	0.003941
3006.000000	0.003969
3005.750000	0.003998
3005.500000	0.004026

3005.250000	0.004053
3005.000000	0.004077
3004.750000	0.004098
3004.500000	0.004115
3004.250000	0.004129
3004.000000	0.004141
3003.750000	0.004154
3003.500000	0.004168
3003.250000	0.004184
3003.000000	0.004202
3002.750000	0.004222
3002.500000	0.004244
3002.250000	0.004266
3002.000000	0.004291
3001.750000	0.004318
3001.500000	0.004348
3001.250000	0.004381
3001.000000	0.004416
3000.750000	0.004452
3000.500000	0.004486
3000.250000	0.004518
3000.000000	0.004546
2999.750000	0.004571
2999.500000	0.004593
2999.250000	0.004614
2999.000000	0.004634

2998.750000	0.004655
2998.500000	0.004676
2998.250000	0.004699
2998.000000	0.004723
2997.750000	0.004751
2997.500000	0.004781
2997.250000	0.004814
2997.000000	0.004850
2996.750000	0.004887
2996.500000	0.004924
2996.250000	0.004959
2996.000000	0.004991
2995.750000	0.005020
2995.500000	0.005044
2995.250000	0.005065
2995.000000	0.005083
2994.750000	0.005099
2994.500000	0.005115
2994.250000	0.005132
2994.000000	0.005152
2993.750000	0.005175
2993.500000	0.005202
2993.250000	0.005233
2993.000000	0.005267
2992.750000	0.005301
2992.500000	0.005336

2992.250000	0.005369
2992.000000	0.005402
2991.750000	0.005434
2991.500000	0.005465
2991.250000	0.005497
2991.000000	0.005528
2990.750000	0.005557
2990.500000	0.005584
2990.250000	0.005608
2990.000000	0.005629
2989.750000	0.005648
2989.500000	0.005666
2989.250000	0.005684
2989.000000	0.005703
2988.750000	0.005723
2988.500000	0.005743
2988.250000	0.005763
2988.000000	0.005782
2987.750000	0.005799
2987.500000	0.005816
2987.250000	0.005833
2987.000000	0.005851
2986.750000	0.005871
2986.500000	0.005893
2986.250000	0.005916
2986.000000	0.005942

2985.750000	0.005969
2985.500000	0.005999
2985.250000	0.006029
2985.000000	0.006060
2984.750000	0.006090
2984.500000	0.006119
2984.250000	0.006144
2984.000000	0.006166
2983.750000	0.006184
2983.500000	0.006197
2983.250000	0.006208
2983.000000	0.006215
2982.750000	0.006219
2982.500000	0.006221
2982.250000	0.006220
2982.000000	0.006218
2981.750000	0.006215
2981.500000	0.006214
2981.250000	0.006215
2981.000000	0.006221
2980.750000	0.006231
2980.500000	0.006245
2980.250000	0.006261
2980.000000	0.006279
2979.750000	0.006296
2979.500000	0.006312

2979.250000	0.006326
2979.000000	0.006338
2978.750000	0.006348
2978.500000	0.006356
2978.250000	0.006362
2978.000000	0.006368
2977.750000	0.006374
2977.500000	0.006380
2977.250000	0.006386
2977.000000	0.006393
2976.750000	0.006401
2976.500000	0.006410
2976.250000	0.006420
2976.000000	0.006431
2975.750000	0.006444
2975.500000	0.006460
2975.250000	0.006481
2975.000000	0.006507
2974.750000	0.006537
2974.500000	0.006568
2974.250000	0.006600
2974.000000	0.006628
2973.750000	0.006653
2973.500000	0.006672
2973.250000	0.006688
2973.000000	0.006703

2972.750000	0.006719
2972.500000	0.006739
2972.250000	0.006762
2972.000000	0.006791
2971.750000	0.006823
2971.500000	0.006857
2971.250000	0.006893
2971.000000	0.006930
2970.750000	0.006965
2970.500000	0.007000
2970.250000	0.007033
2970.000000	0.007063
2969.750000	0.007090
2969.500000	0.007113
2969.250000	0.007130
2969.000000	0.007143
2968.750000	0.007151
2968.500000	0.007156
2968.250000	0.007160
2968.000000	0.007163
2967.750000	0.007168
2967.500000	0.007174
2967.250000	0.007183
2967.000000	0.007193
2966.750000	0.007205
2966.500000	0.007218

2966.250000	0.007234
2966.000000	0.007252
2965.750000	0.007273
2965.500000	0.007298
2965.250000	0.007328
2965.000000	0.007362
2964.750000	0.007397
2964.500000	0.007433
2964.250000	0.007466
2964.000000	0.007494
2963.750000	0.007518
2963.500000	0.007538
2963.250000	0.007554
2963.000000	0.007568
2962.750000	0.007581
2962.500000	0.007594
2962.250000	0.007607
2962.000000	0.007621
2961.750000	0.007636
2961.500000	0.007653
2961.250000	0.007671
2961.000000	0.007693
2960.750000	0.007718
2960.500000	0.007746
2960.250000	0.007777
2960.000000	0.007808

2959.750000	0.007840
2959.500000	0.007869
2959.250000	0.007897
2959.000000	0.007921
2958.750000	0.007944
2958.500000	0.007964
2958.250000	0.007983
2958.000000	0.008000
2957.750000	0.008015
2957.500000	0.008029
2957.250000	0.008042
2957.000000	0.008055
2956.750000	0.008068
2956.500000	0.008083
2956.250000	0.008101
2956.000000	0.008121
2955.750000	0.008143
2955.500000	0.008165
2955.250000	0.008187
2955.000000	0.008207
2954.750000	0.008225
2954.500000	0.008240
2954.250000	0.008253
2954.000000	0.008265
2953.750000	0.008276
2953.500000	0.008289

2953.250000	0.008304
2953.000000	0.008323
2952.750000	0.008346
2952.500000	0.008373
2952.250000	0.008404
2952.000000	0.008437
2951.750000	0.008471
2951.500000	0.008503
2951.250000	0.008534
2951.000000	0.008562
2950.750000	0.008588
2950.500000	0.008613
2950.250000	0.008638
2950.000000	0.008665
2949.750000	0.008693
2949.500000	0.008724
2949.250000	0.008755
2949.000000	0.008786
2948.750000	0.008816
2948.500000	0.008845
2948.250000	0.008872
2948.000000	0.008900
2947.750000	0.008928
2947.500000	0.008959
2947.250000	0.008993
2947.000000	0.009031

2946.750000	0.009072
2946.500000	0.009116
2946.250000	0.009161
2946.000000	0.009207
2945.750000	0.009252
2945.500000	0.009296
2945.250000	0.009338
2945.000000	0.009377
2944.750000	0.009415
2944.500000	0.009451
2944.250000	0.009484
2944.000000	0.009517
2943.750000	0.009550
2943.500000	0.009584
2943.250000	0.009618
2943.000000	0.009654
2942.750000	0.009691
2942.500000	0.009730
2942.250000	0.009769
2942.000000	0.009810
2941.750000	0.009852
2941.500000	0.009895
2941.250000	0.009939
2941.000000	0.009982
2940.750000	0.010025
2940.500000	0.010066

2940.250000	0.010105
2940.000000	0.010143
2939.750000	0.010178
2939.500000	0.010211
2939.250000	0.010243
2939.000000	0.010273
2938.750000	0.010303
2938.500000	0.010332
2938.250000	0.010360
2938.000000	0.010388
2937.750000	0.010415
2937.500000	0.010440
2937.250000	0.010463
2937.000000	0.010483
2936.750000	0.010500
2936.500000	0.010514
2936.250000	0.010526
2936.000000	0.010537
2935.750000	0.010548
2935.500000	0.010559
2935.250000	0.010572
2935.000000	0.010587
2934.750000	0.010604
2934.500000	0.010623
2934.250000	0.010644
2934.000000	0.010666

2933.750000	0.010689
2933.500000	0.010713
2933.250000	0.010736
2933.000000	0.010757
2932.750000	0.010775
2932.500000	0.010788
2932.250000	0.010797
2932.000000	0.010800
2931.750000	0.010799
2931.500000	0.010794
2931.250000	0.010787
2931.000000	0.010779
2930.750000	0.010771
2930.500000	0.010765
2930.250000	0.010763
2930.000000	0.010764
2929.750000	0.010770
2929.500000	0.010780
2929.250000	0.010794
2929.000000	0.010808
2928.750000	0.010821
2928.500000	0.010830
2928.250000	0.010834
2928.000000	0.010835
2927.750000	0.010832
2927.500000	0.010827

2927.250000	0.010821
2927.000000	0.010814
2926.750000	0.010805
2926.500000	0.010794
2926.250000	0.010778
2926.000000	0.010759
2925.750000	0.010736
2925.500000	0.010712
2925.250000	0.010688
2925.000000	0.010667
2924.750000	0.010648
2924.500000	0.010631
2924.250000	0.010614
2924.000000	0.010595
2923.750000	0.010572
2923.500000	0.010546
2923.250000	0.010516
2923.000000	0.010484
2922.750000	0.010450
2922.500000	0.010414
2922.250000	0.010376
2922.000000	0.010336
2921.750000	0.010293
2921.500000	0.010248
2921.250000	0.010201
2921.000000	0.010151

2920.750000	0.010100
2920.500000	0.010046
2920.250000	0.009989
2920.000000	0.009928
2919.750000	0.009862
2919.500000	0.009792
2919.250000	0.009718
2919.000000	0.009642
2918.750000	0.009566
2918.500000	0.009490
2918.250000	0.009415
2918.000000	0.009341
2917.750000	0.009267
2917.500000	0.009192
2917.250000	0.009118
2917.000000	0.009043
2916.750000	0.008969
2916.500000	0.008896
2916.250000	0.008825
2916.000000	0.008755
2915.750000	0.008686
2915.500000	0.008617
2915.250000	0.008547
2915.000000	0.008477
2914.750000	0.008406
2914.500000	0.008335

2914.250000	0.008265
2914.000000	0.008196
2913.750000	0.008127
2913.500000	0.008059
2913.250000	0.007992
2913.000000	0.007924
2912.750000	0.007857
2912.500000	0.007792
2912.250000	0.007730
2912.000000	0.007672
2911.750000	0.007618
2911.500000	0.007567
2911.250000	0.007519
2911.000000	0.007471
2910.750000	0.007422
2910.500000	0.007372
2910.250000	0.007322
2910.000000	0.007271
2909.750000	0.007222
2909.500000	0.007175
2909.250000	0.007132
2909.000000	0.007093
2908.750000	0.007057
2908.500000	0.007024
2908.250000	0.006993
2908.000000	0.006964

2907.750000	0.006935
2907.500000	0.006905
2907.250000	0.006875
2907.000000	0.006843
2906.750000	0.006809
2906.500000	0.006776
2906.250000	0.006742
2906.000000	0.006710
2905.750000	0.006680
2905.500000	0.006653
2905.250000	0.006628
2905.000000	0.006604
2904.750000	0.006578
2904.500000	0.006550
2904.250000	0.006520
2904.000000	0.006489
2903.750000	0.006458
2903.500000	0.006431
2903.250000	0.006407
2903.000000	0.006389
2902.750000	0.006374
2902.500000	0.006361
2902.250000	0.006347
2902.000000	0.006332
2901.750000	0.006315
2901.500000	0.006294

2901.250000	0.006272
2901.000000	0.006248
2900.750000	0.006224
2900.500000	0.006200
2900.250000	0.006177
2900.000000	0.006154
2899.750000	0.006132
2899.500000	0.006111
2899.250000	0.006094
2899.000000	0.006079
2898.750000	0.006068
2898.500000	0.006058
2898.250000	0.006050
2898.000000	0.006042
2897.750000	0.006032
2897.500000	0.006021
2897.250000	0.006008
2897.000000	0.005993
2896.750000	0.005977
2896.500000	0.005959
2896.250000	0.005941
2896.000000	0.005922
2895.750000	0.005904
2895.500000	0.005887
2895.250000	0.005873
2895.000000	0.005862

2894.750000	0.005854
2894.500000	0.005850
2894.250000	0.005846
2894.000000	0.005843
2893.750000	0.005838
2893.500000	0.005829
2893.250000	0.005818
2893.000000	0.005804
2892.750000	0.005788
2892.500000	0.005772
2892.250000	0.005757
2892.000000	0.005744
2891.750000	0.005733
2891.500000	0.005726
2891.250000	0.005720
2891.000000	0.005715
2890.750000	0.005711
2890.500000	0.005706
2890.250000	0.005700
2890.000000	0.005693
2889.750000	0.005685
2889.500000	0.005677
2889.250000	0.005670
2889.000000	0.005665
2888.750000	0.005662
2888.500000	0.005659

2888.250000	0.005655
2888.000000	0.005650
2887.750000	0.005642
2887.500000	0.005632
2887.250000	0.005621
2887.000000	0.005609
2886.750000	0.005598
2886.500000	0.005590
2886.250000	0.005584
2886.000000	0.005581
2885.750000	0.005579
2885.500000	0.005578
2885.250000	0.005577
2885.000000	0.005576
2884.750000	0.005575
2884.500000	0.005575
2884.250000	0.005575
2884.000000	0.005577
2883.750000	0.005578
2883.500000	0.005579
2883.250000	0.005577
2883.000000	0.005574
2882.750000	0.005569
2882.500000	0.005562
2882.250000	0.005555
2882.000000	0.005549

2881.750000	0.005544
2881.500000	0.005541
2881.250000	0.005539
2881.000000	0.005538
2880.750000	0.005538
2880.500000	0.005539
2880.250000	0.005541
2880.000000	0.005544
2879.750000	0.005548
2879.500000	0.005555
2879.250000	0.005562
2879.000000	0.005570
2878.750000	0.005578
2878.500000	0.005585
2878.250000	0.005590
2878.000000	0.005594
2877.750000	0.005596
2877.500000	0.005598
2877.250000	0.005600
2877.000000	0.005602
2876.750000	0.005605
2876.500000	0.005610
2876.250000	0.005615
2876.000000	0.005623
2875.750000	0.005631
2875.500000	0.005640

2875.250000	0.005648
2875.000000	0.005655
2874.750000	0.005660
2874.500000	0.005663
2874.250000	0.005663
2874.000000	0.005662
2873.750000	0.005661
2873.500000	0.005662
2873.250000	0.005665
2873.000000	0.005672
2872.750000	0.005681
2872.500000	0.005692
2872.250000	0.005704
2872.000000	0.005717
2871.750000	0.005730
2871.500000	0.005743
2871.250000	0.005756
2871.000000	0.005766
2870.750000	0.005774
2870.500000	0.005778
2870.250000	0.005777
2870.000000	0.005772
2869.750000	0.005764
2869.500000	0.005756
2869.250000	0.005749
2869.000000	0.005745

2868.750000	0.005747
2868.500000	0.005753
2868.250000	0.005763
2868.000000	0.005773
2867.750000	0.005783
2867.500000	0.005791
2867.250000	0.005797
2867.000000	0.005801
2866.750000	0.005805
2866.500000	0.005809
2866.250000	0.005814
2866.000000	0.005822
2865.750000	0.005830
2865.500000	0.005838
2865.250000	0.005845
2865.000000	0.005851
2864.750000	0.005854
2864.500000	0.005856
2864.250000	0.005856
2864.000000	0.005857
2863.750000	0.005859
2863.500000	0.005864
2863.250000	0.005871
2863.000000	0.005880
2862.750000	0.005891
2862.500000	0.005901

2862.250000	0.005910
2862.000000	0.005918
2861.750000	0.005924
2861.500000	0.005929
2861.250000	0.005936
2861.000000	0.005943
2860.750000	0.005952
2860.500000	0.005961
2860.250000	0.005969
2860.000000	0.005974
2859.750000	0.005975
2859.500000	0.005973
2859.250000	0.005969
2859.000000	0.005965
2858.750000	0.005964
2858.500000	0.005965
2858.250000	0.005970
2858.000000	0.005975
2857.750000	0.005980
2857.500000	0.005981
2857.250000	0.005979
2857.000000	0.005973
2856.750000	0.005964
2856.500000	0.005954
2856.250000	0.005944
2856.000000	0.005935

2855.750000	0.005925
2855.500000	0.005914
2855.250000	0.005901
2855.000000	0.005884
2854.750000	0.005865
2854.500000	0.005845
2854.250000	0.005824
2854.000000	0.005804
2853.750000	0.005786
2853.500000	0.005770
2853.250000	0.005754
2853.000000	0.005739
2852.750000	0.005723
2852.500000	0.005707
2852.250000	0.005690
2852.000000	0.005673
2851.750000	0.005655
2851.500000	0.005636
2851.250000	0.005615
2851.000000	0.005591
2850.750000	0.005562
2850.500000	0.005527
2850.250000	0.005486
2850.000000	0.005439
2849.750000	0.005387
2849.500000	0.005329

2849.250000	0.005266
2849.000000	0.005200
2848.750000	0.005131
2848.500000	0.005060
2848.250000	0.004987
2848.000000	0.004914
2847.750000	0.004841
2847.500000	0.004769
2847.250000	0.004698
2847.000000	0.004627
2846.750000	0.004557
2846.500000	0.004487
2846.250000	0.004416
2846.000000	0.004344
2845.750000	0.004271
2845.500000	0.004198
2845.250000	0.004126
2845.000000	0.004054
2844.750000	0.003985
2844.500000	0.003919
2844.250000	0.003855
2844.000000	0.003794
2843.750000	0.003734
2843.500000	0.003677
2843.250000	0.003621
2843.000000	0.003567

2842.750000	0.003515
2842.500000	0.003466
2842.250000	0.003421
2842.000000	0.003379
2841.750000	0.003342
2841.500000	0.003308
2841.250000	0.003278
2841.000000	0.003249
2840.750000	0.003222
2840.500000	0.003194
2840.250000	0.003164
2840.000000	0.003133
2839.750000	0.003099
2839.500000	0.003063
2839.250000	0.003026
2839.000000	0.002989
2838.750000	0.002954
2838.500000	0.002922
2838.250000	0.002894
2838.000000	0.002870
2837.750000	0.002850
2837.500000	0.002833
2837.250000	0.002818
2837.000000	0.002805
2836.750000	0.002792
2836.500000	0.002779

2836.250000	0.002768
2836.000000	0.002757
2835.750000	0.002746
2835.500000	0.002736
2835.250000	0.002725
2835.000000	0.002713
2834.750000	0.002699
2834.500000	0.002684
2834.250000	0.002668
2834.000000	0.002652
2833.750000	0.002637
2833.500000	0.002623
2833.250000	0.002611
2833.000000	0.002601
2832.750000	0.002592
2832.500000	0.002585
2832.250000	0.002579
2832.000000	0.002575
2831.750000	0.002572
2831.500000	0.002570
2831.250000	0.002569
2831.000000	0.002569
2830.750000	0.002568
2830.500000	0.002565
2830.250000	0.002561
2830.000000	0.002554

2829.750000	0.002546
2829.500000	0.002535
2829.250000	0.002524
2829.000000	0.002512
2828.750000	0.002501
2828.500000	0.002492
2828.250000	0.002486
2828.000000	0.002483
2827.750000	0.002481
2827.500000	0.002481
2827.250000	0.002480
2827.000000	0.002478
2826.750000	0.002474
2826.500000	0.002468
2826.250000	0.002460
2826.000000	0.002450
2825.750000	0.002439
2825.500000	0.002429
2825.250000	0.002418
2825.000000	0.002407
2824.750000	0.002396
2824.500000	0.002385
2824.250000	0.002375
2824.000000	0.002366
2823.750000	0.002359
2823.500000	0.002355

2823.250000	0.002353
2823.000000	0.002354
2822.750000	0.002356
2822.500000	0.002358
2822.250000	0.002360
2822.000000	0.002361
2821.750000	0.002360
2821.500000	0.002357
2821.250000	0.002350
2821.000000	0.002340
2820.750000	0.002327
2820.500000	0.002311
2820.250000	0.002294
2820.000000	0.002278
2819.750000	0.002265
2819.500000	0.002256
2819.250000	0.002252
2819.000000	0.002252
2818.750000	0.002254
2818.500000	0.002258
2818.250000	0.002261
2818.000000	0.002264
2817.750000	0.002265
2817.500000	0.002266
2817.250000	0.002265
2817.000000	0.002263

2816.750000	0.002259
2816.500000	0.002254
2816.250000	0.002247
2816.000000	0.002240
2815.750000	0.002233
2815.500000	0.002227
2815.250000	0.002225
2815.000000	0.002224
2814.750000	0.002223
2814.500000	0.002221
2814.250000	0.002216
2814.000000	0.002207
2813.750000	0.002196
2813.500000	0.002183
2813.250000	0.002172
2813.000000	0.002164
2812.750000	0.002161
2812.500000	0.002160
2812.250000	0.002162
2812.000000	0.002164
2811.750000	0.002164
2811.500000	0.002162
2811.250000	0.002158
2811.000000	0.002152
2810.750000	0.002145
2810.500000	0.002139

2810.250000	0.002133
2810.000000	0.002128
2809.750000	0.002123
2809.500000	0.002117
2809.250000	0.002112
2809.000000	0.002107
2808.750000	0.002102
2808.500000	0.002097
2808.250000	0.002093
2808.000000	0.002089
2807.750000	0.002084
2807.500000	0.002080
2807.250000	0.002076
2807.000000	0.002073
2806.750000	0.002071
2806.500000	0.002072
2806.250000	0.002074
2806.000000	0.002077
2805.750000	0.002080
2805.500000	0.002082
2805.250000	0.002083
2805.000000	0.002082
2804.750000	0.002079
2804.500000	0.002075
2804.250000	0.002070
2804.000000	0.002065

2803.750000	0.002062
2803.500000	0.002059
2803.250000	0.002059
2803.000000	0.002060
2802.750000	0.002061
2802.500000	0.002063
2802.250000	0.002063
2802.000000	0.002062
2801.750000	0.002058
2801.500000	0.002051
2801.250000	0.002041
2801.000000	0.002028
2800.750000	0.002014
2800.500000	0.002000
2800.250000	0.001987
2800.000000	0.001976
2799.750000	0.001968
2799.500000	0.001965
2799.250000	0.001965
2799.000000	0.001969
2798.750000	0.001976
2798.500000	0.001984
2798.250000	0.001992
2798.000000	0.002000
2797.750000	0.002007
2797.500000	0.002013

2797.250000	0.002018
2797.000000	0.002020
2796.750000	0.002021
2796.500000	0.002019
2796.250000	0.002016
2796.000000	0.002010
2795.750000	0.002002
2795.500000	0.001995
2795.250000	0.001988
2795.000000	0.001982
2794.750000	0.001978
2794.500000	0.001975
2794.250000	0.001974
2794.000000	0.001973
2793.750000	0.001972
2793.500000	0.001970
2793.250000	0.001968
2793.000000	0.001965
2792.750000	0.001963
2792.500000	0.001960
2792.250000	0.001956
2792.000000	0.001953
2791.750000	0.001949
2791.500000	0.001944
2791.250000	0.001940
2791.000000	0.001936

2790.750000	0.001932
2790.500000	0.001928
2790.250000	0.001925
2790.000000	0.001921
2789.750000	0.001918
2789.500000	0.001915
2789.250000	0.001913
2789.000000	0.001912
2788.750000	0.001914
2788.500000	0.001916
2788.250000	0.001918
2788.000000	0.001921
2787.750000	0.001922
2787.500000	0.001922
2787.250000	0.001920
2787.000000	0.001918
2786.750000	0.001914
2786.500000	0.001911
2786.250000	0.001908
2786.000000	0.001905
2785.750000	0.001905
2785.500000	0.001906
2785.250000	0.001909
2785.000000	0.001914
2784.750000	0.001920
2784.500000	0.001926

2784.250000	0.001932
2784.000000	0.001936
2783.750000	0.001938
2783.500000	0.001938
2783.250000	0.001936
2783.000000	0.001932
2782.750000	0.001929
2782.500000	0.001925
2782.250000	0.001923
2782.000000	0.001920
2781.750000	0.001918
2781.500000	0.001917
2781.250000	0.001915
2781.000000	0.001914
2780.750000	0.001912
2780.500000	0.001910
2780.250000	0.001907
2780.000000	0.001902
2779.750000	0.001897
2779.500000	0.001890
2779.250000	0.001883
2779.000000	0.001877
2778.750000	0.001873
2778.500000	0.001871
2778.250000	0.001870
2778.000000	0.001869

2777.750000	0.001869
2777.500000	0.001868
2777.250000	0.001866
2777.000000	0.001865
2776.750000	0.001865
2776.500000	0.001867
2776.250000	0.001872
2776.000000	0.001879
2775.750000	0.001887
2775.500000	0.001895
2775.250000	0.001901
2775.000000	0.001904
2774.750000	0.001903
2774.500000	0.001901
2774.250000	0.001896
2774.000000	0.001891
2773.750000	0.001887
2773.500000	0.001883
2773.250000	0.001880
2773.000000	0.001878
2772.750000	0.001875
2772.500000	0.001871
2772.250000	0.001865
2772.000000	0.001858
2771.750000	0.001850
2771.500000	0.001843

2771.250000	0.001838
2771.000000	0.001836
2770.750000	0.001838
2770.500000	0.001842
2770.250000	0.001848
2770.000000	0.001853
2769.750000	0.001856
2769.500000	0.001857
2769.250000	0.001854
2769.000000	0.001849
2768.750000	0.001843
2768.500000	0.001836
2768.250000	0.001830
2768.000000	0.001824
2767.750000	0.001820
2767.500000	0.001818
2767.250000	0.001816
2767.000000	0.001816
2766.750000	0.001816
2766.500000	0.001818
2766.250000	0.001820
2766.000000	0.001823
2765.750000	0.001826
2765.500000	0.001830
2765.250000	0.001835
2765.000000	0.001841

2764.750000	0.001847
2764.500000	0.001852
2764.250000	0.001857
2764.000000	0.001859
2763.750000	0.001858
2763.500000	0.001854
2763.250000	0.001847
2763.000000	0.001839
2762.750000	0.001832
2762.500000	0.001827
2762.250000	0.001824
2762.000000	0.001823
2761.750000	0.001824
2761.500000	0.001825
2761.250000	0.001825
2761.000000	0.001824
2760.750000	0.001822
2760.500000	0.001819
2760.250000	0.001817
2760.000000	0.001814
2759.750000	0.001813
2759.500000	0.001812
2759.250000	0.001811
2759.000000	0.001810
2758.750000	0.001809
2758.500000	0.001807

2758.250000	0.001804
2758.000000	0.001800
2757.750000	0.001796
2757.500000	0.001792
2757.250000	0.001788
2757.000000	0.001784
2756.750000	0.001781
2756.500000	0.001779
2756.250000	0.001778
2756.000000	0.001778
2755.750000	0.001779
2755.500000	0.001779
2755.250000	0.001779
2755.000000	0.001778
2754.750000	0.001777
2754.500000	0.001776
2754.250000	0.001777
2754.000000	0.001779
2753.750000	0.001782
2753.500000	0.001786
2753.250000	0.001791
2753.000000	0.001794
2752.750000	0.001796
2752.500000	0.001795
2752.250000	0.001792
2752.000000	0.001787

2751.750000	0.001780
2751.500000	0.001773
2751.250000	0.001767
2751.000000	0.001763
2750.750000	0.001762
2750.500000	0.001763
2750.250000	0.001767
2750.000000	0.001771
2749.750000	0.001774
2749.500000	0.001775
2749.250000	0.001773
2749.000000	0.001768
2748.750000	0.001762
2748.500000	0.001756
2748.250000	0.001751
2748.000000	0.001749
2747.750000	0.001749
2747.500000	0.001752
2747.250000	0.001757
2747.000000	0.001763
2746.750000	0.001769
2746.500000	0.001775
2746.250000	0.001781
2746.000000	0.001786
2745.750000	0.001789
2745.500000	0.001790

2745.250000	0.001788
2745.000000	0.001782
2744.750000	0.001773
2744.500000	0.001762
2744.250000	0.001750
2744.000000	0.001740
2743.750000	0.001733
2743.500000	0.001729
2743.250000	0.001729
2743.000000	0.001732
2742.750000	0.001737
2742.500000	0.001745
2742.250000	0.001753
2742.000000	0.001762
2741.750000	0.001771
2741.500000	0.001778
2741.250000	0.001783
2741.000000	0.001785
2740.750000	0.001784
2740.500000	0.001779
2740.250000	0.001772
2740.000000	0.001762
2739.750000	0.001753
2739.500000	0.001744
2739.250000	0.001736
2739.000000	0.001731

2738.750000	0.001726
2738.500000	0.001723
2738.250000	0.001721
2738.000000	0.001721
2737.750000	0.001722
2737.500000	0.001726
2737.250000	0.001732
2737.000000	0.001739
2736.750000	0.001747
2736.500000	0.001753
2736.250000	0.001756
2736.000000	0.001754
2735.750000	0.001747
2735.500000	0.001737
2735.250000	0.001724
2735.000000	0.001711
2734.750000	0.001700
2734.500000	0.001692
2734.250000	0.001686
2734.000000	0.001683
2733.750000	0.001681
2733.500000	0.001679
2733.250000	0.001678
2733.000000	0.001677
2732.750000	0.001677
2732.500000	0.001678

2732.250000	0.001681
2732.000000	0.001686
2731.750000	0.001692
2731.500000	0.001697
2731.250000	0.001700
2731.000000	0.001701
2730.750000	0.001699
2730.500000	0.001694
2730.250000	0.001687
2730.000000	0.001680
2729.750000	0.001672
2729.500000	0.001667
2729.250000	0.001663
2729.000000	0.001662
2728.750000	0.001662
2728.500000	0.001664
2728.250000	0.001666
2728.000000	0.001667
2727.750000	0.001665
2727.500000	0.001660
2727.250000	0.001652
2727.000000	0.001642
2726.750000	0.001632
2726.500000	0.001623
2726.250000	0.001618
2726.000000	0.001617

2725.750000	0.001620
2725.500000	0.001627
2725.250000	0.001636
2725.000000	0.001644
2724.750000	0.001651
2724.500000	0.001655
2724.250000	0.001655
2724.000000	0.001651
2723.750000	0.001645
2723.500000	0.001636
2723.250000	0.001627
2723.000000	0.001618
2722.750000	0.001612
2722.500000	0.001608
2722.250000	0.001608
2722.000000	0.001611
2721.750000	0.001617
2721.500000	0.001623
2721.250000	0.001629
2721.000000	0.001634
2720.750000	0.001636
2720.500000	0.001635
2720.250000	0.001633
2720.000000	0.001630
2719.750000	0.001627
2719.500000	0.001625

2719.250000	0.001624
2719.000000	0.001623
2718.750000	0.001622
2718.500000	0.001621
2718.250000	0.001618
2718.000000	0.001615
2717.750000	0.001611
2717.500000	0.001606
2717.250000	0.001601
2717.000000	0.001597
2716.750000	0.001594
2716.500000	0.001592
2716.250000	0.001591
2716.000000	0.001591
2715.750000	0.001593
2715.500000	0.001595
2715.250000	0.001597
2715.000000	0.001598
2714.750000	0.001599
2714.500000	0.001598
2714.250000	0.001597
2714.000000	0.001597
2713.750000	0.001597
2713.500000	0.001600
2713.250000	0.001603
2713.000000	0.001608

2712.750000	0.001613
2712.500000	0.001616
2712.250000	0.001617
2712.000000	0.001614
2711.750000	0.001608
2711.500000	0.001600
2711.250000	0.001590
2711.000000	0.001581
2710.750000	0.001573
2710.500000	0.001567
2710.250000	0.001563
2710.000000	0.001562
2709.750000	0.001563
2709.500000	0.001565
2709.250000	0.001568
2709.000000	0.001571
2708.750000	0.001574
2708.500000	0.001578
2708.250000	0.001580
2708.000000	0.001581
2707.750000	0.001580
2707.500000	0.001578
2707.250000	0.001575
2707.000000	0.001571
2706.750000	0.001567
2706.500000	0.001563

2706.250000	0.001559
2706.000000	0.001556
2705.750000	0.001552
2705.500000	0.001548
2705.250000	0.001543
2705.000000	0.001538
2704.750000	0.001534
2704.500000	0.001531
2704.250000	0.001532
2704.000000	0.001535
2703.750000	0.001542
2703.500000	0.001552
2703.250000	0.001562
2703.000000	0.001573
2702.750000	0.001581
2702.500000	0.001586
2702.250000	0.001587
2702.000000	0.001584
2701.750000	0.001577
2701.500000	0.001569
2701.250000	0.001561
2701.000000	0.001554
2700.750000	0.001549
2700.500000	0.001549
2700.250000	0.001552
2700.000000	0.001558

2699.750000	0.001565
2699.500000	0.001571
2699.250000	0.001575
2699.000000	0.001574
2698.750000	0.001570
2698.500000	0.001561
2698.250000	0.001551
2698.000000	0.001541
2697.750000	0.001532
2697.500000	0.001527
2697.250000	0.001525
2697.000000	0.001526
2696.750000	0.001529
2696.500000	0.001534
2696.250000	0.001538
2696.000000	0.001542
2695.750000	0.001544
2695.500000	0.001544
2695.250000	0.001542
2695.000000	0.001538
2694.750000	0.001531
2694.500000	0.001524
2694.250000	0.001518
2694.000000	0.001512
2693.750000	0.001510
2693.500000	0.001511

2693.250000	0.001515
2693.000000	0.001522
2692.750000	0.001529
2692.500000	0.001535
2692.250000	0.001539
2692.000000	0.001542
2691.750000	0.001542
2691.500000	0.001541
2691.250000	0.001540
2691.000000	0.001541
2690.750000	0.001542
2690.500000	0.001544
2690.250000	0.001546
2690.000000	0.001548
2689.750000	0.001548
2689.500000	0.001547
2689.250000	0.001544
2689.000000	0.001539
2688.750000	0.001534
2688.500000	0.001529
2688.250000	0.001523
2688.000000	0.001517
2687.750000	0.001511
2687.500000	0.001505
2687.250000	0.001499
2687.000000	0.001494

2686.750000	0.001489
2686.500000	0.001486
2686.250000	0.001486
2686.000000	0.001487
2685.750000	0.001491
2685.500000	0.001498
2685.250000	0.001505
2685.000000	0.001513
2684.750000	0.001520
2684.500000	0.001526
2684.250000	0.001529
2684.000000	0.001530
2683.750000	0.001527
2683.500000	0.001523
2683.250000	0.001516
2683.000000	0.001508
2682.750000	0.001500
2682.500000	0.001492
2682.250000	0.001485
2682.000000	0.001480
2681.750000	0.001476
2681.500000	0.001474
2681.250000	0.001475
2681.000000	0.001477
2680.750000	0.001480
2680.500000	0.001483

2680.250000	0.001485
2680.000000	0.001485
2679.750000	0.001484
2679.500000	0.001482
2679.250000	0.001481
2679.000000	0.001480
2678.750000	0.001480
2678.500000	0.001480
2678.250000	0.001479
2678.000000	0.001477
2677.750000	0.001473
2677.500000	0.001469
2677.250000	0.001465
2677.000000	0.001463
2676.750000	0.001462
2676.500000	0.001464
2676.250000	0.001467
2676.000000	0.001471
2675.750000	0.001473
2675.500000	0.001472
2675.250000	0.001469
2675.000000	0.001464
2674.750000	0.001457
2674.500000	0.001451
2674.250000	0.001446
2674.000000	0.001442

2673.750000	0.001439
2673.500000	0.001438
2673.250000	0.001437
2673.000000	0.001439
2672.750000	0.001442
2672.500000	0.001446
2672.250000	0.001453
2672.000000	0.001460
2671.750000	0.001468
2671.500000	0.001475
2671.250000	0.001481
2671.000000	0.001483
2670.750000	0.001483
2670.500000	0.001479
2670.250000	0.001473
2670.000000	0.001465
2669.750000	0.001457
2669.500000	0.001451
2669.250000	0.001447
2669.000000	0.001447
2668.750000	0.001450
2668.500000	0.001455
2668.250000	0.001460
2668.000000	0.001465
2667.750000	0.001468
2667.500000	0.001469

2667.250000	0.001468
2667.000000	0.001466
2666.750000	0.001465
2666.500000	0.001465
2666.250000	0.001466
2666.000000	0.001469
2665.750000	0.001472
2665.500000	0.001474
2665.250000	0.001476
2665.000000	0.001479
2664.750000	0.001481
2664.500000	0.001484
2664.250000	0.001488
2664.000000	0.001493
2663.750000	0.001498
2663.500000	0.001504
2663.250000	0.001508
2663.000000	0.001513
2662.750000	0.001516
2662.500000	0.001519
2662.250000	0.001520
2662.000000	0.001521
2661.750000	0.001522
2661.500000	0.001522
2661.250000	0.001522
2661.000000	0.001523

2660.750000	0.001525
2660.500000	0.001528
2660.250000	0.001532
2660.000000	0.001536
2659.750000	0.001538
2659.500000	0.001538
2659.250000	0.001536
2659.000000	0.001531
2658.750000	0.001525
2658.500000	0.001520
2658.250000	0.001517
2658.000000	0.001517
2657.750000	0.001520
2657.500000	0.001524
2657.250000	0.001530
2657.000000	0.001536
2656.750000	0.001541
2656.500000	0.001543
2656.250000	0.001544
2656.000000	0.001543
2655.750000	0.001541
2655.500000	0.001537
2655.250000	0.001532
2655.000000	0.001527
2654.750000	0.001522
2654.500000	0.001519

2654.250000	0.001517
2654.000000	0.001518
2653.750000	0.001521
2653.500000	0.001525
2653.250000	0.001531
2653.000000	0.001538
2652.750000	0.001545
2652.500000	0.001552
2652.250000	0.001558
2652.000000	0.001564
2651.750000	0.001568
2651.500000	0.001569
2651.250000	0.001569
2651.000000	0.001565
2650.750000	0.001561
2650.500000	0.001555
2650.250000	0.001548
2650.000000	0.001543
2649.750000	0.001539
2649.500000	0.001535
2649.250000	0.001532
2649.000000	0.001528
2648.750000	0.001523
2648.500000	0.001517
2648.250000	0.001511
2648.000000	0.001504

2647.750000	0.001499
2647.500000	0.001496
2647.250000	0.001495
2647.000000	0.001497
2646.750000	0.001501
2646.500000	0.001505
2646.250000	0.001511
2646.000000	0.001515
2645.750000	0.001519
2645.500000	0.001522
2645.250000	0.001523
2645.000000	0.001523
2644.750000	0.001522
2644.500000	0.001520
2644.250000	0.001518
2644.000000	0.001514
2643.750000	0.001511
2643.500000	0.001507
2643.250000	0.001503
2643.000000	0.001500
2642.750000	0.001496
2642.500000	0.001492
2642.250000	0.001488
2642.000000	0.001484
2641.750000	0.001481
2641.500000	0.001478

2641.250000	0.001476
2641.000000	0.001475
2640.750000	0.001475
2640.500000	0.001475
2640.250000	0.001474
2640.000000	0.001473
2639.750000	0.001470
2639.500000	0.001466
2639.250000	0.001461
2639.000000	0.001455
2638.750000	0.001449
2638.500000	0.001442
2638.250000	0.001436
2638.000000	0.001431
2637.750000	0.001427
2637.500000	0.001424
2637.250000	0.001423
2637.000000	0.001424
2636.750000	0.001428
2636.500000	0.001433
2636.250000	0.001441
2636.000000	0.001449
2635.750000	0.001456
2635.500000	0.001462
2635.250000	0.001466
2635.000000	0.001467

2634.750000	0.001466
2634.500000	0.001462
2634.250000	0.001457
2634.000000	0.001453
2633.750000	0.001450
2633.500000	0.001450
2633.250000	0.001452
2633.000000	0.001457
2632.750000	0.001463
2632.500000	0.001469
2632.250000	0.001475
2632.000000	0.001477
2631.750000	0.001477
2631.500000	0.001473
2631.250000	0.001466
2631.000000	0.001457
2630.750000	0.001447
2630.500000	0.001438
2630.250000	0.001432
2630.000000	0.001428
2629.750000	0.001428
2629.500000	0.001432
2629.250000	0.001437
2629.000000	0.001443
2628.750000	0.001447
2628.500000	0.001447

2628.250000	0.001442
2628.000000	0.001432
2627.750000	0.001418
2627.500000	0.001402
2627.250000	0.001387
2627.000000	0.001376
2626.750000	0.001369
2626.500000	0.001366
2626.250000	0.001368
2626.000000	0.001373
2625.750000	0.001379
2625.500000	0.001385
2625.250000	0.001392
2625.000000	0.001398
2624.750000	0.001403
2624.500000	0.001408
2624.250000	0.001411
2624.000000	0.001413
2623.750000	0.001412
2623.500000	0.001409
2623.250000	0.001405
2623.000000	0.001402
2622.750000	0.001402
2622.500000	0.001404
2622.250000	0.001408
2622.000000	0.001413

2621.750000	0.001417
2621.500000	0.001418
2621.250000	0.001415
2621.000000	0.001409
2620.750000	0.001399
2620.500000	0.001387
2620.250000	0.001376
2620.000000	0.001365
2619.750000	0.001355
2619.500000	0.001348
2619.250000	0.001344
2619.000000	0.001344
2618.750000	0.001348
2618.500000	0.001358
2618.250000	0.001372
2618.000000	0.001389
2617.750000	0.001403
2617.500000	0.001414
2617.250000	0.001417
2617.000000	0.001413
2616.750000	0.001403
2616.500000	0.001389
2616.250000	0.001374
2616.000000	0.001362
2615.750000	0.001356
2615.500000	0.001354

2615.250000	0.001357
2615.000000	0.001362
2614.750000	0.001368
2614.500000	0.001372
2614.250000	0.001373
2614.000000	0.001372
2613.750000	0.001367
2613.500000	0.001361
2613.250000	0.001356
2613.000000	0.001352
2612.750000	0.001353
2612.500000	0.001360
2612.250000	0.001371
2612.000000	0.001386
2611.750000	0.001402
2611.500000	0.001416
2611.250000	0.001425
2611.000000	0.001428
2610.750000	0.001425
2610.500000	0.001416
2610.250000	0.001403
2610.000000	0.001387
2609.750000	0.001370
2609.500000	0.001352
2609.250000	0.001333
2609.000000	0.001314

2608.750000	0.001296
2608.500000	0.001281
2608.250000	0.001271
2608.000000	0.001268
2607.750000	0.001270
2607.500000	0.001278
2607.250000	0.001289
2607.000000	0.001301
2606.750000	0.001310
2606.500000	0.001317
2606.250000	0.001321
2606.000000	0.001322
2605.750000	0.001323
2605.500000	0.001325
2605.250000	0.001327
2605.000000	0.001329
2604.750000	0.001332
2604.500000	0.001333
2604.250000	0.001333
2604.000000	0.001330
2603.750000	0.001325
2603.500000	0.001318
2603.250000	0.001309
2603.000000	0.001299
2602.750000	0.001289
2602.500000	0.001281

2602.250000	0.001274
2602.000000	0.001269
2601.750000	0.001268
2601.500000	0.001268
2601.250000	0.001269
2601.000000	0.001271
2600.750000	0.001272
2600.500000	0.001272
2600.250000	0.001272
2600.000000	0.001273
2599.750000	0.001275
2599.500000	0.001279
2599.250000	0.001284
2599.000000	0.001289
2598.750000	0.001293
2598.500000	0.001296
2598.250000	0.001295
2598.000000	0.001292
2597.750000	0.001287
2597.500000	0.001280
2597.250000	0.001272
2597.000000	0.001265
2596.750000	0.001259
2596.500000	0.001255
2596.250000	0.001254
2596.000000	0.001256

2595.750000	0.001261
2595.500000	0.001268
2595.250000	0.001276
2595.000000	0.001284
2594.750000	0.001289
2594.500000	0.001292
2594.250000	0.001292
2594.000000	0.001289
2593.750000	0.001285
2593.500000	0.001282
2593.250000	0.001279
2593.000000	0.001278
2592.750000	0.001278
2592.500000	0.001280
2592.250000	0.001283
2592.000000	0.001288
2591.750000	0.001294
2591.500000	0.001304
2591.250000	0.001317
2591.000000	0.001333
2590.750000	0.001351
2590.500000	0.001369
2590.250000	0.001384
2590.000000	0.001392
2589.750000	0.001392
2589.500000	0.001384

2589.250000	0.001368
2589.000000	0.001348
2588.750000	0.001328
2588.500000	0.001310
2588.250000	0.001298
2588.000000	0.001291
2587.750000	0.001289
2587.500000	0.001291
2587.250000	0.001294
2587.000000	0.001296
2586.750000	0.001297
2586.500000	0.001297
2586.250000	0.001295
2586.000000	0.001290
2585.750000	0.001283
2585.500000	0.001274
2585.250000	0.001261
2585.000000	0.001247
2584.750000	0.001234
2584.500000	0.001225
2584.250000	0.001221
2584.000000	0.001225
2583.750000	0.001236
2583.500000	0.001252
2583.250000	0.001269
2583.000000	0.001285

2582.750000	0.001296
2582.500000	0.001303
2582.250000	0.001305
2582.000000	0.001304
2581.750000	0.001302
2581.500000	0.001300
2581.250000	0.001298
2581.000000	0.001295
2580.750000	0.001292
2580.500000	0.001289
2580.250000	0.001287
2580.000000	0.001285
2579.750000	0.001284
2579.500000	0.001286
2579.250000	0.001288
2579.000000	0.001290
2578.750000	0.001291
2578.500000	0.001288
2578.250000	0.001283
2578.000000	0.001274
2577.750000	0.001264
2577.500000	0.001252
2577.250000	0.001239
2577.000000	0.001226
2576.750000	0.001214
2576.500000	0.001203

2576.250000	0.001197
2576.000000	0.001195
2575.750000	0.001201
2575.500000	0.001213
2575.250000	0.001231
2575.000000	0.001250
2574.750000	0.001268
2574.500000	0.001281
2574.250000	0.001287
2574.000000	0.001286
2573.750000	0.001280
2573.500000	0.001273
2573.250000	0.001266
2573.000000	0.001262
2572.750000	0.001260
2572.500000	0.001260
2572.250000	0.001260
2572.000000	0.001258
2571.750000	0.001254
2571.500000	0.001247
2571.250000	0.001239
2571.000000	0.001230
2570.750000	0.001224
2570.500000	0.001220
2570.250000	0.001220
2570.000000	0.001224

2569.750000	0.001231
2569.500000	0.001241
2569.250000	0.001251
2569.000000	0.001260
2568.750000	0.001267
2568.500000	0.001270
2568.250000	0.001267
2568.000000	0.001260
2567.750000	0.001248
2567.500000	0.001234
2567.250000	0.001218
2567.000000	0.001205
2566.750000	0.001194
2566.500000	0.001188
2566.250000	0.001186
2566.000000	0.001188
2565.750000	0.001193
2565.500000	0.001200
2565.250000	0.001205
2565.000000	0.001210
2564.750000	0.001213
2564.500000	0.001215
2564.250000	0.001216
2564.000000	0.001217
2563.750000	0.001217
2563.500000	0.001219

2563.250000	0.001220
2563.000000	0.001220
2562.750000	0.001220
2562.500000	0.001219
2562.250000	0.001218
2562.000000	0.001219
2561.750000	0.001222
2561.500000	0.001229
2561.250000	0.001239
2561.000000	0.001249
2560.750000	0.001259
2560.500000	0.001264
2560.250000	0.001264
2560.000000	0.001259
2559.750000	0.001251
2559.500000	0.001241
2559.250000	0.001233
2559.000000	0.001230
2558.750000	0.001231
2558.500000	0.001237
2558.250000	0.001244
2558.000000	0.001252
2557.750000	0.001258
2557.500000	0.001260
2557.250000	0.001258
2557.000000	0.001253

2556.750000	0.001246
2556.500000	0.001238
2556.250000	0.001229
2556.000000	0.001221
2555.750000	0.001213
2555.500000	0.001206
2555.250000	0.001200
2555.000000	0.001196
2554.750000	0.001194
2554.500000	0.001195
2554.250000	0.001199
2554.000000	0.001205
2553.750000	0.001212
2553.500000	0.001217
2553.250000	0.001219
2553.000000	0.001216
2552.750000	0.001207
2552.500000	0.001195
2552.250000	0.001180
2552.000000	0.001165
2551.750000	0.001155
2551.500000	0.001150
2551.250000	0.001153
2551.000000	0.001162
2550.750000	0.001176
2550.500000	0.001192

2550.250000	0.001206
2550.000000	0.001219
2549.750000	0.001227
2549.500000	0.001233
2549.250000	0.001237
2549.000000	0.001240
2548.750000	0.001241
2548.500000	0.001240
2548.250000	0.001237
2548.000000	0.001232
2547.750000	0.001224
2547.500000	0.001216
2547.250000	0.001208
2547.000000	0.001202
2546.750000	0.001199
2546.500000	0.001199
2546.250000	0.001198
2546.000000	0.001197
2545.750000	0.001194
2545.500000	0.001188
2545.250000	0.001180
2545.000000	0.001170
2544.750000	0.001160
2544.500000	0.001152
2544.250000	0.001144
2544.000000	0.001138

2543.750000	0.001133
2543.500000	0.001129
2543.250000	0.001125
2543.000000	0.001124
2542.750000	0.001123
2542.500000	0.001123
2542.250000	0.001122
2542.000000	0.001120
2541.750000	0.001115
2541.500000	0.001107
2541.250000	0.001098
2541.000000	0.001090
2540.750000	0.001084
2540.500000	0.001083
2540.250000	0.001087
2540.000000	0.001096
2539.750000	0.001109
2539.500000	0.001122
2539.250000	0.001136
2539.000000	0.001147
2538.750000	0.001156
2538.500000	0.001163
2538.250000	0.001168
2538.000000	0.001173
2537.750000	0.001178
2537.500000	0.001184

2537.250000	0.001190
2537.000000	0.001196
2536.750000	0.001202
2536.500000	0.001208
2536.250000	0.001213
2536.000000	0.001216
2535.750000	0.001217
2535.500000	0.001217
2535.250000	0.001216
2535.000000	0.001213
2534.750000	0.001210
2534.500000	0.001206
2534.250000	0.001199
2534.000000	0.001190
2533.750000	0.001178
2533.500000	0.001165
2533.250000	0.001152
2533.000000	0.001142
2532.750000	0.001138
2532.500000	0.001142
2532.250000	0.001153
2532.000000	0.001170
2531.750000	0.001190
2531.500000	0.001207
2531.250000	0.001220
2531.000000	0.001225

2530.750000	0.001222
2530.500000	0.001214
2530.250000	0.001204
2530.000000	0.001193
2529.750000	0.001185
2529.500000	0.001181
2529.250000	0.001182
2529.000000	0.001186
2528.750000	0.001193
2528.500000	0.001200
2528.250000	0.001207
2528.000000	0.001210
2527.750000	0.001210
2527.500000	0.001206
2527.250000	0.001197
2527.000000	0.001185
2526.750000	0.001171
2526.500000	0.001160
2526.250000	0.001153
2526.000000	0.001153
2525.750000	0.001159
2525.500000	0.001170
2525.250000	0.001184
2525.000000	0.001198
2524.750000	0.001209
2524.500000	0.001214

2524.250000	0.001214
2524.000000	0.001209
2523.750000	0.001201
2523.500000	0.001192
2523.250000	0.001185
2523.000000	0.001179
2522.750000	0.001176
2522.500000	0.001173
2522.250000	0.001171
2522.000000	0.001167
2521.750000	0.001161
2521.500000	0.001153
2521.250000	0.001145
2521.000000	0.001138
2520.750000	0.001136
2520.500000	0.001138
2520.250000	0.001144
2520.000000	0.001153
2519.750000	0.001162
2519.500000	0.001166
2519.250000	0.001165
2519.000000	0.001156
2518.750000	0.001142
2518.500000	0.001125
2518.250000	0.001109
2518.000000	0.001096

2517.750000	0.001088
2517.500000	0.001086
2517.250000	0.001089
2517.000000	0.001096
2516.750000	0.001104
2516.500000	0.001113
2516.250000	0.001123
2516.000000	0.001134
2515.750000	0.001146
2515.500000	0.001158
2515.250000	0.001169
2515.000000	0.001178
2514.750000	0.001183
2514.500000	0.001183
2514.250000	0.001178
2514.000000	0.001170
2513.750000	0.001161
2513.500000	0.001153
2513.250000	0.001147
2513.000000	0.001146
2512.750000	0.001148
2512.500000	0.001153
2512.250000	0.001160
2512.000000	0.001167
2511.750000	0.001172
2511.500000	0.001174

2511.250000	0.001172
2511.000000	0.001165
2510.750000	0.001155
2510.500000	0.001143
2510.250000	0.001132
2510.000000	0.001127
2509.750000	0.001128
2509.500000	0.001137
2509.250000	0.001152
2509.000000	0.001171
2508.750000	0.001187
2508.500000	0.001197
2508.250000	0.001198
2508.000000	0.001187
2507.750000	0.001168
2507.500000	0.001144
2507.250000	0.001120
2507.000000	0.001101
2506.750000	0.001091
2506.500000	0.001090
2506.250000	0.001098
2506.000000	0.001113
2505.750000	0.001130
2505.500000	0.001146
2505.250000	0.001159
2505.000000	0.001168

2504.750000	0.001173
2504.500000	0.001175
2504.250000	0.001176
2504.000000	0.001176
2503.750000	0.001178
2503.500000	0.001182
2503.250000	0.001187
2503.000000	0.001192
2502.750000	0.001195
2502.500000	0.001197
2502.250000	0.001195
2502.000000	0.001190
2501.750000	0.001183
2501.500000	0.001174
2501.250000	0.001166
2501.000000	0.001160
2500.750000	0.001157
2500.500000	0.001158
2500.250000	0.001163
2500.000000	0.001170
2499.750000	0.001179
2499.500000	0.001187
2499.250000	0.001194
2499.000000	0.001199
2498.750000	0.001200
2498.500000	0.001198

2498.250000	0.001193
2498.000000	0.001185
2497.750000	0.001176
2497.500000	0.001167
2497.250000	0.001159
2497.000000	0.001152
2496.750000	0.001148
2496.500000	0.001146
2496.250000	0.001145
2496.000000	0.001145
2495.750000	0.001146
2495.500000	0.001147
2495.250000	0.001148
2495.000000	0.001150
2494.750000	0.001153
2494.500000	0.001157
2494.250000	0.001159
2494.000000	0.001160
2493.750000	0.001157
2493.500000	0.001152
2493.250000	0.001144
2493.000000	0.001135
2492.750000	0.001128
2492.500000	0.001123
2492.250000	0.001122
2492.000000	0.001125

2491.750000	0.001132
2491.500000	0.001140
2491.250000	0.001148
2491.000000	0.001156
2490.750000	0.001162
2490.500000	0.001167
2490.250000	0.001172
2490.000000	0.001177
2489.750000	0.001181
2489.500000	0.001185
2489.250000	0.001188
2489.000000	0.001188
2488.750000	0.001184
2488.500000	0.001176
2488.250000	0.001164
2488.000000	0.001150
2487.750000	0.001135
2487.500000	0.001120
2487.250000	0.001107
2487.000000	0.001098
2486.750000	0.001092
2486.500000	0.001090
2486.250000	0.001092
2486.000000	0.001098
2485.750000	0.001108
2485.500000	0.001121

2485.250000	0.001136
2485.000000	0.001152
2484.750000	0.001168
2484.500000	0.001181
2484.250000	0.001190
2484.000000	0.001195
2483.750000	0.001196
2483.500000	0.001192
2483.250000	0.001184
2483.000000	0.001174
2482.750000	0.001161
2482.500000	0.001146
2482.250000	0.001131
2482.000000	0.001117
2481.750000	0.001104
2481.500000	0.001095
2481.250000	0.001089
2481.000000	0.001089
2480.750000	0.001093
2480.500000	0.001101
2480.250000	0.001112
2480.000000	0.001123
2479.750000	0.001132
2479.500000	0.001138
2479.250000	0.001140
2479.000000	0.001139

2478.750000	0.001136
2478.500000	0.001132
2478.250000	0.001131
2478.000000	0.001132
2477.750000	0.001138
2477.500000	0.001147
2477.250000	0.001158
2477.000000	0.001170
2476.750000	0.001179
2476.500000	0.001184
2476.250000	0.001183
2476.000000	0.001177
2475.750000	0.001165
2475.500000	0.001151
2475.250000	0.001136
2475.000000	0.001123
2474.750000	0.001112
2474.500000	0.001104
2474.250000	0.001098
2474.000000	0.001093
2473.750000	0.001087
2473.500000	0.001081
2473.250000	0.001075
2473.000000	0.001069
2472.750000	0.001064
2472.500000	0.001061

2472.250000	0.001060
2472.000000	0.001061
2471.750000	0.001064
2471.500000	0.001068
2471.250000	0.001073
2471.000000	0.001080
2470.750000	0.001090
2470.500000	0.001102
2470.250000	0.001115
2470.000000	0.001129
2469.750000	0.001141
2469.500000	0.001151
2469.250000	0.001156
2469.000000	0.001156
2468.750000	0.001151
2468.500000	0.001143
2468.250000	0.001134
2468.000000	0.001125
2467.750000	0.001119
2467.500000	0.001117
2467.250000	0.001119
2467.000000	0.001124
2466.750000	0.001131
2466.500000	0.001139
2466.250000	0.001145
2466.000000	0.001147

2465.750000	0.001144
2465.500000	0.001136
2465.250000	0.001123
2465.000000	0.001106
2464.750000	0.001087
2464.500000	0.001069
2464.250000	0.001054
2464.000000	0.001045
2463.750000	0.001043
2463.500000	0.001050
2463.250000	0.001065
2463.000000	0.001084
2462.750000	0.001106
2462.500000	0.001126
2462.250000	0.001142
2462.000000	0.001152
2461.750000	0.001156
2461.500000	0.001155
2461.250000	0.001151
2461.000000	0.001146
2460.750000	0.001143
2460.500000	0.001141
2460.250000	0.001142
2460.000000	0.001144
2459.750000	0.001148
2459.500000	0.001152

2459.250000	0.001156
2459.000000	0.001159
2458.750000	0.001161
2458.500000	0.001162
2458.250000	0.001160
2458.000000	0.001157
2457.750000	0.001154
2457.500000	0.001150
2457.250000	0.001148
2457.000000	0.001148
2456.750000	0.001151
2456.500000	0.001156
2456.250000	0.001163
2456.000000	0.001169
2455.750000	0.001173
2455.500000	0.001175
2455.250000	0.001175
2455.000000	0.001173
2454.750000	0.001170
2454.500000	0.001169
2454.250000	0.001168
2454.000000	0.001169
2453.750000	0.001172
2453.500000	0.001176
2453.250000	0.001181
2453.000000	0.001186

2452.750000	0.001190
2452.500000	0.001193
2452.250000	0.001193
2452.000000	0.001189
2451.750000	0.001182
2451.500000	0.001170
2451.250000	0.001157
2451.000000	0.001143
2450.750000	0.001131
2450.500000	0.001123
2450.250000	0.001119
2450.000000	0.001117
2449.750000	0.001117
2449.500000	0.001116
2449.250000	0.001114
2449.000000	0.001110
2448.750000	0.001107
2448.500000	0.001106
2448.250000	0.001109
2448.000000	0.001114
2447.750000	0.001123
2447.500000	0.001131
2447.250000	0.001137
2447.000000	0.001139
2446.750000	0.001136
2446.500000	0.001129

2446.250000	0.001119
2446.000000	0.001110
2445.750000	0.001104
2445.500000	0.001101
2445.250000	0.001101
2445.000000	0.001103
2444.750000	0.001105
2444.500000	0.001104
2444.250000	0.001099
2444.000000	0.001090
2443.750000	0.001078
2443.500000	0.001065
2443.250000	0.001053
2443.000000	0.001045
2442.750000	0.001042
2442.500000	0.001044
2442.250000	0.001050
2442.000000	0.001058
2441.750000	0.001065
2441.500000	0.001071
2441.250000	0.001073
2441.000000	0.001071
2440.750000	0.001067
2440.500000	0.001063
2440.250000	0.001060
2440.000000	0.001062

2439.750000	0.001068
2439.500000	0.001079
2439.250000	0.001092
2439.000000	0.001107
2438.750000	0.001121
2438.500000	0.001133
2438.250000	0.001142
2438.000000	0.001149
2437.750000	0.001153
2437.500000	0.001156
2437.250000	0.001157
2437.000000	0.001158
2436.750000	0.001157
2436.500000	0.001154
2436.250000	0.001151
2436.000000	0.001146
2435.750000	0.001143
2435.500000	0.001140
2435.250000	0.001139
2435.000000	0.001140
2434.750000	0.001140
2434.500000	0.001140
2434.250000	0.001136
2434.000000	0.001129
2433.750000	0.001119
2433.500000	0.001108

2433.250000	0.001097
2433.000000	0.001088
2432.750000	0.001083
2432.500000	0.001080
2432.250000	0.001080
2432.000000	0.001081
2431.750000	0.001083
2431.500000	0.001085
2431.250000	0.001088
2431.000000	0.001091
2430.750000	0.001095
2430.500000	0.001100
2430.250000	0.001105
2430.000000	0.001109
2429.750000	0.001110
2429.500000	0.001108
2429.250000	0.001103
2429.000000	0.001096
2428.750000	0.001088
2428.500000	0.001080
2428.250000	0.001071
2428.000000	0.001064
2427.750000	0.001058
2427.500000	0.001054
2427.250000	0.001052
2427.000000	0.001053

2426.750000	0.001055
2426.500000	0.001056
2426.250000	0.001055
2426.000000	0.001052
2425.750000	0.001044
2425.500000	0.001034
2425.250000	0.001023
2425.000000	0.001014
2424.750000	0.001011
2424.500000	0.001013
2424.250000	0.001020
2424.000000	0.001029
2423.750000	0.001039
2423.500000	0.001045
2423.250000	0.001046
2423.000000	0.001043
2422.750000	0.001037
2422.500000	0.001029
2422.250000	0.001020
2422.000000	0.001012
2421.750000	0.001005
2421.500000	0.000998
2421.250000	0.000993
2421.000000	0.000988
2420.750000	0.000985
2420.500000	0.000984

2420.250000	0.000986
2420.000000	0.000989
2419.750000	0.000992
2419.500000	0.000996
2419.250000	0.001000
2419.000000	0.001004
2418.750000	0.001010
2418.500000	0.001017
2418.250000	0.001024
2418.000000	0.001031
2417.750000	0.001037
2417.500000	0.001038
2417.250000	0.001036
2417.000000	0.001031
2416.750000	0.001025
2416.500000	0.001022
2416.250000	0.001024
2416.000000	0.001031
2415.750000	0.001041
2415.500000	0.001053
2415.250000	0.001062
2415.000000	0.001068
2414.750000	0.001068
2414.500000	0.001065
2414.250000	0.001060
2414.000000	0.001054

2413.750000	0.001050
2413.500000	0.001048
2413.250000	0.001047
2413.000000	0.001045
2412.750000	0.001041
2412.500000	0.001036
2412.250000	0.001030
2412.000000	0.001024
2411.750000	0.001019
2411.500000	0.001016
2411.250000	0.001017
2411.000000	0.001019
2410.750000	0.001024
2410.500000	0.001029
2410.250000	0.001034
2410.000000	0.001039
2409.750000	0.001044
2409.500000	0.001049
2409.250000	0.001053
2409.000000	0.001056
2408.750000	0.001057
2408.500000	0.001054
2408.250000	0.001047
2408.000000	0.001036
2407.750000	0.001020
2407.500000	0.001002

2407.250000	0.000983
2407.000000	0.000966
2406.750000	0.000952
2406.500000	0.000943
2406.250000	0.000938
2406.000000	0.000937
2405.750000	0.000938
2405.500000	0.000942
2405.250000	0.000946
2405.000000	0.000951
2404.750000	0.000957
2404.500000	0.000964
2404.250000	0.000971
2404.000000	0.000979
2403.750000	0.000986
2403.500000	0.000991
2403.250000	0.000996
2403.000000	0.000999
2402.750000	0.001002
2402.500000	0.001005
2402.250000	0.001009
2402.000000	0.001012
2401.750000	0.001014
2401.500000	0.001014
2401.250000	0.001011
2401.000000	0.001007

2400.750000	0.001001
2400.500000	0.000995
2400.250000	0.000991
2400.000000	0.000990
2399.750000	0.000992
2399.500000	0.000996
2399.250000	0.001002
2399.000000	0.001009
2398.750000	0.001017
2398.500000	0.001024
2398.250000	0.001030
2398.000000	0.001034
2397.750000	0.001034
2397.500000	0.001030
2397.250000	0.001021
2397.000000	0.001007
2396.750000	0.000992
2396.500000	0.000976
2396.250000	0.000963
2396.000000	0.000954
2395.750000	0.000952
2395.500000	0.000955
2395.250000	0.000961
2395.000000	0.000970
2394.750000	0.000979
2394.500000	0.000987

2394.250000	0.000994
2394.000000	0.001001
2393.750000	0.001007
2393.500000	0.001015
2393.250000	0.001022
2393.000000	0.001030
2392.750000	0.001036
2392.500000	0.001038
2392.250000	0.001035
2392.000000	0.001027
2391.750000	0.001013
2391.500000	0.000995
2391.250000	0.000977
2391.000000	0.000962
2390.750000	0.000953
2390.500000	0.000951
2390.250000	0.000956
2390.000000	0.000966
2389.750000	0.000977
2389.500000	0.000989
2389.250000	0.000998
2389.000000	0.001004
2388.750000	0.001007
2388.500000	0.001008
2388.250000	0.001007
2388.000000	0.001006

2387.750000	0.001003
2387.500000	0.000998
2387.250000	0.000990
2387.000000	0.000980
2386.750000	0.000968
2386.500000	0.000955
2386.250000	0.000943
2386.000000	0.000934
2385.750000	0.000929
2385.500000	0.000927
2385.250000	0.000928
2385.000000	0.000932
2384.750000	0.000936
2384.500000	0.000939
2384.250000	0.000940
2384.000000	0.000937
2383.750000	0.000930
2383.500000	0.000918
2383.250000	0.000904
2383.000000	0.000889
2382.750000	0.000877
2382.500000	0.000870
2382.250000	0.000869
2382.000000	0.000874
2381.750000	0.000881
2381.500000	0.000888

2381.250000	0.000890
2381.000000	0.000885
2380.750000	0.000873
2380.500000	0.000856
2380.250000	0.000836
2380.000000	0.000816
2379.750000	0.000799
2379.500000	0.000784
2379.250000	0.000772
2379.000000	0.000763
2378.750000	0.000756
2378.500000	0.000751
2378.250000	0.000750
2378.000000	0.000754
2377.750000	0.000762
2377.500000	0.000774
2377.250000	0.000790
2377.000000	0.000808
2376.750000	0.000824
2376.500000	0.000837
2376.250000	0.000846
2376.000000	0.000849
2375.750000	0.000846
2375.500000	0.000836
2375.250000	0.000820
2375.000000	0.000802

2374.750000	0.000784
2374.500000	0.000767
2374.250000	0.000754
2374.000000	0.000747
2373.750000	0.000745
2373.500000	0.000746
2373.250000	0.000748
2373.000000	0.000747
2372.750000	0.000744
2372.500000	0.000740
2372.250000	0.000736
2372.000000	0.000735
2371.750000	0.000738
2371.500000	0.000745
2371.250000	0.000757
2371.000000	0.000768
2370.750000	0.000777
2370.500000	0.000781
2370.250000	0.000780
2370.000000	0.000776
2369.750000	0.000769
2369.500000	0.000760
2369.250000	0.000751
2369.000000	0.000742
2368.750000	0.000736
2368.500000	0.000733

2368.250000	0.000733
2368.000000	0.000737
2367.750000	0.000745
2367.500000	0.000757
2367.250000	0.000771
2367.000000	0.000782
2366.750000	0.000786
2366.500000	0.000783
2366.250000	0.000774
2366.000000	0.000760
2365.750000	0.000743
2365.500000	0.000722
2365.250000	0.000704
2365.000000	0.000691
2364.750000	0.000686
2364.500000	0.000686
2364.250000	0.000687
2364.000000	0.000688
2363.750000	0.000688
2363.500000	0.000686
2363.250000	0.000680
2363.000000	0.000669
2362.750000	0.000659
2362.500000	0.000655
2362.250000	0.000662
2362.000000	0.000681

2361.750000	0.000710
2361.500000	0.000743
2361.250000	0.000776
2361.000000	0.000803
2360.750000	0.000820
2360.500000	0.000826
2360.250000	0.000824
2360.000000	0.000816
2359.750000	0.000807
2359.500000	0.000799
2359.250000	0.000797
2359.000000	0.000799
2358.750000	0.000800
2358.500000	0.000796
2358.250000	0.000784
2358.000000	0.000765
2357.750000	0.000744
2357.500000	0.000724
2357.250000	0.000708
2357.000000	0.000697
2356.750000	0.000695
2356.500000	0.000706
2356.250000	0.000731
2356.000000	0.000766
2355.750000	0.000803
2355.500000	0.000834

2355.250000	0.000857
2355.000000	0.000875
2354.750000	0.000889
2354.500000	0.000899
2354.250000	0.000902
2354.000000	0.000899
2353.750000	0.000891
2353.500000	0.000883
2353.250000	0.000875
2353.000000	0.000867
2352.750000	0.000862
2352.500000	0.000862
2352.250000	0.000869
2352.000000	0.000884
2351.750000	0.000904
2351.500000	0.000926
2351.250000	0.000947
2351.000000	0.000965
2350.750000	0.000976
2350.500000	0.000977
2350.250000	0.000967
2350.000000	0.000948
2349.750000	0.000927
2349.500000	0.000910
2349.250000	0.000899
2349.000000	0.000894

2348.750000	0.000890
2348.500000	0.000884
2348.250000	0.000873
2348.000000	0.000860
2347.750000	0.000845
2347.500000	0.000830
2347.250000	0.000814
2347.000000	0.000799
2346.750000	0.000787
2346.500000	0.000782
2346.250000	0.000787
2346.000000	0.000801
2345.750000	0.000823
2345.500000	0.000847
2345.250000	0.000869
2345.000000	0.000890
2344.750000	0.000908
2344.500000	0.000926
2344.250000	0.000940
2344.000000	0.000948
2343.750000	0.000948
2343.500000	0.000938
2343.250000	0.000925
2343.000000	0.000913
2342.750000	0.000908
2342.500000	0.000914

2342.250000	0.000928
2342.000000	0.000943
2341.750000	0.000954
2341.500000	0.000957
2341.250000	0.000954
2341.000000	0.000948
2340.750000	0.000941
2340.500000	0.000934
2340.250000	0.000927
2340.000000	0.000920
2339.750000	0.000919
2339.500000	0.000927
2339.250000	0.000947
2339.000000	0.000974
2338.750000	0.001003
2338.500000	0.001027
2338.250000	0.001044
2338.000000	0.001053
2337.750000	0.001059
2337.500000	0.001062
2337.250000	0.001061
2337.000000	0.001053
2336.750000	0.001034
2336.500000	0.001006
2336.250000	0.000975
2336.000000	0.000950

2335.750000	0.000938
2335.500000	0.000941
2335.250000	0.000955
2335.000000	0.000972
2334.750000	0.000988
2334.500000	0.001000
2334.250000	0.001008
2334.000000	0.001014
2333.750000	0.001020
2333.500000	0.001023
2333.250000	0.001022
2333.000000	0.001016
2332.750000	0.001003
2332.500000	0.000985
2332.250000	0.000965
2332.000000	0.000947
2331.750000	0.000935
2331.500000	0.000931
2331.250000	0.000935
2331.000000	0.000945
2330.750000	0.000959
2330.500000	0.000974
2330.250000	0.000991
2330.000000	0.001009
2329.750000	0.001028
2329.500000	0.001046

2329.250000	0.001061
2329.000000	0.001068
2328.750000	0.001068
2328.500000	0.001062
2328.250000	0.001057
2328.000000	0.001058
2327.750000	0.001068
2327.500000	0.001088
2327.250000	0.001113
2327.000000	0.001141
2326.750000	0.001172
2326.500000	0.001207
2326.250000	0.001249
2326.000000	0.001300
2325.750000	0.001353
2325.500000	0.001402
2325.250000	0.001437
2325.000000	0.001453
2324.750000	0.001452
2324.500000	0.001438
2324.250000	0.001421
2324.000000	0.001405
2323.750000	0.001390
2323.500000	0.001376
2323.250000	0.001358
2323.000000	0.001339

2322.750000	0.001321
2322.500000	0.001312
2322.250000	0.001314
2322.000000	0.001324
2321.750000	0.001336
2321.500000	0.001341
2321.250000	0.001333
2321.000000	0.001313
2320.750000	0.001288
2320.500000	0.001266
2320.250000	0.001253
2320.000000	0.001248
2319.750000	0.001245
2319.500000	0.001236
2319.250000	0.001217
2319.000000	0.001189
2318.750000	0.001159
2318.500000	0.001138
2318.250000	0.001131
2318.000000	0.001139
2317.750000	0.001156
2317.500000	0.001176
2317.250000	0.001192
2317.000000	0.001203
2316.750000	0.001214
2316.500000	0.001228

2316.250000	0.001245
2316.000000	0.001264
2315.750000	0.001275
2315.500000	0.001273
2315.250000	0.001255
2315.000000	0.001226
2314.750000	0.001196
2314.500000	0.001174
2314.250000	0.001166
2314.000000	0.001171
2313.750000	0.001183
2313.500000	0.001192
2313.250000	0.001193
2313.000000	0.001186
2312.750000	0.001176
2312.500000	0.001166
2312.250000	0.001161
2312.000000	0.001159
2311.750000	0.001155
2311.500000	0.001143
2311.250000	0.001121
2311.000000	0.001095
2310.750000	0.001071
2310.500000	0.001054
2310.250000	0.001050
2310.000000	0.001063

2309.750000	0.001083
2309.500000	0.001090
2309.250000	0.001085
2309.000000	0.001082
2308.750000	0.001080
2308.500000	0.001055
2308.250000	0.001028
2308.000000	0.001062
2307.750000	0.001173
2307.500000	0.001288
2307.250000	0.001345
2307.000000	0.001363
2306.750000	0.001376
2306.500000	0.001357
2306.250000	0.001272
2306.000000	0.001165
2305.750000	0.001111
2305.500000	0.001121
2305.250000	0.001146
2305.000000	0.001151
2304.750000	0.001141
2304.500000	0.001131
2304.250000	0.001125
2304.000000	0.001124
2303.750000	0.001126
2303.500000	0.001132

2303.250000	0.001139
2303.000000	0.001144
2302.750000	0.001143
2302.500000	0.001137
2302.250000	0.001130
2302.000000	0.001127
2301.750000	0.001131
2301.500000	0.001141
2301.250000	0.001154
2301.000000	0.001167
2300.750000	0.001176
2300.500000	0.001179
2300.250000	0.001177
2300.000000	0.001173
2299.750000	0.001170
2299.500000	0.001172
2299.250000	0.001177
2299.000000	0.001183
2298.750000	0.001188
2298.500000	0.001188
2298.250000	0.001183
2298.000000	0.001178
2297.750000	0.001177
2297.500000	0.001184
2297.250000	0.001201
2297.000000	0.001228

2296.750000	0.001259
2296.500000	0.001290
2296.250000	0.001313
2296.000000	0.001326
2295.750000	0.001328
2295.500000	0.001321
2295.250000	0.001311
2295.000000	0.001299
2294.750000	0.001290
2294.500000	0.001283
2294.250000	0.001278
2294.000000	0.001277
2293.750000	0.001280
2293.500000	0.001287
2293.250000	0.001299
2293.000000	0.001314
2292.750000	0.001331
2292.500000	0.001345
2292.250000	0.001354
2292.000000	0.001354
2291.750000	0.001347
2291.500000	0.001337
2291.250000	0.001327
2291.000000	0.001323
2290.750000	0.001328
2290.500000	0.001341

2290.250000	0.001359
2290.000000	0.001379
2289.750000	0.001397
2289.500000	0.001409
2289.250000	0.001414
2289.000000	0.001415
2288.750000	0.001414
2288.500000	0.001412
2288.250000	0.001410
2288.000000	0.001410
2287.750000	0.001411
2287.500000	0.001413
2287.250000	0.001414
2287.000000	0.001415
2286.750000	0.001415
2286.500000	0.001416
2286.250000	0.001418
2286.000000	0.001420
2285.750000	0.001425
2285.500000	0.001431
2285.250000	0.001440
2285.000000	0.001450
2284.750000	0.001460
2284.500000	0.001466
2284.250000	0.001467
2284.000000	0.001459

2283.750000	0.001443
2283.500000	0.001421
2283.250000	0.001395
2283.000000	0.001371
2282.750000	0.001352
2282.500000	0.001341
2282.250000	0.001338
2282.000000	0.001341
2281.750000	0.001347
2281.500000	0.001354
2281.250000	0.001358
2281.000000	0.001361
2280.750000	0.001362
2280.500000	0.001362
2280.250000	0.001361
2280.000000	0.001359
2279.750000	0.001355
2279.500000	0.001347
2279.250000	0.001335
2279.000000	0.001322
2278.750000	0.001310
2278.500000	0.001303
2278.250000	0.001302
2278.000000	0.001311
2277.750000	0.001327
2277.500000	0.001350

2277.250000	0.001375
2277.000000	0.001399
2276.750000	0.001418
2276.500000	0.001429
2276.250000	0.001430
2276.000000	0.001422
2275.750000	0.001406
2275.500000	0.001382
2275.250000	0.001354
2275.000000	0.001323
2274.750000	0.001292
2274.500000	0.001263
2274.250000	0.001239
2274.000000	0.001221
2273.750000	0.001213
2273.500000	0.001213
2273.250000	0.001221
2273.000000	0.001234
2272.750000	0.001250
2272.500000	0.001266
2272.250000	0.001280
2272.000000	0.001292
2271.750000	0.001301
2271.500000	0.001308
2271.250000	0.001313
2271.000000	0.001315

2270.750000	0.001315
2270.500000	0.001309
2270.250000	0.001298
2270.000000	0.001283
2269.750000	0.001265
2269.500000	0.001246
2269.250000	0.001231
2269.000000	0.001222
2268.750000	0.001219
2268.500000	0.001223
2268.250000	0.001230
2268.000000	0.001238
2267.750000	0.001246
2267.500000	0.001254
2267.250000	0.001261
2267.000000	0.001271
2266.750000	0.001282
2266.500000	0.001294
2266.250000	0.001304
2266.000000	0.001309
2265.750000	0.001307
2265.500000	0.001298
2265.250000	0.001285
2265.000000	0.001270
2264.750000	0.001257
2264.500000	0.001249

2264.250000	0.001245
2264.000000	0.001243
2263.750000	0.001241
2263.500000	0.001237
2263.250000	0.001229
2263.000000	0.001218
2262.750000	0.001206
2262.500000	0.001193
2262.250000	0.001184
2262.000000	0.001178
2261.750000	0.001177
2261.500000	0.001180
2261.250000	0.001189
2261.000000	0.001203
2260.750000	0.001224
2260.500000	0.001249
2260.250000	0.001275
2260.000000	0.001298
2259.750000	0.001315
2259.500000	0.001322
2259.250000	0.001319
2259.000000	0.001310
2258.750000	0.001298
2258.500000	0.001289
2258.250000	0.001284
2258.000000	0.001284

2257.750000	0.001286
2257.500000	0.001287
2257.250000	0.001283
2257.000000	0.001274
2256.750000	0.001259
2256.500000	0.001243
2256.250000	0.001227
2256.000000	0.001216
2255.750000	0.001210
2255.500000	0.001207
2255.250000	0.001207
2255.000000	0.001206
2254.750000	0.001201
2254.500000	0.001193
2254.250000	0.001182
2254.000000	0.001170
2253.750000	0.001160
2253.500000	0.001155
2253.250000	0.001157
2253.000000	0.001167
2252.750000	0.001183
2252.500000	0.001205
2252.250000	0.001227
2252.000000	0.001247
2251.750000	0.001260
2251.500000	0.001265

2251.250000	0.001262
2251.000000	0.001253
2250.750000	0.001241
2250.500000	0.001229
2250.250000	0.001218
2250.000000	0.001212
2249.750000	0.001209
2249.500000	0.001211
2249.250000	0.001216
2249.000000	0.001223
2248.750000	0.001231
2248.500000	0.001239
2248.250000	0.001245
2248.000000	0.001247
2247.750000	0.001243
2247.500000	0.001235
2247.250000	0.001221
2247.000000	0.001206
2246.750000	0.001191
2246.500000	0.001180
2246.250000	0.001177
2246.000000	0.001182
2245.750000	0.001196
2245.500000	0.001218
2245.250000	0.001244
2245.000000	0.001271

2244.750000	0.001296
2244.500000	0.001315
2244.250000	0.001327
2244.000000	0.001331
2243.750000	0.001327
2243.500000	0.001320
2243.250000	0.001311
2243.000000	0.001305
2242.750000	0.001304
2242.500000	0.001308
2242.250000	0.001319
2242.000000	0.001332
2241.750000	0.001345
2241.500000	0.001353
2241.250000	0.001356
2241.000000	0.001351
2240.750000	0.001338
2240.500000	0.001321
2240.250000	0.001303
2240.000000	0.001285
2239.750000	0.001271
2239.500000	0.001262
2239.250000	0.001257
2239.000000	0.001255
2238.750000	0.001253
2238.500000	0.001249

2238.250000	0.001241
2238.000000	0.001231
2237.750000	0.001219
2237.500000	0.001208
2237.250000	0.001202
2237.000000	0.001201
2236.750000	0.001207
2236.500000	0.001219
2236.250000	0.001232
2236.000000	0.001244
2235.750000	0.001252
2235.500000	0.001254
2235.250000	0.001251
2235.000000	0.001244
2234.750000	0.001237
2234.500000	0.001230
2234.250000	0.001228
2234.000000	0.001230
2233.750000	0.001238
2233.500000	0.001250
2233.250000	0.001265
2233.000000	0.001283
2232.750000	0.001302
2232.500000	0.001320
2232.250000	0.001334
2232.000000	0.001342

2231.750000	0.001343
2231.500000	0.001337
2231.250000	0.001325
2231.000000	0.001309
2230.750000	0.001292
2230.500000	0.001278
2230.250000	0.001269
2230.000000	0.001267
2229.750000	0.001271
2229.500000	0.001282
2229.250000	0.001298
2229.000000	0.001316
2228.750000	0.001334
2228.500000	0.001348
2228.250000	0.001356
2228.000000	0.001357
2227.750000	0.001351
2227.500000	0.001339
2227.250000	0.001324
2227.000000	0.001309
2226.750000	0.001299
2226.500000	0.001293
2226.250000	0.001293
2226.000000	0.001295
2225.750000	0.001298
2225.500000	0.001299

2225.250000	0.001299
2225.000000	0.001296
2224.750000	0.001293
2224.500000	0.001290
2224.250000	0.001286
2224.000000	0.001281
2223.750000	0.001275
2223.500000	0.001269
2223.250000	0.001264
2223.000000	0.001264
2222.750000	0.001269
2222.500000	0.001281
2222.250000	0.001297
2222.000000	0.001314
2221.750000	0.001325
2221.500000	0.001328
2221.250000	0.001322
2221.000000	0.001309
2220.750000	0.001294
2220.500000	0.001284
2220.250000	0.001282
2220.000000	0.001290
2219.750000	0.001305
2219.500000	0.001321
2219.250000	0.001332
2219.000000	0.001332

2218.750000	0.001321
2218.500000	0.001299
2218.250000	0.001272
2218.000000	0.001245
2217.750000	0.001224
2217.500000	0.001213
2217.250000	0.001214
2217.000000	0.001227
2216.750000	0.001248
2216.500000	0.001276
2216.250000	0.001305
2216.000000	0.001333
2215.750000	0.001355
2215.500000	0.001371
2215.250000	0.001381
2215.000000	0.001385
2214.750000	0.001388
2214.500000	0.001390
2214.250000	0.001394
2214.000000	0.001400
2213.750000	0.001407
2213.500000	0.001413
2213.250000	0.001419
2213.000000	0.001424
2212.750000	0.001429
2212.500000	0.001435

2212.250000	0.001441
2212.000000	0.001445
2211.750000	0.001445
2211.500000	0.001438
2211.250000	0.001419
2211.000000	0.001391
2210.750000	0.001355
2210.500000	0.001318
2210.250000	0.001284
2210.000000	0.001260
2209.750000	0.001245
2209.500000	0.001240
2209.250000	0.001238
2209.000000	0.001236
2208.750000	0.001228
2208.500000	0.001214
2208.250000	0.001195
2208.000000	0.001177
2207.750000	0.001165
2207.500000	0.001163
2207.250000	0.001177
2207.000000	0.001205
2206.750000	0.001247
2206.500000	0.001296
2206.250000	0.001347
2206.000000	0.001392

2205.750000	0.001424
2205.500000	0.001439
2205.250000	0.001432
2205.000000	0.001406
2204.750000	0.001364
2204.500000	0.001312
2204.250000	0.001261
2204.000000	0.001219
2203.750000	0.001194
2203.500000	0.001188
2203.250000	0.001203
2203.000000	0.001233
2202.750000	0.001272
2202.500000	0.001311
2202.250000	0.001343
2202.000000	0.001363
2201.750000	0.001367
2201.500000	0.001359
2201.250000	0.001343
2201.000000	0.001324
2200.750000	0.001308
2200.500000	0.001300
2200.250000	0.001303
2200.000000	0.001314
2199.750000	0.001330
2199.500000	0.001347

2199.250000	0.001357
2199.000000	0.001359
2198.750000	0.001350
2198.500000	0.001332
2198.250000	0.001311
2198.000000	0.001293
2197.750000	0.001281
2197.500000	0.001279
2197.250000	0.001288
2197.000000	0.001305
2196.750000	0.001323
2196.500000	0.001338
2196.250000	0.001346
2196.000000	0.001343
2195.750000	0.001333
2195.500000	0.001319
2195.250000	0.001307
2195.000000	0.001303
2194.750000	0.001309
2194.500000	0.001326
2194.250000	0.001350
2194.000000	0.001376
2193.750000	0.001399
2193.500000	0.001414
2193.250000	0.001423
2193.000000	0.001426

2192.750000	0.001429
2192.500000	0.001436
2192.250000	0.001450
2192.000000	0.001468
2191.750000	0.001489
2191.500000	0.001505
2191.250000	0.001512
2191.000000	0.001506
2190.750000	0.001489
2190.500000	0.001464
2190.250000	0.001439
2190.000000	0.001420
2189.750000	0.001412
2189.500000	0.001417
2189.250000	0.001435
2189.000000	0.001460
2188.750000	0.001487
2188.500000	0.001512
2188.250000	0.001529
2188.000000	0.001538
2187.750000	0.001538
2187.500000	0.001531
2187.250000	0.001521
2187.000000	0.001510
2186.750000	0.001501
2186.500000	0.001497

2186.250000	0.001500
2186.000000	0.001510
2185.750000	0.001527
2185.500000	0.001550
2185.250000	0.001573
2185.000000	0.001594
2184.750000	0.001607
2184.500000	0.001608
2184.250000	0.001595
2184.000000	0.001568
2183.750000	0.001529
2183.500000	0.001482
2183.250000	0.001432
2183.000000	0.001384
2182.750000	0.001341
2182.500000	0.001303
2182.250000	0.001270
2182.000000	0.001242
2181.750000	0.001217
2181.500000	0.001196
2181.250000	0.001182
2181.000000	0.001178
2180.750000	0.001187
2180.500000	0.001210
2180.250000	0.001244
2180.000000	0.001284

2179.750000	0.001323
2179.500000	0.001353
2179.250000	0.001371
2179.000000	0.001373
2178.750000	0.001362
2178.500000	0.001343
2178.250000	0.001323
2178.000000	0.001308
2177.750000	0.001305
2177.500000	0.001314
2177.250000	0.001334
2177.000000	0.001363
2176.750000	0.001393
2176.500000	0.001418
2176.250000	0.001432
2176.000000	0.001431
2175.750000	0.001416
2175.500000	0.001388
2175.250000	0.001352
2175.000000	0.001315
2174.750000	0.001282
2174.500000	0.001257
2174.250000	0.001241
2174.000000	0.001232
2173.750000	0.001227
2173.500000	0.001224

2173.250000	0.001222
2173.000000	0.001220
2172.750000	0.001223
2172.500000	0.001234
2172.250000	0.001255
2172.000000	0.001289
2171.750000	0.001331
2171.500000	0.001377
2171.250000	0.001419
2171.000000	0.001452
2170.750000	0.001474
2170.500000	0.001484
2170.250000	0.001489
2170.000000	0.001495
2169.750000	0.001510
2169.500000	0.001540
2169.250000	0.001584
2169.000000	0.001640
2168.750000	0.001701
2168.500000	0.001757
2168.250000	0.001800
2168.000000	0.001826
2167.750000	0.001834
2167.500000	0.001827
2167.250000	0.001812
2167.000000	0.001795

2166.750000	0.001780
2166.500000	0.001768
2166.250000	0.001758
2166.000000	0.001748
2165.750000	0.001732
2165.500000	0.001711
2165.250000	0.001685
2165.000000	0.001658
2164.750000	0.001636
2164.500000	0.001624
2164.250000	0.001629
2164.000000	0.001651
2163.750000	0.001692
2163.500000	0.001749
2163.250000	0.001817
2163.000000	0.001890
2162.750000	0.001962
2162.500000	0.002026
2162.250000	0.002078
2162.000000	0.002114
2161.750000	0.002134
2161.500000	0.002137
2161.250000	0.002124
2161.000000	0.002093
2160.750000	0.002039
2160.500000	0.001956

2160.250000	0.001836
2160.000000	0.001678
2159.750000	0.001485
2159.500000	0.001269
2159.250000	0.001049
2159.000000	0.000845
2158.750000	0.000678
2158.500000	0.000558
2158.250000	0.000485
2158.000000	0.000450
2157.750000	0.000440
2157.500000	0.000444
2157.250000	0.000452
2157.000000	0.000463
2156.750000	0.000481
2156.500000	0.000508
2156.250000	0.000545
2156.000000	0.000591
2155.750000	0.000639
2155.500000	0.000685
2155.250000	0.000724
2155.000000	0.000756
2154.750000	0.000782
2154.500000	0.000808
2154.250000	0.000835
2154.000000	0.000864

2153.750000	0.000890
2153.500000	0.000908
2153.250000	0.000911
2153.000000	0.000895
2152.750000	0.000860
2152.500000	0.000809
2152.250000	0.000749
2152.000000	0.000691
2151.750000	0.000641
2151.500000	0.000607
2151.250000	0.000593
2151.000000	0.000600
2150.750000	0.000628
2150.500000	0.000671
2150.250000	0.000724
2150.000000	0.000782
2149.750000	0.000836
2149.500000	0.000881
2149.250000	0.000913
2149.000000	0.000931
2148.750000	0.000936
2148.500000	0.000928
2148.250000	0.000911
2148.000000	0.000887
2147.750000	0.000858
2147.500000	0.000829

2147.250000	0.000803
2147.000000	0.000784
2146.750000	0.000775
2146.500000	0.000779
2146.250000	0.000795
2146.000000	0.000818
2145.750000	0.000843
2145.500000	0.000863
2145.250000	0.000874
2145.000000	0.000872
2144.750000	0.000859
2144.500000	0.000839
2144.250000	0.000816
2144.000000	0.000795
2143.750000	0.000778
2143.500000	0.000767
2143.250000	0.000761
2143.000000	0.000760
2142.750000	0.000763
2142.500000	0.000770
2142.250000	0.000780
2142.000000	0.000793
2141.750000	0.000808
2141.500000	0.000822
2141.250000	0.000834
2141.000000	0.000844

2140.750000	0.000852
2140.500000	0.000860
2140.250000	0.000869
2140.000000	0.000877
2139.750000	0.000883
2139.500000	0.000885
2139.250000	0.000880
2139.000000	0.000869
2138.750000	0.000854
2138.500000	0.000837
2138.250000	0.000825
2138.000000	0.000821
2137.750000	0.000826
2137.500000	0.000837
2137.250000	0.000848
2137.000000	0.000853
2136.750000	0.000847
2136.500000	0.000828
2136.250000	0.000798
2136.000000	0.000763
2135.750000	0.000729
2135.500000	0.000699
2135.250000	0.000676
2135.000000	0.000659
2134.750000	0.000644
2134.500000	0.000629

2134.250000	0.000610
2134.000000	0.000587
2133.750000	0.000563
2133.500000	0.000542
2133.250000	0.000527
2133.000000	0.000520
2132.750000	0.000522
2132.500000	0.000532
2132.250000	0.000548
2132.000000	0.000567
2131.750000	0.000588
2131.500000	0.000610
2131.250000	0.000633
2131.000000	0.000659
2130.750000	0.000686
2130.500000	0.000715
2130.250000	0.000746
2130.000000	0.000779
2129.750000	0.000812
2129.500000	0.000844
2129.250000	0.000873
2129.000000	0.000897
2128.750000	0.000913
2128.500000	0.000918
2128.250000	0.000912
2128.000000	0.000897

2127.750000	0.000877
2127.500000	0.000855
2127.250000	0.000839
2127.000000	0.000830
2126.750000	0.000831
2126.500000	0.000840
2126.250000	0.000855
2126.000000	0.000871
2125.750000	0.000886
2125.500000	0.000895
2125.250000	0.000899
2125.000000	0.000897
2124.750000	0.000890
2124.500000	0.000878
2124.250000	0.000863
2124.000000	0.000848
2123.750000	0.000834
2123.500000	0.000825
2123.250000	0.000824
2123.000000	0.000831
2122.750000	0.000845
2122.500000	0.000865
2122.250000	0.000887
2122.000000	0.000906
2121.750000	0.000919
2121.500000	0.000927

2121.250000	0.000931
2121.000000	0.000933
2120.750000	0.000936
2120.500000	0.000941
2120.250000	0.000948
2120.000000	0.000955
2119.750000	0.000961
2119.500000	0.000965
2119.250000	0.000967
2119.000000	0.000967
2118.750000	0.000968
2118.500000	0.000972
2118.250000	0.000978
2118.000000	0.000988
2117.750000	0.000999
2117.500000	0.001010
2117.250000	0.001022
2117.000000	0.001033
2116.750000	0.001044
2116.500000	0.001055
2116.250000	0.001065
2116.000000	0.001073
2115.750000	0.001078
2115.500000	0.001079
2115.250000	0.001079
2115.000000	0.001079

2114.750000	0.001081
2114.500000	0.001088
2114.250000	0.001100
2114.000000	0.001117
2113.750000	0.001136
2113.500000	0.001155
2113.250000	0.001171
2113.000000	0.001182
2112.750000	0.001186
2112.500000	0.001182
2112.250000	0.001171
2112.000000	0.001153
2111.750000	0.001130
2111.500000	0.001105
2111.250000	0.001082
2111.000000	0.001064
2110.750000	0.001054
2110.500000	0.001053
2110.250000	0.001061
2110.000000	0.001076
2109.750000	0.001095
2109.500000	0.001113
2109.250000	0.001128
2109.000000	0.001138
2108.750000	0.001143
2108.500000	0.001141

2108.250000	0.001132
2108.000000	0.001116
2107.750000	0.001094
2107.500000	0.001067
2107.250000	0.001038
2107.000000	0.001012
2106.750000	0.000994
2106.500000	0.000989
2106.250000	0.000998
2106.000000	0.001021
2105.750000	0.001052
2105.500000	0.001086
2105.250000	0.001116
2105.000000	0.001135
2104.750000	0.001140
2104.500000	0.001132
2104.250000	0.001112
2104.000000	0.001085
2103.750000	0.001054
2103.500000	0.001024
2103.250000	0.000999
2103.000000	0.000979
2102.750000	0.000967
2102.500000	0.000963
2102.250000	0.000967
2102.000000	0.000979

2101.750000	0.000995
2101.500000	0.001015
2101.250000	0.001034
2101.000000	0.001050
2100.750000	0.001060
2100.500000	0.001063
2100.250000	0.001059
2100.000000	0.001050
2099.750000	0.001041
2099.500000	0.001035
2099.250000	0.001037
2099.000000	0.001047
2098.750000	0.001065
2098.500000	0.001087
2098.250000	0.001107
2098.000000	0.001120
2097.750000	0.001123
2097.500000	0.001114
2097.250000	0.001096
2097.000000	0.001073
2096.750000	0.001046
2096.500000	0.001020
2096.250000	0.000994
2096.000000	0.000967
2095.750000	0.000938
2095.500000	0.000909

2095.250000	0.000882
2095.000000	0.000862
2094.750000	0.000853
2094.500000	0.000858
2094.250000	0.000876
2094.000000	0.000903
2093.750000	0.000933
2093.500000	0.000960
2093.250000	0.000978
2093.000000	0.000987
2092.750000	0.000987
2092.500000	0.000982
2092.250000	0.000973
2092.000000	0.000964
2091.750000	0.000955
2091.500000	0.000945
2091.250000	0.000935
2091.000000	0.000925
2090.750000	0.000915
2090.500000	0.000907
2090.250000	0.000901
2090.000000	0.000899
2089.750000	0.000899
2089.500000	0.000904
2089.250000	0.000913
2089.000000	0.000925

2088.750000	0.000942
2088.500000	0.000962
2088.250000	0.000982
2088.000000	0.001001
2087.750000	0.001015
2087.500000	0.001023
2087.250000	0.001025
2087.000000	0.001026
2086.750000	0.001030
2086.500000	0.001039
2086.250000	0.001056
2086.000000	0.001078
2085.750000	0.001101
2085.500000	0.001121
2085.250000	0.001133
2085.000000	0.001136
2084.750000	0.001131
2084.500000	0.001124
2084.250000	0.001118
2084.000000	0.001118
2083.750000	0.001123
2083.500000	0.001133
2083.250000	0.001143
2083.000000	0.001149
2082.750000	0.001150
2082.500000	0.001143

2082.250000	0.001129
2082.000000	0.001110
2081.750000	0.001086
2081.500000	0.001061
2081.250000	0.001036
2081.000000	0.001014
2080.750000	0.000996
2080.500000	0.000986
2080.250000	0.000982
2080.000000	0.000985
2079.750000	0.000992
2079.500000	0.001000
2079.250000	0.001006
2079.000000	0.001008
2078.750000	0.001008
2078.500000	0.001007
2078.250000	0.001009
2078.000000	0.001016
2077.750000	0.001027
2077.500000	0.001044
2077.250000	0.001063
2077.000000	0.001081
2076.750000	0.001096
2076.500000	0.001105
2076.250000	0.001107
2076.000000	0.001102

2075.750000	0.001089
2075.500000	0.001071
2075.250000	0.001051
2075.000000	0.001032
2074.750000	0.001018
2074.500000	0.001013
2074.250000	0.001017
2074.000000	0.001029
2073.750000	0.001046
2073.500000	0.001063
2073.250000	0.001077
2073.000000	0.001086
2072.750000	0.001088
2072.500000	0.001086
2072.250000	0.001082
2072.000000	0.001079
2071.750000	0.001076
2071.500000	0.001073
2071.250000	0.001070
2071.000000	0.001066
2070.750000	0.001063
2070.500000	0.001062
2070.250000	0.001063
2070.000000	0.001067
2069.750000	0.001073
2069.500000	0.001078

2069.250000	0.001079
2069.000000	0.001075
2068.750000	0.001067
2068.500000	0.001057
2068.250000	0.001048
2068.000000	0.001042
2067.750000	0.001040
2067.500000	0.001040
2067.250000	0.001040
2067.000000	0.001037
2066.750000	0.001030
2066.500000	0.001022
2066.250000	0.001014
2066.000000	0.001010
2065.750000	0.001012
2065.500000	0.001021
2065.250000	0.001034
2065.000000	0.001049
2064.750000	0.001060
2064.500000	0.001067
2064.250000	0.001068
2064.000000	0.001063
2063.750000	0.001053
2063.500000	0.001040
2063.250000	0.001025
2063.000000	0.001010

2062.750000	0.000994
2062.500000	0.000979
2062.250000	0.000964
2062.000000	0.000953
2061.750000	0.000943
2061.500000	0.000938
2061.250000	0.000937
2061.000000	0.000942
2060.750000	0.000952
2060.500000	0.000970
2060.250000	0.000993
2060.000000	0.001021
2059.750000	0.001050
2059.500000	0.001077
2059.250000	0.001100
2059.000000	0.001115
2058.750000	0.001123
2058.500000	0.001125
2058.250000	0.001121
2058.000000	0.001113
2057.750000	0.001104
2057.500000	0.001094
2057.250000	0.001084
2057.000000	0.001077
2056.750000	0.001074
2056.500000	0.001075

2056.250000	0.001082
2056.000000	0.001092
2055.750000	0.001103
2055.500000	0.001110
2055.250000	0.001109
2055.000000	0.001099
2054.750000	0.001080
2054.500000	0.001057
2054.250000	0.001036
2054.000000	0.001024
2053.750000	0.001027
2053.500000	0.001046
2053.250000	0.001082
2053.000000	0.001132
2052.750000	0.001194
2052.500000	0.001261
2052.250000	0.001332
2052.000000	0.001401
2051.750000	0.001463
2051.500000	0.001513
2051.250000	0.001546
2051.000000	0.001555
2050.750000	0.001537
2050.500000	0.001493
2050.250000	0.001425
2050.000000	0.001340

2049.750000	0.001248
2049.500000	0.001159
2049.250000	0.001081
2049.000000	0.001020
2048.750000	0.000980
2048.500000	0.000960
2048.250000	0.000958
2048.000000	0.000971
2047.750000	0.000993
2047.500000	0.001017
2047.250000	0.001040
2047.000000	0.001057
2046.750000	0.001065
2046.500000	0.001067
2046.250000	0.001063
2046.000000	0.001059
2045.750000	0.001057
2045.500000	0.001062
2045.250000	0.001075
2045.000000	0.001094
2044.750000	0.001117
2044.500000	0.001138
2044.250000	0.001151
2044.000000	0.001153
2043.750000	0.001142
2043.500000	0.001117

2043.250000	0.001082
2043.000000	0.001044
2042.750000	0.001008
2042.500000	0.000981
2042.250000	0.000967
2042.000000	0.000969
2041.750000	0.000985
2041.500000	0.001013
2041.250000	0.001047
2041.000000	0.001082
2040.750000	0.001110
2040.500000	0.001129
2040.250000	0.001136
2040.000000	0.001130
2039.750000	0.001116
2039.500000	0.001099
2039.250000	0.001085
2039.000000	0.001082
2038.750000	0.001093
2038.500000	0.001121
2038.250000	0.001163
2038.000000	0.001214
2037.750000	0.001266
2037.500000	0.001313
2037.250000	0.001349
2037.000000	0.001371

2036.750000	0.001380
2036.500000	0.001374
2036.250000	0.001357
2036.000000	0.001327
2035.750000	0.001287
2035.500000	0.001237
2035.250000	0.001181
2035.000000	0.001123
2034.750000	0.001070
2034.500000	0.001028
2034.250000	0.001001
2034.000000	0.000993
2033.750000	0.001003
2033.500000	0.001027
2033.250000	0.001064
2033.000000	0.001111
2032.750000	0.001163
2032.500000	0.001217
2032.250000	0.001268
2032.000000	0.001308
2031.750000	0.001331
2031.500000	0.001332
2031.250000	0.001312
2031.000000	0.001276
2030.750000	0.001233
2030.500000	0.001196

2030.250000	0.001173
2030.000000	0.001169
2029.750000	0.001182
2029.500000	0.001207
2029.250000	0.001233
2029.000000	0.001253
2028.750000	0.001263
2028.500000	0.001263
2028.250000	0.001257
2028.000000	0.001248
2027.750000	0.001238
2027.500000	0.001228
2027.250000	0.001216
2027.000000	0.001200
2026.750000	0.001179
2026.500000	0.001155
2026.250000	0.001132
2026.000000	0.001113
2025.750000	0.001102
2025.500000	0.001097
2025.250000	0.001095
2025.000000	0.001091
2024.750000	0.001077
2024.500000	0.001052
2024.250000	0.001012
2024.000000	0.000962

2023.750000	0.000908
2023.500000	0.000855
2023.250000	0.000811
2023.000000	0.000783
2022.750000	0.000770
2022.500000	0.000772
2022.250000	0.000785
2022.000000	0.000801
2021.750000	0.000815
2021.500000	0.000823
2021.250000	0.000827
2021.000000	0.000830
2020.750000	0.000839
2020.500000	0.000858
2020.250000	0.000890
2020.000000	0.000930
2019.750000	0.000970
2019.500000	0.001002
2019.250000	0.001017
2019.000000	0.001013
2018.750000	0.000996
2018.500000	0.000974
2018.250000	0.000956
2018.000000	0.000952
2017.750000	0.000963
2017.500000	0.000983

2017.250000	0.001003
2017.000000	0.001011
2016.750000	0.000997
2016.500000	0.000960
2016.250000	0.000903
2016.000000	0.000838
2015.750000	0.000776
2015.500000	0.000728
2015.250000	0.000700
2015.000000	0.000695
2014.750000	0.000713
2014.500000	0.000749
2014.250000	0.000796
2014.000000	0.000847
2013.750000	0.000894
2013.500000	0.000932
2013.250000	0.000959
2013.000000	0.000975
2012.750000	0.000985
2012.500000	0.000995
2012.250000	0.001012
2012.000000	0.001039
2011.750000	0.001074
2011.500000	0.001110
2011.250000	0.001140
2011.000000	0.001152

2010.750000	0.001141
2010.500000	0.001104
2010.250000	0.001044
2010.000000	0.000972
2009.750000	0.000900
2009.500000	0.000840
2009.250000	0.000803
2009.000000	0.000791
2008.750000	0.000802
2008.500000	0.000829
2008.250000	0.000858
2008.000000	0.000879
2007.750000	0.000884
2007.500000	0.000868
2007.250000	0.000837
2007.000000	0.000799
2006.750000	0.000764
2006.500000	0.000740
2006.250000	0.000730
2006.000000	0.000735
2005.750000	0.000748
2005.500000	0.000762
2005.250000	0.000774
2005.000000	0.000781
2004.750000	0.000786
2004.500000	0.000790

2004.250000	0.000794
2004.000000	0.000796
2003.750000	0.000791
2003.500000	0.000773
2003.250000	0.000735
2003.000000	0.000678
2002.750000	0.000606
2002.500000	0.000531
2002.250000	0.000466
2002.000000	0.000422
2001.750000	0.000408
2001.500000	0.000426
2001.250000	0.000470
2001.000000	0.000533
2000.750000	0.000604
2000.500000	0.000673
2000.250000	0.000734
2000.000000	0.000782
1999.750000	0.000816
1999.500000	0.000838
1999.250000	0.000849
1999.000000	0.000853
1998.750000	0.000856
1998.500000	0.000861
1998.250000	0.000874
1998.000000	0.000895

1997.750000	0.000925
1997.500000	0.000961
1997.250000	0.000999
1997.000000	0.001032
1996.750000	0.001057
1996.500000	0.001070
1996.250000	0.001072
1996.000000	0.001067
1995.750000	0.001061
1995.500000	0.001058
1995.250000	0.001062
1995.000000	0.001073
1994.750000	0.001086
1994.500000	0.001094
1994.250000	0.001088
1994.000000	0.001064
1993.750000	0.001019
1993.500000	0.000955
1993.250000	0.000881
1993.000000	0.000808
1992.750000	0.000749
1992.500000	0.000715
1992.250000	0.000711
1992.000000	0.000733
1991.750000	0.000772
1991.500000	0.000819

1991.250000	0.000865
1991.000000	0.000909
1990.750000	0.000954
1990.500000	0.001002
1990.250000	0.001055
1990.000000	0.001110
1989.750000	0.001161
1989.500000	0.001198
1989.250000	0.001211
1989.000000	0.001194
1988.750000	0.001147
1988.500000	0.001077
1988.250000	0.000996
1988.000000	0.000920
1987.750000	0.000861
1987.500000	0.000828
1987.250000	0.000822
1987.000000	0.000842
1986.750000	0.000882
1986.500000	0.000933
1986.250000	0.000988
1986.000000	0.001041
1985.750000	0.001085
1985.500000	0.001116
1985.250000	0.001134
1985.000000	0.001139

1984.750000	0.001136
1984.500000	0.001129
1984.250000	0.001122
1984.000000	0.001119
1983.750000	0.001119
1983.500000	0.001122
1983.250000	0.001125
1983.000000	0.001129
1982.750000	0.001135
1982.500000	0.001147
1982.250000	0.001168
1982.000000	0.001202
1981.750000	0.001249
1981.500000	0.001308
1981.250000	0.001374
1981.000000	0.001443
1980.750000	0.001510
1980.500000	0.001572
1980.250000	0.001624
1980.000000	0.001661
1979.750000	0.001673
1979.500000	0.001652
1979.250000	0.001590
1979.000000	0.001486
1978.750000	0.001344
1978.500000	0.001179

1978.250000	0.001012
1978.000000	0.000864
1977.750000	0.000754
1977.500000	0.000691
1977.250000	0.000674
1977.000000	0.000696
1976.750000	0.000744
1976.500000	0.000806
1976.250000	0.000871
1976.000000	0.000930
1975.750000	0.000975
1975.500000	0.001000
1975.250000	0.000998
1975.000000	0.000967
1974.750000	0.000908
1974.500000	0.000829
1974.250000	0.000739
1974.000000	0.000655
1973.750000	0.000588
1973.500000	0.000547
1973.250000	0.000533
1973.000000	0.000541
1972.750000	0.000562
1972.500000	0.000587
1972.250000	0.000607
1972.000000	0.000618

1971.750000	0.000621
1971.500000	0.000617
1971.250000	0.000613
1971.000000	0.000610
1970.750000	0.000612
1970.500000	0.000620
1970.250000	0.000633
1970.000000	0.000651
1969.750000	0.000673
1969.500000	0.000700
1969.250000	0.000729
1969.000000	0.000757
1968.750000	0.000781
1968.500000	0.000796
1968.250000	0.000798
1968.000000	0.000785
1967.750000	0.000758
1967.500000	0.000722
1967.250000	0.000683
1967.000000	0.000649
1966.750000	0.000627
1966.500000	0.000620
1966.250000	0.000628
1966.000000	0.000650
1965.750000	0.000681
1965.500000	0.000719

1965.250000	0.000759
1965.000000	0.000799
1964.750000	0.000837
1964.500000	0.000871
1964.250000	0.000895
1964.000000	0.000908
1963.750000	0.000907
1963.500000	0.000892
1963.250000	0.000867
1963.000000	0.000838
1962.750000	0.000810
1962.500000	0.000790
1962.250000	0.000780
1962.000000	0.000777
1961.750000	0.000777
1961.500000	0.000775
1961.250000	0.000764
1961.000000	0.000744
1960.750000	0.000717
1960.500000	0.000686
1960.250000	0.000658
1960.000000	0.000634
1959.750000	0.000617
1959.500000	0.000602
1959.250000	0.000584
1959.000000	0.000561

1958.750000	0.000530
1958.500000	0.000496
1958.250000	0.000466
1958.000000	0.000445
1957.750000	0.000442
1957.500000	0.000458
1957.250000	0.000492
1957.000000	0.000538
1956.750000	0.000590
1956.500000	0.000641
1956.250000	0.000685
1956.000000	0.000720
1955.750000	0.000749
1955.500000	0.000773
1955.250000	0.000797
1955.000000	0.000819
1954.750000	0.000840
1954.500000	0.000855
1954.250000	0.000860
1954.000000	0.000851
1953.750000	0.000828
1953.500000	0.000795
1953.250000	0.000758
1953.000000	0.000723
1952.750000	0.000699
1952.500000	0.000689

1952.250000	0.000693
1952.000000	0.000705
1951.750000	0.000719
1951.500000	0.000728
1951.250000	0.000724
1951.000000	0.000707
1950.750000	0.000678
1950.500000	0.000641
1950.250000	0.000602
1950.000000	0.000567
1949.750000	0.000542
1949.500000	0.000529
1949.250000	0.000530
1949.000000	0.000543
1948.750000	0.000567
1948.500000	0.000601
1948.250000	0.000640
1948.000000	0.000680
1947.750000	0.000717
1947.500000	0.000746
1947.250000	0.000764
1947.000000	0.000769
1946.750000	0.000761
1946.500000	0.000743
1946.250000	0.000720
1946.000000	0.000695

1945.750000	0.000673
1945.500000	0.000655
1945.250000	0.000642
1945.000000	0.000635
1944.750000	0.000632
1944.500000	0.000632
1944.250000	0.000631
1944.000000	0.000627
1943.750000	0.000618
1943.500000	0.000601
1943.250000	0.000578
1943.000000	0.000551
1942.750000	0.000525
1942.500000	0.000503
1942.250000	0.000491
1942.000000	0.000491
1941.750000	0.000502
1941.500000	0.000525
1941.250000	0.000554
1941.000000	0.000587
1940.750000	0.000621
1940.500000	0.000652
1940.250000	0.000681
1940.000000	0.000706
1939.750000	0.000727
1939.500000	0.000746

1939.250000	0.000761
1939.000000	0.000776
1938.750000	0.000790
1938.500000	0.000804
1938.250000	0.000815
1938.000000	0.000821
1937.750000	0.000817
1937.500000	0.000799
1937.250000	0.000768
1937.000000	0.000725
1936.750000	0.000677
1936.500000	0.000631
1936.250000	0.000596
1936.000000	0.000574
1935.750000	0.000568
1935.500000	0.000574
1935.250000	0.000586
1935.000000	0.000599
1934.750000	0.000610
1934.500000	0.000618
1934.250000	0.000622
1934.000000	0.000624
1933.750000	0.000624
1933.500000	0.000623
1933.250000	0.000619
1933.000000	0.000613

1932.750000	0.000606
1932.500000	0.000601
1932.250000	0.000602
1932.000000	0.000610
1931.750000	0.000627
1931.500000	0.000651
1931.250000	0.000676
1931.000000	0.000698
1930.750000	0.000712
1930.500000	0.000717
1930.250000	0.000711
1930.000000	0.000698
1929.750000	0.000681
1929.500000	0.000663
1929.250000	0.000647
1929.000000	0.000634
1928.750000	0.000623
1928.500000	0.000614
1928.250000	0.000604
1928.000000	0.000594
1927.750000	0.000583
1927.500000	0.000572
1927.250000	0.000564
1927.000000	0.000560
1926.750000	0.000563
1926.500000	0.000573

1926.250000	0.000591
1926.000000	0.000614
1925.750000	0.000639
1925.500000	0.000662
1925.250000	0.000679
1925.000000	0.000689
1924.750000	0.000690
1924.500000	0.000683
1924.250000	0.000667
1924.000000	0.000645
1923.750000	0.000618
1923.500000	0.000588
1923.250000	0.000560
1923.000000	0.000535
1922.750000	0.000517
1922.500000	0.000508
1922.250000	0.000507
1922.000000	0.000515
1921.750000	0.000529
1921.500000	0.000548
1921.250000	0.000567
1921.000000	0.000584
1920.750000	0.000598
1920.500000	0.000609
1920.250000	0.000619
1920.000000	0.000632

1919.750000	0.000650
1919.500000	0.000673
1919.250000	0.000702
1919.000000	0.000733
1918.750000	0.000764
1918.500000	0.000787
1918.250000	0.000797
1918.000000	0.000788
1917.750000	0.000760
1917.500000	0.000720
1917.250000	0.000676
1917.000000	0.000639
1916.750000	0.000616
1916.500000	0.000606
1916.250000	0.000607
1916.000000	0.000614
1915.750000	0.000621
1915.500000	0.000622
1915.250000	0.000615
1915.000000	0.000600
1914.750000	0.000578
1914.500000	0.000554
1914.250000	0.000534
1914.000000	0.000523
1913.750000	0.000523
1913.500000	0.000532

1913.250000	0.000549
1913.000000	0.000569
1912.750000	0.000587
1912.500000	0.000601
1912.250000	0.000608
1912.000000	0.000609
1911.750000	0.000606
1911.500000	0.000600
1911.250000	0.000592
1911.000000	0.000582
1910.750000	0.000571
1910.500000	0.000560
1910.250000	0.000552
1910.000000	0.000546
1909.750000	0.000546
1909.500000	0.000551
1909.250000	0.000560
1909.000000	0.000570
1908.750000	0.000581
1908.500000	0.000589
1908.250000	0.000595
1908.000000	0.000600
1907.750000	0.000605
1907.500000	0.000613
1907.250000	0.000625
1907.000000	0.000641

1906.750000	0.000660
1906.500000	0.000678
1906.250000	0.000690
1906.000000	0.000694
1905.750000	0.000686
1905.500000	0.000666
1905.250000	0.000637
1905.000000	0.000603
1904.750000	0.000571
1904.500000	0.000548
1904.250000	0.000537
1904.000000	0.000540
1903.750000	0.000555
1903.500000	0.000579
1903.250000	0.000605
1903.000000	0.000628
1902.750000	0.000647
1902.500000	0.000660
1902.250000	0.000668
1902.000000	0.000671
1901.750000	0.000672
1901.500000	0.000670
1901.250000	0.000665
1901.000000	0.000656
1900.750000	0.000645
1900.500000	0.000632

1900.250000	0.000621
1900.000000	0.000614
1899.750000	0.000612
1899.500000	0.000615
1899.250000	0.000621
1899.000000	0.000626
1898.750000	0.000630
1898.500000	0.000631
1898.250000	0.000629
1898.000000	0.000628
1897.750000	0.000630
1897.500000	0.000636
1897.250000	0.000648
1897.000000	0.000665
1896.750000	0.000685
1896.500000	0.000705
1896.250000	0.000724
1896.000000	0.000739
1895.750000	0.000749
1895.500000	0.000755
1895.250000	0.000756
1895.000000	0.000752
1894.750000	0.000745
1894.500000	0.000736
1894.250000	0.000725
1894.000000	0.000713

1893.750000	0.000699
1893.500000	0.000684
1893.250000	0.000670
1893.000000	0.000657
1892.750000	0.000647
1892.500000	0.000643
1892.250000	0.000644
1892.000000	0.000651
1891.750000	0.000665
1891.500000	0.000683
1891.250000	0.000702
1891.000000	0.000719
1890.750000	0.000730
1890.500000	0.000731
1890.250000	0.000721
1890.000000	0.000699
1889.750000	0.000668
1889.500000	0.000635
1889.250000	0.000608
1889.000000	0.000592
1888.750000	0.000592
1888.500000	0.000605
1888.250000	0.000627
1888.000000	0.000651
1887.750000	0.000671
1887.500000	0.000683

1887.250000	0.000687
1887.000000	0.000685
1886.750000	0.000679
1886.500000	0.000674
1886.250000	0.000669
1886.000000	0.000668
1885.750000	0.000667
1885.500000	0.000666
1885.250000	0.000663
1885.000000	0.000658
1884.750000	0.000651
1884.500000	0.000644
1884.250000	0.000638
1884.000000	0.000634
1883.750000	0.000634
1883.500000	0.000635
1883.250000	0.000639
1883.000000	0.000644
1882.750000	0.000651
1882.500000	0.000657
1882.250000	0.000665
1882.000000	0.000672
1881.750000	0.000679
1881.500000	0.000682
1881.250000	0.000682
1881.000000	0.000677

1880.750000	0.000667
1880.500000	0.000654
1880.250000	0.000638
1880.000000	0.000622
1879.750000	0.000608
1879.500000	0.000598
1879.250000	0.000592
1879.000000	0.000590
1878.750000	0.000591
1878.500000	0.000596
1878.250000	0.000601
1878.000000	0.000608
1877.750000	0.000614
1877.500000	0.000619
1877.250000	0.000623
1877.000000	0.000627
1876.750000	0.000633
1876.500000	0.000642
1876.250000	0.000655
1876.000000	0.000671
1875.750000	0.000688
1875.500000	0.000704
1875.250000	0.000716
1875.000000	0.000722
1874.750000	0.000722
1874.500000	0.000715

1874.250000	0.000705
1874.000000	0.000693
1873.750000	0.000681
1873.500000	0.000669
1873.250000	0.000658
1873.000000	0.000648
1872.750000	0.000639
1872.500000	0.000631
1872.250000	0.000625
1872.000000	0.000620
1871.750000	0.000619
1871.500000	0.000623
1871.250000	0.000633
1871.000000	0.000650
1870.750000	0.000673
1870.500000	0.000700
1870.250000	0.000729
1870.000000	0.000759
1869.750000	0.000785
1869.500000	0.000802
1869.250000	0.000806
1869.000000	0.000793
1868.750000	0.000765
1868.500000	0.000728
1868.250000	0.000689
1868.000000	0.000655

1867.750000	0.000629
1867.500000	0.000612
1867.250000	0.000604
1867.000000	0.000601
1866.750000	0.000601
1866.500000	0.000604
1866.250000	0.000607
1866.000000	0.000612
1865.750000	0.000618
1865.500000	0.000624
1865.250000	0.000630
1865.000000	0.000633
1864.750000	0.000633
1864.500000	0.000630
1864.250000	0.000625
1864.000000	0.000618
1863.750000	0.000611
1863.500000	0.000606
1863.250000	0.000604
1863.000000	0.000606
1862.750000	0.000610
1862.500000	0.000613
1862.250000	0.000613
1862.000000	0.000610
1861.750000	0.000604
1861.500000	0.000596

1861.250000	0.000590
1861.000000	0.000585
1860.750000	0.000584
1860.500000	0.000584
1860.250000	0.000586
1860.000000	0.000587
1859.750000	0.000587
1859.500000	0.000585
1859.250000	0.000581
1859.000000	0.000578
1858.750000	0.000574
1858.500000	0.000571
1858.250000	0.000569
1858.000000	0.000568
1857.750000	0.000566
1857.500000	0.000565
1857.250000	0.000564
1857.000000	0.000562
1856.750000	0.000562
1856.500000	0.000563
1856.250000	0.000566
1856.000000	0.000571
1855.750000	0.000576
1855.500000	0.000582
1855.250000	0.000587
1855.000000	0.000590

1854.750000	0.000591
1854.500000	0.000589
1854.250000	0.000586
1854.000000	0.000582
1853.750000	0.000578
1853.500000	0.000573
1853.250000	0.000569
1853.000000	0.000564
1852.750000	0.000560
1852.500000	0.000555
1852.250000	0.000552
1852.000000	0.000548
1851.750000	0.000546
1851.500000	0.000545
1851.250000	0.000544
1851.000000	0.000542
1850.750000	0.000539
1850.500000	0.000535
1850.250000	0.000530
1850.000000	0.000525
1849.750000	0.000523
1849.500000	0.000526
1849.250000	0.000533
1849.000000	0.000542
1848.750000	0.000551
1848.500000	0.000557

1848.250000	0.000556
1848.000000	0.000549
1847.750000	0.000536
1847.500000	0.000519
1847.250000	0.000503
1847.000000	0.000490
1846.750000	0.000482
1846.500000	0.000479
1846.250000	0.000482
1846.000000	0.000490
1845.750000	0.000503
1845.500000	0.000520
1845.250000	0.000539
1845.000000	0.000562
1844.750000	0.000586
1844.500000	0.000607
1844.250000	0.000617
1844.000000	0.000610
1843.750000	0.000588
1843.500000	0.000561
1843.250000	0.000540
1843.000000	0.000532
1842.750000	0.000533
1842.500000	0.000538
1842.250000	0.000542
1842.000000	0.000543

1841.750000	0.000541
1841.500000	0.000538
1841.250000	0.000537
1841.000000	0.000539
1840.750000	0.000546
1840.500000	0.000555
1840.250000	0.000564
1840.000000	0.000570
1839.750000	0.000572
1839.500000	0.000570
1839.250000	0.000565
1839.000000	0.000560
1838.750000	0.000556
1838.500000	0.000554
1838.250000	0.000551
1838.000000	0.000548
1837.750000	0.000542
1837.500000	0.000532
1837.250000	0.000519
1837.000000	0.000504
1836.750000	0.000490
1836.500000	0.000480
1836.250000	0.000475
1836.000000	0.000477
1835.750000	0.000485
1835.500000	0.000496

1835.250000	0.000508
1835.000000	0.000520
1834.750000	0.000530
1834.500000	0.000537
1834.250000	0.000542
1834.000000	0.000543
1833.750000	0.000543
1833.500000	0.000540
1833.250000	0.000536
1833.000000	0.000532
1832.750000	0.000530
1832.500000	0.000533
1832.250000	0.000542
1832.000000	0.000557
1831.750000	0.000578
1831.500000	0.000602
1831.250000	0.000626
1831.000000	0.000647
1830.750000	0.000663
1830.500000	0.000674
1830.250000	0.000678
1830.000000	0.000673
1829.750000	0.000656
1829.500000	0.000629
1829.250000	0.000599
1829.000000	0.000573

1828.750000	0.000559
1828.500000	0.000557
1828.250000	0.000563
1828.000000	0.000570
1827.750000	0.000576
1827.500000	0.000579
1827.250000	0.000579
1827.000000	0.000578
1826.750000	0.000576
1826.500000	0.000575
1826.250000	0.000577
1826.000000	0.000580
1825.750000	0.000586
1825.500000	0.000591
1825.250000	0.000592
1825.000000	0.000586
1824.750000	0.000576
1824.500000	0.000568
1824.250000	0.000566
1824.000000	0.000571
1823.750000	0.000580
1823.500000	0.000588
1823.250000	0.000592
1823.000000	0.000588
1822.750000	0.000580
1822.500000	0.000570

1822.250000	0.000562
1822.000000	0.000557
1821.750000	0.000557
1821.500000	0.000562
1821.250000	0.000568
1821.000000	0.000575
1820.750000	0.000579
1820.500000	0.000580
1820.250000	0.000577
1820.000000	0.000574
1819.750000	0.000569
1819.500000	0.000563
1819.250000	0.000559
1819.000000	0.000554
1818.750000	0.000549
1818.500000	0.000543
1818.250000	0.000534
1818.000000	0.000523
1817.750000	0.000510
1817.500000	0.000496
1817.250000	0.000484
1817.000000	0.000475
1816.750000	0.000472
1816.500000	0.000476
1816.250000	0.000486
1816.000000	0.000502

1815.750000	0.000520
1815.500000	0.000538
1815.250000	0.000554
1815.000000	0.000565
1814.750000	0.000573
1814.500000	0.000578
1814.250000	0.000581
1814.000000	0.000584
1813.750000	0.000586
1813.500000	0.000588
1813.250000	0.000586
1813.000000	0.000582
1812.750000	0.000576
1812.500000	0.000569
1812.250000	0.000562
1812.000000	0.000558
1811.750000	0.000558
1811.500000	0.000561
1811.250000	0.000567
1811.000000	0.000573
1810.750000	0.000578
1810.500000	0.000581
1810.250000	0.000583
1810.000000	0.000584
1809.750000	0.000588
1809.500000	0.000594

1809.250000	0.000601
1809.000000	0.000607
1808.750000	0.000609
1808.500000	0.000607
1808.250000	0.000601
1808.000000	0.000592
1807.750000	0.000582
1807.500000	0.000572
1807.250000	0.000564
1807.000000	0.000558
1806.750000	0.000555
1806.500000	0.000553
1806.250000	0.000554
1806.000000	0.000556
1805.750000	0.000561
1805.500000	0.000567
1805.250000	0.000575
1805.000000	0.000586
1804.750000	0.000598
1804.500000	0.000612
1804.250000	0.000626
1804.000000	0.000639
1803.750000	0.000650
1803.500000	0.000656
1803.250000	0.000657
1803.000000	0.000650

1802.750000	0.000638
1802.500000	0.000619
1802.250000	0.000597
1802.000000	0.000575
1801.750000	0.000556
1801.500000	0.000545
1801.250000	0.000543
1801.000000	0.000549
1800.750000	0.000561
1800.500000	0.000576
1800.250000	0.000591
1800.000000	0.000605
1799.750000	0.000614
1799.500000	0.000620
1799.250000	0.000623
1799.000000	0.000624
1798.750000	0.000626
1798.500000	0.000631
1798.250000	0.000639
1798.000000	0.000648
1797.750000	0.000658
1797.500000	0.000665
1797.250000	0.000670
1797.000000	0.000670
1796.750000	0.000667
1796.500000	0.000662

1796.250000	0.000656
1796.000000	0.000650
1795.750000	0.000645
1795.500000	0.000641
1795.250000	0.000640
1795.000000	0.000644
1794.750000	0.000655
1794.500000	0.000673
1794.250000	0.000695
1794.000000	0.000719
1793.750000	0.000740
1793.500000	0.000754
1793.250000	0.000759
1793.000000	0.000754
1792.750000	0.000739
1792.500000	0.000716
1792.250000	0.000690
1792.000000	0.000669
1791.750000	0.000657
1791.500000	0.000655
1791.250000	0.000660
1791.000000	0.000667
1790.750000	0.000673
1790.500000	0.000677
1790.250000	0.000679
1790.000000	0.000683

1789.750000	0.000690
1789.500000	0.000699
1789.250000	0.000711
1789.000000	0.000722
1788.750000	0.000731
1788.500000	0.000738
1788.250000	0.000742
1788.000000	0.000742
1787.750000	0.000741
1787.500000	0.000738
1787.250000	0.000735
1787.000000	0.000732
1786.750000	0.000731
1786.500000	0.000732
1786.250000	0.000735
1786.000000	0.000742
1785.750000	0.000749
1785.500000	0.000755
1785.250000	0.000759
1785.000000	0.000759
1784.750000	0.000756
1784.500000	0.000752
1784.250000	0.000750
1784.000000	0.000751
1783.750000	0.000757
1783.500000	0.000763

1783.250000	0.000770
1783.000000	0.000773
1782.750000	0.000774
1782.500000	0.000773
1782.250000	0.000772
1782.000000	0.000772
1781.750000	0.000775
1781.500000	0.000780
1781.250000	0.000785
1781.000000	0.000790
1780.750000	0.000793
1780.500000	0.000793
1780.250000	0.000792
1780.000000	0.000791
1779.750000	0.000792
1779.500000	0.000795
1779.250000	0.000798
1779.000000	0.000801
1778.750000	0.000803
1778.500000	0.000803
1778.250000	0.000804
1778.000000	0.000806
1777.750000	0.000811
1777.500000	0.000820
1777.250000	0.000832
1777.000000	0.000845

1776.750000	0.000858
1776.500000	0.000871
1776.250000	0.000881
1776.000000	0.000886
1775.750000	0.000886
1775.500000	0.000879
1775.250000	0.000870
1775.000000	0.000866
1774.750000	0.000871
1774.500000	0.000888
1774.250000	0.000916
1774.000000	0.000950
1773.750000	0.000985
1773.500000	0.001014
1773.250000	0.001038
1773.000000	0.001056
1772.750000	0.001071
1772.500000	0.001084
1772.250000	0.001093
1772.000000	0.001098
1771.750000	0.001100
1771.500000	0.001101
1771.250000	0.001104
1771.000000	0.001109
1770.750000	0.001116
1770.500000	0.001125

1770.250000	0.001136
1770.000000	0.001150
1769.750000	0.001167
1769.500000	0.001183
1769.250000	0.001197
1769.000000	0.001206
1768.750000	0.001208
1768.500000	0.001204
1768.250000	0.001194
1768.000000	0.001180
1767.750000	0.001168
1767.500000	0.001164
1767.250000	0.001173
1767.000000	0.001195
1766.750000	0.001226
1766.500000	0.001260
1766.250000	0.001293
1766.000000	0.001322
1765.750000	0.001346
1765.500000	0.001366
1765.250000	0.001383
1765.000000	0.001399
1764.750000	0.001414
1764.500000	0.001428
1764.250000	0.001441
1764.000000	0.001455

1763.750000	0.001470
1763.500000	0.001488
1763.250000	0.001509
1763.000000	0.001534
1762.750000	0.001563
1762.500000	0.001596
1762.250000	0.001633
1762.000000	0.001671
1761.750000	0.001704
1761.500000	0.001725
1761.250000	0.001735
1761.000000	0.001740
1760.750000	0.001751
1760.500000	0.001771
1760.250000	0.001797
1760.000000	0.001826
1759.750000	0.001853
1759.500000	0.001877
1759.250000	0.001897
1759.000000	0.001913
1758.750000	0.001928
1758.500000	0.001945
1758.250000	0.001967
1758.000000	0.001993
1757.750000	0.002026
1757.500000	0.002063

1757.250000	0.002104
1757.000000	0.002147
1756.750000	0.002188
1756.500000	0.002227
1756.250000	0.002265
1756.000000	0.002304
1755.750000	0.002346
1755.500000	0.002391
1755.250000	0.002439
1755.000000	0.002486
1754.750000	0.002532
1754.500000	0.002575
1754.250000	0.002614
1754.000000	0.002649
1753.750000	0.002681
1753.500000	0.002711
1753.250000	0.002739
1753.000000	0.002766
1752.750000	0.002793
1752.500000	0.002822
1752.250000	0.002856
1752.000000	0.002904
1751.750000	0.002970
1751.500000	0.003055
1751.250000	0.003148
1751.000000	0.003236

1750.750000	0.003313
1750.500000	0.003382
1750.250000	0.003452
1750.000000	0.003528
1749.750000	0.003608
1749.500000	0.003687
1749.250000	0.003764
1749.000000	0.003838
1748.750000	0.003911
1748.500000	0.003986
1748.250000	0.004066
1748.000000	0.004153
1747.750000	0.004248
1747.500000	0.004351
1747.250000	0.004460
1747.000000	0.004573
1746.750000	0.004687
1746.500000	0.004801
1746.250000	0.004912
1746.000000	0.005019
1745.750000	0.005122
1745.500000	0.005219
1745.250000	0.005312
1745.000000	0.005403
1744.750000	0.005494
1744.500000	0.005590

1744.250000	0.005697
1744.000000	0.005820
1743.750000	0.005963
1743.500000	0.006128
1743.250000	0.006307
1743.000000	0.006490
1742.750000	0.006666
1742.500000	0.006830
1742.250000	0.006979
1742.000000	0.007114
1741.750000	0.007238
1741.500000	0.007354
1741.250000	0.007466
1741.000000	0.007583
1740.750000	0.007719
1740.500000	0.007891
1740.250000	0.008117
1740.000000	0.008397
1739.750000	0.008707
1739.500000	0.009010
1739.250000	0.009272
1739.000000	0.009485
1738.750000	0.009660
1738.500000	0.009813
1738.250000	0.009957
1738.000000	0.010096

1737.750000	0.010235
1737.500000	0.010374
1737.250000	0.010512
1737.000000	0.010645
1736.750000	0.010772
1736.500000	0.010891
1736.250000	0.010998
1736.000000	0.011088
1735.750000	0.011162
1735.500000	0.011227
1735.250000	0.011282
1735.000000	0.011342
1734.750000	0.011469
1734.500000	0.011755
1734.250000	0.012242
1734.000000	0.012858
1733.750000	0.013451
1733.500000	0.013898
1733.250000	0.014175
1733.000000	0.014336
1732.750000	0.014450
1732.500000	0.014551
1732.250000	0.014642
1732.000000	0.014723
1731.750000	0.014801
1731.500000	0.014879

1731.250000	0.014959
1731.000000	0.015049
1730.750000	0.015161
1730.500000	0.015313
1730.250000	0.015510
1730.000000	0.015747
1729.750000	0.016005
1729.500000	0.016264
1729.250000	0.016506
1729.000000	0.016723
1728.750000	0.016916
1728.500000	0.017086
1728.250000	0.017241
1728.000000	0.017385
1727.750000	0.017523
1727.500000	0.017660
1727.250000	0.017800
1727.000000	0.017943
1726.750000	0.018087
1726.500000	0.018230
1726.250000	0.018370
1726.000000	0.018505
1725.750000	0.018636
1725.500000	0.018761
1725.250000	0.018882
1725.000000	0.018999

1724.750000	0.019113
1724.500000	0.019226
1724.250000	0.019343
1724.000000	0.019465
1723.750000	0.019597
1723.500000	0.019739
1723.250000	0.019884
1723.000000	0.020027
1722.750000	0.020160
1722.500000	0.020281
1722.250000	0.020387
1722.000000	0.020478
1721.750000	0.020555
1721.500000	0.020619
1721.250000	0.020671
1721.000000	0.020709
1720.750000	0.020734
1720.500000	0.020743
1720.250000	0.020737
1720.000000	0.020713
1719.750000	0.020674
1719.500000	0.020621
1719.250000	0.020557
1719.000000	0.020489
1718.750000	0.020439
1718.500000	0.020445

1718.250000	0.020552
1718.000000	0.020772
1717.750000	0.021061
1717.500000	0.021337
1717.250000	0.021531
1717.000000	0.021632
1716.750000	0.021672
1716.500000	0.021676
1716.250000	0.021656
1716.000000	0.021614
1715.750000	0.021557
1715.500000	0.021495
1715.250000	0.021443
1715.000000	0.021412
1714.750000	0.021409
1714.500000	0.021424
1714.250000	0.021445
1714.000000	0.021458
1713.750000	0.021457
1713.500000	0.021438
1713.250000	0.021403
1713.000000	0.021350
1712.750000	0.021283
1712.500000	0.021204
1712.250000	0.021117
1712.000000	0.021025

1711.750000	0.020930
1711.500000	0.020835
1711.250000	0.020744
1711.000000	0.020661
1710.750000	0.020590
1710.500000	0.020530
1710.250000	0.020483
1710.000000	0.020444
1709.750000	0.020411
1709.500000	0.020379
1709.250000	0.020343
1709.000000	0.020299
1708.750000	0.020244
1708.500000	0.020175
1708.250000	0.020092
1708.000000	0.019996
1707.750000	0.019891
1707.500000	0.019782
1707.250000	0.019679
1707.000000	0.019592
1706.750000	0.019530
1706.500000	0.019492
1706.250000	0.019476
1706.000000	0.019469
1705.750000	0.019461
1705.500000	0.019446

1705.250000	0.019420
1705.000000	0.019384
1704.750000	0.019341
1704.500000	0.019291
1704.250000	0.019236
1704.000000	0.019178
1703.750000	0.019114
1703.500000	0.019042
1703.250000	0.018959
1703.000000	0.018865
1702.750000	0.018759
1702.500000	0.018643
1702.250000	0.018507
1702.000000	0.018344
1701.750000	0.018161
1701.500000	0.017963
1701.250000	0.017719
1701.000000	0.017421
1700.750000	0.017193
1700.500000	0.017230
1700.250000	0.017564
1700.000000	0.017966
1699.750000	0.018170
1699.500000	0.018131
1699.250000	0.017983
1699.000000	0.017832

1698.750000	0.017681
1698.500000	0.017512
1698.250000	0.017333
1698.000000	0.017152
1697.750000	0.016965
1697.500000	0.016764
1697.250000	0.016549
1697.000000	0.016317
1696.750000	0.016069
1696.500000	0.015818
1696.250000	0.015599
1696.000000	0.015446
1695.750000	0.015364
1695.500000	0.015311
1695.250000	0.015239
1695.000000	0.015138
1694.750000	0.015020
1694.500000	0.014897
1694.250000	0.014761
1694.000000	0.014604
1693.750000	0.014426
1693.500000	0.014230
1693.250000	0.014023
1693.000000	0.013809
1692.750000	0.013596
1692.500000	0.013388

1692.250000	0.013187
1692.000000	0.012994
1691.750000	0.012806
1691.500000	0.012620
1691.250000	0.012433
1691.000000	0.012244
1690.750000	0.012052
1690.500000	0.011856
1690.250000	0.011660
1690.000000	0.011467
1689.750000	0.011281
1689.500000	0.011102
1689.250000	0.010928
1689.000000	0.010756
1688.750000	0.010582
1688.500000	0.010404
1688.250000	0.010225
1688.000000	0.010049
1687.750000	0.009883
1687.500000	0.009730
1687.250000	0.009593
1687.000000	0.009470
1686.750000	0.009357
1686.500000	0.009245
1686.250000	0.009124
1686.000000	0.008985

1685.750000	0.008823
1685.500000	0.008636
1685.250000	0.008426
1685.000000	0.008209
1684.750000	0.008031
1684.500000	0.007939
1684.250000	0.007921
1684.000000	0.007908
1683.750000	0.007846
1683.500000	0.007748
1683.250000	0.007664
1683.000000	0.007613
1682.750000	0.007574
1682.500000	0.007527
1682.250000	0.007466
1682.000000	0.007397
1681.750000	0.007319
1681.500000	0.007232
1681.250000	0.007137
1681.000000	0.007035
1680.750000	0.006928
1680.500000	0.006819
1680.250000	0.006714
1680.000000	0.006619
1679.750000	0.006539
1679.500000	0.006476

1679.250000	0.006426
1679.000000	0.006386
1678.750000	0.006349
1678.500000	0.006309
1678.250000	0.006263
1678.000000	0.006210
1677.750000	0.006152
1677.500000	0.006090
1677.250000	0.006027
1677.000000	0.005965
1676.750000	0.005904
1676.500000	0.005842
1676.250000	0.005778
1676.000000	0.005715
1675.750000	0.005662
1675.500000	0.005626
1675.250000	0.005602
1675.000000	0.005573
1674.750000	0.005527
1674.500000	0.005465
1674.250000	0.005399
1674.000000	0.005334
1673.750000	0.005271
1673.500000	0.005206
1673.250000	0.005139
1673.000000	0.005071

1672.750000	0.005001
1672.500000	0.004930
1672.250000	0.004861
1672.000000	0.004795
1671.750000	0.004734
1671.500000	0.004676
1671.250000	0.004624
1671.000000	0.004578
1670.750000	0.004539
1670.500000	0.004507
1670.250000	0.004477
1670.000000	0.004446
1669.750000	0.004409
1669.500000	0.004364
1669.250000	0.004307
1669.000000	0.004234
1668.750000	0.004149
1668.500000	0.004060
1668.250000	0.003980
1668.000000	0.003915
1667.750000	0.003864
1667.500000	0.003820
1667.250000	0.003779
1667.000000	0.003737
1666.750000	0.003694
1666.500000	0.003652

1666.250000	0.003610
1666.000000	0.003569
1665.750000	0.003529
1665.500000	0.003489
1665.250000	0.003448
1665.000000	0.003408
1664.750000	0.003368
1664.500000	0.003330
1664.250000	0.003294
1664.000000	0.003260
1663.750000	0.003226
1663.500000	0.003195
1663.250000	0.003167
1663.000000	0.003143
1662.750000	0.003120
1662.500000	0.003094
1662.250000	0.003063
1662.000000	0.003029
1661.750000	0.002997
1661.500000	0.002967
1661.250000	0.002941
1661.000000	0.002914
1660.750000	0.002887
1660.500000	0.002859
1660.250000	0.002830
1660.000000	0.002800

1659.750000	0.002773
1659.500000	0.002747
1659.250000	0.002723
1659.000000	0.002701
1658.750000	0.002679
1658.500000	0.002659
1658.250000	0.002640
1658.000000	0.002623
1657.750000	0.002608
1657.500000	0.002593
1657.250000	0.002578
1657.000000	0.002565
1656.750000	0.002554
1656.500000	0.002543
1656.250000	0.002535
1656.000000	0.002528
1655.750000	0.002524
1655.500000	0.002522
1655.250000	0.002513
1655.000000	0.002493
1654.750000	0.002465
1654.500000	0.002430
1654.250000	0.002375
1654.000000	0.002293
1653.750000	0.002226
1653.500000	0.002247

1653.250000	0.002367
1653.000000	0.002497
1652.750000	0.002543
1652.500000	0.002500
1652.250000	0.002437
1652.000000	0.002403
1651.750000	0.002399
1651.500000	0.002410
1651.250000	0.002430
1651.000000	0.002457
1650.750000	0.002486
1650.500000	0.002509
1650.250000	0.002523
1650.000000	0.002530
1649.750000	0.002534
1649.500000	0.002539
1649.250000	0.002547
1649.000000	0.002556
1648.750000	0.002565
1648.500000	0.002575
1648.250000	0.002585
1648.000000	0.002595
1647.750000	0.002608
1647.500000	0.002626
1647.250000	0.002654
1647.000000	0.002693

1646.750000	0.002741
1646.500000	0.002794
1646.250000	0.002842
1646.000000	0.002878
1645.750000	0.002897
1645.500000	0.002905
1645.250000	0.002910
1645.000000	0.002919
1644.750000	0.002934
1644.500000	0.002954
1644.250000	0.002975
1644.000000	0.002993
1643.750000	0.003007
1643.500000	0.003017
1643.250000	0.003024
1643.000000	0.003030
1642.750000	0.003038
1642.500000	0.003049
1642.250000	0.003064
1642.000000	0.003083
1641.750000	0.003103
1641.500000	0.003126
1641.250000	0.003152
1641.000000	0.003179
1640.750000	0.003208
1640.500000	0.003235

1640.250000	0.003260
1640.000000	0.003282
1639.750000	0.003300
1639.500000	0.003317
1639.250000	0.003334
1639.000000	0.003349
1638.750000	0.003362
1638.500000	0.003372
1638.250000	0.003378
1638.000000	0.003377
1637.750000	0.003371
1637.500000	0.003360
1637.250000	0.003350
1637.000000	0.003342
1636.750000	0.003336
1636.500000	0.003330
1636.250000	0.003326
1636.000000	0.003330
1635.750000	0.003344
1635.500000	0.003354
1635.250000	0.003344
1635.000000	0.003307
1634.750000	0.003255
1634.500000	0.003210
1634.250000	0.003180
1634.000000	0.003162

1633.750000	0.003144
1633.500000	0.003119
1633.250000	0.003087
1633.000000	0.003053
1632.750000	0.003018
1632.500000	0.002984
1632.250000	0.002952
1632.000000	0.002924
1631.750000	0.002897
1631.500000	0.002872
1631.250000	0.002846
1631.000000	0.002820
1630.750000	0.002792
1630.500000	0.002763
1630.250000	0.002734
1630.000000	0.002704
1629.750000	0.002676
1629.500000	0.002651
1629.250000	0.002627
1629.000000	0.002605
1628.750000	0.002583
1628.500000	0.002559
1628.250000	0.002534
1628.000000	0.002504
1627.750000	0.002471
1627.500000	0.002437

1627.250000	0.002405
1627.000000	0.002377
1626.750000	0.002354
1626.500000	0.002334
1626.250000	0.002313
1626.000000	0.002292
1625.750000	0.002268
1625.500000	0.002244
1625.250000	0.002223
1625.000000	0.002205
1624.750000	0.002191
1624.500000	0.002181
1624.250000	0.002175
1624.000000	0.002171
1623.750000	0.002165
1623.500000	0.002151
1623.250000	0.002122
1623.000000	0.002079
1622.750000	0.002030
1622.500000	0.001985
1622.250000	0.001951
1622.000000	0.001928
1621.750000	0.001912
1621.500000	0.001900
1621.250000	0.001889
1621.000000	0.001877

1620.750000	0.001863
1620.500000	0.001850
1620.250000	0.001838
1620.000000	0.001828
1619.750000	0.001821
1619.500000	0.001816
1619.250000	0.001814
1619.000000	0.001814
1618.750000	0.001817
1618.500000	0.001823
1618.250000	0.001830
1618.000000	0.001835
1617.750000	0.001837
1617.500000	0.001838
1617.250000	0.001848
1617.000000	0.001872
1616.750000	0.001906
1616.500000	0.001928
1616.250000	0.001928
1616.000000	0.001913
1615.750000	0.001903
1615.500000	0.001910
1615.250000	0.001931
1615.000000	0.001958
1614.750000	0.001982
1614.500000	0.002002

1614.250000	0.002017
1614.000000	0.002030
1613.750000	0.002044
1613.500000	0.002062
1613.250000	0.002085
1613.000000	0.002111
1612.750000	0.002139
1612.500000	0.002167
1612.250000	0.002195
1612.000000	0.002225
1611.750000	0.002256
1611.500000	0.002291
1611.250000	0.002328
1611.000000	0.002367
1610.750000	0.002407
1610.500000	0.002445
1610.250000	0.002482
1610.000000	0.002517
1609.750000	0.002552
1609.500000	0.002587
1609.250000	0.002624
1609.000000	0.002664
1608.750000	0.002707
1608.500000	0.002754
1608.250000	0.002803
1608.000000	0.002854

1607.750000	0.002907
1607.500000	0.002962
1607.250000	0.003019
1607.000000	0.003081
1606.750000	0.003148
1606.500000	0.003219
1606.250000	0.003295
1606.000000	0.003374
1605.750000	0.003455
1605.500000	0.003537
1605.250000	0.003618
1605.000000	0.003697
1604.750000	0.003774
1604.500000	0.003848
1604.250000	0.003920
1604.000000	0.003989
1603.750000	0.004054
1603.500000	0.004116
1603.250000	0.004172
1603.000000	0.004222
1602.750000	0.004265
1602.500000	0.004300
1602.250000	0.004326
1602.000000	0.004345
1601.750000	0.004356
1601.500000	0.004357

1601.250000	0.004349
1601.000000	0.004332
1600.750000	0.004304
1600.500000	0.004267
1600.250000	0.004222
1600.000000	0.004169
1599.750000	0.004109
1599.500000	0.004043
1599.250000	0.003969
1599.000000	0.003889
1598.750000	0.003801
1598.500000	0.003707
1598.250000	0.003610
1598.000000	0.003510
1597.750000	0.003409
1597.500000	0.003307
1597.250000	0.003204
1597.000000	0.003100
1596.750000	0.002996
1596.500000	0.002892
1596.250000	0.002789
1596.000000	0.002688
1595.750000	0.002590
1595.500000	0.002496
1595.250000	0.002405
1595.000000	0.002318

1594.750000	0.002234
1594.500000	0.002153
1594.250000	0.002077
1594.000000	0.002006
1593.750000	0.001940
1593.500000	0.001881
1593.250000	0.001827
1593.000000	0.001779
1592.750000	0.001735
1592.500000	0.001694
1592.250000	0.001655
1592.000000	0.001618
1591.750000	0.001582
1591.500000	0.001547
1591.250000	0.001515
1591.000000	0.001485
1590.750000	0.001457
1590.500000	0.001431
1590.250000	0.001407
1590.000000	0.001384
1589.750000	0.001362
1589.500000	0.001342
1589.250000	0.001324
1589.000000	0.001310
1588.750000	0.001299
1588.500000	0.001291

1588.250000	0.001287
1588.000000	0.001284
1587.750000	0.001283
1587.500000	0.001281
1587.250000	0.001279
1587.000000	0.001276
1586.750000	0.001273
1586.500000	0.001270
1586.250000	0.001268
1586.000000	0.001268
1585.750000	0.001271
1585.500000	0.001275
1585.250000	0.001282
1585.000000	0.001289
1584.750000	0.001297
1584.500000	0.001304
1584.250000	0.001310
1584.000000	0.001315
1583.750000	0.001319
1583.500000	0.001322
1583.250000	0.001326
1583.000000	0.001330
1582.750000	0.001337
1582.500000	0.001346
1582.250000	0.001356
1582.000000	0.001366

1581.750000	0.001377
1581.500000	0.001388
1581.250000	0.001398
1581.000000	0.001406
1580.750000	0.001414
1580.500000	0.001420
1580.250000	0.001427
1580.000000	0.001435
1579.750000	0.001444
1579.500000	0.001455
1579.250000	0.001466
1579.000000	0.001477
1578.750000	0.001486
1578.500000	0.001495
1578.250000	0.001502
1578.000000	0.001509
1577.750000	0.001515
1577.500000	0.001517
1577.250000	0.001513
1577.000000	0.001505
1576.750000	0.001498
1576.500000	0.001500
1576.250000	0.001505
1576.000000	0.001498
1575.750000	0.001474
1575.500000	0.001444

1575.250000	0.001425
1575.000000	0.001427
1574.750000	0.001445
1574.500000	0.001467
1574.250000	0.001486
1574.000000	0.001498
1573.750000	0.001503
1573.500000	0.001502
1573.250000	0.001498
1573.000000	0.001494
1572.750000	0.001491
1572.500000	0.001488
1572.250000	0.001485
1572.000000	0.001481
1571.750000	0.001478
1571.500000	0.001476
1571.250000	0.001478
1571.000000	0.001483
1570.750000	0.001491
1570.500000	0.001500
1570.250000	0.001513
1570.000000	0.001536
1569.750000	0.001565
1569.500000	0.001589
1569.250000	0.001597
1569.000000	0.001590

1568.750000	0.001578
1568.500000	0.001571
1568.250000	0.001574
1568.000000	0.001583
1567.750000	0.001592
1567.500000	0.001600
1567.250000	0.001604
1567.000000	0.001607
1566.750000	0.001610
1566.500000	0.001614
1566.250000	0.001621
1566.000000	0.001631
1565.750000	0.001646
1565.500000	0.001663
1565.250000	0.001683
1565.000000	0.001704
1564.750000	0.001723
1564.500000	0.001740
1564.250000	0.001756
1564.000000	0.001774
1563.750000	0.001794
1563.500000	0.001817
1563.250000	0.001839
1563.000000	0.001859
1562.750000	0.001876
1562.500000	0.001891

1562.250000	0.001906
1562.000000	0.001920
1561.750000	0.001934
1561.500000	0.001945
1561.250000	0.001952
1561.000000	0.001953
1560.750000	0.001950
1560.500000	0.001946
1560.250000	0.001947
1560.000000	0.001965
1559.750000	0.002017
1559.500000	0.002122
1559.250000	0.002278
1559.000000	0.002452
1558.750000	0.002594
1558.500000	0.002667
1558.250000	0.002674
1558.000000	0.002653
1557.750000	0.002645
1557.500000	0.002666
1557.250000	0.002703
1557.000000	0.002740
1556.750000	0.002767
1556.500000	0.002785
1556.250000	0.002794
1556.000000	0.002797

1555.750000	0.002796
1555.500000	0.002792
1555.250000	0.002792
1555.000000	0.002803
1554.750000	0.002838
1554.500000	0.002901
1554.250000	0.002984
1554.000000	0.003069
1553.750000	0.003142
1553.500000	0.003199
1553.250000	0.003247
1553.000000	0.003291
1552.750000	0.003330
1552.500000	0.003363
1552.250000	0.003392
1552.000000	0.003417
1551.750000	0.003438
1551.500000	0.003456
1551.250000	0.003473
1551.000000	0.003489
1550.750000	0.003509
1550.500000	0.003536
1550.250000	0.003575
1550.000000	0.003625
1549.750000	0.003683
1549.500000	0.003745

1549.250000	0.003809
1549.000000	0.003875
1548.750000	0.003942
1548.500000	0.004007
1548.250000	0.004066
1548.000000	0.004117
1547.750000	0.004159
1547.500000	0.004196
1547.250000	0.004228
1547.000000	0.004259
1546.750000	0.004288
1546.500000	0.004318
1546.250000	0.004351
1546.000000	0.004389
1545.750000	0.004438
1545.500000	0.004502
1545.250000	0.004579
1545.000000	0.004657
1544.750000	0.004726
1544.500000	0.004781
1544.250000	0.004824
1544.000000	0.004861
1543.750000	0.004894
1543.500000	0.004927
1543.250000	0.004958
1543.000000	0.004992

1542.750000	0.005037
1542.500000	0.005101
1542.250000	0.005188
1542.000000	0.005289
1541.750000	0.005391
1541.500000	0.005480
1541.250000	0.005549
1541.000000	0.005599
1540.750000	0.005629
1540.500000	0.005641
1540.250000	0.005651
1540.000000	0.005685
1539.750000	0.005763
1539.500000	0.005871
1539.250000	0.005972
1539.000000	0.006037
1538.750000	0.006066
1538.500000	0.006082
1538.250000	0.006109
1538.000000	0.006150
1537.750000	0.006197
1537.500000	0.006240
1537.250000	0.006277
1537.000000	0.006307
1536.750000	0.006329
1536.500000	0.006343

1536.250000	0.006349
1536.000000	0.006354
1535.750000	0.006359
1535.500000	0.006367
1535.250000	0.006377
1535.000000	0.006390
1534.750000	0.006403
1534.500000	0.006421
1534.250000	0.006450
1534.000000	0.006503
1533.750000	0.006580
1533.500000	0.006667
1533.250000	0.006746
1533.000000	0.006804
1532.750000	0.006840
1532.500000	0.006862
1532.250000	0.006876
1532.000000	0.006884
1531.750000	0.006887
1531.500000	0.006888
1531.250000	0.006890
1531.000000	0.006896
1530.750000	0.006906
1530.500000	0.006920
1530.250000	0.006936
1530.000000	0.006951

1529.750000	0.006961
1529.500000	0.006967
1529.250000	0.006968
1529.000000	0.006967
1528.750000	0.006966
1528.500000	0.006966
1528.250000	0.006970
1528.000000	0.006976
1527.750000	0.006984
1527.500000	0.006992
1527.250000	0.006999
1527.000000	0.007006
1526.750000	0.007011
1526.500000	0.007016
1526.250000	0.007020
1526.000000	0.007025
1525.750000	0.007032
1525.500000	0.007040
1525.250000	0.007048
1525.000000	0.007053
1524.750000	0.007054
1524.500000	0.007053
1524.250000	0.007052
1524.000000	0.007052
1523.750000	0.007051
1523.500000	0.007045

1523.250000	0.007032
1523.000000	0.007014
1522.750000	0.006992
1522.500000	0.006970
1522.250000	0.006954
1522.000000	0.006947
1521.750000	0.006957
1521.500000	0.006986
1521.250000	0.007021
1521.000000	0.007046
1520.750000	0.007047
1520.500000	0.007030
1520.250000	0.007012
1520.000000	0.007007
1519.750000	0.007016
1519.500000	0.007031
1519.250000	0.007044
1519.000000	0.007047
1518.750000	0.007040
1518.500000	0.007020
1518.250000	0.006989
1518.000000	0.006951
1517.750000	0.006921
1517.500000	0.006919
1517.250000	0.006958
1517.000000	0.007032

1516.750000	0.007122
1516.500000	0.007208
1516.250000	0.007280
1516.000000	0.007338
1515.750000	0.007388
1515.500000	0.007434
1515.250000	0.007477
1515.000000	0.007519
1514.750000	0.007562
1514.500000	0.007607
1514.250000	0.007653
1514.000000	0.007698
1513.750000	0.007741
1513.500000	0.007782
1513.250000	0.007822
1513.000000	0.007863
1512.750000	0.007910
1512.500000	0.007964
1512.250000	0.008023
1512.000000	0.008080
1511.750000	0.008127
1511.500000	0.008162
1511.250000	0.008183
1511.000000	0.008189
1510.750000	0.008180
1510.500000	0.008156

1510.250000	0.008119
1510.000000	0.008073
1509.750000	0.008021
1509.500000	0.007970
1509.250000	0.007922
1509.000000	0.007877
1508.750000	0.007833
1508.500000	0.007784
1508.250000	0.007725
1508.000000	0.007649
1507.750000	0.007557
1507.500000	0.007452
1507.250000	0.007344
1507.000000	0.007238
1506.750000	0.007123
1506.500000	0.006980
1506.250000	0.006804
1506.000000	0.006615
1505.750000	0.006442
1505.500000	0.006300
1505.250000	0.006186
1505.000000	0.006087
1504.750000	0.005993
1504.500000	0.005903
1504.250000	0.005815
1504.000000	0.005726

1503.750000	0.005632
1503.500000	0.005531
1503.250000	0.005422
1503.000000	0.005304
1502.750000	0.005180
1502.500000	0.005052
1502.250000	0.004925
1502.000000	0.004802
1501.750000	0.004685
1501.500000	0.004577
1501.250000	0.004479
1501.000000	0.004392
1500.750000	0.004315
1500.500000	0.004248
1500.250000	0.004186
1500.000000	0.004125
1499.750000	0.004061
1499.500000	0.003990
1499.250000	0.003911
1499.000000	0.003827
1498.750000	0.003743
1498.500000	0.003665
1498.250000	0.003601
1498.000000	0.003552
1497.750000	0.003515
1497.500000	0.003483

1497.250000	0.003449
1497.000000	0.003407
1496.750000	0.003355
1496.500000	0.003297
1496.250000	0.003237
1496.000000	0.003180
1495.750000	0.003132
1495.500000	0.003099
1495.250000	0.003080
1495.000000	0.003075
1494.750000	0.003075
1494.500000	0.003075
1494.250000	0.003070
1494.000000	0.003060
1493.750000	0.003046
1493.500000	0.003031
1493.250000	0.003017
1493.000000	0.003008
1492.750000	0.003002
1492.500000	0.003001
1492.250000	0.003001
1492.000000	0.003002
1491.750000	0.003002
1491.500000	0.003002
1491.250000	0.003004
1491.000000	0.003011

1490.750000	0.003025
1490.500000	0.003044
1490.250000	0.003064
1490.000000	0.003074
1489.750000	0.003072
1489.500000	0.003062
1489.250000	0.003055
1489.000000	0.003057
1488.750000	0.003069
1488.500000	0.003090
1488.250000	0.003113
1488.000000	0.003136
1487.750000	0.003155
1487.500000	0.003171
1487.250000	0.003183
1487.000000	0.003190
1486.750000	0.003194
1486.500000	0.003196
1486.250000	0.003197
1486.000000	0.003200
1485.750000	0.003203
1485.500000	0.003207
1485.250000	0.003211
1485.000000	0.003215
1484.750000	0.003222
1484.500000	0.003229

1484.250000	0.003239
1484.000000	0.003249
1483.750000	0.003261
1483.500000	0.003273
1483.250000	0.003284
1483.000000	0.003295
1482.750000	0.003305
1482.500000	0.003313
1482.250000	0.003321
1482.000000	0.003327
1481.750000	0.003334
1481.500000	0.003344
1481.250000	0.003357
1481.000000	0.003375
1480.750000	0.003397
1480.500000	0.003421
1480.250000	0.003445
1480.000000	0.003469
1479.750000	0.003490
1479.500000	0.003508
1479.250000	0.003522
1479.000000	0.003535
1478.750000	0.003546
1478.500000	0.003556
1478.250000	0.003568
1478.000000	0.003580

1477.750000	0.003593
1477.500000	0.003606
1477.250000	0.003620
1477.000000	0.003639
1476.750000	0.003664
1476.500000	0.003697
1476.250000	0.003733
1476.000000	0.003770
1475.750000	0.003805
1475.500000	0.003838
1475.250000	0.003873
1475.000000	0.003907
1474.750000	0.003937
1474.500000	0.003960
1474.250000	0.003974
1474.000000	0.003985
1473.750000	0.003998
1473.500000	0.004023
1473.250000	0.004067
1473.000000	0.004136
1472.750000	0.004226
1472.500000	0.004326
1472.250000	0.004419
1472.000000	0.004494
1471.750000	0.004549
1471.500000	0.004589

1471.250000	0.004624
1471.000000	0.004659
1470.750000	0.004695
1470.500000	0.004731
1470.250000	0.004765
1470.000000	0.004797
1469.750000	0.004827
1469.500000	0.004855
1469.250000	0.004881
1469.000000	0.004908
1468.750000	0.004936
1468.500000	0.004965
1468.250000	0.004995
1468.000000	0.005025
1467.750000	0.005052
1467.500000	0.005076
1467.250000	0.005097
1467.000000	0.005116
1466.750000	0.005133
1466.500000	0.005149
1466.250000	0.005164
1466.000000	0.005180
1465.750000	0.005203
1465.500000	0.005239
1465.250000	0.005294
1465.000000	0.005366

1464.750000	0.005443
1464.500000	0.005513
1464.250000	0.005564
1464.000000	0.005599
1463.750000	0.005622
1463.500000	0.005641
1463.250000	0.005656
1463.000000	0.005669
1462.750000	0.005680
1462.500000	0.005690
1462.250000	0.005700
1462.000000	0.005712
1461.750000	0.005724
1461.500000	0.005737
1461.250000	0.005750
1461.000000	0.005762
1460.750000	0.005772
1460.500000	0.005781
1460.250000	0.005788
1460.000000	0.005794
1459.750000	0.005799
1459.500000	0.005806
1459.250000	0.005814
1459.000000	0.005826
1458.750000	0.005841
1458.500000	0.005860

1458.250000	0.005881
1458.000000	0.005902
1457.750000	0.005927
1457.500000	0.005970
1457.250000	0.006041
1457.000000	0.006141
1456.750000	0.006251
1456.500000	0.006342
1456.250000	0.006396
1456.000000	0.006415
1455.750000	0.006416
1455.500000	0.006413
1455.250000	0.006410
1455.000000	0.006406
1454.750000	0.006401
1454.500000	0.006396
1454.250000	0.006390
1454.000000	0.006385
1453.750000	0.006379
1453.500000	0.006373
1453.250000	0.006366
1453.000000	0.006357
1452.750000	0.006347
1452.500000	0.006336
1452.250000	0.006326
1452.000000	0.006318

1451.750000	0.006314
1451.500000	0.006312
1451.250000	0.006312
1451.000000	0.006310
1450.750000	0.006307
1450.500000	0.006300
1450.250000	0.006288
1450.000000	0.006273
1449.750000	0.006254
1449.500000	0.006232
1449.250000	0.006208
1449.000000	0.006182
1448.750000	0.006157
1448.500000	0.006135
1448.250000	0.006118
1448.000000	0.006106
1447.750000	0.006097
1447.500000	0.006089
1447.250000	0.006081
1447.000000	0.006072
1446.750000	0.006062
1446.500000	0.006049
1446.250000	0.006033
1446.000000	0.006013
1445.750000	0.005989
1445.500000	0.005963

1445.250000	0.005936
1445.000000	0.005909
1444.750000	0.005884
1444.500000	0.005860
1444.250000	0.005839
1444.000000	0.005821
1443.750000	0.005806
1443.500000	0.005793
1443.250000	0.005782
1443.000000	0.005772
1442.750000	0.005761
1442.500000	0.005749
1442.250000	0.005733
1442.000000	0.005714
1441.750000	0.005694
1441.500000	0.005673
1441.250000	0.005652
1441.000000	0.005632
1440.750000	0.005614
1440.500000	0.005598
1440.250000	0.005584
1440.000000	0.005570
1439.750000	0.005555
1439.500000	0.005540
1439.250000	0.005522
1439.000000	0.005504

1438.750000	0.005484
1438.500000	0.005464
1438.250000	0.005443
1438.000000	0.005420
1437.750000	0.005391
1437.500000	0.005359
1437.250000	0.005330
1437.000000	0.005315
1436.750000	0.005312
1436.500000	0.005310
1436.250000	0.005296
1436.000000	0.005268
1435.750000	0.005235
1435.500000	0.005204
1435.250000	0.005177
1435.000000	0.005150
1434.750000	0.005119
1434.500000	0.005083
1434.250000	0.005041
1434.000000	0.004996
1433.750000	0.004949
1433.500000	0.004903
1433.250000	0.004859
1433.000000	0.004818
1432.750000	0.004780
1432.500000	0.004745

1432.250000	0.004713
1432.000000	0.004686
1431.750000	0.004662
1431.500000	0.004639
1431.250000	0.004616
1431.000000	0.004591
1430.750000	0.004562
1430.500000	0.004532
1430.250000	0.004501
1430.000000	0.004471
1429.750000	0.004444
1429.500000	0.004422
1429.250000	0.004403
1429.000000	0.004388
1428.750000	0.004375
1428.500000	0.004362
1428.250000	0.004349
1428.000000	0.004336
1427.750000	0.004322
1427.500000	0.004309
1427.250000	0.004298
1427.000000	0.004288
1426.750000	0.004279
1426.500000	0.004272
1426.250000	0.004265
1426.000000	0.004260

1425.750000	0.004255
1425.500000	0.004251
1425.250000	0.004247
1425.000000	0.004243
1424.750000	0.004242
1424.500000	0.004244
1424.250000	0.004252
1424.000000	0.004265
1423.750000	0.004283
1423.500000	0.004305
1423.250000	0.004328
1423.000000	0.004352
1422.750000	0.004376
1422.500000	0.004396
1422.250000	0.004412
1422.000000	0.004422
1421.750000	0.004428
1421.500000	0.004430
1421.250000	0.004430
1421.000000	0.004429
1420.750000	0.004428
1420.500000	0.004423
1420.250000	0.004419
1420.000000	0.004421
1419.750000	0.004440
1419.500000	0.004480

1419.250000	0.004531
1419.000000	0.004578
1418.750000	0.004608
1418.500000	0.004623
1418.250000	0.004631
1418.000000	0.004638
1417.750000	0.004643
1417.500000	0.004644
1417.250000	0.004641
1417.000000	0.004638
1416.750000	0.004635
1416.500000	0.004636
1416.250000	0.004641
1416.000000	0.004649
1415.750000	0.004660
1415.500000	0.004673
1415.250000	0.004685
1415.000000	0.004697
1414.750000	0.004709
1414.500000	0.004720
1414.250000	0.004730
1414.000000	0.004740
1413.750000	0.004749
1413.500000	0.004756
1413.250000	0.004763
1413.000000	0.004769

1412.750000	0.004775
1412.500000	0.004782
1412.250000	0.004790
1412.000000	0.004801
1411.750000	0.004814
1411.500000	0.004829
1411.250000	0.004848
1411.000000	0.004868
1410.750000	0.004889
1410.500000	0.004911
1410.250000	0.004931
1410.000000	0.004951
1409.750000	0.004969
1409.500000	0.004987
1409.250000	0.005004
1409.000000	0.005021
1408.750000	0.005038
1408.500000	0.005054
1408.250000	0.005070
1408.000000	0.005085
1407.750000	0.005099
1407.500000	0.005112
1407.250000	0.005124
1407.000000	0.005135
1406.750000	0.005145
1406.500000	0.005154

1406.250000	0.005162
1406.000000	0.005170
1405.750000	0.005180
1405.500000	0.005192
1405.250000	0.005207
1405.000000	0.005224
1404.750000	0.005241
1404.500000	0.005257
1404.250000	0.005272
1404.000000	0.005285
1403.750000	0.005296
1403.500000	0.005306
1403.250000	0.005312
1403.000000	0.005316
1402.750000	0.005317
1402.500000	0.005317
1402.250000	0.005314
1402.000000	0.005310
1401.750000	0.005304
1401.500000	0.005297
1401.250000	0.005289
1401.000000	0.005281
1400.750000	0.005273
1400.500000	0.005264
1400.250000	0.005255
1400.000000	0.005247

1399.750000	0.005240
1399.500000	0.005237
1399.250000	0.005238
1399.000000	0.005241
1398.750000	0.005244
1398.500000	0.005244
1398.250000	0.005241
1398.000000	0.005236
1397.750000	0.005228
1397.500000	0.005219
1397.250000	0.005207
1397.000000	0.005194
1396.750000	0.005179
1396.500000	0.005163
1396.250000	0.005148
1396.000000	0.005134
1395.750000	0.005123
1395.500000	0.005120
1395.250000	0.005126
1395.000000	0.005144
1394.750000	0.005172
1394.500000	0.005205
1394.250000	0.005237
1394.000000	0.005265
1393.750000	0.005286
1393.500000	0.005303

1393.250000	0.005316
1393.000000	0.005327
1392.750000	0.005336
1392.500000	0.005344
1392.250000	0.005351
1392.000000	0.005357
1391.750000	0.005363
1391.500000	0.005370
1391.250000	0.005379
1391.000000	0.005390
1390.750000	0.005403
1390.500000	0.005418
1390.250000	0.005433
1390.000000	0.005449
1389.750000	0.005465
1389.500000	0.005481
1389.250000	0.005496
1389.000000	0.005508
1388.750000	0.005519
1388.500000	0.005528
1388.250000	0.005540
1388.000000	0.005556
1387.750000	0.005581
1387.500000	0.005613
1387.250000	0.005649
1387.000000	0.005685

1386.750000	0.005718
1386.500000	0.005747
1386.250000	0.005775
1386.000000	0.005802
1385.750000	0.005828
1385.500000	0.005854
1385.250000	0.005879
1385.000000	0.005903
1384.750000	0.005927
1384.500000	0.005950
1384.250000	0.005972
1384.000000	0.005994
1383.750000	0.006013
1383.500000	0.006030
1383.250000	0.006043
1383.000000	0.006055
1382.750000	0.006065
1382.500000	0.006074
1382.250000	0.006084
1382.000000	0.006096
1381.750000	0.006108
1381.500000	0.006121
1381.250000	0.006133
1381.000000	0.006143
1380.750000	0.006150
1380.500000	0.006153

1380.250000	0.006152
1380.000000	0.006147
1379.750000	0.006138
1379.500000	0.006127
1379.250000	0.006113
1379.000000	0.006098
1378.750000	0.006082
1378.500000	0.006065
1378.250000	0.006047
1378.000000	0.006028
1377.750000	0.006010
1377.500000	0.005991
1377.250000	0.005971
1377.000000	0.005950
1376.750000	0.005926
1376.500000	0.005899
1376.250000	0.005867
1376.000000	0.005831
1375.750000	0.005793
1375.500000	0.005753
1375.250000	0.005713
1375.000000	0.005676
1374.750000	0.005640
1374.500000	0.005607
1374.250000	0.005574
1374.000000	0.005541

1373.750000	0.005507
1373.500000	0.005474
1373.250000	0.005443
1373.000000	0.005415
1372.750000	0.005392
1372.500000	0.005371
1372.250000	0.005353
1372.000000	0.005335
1371.750000	0.005318
1371.500000	0.005301
1371.250000	0.005283
1371.000000	0.005265
1370.750000	0.005247
1370.500000	0.005228
1370.250000	0.005210
1370.000000	0.005191
1369.750000	0.005172
1369.500000	0.005153
1369.250000	0.005136
1369.000000	0.005120
1368.750000	0.005106
1368.500000	0.005095
1368.250000	0.005087
1368.000000	0.005081
1367.750000	0.005079
1367.500000	0.005081

1367.250000	0.005086
1367.000000	0.005095
1366.750000	0.005106
1366.500000	0.005119
1366.250000	0.005132
1366.000000	0.005142
1365.750000	0.005146
1365.500000	0.005144
1365.250000	0.005135
1365.000000	0.005119
1364.750000	0.005096
1364.500000	0.005067
1364.250000	0.005033
1364.000000	0.004996
1363.750000	0.004957
1363.500000	0.004921
1363.250000	0.004889
1363.000000	0.004863
1362.750000	0.004842
1362.500000	0.004827
1362.250000	0.004814
1362.000000	0.004802
1361.750000	0.004788
1361.500000	0.004768
1361.250000	0.004743
1361.000000	0.004713

1360.750000	0.004680
1360.500000	0.004648
1360.250000	0.004616
1360.000000	0.004587
1359.750000	0.004558
1359.500000	0.004528
1359.250000	0.004497
1359.000000	0.004464
1358.750000	0.004430
1358.500000	0.004398
1358.250000	0.004367
1358.000000	0.004341
1357.750000	0.004319
1357.500000	0.004299
1357.250000	0.004283
1357.000000	0.004269
1356.750000	0.004256
1356.500000	0.004244
1356.250000	0.004232
1356.000000	0.004219
1355.750000	0.004207
1355.500000	0.004195
1355.250000	0.004183
1355.000000	0.004173
1354.750000	0.004165
1354.500000	0.004159

1354.250000	0.004154
1354.000000	0.004150
1353.750000	0.004145
1353.500000	0.004139
1353.250000	0.004133
1353.000000	0.004126
1352.750000	0.004119
1352.500000	0.004112
1352.250000	0.004104
1352.000000	0.004096
1351.750000	0.004087
1351.500000	0.004077
1351.250000	0.004067
1351.000000	0.004056
1350.750000	0.004045
1350.500000	0.004032
1350.250000	0.004019
1350.000000	0.004005
1349.750000	0.003989
1349.500000	0.003972
1349.250000	0.003954
1349.000000	0.003936
1348.750000	0.003918
1348.500000	0.003900
1348.250000	0.003883
1348.000000	0.003866

1347.750000	0.003850
1347.500000	0.003834
1347.250000	0.003818
1347.000000	0.003801
1346.750000	0.003784
1346.500000	0.003767
1346.250000	0.003750
1346.000000	0.003732
1345.750000	0.003715
1345.500000	0.003698
1345.250000	0.003683
1345.000000	0.003668
1344.750000	0.003655
1344.500000	0.003643
1344.250000	0.003631
1344.000000	0.003620
1343.750000	0.003608
1343.500000	0.003595
1343.250000	0.003582
1343.000000	0.003569
1342.750000	0.003556
1342.500000	0.003545
1342.250000	0.003535
1342.000000	0.003525
1341.750000	0.003516
1341.500000	0.003506

1341.250000	0.003495
1341.000000	0.003482
1340.750000	0.003468
1340.500000	0.003454
1340.250000	0.003439
1340.000000	0.003426
1339.750000	0.003413
1339.500000	0.003404
1339.250000	0.003397
1339.000000	0.003395
1338.750000	0.003397
1338.500000	0.003403
1338.250000	0.003412
1338.000000	0.003424
1337.750000	0.003436
1337.500000	0.003449
1337.250000	0.003460
1337.000000	0.003470
1336.750000	0.003480
1336.500000	0.003489
1336.250000	0.003498
1336.000000	0.003509
1335.750000	0.003521
1335.500000	0.003534
1335.250000	0.003547
1335.000000	0.003561

1334.750000	0.003575
1334.500000	0.003588
1334.250000	0.003602
1334.000000	0.003616
1333.750000	0.003631
1333.500000	0.003648
1333.250000	0.003666
1333.000000	0.003685
1332.750000	0.003703
1332.500000	0.003722
1332.250000	0.003739
1332.000000	0.003757
1331.750000	0.003774
1331.500000	0.003792
1331.250000	0.003810
1331.000000	0.003829
1330.750000	0.003849
1330.500000	0.003868
1330.250000	0.003887
1330.000000	0.003906
1329.750000	0.003924
1329.500000	0.003941
1329.250000	0.003959
1329.000000	0.003976
1328.750000	0.003994
1328.500000	0.004012

1328.250000	0.004030
1328.000000	0.004048
1327.750000	0.004066
1327.500000	0.004085
1327.250000	0.004104
1327.000000	0.004124
1326.750000	0.004145
1326.500000	0.004166
1326.250000	0.004186
1326.000000	0.004205
1325.750000	0.004222
1325.500000	0.004236
1325.250000	0.004248
1325.000000	0.004260
1324.750000	0.004273
1324.500000	0.004288
1324.250000	0.004305
1324.000000	0.004325
1323.750000	0.004346
1323.500000	0.004367
1323.250000	0.004387
1323.000000	0.004405
1322.750000	0.004420
1322.500000	0.004432
1322.250000	0.004441
1322.000000	0.004448

1321.750000	0.004453
1321.500000	0.004457
1321.250000	0.004460
1321.000000	0.004463
1320.750000	0.004466
1320.500000	0.004469
1320.250000	0.004471
1320.000000	0.004472
1319.750000	0.004473
1319.500000	0.004474
1319.250000	0.004474
1319.000000	0.004474
1318.750000	0.004474
1318.500000	0.004474
1318.250000	0.004473
1318.000000	0.004470
1317.750000	0.004466
1317.500000	0.004460
1317.250000	0.004453
1317.000000	0.004446
1316.750000	0.004438
1316.500000	0.004432
1316.250000	0.004426
1316.000000	0.004422
1315.750000	0.004418
1315.500000	0.004415

1315.250000	0.004412
1315.000000	0.004408
1314.750000	0.004404
1314.500000	0.004399
1314.250000	0.004395
1314.000000	0.004393
1313.750000	0.004391
1313.500000	0.004390
1313.250000	0.004391
1313.000000	0.004391
1312.750000	0.004391
1312.500000	0.004390
1312.250000	0.004387
1312.000000	0.004382
1311.750000	0.004377
1311.500000	0.004371
1311.250000	0.004365
1311.000000	0.004360
1310.750000	0.004356
1310.500000	0.004353
1310.250000	0.004350
1310.000000	0.004348
1309.750000	0.004345
1309.500000	0.004343
1309.250000	0.004343
1309.000000	0.004343

1308.750000	0.004346
1308.500000	0.004352
1308.250000	0.004359
1308.000000	0.004368
1307.750000	0.004380
1307.500000	0.004393
1307.250000	0.004408
1307.000000	0.004426
1306.750000	0.004445
1306.500000	0.004465
1306.250000	0.004486
1306.000000	0.004507
1305.750000	0.004529
1305.500000	0.004550
1305.250000	0.004573
1305.000000	0.004596
1304.750000	0.004622
1304.500000	0.004649
1304.250000	0.004678
1304.000000	0.004708
1303.750000	0.004739
1303.500000	0.004771
1303.250000	0.004803
1303.000000	0.004837
1302.750000	0.004871
1302.500000	0.004906

1302.250000	0.004940
1302.000000	0.004974
1301.750000	0.005008
1301.500000	0.005041
1301.250000	0.005075
1301.000000	0.005108
1300.750000	0.005141
1300.500000	0.005175
1300.250000	0.005208
1300.000000	0.005241
1299.750000	0.005275
1299.500000	0.005309
1299.250000	0.005344
1299.000000	0.005380
1298.750000	0.005415
1298.500000	0.005450
1298.250000	0.005482
1298.000000	0.005512
1297.750000	0.005538
1297.500000	0.005561
1297.250000	0.005582
1297.000000	0.005600
1296.750000	0.005617
1296.500000	0.005632
1296.250000	0.005645
1296.000000	0.005655

1295.750000	0.005661
1295.500000	0.005664
1295.250000	0.005662
1295.000000	0.005658
1294.750000	0.005650
1294.500000	0.005638
1294.250000	0.005624
1294.000000	0.005607
1293.750000	0.005586
1293.500000	0.005561
1293.250000	0.005534
1293.000000	0.005503
1292.750000	0.005470
1292.500000	0.005435
1292.250000	0.005398
1292.000000	0.005360
1291.750000	0.005320
1291.500000	0.005281
1291.250000	0.005242
1291.000000	0.005204
1290.750000	0.005169
1290.500000	0.005137
1290.250000	0.005107
1290.000000	0.005079
1289.750000	0.005051
1289.500000	0.005022

1289.250000	0.004992
1289.000000	0.004960
1288.750000	0.004926
1288.500000	0.004891
1288.250000	0.004856
1288.000000	0.004821
1287.750000	0.004788
1287.500000	0.004757
1287.250000	0.004729
1287.000000	0.004704
1286.750000	0.004682
1286.500000	0.004661
1286.250000	0.004641
1286.000000	0.004624
1285.750000	0.004607
1285.500000	0.004592
1285.250000	0.004580
1285.000000	0.004570
1284.750000	0.004563
1284.500000	0.004558
1284.250000	0.004555
1284.000000	0.004553
1283.750000	0.004553
1283.500000	0.004554
1283.250000	0.004556
1283.000000	0.004561

1282.750000	0.004569
1282.500000	0.004581
1282.250000	0.004595
1282.000000	0.004611
1281.750000	0.004628
1281.500000	0.004646
1281.250000	0.004665
1281.000000	0.004684
1280.750000	0.004704
1280.500000	0.004725
1280.250000	0.004749
1280.000000	0.004774
1279.750000	0.004802
1279.500000	0.004832
1279.250000	0.004863
1279.000000	0.004895
1278.750000	0.004927
1278.500000	0.004959
1278.250000	0.004989
1278.000000	0.005019
1277.750000	0.005048
1277.500000	0.005077
1277.250000	0.005107
1277.000000	0.005138
1276.750000	0.005171
1276.500000	0.005208

1276.250000	0.005247
1276.000000	0.005289
1275.750000	0.005333
1275.500000	0.005378
1275.250000	0.005425
1275.000000	0.005471
1274.750000	0.005517
1274.500000	0.005563
1274.250000	0.005607
1274.000000	0.005651
1273.750000	0.005695
1273.500000	0.005738
1273.250000	0.005781
1273.000000	0.005823
1272.750000	0.005864
1272.500000	0.005903
1272.250000	0.005941
1272.000000	0.005978
1271.750000	0.006013
1271.500000	0.006048
1271.250000	0.006083
1271.000000	0.006119
1270.750000	0.006155
1270.500000	0.006193
1270.250000	0.006230
1270.000000	0.006268

1269.750000	0.006305
1269.500000	0.006341
1269.250000	0.006377
1269.000000	0.006411
1268.750000	0.006445
1268.500000	0.006478
1268.250000	0.006510
1268.000000	0.006542
1267.750000	0.006574
1267.500000	0.006605
1267.250000	0.006638
1267.000000	0.006671
1266.750000	0.006706
1266.500000	0.006743
1266.250000	0.006781
1266.000000	0.006822
1265.750000	0.006863
1265.500000	0.006904
1265.250000	0.006945
1265.000000	0.006985
1264.750000	0.007024
1264.500000	0.007064
1264.250000	0.007103
1264.000000	0.007142
1263.750000	0.007183
1263.500000	0.007225

1263.250000	0.007269
1263.000000	0.007315
1262.750000	0.007363
1262.500000	0.007412
1262.250000	0.007461
1262.000000	0.007511
1261.750000	0.007560
1261.500000	0.007609
1261.250000	0.007656
1261.000000	0.007703
1260.750000	0.007750
1260.500000	0.007796
1260.250000	0.007843
1260.000000	0.007891
1259.750000	0.007941
1259.500000	0.007991
1259.250000	0.008042
1259.000000	0.008093
1258.750000	0.008144
1258.500000	0.008195
1258.250000	0.008245
1258.000000	0.008296
1257.750000	0.008346
1257.500000	0.008397
1257.250000	0.008448
1257.000000	0.008500

1256.750000	0.008553
1256.500000	0.008606
1256.250000	0.008659
1256.000000	0.008712
1255.750000	0.008765
1255.500000	0.008818
1255.250000	0.008869
1255.000000	0.008920
1254.750000	0.008970
1254.500000	0.009018
1254.250000	0.009066
1254.000000	0.009113
1253.750000	0.009158
1253.500000	0.009203
1253.250000	0.009246
1253.000000	0.009289
1252.750000	0.009331
1252.500000	0.009372
1252.250000	0.009413
1252.000000	0.009452
1251.750000	0.009490
1251.500000	0.009526
1251.250000	0.009559
1251.000000	0.009589
1250.750000	0.009617
1250.500000	0.009641

1250.250000	0.009665
1250.000000	0.009688
1249.750000	0.009711
1249.500000	0.009734
1249.250000	0.009757
1249.000000	0.009780
1248.750000	0.009801
1248.500000	0.009819
1248.250000	0.009836
1248.000000	0.009850
1247.750000	0.009863
1247.500000	0.009874
1247.250000	0.009885
1247.000000	0.009894
1246.750000	0.009903
1246.500000	0.009911
1246.250000	0.009918
1246.000000	0.009924
1245.750000	0.009930
1245.500000	0.009934
1245.250000	0.009937
1245.000000	0.009938
1244.750000	0.009936
1244.500000	0.009933
1244.250000	0.009929
1244.000000	0.009925

1243.750000	0.009923
1243.500000	0.009921
1243.250000	0.009922
1243.000000	0.009924
1242.750000	0.009927
1242.500000	0.009930
1242.250000	0.009934
1242.000000	0.009937
1241.750000	0.009941
1241.500000	0.009944
1241.250000	0.009947
1241.000000	0.009949
1240.750000	0.009948
1240.500000	0.009946
1240.250000	0.009943
1240.000000	0.009939
1239.750000	0.009935
1239.500000	0.009932
1239.250000	0.009931
1239.000000	0.009930
1238.750000	0.009929
1238.500000	0.009928
1238.250000	0.009925
1238.000000	0.009921
1237.750000	0.009916
1237.500000	0.009911

1237.250000	0.009907
1237.000000	0.009904
1236.750000	0.009901
1236.500000	0.009899
1236.250000	0.009896
1236.000000	0.009892
1235.750000	0.009886
1235.500000	0.009880
1235.250000	0.009873
1235.000000	0.009865
1234.750000	0.009856
1234.500000	0.009846
1234.250000	0.009834
1234.000000	0.009821
1233.750000	0.009805
1233.500000	0.009788
1233.250000	0.009769
1233.000000	0.009750
1232.750000	0.009730
1232.500000	0.009710
1232.250000	0.009689
1232.000000	0.009667
1231.750000	0.009644
1231.500000	0.009619
1231.250000	0.009592
1231.000000	0.009561

1230.750000	0.009528
1230.500000	0.009493
1230.250000	0.009454
1230.000000	0.009415
1229.750000	0.009373
1229.500000	0.009331
1229.250000	0.009288
1229.000000	0.009244
1228.750000	0.009199
1228.500000	0.009154
1228.250000	0.009109
1228.000000	0.009063
1227.750000	0.009018
1227.500000	0.008974
1227.250000	0.008931
1227.000000	0.008887
1226.750000	0.008843
1226.500000	0.008797
1226.250000	0.008749
1226.000000	0.008700
1225.750000	0.008648
1225.500000	0.008597
1225.250000	0.008547
1225.000000	0.008498
1224.750000	0.008452
1224.500000	0.008407

1224.250000	0.008364
1224.000000	0.008321
1223.750000	0.008278
1223.500000	0.008234
1223.250000	0.008190
1223.000000	0.008145
1222.750000	0.008100
1222.500000	0.008057
1222.250000	0.008016
1222.000000	0.007978
1221.750000	0.007943
1221.500000	0.007914
1221.250000	0.007889
1221.000000	0.007869
1220.750000	0.007854
1220.500000	0.007844
1220.250000	0.007837
1220.000000	0.007832
1219.750000	0.007830
1219.500000	0.007829
1219.250000	0.007829
1219.000000	0.007830
1218.750000	0.007832
1218.500000	0.007834
1218.250000	0.007835
1218.000000	0.007835

1217.750000	0.007832
1217.500000	0.007825
1217.250000	0.007812
1217.000000	0.007795
1216.750000	0.007772
1216.500000	0.007744
1216.250000	0.007714
1216.000000	0.007682
1215.750000	0.007650
1215.500000	0.007619
1215.250000	0.007590
1215.000000	0.007563
1214.750000	0.007538
1214.500000	0.007515
1214.250000	0.007494
1214.000000	0.007475
1213.750000	0.007456
1213.500000	0.007440
1213.250000	0.007427
1213.000000	0.007416
1212.750000	0.007409
1212.500000	0.007405
1212.250000	0.007403
1212.000000	0.007403
1211.750000	0.007403
1211.500000	0.007402

1211.250000	0.007400
1211.000000	0.007396
1210.750000	0.007392
1210.500000	0.007388
1210.250000	0.007386
1210.000000	0.007386
1209.750000	0.007389
1209.500000	0.007394
1209.250000	0.007402
1209.000000	0.007412
1208.750000	0.007422
1208.500000	0.007435
1208.250000	0.007448
1208.000000	0.007464
1207.750000	0.007481
1207.500000	0.007500
1207.250000	0.007519
1207.000000	0.007539
1206.750000	0.007557
1206.500000	0.007574
1206.250000	0.007590
1206.000000	0.007605
1205.750000	0.007620
1205.500000	0.007635
1205.250000	0.007652
1205.000000	0.007671

1204.750000	0.007692
1204.500000	0.007714
1204.250000	0.007736
1204.000000	0.007757
1203.750000	0.007778
1203.500000	0.007799
1203.250000	0.007819
1203.000000	0.007839
1202.750000	0.007861
1202.500000	0.007884
1202.250000	0.007910
1202.000000	0.007937
1201.750000	0.007967
1201.500000	0.007997
1201.250000	0.008027
1201.000000	0.008058
1200.750000	0.008087
1200.500000	0.008114
1200.250000	0.008138
1200.000000	0.008160
1199.750000	0.008181
1199.500000	0.008199
1199.250000	0.008217
1199.000000	0.008236
1198.750000	0.008257
1198.500000	0.008281

1198.250000	0.008308
1198.000000	0.008339
1197.750000	0.008372
1197.500000	0.008407
1197.250000	0.008441
1197.000000	0.008474
1196.750000	0.008505
1196.500000	0.008535
1196.250000	0.008563
1196.000000	0.008591
1195.750000	0.008619
1195.500000	0.008647
1195.250000	0.008676
1195.000000	0.008705
1194.750000	0.008736
1194.500000	0.008767
1194.250000	0.008798
1194.000000	0.008831
1193.750000	0.008864
1193.500000	0.008898
1193.250000	0.008932
1193.000000	0.008967
1192.750000	0.009003
1192.500000	0.009040
1192.250000	0.009078
1192.000000	0.009117

1191.750000	0.009158
1191.500000	0.009199
1191.250000	0.009242
1191.000000	0.009285
1190.750000	0.009328
1190.500000	0.009371
1190.250000	0.009415
1190.000000	0.009458
1189.750000	0.009502
1189.500000	0.009545
1189.250000	0.009589
1189.000000	0.009632
1188.750000	0.009676
1188.500000	0.009721
1188.250000	0.009767
1188.000000	0.009817
1187.750000	0.009869
1187.500000	0.009924
1187.250000	0.009981
1187.000000	0.010038
1186.750000	0.010094
1186.500000	0.010148
1186.250000	0.010199
1186.000000	0.010248
1185.750000	0.010297
1185.500000	0.010346

1185.250000	0.010397
1185.000000	0.010450
1184.750000	0.010505
1184.500000	0.010562
1184.250000	0.010620
1184.000000	0.010677
1183.750000	0.010733
1183.500000	0.010787
1183.250000	0.010839
1183.000000	0.010888
1182.750000	0.010934
1182.500000	0.010978
1182.250000	0.011021
1182.000000	0.011063
1181.750000	0.011106
1181.500000	0.011149
1181.250000	0.011193
1181.000000	0.011237
1180.750000	0.011280
1180.500000	0.011322
1180.250000	0.011362
1180.000000	0.011400
1179.750000	0.011438
1179.500000	0.011475
1179.250000	0.011514
1179.000000	0.011555

1178.750000	0.011597
1178.500000	0.011642
1178.250000	0.011687
1178.000000	0.011733
1177.750000	0.011778
1177.500000	0.011821
1177.250000	0.011864
1177.000000	0.011905
1176.750000	0.011947
1176.500000	0.011989
1176.250000	0.012034
1176.000000	0.012081
1175.750000	0.012131
1175.500000	0.012183
1175.250000	0.012237
1175.000000	0.012291
1174.750000	0.012346
1174.500000	0.012401
1174.250000	0.012457
1174.000000	0.012515
1173.750000	0.012573
1173.500000	0.012631
1173.250000	0.012689
1173.000000	0.012746
1172.750000	0.012800
1172.500000	0.012853

1172.250000	0.012906
1172.000000	0.012960
1171.750000	0.013018
1171.500000	0.013079
1171.250000	0.013144
1171.000000	0.013211
1170.750000	0.013279
1170.500000	0.013346
1170.250000	0.013412
1170.000000	0.013476
1169.750000	0.013538
1169.500000	0.013600
1169.250000	0.013662
1169.000000	0.013722
1168.750000	0.013781
1168.500000	0.013838
1168.250000	0.013891
1168.000000	0.013942
1167.750000	0.013991
1167.500000	0.014039
1167.250000	0.014085
1167.000000	0.014129
1166.750000	0.014169
1166.500000	0.014205
1166.250000	0.014235
1166.000000	0.014261

1165.750000	0.014283
1165.500000	0.014304
1165.250000	0.014324
1165.000000	0.014344
1164.750000	0.014366
1164.500000	0.014388
1164.250000	0.014408
1164.000000	0.014425
1163.750000	0.014439
1163.500000	0.014448
1163.250000	0.014452
1163.000000	0.014454
1162.750000	0.014453
1162.500000	0.014450
1162.250000	0.014448
1162.000000	0.014447
1161.750000	0.014446
1161.500000	0.014448
1161.250000	0.014450
1161.000000	0.014453
1160.750000	0.014457
1160.500000	0.014462
1160.250000	0.014468
1160.000000	0.014475
1159.750000	0.014484
1159.500000	0.014493

1159.250000	0.014503
1159.000000	0.014513
1158.750000	0.014523
1158.500000	0.014532
1158.250000	0.014541
1158.000000	0.014550
1157.750000	0.014561
1157.500000	0.014573
1157.250000	0.014588
1157.000000	0.014605
1156.750000	0.014623
1156.500000	0.014643
1156.250000	0.014664
1156.000000	0.014686
1155.750000	0.014709
1155.500000	0.014733
1155.250000	0.014758
1155.000000	0.014784
1154.750000	0.014811
1154.500000	0.014839
1154.250000	0.014868
1154.000000	0.014897
1153.750000	0.014926
1153.500000	0.014953
1153.250000	0.014980
1153.000000	0.015004

1152.750000	0.015027
1152.500000	0.015048
1152.250000	0.015068
1152.000000	0.015087
1151.750000	0.015107
1151.500000	0.015128
1151.250000	0.015150
1151.000000	0.015172
1150.750000	0.015195
1150.500000	0.015217
1150.250000	0.015238
1150.000000	0.015258
1149.750000	0.015276
1149.500000	0.015292
1149.250000	0.015305
1149.000000	0.015316
1148.750000	0.015324
1148.500000	0.015331
1148.250000	0.015337
1148.000000	0.015345
1147.750000	0.015355
1147.500000	0.015367
1147.250000	0.015382
1147.000000	0.015397
1146.750000	0.015413
1146.500000	0.015428

1146.250000	0.015441
1146.000000	0.015454
1145.750000	0.015465
1145.500000	0.015474
1145.250000	0.015481
1145.000000	0.015485
1144.750000	0.015487
1144.500000	0.015486
1144.250000	0.015483
1144.000000	0.015480
1143.750000	0.015479
1143.500000	0.015480
1143.250000	0.015483
1143.000000	0.015489
1142.750000	0.015495
1142.500000	0.015501
1142.250000	0.015507
1142.000000	0.015513
1141.750000	0.015519
1141.500000	0.015527
1141.250000	0.015536
1141.000000	0.015545
1140.750000	0.015555
1140.500000	0.015564
1140.250000	0.015571
1140.000000	0.015576

1139.750000	0.015579
1139.500000	0.015578
1139.250000	0.015576
1139.000000	0.015572
1138.750000	0.015566
1138.500000	0.015559
1138.250000	0.015553
1138.000000	0.015547
1137.750000	0.015544
1137.500000	0.015542
1137.250000	0.015543
1137.000000	0.015546
1136.750000	0.015549
1136.500000	0.015553
1136.250000	0.015558
1136.000000	0.015562
1135.750000	0.015566
1135.500000	0.015569
1135.250000	0.015572
1135.000000	0.015574
1134.750000	0.015576
1134.500000	0.015579
1134.250000	0.015584
1134.000000	0.015591
1133.750000	0.015602
1133.500000	0.015615

1133.250000	0.015630
1133.000000	0.015645
1132.750000	0.015658
1132.500000	0.015666
1132.250000	0.015671
1132.000000	0.015673
1131.750000	0.015672
1131.500000	0.015671
1131.250000	0.015671
1131.000000	0.015672
1130.750000	0.015675
1130.500000	0.015679
1130.250000	0.015685
1130.000000	0.015693
1129.750000	0.015702
1129.500000	0.015712
1129.250000	0.015724
1129.000000	0.015736
1128.750000	0.015746
1128.500000	0.015754
1128.250000	0.015760
1128.000000	0.015763
1127.750000	0.015766
1127.500000	0.015771
1127.250000	0.015778
1127.000000	0.015789

1126.750000	0.015804
1126.500000	0.015822
1126.250000	0.015841
1126.000000	0.015860
1125.750000	0.015879
1125.500000	0.015897
1125.250000	0.015914
1125.000000	0.015932
1124.750000	0.015951
1124.500000	0.015973
1124.250000	0.015997
1124.000000	0.016023
1123.750000	0.016052
1123.500000	0.016081
1123.250000	0.016108
1123.000000	0.016134
1122.750000	0.016155
1122.500000	0.016172
1122.250000	0.016186
1122.000000	0.016198
1121.750000	0.016210
1121.500000	0.016224
1121.250000	0.016241
1121.000000	0.016262
1120.750000	0.016285
1120.500000	0.016311

1120.250000	0.016339
1120.000000	0.016368
1119.750000	0.016398
1119.500000	0.016426
1119.250000	0.016454
1119.000000	0.016478
1118.750000	0.016500
1118.500000	0.016519
1118.250000	0.016535
1118.000000	0.016550
1117.750000	0.016564
1117.500000	0.016579
1117.250000	0.016596
1117.000000	0.016615
1116.750000	0.016634
1116.500000	0.016655
1116.250000	0.016675
1116.000000	0.016694
1115.750000	0.016713
1115.500000	0.016729
1115.250000	0.016744
1115.000000	0.016755
1114.750000	0.016765
1114.500000	0.016772
1114.250000	0.016779
1114.000000	0.016787

1113.750000	0.016797
1113.500000	0.016811
1113.250000	0.016827
1113.000000	0.016845
1112.750000	0.016865
1112.500000	0.016884
1112.250000	0.016902
1112.000000	0.016920
1111.750000	0.016936
1111.500000	0.016952
1111.250000	0.016967
1111.000000	0.016981
1110.750000	0.016993
1110.500000	0.017002
1110.250000	0.017010
1110.000000	0.017017
1109.750000	0.017025
1109.500000	0.017035
1109.250000	0.017047
1109.000000	0.017062
1108.750000	0.017076
1108.500000	0.017089
1108.250000	0.017099
1108.000000	0.017105
1107.750000	0.017108
1107.500000	0.017108

1107.250000	0.017107
1107.000000	0.017108
1106.750000	0.017112
1106.500000	0.017119
1106.250000	0.017129
1106.000000	0.017142
1105.750000	0.017156
1105.500000	0.017169
1105.250000	0.017180
1105.000000	0.017187
1104.750000	0.017192
1104.500000	0.017193
1104.250000	0.017191
1104.000000	0.017190
1103.750000	0.017188
1103.500000	0.017189
1103.250000	0.017193
1103.000000	0.017200
1102.750000	0.017210
1102.500000	0.017222
1102.250000	0.017235
1102.000000	0.017246
1101.750000	0.017256
1101.500000	0.017261
1101.250000	0.017263
1101.000000	0.017262

1100.750000	0.017258
1100.500000	0.017254
1100.250000	0.017251
1100.000000	0.017252
1099.750000	0.017257
1099.500000	0.017267
1099.250000	0.017280
1099.000000	0.017295
1098.750000	0.017309
1098.500000	0.017319
1098.250000	0.017326
1098.000000	0.017329
1097.750000	0.017328
1097.500000	0.017325
1097.250000	0.017320
1097.000000	0.017316
1096.750000	0.017313
1096.500000	0.017314
1096.250000	0.017317
1096.000000	0.017325
1095.750000	0.017336
1095.500000	0.017349
1095.250000	0.017361
1095.000000	0.017371
1094.750000	0.017376
1094.500000	0.017377

1094.250000	0.017375
1094.000000	0.017371
1093.750000	0.017368
1093.500000	0.017369
1093.250000	0.017373
1093.000000	0.017383
1092.750000	0.017396
1092.500000	0.017412
1092.250000	0.017429
1092.000000	0.017449
1091.750000	0.017469
1091.500000	0.017490
1091.250000	0.017510
1091.000000	0.017528
1090.750000	0.017542
1090.500000	0.017552
1090.250000	0.017558
1090.000000	0.017560
1089.750000	0.017562
1089.500000	0.017563
1089.250000	0.017567
1089.000000	0.017574
1088.750000	0.017583
1088.500000	0.017593
1088.250000	0.017605
1088.000000	0.017616

1087.750000	0.017627
1087.500000	0.017638
1087.250000	0.017650
1087.000000	0.017661
1086.750000	0.017674
1086.500000	0.017688
1086.250000	0.017703
1086.000000	0.017721
1085.750000	0.017740
1085.500000	0.017760
1085.250000	0.017780
1085.000000	0.017798
1084.750000	0.017814
1084.500000	0.017827
1084.250000	0.017836
1084.000000	0.017843
1083.750000	0.017849
1083.500000	0.017855
1083.250000	0.017864
1083.000000	0.017874
1082.750000	0.017887
1082.500000	0.017902
1082.250000	0.017918
1082.000000	0.017934
1081.750000	0.017951
1081.500000	0.017967

1081.250000	0.017981
1081.000000	0.017993
1080.750000	0.018003
1080.500000	0.018012
1080.250000	0.018021
1080.000000	0.018031
1079.750000	0.018044
1079.500000	0.018061
1079.250000	0.018080
1079.000000	0.018102
1078.750000	0.018124
1078.500000	0.018144
1078.250000	0.018162
1078.000000	0.018177
1077.750000	0.018189
1077.500000	0.018199
1077.250000	0.018207
1077.000000	0.018215
1076.750000	0.018221
1076.500000	0.018227
1076.250000	0.018234
1076.000000	0.018241
1075.750000	0.018250
1075.500000	0.018262
1075.250000	0.018274
1075.000000	0.018288

1074.750000	0.018301
1074.500000	0.018313
1074.250000	0.018323
1074.000000	0.018330
1073.750000	0.018334
1073.500000	0.018338
1073.250000	0.018340
1073.000000	0.018343
1072.750000	0.018347
1072.500000	0.018353
1072.250000	0.018361
1072.000000	0.018372
1071.750000	0.018385
1071.500000	0.018400
1071.250000	0.018414
1071.000000	0.018427
1070.750000	0.018437
1070.500000	0.018444
1070.250000	0.018447
1070.000000	0.018450
1069.750000	0.018455
1069.500000	0.018462
1069.250000	0.018474
1069.000000	0.018490
1068.750000	0.018507
1068.500000	0.018526

1068.250000	0.018542
1068.000000	0.018556
1067.750000	0.018565
1067.500000	0.018572
1067.250000	0.018576
1067.000000	0.018579
1066.750000	0.018581
1066.500000	0.018583
1066.250000	0.018587
1066.000000	0.018592
1065.750000	0.018600
1065.500000	0.018609
1065.250000	0.018621
1065.000000	0.018633
1064.750000	0.018645
1064.500000	0.018655
1064.250000	0.018663
1064.000000	0.018669
1063.750000	0.018671
1063.500000	0.018671
1063.250000	0.018670
1063.000000	0.018667
1062.750000	0.018665
1062.500000	0.018664
1062.250000	0.018666
1062.000000	0.018671

1061.750000	0.018680
1061.500000	0.018691
1061.250000	0.018704
1061.000000	0.018717
1060.750000	0.018727
1060.500000	0.018733
1060.250000	0.018734
1060.000000	0.018730
1059.750000	0.018723
1059.500000	0.018715
1059.250000	0.018708
1059.000000	0.018703
1058.750000	0.018701
1058.500000	0.018702
1058.250000	0.018706
1058.000000	0.018712
1057.750000	0.018719
1057.500000	0.018727
1057.250000	0.018735
1057.000000	0.018743
1056.750000	0.018751
1056.500000	0.018757
1056.250000	0.018762
1056.000000	0.018765
1055.750000	0.018768
1055.500000	0.018771

1055.250000	0.018772
1055.000000	0.018773
1054.750000	0.018772
1054.500000	0.018770
1054.250000	0.018767
1054.000000	0.018763
1053.750000	0.018759
1053.500000	0.018755
1053.250000	0.018752
1053.000000	0.018749
1052.750000	0.018747
1052.500000	0.018746
1052.250000	0.018745
1052.000000	0.018746
1051.750000	0.018748
1051.500000	0.018750
1051.250000	0.018753
1051.000000	0.018756
1050.750000	0.018758
1050.500000	0.018758
1050.250000	0.018758
1050.000000	0.018759
1049.750000	0.018762
1049.500000	0.018768
1049.250000	0.018778
1049.000000	0.018791

1048.750000	0.018806
1048.500000	0.018819
1048.250000	0.018830
1048.000000	0.018838
1047.750000	0.018841
1047.500000	0.018840
1047.250000	0.018836
1047.000000	0.018830
1046.750000	0.018824
1046.500000	0.018817
1046.250000	0.018812
1046.000000	0.018809
1045.750000	0.018809
1045.500000	0.018812
1045.250000	0.018816
1045.000000	0.018823
1044.750000	0.018829
1044.500000	0.018836
1044.250000	0.018841
1044.000000	0.018844
1043.750000	0.018846
1043.500000	0.018848
1043.250000	0.018849
1043.000000	0.018850
1042.750000	0.018852
1042.500000	0.018854

1042.250000	0.018858
1042.000000	0.018863
1041.750000	0.018870
1041.500000	0.018878
1041.250000	0.018886
1041.000000	0.018895
1040.750000	0.018902
1040.500000	0.018907
1040.250000	0.018909
1040.000000	0.018908
1039.750000	0.018905
1039.500000	0.018899
1039.250000	0.018893
1039.000000	0.018887
1038.750000	0.018882
1038.500000	0.018880
1038.250000	0.018881
1038.000000	0.018884
1037.750000	0.018889
1037.500000	0.018893
1037.250000	0.018897
1037.000000	0.018898
1036.750000	0.018897
1036.500000	0.018893
1036.250000	0.018888
1036.000000	0.018884

1035.750000	0.018881
1035.500000	0.018882
1035.250000	0.018887
1035.000000	0.018894
1034.750000	0.018903
1034.500000	0.018913
1034.250000	0.018922
1034.000000	0.018930
1033.750000	0.018936
1033.500000	0.018939
1033.250000	0.018938
1033.000000	0.018934
1032.750000	0.018927
1032.500000	0.018918
1032.250000	0.018908
1032.000000	0.018899
1031.750000	0.018891
1031.500000	0.018886
1031.250000	0.018882
1031.000000	0.018880
1030.750000	0.018880
1030.500000	0.018880
1030.250000	0.018880
1030.000000	0.018882
1029.750000	0.018884
1029.500000	0.018886

1029.250000	0.018888
1029.000000	0.018889
1028.750000	0.018887
1028.500000	0.018883
1028.250000	0.018876
1028.000000	0.018869
1027.750000	0.018861
1027.500000	0.018854
1027.250000	0.018848
1027.000000	0.018843
1026.750000	0.018838
1026.500000	0.018833
1026.250000	0.018828
1026.000000	0.018822
1025.750000	0.018816
1025.500000	0.018811
1025.250000	0.018805
1025.000000	0.018800
1024.750000	0.018794
1024.500000	0.018788
1024.250000	0.018781
1024.000000	0.018773
1023.750000	0.018765
1023.500000	0.018757
1023.250000	0.018748
1023.000000	0.018738

1022.750000	0.018725
1022.500000	0.018712
1022.250000	0.018700
1022.000000	0.018690
1021.750000	0.018684
1021.500000	0.018683
1021.250000	0.018685
1021.000000	0.018690
1020.750000	0.018693
1020.500000	0.018692
1020.250000	0.018686
1020.000000	0.018674
1019.750000	0.018659
1019.500000	0.018642
1019.250000	0.018626
1019.000000	0.018612
1018.750000	0.018603
1018.500000	0.018597
1018.250000	0.018595
1018.000000	0.018595
1017.750000	0.018598
1017.500000	0.018602
1017.250000	0.018606
1017.000000	0.018610
1016.750000	0.018612
1016.500000	0.018612

1016.250000	0.018610
1016.000000	0.018605
1015.750000	0.018601
1015.500000	0.018596
1015.250000	0.018592
1015.000000	0.018589
1014.750000	0.018586
1014.500000	0.018584
1014.250000	0.018581
1014.000000	0.018577
1013.750000	0.018573
1013.500000	0.018569
1013.250000	0.018565
1013.000000	0.018562
1012.750000	0.018560
1012.500000	0.018559
1012.250000	0.018559
1012.000000	0.018560
1011.750000	0.018562
1011.500000	0.018565
1011.250000	0.018569
1011.000000	0.018571
1010.750000	0.018571
1010.500000	0.018569
1010.250000	0.018562
1010.000000	0.018551

1009.750000	0.018537
1009.500000	0.018520
1009.250000	0.018503
1009.000000	0.018484
1008.750000	0.018465
1008.500000	0.018447
1008.250000	0.018429
1008.000000	0.018412
1007.750000	0.018397
1007.500000	0.018384
1007.250000	0.018373
1007.000000	0.018364
1006.750000	0.018355
1006.500000	0.018346
1006.250000	0.018337
1006.000000	0.018326
1005.750000	0.018313
1005.500000	0.018299
1005.250000	0.018284
1005.000000	0.018269
1004.750000	0.018254
1004.500000	0.018239
1004.250000	0.018225
1004.000000	0.018210
1003.750000	0.018195
1003.500000	0.018179

1003.250000	0.018162
1003.000000	0.018143
1002.750000	0.018124
1002.500000	0.018103
1002.250000	0.018080
1002.000000	0.018058
1001.750000	0.018035
1001.500000	0.018012
1001.250000	0.017990
1001.000000	0.017969
1000.750000	0.017948
1000.500000	0.017927
1000.250000	0.017908
1000.000000	0.017889
999.750000	0.017872
999.500000	0.017854
999.250000	0.017836
999.000000	0.017817
998.750000	0.017798
998.500000	0.017777
998.250000	0.017756
998.000000	0.017735
997.750000	0.017714
997.500000	0.017695
997.250000	0.017677
997.000000	0.017660

996.750000	0.017642
996.500000	0.017625
996.250000	0.017609
996.000000	0.017593
995.750000	0.017580
995.500000	0.017569
995.250000	0.017560
995.000000	0.017553
994.750000	0.017546
994.500000	0.017538
994.250000	0.017527
994.000000	0.017514
993.750000	0.017498
993.500000	0.017479
993.250000	0.017459
993.000000	0.017437
992.750000	0.017415
992.500000	0.017394
992.250000	0.017374
992.000000	0.017356
991.750000	0.017342
991.500000	0.017330
991.250000	0.017321
991.000000	0.017314
990.750000	0.017308
990.500000	0.017303

990.250000	0.017298
990.000000	0.017295
989.750000	0.017292
989.500000	0.017292
989.250000	0.017293
989.000000	0.017294
988.750000	0.017294
988.500000	0.017293
988.250000	0.017290
988.000000	0.017284
987.750000	0.017277
987.500000	0.017269
987.250000	0.017261
987.000000	0.017253
986.750000	0.017246
986.500000	0.017238
986.250000	0.017231
986.000000	0.017225
985.750000	0.017219
985.500000	0.017215
985.250000	0.017212
985.000000	0.017210
984.750000	0.017207
984.500000	0.017204
984.250000	0.017200
984.000000	0.017194

983.750000	0.017186
983.500000	0.017176
983.250000	0.017163
983.000000	0.017147
982.750000	0.017125
982.500000	0.017098
982.250000	0.017066
982.000000	0.017032
981.750000	0.016998
981.500000	0.016967
981.250000	0.016942
981.000000	0.016924
980.750000	0.016912
980.500000	0.016905
980.250000	0.016900
980.000000	0.016895
979.750000	0.016886
979.500000	0.016873
979.250000	0.016854
979.000000	0.016830
978.750000	0.016801
978.500000	0.016769
978.250000	0.016737
978.000000	0.016706
977.750000	0.016678
977.500000	0.016653

977.250000	0.016633
977.000000	0.016616
976.750000	0.016600
976.500000	0.016583
976.250000	0.016565
976.000000	0.016544
975.750000	0.016520
975.500000	0.016493
975.250000	0.016466
975.000000	0.016437
974.750000	0.016410
974.500000	0.016383
974.250000	0.016359
974.000000	0.016337
973.750000	0.016319
973.500000	0.016303
973.250000	0.016289
973.000000	0.016275
972.750000	0.016261
972.500000	0.016244
972.250000	0.016224
972.000000	0.016200
971.750000	0.016174
971.500000	0.016146
971.250000	0.016119
971.000000	0.016094

970.750000	0.016072
970.500000	0.016053
970.250000	0.016036
970.000000	0.016022
969.750000	0.016008
969.500000	0.015995
969.250000	0.015980
969.000000	0.015965
968.750000	0.015949
968.500000	0.015931
968.250000	0.015913
968.000000	0.015896
967.750000	0.015880
967.500000	0.015866
967.250000	0.015855
967.000000	0.015847
966.750000	0.015840
966.500000	0.015833
966.250000	0.015825
966.000000	0.015814
965.750000	0.015799
965.500000	0.015780
965.250000	0.015757
965.000000	0.015732
964.750000	0.015705
964.500000	0.015680

964.250000	0.015659
964.000000	0.015641
963.750000	0.015628
963.500000	0.015619
963.250000	0.015612
963.000000	0.015605
962.750000	0.015596
962.500000	0.015584
962.250000	0.015567
962.000000	0.015545
961.750000	0.015520
961.500000	0.015492
961.250000	0.015464
961.000000	0.015437
960.750000	0.015413
960.500000	0.015393
960.250000	0.015378
960.000000	0.015366
959.750000	0.015356
959.500000	0.015346
959.250000	0.015335
959.000000	0.015322
958.750000	0.015305
958.500000	0.015288
958.250000	0.015269
958.000000	0.015251

957.750000	0.015233
957.500000	0.015217
957.250000	0.015201
957.000000	0.015184
956.750000	0.015167
956.500000	0.015149
956.250000	0.015130
956.000000	0.015111
955.750000	0.015092
955.500000	0.015072
955.250000	0.015053
955.000000	0.015033
954.750000	0.015013
954.500000	0.014993
954.250000	0.014975
954.000000	0.014959
953.750000	0.014947
953.500000	0.014937
953.250000	0.014929
953.000000	0.014921
952.750000	0.014911
952.500000	0.014898
952.250000	0.014882
952.000000	0.014864
951.750000	0.014845
951.500000	0.014825

951.250000	0.014806
951.000000	0.014788
950.750000	0.014770
950.500000	0.014752
950.250000	0.014733
950.000000	0.014715
949.750000	0.014698
949.500000	0.014682
949.250000	0.014668
949.000000	0.014655
948.750000	0.014643
948.500000	0.014632
948.250000	0.014620
948.000000	0.014609
947.750000	0.014597
947.500000	0.014586
947.250000	0.014573
947.000000	0.014560
946.750000	0.014545
946.500000	0.014530
946.250000	0.014515
946.000000	0.014500
945.750000	0.014488
945.500000	0.014478
945.250000	0.014468
945.000000	0.014458

944.750000	0.014446
944.500000	0.014431
944.250000	0.014413
944.000000	0.014393
943.750000	0.014374
943.500000	0.014356
943.250000	0.014341
943.000000	0.014329
942.750000	0.014318
942.500000	0.014306
942.250000	0.014291
942.000000	0.014272
941.750000	0.014248
941.500000	0.014220
941.250000	0.014191
941.000000	0.014161
940.750000	0.014134
940.500000	0.014110
940.250000	0.014091
940.000000	0.014075
939.750000	0.014062
939.500000	0.014050
939.250000	0.014038
939.000000	0.014024
938.750000	0.014009
938.500000	0.013991

938.250000	0.013972
938.000000	0.013953
937.750000	0.013934
937.500000	0.013916
937.250000	0.013898
937.000000	0.013880
936.750000	0.013859
936.500000	0.013837
936.250000	0.013813
936.000000	0.013786
935.750000	0.013758
935.500000	0.013729
935.250000	0.013700
935.000000	0.013670
934.750000	0.013640
934.500000	0.013611
934.250000	0.013584
934.000000	0.013560
933.750000	0.013539
933.500000	0.013521
933.250000	0.013505
933.000000	0.013490
932.750000	0.013473
932.500000	0.013453
932.250000	0.013428
932.000000	0.013400

931.750000	0.013368
931.500000	0.013336
931.250000	0.013305
931.000000	0.013277
930.750000	0.013251
930.500000	0.013228
930.250000	0.013208
930.000000	0.013189
929.750000	0.013171
929.500000	0.013153
929.250000	0.013133
929.000000	0.013110
928.750000	0.013084
928.500000	0.013054
928.250000	0.013022
928.000000	0.012990
927.750000	0.012959
927.500000	0.012931
927.250000	0.012907
927.000000	0.012886
926.750000	0.012869
926.500000	0.012853
926.250000	0.012839
926.000000	0.012824
925.750000	0.012810
925.500000	0.012796

925.250000	0.012781
925.000000	0.012767
924.750000	0.012751
924.500000	0.012735
924.250000	0.012716
924.000000	0.012696
923.750000	0.012674
923.500000	0.012650
923.250000	0.012623
923.000000	0.012595
922.750000	0.012565
922.500000	0.012534
922.250000	0.012502
922.000000	0.012471
921.750000	0.012442
921.500000	0.012416
921.250000	0.012393
921.000000	0.012373
920.750000	0.012355
920.500000	0.012338
920.250000	0.012320
920.000000	0.012301
919.750000	0.012281
919.500000	0.012260
919.250000	0.012236
919.000000	0.012211

918.750000	0.012182
918.500000	0.012151
918.250000	0.012118
918.000000	0.012083
917.750000	0.012047
917.500000	0.012012
917.250000	0.011978
917.000000	0.011946
916.750000	0.011915
916.500000	0.011884
916.250000	0.011853
916.000000	0.011822
915.750000	0.011791
915.500000	0.011760
915.250000	0.011727
915.000000	0.011693
914.750000	0.011656
914.500000	0.011615
914.250000	0.011572
914.000000	0.011528
913.750000	0.011485
913.500000	0.011445
913.250000	0.011410
913.000000	0.011380
912.750000	0.011354
912.500000	0.011329

912.250000	0.011303
912.000000	0.011273
911.750000	0.011237
911.500000	0.011197
911.250000	0.011151
911.000000	0.011103
910.750000	0.011053
910.500000	0.011005
910.250000	0.010960
910.000000	0.010919
909.750000	0.010883
909.500000	0.010851
909.250000	0.010823
909.000000	0.010796
908.750000	0.010768
908.500000	0.010737
908.250000	0.010702
908.000000	0.010662
907.750000	0.010618
907.500000	0.010572
907.250000	0.010527
907.000000	0.010483
906.750000	0.010443
906.500000	0.010407
906.250000	0.010373
906.000000	0.010343

905.750000	0.010314
905.500000	0.010286
905.250000	0.010258
905.000000	0.010229
904.750000	0.010199
904.500000	0.010166
904.250000	0.010129
904.000000	0.010089
903.750000	0.010046
903.500000	0.010000
903.250000	0.009953
903.000000	0.009906
902.750000	0.009861
902.500000	0.009819
902.250000	0.009782
902.000000	0.009749
901.750000	0.009720
901.500000	0.009693
901.250000	0.009667
901.000000	0.009639
900.750000	0.009608
900.500000	0.009573
900.250000	0.009534
900.000000	0.009493
899.750000	0.009450
899.500000	0.009407

899.250000	0.009366
899.000000	0.009328
898.750000	0.009293
898.500000	0.009260
898.250000	0.009230
898.000000	0.009200
897.750000	0.009172
897.500000	0.009143
897.250000	0.009114
897.000000	0.009084
896.750000	0.009052
896.500000	0.009019
896.250000	0.008985
896.000000	0.008949
895.750000	0.008913
895.500000	0.008876
895.250000	0.008840
895.000000	0.008804
894.750000	0.008767
894.500000	0.008730
894.250000	0.008693
894.000000	0.008655
893.750000	0.008619
893.500000	0.008585
893.250000	0.008552
893.000000	0.008523

892.750000	0.008496
892.500000	0.008471
892.250000	0.008447
892.000000	0.008425
891.750000	0.008402
891.500000	0.008379
891.250000	0.008354
891.000000	0.008327
890.750000	0.008296
890.500000	0.008261
890.250000	0.008223
890.000000	0.008182
889.750000	0.008141
889.500000	0.008102
889.250000	0.008066
889.000000	0.008034
888.750000	0.008006
888.500000	0.007981
888.250000	0.007959
888.000000	0.007938
887.750000	0.007917
887.500000	0.007894
887.250000	0.007868
887.000000	0.007840
886.750000	0.007810
886.500000	0.007778

886.250000	0.007746
886.000000	0.007715
885.750000	0.007685
885.500000	0.007658
885.250000	0.007632
885.000000	0.007606
884.750000	0.007580
884.500000	0.007553
884.250000	0.007526
884.000000	0.007498
883.750000	0.007472
883.500000	0.007448
883.250000	0.007425
883.000000	0.007403
882.750000	0.007381
882.500000	0.007357
882.250000	0.007331
882.000000	0.007304
881.750000	0.007275
881.500000	0.007248
881.250000	0.007223
881.000000	0.007200
880.750000	0.007179
880.500000	0.007157
880.250000	0.007134
880.000000	0.007109

879.750000	0.007081
879.500000	0.007052
879.250000	0.007023
879.000000	0.006994
878.750000	0.006965
878.500000	0.006935
878.250000	0.006904
878.000000	0.006872
877.750000	0.006838
877.500000	0.006804
877.250000	0.006770
877.000000	0.006739
876.750000	0.006711
876.500000	0.006687
876.250000	0.006664
876.000000	0.006643
875.750000	0.006622
875.500000	0.006601
875.250000	0.006578
875.000000	0.006554
874.750000	0.006528
874.500000	0.006501
874.250000	0.006472
874.000000	0.006442
873.750000	0.006412
873.500000	0.006383

873.250000	0.006356
873.000000	0.006332
872.750000	0.006309
872.500000	0.006288
872.250000	0.006267
872.000000	0.006244
871.750000	0.006220
871.500000	0.006194
871.250000	0.006168
871.000000	0.006142
870.750000	0.006117
870.500000	0.006095
870.250000	0.006076
870.000000	0.006058
869.750000	0.006042
869.500000	0.006027
869.250000	0.006010
869.000000	0.005992
868.750000	0.005972
868.500000	0.005951
868.250000	0.005929
868.000000	0.005907
867.750000	0.005885
867.500000	0.005865
867.250000	0.005846
867.000000	0.005828

866.750000	0.005811
866.500000	0.005793
866.250000	0.005774
866.000000	0.005753
865.750000	0.005730
865.500000	0.005708
865.250000	0.005686
865.000000	0.005665
864.750000	0.005647
864.500000	0.005630
864.250000	0.005615
864.000000	0.005600
863.750000	0.005583
863.500000	0.005565
863.250000	0.005545
863.000000	0.005524
862.750000	0.005501
862.500000	0.005478
862.250000	0.005456
862.000000	0.005436
861.750000	0.005418
861.500000	0.005402
861.250000	0.005388
861.000000	0.005374
860.750000	0.005359
860.500000	0.005344

860.250000	0.005327
860.000000	0.005309
859.750000	0.005291
859.500000	0.005274
859.250000	0.005257
859.000000	0.005242
858.750000	0.005227
858.500000	0.005212
858.250000	0.005196
858.000000	0.005181
857.750000	0.005166
857.500000	0.005153
857.250000	0.005141
857.000000	0.005131
856.750000	0.005123
856.500000	0.005116
856.250000	0.005109
856.000000	0.005103
855.750000	0.005097
855.500000	0.005092
855.250000	0.005086
855.000000	0.005081
854.750000	0.005075
854.500000	0.005068
854.250000	0.005058
854.000000	0.005045

853.750000	0.005029
853.500000	0.005010
853.250000	0.004990
853.000000	0.004971
852.750000	0.004953
852.500000	0.004939
852.250000	0.004929
852.000000	0.004923
851.750000	0.004920
851.500000	0.004919
851.250000	0.004918
851.000000	0.004916
850.750000	0.004912
850.500000	0.004906
850.250000	0.004899
850.000000	0.004892
849.750000	0.004885
849.500000	0.004881
849.250000	0.004879
849.000000	0.004880
848.750000	0.004881
848.500000	0.004882
848.250000	0.004883
848.000000	0.004882
847.750000	0.004881
847.500000	0.004881

847.250000	0.004881
847.000000	0.004883
846.750000	0.004885
846.500000	0.004888
846.250000	0.004892
846.000000	0.004896
845.750000	0.004902
845.500000	0.004911
845.250000	0.004924
845.000000	0.004940
844.750000	0.004959
844.500000	0.004979
844.250000	0.004998
844.000000	0.005017
843.750000	0.005032
843.500000	0.005046
843.250000	0.005059
843.000000	0.005071
842.750000	0.005083
842.500000	0.005094
842.250000	0.005106
842.000000	0.005117
841.750000	0.005127
841.500000	0.005137
841.250000	0.005148
841.000000	0.005159

840.750000	0.005171
840.500000	0.005183
840.250000	0.005195
840.000000	0.005207
839.750000	0.005217
839.500000	0.005226
839.250000	0.005232
839.000000	0.005237
838.750000	0.005238
838.500000	0.005238
838.250000	0.005235
838.000000	0.005232
837.750000	0.005228
837.500000	0.005226
837.250000	0.005227
837.000000	0.005231
836.750000	0.005238
836.500000	0.005247
836.250000	0.005257
836.000000	0.005265
835.750000	0.005271
835.500000	0.005274
835.250000	0.005275
835.000000	0.005275
834.750000	0.005274
834.500000	0.005273

834.250000	0.005274
834.000000	0.005275
833.750000	0.005276
833.500000	0.005278
833.250000	0.005278
833.000000	0.005276
832.750000	0.005273
832.500000	0.005269
832.250000	0.005263
832.000000	0.005258
831.750000	0.005252
831.500000	0.005248
831.250000	0.005244
831.000000	0.005241
830.750000	0.005237
830.500000	0.005231
830.250000	0.005222
830.000000	0.005211
829.750000	0.005196
829.500000	0.005178
829.250000	0.005160
829.000000	0.005142
828.750000	0.005124
828.500000	0.005106
828.250000	0.005089
828.000000	0.005071

827.750000	0.005051
827.500000	0.005030
827.250000	0.005006
827.000000	0.004980
826.750000	0.004952
826.500000	0.004923
826.250000	0.004893
826.000000	0.004864
825.750000	0.004838
825.500000	0.004815
825.250000	0.004795
825.000000	0.004779
824.750000	0.004765
824.500000	0.004752
824.250000	0.004739
824.000000	0.004725
823.750000	0.004712
823.500000	0.004701
823.250000	0.004692
823.000000	0.004687
822.750000	0.004686
822.500000	0.004687
822.250000	0.004690
822.000000	0.004694
821.750000	0.004697
821.500000	0.004703

821.250000	0.004710
821.000000	0.004721
820.750000	0.004735
820.500000	0.004753
820.250000	0.004772
820.000000	0.004790
819.750000	0.004808
819.500000	0.004825
819.250000	0.004842
819.000000	0.004859
818.750000	0.004879
818.500000	0.004901
818.250000	0.004925
818.000000	0.004950
817.750000	0.004975
817.500000	0.004999
817.250000	0.005023
817.000000	0.005046
816.750000	0.005069
816.500000	0.005093
816.250000	0.005115
816.000000	0.005137
815.750000	0.005157
815.500000	0.005175
815.250000	0.005192
815.000000	0.005207

814.750000	0.005221
814.500000	0.005234
814.250000	0.005246
814.000000	0.005256
813.750000	0.005264
813.500000	0.005269
813.250000	0.005271
813.000000	0.005270
812.750000	0.005267
812.500000	0.005263
812.250000	0.005258
812.000000	0.005251
811.750000	0.005241
811.500000	0.005229
811.250000	0.005215
811.000000	0.005197
810.750000	0.005176
810.500000	0.005153
810.250000	0.005129
810.000000	0.005103
809.750000	0.005076
809.500000	0.005049
809.250000	0.005021
809.000000	0.004994
808.750000	0.004968
808.500000	0.004943

808.250000	0.004918
808.000000	0.004896
807.750000	0.004875
807.500000	0.004857
807.250000	0.004841
807.000000	0.004827
806.750000	0.004815
806.500000	0.004803
806.250000	0.004791
806.000000	0.004775
805.750000	0.004756
805.500000	0.004734
805.250000	0.004708
805.000000	0.004682
804.750000	0.004657
804.500000	0.004635
804.250000	0.004617
804.000000	0.004605
803.750000	0.004597
803.500000	0.004593
803.250000	0.004591
803.000000	0.004590
802.750000	0.004587
802.500000	0.004582
802.250000	0.004574
802.000000	0.004562

801.750000	0.004548
801.500000	0.004531
801.250000	0.004514
801.000000	0.004498
800.750000	0.004483
800.500000	0.004470
800.250000	0.004459
800.000000	0.004447
799.750000	0.004435
799.500000	0.004420
799.250000	0.004403
799.000000	0.004384
798.750000	0.004364
798.500000	0.004345
798.250000	0.004328
798.000000	0.004314
797.750000	0.004303
797.500000	0.004294
797.250000	0.004287
797.000000	0.004279
796.750000	0.004269
796.500000	0.004257
796.250000	0.004241
796.000000	0.004223
795.750000	0.004204
795.500000	0.004184

795.250000	0.004165
795.000000	0.004149
794.750000	0.004135
794.500000	0.004124
794.250000	0.004115
794.000000	0.004106
793.750000	0.004098
793.500000	0.004088
793.250000	0.004077
793.000000	0.004063
792.750000	0.004046
792.500000	0.004027
792.250000	0.004005
792.000000	0.003983
791.750000	0.003961
791.500000	0.003941
791.250000	0.003923
791.000000	0.003909
790.750000	0.003899
790.500000	0.003891
790.250000	0.003885
790.000000	0.003880
789.750000	0.003874
789.500000	0.003869
789.250000	0.003862
789.000000	0.003856

788.750000	0.003850
788.500000	0.003846
788.250000	0.003841
788.000000	0.003837
787.750000	0.003831
787.500000	0.003823
787.250000	0.003814
787.000000	0.003802
786.750000	0.003790
786.500000	0.003778
786.250000	0.003769
786.000000	0.003763
785.750000	0.003761
785.500000	0.003763
785.250000	0.003769
785.000000	0.003780
784.750000	0.003794
784.500000	0.003811
784.250000	0.003831
784.000000	0.003854
783.750000	0.003878
783.500000	0.003904
783.250000	0.003930
783.000000	0.003956
782.750000	0.003981
782.500000	0.004005

782.250000	0.004027
782.000000	0.004049
781.750000	0.004071
781.500000	0.004093
781.250000	0.004117
781.000000	0.004144
780.750000	0.004173
780.500000	0.004203
780.250000	0.004235
780.000000	0.004265
779.750000	0.004294
779.500000	0.004321
779.250000	0.004344
779.000000	0.004366
778.750000	0.004385
778.500000	0.004403
778.250000	0.004420
778.000000	0.004437
777.750000	0.004454
777.500000	0.004470
777.250000	0.004486
777.000000	0.004500
776.750000	0.004512
776.500000	0.004521
776.250000	0.004526
776.000000	0.004527

775.750000	0.004526
775.500000	0.004522
775.250000	0.004519
775.000000	0.004515
774.750000	0.004513
774.500000	0.004511
774.250000	0.004508
774.000000	0.004503
773.750000	0.004493
773.500000	0.004480
773.250000	0.004461
773.000000	0.004437
772.750000	0.004410
772.500000	0.004379
772.250000	0.004345
772.000000	0.004309
771.750000	0.004271
771.500000	0.004232
771.250000	0.004191
771.000000	0.004151
770.750000	0.004112
770.500000	0.004074
770.250000	0.004039
770.000000	0.004006
769.750000	0.003974
769.500000	0.003944

769.250000	0.003914
769.000000	0.003885
768.750000	0.003856
768.500000	0.003828
768.250000	0.003802
768.000000	0.003777
767.750000	0.003754
767.500000	0.003734
767.250000	0.003717
767.000000	0.003702
766.750000	0.003691
766.500000	0.003682
766.250000	0.003676
766.000000	0.003671
765.750000	0.003668
765.500000	0.003665
765.250000	0.003664
765.000000	0.003662
764.750000	0.003661
764.500000	0.003660
764.250000	0.003659
764.000000	0.003658
763.750000	0.003658
763.500000	0.003659
763.250000	0.003661
763.000000	0.003666

762.750000	0.003674
762.500000	0.003682
762.250000	0.003690
762.000000	0.003694
761.750000	0.003693
761.500000	0.003684
761.250000	0.003667
761.000000	0.003642
760.750000	0.003611
760.500000	0.003575
760.250000	0.003537
760.000000	0.003500
759.750000	0.003465
759.500000	0.003433
759.250000	0.003404
759.000000	0.003378
758.750000	0.003354
758.500000	0.003334
758.250000	0.003316
758.000000	0.003300
757.750000	0.003285
757.500000	0.003270
757.250000	0.003255
757.000000	0.003239
756.750000	0.003222
756.500000	0.003203

756.250000	0.003184
756.000000	0.003165
755.750000	0.003146
755.500000	0.003128
755.250000	0.003111
755.000000	0.003096
754.750000	0.003083
754.500000	0.003072
754.250000	0.003065
754.000000	0.003061
753.750000	0.003063
753.500000	0.003068
753.250000	0.003075
753.000000	0.003085
752.750000	0.003093
752.500000	0.003100
752.250000	0.003103
752.000000	0.003102
751.750000	0.003097
751.500000	0.003090
751.250000	0.003081
751.000000	0.003073
750.750000	0.003066
750.500000	0.003060
750.250000	0.003055
750.000000	0.003052

749.750000	0.003049
749.500000	0.003047
749.250000	0.003047
749.000000	0.003049
748.750000	0.003053
748.500000	0.003061
748.250000	0.003070
748.000000	0.003081
747.750000	0.003091
747.500000	0.003098
747.250000	0.003102
747.000000	0.003103
746.750000	0.003100
746.500000	0.003094
746.250000	0.003085
746.000000	0.003073
745.750000	0.003058
745.500000	0.003040
745.250000	0.003017
745.000000	0.002992
744.750000	0.002963
744.500000	0.002933
744.250000	0.002904
744.000000	0.002876
743.750000	0.002850
743.500000	0.002827

743.250000	0.002806
743.000000	0.002786
742.750000	0.002765
742.500000	0.002745
742.250000	0.002723
742.000000	0.002702
741.750000	0.002681
741.500000	0.002661
741.250000	0.002643
741.000000	0.002626
740.750000	0.002609
740.500000	0.002593
740.250000	0.002576
740.000000	0.002561
739.750000	0.002546
739.500000	0.002533
739.250000	0.002523
739.000000	0.002516
738.750000	0.002511
738.500000	0.002507
738.250000	0.002504
738.000000	0.002502
737.750000	0.002500
737.500000	0.002498
737.250000	0.002498
737.000000	0.002501

736.750000	0.002506
736.500000	0.002512
736.250000	0.002518
736.000000	0.002523
735.750000	0.002523
735.500000	0.002519
735.250000	0.002510
735.000000	0.002497
734.750000	0.002482
734.500000	0.002468
734.250000	0.002456
734.000000	0.002447
733.750000	0.002442
733.500000	0.002440
733.250000	0.002441
733.000000	0.002443
732.750000	0.002446
732.500000	0.002449
732.250000	0.002451
732.000000	0.002453
731.750000	0.002453
731.500000	0.002452
731.250000	0.002450
731.000000	0.002448
730.750000	0.002445
730.500000	0.002441

730.250000	0.002437
730.000000	0.002434
729.750000	0.002433
729.500000	0.002432
729.250000	0.002433
729.000000	0.002435
728.750000	0.002436
728.500000	0.002436
728.250000	0.002432
728.000000	0.002423
727.750000	0.002411
727.500000	0.002396
727.250000	0.002380
727.000000	0.002365
726.750000	0.002352
726.500000	0.002342
726.250000	0.002336
726.000000	0.002332
725.750000	0.002329
725.500000	0.002327
725.250000	0.002324
725.000000	0.002321
724.750000	0.002318
724.500000	0.002314
724.250000	0.002310
724.000000	0.002306

723.750000	0.002302
723.500000	0.002296
723.250000	0.002290
723.000000	0.002282
722.750000	0.002273
722.500000	0.002263
722.250000	0.002251
722.000000	0.002237
721.750000	0.002221
721.500000	0.002205
721.250000	0.002189
721.000000	0.002174
720.750000	0.002162
720.500000	0.002154
720.250000	0.002148
720.000000	0.002144
719.750000	0.002139
719.500000	0.002132
719.250000	0.002121
719.000000	0.002106
718.750000	0.002089
718.500000	0.002070
718.250000	0.002053
718.000000	0.002039
717.750000	0.002030
717.500000	0.002024

717.250000	0.002022
717.000000	0.002022
716.750000	0.002022
716.500000	0.002022
716.250000	0.002020
716.000000	0.002017
715.750000	0.002013
715.500000	0.002010
715.250000	0.002009
715.000000	0.002010
714.750000	0.002012
714.500000	0.002016
714.250000	0.002020
714.000000	0.002023
713.750000	0.002024
713.500000	0.002022
713.250000	0.002016
713.000000	0.002009
712.750000	0.002001
712.500000	0.001993
712.250000	0.001986
712.000000	0.001980
711.750000	0.001975
711.500000	0.001970
711.250000	0.001967
711.000000	0.001965

710.750000	0.001964
710.500000	0.001963
710.250000	0.001963
710.000000	0.001964
709.750000	0.001963
709.500000	0.001962
709.250000	0.001958
709.000000	0.001952
708.750000	0.001944
708.500000	0.001936
708.250000	0.001929
708.000000	0.001924
707.750000	0.001922
707.500000	0.001924
707.250000	0.001930
707.000000	0.001939
706.750000	0.001950
706.500000	0.001960
706.250000	0.001968
706.000000	0.001972
705.750000	0.001970
705.500000	0.001964
705.250000	0.001954
705.000000	0.001943
704.750000	0.001934
704.500000	0.001928

704.250000	0.001926
704.000000	0.001929
703.750000	0.001935
703.500000	0.001943
703.250000	0.001952
703.000000	0.001960
702.750000	0.001967
702.500000	0.001974
702.250000	0.001980
702.000000	0.001985
701.750000	0.001989
701.500000	0.001991
701.250000	0.001988
701.000000	0.001982
700.750000	0.001972
700.500000	0.001960
700.250000	0.001948
700.000000	0.001939
699.750000	0.001935
699.500000	0.001936
699.250000	0.001942
699.000000	0.001951
698.750000	0.001962
698.500000	0.001971
698.250000	0.001976
698.000000	0.001979

697.750000	0.001977
697.500000	0.001974
697.250000	0.001969
697.000000	0.001964
696.750000	0.001961
696.500000	0.001958
696.250000	0.001957
696.000000	0.001957
695.750000	0.001957
695.500000	0.001956
695.250000	0.001955
695.000000	0.001952
694.750000	0.001948
694.500000	0.001942
694.250000	0.001936
694.000000	0.001930
693.750000	0.001926
693.500000	0.001924
693.250000	0.001924
693.000000	0.001926
692.750000	0.001929
692.500000	0.001931
692.250000	0.001930
692.000000	0.001926
691.750000	0.001917
691.500000	0.001904

691.250000	0.001886
691.000000	0.001865
690.750000	0.001844
690.500000	0.001822
690.250000	0.001803
690.000000	0.001786
689.750000	0.001773
689.500000	0.001765
689.250000	0.001761
689.000000	0.001761
688.750000	0.001764
688.500000	0.001768
688.250000	0.001770
688.000000	0.001769
687.750000	0.001766
687.500000	0.001761
687.250000	0.001755
687.000000	0.001750
686.750000	0.001748
686.500000	0.001748
686.250000	0.001751
686.000000	0.001755
685.750000	0.001759
685.500000	0.001761
685.250000	0.001761
685.000000	0.001759

684.750000	0.001756
684.500000	0.001754
684.250000	0.001753
684.000000	0.001753
683.750000	0.001754
683.500000	0.001756
683.250000	0.001757
683.000000	0.001757
682.750000	0.001756
682.500000	0.001754
682.250000	0.001751
682.000000	0.001748
681.750000	0.001744
681.500000	0.001740
681.250000	0.001734
681.000000	0.001727
680.750000	0.001717
680.500000	0.001706
680.250000	0.001694
680.000000	0.001683
679.750000	0.001673
679.500000	0.001666
679.250000	0.001664
679.000000	0.001665
678.750000	0.001670
678.500000	0.001676

678.250000	0.001681
678.000000	0.001685
677.750000	0.001687
677.500000	0.001687
677.250000	0.001686
677.000000	0.001686
676.750000	0.001688
676.500000	0.001691
676.250000	0.001697
676.000000	0.001704
675.750000	0.001710
675.500000	0.001715
675.250000	0.001718
675.000000	0.001719
674.750000	0.001717
674.500000	0.001712
674.250000	0.001704
674.000000	0.001694
673.750000	0.001681
673.500000	0.001668
673.250000	0.001656
673.000000	0.001647
672.750000	0.001644
672.500000	0.001645
672.250000	0.001650
672.000000	0.001656

671.750000	0.001660
671.500000	0.001661
671.250000	0.001656
671.000000	0.001645
670.750000	0.001630
670.500000	0.001612
670.250000	0.001594
670.000000	0.001573
669.750000	0.001551
669.500000	0.001530
669.250000	0.001509
669.000000	0.001485
668.750000	0.001463
668.500000	0.001460
668.250000	0.001497
668.000000	0.001573
667.750000	0.001661
667.500000	0.001725
667.250000	0.001744
667.000000	0.001726
666.750000	0.001695
666.500000	0.001665
666.250000	0.001641
666.000000	0.001620
665.750000	0.001602
665.500000	0.001587

665.250000	0.001575
665.000000	0.001565
664.750000	0.001557
664.500000	0.001551
664.250000	0.001547
664.000000	0.001546
663.750000	0.001547
663.500000	0.001550
663.250000	0.001554
663.000000	0.001558
662.750000	0.001562
662.500000	0.001564
662.250000	0.001565
662.000000	0.001564
661.750000	0.001560
661.500000	0.001554
661.250000	0.001545
661.000000	0.001535
660.750000	0.001525
660.500000	0.001514
660.250000	0.001505
660.000000	0.001498
659.750000	0.001493
659.500000	0.001490
659.250000	0.001489
659.000000	0.001489

658.750000	0.001487
658.500000	0.001482
658.250000	0.001475
658.000000	0.001465
657.750000	0.001451
657.500000	0.001437
657.250000	0.001424
657.000000	0.001414
656.750000	0.001408
656.500000	0.001407
656.250000	0.001410
656.000000	0.001416
655.750000	0.001423
655.500000	0.001428
655.250000	0.001429
655.000000	0.001425
654.750000	0.001417
654.500000	0.001406
654.250000	0.001394
654.000000	0.001382
653.750000	0.001372
653.500000	0.001365
653.250000	0.001362
653.000000	0.001361
652.750000	0.001363
652.500000	0.001365

652.250000	0.001367
652.000000	0.001366
651.750000	0.001362
651.500000	0.001353
651.250000	0.001341
651.000000	0.001326
650.750000	0.001311
650.500000	0.001298
650.250000	0.001289
650.000000	0.001283
649.750000	0.001282
649.500000	0.001284
649.250000	0.001287
649.000000	0.001289
648.750000	0.001290
648.500000	0.001287
648.250000	0.001282
648.000000	0.001274
647.750000	0.001262
647.500000	0.001248
647.250000	0.001233
647.000000	0.001216
646.750000	0.001199
646.500000	0.001184
646.250000	0.001172
646.000000	0.001161

645.750000	0.001153
645.500000	0.001146
645.250000	0.001139
645.000000	0.001131
644.750000	0.001122
644.500000	0.001113
644.250000	0.001104
644.000000	0.001096
643.750000	0.001090
643.500000	0.001086
643.250000	0.001083
643.000000	0.001082
642.750000	0.001080
642.500000	0.001078
642.250000	0.001075
642.000000	0.001068
641.750000	0.001059
641.500000	0.001047
641.250000	0.001034
641.000000	0.001019
640.750000	0.001006
640.500000	0.000995
640.250000	0.000990
640.000000	0.000990
639.750000	0.000996
639.500000	0.001008

639.250000	0.001022
639.000000	0.001037
638.750000	0.001052
638.500000	0.001063
638.250000	0.001070
638.000000	0.001072
637.750000	0.001070
637.500000	0.001062
637.250000	0.001052
637.000000	0.001038
636.750000	0.001024
636.500000	0.001010
636.250000	0.000997
636.000000	0.000987
635.750000	0.000980
635.500000	0.000975
635.250000	0.000973
635.000000	0.000971
634.750000	0.000970
634.500000	0.000969
634.250000	0.000968
634.000000	0.000966
633.750000	0.000965
633.500000	0.000964
633.250000	0.000964
633.000000	0.000964

632.750000	0.000965
632.500000	0.000965
632.250000	0.000965
632.000000	0.000965
631.750000	0.000963
631.500000	0.000960
631.250000	0.000955
631.000000	0.000948
630.750000	0.000939
630.500000	0.000929
630.250000	0.000916
630.000000	0.000904
629.750000	0.000891
629.500000	0.000881
629.250000	0.000873
629.000000	0.000868
628.750000	0.000866
628.500000	0.000865
628.250000	0.000866
628.000000	0.000866
627.750000	0.000865
627.500000	0.000862
627.250000	0.000859
627.000000	0.000856
626.750000	0.000855
626.500000	0.000856

626.250000	0.000860
626.000000	0.000866
625.750000	0.000871
625.500000	0.000874
625.250000	0.000874
625.000000	0.000871
624.750000	0.000866
624.500000	0.000859
624.250000	0.000853
624.000000	0.000849
623.750000	0.000847
623.500000	0.000848
623.250000	0.000849
623.000000	0.000852
622.750000	0.000857
622.500000	0.000864
622.250000	0.000873
622.000000	0.000884
621.750000	0.000895
621.500000	0.000905
621.250000	0.000912
621.000000	0.000915
620.750000	0.000913
620.500000	0.000909
620.250000	0.000905
620.000000	0.000902

619.750000	0.000904
619.500000	0.000910
619.250000	0.000920
619.000000	0.000930
618.750000	0.000940
618.500000	0.000945
618.250000	0.000945
618.000000	0.000938
617.750000	0.000928
617.500000	0.000915
617.250000	0.000901
617.000000	0.000890
616.750000	0.000883
616.500000	0.000879
616.250000	0.000879
616.000000	0.000884
615.750000	0.000891
615.500000	0.000900
615.250000	0.000911
615.000000	0.000922
614.750000	0.000932
614.500000	0.000940
614.250000	0.000945
614.000000	0.000948
613.750000	0.000947
613.500000	0.000945

613.250000	0.000941
613.000000	0.000936
612.750000	0.000931
612.500000	0.000924
612.250000	0.000916
612.000000	0.000907
611.750000	0.000897
611.500000	0.000887
611.250000	0.000877
611.000000	0.000869
610.750000	0.000863
610.500000	0.000860
610.250000	0.000860
610.000000	0.000861
609.750000	0.000865
609.500000	0.000871
609.250000	0.000878
609.000000	0.000885
608.750000	0.000893
608.500000	0.000901
608.250000	0.000908
608.000000	0.000913
607.750000	0.000916
607.500000	0.000918
607.250000	0.000918
607.000000	0.000919

606.750000	0.000919
606.500000	0.000920
606.250000	0.000922
606.000000	0.000923
605.750000	0.000923
605.500000	0.000921
605.250000	0.000917
605.000000	0.000910
604.750000	0.000902
604.500000	0.000893
604.250000	0.000886
604.000000	0.000880
603.750000	0.000878
603.500000	0.000878
603.250000	0.000882
603.000000	0.000889
602.750000	0.000899
602.500000	0.000911
602.250000	0.000923
602.000000	0.000934
601.750000	0.000942
601.500000	0.000947
601.250000	0.000949
601.000000	0.000951
600.750000	0.000953
600.500000	0.000957

600.250000	0.000963
600.000000	0.000971
599.750000	0.000977
599.500000	0.000979
599.250000	0.000977
599.000000	0.000970
598.750000	0.000960
598.500000	0.000950
598.250000	0.000943
598.000000	0.000942
597.750000	0.000945
597.500000	0.000952
597.250000	0.000961
597.000000	0.000970
596.750000	0.000976
596.500000	0.000981
596.250000	0.000985
596.000000	0.000990
595.750000	0.000996
595.500000	0.001005
595.250000	0.001015
595.000000	0.001024
594.750000	0.001030
594.500000	0.001032
594.250000	0.001030
594.000000	0.001025

593.750000	0.001018
593.500000	0.001010
593.250000	0.001004
593.000000	0.001002
592.750000	0.001003
592.500000	0.001009
592.250000	0.001019
592.000000	0.001031
591.750000	0.001042
591.500000	0.001048
591.250000	0.001046
591.000000	0.001037
590.750000	0.001020
590.500000	0.001000
590.250000	0.000980
590.000000	0.000963
589.750000	0.000953
589.500000	0.000949
589.250000	0.000952
589.000000	0.000959
588.750000	0.000967
588.500000	0.000975
588.250000	0.000983
588.000000	0.000989
587.750000	0.000995
587.500000	0.001001

587.250000	0.001007
587.000000	0.001011
586.750000	0.001013
586.500000	0.001011
586.250000	0.001004
586.000000	0.000994
585.750000	0.000981
585.500000	0.000969
585.250000	0.000960
585.000000	0.000954
584.750000	0.000953
584.500000	0.000958
584.250000	0.000968
584.000000	0.000981
583.750000	0.000997
583.500000	0.001014
583.250000	0.001030
583.000000	0.001044
582.750000	0.001054
582.500000	0.001057
582.250000	0.001054
582.000000	0.001044
581.750000	0.001030
581.500000	0.001012
581.250000	0.000995
581.000000	0.000980

580.750000	0.000969
580.500000	0.000964
580.250000	0.000964
580.000000	0.000969
579.750000	0.000976
579.500000	0.000985
579.250000	0.000995
579.000000	0.001007
578.750000	0.001019
578.500000	0.001032
578.250000	0.001045
578.000000	0.001056
577.750000	0.001063
577.500000	0.001067
577.250000	0.001065
577.000000	0.001058
576.750000	0.001048
576.500000	0.001035
576.250000	0.001024
576.000000	0.001016
575.750000	0.001013
575.500000	0.001014
575.250000	0.001021
575.000000	0.001030
574.750000	0.001040
574.500000	0.001049

574.250000	0.001055
574.000000	0.001058
573.750000	0.001058
573.500000	0.001054
573.250000	0.001047
573.000000	0.001038
572.750000	0.001030
572.500000	0.001023
572.250000	0.001019
572.000000	0.001016
571.750000	0.001015
571.500000	0.001012
571.250000	0.001007
571.000000	0.000998
570.750000	0.000984
570.500000	0.000966
570.250000	0.000947
570.000000	0.000930
569.750000	0.000917
569.500000	0.000912
569.250000	0.000914
569.000000	0.000924
568.750000	0.000939
568.500000	0.000957
568.250000	0.000974
568.000000	0.000987

567.750000	0.000996
567.500000	0.000998
567.250000	0.000995
567.000000	0.000986
566.750000	0.000974
566.500000	0.000961
566.250000	0.000949
566.000000	0.000940
565.750000	0.000935
565.500000	0.000933
565.250000	0.000936
565.000000	0.000941
564.750000	0.000948
564.500000	0.000956
564.250000	0.000965
564.000000	0.000973
563.750000	0.000979
563.500000	0.000984
563.250000	0.000985
563.000000	0.000983
562.750000	0.000977
562.500000	0.000968
562.250000	0.000957
562.000000	0.000945
561.750000	0.000934
561.500000	0.000926

561.250000	0.000922
561.000000	0.000923
560.750000	0.000929
560.500000	0.000939
560.250000	0.000953
560.000000	0.000970
559.750000	0.000987
559.500000	0.001002
559.250000	0.001013
559.000000	0.001018
558.750000	0.001015
558.500000	0.001005
558.250000	0.000991
558.000000	0.000974
557.750000	0.000959
557.500000	0.000949
557.250000	0.000945
557.000000	0.000947
556.750000	0.000956
556.500000	0.000966
556.250000	0.000976
556.000000	0.000983
555.750000	0.000984
555.500000	0.000980
555.250000	0.000972
555.000000	0.000963

554.750000	0.000957
554.500000	0.000954
554.250000	0.000956
554.000000	0.000962
553.750000	0.000969
553.500000	0.000975
553.250000	0.000976
553.000000	0.000971
552.750000	0.000958
552.500000	0.000940
552.250000	0.000918
552.000000	0.000896
551.750000	0.000876
551.500000	0.000859
551.250000	0.000847
551.000000	0.000838
550.750000	0.000830
550.500000	0.000824
550.250000	0.000818
550.000000	0.000814
549.750000	0.000811
549.500000	0.000813
549.250000	0.000818
549.000000	0.000828
548.750000	0.000839
548.500000	0.000850

548.250000	0.000858
548.000000	0.000860
547.750000	0.000854
547.500000	0.000839
547.250000	0.000815
547.000000	0.000785
546.750000	0.000751
546.500000	0.000719
546.250000	0.000692
546.000000	0.000673
545.750000	0.000665
545.500000	0.000668
545.250000	0.000678
545.000000	0.000693
544.750000	0.000708
544.500000	0.000720
544.250000	0.000727
544.000000	0.000728
543.750000	0.000724
543.500000	0.000718
543.250000	0.000712
543.000000	0.000708
542.750000	0.000706
542.500000	0.000707
542.250000	0.000709
542.000000	0.000711

541.750000	0.000711
541.500000	0.000709
541.250000	0.000703
541.000000	0.000694
540.750000	0.000682
540.500000	0.000669
540.250000	0.000654
540.000000	0.000640
539.750000	0.000626
539.500000	0.000614
539.250000	0.000603
539.000000	0.000593
538.750000	0.000584
538.500000	0.000574
538.250000	0.000562
538.000000	0.000550
537.750000	0.000536
537.500000	0.000524
537.250000	0.000514
537.000000	0.000509
536.750000	0.000510
536.500000	0.000516
536.250000	0.000526
536.000000	0.000538
535.750000	0.000551
535.500000	0.000562

535.250000	0.000572
535.000000	0.000580
534.750000	0.000586
534.500000	0.000591
534.250000	0.000594
534.000000	0.000595
533.750000	0.000593
533.500000	0.000589
533.250000	0.000582
533.000000	0.000574
532.750000	0.000565
532.500000	0.000554
532.250000	0.000542
532.000000	0.000528
531.750000	0.000512
531.500000	0.000493
531.250000	0.000476
531.000000	0.000463
530.750000	0.000458
530.500000	0.000465
530.250000	0.000485
530.000000	0.000517
529.750000	0.000555
529.500000	0.000593
529.250000	0.000624
529.000000	0.000644

528.750000	0.000650
528.500000	0.000643
528.250000	0.000625
528.000000	0.000600
527.750000	0.000573
527.500000	0.000546
527.250000	0.000521
527.000000	0.000499
526.750000	0.000482
526.500000	0.000472
526.250000	0.000469
526.000000	0.000474
525.750000	0.000485
525.500000	0.000500
525.250000	0.000516
525.000000	0.000529
524.750000	0.000539
524.500000	0.000546
524.250000	0.000551
524.000000	0.000555
523.750000	0.000558
523.500000	0.000561
523.250000	0.000564
523.000000	0.000565
522.750000	0.000564
522.500000	0.000561

522.250000	0.000555
522.000000	0.000550
521.750000	0.000547
521.500000	0.000548
521.250000	0.000554
521.000000	0.000565
520.750000	0.000577
520.500000	0.000588
520.250000	0.000594
520.000000	0.000591
519.750000	0.000578
519.500000	0.000556
519.250000	0.000528
519.000000	0.000500
518.750000	0.000476
518.500000	0.000461
518.250000	0.000455
518.000000	0.000459
517.750000	0.000472
517.500000	0.000491
517.250000	0.000513
517.000000	0.000533
516.750000	0.000549
516.500000	0.000558
516.250000	0.000560
516.000000	0.000557

515.750000	0.000553
515.500000	0.000548
515.250000	0.000545
515.000000	0.000545
514.750000	0.000548
514.500000	0.000552
514.250000	0.000556
514.000000	0.000557
513.750000	0.000555
513.500000	0.000549
513.250000	0.000540
513.000000	0.000530
512.750000	0.000521
512.500000	0.000517
512.250000	0.000516
512.000000	0.000520
511.750000	0.000526
511.500000	0.000532
511.250000	0.000537
511.000000	0.000541
510.750000	0.000545
510.500000	0.000550
510.250000	0.000558
510.000000	0.000569
509.750000	0.000583
509.500000	0.000598

509.250000	0.000611
509.000000	0.000621
508.750000	0.000626
508.500000	0.000627
508.250000	0.000626
508.000000	0.000623
507.750000	0.000622
507.500000	0.000623
507.250000	0.000626
507.000000	0.000632
506.750000	0.000638
506.500000	0.000642
506.250000	0.000645
506.000000	0.000646
505.750000	0.000646
505.500000	0.000647
505.250000	0.000649
505.000000	0.000653
504.750000	0.000661
504.500000	0.000670
504.250000	0.000680
504.000000	0.000688
503.750000	0.000695
503.500000	0.000701
503.250000	0.000708
503.000000	0.000718

502.750000	0.000734
502.500000	0.000758
502.250000	0.000787
502.000000	0.000817
501.750000	0.000840
501.500000	0.000853
501.250000	0.000855
501.000000	0.000848
500.750000	0.000837
500.500000	0.000824
500.250000	0.000813
500.000000	0.000807
499.750000	0.000806
499.500000	0.000811
499.250000	0.000821
499.000000	0.000837
498.750000	0.000856
498.500000	0.000879
498.250000	0.000902
498.000000	0.000926
497.750000	0.000949
497.500000	0.000969
497.250000	0.000986
497.000000	0.001002
496.750000	0.001018
496.500000	0.001035

496.250000	0.001053
496.000000	0.001074
495.750000	0.001096
495.500000	0.001116
495.250000	0.001135
495.000000	0.001149
494.750000	0.001161
494.500000	0.001170
494.250000	0.001179
494.000000	0.001189
493.750000	0.001202
493.500000	0.001217
493.250000	0.001235
493.000000	0.001255
492.750000	0.001273
492.500000	0.001289
492.250000	0.001301
492.000000	0.001308
491.750000	0.001312
491.500000	0.001316
491.250000	0.001322
491.000000	0.001335
490.750000	0.001358
490.500000	0.001390
490.250000	0.001430
490.000000	0.001473

489.750000	0.001513
489.500000	0.001544
489.250000	0.001562
489.000000	0.001567
488.750000	0.001562
488.500000	0.001551
488.250000	0.001542
488.000000	0.001537
487.750000	0.001540
487.500000	0.001550
487.250000	0.001566
487.000000	0.001586
486.750000	0.001609
486.500000	0.001635
486.250000	0.001665
486.000000	0.001699
485.750000	0.001736
485.500000	0.001773
485.250000	0.001806
485.000000	0.001832
484.750000	0.001850
484.500000	0.001858
484.250000	0.001859
484.000000	0.001857
483.750000	0.001857
483.500000	0.001865

483.250000	0.001881
483.000000	0.001909
482.750000	0.001945
482.500000	0.001988
482.250000	0.002036
482.000000	0.002083
481.750000	0.002128
481.500000	0.002164
481.250000	0.002190
481.000000	0.002204
480.750000	0.002208
480.500000	0.002207
480.250000	0.002207
480.000000	0.002213
479.750000	0.002228
479.500000	0.002254
479.250000	0.002290
479.000000	0.002332
478.750000	0.002376
478.500000	0.002417
478.250000	0.002454
478.000000	0.002485
477.750000	0.002510
477.500000	0.002530
477.250000	0.002547
477.000000	0.002563

476.750000	0.002579
476.500000	0.002596
476.250000	0.002615
476.000000	0.002632
475.750000	0.002646
475.500000	0.002654
475.250000	0.002654
475.000000	0.002646
474.750000	0.002633
474.500000	0.002619
474.250000	0.002606
474.000000	0.002600
473.750000	0.002604
473.500000	0.002619
473.250000	0.002650
473.000000	0.002696
472.750000	0.002755
472.500000	0.002822
472.250000	0.002885
472.000000	0.002938
471.750000	0.002975
471.500000	0.002995
471.250000	0.002999
471.000000	0.002990
470.750000	0.002971
470.500000	0.002945

470.250000	0.002918
470.000000	0.002896
469.750000	0.002884
469.500000	0.002883
469.250000	0.002893
469.000000	0.002910
468.750000	0.002931
468.500000	0.002952
468.250000	0.002972
468.000000	0.002990
467.750000	0.003011
467.500000	0.003035
467.250000	0.003062
467.000000	0.003091
466.750000	0.003117
466.500000	0.003137
466.250000	0.003148
466.000000	0.003148
465.750000	0.003138
465.500000	0.003119
465.250000	0.003095
465.000000	0.003069
464.750000	0.003043
464.500000	0.003018
464.250000	0.002994
464.000000	0.002972

463.750000	0.002953
463.500000	0.002934
463.250000	0.002917
463.000000	0.002901
462.750000	0.002887
462.500000	0.002878
462.250000	0.002877
462.000000	0.002890
461.750000	0.002921
461.500000	0.002971
461.250000	0.003035
461.000000	0.003104
460.750000	0.003170
460.500000	0.003221
460.250000	0.003253
460.000000	0.003266
459.750000	0.003261
459.500000	0.003247
459.250000	0.003229
459.000000	0.003215
458.750000	0.003209
458.500000	0.003218
458.250000	0.003243
458.000000	0.003278
457.750000	0.003313
457.500000	0.003336

457.250000	0.003343
457.000000	0.003338
456.750000	0.003328
456.500000	0.003319
456.250000	0.003311
456.000000	0.003301
455.750000	0.003285
455.500000	0.003262
455.250000	0.003232
455.000000	0.003197
454.750000	0.003163
454.500000	0.003135
454.250000	0.003117
454.000000	0.003113
453.750000	0.003121
453.500000	0.003136
453.250000	0.003153
453.000000	0.003165
452.750000	0.003166
452.500000	0.003156
452.250000	0.003137
452.000000	0.003114
451.750000	0.003092
451.500000	0.003075
451.250000	0.003065
451.000000	0.003064

450.750000	0.003072
450.500000	0.003089
450.250000	0.003114
450.000000	0.003143
449.750000	0.003170
449.500000	0.003191
449.250000	0.003198
449.000000	0.003188
448.750000	0.003160
448.500000	0.003115
448.250000	0.003058
448.000000	0.002994
447.750000	0.002932
447.500000	0.002880
447.250000	0.002842
447.000000	0.002826
446.750000	0.002830
446.500000	0.002854
446.250000	0.002892
446.000000	0.002936
445.750000	0.002980
445.500000	0.003020
445.250000	0.003050
445.000000	0.003071
444.750000	0.003081
444.500000	0.003081

444.250000	0.003074
444.000000	0.003061
443.750000	0.003046
443.500000	0.003031
443.250000	0.003015
443.000000	0.003000
442.750000	0.002984
442.500000	0.002966
442.250000	0.002948
442.000000	0.002929
441.750000	0.002913
441.500000	0.002901
441.250000	0.002892
441.000000	0.002886
440.750000	0.002879
440.500000	0.002869
440.250000	0.002852
440.000000	0.002827
439.750000	0.002792
439.500000	0.002750
439.250000	0.002705
439.000000	0.002661
438.750000	0.002626
438.500000	0.002604
438.250000	0.002596
438.000000	0.002603

437.750000	0.002621
437.500000	0.002647
437.250000	0.002677
437.000000	0.002705
436.750000	0.002729
436.500000	0.002747
436.250000	0.002759
436.000000	0.002767
435.750000	0.002770
435.500000	0.002769
435.250000	0.002765
435.000000	0.002755
434.750000	0.002740
434.500000	0.002720
434.250000	0.002695
434.000000	0.002666
433.750000	0.002633
433.500000	0.002598
433.250000	0.002561
433.000000	0.002523
432.750000	0.002485
432.500000	0.002452
432.250000	0.002426
432.000000	0.002411
431.750000	0.002409
431.500000	0.002417

431.250000	0.002432
431.000000	0.002448
430.750000	0.002462
430.500000	0.002470
430.250000	0.002473
430.000000	0.002473
429.750000	0.002472
429.500000	0.002470
429.250000	0.002466
429.000000	0.002459
428.750000	0.002445
428.500000	0.002426
428.250000	0.002401
428.000000	0.002375
427.750000	0.002350
427.500000	0.002327
427.250000	0.002307
427.000000	0.002288
426.750000	0.002267
426.500000	0.002242
426.250000	0.002215
426.000000	0.002187
425.750000	0.002162
425.500000	0.002142
425.250000	0.002127
425.000000	0.002114

424.750000	0.002100
424.500000	0.002079
424.250000	0.002052
424.000000	0.002017
423.750000	0.001979
423.500000	0.001945
423.250000	0.001922
423.000000	0.001917
422.750000	0.001931
422.500000	0.001960
422.250000	0.001996
422.000000	0.002033
421.750000	0.002066
421.500000	0.002093
421.250000	0.002114
421.000000	0.002129
420.750000	0.002136
420.500000	0.002134
420.250000	0.002122
420.000000	0.002097
419.750000	0.002061
419.500000	0.002018
419.250000	0.001978
419.000000	0.001952
418.750000	0.001945
418.500000	0.001947

418.250000	0.001946
418.000000	0.001935
417.750000	0.001911
417.500000	0.001877
417.250000	0.001834
417.000000	0.001786
416.750000	0.001735
416.500000	0.001683
416.250000	0.001634
416.000000	0.001590
415.750000	0.001554
415.500000	0.001529
415.250000	0.001516
415.000000	0.001514
414.750000	0.001522
414.500000	0.001538
414.250000	0.001555
414.000000	0.001568
413.750000	0.001569
413.500000	0.001551
413.250000	0.001510
413.000000	0.001446
412.750000	0.001366
412.500000	0.001280
412.250000	0.001204
412.000000	0.001151

411.750000	0.001128
411.500000	0.001136
411.250000	0.001166
411.000000	0.001205
410.750000	0.001241
410.500000	0.001262
410.250000	0.001267
410.000000	0.001255
409.750000	0.001234
409.500000	0.001207
409.250000	0.001174
409.000000	0.001134
408.750000	0.001084
408.500000	0.001021
408.250000	0.000948
408.000000	0.000872
407.750000	0.000804
407.500000	0.000751
407.250000	0.000720
407.000000	0.000710
406.750000	0.000715
406.500000	0.000726
406.250000	0.000736
406.000000	0.000741
405.750000	0.000741
405.500000	0.000739

405.250000	0.000739
405.000000	0.000745
404.750000	0.000758
404.500000	0.000780
404.250000	0.000808
404.000000	0.000838
403.750000	0.000863
403.500000	0.000877
403.250000	0.000873
403.000000	0.000843
402.750000	0.000786
402.500000	0.000709
402.250000	0.000621
402.000000	0.000532
401.750000	0.000455
401.500000	0.000407
401.250000	0.000399
401.000000	0.000424
400.750000	0.000463
400.500000	0.000499
400.250000	0.000523
400.000000	0.000540

A1_2mm_b

PE IR SUBTECH SPECTRUM ASCII PEDS 4.00

-1

A1_2mm_b_1_1_1.ASC

21/07/23

16:16:33.00

21/12/06

15:42:15.00

ir

Sample 019 By ir Date venerd, luglio 23 2021

400.000000

#HDR

-1

-1

#GR

cm-1

A

1.0

0.0

4000.000000

-0.250000

14401

8

0.041622

0.000345

#DATA

4000.000000 0.000657

3999.750000	0.000653
3999.500000	0.000649
3999.250000	0.000645
3999.000000	0.000640
3998.750000	0.000634
3998.500000	0.000630
3998.250000	0.000627
3998.000000	0.000628
3997.750000	0.000633
3997.500000	0.000641
3997.250000	0.000649
3997.000000	0.000658
3996.750000	0.000665
3996.500000	0.000668
3996.250000	0.000666
3996.000000	0.000662
3995.750000	0.000658
3995.500000	0.000657
3995.250000	0.000657
3995.000000	0.000659
3994.750000	0.000661
3994.500000	0.000664
3994.250000	0.000667
3994.000000	0.000670
3993.750000	0.000672
3993.500000	0.000675

3993.250000	0.000678
3993.000000	0.000681
3992.750000	0.000684
3992.500000	0.000688
3992.250000	0.000691
3992.000000	0.000695
3991.750000	0.000698
3991.500000	0.000702
3991.250000	0.000706
3991.000000	0.000709
3990.750000	0.000709
3990.500000	0.000705
3990.250000	0.000697
3990.000000	0.000685
3989.750000	0.000671
3989.500000	0.000655
3989.250000	0.000641
3989.000000	0.000631
3988.750000	0.000626
3988.500000	0.000627
3988.250000	0.000633
3988.000000	0.000642
3987.750000	0.000654
3987.500000	0.000669
3987.250000	0.000684
3987.000000	0.000699

3986.750000	0.000713
3986.500000	0.000724
3986.250000	0.000732
3986.000000	0.000734
3985.750000	0.000731
3985.500000	0.000722
3985.250000	0.000710
3985.000000	0.000696
3984.750000	0.000684
3984.500000	0.000675
3984.250000	0.000672
3984.000000	0.000676
3983.750000	0.000685
3983.500000	0.000698
3983.250000	0.000712
3983.000000	0.000726
3982.750000	0.000736
3982.500000	0.000742
3982.250000	0.000743
3982.000000	0.000739
3981.750000	0.000732
3981.500000	0.000722
3981.250000	0.000713
3981.000000	0.000706
3980.750000	0.000703
3980.500000	0.000704

3980.250000	0.000709
3980.000000	0.000715
3979.750000	0.000723
3979.500000	0.000729
3979.250000	0.000734
3979.000000	0.000736
3978.750000	0.000739
3978.500000	0.000743
3978.250000	0.000749
3978.000000	0.000759
3977.750000	0.000772
3977.500000	0.000786
3977.250000	0.000801
3977.000000	0.000812
3976.750000	0.000819
3976.500000	0.000819
3976.250000	0.000815
3976.000000	0.000805
3975.750000	0.000794
3975.500000	0.000781
3975.250000	0.000769
3975.000000	0.000760
3974.750000	0.000753
3974.500000	0.000749
3974.250000	0.000749
3974.000000	0.000753

3973.750000	0.000762
3973.500000	0.000774
3973.250000	0.000789
3973.000000	0.000805
3972.750000	0.000820
3972.500000	0.000832
3972.250000	0.000840
3972.000000	0.000844
3971.750000	0.000843
3971.500000	0.000840
3971.250000	0.000835
3971.000000	0.000830
3970.750000	0.000825
3970.500000	0.000819
3970.250000	0.000812
3970.000000	0.000804
3969.750000	0.000795
3969.500000	0.000786
3969.250000	0.000777
3969.000000	0.000770
3968.750000	0.000766
3968.500000	0.000765
3968.250000	0.000768
3968.000000	0.000773
3967.750000	0.000778
3967.500000	0.000782

3967.250000	0.000785
3967.000000	0.000786
3966.750000	0.000786
3966.500000	0.000786
3966.250000	0.000787
3966.000000	0.000791
3965.750000	0.000798
3965.500000	0.000807
3965.250000	0.000818
3965.000000	0.000831
3964.750000	0.000843
3964.500000	0.000854
3964.250000	0.000865
3964.000000	0.000875
3963.750000	0.000885
3963.500000	0.000895
3963.250000	0.000904
3963.000000	0.000912
3962.750000	0.000918
3962.500000	0.000922
3962.250000	0.000922
3962.000000	0.000918
3961.750000	0.000911
3961.500000	0.000901
3961.250000	0.000889
3961.000000	0.000879

3960.750000	0.000870
3960.500000	0.000865
3960.250000	0.000864
3960.000000	0.000866
3959.750000	0.000870
3959.500000	0.000874
3959.250000	0.000876
3959.000000	0.000876
3958.750000	0.000873
3958.500000	0.000870
3958.250000	0.000867
3958.000000	0.000867
3957.750000	0.000869
3957.500000	0.000875
3957.250000	0.000882
3957.000000	0.000888
3956.750000	0.000891
3956.500000	0.000889
3956.250000	0.000882
3956.000000	0.000870
3955.750000	0.000855
3955.500000	0.000840
3955.250000	0.000828
3955.000000	0.000821
3954.750000	0.000822
3954.500000	0.000830

3954.250000	0.000844
3954.000000	0.000862
3953.750000	0.000881
3953.500000	0.000898
3953.250000	0.000910
3953.000000	0.000916
3952.750000	0.000916
3952.500000	0.000913
3952.250000	0.000910
3952.000000	0.000909
3951.750000	0.000914
3951.500000	0.000927
3951.250000	0.000947
3951.000000	0.000971
3950.750000	0.000997
3950.500000	0.001019
3950.250000	0.001035
3950.000000	0.001043
3949.750000	0.001042
3949.500000	0.001037
3949.250000	0.001031
3949.000000	0.001026
3948.750000	0.001025
3948.500000	0.001028
3948.250000	0.001032
3948.000000	0.001034

3947.750000	0.001033
3947.500000	0.001025
3947.250000	0.001012
3947.000000	0.000995
3946.750000	0.000980
3946.500000	0.000969
3946.250000	0.000965
3946.000000	0.000969
3945.750000	0.000981
3945.500000	0.000998
3945.250000	0.001016
3945.000000	0.001033
3944.750000	0.001044
3944.500000	0.001048
3944.250000	0.001044
3944.000000	0.001033
3943.750000	0.001018
3943.500000	0.001001
3943.250000	0.000984
3943.000000	0.000968
3942.750000	0.000954
3942.500000	0.000943
3942.250000	0.000935
3942.000000	0.000932
3941.750000	0.000935
3941.500000	0.000946

3941.250000	0.000964
3941.000000	0.000989
3940.750000	0.001019
3940.500000	0.001051
3940.250000	0.001082
3940.000000	0.001109
3939.750000	0.001130
3939.500000	0.001142
3939.250000	0.001146
3939.000000	0.001142
3938.750000	0.001133
3938.500000	0.001120
3938.250000	0.001105
3938.000000	0.001091
3937.750000	0.001078
3937.500000	0.001067
3937.250000	0.001058
3937.000000	0.001051
3936.750000	0.001048
3936.500000	0.001047
3936.250000	0.001049
3936.000000	0.001052
3935.750000	0.001055
3935.500000	0.001056
3935.250000	0.001055
3935.000000	0.001051

3934.750000	0.001045
3934.500000	0.001037
3934.250000	0.001029
3934.000000	0.001024
3933.750000	0.001025
3933.500000	0.001033
3933.250000	0.001050
3933.000000	0.001073
3932.750000	0.001100
3932.500000	0.001125
3932.250000	0.001143
3932.000000	0.001152
3931.750000	0.001151
3931.500000	0.001143
3931.250000	0.001131
3931.000000	0.001119
3930.750000	0.001109
3930.500000	0.001103
3930.250000	0.001101
3930.000000	0.001103
3929.750000	0.001105
3929.500000	0.001107
3929.250000	0.001107
3929.000000	0.001105
3928.750000	0.001104
3928.500000	0.001104

3928.250000	0.001107
3928.000000	0.001114
3927.750000	0.001125
3927.500000	0.001137
3927.250000	0.001149
3927.000000	0.001158
3926.750000	0.001163
3926.500000	0.001162
3926.250000	0.001156
3926.000000	0.001146
3925.750000	0.001136
3925.500000	0.001128
3925.250000	0.001123
3925.000000	0.001125
3924.750000	0.001130
3924.500000	0.001139
3924.250000	0.001149
3924.000000	0.001158
3923.750000	0.001165
3923.500000	0.001168
3923.250000	0.001168
3923.000000	0.001166
3922.750000	0.001161
3922.500000	0.001155
3922.250000	0.001148
3922.000000	0.001141

3921.750000	0.001136
3921.500000	0.001134
3921.250000	0.001136
3921.000000	0.001145
3920.750000	0.001157
3920.500000	0.001170
3920.250000	0.001178
3920.000000	0.001178
3919.750000	0.001167
3919.500000	0.001148
3919.250000	0.001122
3919.000000	0.001096
3918.750000	0.001074
3918.500000	0.001060
3918.250000	0.001059
3918.000000	0.001072
3917.750000	0.001095
3917.500000	0.001122
3917.250000	0.001144
3917.000000	0.001153
3916.750000	0.001146
3916.500000	0.001125
3916.250000	0.001097
3916.000000	0.001070
3915.750000	0.001052
3915.500000	0.001046

3915.250000	0.001054
3915.000000	0.001075
3914.750000	0.001105
3914.500000	0.001139
3914.250000	0.001172
3914.000000	0.001201
3913.750000	0.001225
3913.500000	0.001242
3913.250000	0.001254
3913.000000	0.001261
3912.750000	0.001264
3912.500000	0.001264
3912.250000	0.001260
3912.000000	0.001252
3911.750000	0.001241
3911.500000	0.001227
3911.250000	0.001211
3911.000000	0.001195
3910.750000	0.001179
3910.500000	0.001166
3910.250000	0.001156
3910.000000	0.001151
3909.750000	0.001149
3909.500000	0.001151
3909.250000	0.001154
3909.000000	0.001158

3908.750000	0.001163
3908.500000	0.001168
3908.250000	0.001175
3908.000000	0.001183
3907.750000	0.001193
3907.500000	0.001203
3907.250000	0.001213
3907.000000	0.001219
3906.750000	0.001219
3906.500000	0.001211
3906.250000	0.001192
3906.000000	0.001164
3905.750000	0.001128
3905.500000	0.001087
3905.250000	0.001047
3905.000000	0.001011
3904.750000	0.000981
3904.500000	0.000958
3904.250000	0.000944
3904.000000	0.000939
3903.750000	0.000945
3903.500000	0.000961
3903.250000	0.000987
3903.000000	0.001020
3902.750000	0.001055
3902.500000	0.001088

3902.250000	0.001113
3902.000000	0.001126
3901.750000	0.001127
3901.500000	0.001116
3901.250000	0.001096
3901.000000	0.001074
3900.750000	0.001054
3900.500000	0.001044
3900.250000	0.001050
3900.000000	0.001078
3899.750000	0.001122
3899.500000	0.001170
3899.250000	0.001207
3899.000000	0.001222
3898.750000	0.001212
3898.500000	0.001183
3898.250000	0.001146
3898.000000	0.001113
3897.750000	0.001094
3897.500000	0.001094
3897.250000	0.001113
3897.000000	0.001145
3896.750000	0.001185
3896.500000	0.001223
3896.250000	0.001255
3896.000000	0.001279

3895.750000	0.001297
3895.500000	0.001311
3895.250000	0.001325
3895.000000	0.001340
3894.750000	0.001355
3894.500000	0.001367
3894.250000	0.001373
3894.000000	0.001369
3893.750000	0.001355
3893.500000	0.001332
3893.250000	0.001301
3893.000000	0.001266
3892.750000	0.001231
3892.500000	0.001202
3892.250000	0.001182
3892.000000	0.001174
3891.750000	0.001172
3891.500000	0.001171
3891.250000	0.001162
3891.000000	0.001144
3890.750000	0.001120
3890.500000	0.001100
3890.250000	0.001091
3890.000000	0.001097
3889.750000	0.001121
3889.500000	0.001160

3889.250000	0.001208
3889.000000	0.001258
3888.750000	0.001300
3888.500000	0.001326
3888.250000	0.001330
3888.000000	0.001308
3887.750000	0.001260
3887.500000	0.001191
3887.250000	0.001111
3887.000000	0.001036
3886.750000	0.000980
3886.500000	0.000957
3886.250000	0.000968
3886.000000	0.001000
3885.750000	0.001038
3885.500000	0.001068
3885.250000	0.001087
3885.000000	0.001100
3884.750000	0.001112
3884.500000	0.001128
3884.250000	0.001151
3884.000000	0.001181
3883.750000	0.001219
3883.500000	0.001261
3883.250000	0.001302
3883.000000	0.001336

3882.750000	0.001357
3882.500000	0.001365
3882.250000	0.001359
3882.000000	0.001344
3881.750000	0.001328
3881.500000	0.001320
3881.250000	0.001325
3881.000000	0.001341
3880.750000	0.001355
3880.500000	0.001355
3880.250000	0.001328
3880.000000	0.001278
3879.750000	0.001214
3879.500000	0.001154
3879.250000	0.001109
3879.000000	0.001089
3878.750000	0.001097
3878.500000	0.001131
3878.250000	0.001185
3878.000000	0.001251
3877.750000	0.001318
3877.500000	0.001379
3877.250000	0.001426
3877.000000	0.001456
3876.750000	0.001470
3876.500000	0.001468

3876.250000	0.001453
3876.000000	0.001429
3875.750000	0.001399
3875.500000	0.001370
3875.250000	0.001347
3875.000000	0.001336
3874.750000	0.001334
3874.500000	0.001336
3874.250000	0.001333
3874.000000	0.001319
3873.750000	0.001293
3873.500000	0.001263
3873.250000	0.001237
3873.000000	0.001223
3872.750000	0.001224
3872.500000	0.001239
3872.250000	0.001263
3872.000000	0.001290
3871.750000	0.001311
3871.500000	0.001323
3871.250000	0.001326
3871.000000	0.001327
3870.750000	0.001336
3870.500000	0.001354
3870.250000	0.001372
3870.000000	0.001375

3869.750000	0.001347
3869.500000	0.001290
3869.250000	0.001219
3869.000000	0.001156
3868.750000	0.001120
3868.500000	0.001121
3868.250000	0.001161
3868.000000	0.001231
3867.750000	0.001319
3867.500000	0.001407
3867.250000	0.001481
3867.000000	0.001527
3866.750000	0.001540
3866.500000	0.001524
3866.250000	0.001488
3866.000000	0.001447
3865.750000	0.001416
3865.500000	0.001399
3865.250000	0.001392
3865.000000	0.001385
3864.750000	0.001371
3864.500000	0.001349
3864.250000	0.001326
3864.000000	0.001311
3863.750000	0.001312
3863.500000	0.001332

3863.250000	0.001373
3863.000000	0.001434
3862.750000	0.001507
3862.500000	0.001584
3862.250000	0.001649
3862.000000	0.001692
3861.750000	0.001706
3861.500000	0.001691
3861.250000	0.001655
3861.000000	0.001610
3860.750000	0.001565
3860.500000	0.001529
3860.250000	0.001508
3860.000000	0.001502
3859.750000	0.001511
3859.500000	0.001530
3859.250000	0.001557
3859.000000	0.001585
3858.750000	0.001613
3858.500000	0.001638
3858.250000	0.001659
3858.000000	0.001676
3857.750000	0.001688
3857.500000	0.001690
3857.250000	0.001680
3857.000000	0.001653

3856.750000	0.001607
3856.500000	0.001539
3856.250000	0.001446
3856.000000	0.001326
3855.750000	0.001178
3855.500000	0.001010
3855.250000	0.000838
3855.000000	0.000691
3854.750000	0.000592
3854.500000	0.000555
3854.250000	0.000570
3854.000000	0.000608
3853.750000	0.000642
3853.500000	0.000658
3853.250000	0.000661
3853.000000	0.000665
3852.750000	0.000683
3852.500000	0.000727
3852.250000	0.000804
3852.000000	0.000909
3851.750000	0.001034
3851.500000	0.001164
3851.250000	0.001287
3851.000000	0.001393
3850.750000	0.001477
3850.500000	0.001541

3850.250000	0.001590
3850.000000	0.001632
3849.750000	0.001670
3849.500000	0.001703
3849.250000	0.001727
3849.000000	0.001739
3848.750000	0.001737
3848.500000	0.001720
3848.250000	0.001694
3848.000000	0.001664
3847.750000	0.001636
3847.500000	0.001613
3847.250000	0.001598
3847.000000	0.001587
3846.750000	0.001580
3846.500000	0.001573
3846.250000	0.001562
3846.000000	0.001548
3845.750000	0.001530
3845.500000	0.001510
3845.250000	0.001494
3845.000000	0.001486
3844.750000	0.001491
3844.500000	0.001509
3844.250000	0.001537
3844.000000	0.001564

3843.750000	0.001582
3843.500000	0.001585
3843.250000	0.001573
3843.000000	0.001550
3842.750000	0.001523
3842.500000	0.001497
3842.250000	0.001481
3842.000000	0.001479
3841.750000	0.001495
3841.500000	0.001527
3841.250000	0.001568
3841.000000	0.001605
3840.750000	0.001627
3840.500000	0.001625
3840.250000	0.001598
3840.000000	0.001550
3839.750000	0.001487
3839.500000	0.001419
3839.250000	0.001357
3839.000000	0.001313
3838.750000	0.001299
3838.500000	0.001314
3838.250000	0.001348
3838.000000	0.001379
3837.750000	0.001392
3837.500000	0.001382

3837.250000	0.001358
3837.000000	0.001328
3836.750000	0.001302
3836.500000	0.001284
3836.250000	0.001282
3836.000000	0.001300
3835.750000	0.001341
3835.500000	0.001395
3835.250000	0.001450
3835.000000	0.001488
3834.750000	0.001499
3834.500000	0.001484
3834.250000	0.001449
3834.000000	0.001407
3833.750000	0.001368
3833.500000	0.001339
3833.250000	0.001329
3833.000000	0.001338
3832.750000	0.001369
3832.500000	0.001419
3832.250000	0.001477
3832.000000	0.001532
3831.750000	0.001569
3831.500000	0.001581
3831.250000	0.001569
3831.000000	0.001538

3830.750000	0.001501
3830.500000	0.001466
3830.250000	0.001441
3830.000000	0.001430
3829.750000	0.001434
3829.500000	0.001451
3829.250000	0.001478
3829.000000	0.001510
3828.750000	0.001541
3828.500000	0.001569
3828.250000	0.001591
3828.000000	0.001606
3827.750000	0.001617
3827.500000	0.001624
3827.250000	0.001626
3827.000000	0.001622
3826.750000	0.001611
3826.500000	0.001594
3826.250000	0.001574
3826.000000	0.001556
3825.750000	0.001544
3825.500000	0.001540
3825.250000	0.001547
3825.000000	0.001561
3824.750000	0.001578
3824.500000	0.001594

3824.250000	0.001602
3824.000000	0.001596
3823.750000	0.001570
3823.500000	0.001522
3823.250000	0.001450
3823.000000	0.001360
3822.750000	0.001261
3822.500000	0.001170
3822.250000	0.001107
3822.000000	0.001093
3821.750000	0.001135
3821.500000	0.001220
3821.250000	0.001315
3821.000000	0.001385
3820.750000	0.001412
3820.500000	0.001401
3820.250000	0.001371
3820.000000	0.001343
3819.750000	0.001331
3819.500000	0.001340
3819.250000	0.001371
3819.000000	0.001417
3818.750000	0.001467
3818.500000	0.001510
3818.250000	0.001535
3818.000000	0.001534

3817.750000	0.001506
3817.500000	0.001457
3817.250000	0.001402
3817.000000	0.001358
3816.750000	0.001337
3816.500000	0.001339
3816.250000	0.001349
3816.000000	0.001349
3815.750000	0.001331
3815.500000	0.001300
3815.250000	0.001269
3815.000000	0.001253
3814.750000	0.001257
3814.500000	0.001284
3814.250000	0.001333
3814.000000	0.001399
3813.750000	0.001473
3813.500000	0.001546
3813.250000	0.001608
3813.000000	0.001651
3812.750000	0.001671
3812.500000	0.001671
3812.250000	0.001656
3812.000000	0.001634
3811.750000	0.001611
3811.500000	0.001594

3811.250000	0.001584
3811.000000	0.001580
3810.750000	0.001579
3810.500000	0.001576
3810.250000	0.001569
3810.000000	0.001554
3809.750000	0.001528
3809.500000	0.001492
3809.250000	0.001445
3809.000000	0.001389
3808.750000	0.001327
3808.500000	0.001267
3808.250000	0.001218
3808.000000	0.001193
3807.750000	0.001200
3807.500000	0.001236
3807.250000	0.001288
3807.000000	0.001334
3806.750000	0.001361
3806.500000	0.001363
3806.250000	0.001351
3806.000000	0.001336
3805.750000	0.001330
3805.500000	0.001340
3805.250000	0.001370
3805.000000	0.001417

3804.750000	0.001477
3804.500000	0.001539
3804.250000	0.001592
3804.000000	0.001625
3803.750000	0.001628
3803.500000	0.001597
3803.250000	0.001532
3803.000000	0.001441
3802.750000	0.001336
3802.500000	0.001239
3802.250000	0.001170
3802.000000	0.001145
3801.750000	0.001162
3801.500000	0.001202
3801.250000	0.001243
3801.000000	0.001272
3800.750000	0.001289
3800.500000	0.001301
3800.250000	0.001314
3800.000000	0.001332
3799.750000	0.001354
3799.500000	0.001382
3799.250000	0.001415
3799.000000	0.001450
3798.750000	0.001482
3798.500000	0.001507

3798.250000	0.001520
3798.000000	0.001523
3797.750000	0.001519
3797.500000	0.001515
3797.250000	0.001516
3797.000000	0.001525
3796.750000	0.001535
3796.500000	0.001541
3796.250000	0.001536
3796.000000	0.001519
3795.750000	0.001491
3795.500000	0.001460
3795.250000	0.001429
3795.000000	0.001405
3794.750000	0.001392
3794.500000	0.001391
3794.250000	0.001402
3794.000000	0.001421
3793.750000	0.001443
3793.500000	0.001464
3793.250000	0.001478
3793.000000	0.001485
3792.750000	0.001483
3792.500000	0.001476
3792.250000	0.001465
3792.000000	0.001455

3791.750000	0.001447
3791.500000	0.001442
3791.250000	0.001442
3791.000000	0.001445
3790.750000	0.001450
3790.500000	0.001458
3790.250000	0.001466
3790.000000	0.001473
3789.750000	0.001479
3789.500000	0.001482
3789.250000	0.001483
3789.000000	0.001481
3788.750000	0.001478
3788.500000	0.001474
3788.250000	0.001468
3788.000000	0.001461
3787.750000	0.001454
3787.500000	0.001446
3787.250000	0.001438
3787.000000	0.001433
3786.750000	0.001431
3786.500000	0.001437
3786.250000	0.001451
3786.000000	0.001473
3785.750000	0.001504
3785.500000	0.001539

3785.250000	0.001572
3785.000000	0.001598
3784.750000	0.001613
3784.500000	0.001614
3784.250000	0.001603
3784.000000	0.001584
3783.750000	0.001562
3783.500000	0.001541
3783.250000	0.001527
3783.000000	0.001519
3782.750000	0.001518
3782.500000	0.001520
3782.250000	0.001522
3782.000000	0.001521
3781.750000	0.001513
3781.500000	0.001497
3781.250000	0.001475
3781.000000	0.001451
3780.750000	0.001429
3780.500000	0.001418
3780.250000	0.001419
3780.000000	0.001431
3779.750000	0.001447
3779.500000	0.001458
3779.250000	0.001457
3779.000000	0.001443

3778.750000	0.001419
3778.500000	0.001394
3778.250000	0.001374
3778.000000	0.001366
3777.750000	0.001373
3777.500000	0.001394
3777.250000	0.001424
3777.000000	0.001458
3776.750000	0.001486
3776.500000	0.001505
3776.250000	0.001510
3776.000000	0.001504
3775.750000	0.001490
3775.500000	0.001474
3775.250000	0.001462
3775.000000	0.001456
3774.750000	0.001458
3774.500000	0.001469
3774.250000	0.001485
3774.000000	0.001503
3773.750000	0.001521
3773.500000	0.001537
3773.250000	0.001547
3773.000000	0.001551
3772.750000	0.001547
3772.500000	0.001533

3772.250000	0.001509
3772.000000	0.001475
3771.750000	0.001432
3771.500000	0.001381
3771.250000	0.001332
3771.000000	0.001292
3770.750000	0.001273
3770.500000	0.001277
3770.250000	0.001301
3770.000000	0.001333
3769.750000	0.001358
3769.500000	0.001369
3769.250000	0.001366
3769.000000	0.001356
3768.750000	0.001349
3768.500000	0.001350
3768.250000	0.001366
3768.000000	0.001396
3767.750000	0.001438
3767.500000	0.001487
3767.250000	0.001534
3767.000000	0.001576
3766.750000	0.001608
3766.500000	0.001630
3766.250000	0.001641
3766.000000	0.001641

3765.750000	0.001628
3765.500000	0.001605
3765.250000	0.001572
3765.000000	0.001536
3764.750000	0.001500
3764.500000	0.001471
3764.250000	0.001452
3764.000000	0.001446
3763.750000	0.001451
3763.500000	0.001464
3763.250000	0.001482
3763.000000	0.001500
3762.750000	0.001513
3762.500000	0.001519
3762.250000	0.001517
3762.000000	0.001508
3761.750000	0.001494
3761.500000	0.001474
3761.250000	0.001454
3761.000000	0.001435
3760.750000	0.001423
3760.500000	0.001422
3760.250000	0.001430
3760.000000	0.001442
3759.750000	0.001449
3759.500000	0.001446

3759.250000	0.001431
3759.000000	0.001409
3758.750000	0.001388
3758.500000	0.001375
3758.250000	0.001375
3758.000000	0.001392
3757.750000	0.001426
3757.500000	0.001474
3757.250000	0.001530
3757.000000	0.001587
3756.750000	0.001635
3756.500000	0.001669
3756.250000	0.001685
3756.000000	0.001686
3755.750000	0.001674
3755.500000	0.001655
3755.250000	0.001630
3755.000000	0.001601
3754.750000	0.001566
3754.500000	0.001523
3754.250000	0.001466
3754.000000	0.001395
3753.750000	0.001310
3753.500000	0.001220
3753.250000	0.001142
3753.000000	0.001093

3752.750000	0.001084
3752.500000	0.001107
3752.250000	0.001144
3752.000000	0.001173
3751.750000	0.001183
3751.500000	0.001172
3751.250000	0.001144
3751.000000	0.001106
3750.750000	0.001069
3750.500000	0.001056
3750.250000	0.001091
3750.000000	0.001180
3749.750000	0.001309
3749.500000	0.001439
3749.250000	0.001539
3749.000000	0.001594
3748.750000	0.001611
3748.500000	0.001606
3748.250000	0.001590
3748.000000	0.001573
3747.750000	0.001559
3747.500000	0.001549
3747.250000	0.001538
3747.000000	0.001514
3746.750000	0.001464
3746.500000	0.001375

3746.250000	0.001240
3746.000000	0.001059
3745.750000	0.000858
3745.500000	0.000684
3745.250000	0.000590
3745.000000	0.000601
3744.750000	0.000682
3744.500000	0.000761
3744.250000	0.000782
3744.000000	0.000752
3743.750000	0.000721
3743.500000	0.000733
3743.250000	0.000802
3743.000000	0.000920
3742.750000	0.001072
3742.500000	0.001242
3742.250000	0.001415
3742.000000	0.001577
3741.750000	0.001711
3741.500000	0.001803
3741.250000	0.001847
3741.000000	0.001846
3740.750000	0.001809
3740.500000	0.001749
3740.250000	0.001677
3740.000000	0.001606

3739.750000	0.001544
3739.500000	0.001501
3739.250000	0.001481
3739.000000	0.001488
3738.750000	0.001517
3738.500000	0.001561
3738.250000	0.001608
3738.000000	0.001647
3737.750000	0.001669
3737.500000	0.001670
3737.250000	0.001650
3737.000000	0.001613
3736.750000	0.001566
3736.500000	0.001521
3736.250000	0.001487
3736.000000	0.001473
3735.750000	0.001477
3735.500000	0.001488
3735.250000	0.001489
3735.000000	0.001471
3734.750000	0.001432
3734.500000	0.001384
3734.250000	0.001339
3734.000000	0.001304
3733.750000	0.001283
3733.500000	0.001278

3733.250000	0.001291
3733.000000	0.001321
3732.750000	0.001364
3732.500000	0.001408
3732.250000	0.001439
3732.000000	0.001448
3731.750000	0.001435
3731.500000	0.001406
3731.250000	0.001372
3731.000000	0.001346
3730.750000	0.001334
3730.500000	0.001341
3730.250000	0.001364
3730.000000	0.001400
3729.750000	0.001439
3729.500000	0.001473
3729.250000	0.001495
3729.000000	0.001498
3728.750000	0.001482
3728.500000	0.001448
3728.250000	0.001403
3728.000000	0.001354
3727.750000	0.001311
3727.500000	0.001280
3727.250000	0.001267
3727.000000	0.001273

3726.750000	0.001292
3726.500000	0.001320
3726.250000	0.001348
3726.000000	0.001371
3725.750000	0.001383
3725.500000	0.001385
3725.250000	0.001377
3725.000000	0.001366
3724.750000	0.001355
3724.500000	0.001350
3724.250000	0.001354
3724.000000	0.001366
3723.750000	0.001387
3723.500000	0.001416
3723.250000	0.001453
3723.000000	0.001492
3722.750000	0.001527
3722.500000	0.001550
3722.250000	0.001552
3722.000000	0.001532
3721.750000	0.001494
3721.500000	0.001448
3721.250000	0.001404
3721.000000	0.001372
3720.750000	0.001359
3720.500000	0.001367

3720.250000	0.001396
3720.000000	0.001440
3719.750000	0.001490
3719.500000	0.001537
3719.250000	0.001571
3719.000000	0.001588
3718.750000	0.001588
3718.500000	0.001576
3718.250000	0.001559
3718.000000	0.001544
3717.750000	0.001538
3717.500000	0.001542
3717.250000	0.001556
3717.000000	0.001576
3716.750000	0.001598
3716.500000	0.001618
3716.250000	0.001631
3716.000000	0.001636
3715.750000	0.001631
3715.500000	0.001617
3715.250000	0.001595
3715.000000	0.001565
3714.750000	0.001528
3714.500000	0.001482
3714.250000	0.001427
3714.000000	0.001362

3713.750000	0.001292
3713.500000	0.001225
3713.250000	0.001174
3713.000000	0.001154
3712.750000	0.001172
3712.500000	0.001221
3712.250000	0.001278
3712.000000	0.001322
3711.750000	0.001340
3711.500000	0.001334
3711.250000	0.001312
3711.000000	0.001288
3710.750000	0.001274
3710.500000	0.001278
3710.250000	0.001306
3710.000000	0.001355
3709.750000	0.001414
3709.500000	0.001464
3709.250000	0.001494
3709.000000	0.001498
3708.750000	0.001483
3708.500000	0.001459
3708.250000	0.001436
3708.000000	0.001422
3707.750000	0.001423
3707.500000	0.001438

3707.250000	0.001465
3707.000000	0.001498
3706.750000	0.001532
3706.500000	0.001561
3706.250000	0.001580
3706.000000	0.001586
3705.750000	0.001579
3705.500000	0.001559
3705.250000	0.001529
3705.000000	0.001494
3704.750000	0.001458
3704.500000	0.001424
3704.250000	0.001394
3704.000000	0.001371
3703.750000	0.001353
3703.500000	0.001341
3703.250000	0.001337
3703.000000	0.001343
3702.750000	0.001359
3702.500000	0.001384
3702.250000	0.001413
3702.000000	0.001435
3701.750000	0.001442
3701.500000	0.001431
3701.250000	0.001405
3701.000000	0.001375

3700.750000	0.001352
3700.500000	0.001344
3700.250000	0.001356
3700.000000	0.001389
3699.750000	0.001436
3699.500000	0.001491
3699.250000	0.001543
3699.000000	0.001585
3698.750000	0.001614
3698.500000	0.001630
3698.250000	0.001635
3698.000000	0.001635
3697.750000	0.001632
3697.500000	0.001631
3697.250000	0.001630
3697.000000	0.001631
3696.750000	0.001631
3696.500000	0.001630
3696.250000	0.001628
3696.000000	0.001626
3695.750000	0.001623
3695.500000	0.001622
3695.250000	0.001622
3695.000000	0.001623
3694.750000	0.001626
3694.500000	0.001632

3694.250000	0.001639
3694.000000	0.001646
3693.750000	0.001651
3693.500000	0.001651
3693.250000	0.001641
3693.000000	0.001621
3692.750000	0.001592
3692.500000	0.001558
3692.250000	0.001527
3692.000000	0.001509
3691.750000	0.001505
3691.500000	0.001510
3691.250000	0.001513
3691.000000	0.001501
3690.750000	0.001469
3690.500000	0.001416
3690.250000	0.001346
3690.000000	0.001270
3689.750000	0.001204
3689.500000	0.001172
3689.250000	0.001193
3689.000000	0.001273
3688.750000	0.001391
3688.500000	0.001512
3688.250000	0.001603
3688.000000	0.001652

3687.750000	0.001664
3687.500000	0.001657
3687.250000	0.001647
3687.000000	0.001644
3686.750000	0.001656
3686.500000	0.001682
3686.250000	0.001721
3686.000000	0.001766
3685.750000	0.001808
3685.500000	0.001839
3685.250000	0.001856
3685.000000	0.001858
3684.750000	0.001851
3684.500000	0.001840
3684.250000	0.001832
3684.000000	0.001830
3683.750000	0.001838
3683.500000	0.001853
3683.250000	0.001873
3683.000000	0.001894
3682.750000	0.001911
3682.500000	0.001921
3682.250000	0.001923
3682.000000	0.001917
3681.750000	0.001905
3681.500000	0.001889

3681.250000	0.001873
3681.000000	0.001858
3680.750000	0.001847
3680.500000	0.001845
3680.250000	0.001853
3680.000000	0.001870
3679.750000	0.001894
3679.500000	0.001920
3679.250000	0.001943
3679.000000	0.001960
3678.750000	0.001966
3678.500000	0.001962
3678.250000	0.001945
3678.000000	0.001915
3677.750000	0.001869
3677.500000	0.001807
3677.250000	0.001729
3677.000000	0.001642
3676.750000	0.001558
3676.500000	0.001500
3676.250000	0.001496
3676.000000	0.001566
3675.750000	0.001694
3675.500000	0.001833
3675.250000	0.001930
3675.000000	0.001961

3674.750000	0.001941
3674.500000	0.001903
3674.250000	0.001877
3674.000000	0.001875
3673.750000	0.001901
3673.500000	0.001946
3673.250000	0.001999
3673.000000	0.002044
3672.750000	0.002066
3672.500000	0.002058
3672.250000	0.002020
3672.000000	0.001963
3671.750000	0.001908
3671.500000	0.001873
3671.250000	0.001870
3671.000000	0.001895
3670.750000	0.001935
3670.500000	0.001973
3670.250000	0.001998
3670.000000	0.002011
3669.750000	0.002018
3669.500000	0.002027
3669.250000	0.002044
3669.000000	0.002070
3668.750000	0.002106
3668.500000	0.002145

3668.250000	0.002185
3668.000000	0.002221
3667.750000	0.002251
3667.500000	0.002276
3667.250000	0.002297
3667.000000	0.002317
3666.750000	0.002334
3666.500000	0.002351
3666.250000	0.002364
3666.000000	0.002374
3665.750000	0.002378
3665.500000	0.002379
3665.250000	0.002379
3665.000000	0.002378
3664.750000	0.002379
3664.500000	0.002382
3664.250000	0.002386
3664.000000	0.002389
3663.750000	0.002391
3663.500000	0.002394
3663.250000	0.002397
3663.000000	0.002403
3662.750000	0.002412
3662.500000	0.002423
3662.250000	0.002433
3662.000000	0.002440

3661.750000	0.002444
3661.500000	0.002443
3661.250000	0.002439
3661.000000	0.002435
3660.750000	0.002434
3660.500000	0.002435
3660.250000	0.002440
3660.000000	0.002445
3659.750000	0.002450
3659.500000	0.002451
3659.250000	0.002449
3659.000000	0.002442
3658.750000	0.002433
3658.500000	0.002422
3658.250000	0.002409
3658.000000	0.002396
3657.750000	0.002385
3657.500000	0.002381
3657.250000	0.002392
3657.000000	0.002420
3656.750000	0.002464
3656.500000	0.002512
3656.250000	0.002552
3656.000000	0.002577
3655.750000	0.002587
3655.500000	0.002589

3655.250000	0.002592
3655.000000	0.002602
3654.750000	0.002619
3654.500000	0.002643
3654.250000	0.002667
3654.000000	0.002686
3653.750000	0.002693
3653.500000	0.002686
3653.250000	0.002663
3653.000000	0.002625
3652.750000	0.002579
3652.500000	0.002532
3652.250000	0.002491
3652.000000	0.002463
3651.750000	0.002453
3651.500000	0.002460
3651.250000	0.002482
3651.000000	0.002511
3650.750000	0.002544
3650.500000	0.002573
3650.250000	0.002598
3650.000000	0.002619
3649.750000	0.002638
3649.500000	0.002659
3649.250000	0.002688
3649.000000	0.002726

3648.750000	0.002773
3648.500000	0.002826
3648.250000	0.002884
3648.000000	0.002944
3647.750000	0.003002
3647.500000	0.003052
3647.250000	0.003089
3647.000000	0.003108
3646.750000	0.003110
3646.500000	0.003101
3646.250000	0.003087
3646.000000	0.003075
3645.750000	0.003069
3645.500000	0.003071
3645.250000	0.003082
3645.000000	0.003101
3644.750000	0.003126
3644.500000	0.003154
3644.250000	0.003182
3644.000000	0.003209
3643.750000	0.003235
3643.500000	0.003260
3643.250000	0.003286
3643.000000	0.003310
3642.750000	0.003332
3642.500000	0.003347

3642.250000	0.003354
3642.000000	0.003350
3641.750000	0.003338
3641.500000	0.003320
3641.250000	0.003300
3641.000000	0.003282
3640.750000	0.003268
3640.500000	0.003260
3640.250000	0.003257
3640.000000	0.003258
3639.750000	0.003262
3639.500000	0.003269
3639.250000	0.003280
3639.000000	0.003298
3638.750000	0.003322
3638.500000	0.003352
3638.250000	0.003385
3638.000000	0.003417
3637.750000	0.003443
3637.500000	0.003460
3637.250000	0.003466
3637.000000	0.003462
3636.750000	0.003451
3636.500000	0.003438
3636.250000	0.003426
3636.000000	0.003418

3635.750000	0.003416
3635.500000	0.003422
3635.250000	0.003436
3635.000000	0.003459
3634.750000	0.003490
3634.500000	0.003525
3634.250000	0.003560
3634.000000	0.003588
3633.750000	0.003604
3633.500000	0.003607
3633.250000	0.003599
3633.000000	0.003585
3632.750000	0.003570
3632.500000	0.003557
3632.250000	0.003546
3632.000000	0.003533
3631.750000	0.003510
3631.500000	0.003469
3631.250000	0.003403
3631.000000	0.003311
3630.750000	0.003200
3630.500000	0.003086
3630.250000	0.002995
3630.000000	0.002959
3629.750000	0.003001
3629.500000	0.003117

3629.250000	0.003273
3629.000000	0.003416
3628.750000	0.003504
3628.500000	0.003528
3628.250000	0.003512
3628.000000	0.003490
3627.750000	0.003487
3627.500000	0.003516
3627.250000	0.003577
3627.000000	0.003661
3626.750000	0.003754
3626.500000	0.003841
3626.250000	0.003911
3626.000000	0.003960
3625.750000	0.003988
3625.500000	0.004001
3625.250000	0.004005
3625.000000	0.004004
3624.750000	0.004002
3624.500000	0.003998
3624.250000	0.003993
3624.000000	0.003988
3623.750000	0.003980
3623.500000	0.003971
3623.250000	0.003958
3623.000000	0.003941

3622.750000	0.003920
3622.500000	0.003895
3622.250000	0.003868
3622.000000	0.003842
3621.750000	0.003818
3621.500000	0.003799
3621.250000	0.003785
3621.000000	0.003781
3620.750000	0.003792
3620.500000	0.003819
3620.250000	0.003864
3620.000000	0.003921
3619.750000	0.003982
3619.500000	0.004041
3619.250000	0.004093
3619.000000	0.004137
3618.750000	0.004178
3618.500000	0.004218
3618.250000	0.004256
3618.000000	0.004289
3617.750000	0.004313
3617.500000	0.004321
3617.250000	0.004313
3617.000000	0.004292
3616.750000	0.004264
3616.500000	0.004240

3616.250000	0.004230
3616.000000	0.004243
3615.750000	0.004280
3615.500000	0.004337
3615.250000	0.004401
3615.000000	0.004454
3614.750000	0.004483
3614.500000	0.004482
3614.250000	0.004453
3614.000000	0.004408
3613.750000	0.004366
3613.500000	0.004345
3613.250000	0.004355
3613.000000	0.004392
3612.750000	0.004439
3612.500000	0.004476
3612.250000	0.004487
3612.000000	0.004465
3611.750000	0.004413
3611.500000	0.004343
3611.250000	0.004267
3611.000000	0.004198
3610.750000	0.004150
3610.500000	0.004135
3610.250000	0.004159
3610.000000	0.004221

3609.750000	0.004306
3609.500000	0.004395
3609.250000	0.004468
3609.000000	0.004515
3608.750000	0.004536
3608.500000	0.004539
3608.250000	0.004537
3608.000000	0.004543
3607.750000	0.004562
3607.500000	0.004596
3607.250000	0.004637
3607.000000	0.004674
3606.750000	0.004696
3606.500000	0.004698
3606.250000	0.004684
3606.000000	0.004661
3605.750000	0.004638
3605.500000	0.004624
3605.250000	0.004622
3605.000000	0.004633
3604.750000	0.004653
3604.500000	0.004676
3604.250000	0.004699
3604.000000	0.004715
3603.750000	0.004725
3603.500000	0.004728

3603.250000	0.004724
3603.000000	0.004717
3602.750000	0.004708
3602.500000	0.004701
3602.250000	0.004698
3602.000000	0.004704
3601.750000	0.004719
3601.500000	0.004743
3601.250000	0.004773
3601.000000	0.004805
3600.750000	0.004834
3600.500000	0.004857
3600.250000	0.004872
3600.000000	0.004881
3599.750000	0.004888
3599.500000	0.004895
3599.250000	0.004905
3599.000000	0.004915
3598.750000	0.004925
3598.500000	0.004930
3598.250000	0.004927
3598.000000	0.004912
3597.750000	0.004888
3597.500000	0.004858
3597.250000	0.004829
3597.000000	0.004808

3596.750000	0.004802
3596.500000	0.004814
3596.250000	0.004843
3596.000000	0.004881
3595.750000	0.004918
3595.500000	0.004943
3595.250000	0.004948
3595.000000	0.004933
3594.750000	0.004907
3594.500000	0.004880
3594.250000	0.004867
3594.000000	0.004875
3593.750000	0.004909
3593.500000	0.004962
3593.250000	0.005021
3593.000000	0.005072
3592.750000	0.005104
3592.500000	0.005111
3592.250000	0.005097
3592.000000	0.005067
3591.750000	0.005029
3591.500000	0.004990
3591.250000	0.004954
3591.000000	0.004923
3590.750000	0.004894
3590.500000	0.004867

3590.250000	0.004841
3590.000000	0.004820
3589.750000	0.004809
3589.500000	0.004810
3589.250000	0.004823
3589.000000	0.004844
3588.750000	0.004863
3588.500000	0.004876
3588.250000	0.004883
3588.000000	0.004890
3587.750000	0.004909
3587.500000	0.004949
3587.250000	0.005012
3587.000000	0.005089
3586.750000	0.005162
3586.500000	0.005215
3586.250000	0.005239
3586.000000	0.005233
3585.750000	0.005205
3585.500000	0.005170
3585.250000	0.005139
3585.000000	0.005124
3584.750000	0.005129
3584.500000	0.005154
3584.250000	0.005193
3584.000000	0.005239

3583.750000	0.005281
3583.500000	0.005315
3583.250000	0.005334
3583.000000	0.005339
3582.750000	0.005330
3582.500000	0.005311
3582.250000	0.005286
3582.000000	0.005261
3581.750000	0.005241
3581.500000	0.005231
3581.250000	0.005233
3581.000000	0.005247
3580.750000	0.005269
3580.500000	0.005292
3580.250000	0.005310
3580.000000	0.005320
3579.750000	0.005320
3579.500000	0.005313
3579.250000	0.005303
3579.000000	0.005296
3578.750000	0.005296
3578.500000	0.005306
3578.250000	0.005325
3578.000000	0.005351
3577.750000	0.005377
3577.500000	0.005399

3577.250000	0.005414
3577.000000	0.005419
3576.750000	0.005416
3576.500000	0.005406
3576.250000	0.005394
3576.000000	0.005384
3575.750000	0.005381
3575.500000	0.005387
3575.250000	0.005403
3575.000000	0.005427
3574.750000	0.005454
3574.500000	0.005479
3574.250000	0.005497
3574.000000	0.005503
3573.750000	0.005498
3573.500000	0.005483
3573.250000	0.005464
3573.000000	0.005445
3572.750000	0.005432
3572.500000	0.005427
3572.250000	0.005433
3572.000000	0.005445
3571.750000	0.005460
3571.500000	0.005472
3571.250000	0.005475
3571.000000	0.005467

3570.750000	0.005446
3570.500000	0.005413
3570.250000	0.005374
3570.000000	0.005335
3569.750000	0.005302
3569.500000	0.005284
3569.250000	0.005286
3569.000000	0.005311
3568.750000	0.005357
3568.500000	0.005417
3568.250000	0.005482
3568.000000	0.005543
3567.750000	0.005601
3567.500000	0.005656
3567.250000	0.005715
3567.000000	0.005781
3566.750000	0.005852
3566.500000	0.005918
3566.250000	0.005969
3566.000000	0.005996
3565.750000	0.005995
3565.500000	0.005970
3565.250000	0.005931
3565.000000	0.005888
3564.750000	0.005853
3564.500000	0.005833

3564.250000	0.005830
3564.000000	0.005841
3563.750000	0.005859
3563.500000	0.005874
3563.250000	0.005879
3563.000000	0.005869
3562.750000	0.005844
3562.500000	0.005808
3562.250000	0.005769
3562.000000	0.005733
3561.750000	0.005709
3561.500000	0.005699
3561.250000	0.005707
3561.000000	0.005730
3560.750000	0.005762
3560.500000	0.005796
3560.250000	0.005824
3560.000000	0.005839
3559.750000	0.005840
3559.500000	0.005829
3559.250000	0.005811
3559.000000	0.005793
3558.750000	0.005783
3558.500000	0.005783
3558.250000	0.005797
3558.000000	0.005820

3557.750000	0.005850
3557.500000	0.005880
3557.250000	0.005904
3557.000000	0.005919
3556.750000	0.005924
3556.500000	0.005919
3556.250000	0.005908
3556.000000	0.005894
3555.750000	0.005879
3555.500000	0.005867
3555.250000	0.005859
3555.000000	0.005856
3554.750000	0.005856
3554.500000	0.005861
3554.250000	0.005870
3554.000000	0.005883
3553.750000	0.005904
3553.500000	0.005932
3553.250000	0.005969
3553.000000	0.006016
3552.750000	0.006069
3552.500000	0.006122
3552.250000	0.006168
3552.000000	0.006200
3551.750000	0.006213
3551.500000	0.006206

3551.250000	0.006182
3551.000000	0.006146
3550.750000	0.006107
3550.500000	0.006069
3550.250000	0.006038
3550.000000	0.006016
3549.750000	0.006002
3549.500000	0.005994
3549.250000	0.005991
3549.000000	0.005991
3548.750000	0.005994
3548.500000	0.006001
3548.250000	0.006014
3548.000000	0.006030
3547.750000	0.006050
3547.500000	0.006069
3547.250000	0.006086
3547.000000	0.006098
3546.750000	0.006107
3546.500000	0.006116
3546.250000	0.006127
3546.000000	0.006145
3545.750000	0.006171
3545.500000	0.006203
3545.250000	0.006237
3545.000000	0.006270

3544.750000	0.006296
3544.500000	0.006313
3544.250000	0.006322
3544.000000	0.006322
3543.750000	0.006318
3543.500000	0.006311
3543.250000	0.006302
3543.000000	0.006292
3542.750000	0.006280
3542.500000	0.006268
3542.250000	0.006255
3542.000000	0.006243
3541.750000	0.006233
3541.500000	0.006228
3541.250000	0.006229
3541.000000	0.006237
3540.750000	0.006250
3540.500000	0.006269
3540.250000	0.006291
3540.000000	0.006314
3539.750000	0.006335
3539.500000	0.006354
3539.250000	0.006369
3539.000000	0.006378
3538.750000	0.006380
3538.500000	0.006378

3538.250000	0.006372
3538.000000	0.006365
3537.750000	0.006362
3537.500000	0.006365
3537.250000	0.006376
3537.000000	0.006394
3536.750000	0.006415
3536.500000	0.006435
3536.250000	0.006446
3536.000000	0.006448
3535.750000	0.006438
3535.500000	0.006421
3535.250000	0.006402
3535.000000	0.006385
3534.750000	0.006376
3534.500000	0.006374
3534.250000	0.006380
3534.000000	0.006388
3533.750000	0.006396
3533.500000	0.006400
3533.250000	0.006398
3533.000000	0.006393
3532.750000	0.006388
3532.500000	0.006386
3532.250000	0.006390
3532.000000	0.006400

3531.750000	0.006416
3531.500000	0.006433
3531.250000	0.006449
3531.000000	0.006460
3530.750000	0.006466
3530.500000	0.006467
3530.250000	0.006467
3530.000000	0.006467
3529.750000	0.006471
3529.500000	0.006478
3529.250000	0.006490
3529.000000	0.006503
3528.750000	0.006514
3528.500000	0.006524
3528.250000	0.006532
3528.000000	0.006539
3527.750000	0.006550
3527.500000	0.006567
3527.250000	0.006592
3527.000000	0.006623
3526.750000	0.006660
3526.500000	0.006696
3526.250000	0.006728
3526.000000	0.006752
3525.750000	0.006764
3525.500000	0.006766

3525.250000	0.006759
3525.000000	0.006749
3524.750000	0.006741
3524.500000	0.006738
3524.250000	0.006745
3524.000000	0.006761
3523.750000	0.006785
3523.500000	0.006812
3523.250000	0.006837
3523.000000	0.006855
3522.750000	0.006862
3522.500000	0.006857
3522.250000	0.006843
3522.000000	0.006820
3521.750000	0.006793
3521.500000	0.006765
3521.250000	0.006740
3521.000000	0.006720
3520.750000	0.006706
3520.500000	0.006698
3520.250000	0.006695
3520.000000	0.006697
3519.750000	0.006702
3519.500000	0.006708
3519.250000	0.006713
3519.000000	0.006716

3518.750000	0.006716
3518.500000	0.006716
3518.250000	0.006714
3518.000000	0.006715
3517.750000	0.006717
3517.500000	0.006723
3517.250000	0.006731
3517.000000	0.006741
3516.750000	0.006751
3516.500000	0.006761
3516.250000	0.006768
3516.000000	0.006774
3515.750000	0.006779
3515.500000	0.006783
3515.250000	0.006787
3515.000000	0.006791
3514.750000	0.006793
3514.500000	0.006793
3514.250000	0.006792
3514.000000	0.006790
3513.750000	0.006789
3513.500000	0.006792
3513.250000	0.006800
3513.000000	0.006815
3512.750000	0.006836
3512.500000	0.006859

3512.250000	0.006879
3512.000000	0.006893
3511.750000	0.006898
3511.500000	0.006895
3511.250000	0.006885
3511.000000	0.006875
3510.750000	0.006872
3510.500000	0.006882
3510.250000	0.006908
3510.000000	0.006948
3509.750000	0.006997
3509.500000	0.007044
3509.250000	0.007080
3509.000000	0.007100
3508.750000	0.007102
3508.500000	0.007089
3508.250000	0.007067
3508.000000	0.007044
3507.750000	0.007027
3507.500000	0.007018
3507.250000	0.007018
3507.000000	0.007024
3506.750000	0.007032
3506.500000	0.007036
3506.250000	0.007035
3506.000000	0.007027

3505.750000	0.007014
3505.500000	0.006999
3505.250000	0.006985
3505.000000	0.006978
3504.750000	0.006978
3504.500000	0.006987
3504.250000	0.007005
3504.000000	0.007031
3503.750000	0.007061
3503.500000	0.007093
3503.250000	0.007125
3503.000000	0.007155
3502.750000	0.007179
3502.500000	0.007198
3502.250000	0.007212
3502.000000	0.007219
3501.750000	0.007221
3501.500000	0.007219
3501.250000	0.007214
3501.000000	0.007207
3500.750000	0.007199
3500.500000	0.007191
3500.250000	0.007184
3500.000000	0.007177
3499.750000	0.007171
3499.500000	0.007164

3499.250000	0.007157
3499.000000	0.007150
3498.750000	0.007144
3498.500000	0.007140
3498.250000	0.007141
3498.000000	0.007147
3497.750000	0.007161
3497.500000	0.007181
3497.250000	0.007206
3497.000000	0.007233
3496.750000	0.007258
3496.500000	0.007278
3496.250000	0.007293
3496.000000	0.007301
3495.750000	0.007304
3495.500000	0.007302
3495.250000	0.007296
3495.000000	0.007287
3494.750000	0.007273
3494.500000	0.007254
3494.250000	0.007232
3494.000000	0.007208
3493.750000	0.007187
3493.500000	0.007173
3493.250000	0.007170
3493.000000	0.007178

3492.750000	0.007198
3492.500000	0.007225
3492.250000	0.007253
3492.000000	0.007275
3491.750000	0.007287
3491.500000	0.007290
3491.250000	0.007285
3491.000000	0.007278
3490.750000	0.007275
3490.500000	0.007279
3490.250000	0.007291
3490.000000	0.007311
3489.750000	0.007333
3489.500000	0.007352
3489.250000	0.007366
3489.000000	0.007371
3488.750000	0.007369
3488.500000	0.007362
3488.250000	0.007354
3488.000000	0.007346
3487.750000	0.007342
3487.500000	0.007342
3487.250000	0.007346
3487.000000	0.007353
3486.750000	0.007364
3486.500000	0.007377

3486.250000	0.007393
3486.000000	0.007412
3485.750000	0.007431
3485.500000	0.007449
3485.250000	0.007463
3485.000000	0.007472
3484.750000	0.007474
3484.500000	0.007470
3484.250000	0.007460
3484.000000	0.007449
3483.750000	0.007437
3483.500000	0.007427
3483.250000	0.007422
3483.000000	0.007422
3482.750000	0.007427
3482.500000	0.007437
3482.250000	0.007452
3482.000000	0.007468
3481.750000	0.007485
3481.500000	0.007501
3481.250000	0.007515
3481.000000	0.007525
3480.750000	0.007532
3480.500000	0.007536
3480.250000	0.007539
3480.000000	0.007543

3479.750000	0.007548
3479.500000	0.007557
3479.250000	0.007568
3479.000000	0.007581
3478.750000	0.007595
3478.500000	0.007608
3478.250000	0.007617
3478.000000	0.007622
3477.750000	0.007622
3477.500000	0.007618
3477.250000	0.007613
3477.000000	0.007610
3476.750000	0.007611
3476.500000	0.007618
3476.250000	0.007633
3476.000000	0.007652
3475.750000	0.007675
3475.500000	0.007696
3475.250000	0.007714
3475.000000	0.007726
3474.750000	0.007734
3474.500000	0.007739
3474.250000	0.007744
3474.000000	0.007751
3473.750000	0.007763
3473.500000	0.007779

3473.250000	0.007798
3473.000000	0.007817
3472.750000	0.007834
3472.500000	0.007847
3472.250000	0.007854
3472.000000	0.007858
3471.750000	0.007860
3471.500000	0.007860
3471.250000	0.007861
3471.000000	0.007862
3470.750000	0.007862
3470.500000	0.007861
3470.250000	0.007857
3470.000000	0.007851
3469.750000	0.007844
3469.500000	0.007839
3469.250000	0.007839
3469.000000	0.007845
3468.750000	0.007859
3468.500000	0.007880
3468.250000	0.007905
3468.000000	0.007933
3467.750000	0.007961
3467.500000	0.007988
3467.250000	0.008014
3467.000000	0.008039

3466.750000	0.008065
3466.500000	0.008093
3466.250000	0.008121
3466.000000	0.008150
3465.750000	0.008175
3465.500000	0.008194
3465.250000	0.008206
3465.000000	0.008209
3464.750000	0.008206
3464.500000	0.008200
3464.250000	0.008193
3464.000000	0.008189
3463.750000	0.008191
3463.500000	0.008198
3463.250000	0.008212
3463.000000	0.008229
3462.750000	0.008247
3462.500000	0.008265
3462.250000	0.008281
3462.000000	0.008295
3461.750000	0.008306
3461.500000	0.008316
3461.250000	0.008325
3461.000000	0.008336
3460.750000	0.008349
3460.500000	0.008364

3460.250000	0.008380
3460.000000	0.008398
3459.750000	0.008415
3459.500000	0.008432
3459.250000	0.008447
3459.000000	0.008461
3458.750000	0.008475
3458.500000	0.008488
3458.250000	0.008501
3458.000000	0.008514
3457.750000	0.008526
3457.500000	0.008536
3457.250000	0.008544
3457.000000	0.008551
3456.750000	0.008558
3456.500000	0.008568
3456.250000	0.008582
3456.000000	0.008602
3455.750000	0.008628
3455.500000	0.008658
3455.250000	0.008688
3455.000000	0.008715
3454.750000	0.008736
3454.500000	0.008749
3454.250000	0.008753
3454.000000	0.008751

3453.750000	0.008744
3453.500000	0.008735
3453.250000	0.008728
3453.000000	0.008726
3452.750000	0.008731
3452.500000	0.008742
3452.250000	0.008759
3452.000000	0.008780
3451.750000	0.008804
3451.500000	0.008826
3451.250000	0.008845
3451.000000	0.008859
3450.750000	0.008867
3450.500000	0.008869
3450.250000	0.008864
3450.000000	0.008855
3449.750000	0.008841
3449.500000	0.008827
3449.250000	0.008814
3449.000000	0.008806
3448.750000	0.008808
3448.500000	0.008822
3448.250000	0.008852
3448.000000	0.008897
3447.750000	0.008953
3447.500000	0.009015

3447.250000	0.009075
3447.000000	0.009127
3446.750000	0.009169
3446.500000	0.009201
3446.250000	0.009225
3446.000000	0.009246
3445.750000	0.009267
3445.500000	0.009288
3445.250000	0.009308
3445.000000	0.009325
3444.750000	0.009337
3444.500000	0.009341
3444.250000	0.009339
3444.000000	0.009334
3443.750000	0.009328
3443.500000	0.009326
3443.250000	0.009329
3443.000000	0.009338
3442.750000	0.009351
3442.500000	0.009366
3442.250000	0.009381
3442.000000	0.009394
3441.750000	0.009404
3441.500000	0.009411
3441.250000	0.009416
3441.000000	0.009417

3440.750000	0.009415
3440.500000	0.009411
3440.250000	0.009406
3440.000000	0.009402
3439.750000	0.009403
3439.500000	0.009412
3439.250000	0.009429
3439.000000	0.009454
3438.750000	0.009487
3438.500000	0.009524
3438.250000	0.009560
3438.000000	0.009593
3437.750000	0.009618
3437.500000	0.009636
3437.250000	0.009646
3437.000000	0.009651
3436.750000	0.009652
3436.500000	0.009653
3436.250000	0.009654
3436.000000	0.009659
3435.750000	0.009667
3435.500000	0.009678
3435.250000	0.009694
3435.000000	0.009714
3434.750000	0.009739
3434.500000	0.009768

3434.250000	0.009800
3434.000000	0.009835
3433.750000	0.009870
3433.500000	0.009902
3433.250000	0.009932
3433.000000	0.009956
3432.750000	0.009976
3432.500000	0.009993
3432.250000	0.010010
3432.000000	0.010028
3431.750000	0.010048
3431.500000	0.010070
3431.250000	0.010093
3431.000000	0.010114
3430.750000	0.010132
3430.500000	0.010144
3430.250000	0.010151
3430.000000	0.010155
3429.750000	0.010160
3429.500000	0.010167
3429.250000	0.010180
3429.000000	0.010200
3428.750000	0.010227
3428.500000	0.010261
3428.250000	0.010300
3428.000000	0.010342

3427.750000	0.010383
3427.500000	0.010422
3427.250000	0.010455
3427.000000	0.010481
3426.750000	0.010501
3426.500000	0.010516
3426.250000	0.010527
3426.000000	0.010540
3425.750000	0.010555
3425.500000	0.010574
3425.250000	0.010596
3425.000000	0.010620
3424.750000	0.010641
3424.500000	0.010657
3424.250000	0.010665
3424.000000	0.010667
3423.750000	0.010663
3423.500000	0.010658
3423.250000	0.010655
3423.000000	0.010657
3422.750000	0.010666
3422.500000	0.010682
3422.250000	0.010706
3422.000000	0.010735
3421.750000	0.010769
3421.500000	0.010809

3421.250000	0.010855
3421.000000	0.010906
3420.750000	0.010962
3420.500000	0.011019
3420.250000	0.011076
3420.000000	0.011128
3419.750000	0.011172
3419.500000	0.011207
3419.250000	0.011233
3419.000000	0.011251
3418.750000	0.011264
3418.500000	0.011274
3418.250000	0.011284
3418.000000	0.011295
3417.750000	0.011309
3417.500000	0.011326
3417.250000	0.011344
3417.000000	0.011364
3416.750000	0.011384
3416.500000	0.011405
3416.250000	0.011427
3416.000000	0.011450
3415.750000	0.011475
3415.500000	0.011500
3415.250000	0.011524
3415.000000	0.011548

3414.750000	0.011569
3414.500000	0.011589
3414.250000	0.011607
3414.000000	0.011625
3413.750000	0.011645
3413.500000	0.011670
3413.250000	0.011699
3413.000000	0.011734
3412.750000	0.011773
3412.500000	0.011815
3412.250000	0.011858
3412.000000	0.011898
3411.750000	0.011936
3411.500000	0.011969
3411.250000	0.011999
3411.000000	0.012026
3410.750000	0.012050
3410.500000	0.012073
3410.250000	0.012096
3410.000000	0.012120
3409.750000	0.012145
3409.500000	0.012173
3409.250000	0.012204
3409.000000	0.012237
3408.750000	0.012274
3408.500000	0.012314

3408.250000	0.012357
3408.000000	0.012404
3407.750000	0.012453
3407.500000	0.012502
3407.250000	0.012549
3407.000000	0.012591
3406.750000	0.012627
3406.500000	0.012655
3406.250000	0.012676
3406.000000	0.012692
3405.750000	0.012706
3405.500000	0.012722
3405.250000	0.012742
3405.000000	0.012767
3404.750000	0.012795
3404.500000	0.012825
3404.250000	0.012854
3404.000000	0.012880
3403.750000	0.012903
3403.500000	0.012923
3403.250000	0.012943
3403.000000	0.012965
3402.750000	0.012992
3402.500000	0.013026
3402.250000	0.013067
3402.000000	0.013113

3401.750000	0.013163
3401.500000	0.013214
3401.250000	0.013264
3401.000000	0.013312
3400.750000	0.013356
3400.500000	0.013398
3400.250000	0.013438
3400.000000	0.013476
3399.750000	0.013514
3399.500000	0.013552
3399.250000	0.013591
3399.000000	0.013630
3398.750000	0.013672
3398.500000	0.013714
3398.250000	0.013759
3398.000000	0.013805
3397.750000	0.013851
3397.500000	0.013894
3397.250000	0.013933
3397.000000	0.013964
3396.750000	0.013987
3396.500000	0.014002
3396.250000	0.014010
3396.000000	0.014015
3395.750000	0.014018
3395.500000	0.014023

3395.250000	0.014031
3395.000000	0.014043
3394.750000	0.014057
3394.500000	0.014075
3394.250000	0.014096
3394.000000	0.014119
3393.750000	0.014144
3393.500000	0.014171
3393.250000	0.014198
3393.000000	0.014222
3392.750000	0.014242
3392.500000	0.014257
3392.250000	0.014268
3392.000000	0.014278
3391.750000	0.014288
3391.500000	0.014303
3391.250000	0.014325
3391.000000	0.014351
3390.750000	0.014380
3390.500000	0.014408
3390.250000	0.014432
3390.000000	0.014449
3389.750000	0.014460
3389.500000	0.014465
3389.250000	0.014467
3389.000000	0.014469

3388.750000	0.014472
3388.500000	0.014477
3388.250000	0.014483
3388.000000	0.014490
3387.750000	0.014499
3387.500000	0.014508
3387.250000	0.014518
3387.000000	0.014529
3386.750000	0.014540
3386.500000	0.014550
3386.250000	0.014558
3386.000000	0.014563
3385.750000	0.014565
3385.500000	0.014566
3385.250000	0.014568
3385.000000	0.014573
3384.750000	0.014582
3384.500000	0.014597
3384.250000	0.014615
3384.000000	0.014633
3383.750000	0.014648
3383.500000	0.014656
3383.250000	0.014655
3383.000000	0.014643
3382.750000	0.014622
3382.500000	0.014595

3382.250000	0.014567
3382.000000	0.014543
3381.750000	0.014527
3381.500000	0.014522
3381.250000	0.014526
3381.000000	0.014537
3380.750000	0.014550
3380.500000	0.014561
3380.250000	0.014566
3380.000000	0.014563
3379.750000	0.014553
3379.500000	0.014541
3379.250000	0.014530
3379.000000	0.014522
3378.750000	0.014520
3378.500000	0.014523
3378.250000	0.014528
3378.000000	0.014532
3377.750000	0.014530
3377.500000	0.014523
3377.250000	0.014511
3377.000000	0.014497
3376.750000	0.014485
3376.500000	0.014477
3376.250000	0.014476
3376.000000	0.014484

3375.750000	0.014499
3375.500000	0.014519
3375.250000	0.014541
3375.000000	0.014563
3374.750000	0.014583
3374.500000	0.014599
3374.250000	0.014609
3374.000000	0.014612
3373.750000	0.014608
3373.500000	0.014597
3373.250000	0.014579
3373.000000	0.014556
3372.750000	0.014530
3372.500000	0.014502
3372.250000	0.014473
3372.000000	0.014446
3371.750000	0.014421
3371.500000	0.014399
3371.250000	0.014380
3371.000000	0.014366
3370.750000	0.014356
3370.500000	0.014349
3370.250000	0.014347
3370.000000	0.014345
3369.750000	0.014345
3369.500000	0.014342

3369.250000	0.014338
3369.000000	0.014331
3368.750000	0.014322
3368.500000	0.014313
3368.250000	0.014305
3368.000000	0.014301
3367.750000	0.014302
3367.500000	0.014307
3367.250000	0.014315
3367.000000	0.014325
3366.750000	0.014332
3366.500000	0.014337
3366.250000	0.014336
3366.000000	0.014331
3365.750000	0.014321
3365.500000	0.014309
3365.250000	0.014295
3365.000000	0.014281
3364.750000	0.014267
3364.500000	0.014252
3364.250000	0.014237
3364.000000	0.014221
3363.750000	0.014205
3363.500000	0.014189
3363.250000	0.014174
3363.000000	0.014160

3362.750000	0.014148
3362.500000	0.014137
3362.250000	0.014129
3362.000000	0.014123
3361.750000	0.014117
3361.500000	0.014112
3361.250000	0.014106
3361.000000	0.014096
3360.750000	0.014083
3360.500000	0.014065
3360.250000	0.014043
3360.000000	0.014019
3359.750000	0.013994
3359.500000	0.013972
3359.250000	0.013956
3359.000000	0.013946
3358.750000	0.013943
3358.500000	0.013946
3358.250000	0.013952
3358.000000	0.013960
3357.750000	0.013966
3357.500000	0.013971
3357.250000	0.013972
3357.000000	0.013970
3356.750000	0.013964
3356.500000	0.013953

3356.250000	0.013937
3356.000000	0.013916
3355.750000	0.013890
3355.500000	0.013862
3355.250000	0.013834
3355.000000	0.013807
3354.750000	0.013784
3354.500000	0.013765
3354.250000	0.013749
3354.000000	0.013734
3353.750000	0.013719
3353.500000	0.013703
3353.250000	0.013686
3353.000000	0.013668
3352.750000	0.013650
3352.500000	0.013634
3352.250000	0.013622
3352.000000	0.013613
3351.750000	0.013607
3351.500000	0.013603
3351.250000	0.013600
3351.000000	0.013598
3350.750000	0.013596
3350.500000	0.013592
3350.250000	0.013588
3350.000000	0.013583

3349.750000	0.013578
3349.500000	0.013572
3349.250000	0.013566
3349.000000	0.013558
3348.750000	0.013550
3348.500000	0.013540
3348.250000	0.013530
3348.000000	0.013520
3347.750000	0.013512
3347.500000	0.013505
3347.250000	0.013500
3347.000000	0.013496
3346.750000	0.013491
3346.500000	0.013484
3346.250000	0.013473
3346.000000	0.013456
3345.750000	0.013435
3345.500000	0.013411
3345.250000	0.013386
3345.000000	0.013363
3344.750000	0.013343
3344.500000	0.013329
3344.250000	0.013319
3344.000000	0.013313
3343.750000	0.013309
3343.500000	0.013307

3343.250000	0.013305
3343.000000	0.013301
3342.750000	0.013297
3342.500000	0.013290
3342.250000	0.013280
3342.000000	0.013267
3341.750000	0.013251
3341.500000	0.013233
3341.250000	0.013214
3341.000000	0.013196
3340.750000	0.013182
3340.500000	0.013174
3340.250000	0.013171
3340.000000	0.013172
3339.750000	0.013175
3339.500000	0.013175
3339.250000	0.013171
3339.000000	0.013163
3338.750000	0.013150
3338.500000	0.013136
3338.250000	0.013122
3338.000000	0.013111
3337.750000	0.013104
3337.500000	0.013098
3337.250000	0.013091
3337.000000	0.013079

3336.750000	0.013060
3336.500000	0.013035
3336.250000	0.013004
3336.000000	0.012974
3335.750000	0.012946
3335.500000	0.012926
3335.250000	0.012914
3335.000000	0.012909
3334.750000	0.012906
3334.500000	0.012901
3334.250000	0.012891
3334.000000	0.012873
3333.750000	0.012848
3333.500000	0.012819
3333.250000	0.012788
3333.000000	0.012760
3332.750000	0.012735
3332.500000	0.012713
3332.250000	0.012695
3332.000000	0.012676
3331.750000	0.012656
3331.500000	0.012634
3331.250000	0.012610
3331.000000	0.012584
3330.750000	0.012560
3330.500000	0.012538

3330.250000	0.012520
3330.000000	0.012504
3329.750000	0.012492
3329.500000	0.012480
3329.250000	0.012469
3329.000000	0.012456
3328.750000	0.012443
3328.500000	0.012429
3328.250000	0.012415
3328.000000	0.012401
3327.750000	0.012389
3327.500000	0.012378
3327.250000	0.012368
3327.000000	0.012357
3326.750000	0.012344
3326.500000	0.012329
3326.250000	0.012310
3326.000000	0.012289
3325.750000	0.012266
3325.500000	0.012245
3325.250000	0.012226
3325.000000	0.012211
3324.750000	0.012200
3324.500000	0.012192
3324.250000	0.012186
3324.000000	0.012181

3323.750000	0.012174
3323.500000	0.012163
3323.250000	0.012148
3323.000000	0.012128
3322.750000	0.012101
3322.500000	0.012069
3322.250000	0.012031
3322.000000	0.011991
3321.750000	0.011950
3321.500000	0.011912
3321.250000	0.011881
3321.000000	0.011857
3320.750000	0.011840
3320.500000	0.011831
3320.250000	0.011825
3320.000000	0.011819
3319.750000	0.011810
3319.500000	0.011797
3319.250000	0.011779
3319.000000	0.011756
3318.750000	0.011729
3318.500000	0.011702
3318.250000	0.011676
3318.000000	0.011653
3317.750000	0.011632
3317.500000	0.011615

3317.250000	0.011599
3317.000000	0.011585
3316.750000	0.011569
3316.500000	0.011551
3316.250000	0.011530
3316.000000	0.011504
3315.750000	0.011475
3315.500000	0.011443
3315.250000	0.011412
3315.000000	0.011383
3314.750000	0.011357
3314.500000	0.011337
3314.250000	0.011324
3314.000000	0.011316
3313.750000	0.011313
3313.500000	0.011314
3313.250000	0.011316
3313.000000	0.011315
3312.750000	0.011309
3312.500000	0.011295
3312.250000	0.011269
3312.000000	0.011234
3311.750000	0.011189
3311.500000	0.011139
3311.250000	0.011087
3311.000000	0.011038

3310.750000	0.010995
3310.500000	0.010960
3310.250000	0.010931
3310.000000	0.010908
3309.750000	0.010888
3309.500000	0.010871
3309.250000	0.010856
3309.000000	0.010842
3308.750000	0.010831
3308.500000	0.010821
3308.250000	0.010812
3308.000000	0.010801
3307.750000	0.010787
3307.500000	0.010768
3307.250000	0.010744
3307.000000	0.010715
3306.750000	0.010682
3306.500000	0.010648
3306.250000	0.010616
3306.000000	0.010588
3305.750000	0.010565
3305.500000	0.010548
3305.250000	0.010534
3305.000000	0.010522
3304.750000	0.010509
3304.500000	0.010492

3304.250000	0.010470
3304.000000	0.010442
3303.750000	0.010410
3303.500000	0.010376
3303.250000	0.010344
3303.000000	0.010317
3302.750000	0.010296
3302.500000	0.010281
3302.250000	0.010270
3302.000000	0.010261
3301.750000	0.010251
3301.500000	0.010237
3301.250000	0.010218
3301.000000	0.010196
3300.750000	0.010171
3300.500000	0.010145
3300.250000	0.010119
3300.000000	0.010093
3299.750000	0.010069
3299.500000	0.010045
3299.250000	0.010024
3299.000000	0.010004
3298.750000	0.009986
3298.500000	0.009971
3298.250000	0.009957
3298.000000	0.009944

3297.750000	0.009929
3297.500000	0.009910
3297.250000	0.009887
3297.000000	0.009860
3296.750000	0.009831
3296.500000	0.009801
3296.250000	0.009773
3296.000000	0.009748
3295.750000	0.009729
3295.500000	0.009714
3295.250000	0.009702
3295.000000	0.009691
3294.750000	0.009678
3294.500000	0.009663
3294.250000	0.009642
3294.000000	0.009617
3293.750000	0.009587
3293.500000	0.009552
3293.250000	0.009515
3293.000000	0.009476
3292.750000	0.009438
3292.500000	0.009402
3292.250000	0.009372
3292.000000	0.009348
3291.750000	0.009331
3291.500000	0.009322

3291.250000	0.009319
3291.000000	0.009319
3290.750000	0.009321
3290.500000	0.009321
3290.250000	0.009315
3290.000000	0.009303
3289.750000	0.009285
3289.500000	0.009261
3289.250000	0.009234
3289.000000	0.009205
3288.750000	0.009176
3288.500000	0.009150
3288.250000	0.009127
3288.000000	0.009107
3287.750000	0.009089
3287.500000	0.009075
3287.250000	0.009063
3287.000000	0.009053
3286.750000	0.009045
3286.500000	0.009038
3286.250000	0.009032
3286.000000	0.009027
3285.750000	0.009020
3285.500000	0.009013
3285.250000	0.009002
3285.000000	0.008988

3284.750000	0.008969
3284.500000	0.008944
3284.250000	0.008914
3284.000000	0.008880
3283.750000	0.008844
3283.500000	0.008809
3283.250000	0.008778
3283.000000	0.008751
3282.750000	0.008730
3282.500000	0.008711
3282.250000	0.008694
3282.000000	0.008676
3281.750000	0.008656
3281.500000	0.008634
3281.250000	0.008612
3281.000000	0.008593
3280.750000	0.008577
3280.500000	0.008567
3280.250000	0.008561
3280.000000	0.008559
3279.750000	0.008557
3279.500000	0.008556
3279.250000	0.008552
3279.000000	0.008547
3278.750000	0.008539
3278.500000	0.008529

3278.250000	0.008516
3278.000000	0.008501
3277.750000	0.008481
3277.500000	0.008457
3277.250000	0.008428
3277.000000	0.008395
3276.750000	0.008359
3276.500000	0.008320
3276.250000	0.008281
3276.000000	0.008243
3275.750000	0.008208
3275.500000	0.008180
3275.250000	0.008160
3275.000000	0.008151
3274.750000	0.008152
3274.500000	0.008162
3274.250000	0.008176
3274.000000	0.008190
3273.750000	0.008199
3273.500000	0.008200
3273.250000	0.008191
3273.000000	0.008172
3272.750000	0.008148
3272.500000	0.008121
3272.250000	0.008093
3272.000000	0.008067

3271.750000	0.008043
3271.500000	0.008020
3271.250000	0.008000
3271.000000	0.007981
3270.750000	0.007966
3270.500000	0.007956
3270.250000	0.007949
3270.000000	0.007946
3269.750000	0.007946
3269.500000	0.007945
3269.250000	0.007942
3269.000000	0.007936
3268.750000	0.007925
3268.500000	0.007910
3268.250000	0.007892
3268.000000	0.007872
3267.750000	0.007852
3267.500000	0.007831
3267.250000	0.007813
3267.000000	0.007797
3266.750000	0.007785
3266.500000	0.007776
3266.250000	0.007769
3266.000000	0.007761
3265.750000	0.007751
3265.500000	0.007736

3265.250000	0.007716
3265.000000	0.007693
3264.750000	0.007670
3264.500000	0.007650
3264.250000	0.007637
3264.000000	0.007634
3263.750000	0.007638
3263.500000	0.007646
3263.250000	0.007653
3263.000000	0.007654
3262.750000	0.007645
3262.500000	0.007628
3262.250000	0.007603
3262.000000	0.007577
3261.750000	0.007553
3261.500000	0.007537
3261.250000	0.007529
3261.000000	0.007530
3260.750000	0.007536
3260.500000	0.007542
3260.250000	0.007544
3260.000000	0.007538
3259.750000	0.007522
3259.500000	0.007496
3259.250000	0.007464
3259.000000	0.007428

3258.750000	0.007393
3258.500000	0.007362
3258.250000	0.007337
3258.000000	0.007319
3257.750000	0.007306
3257.500000	0.007298
3257.250000	0.007290
3257.000000	0.007281
3256.750000	0.007270
3256.500000	0.007257
3256.250000	0.007244
3256.000000	0.007232
3255.750000	0.007224
3255.500000	0.007222
3255.250000	0.007226
3255.000000	0.007235
3254.750000	0.007244
3254.500000	0.007250
3254.250000	0.007249
3254.000000	0.007240
3253.750000	0.007223
3253.500000	0.007200
3253.250000	0.007176
3253.000000	0.007153
3252.750000	0.007136
3252.500000	0.007126

3252.250000	0.007122
3252.000000	0.007123
3251.750000	0.007126
3251.500000	0.007128
3251.250000	0.007128
3251.000000	0.007125
3250.750000	0.007119
3250.500000	0.007112
3250.250000	0.007103
3250.000000	0.007093
3249.750000	0.007083
3249.500000	0.007071
3249.250000	0.007060
3249.000000	0.007048
3248.750000	0.007036
3248.500000	0.007025
3248.250000	0.007013
3248.000000	0.007000
3247.750000	0.006985
3247.500000	0.006966
3247.250000	0.006945
3247.000000	0.006922
3246.750000	0.006898
3246.500000	0.006875
3246.250000	0.006856
3246.000000	0.006842

3245.750000	0.006833
3245.500000	0.006831
3245.250000	0.006835
3245.000000	0.006843
3244.750000	0.006853
3244.500000	0.006863
3244.250000	0.006869
3244.000000	0.006869
3243.750000	0.006862
3243.500000	0.006845
3243.250000	0.006821
3243.000000	0.006792
3242.750000	0.006761
3242.500000	0.006733
3242.250000	0.006711
3242.000000	0.006696
3241.750000	0.006689
3241.500000	0.006689
3241.250000	0.006693
3241.000000	0.006700
3240.750000	0.006708
3240.500000	0.006714
3240.250000	0.006719
3240.000000	0.006721
3239.750000	0.006720
3239.500000	0.006716

3239.250000	0.006709
3239.000000	0.006699
3238.750000	0.006686
3238.500000	0.006672
3238.250000	0.006658
3238.000000	0.006645
3237.750000	0.006633
3237.500000	0.006623
3237.250000	0.006615
3237.000000	0.006609
3236.750000	0.006604
3236.500000	0.006599
3236.250000	0.006593
3236.000000	0.006586
3235.750000	0.006576
3235.500000	0.006563
3235.250000	0.006549
3235.000000	0.006535
3234.750000	0.006524
3234.500000	0.006518
3234.250000	0.006517
3234.000000	0.006522
3233.750000	0.006531
3233.500000	0.006541
3233.250000	0.006551
3233.000000	0.006557

3232.750000	0.006559
3232.500000	0.006555
3232.250000	0.006546
3232.000000	0.006533
3231.750000	0.006518
3231.500000	0.006501
3231.250000	0.006485
3231.000000	0.006470
3230.750000	0.006458
3230.500000	0.006449
3230.250000	0.006443
3230.000000	0.006440
3229.750000	0.006438
3229.500000	0.006437
3229.250000	0.006436
3229.000000	0.006435
3228.750000	0.006435
3228.500000	0.006436
3228.250000	0.006438
3228.000000	0.006441
3227.750000	0.006445
3227.500000	0.006447
3227.250000	0.006444
3227.000000	0.006436
3226.750000	0.006422
3226.500000	0.006404

3226.250000	0.006383
3226.000000	0.006363
3225.750000	0.006348
3225.500000	0.006340
3225.250000	0.006340
3225.000000	0.006346
3224.750000	0.006357
3224.500000	0.006369
3224.250000	0.006378
3224.000000	0.006383
3223.750000	0.006382
3223.500000	0.006373
3223.250000	0.006358
3223.000000	0.006338
3222.750000	0.006315
3222.500000	0.006291
3222.250000	0.006268
3222.000000	0.006247
3221.750000	0.006228
3221.500000	0.006213
3221.250000	0.006201
3221.000000	0.006193
3220.750000	0.006189
3220.500000	0.006190
3220.250000	0.006195
3220.000000	0.006204

3219.750000	0.006214
3219.500000	0.006224
3219.250000	0.006232
3219.000000	0.006236
3218.750000	0.006237
3218.500000	0.006235
3218.250000	0.006229
3218.000000	0.006222
3217.750000	0.006213
3217.500000	0.006203
3217.250000	0.006191
3217.000000	0.006178
3216.750000	0.006163
3216.500000	0.006148
3216.250000	0.006135
3216.000000	0.006128
3215.750000	0.006127
3215.500000	0.006134
3215.250000	0.006148
3215.000000	0.006166
3214.750000	0.006186
3214.500000	0.006203
3214.250000	0.006214
3214.000000	0.006218
3213.750000	0.006214
3213.500000	0.006204

3213.250000	0.006189
3213.000000	0.006172
3212.750000	0.006152
3212.500000	0.006133
3212.250000	0.006113
3212.000000	0.006095
3211.750000	0.006079
3211.500000	0.006069
3211.250000	0.006065
3211.000000	0.006068
3210.750000	0.006076
3210.500000	0.006088
3210.250000	0.006101
3210.000000	0.006110
3209.750000	0.006115
3209.500000	0.006113
3209.250000	0.006107
3209.000000	0.006096
3208.750000	0.006084
3208.500000	0.006072
3208.250000	0.006062
3208.000000	0.006053
3207.750000	0.006046
3207.500000	0.006040
3207.250000	0.006036
3207.000000	0.006032

3206.750000	0.006029
3206.500000	0.006026
3206.250000	0.006023
3206.000000	0.006018
3205.750000	0.006012
3205.500000	0.006004
3205.250000	0.005996
3205.000000	0.005990
3204.750000	0.005988
3204.500000	0.005989
3204.250000	0.005996
3204.000000	0.006005
3203.750000	0.006015
3203.500000	0.006024
3203.250000	0.006030
3203.000000	0.006031
3202.750000	0.006029
3202.500000	0.006023
3202.250000	0.006016
3202.000000	0.006009
3201.750000	0.006002
3201.500000	0.005997
3201.250000	0.005995
3201.000000	0.005994
3200.750000	0.005995
3200.500000	0.005995

3200.250000	0.005993
3200.000000	0.005988
3199.750000	0.005978
3199.500000	0.005964
3199.250000	0.005946
3199.000000	0.005930
3198.750000	0.005916
3198.500000	0.005908
3198.250000	0.005905
3198.000000	0.005908
3197.750000	0.005912
3197.500000	0.005914
3197.250000	0.005912
3197.000000	0.005905
3196.750000	0.005893
3196.500000	0.005880
3196.250000	0.005868
3196.000000	0.005859
3195.750000	0.005854
3195.500000	0.005853
3195.250000	0.005855
3195.000000	0.005857
3194.750000	0.005859
3194.500000	0.005860
3194.250000	0.005861
3194.000000	0.005862

3193.750000	0.005862
3193.500000	0.005864
3193.250000	0.005865
3193.000000	0.005868
3192.750000	0.005871
3192.500000	0.005875
3192.250000	0.005879
3192.000000	0.005883
3191.750000	0.005884
3191.500000	0.005882
3191.250000	0.005877
3191.000000	0.005868
3190.750000	0.005858
3190.500000	0.005849
3190.250000	0.005842
3190.000000	0.005839
3189.750000	0.005838
3189.500000	0.005838
3189.250000	0.005837
3189.000000	0.005831
3188.750000	0.005822
3188.500000	0.005808
3188.250000	0.005793
3188.000000	0.005779
3187.750000	0.005767
3187.500000	0.005759

3187.250000	0.005753
3187.000000	0.005750
3186.750000	0.005749
3186.500000	0.005749
3186.250000	0.005751
3186.000000	0.005756
3185.750000	0.005762
3185.500000	0.005771
3185.250000	0.005779
3185.000000	0.005785
3184.750000	0.005787
3184.500000	0.005784
3184.250000	0.005776
3184.000000	0.005764
3183.750000	0.005751
3183.500000	0.005737
3183.250000	0.005725
3183.000000	0.005715
3182.750000	0.005706
3182.500000	0.005698
3182.250000	0.005690
3182.000000	0.005682
3181.750000	0.005673
3181.500000	0.005664
3181.250000	0.005656
3181.000000	0.005648

3180.750000	0.005643
3180.500000	0.005639
3180.250000	0.005637
3180.000000	0.005638
3179.750000	0.005642
3179.500000	0.005648
3179.250000	0.005656
3179.000000	0.005665
3178.750000	0.005673
3178.500000	0.005679
3178.250000	0.005682
3178.000000	0.005681
3177.750000	0.005674
3177.500000	0.005662
3177.250000	0.005646
3177.000000	0.005626
3176.750000	0.005605
3176.500000	0.005584
3176.250000	0.005566
3176.000000	0.005550
3175.750000	0.005540
3175.500000	0.005534
3175.250000	0.005531
3175.000000	0.005532
3174.750000	0.005535
3174.500000	0.005537

3174.250000	0.005537
3174.000000	0.005534
3173.750000	0.005526
3173.500000	0.005513
3173.250000	0.005496
3173.000000	0.005474
3172.750000	0.005451
3172.500000	0.005427
3172.250000	0.005407
3172.000000	0.005390
3171.750000	0.005380
3171.500000	0.005374
3171.250000	0.005374
3171.000000	0.005376
3170.750000	0.005379
3170.500000	0.005383
3170.250000	0.005386
3170.000000	0.005388
3169.750000	0.005388
3169.500000	0.005388
3169.250000	0.005385
3169.000000	0.005380
3168.750000	0.005371
3168.500000	0.005359
3168.250000	0.005343
3168.000000	0.005324

3167.750000	0.005304
3167.500000	0.005284
3167.250000	0.005266
3167.000000	0.005250
3166.750000	0.005238
3166.500000	0.005227
3166.250000	0.005218
3166.000000	0.005210
3165.750000	0.005202
3165.500000	0.005194
3165.250000	0.005188
3165.000000	0.005183
3164.750000	0.005182
3164.500000	0.005183
3164.250000	0.005186
3164.000000	0.005188
3163.750000	0.005186
3163.500000	0.005179
3163.250000	0.005165
3163.000000	0.005145
3162.750000	0.005122
3162.500000	0.005100
3162.250000	0.005082
3162.000000	0.005072
3161.750000	0.005070
3161.500000	0.005076

3161.250000	0.005085
3161.000000	0.005095
3160.750000	0.005104
3160.500000	0.005107
3160.250000	0.005106
3160.000000	0.005101
3159.750000	0.005092
3159.500000	0.005082
3159.250000	0.005070
3159.000000	0.005059
3158.750000	0.005048
3158.500000	0.005037
3158.250000	0.005028
3158.000000	0.005021
3157.750000	0.005015
3157.500000	0.005009
3157.250000	0.005004
3157.000000	0.004998
3156.750000	0.004990
3156.500000	0.004981
3156.250000	0.004972
3156.000000	0.004964
3155.750000	0.004958
3155.500000	0.004954
3155.250000	0.004954
3155.000000	0.004956

3154.750000	0.004960
3154.500000	0.004965
3154.250000	0.004970
3154.000000	0.004975
3153.750000	0.004981
3153.500000	0.004985
3153.250000	0.004990
3153.000000	0.004992
3152.750000	0.004993
3152.500000	0.004991
3152.250000	0.004985
3152.000000	0.004975
3151.750000	0.004961
3151.500000	0.004943
3151.250000	0.004922
3151.000000	0.004901
3150.750000	0.004881
3150.500000	0.004864
3150.250000	0.004851
3150.000000	0.004843
3149.750000	0.004842
3149.500000	0.004846
3149.250000	0.004853
3149.000000	0.004862
3148.750000	0.004871
3148.500000	0.004877

3148.250000	0.004880
3148.000000	0.004877
3147.750000	0.004870
3147.500000	0.004859
3147.250000	0.004846
3147.000000	0.004832
3146.750000	0.004821
3146.500000	0.004813
3146.250000	0.004809
3146.000000	0.004811
3145.750000	0.004815
3145.500000	0.004819
3145.250000	0.004820
3145.000000	0.004817
3144.750000	0.004808
3144.500000	0.004795
3144.250000	0.004779
3144.000000	0.004762
3143.750000	0.004749
3143.500000	0.004740
3143.250000	0.004736
3143.000000	0.004736
3142.750000	0.004739
3142.500000	0.004744
3142.250000	0.004750
3142.000000	0.004757

3141.750000	0.004764
3141.500000	0.004769
3141.250000	0.004772
3141.000000	0.004772
3140.750000	0.004768
3140.500000	0.004760
3140.250000	0.004749
3140.000000	0.004737
3139.750000	0.004726
3139.500000	0.004718
3139.250000	0.004715
3139.000000	0.004714
3138.750000	0.004716
3138.500000	0.004719
3138.250000	0.004721
3138.000000	0.004721
3137.750000	0.004718
3137.500000	0.004712
3137.250000	0.004703
3137.000000	0.004692
3136.750000	0.004678
3136.500000	0.004664
3136.250000	0.004651
3136.000000	0.004641
3135.750000	0.004634
3135.500000	0.004632

3135.250000	0.004635
3135.000000	0.004642
3134.750000	0.004650
3134.500000	0.004658
3134.250000	0.004665
3134.000000	0.004669
3133.750000	0.004671
3133.500000	0.004670
3133.250000	0.004667
3133.000000	0.004663
3132.750000	0.004659
3132.500000	0.004654
3132.250000	0.004647
3132.000000	0.004640
3131.750000	0.004631
3131.500000	0.004620
3131.250000	0.004608
3131.000000	0.004596
3130.750000	0.004585
3130.500000	0.004578
3130.250000	0.004575
3130.000000	0.004577
3129.750000	0.004584
3129.500000	0.004593
3129.250000	0.004602
3129.000000	0.004607

3128.750000	0.004606
3128.500000	0.004598
3128.250000	0.004586
3128.000000	0.004571
3127.750000	0.004559
3127.500000	0.004554
3127.250000	0.004559
3127.000000	0.004575
3126.750000	0.004600
3126.500000	0.004629
3126.250000	0.004659
3126.000000	0.004684
3125.750000	0.004701
3125.500000	0.004708
3125.250000	0.004706
3125.000000	0.004696
3124.750000	0.004682
3124.500000	0.004666
3124.250000	0.004650
3124.000000	0.004634
3123.750000	0.004620
3123.500000	0.004606
3123.250000	0.004592
3123.000000	0.004577
3122.750000	0.004563
3122.500000	0.004548

3122.250000	0.004535
3122.000000	0.004524
3121.750000	0.004516
3121.500000	0.004510
3121.250000	0.004506
3121.000000	0.004504
3120.750000	0.004504
3120.500000	0.004506
3120.250000	0.004513
3120.000000	0.004525
3119.750000	0.004542
3119.500000	0.004561
3119.250000	0.004581
3119.000000	0.004597
3118.750000	0.004606
3118.500000	0.004606
3118.250000	0.004596
3118.000000	0.004581
3117.750000	0.004562
3117.500000	0.004544
3117.250000	0.004528
3117.000000	0.004516
3116.750000	0.004508
3116.500000	0.004502
3116.250000	0.004497
3116.000000	0.004493

3115.750000	0.004492
3115.500000	0.004497
3115.250000	0.004509
3115.000000	0.004530
3114.750000	0.004560
3114.500000	0.004594
3114.250000	0.004629
3114.000000	0.004659
3113.750000	0.004681
3113.500000	0.004692
3113.250000	0.004690
3113.000000	0.004678
3112.750000	0.004660
3112.500000	0.004638
3112.250000	0.004618
3112.000000	0.004602
3111.750000	0.004595
3111.500000	0.004595
3111.250000	0.004603
3111.000000	0.004617
3110.750000	0.004634
3110.500000	0.004650
3110.250000	0.004664
3110.000000	0.004673
3109.750000	0.004678
3109.500000	0.004678

3109.250000	0.004676
3109.000000	0.004674
3108.750000	0.004675
3108.500000	0.004678
3108.250000	0.004687
3108.000000	0.004698
3107.750000	0.004711
3107.500000	0.004724
3107.250000	0.004734
3107.000000	0.004740
3106.750000	0.004742
3106.500000	0.004739
3106.250000	0.004735
3106.000000	0.004732
3105.750000	0.004733
3105.500000	0.004738
3105.250000	0.004748
3105.000000	0.004761
3104.750000	0.004776
3104.500000	0.004788
3104.250000	0.004795
3104.000000	0.004796
3103.750000	0.004790
3103.500000	0.004779
3103.250000	0.004765
3103.000000	0.004752

3102.750000	0.004742
3102.500000	0.004736
3102.250000	0.004734
3102.000000	0.004735
3101.750000	0.004738
3101.500000	0.004740
3101.250000	0.004741
3101.000000	0.004741
3100.750000	0.004743
3100.500000	0.004747
3100.250000	0.004756
3100.000000	0.004771
3099.750000	0.004790
3099.500000	0.004810
3099.250000	0.004830
3099.000000	0.004845
3098.750000	0.004852
3098.500000	0.004850
3098.250000	0.004839
3098.000000	0.004823
3097.750000	0.004804
3097.500000	0.004786
3097.250000	0.004773
3097.000000	0.004766
3096.750000	0.004765
3096.500000	0.004769

3096.250000	0.004776
3096.000000	0.004784
3095.750000	0.004791
3095.500000	0.004796
3095.250000	0.004799
3095.000000	0.004799
3094.750000	0.004798
3094.500000	0.004795
3094.250000	0.004790
3094.000000	0.004784
3093.750000	0.004776
3093.500000	0.004768
3093.250000	0.004760
3093.000000	0.004754
3092.750000	0.004748
3092.500000	0.004744
3092.250000	0.004742
3092.000000	0.004740
3091.750000	0.004738
3091.500000	0.004735
3091.250000	0.004731
3091.000000	0.004726
3090.750000	0.004722
3090.500000	0.004718
3090.250000	0.004717
3090.000000	0.004720

3089.750000	0.004726
3089.500000	0.004736
3089.250000	0.004749
3089.000000	0.004763
3088.750000	0.004776
3088.500000	0.004788
3088.250000	0.004797
3088.000000	0.004803
3087.750000	0.004806
3087.500000	0.004806
3087.250000	0.004803
3087.000000	0.004799
3086.750000	0.004794
3086.500000	0.004788
3086.250000	0.004784
3086.000000	0.004781
3085.750000	0.004780
3085.500000	0.004783
3085.250000	0.004789
3085.000000	0.004798
3084.750000	0.004810
3084.500000	0.004823
3084.250000	0.004835
3084.000000	0.004845
3083.750000	0.004851
3083.500000	0.004852

3083.250000	0.004850
3083.000000	0.004846
3082.750000	0.004843
3082.500000	0.004843
3082.250000	0.004848
3082.000000	0.004856
3081.750000	0.004868
3081.500000	0.004882
3081.250000	0.004893
3081.000000	0.004902
3080.750000	0.004906
3080.500000	0.004907
3080.250000	0.004907
3080.000000	0.004908
3079.750000	0.004912
3079.500000	0.004920
3079.250000	0.004931
3079.000000	0.004944
3078.750000	0.004955
3078.500000	0.004962
3078.250000	0.004964
3078.000000	0.004961
3077.750000	0.004955
3077.500000	0.004949
3077.250000	0.004946
3077.000000	0.004949

3076.750000	0.004959
3076.500000	0.004975
3076.250000	0.004995
3076.000000	0.005017
3075.750000	0.005035
3075.500000	0.005047
3075.250000	0.005053
3075.000000	0.005051
3074.750000	0.005043
3074.500000	0.005034
3074.250000	0.005025
3074.000000	0.005021
3073.750000	0.005022
3073.500000	0.005028
3073.250000	0.005038
3073.000000	0.005050
3072.750000	0.005060
3072.500000	0.005067
3072.250000	0.005069
3072.000000	0.005067
3071.750000	0.005062
3071.500000	0.005056
3071.250000	0.005049
3071.000000	0.005044
3070.750000	0.005042
3070.500000	0.005042

3070.250000	0.005044
3070.000000	0.005048
3069.750000	0.005052
3069.500000	0.005056
3069.250000	0.005059
3069.000000	0.005062
3068.750000	0.005064
3068.500000	0.005066
3068.250000	0.005069
3068.000000	0.005073
3067.750000	0.005077
3067.500000	0.005080
3067.250000	0.005081
3067.000000	0.005079
3066.750000	0.005075
3066.500000	0.005070
3066.250000	0.005066
3066.000000	0.005065
3065.750000	0.005069
3065.500000	0.005078
3065.250000	0.005091
3065.000000	0.005103
3064.750000	0.005113
3064.500000	0.005119
3064.250000	0.005119
3064.000000	0.005116

3063.750000	0.005112
3063.500000	0.005111
3063.250000	0.005114
3063.000000	0.005123
3062.750000	0.005136
3062.500000	0.005151
3062.250000	0.005164
3062.000000	0.005173
3061.750000	0.005176
3061.500000	0.005174
3061.250000	0.005166
3061.000000	0.005156
3060.750000	0.005146
3060.500000	0.005137
3060.250000	0.005133
3060.000000	0.005134
3059.750000	0.005139
3059.500000	0.005148
3059.250000	0.005159
3059.000000	0.005170
3058.750000	0.005178
3058.500000	0.005182
3058.250000	0.005182
3058.000000	0.005179
3057.750000	0.005174
3057.500000	0.005169

3057.250000	0.005166
3057.000000	0.005165
3056.750000	0.005166
3056.500000	0.005166
3056.250000	0.005165
3056.000000	0.005162
3055.750000	0.005155
3055.500000	0.005145
3055.250000	0.005134
3055.000000	0.005124
3054.750000	0.005116
3054.500000	0.005112
3054.250000	0.005111
3054.000000	0.005113
3053.750000	0.005115
3053.500000	0.005117
3053.250000	0.005117
3053.000000	0.005115
3052.750000	0.005110
3052.500000	0.005104
3052.250000	0.005098
3052.000000	0.005092
3051.750000	0.005088
3051.500000	0.005084
3051.250000	0.005081
3051.000000	0.005080

3050.750000	0.005080
3050.500000	0.005083
3050.250000	0.005088
3050.000000	0.005097
3049.750000	0.005109
3049.500000	0.005121
3049.250000	0.005132
3049.000000	0.005138
3048.750000	0.005138
3048.500000	0.005131
3048.250000	0.005118
3048.000000	0.005104
3047.750000	0.005091
3047.500000	0.005085
3047.250000	0.005086
3047.000000	0.005094
3046.750000	0.005108
3046.500000	0.005124
3046.250000	0.005138
3046.000000	0.005149
3045.750000	0.005155
3045.500000	0.005157
3045.250000	0.005156
3045.000000	0.005154
3044.750000	0.005151
3044.500000	0.005148

3044.250000	0.005144
3044.000000	0.005141
3043.750000	0.005139
3043.500000	0.005140
3043.250000	0.005145
3043.000000	0.005156
3042.750000	0.005172
3042.500000	0.005192
3042.250000	0.005213
3042.000000	0.005232
3041.750000	0.005247
3041.500000	0.005254
3041.250000	0.005255
3041.000000	0.005249
3040.750000	0.005239
3040.500000	0.005229
3040.250000	0.005223
3040.000000	0.005222
3039.750000	0.005228
3039.500000	0.005240
3039.250000	0.005256
3039.000000	0.005273
3038.750000	0.005289
3038.500000	0.005300
3038.250000	0.005306
3038.000000	0.005307

3037.750000	0.005305
3037.500000	0.005300
3037.250000	0.005296
3037.000000	0.005294
3036.750000	0.005295
3036.500000	0.005300
3036.250000	0.005308
3036.000000	0.005320
3035.750000	0.005334
3035.500000	0.005351
3035.250000	0.005368
3035.000000	0.005384
3034.750000	0.005400
3034.500000	0.005413
3034.250000	0.005422
3034.000000	0.005430
3033.750000	0.005436
3033.500000	0.005442
3033.250000	0.005450
3033.000000	0.005462
3032.750000	0.005477
3032.500000	0.005496
3032.250000	0.005517
3032.000000	0.005539
3031.750000	0.005558
3031.500000	0.005575

3031.250000	0.005588
3031.000000	0.005598
3030.750000	0.005605
3030.500000	0.005611
3030.250000	0.005617
3030.000000	0.005625
3029.750000	0.005635
3029.500000	0.005647
3029.250000	0.005661
3029.000000	0.005674
3028.750000	0.005687
3028.500000	0.005698
3028.250000	0.005704
3028.000000	0.005707
3027.750000	0.005705
3027.500000	0.005699
3027.250000	0.005689
3027.000000	0.005677
3026.750000	0.005664
3026.500000	0.005654
3026.250000	0.005647
3026.000000	0.005647
3025.750000	0.005655
3025.500000	0.005669
3025.250000	0.005690
3025.000000	0.005712

3024.750000	0.005735
3024.500000	0.005756
3024.250000	0.005772
3024.000000	0.005786
3023.750000	0.005799
3023.500000	0.005813
3023.250000	0.005829
3023.000000	0.005849
3022.750000	0.005871
3022.500000	0.005894
3022.250000	0.005917
3022.000000	0.005938
3021.750000	0.005958
3021.500000	0.005976
3021.250000	0.005994
3021.000000	0.006011
3020.750000	0.006027
3020.500000	0.006044
3020.250000	0.006059
3020.000000	0.006075
3019.750000	0.006091
3019.500000	0.006109
3019.250000	0.006131
3019.000000	0.006156
3018.750000	0.006186
3018.500000	0.006220

3018.250000	0.006256
3018.000000	0.006294
3017.750000	0.006332
3017.500000	0.006370
3017.250000	0.006408
3017.000000	0.006445
3016.750000	0.006481
3016.500000	0.006514
3016.250000	0.006543
3016.000000	0.006568
3015.750000	0.006588
3015.500000	0.006604
3015.250000	0.006618
3015.000000	0.006633
3014.750000	0.006650
3014.500000	0.006670
3014.250000	0.006695
3014.000000	0.006724
3013.750000	0.006756
3013.500000	0.006789
3013.250000	0.006825
3013.000000	0.006861
3012.750000	0.006899
3012.500000	0.006937
3012.250000	0.006976
3012.000000	0.007016

3011.750000	0.007056
3011.500000	0.007098
3011.250000	0.007142
3011.000000	0.007187
3010.750000	0.007235
3010.500000	0.007285
3010.250000	0.007335
3010.000000	0.007385
3009.750000	0.007432
3009.500000	0.007475
3009.250000	0.007514
3009.000000	0.007547
3008.750000	0.007575
3008.500000	0.007600
3008.250000	0.007623
3008.000000	0.007646
3007.750000	0.007671
3007.500000	0.007698
3007.250000	0.007729
3007.000000	0.007763
3006.750000	0.007802
3006.500000	0.007843
3006.250000	0.007888
3006.000000	0.007934
3005.750000	0.007980
3005.500000	0.008026

3005.250000	0.008069
3005.000000	0.008109
3004.750000	0.008144
3004.500000	0.008177
3004.250000	0.008208
3004.000000	0.008239
3003.750000	0.008272
3003.500000	0.008308
3003.250000	0.008345
3003.000000	0.008384
3002.750000	0.008421
3002.500000	0.008457
3002.250000	0.008490
3002.000000	0.008521
3001.750000	0.008551
3001.500000	0.008584
3001.250000	0.008621
3001.000000	0.008663
3000.750000	0.008709
3000.500000	0.008759
3000.250000	0.008811
3000.000000	0.008862
2999.750000	0.008913
2999.500000	0.008963
2999.250000	0.009012
2999.000000	0.009061

2998.750000	0.009111
2998.500000	0.009161
2998.250000	0.009211
2998.000000	0.009261
2997.750000	0.009310
2997.500000	0.009360
2997.250000	0.009411
2997.000000	0.009465
2996.750000	0.009521
2996.500000	0.009578
2996.250000	0.009636
2996.000000	0.009691
2995.750000	0.009742
2995.500000	0.009788
2995.250000	0.009828
2995.000000	0.009863
2994.750000	0.009893
2994.500000	0.009920
2994.250000	0.009947
2994.000000	0.009974
2993.750000	0.010004
2993.500000	0.010037
2993.250000	0.010074
2993.000000	0.010117
2992.750000	0.010167
2992.500000	0.010224

2992.250000	0.010289
2992.000000	0.010362
2991.750000	0.010440
2991.500000	0.010521
2991.250000	0.010601
2991.000000	0.010676
2990.750000	0.010744
2990.500000	0.010802
2990.250000	0.010850
2990.000000	0.010892
2989.750000	0.010929
2989.500000	0.010964
2989.250000	0.011001
2989.000000	0.011039
2988.750000	0.011080
2988.500000	0.011121
2988.250000	0.011163
2988.000000	0.011203
2987.750000	0.011241
2987.500000	0.011279
2987.250000	0.011315
2987.000000	0.011352
2986.750000	0.011390
2986.500000	0.011430
2986.250000	0.011474
2986.000000	0.011522

2985.750000	0.011573
2985.500000	0.011626
2985.250000	0.011680
2985.000000	0.011732
2984.750000	0.011778
2984.500000	0.011816
2984.250000	0.011846
2984.000000	0.011869
2983.750000	0.011888
2983.500000	0.011906
2983.250000	0.011925
2983.000000	0.011948
2982.750000	0.011975
2982.500000	0.012004
2982.250000	0.012033
2982.000000	0.012062
2981.750000	0.012089
2981.500000	0.012117
2981.250000	0.012146
2981.000000	0.012179
2980.750000	0.012214
2980.500000	0.012253
2980.250000	0.012292
2980.000000	0.012329
2979.750000	0.012362
2979.500000	0.012392

2979.250000	0.012418
2979.000000	0.012441
2978.750000	0.012464
2978.500000	0.012488
2978.250000	0.012511
2978.000000	0.012534
2977.750000	0.012557
2977.500000	0.012579
2977.250000	0.012599
2977.000000	0.012619
2976.750000	0.012641
2976.500000	0.012665
2976.250000	0.012694
2976.000000	0.012729
2975.750000	0.012770
2975.500000	0.012820
2975.250000	0.012879
2975.000000	0.012945
2974.750000	0.013019
2974.500000	0.013097
2974.250000	0.013178
2974.000000	0.013260
2973.750000	0.013342
2973.500000	0.013423
2973.250000	0.013505
2973.000000	0.013590

2972.750000	0.013679
2972.500000	0.013775
2972.250000	0.013879
2972.000000	0.013990
2971.750000	0.014108
2971.500000	0.014229
2971.250000	0.014348
2971.000000	0.014462
2970.750000	0.014566
2970.500000	0.014656
2970.250000	0.014729
2970.000000	0.014784
2969.750000	0.014821
2969.500000	0.014843
2969.250000	0.014853
2969.000000	0.014856
2968.750000	0.014856
2968.500000	0.014857
2968.250000	0.014862
2968.000000	0.014874
2967.750000	0.014895
2967.500000	0.014925
2967.250000	0.014962
2967.000000	0.015006
2966.750000	0.015054
2966.500000	0.015105

2966.250000	0.015158
2966.000000	0.015212
2965.750000	0.015269
2965.500000	0.015329
2965.250000	0.015393
2965.000000	0.015460
2964.750000	0.015529
2964.500000	0.015597
2964.250000	0.015663
2964.000000	0.015728
2963.750000	0.015793
2963.500000	0.015860
2963.250000	0.015930
2963.000000	0.016006
2962.750000	0.016086
2962.500000	0.016167
2962.250000	0.016248
2962.000000	0.016324
2961.750000	0.016394
2961.500000	0.016459
2961.250000	0.016519
2961.000000	0.016577
2960.750000	0.016634
2960.500000	0.016692
2960.250000	0.016749
2960.000000	0.016805

2959.750000	0.016859
2959.500000	0.016912
2959.250000	0.016964
2959.000000	0.017017
2958.750000	0.017071
2958.500000	0.017126
2958.250000	0.017181
2958.000000	0.017233
2957.750000	0.017281
2957.500000	0.017324
2957.250000	0.017363
2957.000000	0.017400
2956.750000	0.017438
2956.500000	0.017479
2956.250000	0.017526
2956.000000	0.017577
2955.750000	0.017633
2955.500000	0.017689
2955.250000	0.017744
2955.000000	0.017794
2954.750000	0.017837
2954.500000	0.017872
2954.250000	0.017902
2954.000000	0.017928
2953.750000	0.017953
2953.500000	0.017980

2953.250000	0.018010
2953.000000	0.018045
2952.750000	0.018083
2952.500000	0.018123
2952.250000	0.018164
2952.000000	0.018204
2951.750000	0.018244
2951.500000	0.018284
2951.250000	0.018326
2951.000000	0.018370
2950.750000	0.018418
2950.500000	0.018468
2950.250000	0.018520
2950.000000	0.018571
2949.750000	0.018623
2949.500000	0.018674
2949.250000	0.018728
2949.000000	0.018784
2948.750000	0.018845
2948.500000	0.018912
2948.250000	0.018984
2948.000000	0.019062
2947.750000	0.019143
2947.500000	0.019226
2947.250000	0.019312
2947.000000	0.019399

2946.750000	0.019487
2946.500000	0.019577
2946.250000	0.019668
2946.000000	0.019760
2945.750000	0.019853
2945.500000	0.019947
2945.250000	0.020043
2945.000000	0.020140
2944.750000	0.020240
2944.500000	0.020342
2944.250000	0.020445
2944.000000	0.020548
2943.750000	0.020651
2943.500000	0.020752
2943.250000	0.020852
2943.000000	0.020952
2942.750000	0.021052
2942.500000	0.021154
2942.250000	0.021258
2942.000000	0.021365
2941.750000	0.021474
2941.500000	0.021586
2941.250000	0.021699
2941.000000	0.021814
2940.750000	0.021929
2940.500000	0.022046

2940.250000	0.022165
2940.000000	0.022284
2939.750000	0.022406
2939.500000	0.022528
2939.250000	0.022651
2939.000000	0.022773
2938.750000	0.022893
2938.500000	0.023009
2938.250000	0.023122
2938.000000	0.023232
2937.750000	0.023339
2937.500000	0.023447
2937.250000	0.023556
2937.000000	0.023668
2936.750000	0.023782
2936.500000	0.023897
2936.250000	0.024011
2936.000000	0.024122
2935.750000	0.024227
2935.500000	0.024329
2935.250000	0.024428
2935.000000	0.024528
2934.750000	0.024630
2934.500000	0.024736
2934.250000	0.024847
2934.000000	0.024961

2933.750000	0.025075
2933.500000	0.025188
2933.250000	0.025296
2933.000000	0.025398
2932.750000	0.025493
2932.500000	0.025580
2932.250000	0.025661
2932.000000	0.025737
2931.750000	0.025811
2931.500000	0.025884
2931.250000	0.025959
2931.000000	0.026037
2930.750000	0.026118
2930.500000	0.026200
2930.250000	0.026283
2930.000000	0.026365
2929.750000	0.026443
2929.500000	0.026515
2929.250000	0.026581
2929.000000	0.026641
2928.750000	0.026695
2928.500000	0.026744
2928.250000	0.026790
2928.000000	0.026836
2927.750000	0.026882
2927.500000	0.026930

2927.250000	0.026977
2927.000000	0.027021
2926.750000	0.027060
2926.500000	0.027090
2926.250000	0.027109
2926.000000	0.027116
2925.750000	0.027114
2925.500000	0.027105
2925.250000	0.027093
2925.000000	0.027080
2924.750000	0.027069
2924.500000	0.027056
2924.250000	0.027039
2924.000000	0.027016
2923.750000	0.026982
2923.500000	0.026939
2923.250000	0.026887
2923.000000	0.026827
2922.750000	0.026763
2922.500000	0.026696
2922.250000	0.026625
2922.000000	0.026549
2921.750000	0.026466
2921.500000	0.026374
2921.250000	0.026273
2921.000000	0.026161

2920.750000	0.026038
2920.500000	0.025906
2920.250000	0.025764
2920.000000	0.025612
2919.750000	0.025449
2919.500000	0.025277
2919.250000	0.025096
2919.000000	0.024908
2918.750000	0.024713
2918.500000	0.024512
2918.250000	0.024306
2918.000000	0.024095
2917.750000	0.023879
2917.500000	0.023659
2917.250000	0.023434
2917.000000	0.023208
2916.750000	0.022982
2916.500000	0.022758
2916.250000	0.022537
2916.000000	0.022319
2915.750000	0.022104
2915.500000	0.021891
2915.250000	0.021679
2915.000000	0.021467
2914.750000	0.021256
2914.500000	0.021045

2914.250000	0.020835
2914.000000	0.020627
2913.750000	0.020422
2913.500000	0.020220
2913.250000	0.020020
2913.000000	0.019823
2912.750000	0.019629
2912.500000	0.019437
2912.250000	0.019248
2912.000000	0.019063
2911.750000	0.018882
2911.500000	0.018704
2911.250000	0.018531
2911.000000	0.018361
2910.750000	0.018196
2910.500000	0.018035
2910.250000	0.017878
2910.000000	0.017727
2909.750000	0.017581
2909.500000	0.017444
2909.250000	0.017314
2909.000000	0.017193
2908.750000	0.017079
2908.500000	0.016971
2908.250000	0.016866
2908.000000	0.016763

2907.750000	0.016660
2907.500000	0.016554
2907.250000	0.016446
2907.000000	0.016335
2906.750000	0.016222
2906.500000	0.016111
2906.250000	0.016002
2906.000000	0.015897
2905.750000	0.015798
2905.500000	0.015705
2905.250000	0.015615
2905.000000	0.015528
2904.750000	0.015442
2904.500000	0.015356
2904.250000	0.015269
2904.000000	0.015184
2903.750000	0.015103
2903.500000	0.015028
2903.250000	0.014960
2903.000000	0.014899
2902.750000	0.014842
2902.500000	0.014786
2902.250000	0.014727
2902.000000	0.014664
2901.750000	0.014594
2901.500000	0.014520

2901.250000	0.014444
2901.000000	0.014368
2900.750000	0.014296
2900.500000	0.014228
2900.250000	0.014166
2900.000000	0.014108
2899.750000	0.014052
2899.500000	0.013998
2899.250000	0.013944
2899.000000	0.013890
2898.750000	0.013836
2898.500000	0.013781
2898.250000	0.013728
2898.000000	0.013675
2897.750000	0.013622
2897.500000	0.013571
2897.250000	0.013520
2897.000000	0.013468
2896.750000	0.013414
2896.500000	0.013358
2896.250000	0.013300
2896.000000	0.013241
2895.750000	0.013184
2895.500000	0.013131
2895.250000	0.013083
2895.000000	0.013043

2894.750000	0.013010
2894.500000	0.012982
2894.250000	0.012956
2894.000000	0.012929
2893.750000	0.012899
2893.500000	0.012864
2893.250000	0.012825
2893.000000	0.012783
2892.750000	0.012740
2892.500000	0.012696
2892.250000	0.012654
2892.000000	0.012613
2891.750000	0.012573
2891.500000	0.012535
2891.250000	0.012499
2891.000000	0.012462
2890.750000	0.012425
2890.500000	0.012387
2890.250000	0.012348
2890.000000	0.012306
2889.750000	0.012264
2889.500000	0.012223
2889.250000	0.012185
2889.000000	0.012150
2888.750000	0.012121
2888.500000	0.012095

2888.250000	0.012071
2888.000000	0.012048
2887.750000	0.012021
2887.500000	0.011992
2887.250000	0.011959
2887.000000	0.011923
2886.750000	0.011887
2886.500000	0.011851
2886.250000	0.011819
2886.000000	0.011791
2885.750000	0.011768
2885.500000	0.011751
2885.250000	0.011738
2885.000000	0.011729
2884.750000	0.011725
2884.500000	0.011723
2884.250000	0.011721
2884.000000	0.011719
2883.750000	0.011714
2883.500000	0.011707
2883.250000	0.011696
2883.000000	0.011682
2882.750000	0.011666
2882.500000	0.011648
2882.250000	0.011630
2882.000000	0.011612

2881.750000	0.011595
2881.500000	0.011580
2881.250000	0.011568
2881.000000	0.011560
2880.750000	0.011555
2880.500000	0.011554
2880.250000	0.011556
2880.000000	0.011561
2879.750000	0.011569
2879.500000	0.011578
2879.250000	0.011590
2879.000000	0.011603
2878.750000	0.011618
2878.500000	0.011634
2878.250000	0.011650
2878.000000	0.011667
2877.750000	0.011683
2877.500000	0.011700
2877.250000	0.011717
2877.000000	0.011735
2876.750000	0.011756
2876.500000	0.011780
2876.250000	0.011806
2876.000000	0.011834
2875.750000	0.011864
2875.500000	0.011894

2875.250000	0.011924
2875.000000	0.011952
2874.750000	0.011979
2874.500000	0.012003
2874.250000	0.012024
2874.000000	0.012043
2873.750000	0.012061
2873.500000	0.012080
2873.250000	0.012102
2873.000000	0.012126
2872.750000	0.012152
2872.500000	0.012179
2872.250000	0.012204
2872.000000	0.012225
2871.750000	0.012241
2871.500000	0.012254
2871.250000	0.012264
2871.000000	0.012272
2870.750000	0.012280
2870.500000	0.012289
2870.250000	0.012299
2870.000000	0.012309
2869.750000	0.012319
2869.500000	0.012330
2869.250000	0.012343
2869.000000	0.012358

2868.750000	0.012377
2868.500000	0.012397
2868.250000	0.012419
2868.000000	0.012439
2867.750000	0.012455
2867.500000	0.012468
2867.250000	0.012479
2867.000000	0.012490
2866.750000	0.012505
2866.500000	0.012527
2866.250000	0.012556
2866.000000	0.012594
2865.750000	0.012638
2865.500000	0.012687
2865.250000	0.012738
2865.000000	0.012790
2864.750000	0.012841
2864.500000	0.012890
2864.250000	0.012938
2864.000000	0.012986
2863.750000	0.013033
2863.500000	0.013083
2863.250000	0.013135
2863.000000	0.013192
2862.750000	0.013256
2862.500000	0.013326

2862.250000	0.013403
2862.000000	0.013484
2861.750000	0.013569
2861.500000	0.013656
2861.250000	0.013743
2861.000000	0.013829
2860.750000	0.013912
2860.500000	0.013993
2860.250000	0.014068
2860.000000	0.014139
2859.750000	0.014205
2859.500000	0.014266
2859.250000	0.014326
2859.000000	0.014386
2858.750000	0.014449
2858.500000	0.014514
2858.250000	0.014581
2858.000000	0.014650
2857.750000	0.014717
2857.500000	0.014782
2857.250000	0.014845
2857.000000	0.014904
2856.750000	0.014962
2856.500000	0.015018
2856.250000	0.015072
2856.000000	0.015121

2855.750000	0.015163
2855.500000	0.015195
2855.250000	0.015215
2855.000000	0.015223
2854.750000	0.015220
2854.500000	0.015208
2854.250000	0.015190
2854.000000	0.015168
2853.750000	0.015144
2853.500000	0.015118
2853.250000	0.015090
2853.000000	0.015060
2852.750000	0.015026
2852.500000	0.014986
2852.250000	0.014938
2852.000000	0.014882
2851.750000	0.014816
2851.500000	0.014739
2851.250000	0.014652
2851.000000	0.014555
2850.750000	0.014449
2850.500000	0.014333
2850.250000	0.014208
2850.000000	0.014071
2849.750000	0.013923
2849.500000	0.013761

2849.250000	0.013588
2849.000000	0.013403
2848.750000	0.013208
2848.500000	0.013005
2848.250000	0.012795
2848.000000	0.012579
2847.750000	0.012358
2847.500000	0.012132
2847.250000	0.011902
2847.000000	0.011668
2846.750000	0.011432
2846.500000	0.011197
2846.250000	0.010963
2846.000000	0.010732
2845.750000	0.010507
2845.500000	0.010286
2845.250000	0.010072
2845.000000	0.009864
2844.750000	0.009661
2844.500000	0.009464
2844.250000	0.009272
2844.000000	0.009084
2843.750000	0.008901
2843.500000	0.008722
2843.250000	0.008549
2843.000000	0.008381

2842.750000	0.008222
2842.500000	0.008071
2842.250000	0.007929
2842.000000	0.007797
2841.750000	0.007672
2841.500000	0.007553
2841.250000	0.007438
2841.000000	0.007327
2840.750000	0.007217
2840.500000	0.007108
2840.250000	0.007000
2840.000000	0.006894
2839.750000	0.006791
2839.500000	0.006691
2839.250000	0.006596
2839.000000	0.006506
2838.750000	0.006424
2838.500000	0.006349
2838.250000	0.006280
2838.000000	0.006217
2837.750000	0.006157
2837.500000	0.006101
2837.250000	0.006047
2837.000000	0.005994
2836.750000	0.005943
2836.500000	0.005895

2836.250000	0.005850
2836.000000	0.005808
2835.750000	0.005768
2835.500000	0.005729
2835.250000	0.005689
2835.000000	0.005649
2834.750000	0.005607
2834.500000	0.005564
2834.250000	0.005520
2834.000000	0.005478
2833.750000	0.005438
2833.500000	0.005402
2833.250000	0.005370
2833.000000	0.005343
2832.750000	0.005319
2832.500000	0.005298
2832.250000	0.005278
2832.000000	0.005258
2831.750000	0.005236
2831.500000	0.005212
2831.250000	0.005186
2831.000000	0.005160
2830.750000	0.005134
2830.500000	0.005110
2830.250000	0.005089
2830.000000	0.005070

2829.750000	0.005052
2829.500000	0.005033
2829.250000	0.005012
2829.000000	0.004988
2828.750000	0.004962
2828.500000	0.004934
2828.250000	0.004906
2828.000000	0.004880
2827.750000	0.004858
2827.500000	0.004838
2827.250000	0.004821
2827.000000	0.004805
2826.750000	0.004789
2826.500000	0.004770
2826.250000	0.004749
2826.000000	0.004727
2825.750000	0.004702
2825.500000	0.004678
2825.250000	0.004656
2825.000000	0.004634
2824.750000	0.004615
2824.500000	0.004597
2824.250000	0.004578
2824.000000	0.004558
2823.750000	0.004535
2823.500000	0.004511

2823.250000	0.004486
2823.000000	0.004462
2822.750000	0.004442
2822.500000	0.004427
2822.250000	0.004418
2822.000000	0.004414
2821.750000	0.004414
2821.500000	0.004413
2821.250000	0.004408
2821.000000	0.004396
2820.750000	0.004376
2820.500000	0.004348
2820.250000	0.004314
2820.000000	0.004279
2819.750000	0.004247
2819.500000	0.004220
2819.250000	0.004203
2819.000000	0.004194
2818.750000	0.004195
2818.500000	0.004201
2818.250000	0.004211
2818.000000	0.004219
2817.750000	0.004224
2817.500000	0.004224
2817.250000	0.004218
2817.000000	0.004206

2816.750000	0.004190
2816.500000	0.004172
2816.250000	0.004152
2816.000000	0.004132
2815.750000	0.004113
2815.500000	0.004095
2815.250000	0.004077
2815.000000	0.004061
2814.750000	0.004046
2814.500000	0.004031
2814.250000	0.004016
2814.000000	0.004002
2813.750000	0.003987
2813.500000	0.003974
2813.250000	0.003962
2813.000000	0.003952
2812.750000	0.003942
2812.500000	0.003934
2812.250000	0.003924
2812.000000	0.003913
2811.750000	0.003900
2811.500000	0.003885
2811.250000	0.003868
2811.000000	0.003852
2810.750000	0.003837
2810.500000	0.003824

2810.250000	0.003815
2810.000000	0.003807
2809.750000	0.003801
2809.500000	0.003796
2809.250000	0.003791
2809.000000	0.003788
2808.750000	0.003786
2808.500000	0.003785
2808.250000	0.003785
2808.000000	0.003786
2807.750000	0.003785
2807.500000	0.003782
2807.250000	0.003775
2807.000000	0.003765
2806.750000	0.003752
2806.500000	0.003737
2806.250000	0.003722
2806.000000	0.003709
2805.750000	0.003696
2805.500000	0.003685
2805.250000	0.003674
2805.000000	0.003664
2804.750000	0.003655
2804.500000	0.003646
2804.250000	0.003639
2804.000000	0.003634

2803.750000	0.003632
2803.500000	0.003632
2803.250000	0.003635
2803.000000	0.003638
2802.750000	0.003642
2802.500000	0.003644
2802.250000	0.003645
2802.000000	0.003642
2801.750000	0.003635
2801.500000	0.003626
2801.250000	0.003613
2801.000000	0.003598
2800.750000	0.003582
2800.500000	0.003565
2800.250000	0.003550
2800.000000	0.003536
2799.750000	0.003526
2799.500000	0.003519
2799.250000	0.003516
2799.000000	0.003517
2798.750000	0.003522
2798.500000	0.003531
2798.250000	0.003541
2798.000000	0.003550
2797.750000	0.003558
2797.500000	0.003563

2797.250000	0.003564
2797.000000	0.003560
2796.750000	0.003552
2796.500000	0.003541
2796.250000	0.003527
2796.000000	0.003511
2795.750000	0.003494
2795.500000	0.003478
2795.250000	0.003464
2795.000000	0.003452
2794.750000	0.003444
2794.500000	0.003438
2794.250000	0.003435
2794.000000	0.003435
2793.750000	0.003435
2793.500000	0.003436
2793.250000	0.003436
2793.000000	0.003434
2792.750000	0.003430
2792.500000	0.003424
2792.250000	0.003416
2792.000000	0.003405
2791.750000	0.003392
2791.500000	0.003379
2791.250000	0.003365
2791.000000	0.003353

2790.750000	0.003343
2790.500000	0.003336
2790.250000	0.003332
2790.000000	0.003331
2789.750000	0.003330
2789.500000	0.003330
2789.250000	0.003328
2789.000000	0.003325
2788.750000	0.003319
2788.500000	0.003311
2788.250000	0.003301
2788.000000	0.003290
2787.750000	0.003280
2787.500000	0.003270
2787.250000	0.003262
2787.000000	0.003258
2786.750000	0.003257
2786.500000	0.003260
2786.250000	0.003266
2786.000000	0.003274
2785.750000	0.003283
2785.500000	0.003292
2785.250000	0.003300
2785.000000	0.003305
2784.750000	0.003309
2784.500000	0.003311

2784.250000	0.003310
2784.000000	0.003307
2783.750000	0.003301
2783.500000	0.003294
2783.250000	0.003285
2783.000000	0.003275
2782.750000	0.003265
2782.500000	0.003257
2782.250000	0.003249
2782.000000	0.003243
2781.750000	0.003239
2781.500000	0.003235
2781.250000	0.003233
2781.000000	0.003232
2780.750000	0.003231
2780.500000	0.003231
2780.250000	0.003231
2780.000000	0.003229
2779.750000	0.003225
2779.500000	0.003219
2779.250000	0.003210
2779.000000	0.003200
2778.750000	0.003190
2778.500000	0.003181
2778.250000	0.003172
2778.000000	0.003165

2777.750000	0.003159
2777.500000	0.003152
2777.250000	0.003145
2777.000000	0.003140
2776.750000	0.003135
2776.500000	0.003133
2776.250000	0.003132
2776.000000	0.003132
2775.750000	0.003131
2775.500000	0.003129
2775.250000	0.003124
2775.000000	0.003117
2774.750000	0.003111
2774.500000	0.003107
2774.250000	0.003108
2774.000000	0.003114
2773.750000	0.003124
2773.500000	0.003137
2773.250000	0.003148
2773.000000	0.003156
2772.750000	0.003158
2772.500000	0.003154
2772.250000	0.003145
2772.000000	0.003133
2771.750000	0.003120
2771.500000	0.003109

2771.250000	0.003099
2771.000000	0.003093
2770.750000	0.003089
2770.500000	0.003087
2770.250000	0.003086
2770.000000	0.003083
2769.750000	0.003079
2769.500000	0.003072
2769.250000	0.003064
2769.000000	0.003056
2768.750000	0.003048
2768.500000	0.003044
2768.250000	0.003042
2768.000000	0.003044
2767.750000	0.003047
2767.500000	0.003052
2767.250000	0.003056
2767.000000	0.003058
2766.750000	0.003059
2766.500000	0.003058
2766.250000	0.003057
2766.000000	0.003057
2765.750000	0.003059
2765.500000	0.003063
2765.250000	0.003068
2765.000000	0.003075

2764.750000	0.003081
2764.500000	0.003085
2764.250000	0.003085
2764.000000	0.003081
2763.750000	0.003073
2763.500000	0.003061
2763.250000	0.003047
2763.000000	0.003034
2762.750000	0.003023
2762.500000	0.003018
2762.250000	0.003020
2762.000000	0.003026
2761.750000	0.003037
2761.500000	0.003050
2761.250000	0.003061
2761.000000	0.003069
2760.750000	0.003073
2760.500000	0.003073
2760.250000	0.003069
2760.000000	0.003063
2759.750000	0.003055
2759.500000	0.003048
2759.250000	0.003040
2759.000000	0.003034
2758.750000	0.003029
2758.500000	0.003024

2758.250000	0.003020
2758.000000	0.003015
2757.750000	0.003009
2757.500000	0.003001
2757.250000	0.002992
2757.000000	0.002983
2756.750000	0.002974
2756.500000	0.002967
2756.250000	0.002964
2756.000000	0.002966
2755.750000	0.002971
2755.500000	0.002979
2755.250000	0.002988
2755.000000	0.002995
2754.750000	0.003000
2754.500000	0.003002
2754.250000	0.003002
2754.000000	0.002999
2753.750000	0.002995
2753.500000	0.002991
2753.250000	0.002986
2753.000000	0.002983
2752.750000	0.002980
2752.500000	0.002978
2752.250000	0.002977
2752.000000	0.002975

2751.750000	0.002973
2751.500000	0.002969
2751.250000	0.002964
2751.000000	0.002957
2750.750000	0.002951
2750.500000	0.002944
2750.250000	0.002939
2750.000000	0.002936
2749.750000	0.002933
2749.500000	0.002931
2749.250000	0.002928
2749.000000	0.002924
2748.750000	0.002919
2748.500000	0.002913
2748.250000	0.002909
2748.000000	0.002906
2747.750000	0.002907
2747.500000	0.002910
2747.250000	0.002917
2747.000000	0.002925
2746.750000	0.002935
2746.500000	0.002945
2746.250000	0.002954
2746.000000	0.002959
2745.750000	0.002960
2745.500000	0.002956

2745.250000	0.002945
2745.000000	0.002929
2744.750000	0.002909
2744.500000	0.002890
2744.250000	0.002875
2744.000000	0.002865
2743.750000	0.002863
2743.500000	0.002867
2743.250000	0.002875
2743.000000	0.002885
2742.750000	0.002894
2742.500000	0.002901
2742.250000	0.002906
2742.000000	0.002910
2741.750000	0.002914
2741.500000	0.002918
2741.250000	0.002922
2741.000000	0.002926
2740.750000	0.002927
2740.500000	0.002925
2740.250000	0.002920
2740.000000	0.002911
2739.750000	0.002901
2739.500000	0.002890
2739.250000	0.002879
2739.000000	0.002869

2738.750000	0.002859
2738.500000	0.002849
2738.250000	0.002841
2738.000000	0.002834
2737.750000	0.002829
2737.500000	0.002827
2737.250000	0.002830
2737.000000	0.002836
2736.750000	0.002844
2736.500000	0.002852
2736.250000	0.002857
2736.000000	0.002858
2735.750000	0.002855
2735.500000	0.002847
2735.250000	0.002838
2735.000000	0.002829
2734.750000	0.002823
2734.500000	0.002822
2734.250000	0.002827
2734.000000	0.002836
2733.750000	0.002847
2733.500000	0.002857
2733.250000	0.002865
2733.000000	0.002869
2732.750000	0.002869
2732.500000	0.002866

2732.250000	0.002862
2732.000000	0.002856
2731.750000	0.002851
2731.500000	0.002847
2731.250000	0.002842
2731.000000	0.002835
2730.750000	0.002827
2730.500000	0.002818
2730.250000	0.002807
2730.000000	0.002797
2729.750000	0.002788
2729.500000	0.002781
2729.250000	0.002775
2729.000000	0.002771
2728.750000	0.002767
2728.500000	0.002762
2728.250000	0.002755
2728.000000	0.002748
2727.750000	0.002740
2727.500000	0.002733
2727.250000	0.002727
2727.000000	0.002724
2726.750000	0.002723
2726.500000	0.002726
2726.250000	0.002730
2726.000000	0.002737

2725.750000	0.002744
2725.500000	0.002751
2725.250000	0.002757
2725.000000	0.002760
2724.750000	0.002759
2724.500000	0.002754
2724.250000	0.002744
2724.000000	0.002731
2723.750000	0.002716
2723.500000	0.002701
2723.250000	0.002689
2723.000000	0.002680
2722.750000	0.002676
2722.500000	0.002678
2722.250000	0.002682
2722.000000	0.002689
2721.750000	0.002695
2721.500000	0.002699
2721.250000	0.002700
2721.000000	0.002697
2720.750000	0.002692
2720.500000	0.002686
2720.250000	0.002680
2720.000000	0.002675
2719.750000	0.002673
2719.500000	0.002673

2719.250000	0.002673
2719.000000	0.002673
2718.750000	0.002671
2718.500000	0.002668
2718.250000	0.002663
2718.000000	0.002658
2717.750000	0.002653
2717.500000	0.002649
2717.250000	0.002646
2717.000000	0.002643
2716.750000	0.002641
2716.500000	0.002639
2716.250000	0.002636
2716.000000	0.002634
2715.750000	0.002632
2715.500000	0.002631
2715.250000	0.002631
2715.000000	0.002631
2714.750000	0.002631
2714.500000	0.002630
2714.250000	0.002628
2714.000000	0.002625
2713.750000	0.002621
2713.500000	0.002616
2713.250000	0.002612
2713.000000	0.002608

2712.750000	0.002606
2712.500000	0.002604
2712.250000	0.002601
2712.000000	0.002599
2711.750000	0.002595
2711.500000	0.002591
2711.250000	0.002586
2711.000000	0.002582
2710.750000	0.002579
2710.500000	0.002577
2710.250000	0.002576
2710.000000	0.002575
2709.750000	0.002576
2709.500000	0.002576
2709.250000	0.002575
2709.000000	0.002575
2708.750000	0.002574
2708.500000	0.002572
2708.250000	0.002571
2708.000000	0.002569
2707.750000	0.002568
2707.500000	0.002567
2707.250000	0.002566
2707.000000	0.002565
2706.750000	0.002562
2706.500000	0.002559

2706.250000	0.002553
2706.000000	0.002545
2705.750000	0.002536
2705.500000	0.002527
2705.250000	0.002519
2705.000000	0.002513
2704.750000	0.002511
2704.500000	0.002512
2704.250000	0.002516
2704.000000	0.002522
2703.750000	0.002529
2703.500000	0.002535
2703.250000	0.002540
2703.000000	0.002544
2702.750000	0.002545
2702.500000	0.002544
2702.250000	0.002540
2702.000000	0.002534
2701.750000	0.002526
2701.500000	0.002516
2701.250000	0.002506
2701.000000	0.002497
2700.750000	0.002490
2700.500000	0.002487
2700.250000	0.002486
2700.000000	0.002487

2699.750000	0.002489
2699.500000	0.002491
2699.250000	0.002491
2699.000000	0.002489
2698.750000	0.002486
2698.500000	0.002482
2698.250000	0.002479
2698.000000	0.002476
2697.750000	0.002475
2697.500000	0.002476
2697.250000	0.002478
2697.000000	0.002481
2696.750000	0.002484
2696.500000	0.002489
2696.250000	0.002494
2696.000000	0.002497
2695.750000	0.002499
2695.500000	0.002498
2695.250000	0.002493
2695.000000	0.002484
2694.750000	0.002474
2694.500000	0.002464
2694.250000	0.002455
2694.000000	0.002450
2693.750000	0.002448
2693.500000	0.002450

2693.250000	0.002453
2693.000000	0.002457
2692.750000	0.002460
2692.500000	0.002461
2692.250000	0.002459
2692.000000	0.002456
2691.750000	0.002452
2691.500000	0.002447
2691.250000	0.002441
2691.000000	0.002435
2690.750000	0.002431
2690.500000	0.002427
2690.250000	0.002426
2690.000000	0.002428
2689.750000	0.002432
2689.500000	0.002439
2689.250000	0.002449
2689.000000	0.002459
2688.750000	0.002470
2688.500000	0.002480
2688.250000	0.002488
2688.000000	0.002494
2687.750000	0.002497
2687.500000	0.002496
2687.250000	0.002493
2687.000000	0.002486

2686.750000	0.002478
2686.500000	0.002469
2686.250000	0.002460
2686.000000	0.002452
2685.750000	0.002446
2685.500000	0.002442
2685.250000	0.002440
2685.000000	0.002439
2684.750000	0.002440
2684.500000	0.002442
2684.250000	0.002444
2684.000000	0.002448
2683.750000	0.002450
2683.500000	0.002452
2683.250000	0.002452
2683.000000	0.002449
2682.750000	0.002444
2682.500000	0.002436
2682.250000	0.002428
2682.000000	0.002420
2681.750000	0.002413
2681.500000	0.002408
2681.250000	0.002404
2681.000000	0.002402
2680.750000	0.002400
2680.500000	0.002399

2680.250000	0.002399
2680.000000	0.002400
2679.750000	0.002403
2679.500000	0.002406
2679.250000	0.002409
2679.000000	0.002411
2678.750000	0.002413
2678.500000	0.002412
2678.250000	0.002410
2678.000000	0.002406
2677.750000	0.002401
2677.500000	0.002394
2677.250000	0.002387
2677.000000	0.002381
2676.750000	0.002374
2676.500000	0.002368
2676.250000	0.002364
2676.000000	0.002360
2675.750000	0.002357
2675.500000	0.002354
2675.250000	0.002352
2675.000000	0.002349
2674.750000	0.002345
2674.500000	0.002341
2674.250000	0.002337
2674.000000	0.002333

2673.750000	0.002330
2673.500000	0.002328
2673.250000	0.002329
2673.000000	0.002330
2672.750000	0.002332
2672.500000	0.002336
2672.250000	0.002340
2672.000000	0.002344
2671.750000	0.002350
2671.500000	0.002356
2671.250000	0.002362
2671.000000	0.002367
2670.750000	0.002370
2670.500000	0.002371
2670.250000	0.002368
2670.000000	0.002362
2669.750000	0.002354
2669.500000	0.002345
2669.250000	0.002336
2669.000000	0.002329
2668.750000	0.002325
2668.500000	0.002324
2668.250000	0.002326
2668.000000	0.002331
2667.750000	0.002337
2667.500000	0.002343

2667.250000	0.002348
2667.000000	0.002353
2666.750000	0.002356
2666.500000	0.002360
2666.250000	0.002365
2666.000000	0.002372
2665.750000	0.002380
2665.500000	0.002389
2665.250000	0.002398
2665.000000	0.002405
2664.750000	0.002411
2664.500000	0.002413
2664.250000	0.002413
2664.000000	0.002410
2663.750000	0.002407
2663.500000	0.002403
2663.250000	0.002400
2663.000000	0.002398
2662.750000	0.002396
2662.500000	0.002396
2662.250000	0.002397
2662.000000	0.002398
2661.750000	0.002400
2661.500000	0.002402
2661.250000	0.002403
2661.000000	0.002405

2660.750000	0.002407
2660.500000	0.002408
2660.250000	0.002409
2660.000000	0.002410
2659.750000	0.002410
2659.500000	0.002409
2659.250000	0.002406
2659.000000	0.002401
2658.750000	0.002394
2658.500000	0.002386
2658.250000	0.002378
2658.000000	0.002372
2657.750000	0.002368
2657.500000	0.002368
2657.250000	0.002370
2657.000000	0.002375
2656.750000	0.002379
2656.500000	0.002383
2656.250000	0.002384
2656.000000	0.002383
2655.750000	0.002380
2655.500000	0.002376
2655.250000	0.002371
2655.000000	0.002368
2654.750000	0.002365
2654.500000	0.002363

2654.250000	0.002362
2654.000000	0.002362
2653.750000	0.002363
2653.500000	0.002365
2653.250000	0.002368
2653.000000	0.002372
2652.750000	0.002376
2652.500000	0.002380
2652.250000	0.002381
2652.000000	0.002380
2651.750000	0.002377
2651.500000	0.002372
2651.250000	0.002366
2651.000000	0.002362
2650.750000	0.002359
2650.500000	0.002359
2650.250000	0.002362
2650.000000	0.002366
2649.750000	0.002371
2649.500000	0.002377
2649.250000	0.002381
2649.000000	0.002384
2648.750000	0.002386
2648.500000	0.002385
2648.250000	0.002383
2648.000000	0.002378

2647.750000	0.002372
2647.500000	0.002365
2647.250000	0.002356
2647.000000	0.002348
2646.750000	0.002340
2646.500000	0.002333
2646.250000	0.002326
2646.000000	0.002320
2645.750000	0.002313
2645.500000	0.002305
2645.250000	0.002298
2645.000000	0.002292
2644.750000	0.002289
2644.500000	0.002288
2644.250000	0.002292
2644.000000	0.002298
2643.750000	0.002306
2643.500000	0.002313
2643.250000	0.002317
2643.000000	0.002317
2642.750000	0.002313
2642.500000	0.002305
2642.250000	0.002296
2642.000000	0.002287
2641.750000	0.002280
2641.500000	0.002274

2641.250000	0.002270
2641.000000	0.002266
2640.750000	0.002263
2640.500000	0.002260
2640.250000	0.002258
2640.000000	0.002256
2639.750000	0.002256
2639.500000	0.002258
2639.250000	0.002260
2639.000000	0.002262
2638.750000	0.002264
2638.500000	0.002264
2638.250000	0.002264
2638.000000	0.002262
2637.750000	0.002260
2637.500000	0.002258
2637.250000	0.002257
2637.000000	0.002256
2636.750000	0.002257
2636.500000	0.002258
2636.250000	0.002261
2636.000000	0.002264
2635.750000	0.002267
2635.500000	0.002270
2635.250000	0.002270
2635.000000	0.002267

2634.750000	0.002260
2634.500000	0.002250
2634.250000	0.002239
2634.000000	0.002228
2633.750000	0.002220
2633.500000	0.002215
2633.250000	0.002216
2633.000000	0.002221
2632.750000	0.002229
2632.500000	0.002239
2632.250000	0.002248
2632.000000	0.002255
2631.750000	0.002258
2631.500000	0.002254
2631.250000	0.002245
2631.000000	0.002229
2630.750000	0.002210
2630.500000	0.002191
2630.250000	0.002174
2630.000000	0.002163
2629.750000	0.002159
2629.500000	0.002164
2629.250000	0.002174
2629.000000	0.002186
2628.750000	0.002195
2628.500000	0.002198

2628.250000	0.002192
2628.000000	0.002179
2627.750000	0.002160
2627.500000	0.002139
2627.250000	0.002120
2627.000000	0.002107
2626.750000	0.002100
2626.500000	0.002099
2626.250000	0.002104
2626.000000	0.002113
2625.750000	0.002123
2625.500000	0.002134
2625.250000	0.002145
2625.000000	0.002156
2624.750000	0.002165
2624.500000	0.002171
2624.250000	0.002175
2624.000000	0.002175
2623.750000	0.002173
2623.500000	0.002168
2623.250000	0.002164
2623.000000	0.002161
2622.750000	0.002161
2622.500000	0.002163
2622.250000	0.002167
2622.000000	0.002170

2621.750000	0.002172
2621.500000	0.002171
2621.250000	0.002166
2621.000000	0.002157
2620.750000	0.002146
2620.500000	0.002134
2620.250000	0.002124
2620.000000	0.002115
2619.750000	0.002109
2619.500000	0.002105
2619.250000	0.002103
2619.000000	0.002101
2618.750000	0.002097
2618.500000	0.002091
2618.250000	0.002083
2618.000000	0.002073
2617.750000	0.002063
2617.500000	0.002053
2617.250000	0.002046
2617.000000	0.002041
2616.750000	0.002040
2616.500000	0.002043
2616.250000	0.002049
2616.000000	0.002056
2615.750000	0.002064
2615.500000	0.002070

2615.250000	0.002075
2615.000000	0.002076
2614.750000	0.002075
2614.500000	0.002072
2614.250000	0.002069
2614.000000	0.002068
2613.750000	0.002068
2613.500000	0.002072
2613.250000	0.002078
2613.000000	0.002084
2612.750000	0.002090
2612.500000	0.002093
2612.250000	0.002091
2612.000000	0.002086
2611.750000	0.002077
2611.500000	0.002065
2611.250000	0.002054
2611.000000	0.002046
2610.750000	0.002042
2610.500000	0.002043
2610.250000	0.002048
2610.000000	0.002057
2609.750000	0.002065
2609.500000	0.002072
2609.250000	0.002075
2609.000000	0.002072

2608.750000	0.002065
2608.500000	0.002055
2608.250000	0.002044
2608.000000	0.002036
2607.750000	0.002033
2607.500000	0.002035
2607.250000	0.002042
2607.000000	0.002050
2606.750000	0.002057
2606.500000	0.002060
2606.250000	0.002057
2606.000000	0.002047
2605.750000	0.002033
2605.500000	0.002016
2605.250000	0.002000
2605.000000	0.001988
2604.750000	0.001982
2604.500000	0.001981
2604.250000	0.001984
2604.000000	0.001990
2603.750000	0.001994
2603.500000	0.001997
2603.250000	0.001996
2603.000000	0.001992
2602.750000	0.001987
2602.500000	0.001982

2602.250000	0.001979
2602.000000	0.001976
2601.750000	0.001975
2601.500000	0.001973
2601.250000	0.001969
2601.000000	0.001964
2600.750000	0.001957
2600.500000	0.001950
2600.250000	0.001944
2600.000000	0.001941
2599.750000	0.001942
2599.500000	0.001947
2599.250000	0.001954
2599.000000	0.001962
2598.750000	0.001970
2598.500000	0.001976
2598.250000	0.001980
2598.000000	0.001983
2597.750000	0.001984
2597.500000	0.001984
2597.250000	0.001983
2597.000000	0.001982
2596.750000	0.001980
2596.500000	0.001979
2596.250000	0.001980
2596.000000	0.001982

2595.750000	0.001987
2595.500000	0.001992
2595.250000	0.001997
2595.000000	0.002001
2594.750000	0.002002
2594.500000	0.002001
2594.250000	0.001997
2594.000000	0.001994
2593.750000	0.001991
2593.500000	0.001991
2593.250000	0.001992
2593.000000	0.001996
2592.750000	0.002001
2592.500000	0.002006
2592.250000	0.002011
2592.000000	0.002015
2591.750000	0.002021
2591.500000	0.002029
2591.250000	0.002038
2591.000000	0.002049
2590.750000	0.002060
2590.500000	0.002068
2590.250000	0.002072
2590.000000	0.002071
2589.750000	0.002065
2589.500000	0.002055

2589.250000	0.002043
2589.000000	0.002032
2588.750000	0.002022
2588.500000	0.002016
2588.250000	0.002014
2588.000000	0.002014
2587.750000	0.002016
2587.500000	0.002017
2587.250000	0.002015
2587.000000	0.002010
2586.750000	0.002003
2586.500000	0.001993
2586.250000	0.001983
2586.000000	0.001973
2585.750000	0.001964
2585.500000	0.001956
2585.250000	0.001949
2585.000000	0.001943
2584.750000	0.001936
2584.500000	0.001931
2584.250000	0.001926
2584.000000	0.001924
2583.750000	0.001925
2583.500000	0.001929
2583.250000	0.001936
2583.000000	0.001943

2582.750000	0.001949
2582.500000	0.001953
2582.250000	0.001954
2582.000000	0.001954
2581.750000	0.001952
2581.500000	0.001950
2581.250000	0.001949
2581.000000	0.001950
2580.750000	0.001951
2580.500000	0.001953
2580.250000	0.001955
2580.000000	0.001957
2579.750000	0.001960
2579.500000	0.001962
2579.250000	0.001965
2579.000000	0.001967
2578.750000	0.001966
2578.500000	0.001963
2578.250000	0.001956
2578.000000	0.001948
2577.750000	0.001938
2577.500000	0.001928
2577.250000	0.001918
2577.000000	0.001909
2576.750000	0.001902
2576.500000	0.001897

2576.250000	0.001895
2576.000000	0.001896
2575.750000	0.001901
2575.500000	0.001909
2575.250000	0.001921
2575.000000	0.001934
2574.750000	0.001947
2574.500000	0.001959
2574.250000	0.001969
2574.000000	0.001978
2573.750000	0.001987
2573.500000	0.001998
2573.250000	0.002011
2573.000000	0.002023
2572.750000	0.002032
2572.500000	0.002035
2572.250000	0.002029
2572.000000	0.002014
2571.750000	0.001990
2571.500000	0.001962
2571.250000	0.001932
2571.000000	0.001903
2570.750000	0.001877
2570.500000	0.001855
2570.250000	0.001838
2570.000000	0.001826

2569.750000	0.001822
2569.500000	0.001827
2569.250000	0.001841
2569.000000	0.001863
2568.750000	0.001891
2568.500000	0.001918
2568.250000	0.001939
2568.000000	0.001950
2567.750000	0.001949
2567.500000	0.001938
2567.250000	0.001921
2567.000000	0.001904
2566.750000	0.001891
2566.500000	0.001888
2566.250000	0.001892
2566.000000	0.001903
2565.750000	0.001916
2565.500000	0.001927
2565.250000	0.001934
2565.000000	0.001936
2564.750000	0.001936
2564.500000	0.001934
2564.250000	0.001934
2564.000000	0.001937
2563.750000	0.001942
2563.500000	0.001948

2563.250000	0.001953
2563.000000	0.001956
2562.750000	0.001958
2562.500000	0.001960
2562.250000	0.001963
2562.000000	0.001971
2561.750000	0.001983
2561.500000	0.002000
2561.250000	0.002018
2561.000000	0.002032
2560.750000	0.002040
2560.500000	0.002038
2560.250000	0.002025
2560.000000	0.002003
2559.750000	0.001976
2559.500000	0.001947
2559.250000	0.001922
2559.000000	0.001902
2558.750000	0.001888
2558.500000	0.001880
2558.250000	0.001876
2558.000000	0.001873
2557.750000	0.001872
2557.500000	0.001870
2557.250000	0.001869
2557.000000	0.001868

2556.750000	0.001868
2556.500000	0.001869
2556.250000	0.001872
2556.000000	0.001877
2555.750000	0.001886
2555.500000	0.001896
2555.250000	0.001908
2555.000000	0.001920
2554.750000	0.001930
2554.500000	0.001936
2554.250000	0.001937
2554.000000	0.001932
2553.750000	0.001923
2553.500000	0.001911
2553.250000	0.001896
2553.000000	0.001881
2552.750000	0.001867
2552.500000	0.001855
2552.250000	0.001846
2552.000000	0.001841
2551.750000	0.001843
2551.500000	0.001850
2551.250000	0.001863
2551.000000	0.001877
2550.750000	0.001890
2550.500000	0.001899

2550.250000	0.001900
2550.000000	0.001895
2549.750000	0.001885
2549.500000	0.001876
2549.250000	0.001870
2549.000000	0.001872
2548.750000	0.001882
2548.500000	0.001898
2548.250000	0.001917
2548.000000	0.001934
2547.750000	0.001944
2547.500000	0.001946
2547.250000	0.001938
2547.000000	0.001923
2546.750000	0.001901
2546.500000	0.001876
2546.250000	0.001852
2546.000000	0.001830
2545.750000	0.001812
2545.500000	0.001801
2545.250000	0.001796
2545.000000	0.001798
2544.750000	0.001803
2544.500000	0.001810
2544.250000	0.001816
2544.000000	0.001820

2543.750000	0.001819
2543.500000	0.001814
2543.250000	0.001806
2543.000000	0.001796
2542.750000	0.001786
2542.500000	0.001775
2542.250000	0.001764
2542.000000	0.001755
2541.750000	0.001748
2541.500000	0.001745
2541.250000	0.001747
2541.000000	0.001755
2540.750000	0.001768
2540.500000	0.001783
2540.250000	0.001796
2540.000000	0.001802
2539.750000	0.001800
2539.500000	0.001787
2539.250000	0.001765
2539.000000	0.001738
2538.750000	0.001710
2538.500000	0.001688
2538.250000	0.001677
2538.000000	0.001681
2537.750000	0.001699
2537.500000	0.001731

2537.250000	0.001771
2537.000000	0.001815
2536.750000	0.001854
2536.500000	0.001884
2536.250000	0.001900
2536.000000	0.001901
2535.750000	0.001890
2535.500000	0.001869
2535.250000	0.001845
2535.000000	0.001823
2534.750000	0.001804
2534.500000	0.001791
2534.250000	0.001783
2534.000000	0.001776
2533.750000	0.001770
2533.500000	0.001764
2533.250000	0.001759
2533.000000	0.001757
2532.750000	0.001761
2532.500000	0.001771
2532.250000	0.001787
2532.000000	0.001806
2531.750000	0.001826
2531.500000	0.001842
2531.250000	0.001855
2531.000000	0.001863

2530.750000	0.001867
2530.500000	0.001868
2530.250000	0.001866
2530.000000	0.001862
2529.750000	0.001856
2529.500000	0.001850
2529.250000	0.001843
2529.000000	0.001838
2528.750000	0.001835
2528.500000	0.001833
2528.250000	0.001833
2528.000000	0.001832
2527.750000	0.001827
2527.500000	0.001817
2527.250000	0.001801
2527.000000	0.001780
2526.750000	0.001758
2526.500000	0.001738
2526.250000	0.001724
2526.000000	0.001721
2525.750000	0.001728
2525.500000	0.001745
2525.250000	0.001767
2525.000000	0.001792
2524.750000	0.001814
2524.500000	0.001832

2524.250000	0.001844
2524.000000	0.001850
2523.750000	0.001851
2523.500000	0.001849
2523.250000	0.001846
2523.000000	0.001844
2522.750000	0.001843
2522.500000	0.001842
2522.250000	0.001841
2522.000000	0.001838
2521.750000	0.001834
2521.500000	0.001829
2521.250000	0.001824
2521.000000	0.001820
2520.750000	0.001818
2520.500000	0.001819
2520.250000	0.001821
2520.000000	0.001822
2519.750000	0.001820
2519.500000	0.001810
2519.250000	0.001794
2519.000000	0.001770
2518.750000	0.001741
2518.500000	0.001710
2518.250000	0.001682
2518.000000	0.001661

2517.750000	0.001648
2517.500000	0.001645
2517.250000	0.001652
2517.000000	0.001668
2516.750000	0.001689
2516.500000	0.001714
2516.250000	0.001740
2516.000000	0.001764
2515.750000	0.001784
2515.500000	0.001800
2515.250000	0.001810
2515.000000	0.001815
2514.750000	0.001817
2514.500000	0.001816
2514.250000	0.001815
2514.000000	0.001814
2513.750000	0.001812
2513.500000	0.001809
2513.250000	0.001803
2513.000000	0.001793
2512.750000	0.001780
2512.500000	0.001765
2512.250000	0.001750
2512.000000	0.001737
2511.750000	0.001728
2511.500000	0.001725

2511.250000	0.001726
2511.000000	0.001730
2510.750000	0.001736
2510.500000	0.001743
2510.250000	0.001750
2510.000000	0.001757
2509.750000	0.001766
2509.500000	0.001776
2509.250000	0.001786
2509.000000	0.001795
2508.750000	0.001800
2508.500000	0.001800
2508.250000	0.001794
2508.000000	0.001781
2507.750000	0.001765
2507.500000	0.001746
2507.250000	0.001727
2507.000000	0.001712
2506.750000	0.001700
2506.500000	0.001694
2506.250000	0.001692
2506.000000	0.001694
2505.750000	0.001701
2505.500000	0.001710
2505.250000	0.001721
2505.000000	0.001732

2504.750000	0.001743
2504.500000	0.001754
2504.250000	0.001765
2504.000000	0.001777
2503.750000	0.001790
2503.500000	0.001806
2503.250000	0.001822
2503.000000	0.001839
2502.750000	0.001854
2502.500000	0.001865
2502.250000	0.001870
2502.000000	0.001870
2501.750000	0.001864
2501.500000	0.001852
2501.250000	0.001837
2501.000000	0.001820
2500.750000	0.001803
2500.500000	0.001788
2500.250000	0.001775
2500.000000	0.001766
2499.750000	0.001762
2499.500000	0.001761
2499.250000	0.001762
2499.000000	0.001763
2498.750000	0.001760
2498.500000	0.001753

2498.250000	0.001742
2498.000000	0.001727
2497.750000	0.001714
2497.500000	0.001704
2497.250000	0.001702
2497.000000	0.001708
2496.750000	0.001722
2496.500000	0.001740
2496.250000	0.001759
2496.000000	0.001776
2495.750000	0.001786
2495.500000	0.001786
2495.250000	0.001776
2495.000000	0.001756
2494.750000	0.001726
2494.500000	0.001691
2494.250000	0.001656
2494.000000	0.001628
2493.750000	0.001612
2493.500000	0.001612
2493.250000	0.001628
2493.000000	0.001657
2492.750000	0.001692
2492.500000	0.001724
2492.250000	0.001748
2492.000000	0.001761

2491.750000	0.001765
2491.500000	0.001767
2491.250000	0.001772
2491.000000	0.001785
2490.750000	0.001809
2490.500000	0.001837
2490.250000	0.001865
2490.000000	0.001885
2489.750000	0.001890
2489.500000	0.001881
2489.250000	0.001860
2489.000000	0.001832
2488.750000	0.001803
2488.500000	0.001778
2488.250000	0.001759
2488.000000	0.001745
2487.750000	0.001735
2487.500000	0.001725
2487.250000	0.001716
2487.000000	0.001706
2486.750000	0.001698
2486.500000	0.001693
2486.250000	0.001692
2486.000000	0.001698
2485.750000	0.001709
2485.500000	0.001727

2485.250000	0.001748
2485.000000	0.001772
2484.750000	0.001797
2484.500000	0.001820
2484.250000	0.001838
2484.000000	0.001849
2483.750000	0.001853
2483.500000	0.001848
2483.250000	0.001837
2483.000000	0.001822
2482.750000	0.001804
2482.500000	0.001787
2482.250000	0.001771
2482.000000	0.001759
2481.750000	0.001749
2481.500000	0.001741
2481.250000	0.001737
2481.000000	0.001734
2480.750000	0.001732
2480.500000	0.001730
2480.250000	0.001726
2480.000000	0.001720
2479.750000	0.001713
2479.500000	0.001705
2479.250000	0.001698
2479.000000	0.001694

2478.750000	0.001693
2478.500000	0.001696
2478.250000	0.001702
2478.000000	0.001712
2477.750000	0.001724
2477.500000	0.001738
2477.250000	0.001754
2477.000000	0.001772
2476.750000	0.001790
2476.500000	0.001806
2476.250000	0.001816
2476.000000	0.001819
2475.750000	0.001814
2475.500000	0.001799
2475.250000	0.001778
2475.000000	0.001755
2474.750000	0.001733
2474.500000	0.001717
2474.250000	0.001708
2474.000000	0.001706
2473.750000	0.001710
2473.500000	0.001716
2473.250000	0.001724
2473.000000	0.001730
2472.750000	0.001734
2472.500000	0.001735

2472.250000	0.001735
2472.000000	0.001735
2471.750000	0.001735
2471.500000	0.001737
2471.250000	0.001742
2471.000000	0.001750
2470.750000	0.001761
2470.500000	0.001773
2470.250000	0.001785
2470.000000	0.001794
2469.750000	0.001798
2469.500000	0.001796
2469.250000	0.001789
2469.000000	0.001777
2468.750000	0.001764
2468.500000	0.001750
2468.250000	0.001740
2468.000000	0.001733
2467.750000	0.001730
2467.500000	0.001731
2467.250000	0.001734
2467.000000	0.001738
2466.750000	0.001741
2466.500000	0.001742
2466.250000	0.001740
2466.000000	0.001734

2465.750000	0.001726
2465.500000	0.001714
2465.250000	0.001702
2465.000000	0.001689
2464.750000	0.001675
2464.500000	0.001662
2464.250000	0.001649
2464.000000	0.001636
2463.750000	0.001626
2463.500000	0.001618
2463.250000	0.001616
2463.000000	0.001619
2462.750000	0.001629
2462.500000	0.001643
2462.250000	0.001660
2462.000000	0.001677
2461.750000	0.001691
2461.500000	0.001701
2461.250000	0.001708
2461.000000	0.001712
2460.750000	0.001715
2460.500000	0.001718
2460.250000	0.001723
2460.000000	0.001728
2459.750000	0.001733
2459.500000	0.001738

2459.250000	0.001741
2459.000000	0.001742
2458.750000	0.001742
2458.500000	0.001741
2458.250000	0.001740
2458.000000	0.001737
2457.750000	0.001735
2457.500000	0.001733
2457.250000	0.001733
2457.000000	0.001737
2456.750000	0.001745
2456.500000	0.001755
2456.250000	0.001768
2456.000000	0.001779
2455.750000	0.001786
2455.500000	0.001786
2455.250000	0.001779
2455.000000	0.001767
2454.750000	0.001751
2454.500000	0.001737
2454.250000	0.001726
2454.000000	0.001722
2453.750000	0.001725
2453.500000	0.001735
2453.250000	0.001748
2453.000000	0.001761

2452.750000	0.001772
2452.500000	0.001777
2452.250000	0.001777
2452.000000	0.001770
2451.750000	0.001758
2451.500000	0.001744
2451.250000	0.001731
2451.000000	0.001721
2450.750000	0.001716
2450.500000	0.001716
2450.250000	0.001721
2450.000000	0.001727
2449.750000	0.001733
2449.500000	0.001737
2449.250000	0.001740
2449.000000	0.001741
2448.750000	0.001742
2448.500000	0.001744
2448.250000	0.001749
2448.000000	0.001754
2447.750000	0.001758
2447.500000	0.001760
2447.250000	0.001756
2447.000000	0.001748
2446.750000	0.001736
2446.500000	0.001723

2446.250000	0.001712
2446.000000	0.001706
2445.750000	0.001704
2445.500000	0.001707
2445.250000	0.001712
2445.000000	0.001715
2444.750000	0.001714
2444.500000	0.001706
2444.250000	0.001691
2444.000000	0.001673
2443.750000	0.001653
2443.500000	0.001635
2443.250000	0.001622
2443.000000	0.001615
2442.750000	0.001614
2442.500000	0.001620
2442.250000	0.001629
2442.000000	0.001640
2441.750000	0.001652
2441.500000	0.001664
2441.250000	0.001676
2441.000000	0.001685
2440.750000	0.001691
2440.500000	0.001693
2440.250000	0.001690
2440.000000	0.001683

2439.750000	0.001673
2439.500000	0.001662
2439.250000	0.001653
2439.000000	0.001648
2438.750000	0.001650
2438.500000	0.001659
2438.250000	0.001672
2438.000000	0.001687
2437.750000	0.001702
2437.500000	0.001714
2437.250000	0.001721
2437.000000	0.001722
2436.750000	0.001718
2436.500000	0.001712
2436.250000	0.001707
2436.000000	0.001705
2435.750000	0.001709
2435.500000	0.001720
2435.250000	0.001737
2435.000000	0.001757
2434.750000	0.001776
2434.500000	0.001788
2434.250000	0.001791
2434.000000	0.001783
2433.750000	0.001765
2433.500000	0.001739

2433.250000	0.001710
2433.000000	0.001685
2432.750000	0.001665
2432.500000	0.001655
2432.250000	0.001652
2432.000000	0.001657
2431.750000	0.001667
2431.500000	0.001679
2431.250000	0.001692
2431.000000	0.001705
2430.750000	0.001716
2430.500000	0.001723
2430.250000	0.001726
2430.000000	0.001723
2429.750000	0.001713
2429.500000	0.001699
2429.250000	0.001680
2429.000000	0.001659
2428.750000	0.001638
2428.500000	0.001619
2428.250000	0.001604
2428.000000	0.001593
2427.750000	0.001589
2427.500000	0.001591
2427.250000	0.001600
2427.000000	0.001614

2426.750000	0.001632
2426.500000	0.001650
2426.250000	0.001665
2426.000000	0.001674
2425.750000	0.001674
2425.500000	0.001667
2425.250000	0.001655
2425.000000	0.001642
2424.750000	0.001630
2424.500000	0.001623
2424.250000	0.001621
2424.000000	0.001622
2423.750000	0.001623
2423.500000	0.001623
2423.250000	0.001621
2423.000000	0.001615
2422.750000	0.001608
2422.500000	0.001600
2422.250000	0.001592
2422.000000	0.001583
2421.750000	0.001573
2421.500000	0.001562
2421.250000	0.001550
2421.000000	0.001538
2420.750000	0.001527
2420.500000	0.001519

2420.250000	0.001513
2420.000000	0.001512
2419.750000	0.001514
2419.500000	0.001520
2419.250000	0.001529
2419.000000	0.001541
2418.750000	0.001555
2418.500000	0.001569
2418.250000	0.001583
2418.000000	0.001594
2417.750000	0.001601
2417.500000	0.001603
2417.250000	0.001602
2417.000000	0.001600
2416.750000	0.001599
2416.500000	0.001602
2416.250000	0.001609
2416.000000	0.001621
2415.750000	0.001634
2415.500000	0.001648
2415.250000	0.001659
2415.000000	0.001666
2414.750000	0.001666
2414.500000	0.001661
2414.250000	0.001650
2414.000000	0.001635

2413.750000	0.001618
2413.500000	0.001601
2413.250000	0.001589
2413.000000	0.001581
2412.750000	0.001581
2412.500000	0.001586
2412.250000	0.001595
2412.000000	0.001604
2411.750000	0.001612
2411.500000	0.001616
2411.250000	0.001615
2411.000000	0.001610
2410.750000	0.001602
2410.500000	0.001595
2410.250000	0.001588
2410.000000	0.001583
2409.750000	0.001580
2409.500000	0.001579
2409.250000	0.001578
2409.000000	0.001578
2408.750000	0.001578
2408.500000	0.001579
2408.250000	0.001580
2408.000000	0.001579
2407.750000	0.001577
2407.500000	0.001572

2407.250000	0.001566
2407.000000	0.001560
2406.750000	0.001556
2406.500000	0.001556
2406.250000	0.001561
2406.000000	0.001571
2405.750000	0.001585
2405.500000	0.001602
2405.250000	0.001618
2405.000000	0.001633
2404.750000	0.001645
2404.500000	0.001652
2404.250000	0.001654
2404.000000	0.001649
2403.750000	0.001637
2403.500000	0.001620
2403.250000	0.001600
2403.000000	0.001580
2402.750000	0.001563
2402.500000	0.001552
2402.250000	0.001549
2402.000000	0.001552
2401.750000	0.001558
2401.500000	0.001566
2401.250000	0.001572
2401.000000	0.001575

2400.750000	0.001577
2400.500000	0.001577
2400.250000	0.001579
2400.000000	0.001583
2399.750000	0.001589
2399.500000	0.001595
2399.250000	0.001599
2399.000000	0.001600
2398.750000	0.001596
2398.500000	0.001588
2398.250000	0.001577
2398.000000	0.001563
2397.750000	0.001549
2397.500000	0.001535
2397.250000	0.001520
2397.000000	0.001506
2396.750000	0.001492
2396.500000	0.001479
2396.250000	0.001469
2396.000000	0.001464
2395.750000	0.001465
2395.500000	0.001473
2395.250000	0.001488
2395.000000	0.001507
2394.750000	0.001529
2394.500000	0.001551

2394.250000	0.001571
2394.000000	0.001588
2393.750000	0.001600
2393.500000	0.001608
2393.250000	0.001612
2393.000000	0.001612
2392.750000	0.001608
2392.500000	0.001599
2392.250000	0.001585
2392.000000	0.001567
2391.750000	0.001547
2391.500000	0.001525
2391.250000	0.001507
2391.000000	0.001495
2390.750000	0.001491
2390.500000	0.001494
2390.250000	0.001503
2390.000000	0.001513
2389.750000	0.001521
2389.500000	0.001524
2389.250000	0.001521
2389.000000	0.001513
2388.750000	0.001504
2388.500000	0.001498
2388.250000	0.001497
2388.000000	0.001502

2387.750000	0.001513
2387.500000	0.001527
2387.250000	0.001541
2387.000000	0.001552
2386.750000	0.001556
2386.500000	0.001554
2386.250000	0.001547
2386.000000	0.001535
2385.750000	0.001523
2385.500000	0.001514
2385.250000	0.001510
2385.000000	0.001513
2384.750000	0.001523
2384.500000	0.001536
2384.250000	0.001549
2384.000000	0.001557
2383.750000	0.001557
2383.500000	0.001547
2383.250000	0.001530
2383.000000	0.001511
2382.750000	0.001493
2382.500000	0.001484
2382.250000	0.001486
2382.000000	0.001498
2381.750000	0.001519
2381.500000	0.001543

2381.250000	0.001563
2381.000000	0.001576
2380.750000	0.001579
2380.500000	0.001573
2380.250000	0.001558
2380.000000	0.001538
2379.750000	0.001515
2379.500000	0.001490
2379.250000	0.001465
2379.000000	0.001440
2378.750000	0.001415
2378.500000	0.001393
2378.250000	0.001375
2378.000000	0.001364
2377.750000	0.001361
2377.500000	0.001367
2377.250000	0.001380
2377.000000	0.001397
2376.750000	0.001416
2376.500000	0.001432
2376.250000	0.001444
2376.000000	0.001450
2375.750000	0.001451
2375.500000	0.001449
2375.250000	0.001446
2375.000000	0.001444

2374.750000	0.001445
2374.500000	0.001450
2374.250000	0.001457
2374.000000	0.001466
2373.750000	0.001475
2373.500000	0.001482
2373.250000	0.001486
2373.000000	0.001488
2372.750000	0.001486
2372.500000	0.001480
2372.250000	0.001471
2372.000000	0.001459
2371.750000	0.001447
2371.500000	0.001437
2371.250000	0.001432
2371.000000	0.001432
2370.750000	0.001438
2370.500000	0.001443
2370.250000	0.001441
2370.000000	0.001424
2369.750000	0.001387
2369.500000	0.001332
2369.250000	0.001267
2369.000000	0.001208
2368.750000	0.001173
2368.500000	0.001175

2368.250000	0.001223
2368.000000	0.001312
2367.750000	0.001428
2367.500000	0.001552
2367.250000	0.001660
2367.000000	0.001734
2366.750000	0.001764
2366.500000	0.001751
2366.250000	0.001704
2366.000000	0.001638
2365.750000	0.001569
2365.500000	0.001511
2365.250000	0.001472
2365.000000	0.001457
2364.750000	0.001462
2364.500000	0.001482
2364.250000	0.001507
2364.000000	0.001527
2363.750000	0.001537
2363.500000	0.001532
2363.250000	0.001514
2363.000000	0.001490
2362.750000	0.001470
2362.500000	0.001464
2362.250000	0.001477
2362.000000	0.001511

2361.750000	0.001559
2361.500000	0.001611
2361.250000	0.001656
2361.000000	0.001684
2360.750000	0.001688
2360.500000	0.001670
2360.250000	0.001635
2360.000000	0.001589
2359.750000	0.001541
2359.500000	0.001498
2359.250000	0.001464
2359.000000	0.001441
2358.750000	0.001428
2358.500000	0.001423
2358.250000	0.001423
2358.000000	0.001424
2357.750000	0.001424
2357.500000	0.001423
2357.250000	0.001420
2357.000000	0.001418
2356.750000	0.001421
2356.500000	0.001431
2356.250000	0.001450
2356.000000	0.001476
2355.750000	0.001503
2355.500000	0.001527

2355.250000	0.001544
2355.000000	0.001552
2354.750000	0.001550
2354.500000	0.001541
2354.250000	0.001528
2354.000000	0.001513
2353.750000	0.001499
2353.500000	0.001486
2353.250000	0.001474
2353.000000	0.001463
2352.750000	0.001456
2352.500000	0.001454
2352.250000	0.001459
2352.000000	0.001474
2351.750000	0.001496
2351.500000	0.001522
2351.250000	0.001546
2351.000000	0.001561
2350.750000	0.001564
2350.500000	0.001552
2350.250000	0.001529
2350.000000	0.001498
2349.750000	0.001466
2349.500000	0.001439
2349.250000	0.001422
2349.000000	0.001416

2348.750000	0.001421
2348.500000	0.001436
2348.250000	0.001456
2348.000000	0.001480
2347.750000	0.001504
2347.500000	0.001523
2347.250000	0.001536
2347.000000	0.001541
2346.750000	0.001540
2346.500000	0.001534
2346.250000	0.001527
2346.000000	0.001520
2345.750000	0.001515
2345.500000	0.001512
2345.250000	0.001511
2345.000000	0.001512
2344.750000	0.001517
2344.500000	0.001526
2344.250000	0.001539
2344.000000	0.001553
2343.750000	0.001567
2343.500000	0.001577
2343.250000	0.001582
2343.000000	0.001580
2342.750000	0.001573
2342.500000	0.001564

2342.250000	0.001555
2342.000000	0.001550
2341.750000	0.001549
2341.500000	0.001552
2341.250000	0.001557
2341.000000	0.001563
2340.750000	0.001566
2340.500000	0.001566
2340.250000	0.001562
2340.000000	0.001556
2339.750000	0.001551
2339.500000	0.001548
2339.250000	0.001550
2339.000000	0.001557
2338.750000	0.001570
2338.500000	0.001587
2338.250000	0.001609
2338.000000	0.001634
2337.750000	0.001658
2337.500000	0.001680
2337.250000	0.001694
2337.000000	0.001699
2336.750000	0.001693
2336.500000	0.001679
2336.250000	0.001660
2336.000000	0.001641

2335.750000	0.001628
2335.500000	0.001622
2335.250000	0.001623
2335.000000	0.001632
2334.750000	0.001645
2334.500000	0.001661
2334.250000	0.001679
2334.000000	0.001698
2333.750000	0.001717
2333.500000	0.001734
2333.250000	0.001746
2333.000000	0.001750
2332.750000	0.001745
2332.500000	0.001732
2332.250000	0.001714
2332.000000	0.001697
2331.750000	0.001684
2331.500000	0.001679
2331.250000	0.001683
2331.000000	0.001696
2330.750000	0.001716
2330.500000	0.001738
2330.250000	0.001761
2330.000000	0.001782
2329.750000	0.001800
2329.500000	0.001813

2329.250000	0.001819
2329.000000	0.001818
2328.750000	0.001810
2328.500000	0.001798
2328.250000	0.001785
2328.000000	0.001776
2327.750000	0.001775
2327.500000	0.001788
2327.250000	0.001817
2327.000000	0.001862
2326.750000	0.001923
2326.500000	0.001995
2326.250000	0.002073
2326.000000	0.002151
2325.750000	0.002223
2325.500000	0.002281
2325.250000	0.002324
2325.000000	0.002348
2324.750000	0.002355
2324.500000	0.002348
2324.250000	0.002331
2324.000000	0.002308
2323.750000	0.002283
2323.500000	0.002259
2323.250000	0.002236
2323.000000	0.002214

2322.750000	0.002193
2322.500000	0.002171
2322.250000	0.002148
2322.000000	0.002123
2321.750000	0.002098
2321.500000	0.002073
2321.250000	0.002052
2321.000000	0.002036
2320.750000	0.002028
2320.500000	0.002027
2320.250000	0.002031
2320.000000	0.002037
2319.750000	0.002042
2319.500000	0.002044
2319.250000	0.002040
2319.000000	0.002032
2318.750000	0.002020
2318.500000	0.002007
2318.250000	0.001994
2318.000000	0.001984
2317.750000	0.001978
2317.500000	0.001979
2317.250000	0.001987
2317.000000	0.002002
2316.750000	0.002019
2316.500000	0.002037

2316.250000	0.002050
2316.000000	0.002054
2315.750000	0.002046
2315.500000	0.002026
2315.250000	0.001995
2315.000000	0.001956
2314.750000	0.001915
2314.500000	0.001877
2314.250000	0.001846
2314.000000	0.001824
2313.750000	0.001814
2313.500000	0.001814
2313.250000	0.001825
2313.000000	0.001844
2312.750000	0.001867
2312.500000	0.001891
2312.250000	0.001912
2312.000000	0.001927
2311.750000	0.001935
2311.500000	0.001933
2311.250000	0.001922
2311.000000	0.001903
2310.750000	0.001878
2310.500000	0.001851
2310.250000	0.001823
2310.000000	0.001797

2309.750000	0.001774
2309.500000	0.001757
2309.250000	0.001746
2309.000000	0.001743
2308.750000	0.001748
2308.500000	0.001760
2308.250000	0.001775
2308.000000	0.001792
2307.750000	0.001807
2307.500000	0.001819
2307.250000	0.001826
2307.000000	0.001829
2306.750000	0.001829
2306.500000	0.001827
2306.250000	0.001826
2306.000000	0.001825
2305.750000	0.001825
2305.500000	0.001826
2305.250000	0.001826
2305.000000	0.001825
2304.750000	0.001823
2304.500000	0.001821
2304.250000	0.001821
2304.000000	0.001824
2303.750000	0.001832
2303.500000	0.001845

2303.250000	0.001862
2303.000000	0.001881
2302.750000	0.001897
2302.500000	0.001907
2302.250000	0.001908
2302.000000	0.001898
2301.750000	0.001880
2301.500000	0.001854
2301.250000	0.001827
2301.000000	0.001801
2300.750000	0.001780
2300.500000	0.001767
2300.250000	0.001764
2300.000000	0.001769
2299.750000	0.001782
2299.500000	0.001801
2299.250000	0.001824
2299.000000	0.001847
2298.750000	0.001868
2298.500000	0.001883
2298.250000	0.001890
2298.000000	0.001890
2297.750000	0.001882
2297.500000	0.001872
2297.250000	0.001863
2297.000000	0.001859

2296.750000	0.001861
2296.500000	0.001870
2296.250000	0.001883
2296.000000	0.001897
2295.750000	0.001907
2295.500000	0.001911
2295.250000	0.001910
2295.000000	0.001905
2294.750000	0.001900
2294.500000	0.001896
2294.250000	0.001896
2294.000000	0.001903
2293.750000	0.001916
2293.500000	0.001934
2293.250000	0.001957
2293.000000	0.001983
2292.750000	0.002008
2292.500000	0.002032
2292.250000	0.002051
2292.000000	0.002064
2291.750000	0.002072
2291.500000	0.002075
2291.250000	0.002074
2291.000000	0.002072
2290.750000	0.002069
2290.500000	0.002068

2290.250000	0.002067
2290.000000	0.002065
2289.750000	0.002063
2289.500000	0.002059
2289.250000	0.002053
2289.000000	0.002047
2288.750000	0.002042
2288.500000	0.002038
2288.250000	0.002037
2288.000000	0.002038
2287.750000	0.002039
2287.500000	0.002040
2287.250000	0.002039
2287.000000	0.002035
2286.750000	0.002028
2286.500000	0.002019
2286.250000	0.002010
2286.000000	0.002004
2285.750000	0.002003
2285.500000	0.002010
2285.250000	0.002026
2285.000000	0.002051
2284.750000	0.002079
2284.500000	0.002104
2284.250000	0.002120
2284.000000	0.002119

2283.750000	0.002099
2283.500000	0.002059
2283.250000	0.002006
2283.000000	0.001946
2282.750000	0.001889
2282.500000	0.001842
2282.250000	0.001810
2282.000000	0.001796
2281.750000	0.001796
2281.500000	0.001807
2281.250000	0.001825
2281.000000	0.001845
2280.750000	0.001865
2280.500000	0.001884
2280.250000	0.001901
2280.000000	0.001915
2279.750000	0.001928
2279.500000	0.001940
2279.250000	0.001950
2279.000000	0.001958
2278.750000	0.001964
2278.500000	0.001968
2278.250000	0.001970
2278.000000	0.001970
2277.750000	0.001967
2277.500000	0.001963

2277.250000	0.001958
2277.000000	0.001955
2276.750000	0.001953
2276.500000	0.001953
2276.250000	0.001954
2276.000000	0.001954
2275.750000	0.001951
2275.500000	0.001944
2275.250000	0.001932
2275.000000	0.001917
2274.750000	0.001898
2274.500000	0.001879
2274.250000	0.001860
2274.000000	0.001844
2273.750000	0.001830
2273.500000	0.001821
2273.250000	0.001815
2273.000000	0.001815
2272.750000	0.001821
2272.500000	0.001833
2272.250000	0.001850
2272.000000	0.001872
2271.750000	0.001894
2271.500000	0.001915
2271.250000	0.001929
2271.000000	0.001934

2270.750000	0.001927
2270.500000	0.001908
2270.250000	0.001877
2270.000000	0.001836
2269.750000	0.001789
2269.500000	0.001740
2269.250000	0.001694
2269.000000	0.001655
2268.750000	0.001627
2268.500000	0.001613
2268.250000	0.001614
2268.000000	0.001629
2267.750000	0.001656
2267.500000	0.001692
2267.250000	0.001735
2267.000000	0.001779
2266.750000	0.001822
2266.500000	0.001857
2266.250000	0.001881
2266.000000	0.001892
2265.750000	0.001888
2265.500000	0.001872
2265.250000	0.001846
2265.000000	0.001816
2264.750000	0.001785
2264.500000	0.001758

2264.250000	0.001735
2264.000000	0.001716
2263.750000	0.001701
2263.500000	0.001687
2263.250000	0.001675
2263.000000	0.001664
2262.750000	0.001656
2262.500000	0.001650
2262.250000	0.001650
2262.000000	0.001653
2261.750000	0.001662
2261.500000	0.001675
2261.250000	0.001694
2261.000000	0.001717
2260.750000	0.001744
2260.500000	0.001774
2260.250000	0.001805
2260.000000	0.001835
2259.750000	0.001862
2259.500000	0.001885
2259.250000	0.001903
2259.000000	0.001917
2258.750000	0.001927
2258.500000	0.001932
2258.250000	0.001932
2258.000000	0.001926

2257.750000	0.001911
2257.500000	0.001888
2257.250000	0.001860
2257.000000	0.001829
2256.750000	0.001801
2256.500000	0.001779
2256.250000	0.001766
2256.000000	0.001761
2255.750000	0.001763
2255.500000	0.001767
2255.250000	0.001771
2255.000000	0.001773
2254.750000	0.001774
2254.500000	0.001776
2254.250000	0.001781
2254.000000	0.001791
2253.750000	0.001804
2253.500000	0.001820
2253.250000	0.001835
2253.000000	0.001846
2252.750000	0.001849
2252.500000	0.001845
2252.250000	0.001835
2252.000000	0.001823
2251.750000	0.001812
2251.500000	0.001806

2251.250000	0.001807
2251.000000	0.001814
2250.750000	0.001822
2250.500000	0.001829
2250.250000	0.001829
2250.000000	0.001820
2249.750000	0.001801
2249.500000	0.001775
2249.250000	0.001747
2249.000000	0.001724
2248.750000	0.001709
2248.500000	0.001705
2248.250000	0.001714
2248.000000	0.001732
2247.750000	0.001755
2247.500000	0.001776
2247.250000	0.001792
2247.000000	0.001796
2246.750000	0.001787
2246.500000	0.001765
2246.250000	0.001733
2246.000000	0.001699
2245.750000	0.001670
2245.500000	0.001656
2245.250000	0.001665
2245.000000	0.001701

2244.750000	0.001762
2244.500000	0.001841
2244.250000	0.001927
2244.000000	0.002006
2243.750000	0.002068
2243.500000	0.002106
2243.250000	0.002118
2243.000000	0.002111
2242.750000	0.002092
2242.500000	0.002070
2242.250000	0.002051
2242.000000	0.002036
2241.750000	0.002024
2241.500000	0.002011
2241.250000	0.001993
2241.000000	0.001966
2240.750000	0.001932
2240.500000	0.001894
2240.250000	0.001858
2240.000000	0.001832
2239.750000	0.001819
2239.500000	0.001823
2239.250000	0.001840
2239.000000	0.001866
2238.750000	0.001895
2238.500000	0.001919

2238.250000	0.001933
2238.000000	0.001933
2237.750000	0.001920
2237.500000	0.001899
2237.250000	0.001876
2237.000000	0.001857
2236.750000	0.001847
2236.500000	0.001848
2236.250000	0.001857
2236.000000	0.001871
2235.750000	0.001883
2235.500000	0.001888
2235.250000	0.001885
2235.000000	0.001874
2234.750000	0.001857
2234.500000	0.001841
2234.250000	0.001828
2234.000000	0.001823
2233.750000	0.001825
2233.500000	0.001834
2233.250000	0.001846
2233.000000	0.001858
2232.750000	0.001867
2232.500000	0.001870
2232.250000	0.001866
2232.000000	0.001856

2231.750000	0.001841
2231.500000	0.001823
2231.250000	0.001804
2231.000000	0.001786
2230.750000	0.001770
2230.500000	0.001758
2230.250000	0.001750
2230.000000	0.001748
2229.750000	0.001752
2229.500000	0.001763
2229.250000	0.001781
2229.000000	0.001804
2228.750000	0.001829
2228.500000	0.001852
2228.250000	0.001868
2228.000000	0.001872
2227.750000	0.001865
2227.500000	0.001847
2227.250000	0.001822
2227.000000	0.001794
2226.750000	0.001768
2226.500000	0.001745
2226.250000	0.001727
2226.000000	0.001713
2225.750000	0.001701
2225.500000	0.001692

2225.250000	0.001686
2225.000000	0.001685
2224.750000	0.001690
2224.500000	0.001701
2224.250000	0.001717
2224.000000	0.001734
2223.750000	0.001750
2223.500000	0.001764
2223.250000	0.001775
2223.000000	0.001785
2222.750000	0.001794
2222.500000	0.001805
2222.250000	0.001816
2222.000000	0.001825
2221.750000	0.001832
2221.500000	0.001835
2221.250000	0.001836
2221.000000	0.001840
2220.750000	0.001851
2220.500000	0.001872
2220.250000	0.001903
2220.000000	0.001941
2219.750000	0.001979
2219.500000	0.002010
2219.250000	0.002027
2219.000000	0.002027

2218.750000	0.002008
2218.500000	0.001973
2218.250000	0.001927
2218.000000	0.001877
2217.750000	0.001832
2217.500000	0.001796
2217.250000	0.001775
2217.000000	0.001771
2216.750000	0.001781
2216.500000	0.001801
2216.250000	0.001824
2216.000000	0.001842
2215.750000	0.001852
2215.500000	0.001851
2215.250000	0.001843
2215.000000	0.001833
2214.750000	0.001824
2214.500000	0.001821
2214.250000	0.001821
2214.000000	0.001822
2213.750000	0.001820
2213.500000	0.001813
2213.250000	0.001802
2213.000000	0.001792
2212.750000	0.001788
2212.500000	0.001796

2212.250000	0.001816
2212.000000	0.001842
2211.750000	0.001866
2211.500000	0.001880
2211.250000	0.001879
2211.000000	0.001862
2210.750000	0.001834
2210.500000	0.001805
2210.250000	0.001785
2210.000000	0.001777
2209.750000	0.001784
2209.500000	0.001800
2209.250000	0.001818
2209.000000	0.001832
2208.750000	0.001840
2208.500000	0.001842
2208.250000	0.001842
2208.000000	0.001846
2207.750000	0.001857
2207.500000	0.001877
2207.250000	0.001905
2207.000000	0.001934
2206.750000	0.001959
2206.500000	0.001973
2206.250000	0.001971
2206.000000	0.001953

2205.750000	0.001919
2205.500000	0.001875
2205.250000	0.001827
2205.000000	0.001782
2204.750000	0.001749
2204.500000	0.001735
2204.250000	0.001744
2204.000000	0.001777
2203.750000	0.001831
2203.500000	0.001897
2203.250000	0.001966
2203.000000	0.002028
2202.750000	0.002073
2202.500000	0.002094
2202.250000	0.002089
2202.000000	0.002058
2201.750000	0.002007
2201.500000	0.001946
2201.250000	0.001882
2201.000000	0.001826
2200.750000	0.001784
2200.500000	0.001759
2200.250000	0.001752
2200.000000	0.001757
2199.750000	0.001772
2199.500000	0.001791

2199.250000	0.001811
2199.000000	0.001828
2198.750000	0.001842
2198.500000	0.001852
2198.250000	0.001856
2198.000000	0.001856
2197.750000	0.001850
2197.500000	0.001840
2197.250000	0.001828
2197.000000	0.001815
2196.750000	0.001803
2196.500000	0.001791
2196.250000	0.001777
2196.000000	0.001760
2195.750000	0.001739
2195.500000	0.001715
2195.250000	0.001694
2195.000000	0.001681
2194.750000	0.001685
2194.500000	0.001710
2194.250000	0.001756
2194.000000	0.001817
2193.750000	0.001885
2193.500000	0.001951
2193.250000	0.002009
2193.000000	0.002056

2192.750000	0.002092
2192.500000	0.002120
2192.250000	0.002140
2192.000000	0.002153
2191.750000	0.002152
2191.500000	0.002136
2191.250000	0.002101
2191.000000	0.002050
2190.750000	0.001994
2190.500000	0.001946
2190.250000	0.001919
2190.000000	0.001924
2189.750000	0.001962
2189.500000	0.002025
2189.250000	0.002101
2189.000000	0.002171
2188.750000	0.002221
2188.500000	0.002244
2188.250000	0.002237
2188.000000	0.002208
2187.750000	0.002168
2187.500000	0.002129
2187.250000	0.002098
2187.000000	0.002080
2186.750000	0.002074
2186.500000	0.002075

2186.250000	0.002079
2186.000000	0.002083
2185.750000	0.002086
2185.500000	0.002093
2185.250000	0.002105
2185.000000	0.002126
2184.750000	0.002155
2184.500000	0.002190
2184.250000	0.002224
2184.000000	0.002251
2183.750000	0.002265
2183.500000	0.002263
2183.250000	0.002246
2183.000000	0.002216
2182.750000	0.002180
2182.500000	0.002141
2182.250000	0.002103
2182.000000	0.002066
2181.750000	0.002031
2181.500000	0.001994
2181.250000	0.001956
2181.000000	0.001914
2180.750000	0.001871
2180.500000	0.001828
2180.250000	0.001790
2180.000000	0.001759

2179.750000	0.001741
2179.500000	0.001737
2179.250000	0.001751
2179.000000	0.001781
2178.750000	0.001823
2178.500000	0.001872
2178.250000	0.001921
2178.000000	0.001961
2177.750000	0.001988
2177.500000	0.002000
2177.250000	0.002000
2177.000000	0.001991
2176.750000	0.001979
2176.500000	0.001966
2176.250000	0.001954
2176.000000	0.001938
2175.750000	0.001915
2175.500000	0.001880
2175.250000	0.001835
2175.000000	0.001783
2174.750000	0.001734
2174.500000	0.001697
2174.250000	0.001681
2174.000000	0.001688
2173.750000	0.001718
2173.500000	0.001764

2173.250000	0.001815
2173.000000	0.001862
2172.750000	0.001897
2172.500000	0.001916
2172.250000	0.001918
2172.000000	0.001907
2171.750000	0.001889
2171.500000	0.001869
2171.250000	0.001853
2171.000000	0.001843
2170.750000	0.001842
2170.500000	0.001848
2170.250000	0.001863
2170.000000	0.001889
2169.750000	0.001926
2169.500000	0.001978
2169.250000	0.002045
2169.000000	0.002125
2168.750000	0.002209
2168.500000	0.002289
2168.250000	0.002350
2168.000000	0.002385
2167.750000	0.002388
2167.500000	0.002363
2167.250000	0.002323
2167.000000	0.002282

2166.750000	0.002256
2166.500000	0.002253
2166.250000	0.002275
2166.000000	0.002314
2165.750000	0.002357
2165.500000	0.002393
2165.250000	0.002411
2165.000000	0.002411
2164.750000	0.002397
2164.500000	0.002379
2164.250000	0.002368
2164.000000	0.002372
2163.750000	0.002395
2163.500000	0.002437
2163.250000	0.002498
2163.000000	0.002575
2162.750000	0.002664
2162.500000	0.002763
2162.250000	0.002863
2162.000000	0.002957
2161.750000	0.003032
2161.500000	0.003077
2161.250000	0.003081
2161.000000	0.003036
2160.750000	0.002939
2160.500000	0.002790

2160.250000	0.002597
2160.000000	0.002366
2159.750000	0.002110
2159.500000	0.001840
2159.250000	0.001570
2159.000000	0.001313
2158.750000	0.001082
2158.500000	0.000885
2158.250000	0.000728
2158.000000	0.000612
2157.750000	0.000535
2157.500000	0.000498
2157.250000	0.000498
2157.000000	0.000536
2156.750000	0.000607
2156.500000	0.000705
2156.250000	0.000816
2156.000000	0.000926
2155.750000	0.001018
2155.500000	0.001081
2155.250000	0.001109
2155.000000	0.001109
2154.750000	0.001090
2154.500000	0.001068
2154.250000	0.001054
2154.000000	0.001057

2153.750000	0.001076
2153.500000	0.001105
2153.250000	0.001137
2153.000000	0.001165
2152.750000	0.001184
2152.500000	0.001193
2152.250000	0.001194
2152.000000	0.001192
2151.750000	0.001190
2151.500000	0.001195
2151.250000	0.001209
2151.000000	0.001237
2150.750000	0.001280
2150.500000	0.001339
2150.250000	0.001408
2150.000000	0.001482
2149.750000	0.001551
2149.500000	0.001608
2149.250000	0.001645
2149.000000	0.001659
2148.750000	0.001654
2148.500000	0.001633
2148.250000	0.001605
2148.000000	0.001574
2147.750000	0.001547
2147.500000	0.001523

2147.250000	0.001503
2147.000000	0.001486
2146.750000	0.001470
2146.500000	0.001452
2146.250000	0.001433
2146.000000	0.001410
2145.750000	0.001381
2145.500000	0.001347
2145.250000	0.001307
2145.000000	0.001268
2144.750000	0.001234
2144.500000	0.001213
2144.250000	0.001211
2144.000000	0.001231
2143.750000	0.001269
2143.500000	0.001319
2143.250000	0.001368
2143.000000	0.001405
2142.750000	0.001422
2142.500000	0.001413
2142.250000	0.001381
2142.000000	0.001333
2141.750000	0.001279
2141.500000	0.001230
2141.250000	0.001193
2141.000000	0.001173

2140.750000	0.001171
2140.500000	0.001183
2140.250000	0.001203
2140.000000	0.001225
2139.750000	0.001244
2139.500000	0.001257
2139.250000	0.001265
2139.000000	0.001270
2138.750000	0.001276
2138.500000	0.001287
2138.250000	0.001305
2138.000000	0.001332
2137.750000	0.001364
2137.500000	0.001398
2137.250000	0.001427
2137.000000	0.001444
2136.750000	0.001444
2136.500000	0.001422
2136.250000	0.001378
2136.000000	0.001315
2135.750000	0.001241
2135.500000	0.001168
2135.250000	0.001104
2135.000000	0.001060
2134.750000	0.001040
2134.500000	0.001044

2134.250000	0.001067
2134.000000	0.001099
2133.750000	0.001130
2133.500000	0.001152
2133.250000	0.001158
2133.000000	0.001149
2132.750000	0.001129
2132.500000	0.001106
2132.250000	0.001088
2132.000000	0.001082
2131.750000	0.001092
2131.500000	0.001118
2131.250000	0.001156
2131.000000	0.001199
2130.750000	0.001241
2130.500000	0.001273
2130.250000	0.001292
2130.000000	0.001297
2129.750000	0.001291
2129.500000	0.001280
2129.250000	0.001269
2129.000000	0.001264
2128.750000	0.001265
2128.500000	0.001272
2128.250000	0.001279
2128.000000	0.001281

2127.750000	0.001273
2127.500000	0.001255
2127.250000	0.001230
2127.000000	0.001206
2126.750000	0.001189
2126.500000	0.001186
2126.250000	0.001200
2126.000000	0.001231
2125.750000	0.001275
2125.500000	0.001326
2125.250000	0.001377
2125.000000	0.001423
2124.750000	0.001459
2124.500000	0.001482
2124.250000	0.001493
2124.000000	0.001492
2123.750000	0.001483
2123.500000	0.001470
2123.250000	0.001458
2123.000000	0.001453
2122.750000	0.001458
2122.500000	0.001471
2122.250000	0.001488
2122.000000	0.001502
2121.750000	0.001502
2121.500000	0.001483

2121.250000	0.001445
2121.000000	0.001393
2120.750000	0.001340
2120.500000	0.001299
2120.250000	0.001282
2120.000000	0.001298
2119.750000	0.001346
2119.500000	0.001417
2119.250000	0.001499
2119.000000	0.001576
2118.750000	0.001637
2118.500000	0.001676
2118.250000	0.001692
2118.000000	0.001690
2117.750000	0.001676
2117.500000	0.001657
2117.250000	0.001639
2117.000000	0.001624
2116.750000	0.001611
2116.500000	0.001599
2116.250000	0.001587
2116.000000	0.001574
2115.750000	0.001561
2115.500000	0.001552
2115.250000	0.001549
2115.000000	0.001554

2114.750000	0.001569
2114.500000	0.001592
2114.250000	0.001620
2114.000000	0.001648
2113.750000	0.001672
2113.500000	0.001687
2113.250000	0.001689
2113.000000	0.001678
2112.750000	0.001653
2112.500000	0.001618
2112.250000	0.001575
2112.000000	0.001531
2111.750000	0.001491
2111.500000	0.001460
2111.250000	0.001441
2111.000000	0.001436
2110.750000	0.001442
2110.500000	0.001457
2110.250000	0.001474
2110.000000	0.001491
2109.750000	0.001504
2109.500000	0.001514
2109.250000	0.001522
2109.000000	0.001528
2108.750000	0.001532
2108.500000	0.001533

2108.250000	0.001527
2108.000000	0.001513
2107.750000	0.001491
2107.500000	0.001464
2107.250000	0.001440
2107.000000	0.001428
2106.750000	0.001437
2106.500000	0.001468
2106.250000	0.001520
2106.000000	0.001583
2105.750000	0.001645
2105.500000	0.001693
2105.250000	0.001720
2105.000000	0.001722
2104.750000	0.001704
2104.500000	0.001673
2104.250000	0.001636
2104.000000	0.001600
2103.750000	0.001569
2103.500000	0.001544
2103.250000	0.001527
2103.000000	0.001518
2102.750000	0.001519
2102.500000	0.001531
2102.250000	0.001552
2102.000000	0.001582

2101.750000	0.001614
2101.500000	0.001641
2101.250000	0.001655
2101.000000	0.001654
2100.750000	0.001635
2100.500000	0.001603
2100.250000	0.001564
2100.000000	0.001526
2099.750000	0.001496
2099.500000	0.001478
2099.250000	0.001472
2099.000000	0.001477
2098.750000	0.001487
2098.500000	0.001499
2098.250000	0.001507
2098.000000	0.001508
2097.750000	0.001500
2097.500000	0.001485
2097.250000	0.001464
2097.000000	0.001441
2096.750000	0.001420
2096.500000	0.001403
2096.250000	0.001393
2096.000000	0.001388
2095.750000	0.001388
2095.500000	0.001387

2095.250000	0.001384
2095.000000	0.001377
2094.750000	0.001366
2094.500000	0.001352
2094.250000	0.001338
2094.000000	0.001328
2093.750000	0.001322
2093.500000	0.001321
2093.250000	0.001324
2093.000000	0.001329
2092.750000	0.001335
2092.500000	0.001341
2092.250000	0.001347
2092.000000	0.001354
2091.750000	0.001363
2091.500000	0.001375
2091.250000	0.001391
2091.000000	0.001411
2090.750000	0.001432
2090.500000	0.001453
2090.250000	0.001469
2090.000000	0.001478
2089.750000	0.001476
2089.500000	0.001464
2089.250000	0.001442
2089.000000	0.001415

2088.750000	0.001385
2088.500000	0.001359
2088.250000	0.001338
2088.000000	0.001327
2087.750000	0.001325
2087.500000	0.001332
2087.250000	0.001347
2087.000000	0.001370
2086.750000	0.001397
2086.500000	0.001429
2086.250000	0.001462
2086.000000	0.001495
2085.750000	0.001527
2085.500000	0.001555
2085.250000	0.001578
2085.000000	0.001595
2084.750000	0.001608
2084.500000	0.001615
2084.250000	0.001618
2084.000000	0.001618
2083.750000	0.001613
2083.500000	0.001605
2083.250000	0.001593
2083.000000	0.001575
2082.750000	0.001552
2082.500000	0.001525

2082.250000	0.001492
2082.000000	0.001455
2081.750000	0.001414
2081.500000	0.001374
2081.250000	0.001338
2081.000000	0.001311
2080.750000	0.001298
2080.500000	0.001302
2080.250000	0.001323
2080.000000	0.001359
2079.750000	0.001404
2079.500000	0.001453
2079.250000	0.001498
2079.000000	0.001536
2078.750000	0.001564
2078.500000	0.001583
2078.250000	0.001597
2078.000000	0.001607
2077.750000	0.001616
2077.500000	0.001624
2077.250000	0.001628
2077.000000	0.001626
2076.750000	0.001616
2076.500000	0.001598
2076.250000	0.001574
2076.000000	0.001550

2075.750000	0.001529
2075.500000	0.001517
2075.250000	0.001518
2075.000000	0.001529
2074.750000	0.001549
2074.500000	0.001572
2074.250000	0.001594
2074.000000	0.001609
2073.750000	0.001615
2073.500000	0.001612
2073.250000	0.001603
2073.000000	0.001588
2072.750000	0.001571
2072.500000	0.001551
2072.250000	0.001528
2072.000000	0.001504
2071.750000	0.001478
2071.500000	0.001453
2071.250000	0.001431
2071.000000	0.001416
2070.750000	0.001408
2070.500000	0.001406
2070.250000	0.001407
2070.000000	0.001407
2069.750000	0.001404
2069.500000	0.001396

2069.250000	0.001387
2069.000000	0.001381
2068.750000	0.001382
2068.500000	0.001393
2068.250000	0.001412
2068.000000	0.001436
2067.750000	0.001458
2067.500000	0.001472
2067.250000	0.001475
2067.000000	0.001468
2066.750000	0.001455
2066.500000	0.001440
2066.250000	0.001428
2066.000000	0.001419
2065.750000	0.001414
2065.500000	0.001408
2065.250000	0.001399
2065.000000	0.001385
2064.750000	0.001368
2064.500000	0.001350
2064.250000	0.001335
2064.000000	0.001324
2063.750000	0.001318
2063.500000	0.001314
2063.250000	0.001310
2063.000000	0.001303

2062.750000	0.001291
2062.500000	0.001276
2062.250000	0.001259
2062.000000	0.001243
2061.750000	0.001232
2061.500000	0.001228
2061.250000	0.001235
2061.000000	0.001253
2060.750000	0.001282
2060.500000	0.001319
2060.250000	0.001361
2060.000000	0.001404
2059.750000	0.001444
2059.500000	0.001476
2059.250000	0.001500
2059.000000	0.001515
2058.750000	0.001521
2058.500000	0.001522
2058.250000	0.001516
2058.000000	0.001506
2057.750000	0.001493
2057.500000	0.001478
2057.250000	0.001464
2057.000000	0.001456
2056.750000	0.001457
2056.500000	0.001468

2056.250000	0.001489
2056.000000	0.001516
2055.750000	0.001544
2055.500000	0.001567
2055.250000	0.001581
2055.000000	0.001581
2054.750000	0.001566
2054.500000	0.001538
2054.250000	0.001499
2054.000000	0.001454
2053.750000	0.001409
2053.500000	0.001373
2053.250000	0.001358
2053.000000	0.001371
2052.750000	0.001419
2052.500000	0.001502
2052.250000	0.001612
2052.000000	0.001736
2051.750000	0.001856
2051.500000	0.001953
2051.250000	0.002015
2051.000000	0.002034
2050.750000	0.002013
2050.500000	0.001959
2050.250000	0.001884
2050.000000	0.001799

2049.750000	0.001712
2049.500000	0.001628
2049.250000	0.001550
2049.000000	0.001480
2048.750000	0.001422
2048.500000	0.001383
2048.250000	0.001365
2048.000000	0.001371
2047.750000	0.001397
2047.500000	0.001438
2047.250000	0.001483
2047.000000	0.001525
2046.750000	0.001557
2046.500000	0.001577
2046.250000	0.001587
2046.000000	0.001591
2045.750000	0.001593
2045.500000	0.001594
2045.250000	0.001594
2045.000000	0.001592
2044.750000	0.001585
2044.500000	0.001572
2044.250000	0.001552
2044.000000	0.001526
2043.750000	0.001494
2043.500000	0.001460

2043.250000	0.001427
2043.000000	0.001400
2042.750000	0.001380
2042.500000	0.001371
2042.250000	0.001373
2042.000000	0.001382
2041.750000	0.001395
2041.500000	0.001408
2041.250000	0.001418
2041.000000	0.001424
2040.750000	0.001427
2040.500000	0.001429
2040.250000	0.001432
2040.000000	0.001435
2039.750000	0.001437
2039.500000	0.001438
2039.250000	0.001437
2039.000000	0.001436
2038.750000	0.001442
2038.500000	0.001460
2038.250000	0.001496
2038.000000	0.001554
2037.750000	0.001631
2037.500000	0.001718
2037.250000	0.001806
2037.000000	0.001883

2036.750000	0.001937
2036.500000	0.001963
2036.250000	0.001958
2036.000000	0.001927
2035.750000	0.001874
2035.500000	0.001808
2035.250000	0.001735
2035.000000	0.001663
2034.750000	0.001595
2034.500000	0.001533
2034.250000	0.001480
2034.000000	0.001437
2033.750000	0.001404
2033.500000	0.001385
2033.250000	0.001380
2033.000000	0.001390
2032.750000	0.001414
2032.500000	0.001445
2032.250000	0.001478
2032.000000	0.001504
2031.750000	0.001517
2031.500000	0.001516
2031.250000	0.001503
2031.000000	0.001487
2030.750000	0.001476
2030.500000	0.001476

2030.250000	0.001489
2030.000000	0.001513
2029.750000	0.001539
2029.500000	0.001560
2029.250000	0.001569
2029.000000	0.001565
2028.750000	0.001550
2028.500000	0.001530
2028.250000	0.001510
2028.000000	0.001494
2027.750000	0.001481
2027.500000	0.001469
2027.250000	0.001452
2027.000000	0.001428
2026.750000	0.001395
2026.500000	0.001354
2026.250000	0.001309
2026.000000	0.001267
2025.750000	0.001232
2025.500000	0.001211
2025.250000	0.001205
2025.000000	0.001215
2024.750000	0.001237
2024.500000	0.001267
2024.250000	0.001296
2024.000000	0.001314

2023.750000	0.001318
2023.500000	0.001305
2023.250000	0.001279
2023.000000	0.001249
2022.750000	0.001224
2022.500000	0.001211
2022.250000	0.001212
2022.000000	0.001224
2021.750000	0.001240
2021.500000	0.001254
2021.250000	0.001264
2021.000000	0.001274
2020.750000	0.001289
2020.500000	0.001315
2020.250000	0.001354
2020.000000	0.001398
2019.750000	0.001438
2019.500000	0.001460
2019.250000	0.001458
2019.000000	0.001433
2018.750000	0.001394
2018.500000	0.001356
2018.250000	0.001331
2018.000000	0.001324
2017.750000	0.001331
2017.500000	0.001338

2017.250000	0.001328
2017.000000	0.001290
2016.750000	0.001224
2016.500000	0.001142
2016.250000	0.001065
2016.000000	0.001014
2015.750000	0.001002
2015.500000	0.001033
2015.250000	0.001096
2015.000000	0.001177
2014.750000	0.001258
2014.500000	0.001324
2014.250000	0.001368
2014.000000	0.001390
2013.750000	0.001392
2013.500000	0.001383
2013.250000	0.001371
2013.000000	0.001363
2012.750000	0.001362
2012.500000	0.001372
2012.250000	0.001391
2012.000000	0.001417
2011.750000	0.001444
2011.500000	0.001467
2011.250000	0.001481
2011.000000	0.001480

2010.750000	0.001464
2010.500000	0.001434
2010.250000	0.001396
2010.000000	0.001356
2009.750000	0.001325
2009.500000	0.001307
2009.250000	0.001307
2009.000000	0.001323
2008.750000	0.001349
2008.500000	0.001374
2008.250000	0.001388
2008.000000	0.001379
2007.750000	0.001343
2007.500000	0.001279
2007.250000	0.001197
2007.000000	0.001108
2006.750000	0.001025
2006.500000	0.000957
2006.250000	0.000911
2006.000000	0.000884
2005.750000	0.000874
2005.500000	0.000873
2005.250000	0.000881
2005.000000	0.000895
2004.750000	0.000918
2004.500000	0.000950

2004.250000	0.000990
2004.000000	0.001030
2003.750000	0.001062
2003.500000	0.001077
2003.250000	0.001071
2003.000000	0.001044
2002.750000	0.001005
2002.500000	0.000966
2002.250000	0.000938
2002.000000	0.000930
2001.750000	0.000943
2001.500000	0.000972
2001.250000	0.001009
2001.000000	0.001043
2000.750000	0.001068
2000.500000	0.001081
2000.250000	0.001082
2000.000000	0.001073
1999.750000	0.001058
1999.500000	0.001039
1999.250000	0.001017
1999.000000	0.000993
1998.750000	0.000970
1998.500000	0.000953
1998.250000	0.000948
1998.000000	0.000960

1997.750000	0.000996
1997.500000	0.001055
1997.250000	0.001134
1997.000000	0.001227
1996.750000	0.001323
1996.500000	0.001410
1996.250000	0.001479
1996.000000	0.001527
1995.750000	0.001552
1995.500000	0.001560
1995.250000	0.001557
1995.000000	0.001548
1994.750000	0.001538
1994.500000	0.001527
1994.250000	0.001510
1994.000000	0.001483
1993.750000	0.001443
1993.500000	0.001389
1993.250000	0.001325
1993.000000	0.001257
1992.750000	0.001193
1992.500000	0.001139
1992.250000	0.001098
1992.000000	0.001068
1991.750000	0.001043
1991.500000	0.001025

1991.250000	0.001015
1991.000000	0.001021
1990.750000	0.001051
1990.500000	0.001108
1990.250000	0.001191
1990.000000	0.001289
1989.750000	0.001386
1989.500000	0.001462
1989.250000	0.001500
1989.000000	0.001490
1988.750000	0.001432
1988.500000	0.001338
1988.250000	0.001228
1988.000000	0.001121
1987.750000	0.001037
1987.500000	0.000984
1987.250000	0.000965
1987.000000	0.000978
1986.750000	0.001017
1986.500000	0.001077
1986.250000	0.001150
1986.000000	0.001228
1985.750000	0.001305
1985.500000	0.001369
1985.250000	0.001413
1985.000000	0.001429

1984.750000	0.001416
1984.500000	0.001376
1984.250000	0.001318
1984.000000	0.001255
1983.750000	0.001198
1983.500000	0.001160
1983.250000	0.001150
1983.000000	0.001172
1982.750000	0.001228
1982.500000	0.001311
1982.250000	0.001413
1982.000000	0.001519
1981.750000	0.001615
1981.500000	0.001689
1981.250000	0.001736
1981.000000	0.001757
1980.750000	0.001760
1980.500000	0.001756
1980.250000	0.001755
1980.000000	0.001762
1979.750000	0.001775
1979.500000	0.001783
1979.250000	0.001776
1979.000000	0.001746
1978.750000	0.001689
1978.500000	0.001612

1978.250000	0.001524
1978.000000	0.001436
1977.750000	0.001353
1977.500000	0.001278
1977.250000	0.001208
1977.000000	0.001140
1976.750000	0.001077
1976.500000	0.001025
1976.250000	0.000993
1976.000000	0.000986
1975.750000	0.001006
1975.500000	0.001044
1975.250000	0.001086
1975.000000	0.001114
1974.750000	0.001119
1974.500000	0.001097
1974.250000	0.001055
1974.000000	0.001008
1973.750000	0.000973
1973.500000	0.000962
1973.250000	0.000978
1973.000000	0.001016
1972.750000	0.001062
1972.500000	0.001103
1972.250000	0.001125
1972.000000	0.001122

1971.750000	0.001094
1971.500000	0.001046
1971.250000	0.000987
1971.000000	0.000928
1970.750000	0.000875
1970.500000	0.000836
1970.250000	0.000814
1970.000000	0.000810
1969.750000	0.000825
1969.500000	0.000854
1969.250000	0.000895
1969.000000	0.000941
1968.750000	0.000986
1968.500000	0.001023
1968.250000	0.001045
1968.000000	0.001049
1967.750000	0.001036
1967.500000	0.001008
1967.250000	0.000972
1967.000000	0.000935
1966.750000	0.000905
1966.500000	0.000887
1966.250000	0.000883
1966.000000	0.000893
1965.750000	0.000917
1965.500000	0.000953

1965.250000	0.000999
1965.000000	0.001053
1964.750000	0.001111
1964.500000	0.001166
1964.250000	0.001209
1964.000000	0.001234
1963.750000	0.001235
1963.500000	0.001211
1963.250000	0.001170
1963.000000	0.001122
1962.750000	0.001080
1962.500000	0.001055
1962.250000	0.001053
1962.000000	0.001072
1961.750000	0.001104
1961.500000	0.001139
1961.250000	0.001164
1961.000000	0.001171
1960.750000	0.001153
1960.500000	0.001112
1960.250000	0.001050
1960.000000	0.000975
1959.750000	0.000894
1959.500000	0.000812
1959.250000	0.000736
1959.000000	0.000675

1958.750000	0.000634
1958.500000	0.000616
1958.250000	0.000624
1958.000000	0.000654
1957.750000	0.000699
1957.500000	0.000751
1957.250000	0.000802
1957.000000	0.000848
1956.750000	0.000888
1956.500000	0.000921
1956.250000	0.000951
1956.000000	0.000979
1955.750000	0.001005
1955.500000	0.001026
1955.250000	0.001038
1955.000000	0.001037
1954.750000	0.001021
1954.500000	0.000991
1954.250000	0.000950
1954.000000	0.000905
1953.750000	0.000860
1953.500000	0.000822
1953.250000	0.000797
1953.000000	0.000787
1952.750000	0.000793
1952.500000	0.000814

1952.250000	0.000844
1952.000000	0.000875
1951.750000	0.000899
1951.500000	0.000910
1951.250000	0.000903
1951.000000	0.000880
1950.750000	0.000846
1950.500000	0.000810
1950.250000	0.000779
1950.000000	0.000760
1949.750000	0.000755
1949.500000	0.000763
1949.250000	0.000781
1949.000000	0.000805
1948.750000	0.000831
1948.500000	0.000856
1948.250000	0.000878
1948.000000	0.000893
1947.750000	0.000898
1947.500000	0.000889
1947.250000	0.000862
1947.000000	0.000818
1946.750000	0.000759
1946.500000	0.000694
1946.250000	0.000635
1946.000000	0.000592

1945.750000	0.000572
1945.500000	0.000578
1945.250000	0.000607
1945.000000	0.000653
1944.750000	0.000707
1944.500000	0.000761
1944.250000	0.000808
1944.000000	0.000839
1943.750000	0.000851
1943.500000	0.000839
1943.250000	0.000806
1943.000000	0.000756
1942.750000	0.000698
1942.500000	0.000643
1942.250000	0.000602
1942.000000	0.000583
1941.750000	0.000588
1941.500000	0.000613
1941.250000	0.000649
1941.000000	0.000689
1940.750000	0.000726
1940.500000	0.000758
1940.250000	0.000785
1940.000000	0.000811
1939.750000	0.000836
1939.500000	0.000862

1939.250000	0.000887
1939.000000	0.000910
1938.750000	0.000930
1938.500000	0.000944
1938.250000	0.000954
1938.000000	0.000957
1937.750000	0.000956
1937.500000	0.000948
1937.250000	0.000935
1937.000000	0.000916
1936.750000	0.000893
1936.500000	0.000868
1936.250000	0.000843
1936.000000	0.000821
1935.750000	0.000801
1935.500000	0.000783
1935.250000	0.000768
1935.000000	0.000754
1934.750000	0.000744
1934.500000	0.000739
1934.250000	0.000741
1934.000000	0.000750
1933.750000	0.000766
1933.500000	0.000786
1933.250000	0.000809
1933.000000	0.000833

1932.750000	0.000857
1932.500000	0.000884
1932.250000	0.000913
1932.000000	0.000945
1931.750000	0.000979
1931.500000	0.001013
1931.250000	0.001041
1931.000000	0.001061
1930.750000	0.001070
1930.500000	0.001067
1930.250000	0.001052
1930.000000	0.001028
1929.750000	0.000998
1929.500000	0.000963
1929.250000	0.000926
1929.000000	0.000887
1928.750000	0.000848
1928.500000	0.000812
1928.250000	0.000780
1928.000000	0.000755
1927.750000	0.000740
1927.500000	0.000734
1927.250000	0.000739
1927.000000	0.000754
1926.750000	0.000778
1926.500000	0.000806

1926.250000	0.000835
1926.000000	0.000860
1925.750000	0.000879
1925.500000	0.000889
1925.250000	0.000890
1925.000000	0.000883
1924.750000	0.000871
1924.500000	0.000856
1924.250000	0.000841
1924.000000	0.000827
1923.750000	0.000815
1923.500000	0.000804
1923.250000	0.000792
1923.000000	0.000780
1922.750000	0.000767
1922.500000	0.000755
1922.250000	0.000748
1922.000000	0.000749
1921.750000	0.000760
1921.500000	0.000778
1921.250000	0.000801
1921.000000	0.000823
1920.750000	0.000843
1920.500000	0.000861
1920.250000	0.000878
1920.000000	0.000894

1919.750000	0.000911
1919.500000	0.000927
1919.250000	0.000941
1919.000000	0.000950
1918.750000	0.000954
1918.500000	0.000953
1918.250000	0.000947
1918.000000	0.000937
1917.750000	0.000924
1917.500000	0.000912
1917.250000	0.000906
1917.000000	0.000905
1916.750000	0.000910
1916.500000	0.000914
1916.250000	0.000912
1916.000000	0.000903
1915.750000	0.000884
1915.500000	0.000857
1915.250000	0.000823
1915.000000	0.000786
1914.750000	0.000751
1914.500000	0.000721
1914.250000	0.000702
1914.000000	0.000699
1913.750000	0.000714
1913.500000	0.000746

1913.250000	0.000789
1913.000000	0.000837
1912.750000	0.000881
1912.500000	0.000912
1912.250000	0.000925
1912.000000	0.000920
1911.750000	0.000900
1911.500000	0.000870
1911.250000	0.000836
1911.000000	0.000802
1910.750000	0.000775
1910.500000	0.000756
1910.250000	0.000748
1910.000000	0.000749
1909.750000	0.000756
1909.500000	0.000764
1909.250000	0.000770
1909.000000	0.000768
1908.750000	0.000758
1908.500000	0.000740
1908.250000	0.000718
1908.000000	0.000697
1907.750000	0.000685
1907.500000	0.000687
1907.250000	0.000706
1907.000000	0.000742

1906.750000	0.000790
1906.500000	0.000841
1906.250000	0.000888
1906.000000	0.000922
1905.750000	0.000937
1905.500000	0.000931
1905.250000	0.000905
1905.000000	0.000867
1904.750000	0.000823
1904.500000	0.000781
1904.250000	0.000748
1904.000000	0.000730
1903.750000	0.000725
1903.500000	0.000734
1903.250000	0.000753
1903.000000	0.000777
1902.750000	0.000802
1902.500000	0.000826
1902.250000	0.000843
1902.000000	0.000853
1901.750000	0.000851
1901.500000	0.000838
1901.250000	0.000814
1901.000000	0.000785
1900.750000	0.000756
1900.500000	0.000736

1900.250000	0.000731
1900.000000	0.000744
1899.750000	0.000775
1899.500000	0.000817
1899.250000	0.000862
1899.000000	0.000902
1898.750000	0.000931
1898.500000	0.000944
1898.250000	0.000943
1898.000000	0.000932
1897.750000	0.000916
1897.500000	0.000898
1897.250000	0.000883
1897.000000	0.000871
1896.750000	0.000862
1896.500000	0.000855
1896.250000	0.000849
1896.000000	0.000845
1895.750000	0.000843
1895.500000	0.000844
1895.250000	0.000846
1895.000000	0.000850
1894.750000	0.000856
1894.500000	0.000862
1894.250000	0.000870
1894.000000	0.000877

1893.750000	0.000882
1893.500000	0.000884
1893.250000	0.000884
1893.000000	0.000880
1892.750000	0.000875
1892.500000	0.000872
1892.250000	0.000872
1892.000000	0.000878
1891.750000	0.000892
1891.500000	0.000911
1891.250000	0.000933
1891.000000	0.000951
1890.750000	0.000959
1890.500000	0.000952
1890.250000	0.000928
1890.000000	0.000888
1889.750000	0.000834
1889.500000	0.000775
1889.250000	0.000722
1889.000000	0.000682
1888.750000	0.000661
1888.500000	0.000658
1888.250000	0.000668
1888.000000	0.000683
1887.750000	0.000699
1887.500000	0.000714

1887.250000	0.000728
1887.000000	0.000744
1886.750000	0.000765
1886.500000	0.000789
1886.250000	0.000816
1886.000000	0.000842
1885.750000	0.000865
1885.500000	0.000881
1885.250000	0.000888
1885.000000	0.000887
1884.750000	0.000881
1884.500000	0.000871
1884.250000	0.000862
1884.000000	0.000855
1883.750000	0.000849
1883.500000	0.000847
1883.250000	0.000846
1883.000000	0.000845
1882.750000	0.000844
1882.500000	0.000842
1882.250000	0.000840
1882.000000	0.000838
1881.750000	0.000839
1881.500000	0.000841
1881.250000	0.000843
1881.000000	0.000844

1880.750000	0.000842
1880.500000	0.000834
1880.250000	0.000821
1880.000000	0.000805
1879.750000	0.000787
1879.500000	0.000772
1879.250000	0.000760
1879.000000	0.000753
1878.750000	0.000750
1878.500000	0.000751
1878.250000	0.000755
1878.000000	0.000760
1877.750000	0.000767
1877.500000	0.000777
1877.250000	0.000791
1877.000000	0.000810
1876.750000	0.000833
1876.500000	0.000858
1876.250000	0.000883
1876.000000	0.000905
1875.750000	0.000921
1875.500000	0.000929
1875.250000	0.000929
1875.000000	0.000922
1874.750000	0.000909
1874.500000	0.000893

1874.250000	0.000877
1874.000000	0.000863
1873.750000	0.000851
1873.500000	0.000844
1873.250000	0.000843
1873.000000	0.000846
1872.750000	0.000854
1872.500000	0.000862
1872.250000	0.000868
1872.000000	0.000868
1871.750000	0.000861
1871.500000	0.000851
1871.250000	0.000838
1871.000000	0.000825
1870.750000	0.000818
1870.500000	0.000817
1870.250000	0.000827
1870.000000	0.000848
1869.750000	0.000877
1869.500000	0.000908
1869.250000	0.000929
1869.000000	0.000932
1868.750000	0.000912
1868.500000	0.000874
1868.250000	0.000830
1868.000000	0.000790

1867.750000	0.000764
1867.500000	0.000754
1867.250000	0.000762
1867.000000	0.000782
1866.750000	0.000809
1866.500000	0.000837
1866.250000	0.000860
1866.000000	0.000875
1865.750000	0.000881
1865.500000	0.000878
1865.250000	0.000867
1865.000000	0.000851
1864.750000	0.000831
1864.500000	0.000810
1864.250000	0.000787
1864.000000	0.000766
1863.750000	0.000749
1863.500000	0.000737
1863.250000	0.000734
1863.000000	0.000739
1862.750000	0.000752
1862.500000	0.000768
1862.250000	0.000783
1862.000000	0.000791
1861.750000	0.000793
1861.500000	0.000788

1861.250000	0.000779
1861.000000	0.000769
1860.750000	0.000760
1860.500000	0.000754
1860.250000	0.000749
1860.000000	0.000745
1859.750000	0.000738
1859.500000	0.000728
1859.250000	0.000712
1859.000000	0.000692
1858.750000	0.000671
1858.500000	0.000653
1858.250000	0.000640
1858.000000	0.000636
1857.750000	0.000642
1857.500000	0.000658
1857.250000	0.000681
1857.000000	0.000708
1856.750000	0.000735
1856.500000	0.000758
1856.250000	0.000773
1856.000000	0.000778
1855.750000	0.000774
1855.500000	0.000762
1855.250000	0.000743
1855.000000	0.000722

1854.750000	0.000702
1854.500000	0.000686
1854.250000	0.000676
1854.000000	0.000675
1853.750000	0.000683
1853.500000	0.000698
1853.250000	0.000719
1853.000000	0.000743
1852.750000	0.000766
1852.500000	0.000784
1852.250000	0.000795
1852.000000	0.000798
1851.750000	0.000792
1851.500000	0.000779
1851.250000	0.000762
1851.000000	0.000743
1850.750000	0.000723
1850.500000	0.000707
1850.250000	0.000695
1850.000000	0.000689
1849.750000	0.000691
1849.500000	0.000700
1849.250000	0.000715
1849.000000	0.000733
1848.750000	0.000748
1848.500000	0.000755

1848.250000	0.000750
1848.000000	0.000731
1847.750000	0.000702
1847.500000	0.000669
1847.250000	0.000638
1847.000000	0.000617
1846.750000	0.000609
1846.500000	0.000614
1846.250000	0.000630
1846.000000	0.000651
1845.750000	0.000673
1845.500000	0.000690
1845.250000	0.000701
1845.000000	0.000704
1844.750000	0.000701
1844.500000	0.000689
1844.250000	0.000670
1844.000000	0.000646
1843.750000	0.000628
1843.500000	0.000629
1843.250000	0.000652
1843.000000	0.000691
1842.750000	0.000731
1842.500000	0.000758
1842.250000	0.000769
1842.000000	0.000763

1841.750000	0.000746
1841.500000	0.000725
1841.250000	0.000708
1841.000000	0.000699
1840.750000	0.000698
1840.500000	0.000702
1840.250000	0.000708
1840.000000	0.000711
1839.750000	0.000710
1839.500000	0.000707
1839.250000	0.000704
1839.000000	0.000703
1838.750000	0.000705
1838.500000	0.000709
1838.250000	0.000713
1838.000000	0.000713
1837.750000	0.000707
1837.500000	0.000693
1837.250000	0.000672
1837.000000	0.000647
1836.750000	0.000626
1836.500000	0.000612
1836.250000	0.000611
1836.000000	0.000622
1835.750000	0.000643
1835.500000	0.000669

1835.250000	0.000694
1835.000000	0.000716
1834.750000	0.000729
1834.500000	0.000734
1834.250000	0.000729
1834.000000	0.000717
1833.750000	0.000700
1833.500000	0.000680
1833.250000	0.000661
1833.000000	0.000646
1832.750000	0.000640
1832.500000	0.000645
1832.250000	0.000663
1832.000000	0.000691
1831.750000	0.000725
1831.500000	0.000759
1831.250000	0.000787
1831.000000	0.000805
1830.750000	0.000809
1830.500000	0.000800
1830.250000	0.000778
1830.000000	0.000742
1829.750000	0.000696
1829.500000	0.000648
1829.250000	0.000611
1829.000000	0.000595

1828.750000	0.000602
1828.500000	0.000625
1828.250000	0.000653
1828.000000	0.000677
1827.750000	0.000694
1827.500000	0.000703
1827.250000	0.000707
1827.000000	0.000711
1826.750000	0.000716
1826.500000	0.000721
1826.250000	0.000727
1826.000000	0.000731
1825.750000	0.000727
1825.500000	0.000713
1825.250000	0.000685
1825.000000	0.000650
1824.750000	0.000619
1824.500000	0.000603
1824.250000	0.000609
1824.000000	0.000634
1823.750000	0.000665
1823.500000	0.000691
1823.250000	0.000705
1823.000000	0.000707
1822.750000	0.000700
1822.500000	0.000691

1822.250000	0.000684
1822.000000	0.000682
1821.750000	0.000687
1821.500000	0.000695
1821.250000	0.000705
1821.000000	0.000713
1820.750000	0.000717
1820.500000	0.000719
1820.250000	0.000718
1820.000000	0.000716
1819.750000	0.000713
1819.500000	0.000709
1819.250000	0.000704
1819.000000	0.000699
1818.750000	0.000692
1818.500000	0.000683
1818.250000	0.000673
1818.000000	0.000660
1817.750000	0.000647
1817.500000	0.000635
1817.250000	0.000627
1817.000000	0.000623
1816.750000	0.000627
1816.500000	0.000638
1816.250000	0.000655
1816.000000	0.000674

1815.750000	0.000692
1815.500000	0.000707
1815.250000	0.000716
1815.000000	0.000720
1814.750000	0.000722
1814.500000	0.000724
1814.250000	0.000728
1814.000000	0.000735
1813.750000	0.000744
1813.500000	0.000751
1813.250000	0.000753
1813.000000	0.000750
1812.750000	0.000741
1812.500000	0.000730
1812.250000	0.000720
1812.000000	0.000714
1811.750000	0.000713
1811.500000	0.000717
1811.250000	0.000725
1811.000000	0.000732
1810.750000	0.000736
1810.500000	0.000737
1810.250000	0.000737
1810.000000	0.000739
1809.750000	0.000748
1809.500000	0.000762

1809.250000	0.000780
1809.000000	0.000795
1808.750000	0.000805
1808.500000	0.000807
1808.250000	0.000802
1808.000000	0.000792
1807.750000	0.000780
1807.500000	0.000771
1807.250000	0.000766
1807.000000	0.000766
1806.750000	0.000771
1806.500000	0.000780
1806.250000	0.000792
1806.000000	0.000805
1805.750000	0.000818
1805.500000	0.000831
1805.250000	0.000843
1805.000000	0.000855
1804.750000	0.000869
1804.500000	0.000884
1804.250000	0.000901
1804.000000	0.000916
1803.750000	0.000928
1803.500000	0.000934
1803.250000	0.000932
1803.000000	0.000923

1802.750000	0.000905
1802.500000	0.000881
1802.250000	0.000854
1802.000000	0.000829
1801.750000	0.000810
1801.500000	0.000804
1801.250000	0.000811
1801.000000	0.000829
1800.750000	0.000855
1800.500000	0.000884
1800.250000	0.000910
1800.000000	0.000929
1799.750000	0.000941
1799.500000	0.000945
1799.250000	0.000944
1799.000000	0.000943
1798.750000	0.000949
1798.500000	0.000962
1798.250000	0.000982
1798.000000	0.001004
1797.750000	0.001026
1797.500000	0.001044
1797.250000	0.001056
1797.000000	0.001062
1796.750000	0.001062
1796.500000	0.001057

1796.250000	0.001050
1796.000000	0.001040
1795.750000	0.001028
1795.500000	0.001017
1795.250000	0.001009
1795.000000	0.001011
1794.750000	0.001025
1794.500000	0.001049
1794.250000	0.001078
1794.000000	0.001106
1793.750000	0.001126
1793.500000	0.001134
1793.250000	0.001130
1793.000000	0.001114
1792.750000	0.001088
1792.500000	0.001058
1792.250000	0.001031
1792.000000	0.001017
1791.750000	0.001018
1791.500000	0.001032
1791.250000	0.001049
1791.000000	0.001062
1790.750000	0.001070
1790.500000	0.001075
1790.250000	0.001083
1790.000000	0.001100

1789.750000	0.001125
1789.500000	0.001158
1789.250000	0.001191
1789.000000	0.001221
1788.750000	0.001244
1788.500000	0.001260
1788.250000	0.001269
1788.000000	0.001276
1787.750000	0.001281
1787.500000	0.001288
1787.250000	0.001297
1787.000000	0.001308
1786.750000	0.001321
1786.500000	0.001333
1786.250000	0.001343
1786.000000	0.001349
1785.750000	0.001349
1785.500000	0.001341
1785.250000	0.001324
1785.000000	0.001303
1784.750000	0.001283
1784.500000	0.001271
1784.250000	0.001274
1784.000000	0.001292
1783.750000	0.001321
1783.500000	0.001355

1783.250000	0.001387
1783.000000	0.001413
1782.750000	0.001431
1782.500000	0.001443
1782.250000	0.001450
1782.000000	0.001455
1781.750000	0.001457
1781.500000	0.001457
1781.250000	0.001455
1781.000000	0.001450
1780.750000	0.001445
1780.500000	0.001441
1780.250000	0.001444
1780.000000	0.001455
1779.750000	0.001475
1779.500000	0.001501
1779.250000	0.001527
1779.000000	0.001550
1778.750000	0.001568
1778.500000	0.001580
1778.250000	0.001590
1778.000000	0.001602
1777.750000	0.001618
1777.500000	0.001640
1777.250000	0.001666
1777.000000	0.001694

1776.750000	0.001722
1776.500000	0.001747
1776.250000	0.001767
1776.000000	0.001781
1775.750000	0.001787
1775.500000	0.001788
1775.250000	0.001792
1775.000000	0.001806
1774.750000	0.001837
1774.500000	0.001883
1774.250000	0.001938
1774.000000	0.001994
1773.750000	0.002042
1773.500000	0.002077
1773.250000	0.002100
1773.000000	0.002116
1772.750000	0.002131
1772.500000	0.002146
1772.250000	0.002160
1772.000000	0.002174
1771.750000	0.002189
1771.500000	0.002209
1771.250000	0.002236
1771.000000	0.002268
1770.750000	0.002306
1770.500000	0.002347

1770.250000	0.002392
1770.000000	0.002439
1769.750000	0.002482
1769.500000	0.002519
1769.250000	0.002544
1769.000000	0.002558
1768.750000	0.002562
1768.500000	0.002561
1768.250000	0.002560
1768.000000	0.002565
1767.750000	0.002583
1767.500000	0.002621
1767.250000	0.002682
1767.000000	0.002760
1766.750000	0.002845
1766.500000	0.002927
1766.250000	0.003000
1766.000000	0.003061
1765.750000	0.003112
1765.500000	0.003157
1765.250000	0.003200
1765.000000	0.003243
1764.750000	0.003286
1764.500000	0.003331
1764.250000	0.003376
1764.000000	0.003421

1763.750000	0.003467
1763.500000	0.003514
1763.250000	0.003562
1763.000000	0.003611
1762.750000	0.003664
1762.500000	0.003722
1762.250000	0.003790
1762.000000	0.003866
1761.750000	0.003942
1761.500000	0.004010
1761.250000	0.004072
1761.000000	0.004134
1760.750000	0.004204
1760.500000	0.004284
1760.250000	0.004365
1760.000000	0.004441
1759.750000	0.004510
1759.500000	0.004572
1759.250000	0.004628
1759.000000	0.004682
1758.750000	0.004737
1758.500000	0.004795
1758.250000	0.004858
1758.000000	0.004926
1757.750000	0.004998
1757.500000	0.005074

1757.250000	0.005156
1757.000000	0.005241
1756.750000	0.005330
1756.500000	0.005424
1756.250000	0.005524
1756.000000	0.005635
1755.750000	0.005756
1755.500000	0.005884
1755.250000	0.006016
1755.000000	0.006146
1754.750000	0.006271
1754.500000	0.006390
1754.250000	0.006503
1754.000000	0.006610
1753.750000	0.006709
1753.500000	0.006801
1753.250000	0.006886
1753.000000	0.006967
1752.750000	0.007045
1752.500000	0.007125
1752.250000	0.007216
1752.000000	0.007330
1751.750000	0.007477
1751.500000	0.007650
1751.250000	0.007832
1751.000000	0.008001

1750.750000	0.008152
1750.500000	0.008294
1750.250000	0.008440
1750.000000	0.008595
1749.750000	0.008754
1749.500000	0.008913
1749.250000	0.009072
1749.000000	0.009233
1748.750000	0.009397
1748.500000	0.009564
1748.250000	0.009735
1748.000000	0.009910
1747.750000	0.010092
1747.500000	0.010281
1747.250000	0.010479
1747.000000	0.010683
1746.750000	0.010893
1746.500000	0.011103
1746.250000	0.011312
1746.000000	0.011516
1745.750000	0.011713
1745.500000	0.011902
1745.250000	0.012086
1745.000000	0.012272
1744.750000	0.012465
1744.500000	0.012673

1744.250000	0.012903
1744.000000	0.013162
1743.750000	0.013449
1743.500000	0.013755
1743.250000	0.014065
1743.000000	0.014361
1742.750000	0.014632
1742.500000	0.014877
1742.250000	0.015100
1742.000000	0.015308
1741.750000	0.015507
1741.500000	0.015705
1741.250000	0.015913
1741.000000	0.016151
1740.750000	0.016447
1740.500000	0.016832
1740.250000	0.017318
1740.000000	0.017879
1739.750000	0.018447
1739.500000	0.018947
1739.250000	0.019341
1739.000000	0.019639
1738.750000	0.019887
1738.500000	0.020120
1738.250000	0.020360
1738.000000	0.020612

1737.750000	0.020874
1737.500000	0.021140
1737.250000	0.021397
1737.000000	0.021634
1736.750000	0.021845
1736.500000	0.022028
1736.250000	0.022182
1736.000000	0.022311
1735.750000	0.022432
1735.500000	0.022568
1735.250000	0.022741
1735.000000	0.022998
1734.750000	0.023433
1734.500000	0.024135
1734.250000	0.025062
1734.000000	0.026000
1733.750000	0.026716
1733.500000	0.027125
1733.250000	0.027314
1733.000000	0.027416
1732.750000	0.027512
1732.500000	0.027623
1732.250000	0.027745
1732.000000	0.027881
1731.750000	0.028040
1731.500000	0.028234

1731.250000	0.028474
1731.000000	0.028773
1730.750000	0.029148
1730.500000	0.029599
1730.250000	0.030100
1730.000000	0.030601
1729.750000	0.031055
1729.500000	0.031439
1729.250000	0.031756
1729.000000	0.032029
1728.750000	0.032284
1728.500000	0.032542
1728.250000	0.032812
1728.000000	0.033096
1727.750000	0.033393
1727.500000	0.033700
1727.250000	0.034011
1727.000000	0.034322
1726.750000	0.034627
1726.500000	0.034922
1726.250000	0.035204
1726.000000	0.035472
1725.750000	0.035726
1725.500000	0.035967
1725.250000	0.036199
1725.000000	0.036428

1724.750000	0.036661
1724.500000	0.036906
1724.250000	0.037168
1724.000000	0.037445
1723.750000	0.037730
1723.500000	0.038006
1723.250000	0.038257
1723.000000	0.038471
1722.750000	0.038651
1722.500000	0.038806
1722.250000	0.038950
1722.000000	0.039091
1721.750000	0.039229
1721.500000	0.039358
1721.250000	0.039466
1721.000000	0.039546
1720.750000	0.039593
1720.500000	0.039610
1720.250000	0.039607
1720.000000	0.039596
1719.750000	0.039595
1719.500000	0.039625
1719.250000	0.039703
1719.000000	0.039849
1718.750000	0.040097
1718.500000	0.040472

1718.250000	0.040936
1718.000000	0.041367
1717.750000	0.041620
1717.500000	0.041622
1717.250000	0.041421
1717.000000	0.041134
1716.750000	0.040866
1716.500000	0.040665
1716.250000	0.040532
1716.000000	0.040468
1715.750000	0.040483
1715.500000	0.040579
1715.250000	0.040729
1715.000000	0.040882
1714.750000	0.040976
1714.500000	0.040971
1714.250000	0.040865
1714.000000	0.040696
1713.750000	0.040515
1713.500000	0.040354
1713.250000	0.040218
1713.000000	0.040100
1712.750000	0.039988
1712.500000	0.039873
1712.250000	0.039753
1712.000000	0.039633

1711.750000	0.039521
1711.500000	0.039427
1711.250000	0.039357
1711.000000	0.039306
1710.750000	0.039263
1710.500000	0.039208
1710.250000	0.039124
1710.000000	0.039006
1709.750000	0.038857
1709.500000	0.038688
1709.250000	0.038510
1709.000000	0.038330
1708.750000	0.038151
1708.500000	0.037973
1708.250000	0.037799
1708.000000	0.037640
1707.750000	0.037509
1707.500000	0.037419
1707.250000	0.037377
1707.000000	0.037372
1706.750000	0.037374
1706.500000	0.037337
1706.250000	0.037230
1706.000000	0.037051
1705.750000	0.036830
1705.500000	0.036611

1705.250000	0.036430
1705.000000	0.036301
1704.750000	0.036209
1704.500000	0.036123
1704.250000	0.036006
1704.000000	0.035839
1703.750000	0.035624
1703.500000	0.035378
1703.250000	0.035128
1703.000000	0.034892
1702.750000	0.034667
1702.500000	0.034442
1702.250000	0.034223
1702.000000	0.034021
1701.750000	0.033824
1701.500000	0.033624
1701.250000	0.033497
1701.000000	0.033598
1700.750000	0.034008
1700.500000	0.034564
1700.250000	0.034877
1700.000000	0.034630
1699.750000	0.033882
1699.500000	0.033026
1699.250000	0.032406
1699.000000	0.032047

1698.750000	0.031781
1698.500000	0.031511
1698.250000	0.031255
1698.000000	0.031028
1697.750000	0.030800
1697.500000	0.030554
1697.250000	0.030305
1697.000000	0.030076
1696.750000	0.029900
1696.500000	0.029813
1696.250000	0.029786
1696.000000	0.029696
1695.750000	0.029412
1695.500000	0.028913
1695.250000	0.028305
1695.000000	0.027733
1694.750000	0.027279
1694.500000	0.026933
1694.250000	0.026640
1694.000000	0.026363
1693.750000	0.026090
1693.500000	0.025816
1693.250000	0.025531
1693.000000	0.025228
1692.750000	0.024912
1692.500000	0.024587

1692.250000	0.024257
1692.000000	0.023925
1691.750000	0.023595
1691.500000	0.023269
1691.250000	0.022945
1691.000000	0.022621
1690.750000	0.022292
1690.500000	0.021953
1690.250000	0.021603
1690.000000	0.021245
1689.750000	0.020886
1689.500000	0.020532
1689.250000	0.020188
1689.000000	0.019854
1688.750000	0.019527
1688.500000	0.019202
1688.250000	0.018875
1688.000000	0.018546
1687.750000	0.018219
1687.500000	0.017903
1687.250000	0.017605
1687.000000	0.017332
1686.750000	0.017080
1686.500000	0.016840
1686.250000	0.016606
1686.000000	0.016372

1685.750000	0.016133
1685.500000	0.015889
1685.250000	0.015662
1685.000000	0.015494
1684.750000	0.015407
1684.500000	0.015358
1684.250000	0.015239
1684.000000	0.014963
1683.750000	0.014559
1683.500000	0.014158
1683.250000	0.013875
1683.000000	0.013715
1682.750000	0.013610
1682.500000	0.013508
1682.250000	0.013402
1682.000000	0.013295
1681.750000	0.013184
1681.500000	0.013066
1681.250000	0.012945
1681.000000	0.012825
1680.750000	0.012704
1680.500000	0.012577
1680.250000	0.012441
1680.000000	0.012297
1679.750000	0.012151
1679.500000	0.012012

1679.250000	0.011890
1679.000000	0.011786
1678.750000	0.011696
1678.500000	0.011613
1678.250000	0.011527
1678.000000	0.011432
1677.750000	0.011325
1677.500000	0.011205
1677.250000	0.011077
1677.000000	0.010948
1676.750000	0.010823
1676.500000	0.010707
1676.250000	0.010614
1676.000000	0.010558
1675.750000	0.010543
1675.500000	0.010541
1675.250000	0.010509
1675.000000	0.010416
1674.750000	0.010266
1674.500000	0.010099
1674.250000	0.009949
1674.000000	0.009824
1673.750000	0.009710
1673.500000	0.009596
1673.250000	0.009481
1673.000000	0.009367

1672.750000	0.009257
1672.500000	0.009152
1672.250000	0.009054
1672.000000	0.008962
1671.750000	0.008870
1671.500000	0.008774
1671.250000	0.008673
1671.000000	0.008571
1670.750000	0.008476
1670.500000	0.008394
1670.250000	0.008326
1670.000000	0.008272
1669.750000	0.008227
1669.500000	0.008179
1669.250000	0.008106
1669.000000	0.007988
1668.750000	0.007830
1668.500000	0.007659
1668.250000	0.007507
1668.000000	0.007388
1667.750000	0.007297
1667.500000	0.007222
1667.250000	0.007152
1667.000000	0.007083
1666.750000	0.007015
1666.500000	0.006948

1666.250000	0.006882
1666.000000	0.006816
1665.750000	0.006748
1665.500000	0.006679
1665.250000	0.006606
1665.000000	0.006530
1664.750000	0.006452
1664.500000	0.006376
1664.250000	0.006303
1664.000000	0.006236
1663.750000	0.006181
1663.500000	0.006141
1663.250000	0.006119
1663.000000	0.006105
1662.750000	0.006085
1662.500000	0.006046
1662.250000	0.005986
1662.000000	0.005915
1661.750000	0.005845
1661.500000	0.005784
1661.250000	0.005729
1661.000000	0.005677
1660.750000	0.005628
1660.500000	0.005581
1660.250000	0.005537
1660.000000	0.005496

1659.750000	0.005460
1659.500000	0.005427
1659.250000	0.005395
1659.000000	0.005362
1658.750000	0.005327
1658.500000	0.005290
1658.250000	0.005253
1658.000000	0.005217
1657.750000	0.005183
1657.500000	0.005150
1657.250000	0.005118
1657.000000	0.005088
1656.750000	0.005059
1656.500000	0.005026
1656.250000	0.004990
1656.000000	0.004951
1655.750000	0.004912
1655.500000	0.004869
1655.250000	0.004816
1655.000000	0.004747
1654.750000	0.004671
1654.500000	0.004595
1654.250000	0.004499
1654.000000	0.004377
1653.750000	0.004309
1653.500000	0.004417

1653.250000	0.004695
1653.000000	0.004966
1652.750000	0.005065
1652.500000	0.005021
1652.250000	0.004974
1652.000000	0.005000
1651.750000	0.005063
1651.500000	0.005113
1651.250000	0.005145
1651.000000	0.005167
1650.750000	0.005182
1650.500000	0.005184
1650.250000	0.005176
1650.000000	0.005161
1649.750000	0.005147
1649.500000	0.005142
1649.250000	0.005145
1649.000000	0.005160
1648.750000	0.005190
1648.500000	0.005235
1648.250000	0.005293
1648.000000	0.005354
1647.750000	0.005410
1647.500000	0.005460
1647.250000	0.005508
1647.000000	0.005567

1646.750000	0.005649
1646.500000	0.005753
1646.250000	0.005865
1646.000000	0.005960
1645.750000	0.006025
1645.500000	0.006062
1645.250000	0.006085
1645.000000	0.006106
1644.750000	0.006128
1644.500000	0.006150
1644.250000	0.006168
1644.000000	0.006182
1643.750000	0.006193
1643.500000	0.006204
1643.250000	0.006217
1643.000000	0.006236
1642.750000	0.006262
1642.500000	0.006293
1642.250000	0.006329
1642.000000	0.006367
1641.750000	0.006405
1641.500000	0.006442
1641.250000	0.006480
1641.000000	0.006520
1640.750000	0.006561
1640.500000	0.006600

1640.250000	0.006636
1640.000000	0.006666
1639.750000	0.006690
1639.500000	0.006711
1639.250000	0.006728
1639.000000	0.006742
1638.750000	0.006755
1638.500000	0.006765
1638.250000	0.006773
1638.000000	0.006775
1637.750000	0.006766
1637.500000	0.006745
1637.250000	0.006712
1637.000000	0.006675
1636.750000	0.006633
1636.500000	0.006594
1636.250000	0.006578
1636.000000	0.006614
1635.750000	0.006694
1635.500000	0.006762
1635.250000	0.006759
1635.000000	0.006676
1634.750000	0.006559
1634.500000	0.006461
1634.250000	0.006401
1634.000000	0.006363

1633.750000	0.006322
1633.500000	0.006269
1633.250000	0.006206
1633.000000	0.006143
1632.750000	0.006080
1632.500000	0.006020
1632.250000	0.005964
1632.000000	0.005911
1631.750000	0.005861
1631.500000	0.005811
1631.250000	0.005760
1631.000000	0.005710
1630.750000	0.005660
1630.500000	0.005610
1630.250000	0.005559
1630.000000	0.005507
1629.750000	0.005455
1629.500000	0.005404
1629.250000	0.005353
1629.000000	0.005305
1628.750000	0.005259
1628.500000	0.005216
1628.250000	0.005175
1628.000000	0.005131
1627.750000	0.005081
1627.500000	0.005026

1627.250000	0.004968
1627.000000	0.004911
1626.750000	0.004857
1626.500000	0.004805
1626.250000	0.004752
1626.000000	0.004695
1625.750000	0.004635
1625.500000	0.004574
1625.250000	0.004513
1625.000000	0.004457
1624.750000	0.004407
1624.500000	0.004367
1624.250000	0.004342
1624.000000	0.004335
1623.750000	0.004338
1623.500000	0.004335
1623.250000	0.004307
1623.000000	0.004249
1622.750000	0.004173
1622.500000	0.004097
1622.250000	0.004034
1622.000000	0.003985
1621.750000	0.003946
1621.500000	0.003914
1621.250000	0.003885
1621.000000	0.003859

1620.750000	0.003835
1620.500000	0.003815
1620.250000	0.003798
1620.000000	0.003783
1619.750000	0.003770
1619.500000	0.003758
1619.250000	0.003744
1619.000000	0.003729
1618.750000	0.003712
1618.500000	0.003693
1618.250000	0.003671
1618.000000	0.003644
1617.750000	0.003619
1617.500000	0.003608
1617.250000	0.003632
1617.000000	0.003699
1616.750000	0.003785
1616.500000	0.003848
1616.250000	0.003867
1616.000000	0.003861
1615.750000	0.003863
1615.500000	0.003887
1615.250000	0.003924
1615.000000	0.003958
1614.750000	0.003985
1614.500000	0.004007

1614.250000	0.004028
1614.000000	0.004049
1613.750000	0.004075
1613.500000	0.004109
1613.250000	0.004149
1613.000000	0.004194
1612.750000	0.004240
1612.500000	0.004286
1612.250000	0.004334
1612.000000	0.004385
1611.750000	0.004440
1611.500000	0.004499
1611.250000	0.004563
1611.000000	0.004629
1610.750000	0.004696
1610.500000	0.004763
1610.250000	0.004830
1610.000000	0.004897
1609.750000	0.004965
1609.500000	0.005033
1609.250000	0.005101
1609.000000	0.005172
1608.750000	0.005245
1608.500000	0.005324
1608.250000	0.005408
1608.000000	0.005499

1607.750000	0.005598
1607.500000	0.005703
1607.250000	0.005813
1607.000000	0.005928
1606.750000	0.006046
1606.500000	0.006165
1606.250000	0.006286
1606.000000	0.006409
1605.750000	0.006533
1605.500000	0.006660
1605.250000	0.006787
1605.000000	0.006915
1604.750000	0.007043
1604.500000	0.007170
1604.250000	0.007297
1604.000000	0.007424
1603.750000	0.007548
1603.500000	0.007666
1603.250000	0.007776
1603.000000	0.007874
1602.750000	0.007955
1602.500000	0.008020
1602.250000	0.008068
1602.000000	0.008103
1601.750000	0.008124
1601.500000	0.008133

1601.250000	0.008127
1601.000000	0.008106
1600.750000	0.008066
1600.500000	0.008009
1600.250000	0.007935
1600.000000	0.007845
1599.750000	0.007741
1599.500000	0.007624
1599.250000	0.007493
1599.000000	0.007350
1598.750000	0.007193
1598.500000	0.007026
1598.250000	0.006851
1598.000000	0.006671
1597.750000	0.006490
1597.500000	0.006307
1597.250000	0.006125
1597.000000	0.005942
1596.750000	0.005759
1596.500000	0.005576
1596.250000	0.005395
1596.000000	0.005217
1595.750000	0.005044
1595.500000	0.004878
1595.250000	0.004719
1595.000000	0.004567

1594.750000	0.004420
1594.500000	0.004279
1594.250000	0.004144
1594.000000	0.004016
1593.750000	0.003897
1593.500000	0.003789
1593.250000	0.003690
1593.000000	0.003602
1592.750000	0.003520
1592.500000	0.003444
1592.250000	0.003372
1592.000000	0.003302
1591.750000	0.003234
1591.500000	0.003168
1591.250000	0.003104
1591.000000	0.003043
1590.750000	0.002987
1590.500000	0.002934
1590.250000	0.002885
1590.000000	0.002842
1589.750000	0.002803
1589.500000	0.002770
1589.250000	0.002741
1589.000000	0.002716
1588.750000	0.002695
1588.500000	0.002677

1588.250000	0.002661
1588.000000	0.002648
1587.750000	0.002637
1587.500000	0.002629
1587.250000	0.002624
1587.000000	0.002621
1586.750000	0.002622
1586.500000	0.002626
1586.250000	0.002631
1586.000000	0.002638
1585.750000	0.002646
1585.500000	0.002655
1585.250000	0.002664
1585.000000	0.002673
1584.750000	0.002682
1584.500000	0.002691
1584.250000	0.002700
1584.000000	0.002708
1583.750000	0.002717
1583.500000	0.002727
1583.250000	0.002740
1583.000000	0.002754
1582.750000	0.002769
1582.500000	0.002785
1582.250000	0.002800
1582.000000	0.002814

1581.750000	0.002826
1581.500000	0.002837
1581.250000	0.002849
1581.000000	0.002862
1580.750000	0.002876
1580.500000	0.002891
1580.250000	0.002908
1580.000000	0.002928
1579.750000	0.002949
1579.500000	0.002970
1579.250000	0.002989
1579.000000	0.003005
1578.750000	0.003015
1578.500000	0.003018
1578.250000	0.003015
1578.000000	0.003005
1577.750000	0.002986
1577.500000	0.002958
1577.250000	0.002924
1577.000000	0.002895
1576.750000	0.002887
1576.500000	0.002909
1576.250000	0.002947
1576.000000	0.002971
1575.750000	0.002967
1575.500000	0.002951

1575.250000	0.002950
1575.000000	0.002972
1574.750000	0.003000
1574.500000	0.003020
1574.250000	0.003025
1574.000000	0.003017
1573.750000	0.003001
1573.500000	0.002980
1573.250000	0.002959
1573.000000	0.002942
1572.750000	0.002930
1572.500000	0.002921
1572.250000	0.002913
1572.000000	0.002904
1571.750000	0.002892
1571.500000	0.002879
1571.250000	0.002865
1571.000000	0.002851
1570.750000	0.002838
1570.500000	0.002832
1570.250000	0.002846
1570.000000	0.002891
1569.750000	0.002960
1569.500000	0.003027
1569.250000	0.003067
1569.000000	0.003075

1568.750000	0.003070
1568.500000	0.003068
1568.250000	0.003075
1568.000000	0.003085
1567.750000	0.003092
1567.500000	0.003094
1567.250000	0.003090
1567.000000	0.003086
1566.750000	0.003082
1566.500000	0.003083
1566.250000	0.003091
1566.000000	0.003110
1565.750000	0.003141
1565.500000	0.003185
1565.250000	0.003239
1565.000000	0.003295
1564.750000	0.003346
1564.500000	0.003385
1564.250000	0.003413
1564.000000	0.003437
1563.750000	0.003462
1563.500000	0.003488
1563.250000	0.003514
1563.000000	0.003537
1562.750000	0.003557
1562.500000	0.003574

1562.250000	0.003587
1562.000000	0.003596
1561.750000	0.003597
1561.500000	0.003590
1561.250000	0.003572
1561.000000	0.003546
1560.750000	0.003519
1560.500000	0.003503
1560.250000	0.003513
1560.000000	0.003567
1559.750000	0.003679
1559.500000	0.003861
1559.250000	0.004107
1559.000000	0.004385
1558.750000	0.004631
1558.500000	0.004780
1558.250000	0.004818
1558.000000	0.004797
1557.750000	0.004787
1557.500000	0.004826
1557.250000	0.004905
1557.000000	0.004991
1556.750000	0.005062
1556.500000	0.005108
1556.250000	0.005133
1556.000000	0.005144

1555.750000	0.005145
1555.500000	0.005145
1555.250000	0.005162
1555.000000	0.005211
1554.750000	0.005303
1554.500000	0.005431
1554.250000	0.005572
1554.000000	0.005698
1553.750000	0.005798
1553.500000	0.005878
1553.250000	0.005953
1553.000000	0.006027
1552.750000	0.006096
1552.500000	0.006156
1552.250000	0.006207
1552.000000	0.006249
1551.750000	0.006285
1551.500000	0.006317
1551.250000	0.006350
1551.000000	0.006392
1550.750000	0.006453
1550.500000	0.006542
1550.250000	0.006656
1550.000000	0.006780
1549.750000	0.006896
1549.500000	0.006995

1549.250000	0.007082
1549.000000	0.007167
1548.750000	0.007258
1548.500000	0.007353
1548.250000	0.007446
1548.000000	0.007532
1547.750000	0.007608
1547.500000	0.007674
1547.250000	0.007731
1547.000000	0.007781
1546.750000	0.007830
1546.500000	0.007881
1546.250000	0.007942
1546.000000	0.008023
1545.750000	0.008134
1545.500000	0.008272
1545.250000	0.008423
1545.000000	0.008561
1544.750000	0.008666
1544.500000	0.008736
1544.250000	0.008784
1544.000000	0.008823
1543.750000	0.008862
1543.500000	0.008905
1543.250000	0.008955
1543.000000	0.009021

1542.750000	0.009113
1542.500000	0.009239
1542.250000	0.009392
1542.000000	0.009547
1541.750000	0.009678
1541.500000	0.009769
1541.250000	0.009825
1541.000000	0.009858
1540.750000	0.009876
1540.500000	0.009897
1540.250000	0.009970
1540.000000	0.010150
1539.750000	0.010430
1539.500000	0.010719
1539.250000	0.010908
1539.000000	0.010964
1538.750000	0.010942
1538.500000	0.010923
1538.250000	0.010947
1538.000000	0.011007
1537.750000	0.011078
1537.500000	0.011144
1537.250000	0.011206
1537.000000	0.011264
1536.750000	0.011315
1536.500000	0.011357

1536.250000	0.011393
1536.000000	0.011422
1535.750000	0.011445
1535.500000	0.011461
1535.250000	0.011473
1535.000000	0.011488
1534.750000	0.011515
1534.500000	0.011572
1534.250000	0.011681
1534.000000	0.011847
1533.750000	0.012042
1533.500000	0.012212
1533.250000	0.012313
1533.000000	0.012344
1532.750000	0.012339
1532.500000	0.012331
1532.250000	0.012334
1532.000000	0.012346
1531.750000	0.012359
1531.500000	0.012371
1531.250000	0.012381
1531.000000	0.012389
1530.750000	0.012400
1530.500000	0.012415
1530.250000	0.012435
1530.000000	0.012459

1529.750000	0.012486
1529.500000	0.012513
1529.250000	0.012542
1529.000000	0.012574
1528.750000	0.012607
1528.500000	0.012639
1528.250000	0.012666
1528.000000	0.012681
1527.750000	0.012681
1527.500000	0.012663
1527.250000	0.012634
1527.000000	0.012602
1526.750000	0.012579
1526.500000	0.012574
1526.250000	0.012595
1526.000000	0.012644
1525.750000	0.012709
1525.500000	0.012767
1525.250000	0.012791
1525.000000	0.012772
1524.750000	0.012718
1524.500000	0.012651
1524.250000	0.012591
1524.000000	0.012546
1523.750000	0.012517
1523.500000	0.012498

1523.250000	0.012488
1523.000000	0.012482
1522.750000	0.012480
1522.500000	0.012478
1522.250000	0.012478
1522.000000	0.012491
1521.750000	0.012526
1521.500000	0.012587
1521.250000	0.012647
1521.000000	0.012669
1520.750000	0.012630
1520.500000	0.012549
1520.250000	0.012467
1520.000000	0.012415
1519.750000	0.012396
1519.500000	0.012396
1519.250000	0.012399
1519.000000	0.012397
1518.750000	0.012388
1518.500000	0.012376
1518.250000	0.012370
1518.000000	0.012386
1517.750000	0.012446
1517.500000	0.012563
1517.250000	0.012718
1517.000000	0.012864

1516.750000	0.012963
1516.500000	0.013015
1516.250000	0.013047
1516.000000	0.013090
1515.750000	0.013157
1515.500000	0.013242
1515.250000	0.013337
1515.000000	0.013432
1514.750000	0.013521
1514.500000	0.013601
1514.250000	0.013673
1514.000000	0.013741
1513.750000	0.013812
1513.500000	0.013894
1513.250000	0.013994
1513.000000	0.014117
1512.750000	0.014259
1512.500000	0.014407
1512.250000	0.014538
1512.000000	0.014634
1511.750000	0.014689
1511.500000	0.014712
1511.250000	0.014718
1511.000000	0.014717
1510.750000	0.014714
1510.500000	0.014707

1510.250000	0.014694
1510.000000	0.014672
1509.750000	0.014635
1509.500000	0.014582
1509.250000	0.014511
1509.000000	0.014423
1508.750000	0.014321
1508.500000	0.014208
1508.250000	0.014086
1508.000000	0.013957
1507.750000	0.013825
1507.500000	0.013700
1507.250000	0.013583
1507.000000	0.013446
1506.750000	0.013230
1506.500000	0.012887
1506.250000	0.012432
1506.000000	0.011953
1505.750000	0.011546
1505.500000	0.011245
1505.250000	0.011028
1505.000000	0.010854
1504.750000	0.010697
1504.500000	0.010550
1504.250000	0.010406
1504.000000	0.010259

1503.750000	0.010106
1503.500000	0.009943
1503.250000	0.009770
1503.000000	0.009586
1502.750000	0.009392
1502.500000	0.009190
1502.250000	0.008985
1502.000000	0.008781
1501.750000	0.008579
1501.500000	0.008384
1501.250000	0.008201
1501.000000	0.008032
1500.750000	0.007881
1500.500000	0.007746
1500.250000	0.007626
1500.000000	0.007516
1499.750000	0.007411
1499.500000	0.007305
1499.250000	0.007191
1499.000000	0.007060
1498.750000	0.006910
1498.500000	0.006752
1498.250000	0.006602
1498.000000	0.006473
1497.750000	0.006369
1497.500000	0.006286

1497.250000	0.006216
1497.000000	0.006157
1496.750000	0.006106
1496.500000	0.006057
1496.250000	0.005997
1496.000000	0.005918
1495.750000	0.005828
1495.500000	0.005746
1495.250000	0.005687
1495.000000	0.005653
1494.750000	0.005635
1494.500000	0.005621
1494.250000	0.005605
1494.000000	0.005587
1493.750000	0.005567
1493.500000	0.005548
1493.250000	0.005531
1493.000000	0.005519
1492.750000	0.005510
1492.500000	0.005505
1492.250000	0.005500
1492.000000	0.005495
1491.750000	0.005489
1491.500000	0.005487
1491.250000	0.005493
1491.000000	0.005516

1490.750000	0.005559
1490.500000	0.005614
1490.250000	0.005663
1490.000000	0.005685
1489.750000	0.005673
1489.500000	0.005637
1489.250000	0.005602
1489.000000	0.005582
1488.750000	0.005584
1488.500000	0.005604
1488.250000	0.005635
1488.000000	0.005675
1487.750000	0.005720
1487.500000	0.005763
1487.250000	0.005799
1487.000000	0.005822
1486.750000	0.005833
1486.500000	0.005838
1486.250000	0.005842
1486.000000	0.005850
1485.750000	0.005865
1485.500000	0.005886
1485.250000	0.005911
1485.000000	0.005939
1484.750000	0.005968
1484.500000	0.005996

1484.250000	0.006021
1484.000000	0.006042
1483.750000	0.006059
1483.500000	0.006074
1483.250000	0.006089
1483.000000	0.006103
1482.750000	0.006119
1482.500000	0.006137
1482.250000	0.006157
1482.000000	0.006178
1481.750000	0.006203
1481.500000	0.006231
1481.250000	0.006263
1481.000000	0.006298
1480.750000	0.006334
1480.500000	0.006371
1480.250000	0.006409
1480.000000	0.006447
1479.750000	0.006486
1479.500000	0.006525
1479.250000	0.006562
1479.000000	0.006597
1478.750000	0.006630
1478.500000	0.006661
1478.250000	0.006688
1478.000000	0.006713

1477.750000	0.006737
1477.500000	0.006763
1477.250000	0.006799
1477.000000	0.006850
1476.750000	0.006917
1476.500000	0.006993
1476.250000	0.007063
1476.000000	0.007117
1475.750000	0.007154
1475.500000	0.007186
1475.250000	0.007221
1475.000000	0.007263
1474.750000	0.007305
1474.500000	0.007346
1474.250000	0.007386
1474.000000	0.007433
1473.750000	0.007495
1473.500000	0.007581
1473.250000	0.007696
1473.000000	0.007844
1472.750000	0.008018
1472.500000	0.008204
1472.250000	0.008379
1472.000000	0.008527
1471.750000	0.008644
1471.500000	0.008737

1471.250000	0.008821
1471.000000	0.008906
1470.750000	0.008994
1470.500000	0.009086
1470.250000	0.009181
1470.000000	0.009279
1469.750000	0.009379
1469.500000	0.009480
1469.250000	0.009578
1469.000000	0.009673
1468.750000	0.009765
1468.500000	0.009851
1468.250000	0.009932
1468.000000	0.010005
1467.750000	0.010069
1467.500000	0.010123
1467.250000	0.010165
1467.000000	0.010197
1466.750000	0.010221
1466.500000	0.010241
1466.250000	0.010264
1466.000000	0.010299
1465.750000	0.010360
1465.500000	0.010457
1465.250000	0.010589
1465.000000	0.010733

1464.750000	0.010857
1464.500000	0.010942
1464.250000	0.010987
1464.000000	0.011009
1463.750000	0.011029
1463.500000	0.011055
1463.250000	0.011088
1463.000000	0.011124
1462.750000	0.011163
1462.500000	0.011202
1462.250000	0.011241
1462.000000	0.011278
1461.750000	0.011312
1461.500000	0.011341
1461.250000	0.011365
1461.000000	0.011385
1460.750000	0.011402
1460.500000	0.011419
1460.250000	0.011437
1460.000000	0.011461
1459.750000	0.011491
1459.500000	0.011528
1459.250000	0.011570
1459.000000	0.011614
1458.750000	0.011655
1458.500000	0.011690

1458.250000	0.011717
1458.000000	0.011743
1457.750000	0.011789
1457.500000	0.011881
1457.250000	0.012031
1457.000000	0.012216
1456.750000	0.012386
1456.500000	0.012499
1456.250000	0.012544
1456.000000	0.012546
1455.750000	0.012536
1455.500000	0.012530
1455.250000	0.012528
1455.000000	0.012523
1454.750000	0.012515
1454.500000	0.012503
1454.250000	0.012490
1454.000000	0.012478
1453.750000	0.012468
1453.500000	0.012465
1453.250000	0.012467
1453.000000	0.012473
1452.750000	0.012480
1452.500000	0.012483
1452.250000	0.012478
1452.000000	0.012464

1451.750000	0.012443
1451.500000	0.012419
1451.250000	0.012395
1451.000000	0.012374
1450.750000	0.012356
1450.500000	0.012338
1450.250000	0.012317
1450.000000	0.012290
1449.750000	0.012257
1449.500000	0.012221
1449.250000	0.012186
1449.000000	0.012157
1448.750000	0.012137
1448.500000	0.012127
1448.250000	0.012119
1448.000000	0.012101
1447.750000	0.012063
1447.500000	0.012006
1447.250000	0.011939
1447.000000	0.011875
1446.750000	0.011821
1446.500000	0.011778
1446.250000	0.011743
1446.000000	0.011712
1445.750000	0.011683
1445.500000	0.011653

1445.250000	0.011621
1445.000000	0.011588
1444.750000	0.011553
1444.500000	0.011517
1444.250000	0.011483
1444.000000	0.011450
1443.750000	0.011420
1443.500000	0.011393
1443.250000	0.011368
1443.000000	0.011345
1442.750000	0.011323
1442.500000	0.011300
1442.250000	0.011276
1442.000000	0.011249
1441.750000	0.011221
1441.500000	0.011192
1441.250000	0.011161
1441.000000	0.011130
1440.750000	0.011100
1440.500000	0.011072
1440.250000	0.011046
1440.000000	0.011018
1439.750000	0.010988
1439.500000	0.010954
1439.250000	0.010915
1439.000000	0.010871

1438.750000	0.010822
1438.500000	0.010772
1438.250000	0.010720
1438.000000	0.010669
1437.750000	0.010622
1437.500000	0.010593
1437.250000	0.010591
1437.000000	0.010612
1436.750000	0.010625
1436.500000	0.010593
1436.250000	0.010506
1436.000000	0.010393
1435.750000	0.010293
1435.500000	0.010224
1435.250000	0.010177
1435.000000	0.010139
1434.750000	0.010099
1434.500000	0.010055
1434.250000	0.010006
1434.000000	0.009951
1433.750000	0.009890
1433.500000	0.009823
1433.250000	0.009754
1433.000000	0.009683
1432.750000	0.009611
1432.500000	0.009539

1432.250000	0.009470
1432.000000	0.009405
1431.750000	0.009345
1431.500000	0.009290
1431.250000	0.009239
1431.000000	0.009193
1430.750000	0.009150
1430.500000	0.009110
1430.250000	0.009070
1430.000000	0.009026
1429.750000	0.008974
1429.500000	0.008918
1429.250000	0.008863
1429.000000	0.008815
1428.750000	0.008777
1428.500000	0.008747
1428.250000	0.008723
1428.000000	0.008702
1427.750000	0.008681
1427.500000	0.008660
1427.250000	0.008640
1427.000000	0.008622
1426.750000	0.008608
1426.500000	0.008597
1426.250000	0.008590
1426.000000	0.008583

1425.750000	0.008577
1425.500000	0.008571
1425.250000	0.008565
1425.000000	0.008563
1424.750000	0.008570
1424.500000	0.008589
1424.250000	0.008619
1424.000000	0.008655
1423.750000	0.008688
1423.500000	0.008714
1423.250000	0.008736
1423.000000	0.008758
1422.750000	0.008784
1422.500000	0.008813
1422.250000	0.008842
1422.000000	0.008867
1421.750000	0.008883
1421.500000	0.008888
1421.250000	0.008884
1421.000000	0.008874
1420.750000	0.008860
1420.500000	0.008848
1420.250000	0.008854
1420.000000	0.008897
1419.750000	0.008987
1419.500000	0.009107

1419.250000	0.009218
1419.000000	0.009285
1418.750000	0.009299
1418.500000	0.009286
1418.250000	0.009274
1418.000000	0.009276
1417.750000	0.009286
1417.500000	0.009295
1417.250000	0.009299
1417.000000	0.009299
1416.750000	0.009296
1416.500000	0.009292
1416.250000	0.009289
1416.000000	0.009291
1415.750000	0.009298
1415.500000	0.009309
1415.250000	0.009322
1415.000000	0.009337
1414.750000	0.009353
1414.500000	0.009371
1414.250000	0.009391
1414.000000	0.009410
1413.750000	0.009429
1413.500000	0.009446
1413.250000	0.009463
1413.000000	0.009477

1412.750000	0.009492
1412.500000	0.009507
1412.250000	0.009524
1412.000000	0.009544
1411.750000	0.009567
1411.500000	0.009593
1411.250000	0.009622
1411.000000	0.009653
1410.750000	0.009685
1410.500000	0.009717
1410.250000	0.009750
1410.000000	0.009784
1409.750000	0.009816
1409.500000	0.009847
1409.250000	0.009875
1409.000000	0.009900
1408.750000	0.009924
1408.500000	0.009949
1408.250000	0.009974
1408.000000	0.009999
1407.750000	0.010024
1407.500000	0.010046
1407.250000	0.010064
1407.000000	0.010079
1406.750000	0.010090
1406.500000	0.010102

1406.250000	0.010117
1406.000000	0.010138
1405.750000	0.010169
1405.500000	0.010208
1405.250000	0.010248
1405.000000	0.010282
1404.750000	0.010302
1404.500000	0.010308
1404.250000	0.010306
1404.000000	0.010301
1403.750000	0.010300
1403.500000	0.010303
1403.250000	0.010308
1403.000000	0.010315
1402.750000	0.010321
1402.500000	0.010326
1402.250000	0.010326
1402.000000	0.010322
1401.750000	0.010313
1401.500000	0.010299
1401.250000	0.010280
1401.000000	0.010259
1400.750000	0.010237
1400.500000	0.010218
1400.250000	0.010204
1400.000000	0.010200

1399.750000	0.010207
1399.500000	0.010220
1399.250000	0.010231
1399.000000	0.010230
1398.750000	0.010209
1398.500000	0.010172
1398.250000	0.010127
1398.000000	0.010086
1397.750000	0.010052
1397.500000	0.010026
1397.250000	0.010007
1397.000000	0.009992
1396.750000	0.009979
1396.500000	0.009966
1396.250000	0.009956
1396.000000	0.009949
1395.750000	0.009951
1395.500000	0.009966
1395.250000	0.009998
1395.000000	0.010044
1394.750000	0.010094
1394.500000	0.010137
1394.250000	0.010165
1394.000000	0.010182
1393.750000	0.010192
1393.500000	0.010202

1393.250000	0.010217
1393.000000	0.010237
1392.750000	0.010261
1392.500000	0.010289
1392.250000	0.010321
1392.000000	0.010356
1391.750000	0.010393
1391.500000	0.010432
1391.250000	0.010472
1391.000000	0.010513
1390.750000	0.010552
1390.500000	0.010589
1390.250000	0.010623
1390.000000	0.010654
1389.750000	0.010681
1389.500000	0.010706
1389.250000	0.010729
1389.000000	0.010751
1388.750000	0.010776
1388.500000	0.010809
1388.250000	0.010857
1388.000000	0.010924
1387.750000	0.011008
1387.500000	0.011098
1387.250000	0.011180
1387.000000	0.011245

1386.750000	0.011291
1386.500000	0.011328
1386.250000	0.011365
1386.000000	0.011409
1385.750000	0.011460
1385.500000	0.011517
1385.250000	0.011580
1385.000000	0.011646
1384.750000	0.011714
1384.500000	0.011783
1384.250000	0.011849
1384.000000	0.011913
1383.750000	0.011971
1383.500000	0.012025
1383.250000	0.012072
1383.000000	0.012116
1382.750000	0.012158
1382.500000	0.012200
1382.250000	0.012243
1382.000000	0.012289
1381.750000	0.012337
1381.500000	0.012384
1381.250000	0.012429
1381.000000	0.012469
1380.750000	0.012501
1380.500000	0.012526

1380.250000	0.012543
1380.000000	0.012552
1379.750000	0.012554
1379.500000	0.012551
1379.250000	0.012542
1379.000000	0.012528
1378.750000	0.012512
1378.500000	0.012493
1378.250000	0.012470
1378.000000	0.012444
1377.750000	0.012414
1377.500000	0.012379
1377.250000	0.012339
1377.000000	0.012295
1376.750000	0.012248
1376.500000	0.012197
1376.250000	0.012144
1376.000000	0.012089
1375.750000	0.012032
1375.500000	0.011974
1375.250000	0.011913
1375.000000	0.011848
1374.750000	0.011780
1374.500000	0.011707
1374.250000	0.011630
1374.000000	0.011550

1373.750000	0.011469
1373.500000	0.011390
1373.250000	0.011317
1373.000000	0.011253
1372.750000	0.011200
1372.500000	0.011157
1372.250000	0.011125
1372.000000	0.011100
1371.750000	0.011080
1371.500000	0.011062
1371.250000	0.011044
1371.000000	0.011024
1370.750000	0.011000
1370.500000	0.010973
1370.250000	0.010944
1370.000000	0.010913
1369.750000	0.010881
1369.500000	0.010849
1369.250000	0.010816
1369.000000	0.010784
1368.750000	0.010753
1368.500000	0.010725
1368.250000	0.010702
1368.000000	0.010685
1367.750000	0.010678
1367.500000	0.010682

1367.250000	0.010698
1367.000000	0.010727
1366.750000	0.010767
1366.500000	0.010814
1366.250000	0.010864
1366.000000	0.010907
1365.750000	0.010935
1365.500000	0.010941
1365.250000	0.010921
1365.000000	0.010876
1364.750000	0.010812
1364.500000	0.010734
1364.250000	0.010649
1364.000000	0.010565
1363.750000	0.010485
1363.500000	0.010410
1363.250000	0.010339
1363.000000	0.010267
1362.750000	0.010197
1362.500000	0.010132
1362.250000	0.010078
1362.000000	0.010038
1361.750000	0.010006
1361.500000	0.009976
1361.250000	0.009940
1361.000000	0.009892

1360.750000	0.009832
1360.500000	0.009764
1360.250000	0.009693
1360.000000	0.009625
1359.750000	0.009563
1359.500000	0.009507
1359.250000	0.009456
1359.000000	0.009408
1358.750000	0.009360
1358.500000	0.009313
1358.250000	0.009266
1358.000000	0.009222
1357.750000	0.009180
1357.500000	0.009143
1357.250000	0.009110
1357.000000	0.009082
1356.750000	0.009056
1356.500000	0.009033
1356.250000	0.009013
1356.000000	0.008994
1355.750000	0.008978
1355.500000	0.008964
1355.250000	0.008950
1355.000000	0.008937
1354.750000	0.008922
1354.500000	0.008907

1354.250000	0.008890
1354.000000	0.008873
1353.750000	0.008856
1353.500000	0.008841
1353.250000	0.008827
1353.000000	0.008813
1352.750000	0.008796
1352.500000	0.008777
1352.250000	0.008755
1352.000000	0.008730
1351.750000	0.008704
1351.500000	0.008677
1351.250000	0.008651
1351.000000	0.008624
1350.750000	0.008597
1350.500000	0.008567
1350.250000	0.008534
1350.000000	0.008499
1349.750000	0.008461
1349.500000	0.008422
1349.250000	0.008381
1349.000000	0.008340
1348.750000	0.008299
1348.500000	0.008260
1348.250000	0.008222
1348.000000	0.008187

1347.750000	0.008152
1347.500000	0.008118
1347.250000	0.008083
1347.000000	0.008045
1346.750000	0.008004
1346.500000	0.007960
1346.250000	0.007914
1346.000000	0.007868
1345.750000	0.007825
1345.500000	0.007785
1345.250000	0.007748
1345.000000	0.007715
1344.750000	0.007684
1344.500000	0.007654
1344.250000	0.007624
1344.000000	0.007594
1343.750000	0.007564
1343.500000	0.007534
1343.250000	0.007502
1343.000000	0.007469
1342.750000	0.007436
1342.500000	0.007402
1342.250000	0.007370
1342.000000	0.007340
1341.750000	0.007312
1341.500000	0.007286

1341.250000	0.007260
1341.000000	0.007234
1340.750000	0.007207
1340.500000	0.007178
1340.250000	0.007147
1340.000000	0.007116
1339.750000	0.007087
1339.500000	0.007062
1339.250000	0.007042
1339.000000	0.007027
1338.750000	0.007018
1338.500000	0.007012
1338.250000	0.007011
1338.000000	0.007014
1337.750000	0.007021
1337.500000	0.007032
1337.250000	0.007045
1337.000000	0.007061
1336.750000	0.007076
1336.500000	0.007091
1336.250000	0.007104
1336.000000	0.007115
1335.750000	0.007128
1335.500000	0.007142
1335.250000	0.007158
1335.000000	0.007177

1334.750000	0.007199
1334.500000	0.007222
1334.250000	0.007247
1334.000000	0.007274
1333.750000	0.007303
1333.500000	0.007333
1333.250000	0.007365
1333.000000	0.007398
1332.750000	0.007430
1332.500000	0.007461
1332.250000	0.007492
1332.000000	0.007522
1331.750000	0.007551
1331.500000	0.007581
1331.250000	0.007611
1331.000000	0.007643
1330.750000	0.007675
1330.500000	0.007707
1330.250000	0.007741
1330.000000	0.007774
1329.750000	0.007809
1329.500000	0.007845
1329.250000	0.007883
1329.000000	0.007922
1328.750000	0.007962
1328.500000	0.008001

1328.250000	0.008039
1328.000000	0.008074
1327.750000	0.008107
1327.500000	0.008139
1327.250000	0.008171
1327.000000	0.008203
1326.750000	0.008237
1326.500000	0.008271
1326.250000	0.008306
1326.000000	0.008340
1325.750000	0.008372
1325.500000	0.008402
1325.250000	0.008430
1325.000000	0.008456
1324.750000	0.008481
1324.500000	0.008508
1324.250000	0.008535
1324.000000	0.008565
1323.750000	0.008597
1323.500000	0.008631
1323.250000	0.008664
1323.000000	0.008697
1322.750000	0.008726
1322.500000	0.008752
1322.250000	0.008774
1322.000000	0.008793

1321.750000	0.008809
1321.500000	0.008821
1321.250000	0.008832
1321.000000	0.008839
1320.750000	0.008844
1320.500000	0.008846
1320.250000	0.008846
1320.000000	0.008844
1319.750000	0.008840
1319.500000	0.008836
1319.250000	0.008832
1319.000000	0.008827
1318.750000	0.008821
1318.500000	0.008813
1318.250000	0.008803
1318.000000	0.008792
1317.750000	0.008780
1317.500000	0.008768
1317.250000	0.008755
1317.000000	0.008743
1316.750000	0.008731
1316.500000	0.008718
1316.250000	0.008704
1316.000000	0.008691
1315.750000	0.008677
1315.500000	0.008664

1315.250000	0.008652
1315.000000	0.008639
1314.750000	0.008627
1314.500000	0.008614
1314.250000	0.008601
1314.000000	0.008590
1313.750000	0.008581
1313.500000	0.008574
1313.250000	0.008569
1313.000000	0.008565
1312.750000	0.008561
1312.500000	0.008555
1312.250000	0.008546
1312.000000	0.008536
1311.750000	0.008524
1311.500000	0.008512
1311.250000	0.008502
1311.000000	0.008494
1310.750000	0.008488
1310.500000	0.008483
1310.250000	0.008478
1310.000000	0.008473
1309.750000	0.008468
1309.500000	0.008464
1309.250000	0.008462
1309.000000	0.008462

1308.750000	0.008465
1308.500000	0.008471
1308.250000	0.008480
1308.000000	0.008491
1307.750000	0.008506
1307.500000	0.008524
1307.250000	0.008547
1307.000000	0.008576
1306.750000	0.008609
1306.500000	0.008647
1306.250000	0.008688
1306.000000	0.008732
1305.750000	0.008778
1305.500000	0.008826
1305.250000	0.008875
1305.000000	0.008925
1304.750000	0.008976
1304.500000	0.009028
1304.250000	0.009079
1304.000000	0.009131
1303.750000	0.009184
1303.500000	0.009240
1303.250000	0.009299
1303.000000	0.009362
1302.750000	0.009428
1302.500000	0.009496

1302.250000	0.009566
1302.000000	0.009636
1301.750000	0.009705
1301.500000	0.009774
1301.250000	0.009843
1301.000000	0.009912
1300.750000	0.009979
1300.500000	0.010046
1300.250000	0.010113
1300.000000	0.010178
1299.750000	0.010244
1299.500000	0.010310
1299.250000	0.010378
1299.000000	0.010446
1298.750000	0.010513
1298.500000	0.010579
1298.250000	0.010639
1298.000000	0.010694
1297.750000	0.010743
1297.500000	0.010785
1297.250000	0.010822
1297.000000	0.010854
1296.750000	0.010880
1296.500000	0.010902
1296.250000	0.010918
1296.000000	0.010929

1295.750000	0.010934
1295.500000	0.010935
1295.250000	0.010932
1295.000000	0.010924
1294.750000	0.010912
1294.500000	0.010895
1294.250000	0.010873
1294.000000	0.010845
1293.750000	0.010811
1293.500000	0.010772
1293.250000	0.010726
1293.000000	0.010675
1292.750000	0.010619
1292.500000	0.010556
1292.250000	0.010488
1292.000000	0.010416
1291.750000	0.010341
1291.500000	0.010264
1291.250000	0.010187
1291.000000	0.010111
1290.750000	0.010036
1290.500000	0.009963
1290.250000	0.009890
1290.000000	0.009818
1289.750000	0.009747
1289.500000	0.009678

1289.250000	0.009610
1289.000000	0.009543
1288.750000	0.009479
1288.500000	0.009416
1288.250000	0.009354
1288.000000	0.009293
1287.750000	0.009234
1287.500000	0.009176
1287.250000	0.009122
1287.000000	0.009072
1286.750000	0.009025
1286.500000	0.008982
1286.250000	0.008943
1286.000000	0.008906
1285.750000	0.008872
1285.500000	0.008842
1285.250000	0.008816
1285.000000	0.008796
1284.750000	0.008782
1284.500000	0.008772
1284.250000	0.008766
1284.000000	0.008761
1283.750000	0.008759
1283.500000	0.008757
1283.250000	0.008756
1283.000000	0.008757

1282.750000	0.008760
1282.500000	0.008767
1282.250000	0.008776
1282.000000	0.008789
1281.750000	0.008804
1281.500000	0.008823
1281.250000	0.008844
1281.000000	0.008869
1280.750000	0.008898
1280.500000	0.008930
1280.250000	0.008967
1280.000000	0.009007
1279.750000	0.009050
1279.500000	0.009096
1279.250000	0.009143
1279.000000	0.009192
1278.750000	0.009242
1278.500000	0.009293
1278.250000	0.009345
1278.000000	0.009397
1277.750000	0.009451
1277.500000	0.009508
1277.250000	0.009569
1277.000000	0.009633
1276.750000	0.009701
1276.500000	0.009771

1276.250000	0.009843
1276.000000	0.009915
1275.750000	0.009986
1275.500000	0.010056
1275.250000	0.010125
1275.000000	0.010193
1274.750000	0.010260
1274.500000	0.010328
1274.250000	0.010395
1274.000000	0.010464
1273.750000	0.010533
1273.500000	0.010604
1273.250000	0.010676
1273.000000	0.010748
1272.750000	0.010820
1272.500000	0.010892
1272.250000	0.010961
1272.000000	0.011028
1271.750000	0.011093
1271.500000	0.011155
1271.250000	0.011217
1271.000000	0.011278
1270.750000	0.011339
1270.500000	0.011401
1270.250000	0.011463
1270.000000	0.011526

1269.750000	0.011588
1269.500000	0.011651
1269.250000	0.011713
1269.000000	0.011775
1268.750000	0.011836
1268.500000	0.011896
1268.250000	0.011954
1268.000000	0.012012
1267.750000	0.012069
1267.500000	0.012127
1267.250000	0.012187
1267.000000	0.012249
1266.750000	0.012314
1266.500000	0.012382
1266.250000	0.012453
1266.000000	0.012526
1265.750000	0.012600
1265.500000	0.012675
1265.250000	0.012749
1265.000000	0.012821
1264.750000	0.012892
1264.500000	0.012961
1264.250000	0.013029
1264.000000	0.013098
1263.750000	0.013169
1263.500000	0.013242

1263.250000	0.013320
1263.000000	0.013400
1262.750000	0.013484
1262.500000	0.013570
1262.250000	0.013656
1262.000000	0.013744
1261.750000	0.013832
1261.500000	0.013919
1261.250000	0.014007
1261.000000	0.014095
1260.750000	0.014181
1260.500000	0.014266
1260.250000	0.014350
1260.000000	0.014432
1259.750000	0.014516
1259.500000	0.014600
1259.250000	0.014688
1259.000000	0.014778
1258.750000	0.014870
1258.500000	0.014964
1258.250000	0.015058
1258.000000	0.015151
1257.750000	0.015242
1257.500000	0.015333
1257.250000	0.015424
1257.000000	0.015515

1256.750000	0.015608
1256.500000	0.015703
1256.250000	0.015800
1256.000000	0.015899
1255.750000	0.016000
1255.500000	0.016100
1255.250000	0.016200
1255.000000	0.016298
1254.750000	0.016393
1254.500000	0.016483
1254.250000	0.016569
1254.000000	0.016649
1253.750000	0.016725
1253.500000	0.016799
1253.250000	0.016871
1253.000000	0.016944
1252.750000	0.017017
1252.500000	0.017092
1252.250000	0.017168
1252.000000	0.017243
1251.750000	0.017317
1251.500000	0.017388
1251.250000	0.017455
1251.000000	0.017515
1250.750000	0.017570
1250.500000	0.017617

1250.250000	0.017659
1250.000000	0.017697
1249.750000	0.017732
1249.500000	0.017768
1249.250000	0.017806
1249.000000	0.017846
1248.750000	0.017890
1248.500000	0.017935
1248.250000	0.017980
1248.000000	0.018023
1247.750000	0.018060
1247.500000	0.018090
1247.250000	0.018112
1247.000000	0.018126
1246.750000	0.018135
1246.500000	0.018140
1246.250000	0.018144
1246.000000	0.018151
1245.750000	0.018162
1245.500000	0.018176
1245.250000	0.018194
1245.000000	0.018214
1244.750000	0.018231
1244.500000	0.018246
1244.250000	0.018255
1244.000000	0.018260

1243.750000	0.018263
1243.500000	0.018264
1243.250000	0.018267
1243.000000	0.018273
1242.750000	0.018283
1242.500000	0.018296
1242.250000	0.018312
1242.000000	0.018328
1241.750000	0.018344
1241.500000	0.018358
1241.250000	0.018370
1241.000000	0.018377
1240.750000	0.018382
1240.500000	0.018385
1240.250000	0.018388
1240.000000	0.018391
1239.750000	0.018395
1239.500000	0.018402
1239.250000	0.018411
1239.000000	0.018420
1238.750000	0.018428
1238.500000	0.018436
1238.250000	0.018443
1238.000000	0.018451
1237.750000	0.018460
1237.500000	0.018473

1237.250000	0.018490
1237.000000	0.018510
1236.750000	0.018532
1236.500000	0.018553
1236.250000	0.018572
1236.000000	0.018587
1235.750000	0.018598
1235.500000	0.018607
1235.250000	0.018612
1235.000000	0.018615
1234.750000	0.018616
1234.500000	0.018614
1234.250000	0.018610
1234.000000	0.018602
1233.750000	0.018591
1233.500000	0.018576
1233.250000	0.018557
1233.000000	0.018535
1232.750000	0.018510
1232.500000	0.018483
1232.250000	0.018454
1232.000000	0.018424
1231.750000	0.018391
1231.500000	0.018356
1231.250000	0.018319
1231.000000	0.018279

1230.750000	0.018235
1230.500000	0.018187
1230.250000	0.018136
1230.000000	0.018081
1229.750000	0.018022
1229.500000	0.017960
1229.250000	0.017895
1229.000000	0.017825
1228.750000	0.017750
1228.500000	0.017671
1228.250000	0.017587
1228.000000	0.017499
1227.750000	0.017409
1227.500000	0.017317
1227.250000	0.017226
1227.000000	0.017134
1226.750000	0.017041
1226.500000	0.016947
1226.250000	0.016850
1226.000000	0.016751
1225.750000	0.016650
1225.500000	0.016550
1225.250000	0.016450
1225.000000	0.016352
1224.750000	0.016257
1224.500000	0.016163

1224.250000	0.016070
1224.000000	0.015977
1223.750000	0.015884
1223.500000	0.015791
1223.250000	0.015697
1223.000000	0.015604
1222.750000	0.015512
1222.500000	0.015423
1222.250000	0.015339
1222.000000	0.015261
1221.750000	0.015194
1221.500000	0.015141
1221.250000	0.015103
1221.000000	0.015082
1220.750000	0.015079
1220.500000	0.015091
1220.250000	0.015116
1220.000000	0.015151
1219.750000	0.015192
1219.500000	0.015237
1219.250000	0.015283
1219.000000	0.015329
1218.750000	0.015372
1218.500000	0.015411
1218.250000	0.015443
1218.000000	0.015466

1217.750000	0.015477
1217.500000	0.015474
1217.250000	0.015457
1217.000000	0.015426
1216.750000	0.015381
1216.500000	0.015324
1216.250000	0.015258
1216.000000	0.015184
1215.750000	0.015105
1215.500000	0.015023
1215.250000	0.014941
1215.000000	0.014860
1214.750000	0.014784
1214.500000	0.014714
1214.250000	0.014650
1214.000000	0.014595
1213.750000	0.014547
1213.500000	0.014507
1213.250000	0.014472
1213.000000	0.014443
1212.750000	0.014418
1212.500000	0.014397
1212.250000	0.014380
1212.000000	0.014367
1211.750000	0.014357
1211.500000	0.014350

1211.250000	0.014346
1211.000000	0.014344
1210.750000	0.014345
1210.500000	0.014347
1210.250000	0.014350
1210.000000	0.014355
1209.750000	0.014361
1209.500000	0.014368
1209.250000	0.014377
1209.000000	0.014387
1208.750000	0.014401
1208.500000	0.014420
1208.250000	0.014444
1208.000000	0.014475
1207.750000	0.014512
1207.500000	0.014554
1207.250000	0.014599
1207.000000	0.014644
1206.750000	0.014688
1206.500000	0.014729
1206.250000	0.014766
1206.000000	0.014798
1205.750000	0.014828
1205.500000	0.014854
1205.250000	0.014880
1205.000000	0.014905

1204.750000	0.014931
1204.500000	0.014958
1204.250000	0.014987
1204.000000	0.015018
1203.750000	0.015051
1203.500000	0.015085
1203.250000	0.015120
1203.000000	0.015154
1202.750000	0.015188
1202.500000	0.015220
1202.250000	0.015252
1202.000000	0.015283
1201.750000	0.015316
1201.500000	0.015350
1201.250000	0.015385
1201.000000	0.015423
1200.750000	0.015460
1200.500000	0.015497
1200.250000	0.015532
1200.000000	0.015565
1199.750000	0.015596
1199.500000	0.015625
1199.250000	0.015653
1199.000000	0.015681
1198.750000	0.015709
1198.500000	0.015740

1198.250000	0.015772
1198.000000	0.015807
1197.750000	0.015844
1197.500000	0.015883
1197.250000	0.015924
1197.000000	0.015966
1196.750000	0.016009
1196.500000	0.016051
1196.250000	0.016093
1196.000000	0.016134
1195.750000	0.016174
1195.500000	0.016211
1195.250000	0.016247
1195.000000	0.016282
1194.750000	0.016316
1194.500000	0.016352
1194.250000	0.016391
1194.000000	0.016435
1193.750000	0.016485
1193.500000	0.016541
1193.250000	0.016603
1193.000000	0.016669
1192.750000	0.016738
1192.500000	0.016807
1192.250000	0.016875
1192.000000	0.016940

1191.750000	0.017003
1191.500000	0.017064
1191.250000	0.017125
1191.000000	0.017186
1190.750000	0.017251
1190.500000	0.017319
1190.250000	0.017391
1190.000000	0.017466
1189.750000	0.017545
1189.500000	0.017626
1189.250000	0.017707
1189.000000	0.017789
1188.750000	0.017870
1188.500000	0.017952
1188.250000	0.018036
1188.000000	0.018123
1187.750000	0.018212
1187.500000	0.018304
1187.250000	0.018398
1187.000000	0.018490
1186.750000	0.018581
1186.500000	0.018669
1186.250000	0.018753
1186.000000	0.018836
1185.750000	0.018919
1185.500000	0.019002

1185.250000	0.019089
1185.000000	0.019178
1184.750000	0.019270
1184.500000	0.019365
1184.250000	0.019461
1184.000000	0.019559
1183.750000	0.019656
1183.500000	0.019752
1183.250000	0.019845
1183.000000	0.019935
1182.750000	0.020021
1182.500000	0.020103
1182.250000	0.020182
1182.000000	0.020260
1181.750000	0.020338
1181.500000	0.020417
1181.250000	0.020498
1181.000000	0.020580
1180.750000	0.020662
1180.500000	0.020742
1180.250000	0.020818
1180.000000	0.020891
1179.750000	0.020959
1179.500000	0.021023
1179.250000	0.021084
1179.000000	0.021144

1178.750000	0.021204
1178.500000	0.021266
1178.250000	0.021332
1178.000000	0.021403
1177.750000	0.021480
1177.500000	0.021561
1177.250000	0.021645
1177.000000	0.021731
1176.750000	0.021816
1176.500000	0.021900
1176.250000	0.021982
1176.000000	0.022065
1175.750000	0.022148
1175.500000	0.022235
1175.250000	0.022324
1175.000000	0.022416
1174.750000	0.022511
1174.500000	0.022607
1174.250000	0.022704
1174.000000	0.022802
1173.750000	0.022902
1173.500000	0.023003
1173.250000	0.023105
1173.000000	0.023209
1172.750000	0.023314
1172.500000	0.023421

1172.250000	0.023529
1172.000000	0.023640
1171.750000	0.023753
1171.500000	0.023869
1171.250000	0.023988
1171.000000	0.024108
1170.750000	0.024228
1170.500000	0.024347
1170.250000	0.024464
1170.000000	0.024579
1169.750000	0.024692
1169.500000	0.024802
1169.250000	0.024909
1169.000000	0.025012
1168.750000	0.025111
1168.500000	0.025206
1168.250000	0.025299
1168.000000	0.025390
1167.750000	0.025482
1167.500000	0.025574
1167.250000	0.025667
1167.000000	0.025758
1166.750000	0.025844
1166.500000	0.025923
1166.250000	0.025993
1166.000000	0.026055

1165.750000	0.026108
1165.500000	0.026155
1165.250000	0.026197
1165.000000	0.026235
1164.750000	0.026270
1164.500000	0.026301
1164.250000	0.026328
1164.000000	0.026350
1163.750000	0.026368
1163.500000	0.026382
1163.250000	0.026392
1163.000000	0.026398
1162.750000	0.026401
1162.500000	0.026402
1162.250000	0.026400
1162.000000	0.026397
1161.750000	0.026394
1161.500000	0.026390
1161.250000	0.026387
1161.000000	0.026385
1160.750000	0.026385
1160.500000	0.026387
1160.250000	0.026392
1160.000000	0.026402
1159.750000	0.026416
1159.500000	0.026436

1159.250000	0.026459
1159.000000	0.026485
1158.750000	0.026510
1158.500000	0.026535
1158.250000	0.026557
1158.000000	0.026579
1157.750000	0.026600
1157.500000	0.026623
1157.250000	0.026647
1157.000000	0.026674
1156.750000	0.026704
1156.500000	0.026735
1156.250000	0.026770
1156.000000	0.026806
1155.750000	0.026845
1155.500000	0.026888
1155.250000	0.026932
1155.000000	0.026979
1154.750000	0.027027
1154.500000	0.027074
1154.250000	0.027122
1154.000000	0.027169
1153.750000	0.027216
1153.500000	0.027262
1153.250000	0.027307
1153.000000	0.027350

1152.750000	0.027391
1152.500000	0.027428
1152.250000	0.027461
1152.000000	0.027491
1151.750000	0.027520
1151.500000	0.027549
1151.250000	0.027579
1151.000000	0.027611
1150.750000	0.027646
1150.500000	0.027682
1150.250000	0.027720
1150.000000	0.027759
1149.750000	0.027798
1149.500000	0.027835
1149.250000	0.027870
1149.000000	0.027901
1148.750000	0.027928
1148.500000	0.027950
1148.250000	0.027969
1148.000000	0.027985
1147.750000	0.028000
1147.500000	0.028014
1147.250000	0.028028
1147.000000	0.028041
1146.750000	0.028053
1146.500000	0.028063

1146.250000	0.028073
1146.000000	0.028083
1145.750000	0.028092
1145.500000	0.028101
1145.250000	0.028108
1145.000000	0.028114
1144.750000	0.028115
1144.500000	0.028114
1144.250000	0.028110
1144.000000	0.028107
1143.750000	0.028106
1143.500000	0.028110
1143.250000	0.028120
1143.000000	0.028136
1142.750000	0.028155
1142.500000	0.028176
1142.250000	0.028195
1142.000000	0.028211
1141.750000	0.028223
1141.500000	0.028230
1141.250000	0.028233
1141.000000	0.028231
1140.750000	0.028227
1140.500000	0.028220
1140.250000	0.028213
1140.000000	0.028206

1139.750000	0.028200
1139.500000	0.028195
1139.250000	0.028191
1139.000000	0.028187
1138.750000	0.028181
1138.500000	0.028172
1138.250000	0.028162
1138.000000	0.028152
1137.750000	0.028143
1137.500000	0.028137
1137.250000	0.028134
1137.000000	0.028135
1136.750000	0.028139
1136.500000	0.028145
1136.250000	0.028152
1136.000000	0.028158
1135.750000	0.028163
1135.500000	0.028167
1135.250000	0.028170
1135.000000	0.028171
1134.750000	0.028173
1134.500000	0.028175
1134.250000	0.028179
1134.000000	0.028186
1133.750000	0.028198
1133.500000	0.028214

1133.250000	0.028232
1133.000000	0.028251
1132.750000	0.028268
1132.500000	0.028283
1132.250000	0.028295
1132.000000	0.028304
1131.750000	0.028312
1131.500000	0.028319
1131.250000	0.028326
1131.000000	0.028331
1130.750000	0.028335
1130.500000	0.028337
1130.250000	0.028337
1130.000000	0.028337
1129.750000	0.028340
1129.500000	0.028346
1129.250000	0.028359
1129.000000	0.028376
1128.750000	0.028396
1128.500000	0.028416
1128.250000	0.028434
1128.000000	0.028449
1127.750000	0.028459
1127.500000	0.028468
1127.250000	0.028477
1127.000000	0.028488

1126.750000	0.028504
1126.500000	0.028526
1126.250000	0.028555
1126.000000	0.028591
1125.750000	0.028633
1125.500000	0.028678
1125.250000	0.028725
1125.000000	0.028769
1124.750000	0.028808
1124.500000	0.028839
1124.250000	0.028862
1124.000000	0.028877
1123.750000	0.028886
1123.500000	0.028895
1123.250000	0.028908
1123.000000	0.028927
1122.750000	0.028956
1122.500000	0.028995
1122.250000	0.029041
1122.000000	0.029091
1121.750000	0.029140
1121.500000	0.029185
1121.250000	0.029223
1121.000000	0.029253
1120.750000	0.029279
1120.500000	0.029305

1120.250000	0.029335
1120.000000	0.029372
1119.750000	0.029418
1119.500000	0.029471
1119.250000	0.029528
1119.000000	0.029583
1118.750000	0.029634
1118.500000	0.029678
1118.250000	0.029715
1118.000000	0.029747
1117.750000	0.029776
1117.500000	0.029806
1117.250000	0.029837
1117.000000	0.029869
1116.750000	0.029902
1116.500000	0.029935
1116.250000	0.029967
1116.000000	0.029999
1115.750000	0.030032
1115.500000	0.030064
1115.250000	0.030096
1115.000000	0.030126
1114.750000	0.030154
1114.500000	0.030178
1114.250000	0.030200
1114.000000	0.030221

1113.750000	0.030242
1113.500000	0.030265
1113.250000	0.030291
1113.000000	0.030319
1112.750000	0.030348
1112.500000	0.030379
1112.250000	0.030410
1112.000000	0.030443
1111.750000	0.030477
1111.500000	0.030513
1111.250000	0.030548
1111.000000	0.030580
1110.750000	0.030606
1110.500000	0.030625
1110.250000	0.030637
1110.000000	0.030645
1109.750000	0.030651
1109.500000	0.030659
1109.250000	0.030672
1109.000000	0.030690
1108.750000	0.030712
1108.500000	0.030735
1108.250000	0.030755
1108.000000	0.030770
1107.750000	0.030779
1107.500000	0.030783

1107.250000	0.030784
1107.000000	0.030784
1106.750000	0.030786
1106.500000	0.030791
1106.250000	0.030801
1106.000000	0.030816
1105.750000	0.030834
1105.500000	0.030855
1105.250000	0.030874
1105.000000	0.030891
1104.750000	0.030904
1104.500000	0.030912
1104.250000	0.030916
1104.000000	0.030919
1103.750000	0.030920
1103.500000	0.030924
1103.250000	0.030930
1103.000000	0.030939
1102.750000	0.030952
1102.500000	0.030968
1102.250000	0.030985
1102.000000	0.031004
1101.750000	0.031023
1101.500000	0.031040
1101.250000	0.031053
1101.000000	0.031061

1100.750000	0.031064
1100.500000	0.031061
1100.250000	0.031058
1100.000000	0.031056
1099.750000	0.031059
1099.500000	0.031070
1099.250000	0.031088
1099.000000	0.031113
1098.750000	0.031140
1098.500000	0.031166
1098.250000	0.031187
1098.000000	0.031204
1097.750000	0.031215
1097.500000	0.031221
1097.250000	0.031225
1097.000000	0.031227
1096.750000	0.031231
1096.500000	0.031237
1096.250000	0.031247
1096.000000	0.031261
1095.750000	0.031281
1095.500000	0.031305
1095.250000	0.031331
1095.000000	0.031356
1094.750000	0.031376
1094.500000	0.031389

1094.250000	0.031396
1094.000000	0.031398
1093.750000	0.031398
1093.500000	0.031399
1093.250000	0.031406
1093.000000	0.031420
1092.750000	0.031440
1092.500000	0.031466
1092.250000	0.031497
1092.000000	0.031530
1091.750000	0.031565
1091.500000	0.031600
1091.250000	0.031633
1091.000000	0.031662
1090.750000	0.031686
1090.500000	0.031704
1090.250000	0.031716
1090.000000	0.031723
1089.750000	0.031726
1089.500000	0.031728
1089.250000	0.031731
1089.000000	0.031737
1088.750000	0.031748
1088.500000	0.031763
1088.250000	0.031781
1088.000000	0.031802

1087.750000	0.031825
1087.500000	0.031849
1087.250000	0.031872
1087.000000	0.031895
1086.750000	0.031917
1086.500000	0.031940
1086.250000	0.031963
1086.000000	0.031988
1085.750000	0.032015
1085.500000	0.032047
1085.250000	0.032081
1085.000000	0.032118
1084.750000	0.032157
1084.500000	0.032195
1084.250000	0.032231
1084.000000	0.032264
1083.750000	0.032293
1083.500000	0.032317
1083.250000	0.032336
1083.000000	0.032352
1082.750000	0.032366
1082.500000	0.032380
1082.250000	0.032397
1082.000000	0.032418
1081.750000	0.032443
1081.500000	0.032472

1081.250000	0.032501
1081.000000	0.032528
1080.750000	0.032551
1080.500000	0.032571
1080.250000	0.032588
1080.000000	0.032606
1079.750000	0.032627
1079.500000	0.032654
1079.250000	0.032686
1079.000000	0.032721
1078.750000	0.032756
1078.500000	0.032789
1078.250000	0.032816
1078.000000	0.032839
1077.750000	0.032857
1077.500000	0.032872
1077.250000	0.032885
1077.000000	0.032896
1076.750000	0.032907
1076.500000	0.032917
1076.250000	0.032928
1076.000000	0.032941
1075.750000	0.032958
1075.500000	0.032981
1075.250000	0.033008
1075.000000	0.033039

1074.750000	0.033071
1074.500000	0.033100
1074.250000	0.033125
1074.000000	0.033145
1073.750000	0.033159
1073.500000	0.033170
1073.250000	0.033178
1073.000000	0.033186
1072.750000	0.033195
1072.500000	0.033204
1072.250000	0.033216
1072.000000	0.033230
1071.750000	0.033247
1071.500000	0.033268
1071.250000	0.033290
1071.000000	0.033314
1070.750000	0.033338
1070.500000	0.033359
1070.250000	0.033377
1070.000000	0.033391
1069.750000	0.033401
1069.500000	0.033408
1069.250000	0.033413
1069.000000	0.033418
1068.750000	0.033423
1068.500000	0.033431

1068.250000	0.033442
1068.000000	0.033458
1067.750000	0.033480
1067.500000	0.033504
1067.250000	0.033531
1067.000000	0.033556
1066.750000	0.033576
1066.500000	0.033591
1066.250000	0.033599
1066.000000	0.033601
1065.750000	0.033602
1065.500000	0.033603
1065.250000	0.033607
1065.000000	0.033615
1064.750000	0.033628
1064.500000	0.033643
1064.250000	0.033659
1064.000000	0.033672
1063.750000	0.033682
1063.500000	0.033686
1063.250000	0.033684
1063.000000	0.033677
1062.750000	0.033667
1062.500000	0.033657
1062.250000	0.033651
1062.000000	0.033651

1061.750000	0.033660
1061.500000	0.033678
1061.250000	0.033704
1061.000000	0.033734
1060.750000	0.033764
1060.500000	0.033790
1060.250000	0.033807
1060.000000	0.033815
1059.750000	0.033812
1059.500000	0.033801
1059.250000	0.033784
1059.000000	0.033765
1058.750000	0.033747
1058.500000	0.033733
1058.250000	0.033726
1058.000000	0.033724
1057.750000	0.033728
1057.500000	0.033734
1057.250000	0.033741
1057.000000	0.033746
1056.750000	0.033748
1056.500000	0.033747
1056.250000	0.033745
1056.000000	0.033744
1055.750000	0.033745
1055.500000	0.033749

1055.250000	0.033756
1055.000000	0.033765
1054.750000	0.033775
1054.500000	0.033783
1054.250000	0.033789
1054.000000	0.033794
1053.750000	0.033796
1053.500000	0.033795
1053.250000	0.033792
1053.000000	0.033787
1052.750000	0.033779
1052.500000	0.033771
1052.250000	0.033765
1052.000000	0.033762
1051.750000	0.033762
1051.500000	0.033767
1051.250000	0.033773
1051.000000	0.033778
1050.750000	0.033780
1050.500000	0.033779
1050.250000	0.033775
1050.000000	0.033773
1049.750000	0.033774
1049.500000	0.033783
1049.250000	0.033797
1049.000000	0.033816

1048.750000	0.033835
1048.500000	0.033851
1048.250000	0.033861
1048.000000	0.033865
1047.750000	0.033862
1047.500000	0.033854
1047.250000	0.033844
1047.000000	0.033834
1046.750000	0.033823
1046.500000	0.033814
1046.250000	0.033807
1046.000000	0.033803
1045.750000	0.033804
1045.500000	0.033808
1045.250000	0.033816
1045.000000	0.033826
1044.750000	0.033837
1044.500000	0.033846
1044.250000	0.033852
1044.000000	0.033854
1043.750000	0.033854
1043.500000	0.033852
1043.250000	0.033850
1043.000000	0.033847
1042.750000	0.033847
1042.500000	0.033849

1042.250000	0.033854
1042.000000	0.033864
1041.750000	0.033879
1041.500000	0.033899
1041.250000	0.033921
1041.000000	0.033943
1040.750000	0.033962
1040.500000	0.033975
1040.250000	0.033980
1040.000000	0.033977
1039.750000	0.033966
1039.500000	0.033952
1039.250000	0.033935
1039.000000	0.033919
1038.750000	0.033906
1038.500000	0.033897
1038.250000	0.033892
1038.000000	0.033891
1037.750000	0.033893
1037.500000	0.033896
1037.250000	0.033899
1037.000000	0.033900
1036.750000	0.033899
1036.500000	0.033895
1036.250000	0.033891
1036.000000	0.033888

1035.750000	0.033887
1035.500000	0.033890
1035.250000	0.033898
1035.000000	0.033909
1034.750000	0.033922
1034.500000	0.033936
1034.250000	0.033949
1034.000000	0.033960
1033.750000	0.033967
1033.500000	0.033971
1033.250000	0.033968
1033.000000	0.033959
1032.750000	0.033943
1032.500000	0.033922
1032.250000	0.033899
1032.000000	0.033876
1031.750000	0.033858
1031.500000	0.033846
1031.250000	0.033842
1031.000000	0.033845
1030.750000	0.033853
1030.500000	0.033863
1030.250000	0.033871
1030.000000	0.033876
1029.750000	0.033874
1029.500000	0.033865

1029.250000	0.033850
1029.000000	0.033831
1028.750000	0.033808
1028.500000	0.033786
1028.250000	0.033767
1028.000000	0.033753
1027.750000	0.033745
1027.500000	0.033741
1027.250000	0.033739
1027.000000	0.033735
1026.750000	0.033728
1026.500000	0.033714
1026.250000	0.033695
1026.000000	0.033672
1025.750000	0.033649
1025.500000	0.033628
1025.250000	0.033611
1025.000000	0.033600
1024.750000	0.033595
1024.500000	0.033594
1024.250000	0.033596
1024.000000	0.033598
1023.750000	0.033598
1023.500000	0.033596
1023.250000	0.033588
1023.000000	0.033576

1022.750000	0.033558
1022.500000	0.033539
1022.250000	0.033520
1022.000000	0.033503
1021.750000	0.033492
1021.500000	0.033486
1021.250000	0.033485
1021.000000	0.033484
1020.750000	0.033482
1020.500000	0.033475
1020.250000	0.033462
1020.000000	0.033444
1019.750000	0.033421
1019.500000	0.033396
1019.250000	0.033370
1019.000000	0.033345
1018.750000	0.033320
1018.500000	0.033298
1018.250000	0.033279
1018.000000	0.033264
1017.750000	0.033255
1017.500000	0.033251
1017.250000	0.033251
1017.000000	0.033253
1016.750000	0.033254
1016.500000	0.033254

1016.250000	0.033251
1016.000000	0.033245
1015.750000	0.033239
1015.500000	0.033232
1015.250000	0.033227
1015.000000	0.033223
1014.750000	0.033219
1014.500000	0.033216
1014.250000	0.033211
1014.000000	0.033206
1013.750000	0.033201
1013.500000	0.033197
1013.250000	0.033192
1013.000000	0.033187
1012.750000	0.033180
1012.500000	0.033172
1012.250000	0.033160
1012.000000	0.033145
1011.750000	0.033129
1011.500000	0.033112
1011.250000	0.033094
1011.000000	0.033078
1010.750000	0.033064
1010.500000	0.033050
1010.250000	0.033037
1010.000000	0.033025

1009.750000	0.033013
1009.500000	0.033001
1009.250000	0.032986
1009.000000	0.032969
1008.750000	0.032948
1008.500000	0.032923
1008.250000	0.032895
1008.000000	0.032864
1007.750000	0.032830
1007.500000	0.032796
1007.250000	0.032761
1007.000000	0.032726
1006.750000	0.032693
1006.500000	0.032660
1006.250000	0.032629
1006.000000	0.032599
1005.750000	0.032569
1005.500000	0.032538
1005.250000	0.032503
1005.000000	0.032464
1004.750000	0.032421
1004.500000	0.032373
1004.250000	0.032325
1004.000000	0.032278
1003.750000	0.032237
1003.500000	0.032202

1003.250000	0.032174
1003.000000	0.032150
1002.750000	0.032129
1002.500000	0.032107
1002.250000	0.032079
1002.000000	0.032046
1001.750000	0.032006
1001.500000	0.031961
1001.250000	0.031912
1001.000000	0.031861
1000.750000	0.031812
1000.500000	0.031766
1000.250000	0.031726
1000.000000	0.031693
999.750000	0.031667
999.500000	0.031646
999.250000	0.031628
999.000000	0.031608
998.750000	0.031582
998.500000	0.031549
998.250000	0.031507
998.000000	0.031456
997.750000	0.031399
997.500000	0.031341
997.250000	0.031286
997.000000	0.031236

996.750000	0.031195
996.500000	0.031164
996.250000	0.031141
996.000000	0.031126
995.750000	0.031114
995.500000	0.031103
995.250000	0.031091
995.000000	0.031074
994.750000	0.031052
994.500000	0.031025
994.250000	0.030995
994.000000	0.030963
993.750000	0.030931
993.500000	0.030901
993.250000	0.030873
993.000000	0.030846
992.750000	0.030819
992.500000	0.030792
992.250000	0.030761
992.000000	0.030728
991.750000	0.030693
991.500000	0.030655
991.250000	0.030616
991.000000	0.030580
990.750000	0.030546
990.500000	0.030519

990.250000	0.030499
990.000000	0.030487
989.750000	0.030484
989.500000	0.030489
989.250000	0.030500
989.000000	0.030515
988.750000	0.030532
988.500000	0.030548
988.250000	0.030561
988.000000	0.030568
987.750000	0.030567
987.500000	0.030555
987.250000	0.030533
987.000000	0.030501
986.750000	0.030463
986.500000	0.030425
986.250000	0.030391
986.000000	0.030366
985.750000	0.030349
985.500000	0.030341
985.250000	0.030338
985.000000	0.030335
984.750000	0.030330
984.500000	0.030323
984.250000	0.030314
984.000000	0.030305

983.750000	0.030298
983.500000	0.030291
983.250000	0.030282
983.000000	0.030266
982.750000	0.030240
982.500000	0.030202
982.250000	0.030153
982.000000	0.030095
981.750000	0.030034
981.500000	0.029972
981.250000	0.029913
981.000000	0.029858
980.750000	0.029807
980.500000	0.029759
980.250000	0.029717
980.000000	0.029679
979.750000	0.029648
979.500000	0.029621
979.250000	0.029596
979.000000	0.029569
978.750000	0.029537
978.500000	0.029496
978.250000	0.029447
978.000000	0.029393
977.750000	0.029338
977.500000	0.029287

977.250000	0.029244
977.000000	0.029210
976.750000	0.029183
976.500000	0.029159
976.250000	0.029134
976.000000	0.029105
975.750000	0.029069
975.500000	0.029026
975.250000	0.028976
975.000000	0.028922
974.750000	0.028866
974.500000	0.028811
974.250000	0.028760
974.000000	0.028716
973.750000	0.028678
973.500000	0.028647
973.250000	0.028621
973.000000	0.028598
972.750000	0.028575
972.500000	0.028550
972.250000	0.028522
972.000000	0.028490
971.750000	0.028457
971.500000	0.028422
971.250000	0.028386
971.000000	0.028349

970.750000	0.028311
970.500000	0.028272
970.250000	0.028231
970.000000	0.028189
969.750000	0.028147
969.500000	0.028104
969.250000	0.028061
969.000000	0.028017
968.750000	0.027972
968.500000	0.027927
968.250000	0.027882
968.000000	0.027841
967.750000	0.027806
967.500000	0.027779
967.250000	0.027761
967.000000	0.027751
966.750000	0.027746
966.500000	0.027742
966.250000	0.027736
966.000000	0.027725
965.750000	0.027706
965.500000	0.027680
965.250000	0.027647
965.000000	0.027608
964.750000	0.027565
964.500000	0.027521

964.250000	0.027478
964.000000	0.027437
963.750000	0.027401
963.500000	0.027369
963.250000	0.027341
963.000000	0.027315
962.750000	0.027291
962.500000	0.027267
962.250000	0.027242
962.000000	0.027214
961.750000	0.027185
961.500000	0.027154
961.250000	0.027120
961.000000	0.027085
960.750000	0.027050
960.500000	0.027014
960.250000	0.026980
960.000000	0.026949
959.750000	0.026921
959.500000	0.026898
959.250000	0.026877
959.000000	0.026857
958.750000	0.026836
958.500000	0.026812
958.250000	0.026786
958.000000	0.026756

957.750000	0.026723
957.500000	0.026688
957.250000	0.026652
957.000000	0.026615
956.750000	0.026577
956.500000	0.026540
956.250000	0.026505
956.000000	0.026474
955.750000	0.026445
955.500000	0.026419
955.250000	0.026393
955.000000	0.026365
954.750000	0.026334
954.500000	0.026299
954.250000	0.026262
954.000000	0.026225
953.750000	0.026190
953.500000	0.026161
953.250000	0.026136
953.000000	0.026115
952.750000	0.026095
952.500000	0.026073
952.250000	0.026048
952.000000	0.026019
951.750000	0.025986
951.500000	0.025953

951.250000	0.025920
951.000000	0.025888
950.750000	0.025858
950.500000	0.025830
950.250000	0.025802
950.000000	0.025775
949.750000	0.025750
949.500000	0.025725
949.250000	0.025701
949.000000	0.025676
948.750000	0.025651
948.500000	0.025624
948.250000	0.025595
948.000000	0.025565
947.750000	0.025534
947.500000	0.025504
947.250000	0.025475
947.000000	0.025448
946.750000	0.025424
946.500000	0.025403
946.250000	0.025386
946.000000	0.025372
945.750000	0.025360
945.500000	0.025350
945.250000	0.025339
945.000000	0.025326

944.750000	0.025308
944.500000	0.025284
944.250000	0.025254
944.000000	0.025220
943.750000	0.025184
943.500000	0.025149
943.250000	0.025116
943.000000	0.025086
942.750000	0.025059
942.500000	0.025034
942.250000	0.025010
942.000000	0.024985
941.750000	0.024958
941.500000	0.024928
941.250000	0.024895
941.000000	0.024860
940.750000	0.024825
940.500000	0.024791
940.250000	0.024761
940.000000	0.024734
939.750000	0.024711
939.500000	0.024690
939.250000	0.024668
939.000000	0.024640
938.750000	0.024606
938.500000	0.024565

938.250000	0.024517
938.000000	0.024466
937.750000	0.024415
937.500000	0.024366
937.250000	0.024321
937.000000	0.024278
936.750000	0.024236
936.500000	0.024195
936.250000	0.024154
936.000000	0.024112
935.750000	0.024071
935.500000	0.024031
935.250000	0.023989
935.000000	0.023946
934.750000	0.023899
934.500000	0.023847
934.250000	0.023791
934.000000	0.023733
933.750000	0.023677
933.500000	0.023626
933.250000	0.023580
933.000000	0.023541
932.750000	0.023506
932.500000	0.023472
932.250000	0.023436
932.000000	0.023397

931.750000	0.023352
931.500000	0.023303
931.250000	0.023252
931.000000	0.023201
930.750000	0.023152
930.500000	0.023107
930.250000	0.023066
930.000000	0.023029
929.750000	0.022996
929.500000	0.022964
929.250000	0.022933
929.000000	0.022899
928.750000	0.022862
928.500000	0.022821
928.250000	0.022778
928.000000	0.022733
927.750000	0.022686
927.500000	0.022639
927.250000	0.022592
927.000000	0.022543
926.750000	0.022494
926.500000	0.022444
926.250000	0.022396
926.000000	0.022353
925.750000	0.022315
925.500000	0.022283

925.250000	0.022257
925.000000	0.022233
924.750000	0.022210
924.500000	0.022184
924.250000	0.022155
924.000000	0.022122
923.750000	0.022087
923.500000	0.022050
923.250000	0.022014
923.000000	0.021977
922.750000	0.021938
922.500000	0.021896
922.250000	0.021850
922.000000	0.021802
921.750000	0.021752
921.500000	0.021702
921.250000	0.021653
921.000000	0.021605
920.750000	0.021558
920.500000	0.021511
920.250000	0.021464
920.000000	0.021418
919.750000	0.021371
919.500000	0.021325
919.250000	0.021279
919.000000	0.021231

918.750000	0.021182
918.500000	0.021131
918.250000	0.021077
918.000000	0.021022
917.750000	0.020967
917.500000	0.020915
917.250000	0.020864
917.000000	0.020815
916.750000	0.020766
916.500000	0.020716
916.250000	0.020663
916.000000	0.020606
915.750000	0.020547
915.500000	0.020485
915.250000	0.020423
915.000000	0.020358
914.750000	0.020292
914.500000	0.020224
914.250000	0.020155
914.000000	0.020086
913.750000	0.020018
913.500000	0.019953
913.250000	0.019892
913.000000	0.019835
912.750000	0.019780
912.500000	0.019726

912.250000	0.019671
912.000000	0.019614
911.750000	0.019554
911.500000	0.019492
911.250000	0.019426
911.000000	0.019358
910.750000	0.019285
910.500000	0.019209
910.250000	0.019130
910.000000	0.019050
909.750000	0.018970
909.500000	0.018892
909.250000	0.018818
909.000000	0.018749
908.750000	0.018685
908.500000	0.018624
908.250000	0.018563
908.000000	0.018503
907.750000	0.018441
907.500000	0.018377
907.250000	0.018311
907.000000	0.018244
906.750000	0.018175
906.500000	0.018105
906.250000	0.018036
906.000000	0.017967

905.750000	0.017899
905.500000	0.017834
905.250000	0.017771
905.000000	0.017711
904.750000	0.017652
904.500000	0.017595
904.250000	0.017537
904.000000	0.017479
903.750000	0.017419
903.500000	0.017358
903.250000	0.017295
903.000000	0.017229
902.750000	0.017161
902.500000	0.017092
902.250000	0.017023
902.000000	0.016955
901.750000	0.016889
901.500000	0.016825
901.250000	0.016764
901.000000	0.016704
900.750000	0.016643
900.500000	0.016580
900.250000	0.016515
900.000000	0.016449
899.750000	0.016383
899.500000	0.016319

899.250000	0.016259
899.000000	0.016202
898.750000	0.016148
898.500000	0.016095
898.250000	0.016042
898.000000	0.015987
897.750000	0.015930
897.500000	0.015869
897.250000	0.015806
897.000000	0.015741
896.750000	0.015676
896.500000	0.015612
896.250000	0.015550
896.000000	0.015490
895.750000	0.015434
895.500000	0.015381
895.250000	0.015329
895.000000	0.015277
894.750000	0.015224
894.500000	0.015168
894.250000	0.015109
894.000000	0.015047
893.750000	0.014983
893.500000	0.014918
893.250000	0.014853
893.000000	0.014789

892.750000	0.014727
892.500000	0.014668
892.250000	0.014613
892.000000	0.014563
891.750000	0.014517
891.500000	0.014477
891.250000	0.014440
891.000000	0.014405
890.750000	0.014367
890.500000	0.014324
890.250000	0.014275
890.000000	0.014218
889.750000	0.014156
889.500000	0.014090
889.250000	0.014025
889.000000	0.013963
888.750000	0.013907
888.500000	0.013858
888.250000	0.013817
888.000000	0.013781
887.750000	0.013747
887.500000	0.013713
887.250000	0.013675
887.000000	0.013631
886.750000	0.013578
886.500000	0.013519

886.250000	0.013456
886.000000	0.013392
885.750000	0.013330
885.500000	0.013273
885.250000	0.013221
885.000000	0.013175
884.750000	0.013131
884.500000	0.013090
884.250000	0.013048
884.000000	0.013007
883.750000	0.012966
883.500000	0.012927
883.250000	0.012889
883.000000	0.012853
882.750000	0.012816
882.500000	0.012777
882.250000	0.012735
882.000000	0.012689
881.750000	0.012641
881.500000	0.012592
881.250000	0.012543
881.000000	0.012495
880.750000	0.012450
880.500000	0.012406
880.250000	0.012362
880.000000	0.012317

879.750000	0.012271
879.500000	0.012222
879.250000	0.012171
879.000000	0.012117
878.750000	0.012062
878.500000	0.012005
878.250000	0.011949
878.000000	0.011892
877.750000	0.011838
877.500000	0.011785
877.250000	0.011735
877.000000	0.011687
876.750000	0.011639
876.500000	0.011589
876.250000	0.011538
876.000000	0.011484
875.750000	0.011428
875.500000	0.011372
875.250000	0.011316
875.000000	0.011261
874.750000	0.011209
874.500000	0.011160
874.250000	0.011113
874.000000	0.011070
873.750000	0.011029
873.500000	0.010993

873.250000	0.010961
873.000000	0.010932
872.750000	0.010907
872.500000	0.010883
872.250000	0.010859
872.000000	0.010831
871.750000	0.010800
871.500000	0.010762
871.250000	0.010719
871.000000	0.010672
870.750000	0.010621
870.500000	0.010570
870.250000	0.010520
870.000000	0.010474
869.750000	0.010431
869.500000	0.010393
869.250000	0.010359
869.000000	0.010325
868.750000	0.010292
868.500000	0.010257
868.250000	0.010221
868.000000	0.010184
867.750000	0.010149
867.500000	0.010117
867.250000	0.010089
867.000000	0.010063

866.750000	0.010040
866.500000	0.010016
866.250000	0.009989
866.000000	0.009958
865.750000	0.009923
865.500000	0.009885
865.250000	0.009845
865.000000	0.009806
864.750000	0.009770
864.500000	0.009739
864.250000	0.009711
864.000000	0.009685
863.750000	0.009661
863.500000	0.009635
863.250000	0.009607
863.000000	0.009576
862.750000	0.009543
862.500000	0.009509
862.250000	0.009476
862.000000	0.009446
861.750000	0.009418
861.500000	0.009393
861.250000	0.009370
861.000000	0.009346
860.750000	0.009321
860.500000	0.009293

860.250000	0.009264
860.000000	0.009235
859.750000	0.009205
859.500000	0.009176
859.250000	0.009146
859.000000	0.009116
858.750000	0.009085
858.500000	0.009050
858.250000	0.009015
858.000000	0.008979
857.750000	0.008946
857.500000	0.008917
857.250000	0.008893
857.000000	0.008874
856.750000	0.008859
856.500000	0.008845
856.250000	0.008831
856.000000	0.008815
855.750000	0.008800
855.500000	0.008784
855.250000	0.008770
855.000000	0.008759
854.750000	0.008750
854.500000	0.008742
854.250000	0.008734
854.000000	0.008725

853.750000	0.008713
853.500000	0.008697
853.250000	0.008678
853.000000	0.008655
852.750000	0.008630
852.500000	0.008604
852.250000	0.008578
852.000000	0.008555
851.750000	0.008535
851.500000	0.008519
851.250000	0.008506
851.000000	0.008495
850.750000	0.008484
850.500000	0.008472
850.250000	0.008459
850.000000	0.008445
849.750000	0.008432
849.500000	0.008422
849.250000	0.008417
849.000000	0.008417
848.750000	0.008422
848.500000	0.008428
848.250000	0.008435
848.000000	0.008442
847.750000	0.008446
847.500000	0.008450

847.250000	0.008454
847.000000	0.008460
846.750000	0.008466
846.500000	0.008475
846.250000	0.008485
846.000000	0.008496
845.750000	0.008507
845.500000	0.008519
845.250000	0.008534
845.000000	0.008551
844.750000	0.008571
844.500000	0.008596
844.250000	0.008624
844.000000	0.008654
843.750000	0.008687
843.500000	0.008719
843.250000	0.008750
843.000000	0.008778
842.750000	0.008802
842.500000	0.008821
842.250000	0.008835
842.000000	0.008846
841.750000	0.008856
841.500000	0.008865
841.250000	0.008875
841.000000	0.008888

840.750000	0.008902
840.500000	0.008918
840.250000	0.008934
840.000000	0.008950
839.750000	0.008966
839.500000	0.008981
839.250000	0.008996
839.000000	0.009010
838.750000	0.009023
838.500000	0.009033
838.250000	0.009039
838.000000	0.009042
837.750000	0.009041
837.500000	0.009039
837.250000	0.009038
837.000000	0.009041
836.750000	0.009047
836.500000	0.009057
836.250000	0.009070
836.000000	0.009081
835.750000	0.009091
835.500000	0.009096
835.250000	0.009098
835.000000	0.009096
834.750000	0.009094
834.500000	0.009093

834.250000	0.009093
834.000000	0.009096
833.750000	0.009099
833.500000	0.009102
833.250000	0.009103
833.000000	0.009102
832.750000	0.009096
832.500000	0.009088
832.250000	0.009078
832.000000	0.009068
831.750000	0.009058
831.500000	0.009050
831.250000	0.009044
831.000000	0.009039
830.750000	0.009034
830.500000	0.009026
830.250000	0.009015
830.000000	0.008998
829.750000	0.008975
829.500000	0.008945
829.250000	0.008910
829.000000	0.008871
828.750000	0.008830
828.500000	0.008788
828.250000	0.008749
828.000000	0.008712

827.750000	0.008678
827.500000	0.008645
827.250000	0.008610
827.000000	0.008572
826.750000	0.008529
826.500000	0.008483
826.250000	0.008435
826.000000	0.008389
825.750000	0.008348
825.500000	0.008314
825.250000	0.008287
825.000000	0.008265
824.750000	0.008243
824.500000	0.008220
824.250000	0.008194
824.000000	0.008164
823.750000	0.008134
823.500000	0.008107
823.250000	0.008087
823.000000	0.008077
822.750000	0.008077
822.500000	0.008087
822.250000	0.008103
822.000000	0.008123
821.750000	0.008146
821.500000	0.008169

821.250000	0.008194
821.000000	0.008220
820.750000	0.008247
820.500000	0.008274
820.250000	0.008301
820.000000	0.008328
819.750000	0.008355
819.500000	0.008381
819.250000	0.008409
819.000000	0.008439
818.750000	0.008473
818.500000	0.008510
818.250000	0.008551
818.000000	0.008594
817.750000	0.008640
817.500000	0.008688
817.250000	0.008738
817.000000	0.008789
816.750000	0.008841
816.500000	0.008891
816.250000	0.008938
816.000000	0.008981
815.750000	0.009019
815.500000	0.009050
815.250000	0.009076
815.000000	0.009097

814.750000	0.009115
814.500000	0.009133
814.250000	0.009151
814.000000	0.009168
813.750000	0.009184
813.500000	0.009197
813.250000	0.009205
813.000000	0.009206
812.750000	0.009199
812.500000	0.009184
812.250000	0.009163
812.000000	0.009136
811.750000	0.009106
811.500000	0.009074
811.250000	0.009042
811.000000	0.009008
810.750000	0.008974
810.500000	0.008939
810.250000	0.008903
810.000000	0.008865
809.750000	0.008825
809.500000	0.008784
809.250000	0.008741
809.000000	0.008695
808.750000	0.008649
808.500000	0.008602

808.250000	0.008557
808.000000	0.008516
807.750000	0.008478
807.500000	0.008446
807.250000	0.008417
807.000000	0.008391
806.750000	0.008364
806.500000	0.008334
806.250000	0.008300
806.000000	0.008261
805.750000	0.008219
805.500000	0.008176
805.250000	0.008135
805.000000	0.008096
804.750000	0.008061
804.500000	0.008029
804.250000	0.008002
804.000000	0.007978
803.750000	0.007957
803.500000	0.007940
803.250000	0.007924
803.000000	0.007911
802.750000	0.007898
802.500000	0.007884
802.250000	0.007869
802.000000	0.007851

801.750000	0.007830
801.500000	0.007807
801.250000	0.007782
801.000000	0.007757
800.750000	0.007733
800.500000	0.007710
800.250000	0.007687
800.000000	0.007664
799.750000	0.007641
799.500000	0.007618
799.250000	0.007594
799.000000	0.007569
798.750000	0.007543
798.500000	0.007517
798.250000	0.007491
798.000000	0.007466
797.750000	0.007441
797.500000	0.007418
797.250000	0.007395
797.000000	0.007373
796.750000	0.007350
796.500000	0.007326
796.250000	0.007301
796.000000	0.007275
795.750000	0.007248
795.500000	0.007221

795.250000	0.007195
795.000000	0.007169
794.750000	0.007144
794.500000	0.007119
794.250000	0.007094
794.000000	0.007069
793.750000	0.007045
793.500000	0.007021
793.250000	0.006997
793.000000	0.006974
792.750000	0.006950
792.500000	0.006924
792.250000	0.006898
792.000000	0.006870
791.750000	0.006843
791.500000	0.006818
791.250000	0.006797
791.000000	0.006781
790.750000	0.006768
790.500000	0.006759
790.250000	0.006749
790.000000	0.006737
789.750000	0.006723
789.500000	0.006705
789.250000	0.006685
789.000000	0.006666

788.750000	0.006649
788.500000	0.006636
788.250000	0.006626
788.000000	0.006619
787.750000	0.006612
787.500000	0.006605
787.250000	0.006597
787.000000	0.006587
786.750000	0.006577
786.500000	0.006568
786.250000	0.006561
786.000000	0.006558
785.750000	0.006559
785.500000	0.006566
785.250000	0.006578
785.000000	0.006595
784.750000	0.006617
784.500000	0.006644
784.250000	0.006675
784.000000	0.006708
783.750000	0.006744
783.500000	0.006779
783.250000	0.006813
783.000000	0.006845
782.750000	0.006875
782.500000	0.006904

782.250000	0.006934
782.000000	0.006965
781.750000	0.007001
781.500000	0.007041
781.250000	0.007087
781.000000	0.007136
780.750000	0.007186
780.500000	0.007237
780.250000	0.007285
780.000000	0.007330
779.750000	0.007372
779.500000	0.007411
779.250000	0.007450
779.000000	0.007488
778.750000	0.007526
778.500000	0.007565
778.250000	0.007603
778.000000	0.007639
777.750000	0.007670
777.500000	0.007695
777.250000	0.007713
777.000000	0.007724
776.750000	0.007729
776.500000	0.007730
776.250000	0.007729
776.000000	0.007729

775.750000	0.007730
775.500000	0.007734
775.250000	0.007738
775.000000	0.007740
774.750000	0.007738
774.500000	0.007730
774.250000	0.007714
774.000000	0.007691
773.750000	0.007663
773.500000	0.007631
773.250000	0.007596
773.000000	0.007559
772.750000	0.007520
772.500000	0.007478
772.250000	0.007430
772.000000	0.007377
771.750000	0.007317
771.500000	0.007252
771.250000	0.007185
771.000000	0.007116
770.750000	0.007049
770.500000	0.006985
770.250000	0.006924
770.000000	0.006866
769.750000	0.006811
769.500000	0.006758

769.250000	0.006706
769.000000	0.006656
768.750000	0.006607
768.500000	0.006561
768.250000	0.006518
768.000000	0.006479
767.750000	0.006444
767.500000	0.006413
767.250000	0.006386
767.000000	0.006364
766.750000	0.006347
766.500000	0.006333
766.250000	0.006323
766.000000	0.006316
765.750000	0.006313
765.500000	0.006311
765.250000	0.006312
765.000000	0.006315
764.750000	0.006318
764.500000	0.006323
764.250000	0.006328
764.000000	0.006333
763.750000	0.006339
763.500000	0.006346
763.250000	0.006353
763.000000	0.006360

762.750000	0.006365
762.500000	0.006367
762.250000	0.006364
762.000000	0.006355
761.750000	0.006339
761.500000	0.006315
761.250000	0.006284
761.000000	0.006247
760.750000	0.006206
760.500000	0.006163
760.250000	0.006118
760.000000	0.006074
759.750000	0.006030
759.500000	0.005986
759.250000	0.005943
759.000000	0.005899
758.750000	0.005856
758.500000	0.005815
758.250000	0.005776
758.000000	0.005741
757.750000	0.005710
757.500000	0.005682
757.250000	0.005656
757.000000	0.005630
756.750000	0.005601
756.500000	0.005570

756.250000	0.005537
756.000000	0.005502
755.750000	0.005468
755.500000	0.005437
755.250000	0.005410
755.000000	0.005388
754.750000	0.005371
754.500000	0.005359
754.250000	0.005352
754.000000	0.005348
753.750000	0.005347
753.500000	0.005348
753.250000	0.005349
753.000000	0.005351
752.750000	0.005349
752.500000	0.005345
752.250000	0.005338
752.000000	0.005328
751.750000	0.005318
751.500000	0.005310
751.250000	0.005305
751.000000	0.005305
750.750000	0.005309
750.500000	0.005316
750.250000	0.005324
750.000000	0.005332

749.750000	0.005337
749.500000	0.005340
749.250000	0.005340
749.000000	0.005339
748.750000	0.005337
748.500000	0.005335
748.250000	0.005336
748.000000	0.005339
747.750000	0.005343
747.500000	0.005348
747.250000	0.005352
747.000000	0.005353
746.750000	0.005348
746.500000	0.005337
746.250000	0.005319
746.000000	0.005294
745.750000	0.005266
745.500000	0.005234
745.250000	0.005200
745.000000	0.005165
744.750000	0.005130
744.500000	0.005094
744.250000	0.005058
744.000000	0.005023
743.750000	0.004989
743.500000	0.004958

743.250000	0.004929
743.000000	0.004902
742.750000	0.004874
742.500000	0.004844
742.250000	0.004810
742.000000	0.004773
741.750000	0.004734
741.500000	0.004693
741.250000	0.004653
741.000000	0.004616
740.750000	0.004583
740.500000	0.004555
740.250000	0.004530
740.000000	0.004510
739.750000	0.004493
739.500000	0.004480
739.250000	0.004472
739.000000	0.004467
738.750000	0.004465
738.500000	0.004466
738.250000	0.004466
738.000000	0.004466
737.750000	0.004465
737.500000	0.004462
737.250000	0.004458
737.000000	0.004453

736.750000	0.004449
736.500000	0.004446
736.250000	0.004443
736.000000	0.004440
735.750000	0.004435
735.500000	0.004429
735.250000	0.004422
735.000000	0.004413
734.750000	0.004403
734.500000	0.004392
734.250000	0.004381
734.000000	0.004369
733.750000	0.004356
733.500000	0.004342
733.250000	0.004328
733.000000	0.004315
732.750000	0.004304
732.500000	0.004297
732.250000	0.004294
732.000000	0.004298
731.750000	0.004306
731.500000	0.004319
731.250000	0.004334
731.000000	0.004348
730.750000	0.004361
730.500000	0.004370

730.250000	0.004376
730.000000	0.004379
729.750000	0.004381
729.500000	0.004381
729.250000	0.004382
729.000000	0.004380
728.750000	0.004376
728.500000	0.004366
728.250000	0.004352
728.000000	0.004332
727.750000	0.004309
727.500000	0.004285
727.250000	0.004261
727.000000	0.004241
726.750000	0.004225
726.500000	0.004213
726.250000	0.004204
726.000000	0.004199
725.750000	0.004197
725.500000	0.004199
725.250000	0.004204
725.000000	0.004211
724.750000	0.004218
724.500000	0.004223
724.250000	0.004225
724.000000	0.004221

723.750000	0.004211
723.500000	0.004197
723.250000	0.004180
723.000000	0.004162
722.750000	0.004144
722.500000	0.004128
722.250000	0.004113
722.000000	0.004099
721.750000	0.004085
721.500000	0.004071
721.250000	0.004058
721.000000	0.004045
720.750000	0.004031
720.500000	0.004016
720.250000	0.003999
720.000000	0.003980
719.750000	0.003959
719.500000	0.003937
719.250000	0.003915
719.000000	0.003894
718.750000	0.003875
718.500000	0.003857
718.250000	0.003839
718.000000	0.003822
717.750000	0.003805
717.500000	0.003786

717.250000	0.003768
717.000000	0.003751
716.750000	0.003735
716.500000	0.003720
716.250000	0.003708
716.000000	0.003697
715.750000	0.003688
715.500000	0.003680
715.250000	0.003672
715.000000	0.003666
714.750000	0.003661
714.500000	0.003656
714.250000	0.003651
714.000000	0.003646
713.750000	0.003639
713.500000	0.003632
713.250000	0.003626
713.000000	0.003619
712.750000	0.003615
712.500000	0.003611
712.250000	0.003607
712.000000	0.003602
711.750000	0.003596
711.500000	0.003589
711.250000	0.003580
711.000000	0.003571

710.750000	0.003563
710.500000	0.003558
710.250000	0.003554
710.000000	0.003552
709.750000	0.003550
709.500000	0.003549
709.250000	0.003548
709.000000	0.003547
708.750000	0.003546
708.500000	0.003546
708.250000	0.003548
708.000000	0.003554
707.750000	0.003562
707.500000	0.003574
707.250000	0.003589
707.000000	0.003606
706.750000	0.003623
706.500000	0.003636
706.250000	0.003643
706.000000	0.003641
705.750000	0.003628
705.500000	0.003606
705.250000	0.003577
705.000000	0.003545
704.750000	0.003514
704.500000	0.003489

704.250000	0.003472
704.000000	0.003463
703.750000	0.003461
703.500000	0.003465
703.250000	0.003471
703.000000	0.003479
702.750000	0.003486
702.500000	0.003494
702.250000	0.003501
702.000000	0.003507
701.750000	0.003511
701.500000	0.003513
701.250000	0.003510
701.000000	0.003504
700.750000	0.003493
700.500000	0.003480
700.250000	0.003467
700.000000	0.003457
699.750000	0.003452
699.500000	0.003453
699.250000	0.003461
699.000000	0.003474
698.750000	0.003490
698.500000	0.003506
698.250000	0.003520
698.000000	0.003530

697.750000	0.003535
697.500000	0.003534
697.250000	0.003529
697.000000	0.003519
696.750000	0.003507
696.500000	0.003493
696.250000	0.003479
696.000000	0.003466
695.750000	0.003455
695.500000	0.003449
695.250000	0.003447
695.000000	0.003448
694.750000	0.003450
694.500000	0.003452
694.250000	0.003452
694.000000	0.003447
693.750000	0.003439
693.500000	0.003428
693.250000	0.003417
693.000000	0.003408
692.750000	0.003402
692.500000	0.003397
692.250000	0.003393
692.000000	0.003386
691.750000	0.003375
691.500000	0.003358

691.250000	0.003335
691.000000	0.003308
690.750000	0.003281
690.500000	0.003255
690.250000	0.003233
690.000000	0.003216
689.750000	0.003204
689.500000	0.003196
689.250000	0.003192
689.000000	0.003191
688.750000	0.003191
688.500000	0.003190
688.250000	0.003187
688.000000	0.003181
687.750000	0.003172
687.500000	0.003160
687.250000	0.003146
687.000000	0.003131
686.750000	0.003116
686.500000	0.003103
686.250000	0.003090
686.000000	0.003079
685.750000	0.003069
685.500000	0.003060
685.250000	0.003054
685.000000	0.003050

684.750000	0.003051
684.500000	0.003055
684.250000	0.003064
684.000000	0.003073
683.750000	0.003082
683.500000	0.003088
683.250000	0.003089
683.000000	0.003086
682.750000	0.003078
682.500000	0.003067
682.250000	0.003055
682.000000	0.003044
681.750000	0.003033
681.500000	0.003024
681.250000	0.003015
681.000000	0.003007
680.750000	0.003000
680.500000	0.002993
680.250000	0.002987
680.000000	0.002984
679.750000	0.002985
679.500000	0.002989
679.250000	0.002997
679.000000	0.003005
678.750000	0.003013
678.500000	0.003018

678.250000	0.003018
678.000000	0.003012
677.750000	0.003003
677.500000	0.002990
677.250000	0.002977
677.000000	0.002966
676.750000	0.002957
676.500000	0.002953
676.250000	0.002952
676.000000	0.002953
675.750000	0.002956
675.500000	0.002961
675.250000	0.002967
675.000000	0.002975
674.750000	0.002983
674.500000	0.002989
674.250000	0.002990
674.000000	0.002986
673.750000	0.002974
673.500000	0.002954
673.250000	0.002931
673.000000	0.002907
672.750000	0.002887
672.500000	0.002874
672.250000	0.002868
672.000000	0.002870

671.750000	0.002876
671.500000	0.002881
671.250000	0.002881
671.000000	0.002874
670.750000	0.002860
670.500000	0.002836
670.250000	0.002802
670.000000	0.002764
669.750000	0.002727
669.500000	0.002696
669.250000	0.002673
669.000000	0.002666
668.750000	0.002693
668.500000	0.002766
668.250000	0.002869
668.000000	0.002965
667.750000	0.003016
667.500000	0.003013
667.250000	0.002974
667.000000	0.002920
666.750000	0.002866
666.500000	0.002816
666.250000	0.002776
666.000000	0.002750
665.750000	0.002737
665.500000	0.002733

665.250000	0.002734
665.000000	0.002734
664.750000	0.002734
664.500000	0.002732
664.250000	0.002727
664.000000	0.002720
663.750000	0.002714
663.500000	0.002708
663.250000	0.002703
663.000000	0.002699
662.750000	0.002694
662.500000	0.002690
662.250000	0.002684
662.000000	0.002678
661.750000	0.002669
661.500000	0.002658
661.250000	0.002646
661.000000	0.002633
660.750000	0.002621
660.500000	0.002612
660.250000	0.002606
660.000000	0.002603
659.750000	0.002605
659.500000	0.002608
659.250000	0.002612
659.000000	0.002616

658.750000	0.002616
658.500000	0.002613
658.250000	0.002606
658.000000	0.002596
657.750000	0.002583
657.500000	0.002569
657.250000	0.002556
657.000000	0.002544
656.750000	0.002536
656.500000	0.002531
656.250000	0.002528
656.000000	0.002525
655.750000	0.002522
655.500000	0.002517
655.250000	0.002510
655.000000	0.002501
654.750000	0.002492
654.500000	0.002483
654.250000	0.002475
654.000000	0.002468
653.750000	0.002460
653.500000	0.002452
653.250000	0.002441
653.000000	0.002427
652.750000	0.002411
652.500000	0.002393

652.250000	0.002375
652.000000	0.002355
651.750000	0.002336
651.500000	0.002317
651.250000	0.002300
651.000000	0.002285
650.750000	0.002273
650.500000	0.002264
650.250000	0.002258
650.000000	0.002254
649.750000	0.002252
649.500000	0.002251
649.250000	0.002250
649.000000	0.002249
648.750000	0.002247
648.500000	0.002244
648.250000	0.002239
648.000000	0.002231
647.750000	0.002217
647.500000	0.002198
647.250000	0.002175
647.000000	0.002148
646.750000	0.002121
646.500000	0.002096
646.250000	0.002075
646.000000	0.002058

645.750000	0.002044
645.500000	0.002031
645.250000	0.002018
645.000000	0.002002
644.750000	0.001986
644.500000	0.001968
644.250000	0.001953
644.000000	0.001942
643.750000	0.001936
643.500000	0.001936
643.250000	0.001939
643.000000	0.001945
642.750000	0.001950
642.500000	0.001954
642.250000	0.001954
642.000000	0.001951
641.750000	0.001944
641.500000	0.001934
641.250000	0.001921
641.000000	0.001905
640.750000	0.001888
640.500000	0.001869
640.250000	0.001850
640.000000	0.001832
639.750000	0.001815
639.500000	0.001801

639.250000	0.001790
639.000000	0.001783
638.750000	0.001781
638.500000	0.001781
638.250000	0.001783
638.000000	0.001785
637.750000	0.001785
637.500000	0.001783
637.250000	0.001779
637.000000	0.001774
636.750000	0.001769
636.500000	0.001765
636.250000	0.001764
636.000000	0.001764
635.750000	0.001763
635.500000	0.001760
635.250000	0.001753
635.000000	0.001743
634.750000	0.001729
634.500000	0.001716
634.250000	0.001705
634.000000	0.001696
633.750000	0.001692
633.500000	0.001688
633.250000	0.001685
633.000000	0.001680

632.750000	0.001671
632.500000	0.001658
632.250000	0.001644
632.000000	0.001629
631.750000	0.001614
631.500000	0.001600
631.250000	0.001587
631.000000	0.001575
630.750000	0.001562
630.500000	0.001550
630.250000	0.001538
630.000000	0.001528
629.750000	0.001522
629.500000	0.001519
629.250000	0.001519
629.000000	0.001520
628.750000	0.001520
628.500000	0.001518
628.250000	0.001511
628.000000	0.001499
627.750000	0.001483
627.500000	0.001466
627.250000	0.001451
627.000000	0.001438
626.750000	0.001429
626.500000	0.001422

626.250000	0.001418
626.000000	0.001413
625.750000	0.001407
625.500000	0.001401
625.250000	0.001396
625.000000	0.001395
624.750000	0.001401
624.500000	0.001416
624.250000	0.001437
624.000000	0.001464
623.750000	0.001490
623.500000	0.001513
623.250000	0.001528
623.000000	0.001536
622.750000	0.001538
622.500000	0.001535
622.250000	0.001530
622.000000	0.001525
621.750000	0.001518
621.500000	0.001509
621.250000	0.001496
621.000000	0.001478
620.750000	0.001457
620.500000	0.001437
620.250000	0.001424
620.000000	0.001421

619.750000	0.001431
619.500000	0.001452
619.250000	0.001479
619.000000	0.001506
618.750000	0.001529
618.500000	0.001542
618.250000	0.001546
618.000000	0.001540
617.750000	0.001527
617.500000	0.001512
617.250000	0.001497
617.000000	0.001483
616.750000	0.001471
616.500000	0.001462
616.250000	0.001456
616.000000	0.001453
615.750000	0.001454
615.500000	0.001458
615.250000	0.001466
615.000000	0.001475
614.750000	0.001484
614.500000	0.001490
614.250000	0.001493
614.000000	0.001492
613.750000	0.001489
613.500000	0.001486

613.250000	0.001482
613.000000	0.001481
612.750000	0.001481
612.500000	0.001483
612.250000	0.001484
612.000000	0.001485
611.750000	0.001482
611.500000	0.001475
611.250000	0.001462
611.000000	0.001445
610.750000	0.001423
610.500000	0.001401
610.250000	0.001381
610.000000	0.001366
609.750000	0.001358
609.500000	0.001358
609.250000	0.001366
609.000000	0.001379
608.750000	0.001395
608.500000	0.001410
608.250000	0.001422
608.000000	0.001431
607.750000	0.001435
607.500000	0.001436
607.250000	0.001435
607.000000	0.001434

606.750000	0.001434
606.500000	0.001434
606.250000	0.001434
606.000000	0.001434
605.750000	0.001434
605.500000	0.001432
605.250000	0.001430
605.000000	0.001426
604.750000	0.001421
604.500000	0.001416
604.250000	0.001411
604.000000	0.001407
603.750000	0.001403
603.500000	0.001398
603.250000	0.001393
603.000000	0.001387
602.750000	0.001380
602.500000	0.001374
602.250000	0.001371
602.000000	0.001371
601.750000	0.001377
601.500000	0.001389
601.250000	0.001406
601.000000	0.001427
600.750000	0.001450
600.500000	0.001472

600.250000	0.001491
600.000000	0.001507
599.750000	0.001517
599.500000	0.001523
599.250000	0.001525
599.000000	0.001523
598.750000	0.001519
598.500000	0.001514
598.250000	0.001509
598.000000	0.001504
597.750000	0.001501
597.500000	0.001498
597.250000	0.001496
597.000000	0.001495
596.750000	0.001495
596.500000	0.001494
596.250000	0.001495
596.000000	0.001496
595.750000	0.001499
595.500000	0.001503
595.250000	0.001506
595.000000	0.001509
594.750000	0.001510
594.500000	0.001509
594.250000	0.001508
594.000000	0.001507

593.750000	0.001507
593.500000	0.001508
593.250000	0.001511
593.000000	0.001514
592.750000	0.001516
592.500000	0.001516
592.250000	0.001514
592.000000	0.001510
591.750000	0.001506
591.500000	0.001502
591.250000	0.001498
591.000000	0.001496
590.750000	0.001493
590.500000	0.001490
590.250000	0.001485
590.000000	0.001477
589.750000	0.001466
589.500000	0.001454
589.250000	0.001440
589.000000	0.001427
588.750000	0.001417
588.500000	0.001410
588.250000	0.001409
588.000000	0.001413
587.750000	0.001424
587.500000	0.001438

587.250000	0.001455
587.000000	0.001470
586.750000	0.001483
586.500000	0.001490
586.250000	0.001491
586.000000	0.001487
585.750000	0.001481
585.500000	0.001473
585.250000	0.001466
585.000000	0.001461
584.750000	0.001459
584.500000	0.001458
584.250000	0.001458
584.000000	0.001459
583.750000	0.001459
583.500000	0.001458
583.250000	0.001457
583.000000	0.001455
582.750000	0.001451
582.500000	0.001445
582.250000	0.001437
582.000000	0.001427
581.750000	0.001414
581.500000	0.001399
581.250000	0.001384
581.000000	0.001369

580.750000	0.001355
580.500000	0.001344
580.250000	0.001339
580.000000	0.001340
579.750000	0.001349
579.500000	0.001365
579.250000	0.001387
579.000000	0.001413
578.750000	0.001440
578.500000	0.001465
578.250000	0.001486
578.000000	0.001500
577.750000	0.001508
577.500000	0.001511
577.250000	0.001510
577.000000	0.001508
576.750000	0.001505
576.500000	0.001501
576.250000	0.001495
576.000000	0.001485
575.750000	0.001471
575.500000	0.001455
575.250000	0.001439
575.000000	0.001425
574.750000	0.001416
574.500000	0.001412

574.250000	0.001413
574.000000	0.001419
573.750000	0.001427
573.500000	0.001436
573.250000	0.001445
573.000000	0.001453
572.750000	0.001458
572.500000	0.001460
572.250000	0.001460
572.000000	0.001455
571.750000	0.001446
571.500000	0.001433
571.250000	0.001416
571.000000	0.001397
570.750000	0.001377
570.500000	0.001359
570.250000	0.001347
570.000000	0.001341
569.750000	0.001342
569.500000	0.001349
569.250000	0.001359
569.000000	0.001370
568.750000	0.001377
568.500000	0.001379
568.250000	0.001375
568.000000	0.001365

567.750000	0.001351
567.500000	0.001335
567.250000	0.001318
567.000000	0.001303
566.750000	0.001289
566.500000	0.001277
566.250000	0.001266
566.000000	0.001257
565.750000	0.001251
565.500000	0.001247
565.250000	0.001248
565.000000	0.001254
564.750000	0.001265
564.500000	0.001279
564.250000	0.001294
564.000000	0.001308
563.750000	0.001319
563.500000	0.001327
563.250000	0.001329
563.000000	0.001326
562.750000	0.001318
562.500000	0.001307
562.250000	0.001292
562.000000	0.001275
561.750000	0.001259
561.500000	0.001247

561.250000	0.001241
561.000000	0.001244
560.750000	0.001256
560.500000	0.001278
560.250000	0.001308
560.000000	0.001342
559.750000	0.001377
559.500000	0.001410
559.250000	0.001436
559.000000	0.001453
558.750000	0.001458
558.500000	0.001453
558.250000	0.001436
558.000000	0.001410
557.750000	0.001380
557.500000	0.001349
557.250000	0.001324
557.000000	0.001306
556.750000	0.001299
556.500000	0.001300
556.250000	0.001307
556.000000	0.001314
555.750000	0.001317
555.500000	0.001314
555.250000	0.001304
555.000000	0.001292

554.750000	0.001282
554.500000	0.001279
554.250000	0.001284
554.000000	0.001297
553.750000	0.001313
553.500000	0.001327
553.250000	0.001334
553.000000	0.001331
552.750000	0.001317
552.500000	0.001297
552.250000	0.001274
552.000000	0.001254
551.750000	0.001240
551.500000	0.001231
551.250000	0.001226
551.000000	0.001219
550.750000	0.001205
550.500000	0.001181
550.250000	0.001146
550.000000	0.001104
549.750000	0.001061
549.500000	0.001023
549.250000	0.000998
549.000000	0.000987
548.750000	0.000993
548.500000	0.001012

548.250000	0.001039
548.000000	0.001068
547.750000	0.001091
547.500000	0.001105
547.250000	0.001106
547.000000	0.001094
546.750000	0.001073
546.500000	0.001047
546.250000	0.001024
546.000000	0.001006
545.750000	0.000998
545.500000	0.000999
545.250000	0.001007
545.000000	0.001017
544.750000	0.001026
544.500000	0.001029
544.250000	0.001024
544.000000	0.001013
543.750000	0.000998
543.500000	0.000982
543.250000	0.000969
543.000000	0.000962
542.750000	0.000962
542.500000	0.000969
542.250000	0.000982
542.000000	0.000996

541.750000	0.001009
541.500000	0.001017
541.250000	0.001019
541.000000	0.001016
540.750000	0.001010
540.500000	0.001001
540.250000	0.000992
540.000000	0.000984
539.750000	0.000974
539.500000	0.000962
539.250000	0.000945
539.000000	0.000922
538.750000	0.000893
538.500000	0.000861
538.250000	0.000828
538.000000	0.000798
537.750000	0.000773
537.500000	0.000754
537.250000	0.000741
537.000000	0.000732
536.750000	0.000723
536.500000	0.000714
536.250000	0.000703
536.000000	0.000692
535.750000	0.000684
535.500000	0.000681

535.250000	0.000686
535.000000	0.000698
534.750000	0.000717
534.500000	0.000738
534.250000	0.000757
534.000000	0.000772
533.750000	0.000780
533.500000	0.000780
533.250000	0.000773
533.000000	0.000760
532.750000	0.000743
532.500000	0.000723
532.250000	0.000699
532.000000	0.000673
531.750000	0.000645
531.500000	0.000620
531.250000	0.000602
531.000000	0.000595
530.750000	0.000604
530.500000	0.000629
530.250000	0.000669
530.000000	0.000717
529.750000	0.000765
529.500000	0.000806
529.250000	0.000833
529.000000	0.000846

528.750000	0.000845
528.500000	0.000835
528.250000	0.000820
528.000000	0.000807
527.750000	0.000795
527.500000	0.000785
527.250000	0.000775
527.000000	0.000762
526.750000	0.000745
526.500000	0.000725
526.250000	0.000705
526.000000	0.000689
525.750000	0.000680
525.500000	0.000682
525.250000	0.000692
525.000000	0.000710
524.750000	0.000733
524.500000	0.000757
524.250000	0.000782
524.000000	0.000805
523.750000	0.000823
523.500000	0.000833
523.250000	0.000833
523.000000	0.000819
522.750000	0.000794
522.500000	0.000760

522.250000	0.000722
522.000000	0.000686
521.750000	0.000658
521.500000	0.000640
521.250000	0.000633
521.000000	0.000633
520.750000	0.000637
520.500000	0.000641
520.250000	0.000642
520.000000	0.000639
519.750000	0.000633
519.500000	0.000628
519.250000	0.000626
519.000000	0.000630
518.750000	0.000642
518.500000	0.000660
518.250000	0.000681
518.000000	0.000704
517.750000	0.000724
517.500000	0.000739
517.250000	0.000746
517.000000	0.000746
516.750000	0.000739
516.500000	0.000729
516.250000	0.000718
516.000000	0.000711

515.750000	0.000708
515.500000	0.000710
515.250000	0.000716
515.000000	0.000726
514.750000	0.000737
514.500000	0.000748
514.250000	0.000756
514.000000	0.000761
513.750000	0.000762
513.500000	0.000759
513.250000	0.000754
513.000000	0.000747
512.750000	0.000741
512.500000	0.000738
512.250000	0.000738
512.000000	0.000744
511.750000	0.000756
511.500000	0.000774
511.250000	0.000795
511.000000	0.000819
510.750000	0.000841
510.500000	0.000858
510.250000	0.000865
510.000000	0.000859
509.750000	0.000839
509.500000	0.000807

509.250000	0.000768
509.000000	0.000728
508.750000	0.000692
508.500000	0.000667
508.250000	0.000658
508.000000	0.000668
507.750000	0.000697
507.500000	0.000743
507.250000	0.000800
507.000000	0.000859
506.750000	0.000912
506.500000	0.000954
506.250000	0.000981
506.000000	0.000995
505.750000	0.000999
505.500000	0.000997
505.250000	0.000992
505.000000	0.000984
504.750000	0.000976
504.500000	0.000969
504.250000	0.000961
504.000000	0.000953
503.750000	0.000946
503.500000	0.000939
503.250000	0.000934
503.000000	0.000934

502.750000	0.000941
502.500000	0.000958
502.250000	0.000984
502.000000	0.001019
501.750000	0.001059
501.500000	0.001100
501.250000	0.001138
501.000000	0.001166
500.750000	0.001183
500.500000	0.001185
500.250000	0.001172
500.000000	0.001147
499.750000	0.001118
499.500000	0.001093
499.250000	0.001085
499.000000	0.001102
498.750000	0.001146
498.500000	0.001214
498.250000	0.001296
498.000000	0.001379
497.750000	0.001448
497.500000	0.001496
497.250000	0.001519
497.000000	0.001522
496.750000	0.001514
496.500000	0.001505

496.250000	0.001506
496.000000	0.001524
495.750000	0.001560
495.500000	0.001612
495.250000	0.001676
495.000000	0.001746
494.750000	0.001814
494.500000	0.001875
494.250000	0.001923
494.000000	0.001955
493.750000	0.001968
493.500000	0.001964
493.250000	0.001947
493.000000	0.001923
492.750000	0.001899
492.500000	0.001884
492.250000	0.001882
492.000000	0.001894
491.750000	0.001919
491.500000	0.001951
491.250000	0.001985
491.000000	0.002018
490.750000	0.002050
490.500000	0.002081
490.250000	0.002113
490.000000	0.002150

489.750000	0.002191
489.500000	0.002237
489.250000	0.002286
489.000000	0.002337
488.750000	0.002387
488.500000	0.002435
488.250000	0.002480
488.000000	0.002518
487.750000	0.002549
487.500000	0.002571
487.250000	0.002583
487.000000	0.002587
486.750000	0.002589
486.500000	0.002593
486.250000	0.002606
486.000000	0.002632
485.750000	0.002670
485.500000	0.002718
485.250000	0.002769
485.000000	0.002818
484.750000	0.002859
484.500000	0.002889
484.250000	0.002909
484.000000	0.002920
483.750000	0.002931
483.500000	0.002947

483.250000	0.002974
483.000000	0.003013
482.750000	0.003062
482.500000	0.003116
482.250000	0.003172
482.000000	0.003222
481.750000	0.003263
481.500000	0.003293
481.250000	0.003310
481.000000	0.003318
480.750000	0.003322
480.500000	0.003326
480.250000	0.003335
480.000000	0.003351
479.750000	0.003374
479.500000	0.003402
479.250000	0.003436
479.000000	0.003474
478.750000	0.003517
478.500000	0.003565
478.250000	0.003618
478.000000	0.003673
477.750000	0.003728
477.500000	0.003778
477.250000	0.003822
477.000000	0.003859

476.750000	0.003891
476.500000	0.003920
476.250000	0.003948
476.000000	0.003975
475.750000	0.003999
475.500000	0.004017
475.250000	0.004024
475.000000	0.004021
474.750000	0.004008
474.500000	0.003990
474.250000	0.003971
474.000000	0.003955
473.750000	0.003948
473.500000	0.003954
473.250000	0.003979
473.000000	0.004027
472.750000	0.004100
472.500000	0.004192
472.250000	0.004292
472.000000	0.004385
471.750000	0.004460
471.500000	0.004511
471.250000	0.004538
471.000000	0.004541
470.750000	0.004525
470.500000	0.004497

470.250000	0.004463
470.000000	0.004432
469.750000	0.004412
469.500000	0.004407
469.250000	0.004419
469.000000	0.004446
468.750000	0.004486
468.500000	0.004536
468.250000	0.004592
468.000000	0.004652
467.750000	0.004714
467.500000	0.004774
467.250000	0.004831
467.000000	0.004879
466.750000	0.004915
466.500000	0.004938
466.250000	0.004945
466.000000	0.004941
465.750000	0.004929
465.500000	0.004917
465.250000	0.004910
465.000000	0.004912
464.750000	0.004924
464.500000	0.004941
464.250000	0.004957
464.000000	0.004965

463.750000	0.004960
463.500000	0.004939
463.250000	0.004906
463.000000	0.004869
462.750000	0.004836
462.500000	0.004816
462.250000	0.004818
462.000000	0.004843
461.750000	0.004889
461.500000	0.004943
461.250000	0.004994
461.000000	0.005027
460.750000	0.005038
460.500000	0.005026
460.250000	0.004997
460.000000	0.004959
459.750000	0.004918
459.500000	0.004882
459.250000	0.004852
459.000000	0.004831
458.750000	0.004821
458.500000	0.004825
458.250000	0.004845
458.000000	0.004879
457.750000	0.004923
457.500000	0.004966

457.250000	0.005002
457.000000	0.005030
456.750000	0.005051
456.500000	0.005066
456.250000	0.005073
456.000000	0.005067
455.750000	0.005047
455.500000	0.005011
455.250000	0.004965
455.000000	0.004914
454.750000	0.004869
454.500000	0.004839
454.250000	0.004831
454.000000	0.004846
453.750000	0.004881
453.500000	0.004927
453.250000	0.004971
453.000000	0.005002
452.750000	0.005014
452.500000	0.005007
452.250000	0.004989
452.000000	0.004969
451.750000	0.004955
451.500000	0.004949
451.250000	0.004950
451.000000	0.004953

450.750000	0.004951
450.500000	0.004939
450.250000	0.004912
450.000000	0.004871
449.750000	0.004817
449.500000	0.004757
449.250000	0.004696
449.000000	0.004642
448.750000	0.004599
448.500000	0.004569
448.250000	0.004553
448.000000	0.004547
447.750000	0.004547
447.500000	0.004551
447.250000	0.004555
447.000000	0.004560
446.750000	0.004568
446.500000	0.004583
446.250000	0.004608
446.000000	0.004641
445.750000	0.004678
445.500000	0.004713
445.250000	0.004739
445.000000	0.004753
444.750000	0.004756
444.500000	0.004750

444.250000	0.004738
444.000000	0.004727
443.750000	0.004717
443.500000	0.004708
443.250000	0.004696
443.000000	0.004678
442.750000	0.004651
442.500000	0.004617
442.250000	0.004579
442.000000	0.004543
441.750000	0.004510
441.500000	0.004478
441.250000	0.004443
441.000000	0.004401
440.750000	0.004348
440.500000	0.004284
440.250000	0.004215
440.000000	0.004147
439.750000	0.004091
439.500000	0.004055
439.250000	0.004042
439.000000	0.004053
438.750000	0.004080
438.500000	0.004113
438.250000	0.004141
438.000000	0.004155

437.750000	0.004152
437.500000	0.004130
437.250000	0.004094
437.000000	0.004049
436.750000	0.004006
436.500000	0.003973
436.250000	0.003956
436.000000	0.003959
435.750000	0.003977
435.500000	0.004004
435.250000	0.004032
435.000000	0.004052
434.750000	0.004060
434.500000	0.004052
434.250000	0.004031
434.000000	0.003998
433.750000	0.003958
433.500000	0.003917
433.250000	0.003882
433.000000	0.003855
432.750000	0.003840
432.500000	0.003837
432.250000	0.003847
432.000000	0.003865
431.750000	0.003888
431.500000	0.003908

431.250000	0.003921
431.000000	0.003921
430.750000	0.003907
430.500000	0.003880
430.250000	0.003846
430.000000	0.003806
429.750000	0.003766
429.500000	0.003728
429.250000	0.003695
429.000000	0.003667
428.750000	0.003644
428.500000	0.003628
428.250000	0.003615
428.000000	0.003604
427.750000	0.003590
427.500000	0.003570
427.250000	0.003545
427.000000	0.003518
426.750000	0.003496
426.500000	0.003486
426.250000	0.003495
426.000000	0.003522
425.750000	0.003561
425.500000	0.003591
425.250000	0.003593
425.000000	0.003552

424.750000	0.003468
424.500000	0.003353
424.250000	0.003224
424.000000	0.003104
423.750000	0.003017
423.500000	0.002982
423.250000	0.003004
423.000000	0.003068
422.750000	0.003143
422.500000	0.003195
422.250000	0.003202
422.000000	0.003161
421.750000	0.003085
421.500000	0.002989
421.250000	0.002884
421.000000	0.002773
420.750000	0.002660
420.500000	0.002548
420.250000	0.002437
420.000000	0.002331
419.750000	0.002254
419.500000	0.002254
419.250000	0.002387
419.000000	0.002673
418.750000	0.003051
418.500000	0.003405

418.250000	0.003632
418.000000	0.003703
417.750000	0.003655
417.500000	0.003535
417.250000	0.003371
417.000000	0.003181
416.750000	0.002984
416.500000	0.002795
416.250000	0.002627
416.000000	0.002489
415.750000	0.002388
415.500000	0.002328
415.250000	0.002306
415.000000	0.002313
414.750000	0.002337
414.500000	0.002368
414.250000	0.002395
414.000000	0.002411
413.750000	0.002410
413.500000	0.002388
413.250000	0.002346
413.000000	0.002286
412.750000	0.002215
412.500000	0.002145
412.250000	0.002084
412.000000	0.002039

411.750000	0.002010
411.500000	0.001993
411.250000	0.001977
411.000000	0.001956
410.750000	0.001930
410.500000	0.001904
410.250000	0.001892
410.000000	0.001901
409.750000	0.001934
409.500000	0.001977
409.250000	0.002005
409.000000	0.001988
408.750000	0.001905
408.500000	0.001754
408.250000	0.001558
408.000000	0.001359
407.750000	0.001208
407.500000	0.001150
407.250000	0.001209
407.000000	0.001378
406.750000	0.001621
406.500000	0.001886
406.250000	0.002116
406.000000	0.002267
405.750000	0.002322
405.500000	0.002285

405.250000	0.002182
405.000000	0.002049
404.750000	0.001918
404.500000	0.001813
404.250000	0.001740
404.000000	0.001694
403.750000	0.001657
403.500000	0.001609
403.250000	0.001531
403.000000	0.001413
402.750000	0.001253
402.500000	0.001062
402.250000	0.000859
402.000000	0.000667
401.750000	0.000505
401.500000	0.000393
401.250000	0.000345
401.000000	0.000370
400.750000	0.000458
400.500000	0.000583
400.250000	0.000719
400.000000	0.000853

A1_2mm_bdown

A1_2mm_c

PE IR SUBTECH SPECTRUM ASCII PEDS 4.00

-1

A1_2mm_c_1_1_1.ASC

21/07/23

16:20:39.00

21/12/06

15:42:15.00

ir

Sample 021 By ir Date venerd, luglio 23 2021

400.000000

#HDR

-1

-1

#GR

cm-1

A

1.0

0.0

4000.000000

-0.250000

14401

8

0.038050

0.000257zaz

#DATA

4000.000000 0.000512

3999.750000	0.000521
3999.500000	0.000531
3999.250000	0.000541
3999.000000	0.000549
3998.750000	0.000556
3998.500000	0.000560
3998.250000	0.000563
3998.000000	0.000562
3997.750000	0.000558
3997.500000	0.000550
3997.250000	0.000539
3997.000000	0.000526
3996.750000	0.000513
3996.500000	0.000503
3996.250000	0.000497
3996.000000	0.000497
3995.750000	0.000504
3995.500000	0.000516
3995.250000	0.000533
3995.000000	0.000551
3994.750000	0.000567
3994.500000	0.000581
3994.250000	0.000591
3994.000000	0.000598
3993.750000	0.000602
3993.500000	0.000604

3993.250000	0.000605
3993.000000	0.000606
3992.750000	0.000605
3992.500000	0.000602
3992.250000	0.000599
3992.000000	0.000594
3991.750000	0.000590
3991.500000	0.000587
3991.250000	0.000587
3991.000000	0.000593
3990.750000	0.000603
3990.500000	0.000618
3990.250000	0.000635
3990.000000	0.000654
3989.750000	0.000670
3989.500000	0.000684
3989.250000	0.000693
3989.000000	0.000697
3988.750000	0.000698
3988.500000	0.000695
3988.250000	0.000690
3988.000000	0.000683
3987.750000	0.000673
3987.500000	0.000662
3987.250000	0.000648
3987.000000	0.000632

3986.750000	0.000616
3986.500000	0.000600
3986.250000	0.000588
3986.000000	0.000579
3985.750000	0.000574
3985.500000	0.000573
3985.250000	0.000574
3985.000000	0.000575
3984.750000	0.000573
3984.500000	0.000567
3984.250000	0.000557
3984.000000	0.000544
3983.750000	0.000529
3983.500000	0.000515
3983.250000	0.000505
3983.000000	0.000500
3982.750000	0.000502
3982.500000	0.000508
3982.250000	0.000519
3982.000000	0.000532
3981.750000	0.000546
3981.500000	0.000560
3981.250000	0.000572
3981.000000	0.000582
3980.750000	0.000590
3980.500000	0.000596

3980.250000	0.000598
3980.000000	0.000598
3979.750000	0.000596
3979.500000	0.000592
3979.250000	0.000586
3979.000000	0.000580
3978.750000	0.000574
3978.500000	0.000568
3978.250000	0.000562
3978.000000	0.000557
3977.750000	0.000553
3977.500000	0.000550
3977.250000	0.000549
3977.000000	0.000551
3976.750000	0.000555
3976.500000	0.000560
3976.250000	0.000565
3976.000000	0.000570
3975.750000	0.000574
3975.500000	0.000577
3975.250000	0.000579
3975.000000	0.000582
3974.750000	0.000588
3974.500000	0.000598
3974.250000	0.000612
3974.000000	0.000631

3973.750000	0.000652
3973.500000	0.000673
3973.250000	0.000691
3973.000000	0.000703
3972.750000	0.000708
3972.500000	0.000705
3972.250000	0.000696
3972.000000	0.000685
3971.750000	0.000673
3971.500000	0.000664
3971.250000	0.000658
3971.000000	0.000656
3970.750000	0.000657
3970.500000	0.000659
3970.250000	0.000659
3970.000000	0.000655
3969.750000	0.000648
3969.500000	0.000636
3969.250000	0.000622
3969.000000	0.000609
3968.750000	0.000597
3968.500000	0.000590
3968.250000	0.000588
3968.000000	0.000591
3967.750000	0.000598
3967.500000	0.000608

3967.250000	0.000617
3967.000000	0.000624
3966.750000	0.000627
3966.500000	0.000625
3966.250000	0.000618
3966.000000	0.000607
3965.750000	0.000593
3965.500000	0.000578
3965.250000	0.000567
3965.000000	0.000560
3964.750000	0.000560
3964.500000	0.000567
3964.250000	0.000580
3964.000000	0.000599
3963.750000	0.000619
3963.500000	0.000640
3963.250000	0.000657
3963.000000	0.000669
3962.750000	0.000674
3962.500000	0.000673
3962.250000	0.000665
3962.000000	0.000654
3961.750000	0.000641
3961.500000	0.000629
3961.250000	0.000620
3961.000000	0.000615

3960.750000	0.000616
3960.500000	0.000620
3960.250000	0.000627
3960.000000	0.000635
3959.750000	0.000643
3959.500000	0.000650
3959.250000	0.000655
3959.000000	0.000658
3958.750000	0.000659
3958.500000	0.000659
3958.250000	0.000658
3958.000000	0.000656
3957.750000	0.000654
3957.500000	0.000649
3957.250000	0.000644
3957.000000	0.000637
3956.750000	0.000628
3956.500000	0.000618
3956.250000	0.000606
3956.000000	0.000594
3955.750000	0.000582
3955.500000	0.000570
3955.250000	0.000560
3955.000000	0.000551
3954.750000	0.000546
3954.500000	0.000544

3954.250000	0.000546
3954.000000	0.000552
3953.750000	0.000561
3953.500000	0.000570
3953.250000	0.000578
3953.000000	0.000583
3952.750000	0.000584
3952.500000	0.000581
3952.250000	0.000577
3952.000000	0.000573
3951.750000	0.000572
3951.500000	0.000575
3951.250000	0.000584
3951.000000	0.000599
3950.750000	0.000618
3950.500000	0.000640
3950.250000	0.000662
3950.000000	0.000685
3949.750000	0.000708
3949.500000	0.000732
3949.250000	0.000756
3949.000000	0.000779
3948.750000	0.000800
3948.500000	0.000815
3948.250000	0.000823
3948.000000	0.000820

3947.750000	0.000808
3947.500000	0.000790
3947.250000	0.000767
3947.000000	0.000746
3946.750000	0.000730
3946.500000	0.000720
3946.250000	0.000716
3946.000000	0.000717
3945.750000	0.000721
3945.500000	0.000726
3945.250000	0.000731
3945.000000	0.000735
3944.750000	0.000738
3944.500000	0.000740
3944.250000	0.000740
3944.000000	0.000737
3943.750000	0.000729
3943.500000	0.000715
3943.250000	0.000693
3943.000000	0.000666
3942.750000	0.000639
3942.500000	0.000616
3942.250000	0.000604
3942.000000	0.000606
3941.750000	0.000622
3941.500000	0.000647

3941.250000	0.000677
3941.000000	0.000706
3940.750000	0.000730
3940.500000	0.000746
3940.250000	0.000753
3940.000000	0.000750
3939.750000	0.000739
3939.500000	0.000721
3939.250000	0.000698
3939.000000	0.000674
3938.750000	0.000651
3938.500000	0.000629
3938.250000	0.000612
3938.000000	0.000600
3937.750000	0.000593
3937.500000	0.000591
3937.250000	0.000594
3937.000000	0.000601
3936.750000	0.000610
3936.500000	0.000622
3936.250000	0.000634
3936.000000	0.000647
3935.750000	0.000658
3935.500000	0.000666
3935.250000	0.000672
3935.000000	0.000675

3934.750000	0.000678
3934.500000	0.000680
3934.250000	0.000684
3934.000000	0.000692
3933.750000	0.000701
3933.500000	0.000713
3933.250000	0.000723
3933.000000	0.000731
3932.750000	0.000733
3932.500000	0.000731
3932.250000	0.000727
3932.000000	0.000724
3931.750000	0.000726
3931.500000	0.000734
3931.250000	0.000749
3931.000000	0.000767
3930.750000	0.000786
3930.500000	0.000801
3930.250000	0.000810
3930.000000	0.000811
3929.750000	0.000802
3929.500000	0.000786
3929.250000	0.000765
3929.000000	0.000742
3928.750000	0.000719
3928.500000	0.000699

3928.250000	0.000684
3928.000000	0.000674
3927.750000	0.000668
3927.500000	0.000666
3927.250000	0.000667
3927.000000	0.000671
3926.750000	0.000676
3926.500000	0.000682
3926.250000	0.000690
3926.000000	0.000699
3925.750000	0.000710
3925.500000	0.000723
3925.250000	0.000736
3925.000000	0.000748
3924.750000	0.000759
3924.500000	0.000766
3924.250000	0.000769
3924.000000	0.000766
3923.750000	0.000759
3923.500000	0.000747
3923.250000	0.000733
3923.000000	0.000719
3922.750000	0.000706
3922.500000	0.000697
3922.250000	0.000694
3922.000000	0.000695

3921.750000	0.000702
3921.500000	0.000712
3921.250000	0.000723
3921.000000	0.000735
3920.750000	0.000742
3920.500000	0.000744
3920.250000	0.000738
3920.000000	0.000728
3919.750000	0.000719
3919.500000	0.000714
3919.250000	0.000717
3919.000000	0.000730
3918.750000	0.000750
3918.500000	0.000777
3918.250000	0.000808
3918.000000	0.000842
3917.750000	0.000871
3917.500000	0.000892
3917.250000	0.000898
3917.000000	0.000890
3916.750000	0.000870
3916.500000	0.000843
3916.250000	0.000815
3916.000000	0.000790
3915.750000	0.000769
3915.500000	0.000751

3915.250000	0.000738
3915.000000	0.000729
3914.750000	0.000726
3914.500000	0.000725
3914.250000	0.000727
3914.000000	0.000728
3913.750000	0.000726
3913.500000	0.000718
3913.250000	0.000704
3913.000000	0.000684
3912.750000	0.000659
3912.500000	0.000632
3912.250000	0.000605
3912.000000	0.000580
3911.750000	0.000558
3911.500000	0.000541
3911.250000	0.000528
3911.000000	0.000519
3910.750000	0.000514
3910.500000	0.000511
3910.250000	0.000513
3910.000000	0.000518
3909.750000	0.000527
3909.500000	0.000537
3909.250000	0.000548
3909.000000	0.000559

3908.750000	0.000567
3908.500000	0.000573
3908.250000	0.000579
3908.000000	0.000584
3907.750000	0.000589
3907.500000	0.000593
3907.250000	0.000594
3907.000000	0.000592
3906.750000	0.000587
3906.500000	0.000579
3906.250000	0.000573
3906.000000	0.000575
3905.750000	0.000587
3905.500000	0.000614
3905.250000	0.000654
3905.000000	0.000701
3904.750000	0.000750
3904.500000	0.000794
3904.250000	0.000832
3904.000000	0.000867
3903.750000	0.000903
3903.500000	0.000941
3903.250000	0.000979
3903.000000	0.001014
3902.750000	0.001040
3902.500000	0.001055

3902.250000	0.001055
3902.000000	0.001046
3901.750000	0.001035
3901.500000	0.001032
3901.250000	0.001042
3901.000000	0.001067
3900.750000	0.001098
3900.500000	0.001128
3900.250000	0.001149
3900.000000	0.001154
3899.750000	0.001139
3899.500000	0.001102
3899.250000	0.001048
3899.000000	0.000986
3898.750000	0.000927
3898.500000	0.000881
3898.250000	0.000849
3898.000000	0.000828
3897.750000	0.000814
3897.500000	0.000803
3897.250000	0.000795
3897.000000	0.000789
3896.750000	0.000786
3896.500000	0.000787
3896.250000	0.000788
3896.000000	0.000789

3895.750000	0.000789
3895.500000	0.000787
3895.250000	0.000782
3895.000000	0.000775
3894.750000	0.000764
3894.500000	0.000751
3894.250000	0.000736
3894.000000	0.000720
3893.750000	0.000705
3893.500000	0.000693
3893.250000	0.000685
3893.000000	0.000680
3892.750000	0.000678
3892.500000	0.000680
3892.250000	0.000684
3892.000000	0.000691
3891.750000	0.000697
3891.500000	0.000703
3891.250000	0.000710
3891.000000	0.000722
3890.750000	0.000743
3890.500000	0.000771
3890.250000	0.000801
3890.000000	0.000826
3889.750000	0.000841
3889.500000	0.000843

3889.250000	0.000834
3889.000000	0.000816
3888.750000	0.000791
3888.500000	0.000762
3888.250000	0.000731
3888.000000	0.000699
3887.750000	0.000668
3887.500000	0.000640
3887.250000	0.000618
3887.000000	0.000607
3886.750000	0.000609
3886.500000	0.000625
3886.250000	0.000649
3886.000000	0.000675
3885.750000	0.000698
3885.500000	0.000721
3885.250000	0.000752
3885.000000	0.000790
3884.750000	0.000832
3884.500000	0.000868
3884.250000	0.000892
3884.000000	0.000905
3883.750000	0.000907
3883.500000	0.000905
3883.250000	0.000900
3883.000000	0.000895

3882.750000	0.000891
3882.500000	0.000890
3882.250000	0.000890
3882.000000	0.000892
3881.750000	0.000896
3881.500000	0.000900
3881.250000	0.000901
3881.000000	0.000894
3880.750000	0.000872
3880.500000	0.000835
3880.250000	0.000792
3880.000000	0.000757
3879.750000	0.000742
3879.500000	0.000746
3879.250000	0.000762
3879.000000	0.000780
3878.750000	0.000794
3878.500000	0.000798
3878.250000	0.000793
3878.000000	0.000780
3877.750000	0.000760
3877.500000	0.000735
3877.250000	0.000710
3877.000000	0.000686
3876.750000	0.000669
3876.500000	0.000661

3876.250000	0.000664
3876.000000	0.000676
3875.750000	0.000696
3875.500000	0.000721
3875.250000	0.000746
3875.000000	0.000764
3874.750000	0.000768
3874.500000	0.000754
3874.250000	0.000725
3874.000000	0.000690
3873.750000	0.000665
3873.500000	0.000662
3873.250000	0.000681
3873.000000	0.000718
3872.750000	0.000765
3872.500000	0.000814
3872.250000	0.000858
3872.000000	0.000894
3871.750000	0.000918
3871.500000	0.000931
3871.250000	0.000938
3871.000000	0.000946
3870.750000	0.000963
3870.500000	0.000987
3870.250000	0.001010
3870.000000	0.001016

3869.750000	0.000999
3869.500000	0.000969
3869.250000	0.000941
3869.000000	0.000927
3868.750000	0.000928
3868.500000	0.000941
3868.250000	0.000959
3868.000000	0.000982
3867.750000	0.001008
3867.500000	0.001035
3867.250000	0.001059
3867.000000	0.001077
3866.750000	0.001086
3866.500000	0.001086
3866.250000	0.001077
3866.000000	0.001062
3865.750000	0.001041
3865.500000	0.001013
3865.250000	0.000975
3865.000000	0.000929
3864.750000	0.000884
3864.500000	0.000849
3864.250000	0.000832
3864.000000	0.000834
3863.750000	0.000846
3863.500000	0.000861

3863.250000	0.000875
3863.000000	0.000887
3862.750000	0.000896
3862.500000	0.000904
3862.250000	0.000911
3862.000000	0.000914
3861.750000	0.000914
3861.500000	0.000910
3861.250000	0.000905
3861.000000	0.000899
3860.750000	0.000888
3860.500000	0.000870
3860.250000	0.000845
3860.000000	0.000813
3859.750000	0.000780
3859.500000	0.000748
3859.250000	0.000724
3859.000000	0.000708
3858.750000	0.000702
3858.500000	0.000705
3858.250000	0.000714
3858.000000	0.000725
3857.750000	0.000733
3857.500000	0.000734
3857.250000	0.000729
3857.000000	0.000719

3856.750000	0.000707
3856.500000	0.000697
3856.250000	0.000690
3856.000000	0.000683
3855.750000	0.000674
3855.500000	0.000663
3855.250000	0.000658
3855.000000	0.000671
3854.750000	0.000710
3854.500000	0.000768
3854.250000	0.000832
3854.000000	0.000891
3853.750000	0.000946
3853.500000	0.001005
3853.250000	0.001072
3853.000000	0.001133
3852.750000	0.001172
3852.500000	0.001185
3852.250000	0.001181
3852.000000	0.001176
3851.750000	0.001177
3851.500000	0.001185
3851.250000	0.001194
3851.000000	0.001195
3850.750000	0.001180
3850.500000	0.001148

3850.250000	0.001101
3850.000000	0.001046
3849.750000	0.000989
3849.500000	0.000935
3849.250000	0.000888
3849.000000	0.000848
3848.750000	0.000814
3848.500000	0.000786
3848.250000	0.000762
3848.000000	0.000742
3847.750000	0.000724
3847.500000	0.000708
3847.250000	0.000695
3847.000000	0.000684
3846.750000	0.000676
3846.500000	0.000670
3846.250000	0.000668
3846.000000	0.000668
3845.750000	0.000671
3845.500000	0.000678
3845.250000	0.000687
3845.000000	0.000700
3844.750000	0.000714
3844.500000	0.000729
3844.250000	0.000740
3844.000000	0.000744

3843.750000	0.000742
3843.500000	0.000737
3843.250000	0.000734
3843.000000	0.000736
3842.750000	0.000742
3842.500000	0.000751
3842.250000	0.000760
3842.000000	0.000771
3841.750000	0.000785
3841.500000	0.000802
3841.250000	0.000818
3841.000000	0.000831
3840.750000	0.000837
3840.500000	0.000838
3840.250000	0.000842
3840.000000	0.000854
3839.750000	0.000875
3839.500000	0.000904
3839.250000	0.000939
3839.000000	0.000980
3838.750000	0.001024
3838.500000	0.001065
3838.250000	0.001089
3838.000000	0.001085
3837.750000	0.001056
3837.500000	0.001017

3837.250000	0.000987
3837.000000	0.000975
3836.750000	0.000976
3836.500000	0.000982
3836.250000	0.000986
3836.000000	0.000987
3835.750000	0.000982
3835.500000	0.000969
3835.250000	0.000944
3835.000000	0.000908
3834.750000	0.000867
3834.500000	0.000831
3834.250000	0.000809
3834.000000	0.000802
3833.750000	0.000807
3833.500000	0.000818
3833.250000	0.000830
3833.000000	0.000839
3832.750000	0.000845
3832.500000	0.000846
3832.250000	0.000841
3832.000000	0.000826
3831.750000	0.000803
3831.500000	0.000777
3831.250000	0.000755
3831.000000	0.000743

3830.750000	0.000741
3830.500000	0.000747
3830.250000	0.000755
3830.000000	0.000763
3829.750000	0.000769
3829.500000	0.000775
3829.250000	0.000782
3829.000000	0.000790
3828.750000	0.000800
3828.500000	0.000811
3828.250000	0.000821
3828.000000	0.000830
3827.750000	0.000835
3827.500000	0.000834
3827.250000	0.000826
3827.000000	0.000811
3826.750000	0.000791
3826.500000	0.000771
3826.250000	0.000757
3826.000000	0.000751
3825.750000	0.000755
3825.500000	0.000766
3825.250000	0.000780
3825.000000	0.000791
3824.750000	0.000797
3824.500000	0.000796

3824.250000	0.000788
3824.000000	0.000773
3823.750000	0.000756
3823.500000	0.000738
3823.250000	0.000724
3823.000000	0.000716
3822.750000	0.000719
3822.500000	0.000735
3822.250000	0.000770
3822.000000	0.000822
3821.750000	0.000884
3821.500000	0.000942
3821.250000	0.000982
3821.000000	0.001002
3820.750000	0.001010
3820.500000	0.001019
3820.250000	0.001034
3820.000000	0.001052
3819.750000	0.001065
3819.500000	0.001068
3819.250000	0.001063
3819.000000	0.001051
3818.750000	0.001035
3818.500000	0.001016
3818.250000	0.000995
3818.000000	0.000973

3817.750000	0.000950
3817.500000	0.000927
3817.250000	0.000906
3817.000000	0.000891
3816.750000	0.000878
3816.500000	0.000861
3816.250000	0.000831
3816.000000	0.000790
3815.750000	0.000749
3815.500000	0.000725
3815.250000	0.000725
3815.000000	0.000743
3814.750000	0.000767
3814.500000	0.000788
3814.250000	0.000804
3814.000000	0.000817
3813.750000	0.000828
3813.500000	0.000838
3813.250000	0.000844
3813.000000	0.000845
3812.750000	0.000839
3812.500000	0.000826
3812.250000	0.000805
3812.000000	0.000780
3811.750000	0.000754
3811.500000	0.000728

3811.250000	0.000706
3811.000000	0.000690
3810.750000	0.000680
3810.500000	0.000674
3810.250000	0.000671
3810.000000	0.000667
3809.750000	0.000662
3809.500000	0.000654
3809.250000	0.000645
3809.000000	0.000638
3808.750000	0.000636
3808.500000	0.000641
3808.250000	0.000656
3808.000000	0.000680
3807.750000	0.000708
3807.500000	0.000730
3807.250000	0.000735
3807.000000	0.000722
3806.750000	0.000700
3806.500000	0.000683
3806.250000	0.000684
3806.000000	0.000703
3805.750000	0.000735
3805.500000	0.000773
3805.250000	0.000811
3805.000000	0.000846

3804.750000	0.000875
3804.500000	0.000896
3804.250000	0.000905
3804.000000	0.000901
3803.750000	0.000884
3803.500000	0.000855
3803.250000	0.000818
3803.000000	0.000780
3802.750000	0.000748
3802.500000	0.000731
3802.250000	0.000737
3802.000000	0.000767
3801.750000	0.000809
3801.500000	0.000849
3801.250000	0.000878
3801.000000	0.000901
3800.750000	0.000923
3800.500000	0.000945
3800.250000	0.000964
3800.000000	0.000972
3799.750000	0.000968
3799.500000	0.000955
3799.250000	0.000937
3799.000000	0.000918
3798.750000	0.000899
3798.500000	0.000880

3798.250000	0.000862
3798.000000	0.000843
3797.750000	0.000825
3797.500000	0.000809
3797.250000	0.000796
3797.000000	0.000788
3796.750000	0.000785
3796.500000	0.000787
3796.250000	0.000796
3796.000000	0.000809
3795.750000	0.000824
3795.500000	0.000834
3795.250000	0.000835
3795.000000	0.000821
3794.750000	0.000793
3794.500000	0.000757
3794.250000	0.000718
3794.000000	0.000682
3793.750000	0.000655
3793.500000	0.000638
3793.250000	0.000630
3793.000000	0.000628
3792.750000	0.000631
3792.500000	0.000634
3792.250000	0.000637
3792.000000	0.000636

3791.750000	0.000633
3791.500000	0.000625
3791.250000	0.000613
3791.000000	0.000598
3790.750000	0.000581
3790.500000	0.000565
3790.250000	0.000551
3790.000000	0.000540
3789.750000	0.000534
3789.500000	0.000532
3789.250000	0.000532
3789.000000	0.000533
3788.750000	0.000533
3788.500000	0.000531
3788.250000	0.000525
3788.000000	0.000517
3787.750000	0.000509
3787.500000	0.000502
3787.250000	0.000499
3787.000000	0.000505
3786.750000	0.000521
3786.500000	0.000549
3786.250000	0.000588
3786.000000	0.000634
3785.750000	0.000682
3785.500000	0.000726

3785.250000	0.000760
3785.000000	0.000778
3784.750000	0.000782
3784.500000	0.000774
3784.250000	0.000759
3784.000000	0.000743
3783.750000	0.000728
3783.500000	0.000717
3783.250000	0.000709
3783.000000	0.000702
3782.750000	0.000695
3782.500000	0.000687
3782.250000	0.000679
3782.000000	0.000671
3781.750000	0.000664
3781.500000	0.000659
3781.250000	0.000657
3781.000000	0.000655
3780.750000	0.000655
3780.500000	0.000657
3780.250000	0.000663
3780.000000	0.000670
3779.750000	0.000680
3779.500000	0.000691
3779.250000	0.000703
3779.000000	0.000717

3778.750000	0.000733
3778.500000	0.000747
3778.250000	0.000756
3778.000000	0.000758
3777.750000	0.000754
3777.500000	0.000746
3777.250000	0.000737
3777.000000	0.000726
3776.750000	0.000713
3776.500000	0.000697
3776.250000	0.000677
3776.000000	0.000654
3775.750000	0.000629
3775.500000	0.000606
3775.250000	0.000586
3775.000000	0.000573
3774.750000	0.000566
3774.500000	0.000565
3774.250000	0.000570
3774.000000	0.000579
3773.750000	0.000589
3773.500000	0.000598
3773.250000	0.000604
3773.000000	0.000603
3772.750000	0.000598
3772.500000	0.000587

3772.250000	0.000574
3772.000000	0.000561
3771.750000	0.000553
3771.500000	0.000554
3771.250000	0.000568
3771.000000	0.000597
3770.750000	0.000642
3770.500000	0.000695
3770.250000	0.000747
3770.000000	0.000787
3769.750000	0.000809
3769.500000	0.000818
3769.250000	0.000818
3769.000000	0.000816
3768.750000	0.000815
3768.500000	0.000816
3768.250000	0.000820
3768.000000	0.000831
3767.750000	0.000850
3767.500000	0.000874
3767.250000	0.000900
3767.000000	0.000923
3766.750000	0.000937
3766.500000	0.000938
3766.250000	0.000923
3766.000000	0.000894

3765.750000	0.000854
3765.500000	0.000812
3765.250000	0.000775
3765.000000	0.000749
3764.750000	0.000735
3764.500000	0.000730
3764.250000	0.000732
3764.000000	0.000737
3763.750000	0.000743
3763.500000	0.000745
3763.250000	0.000744
3763.000000	0.000738
3762.750000	0.000728
3762.500000	0.000716
3762.250000	0.000704
3762.000000	0.000694
3761.750000	0.000688
3761.500000	0.000687
3761.250000	0.000690
3761.000000	0.000699
3760.750000	0.000711
3760.500000	0.000727
3760.250000	0.000742
3760.000000	0.000752
3759.750000	0.000753
3759.500000	0.000750

3759.250000	0.000751
3759.000000	0.000762
3758.750000	0.000786
3758.500000	0.000821
3758.250000	0.000863
3758.000000	0.000908
3757.750000	0.000952
3757.500000	0.000990
3757.250000	0.001016
3757.000000	0.001025
3756.750000	0.001015
3756.500000	0.000990
3756.250000	0.000957
3756.000000	0.000924
3755.750000	0.000898
3755.500000	0.000877
3755.250000	0.000861
3755.000000	0.000845
3754.750000	0.000827
3754.500000	0.000806
3754.250000	0.000781
3754.000000	0.000753
3753.750000	0.000726
3753.500000	0.000705
3753.250000	0.000697
3753.000000	0.000707

3752.750000	0.000730
3752.500000	0.000755
3752.250000	0.000770
3752.000000	0.000777
3751.750000	0.000789
3751.500000	0.000817
3751.250000	0.000862
3751.000000	0.000918
3750.750000	0.000980
3750.500000	0.001052
3750.250000	0.001142
3750.000000	0.001247
3749.750000	0.001347
3749.500000	0.001418
3749.250000	0.001449
3749.000000	0.001450
3748.750000	0.001438
3748.500000	0.001424
3748.250000	0.001408
3748.000000	0.001386
3747.750000	0.001357
3747.500000	0.001323
3747.250000	0.001287
3747.000000	0.001248
3746.750000	0.001207
3746.500000	0.001163

3746.250000	0.001111
3746.000000	0.001049
3745.750000	0.000988
3745.500000	0.000947
3745.250000	0.000944
3745.000000	0.000973
3744.750000	0.001002
3744.500000	0.001004
3744.250000	0.000989
3744.000000	0.000993
3743.750000	0.001043
3743.500000	0.001122
3743.250000	0.001193
3743.000000	0.001240
3742.750000	0.001269
3742.500000	0.001292
3742.250000	0.001309
3742.000000	0.001316
3741.750000	0.001305
3741.500000	0.001269
3741.250000	0.001207
3741.000000	0.001130
3740.750000	0.001052
3740.500000	0.000985
3740.250000	0.000935
3740.000000	0.000901

3739.750000	0.000881
3739.500000	0.000871
3739.250000	0.000867
3739.000000	0.000868
3738.750000	0.000873
3738.500000	0.000885
3738.250000	0.000905
3738.000000	0.000934
3737.750000	0.000973
3737.500000	0.001022
3737.250000	0.001076
3737.000000	0.001132
3736.750000	0.001183
3736.500000	0.001225
3736.250000	0.001256
3736.000000	0.001271
3735.750000	0.001267
3735.500000	0.001238
3735.250000	0.001185
3735.000000	0.001119
3734.750000	0.001056
3734.500000	0.001012
3734.250000	0.000991
3734.000000	0.000992
3733.750000	0.001006
3733.500000	0.001028

3733.250000	0.001054
3733.000000	0.001082
3732.750000	0.001104
3732.500000	0.001111
3732.250000	0.001094
3732.000000	0.001056
3731.750000	0.001006
3731.500000	0.000957
3731.250000	0.000917
3731.000000	0.000887
3730.750000	0.000862
3730.500000	0.000841
3730.250000	0.000822
3730.000000	0.000805
3729.750000	0.000789
3729.500000	0.000773
3729.250000	0.000754
3729.000000	0.000732
3728.750000	0.000708
3728.500000	0.000685
3728.250000	0.000665
3728.000000	0.000653
3727.750000	0.000651
3727.500000	0.000660
3727.250000	0.000677
3727.000000	0.000701

3726.750000	0.000727
3726.500000	0.000752
3726.250000	0.000776
3726.000000	0.000796
3725.750000	0.000811
3725.500000	0.000823
3725.250000	0.000831
3725.000000	0.000836
3724.750000	0.000842
3724.500000	0.000848
3724.250000	0.000856
3724.000000	0.000863
3723.750000	0.000871
3723.500000	0.000878
3723.250000	0.000884
3723.000000	0.000886
3722.750000	0.000882
3722.500000	0.000869
3722.250000	0.000847
3722.000000	0.000821
3721.750000	0.000799
3721.500000	0.000783
3721.250000	0.000776
3721.000000	0.000774
3720.750000	0.000777
3720.500000	0.000781

3720.250000	0.000787
3720.000000	0.000793
3719.750000	0.000797
3719.500000	0.000797
3719.250000	0.000791
3719.000000	0.000781
3718.750000	0.000769
3718.500000	0.000760
3718.250000	0.000755
3718.000000	0.000753
3717.750000	0.000753
3717.500000	0.000754
3717.250000	0.000753
3717.000000	0.000751
3716.750000	0.000748
3716.500000	0.000747
3716.250000	0.000747
3716.000000	0.000748
3715.750000	0.000750
3715.500000	0.000751
3715.250000	0.000748
3715.000000	0.000741
3714.750000	0.000730
3714.500000	0.000714
3714.250000	0.000698
3714.000000	0.000682

3713.750000	0.000670
3713.500000	0.000667
3713.250000	0.000679
3713.000000	0.000711
3712.750000	0.000758
3712.500000	0.000806
3712.250000	0.000840
3712.000000	0.000856
3711.750000	0.000863
3711.500000	0.000874
3711.250000	0.000899
3711.000000	0.000936
3710.750000	0.000984
3710.500000	0.001038
3710.250000	0.001093
3710.000000	0.001141
3709.750000	0.001167
3709.500000	0.001164
3709.250000	0.001130
3709.000000	0.001075
3708.750000	0.001012
3708.500000	0.000952
3708.250000	0.000899
3708.000000	0.000856
3707.750000	0.000824
3707.500000	0.000800

3707.250000	0.000786
3707.000000	0.000778
3706.750000	0.000774
3706.500000	0.000771
3706.250000	0.000765
3706.000000	0.000754
3705.750000	0.000736
3705.500000	0.000712
3705.250000	0.000682
3705.000000	0.000652
3704.750000	0.000624
3704.500000	0.000601
3704.250000	0.000586
3704.000000	0.000579
3703.750000	0.000578
3703.500000	0.000580
3703.250000	0.000584
3703.000000	0.000590
3702.750000	0.000598
3702.500000	0.000609
3702.250000	0.000621
3702.000000	0.000633
3701.750000	0.000645
3701.500000	0.000659
3701.250000	0.000678
3701.000000	0.000704

3700.750000	0.000733
3700.500000	0.000763
3700.250000	0.000789
3700.000000	0.000808
3699.750000	0.000819
3699.500000	0.000820
3699.250000	0.000812
3699.000000	0.000795
3698.750000	0.000773
3698.500000	0.000748
3698.250000	0.000727
3698.000000	0.000713
3697.750000	0.000707
3697.500000	0.000710
3697.250000	0.000718
3697.000000	0.000728
3696.750000	0.000737
3696.500000	0.000743
3696.250000	0.000744
3696.000000	0.000741
3695.750000	0.000737
3695.500000	0.000732
3695.250000	0.000727
3695.000000	0.000725
3694.750000	0.000724
3694.500000	0.000725

3694.250000	0.000726
3694.000000	0.000728
3693.750000	0.000730
3693.500000	0.000732
3693.250000	0.000734
3693.000000	0.000738
3692.750000	0.000745
3692.500000	0.000757
3692.250000	0.000775
3692.000000	0.000798
3691.750000	0.000819
3691.500000	0.000830
3691.250000	0.000827
3691.000000	0.000813
3690.750000	0.000797
3690.500000	0.000788
3690.250000	0.000792
3690.000000	0.000811
3689.750000	0.000847
3689.500000	0.000904
3689.250000	0.000985
3689.000000	0.001078
3688.750000	0.001161
3688.500000	0.001213
3688.250000	0.001227
3688.000000	0.001218

3687.750000	0.001205
3687.500000	0.001198
3687.250000	0.001195
3687.000000	0.001193
3686.750000	0.001190
3686.500000	0.001184
3686.250000	0.001176
3686.000000	0.001164
3685.750000	0.001148
3685.500000	0.001126
3685.250000	0.001100
3685.000000	0.001073
3684.750000	0.001047
3684.500000	0.001023
3684.250000	0.001002
3684.000000	0.000982
3683.750000	0.000962
3683.500000	0.000939
3683.250000	0.000915
3683.000000	0.000892
3682.750000	0.000872
3682.500000	0.000860
3682.250000	0.000858
3682.000000	0.000867
3681.750000	0.000887
3681.500000	0.000915

3681.250000	0.000948
3681.000000	0.000982
3680.750000	0.001012
3680.500000	0.001035
3680.250000	0.001050
3680.000000	0.001057
3679.750000	0.001058
3679.500000	0.001056
3679.250000	0.001056
3679.000000	0.001063
3678.750000	0.001076
3678.500000	0.001097
3678.250000	0.001121
3678.000000	0.001144
3677.750000	0.001162
3677.500000	0.001172
3677.250000	0.001173
3677.000000	0.001166
3676.750000	0.001156
3676.500000	0.001157
3676.250000	0.001182
3676.000000	0.001235
3675.750000	0.001302
3675.500000	0.001358
3675.250000	0.001389
3675.000000	0.001404

3674.750000	0.001420
3674.500000	0.001447
3674.250000	0.001478
3674.000000	0.001504
3673.750000	0.001522
3673.500000	0.001535
3673.250000	0.001544
3673.000000	0.001552
3672.750000	0.001557
3672.500000	0.001559
3672.250000	0.001559
3672.000000	0.001558
3671.750000	0.001559
3671.500000	0.001559
3671.250000	0.001554
3671.000000	0.001535
3670.750000	0.001501
3670.500000	0.001460
3670.250000	0.001428
3670.000000	0.001415
3669.750000	0.001428
3669.500000	0.001463
3669.250000	0.001511
3669.000000	0.001561
3668.750000	0.001605
3668.500000	0.001638

3668.250000	0.001659
3668.000000	0.001669
3667.750000	0.001667
3667.500000	0.001656
3667.250000	0.001636
3667.000000	0.001611
3666.750000	0.001582
3666.500000	0.001553
3666.250000	0.001526
3666.000000	0.001501
3665.750000	0.001478
3665.500000	0.001459
3665.250000	0.001442
3665.000000	0.001428
3664.750000	0.001417
3664.500000	0.001409
3664.250000	0.001404
3664.000000	0.001401
3663.750000	0.001400
3663.500000	0.001402
3663.250000	0.001406
3663.000000	0.001414
3662.750000	0.001425
3662.500000	0.001440
3662.250000	0.001458
3662.000000	0.001476

3661.750000	0.001493
3661.500000	0.001504
3661.250000	0.001507
3661.000000	0.001502
3660.750000	0.001489
3660.500000	0.001472
3660.250000	0.001455
3660.000000	0.001443
3659.750000	0.001440
3659.500000	0.001446
3659.250000	0.001462
3659.000000	0.001484
3658.750000	0.001510
3658.500000	0.001536
3658.250000	0.001560
3658.000000	0.001581
3657.750000	0.001599
3657.500000	0.001619
3657.250000	0.001644
3657.000000	0.001677
3656.750000	0.001713
3656.500000	0.001746
3656.250000	0.001770
3656.000000	0.001785
3655.750000	0.001798
3655.500000	0.001811

3655.250000	0.001825
3655.000000	0.001838
3654.750000	0.001847
3654.500000	0.001850
3654.250000	0.001848
3654.000000	0.001841
3653.750000	0.001829
3653.500000	0.001812
3653.250000	0.001793
3653.000000	0.001775
3652.750000	0.001761
3652.500000	0.001754
3652.250000	0.001756
3652.000000	0.001770
3651.750000	0.001797
3651.500000	0.001839
3651.250000	0.001898
3651.000000	0.001973
3650.750000	0.002064
3650.500000	0.002164
3650.250000	0.002263
3650.000000	0.002352
3649.750000	0.002424
3649.500000	0.002480
3649.250000	0.002525
3649.000000	0.002564

3648.750000	0.002604
3648.500000	0.002644
3648.250000	0.002679
3648.000000	0.002702
3647.750000	0.002705
3647.500000	0.002682
3647.250000	0.002635
3647.000000	0.002576
3646.750000	0.002517
3646.500000	0.002466
3646.250000	0.002425
3646.000000	0.002389
3645.750000	0.002355
3645.500000	0.002321
3645.250000	0.002287
3645.000000	0.002259
3644.750000	0.002241
3644.500000	0.002236
3644.250000	0.002243
3644.000000	0.002259
3643.750000	0.002279
3643.500000	0.002298
3643.250000	0.002310
3643.000000	0.002315
3642.750000	0.002310
3642.500000	0.002299

3642.250000	0.002282
3642.000000	0.002264
3641.750000	0.002248
3641.500000	0.002235
3641.250000	0.002227
3641.000000	0.002226
3640.750000	0.002229
3640.500000	0.002238
3640.250000	0.002250
3640.000000	0.002263
3639.750000	0.002275
3639.500000	0.002284
3639.250000	0.002290
3639.000000	0.002295
3638.750000	0.002300
3638.500000	0.002308
3638.250000	0.002318
3638.000000	0.002330
3637.750000	0.002341
3637.500000	0.002349
3637.250000	0.002350
3637.000000	0.002344
3636.750000	0.002333
3636.500000	0.002319
3636.250000	0.002307
3636.000000	0.002302

3635.750000	0.002306
3635.500000	0.002323
3635.250000	0.002351
3635.000000	0.002389
3634.750000	0.002432
3634.500000	0.002477
3634.250000	0.002517
3634.000000	0.002551
3633.750000	0.002577
3633.500000	0.002599
3633.250000	0.002618
3633.000000	0.002636
3632.750000	0.002653
3632.500000	0.002667
3632.250000	0.002677
3632.000000	0.002681
3631.750000	0.002676
3631.500000	0.002663
3631.250000	0.002641
3631.000000	0.002614
3630.750000	0.002587
3630.500000	0.002570
3630.250000	0.002575
3630.000000	0.002612
3629.750000	0.002685
3629.500000	0.002782

3629.250000	0.002878
3629.000000	0.002949
3628.750000	0.002989
3628.500000	0.003009
3628.250000	0.003025
3628.000000	0.003046
3627.750000	0.003072
3627.500000	0.003099
3627.250000	0.003128
3627.000000	0.003156
3626.750000	0.003179
3626.500000	0.003190
3626.250000	0.003186
3626.000000	0.003168
3625.750000	0.003140
3625.500000	0.003107
3625.250000	0.003077
3625.000000	0.003052
3624.750000	0.003033
3624.500000	0.003020
3624.250000	0.003012
3624.000000	0.003007
3623.750000	0.003004
3623.500000	0.003003
3623.250000	0.003002
3623.000000	0.003000

3622.750000	0.002996
3622.500000	0.002993
3622.250000	0.002990
3622.000000	0.002989
3621.750000	0.002993
3621.500000	0.003002
3621.250000	0.003016
3621.000000	0.003035
3620.750000	0.003059
3620.500000	0.003087
3620.250000	0.003118
3620.000000	0.003152
3619.750000	0.003188
3619.500000	0.003228
3619.250000	0.003274
3619.000000	0.003325
3618.750000	0.003379
3618.500000	0.003428
3618.250000	0.003467
3618.000000	0.003490
3617.750000	0.003496
3617.500000	0.003491
3617.250000	0.003482
3617.000000	0.003478
3616.750000	0.003487
3616.500000	0.003511

3616.250000	0.003549
3616.000000	0.003596
3615.750000	0.003643
3615.500000	0.003681
3615.250000	0.003700
3615.000000	0.003700
3614.750000	0.003684
3614.500000	0.003664
3614.250000	0.003652
3614.000000	0.003656
3613.750000	0.003681
3613.500000	0.003723
3613.250000	0.003771
3613.000000	0.003811
3612.750000	0.003829
3612.500000	0.003819
3612.250000	0.003785
3612.000000	0.003738
3611.750000	0.003686
3611.500000	0.003638
3611.250000	0.003599
3611.000000	0.003571
3610.750000	0.003557
3610.500000	0.003560
3610.250000	0.003581
3610.000000	0.003615

3609.750000	0.003655
3609.500000	0.003693
3609.250000	0.003725
3609.000000	0.003750
3608.750000	0.003774
3608.500000	0.003799
3608.250000	0.003826
3608.000000	0.003852
3607.750000	0.003875
3607.500000	0.003888
3607.250000	0.003887
3607.000000	0.003871
3606.750000	0.003841
3606.500000	0.003803
3606.250000	0.003764
3606.000000	0.003730
3605.750000	0.003704
3605.500000	0.003687
3605.250000	0.003677
3605.000000	0.003675
3604.750000	0.003679
3604.500000	0.003689
3604.250000	0.003702
3604.000000	0.003719
3603.750000	0.003738
3603.500000	0.003756

3603.250000	0.003772
3603.000000	0.003782
3602.750000	0.003788
3602.500000	0.003790
3602.250000	0.003792
3602.000000	0.003794
3601.750000	0.003800
3601.500000	0.003809
3601.250000	0.003821
3601.000000	0.003834
3600.750000	0.003847
3600.500000	0.003857
3600.250000	0.003865
3600.000000	0.003870
3599.750000	0.003871
3599.500000	0.003867
3599.250000	0.003861
3599.000000	0.003852
3598.750000	0.003846
3598.500000	0.003846
3598.250000	0.003854
3598.000000	0.003874
3597.750000	0.003904
3597.500000	0.003941
3597.250000	0.003981
3597.000000	0.004016

3596.750000	0.004041
3596.500000	0.004053
3596.250000	0.004050
3596.000000	0.004034
3595.750000	0.004006
3595.500000	0.003975
3595.250000	0.003945
3595.000000	0.003922
3594.750000	0.003910
3594.500000	0.003912
3594.250000	0.003925
3594.000000	0.003949
3593.750000	0.003978
3593.500000	0.004007
3593.250000	0.004031
3593.000000	0.004047
3592.750000	0.004054
3592.500000	0.004056
3592.250000	0.004053
3592.000000	0.004050
3591.750000	0.004047
3591.500000	0.004045
3591.250000	0.004044
3591.000000	0.004043
3590.750000	0.004043
3590.500000	0.004045

3590.250000	0.004050
3590.000000	0.004062
3589.750000	0.004086
3589.500000	0.004121
3589.250000	0.004168
3589.000000	0.004220
3588.750000	0.004271
3588.500000	0.004315
3588.250000	0.004349
3588.000000	0.004375
3587.750000	0.004393
3587.500000	0.004405
3587.250000	0.004413
3587.000000	0.004417
3586.750000	0.004418
3586.500000	0.004416
3586.250000	0.004414
3586.000000	0.004411
3585.750000	0.004409
3585.500000	0.004404
3585.250000	0.004396
3585.000000	0.004387
3584.750000	0.004377
3584.500000	0.004368
3584.250000	0.004362
3584.000000	0.004360

3583.750000	0.004360
3583.500000	0.004359
3583.250000	0.004355
3583.000000	0.004347
3582.750000	0.004334
3582.500000	0.004316
3582.250000	0.004298
3582.000000	0.004283
3581.750000	0.004275
3581.500000	0.004275
3581.250000	0.004285
3581.000000	0.004302
3580.750000	0.004323
3580.500000	0.004342
3580.250000	0.004358
3580.000000	0.004371
3579.750000	0.004380
3579.500000	0.004390
3579.250000	0.004401
3579.000000	0.004417
3578.750000	0.004434
3578.500000	0.004453
3578.250000	0.004470
3578.000000	0.004483
3577.750000	0.004492
3577.500000	0.004498

3577.250000	0.004502
3577.000000	0.004506
3576.750000	0.004512
3576.500000	0.004519
3576.250000	0.004527
3576.000000	0.004536
3575.750000	0.004543
3575.500000	0.004549
3575.250000	0.004552
3575.000000	0.004552
3574.750000	0.004550
3574.500000	0.004546
3574.250000	0.004542
3574.000000	0.004538
3573.750000	0.004537
3573.500000	0.004541
3573.250000	0.004549
3573.000000	0.004563
3572.750000	0.004580
3572.500000	0.004599
3572.250000	0.004617
3572.000000	0.004629
3571.750000	0.004632
3571.500000	0.004627
3571.250000	0.004615
3571.000000	0.004599

3570.750000	0.004586
3570.500000	0.004582
3570.250000	0.004591
3570.000000	0.004618
3569.750000	0.004662
3569.500000	0.004720
3569.250000	0.004788
3569.000000	0.004859
3568.750000	0.004925
3568.500000	0.004980
3568.250000	0.005022
3568.000000	0.005055
3567.750000	0.005084
3567.500000	0.005117
3567.250000	0.005157
3567.000000	0.005201
3566.750000	0.005244
3566.500000	0.005276
3566.250000	0.005292
3566.000000	0.005289
3565.750000	0.005271
3565.500000	0.005245
3565.250000	0.005215
3565.000000	0.005185
3564.750000	0.005159
3564.500000	0.005136

3564.250000	0.005117
3564.000000	0.005099
3563.750000	0.005080
3563.500000	0.005059
3563.250000	0.005034
3563.000000	0.005008
3562.750000	0.004981
3562.500000	0.004958
3562.250000	0.004941
3562.000000	0.004932
3561.750000	0.004931
3561.500000	0.004938
3561.250000	0.004950
3561.000000	0.004965
3560.750000	0.004977
3560.500000	0.004983
3560.250000	0.004981
3560.000000	0.004973
3559.750000	0.004960
3559.500000	0.004949
3559.250000	0.004944
3559.000000	0.004947
3558.750000	0.004959
3558.500000	0.004979
3558.250000	0.005003
3558.000000	0.005028

3557.750000	0.005051
3557.500000	0.005068
3557.250000	0.005079
3557.000000	0.005086
3556.750000	0.005091
3556.500000	0.005094
3556.250000	0.005098
3556.000000	0.005104
3555.750000	0.005111
3555.500000	0.005120
3555.250000	0.005129
3555.000000	0.005138
3554.750000	0.005149
3554.500000	0.005160
3554.250000	0.005173
3554.000000	0.005188
3553.750000	0.005205
3553.500000	0.005223
3553.250000	0.005242
3553.000000	0.005261
3552.750000	0.005277
3552.500000	0.005289
3552.250000	0.005296
3552.000000	0.005296
3551.750000	0.005292
3551.500000	0.005285

3551.250000	0.005278
3551.000000	0.005274
3550.750000	0.005276
3550.500000	0.005283
3550.250000	0.005295
3550.000000	0.005309
3549.750000	0.005324
3549.500000	0.005336
3549.250000	0.005344
3549.000000	0.005348
3548.750000	0.005351
3548.500000	0.005354
3548.250000	0.005360
3548.000000	0.005372
3547.750000	0.005389
3547.500000	0.005414
3547.250000	0.005445
3547.000000	0.005481
3546.750000	0.005521
3546.500000	0.005563
3546.250000	0.005603
3546.000000	0.005637
3545.750000	0.005660
3545.500000	0.005670
3545.250000	0.005667
3545.000000	0.005652

3544.750000	0.005631
3544.500000	0.005610
3544.250000	0.005593
3544.000000	0.005583
3543.750000	0.005579
3543.500000	0.005580
3543.250000	0.005580
3543.000000	0.005579
3542.750000	0.005575
3542.500000	0.005568
3542.250000	0.005561
3542.000000	0.005555
3541.750000	0.005553
3541.500000	0.005553
3541.250000	0.005555
3541.000000	0.005558
3540.750000	0.005558
3540.500000	0.005557
3540.250000	0.005553
3540.000000	0.005549
3539.750000	0.005547
3539.500000	0.005548
3539.250000	0.005555
3539.000000	0.005567
3538.750000	0.005585
3538.500000	0.005608

3538.250000	0.005634
3538.000000	0.005663
3537.750000	0.005691
3537.500000	0.005718
3537.250000	0.005741
3537.000000	0.005759
3536.750000	0.005770
3536.500000	0.005774
3536.250000	0.005772
3536.000000	0.005766
3535.750000	0.005761
3535.500000	0.005757
3535.250000	0.005758
3535.000000	0.005765
3534.750000	0.005776
3534.500000	0.005791
3534.250000	0.005807
3534.000000	0.005822
3533.750000	0.005834
3533.500000	0.005842
3533.250000	0.005846
3533.000000	0.005848
3532.750000	0.005848
3532.500000	0.005848
3532.250000	0.005849
3532.000000	0.005851

3531.750000	0.005855
3531.500000	0.005861
3531.250000	0.005869
3531.000000	0.005878
3530.750000	0.005889
3530.500000	0.005902
3530.250000	0.005917
3530.000000	0.005933
3529.750000	0.005949
3529.500000	0.005966
3529.250000	0.005982
3529.000000	0.005996
3528.750000	0.006009
3528.500000	0.006020
3528.250000	0.006029
3528.000000	0.006037
3527.750000	0.006042
3527.500000	0.006049
3527.250000	0.006057
3527.000000	0.006069
3526.750000	0.006087
3526.500000	0.006109
3526.250000	0.006135
3526.000000	0.006163
3525.750000	0.006189
3525.500000	0.006212

3525.250000	0.006229
3525.000000	0.006240
3524.750000	0.006247
3524.500000	0.006249
3524.250000	0.006249
3524.000000	0.006247
3523.750000	0.006244
3523.500000	0.006242
3523.250000	0.006239
3523.000000	0.006238
3522.750000	0.006237
3522.500000	0.006238
3522.250000	0.006239
3522.000000	0.006238
3521.750000	0.006236
3521.500000	0.006231
3521.250000	0.006224
3521.000000	0.006217
3520.750000	0.006214
3520.500000	0.006214
3520.250000	0.006221
3520.000000	0.006233
3519.750000	0.006249
3519.500000	0.006266
3519.250000	0.006280
3519.000000	0.006292

3518.750000	0.006299
3518.500000	0.006304
3518.250000	0.006307
3518.000000	0.006309
3517.750000	0.006312
3517.500000	0.006316
3517.250000	0.006319
3517.000000	0.006321
3516.750000	0.006320
3516.500000	0.006316
3516.250000	0.006309
3516.000000	0.006301
3515.750000	0.006294
3515.500000	0.006288
3515.250000	0.006286
3515.000000	0.006287
3514.750000	0.006291
3514.500000	0.006298
3514.250000	0.006306
3514.000000	0.006317
3513.750000	0.006330
3513.500000	0.006345
3513.250000	0.006363
3513.000000	0.006383
3512.750000	0.006405
3512.500000	0.006426

3512.250000	0.006444
3512.000000	0.006458
3511.750000	0.006468
3511.500000	0.006473
3511.250000	0.006476
3511.000000	0.006481
3510.750000	0.006491
3510.500000	0.006507
3510.250000	0.006530
3510.000000	0.006558
3509.750000	0.006588
3509.500000	0.006615
3509.250000	0.006636
3509.000000	0.006649
3508.750000	0.006654
3508.500000	0.006651
3508.250000	0.006643
3508.000000	0.006631
3507.750000	0.006618
3507.500000	0.006606
3507.250000	0.006597
3507.000000	0.006592
3506.750000	0.006590
3506.500000	0.006592
3506.250000	0.006596
3506.000000	0.006602

3505.750000	0.006608
3505.500000	0.006615
3505.250000	0.006624
3505.000000	0.006634
3504.750000	0.006647
3504.500000	0.006662
3504.250000	0.006681
3504.000000	0.006700
3503.750000	0.006719
3503.500000	0.006736
3503.250000	0.006751
3503.000000	0.006763
3502.750000	0.006775
3502.500000	0.006786
3502.250000	0.006795
3502.000000	0.006802
3501.750000	0.006805
3501.500000	0.006804
3501.250000	0.006799
3501.000000	0.006792
3500.750000	0.006785
3500.500000	0.006784
3500.250000	0.006788
3500.000000	0.006800
3499.750000	0.006818
3499.500000	0.006839

3499.250000	0.006859
3499.000000	0.006875
3498.750000	0.006883
3498.500000	0.006885
3498.250000	0.006880
3498.000000	0.006872
3497.750000	0.006865
3497.500000	0.006861
3497.250000	0.006861
3497.000000	0.006866
3496.750000	0.006874
3496.500000	0.006883
3496.250000	0.006891
3496.000000	0.006897
3495.750000	0.006900
3495.500000	0.006903
3495.250000	0.006907
3495.000000	0.006913
3494.750000	0.006924
3494.500000	0.006937
3494.250000	0.006951
3494.000000	0.006964
3493.750000	0.006975
3493.500000	0.006986
3493.250000	0.006998
3493.000000	0.007016

3492.750000	0.007041
3492.500000	0.007076
3492.250000	0.007117
3492.000000	0.007158
3491.750000	0.007194
3491.500000	0.007217
3491.250000	0.007223
3491.000000	0.007215
3490.750000	0.007194
3490.500000	0.007168
3490.250000	0.007142
3490.000000	0.007121
3489.750000	0.007106
3489.500000	0.007096
3489.250000	0.007090
3489.000000	0.007086
3488.750000	0.007084
3488.500000	0.007082
3488.250000	0.007083
3488.000000	0.007088
3487.750000	0.007095
3487.500000	0.007105
3487.250000	0.007115
3487.000000	0.007124
3486.750000	0.007131
3486.500000	0.007136

3486.250000	0.007138
3486.000000	0.007139
3485.750000	0.007140
3485.500000	0.007139
3485.250000	0.007138
3485.000000	0.007136
3484.750000	0.007135
3484.500000	0.007137
3484.250000	0.007143
3484.000000	0.007154
3483.750000	0.007172
3483.500000	0.007194
3483.250000	0.007217
3483.000000	0.007240
3482.750000	0.007258
3482.500000	0.007269
3482.250000	0.007273
3482.000000	0.007270
3481.750000	0.007261
3481.500000	0.007249
3481.250000	0.007238
3481.000000	0.007229
3480.750000	0.007227
3480.500000	0.007232
3480.250000	0.007246
3480.000000	0.007268

3479.750000	0.007295
3479.500000	0.007325
3479.250000	0.007353
3479.000000	0.007377
3478.750000	0.007395
3478.500000	0.007405
3478.250000	0.007410
3478.000000	0.007410
3477.750000	0.007408
3477.500000	0.007404
3477.250000	0.007399
3477.000000	0.007396
3476.750000	0.007395
3476.500000	0.007397
3476.250000	0.007403
3476.000000	0.007412
3475.750000	0.007425
3475.500000	0.007439
3475.250000	0.007454
3475.000000	0.007469
3474.750000	0.007485
3474.500000	0.007501
3474.250000	0.007520
3474.000000	0.007541
3473.750000	0.007563
3473.500000	0.007584

3473.250000	0.007599
3473.000000	0.007607
3472.750000	0.007605
3472.500000	0.007596
3472.250000	0.007581
3472.000000	0.007565
3471.750000	0.007554
3471.500000	0.007550
3471.250000	0.007555
3471.000000	0.007568
3470.750000	0.007587
3470.500000	0.007609
3470.250000	0.007630
3470.000000	0.007646
3469.750000	0.007658
3469.500000	0.007664
3469.250000	0.007667
3469.000000	0.007668
3468.750000	0.007670
3468.500000	0.007674
3468.250000	0.007683
3468.000000	0.007696
3467.750000	0.007715
3467.500000	0.007740
3467.250000	0.007770
3467.000000	0.007805

3466.750000	0.007843
3466.500000	0.007883
3466.250000	0.007922
3466.000000	0.007955
3465.750000	0.007980
3465.500000	0.007994
3465.250000	0.007997
3465.000000	0.007989
3464.750000	0.007975
3464.500000	0.007958
3464.250000	0.007944
3464.000000	0.007937
3463.750000	0.007938
3463.500000	0.007948
3463.250000	0.007966
3463.000000	0.007990
3462.750000	0.008019
3462.500000	0.008049
3462.250000	0.008079
3462.000000	0.008106
3461.750000	0.008129
3461.500000	0.008148
3461.250000	0.008163
3461.000000	0.008175
3460.750000	0.008186
3460.500000	0.008197

3460.250000	0.008209
3460.000000	0.008221
3459.750000	0.008234
3459.500000	0.008247
3459.250000	0.008258
3459.000000	0.008268
3458.750000	0.008279
3458.500000	0.008292
3458.250000	0.008309
3458.000000	0.008330
3457.750000	0.008353
3457.500000	0.008376
3457.250000	0.008396
3457.000000	0.008410
3456.750000	0.008417
3456.500000	0.008418
3456.250000	0.008416
3456.000000	0.008414
3455.750000	0.008415
3455.500000	0.008421
3455.250000	0.008433
3455.000000	0.008449
3454.750000	0.008467
3454.500000	0.008487
3454.250000	0.008505
3454.000000	0.008522

3453.750000	0.008535
3453.500000	0.008546
3453.250000	0.008553
3453.000000	0.008558
3452.750000	0.008561
3452.500000	0.008564
3452.250000	0.008569
3452.000000	0.008576
3451.750000	0.008586
3451.500000	0.008600
3451.250000	0.008615
3451.000000	0.008631
3450.750000	0.008646
3450.500000	0.008660
3450.250000	0.008670
3450.000000	0.008678
3449.750000	0.008683
3449.500000	0.008688
3449.250000	0.008693
3449.000000	0.008703
3448.750000	0.008719
3448.500000	0.008743
3448.250000	0.008776
3448.000000	0.008815
3447.750000	0.008858
3447.500000	0.008897

3447.250000	0.008929
3447.000000	0.008951
3446.750000	0.008964
3446.500000	0.008969
3446.250000	0.008972
3446.000000	0.008976
3445.750000	0.008982
3445.500000	0.008989
3445.250000	0.008997
3445.000000	0.009003
3444.750000	0.009005
3444.500000	0.009005
3444.250000	0.009004
3444.000000	0.009007
3443.750000	0.009017
3443.500000	0.009034
3443.250000	0.009059
3443.000000	0.009087
3442.750000	0.009113
3442.500000	0.009135
3442.250000	0.009150
3442.000000	0.009156
3441.750000	0.009157
3441.500000	0.009152
3441.250000	0.009147
3441.000000	0.009141

3440.750000	0.009138
3440.500000	0.009138
3440.250000	0.009143
3440.000000	0.009155
3439.750000	0.009172
3439.500000	0.009196
3439.250000	0.009226
3439.000000	0.009260
3438.750000	0.009296
3438.500000	0.009330
3438.250000	0.009359
3438.000000	0.009382
3437.750000	0.009398
3437.500000	0.009406
3437.250000	0.009407
3437.000000	0.009404
3436.750000	0.009399
3436.500000	0.009392
3436.250000	0.009386
3436.000000	0.009382
3435.750000	0.009379
3435.500000	0.009379
3435.250000	0.009383
3435.000000	0.009392
3434.750000	0.009408
3434.500000	0.009432

3434.250000	0.009465
3434.000000	0.009504
3433.750000	0.009547
3433.500000	0.009590
3433.250000	0.009630
3433.000000	0.009662
3432.750000	0.009687
3432.500000	0.009704
3432.250000	0.009715
3432.000000	0.009724
3431.750000	0.009734
3431.500000	0.009745
3431.250000	0.009761
3431.000000	0.009780
3430.750000	0.009803
3430.500000	0.009831
3430.250000	0.009862
3430.000000	0.009897
3429.750000	0.009934
3429.500000	0.009970
3429.250000	0.010001
3429.000000	0.010026
3428.750000	0.010043
3428.500000	0.010052
3428.250000	0.010058
3428.000000	0.010062

3427.750000	0.010068
3427.500000	0.010079
3427.250000	0.010094
3427.000000	0.010111
3426.750000	0.010127
3426.500000	0.010143
3426.250000	0.010157
3426.000000	0.010171
3425.750000	0.010188
3425.500000	0.010209
3425.250000	0.010237
3425.000000	0.010269
3424.750000	0.010304
3424.500000	0.010338
3424.250000	0.010367
3424.000000	0.010389
3423.750000	0.010405
3423.500000	0.010416
3423.250000	0.010424
3423.000000	0.010433
3422.750000	0.010443
3422.500000	0.010458
3422.250000	0.010476
3422.000000	0.010497
3421.750000	0.010520
3421.500000	0.010545

3421.250000	0.010572
3421.000000	0.010600
3420.750000	0.010629
3420.500000	0.010662
3420.250000	0.010696
3420.000000	0.010732
3419.750000	0.010766
3419.500000	0.010798
3419.250000	0.010824
3419.000000	0.010843
3418.750000	0.010856
3418.500000	0.010864
3418.250000	0.010869
3418.000000	0.010874
3417.750000	0.010883
3417.500000	0.010895
3417.250000	0.010909
3417.000000	0.010926
3416.750000	0.010941
3416.500000	0.010957
3416.250000	0.010973
3416.000000	0.010991
3415.750000	0.011014
3415.500000	0.011043
3415.250000	0.011078
3415.000000	0.011116

3414.750000	0.011154
3414.500000	0.011189
3414.250000	0.011219
3414.000000	0.011241
3413.750000	0.011258
3413.500000	0.011272
3413.250000	0.011287
3413.000000	0.011304
3412.750000	0.011326
3412.500000	0.011353
3412.250000	0.011385
3412.000000	0.011421
3411.750000	0.011458
3411.500000	0.011495
3411.250000	0.011530
3411.000000	0.011560
3410.750000	0.011587
3410.500000	0.011609
3410.250000	0.011628
3410.000000	0.011646
3409.750000	0.011664
3409.500000	0.011683
3409.250000	0.011706
3409.000000	0.011732
3408.750000	0.011763
3408.500000	0.011798

3408.250000	0.011839
3408.000000	0.011885
3407.750000	0.011935
3407.500000	0.011988
3407.250000	0.012041
3407.000000	0.012089
3406.750000	0.012130
3406.500000	0.012161
3406.250000	0.012180
3406.000000	0.012190
3405.750000	0.012195
3405.500000	0.012197
3405.250000	0.012202
3405.000000	0.012211
3404.750000	0.012226
3404.500000	0.012246
3404.250000	0.012269
3404.000000	0.012295
3403.750000	0.012323
3403.500000	0.012353
3403.250000	0.012386
3403.000000	0.012422
3402.750000	0.012463
3402.500000	0.012506
3402.250000	0.012551
3402.000000	0.012598

3401.750000	0.012644
3401.500000	0.012690
3401.250000	0.012735
3401.000000	0.012778
3400.750000	0.012819
3400.500000	0.012858
3400.250000	0.012894
3400.000000	0.012929
3399.750000	0.012965
3399.500000	0.013003
3399.250000	0.013045
3399.000000	0.013092
3398.750000	0.013142
3398.500000	0.013194
3398.250000	0.013246
3398.000000	0.013292
3397.750000	0.013331
3397.500000	0.013361
3397.250000	0.013381
3397.000000	0.013394
3396.750000	0.013401
3396.500000	0.013406
3396.250000	0.013411
3396.000000	0.013419
3395.750000	0.013432
3395.500000	0.013449

3395.250000	0.013471
3395.000000	0.013498
3394.750000	0.013527
3394.500000	0.013559
3394.250000	0.013592
3394.000000	0.013623
3393.750000	0.013653
3393.500000	0.013678
3393.250000	0.013699
3393.000000	0.013714
3392.750000	0.013723
3392.500000	0.013727
3392.250000	0.013730
3392.000000	0.013733
3391.750000	0.013741
3391.500000	0.013755
3391.250000	0.013776
3391.000000	0.013804
3390.750000	0.013834
3390.500000	0.013863
3390.250000	0.013888
3390.000000	0.013908
3389.750000	0.013921
3389.500000	0.013928
3389.250000	0.013933
3389.000000	0.013936

3388.750000	0.013940
3388.500000	0.013944
3388.250000	0.013950
3388.000000	0.013956
3387.750000	0.013962
3387.500000	0.013969
3387.250000	0.013977
3387.000000	0.013986
3386.750000	0.013995
3386.500000	0.014005
3386.250000	0.014013
3386.000000	0.014018
3385.750000	0.014021
3385.500000	0.014023
3385.250000	0.014024
3385.000000	0.014028
3384.750000	0.014036
3384.500000	0.014048
3384.250000	0.014063
3384.000000	0.014078
3383.750000	0.014091
3383.500000	0.014100
3383.250000	0.014103
3383.000000	0.014100
3382.750000	0.014092
3382.500000	0.014081

3382.250000	0.014069
3382.000000	0.014057
3381.750000	0.014047
3381.500000	0.014036
3381.250000	0.014026
3381.000000	0.014015
3380.750000	0.014002
3380.500000	0.013989
3380.250000	0.013975
3380.000000	0.013963
3379.750000	0.013953
3379.500000	0.013949
3379.250000	0.013949
3379.000000	0.013955
3378.750000	0.013964
3378.500000	0.013974
3378.250000	0.013983
3378.000000	0.013990
3377.750000	0.013993
3377.500000	0.013994
3377.250000	0.013993
3377.000000	0.013992
3376.750000	0.013993
3376.500000	0.013994
3376.250000	0.013997
3376.000000	0.014001

3375.750000	0.014006
3375.500000	0.014013
3375.250000	0.014021
3375.000000	0.014031
3374.750000	0.014043
3374.500000	0.014053
3374.250000	0.014059
3374.000000	0.014057
3373.750000	0.014044
3373.500000	0.014021
3373.250000	0.013989
3373.000000	0.013953
3372.750000	0.013919
3372.500000	0.013889
3372.250000	0.013868
3372.000000	0.013854
3371.750000	0.013845
3371.500000	0.013838
3371.250000	0.013829
3371.000000	0.013818
3370.750000	0.013806
3370.500000	0.013792
3370.250000	0.013780
3370.000000	0.013772
3369.750000	0.013767
3369.500000	0.013765

3369.250000	0.013766
3369.000000	0.013768
3368.750000	0.013770
3368.500000	0.013770
3368.250000	0.013767
3368.000000	0.013759
3367.750000	0.013744
3367.500000	0.013719
3367.250000	0.013685
3367.000000	0.013646
3366.750000	0.013606
3366.500000	0.013571
3366.250000	0.013544
3366.000000	0.013530
3365.750000	0.013527
3365.500000	0.013531
3365.250000	0.013538
3365.000000	0.013543
3364.750000	0.013542
3364.500000	0.013537
3364.250000	0.013531
3364.000000	0.013528
3363.750000	0.013533
3363.500000	0.013547
3363.250000	0.013569
3363.000000	0.013595

3362.750000	0.013618
3362.500000	0.013635
3362.250000	0.013641
3362.000000	0.013637
3361.750000	0.013623
3361.500000	0.013602
3361.250000	0.013578
3361.000000	0.013551
3360.750000	0.013525
3360.500000	0.013499
3360.250000	0.013475
3360.000000	0.013454
3359.750000	0.013439
3359.500000	0.013433
3359.250000	0.013436
3359.000000	0.013448
3358.750000	0.013468
3358.500000	0.013492
3358.250000	0.013516
3358.000000	0.013534
3357.750000	0.013543
3357.500000	0.013541
3357.250000	0.013529
3357.000000	0.013507
3356.750000	0.013479
3356.500000	0.013446

3356.250000	0.013413
3356.000000	0.013380
3355.750000	0.013350
3355.500000	0.013323
3355.250000	0.013302
3355.000000	0.013286
3354.750000	0.013276
3354.500000	0.013271
3354.250000	0.013269
3354.000000	0.013268
3353.750000	0.013265
3353.500000	0.013258
3353.250000	0.013244
3353.000000	0.013225
3352.750000	0.013200
3352.500000	0.013172
3352.250000	0.013144
3352.000000	0.013119
3351.750000	0.013099
3351.500000	0.013085
3351.250000	0.013078
3351.000000	0.013076
3350.750000	0.013077
3350.500000	0.013081
3350.250000	0.013085
3350.000000	0.013089

3349.750000	0.013092
3349.500000	0.013096
3349.250000	0.013099
3349.000000	0.013101
3348.750000	0.013102
3348.500000	0.013101
3348.250000	0.013098
3348.000000	0.013092
3347.750000	0.013085
3347.500000	0.013076
3347.250000	0.013067
3347.000000	0.013059
3346.750000	0.013053
3346.500000	0.013047
3346.250000	0.013040
3346.000000	0.013030
3345.750000	0.013017
3345.500000	0.012999
3345.250000	0.012977
3345.000000	0.012953
3344.750000	0.012928
3344.500000	0.012903
3344.250000	0.012880
3344.000000	0.012859
3343.750000	0.012841
3343.500000	0.012827

3343.250000	0.012816
3343.000000	0.012810
3342.750000	0.012809
3342.500000	0.012812
3342.250000	0.012819
3342.000000	0.012826
3341.750000	0.012830
3341.500000	0.012830
3341.250000	0.012823
3341.000000	0.012809
3340.750000	0.012791
3340.500000	0.012770
3340.250000	0.012750
3340.000000	0.012733
3339.750000	0.012718
3339.500000	0.012706
3339.250000	0.012696
3339.000000	0.012686
3338.750000	0.012678
3338.500000	0.012671
3338.250000	0.012665
3338.000000	0.012663
3337.750000	0.012663
3337.500000	0.012665
3337.250000	0.012667
3337.000000	0.012665

3336.750000	0.012660
3336.500000	0.012649
3336.250000	0.012633
3336.000000	0.012615
3335.750000	0.012595
3335.500000	0.012576
3335.250000	0.012559
3335.000000	0.012543
3334.750000	0.012527
3334.500000	0.012511
3334.250000	0.012494
3334.000000	0.012474
3333.750000	0.012451
3333.500000	0.012427
3333.250000	0.012400
3333.000000	0.012373
3332.750000	0.012345
3332.500000	0.012317
3332.250000	0.012289
3332.000000	0.012263
3331.750000	0.012239
3331.500000	0.012219
3331.250000	0.012204
3331.000000	0.012195
3330.750000	0.012193
3330.500000	0.012195

3330.250000	0.012199
3330.000000	0.012203
3329.750000	0.012202
3329.500000	0.012195
3329.250000	0.012181
3329.000000	0.012159
3328.750000	0.012132
3328.500000	0.012102
3328.250000	0.012072
3328.000000	0.012044
3327.750000	0.012018
3327.500000	0.011995
3327.250000	0.011973
3327.000000	0.011953
3326.750000	0.011935
3326.500000	0.011921
3326.250000	0.011912
3326.000000	0.011909
3325.750000	0.011914
3325.500000	0.011924
3325.250000	0.011935
3325.000000	0.011945
3324.750000	0.011949
3324.500000	0.011943
3324.250000	0.011927
3324.000000	0.011902

3323.750000	0.011870
3323.500000	0.011837
3323.250000	0.011805
3323.000000	0.011777
3322.750000	0.011754
3322.500000	0.011736
3322.250000	0.011722
3322.000000	0.011708
3321.750000	0.011694
3321.500000	0.011678
3321.250000	0.011660
3321.000000	0.011641
3320.750000	0.011622
3320.500000	0.011604
3320.250000	0.011587
3320.000000	0.011570
3319.750000	0.011554
3319.500000	0.011537
3319.250000	0.011517
3319.000000	0.011494
3318.750000	0.011468
3318.500000	0.011439
3318.250000	0.011411
3318.000000	0.011383
3317.750000	0.011358
3317.500000	0.011337

3317.250000	0.011321
3317.000000	0.011309
3316.750000	0.011301
3316.500000	0.011296
3316.250000	0.011290
3316.000000	0.011283
3315.750000	0.011272
3315.500000	0.011255
3315.250000	0.011230
3315.000000	0.011199
3314.750000	0.011162
3314.500000	0.011124
3314.250000	0.011087
3314.000000	0.011055
3313.750000	0.011032
3313.500000	0.011019
3313.250000	0.011013
3313.000000	0.011012
3312.750000	0.011013
3312.500000	0.011009
3312.250000	0.010997
3312.000000	0.010976
3311.750000	0.010945
3311.500000	0.010908
3311.250000	0.010867
3311.000000	0.010829

3310.750000	0.010797
3310.500000	0.010772
3310.250000	0.010757
3310.000000	0.010750
3309.750000	0.010747
3309.500000	0.010746
3309.250000	0.010742
3309.000000	0.010735
3308.750000	0.010722
3308.500000	0.010704
3308.250000	0.010681
3308.000000	0.010656
3307.750000	0.010628
3307.500000	0.010599
3307.250000	0.010569
3307.000000	0.010538
3306.750000	0.010508
3306.500000	0.010480
3306.250000	0.010456
3306.000000	0.010436
3305.750000	0.010420
3305.500000	0.010409
3305.250000	0.010402
3305.000000	0.010394
3304.750000	0.010385
3304.500000	0.010372

3304.250000	0.010353
3304.000000	0.010328
3303.750000	0.010299
3303.500000	0.010267
3303.250000	0.010234
3303.000000	0.010203
3302.750000	0.010176
3302.500000	0.010154
3302.250000	0.010138
3302.000000	0.010127
3301.750000	0.010121
3301.500000	0.010118
3301.250000	0.010117
3301.000000	0.010115
3300.750000	0.010109
3300.500000	0.010098
3300.250000	0.010081
3300.000000	0.010060
3299.750000	0.010035
3299.500000	0.010009
3299.250000	0.009985
3299.000000	0.009963
3298.750000	0.009944
3298.500000	0.009926
3298.250000	0.009906
3298.000000	0.009882

3297.750000	0.009852
3297.500000	0.009817
3297.250000	0.009779
3297.000000	0.009740
3296.750000	0.009703
3296.500000	0.009671
3296.250000	0.009645
3296.000000	0.009625
3295.750000	0.009609
3295.500000	0.009596
3295.250000	0.009584
3295.000000	0.009573
3294.750000	0.009559
3294.500000	0.009544
3294.250000	0.009525
3294.000000	0.009504
3293.750000	0.009478
3293.500000	0.009450
3293.250000	0.009420
3293.000000	0.009389
3292.750000	0.009360
3292.500000	0.009335
3292.250000	0.009314
3292.000000	0.009298
3291.750000	0.009286
3291.500000	0.009277

3291.250000	0.009270
3291.000000	0.009263
3290.750000	0.009254
3290.500000	0.009242
3290.250000	0.009227
3290.000000	0.009209
3289.750000	0.009189
3289.500000	0.009168
3289.250000	0.009146
3289.000000	0.009125
3288.750000	0.009105
3288.500000	0.009088
3288.250000	0.009073
3288.000000	0.009059
3287.750000	0.009046
3287.500000	0.009033
3287.250000	0.009019
3287.000000	0.009005
3286.750000	0.008989
3286.500000	0.008974
3286.250000	0.008960
3286.000000	0.008947
3285.750000	0.008936
3285.500000	0.008926
3285.250000	0.008916
3285.000000	0.008907

3284.750000	0.008898
3284.500000	0.008887
3284.250000	0.008875
3284.000000	0.008860
3283.750000	0.008842
3283.500000	0.008820
3283.250000	0.008795
3283.000000	0.008766
3282.750000	0.008735
3282.500000	0.008705
3282.250000	0.008676
3282.000000	0.008650
3281.750000	0.008629
3281.500000	0.008611
3281.250000	0.008596
3281.000000	0.008584
3280.750000	0.008574
3280.500000	0.008565
3280.250000	0.008557
3280.000000	0.008551
3279.750000	0.008546
3279.500000	0.008543
3279.250000	0.008541
3279.000000	0.008539
3278.750000	0.008536
3278.500000	0.008529

3278.250000	0.008515
3278.000000	0.008495
3277.750000	0.008466
3277.500000	0.008430
3277.250000	0.008388
3277.000000	0.008345
3276.750000	0.008303
3276.500000	0.008266
3276.250000	0.008235
3276.000000	0.008211
3275.750000	0.008194
3275.500000	0.008183
3275.250000	0.008175
3275.000000	0.008169
3274.750000	0.008162
3274.500000	0.008154
3274.250000	0.008144
3274.000000	0.008130
3273.750000	0.008113
3273.500000	0.008092
3273.250000	0.008068
3273.000000	0.008042
3272.750000	0.008017
3272.500000	0.007992
3272.250000	0.007970
3272.000000	0.007951

3271.750000	0.007935
3271.500000	0.007924
3271.250000	0.007917
3271.000000	0.007914
3270.750000	0.007916
3270.500000	0.007922
3270.250000	0.007930
3270.000000	0.007940
3269.750000	0.007946
3269.500000	0.007948
3269.250000	0.007942
3269.000000	0.007928
3268.750000	0.007904
3268.500000	0.007872
3268.250000	0.007835
3268.000000	0.007797
3267.750000	0.007761
3267.500000	0.007729
3267.250000	0.007705
3267.000000	0.007690
3266.750000	0.007683
3266.500000	0.007684
3266.250000	0.007691
3266.000000	0.007700
3265.750000	0.007708
3265.500000	0.007713

3265.250000	0.007711
3265.000000	0.007703
3264.750000	0.007686
3264.500000	0.007664
3264.250000	0.007637
3264.000000	0.007607
3263.750000	0.007575
3263.500000	0.007544
3263.250000	0.007515
3263.000000	0.007488
3262.750000	0.007465
3262.500000	0.007446
3262.250000	0.007431
3262.000000	0.007421
3261.750000	0.007416
3261.500000	0.007415
3261.250000	0.007418
3261.000000	0.007424
3260.750000	0.007431
3260.500000	0.007437
3260.250000	0.007442
3260.000000	0.007441
3259.750000	0.007434
3259.500000	0.007418
3259.250000	0.007394
3259.000000	0.007364

3258.750000	0.007332
3258.500000	0.007301
3258.250000	0.007276
3258.000000	0.007259
3257.750000	0.007249
3257.500000	0.007243
3257.250000	0.007239
3257.000000	0.007232
3256.750000	0.007221
3256.500000	0.007206
3256.250000	0.007191
3256.000000	0.007177
3255.750000	0.007171
3255.500000	0.007174
3255.250000	0.007186
3255.000000	0.007205
3254.750000	0.007227
3254.500000	0.007247
3254.250000	0.007258
3254.000000	0.007259
3253.750000	0.007248
3253.500000	0.007225
3253.250000	0.007192
3253.000000	0.007153
3252.750000	0.007111
3252.500000	0.007069

3252.250000	0.007031
3252.000000	0.006997
3251.750000	0.006971
3251.500000	0.006951
3251.250000	0.006939
3251.000000	0.006934
3250.750000	0.006934
3250.500000	0.006937
3250.250000	0.006943
3250.000000	0.006948
3249.750000	0.006951
3249.500000	0.006951
3249.250000	0.006946
3249.000000	0.006936
3248.750000	0.006924
3248.500000	0.006909
3248.250000	0.006895
3248.000000	0.006881
3247.750000	0.006867
3247.500000	0.006853
3247.250000	0.006838
3247.000000	0.006823
3246.750000	0.006807
3246.500000	0.006792
3246.250000	0.006782
3246.000000	0.006779

3245.750000	0.006784
3245.500000	0.006797
3245.250000	0.006815
3245.000000	0.006835
3244.750000	0.006852
3244.500000	0.006863
3244.250000	0.006865
3244.000000	0.006857
3243.750000	0.006840
3243.500000	0.006815
3243.250000	0.006787
3243.000000	0.006757
3242.750000	0.006728
3242.500000	0.006702
3242.250000	0.006682
3242.000000	0.006668
3241.750000	0.006659
3241.500000	0.006655
3241.250000	0.006654
3241.000000	0.006653
3240.750000	0.006649
3240.500000	0.006643
3240.250000	0.006634
3240.000000	0.006623
3239.750000	0.006613
3239.500000	0.006604

3239.250000	0.006598
3239.000000	0.006593
3238.750000	0.006589
3238.500000	0.006583
3238.250000	0.006571
3238.000000	0.006554
3237.750000	0.006532
3237.500000	0.006506
3237.250000	0.006479
3237.000000	0.006455
3236.750000	0.006436
3236.500000	0.006423
3236.250000	0.006416
3236.000000	0.006413
3235.750000	0.006412
3235.500000	0.006413
3235.250000	0.006413
3235.000000	0.006413
3234.750000	0.006414
3234.500000	0.006415
3234.250000	0.006417
3234.000000	0.006419
3233.750000	0.006422
3233.500000	0.006423
3233.250000	0.006422
3233.000000	0.006419

3232.750000	0.006414
3232.500000	0.006408
3232.250000	0.006401
3232.000000	0.006397
3231.750000	0.006394
3231.500000	0.006392
3231.250000	0.006392
3231.000000	0.006392
3230.750000	0.006390
3230.500000	0.006388
3230.250000	0.006384
3230.000000	0.006380
3229.750000	0.006374
3229.500000	0.006368
3229.250000	0.006359
3229.000000	0.006347
3228.750000	0.006330
3228.500000	0.006309
3228.250000	0.006286
3228.000000	0.006261
3227.750000	0.006239
3227.500000	0.006221
3227.250000	0.006211
3227.000000	0.006208
3226.750000	0.006211
3226.500000	0.006217

3226.250000	0.006222
3226.000000	0.006226
3225.750000	0.006226
3225.500000	0.006224
3225.250000	0.006220
3225.000000	0.006217
3224.750000	0.006214
3224.500000	0.006211
3224.250000	0.006207
3224.000000	0.006201
3223.750000	0.006192
3223.500000	0.006180
3223.250000	0.006166
3223.000000	0.006152
3222.750000	0.006138
3222.500000	0.006125
3222.250000	0.006113
3222.000000	0.006099
3221.750000	0.006085
3221.500000	0.006069
3221.250000	0.006053
3221.000000	0.006040
3220.750000	0.006033
3220.500000	0.006032
3220.250000	0.006039
3220.000000	0.006050

3219.750000	0.006063
3219.500000	0.006074
3219.250000	0.006081
3219.000000	0.006082
3218.750000	0.006079
3218.500000	0.006073
3218.250000	0.006065
3218.000000	0.006058
3217.750000	0.006051
3217.500000	0.006043
3217.250000	0.006033
3217.000000	0.006018
3216.750000	0.005999
3216.500000	0.005976
3216.250000	0.005950
3216.000000	0.005926
3215.750000	0.005904
3215.500000	0.005888
3215.250000	0.005880
3215.000000	0.005880
3214.750000	0.005887
3214.500000	0.005901
3214.250000	0.005919
3214.000000	0.005939
3213.750000	0.005957
3213.500000	0.005973

3213.250000	0.005983
3213.000000	0.005988
3212.750000	0.005986
3212.500000	0.005979
3212.250000	0.005967
3212.000000	0.005951
3211.750000	0.005932
3211.500000	0.005912
3211.250000	0.005893
3211.000000	0.005876
3210.750000	0.005862
3210.500000	0.005853
3210.250000	0.005849
3210.000000	0.005851
3209.750000	0.005857
3209.500000	0.005868
3209.250000	0.005881
3209.000000	0.005894
3208.750000	0.005906
3208.500000	0.005914
3208.250000	0.005917
3208.000000	0.005914
3207.750000	0.005905
3207.500000	0.005891
3207.250000	0.005875
3207.000000	0.005859

3206.750000	0.005845
3206.500000	0.005834
3206.250000	0.005826
3206.000000	0.005821
3205.750000	0.005816
3205.500000	0.005809
3205.250000	0.005800
3205.000000	0.005791
3204.750000	0.005784
3204.500000	0.005782
3204.250000	0.005787
3204.000000	0.005798
3203.750000	0.005815
3203.500000	0.005834
3203.250000	0.005851
3203.000000	0.005862
3202.750000	0.005866
3202.500000	0.005862
3202.250000	0.005852
3202.000000	0.005839
3201.750000	0.005826
3201.500000	0.005815
3201.250000	0.005807
3201.000000	0.005802
3200.750000	0.005800
3200.500000	0.005800

3200.250000	0.005799
3200.000000	0.005797
3199.750000	0.005792
3199.500000	0.005784
3199.250000	0.005774
3199.000000	0.005762
3198.750000	0.005748
3198.500000	0.005734
3198.250000	0.005720
3198.000000	0.005709
3197.750000	0.005699
3197.500000	0.005693
3197.250000	0.005691
3197.000000	0.005694
3196.750000	0.005702
3196.500000	0.005715
3196.250000	0.005731
3196.000000	0.005746
3195.750000	0.005758
3195.500000	0.005765
3195.250000	0.005764
3195.000000	0.005755
3194.750000	0.005741
3194.500000	0.005724
3194.250000	0.005706
3194.000000	0.005688

3193.750000	0.005671
3193.500000	0.005657
3193.250000	0.005644
3193.000000	0.005634
3192.750000	0.005627
3192.500000	0.005625
3192.250000	0.005626
3192.000000	0.005631
3191.750000	0.005637
3191.500000	0.005640
3191.250000	0.005639
3191.000000	0.005633
3190.750000	0.005624
3190.500000	0.005614
3190.250000	0.005608
3190.000000	0.005608
3189.750000	0.005615
3189.500000	0.005625
3189.250000	0.005634
3189.000000	0.005637
3188.750000	0.005629
3188.500000	0.005609
3188.250000	0.005580
3188.000000	0.005545
3187.750000	0.005510
3187.500000	0.005483

3187.250000	0.005468
3187.000000	0.005465
3186.750000	0.005473
3186.500000	0.005491
3186.250000	0.005511
3186.000000	0.005531
3185.750000	0.005544
3185.500000	0.005549
3185.250000	0.005544
3185.000000	0.005530
3184.750000	0.005510
3184.500000	0.005487
3184.250000	0.005464
3184.000000	0.005445
3183.750000	0.005430
3183.500000	0.005420
3183.250000	0.005414
3183.000000	0.005410
3182.750000	0.005407
3182.500000	0.005403
3182.250000	0.005399
3182.000000	0.005395
3181.750000	0.005390
3181.500000	0.005385
3181.250000	0.005380
3181.000000	0.005376

3180.750000	0.005374
3180.500000	0.005374
3180.250000	0.005376
3180.000000	0.005380
3179.750000	0.005386
3179.500000	0.005392
3179.250000	0.005397
3179.000000	0.005399
3178.750000	0.005396
3178.500000	0.005390
3178.250000	0.005381
3178.000000	0.005371
3177.750000	0.005361
3177.500000	0.005350
3177.250000	0.005340
3177.000000	0.005330
3176.750000	0.005318
3176.500000	0.005306
3176.250000	0.005292
3176.000000	0.005280
3175.750000	0.005270
3175.500000	0.005265
3175.250000	0.005264
3175.000000	0.005268
3174.750000	0.005276
3174.500000	0.005286

3174.250000	0.005294
3174.000000	0.005297
3173.750000	0.005294
3173.500000	0.005281
3173.250000	0.005259
3173.000000	0.005229
3172.750000	0.005193
3172.500000	0.005156
3172.250000	0.005121
3172.000000	0.005093
3171.750000	0.005073
3171.500000	0.005062
3171.250000	0.005059
3171.000000	0.005062
3170.750000	0.005067
3170.500000	0.005073
3170.250000	0.005080
3170.000000	0.005085
3169.750000	0.005090
3169.500000	0.005092
3169.250000	0.005091
3169.000000	0.005085
3168.750000	0.005073
3168.500000	0.005055
3168.250000	0.005031
3168.000000	0.005006

3167.750000	0.004982
3167.500000	0.004962
3167.250000	0.004948
3167.000000	0.004942
3166.750000	0.004941
3166.500000	0.004943
3166.250000	0.004944
3166.000000	0.004943
3165.750000	0.004939
3165.500000	0.004931
3165.250000	0.004922
3165.000000	0.004914
3164.750000	0.004908
3164.500000	0.004906
3164.250000	0.004905
3164.000000	0.004905
3163.750000	0.004901
3163.500000	0.004892
3163.250000	0.004877
3163.000000	0.004857
3162.750000	0.004836
3162.500000	0.004816
3162.250000	0.004800
3162.000000	0.004791
3161.750000	0.004787
3161.500000	0.004786

3161.250000	0.004785
3161.000000	0.004780
3160.750000	0.004769
3160.500000	0.004752
3160.250000	0.004732
3160.000000	0.004711
3159.750000	0.004692
3159.500000	0.004678
3159.250000	0.004669
3159.000000	0.004666
3158.750000	0.004668
3158.500000	0.004672
3158.250000	0.004678
3158.000000	0.004685
3157.750000	0.004691
3157.500000	0.004696
3157.250000	0.004699
3157.000000	0.004697
3156.750000	0.004691
3156.500000	0.004681
3156.250000	0.004668
3156.000000	0.004656
3155.750000	0.004645
3155.500000	0.004639
3155.250000	0.004637
3155.000000	0.004641

3154.750000	0.004647
3154.500000	0.004653
3154.250000	0.004657
3154.000000	0.004657
3153.750000	0.004652
3153.500000	0.004642
3153.250000	0.004628
3153.000000	0.004612
3152.750000	0.004596
3152.500000	0.004582
3152.250000	0.004573
3152.000000	0.004568
3151.750000	0.004568
3151.500000	0.004571
3151.250000	0.004575
3151.000000	0.004579
3150.750000	0.004581
3150.500000	0.004580
3150.250000	0.004576
3150.000000	0.004570
3149.750000	0.004563
3149.500000	0.004555
3149.250000	0.004547
3149.000000	0.004538
3148.750000	0.004527
3148.500000	0.004514

3148.250000	0.004499
3148.000000	0.004483
3147.750000	0.004469
3147.500000	0.004458
3147.250000	0.004453
3147.000000	0.004452
3146.750000	0.004456
3146.500000	0.004462
3146.250000	0.004468
3146.000000	0.004470
3145.750000	0.004467
3145.500000	0.004459
3145.250000	0.004446
3145.000000	0.004430
3144.750000	0.004412
3144.500000	0.004394
3144.250000	0.004377
3144.000000	0.004361
3143.750000	0.004347
3143.500000	0.004335
3143.250000	0.004327
3143.000000	0.004322
3142.750000	0.004324
3142.500000	0.004332
3142.250000	0.004346
3142.000000	0.004365

3141.750000	0.004386
3141.500000	0.004404
3141.250000	0.004416
3141.000000	0.004418
3140.750000	0.004410
3140.500000	0.004393
3140.250000	0.004369
3140.000000	0.004344
3139.750000	0.004321
3139.500000	0.004305
3139.250000	0.004296
3139.000000	0.004294
3138.750000	0.004298
3138.500000	0.004307
3138.250000	0.004316
3138.000000	0.004326
3137.750000	0.004333
3137.500000	0.004338
3137.250000	0.004338
3137.000000	0.004334
3136.750000	0.004326
3136.500000	0.004314
3136.250000	0.004300
3136.000000	0.004284
3135.750000	0.004269
3135.500000	0.004257

3135.250000	0.004249
3135.000000	0.004244
3134.750000	0.004244
3134.500000	0.004248
3134.250000	0.004255
3134.000000	0.004264
3133.750000	0.004274
3133.500000	0.004284
3133.250000	0.004294
3133.000000	0.004301
3132.750000	0.004305
3132.500000	0.004304
3132.250000	0.004299
3132.000000	0.004289
3131.750000	0.004275
3131.500000	0.004257
3131.250000	0.004237
3131.000000	0.004216
3130.750000	0.004197
3130.500000	0.004181
3130.250000	0.004170
3130.000000	0.004166
3129.750000	0.004170
3129.500000	0.004180
3129.250000	0.004194
3129.000000	0.004209

3128.750000	0.004222
3128.500000	0.004229
3128.250000	0.004229
3128.000000	0.004220
3127.750000	0.004206
3127.500000	0.004189
3127.250000	0.004172
3127.000000	0.004159
3126.750000	0.004151
3126.500000	0.004148
3126.250000	0.004151
3126.000000	0.004156
3125.750000	0.004162
3125.500000	0.004167
3125.250000	0.004169
3125.000000	0.004169
3124.750000	0.004168
3124.500000	0.004168
3124.250000	0.004171
3124.000000	0.004177
3123.750000	0.004186
3123.500000	0.004196
3123.250000	0.004204
3123.000000	0.004208
3122.750000	0.004206
3122.500000	0.004198

3122.250000	0.004186
3122.000000	0.004172
3121.750000	0.004159
3121.500000	0.004149
3121.250000	0.004145
3121.000000	0.004145
3120.750000	0.004148
3120.500000	0.004152
3120.250000	0.004154
3120.000000	0.004153
3119.750000	0.004147
3119.500000	0.004137
3119.250000	0.004123
3119.000000	0.004107
3118.750000	0.004091
3118.500000	0.004076
3118.250000	0.004064
3118.000000	0.004058
3117.750000	0.004057
3117.500000	0.004063
3117.250000	0.004075
3117.000000	0.004092
3116.750000	0.004111
3116.500000	0.004128
3116.250000	0.004142
3116.000000	0.004152

3115.750000	0.004156
3115.500000	0.004156
3115.250000	0.004157
3115.000000	0.004160
3114.750000	0.004168
3114.500000	0.004183
3114.250000	0.004202
3114.000000	0.004223
3113.750000	0.004243
3113.500000	0.004258
3113.250000	0.004265
3113.000000	0.004263
3112.750000	0.004252
3112.500000	0.004235
3112.250000	0.004213
3112.000000	0.004190
3111.750000	0.004170
3111.500000	0.004154
3111.250000	0.004144
3111.000000	0.004142
3110.750000	0.004146
3110.500000	0.004155
3110.250000	0.004168
3110.000000	0.004182
3109.750000	0.004194
3109.500000	0.004202

3109.250000	0.004206
3109.000000	0.004205
3108.750000	0.004200
3108.500000	0.004195
3108.250000	0.004191
3108.000000	0.004190
3107.750000	0.004191
3107.500000	0.004194
3107.250000	0.004198
3107.000000	0.004201
3106.750000	0.004202
3106.500000	0.004201
3106.250000	0.004200
3106.000000	0.004200
3105.750000	0.004203
3105.500000	0.004212
3105.250000	0.004225
3105.000000	0.004243
3104.750000	0.004263
3104.500000	0.004281
3104.250000	0.004296
3104.000000	0.004306
3103.750000	0.004310
3103.500000	0.004308
3103.250000	0.004301
3103.000000	0.004291

3102.750000	0.004279
3102.500000	0.004267
3102.250000	0.004256
3102.000000	0.004246
3101.750000	0.004236
3101.500000	0.004227
3101.250000	0.004218
3101.000000	0.004211
3100.750000	0.004205
3100.500000	0.004204
3100.250000	0.004207
3100.000000	0.004214
3099.750000	0.004226
3099.500000	0.004239
3099.250000	0.004251
3099.000000	0.004259
3098.750000	0.004261
3098.500000	0.004257
3098.250000	0.004246
3098.000000	0.004231
3097.750000	0.004216
3097.500000	0.004203
3097.250000	0.004195
3097.000000	0.004194
3096.750000	0.004198
3096.500000	0.004207

3096.250000	0.004218
3096.000000	0.004228
3095.750000	0.004235
3095.500000	0.004240
3095.250000	0.004241
3095.000000	0.004242
3094.750000	0.004244
3094.500000	0.004249
3094.250000	0.004257
3094.000000	0.004268
3093.750000	0.004279
3093.500000	0.004287
3093.250000	0.004290
3093.000000	0.004285
3092.750000	0.004272
3092.500000	0.004252
3092.250000	0.004228
3092.000000	0.004206
3091.750000	0.004187
3091.500000	0.004175
3091.250000	0.004171
3091.000000	0.004173
3090.750000	0.004179
3090.500000	0.004187
3090.250000	0.004196
3090.000000	0.004203

3089.750000	0.004211
3089.500000	0.004219
3089.250000	0.004230
3089.000000	0.004243
3088.750000	0.004259
3088.500000	0.004276
3088.250000	0.004292
3088.000000	0.004304
3087.750000	0.004311
3087.500000	0.004312
3087.250000	0.004306
3087.000000	0.004296
3086.750000	0.004284
3086.500000	0.004273
3086.250000	0.004264
3086.000000	0.004261
3085.750000	0.004265
3085.500000	0.004274
3085.250000	0.004289
3085.000000	0.004306
3084.750000	0.004323
3084.500000	0.004336
3084.250000	0.004344
3084.000000	0.004346
3083.750000	0.004342
3083.500000	0.004334

3083.250000	0.004325
3083.000000	0.004318
3082.750000	0.004316
3082.500000	0.004319
3082.250000	0.004328
3082.000000	0.004342
3081.750000	0.004358
3081.500000	0.004374
3081.250000	0.004387
3081.000000	0.004395
3080.750000	0.004399
3080.500000	0.004400
3080.250000	0.004401
3080.000000	0.004404
3079.750000	0.004410
3079.500000	0.004420
3079.250000	0.004432
3079.000000	0.004444
3078.750000	0.004452
3078.500000	0.004454
3078.250000	0.004448
3078.000000	0.004436
3077.750000	0.004420
3077.500000	0.004406
3077.250000	0.004398
3077.000000	0.004398

3076.750000	0.004409
3076.500000	0.004428
3076.250000	0.004453
3076.000000	0.004478
3075.750000	0.004499
3075.500000	0.004512
3075.250000	0.004515
3075.000000	0.004509
3074.750000	0.004497
3074.500000	0.004482
3074.250000	0.004468
3074.000000	0.004458
3073.750000	0.004454
3073.500000	0.004457
3073.250000	0.004464
3073.000000	0.004477
3072.750000	0.004491
3072.500000	0.004506
3072.250000	0.004521
3072.000000	0.004535
3071.750000	0.004547
3071.500000	0.004558
3071.250000	0.004567
3071.000000	0.004574
3070.750000	0.004578
3070.500000	0.004577

3070.250000	0.004572
3070.000000	0.004562
3069.750000	0.004548
3069.500000	0.004531
3069.250000	0.004515
3069.000000	0.004502
3068.750000	0.004493
3068.500000	0.004489
3068.250000	0.004489
3068.000000	0.004492
3067.750000	0.004495
3067.500000	0.004496
3067.250000	0.004493
3067.000000	0.004487
3066.750000	0.004481
3066.500000	0.004476
3066.250000	0.004476
3066.000000	0.004483
3065.750000	0.004496
3065.500000	0.004515
3065.250000	0.004536
3065.000000	0.004556
3064.750000	0.004573
3064.500000	0.004584
3064.250000	0.004589
3064.000000	0.004588

3063.750000	0.004585
3063.500000	0.004582
3063.250000	0.004580
3063.000000	0.004582
3062.750000	0.004588
3062.500000	0.004596
3062.250000	0.004603
3062.000000	0.004608
3061.750000	0.004608
3061.500000	0.004601
3061.250000	0.004589
3061.000000	0.004573
3060.750000	0.004557
3060.500000	0.004545
3060.250000	0.004539
3060.000000	0.004541
3059.750000	0.004548
3059.500000	0.004559
3059.250000	0.004571
3059.000000	0.004580
3058.750000	0.004585
3058.500000	0.004587
3058.250000	0.004587
3058.000000	0.004587
3057.750000	0.004590
3057.500000	0.004596

3057.250000	0.004605
3057.000000	0.004614
3056.750000	0.004620
3056.500000	0.004621
3056.250000	0.004614
3056.000000	0.004601
3055.750000	0.004581
3055.500000	0.004558
3055.250000	0.004534
3055.000000	0.004512
3054.750000	0.004495
3054.500000	0.004484
3054.250000	0.004481
3054.000000	0.004485
3053.750000	0.004494
3053.500000	0.004507
3053.250000	0.004522
3053.000000	0.004536
3052.750000	0.004548
3052.500000	0.004557
3052.250000	0.004563
3052.000000	0.004565
3051.750000	0.004563
3051.500000	0.004559
3051.250000	0.004551
3051.000000	0.004540

3050.750000	0.004528
3050.500000	0.004515
3050.250000	0.004504
3050.000000	0.004496
3049.750000	0.004493
3049.500000	0.004493
3049.250000	0.004497
3049.000000	0.004502
3048.750000	0.004506
3048.500000	0.004509
3048.250000	0.004509
3048.000000	0.004506
3047.750000	0.004503
3047.500000	0.004500
3047.250000	0.004499
3047.000000	0.004501
3046.750000	0.004507
3046.500000	0.004515
3046.250000	0.004526
3046.000000	0.004537
3045.750000	0.004548
3045.500000	0.004557
3045.250000	0.004564
3045.000000	0.004567
3044.750000	0.004567
3044.500000	0.004565

3044.250000	0.004562
3044.000000	0.004560
3043.750000	0.004560
3043.500000	0.004563
3043.250000	0.004570
3043.000000	0.004580
3042.750000	0.004591
3042.500000	0.004602
3042.250000	0.004611
3042.000000	0.004614
3041.750000	0.004610
3041.500000	0.004601
3041.250000	0.004586
3041.000000	0.004570
3040.750000	0.004556
3040.500000	0.004548
3040.250000	0.004549
3040.000000	0.004561
3039.750000	0.004582
3039.500000	0.004609
3039.250000	0.004638
3039.000000	0.004663
3038.750000	0.004682
3038.500000	0.004692
3038.250000	0.004693
3038.000000	0.004688

3037.750000	0.004681
3037.500000	0.004674
3037.250000	0.004670
3037.000000	0.004668
3036.750000	0.004670
3036.500000	0.004673
3036.250000	0.004678
3036.000000	0.004683
3035.750000	0.004690
3035.500000	0.004698
3035.250000	0.004709
3035.000000	0.004723
3034.750000	0.004740
3034.500000	0.004757
3034.250000	0.004775
3034.000000	0.004792
3033.750000	0.004806
3033.500000	0.004817
3033.250000	0.004826
3033.000000	0.004833
3032.750000	0.004839
3032.500000	0.004845
3032.250000	0.004852
3032.000000	0.004861
3031.750000	0.004871
3031.500000	0.004882

3031.250000	0.004891
3031.000000	0.004899
3030.750000	0.004905
3030.500000	0.004909
3030.250000	0.004911
3030.000000	0.004914
3029.750000	0.004917
3029.500000	0.004923
3029.250000	0.004931
3029.000000	0.004941
3028.750000	0.004952
3028.500000	0.004964
3028.250000	0.004975
3028.000000	0.004985
3027.750000	0.004993
3027.500000	0.004998
3027.250000	0.005000
3027.000000	0.005000
3026.750000	0.004997
3026.500000	0.004993
3026.250000	0.004990
3026.000000	0.004989
3025.750000	0.004991
3025.500000	0.004999
3025.250000	0.005011
3025.000000	0.005025

3024.750000	0.005040
3024.500000	0.005055
3024.250000	0.005067
3024.000000	0.005077
3023.750000	0.005087
3023.500000	0.005096
3023.250000	0.005109
3023.000000	0.005127
3022.750000	0.005149
3022.500000	0.005177
3022.250000	0.005207
3022.000000	0.005238
3021.750000	0.005266
3021.500000	0.005289
3021.250000	0.005307
3021.000000	0.005318
3020.750000	0.005324
3020.500000	0.005328
3020.250000	0.005333
3020.000000	0.005341
3019.750000	0.005355
3019.500000	0.005375
3019.250000	0.005400
3019.000000	0.005430
3018.750000	0.005463
3018.500000	0.005497

3018.250000	0.005532
3018.000000	0.005566
3017.750000	0.005598
3017.500000	0.005630
3017.250000	0.005660
3017.000000	0.005687
3016.750000	0.005710
3016.500000	0.005728
3016.250000	0.005741
3016.000000	0.005748
3015.750000	0.005751
3015.500000	0.005751
3015.250000	0.005751
3015.000000	0.005755
3014.750000	0.005766
3014.500000	0.005786
3014.250000	0.005817
3014.000000	0.005856
3013.750000	0.005902
3013.500000	0.005951
3013.250000	0.005998
3013.000000	0.006041
3012.750000	0.006077
3012.500000	0.006106
3012.250000	0.006130
3012.000000	0.006152

3011.750000	0.006173
3011.500000	0.006196
3011.250000	0.006222
3011.000000	0.006250
3010.750000	0.006280
3010.500000	0.006312
3010.250000	0.006346
3010.000000	0.006380
3009.750000	0.006415
3009.500000	0.006451
3009.250000	0.006486
3009.000000	0.006518
3008.750000	0.006549
3008.500000	0.006578
3008.250000	0.006605
3008.000000	0.006633
3007.750000	0.006661
3007.500000	0.006693
3007.250000	0.006727
3007.000000	0.006765
3006.750000	0.006805
3006.500000	0.006849
3006.250000	0.006895
3006.000000	0.006944
3005.750000	0.006993
3005.500000	0.007041

3005.250000	0.007084
3005.000000	0.007119
3004.750000	0.007147
3004.500000	0.007167
3004.250000	0.007185
3004.000000	0.007204
3003.750000	0.007230
3003.500000	0.007263
3003.250000	0.007305
3003.000000	0.007350
3002.750000	0.007395
3002.500000	0.007435
3002.250000	0.007468
3002.000000	0.007496
3001.750000	0.007521
3001.500000	0.007548
3001.250000	0.007580
3001.000000	0.007618
3000.750000	0.007661
3000.500000	0.007706
3000.250000	0.007750
3000.000000	0.007794
2999.750000	0.007836
2999.500000	0.007877
2999.250000	0.007918
2999.000000	0.007960

2998.750000	0.008001
2998.500000	0.008039
2998.250000	0.008074
2998.000000	0.008104
2997.750000	0.008133
2997.500000	0.008163
2997.250000	0.008198
2997.000000	0.008242
2996.750000	0.008294
2996.500000	0.008353
2996.250000	0.008416
2996.000000	0.008478
2995.750000	0.008537
2995.500000	0.008590
2995.250000	0.008636
2995.000000	0.008678
2994.750000	0.008715
2994.500000	0.008749
2994.250000	0.008782
2994.000000	0.008815
2993.750000	0.008850
2993.500000	0.008889
2993.250000	0.008933
2993.000000	0.008983
2992.750000	0.009037
2992.500000	0.009095

2992.250000	0.009152
2992.000000	0.009206
2991.750000	0.009255
2991.500000	0.009298
2991.250000	0.009337
2991.000000	0.009373
2990.750000	0.009408
2990.500000	0.009444
2990.250000	0.009483
2990.000000	0.009525
2989.750000	0.009569
2989.500000	0.009613
2989.250000	0.009658
2989.000000	0.009699
2988.750000	0.009737
2988.500000	0.009769
2988.250000	0.009796
2988.000000	0.009818
2987.750000	0.009837
2987.500000	0.009857
2987.250000	0.009880
2987.000000	0.009909
2986.750000	0.009946
2986.500000	0.009991
2986.250000	0.010043
2986.000000	0.010099

2985.750000	0.010157
2985.500000	0.010215
2985.250000	0.010268
2985.000000	0.010315
2984.750000	0.010353
2984.500000	0.010382
2984.250000	0.010404
2984.000000	0.010421
2983.750000	0.010437
2983.500000	0.010457
2983.250000	0.010482
2983.000000	0.010515
2982.750000	0.010552
2982.500000	0.010589
2982.250000	0.010623
2982.000000	0.010649
2981.750000	0.010667
2981.500000	0.010677
2981.250000	0.010684
2981.000000	0.010691
2980.750000	0.010703
2980.500000	0.010720
2980.250000	0.010743
2980.000000	0.010771
2979.750000	0.010800
2979.500000	0.010829

2979.250000	0.010857
2979.000000	0.010884
2978.750000	0.010911
2978.500000	0.010937
2978.250000	0.010962
2978.000000	0.010985
2977.750000	0.011006
2977.500000	0.011025
2977.250000	0.011042
2977.000000	0.011057
2976.750000	0.011073
2976.500000	0.011090
2976.250000	0.011111
2976.000000	0.011137
2975.750000	0.011169
2975.500000	0.011207
2975.250000	0.011254
2975.000000	0.011308
2974.750000	0.011371
2974.500000	0.011440
2974.250000	0.011512
2974.000000	0.011586
2973.750000	0.011660
2973.500000	0.011732
2973.250000	0.011803
2973.000000	0.011874

2972.750000	0.011948
2972.500000	0.012027
2972.250000	0.012111
2972.000000	0.012199
2971.750000	0.012290
2971.500000	0.012381
2971.250000	0.012469
2971.000000	0.012551
2970.750000	0.012626
2970.500000	0.012694
2970.250000	0.012753
2970.000000	0.012805
2969.750000	0.012851
2969.500000	0.012890
2969.250000	0.012926
2969.000000	0.012956
2968.750000	0.012983
2968.500000	0.013007
2968.250000	0.013028
2968.000000	0.013046
2967.750000	0.013063
2967.500000	0.013081
2967.250000	0.013100
2967.000000	0.013123
2966.750000	0.013149
2966.500000	0.013179

2966.250000	0.013213
2966.000000	0.013250
2965.750000	0.013290
2965.500000	0.013333
2965.250000	0.013379
2965.000000	0.013430
2964.750000	0.013486
2964.500000	0.013545
2964.250000	0.013607
2964.000000	0.013671
2963.750000	0.013734
2963.500000	0.013797
2963.250000	0.013858
2963.000000	0.013919
2962.750000	0.013978
2962.500000	0.014036
2962.250000	0.014091
2962.000000	0.014142
2961.750000	0.014188
2961.500000	0.014231
2961.250000	0.014271
2961.000000	0.014312
2960.750000	0.014355
2960.500000	0.014402
2960.250000	0.014456
2960.000000	0.014514

2959.750000	0.014576
2959.500000	0.014640
2959.250000	0.014704
2959.000000	0.014767
2958.750000	0.014828
2958.500000	0.014885
2958.250000	0.014940
2958.000000	0.014989
2957.750000	0.015034
2957.500000	0.015072
2957.250000	0.015104
2957.000000	0.015133
2956.750000	0.015160
2956.500000	0.015188
2956.250000	0.015220
2956.000000	0.015258
2955.750000	0.015302
2955.500000	0.015352
2955.250000	0.015405
2955.000000	0.015458
2954.750000	0.015509
2954.500000	0.015555
2954.250000	0.015593
2954.000000	0.015624
2953.750000	0.015648
2953.500000	0.015668

2953.250000	0.015687
2953.000000	0.015709
2952.750000	0.015736
2952.500000	0.015769
2952.250000	0.015810
2952.000000	0.015855
2951.750000	0.015904
2951.500000	0.015953
2951.250000	0.016000
2951.000000	0.016045
2950.750000	0.016087
2950.500000	0.016127
2950.250000	0.016168
2950.000000	0.016211
2949.750000	0.016257
2949.500000	0.016309
2949.250000	0.016366
2949.000000	0.016427
2948.750000	0.016492
2948.500000	0.016558
2948.250000	0.016623
2948.000000	0.016687
2947.750000	0.016746
2947.500000	0.016803
2947.250000	0.016858
2947.000000	0.016912

2946.750000	0.016968
2946.500000	0.017028
2946.250000	0.017091
2946.000000	0.017160
2945.750000	0.017233
2945.500000	0.017311
2945.250000	0.017393
2945.000000	0.017480
2944.750000	0.017571
2944.500000	0.017666
2944.250000	0.017764
2944.000000	0.017864
2943.750000	0.017964
2943.500000	0.018063
2943.250000	0.018160
2943.000000	0.018255
2942.750000	0.018349
2942.500000	0.018440
2942.250000	0.018530
2942.000000	0.018619
2941.750000	0.018705
2941.500000	0.018791
2941.250000	0.018875
2941.000000	0.018961
2940.750000	0.019051
2940.500000	0.019145

2940.250000	0.019244
2940.000000	0.019348
2939.750000	0.019454
2939.500000	0.019559
2939.250000	0.019661
2939.000000	0.019758
2938.750000	0.019849
2938.500000	0.019935
2938.250000	0.020017
2938.000000	0.020097
2937.750000	0.020177
2937.500000	0.020258
2937.250000	0.020342
2937.000000	0.020428
2936.750000	0.020517
2936.500000	0.020609
2936.250000	0.020703
2936.000000	0.020799
2935.750000	0.020895
2935.500000	0.020989
2935.250000	0.021079
2935.000000	0.021166
2934.750000	0.021249
2934.500000	0.021329
2934.250000	0.021408
2934.000000	0.021487

2933.750000	0.021568
2933.500000	0.021650
2933.250000	0.021733
2933.000000	0.021816
2932.750000	0.021897
2932.500000	0.021976
2932.250000	0.022053
2932.000000	0.022127
2931.750000	0.022200
2931.500000	0.022271
2931.250000	0.022341
2931.000000	0.022409
2930.750000	0.022476
2930.500000	0.022541
2930.250000	0.022604
2930.000000	0.022665
2929.750000	0.022724
2929.500000	0.022784
2929.250000	0.022843
2929.000000	0.022903
2928.750000	0.022963
2928.500000	0.023020
2928.250000	0.023075
2928.000000	0.023124
2927.750000	0.023167
2927.500000	0.023203

2927.250000	0.023233
2927.000000	0.023258
2926.750000	0.023278
2926.500000	0.023295
2926.250000	0.023310
2926.000000	0.023323
2925.750000	0.023336
2925.500000	0.023348
2925.250000	0.023360
2925.000000	0.023370
2924.750000	0.023377
2924.500000	0.023380
2924.250000	0.023375
2924.000000	0.023363
2923.750000	0.023343
2923.500000	0.023316
2923.250000	0.023282
2923.000000	0.023244
2922.750000	0.023201
2922.500000	0.023154
2922.250000	0.023100
2922.000000	0.023038
2921.750000	0.022967
2921.500000	0.022888
2921.250000	0.022800
2921.000000	0.022707

2920.750000	0.022610
2920.500000	0.022510
2920.250000	0.022408
2920.000000	0.022301
2919.750000	0.022187
2919.500000	0.022063
2919.250000	0.021929
2919.000000	0.021785
2918.750000	0.021631
2918.500000	0.021470
2918.250000	0.021304
2918.000000	0.021135
2917.750000	0.020962
2917.500000	0.020786
2917.250000	0.020605
2917.000000	0.020419
2916.750000	0.020227
2916.500000	0.020030
2916.250000	0.019831
2916.000000	0.019632
2915.750000	0.019433
2915.500000	0.019236
2915.250000	0.019042
2915.000000	0.018850
2914.750000	0.018660
2914.500000	0.018472

2914.250000	0.018285
2914.000000	0.018099
2913.750000	0.017913
2913.500000	0.017725
2913.250000	0.017537
2913.000000	0.017347
2912.750000	0.017158
2912.500000	0.016970
2912.250000	0.016788
2912.000000	0.016612
2911.750000	0.016445
2911.500000	0.016287
2911.250000	0.016138
2911.000000	0.015997
2910.750000	0.015860
2910.500000	0.015726
2910.250000	0.015593
2910.000000	0.015460
2909.750000	0.015328
2909.500000	0.015200
2909.250000	0.015078
2909.000000	0.014963
2908.750000	0.014857
2908.500000	0.014758
2908.250000	0.014666
2908.000000	0.014576

2907.750000	0.014485
2907.500000	0.014392
2907.250000	0.014294
2907.000000	0.014193
2906.750000	0.014090
2906.500000	0.013988
2906.250000	0.013889
2906.000000	0.013796
2905.750000	0.013708
2905.500000	0.013627
2905.250000	0.013549
2905.000000	0.013474
2904.750000	0.013398
2904.500000	0.013322
2904.250000	0.013244
2904.000000	0.013167
2903.750000	0.013092
2903.500000	0.013020
2903.250000	0.012952
2903.000000	0.012890
2902.750000	0.012832
2902.500000	0.012779
2902.250000	0.012729
2902.000000	0.012680
2901.750000	0.012634
2901.500000	0.012588

2901.250000	0.012544
2901.000000	0.012500
2900.750000	0.012455
2900.500000	0.012410
2900.250000	0.012362
2900.000000	0.012312
2899.750000	0.012257
2899.500000	0.012201
2899.250000	0.012143
2899.000000	0.012087
2898.750000	0.012034
2898.500000	0.011986
2898.250000	0.011944
2898.000000	0.011905
2897.750000	0.011867
2897.500000	0.011826
2897.250000	0.011782
2897.000000	0.011732
2896.750000	0.011677
2896.500000	0.011622
2896.250000	0.011567
2896.000000	0.011518
2895.750000	0.011477
2895.500000	0.011442
2895.250000	0.011414
2895.000000	0.011389

2894.750000	0.011365
2894.500000	0.011338
2894.250000	0.011307
2894.000000	0.011272
2893.750000	0.011234
2893.500000	0.011194
2893.250000	0.011154
2893.000000	0.011116
2892.750000	0.011082
2892.500000	0.011051
2892.250000	0.011024
2892.000000	0.011001
2891.750000	0.010979
2891.500000	0.010957
2891.250000	0.010934
2891.000000	0.010908
2890.750000	0.010879
2890.500000	0.010847
2890.250000	0.010813
2890.000000	0.010777
2889.750000	0.010743
2889.500000	0.010709
2889.250000	0.010677
2889.000000	0.010645
2888.750000	0.010614
2888.500000	0.010583

2888.250000	0.010551
2888.000000	0.010519
2887.750000	0.010487
2887.500000	0.010458
2887.250000	0.010432
2887.000000	0.010411
2886.750000	0.010393
2886.500000	0.010380
2886.250000	0.010370
2886.000000	0.010363
2885.750000	0.010357
2885.500000	0.010352
2885.250000	0.010347
2885.000000	0.010340
2884.750000	0.010332
2884.500000	0.010321
2884.250000	0.010308
2884.000000	0.010292
2883.750000	0.010274
2883.500000	0.010254
2883.250000	0.010233
2883.000000	0.010213
2882.750000	0.010194
2882.500000	0.010178
2882.250000	0.010165
2882.000000	0.010157

2881.750000	0.010153
2881.500000	0.010152
2881.250000	0.010154
2881.000000	0.010156
2880.750000	0.010158
2880.500000	0.010159
2880.250000	0.010159
2880.000000	0.010160
2879.750000	0.010163
2879.500000	0.010170
2879.250000	0.010181
2879.000000	0.010196
2878.750000	0.010212
2878.500000	0.010229
2878.250000	0.010245
2878.000000	0.010259
2877.750000	0.010272
2877.500000	0.010285
2877.250000	0.010300
2877.000000	0.010319
2876.750000	0.010340
2876.500000	0.010363
2876.250000	0.010387
2876.000000	0.010410
2875.750000	0.010432
2875.500000	0.010453

2875.250000	0.010473
2875.000000	0.010495
2874.750000	0.010518
2874.500000	0.010541
2874.250000	0.010562
2874.000000	0.010581
2873.750000	0.010596
2873.500000	0.010607
2873.250000	0.010614
2873.000000	0.010621
2872.750000	0.010628
2872.500000	0.010638
2872.250000	0.010650
2872.000000	0.010666
2871.750000	0.010684
2871.500000	0.010704
2871.250000	0.010723
2871.000000	0.010742
2870.750000	0.010759
2870.500000	0.010775
2870.250000	0.010790
2870.000000	0.010803
2869.750000	0.010815
2869.500000	0.010827
2869.250000	0.010839
2869.000000	0.010852

2868.750000	0.010867
2868.500000	0.010883
2868.250000	0.010897
2868.000000	0.010910
2867.750000	0.010919
2867.500000	0.010923
2867.250000	0.010923
2867.000000	0.010922
2866.750000	0.010922
2866.500000	0.010928
2866.250000	0.010942
2866.000000	0.010966
2865.750000	0.010999
2865.500000	0.011040
2865.250000	0.011085
2865.000000	0.011131
2864.750000	0.011177
2864.500000	0.011219
2864.250000	0.011259
2864.000000	0.011296
2863.750000	0.011333
2863.500000	0.011371
2863.250000	0.011411
2863.000000	0.011453
2862.750000	0.011497
2862.500000	0.011543

2862.250000	0.011588
2862.000000	0.011634
2861.750000	0.011679
2861.500000	0.011726
2861.250000	0.011775
2861.000000	0.011826
2860.750000	0.011881
2860.500000	0.011940
2860.250000	0.012000
2860.000000	0.012063
2859.750000	0.012125
2859.500000	0.012186
2859.250000	0.012247
2859.000000	0.012305
2858.750000	0.012362
2858.500000	0.012416
2858.250000	0.012466
2858.000000	0.012513
2857.750000	0.012555
2857.500000	0.012593
2857.250000	0.012629
2857.000000	0.012665
2856.750000	0.012702
2856.500000	0.012741
2856.250000	0.012782
2856.000000	0.012822

2855.750000	0.012859
2855.500000	0.012889
2855.250000	0.012910
2855.000000	0.012920
2854.750000	0.012922
2854.500000	0.012916
2854.250000	0.012906
2854.000000	0.012895
2853.750000	0.012883
2853.500000	0.012872
2853.250000	0.012859
2853.000000	0.012843
2852.750000	0.012822
2852.500000	0.012794
2852.250000	0.012759
2852.000000	0.012717
2851.750000	0.012670
2851.500000	0.012617
2851.250000	0.012559
2851.000000	0.012496
2850.750000	0.012428
2850.500000	0.012353
2850.250000	0.012272
2850.000000	0.012181
2849.750000	0.012082
2849.500000	0.011971

2849.250000	0.011849
2849.000000	0.011713
2848.750000	0.011563
2848.500000	0.011399
2848.250000	0.011223
2848.000000	0.011036
2847.750000	0.010843
2847.500000	0.010647
2847.250000	0.010450
2847.000000	0.010255
2846.750000	0.010063
2846.500000	0.009874
2846.250000	0.009685
2846.000000	0.009497
2845.750000	0.009307
2845.500000	0.009115
2845.250000	0.008923
2845.000000	0.008730
2844.750000	0.008540
2844.500000	0.008354
2844.250000	0.008175
2844.000000	0.008002
2843.750000	0.007838
2843.500000	0.007682
2843.250000	0.007534
2843.000000	0.007392

2842.750000	0.007257
2842.500000	0.007128
2842.250000	0.007004
2842.000000	0.006884
2841.750000	0.006767
2841.500000	0.006653
2841.250000	0.006541
2841.000000	0.006431
2840.750000	0.006322
2840.500000	0.006217
2840.250000	0.006114
2840.000000	0.006016
2839.750000	0.005924
2839.500000	0.005839
2839.250000	0.005761
2839.000000	0.005692
2838.750000	0.005631
2838.500000	0.005576
2838.250000	0.005526
2838.000000	0.005479
2837.750000	0.005435
2837.500000	0.005390
2837.250000	0.005346
2837.000000	0.005303
2836.750000	0.005260
2836.500000	0.005218

2836.250000	0.005178
2836.000000	0.005136
2835.750000	0.005094
2835.500000	0.005048
2835.250000	0.005000
2835.000000	0.004949
2834.750000	0.004897
2834.500000	0.004848
2834.250000	0.004803
2834.000000	0.004765
2833.750000	0.004734
2833.500000	0.004711
2833.250000	0.004693
2833.000000	0.004680
2832.750000	0.004669
2832.500000	0.004658
2832.250000	0.004646
2832.000000	0.004633
2831.750000	0.004618
2831.500000	0.004601
2831.250000	0.004581
2831.000000	0.004559
2830.750000	0.004534
2830.500000	0.004509
2830.250000	0.004483
2830.000000	0.004457

2829.750000	0.004432
2829.500000	0.004407
2829.250000	0.004382
2829.000000	0.004358
2828.750000	0.004335
2828.500000	0.004314
2828.250000	0.004294
2828.000000	0.004278
2827.750000	0.004264
2827.500000	0.004253
2827.250000	0.004243
2827.000000	0.004232
2826.750000	0.004220
2826.500000	0.004205
2826.250000	0.004188
2826.000000	0.004169
2825.750000	0.004148
2825.500000	0.004127
2825.250000	0.004106
2825.000000	0.004085
2824.750000	0.004065
2824.500000	0.004045
2824.250000	0.004026
2824.000000	0.004008
2823.750000	0.003993
2823.500000	0.003980

2823.250000	0.003970
2823.000000	0.003961
2822.750000	0.003953
2822.500000	0.003945
2822.250000	0.003935
2822.000000	0.003923
2821.750000	0.003909
2821.500000	0.003891
2821.250000	0.003871
2821.000000	0.003848
2820.750000	0.003822
2820.500000	0.003796
2820.250000	0.003771
2820.000000	0.003749
2819.750000	0.003734
2819.500000	0.003725
2819.250000	0.003722
2819.000000	0.003725
2818.750000	0.003731
2818.500000	0.003737
2818.250000	0.003741
2818.000000	0.003742
2817.750000	0.003739
2817.500000	0.003734
2817.250000	0.003727
2817.000000	0.003719

2816.750000	0.003710
2816.500000	0.003701
2816.250000	0.003691
2816.000000	0.003679
2815.750000	0.003665
2815.500000	0.003649
2815.250000	0.003633
2815.000000	0.003617
2814.750000	0.003601
2814.500000	0.003586
2814.250000	0.003570
2814.000000	0.003554
2813.750000	0.003538
2813.500000	0.003521
2813.250000	0.003506
2813.000000	0.003491
2812.750000	0.003478
2812.500000	0.003466
2812.250000	0.003455
2812.000000	0.003446
2811.750000	0.003437
2811.500000	0.003429
2811.250000	0.003422
2811.000000	0.003417
2810.750000	0.003414
2810.500000	0.003412

2810.250000	0.003411
2810.000000	0.003410
2809.750000	0.003409
2809.500000	0.003408
2809.250000	0.003408
2809.000000	0.003408
2808.750000	0.003408
2808.500000	0.003407
2808.250000	0.003403
2808.000000	0.003396
2807.750000	0.003383
2807.500000	0.003368
2807.250000	0.003351
2807.000000	0.003336
2806.750000	0.003324
2806.500000	0.003316
2806.250000	0.003312
2806.000000	0.003310
2805.750000	0.003308
2805.500000	0.003304
2805.250000	0.003297
2805.000000	0.003288
2804.750000	0.003279
2804.500000	0.003271
2804.250000	0.003266
2804.000000	0.003265

2803.750000	0.003266
2803.500000	0.003268
2803.250000	0.003270
2803.000000	0.003269
2802.750000	0.003265
2802.500000	0.003259
2802.250000	0.003251
2802.000000	0.003242
2801.750000	0.003231
2801.500000	0.003219
2801.250000	0.003205
2801.000000	0.003189
2800.750000	0.003171
2800.500000	0.003152
2800.250000	0.003132
2800.000000	0.003114
2799.750000	0.003099
2799.500000	0.003089
2799.250000	0.003084
2799.000000	0.003087
2798.750000	0.003095
2798.500000	0.003108
2798.250000	0.003123
2798.000000	0.003137
2797.750000	0.003147
2797.500000	0.003152

2797.250000	0.003150
2797.000000	0.003143
2796.750000	0.003130
2796.500000	0.003117
2796.250000	0.003104
2796.000000	0.003093
2795.750000	0.003085
2795.500000	0.003079
2795.250000	0.003073
2795.000000	0.003067
2794.750000	0.003059
2794.500000	0.003049
2794.250000	0.003038
2794.000000	0.003028
2793.750000	0.003019
2793.500000	0.003013
2793.250000	0.003009
2793.000000	0.003008
2792.750000	0.003007
2792.500000	0.003007
2792.250000	0.003005
2792.000000	0.003001
2791.750000	0.002995
2791.500000	0.002986
2791.250000	0.002975
2791.000000	0.002963

2790.750000	0.002951
2790.500000	0.002940
2790.250000	0.002932
2790.000000	0.002926
2789.750000	0.002923
2789.500000	0.002922
2789.250000	0.002923
2789.000000	0.002925
2788.750000	0.002927
2788.500000	0.002927
2788.250000	0.002925
2788.000000	0.002922
2787.750000	0.002918
2787.500000	0.002914
2787.250000	0.002910
2787.000000	0.002908
2786.750000	0.002908
2786.500000	0.002909
2786.250000	0.002912
2786.000000	0.002916
2785.750000	0.002921
2785.500000	0.002925
2785.250000	0.002927
2785.000000	0.002928
2784.750000	0.002927
2784.500000	0.002923

2784.250000	0.002917
2784.000000	0.002909
2783.750000	0.002899
2783.500000	0.002889
2783.250000	0.002880
2783.000000	0.002871
2782.750000	0.002865
2782.500000	0.002859
2782.250000	0.002854
2782.000000	0.002849
2781.750000	0.002843
2781.500000	0.002835
2781.250000	0.002827
2781.000000	0.002819
2780.750000	0.002813
2780.500000	0.002809
2780.250000	0.002807
2780.000000	0.002808
2779.750000	0.002810
2779.500000	0.002812
2779.250000	0.002812
2779.000000	0.002811
2778.750000	0.002809
2778.500000	0.002804
2778.250000	0.002798
2778.000000	0.002790

2777.750000	0.002782
2777.500000	0.002772
2777.250000	0.002762
2777.000000	0.002752
2776.750000	0.002743
2776.500000	0.002738
2776.250000	0.002735
2776.000000	0.002735
2775.750000	0.002738
2775.500000	0.002743
2775.250000	0.002749
2775.000000	0.002755
2774.750000	0.002760
2774.500000	0.002766
2774.250000	0.002771
2774.000000	0.002777
2773.750000	0.002782
2773.500000	0.002786
2773.250000	0.002788
2773.000000	0.002788
2772.750000	0.002785
2772.500000	0.002780
2772.250000	0.002772
2772.000000	0.002764
2771.750000	0.002757
2771.500000	0.002752

2771.250000	0.002752
2771.000000	0.002755
2770.750000	0.002762
2770.500000	0.002770
2770.250000	0.002778
2770.000000	0.002782
2769.750000	0.002780
2769.500000	0.002771
2769.250000	0.002757
2769.000000	0.002738
2768.750000	0.002719
2768.500000	0.002702
2768.250000	0.002689
2768.000000	0.002682
2767.750000	0.002679
2767.500000	0.002680
2767.250000	0.002682
2767.000000	0.002683
2766.750000	0.002683
2766.500000	0.002682
2766.250000	0.002681
2766.000000	0.002681
2765.750000	0.002684
2765.500000	0.002689
2765.250000	0.002697
2765.000000	0.002706

2764.750000	0.002714
2764.500000	0.002721
2764.250000	0.002725
2764.000000	0.002727
2763.750000	0.002725
2763.500000	0.002721
2763.250000	0.002715
2763.000000	0.002708
2762.750000	0.002703
2762.500000	0.002700
2762.250000	0.002700
2762.000000	0.002703
2761.750000	0.002708
2761.500000	0.002715
2761.250000	0.002719
2761.000000	0.002721
2760.750000	0.002718
2760.500000	0.002711
2760.250000	0.002701
2760.000000	0.002689
2759.750000	0.002676
2759.500000	0.002665
2759.250000	0.002657
2759.000000	0.002652
2758.750000	0.002650
2758.500000	0.002649

2758.250000	0.002649
2758.000000	0.002649
2757.750000	0.002646
2757.500000	0.002641
2757.250000	0.002634
2757.000000	0.002626
2756.750000	0.002617
2756.500000	0.002611
2756.250000	0.002607
2756.000000	0.002605
2755.750000	0.002604
2755.500000	0.002605
2755.250000	0.002605
2755.000000	0.002605
2754.750000	0.002605
2754.500000	0.002605
2754.250000	0.002605
2754.000000	0.002608
2753.750000	0.002612
2753.500000	0.002616
2753.250000	0.002621
2753.000000	0.002624
2752.750000	0.002626
2752.500000	0.002625
2752.250000	0.002622
2752.000000	0.002617

2751.750000	0.002612
2751.500000	0.002607
2751.250000	0.002602
2751.000000	0.002598
2750.750000	0.002594
2750.500000	0.002592
2750.250000	0.002589
2750.000000	0.002587
2749.750000	0.002583
2749.500000	0.002576
2749.250000	0.002567
2749.000000	0.002555
2748.750000	0.002541
2748.500000	0.002529
2748.250000	0.002520
2748.000000	0.002518
2747.750000	0.002523
2747.500000	0.002534
2747.250000	0.002550
2747.000000	0.002568
2746.750000	0.002583
2746.500000	0.002596
2746.250000	0.002604
2746.000000	0.002610
2745.750000	0.002613
2745.500000	0.002617

2745.250000	0.002620
2745.000000	0.002624
2744.750000	0.002627
2744.500000	0.002628
2744.250000	0.002628
2744.000000	0.002625
2743.750000	0.002620
2743.500000	0.002614
2743.250000	0.002607
2743.000000	0.002600
2742.750000	0.002591
2742.500000	0.002583
2742.250000	0.002575
2742.000000	0.002569
2741.750000	0.002565
2741.500000	0.002564
2741.250000	0.002565
2741.000000	0.002567
2740.750000	0.002568
2740.500000	0.002565
2740.250000	0.002560
2740.000000	0.002550
2739.750000	0.002538
2739.500000	0.002526
2739.250000	0.002516
2739.000000	0.002509

2738.750000	0.002507
2738.500000	0.002509
2738.250000	0.002514
2738.000000	0.002522
2737.750000	0.002531
2737.500000	0.002540
2737.250000	0.002551
2737.000000	0.002561
2736.750000	0.002571
2736.500000	0.002578
2736.250000	0.002581
2736.000000	0.002579
2735.750000	0.002572
2735.500000	0.002561
2735.250000	0.002548
2735.000000	0.002535
2734.750000	0.002525
2734.500000	0.002518
2734.250000	0.002515
2734.000000	0.002514
2733.750000	0.002514
2733.500000	0.002513
2733.250000	0.002509
2733.000000	0.002504
2732.750000	0.002498
2732.500000	0.002493

2732.250000	0.002489
2732.000000	0.002486
2731.750000	0.002484
2731.500000	0.002481
2731.250000	0.002477
2731.000000	0.002470
2730.750000	0.002461
2730.500000	0.002450
2730.250000	0.002439
2730.000000	0.002430
2729.750000	0.002424
2729.500000	0.002421
2729.250000	0.002422
2729.000000	0.002426
2728.750000	0.002430
2728.500000	0.002432
2728.250000	0.002432
2728.000000	0.002428
2727.750000	0.002422
2727.500000	0.002415
2727.250000	0.002409
2727.000000	0.002406
2726.750000	0.002406
2726.500000	0.002409
2726.250000	0.002414
2726.000000	0.002420

2725.750000	0.002425
2725.500000	0.002428
2725.250000	0.002430
2725.000000	0.002429
2724.750000	0.002428
2724.500000	0.002425
2724.250000	0.002422
2724.000000	0.002417
2723.750000	0.002409
2723.500000	0.002398
2723.250000	0.002385
2723.000000	0.002370
2722.750000	0.002357
2722.500000	0.002348
2722.250000	0.002344
2722.000000	0.002346
2721.750000	0.002355
2721.500000	0.002368
2721.250000	0.002382
2721.000000	0.002396
2720.750000	0.002407
2720.500000	0.002414
2720.250000	0.002417
2720.000000	0.002415
2719.750000	0.002410
2719.500000	0.002401

2719.250000	0.002390
2719.000000	0.002378
2718.750000	0.002365
2718.500000	0.002353
2718.250000	0.002344
2718.000000	0.002338
2717.750000	0.002335
2717.500000	0.002334
2717.250000	0.002333
2717.000000	0.002332
2716.750000	0.002330
2716.500000	0.002326
2716.250000	0.002322
2716.000000	0.002318
2715.750000	0.002315
2715.500000	0.002315
2715.250000	0.002316
2715.000000	0.002318
2714.750000	0.002320
2714.500000	0.002321
2714.250000	0.002321
2714.000000	0.002321
2713.750000	0.002322
2713.500000	0.002324
2713.250000	0.002329
2713.000000	0.002336

2712.750000	0.002344
2712.500000	0.002352
2712.250000	0.002357
2712.000000	0.002358
2711.750000	0.002355
2711.500000	0.002347
2711.250000	0.002335
2711.000000	0.002321
2710.750000	0.002306
2710.500000	0.002291
2710.250000	0.002279
2710.000000	0.002270
2709.750000	0.002265
2709.500000	0.002265
2709.250000	0.002268
2709.000000	0.002273
2708.750000	0.002278
2708.500000	0.002281
2708.250000	0.002280
2708.000000	0.002274
2707.750000	0.002264
2707.500000	0.002251
2707.250000	0.002237
2707.000000	0.002224
2706.750000	0.002215
2706.500000	0.002209

2706.250000	0.002209
2706.000000	0.002211
2705.750000	0.002216
2705.500000	0.002220
2705.250000	0.002224
2705.000000	0.002226
2704.750000	0.002226
2704.500000	0.002226
2704.250000	0.002226
2704.000000	0.002226
2703.750000	0.002226
2703.500000	0.002226
2703.250000	0.002226
2703.000000	0.002224
2702.750000	0.002222
2702.500000	0.002220
2702.250000	0.002218
2702.000000	0.002216
2701.750000	0.002215
2701.500000	0.002214
2701.250000	0.002214
2701.000000	0.002213
2700.750000	0.002213
2700.500000	0.002212
2700.250000	0.002212
2700.000000	0.002211

2699.750000	0.002211
2699.500000	0.002211
2699.250000	0.002210
2699.000000	0.002207
2698.750000	0.002204
2698.500000	0.002200
2698.250000	0.002197
2698.000000	0.002195
2697.750000	0.002196
2697.500000	0.002199
2697.250000	0.002204
2697.000000	0.002211
2696.750000	0.002218
2696.500000	0.002223
2696.250000	0.002226
2696.000000	0.002226
2695.750000	0.002224
2695.500000	0.002220
2695.250000	0.002214
2695.000000	0.002208
2694.750000	0.002203
2694.500000	0.002199
2694.250000	0.002196
2694.000000	0.002195
2693.750000	0.002196
2693.500000	0.002198

2693.250000	0.002201
2693.000000	0.002203
2692.750000	0.002205
2692.500000	0.002205
2692.250000	0.002203
2692.000000	0.002200
2691.750000	0.002195
2691.500000	0.002190
2691.250000	0.002184
2691.000000	0.002178
2690.750000	0.002173
2690.500000	0.002169
2690.250000	0.002167
2690.000000	0.002166
2689.750000	0.002168
2689.500000	0.002170
2689.250000	0.002174
2689.000000	0.002180
2688.750000	0.002185
2688.500000	0.002191
2688.250000	0.002195
2688.000000	0.002197
2687.750000	0.002195
2687.500000	0.002191
2687.250000	0.002183
2687.000000	0.002172

2686.750000	0.002162
2686.500000	0.002153
2686.250000	0.002147
2686.000000	0.002145
2685.750000	0.002147
2685.500000	0.002152
2685.250000	0.002156
2685.000000	0.002157
2684.750000	0.002155
2684.500000	0.002150
2684.250000	0.002142
2684.000000	0.002133
2683.750000	0.002125
2683.500000	0.002119
2683.250000	0.002115
2683.000000	0.002114
2682.750000	0.002115
2682.500000	0.002117
2682.250000	0.002120
2682.000000	0.002123
2681.750000	0.002127
2681.500000	0.002130
2681.250000	0.002132
2681.000000	0.002133
2680.750000	0.002131
2680.500000	0.002127

2680.250000	0.002120
2680.000000	0.002111
2679.750000	0.002103
2679.500000	0.002096
2679.250000	0.002091
2679.000000	0.002089
2678.750000	0.002089
2678.500000	0.002091
2678.250000	0.002092
2678.000000	0.002092
2677.750000	0.002092
2677.500000	0.002090
2677.250000	0.002087
2677.000000	0.002085
2676.750000	0.002083
2676.500000	0.002082
2676.250000	0.002082
2676.000000	0.002083
2675.750000	0.002085
2675.500000	0.002086
2675.250000	0.002088
2675.000000	0.002090
2674.750000	0.002091
2674.500000	0.002093
2674.250000	0.002094
2674.000000	0.002094

2673.750000	0.002092
2673.500000	0.002087
2673.250000	0.002081
2673.000000	0.002072
2672.750000	0.002062
2672.500000	0.002053
2672.250000	0.002047
2672.000000	0.002045
2671.750000	0.002049
2671.500000	0.002058
2671.250000	0.002070
2671.000000	0.002082
2670.750000	0.002092
2670.500000	0.002098
2670.250000	0.002099
2670.000000	0.002096
2669.750000	0.002091
2669.500000	0.002085
2669.250000	0.002082
2669.000000	0.002081
2668.750000	0.002084
2668.500000	0.002088
2668.250000	0.002093
2668.000000	0.002097
2667.750000	0.002099
2667.500000	0.002099

2667.250000	0.002098
2667.000000	0.002097
2666.750000	0.002095
2666.500000	0.002094
2666.250000	0.002094
2666.000000	0.002094
2665.750000	0.002094
2665.500000	0.002094
2665.250000	0.002095
2665.000000	0.002098
2664.750000	0.002102
2664.500000	0.002109
2664.250000	0.002119
2664.000000	0.002132
2663.750000	0.002145
2663.500000	0.002159
2663.250000	0.002170
2663.000000	0.002177
2662.750000	0.002180
2662.500000	0.002178
2662.250000	0.002171
2662.000000	0.002161
2661.750000	0.002148
2661.500000	0.002135
2661.250000	0.002123
2661.000000	0.002114

2660.750000	0.002108
2660.500000	0.002105
2660.250000	0.002106
2660.000000	0.002110
2659.750000	0.002115
2659.500000	0.002120
2659.250000	0.002124
2659.000000	0.002127
2658.750000	0.002130
2658.500000	0.002133
2658.250000	0.002138
2658.000000	0.002144
2657.750000	0.002153
2657.500000	0.002161
2657.250000	0.002168
2657.000000	0.002173
2656.750000	0.002174
2656.500000	0.002172
2656.250000	0.002168
2656.000000	0.002164
2655.750000	0.002160
2655.500000	0.002157
2655.250000	0.002155
2655.000000	0.002153
2654.750000	0.002150
2654.500000	0.002145

2654.250000	0.002139
2654.000000	0.002132
2653.750000	0.002127
2653.500000	0.002123
2653.250000	0.002122
2653.000000	0.002124
2652.750000	0.002127
2652.500000	0.002131
2652.250000	0.002134
2652.000000	0.002134
2651.750000	0.002133
2651.500000	0.002129
2651.250000	0.002124
2651.000000	0.002118
2650.750000	0.002113
2650.500000	0.002110
2650.250000	0.002110
2650.000000	0.002112
2649.750000	0.002116
2649.500000	0.002120
2649.250000	0.002123
2649.000000	0.002123
2648.750000	0.002117
2648.500000	0.002106
2648.250000	0.002090
2648.000000	0.002071

2647.750000	0.002054
2647.500000	0.002039
2647.250000	0.002030
2647.000000	0.002027
2646.750000	0.002029
2646.500000	0.002034
2646.250000	0.002041
2646.000000	0.002046
2645.750000	0.002049
2645.500000	0.002050
2645.250000	0.002050
2645.000000	0.002049
2644.750000	0.002049
2644.500000	0.002049
2644.250000	0.002050
2644.000000	0.002051
2643.750000	0.002051
2643.500000	0.002049
2643.250000	0.002045
2643.000000	0.002038
2642.750000	0.002030
2642.500000	0.002020
2642.250000	0.002011
2642.000000	0.002004
2641.750000	0.002000
2641.500000	0.001999

2641.250000	0.002002
2641.000000	0.002007
2640.750000	0.002012
2640.500000	0.002016
2640.250000	0.002018
2640.000000	0.002015
2639.750000	0.002009
2639.500000	0.002001
2639.250000	0.001992
2639.000000	0.001984
2638.750000	0.001978
2638.500000	0.001976
2638.250000	0.001976
2638.000000	0.001979
2637.750000	0.001983
2637.500000	0.001986
2637.250000	0.001990
2637.000000	0.001992
2636.750000	0.001993
2636.500000	0.001993
2636.250000	0.001994
2636.000000	0.001995
2635.750000	0.001997
2635.500000	0.001999
2635.250000	0.002003
2635.000000	0.002006

2634.750000	0.002008
2634.500000	0.002007
2634.250000	0.002006
2634.000000	0.002003
2633.750000	0.002001
2633.500000	0.002000
2633.250000	0.002002
2633.000000	0.002005
2632.750000	0.002009
2632.500000	0.002011
2632.250000	0.002009
2632.000000	0.002002
2631.750000	0.001991
2631.500000	0.001977
2631.250000	0.001962
2631.000000	0.001950
2630.750000	0.001943
2630.500000	0.001942
2630.250000	0.001945
2630.000000	0.001951
2629.750000	0.001959
2629.500000	0.001964
2629.250000	0.001967
2629.000000	0.001965
2628.750000	0.001959
2628.500000	0.001949

2628.250000	0.001936
2628.000000	0.001920
2627.750000	0.001904
2627.500000	0.001888
2627.250000	0.001873
2627.000000	0.001861
2626.750000	0.001853
2626.500000	0.001848
2626.250000	0.001848
2626.000000	0.001852
2625.750000	0.001859
2625.500000	0.001869
2625.250000	0.001879
2625.000000	0.001888
2624.750000	0.001896
2624.500000	0.001900
2624.250000	0.001901
2624.000000	0.001899
2623.750000	0.001895
2623.500000	0.001889
2623.250000	0.001885
2623.000000	0.001882
2622.750000	0.001882
2622.500000	0.001886
2622.250000	0.001892
2622.000000	0.001900

2621.750000	0.001908
2621.500000	0.001914
2621.250000	0.001917
2621.000000	0.001915
2620.750000	0.001907
2620.500000	0.001893
2620.250000	0.001872
2620.000000	0.001847
2619.750000	0.001819
2619.500000	0.001791
2619.250000	0.001767
2619.000000	0.001748
2618.750000	0.001738
2618.500000	0.001738
2618.250000	0.001746
2618.000000	0.001763
2617.750000	0.001785
2617.500000	0.001810
2617.250000	0.001837
2617.000000	0.001861
2616.750000	0.001880
2616.500000	0.001894
2616.250000	0.001900
2616.000000	0.001899
2615.750000	0.001890
2615.500000	0.001876

2615.250000	0.001859
2615.000000	0.001840
2614.750000	0.001822
2614.500000	0.001806
2614.250000	0.001793
2614.000000	0.001783
2613.750000	0.001776
2613.500000	0.001773
2613.250000	0.001773
2613.000000	0.001777
2612.750000	0.001785
2612.500000	0.001795
2612.250000	0.001809
2612.000000	0.001823
2611.750000	0.001838
2611.500000	0.001850
2611.250000	0.001860
2611.000000	0.001865
2610.750000	0.001865
2610.500000	0.001860
2610.250000	0.001850
2610.000000	0.001835
2609.750000	0.001815
2609.500000	0.001793
2609.250000	0.001772
2609.000000	0.001752

2608.750000	0.001737
2608.500000	0.001729
2608.250000	0.001727
2608.000000	0.001732
2607.750000	0.001742
2607.500000	0.001756
2607.250000	0.001770
2607.000000	0.001782
2606.750000	0.001792
2606.500000	0.001798
2606.250000	0.001799
2606.000000	0.001795
2605.750000	0.001788
2605.500000	0.001777
2605.250000	0.001764
2605.000000	0.001750
2604.750000	0.001739
2604.500000	0.001729
2604.250000	0.001723
2604.000000	0.001720
2603.750000	0.001718
2603.500000	0.001715
2603.250000	0.001711
2603.000000	0.001703
2602.750000	0.001693
2602.500000	0.001681

2602.250000	0.001668
2602.000000	0.001657
2601.750000	0.001648
2601.500000	0.001642
2601.250000	0.001640
2601.000000	0.001639
2600.750000	0.001640
2600.500000	0.001642
2600.250000	0.001645
2600.000000	0.001649
2599.750000	0.001655
2599.500000	0.001664
2599.250000	0.001674
2599.000000	0.001684
2598.750000	0.001693
2598.500000	0.001697
2598.250000	0.001696
2598.000000	0.001689
2597.750000	0.001679
2597.500000	0.001669
2597.250000	0.001662
2597.000000	0.001662
2596.750000	0.001670
2596.500000	0.001686
2596.250000	0.001706
2596.000000	0.001726

2595.750000	0.001742
2595.500000	0.001750
2595.250000	0.001747
2595.000000	0.001735
2594.750000	0.001715
2594.500000	0.001691
2594.250000	0.001668
2594.000000	0.001649
2593.750000	0.001639
2593.500000	0.001638
2593.250000	0.001645
2593.000000	0.001659
2592.750000	0.001675
2592.500000	0.001691
2592.250000	0.001704
2592.000000	0.001712
2591.750000	0.001716
2591.500000	0.001719
2591.250000	0.001724
2591.000000	0.001731
2590.750000	0.001743
2590.500000	0.001759
2590.250000	0.001775
2590.000000	0.001789
2589.750000	0.001797
2589.500000	0.001798

2589.250000	0.001792
2589.000000	0.001780
2588.750000	0.001765
2588.500000	0.001750
2588.250000	0.001737
2588.000000	0.001729
2587.750000	0.001725
2587.500000	0.001725
2587.250000	0.001728
2587.000000	0.001732
2586.750000	0.001736
2586.500000	0.001738
2586.250000	0.001734
2586.000000	0.001724
2585.750000	0.001708
2585.500000	0.001686
2585.250000	0.001663
2585.000000	0.001642
2584.750000	0.001627
2584.500000	0.001620
2584.250000	0.001624
2584.000000	0.001637
2583.750000	0.001658
2583.500000	0.001682
2583.250000	0.001707
2583.000000	0.001730

2582.750000	0.001752
2582.500000	0.001769
2582.250000	0.001783
2582.000000	0.001792
2581.750000	0.001796
2581.500000	0.001794
2581.250000	0.001788
2581.000000	0.001779
2580.750000	0.001769
2580.500000	0.001760
2580.250000	0.001756
2580.000000	0.001757
2579.750000	0.001761
2579.500000	0.001767
2579.250000	0.001772
2579.000000	0.001774
2578.750000	0.001773
2578.500000	0.001767
2578.250000	0.001758
2578.000000	0.001748
2577.750000	0.001737
2577.500000	0.001726
2577.250000	0.001714
2577.000000	0.001702
2576.750000	0.001690
2576.500000	0.001679

2576.250000	0.001668
2576.000000	0.001661
2575.750000	0.001657
2575.500000	0.001657
2575.250000	0.001659
2575.000000	0.001663
2574.750000	0.001667
2574.500000	0.001671
2574.250000	0.001676
2574.000000	0.001682
2573.750000	0.001691
2573.500000	0.001702
2573.250000	0.001717
2573.000000	0.001732
2572.750000	0.001747
2572.500000	0.001757
2572.250000	0.001760
2572.000000	0.001756
2571.750000	0.001744
2571.500000	0.001726
2571.250000	0.001705
2571.000000	0.001684
2570.750000	0.001666
2570.500000	0.001651
2570.250000	0.001641
2570.000000	0.001635

2569.750000	0.001630
2569.500000	0.001626
2569.250000	0.001622
2569.000000	0.001619
2568.750000	0.001618
2568.500000	0.001618
2568.250000	0.001622
2568.000000	0.001629
2567.750000	0.001638
2567.500000	0.001647
2567.250000	0.001654
2567.000000	0.001659
2566.750000	0.001660
2566.500000	0.001658
2566.250000	0.001652
2566.000000	0.001644
2565.750000	0.001636
2565.500000	0.001628
2565.250000	0.001621
2565.000000	0.001618
2564.750000	0.001619
2564.500000	0.001622
2564.250000	0.001628
2564.000000	0.001635
2563.750000	0.001640
2563.500000	0.001643

2563.250000	0.001643
2563.000000	0.001642
2562.750000	0.001641
2562.500000	0.001642
2562.250000	0.001648
2562.000000	0.001660
2561.750000	0.001676
2561.500000	0.001694
2561.250000	0.001711
2561.000000	0.001723
2560.750000	0.001728
2560.500000	0.001726
2560.250000	0.001716
2560.000000	0.001703
2559.750000	0.001687
2559.500000	0.001673
2559.250000	0.001663
2559.000000	0.001659
2558.750000	0.001660
2558.500000	0.001666
2558.250000	0.001675
2558.000000	0.001685
2557.750000	0.001694
2557.500000	0.001700
2557.250000	0.001701
2557.000000	0.001697

2556.750000	0.001687
2556.500000	0.001672
2556.250000	0.001654
2556.000000	0.001635
2555.750000	0.001615
2555.500000	0.001596
2555.250000	0.001579
2555.000000	0.001566
2554.750000	0.001558
2554.500000	0.001555
2554.250000	0.001559
2554.000000	0.001568
2553.750000	0.001581
2553.500000	0.001596
2553.250000	0.001609
2553.000000	0.001617
2552.750000	0.001619
2552.500000	0.001615
2552.250000	0.001607
2552.000000	0.001598
2551.750000	0.001591
2551.500000	0.001589
2551.250000	0.001592
2551.000000	0.001601
2550.750000	0.001611
2550.500000	0.001620

2550.250000	0.001627
2550.000000	0.001629
2549.750000	0.001630
2549.500000	0.001631
2549.250000	0.001635
2549.000000	0.001644
2548.750000	0.001659
2548.500000	0.001676
2548.250000	0.001695
2548.000000	0.001709
2547.750000	0.001715
2547.500000	0.001710
2547.250000	0.001695
2547.000000	0.001670
2546.750000	0.001638
2546.500000	0.001604
2546.250000	0.001571
2546.000000	0.001544
2545.750000	0.001525
2545.500000	0.001515
2545.250000	0.001513
2545.000000	0.001517
2544.750000	0.001523
2544.500000	0.001529
2544.250000	0.001530
2544.000000	0.001527

2543.750000	0.001519
2543.500000	0.001508
2543.250000	0.001497
2543.000000	0.001490
2542.750000	0.001489
2542.500000	0.001494
2542.250000	0.001503
2542.000000	0.001515
2541.750000	0.001525
2541.500000	0.001530
2541.250000	0.001528
2541.000000	0.001520
2540.750000	0.001505
2540.500000	0.001487
2540.250000	0.001469
2540.000000	0.001452
2539.750000	0.001439
2539.500000	0.001431
2539.250000	0.001428
2539.000000	0.001433
2538.750000	0.001442
2538.500000	0.001457
2538.250000	0.001476
2538.000000	0.001497
2537.750000	0.001519
2537.500000	0.001540

2537.250000	0.001558
2537.000000	0.001574
2536.750000	0.001587
2536.500000	0.001597
2536.250000	0.001603
2536.000000	0.001606
2535.750000	0.001607
2535.500000	0.001605
2535.250000	0.001601
2535.000000	0.001597
2534.750000	0.001593
2534.500000	0.001589
2534.250000	0.001585
2534.000000	0.001580
2533.750000	0.001576
2533.500000	0.001571
2533.250000	0.001567
2533.000000	0.001565
2532.750000	0.001564
2532.500000	0.001567
2532.250000	0.001571
2532.000000	0.001575
2531.750000	0.001577
2531.500000	0.001576
2531.250000	0.001570
2531.000000	0.001560

2530.750000	0.001548
2530.500000	0.001537
2530.250000	0.001529
2530.000000	0.001525
2529.750000	0.001526
2529.500000	0.001532
2529.250000	0.001541
2529.000000	0.001551
2528.750000	0.001562
2528.500000	0.001571
2528.250000	0.001577
2528.000000	0.001577
2527.750000	0.001570
2527.500000	0.001556
2527.250000	0.001534
2527.000000	0.001506
2526.750000	0.001479
2526.500000	0.001455
2526.250000	0.001442
2526.000000	0.001440
2525.750000	0.001451
2525.500000	0.001471
2525.250000	0.001497
2525.000000	0.001521
2524.750000	0.001540
2524.500000	0.001551

2524.250000	0.001553
2524.000000	0.001549
2523.750000	0.001542
2523.500000	0.001535
2523.250000	0.001531
2523.000000	0.001532
2522.750000	0.001537
2522.500000	0.001545
2522.250000	0.001555
2522.000000	0.001563
2521.750000	0.001567
2521.500000	0.001568
2521.250000	0.001563
2521.000000	0.001556
2520.750000	0.001547
2520.500000	0.001539
2520.250000	0.001535
2520.000000	0.001535
2519.750000	0.001539
2519.500000	0.001545
2519.250000	0.001551
2519.000000	0.001554
2518.750000	0.001552
2518.500000	0.001543
2518.250000	0.001530
2518.000000	0.001514

2517.750000	0.001497
2517.500000	0.001482
2517.250000	0.001471
2517.000000	0.001464
2516.750000	0.001459
2516.500000	0.001457
2516.250000	0.001457
2516.000000	0.001457
2515.750000	0.001458
2515.500000	0.001459
2515.250000	0.001462
2515.000000	0.001467
2514.750000	0.001473
2514.500000	0.001481
2514.250000	0.001489
2514.000000	0.001497
2513.750000	0.001505
2513.500000	0.001511
2513.250000	0.001517
2513.000000	0.001522
2512.750000	0.001526
2512.500000	0.001531
2512.250000	0.001535
2512.000000	0.001538
2511.750000	0.001539
2511.500000	0.001538

2511.250000	0.001534
2511.000000	0.001528
2510.750000	0.001520
2510.500000	0.001511
2510.250000	0.001503
2510.000000	0.001497
2509.750000	0.001493
2509.500000	0.001492
2509.250000	0.001493
2509.000000	0.001494
2508.750000	0.001494
2508.500000	0.001492
2508.250000	0.001489
2508.000000	0.001482
2507.750000	0.001474
2507.500000	0.001463
2507.250000	0.001452
2507.000000	0.001442
2506.750000	0.001432
2506.500000	0.001427
2506.250000	0.001426
2506.000000	0.001431
2505.750000	0.001443
2505.500000	0.001461
2505.250000	0.001483
2505.000000	0.001507

2504.750000	0.001528
2504.500000	0.001544
2504.250000	0.001553
2504.000000	0.001555
2503.750000	0.001549
2503.500000	0.001538
2503.250000	0.001524
2503.000000	0.001510
2502.750000	0.001497
2502.500000	0.001487
2502.250000	0.001481
2502.000000	0.001478
2501.750000	0.001478
2501.500000	0.001482
2501.250000	0.001489
2501.000000	0.001498
2500.750000	0.001509
2500.500000	0.001520
2500.250000	0.001529
2500.000000	0.001537
2499.750000	0.001542
2499.500000	0.001544
2499.250000	0.001546
2499.000000	0.001548
2498.750000	0.001552
2498.500000	0.001556

2498.250000	0.001562
2498.000000	0.001567
2497.750000	0.001571
2497.500000	0.001573
2497.250000	0.001572
2497.000000	0.001569
2496.750000	0.001565
2496.500000	0.001562
2496.250000	0.001560
2496.000000	0.001559
2495.750000	0.001558
2495.500000	0.001556
2495.250000	0.001552
2495.000000	0.001543
2494.750000	0.001531
2494.500000	0.001514
2494.250000	0.001492
2494.000000	0.001466
2493.750000	0.001438
2493.500000	0.001411
2493.250000	0.001386
2493.000000	0.001367
2492.750000	0.001357
2492.500000	0.001357
2492.250000	0.001366
2492.000000	0.001383

2491.750000	0.001403
2491.500000	0.001422
2491.250000	0.001437
2491.000000	0.001445
2490.750000	0.001448
2490.500000	0.001447
2490.250000	0.001448
2490.000000	0.001455
2489.750000	0.001470
2489.500000	0.001494
2489.250000	0.001525
2489.000000	0.001558
2488.750000	0.001587
2488.500000	0.001608
2488.250000	0.001615
2488.000000	0.001609
2487.750000	0.001590
2487.500000	0.001560
2487.250000	0.001525
2487.000000	0.001489
2486.750000	0.001458
2486.500000	0.001436
2486.250000	0.001425
2486.000000	0.001425
2485.750000	0.001437
2485.500000	0.001458

2485.250000	0.001484
2485.000000	0.001514
2484.750000	0.001542
2484.500000	0.001566
2484.250000	0.001583
2484.000000	0.001592
2483.750000	0.001592
2483.500000	0.001582
2483.250000	0.001564
2483.000000	0.001539
2482.750000	0.001509
2482.500000	0.001477
2482.250000	0.001447
2482.000000	0.001422
2481.750000	0.001405
2481.500000	0.001397
2481.250000	0.001397
2481.000000	0.001405
2480.750000	0.001418
2480.500000	0.001431
2480.250000	0.001442
2480.000000	0.001448
2479.750000	0.001449
2479.500000	0.001444
2479.250000	0.001436
2479.000000	0.001428

2478.750000	0.001422
2478.500000	0.001422
2478.250000	0.001429
2478.000000	0.001445
2477.750000	0.001468
2477.500000	0.001496
2477.250000	0.001526
2477.000000	0.001554
2476.750000	0.001575
2476.500000	0.001587
2476.250000	0.001589
2476.000000	0.001580
2475.750000	0.001565
2475.500000	0.001547
2475.250000	0.001530
2475.000000	0.001517
2474.750000	0.001510
2474.500000	0.001508
2474.250000	0.001509
2474.000000	0.001510
2473.750000	0.001512
2473.500000	0.001512
2473.250000	0.001513
2473.000000	0.001516
2472.750000	0.001521
2472.500000	0.001529

2472.250000	0.001536
2472.000000	0.001541
2471.750000	0.001540
2471.500000	0.001532
2471.250000	0.001517
2471.000000	0.001498
2470.750000	0.001480
2470.500000	0.001467
2470.250000	0.001460
2470.000000	0.001462
2469.750000	0.001471
2469.500000	0.001483
2469.250000	0.001496
2469.000000	0.001507
2468.750000	0.001515
2468.500000	0.001520
2468.250000	0.001524
2468.000000	0.001528
2467.750000	0.001533
2467.500000	0.001540
2467.250000	0.001547
2467.000000	0.001552
2466.750000	0.001553
2466.500000	0.001550
2466.250000	0.001541
2466.000000	0.001528

2465.750000	0.001512
2465.500000	0.001496
2465.250000	0.001482
2465.000000	0.001472
2464.750000	0.001466
2464.500000	0.001465
2464.250000	0.001468
2464.000000	0.001476
2463.750000	0.001487
2463.500000	0.001501
2463.250000	0.001516
2463.000000	0.001530
2462.750000	0.001541
2462.500000	0.001547
2462.250000	0.001546
2462.000000	0.001538
2461.750000	0.001525
2461.500000	0.001510
2461.250000	0.001497
2461.000000	0.001490
2460.750000	0.001492
2460.500000	0.001503
2460.250000	0.001521
2460.000000	0.001540
2459.750000	0.001557
2459.500000	0.001567

2459.250000	0.001569
2459.000000	0.001562
2458.750000	0.001547
2458.500000	0.001528
2458.250000	0.001506
2458.000000	0.001485
2457.750000	0.001467
2457.500000	0.001456
2457.250000	0.001452
2457.000000	0.001457
2456.750000	0.001472
2456.500000	0.001494
2456.250000	0.001522
2456.000000	0.001551
2455.750000	0.001576
2455.500000	0.001594
2455.250000	0.001603
2455.000000	0.001602
2454.750000	0.001595
2454.500000	0.001585
2454.250000	0.001576
2454.000000	0.001569
2453.750000	0.001566
2453.500000	0.001566
2453.250000	0.001570
2453.000000	0.001574

2452.750000	0.001577
2452.500000	0.001578
2452.250000	0.001573
2452.000000	0.001564
2451.750000	0.001549
2451.500000	0.001530
2451.250000	0.001509
2451.000000	0.001490
2450.750000	0.001473
2450.500000	0.001462
2450.250000	0.001454
2450.000000	0.001449
2449.750000	0.001445
2449.500000	0.001439
2449.250000	0.001432
2449.000000	0.001425
2448.750000	0.001422
2448.500000	0.001425
2448.250000	0.001437
2448.000000	0.001456
2447.750000	0.001479
2447.500000	0.001502
2447.250000	0.001519
2447.000000	0.001529
2446.750000	0.001529
2446.500000	0.001522

2446.250000	0.001510
2446.000000	0.001495
2445.750000	0.001481
2445.500000	0.001466
2445.250000	0.001452
2445.000000	0.001438
2444.750000	0.001424
2444.500000	0.001412
2444.250000	0.001404
2444.000000	0.001400
2443.750000	0.001401
2443.500000	0.001405
2443.250000	0.001412
2443.000000	0.001417
2442.750000	0.001418
2442.500000	0.001415
2442.250000	0.001409
2442.000000	0.001400
2441.750000	0.001391
2441.500000	0.001383
2441.250000	0.001380
2441.000000	0.001380
2440.750000	0.001384
2440.500000	0.001391
2440.250000	0.001401
2440.000000	0.001411

2439.750000	0.001422
2439.500000	0.001431
2439.250000	0.001440
2439.000000	0.001449
2438.750000	0.001459
2438.500000	0.001472
2438.250000	0.001489
2438.000000	0.001510
2437.750000	0.001534
2437.500000	0.001559
2437.250000	0.001582
2437.000000	0.001601
2436.750000	0.001614
2436.500000	0.001621
2436.250000	0.001623
2436.000000	0.001621
2435.750000	0.001616
2435.500000	0.001608
2435.250000	0.001596
2435.000000	0.001579
2434.750000	0.001556
2434.500000	0.001529
2434.250000	0.001500
2434.000000	0.001472
2433.750000	0.001450
2433.500000	0.001434

2433.250000	0.001426
2433.000000	0.001424
2432.750000	0.001426
2432.500000	0.001427
2432.250000	0.001428
2432.000000	0.001427
2431.750000	0.001426
2431.500000	0.001428
2431.250000	0.001435
2431.000000	0.001448
2430.750000	0.001465
2430.500000	0.001485
2430.250000	0.001503
2430.000000	0.001515
2429.750000	0.001521
2429.500000	0.001520
2429.250000	0.001513
2429.000000	0.001503
2428.750000	0.001493
2428.500000	0.001484
2428.250000	0.001478
2428.000000	0.001473
2427.750000	0.001470
2427.500000	0.001467
2427.250000	0.001462
2427.000000	0.001457

2426.750000	0.001451
2426.500000	0.001444
2426.250000	0.001438
2426.000000	0.001432
2425.750000	0.001429
2425.500000	0.001428
2425.250000	0.001430
2425.000000	0.001436
2424.750000	0.001445
2424.500000	0.001455
2424.250000	0.001465
2424.000000	0.001472
2423.750000	0.001475
2423.500000	0.001473
2423.250000	0.001467
2423.000000	0.001460
2422.750000	0.001451
2422.500000	0.001445
2422.250000	0.001440
2422.000000	0.001437
2421.750000	0.001436
2421.500000	0.001434
2421.250000	0.001434
2421.000000	0.001434
2420.750000	0.001434
2420.500000	0.001434

2420.250000	0.001435
2420.000000	0.001433
2419.750000	0.001429
2419.500000	0.001422
2419.250000	0.001413
2419.000000	0.001405
2418.750000	0.001399
2418.500000	0.001398
2418.250000	0.001401
2418.000000	0.001406
2417.750000	0.001409
2417.500000	0.001408
2417.250000	0.001401
2417.000000	0.001390
2416.750000	0.001377
2416.500000	0.001368
2416.250000	0.001366
2416.000000	0.001374
2415.750000	0.001391
2415.500000	0.001414
2415.250000	0.001438
2415.000000	0.001457
2414.750000	0.001466
2414.500000	0.001465
2414.250000	0.001453
2414.000000	0.001432

2413.750000	0.001408
2413.500000	0.001385
2413.250000	0.001366
2413.000000	0.001354
2412.750000	0.001350
2412.500000	0.001355
2412.250000	0.001365
2412.000000	0.001378
2411.750000	0.001388
2411.500000	0.001394
2411.250000	0.001393
2411.000000	0.001385
2410.750000	0.001375
2410.500000	0.001366
2410.250000	0.001362
2410.000000	0.001365
2409.750000	0.001377
2409.500000	0.001394
2409.250000	0.001414
2409.000000	0.001431
2408.750000	0.001441
2408.500000	0.001441
2408.250000	0.001431
2408.000000	0.001412
2407.750000	0.001386
2407.500000	0.001357

2407.250000	0.001329
2407.000000	0.001305
2406.750000	0.001287
2406.500000	0.001276
2406.250000	0.001274
2406.000000	0.001278
2405.750000	0.001289
2405.500000	0.001304
2405.250000	0.001322
2405.000000	0.001342
2404.750000	0.001362
2404.500000	0.001380
2404.250000	0.001396
2404.000000	0.001408
2403.750000	0.001415
2403.500000	0.001416
2403.250000	0.001411
2403.000000	0.001401
2402.750000	0.001387
2402.500000	0.001371
2402.250000	0.001355
2402.000000	0.001339
2401.750000	0.001325
2401.500000	0.001314
2401.250000	0.001306
2401.000000	0.001303

2400.750000	0.001307
2400.500000	0.001318
2400.250000	0.001335
2400.000000	0.001357
2399.750000	0.001381
2399.500000	0.001401
2399.250000	0.001416
2399.000000	0.001422
2398.750000	0.001421
2398.500000	0.001413
2398.250000	0.001402
2398.000000	0.001391
2397.750000	0.001381
2397.500000	0.001374
2397.250000	0.001370
2397.000000	0.001368
2396.750000	0.001367
2396.500000	0.001369
2396.250000	0.001372
2396.000000	0.001379
2395.750000	0.001387
2395.500000	0.001395
2395.250000	0.001402
2395.000000	0.001406
2394.750000	0.001406
2394.500000	0.001403

2394.250000	0.001398
2394.000000	0.001395
2393.750000	0.001394
2393.500000	0.001396
2393.250000	0.001399
2393.000000	0.001402
2392.750000	0.001401
2392.500000	0.001395
2392.250000	0.001381
2392.000000	0.001360
2391.750000	0.001336
2391.500000	0.001311
2391.250000	0.001290
2391.000000	0.001276
2390.750000	0.001270
2390.500000	0.001271
2390.250000	0.001276
2390.000000	0.001282
2389.750000	0.001286
2389.500000	0.001285
2389.250000	0.001280
2389.000000	0.001273
2388.750000	0.001267
2388.500000	0.001266
2388.250000	0.001271
2388.000000	0.001284

2387.750000	0.001300
2387.500000	0.001316
2387.250000	0.001329
2387.000000	0.001335
2386.750000	0.001336
2386.500000	0.001332
2386.250000	0.001327
2386.000000	0.001324
2385.750000	0.001324
2385.500000	0.001328
2385.250000	0.001335
2385.000000	0.001341
2384.750000	0.001346
2384.500000	0.001346
2384.250000	0.001343
2384.000000	0.001336
2383.750000	0.001326
2383.500000	0.001315
2383.250000	0.001303
2383.000000	0.001290
2382.750000	0.001279
2382.500000	0.001271
2382.250000	0.001265
2382.000000	0.001263
2381.750000	0.001263
2381.500000	0.001263

2381.250000	0.001259
2381.000000	0.001249
2380.750000	0.001231
2380.500000	0.001207
2380.250000	0.001180
2380.000000	0.001153
2379.750000	0.001132
2379.500000	0.001118
2379.250000	0.001113
2379.000000	0.001114
2378.750000	0.001118
2378.500000	0.001123
2378.250000	0.001124
2378.000000	0.001122
2377.750000	0.001117
2377.500000	0.001111
2377.250000	0.001107
2377.000000	0.001106
2376.750000	0.001108
2376.500000	0.001111
2376.250000	0.001114
2376.000000	0.001113
2375.750000	0.001108
2375.500000	0.001099
2375.250000	0.001088
2375.000000	0.001076

2374.750000	0.001068
2374.500000	0.001063
2374.250000	0.001062
2374.000000	0.001062
2373.750000	0.001058
2373.500000	0.001049
2373.250000	0.001031
2373.000000	0.001007
2372.750000	0.000980
2372.500000	0.000956
2372.250000	0.000940
2372.000000	0.000936
2371.750000	0.000942
2371.500000	0.000951
2371.250000	0.000953
2371.000000	0.000938
2370.750000	0.000895
2370.500000	0.000822
2370.250000	0.000719
2370.000000	0.000598
2369.750000	0.000474
2369.500000	0.000364
2369.250000	0.000287
2369.000000	0.000257
2368.750000	0.000282
2368.500000	0.000360

2368.250000	0.000482
2368.000000	0.000632
2367.750000	0.000789
2367.500000	0.000934
2367.250000	0.001049
2367.000000	0.001124
2366.750000	0.001157
2366.500000	0.001153
2366.250000	0.001123
2366.000000	0.001078
2365.750000	0.001030
2365.500000	0.000987
2365.250000	0.000951
2365.000000	0.000924
2364.750000	0.000903
2364.500000	0.000886
2364.250000	0.000872
2364.000000	0.000859
2363.750000	0.000847
2363.500000	0.000837
2363.250000	0.000831
2363.000000	0.000828
2362.750000	0.000831
2362.500000	0.000842
2362.250000	0.000861
2362.000000	0.000887

2361.750000	0.000919
2361.500000	0.000954
2361.250000	0.000986
2361.000000	0.001010
2360.750000	0.001024
2360.500000	0.001026
2360.250000	0.001019
2360.000000	0.001006
2359.750000	0.000992
2359.500000	0.000980
2359.250000	0.000972
2359.000000	0.000967
2358.750000	0.000962
2358.500000	0.000955
2358.250000	0.000942
2358.000000	0.000924
2357.750000	0.000901
2357.500000	0.000880
2357.250000	0.000865
2357.000000	0.000862
2356.750000	0.000874
2356.500000	0.000901
2356.250000	0.000942
2356.000000	0.000989
2355.750000	0.001038
2355.500000	0.001082

2355.250000	0.001117
2355.000000	0.001143
2354.750000	0.001162
2354.500000	0.001176
2354.250000	0.001188
2354.000000	0.001200
2353.750000	0.001212
2353.500000	0.001220
2353.250000	0.001223
2353.000000	0.001218
2352.750000	0.001205
2352.500000	0.001187
2352.250000	0.001166
2352.000000	0.001146
2351.750000	0.001131
2351.500000	0.001121
2351.250000	0.001115
2351.000000	0.001109
2350.750000	0.001102
2350.500000	0.001092
2350.250000	0.001081
2350.000000	0.001071
2349.750000	0.001064
2349.500000	0.001061
2349.250000	0.001065
2349.000000	0.001073

2348.750000	0.001085
2348.500000	0.001099
2348.250000	0.001113
2348.000000	0.001126
2347.750000	0.001137
2347.500000	0.001144
2347.250000	0.001146
2347.000000	0.001144
2346.750000	0.001136
2346.500000	0.001123
2346.250000	0.001108
2346.000000	0.001095
2345.750000	0.001085
2345.500000	0.001082
2345.250000	0.001088
2345.000000	0.001103
2344.750000	0.001123
2344.500000	0.001146
2344.250000	0.001167
2344.000000	0.001184
2343.750000	0.001196
2343.500000	0.001202
2343.250000	0.001206
2343.000000	0.001209
2342.750000	0.001213
2342.500000	0.001219

2342.250000	0.001227
2342.000000	0.001236
2341.750000	0.001243
2341.500000	0.001247
2341.250000	0.001247
2341.000000	0.001241
2340.750000	0.001231
2340.500000	0.001220
2340.250000	0.001209
2340.000000	0.001202
2339.750000	0.001202
2339.500000	0.001209
2339.250000	0.001224
2339.000000	0.001246
2338.750000	0.001273
2338.500000	0.001302
2338.250000	0.001331
2338.000000	0.001355
2337.750000	0.001372
2337.500000	0.001377
2337.250000	0.001371
2337.000000	0.001353
2336.750000	0.001326
2336.500000	0.001297
2336.250000	0.001271
2336.000000	0.001254

2335.750000	0.001249
2335.500000	0.001255
2335.250000	0.001273
2335.000000	0.001298
2334.750000	0.001327
2334.500000	0.001357
2334.250000	0.001386
2334.000000	0.001409
2333.750000	0.001424
2333.500000	0.001429
2333.250000	0.001421
2333.000000	0.001402
2332.750000	0.001374
2332.500000	0.001342
2332.250000	0.001312
2332.000000	0.001289
2331.750000	0.001278
2331.500000	0.001279
2331.250000	0.001290
2331.000000	0.001306
2330.750000	0.001321
2330.500000	0.001333
2330.250000	0.001339
2330.000000	0.001339
2329.750000	0.001335
2329.500000	0.001329

2329.250000	0.001324
2329.000000	0.001321
2328.750000	0.001321
2328.500000	0.001323
2328.250000	0.001331
2328.000000	0.001343
2327.750000	0.001364
2327.500000	0.001395
2327.250000	0.001439
2327.000000	0.001496
2326.750000	0.001567
2326.500000	0.001649
2326.250000	0.001737
2326.000000	0.001826
2325.750000	0.001910
2325.500000	0.001983
2325.250000	0.002038
2325.000000	0.002074
2324.750000	0.002090
2324.500000	0.002086
2324.250000	0.002065
2324.000000	0.002030
2323.750000	0.001987
2323.500000	0.001940
2323.250000	0.001894
2323.000000	0.001854

2322.750000	0.001820
2322.500000	0.001793
2322.250000	0.001771
2322.000000	0.001753
2321.750000	0.001736
2321.500000	0.001720
2321.250000	0.001704
2321.000000	0.001689
2320.750000	0.001676
2320.500000	0.001665
2320.250000	0.001658
2320.000000	0.001654
2319.750000	0.001654
2319.500000	0.001656
2319.250000	0.001659
2319.000000	0.001661
2318.750000	0.001660
2318.500000	0.001655
2318.250000	0.001646
2318.000000	0.001635
2317.750000	0.001625
2317.500000	0.001622
2317.250000	0.001627
2317.000000	0.001642
2316.750000	0.001664
2316.500000	0.001687

2316.250000	0.001705
2316.000000	0.001714
2315.750000	0.001710
2315.500000	0.001693
2315.250000	0.001667
2315.000000	0.001639
2314.750000	0.001614
2314.500000	0.001597
2314.250000	0.001591
2314.000000	0.001595
2313.750000	0.001607
2313.500000	0.001622
2313.250000	0.001636
2313.000000	0.001646
2312.750000	0.001649
2312.500000	0.001644
2312.250000	0.001634
2312.000000	0.001619
2311.750000	0.001602
2311.500000	0.001585
2311.250000	0.001570
2311.000000	0.001558
2310.750000	0.001548
2310.500000	0.001541
2310.250000	0.001536
2310.000000	0.001531

2309.750000	0.001529
2309.500000	0.001529
2309.250000	0.001533
2309.000000	0.001541
2308.750000	0.001553
2308.500000	0.001567
2308.250000	0.001580
2308.000000	0.001589
2307.750000	0.001593
2307.500000	0.001593
2307.250000	0.001592
2307.000000	0.001594
2306.750000	0.001602
2306.500000	0.001617
2306.250000	0.001638
2306.000000	0.001662
2305.750000	0.001683
2305.500000	0.001697
2305.250000	0.001702
2305.000000	0.001698
2304.750000	0.001687
2304.500000	0.001672
2304.250000	0.001658
2304.000000	0.001647
2303.750000	0.001640
2303.500000	0.001636

2303.250000	0.001634
2303.000000	0.001632
2302.750000	0.001631
2302.500000	0.001630
2302.250000	0.001629
2302.000000	0.001629
2301.750000	0.001630
2301.500000	0.001631
2301.250000	0.001633
2301.000000	0.001635
2300.750000	0.001638
2300.500000	0.001644
2300.250000	0.001653
2300.000000	0.001665
2299.750000	0.001678
2299.500000	0.001690
2299.250000	0.001697
2299.000000	0.001697
2298.750000	0.001689
2298.500000	0.001675
2298.250000	0.001659
2298.000000	0.001646
2297.750000	0.001640
2297.500000	0.001644
2297.250000	0.001658
2297.000000	0.001678

2296.750000	0.001702
2296.500000	0.001724
2296.250000	0.001743
2296.000000	0.001755
2295.750000	0.001763
2295.500000	0.001765
2295.250000	0.001764
2295.000000	0.001759
2294.750000	0.001753
2294.500000	0.001745
2294.250000	0.001736
2294.000000	0.001730
2293.750000	0.001728
2293.500000	0.001733
2293.250000	0.001745
2293.000000	0.001765
2292.750000	0.001788
2292.500000	0.001811
2292.250000	0.001831
2292.000000	0.001844
2291.750000	0.001850
2291.500000	0.001849
2291.250000	0.001844
2291.000000	0.001836
2290.750000	0.001828
2290.500000	0.001819

2290.250000	0.001810
2290.000000	0.001800
2289.750000	0.001789
2289.500000	0.001777
2289.250000	0.001768
2289.000000	0.001764
2288.750000	0.001771
2288.500000	0.001790
2288.250000	0.001823
2288.000000	0.001868
2287.750000	0.001921
2287.500000	0.001975
2287.250000	0.002023
2287.000000	0.002057
2286.750000	0.002073
2286.500000	0.002070
2286.250000	0.002049
2286.000000	0.002016
2285.750000	0.001980
2285.500000	0.001948
2285.250000	0.001928
2285.000000	0.001923
2284.750000	0.001931
2284.500000	0.001948
2284.250000	0.001965
2284.000000	0.001974

2283.750000	0.001968
2283.500000	0.001944
2283.250000	0.001904
2283.000000	0.001853
2282.750000	0.001802
2282.500000	0.001760
2282.250000	0.001734
2282.000000	0.001727
2281.750000	0.001739
2281.500000	0.001764
2281.250000	0.001795
2281.000000	0.001825
2280.750000	0.001847
2280.500000	0.001858
2280.250000	0.001856
2280.000000	0.001842
2279.750000	0.001818
2279.500000	0.001786
2279.250000	0.001751
2279.000000	0.001715
2278.750000	0.001682
2278.500000	0.001655
2278.250000	0.001639
2278.000000	0.001636
2277.750000	0.001646
2277.500000	0.001668

2277.250000	0.001698
2277.000000	0.001733
2276.750000	0.001766
2276.500000	0.001793
2276.250000	0.001811
2276.000000	0.001817
2275.750000	0.001811
2275.500000	0.001795
2275.250000	0.001770
2275.000000	0.001739
2274.750000	0.001706
2274.500000	0.001674
2274.250000	0.001647
2274.000000	0.001628
2273.750000	0.001617
2273.500000	0.001614
2273.250000	0.001618
2273.000000	0.001625
2272.750000	0.001635
2272.500000	0.001646
2272.250000	0.001657
2272.000000	0.001669
2271.750000	0.001681
2271.500000	0.001691
2271.250000	0.001697
2271.000000	0.001696

2270.750000	0.001685
2270.500000	0.001663
2270.250000	0.001631
2270.000000	0.001592
2269.750000	0.001551
2269.500000	0.001515
2269.250000	0.001488
2269.000000	0.001476
2268.750000	0.001480
2268.500000	0.001498
2268.250000	0.001529
2268.000000	0.001566
2267.750000	0.001606
2267.500000	0.001643
2267.250000	0.001673
2267.000000	0.001694
2266.750000	0.001705
2266.500000	0.001706
2266.250000	0.001701
2266.000000	0.001692
2265.750000	0.001683
2265.500000	0.001676
2265.250000	0.001673
2265.000000	0.001674
2264.750000	0.001677
2264.500000	0.001683

2264.250000	0.001688
2264.000000	0.001692
2263.750000	0.001694
2263.500000	0.001695
2263.250000	0.001696
2263.000000	0.001695
2262.750000	0.001694
2262.500000	0.001691
2262.250000	0.001686
2262.000000	0.001678
2261.750000	0.001669
2261.500000	0.001660
2261.250000	0.001655
2261.000000	0.001654
2260.750000	0.001660
2260.500000	0.001671
2260.250000	0.001688
2260.000000	0.001709
2259.750000	0.001730
2259.500000	0.001751
2259.250000	0.001772
2259.000000	0.001794
2258.750000	0.001817
2258.500000	0.001842
2258.250000	0.001865
2258.000000	0.001883

2257.750000	0.001891
2257.500000	0.001884
2257.250000	0.001861
2257.000000	0.001821
2256.750000	0.001769
2256.500000	0.001715
2256.250000	0.001666
2256.000000	0.001631
2255.750000	0.001615
2255.500000	0.001619
2255.250000	0.001637
2255.000000	0.001664
2254.750000	0.001691
2254.500000	0.001710
2254.250000	0.001718
2254.000000	0.001714
2253.750000	0.001700
2253.500000	0.001679
2253.250000	0.001657
2253.000000	0.001639
2252.750000	0.001626
2252.500000	0.001622
2252.250000	0.001627
2252.000000	0.001640
2251.750000	0.001659
2251.500000	0.001681

2251.250000	0.001703
2251.000000	0.001720
2250.750000	0.001728
2250.500000	0.001726
2250.250000	0.001711
2250.000000	0.001684
2249.750000	0.001647
2249.500000	0.001604
2249.250000	0.001557
2249.000000	0.001511
2248.750000	0.001469
2248.500000	0.001435
2248.250000	0.001411
2248.000000	0.001399
2247.750000	0.001397
2247.500000	0.001403
2247.250000	0.001414
2247.000000	0.001426
2246.750000	0.001438
2246.500000	0.001451
2246.250000	0.001472
2246.000000	0.001510
2245.750000	0.001575
2245.500000	0.001675
2245.250000	0.001814
2245.000000	0.001990

2244.750000	0.002189
2244.500000	0.002393
2244.250000	0.002579
2244.000000	0.002724
2243.750000	0.002808
2243.500000	0.002821
2243.250000	0.002763
2243.000000	0.002644
2242.750000	0.002481
2242.500000	0.002300
2242.250000	0.002124
2242.000000	0.001972
2241.750000	0.001857
2241.500000	0.001783
2241.250000	0.001744
2241.000000	0.001732
2240.750000	0.001735
2240.500000	0.001746
2240.250000	0.001757
2240.000000	0.001766
2239.750000	0.001772
2239.500000	0.001776
2239.250000	0.001777
2239.000000	0.001773
2238.750000	0.001762
2238.500000	0.001743

2238.250000	0.001717
2238.000000	0.001687
2237.750000	0.001655
2237.500000	0.001626
2237.250000	0.001601
2237.000000	0.001581
2236.750000	0.001565
2236.500000	0.001554
2236.250000	0.001549
2236.000000	0.001554
2235.750000	0.001574
2235.500000	0.001611
2235.250000	0.001665
2235.000000	0.001732
2234.750000	0.001800
2234.500000	0.001858
2234.250000	0.001896
2234.000000	0.001906
2233.750000	0.001890
2233.500000	0.001853
2233.250000	0.001806
2233.000000	0.001762
2232.750000	0.001730
2232.500000	0.001714
2232.250000	0.001713
2232.000000	0.001724

2231.750000	0.001740
2231.500000	0.001755
2231.250000	0.001767
2231.000000	0.001776
2230.750000	0.001783
2230.500000	0.001791
2230.250000	0.001802
2230.000000	0.001818
2229.750000	0.001836
2229.500000	0.001855
2229.250000	0.001869
2229.000000	0.001875
2228.750000	0.001869
2228.500000	0.001850
2228.250000	0.001819
2228.000000	0.001780
2227.750000	0.001738
2227.500000	0.001701
2227.250000	0.001674
2227.000000	0.001662
2226.750000	0.001665
2226.500000	0.001683
2226.250000	0.001712
2226.000000	0.001746
2225.750000	0.001781
2225.500000	0.001813

2225.250000	0.001839
2225.000000	0.001859
2224.750000	0.001874
2224.500000	0.001885
2224.250000	0.001892
2224.000000	0.001897
2223.750000	0.001900
2223.500000	0.001902
2223.250000	0.001900
2223.000000	0.001895
2222.750000	0.001885
2222.500000	0.001869
2222.250000	0.001845
2222.000000	0.001815
2221.750000	0.001778
2221.500000	0.001739
2221.250000	0.001701
2221.000000	0.001668
2220.750000	0.001644
2220.500000	0.001631
2220.250000	0.001630
2220.000000	0.001641
2219.750000	0.001661
2219.500000	0.001687
2219.250000	0.001717
2219.000000	0.001747

2218.750000	0.001777
2218.500000	0.001803
2218.250000	0.001826
2218.000000	0.001845
2217.750000	0.001859
2217.500000	0.001867
2217.250000	0.001869
2217.000000	0.001864
2216.750000	0.001852
2216.500000	0.001833
2216.250000	0.001807
2216.000000	0.001778
2215.750000	0.001749
2215.500000	0.001727
2215.250000	0.001717
2215.000000	0.001721
2214.750000	0.001739
2214.500000	0.001767
2214.250000	0.001795
2214.000000	0.001815
2213.750000	0.001817
2213.500000	0.001798
2213.250000	0.001758
2213.000000	0.001704
2212.750000	0.001647
2212.500000	0.001596

2212.250000	0.001561
2212.000000	0.001546
2211.750000	0.001548
2211.500000	0.001561
2211.250000	0.001579
2211.000000	0.001593
2210.750000	0.001599
2210.500000	0.001596
2210.250000	0.001584
2210.000000	0.001569
2209.750000	0.001551
2209.500000	0.001535
2209.250000	0.001521
2209.000000	0.001510
2208.750000	0.001502
2208.500000	0.001498
2208.250000	0.001501
2208.000000	0.001512
2207.750000	0.001535
2207.500000	0.001570
2207.250000	0.001617
2207.000000	0.001674
2206.750000	0.001735
2206.500000	0.001794
2206.250000	0.001843
2206.000000	0.001873

2205.750000	0.001880
2205.500000	0.001862
2205.250000	0.001821
2205.000000	0.001764
2204.750000	0.001702
2204.500000	0.001648
2204.250000	0.001614
2204.000000	0.001609
2203.750000	0.001635
2203.500000	0.001689
2203.250000	0.001763
2203.000000	0.001844
2202.750000	0.001921
2202.500000	0.001983
2202.250000	0.002024
2202.000000	0.002040
2201.750000	0.002034
2201.500000	0.002010
2201.250000	0.001976
2201.000000	0.001938
2200.750000	0.001904
2200.500000	0.001880
2200.250000	0.001869
2200.000000	0.001871
2199.750000	0.001885
2199.500000	0.001906

2199.250000	0.001927
2199.000000	0.001941
2198.750000	0.001942
2198.500000	0.001928
2198.250000	0.001900
2198.000000	0.001863
2197.750000	0.001825
2197.500000	0.001792
2197.250000	0.001771
2197.000000	0.001763
2196.750000	0.001767
2196.500000	0.001777
2196.250000	0.001789
2196.000000	0.001796
2195.750000	0.001794
2195.500000	0.001786
2195.250000	0.001773
2195.000000	0.001763
2194.750000	0.001762
2194.500000	0.001773
2194.250000	0.001798
2194.000000	0.001834
2193.750000	0.001873
2193.500000	0.001907
2193.250000	0.001927
2193.000000	0.001928

2192.750000	0.001909
2192.500000	0.001874
2192.250000	0.001831
2192.000000	0.001791
2191.750000	0.001760
2191.500000	0.001746
2191.250000	0.001747
2191.000000	0.001763
2190.750000	0.001789
2190.500000	0.001818
2190.250000	0.001848
2190.000000	0.001874
2189.750000	0.001894
2189.500000	0.001907
2189.250000	0.001911
2189.000000	0.001908
2188.750000	0.001897
2188.500000	0.001880
2188.250000	0.001864
2188.000000	0.001851
2187.750000	0.001847
2187.500000	0.001853
2187.250000	0.001867
2187.000000	0.001886
2186.750000	0.001903
2186.500000	0.001913

2186.250000	0.001912
2186.000000	0.001899
2185.750000	0.001875
2185.500000	0.001844
2185.250000	0.001809
2185.000000	0.001770
2184.750000	0.001733
2184.500000	0.001699
2184.250000	0.001676
2184.000000	0.001670
2183.750000	0.001687
2183.500000	0.001729
2183.250000	0.001792
2183.000000	0.001865
2182.750000	0.001932
2182.500000	0.001974
2182.250000	0.001980
2182.000000	0.001948
2181.750000	0.001887
2181.500000	0.001815
2181.250000	0.001752
2181.000000	0.001714
2180.750000	0.001710
2180.500000	0.001737
2180.250000	0.001783
2180.000000	0.001834

2179.750000	0.001877
2179.500000	0.001905
2179.250000	0.001915
2179.000000	0.001914
2178.750000	0.001906
2178.500000	0.001897
2178.250000	0.001888
2178.000000	0.001879
2177.750000	0.001867
2177.500000	0.001850
2177.250000	0.001829
2177.000000	0.001806
2176.750000	0.001780
2176.500000	0.001753
2176.250000	0.001721
2176.000000	0.001684
2175.750000	0.001640
2175.500000	0.001590
2175.250000	0.001543
2175.000000	0.001505
2174.750000	0.001486
2174.500000	0.001490
2174.250000	0.001516
2174.000000	0.001560
2173.750000	0.001612
2173.500000	0.001663

2173.250000	0.001706
2173.000000	0.001741
2172.750000	0.001771
2172.500000	0.001801
2172.250000	0.001835
2172.000000	0.001873
2171.750000	0.001913
2171.500000	0.001948
2171.250000	0.001970
2171.000000	0.001974
2170.750000	0.001960
2170.500000	0.001930
2170.250000	0.001896
2170.000000	0.001866
2169.750000	0.001853
2169.500000	0.001863
2169.250000	0.001902
2169.000000	0.001964
2168.750000	0.002044
2168.500000	0.002131
2168.250000	0.002211
2168.000000	0.002273
2167.750000	0.002311
2167.500000	0.002320
2167.250000	0.002303
2167.000000	0.002268

2166.750000	0.002224
2166.500000	0.002180
2166.250000	0.002147
2166.000000	0.002131
2165.750000	0.002133
2165.500000	0.002155
2165.250000	0.002191
2165.000000	0.002234
2164.750000	0.002275
2164.500000	0.002305
2164.250000	0.002313
2164.000000	0.002296
2163.750000	0.002256
2163.500000	0.002200
2163.250000	0.002143
2163.000000	0.002104
2162.750000	0.002101
2162.500000	0.002142
2162.250000	0.002230
2162.000000	0.002353
2161.750000	0.002489
2161.500000	0.002614
2161.250000	0.002699
2161.000000	0.002722
2160.750000	0.002670
2160.500000	0.002537

2160.250000	0.002329
2160.000000	0.002061
2159.750000	0.001753
2159.500000	0.001429
2159.250000	0.001116
2159.000000	0.000837
2158.750000	0.000610
2158.500000	0.000446
2158.250000	0.000348
2158.000000	0.000311
2157.750000	0.000323
2157.500000	0.000372
2157.250000	0.000446
2157.000000	0.000538
2156.750000	0.000639
2156.500000	0.000744
2156.250000	0.000844
2156.000000	0.000929
2155.750000	0.000994
2155.500000	0.001032
2155.250000	0.001045
2155.000000	0.001041
2154.750000	0.001033
2154.500000	0.001034
2154.250000	0.001054
2154.000000	0.001095

2153.750000	0.001152
2153.500000	0.001212
2153.250000	0.001261
2153.000000	0.001287
2152.750000	0.001283
2152.500000	0.001253
2152.250000	0.001204
2152.000000	0.001148
2151.750000	0.001096
2151.500000	0.001054
2151.250000	0.001027
2151.000000	0.001011
2150.750000	0.001002
2150.500000	0.000995
2150.250000	0.000986
2150.000000	0.000972
2149.750000	0.000951
2149.500000	0.000926
2149.250000	0.000897
2149.000000	0.000868
2148.750000	0.000839
2148.500000	0.000814
2148.250000	0.000792
2148.000000	0.000772
2147.750000	0.000756
2147.500000	0.000742

2147.250000	0.000734
2147.000000	0.000738
2146.750000	0.000759
2146.500000	0.000806
2146.250000	0.000880
2146.000000	0.000980
2145.750000	0.001096
2145.500000	0.001214
2145.250000	0.001316
2145.000000	0.001389
2144.750000	0.001425
2144.500000	0.001424
2144.250000	0.001397
2144.000000	0.001356
2143.750000	0.001317
2143.500000	0.001288
2143.250000	0.001277
2143.000000	0.001281
2142.750000	0.001294
2142.500000	0.001310
2142.250000	0.001323
2142.000000	0.001329
2141.750000	0.001327
2141.500000	0.001319
2141.250000	0.001306
2141.000000	0.001289

2140.750000	0.001270
2140.500000	0.001249
2140.250000	0.001226
2140.000000	0.001201
2139.750000	0.001176
2139.500000	0.001149
2139.250000	0.001123
2139.000000	0.001100
2138.750000	0.001080
2138.500000	0.001068
2138.250000	0.001065
2138.000000	0.001074
2137.750000	0.001095
2137.500000	0.001126
2137.250000	0.001162
2137.000000	0.001197
2136.750000	0.001223
2136.500000	0.001237
2136.250000	0.001236
2136.000000	0.001219
2135.750000	0.001190
2135.500000	0.001150
2135.250000	0.001105
2135.000000	0.001056
2134.750000	0.001005
2134.500000	0.000956

2134.250000	0.000909
2134.000000	0.000868
2133.750000	0.000833
2133.500000	0.000807
2133.250000	0.000790
2133.000000	0.000781
2132.750000	0.000782
2132.500000	0.000791
2132.250000	0.000811
2132.000000	0.000841
2131.750000	0.000883
2131.500000	0.000934
2131.250000	0.000992
2131.000000	0.001052
2130.750000	0.001108
2130.500000	0.001158
2130.250000	0.001200
2130.000000	0.001234
2129.750000	0.001263
2129.500000	0.001287
2129.250000	0.001309
2129.000000	0.001326
2128.750000	0.001335
2128.500000	0.001333
2128.250000	0.001317
2128.000000	0.001289

2127.750000	0.001252
2127.500000	0.001211
2127.250000	0.001172
2127.000000	0.001138
2126.750000	0.001110
2126.500000	0.001089
2126.250000	0.001073
2126.000000	0.001063
2125.750000	0.001061
2125.500000	0.001069
2125.250000	0.001088
2125.000000	0.001120
2124.750000	0.001162
2124.500000	0.001208
2124.250000	0.001253
2124.000000	0.001288
2123.750000	0.001310
2123.500000	0.001316
2123.250000	0.001306
2123.000000	0.001284
2122.750000	0.001255
2122.500000	0.001223
2122.250000	0.001195
2122.000000	0.001176
2121.750000	0.001171
2121.500000	0.001183

2121.250000	0.001213
2121.000000	0.001259
2120.750000	0.001314
2120.500000	0.001369
2120.250000	0.001414
2120.000000	0.001443
2119.750000	0.001450
2119.500000	0.001439
2119.250000	0.001416
2119.000000	0.001389
2118.750000	0.001366
2118.500000	0.001352
2118.250000	0.001349
2118.000000	0.001355
2117.750000	0.001365
2117.500000	0.001377
2117.250000	0.001387
2117.000000	0.001395
2116.750000	0.001402
2116.500000	0.001409
2116.250000	0.001416
2116.000000	0.001424
2115.750000	0.001435
2115.500000	0.001447
2115.250000	0.001461
2115.000000	0.001477

2114.750000	0.001494
2114.500000	0.001513
2114.250000	0.001530
2114.000000	0.001545
2113.750000	0.001556
2113.500000	0.001563
2113.250000	0.001566
2113.000000	0.001566
2112.750000	0.001564
2112.500000	0.001560
2112.250000	0.001555
2112.000000	0.001547
2111.750000	0.001539
2111.500000	0.001532
2111.250000	0.001529
2111.000000	0.001533
2110.750000	0.001546
2110.500000	0.001568
2110.250000	0.001595
2110.000000	0.001621
2109.750000	0.001641
2109.500000	0.001649
2109.250000	0.001643
2109.000000	0.001626
2108.750000	0.001600
2108.500000	0.001570

2108.250000	0.001540
2108.000000	0.001513
2107.750000	0.001490
2107.500000	0.001470
2107.250000	0.001454
2107.000000	0.001443
2106.750000	0.001440
2106.500000	0.001445
2106.250000	0.001462
2106.000000	0.001489
2105.750000	0.001523
2105.500000	0.001561
2105.250000	0.001597
2105.000000	0.001626
2104.750000	0.001643
2104.500000	0.001646
2104.250000	0.001635
2104.000000	0.001610
2103.750000	0.001575
2103.500000	0.001534
2103.250000	0.001494
2103.000000	0.001460
2102.750000	0.001439
2102.500000	0.001432
2102.250000	0.001439
2102.000000	0.001456

2101.750000	0.001477
2101.500000	0.001495
2101.250000	0.001504
2101.000000	0.001502
2100.750000	0.001489
2100.500000	0.001470
2100.250000	0.001449
2100.000000	0.001433
2099.750000	0.001424
2099.500000	0.001422
2099.250000	0.001427
2099.000000	0.001435
2098.750000	0.001443
2098.500000	0.001446
2098.250000	0.001446
2098.000000	0.001441
2097.750000	0.001434
2097.500000	0.001427
2097.250000	0.001420
2097.000000	0.001416
2096.750000	0.001412
2096.500000	0.001409
2096.250000	0.001406
2096.000000	0.001403
2095.750000	0.001400
2095.500000	0.001399

2095.250000	0.001400
2095.000000	0.001404
2094.750000	0.001410
2094.500000	0.001416
2094.250000	0.001420
2094.000000	0.001423
2093.750000	0.001423
2093.500000	0.001424
2093.250000	0.001425
2093.000000	0.001429
2092.750000	0.001434
2092.500000	0.001437
2092.250000	0.001435
2092.000000	0.001425
2091.750000	0.001404
2091.500000	0.001376
2091.250000	0.001345
2091.000000	0.001318
2090.750000	0.001304
2090.500000	0.001305
2090.250000	0.001322
2090.000000	0.001351
2089.750000	0.001385
2089.500000	0.001416
2089.250000	0.001438
2089.000000	0.001448

2088.750000	0.001447
2088.500000	0.001439
2088.250000	0.001426
2088.000000	0.001412
2087.750000	0.001399
2087.500000	0.001388
2087.250000	0.001382
2087.000000	0.001382
2086.750000	0.001390
2086.500000	0.001408
2086.250000	0.001436
2086.000000	0.001470
2085.750000	0.001507
2085.500000	0.001541
2085.250000	0.001567
2085.000000	0.001584
2084.750000	0.001592
2084.500000	0.001597
2084.250000	0.001602
2084.000000	0.001611
2083.750000	0.001627
2083.500000	0.001647
2083.250000	0.001667
2083.000000	0.001682
2082.750000	0.001688
2082.500000	0.001681

2082.250000	0.001663
2082.000000	0.001634
2081.750000	0.001599
2081.500000	0.001560
2081.250000	0.001522
2081.000000	0.001486
2080.750000	0.001454
2080.500000	0.001428
2080.250000	0.001408
2080.000000	0.001397
2079.750000	0.001395
2079.500000	0.001402
2079.250000	0.001416
2079.000000	0.001435
2078.750000	0.001457
2078.500000	0.001479
2078.250000	0.001497
2078.000000	0.001512
2077.750000	0.001520
2077.500000	0.001525
2077.250000	0.001525
2077.000000	0.001523
2076.750000	0.001519
2076.500000	0.001514
2076.250000	0.001509
2076.000000	0.001506

2075.750000	0.001507
2075.500000	0.001514
2075.250000	0.001529
2075.000000	0.001552
2074.750000	0.001580
2074.500000	0.001609
2074.250000	0.001633
2074.000000	0.001648
2073.750000	0.001650
2073.500000	0.001640
2073.250000	0.001621
2073.000000	0.001600
2072.750000	0.001583
2072.500000	0.001576
2072.250000	0.001578
2072.000000	0.001588
2071.750000	0.001602
2071.500000	0.001614
2071.250000	0.001620
2071.000000	0.001619
2070.750000	0.001611
2070.500000	0.001599
2070.250000	0.001586
2070.000000	0.001573
2069.750000	0.001562
2069.500000	0.001554

2069.250000	0.001550
2069.000000	0.001550
2068.750000	0.001556
2068.500000	0.001566
2068.250000	0.001577
2068.000000	0.001584
2067.750000	0.001583
2067.500000	0.001570
2067.250000	0.001545
2067.000000	0.001510
2066.750000	0.001474
2066.500000	0.001445
2066.250000	0.001430
2066.000000	0.001433
2065.750000	0.001452
2065.500000	0.001481
2065.250000	0.001509
2065.000000	0.001528
2064.750000	0.001530
2064.500000	0.001512
2064.250000	0.001477
2064.000000	0.001430
2063.750000	0.001376
2063.500000	0.001324
2063.250000	0.001278
2063.000000	0.001243

2062.750000	0.001220
2062.500000	0.001211
2062.250000	0.001214
2062.000000	0.001227
2061.750000	0.001246
2061.500000	0.001269
2061.250000	0.001293
2061.000000	0.001317
2060.750000	0.001343
2060.500000	0.001374
2060.250000	0.001411
2060.000000	0.001454
2059.750000	0.001501
2059.500000	0.001547
2059.250000	0.001585
2059.000000	0.001610
2058.750000	0.001619
2058.500000	0.001613
2058.250000	0.001596
2058.000000	0.001575
2057.750000	0.001559
2057.500000	0.001551
2057.250000	0.001555
2057.000000	0.001567
2056.750000	0.001581
2056.500000	0.001589

2056.250000	0.001584
2056.000000	0.001559
2055.750000	0.001513
2055.500000	0.001449
2055.250000	0.001374
2055.000000	0.001298
2054.750000	0.001230
2054.500000	0.001177
2054.250000	0.001144
2054.000000	0.001133
2053.750000	0.001146
2053.500000	0.001181
2053.250000	0.001240
2053.000000	0.001325
2052.750000	0.001436
2052.500000	0.001572
2052.250000	0.001728
2052.000000	0.001892
2051.750000	0.002049
2051.500000	0.002181
2051.250000	0.002271
2051.000000	0.002307
2050.750000	0.002284
2050.500000	0.002208
2050.250000	0.002092
2050.000000	0.001955

2049.750000	0.001814
2049.500000	0.001685
2049.250000	0.001575
2049.000000	0.001488
2048.750000	0.001419
2048.500000	0.001365
2048.250000	0.001320
2048.000000	0.001283
2047.750000	0.001253
2047.500000	0.001230
2047.250000	0.001217
2047.000000	0.001212
2046.750000	0.001217
2046.500000	0.001228
2046.250000	0.001245
2046.000000	0.001265
2045.750000	0.001288
2045.500000	0.001312
2045.250000	0.001337
2045.000000	0.001364
2044.750000	0.001390
2044.500000	0.001414
2044.250000	0.001429
2044.000000	0.001431
2043.750000	0.001413
2043.500000	0.001372

2043.250000	0.001308
2043.000000	0.001228
2042.750000	0.001142
2042.500000	0.001067
2042.250000	0.001017
2042.000000	0.001007
2041.750000	0.001040
2041.500000	0.001112
2041.250000	0.001209
2041.000000	0.001310
2040.750000	0.001392
2040.500000	0.001441
2040.250000	0.001451
2040.000000	0.001424
2039.750000	0.001376
2039.500000	0.001324
2039.250000	0.001287
2039.000000	0.001276
2038.750000	0.001298
2038.500000	0.001350
2038.250000	0.001424
2038.000000	0.001509
2037.750000	0.001595
2037.500000	0.001674
2037.250000	0.001740
2037.000000	0.001791

2036.750000	0.001823
2036.500000	0.001833
2036.250000	0.001815
2036.000000	0.001768
2035.750000	0.001694
2035.500000	0.001597
2035.250000	0.001490
2035.000000	0.001386
2034.750000	0.001299
2034.500000	0.001239
2034.250000	0.001210
2034.000000	0.001209
2033.750000	0.001229
2033.500000	0.001260
2033.250000	0.001296
2033.000000	0.001332
2032.750000	0.001367
2032.500000	0.001399
2032.250000	0.001429
2032.000000	0.001455
2031.750000	0.001472
2031.500000	0.001479
2031.250000	0.001475
2031.000000	0.001460
2030.750000	0.001439
2030.500000	0.001418

2030.250000	0.001401
2030.000000	0.001391
2029.750000	0.001386
2029.500000	0.001384
2029.250000	0.001381
2029.000000	0.001375
2028.750000	0.001366
2028.500000	0.001357
2028.250000	0.001351
2028.000000	0.001350
2027.750000	0.001357
2027.500000	0.001368
2027.250000	0.001381
2027.000000	0.001391
2026.750000	0.001395
2026.500000	0.001391
2026.250000	0.001378
2026.000000	0.001355
2025.750000	0.001325
2025.500000	0.001290
2025.250000	0.001253
2025.000000	0.001217
2024.750000	0.001185
2024.500000	0.001162
2024.250000	0.001149
2024.000000	0.001147

2023.750000	0.001156
2023.500000	0.001173
2023.250000	0.001198
2023.000000	0.001227
2022.750000	0.001260
2022.500000	0.001293
2022.250000	0.001323
2022.000000	0.001347
2021.750000	0.001360
2021.500000	0.001360
2021.250000	0.001346
2021.000000	0.001319
2020.750000	0.001284
2020.500000	0.001245
2020.250000	0.001208
2020.000000	0.001174
2019.750000	0.001146
2019.500000	0.001125
2019.250000	0.001111
2019.000000	0.001107
2018.750000	0.001116
2018.500000	0.001142
2018.250000	0.001188
2018.000000	0.001253
2017.750000	0.001330
2017.500000	0.001406

2017.250000	0.001466
2017.000000	0.001496
2016.750000	0.001485
2016.500000	0.001435
2016.250000	0.001354
2016.000000	0.001261
2015.750000	0.001176
2015.500000	0.001116
2015.250000	0.001094
2015.000000	0.001113
2014.750000	0.001170
2014.500000	0.001254
2014.250000	0.001351
2014.000000	0.001445
2013.750000	0.001519
2013.500000	0.001566
2013.250000	0.001578
2013.000000	0.001559
2012.750000	0.001514
2012.500000	0.001455
2012.250000	0.001392
2012.000000	0.001338
2011.750000	0.001298
2011.500000	0.001277
2011.250000	0.001270
2011.000000	0.001272

2010.750000	0.001274
2010.500000	0.001269
2010.250000	0.001252
2010.000000	0.001226
2009.750000	0.001196
2009.500000	0.001168
2009.250000	0.001149
2009.000000	0.001138
2008.750000	0.001134
2008.500000	0.001127
2008.250000	0.001109
2008.000000	0.001076
2007.750000	0.001031
2007.500000	0.000982
2007.250000	0.000944
2007.000000	0.000932
2006.750000	0.000953
2006.500000	0.001008
2006.250000	0.001085
2006.000000	0.001169
2005.750000	0.001241
2005.500000	0.001287
2005.250000	0.001299
2005.000000	0.001280
2004.750000	0.001237
2004.500000	0.001179

2004.250000	0.001118
2004.000000	0.001060
2003.750000	0.001009
2003.500000	0.000968
2003.250000	0.000935
2003.000000	0.000908
2002.750000	0.000887
2002.500000	0.000871
2002.250000	0.000860
2002.000000	0.000853
2001.750000	0.000852
2001.500000	0.000857
2001.250000	0.000870
2001.000000	0.000891
2000.750000	0.000919
2000.500000	0.000952
2000.250000	0.000985
2000.000000	0.001015
1999.750000	0.001037
1999.500000	0.001048
1999.250000	0.001047
1999.000000	0.001035
1998.750000	0.001015
1998.500000	0.000990
1998.250000	0.000967
1998.000000	0.000950

1997.750000	0.000946
1997.500000	0.000962
1997.250000	0.001003
1997.000000	0.001069
1996.750000	0.001157
1996.500000	0.001260
1996.250000	0.001363
1996.000000	0.001453
1995.750000	0.001519
1995.500000	0.001554
1995.250000	0.001557
1995.000000	0.001531
1994.750000	0.001481
1994.500000	0.001412
1994.250000	0.001326
1994.000000	0.001225
1993.750000	0.001111
1993.500000	0.000990
1993.250000	0.000871
1993.000000	0.000769
1992.750000	0.000700
1992.500000	0.000675
1992.250000	0.000697
1992.000000	0.000759
1991.750000	0.000849
1991.500000	0.000949

1991.250000	0.001048
1991.000000	0.001141
1990.750000	0.001226
1990.500000	0.001304
1990.250000	0.001373
1990.000000	0.001428
1989.750000	0.001463
1989.500000	0.001471
1989.250000	0.001450
1989.000000	0.001401
1988.750000	0.001334
1988.500000	0.001260
1988.250000	0.001190
1988.000000	0.001132
1987.750000	0.001090
1987.500000	0.001065
1987.250000	0.001057
1987.000000	0.001065
1986.750000	0.001092
1986.500000	0.001140
1986.250000	0.001204
1986.000000	0.001278
1985.750000	0.001350
1985.500000	0.001405
1985.250000	0.001434
1985.000000	0.001431

1984.750000	0.001401
1984.500000	0.001354
1984.250000	0.001304
1984.000000	0.001264
1983.750000	0.001243
1983.500000	0.001244
1983.250000	0.001267
1983.000000	0.001307
1982.750000	0.001361
1982.500000	0.001423
1982.250000	0.001490
1982.000000	0.001558
1981.750000	0.001623
1981.500000	0.001685
1981.250000	0.001741
1981.000000	0.001795
1980.750000	0.001847
1980.500000	0.001900
1980.250000	0.001950
1980.000000	0.001990
1979.750000	0.002010
1979.500000	0.001997
1979.250000	0.001943
1979.000000	0.001847
1978.750000	0.001717
1978.500000	0.001568

1978.250000	0.001421
1978.000000	0.001293
1977.750000	0.001195
1977.500000	0.001131
1977.250000	0.001096
1977.000000	0.001085
1976.750000	0.001091
1976.500000	0.001110
1976.250000	0.001140
1976.000000	0.001178
1975.750000	0.001220
1975.500000	0.001258
1975.250000	0.001285
1975.000000	0.001293
1974.750000	0.001277
1974.500000	0.001239
1974.250000	0.001186
1974.000000	0.001129
1973.750000	0.001081
1973.500000	0.001051
1973.250000	0.001044
1973.000000	0.001059
1972.750000	0.001090
1972.500000	0.001126
1972.250000	0.001155
1972.000000	0.001168

1971.750000	0.001161
1971.500000	0.001136
1971.250000	0.001101
1971.000000	0.001064
1970.750000	0.001035
1970.500000	0.001018
1970.250000	0.001011
1970.000000	0.001007
1969.750000	0.000995
1969.500000	0.000968
1969.250000	0.000922
1969.000000	0.000860
1968.750000	0.000787
1968.500000	0.000716
1968.250000	0.000655
1968.000000	0.000611
1967.750000	0.000587
1967.500000	0.000580
1967.250000	0.000589
1967.000000	0.000610
1966.750000	0.000640
1966.500000	0.000678
1966.250000	0.000722
1966.000000	0.000769
1965.750000	0.000814
1965.500000	0.000856

1965.250000	0.000892
1965.000000	0.000925
1964.750000	0.000956
1964.500000	0.000985
1964.250000	0.001010
1964.000000	0.001028
1963.750000	0.001032
1963.500000	0.001022
1963.250000	0.000997
1963.000000	0.000964
1962.750000	0.000934
1962.500000	0.000916
1962.250000	0.000916
1962.000000	0.000933
1961.750000	0.000961
1961.500000	0.000988
1961.250000	0.001003
1961.000000	0.001000
1960.750000	0.000977
1960.500000	0.000938
1960.250000	0.000888
1960.000000	0.000831
1959.750000	0.000770
1959.500000	0.000706
1959.250000	0.000642
1959.000000	0.000582

1958.750000	0.000533
1958.500000	0.000501
1958.250000	0.000489
1958.000000	0.000494
1957.750000	0.000511
1957.500000	0.000530
1957.250000	0.000545
1957.000000	0.000556
1956.750000	0.000569
1956.500000	0.000593
1956.250000	0.000638
1956.000000	0.000707
1955.750000	0.000798
1955.500000	0.000899
1955.250000	0.000993
1955.000000	0.001067
1954.750000	0.001111
1954.500000	0.001125
1954.250000	0.001114
1954.000000	0.001087
1953.750000	0.001055
1953.500000	0.001023
1953.250000	0.000996
1953.000000	0.000977
1952.750000	0.000967
1952.500000	0.000966

1952.250000	0.000973
1952.000000	0.000985
1951.750000	0.000994
1951.500000	0.000992
1951.250000	0.000971
1951.000000	0.000926
1950.750000	0.000861
1950.500000	0.000785
1950.250000	0.000710
1950.000000	0.000650
1949.750000	0.000614
1949.500000	0.000606
1949.250000	0.000625
1949.000000	0.000665
1948.750000	0.000719
1948.500000	0.000780
1948.250000	0.000843
1948.000000	0.000901
1947.750000	0.000948
1947.500000	0.000979
1947.250000	0.000987
1947.000000	0.000971
1946.750000	0.000934
1946.500000	0.000884
1946.250000	0.000833
1946.000000	0.000792

1945.750000	0.000769
1945.500000	0.000766
1945.250000	0.000777
1945.000000	0.000792
1944.750000	0.000802
1944.500000	0.000798
1944.250000	0.000778
1944.000000	0.000743
1943.750000	0.000700
1943.500000	0.000657
1943.250000	0.000624
1943.000000	0.000609
1942.750000	0.000617
1942.500000	0.000651
1942.250000	0.000708
1942.000000	0.000783
1941.750000	0.000867
1941.500000	0.000947
1941.250000	0.001013
1941.000000	0.001055
1940.750000	0.001069
1940.500000	0.001059
1940.250000	0.001032
1940.000000	0.001000
1939.750000	0.000974
1939.500000	0.000962

1939.250000	0.000968
1939.000000	0.000992
1938.750000	0.001026
1938.500000	0.001061
1938.250000	0.001086
1938.000000	0.001094
1937.750000	0.001081
1937.500000	0.001047
1937.250000	0.000999
1937.000000	0.000944
1936.750000	0.000892
1936.500000	0.000852
1936.250000	0.000829
1936.000000	0.000823
1935.750000	0.000833
1935.500000	0.000852
1935.250000	0.000872
1935.000000	0.000887
1934.750000	0.000892
1934.500000	0.000884
1934.250000	0.000864
1934.000000	0.000837
1933.750000	0.000808
1933.500000	0.000782
1933.250000	0.000764
1933.000000	0.000758

1932.750000	0.000764
1932.500000	0.000782
1932.250000	0.000809
1932.000000	0.000842
1931.750000	0.000877
1931.500000	0.000908
1931.250000	0.000933
1931.000000	0.000952
1930.750000	0.000965
1930.500000	0.000974
1930.250000	0.000981
1930.000000	0.000987
1929.750000	0.000991
1929.500000	0.000992
1929.250000	0.000985
1929.000000	0.000967
1928.750000	0.000937
1928.500000	0.000896
1928.250000	0.000849
1928.000000	0.000801
1927.750000	0.000757
1927.500000	0.000723
1927.250000	0.000701
1927.000000	0.000692
1926.750000	0.000695
1926.500000	0.000708

1926.250000	0.000727
1926.000000	0.000752
1925.750000	0.000777
1925.500000	0.000801
1925.250000	0.000821
1925.000000	0.000832
1924.750000	0.000833
1924.500000	0.000823
1924.250000	0.000802
1924.000000	0.000776
1923.750000	0.000749
1923.500000	0.000728
1923.250000	0.000720
1923.000000	0.000729
1922.750000	0.000754
1922.500000	0.000794
1922.250000	0.000842
1922.000000	0.000890
1921.750000	0.000929
1921.500000	0.000955
1921.250000	0.000966
1921.000000	0.000962
1920.750000	0.000949
1920.500000	0.000931
1920.250000	0.000911
1920.000000	0.000892

1919.750000	0.000873
1919.500000	0.000854
1919.250000	0.000835
1919.000000	0.000818
1918.750000	0.000805
1918.500000	0.000798
1918.250000	0.000798
1918.000000	0.000807
1917.750000	0.000826
1917.500000	0.000852
1917.250000	0.000883
1917.000000	0.000912
1916.750000	0.000934
1916.500000	0.000946
1916.250000	0.000948
1916.000000	0.000946
1915.750000	0.000943
1915.500000	0.000942
1915.250000	0.000944
1915.000000	0.000948
1914.750000	0.000952
1914.500000	0.000956
1914.250000	0.000957
1914.000000	0.000954
1913.750000	0.000948
1913.500000	0.000939

1913.250000	0.000925
1913.000000	0.000909
1912.750000	0.000892
1912.500000	0.000874
1912.250000	0.000858
1912.000000	0.000842
1911.750000	0.000825
1911.500000	0.000805
1911.250000	0.000782
1911.000000	0.000757
1910.750000	0.000735
1910.500000	0.000719
1910.250000	0.000716
1910.000000	0.000728
1909.750000	0.000754
1909.500000	0.000791
1909.250000	0.000831
1909.000000	0.000867
1908.750000	0.000895
1908.500000	0.000914
1908.250000	0.000925
1908.000000	0.000932
1907.750000	0.000938
1907.500000	0.000948
1907.250000	0.000962
1907.000000	0.000980

1906.750000	0.001000
1906.500000	0.001017
1906.250000	0.001029
1906.000000	0.001031
1905.750000	0.001023
1905.500000	0.001003
1905.250000	0.000975
1905.000000	0.000941
1904.750000	0.000907
1904.500000	0.000880
1904.250000	0.000863
1904.000000	0.000858
1903.750000	0.000864
1903.500000	0.000879
1903.250000	0.000897
1903.000000	0.000915
1902.750000	0.000929
1902.500000	0.000939
1902.250000	0.000946
1902.000000	0.000948
1901.750000	0.000948
1901.500000	0.000943
1901.250000	0.000933
1901.000000	0.000919
1900.750000	0.000902
1900.500000	0.000885

1900.250000	0.000871
1900.000000	0.000864
1899.750000	0.000863
1899.500000	0.000868
1899.250000	0.000877
1899.000000	0.000885
1898.750000	0.000892
1898.500000	0.000897
1898.250000	0.000901
1898.000000	0.000909
1897.750000	0.000921
1897.500000	0.000938
1897.250000	0.000959
1897.000000	0.000981
1896.750000	0.001001
1896.500000	0.001016
1896.250000	0.001025
1896.000000	0.001029
1895.750000	0.001029
1895.500000	0.001025
1895.250000	0.001021
1895.000000	0.001018
1894.750000	0.001017
1894.500000	0.001019
1894.250000	0.001023
1894.000000	0.001029

1893.750000	0.001037
1893.500000	0.001044
1893.250000	0.001050
1893.000000	0.001054
1892.750000	0.001057
1892.500000	0.001059
1892.250000	0.001060
1892.000000	0.001057
1891.750000	0.001048
1891.500000	0.001030
1891.250000	0.001000
1891.000000	0.000957
1890.750000	0.000903
1890.500000	0.000845
1890.250000	0.000792
1890.000000	0.000753
1889.750000	0.000737
1889.500000	0.000747
1889.250000	0.000780
1889.000000	0.000827
1888.750000	0.000876
1888.500000	0.000918
1888.250000	0.000947
1888.000000	0.000961
1887.750000	0.000964
1887.500000	0.000962

1887.250000	0.000959
1887.000000	0.000958
1886.750000	0.000960
1886.500000	0.000963
1886.250000	0.000963
1886.000000	0.000959
1885.750000	0.000950
1885.500000	0.000939
1885.250000	0.000927
1885.000000	0.000918
1884.750000	0.000913
1884.500000	0.000913
1884.250000	0.000916
1884.000000	0.000922
1883.750000	0.000928
1883.500000	0.000932
1883.250000	0.000933
1883.000000	0.000929
1882.750000	0.000922
1882.500000	0.000914
1882.250000	0.000908
1882.000000	0.000906
1881.750000	0.000910
1881.500000	0.000922
1881.250000	0.000940
1881.000000	0.000963

1880.750000	0.000985
1880.500000	0.001003
1880.250000	0.001015
1880.000000	0.001019
1879.750000	0.001013
1879.500000	0.001000
1879.250000	0.000982
1879.000000	0.000960
1878.750000	0.000938
1878.500000	0.000918
1878.250000	0.000899
1878.000000	0.000883
1877.750000	0.000870
1877.500000	0.000861
1877.250000	0.000857
1877.000000	0.000860
1876.750000	0.000872
1876.500000	0.000894
1876.250000	0.000925
1876.000000	0.000963
1875.750000	0.001003
1875.500000	0.001038
1875.250000	0.001062
1875.000000	0.001071
1874.750000	0.001062
1874.500000	0.001038

1874.250000	0.001003
1874.000000	0.000965
1873.750000	0.000933
1873.500000	0.000913
1873.250000	0.000909
1873.000000	0.000921
1872.750000	0.000944
1872.500000	0.000971
1872.250000	0.000995
1872.000000	0.001007
1871.750000	0.001003
1871.500000	0.000979
1871.250000	0.000936
1871.000000	0.000877
1870.750000	0.000811
1870.500000	0.000749
1870.250000	0.000702
1870.000000	0.000682
1869.750000	0.000691
1869.500000	0.000726
1869.250000	0.000774
1869.000000	0.000819
1868.750000	0.000848
1868.500000	0.000858
1868.250000	0.000851
1868.000000	0.000836

1867.750000	0.000825
1867.500000	0.000824
1867.250000	0.000838
1867.000000	0.000867
1866.750000	0.000908
1866.500000	0.000955
1866.250000	0.001000
1866.000000	0.001040
1865.750000	0.001071
1865.500000	0.001090
1865.250000	0.001097
1865.000000	0.001090
1864.750000	0.001072
1864.500000	0.001043
1864.250000	0.001008
1864.000000	0.000971
1863.750000	0.000938
1863.500000	0.000916
1863.250000	0.000909
1863.000000	0.000915
1862.750000	0.000934
1862.500000	0.000960
1862.250000	0.000987
1862.000000	0.001010
1861.750000	0.001024
1861.500000	0.001028

1861.250000	0.001022
1861.000000	0.001010
1860.750000	0.000996
1860.500000	0.000983
1860.250000	0.000974
1860.000000	0.000968
1859.750000	0.000966
1859.500000	0.000966
1859.250000	0.000966
1859.000000	0.000966
1858.750000	0.000966
1858.500000	0.000968
1858.250000	0.000970
1858.000000	0.000974
1857.750000	0.000978
1857.500000	0.000981
1857.250000	0.000982
1857.000000	0.000981
1856.750000	0.000978
1856.500000	0.000974
1856.250000	0.000971
1856.000000	0.000969
1855.750000	0.000970
1855.500000	0.000971
1855.250000	0.000970
1855.000000	0.000966

1854.750000	0.000958
1854.500000	0.000946
1854.250000	0.000932
1854.000000	0.000919
1853.750000	0.000907
1853.500000	0.000900
1853.250000	0.000899
1853.000000	0.000902
1852.750000	0.000909
1852.500000	0.000916
1852.250000	0.000922
1852.000000	0.000923
1851.750000	0.000918
1851.500000	0.000906
1851.250000	0.000888
1851.000000	0.000866
1850.750000	0.000845
1850.500000	0.000829
1850.250000	0.000819
1850.000000	0.000819
1849.750000	0.000826
1849.500000	0.000838
1849.250000	0.000852
1849.000000	0.000863
1848.750000	0.000872
1848.500000	0.000875

1848.250000	0.000875
1848.000000	0.000871
1847.750000	0.000865
1847.500000	0.000860
1847.250000	0.000854
1847.000000	0.000846
1846.750000	0.000832
1846.500000	0.000804
1846.250000	0.000759
1846.000000	0.000692
1845.750000	0.000607
1845.500000	0.000514
1845.250000	0.000429
1845.000000	0.000373
1844.750000	0.000369
1844.500000	0.000429
1844.250000	0.000547
1844.000000	0.000702
1843.750000	0.000865
1843.500000	0.001006
1843.250000	0.001102
1843.000000	0.001147
1842.750000	0.001144
1842.500000	0.001107
1842.250000	0.001056
1842.000000	0.001006

1841.750000	0.000968
1841.500000	0.000943
1841.250000	0.000930
1841.000000	0.000924
1840.750000	0.000920
1840.500000	0.000916
1840.250000	0.000909
1840.000000	0.000900
1839.750000	0.000891
1839.500000	0.000881
1839.250000	0.000870
1839.000000	0.000857
1838.750000	0.000844
1838.500000	0.000831
1838.250000	0.000821
1838.000000	0.000814
1837.750000	0.000810
1837.500000	0.000808
1837.250000	0.000806
1837.000000	0.000804
1836.750000	0.000802
1836.500000	0.000801
1836.250000	0.000803
1836.000000	0.000808
1835.750000	0.000817
1835.500000	0.000829

1835.250000	0.000844
1835.000000	0.000859
1834.750000	0.000874
1834.500000	0.000887
1834.250000	0.000896
1834.000000	0.000900
1833.750000	0.000898
1833.500000	0.000891
1833.250000	0.000880
1833.000000	0.000869
1832.750000	0.000858
1832.500000	0.000848
1832.250000	0.000837
1832.000000	0.000821
1831.750000	0.000797
1831.500000	0.000762
1831.250000	0.000717
1831.000000	0.000666
1830.750000	0.000617
1830.500000	0.000578
1830.250000	0.000556
1830.000000	0.000554
1829.750000	0.000574
1829.500000	0.000612
1829.250000	0.000667
1829.000000	0.000730

1828.750000	0.000794
1828.500000	0.000851
1828.250000	0.000896
1828.000000	0.000927
1827.750000	0.000942
1827.500000	0.000942
1827.250000	0.000925
1827.000000	0.000888
1826.750000	0.000831
1826.500000	0.000761
1826.250000	0.000685
1826.000000	0.000617
1825.750000	0.000574
1825.500000	0.000567
1825.250000	0.000601
1825.000000	0.000670
1824.750000	0.000761
1824.500000	0.000858
1824.250000	0.000943
1824.000000	0.001004
1823.750000	0.001036
1823.500000	0.001040
1823.250000	0.001024
1823.000000	0.000997
1822.750000	0.000967
1822.500000	0.000940

1822.250000	0.000918
1822.000000	0.000903
1821.750000	0.000892
1821.500000	0.000885
1821.250000	0.000880
1821.000000	0.000876
1820.750000	0.000873
1820.500000	0.000871
1820.250000	0.000868
1820.000000	0.000863
1819.750000	0.000857
1819.500000	0.000848
1819.250000	0.000838
1819.000000	0.000828
1818.750000	0.000816
1818.500000	0.000806
1818.250000	0.000796
1818.000000	0.000786
1817.750000	0.000777
1817.500000	0.000768
1817.250000	0.000760
1817.000000	0.000754
1816.750000	0.000752
1816.500000	0.000758
1816.250000	0.000771
1816.000000	0.000793

1815.750000	0.000820
1815.500000	0.000850
1815.250000	0.000878
1815.000000	0.000902
1814.750000	0.000917
1814.500000	0.000922
1814.250000	0.000918
1814.000000	0.000908
1813.750000	0.000893
1813.500000	0.000878
1813.250000	0.000865
1813.000000	0.000857
1812.750000	0.000854
1812.500000	0.000855
1812.250000	0.000858
1812.000000	0.000860
1811.750000	0.000861
1811.500000	0.000857
1811.250000	0.000849
1811.000000	0.000838
1810.750000	0.000828
1810.500000	0.000824
1810.250000	0.000830
1810.000000	0.000846
1809.750000	0.000871
1809.500000	0.000898

1809.250000	0.000922
1809.000000	0.000938
1808.750000	0.000947
1808.500000	0.000949
1808.250000	0.000951
1808.000000	0.000954
1807.750000	0.000960
1807.500000	0.000969
1807.250000	0.000978
1807.000000	0.000984
1806.750000	0.000985
1806.500000	0.000980
1806.250000	0.000969
1806.000000	0.000957
1805.750000	0.000947
1805.500000	0.000943
1805.250000	0.000946
1805.000000	0.000958
1804.750000	0.000977
1804.500000	0.000997
1804.250000	0.001015
1804.000000	0.001027
1803.750000	0.001030
1803.500000	0.001024
1803.250000	0.001011
1803.000000	0.000995

1802.750000	0.000980
1802.500000	0.000970
1802.250000	0.000969
1802.000000	0.000976
1801.750000	0.000988
1801.500000	0.001000
1801.250000	0.001005
1801.000000	0.000999
1800.750000	0.000982
1800.500000	0.000960
1800.250000	0.000940
1800.000000	0.000930
1799.750000	0.000938
1799.500000	0.000965
1799.250000	0.001006
1799.000000	0.001055
1798.750000	0.001103
1798.500000	0.001142
1798.250000	0.001166
1798.000000	0.001173
1797.750000	0.001166
1797.500000	0.001149
1797.250000	0.001128
1797.000000	0.001109
1796.750000	0.001095
1796.500000	0.001087

1796.250000	0.001086
1796.000000	0.001091
1795.750000	0.001103
1795.500000	0.001119
1795.250000	0.001139
1795.000000	0.001154
1794.750000	0.001158
1794.500000	0.001144
1794.250000	0.001108
1794.000000	0.001053
1793.750000	0.000988
1793.500000	0.000927
1793.250000	0.000882
1793.000000	0.000868
1792.750000	0.000887
1792.500000	0.000937
1792.250000	0.001007
1792.000000	0.001083
1791.750000	0.001151
1791.500000	0.001205
1791.250000	0.001243
1791.000000	0.001274
1790.750000	0.001304
1790.500000	0.001339
1790.250000	0.001380
1790.000000	0.001419

1789.750000	0.001450
1789.500000	0.001467
1789.250000	0.001467
1789.000000	0.001453
1788.750000	0.001430
1788.500000	0.001405
1788.250000	0.001385
1788.000000	0.001373
1787.750000	0.001369
1787.500000	0.001371
1787.250000	0.001376
1787.000000	0.001380
1786.750000	0.001382
1786.500000	0.001380
1786.250000	0.001374
1786.000000	0.001367
1785.750000	0.001362
1785.500000	0.001364
1785.250000	0.001376
1785.000000	0.001400
1784.750000	0.001434
1784.500000	0.001475
1784.250000	0.001517
1784.000000	0.001554
1783.750000	0.001579
1783.500000	0.001592

1783.250000	0.001592
1783.000000	0.001581
1782.750000	0.001562
1782.500000	0.001534
1782.250000	0.001501
1782.000000	0.001464
1781.750000	0.001427
1781.500000	0.001394
1781.250000	0.001374
1781.000000	0.001373
1780.750000	0.001394
1780.500000	0.001438
1780.250000	0.001498
1780.000000	0.001566
1779.750000	0.001630
1779.500000	0.001683
1779.250000	0.001719
1779.000000	0.001737
1778.750000	0.001742
1778.500000	0.001739
1778.250000	0.001733
1778.000000	0.001729
1777.750000	0.001727
1777.500000	0.001727
1777.250000	0.001725
1777.000000	0.001721

1776.750000	0.001714
1776.500000	0.001707
1776.250000	0.001703
1776.000000	0.001707
1775.750000	0.001724
1775.500000	0.001753
1775.250000	0.001791
1775.000000	0.001829
1774.750000	0.001858
1774.500000	0.001870
1774.250000	0.001860
1774.000000	0.001831
1773.750000	0.001792
1773.500000	0.001754
1773.250000	0.001731
1773.000000	0.001735
1772.750000	0.001770
1772.500000	0.001833
1772.250000	0.001912
1772.000000	0.001992
1771.750000	0.002064
1771.500000	0.002127
1771.250000	0.002184
1771.000000	0.002241
1770.750000	0.002299
1770.500000	0.002355

1770.250000	0.002401
1770.000000	0.002430
1769.750000	0.002438
1769.500000	0.002427
1769.250000	0.002407
1769.000000	0.002393
1768.750000	0.002398
1768.500000	0.002433
1768.250000	0.002499
1768.000000	0.002589
1767.750000	0.002692
1767.500000	0.002794
1767.250000	0.002884
1767.000000	0.002954
1766.750000	0.003004
1766.500000	0.003037
1766.250000	0.003062
1766.000000	0.003086
1765.750000	0.003114
1765.500000	0.003148
1765.250000	0.003186
1765.000000	0.003226
1764.750000	0.003265
1764.500000	0.003301
1764.250000	0.003331
1764.000000	0.003353

1763.750000	0.003364
1763.500000	0.003363
1763.250000	0.003351
1763.000000	0.003334
1762.750000	0.003323
1762.500000	0.003333
1762.250000	0.003377
1762.000000	0.003462
1761.750000	0.003584
1761.500000	0.003730
1761.250000	0.003884
1761.000000	0.004028
1760.750000	0.004149
1760.500000	0.004240
1760.250000	0.004303
1760.000000	0.004344
1759.750000	0.004375
1759.500000	0.004402
1759.250000	0.004430
1759.000000	0.004458
1758.750000	0.004483
1758.500000	0.004504
1758.250000	0.004520
1758.000000	0.004537
1757.750000	0.004563
1757.500000	0.004607

1757.250000	0.004678
1757.000000	0.004778
1756.750000	0.004903
1756.500000	0.005043
1756.250000	0.005188
1756.000000	0.005328
1755.750000	0.005456
1755.500000	0.005569
1755.250000	0.005667
1755.000000	0.005754
1754.750000	0.005836
1754.500000	0.005919
1754.250000	0.006005
1754.000000	0.006092
1753.750000	0.006174
1753.500000	0.006247
1753.250000	0.006307
1753.000000	0.006354
1752.750000	0.006394
1752.500000	0.006439
1752.250000	0.006508
1752.000000	0.006620
1751.750000	0.006788
1751.500000	0.007004
1751.250000	0.007245
1751.000000	0.007478

1750.750000	0.007678
1750.500000	0.007831
1750.250000	0.007938
1750.000000	0.008010
1749.750000	0.008063
1749.500000	0.008116
1749.250000	0.008189
1749.000000	0.008295
1748.750000	0.008435
1748.500000	0.008604
1748.250000	0.008789
1748.000000	0.008981
1747.750000	0.009175
1747.500000	0.009367
1747.250000	0.009559
1747.000000	0.009751
1746.750000	0.009946
1746.500000	0.010143
1746.250000	0.010341
1746.000000	0.010537
1745.750000	0.010729
1745.500000	0.010916
1745.250000	0.011096
1745.000000	0.011272
1744.750000	0.011445
1744.500000	0.011622

1744.250000	0.011811
1744.000000	0.012023
1743.750000	0.012260
1743.500000	0.012523
1743.250000	0.012801
1743.000000	0.013084
1742.750000	0.013359
1742.500000	0.013618
1742.250000	0.013854
1742.000000	0.014065
1741.750000	0.014252
1741.500000	0.014421
1741.250000	0.014584
1741.000000	0.014761
1740.750000	0.014977
1740.500000	0.015256
1740.250000	0.015607
1740.000000	0.016016
1739.750000	0.016450
1739.500000	0.016873
1739.250000	0.017262
1739.000000	0.017611
1738.750000	0.017925
1738.500000	0.018211
1738.250000	0.018478
1738.000000	0.018735

1737.750000	0.018992
1737.500000	0.019249
1737.250000	0.019503
1737.000000	0.019747
1736.750000	0.019972
1736.500000	0.020168
1736.250000	0.020339
1736.000000	0.020501
1735.750000	0.020672
1735.500000	0.020867
1735.250000	0.021114
1735.000000	0.021450
1734.750000	0.021904
1734.500000	0.022452
1734.250000	0.023002
1734.000000	0.023448
1733.750000	0.023749
1733.500000	0.023955
1733.250000	0.024149
1733.000000	0.024370
1732.750000	0.024602
1732.500000	0.024819
1732.250000	0.025021
1732.000000	0.025226
1731.750000	0.025451
1731.500000	0.025704

1731.250000	0.025987
1731.000000	0.026308
1730.750000	0.026663
1730.500000	0.027032
1730.250000	0.027385
1730.000000	0.027699
1729.750000	0.027973
1729.500000	0.028223
1729.250000	0.028470
1729.000000	0.028730
1728.750000	0.029009
1728.500000	0.029304
1728.250000	0.029608
1728.000000	0.029914
1727.750000	0.030219
1727.500000	0.030520
1727.250000	0.030814
1727.000000	0.031099
1726.750000	0.031374
1726.500000	0.031641
1726.250000	0.031900
1726.000000	0.032153
1725.750000	0.032402
1725.500000	0.032649
1725.250000	0.032894
1725.000000	0.033137

1724.750000	0.033376
1724.500000	0.033610
1724.250000	0.033834
1724.000000	0.034042
1723.750000	0.034228
1723.500000	0.034390
1723.250000	0.034534
1723.000000	0.034667
1722.750000	0.034802
1722.500000	0.034945
1722.250000	0.035101
1722.000000	0.035268
1721.750000	0.035438
1721.500000	0.035600
1721.250000	0.035748
1721.000000	0.035883
1720.750000	0.036007
1720.500000	0.036121
1720.250000	0.036235
1720.000000	0.036365
1719.750000	0.036526
1719.500000	0.036722
1719.250000	0.036960
1719.000000	0.037250
1718.750000	0.037583
1718.500000	0.037891

1718.250000	0.038050
1718.000000	0.037955
1717.750000	0.037616
1717.500000	0.037178
1717.250000	0.036818
1717.000000	0.036627
1716.750000	0.036581
1716.500000	0.036610
1716.250000	0.036670
1716.000000	0.036758
1715.750000	0.036876
1715.500000	0.037002
1715.250000	0.037086
1715.000000	0.037082
1714.750000	0.036972
1714.500000	0.036779
1714.250000	0.036557
1714.000000	0.036362
1713.750000	0.036233
1713.500000	0.036174
1713.250000	0.036166
1713.000000	0.036186
1712.750000	0.036218
1712.500000	0.036249
1712.250000	0.036276
1712.000000	0.036299

1711.750000	0.036322
1711.500000	0.036345
1711.250000	0.036362
1711.000000	0.036360
1710.750000	0.036322
1710.500000	0.036237
1710.250000	0.036103
1710.000000	0.035936
1709.750000	0.035758
1709.500000	0.035594
1709.250000	0.035461
1709.000000	0.035363
1708.750000	0.035298
1708.500000	0.035259
1708.250000	0.035242
1708.000000	0.035243
1707.750000	0.035258
1707.500000	0.035281
1707.250000	0.035294
1707.000000	0.035266
1706.750000	0.035164
1706.500000	0.034978
1706.250000	0.034723
1706.000000	0.034441
1705.750000	0.034184
1705.500000	0.033991

1705.250000	0.033860
1705.000000	0.033758
1704.750000	0.033654
1704.500000	0.033539
1704.250000	0.033397
1704.000000	0.033215
1703.750000	0.033023
1703.500000	0.032890
1703.250000	0.032832
1703.000000	0.032798
1702.750000	0.032750
1702.500000	0.032724
1702.250000	0.032764
1702.000000	0.032838
1701.750000	0.032875
1701.500000	0.032924
1701.250000	0.033165
1701.000000	0.033659
1700.750000	0.034064
1700.500000	0.033812
1700.250000	0.032701
1700.000000	0.031205
1699.750000	0.030053
1699.500000	0.029590
1699.250000	0.029616
1699.000000	0.029749

1698.750000	0.029784
1698.500000	0.029750
1698.250000	0.029728
1698.000000	0.029726
1697.750000	0.029704
1697.500000	0.029662
1697.250000	0.029650
1697.000000	0.029701
1696.750000	0.029806
1696.500000	0.029885
1696.250000	0.029781
1696.000000	0.029332
1695.750000	0.028541
1695.500000	0.027637
1695.250000	0.026901
1695.000000	0.026446
1694.750000	0.026193
1694.500000	0.026009
1694.250000	0.025828
1694.000000	0.025653
1693.750000	0.025496
1693.500000	0.025343
1693.250000	0.025172
1693.000000	0.024977
1692.750000	0.024766
1692.500000	0.024538

1692.250000	0.024289
1692.000000	0.024024
1691.750000	0.023756
1691.500000	0.023491
1691.250000	0.023220
1691.000000	0.022938
1690.750000	0.022638
1690.500000	0.022319
1690.250000	0.021980
1690.000000	0.021627
1689.750000	0.021274
1689.500000	0.020930
1689.250000	0.020601
1689.000000	0.020280
1688.750000	0.019962
1688.500000	0.019643
1688.250000	0.019321
1688.000000	0.018996
1687.750000	0.018681
1687.500000	0.018395
1687.250000	0.018146
1687.000000	0.017922
1686.750000	0.017711
1686.500000	0.017515
1686.250000	0.017346
1686.000000	0.017202

1685.750000	0.017069
1685.500000	0.016953
1685.250000	0.016887
1685.000000	0.016878
1684.750000	0.016831
1684.500000	0.016581
1684.250000	0.016037
1684.000000	0.015297
1683.750000	0.014589
1683.500000	0.014102
1683.250000	0.013869
1683.000000	0.013798
1682.750000	0.013785
1682.500000	0.013781
1682.250000	0.013779
1682.000000	0.013773
1681.750000	0.013749
1681.500000	0.013704
1681.250000	0.013647
1681.000000	0.013583
1680.750000	0.013504
1680.500000	0.013397
1680.250000	0.013257
1680.000000	0.013092
1679.750000	0.012921
1679.500000	0.012762

1679.250000	0.012628
1679.000000	0.012519
1678.750000	0.012430
1678.500000	0.012353
1678.250000	0.012281
1678.000000	0.012204
1677.750000	0.012119
1677.500000	0.012026
1677.250000	0.011926
1677.000000	0.011819
1676.750000	0.011708
1676.500000	0.011602
1676.250000	0.011512
1676.000000	0.011436
1675.750000	0.011348
1675.500000	0.011215
1675.250000	0.011030
1675.000000	0.010833
1674.750000	0.010676
1674.500000	0.010583
1674.250000	0.010537
1674.000000	0.010499
1673.750000	0.010445
1673.500000	0.010372
1673.250000	0.010291
1673.000000	0.010208

1672.750000	0.010124
1672.500000	0.010037
1672.250000	0.009947
1672.000000	0.009849
1671.750000	0.009737
1671.500000	0.009613
1671.250000	0.009485
1671.000000	0.009362
1670.750000	0.009247
1670.500000	0.009144
1670.250000	0.009053
1670.000000	0.008975
1669.750000	0.008898
1669.500000	0.008806
1669.250000	0.008682
1669.000000	0.008532
1668.750000	0.008383
1668.500000	0.008263
1668.250000	0.008184
1668.000000	0.008134
1667.750000	0.008097
1667.500000	0.008060
1667.250000	0.008022
1667.000000	0.007984
1666.750000	0.007944
1666.500000	0.007899

1666.250000	0.007848
1666.000000	0.007793
1665.750000	0.007734
1665.500000	0.007674
1665.250000	0.007611
1665.000000	0.007545
1664.750000	0.007475
1664.500000	0.007399
1664.250000	0.007316
1664.000000	0.007232
1663.750000	0.007152
1663.500000	0.007079
1663.250000	0.007011
1663.000000	0.006941
1662.750000	0.006865
1662.500000	0.006791
1662.250000	0.006731
1662.000000	0.006691
1661.750000	0.006671
1661.500000	0.006658
1661.250000	0.006645
1661.000000	0.006627
1660.750000	0.006604
1660.500000	0.006580
1660.250000	0.006556
1660.000000	0.006531

1659.750000	0.006506
1659.500000	0.006482
1659.250000	0.006456
1659.000000	0.006429
1658.750000	0.006400
1658.500000	0.006368
1658.250000	0.006335
1658.000000	0.006302
1657.750000	0.006269
1657.500000	0.006238
1657.250000	0.006209
1657.000000	0.006182
1656.750000	0.006152
1656.500000	0.006112
1656.250000	0.006064
1656.000000	0.006011
1655.750000	0.005949
1655.500000	0.005870
1655.250000	0.005777
1655.000000	0.005684
1654.750000	0.005591
1654.500000	0.005484
1654.250000	0.005374
1654.000000	0.005309
1653.750000	0.005323
1653.500000	0.005378

1653.250000	0.005383
1653.000000	0.005300
1652.750000	0.005214
1652.500000	0.005272
1652.250000	0.005526
1652.000000	0.005863
1651.750000	0.006118
1651.500000	0.006219
1651.250000	0.006212
1651.000000	0.006175
1650.750000	0.006141
1650.500000	0.006111
1650.250000	0.006083
1650.000000	0.006067
1649.750000	0.006066
1649.500000	0.006067
1649.250000	0.006060
1649.000000	0.006049
1648.750000	0.006047
1648.500000	0.006055
1648.250000	0.006068
1648.000000	0.006082
1647.750000	0.006098
1647.500000	0.006122
1647.250000	0.006158
1647.000000	0.006211

1646.750000	0.006282
1646.500000	0.006361
1646.250000	0.006441
1646.000000	0.006522
1645.750000	0.006611
1645.500000	0.006712
1645.250000	0.006813
1645.000000	0.006900
1644.750000	0.006959
1644.500000	0.006990
1644.250000	0.007003
1644.000000	0.007010
1643.750000	0.007021
1643.500000	0.007039
1643.250000	0.007063
1643.000000	0.007092
1642.750000	0.007122
1642.500000	0.007150
1642.250000	0.007175
1642.000000	0.007197
1641.750000	0.007220
1641.500000	0.007245
1641.250000	0.007272
1641.000000	0.007301
1640.750000	0.007330
1640.500000	0.007360

1640.250000	0.007387
1640.000000	0.007412
1639.750000	0.007434
1639.500000	0.007453
1639.250000	0.007467
1639.000000	0.007476
1638.750000	0.007479
1638.500000	0.007480
1638.250000	0.007475
1638.000000	0.007457
1637.750000	0.007420
1637.500000	0.007370
1637.250000	0.007312
1637.000000	0.007248
1636.750000	0.007185
1636.500000	0.007139
1636.250000	0.007121
1636.000000	0.007115
1635.750000	0.007075
1635.500000	0.006964
1635.250000	0.006805
1635.000000	0.006673
1634.750000	0.006631
1634.500000	0.006675
1634.250000	0.006750
1634.000000	0.006803

1633.750000	0.006820
1633.500000	0.006815
1633.250000	0.006805
1633.000000	0.006789
1632.750000	0.006768
1632.500000	0.006740
1632.250000	0.006710
1632.000000	0.006677
1631.750000	0.006641
1631.500000	0.006603
1631.250000	0.006570
1631.000000	0.006541
1630.750000	0.006515
1630.500000	0.006487
1630.250000	0.006456
1630.000000	0.006418
1629.750000	0.006372
1629.500000	0.006316
1629.250000	0.006248
1629.000000	0.006172
1628.750000	0.006089
1628.500000	0.006002
1628.250000	0.005911
1628.000000	0.005820
1627.750000	0.005736
1627.500000	0.005666

1627.250000	0.005619
1627.000000	0.005595
1626.750000	0.005587
1626.500000	0.005586
1626.250000	0.005580
1626.000000	0.005563
1625.750000	0.005533
1625.500000	0.005489
1625.250000	0.005432
1625.000000	0.005364
1624.750000	0.005290
1624.500000	0.005217
1624.250000	0.005150
1624.000000	0.005088
1623.750000	0.005025
1623.500000	0.004954
1623.250000	0.004878
1623.000000	0.004812
1622.750000	0.004768
1622.500000	0.004750
1622.250000	0.004748
1622.000000	0.004751
1621.750000	0.004752
1621.500000	0.004751
1621.250000	0.004749
1621.000000	0.004746

1620.750000	0.004741
1620.500000	0.004733
1620.250000	0.004724
1620.000000	0.004714
1619.750000	0.004704
1619.500000	0.004696
1619.250000	0.004684
1619.000000	0.004664
1618.750000	0.004633
1618.500000	0.004590
1618.250000	0.004538
1618.000000	0.004484
1617.750000	0.004447
1617.500000	0.004446
1617.250000	0.004490
1617.000000	0.004567
1616.750000	0.004652
1616.500000	0.004729
1616.250000	0.004795
1616.000000	0.004855
1615.750000	0.004906
1615.500000	0.004937
1615.250000	0.004941
1615.000000	0.004927
1614.750000	0.004911
1614.500000	0.004909

1614.250000	0.004924
1614.000000	0.004953
1613.750000	0.004991
1613.500000	0.005036
1613.250000	0.005083
1613.000000	0.005131
1612.750000	0.005181
1612.500000	0.005232
1612.250000	0.005288
1612.000000	0.005346
1611.750000	0.005404
1611.500000	0.005462
1611.250000	0.005519
1611.000000	0.005575
1610.750000	0.005630
1610.500000	0.005685
1610.250000	0.005742
1610.000000	0.005800
1609.750000	0.005859
1609.500000	0.005921
1609.250000	0.005986
1609.000000	0.006054
1608.750000	0.006126
1608.500000	0.006205
1608.250000	0.006289
1608.000000	0.006380

1607.750000	0.006476
1607.500000	0.006579
1607.250000	0.006686
1607.000000	0.006797
1606.750000	0.006911
1606.500000	0.007028
1606.250000	0.007149
1606.000000	0.007274
1605.750000	0.007404
1605.500000	0.007537
1605.250000	0.007673
1605.000000	0.007809
1604.750000	0.007944
1604.500000	0.008078
1604.250000	0.008210
1604.000000	0.008339
1603.750000	0.008465
1603.500000	0.008585
1603.250000	0.008697
1603.000000	0.008797
1602.750000	0.008883
1602.500000	0.008954
1602.250000	0.009012
1602.000000	0.009057
1601.750000	0.009090
1601.500000	0.009109

1601.250000	0.009113
1601.000000	0.009099
1600.750000	0.009066
1600.500000	0.009014
1600.250000	0.008946
1600.000000	0.008863
1599.750000	0.008768
1599.500000	0.008661
1599.250000	0.008542
1599.000000	0.008412
1598.750000	0.008271
1598.500000	0.008120
1598.250000	0.007960
1598.000000	0.007794
1597.750000	0.007623
1597.500000	0.007449
1597.250000	0.007271
1597.000000	0.007091
1596.750000	0.006909
1596.500000	0.006728
1596.250000	0.006548
1596.000000	0.006374
1595.750000	0.006205
1595.500000	0.006041
1595.250000	0.005884
1595.000000	0.005730

1594.750000	0.005580
1594.500000	0.005436
1594.250000	0.005298
1594.000000	0.005170
1593.750000	0.005052
1593.500000	0.004944
1593.250000	0.004845
1593.000000	0.004753
1592.750000	0.004664
1592.500000	0.004580
1592.250000	0.004499
1592.000000	0.004424
1591.750000	0.004356
1591.500000	0.004295
1591.250000	0.004241
1591.000000	0.004193
1590.750000	0.004150
1590.500000	0.004110
1590.250000	0.004070
1590.000000	0.004032
1589.750000	0.003994
1589.500000	0.003959
1589.250000	0.003926
1589.000000	0.003898
1588.750000	0.003874
1588.500000	0.003855

1588.250000	0.003840
1588.000000	0.003827
1587.750000	0.003815
1587.500000	0.003803
1587.250000	0.003790
1587.000000	0.003777
1586.750000	0.003764
1586.500000	0.003752
1586.250000	0.003742
1586.000000	0.003736
1585.750000	0.003735
1585.500000	0.003739
1585.250000	0.003747
1585.000000	0.003759
1584.750000	0.003773
1584.500000	0.003787
1584.250000	0.003801
1584.000000	0.003814
1583.750000	0.003826
1583.500000	0.003837
1583.250000	0.003847
1583.000000	0.003857
1582.750000	0.003867
1582.500000	0.003877
1582.250000	0.003887
1582.000000	0.003897

1581.750000	0.003907
1581.500000	0.003918
1581.250000	0.003929
1581.000000	0.003939
1580.750000	0.003950
1580.500000	0.003960
1580.250000	0.003970
1580.000000	0.003980
1579.750000	0.003990
1579.500000	0.004000
1579.250000	0.004012
1579.000000	0.004022
1578.750000	0.004026
1578.500000	0.004019
1578.250000	0.003999
1578.000000	0.003967
1577.750000	0.003926
1577.500000	0.003885
1577.250000	0.003860
1577.000000	0.003870
1576.750000	0.003915
1576.500000	0.003976
1576.250000	0.004028
1576.000000	0.004062
1575.750000	0.004088
1575.500000	0.004116

1575.250000	0.004143
1575.000000	0.004151
1574.750000	0.004127
1574.500000	0.004080
1574.250000	0.004029
1574.000000	0.003990
1573.750000	0.003965
1573.500000	0.003953
1573.250000	0.003948
1573.000000	0.003946
1572.750000	0.003944
1572.500000	0.003939
1572.250000	0.003931
1572.000000	0.003917
1571.750000	0.003895
1571.500000	0.003864
1571.250000	0.003823
1571.000000	0.003772
1570.750000	0.003720
1570.500000	0.003678
1570.250000	0.003656
1570.000000	0.003658
1569.750000	0.003672
1569.500000	0.003687
1569.250000	0.003700
1569.000000	0.003722

1568.750000	0.003761
1568.500000	0.003817
1568.250000	0.003877
1568.000000	0.003928
1567.750000	0.003968
1567.500000	0.004000
1567.250000	0.004024
1567.000000	0.004041
1566.750000	0.004049
1566.500000	0.004049
1566.250000	0.004044
1566.000000	0.004037
1565.750000	0.004035
1565.500000	0.004042
1565.250000	0.004062
1565.000000	0.004092
1564.750000	0.004131
1564.500000	0.004176
1564.250000	0.004226
1564.000000	0.004279
1563.750000	0.004332
1563.500000	0.004381
1563.250000	0.004424
1563.000000	0.004458
1562.750000	0.004482
1562.500000	0.004492

1562.250000	0.004486
1562.000000	0.004465
1561.750000	0.004433
1561.500000	0.004396
1561.250000	0.004358
1561.000000	0.004324
1560.750000	0.004300
1560.500000	0.004294
1560.250000	0.004313
1560.000000	0.004370
1559.750000	0.004474
1559.500000	0.004624
1559.250000	0.004783
1559.000000	0.004890
1558.750000	0.004897
1558.500000	0.004814
1558.250000	0.004719
1558.000000	0.004710
1557.750000	0.004834
1557.500000	0.005062
1557.250000	0.005317
1557.000000	0.005534
1556.750000	0.005684
1556.500000	0.005773
1556.250000	0.005818
1556.000000	0.005838

1555.750000	0.005848
1555.500000	0.005869
1555.250000	0.005918
1555.000000	0.006007
1554.750000	0.006133
1554.500000	0.006279
1554.250000	0.006427
1554.000000	0.006562
1553.750000	0.006677
1553.500000	0.006776
1553.250000	0.006858
1553.000000	0.006925
1552.750000	0.006976
1552.500000	0.007018
1552.250000	0.007057
1552.000000	0.007099
1551.750000	0.007147
1551.500000	0.007199
1551.250000	0.007260
1551.000000	0.007335
1550.750000	0.007427
1550.500000	0.007532
1550.250000	0.007638
1550.000000	0.007728
1549.750000	0.007794
1549.500000	0.007845

1549.250000	0.007896
1549.000000	0.007961
1548.750000	0.008042
1548.500000	0.008133
1548.250000	0.008227
1548.000000	0.008319
1547.750000	0.008408
1547.500000	0.008490
1547.250000	0.008563
1547.000000	0.008629
1546.750000	0.008691
1546.500000	0.008755
1546.250000	0.008829
1546.000000	0.008920
1545.750000	0.009030
1545.500000	0.009149
1545.250000	0.009265
1545.000000	0.009365
1544.750000	0.009443
1544.500000	0.009506
1544.250000	0.009557
1544.000000	0.009602
1543.750000	0.009642
1543.500000	0.009686
1543.250000	0.009742
1543.000000	0.009817

1542.750000	0.009912
1542.500000	0.010023
1542.250000	0.010134
1542.000000	0.010224
1541.750000	0.010282
1541.500000	0.010318
1541.250000	0.010343
1541.000000	0.010362
1540.750000	0.010388
1540.500000	0.010445
1540.250000	0.010563
1540.000000	0.010734
1539.750000	0.010896
1539.500000	0.010975
1539.250000	0.010964
1539.000000	0.010938
1538.750000	0.010982
1538.500000	0.011115
1538.250000	0.011284
1538.000000	0.011432
1537.750000	0.011541
1537.500000	0.011626
1537.250000	0.011704
1537.000000	0.011783
1536.750000	0.011863
1536.500000	0.011947

1536.250000	0.012034
1536.000000	0.012117
1535.750000	0.012186
1535.500000	0.012238
1535.250000	0.012279
1535.000000	0.012318
1534.750000	0.012361
1534.500000	0.012419
1534.250000	0.012488
1534.000000	0.012545
1533.750000	0.012560
1533.500000	0.012523
1533.250000	0.012461
1533.000000	0.012419
1532.750000	0.012428
1532.500000	0.012487
1532.250000	0.012571
1532.000000	0.012656
1531.750000	0.012732
1531.500000	0.012797
1531.250000	0.012854
1531.000000	0.012904
1530.750000	0.012951
1530.500000	0.012998
1530.250000	0.013046
1530.000000	0.013095

1529.750000	0.013141
1529.500000	0.013185
1529.250000	0.013224
1529.000000	0.013258
1528.750000	0.013285
1528.500000	0.013302
1528.250000	0.013306
1528.000000	0.013296
1527.750000	0.013272
1527.500000	0.013240
1527.250000	0.013209
1527.000000	0.013186
1526.750000	0.013176
1526.500000	0.013186
1526.250000	0.013214
1526.000000	0.013252
1525.750000	0.013283
1525.500000	0.013289
1525.250000	0.013262
1525.000000	0.013213
1524.750000	0.013164
1524.500000	0.013128
1524.250000	0.013111
1524.000000	0.013107
1523.750000	0.013109
1523.500000	0.013114

1523.250000	0.013121
1523.000000	0.013129
1522.750000	0.013136
1522.500000	0.013144
1522.250000	0.013161
1522.000000	0.013193
1521.750000	0.013234
1521.500000	0.013250
1521.250000	0.013207
1521.000000	0.013093
1520.750000	0.012941
1520.500000	0.012808
1520.250000	0.012736
1520.000000	0.012728
1519.750000	0.012759
1519.500000	0.012800
1519.250000	0.012839
1519.000000	0.012876
1518.750000	0.012911
1518.500000	0.012948
1518.250000	0.012994
1518.000000	0.013062
1517.750000	0.013149
1517.500000	0.013236
1517.250000	0.013287
1517.000000	0.013283

1516.750000	0.013244
1516.500000	0.013213
1516.250000	0.013228
1516.000000	0.013298
1515.750000	0.013405
1515.500000	0.013527
1515.250000	0.013650
1515.000000	0.013770
1514.750000	0.013886
1514.500000	0.013997
1514.250000	0.014105
1514.000000	0.014213
1513.750000	0.014326
1513.500000	0.014447
1513.250000	0.014576
1513.000000	0.014712
1512.750000	0.014842
1512.500000	0.014951
1512.250000	0.015027
1512.000000	0.015071
1511.750000	0.015098
1511.500000	0.015122
1511.250000	0.015152
1511.000000	0.015189
1510.750000	0.015231
1510.500000	0.015272

1510.250000	0.015306
1510.000000	0.015327
1509.750000	0.015329
1509.500000	0.015307
1509.250000	0.015259
1509.000000	0.015189
1508.750000	0.015111
1508.500000	0.015035
1508.250000	0.014963
1508.000000	0.014891
1507.750000	0.014819
1507.500000	0.014739
1507.250000	0.014606
1507.000000	0.014333
1506.750000	0.013839
1506.500000	0.013137
1506.250000	0.012364
1506.000000	0.011707
1505.750000	0.011287
1505.500000	0.011091
1505.250000	0.011021
1505.000000	0.010984
1504.750000	0.010935
1504.500000	0.010864
1504.250000	0.010770
1504.000000	0.010654

1503.750000	0.010523
1503.500000	0.010385
1503.250000	0.010248
1503.000000	0.010106
1502.750000	0.009952
1502.500000	0.009784
1502.250000	0.009601
1502.000000	0.009406
1501.750000	0.009204
1501.500000	0.009003
1501.250000	0.008810
1501.000000	0.008632
1500.750000	0.008468
1500.500000	0.008314
1500.250000	0.008167
1500.000000	0.008024
1499.750000	0.007879
1499.500000	0.007728
1499.250000	0.007565
1499.000000	0.007388
1498.750000	0.007206
1498.500000	0.007037
1498.250000	0.006898
1498.000000	0.006794
1497.750000	0.006715
1497.500000	0.006656

1497.250000	0.006612
1497.000000	0.006583
1496.750000	0.006558
1496.500000	0.006517
1496.250000	0.006445
1496.000000	0.006346
1495.750000	0.006242
1495.500000	0.006156
1495.250000	0.006095
1495.000000	0.006048
1494.750000	0.006006
1494.500000	0.005966
1494.250000	0.005929
1494.000000	0.005899
1493.750000	0.005875
1493.500000	0.005856
1493.250000	0.005841
1493.000000	0.005829
1492.750000	0.005819
1492.500000	0.005809
1492.250000	0.005798
1492.000000	0.005786
1491.750000	0.005774
1491.500000	0.005765
1491.250000	0.005763
1491.000000	0.005773

1490.750000	0.005795
1490.500000	0.005823
1490.250000	0.005844
1490.000000	0.005851
1489.750000	0.005848
1489.500000	0.005845
1489.250000	0.005849
1489.000000	0.005860
1488.750000	0.005871
1488.500000	0.005875
1488.250000	0.005873
1488.000000	0.005869
1487.750000	0.005868
1487.500000	0.005872
1487.250000	0.005883
1487.000000	0.005901
1486.750000	0.005929
1486.500000	0.005963
1486.250000	0.006001
1486.000000	0.006038
1485.750000	0.006072
1485.500000	0.006101
1485.250000	0.006127
1485.000000	0.006149
1484.750000	0.006169
1484.500000	0.006186

1484.250000	0.006201
1484.000000	0.006214
1483.750000	0.006226
1483.500000	0.006239
1483.250000	0.006254
1483.000000	0.006272
1482.750000	0.006291
1482.500000	0.006309
1482.250000	0.006327
1482.000000	0.006343
1481.750000	0.006357
1481.500000	0.006371
1481.250000	0.006387
1481.000000	0.006404
1480.750000	0.006427
1480.500000	0.006454
1480.250000	0.006486
1480.000000	0.006520
1479.750000	0.006555
1479.500000	0.006589
1479.250000	0.006622
1479.000000	0.006653
1478.750000	0.006683
1478.500000	0.006710
1478.250000	0.006735
1478.000000	0.006757

1477.750000	0.006778
1477.500000	0.006799
1477.250000	0.006827
1477.000000	0.006862
1476.750000	0.006904
1476.500000	0.006943
1476.250000	0.006977
1476.000000	0.007006
1475.750000	0.007039
1475.500000	0.007082
1475.250000	0.007133
1475.000000	0.007188
1474.750000	0.007242
1474.500000	0.007298
1474.250000	0.007360
1474.000000	0.007432
1473.750000	0.007516
1473.500000	0.007607
1473.250000	0.007700
1473.000000	0.007788
1472.750000	0.007863
1472.500000	0.007921
1472.250000	0.007969
1472.000000	0.008023
1471.750000	0.008099
1471.500000	0.008203

1471.250000	0.008331
1471.000000	0.008468
1470.750000	0.008601
1470.500000	0.008726
1470.250000	0.008841
1470.000000	0.008951
1469.750000	0.009058
1469.500000	0.009163
1469.250000	0.009265
1469.000000	0.009363
1468.750000	0.009454
1468.500000	0.009537
1468.250000	0.009613
1468.000000	0.009680
1467.750000	0.009740
1467.500000	0.009790
1467.250000	0.009829
1467.000000	0.009859
1466.750000	0.009882
1466.500000	0.009902
1466.250000	0.009924
1466.000000	0.009954
1465.750000	0.009999
1465.500000	0.010055
1465.250000	0.010108
1465.000000	0.010145

1464.750000	0.010161
1464.500000	0.010171
1464.250000	0.010192
1464.000000	0.010236
1463.750000	0.010296
1463.500000	0.010363
1463.250000	0.010429
1463.000000	0.010491
1462.750000	0.010553
1462.500000	0.010613
1462.250000	0.010670
1462.000000	0.010723
1461.750000	0.010771
1461.500000	0.010812
1461.250000	0.010848
1461.000000	0.010879
1460.750000	0.010906
1460.500000	0.010930
1460.250000	0.010954
1460.000000	0.010978
1459.750000	0.011005
1459.500000	0.011032
1459.250000	0.011056
1459.000000	0.011075
1458.750000	0.011085
1458.500000	0.011087

1458.250000	0.011086
1458.000000	0.011097
1457.750000	0.011134
1457.500000	0.011202
1457.250000	0.011291
1457.000000	0.011379
1456.750000	0.011456
1456.500000	0.011522
1456.250000	0.011582
1456.000000	0.011633
1455.750000	0.011664
1455.500000	0.011668
1455.250000	0.011652
1455.000000	0.011630
1454.750000	0.011614
1454.500000	0.011609
1454.250000	0.011616
1454.000000	0.011632
1453.750000	0.011655
1453.500000	0.011682
1453.250000	0.011707
1453.000000	0.011729
1452.750000	0.011744
1452.500000	0.011749
1452.250000	0.011739
1452.000000	0.011715

1451.750000	0.011680
1451.500000	0.011641
1451.250000	0.011605
1451.000000	0.011574
1450.750000	0.011551
1450.500000	0.011531
1450.250000	0.011513
1450.000000	0.011496
1449.750000	0.011479
1449.500000	0.011462
1449.250000	0.011447
1449.000000	0.011434
1448.750000	0.011419
1448.500000	0.011393
1448.250000	0.011345
1448.000000	0.011269
1447.750000	0.011173
1447.500000	0.011075
1447.250000	0.010991
1447.000000	0.010932
1446.750000	0.010895
1446.500000	0.010872
1446.250000	0.010854
1446.000000	0.010837
1445.750000	0.010818
1445.500000	0.010797

1445.250000	0.010771
1445.000000	0.010743
1444.750000	0.010711
1444.500000	0.010678
1444.250000	0.010643
1444.000000	0.010608
1443.750000	0.010573
1443.500000	0.010539
1443.250000	0.010506
1443.000000	0.010473
1442.750000	0.010442
1442.500000	0.010410
1442.250000	0.010377
1442.000000	0.010342
1441.750000	0.010305
1441.500000	0.010266
1441.250000	0.010227
1441.000000	0.010188
1440.750000	0.010151
1440.500000	0.010114
1440.250000	0.010077
1440.000000	0.010040
1439.750000	0.010004
1439.500000	0.009967
1439.250000	0.009927
1439.000000	0.009884

1438.750000	0.009839
1438.500000	0.009793
1438.250000	0.009746
1438.000000	0.009704
1437.750000	0.009677
1437.500000	0.009673
1437.250000	0.009676
1437.000000	0.009649
1436.750000	0.009562
1436.500000	0.009420
1436.250000	0.009267
1436.000000	0.009148
1435.750000	0.009079
1435.500000	0.009044
1435.250000	0.009016
1435.000000	0.008981
1434.750000	0.008943
1434.500000	0.008908
1434.250000	0.008872
1434.000000	0.008829
1433.750000	0.008778
1433.500000	0.008717
1433.250000	0.008647
1433.000000	0.008571
1432.750000	0.008493
1432.500000	0.008418

1432.250000	0.008349
1432.000000	0.008287
1431.750000	0.008230
1431.500000	0.008176
1431.250000	0.008123
1431.000000	0.008069
1430.750000	0.008012
1430.500000	0.007948
1430.250000	0.007874
1430.000000	0.007791
1429.750000	0.007707
1429.500000	0.007632
1429.250000	0.007576
1429.000000	0.007539
1428.750000	0.007517
1428.500000	0.007504
1428.250000	0.007494
1428.000000	0.007484
1427.750000	0.007472
1427.500000	0.007460
1427.250000	0.007449
1427.000000	0.007438
1426.750000	0.007428
1426.500000	0.007419
1426.250000	0.007409
1426.000000	0.007399

1425.750000	0.007389
1425.500000	0.007382
1425.250000	0.007378
1425.000000	0.007381
1424.750000	0.007391
1424.500000	0.007406
1424.250000	0.007420
1424.000000	0.007429
1423.750000	0.007429
1423.500000	0.007425
1423.250000	0.007427
1423.000000	0.007439
1422.750000	0.007463
1422.500000	0.007496
1422.250000	0.007532
1422.000000	0.007565
1421.750000	0.007590
1421.500000	0.007605
1421.250000	0.007614
1421.000000	0.007619
1420.750000	0.007626
1420.500000	0.007643
1420.250000	0.007684
1420.000000	0.007751
1419.750000	0.007829
1419.500000	0.007890

1419.250000	0.007914
1419.000000	0.007904
1418.750000	0.007883
1418.500000	0.007873
1418.250000	0.007879
1418.000000	0.007894
1417.750000	0.007908
1417.500000	0.007918
1417.250000	0.007927
1417.000000	0.007937
1416.750000	0.007945
1416.500000	0.007952
1416.250000	0.007959
1416.000000	0.007965
1415.750000	0.007971
1415.500000	0.007976
1415.250000	0.007982
1415.000000	0.007989
1414.750000	0.007997
1414.500000	0.008008
1414.250000	0.008020
1414.000000	0.008034
1413.750000	0.008049
1413.500000	0.008064
1413.250000	0.008078
1413.000000	0.008091

1412.750000	0.008102
1412.500000	0.008114
1412.250000	0.008125
1412.000000	0.008136
1411.750000	0.008149
1411.500000	0.008162
1411.250000	0.008178
1411.000000	0.008197
1410.750000	0.008219
1410.500000	0.008245
1410.250000	0.008275
1410.000000	0.008307
1409.750000	0.008339
1409.500000	0.008368
1409.250000	0.008394
1409.000000	0.008418
1408.750000	0.008439
1408.500000	0.008460
1408.250000	0.008483
1408.000000	0.008508
1407.750000	0.008534
1407.500000	0.008560
1407.250000	0.008585
1407.000000	0.008608
1406.750000	0.008629
1406.500000	0.008649

1406.250000	0.008668
1406.000000	0.008688
1405.750000	0.008708
1405.500000	0.008725
1405.250000	0.008734
1405.000000	0.008733
1404.750000	0.008722
1404.500000	0.008711
1404.250000	0.008705
1404.000000	0.008709
1403.750000	0.008721
1403.500000	0.008739
1403.250000	0.008756
1403.000000	0.008771
1402.750000	0.008781
1402.500000	0.008786
1402.250000	0.008787
1402.000000	0.008783
1401.750000	0.008777
1401.500000	0.008769
1401.250000	0.008758
1401.000000	0.008746
1400.750000	0.008731
1400.500000	0.008717
1400.250000	0.008703
1400.000000	0.008692

1399.750000	0.008682
1399.500000	0.008668
1399.250000	0.008646
1399.000000	0.008615
1398.750000	0.008579
1398.500000	0.008548
1398.250000	0.008525
1398.000000	0.008513
1397.750000	0.008508
1397.500000	0.008506
1397.250000	0.008502
1397.000000	0.008497
1396.750000	0.008488
1396.500000	0.008478
1396.250000	0.008468
1396.000000	0.008460
1395.750000	0.008459
1395.500000	0.008465
1395.250000	0.008474
1395.000000	0.008482
1394.750000	0.008483
1394.500000	0.008478
1394.250000	0.008476
1394.000000	0.008483
1393.750000	0.008504
1393.500000	0.008535

1393.250000	0.008572
1393.000000	0.008610
1392.750000	0.008648
1392.500000	0.008687
1392.250000	0.008727
1392.000000	0.008768
1391.750000	0.008808
1391.500000	0.008846
1391.250000	0.008882
1391.000000	0.008914
1390.750000	0.008944
1390.500000	0.008973
1390.250000	0.009005
1390.000000	0.009038
1389.750000	0.009073
1389.500000	0.009108
1389.250000	0.009144
1389.000000	0.009181
1388.750000	0.009224
1388.500000	0.009274
1388.250000	0.009334
1388.000000	0.009404
1387.750000	0.009476
1387.500000	0.009541
1387.250000	0.009595
1387.000000	0.009637

1386.750000	0.009671
1386.500000	0.009705
1386.250000	0.009742
1386.000000	0.009781
1385.750000	0.009823
1385.500000	0.009867
1385.250000	0.009915
1385.000000	0.009966
1384.750000	0.010020
1384.500000	0.010078
1384.250000	0.010138
1384.000000	0.010199
1383.750000	0.010261
1383.500000	0.010319
1383.250000	0.010375
1383.000000	0.010426
1382.750000	0.010473
1382.500000	0.010517
1382.250000	0.010559
1382.000000	0.010600
1381.750000	0.010639
1381.500000	0.010675
1381.250000	0.010707
1381.000000	0.010733
1380.750000	0.010754
1380.500000	0.010771

1380.250000	0.010784
1380.000000	0.010794
1379.750000	0.010799
1379.500000	0.010800
1379.250000	0.010794
1379.000000	0.010781
1378.750000	0.010762
1378.500000	0.010736
1378.250000	0.010706
1378.000000	0.010673
1377.750000	0.010640
1377.500000	0.010606
1377.250000	0.010573
1377.000000	0.010539
1376.750000	0.010505
1376.500000	0.010468
1376.250000	0.010428
1376.000000	0.010382
1375.750000	0.010329
1375.500000	0.010268
1375.250000	0.010198
1375.000000	0.010121
1374.750000	0.010038
1374.500000	0.009950
1374.250000	0.009860
1374.000000	0.009769

1373.750000	0.009682
1373.500000	0.009603
1373.250000	0.009538
1373.000000	0.009487
1372.750000	0.009452
1372.500000	0.009429
1372.250000	0.009417
1372.000000	0.009409
1371.750000	0.009402
1371.500000	0.009393
1371.250000	0.009378
1371.000000	0.009357
1370.750000	0.009330
1370.500000	0.009300
1370.250000	0.009270
1370.000000	0.009242
1369.750000	0.009215
1369.500000	0.009191
1369.250000	0.009168
1369.000000	0.009144
1368.750000	0.009119
1368.500000	0.009093
1368.250000	0.009070
1368.000000	0.009052
1367.750000	0.009042
1367.500000	0.009044

1367.250000	0.009057
1367.000000	0.009084
1366.750000	0.009121
1366.500000	0.009165
1366.250000	0.009210
1366.000000	0.009250
1365.750000	0.009276
1365.500000	0.009284
1365.250000	0.009272
1365.000000	0.009241
1364.750000	0.009196
1364.500000	0.009144
1364.250000	0.009090
1364.000000	0.009039
1363.750000	0.008991
1363.500000	0.008944
1363.250000	0.008893
1363.000000	0.008835
1362.750000	0.008774
1362.500000	0.008716
1362.250000	0.008665
1362.000000	0.008622
1361.750000	0.008582
1361.500000	0.008539
1361.250000	0.008487
1361.000000	0.008426

1360.750000	0.008356
1360.500000	0.008283
1360.250000	0.008210
1360.000000	0.008142
1359.750000	0.008080
1359.500000	0.008023
1359.250000	0.007971
1359.000000	0.007922
1358.750000	0.007877
1358.500000	0.007835
1358.250000	0.007795
1358.000000	0.007759
1357.750000	0.007726
1357.500000	0.007697
1357.250000	0.007670
1357.000000	0.007646
1356.750000	0.007625
1356.500000	0.007607
1356.250000	0.007592
1356.000000	0.007580
1355.750000	0.007571
1355.500000	0.007561
1355.250000	0.007549
1355.000000	0.007534
1354.750000	0.007515
1354.500000	0.007493

1354.250000	0.007469
1354.000000	0.007447
1353.750000	0.007427
1353.500000	0.007411
1353.250000	0.007399
1353.000000	0.007390
1352.750000	0.007384
1352.500000	0.007380
1352.250000	0.007375
1352.000000	0.007370
1351.750000	0.007362
1351.500000	0.007350
1351.250000	0.007333
1351.000000	0.007310
1350.750000	0.007281
1350.500000	0.007248
1350.250000	0.007212
1350.000000	0.007175
1349.750000	0.007136
1349.500000	0.007098
1349.250000	0.007059
1349.000000	0.007021
1348.750000	0.006984
1348.500000	0.006949
1348.250000	0.006917
1348.000000	0.006888

1347.750000	0.006861
1347.500000	0.006835
1347.250000	0.006808
1347.000000	0.006780
1346.750000	0.006749
1346.500000	0.006717
1346.250000	0.006684
1346.000000	0.006651
1345.750000	0.006618
1345.500000	0.006586
1345.250000	0.006553
1345.000000	0.006521
1344.750000	0.006490
1344.500000	0.006460
1344.250000	0.006431
1344.000000	0.006403
1343.750000	0.006375
1343.500000	0.006346
1343.250000	0.006317
1343.000000	0.006287
1342.750000	0.006258
1342.500000	0.006232
1342.250000	0.006212
1342.000000	0.006197
1341.750000	0.006189
1341.500000	0.006185

1341.250000	0.006185
1341.000000	0.006186
1340.750000	0.006187
1340.500000	0.006184
1340.250000	0.006178
1340.000000	0.006168
1339.750000	0.006155
1339.500000	0.006140
1339.250000	0.006124
1339.000000	0.006107
1338.750000	0.006092
1338.500000	0.006080
1338.250000	0.006071
1338.000000	0.006067
1337.750000	0.006067
1337.500000	0.006070
1337.250000	0.006075
1337.000000	0.006081
1336.750000	0.006088
1336.500000	0.006094
1336.250000	0.006102
1336.000000	0.006112
1335.750000	0.006126
1335.500000	0.006142
1335.250000	0.006162
1335.000000	0.006183

1334.750000	0.006205
1334.500000	0.006227
1334.250000	0.006248
1334.000000	0.006268
1333.750000	0.006288
1333.500000	0.006308
1333.250000	0.006329
1333.000000	0.006353
1332.750000	0.006379
1332.500000	0.006408
1332.250000	0.006440
1332.000000	0.006474
1331.750000	0.006511
1331.500000	0.006549
1331.250000	0.006587
1331.000000	0.006626
1330.750000	0.006664
1330.500000	0.006702
1330.250000	0.006740
1330.000000	0.006779
1329.750000	0.006817
1329.500000	0.006855
1329.250000	0.006893
1329.000000	0.006930
1328.750000	0.006964
1328.500000	0.006997

1328.250000	0.007026
1328.000000	0.007055
1327.750000	0.007083
1327.500000	0.007111
1327.250000	0.007142
1327.000000	0.007174
1326.750000	0.007208
1326.500000	0.007243
1326.250000	0.007278
1326.000000	0.007313
1325.750000	0.007348
1325.500000	0.007382
1325.250000	0.007415
1325.000000	0.007450
1324.750000	0.007485
1324.500000	0.007522
1324.250000	0.007560
1324.000000	0.007599
1323.750000	0.007638
1323.500000	0.007675
1323.250000	0.007710
1323.000000	0.007742
1322.750000	0.007770
1322.500000	0.007794
1322.250000	0.007813
1322.000000	0.007829

1321.750000	0.007842
1321.500000	0.007852
1321.250000	0.007861
1321.000000	0.007868
1320.750000	0.007876
1320.500000	0.007885
1320.250000	0.007897
1320.000000	0.007909
1319.750000	0.007923
1319.500000	0.007936
1319.250000	0.007945
1319.000000	0.007951
1318.750000	0.007953
1318.500000	0.007950
1318.250000	0.007946
1318.000000	0.007940
1317.750000	0.007933
1317.500000	0.007925
1317.250000	0.007915
1317.000000	0.007903
1316.750000	0.007890
1316.500000	0.007878
1316.250000	0.007868
1316.000000	0.007862
1315.750000	0.007861
1315.500000	0.007865

1315.250000	0.007873
1315.000000	0.007881
1314.750000	0.007887
1314.500000	0.007891
1314.250000	0.007892
1314.000000	0.007891
1313.750000	0.007889
1313.500000	0.007888
1313.250000	0.007886
1313.000000	0.007885
1312.750000	0.007881
1312.500000	0.007875
1312.250000	0.007866
1312.000000	0.007854
1311.750000	0.007842
1311.500000	0.007830
1311.250000	0.007821
1311.000000	0.007814
1310.750000	0.007811
1310.500000	0.007809
1310.250000	0.007809
1310.000000	0.007811
1309.750000	0.007815
1309.500000	0.007819
1309.250000	0.007825
1309.000000	0.007831

1308.750000	0.007837
1308.500000	0.007844
1308.250000	0.007851
1308.000000	0.007859
1307.750000	0.007870
1307.500000	0.007883
1307.250000	0.007901
1307.000000	0.007923
1306.750000	0.007951
1306.500000	0.007984
1306.250000	0.008023
1306.000000	0.008067
1305.750000	0.008115
1305.500000	0.008167
1305.250000	0.008222
1305.000000	0.008277
1304.750000	0.008331
1304.500000	0.008382
1304.250000	0.008430
1304.000000	0.008476
1303.750000	0.008520
1303.500000	0.008565
1303.250000	0.008612
1303.000000	0.008661
1302.750000	0.008714
1302.500000	0.008770

1302.250000	0.008829
1302.000000	0.008892
1301.750000	0.008959
1301.500000	0.009029
1301.250000	0.009102
1301.000000	0.009177
1300.750000	0.009251
1300.500000	0.009322
1300.250000	0.009391
1300.000000	0.009455
1299.750000	0.009515
1299.500000	0.009571
1299.250000	0.009626
1299.000000	0.009680
1298.750000	0.009734
1298.500000	0.009787
1298.250000	0.009840
1298.000000	0.009891
1297.750000	0.009941
1297.500000	0.009987
1297.250000	0.010030
1297.000000	0.010068
1296.750000	0.010102
1296.500000	0.010133
1296.250000	0.010161
1296.000000	0.010185

1295.750000	0.010207
1295.500000	0.010226
1295.250000	0.010239
1295.000000	0.010246
1294.750000	0.010243
1294.500000	0.010232
1294.250000	0.010210
1294.000000	0.010179
1293.750000	0.010142
1293.500000	0.010098
1293.250000	0.010051
1293.000000	0.010002
1292.750000	0.009951
1292.500000	0.009900
1292.250000	0.009848
1292.000000	0.009795
1291.750000	0.009741
1291.500000	0.009686
1291.250000	0.009628
1291.000000	0.009568
1290.750000	0.009506
1290.500000	0.009442
1290.250000	0.009379
1290.000000	0.009317
1289.750000	0.009259
1289.500000	0.009205

1289.250000	0.009154
1289.000000	0.009107
1288.750000	0.009061
1288.500000	0.009016
1288.250000	0.008972
1288.000000	0.008927
1287.750000	0.008882
1287.500000	0.008836
1287.250000	0.008791
1287.000000	0.008747
1286.750000	0.008704
1286.500000	0.008663
1286.250000	0.008626
1286.000000	0.008593
1285.750000	0.008566
1285.500000	0.008544
1285.250000	0.008527
1285.000000	0.008514
1284.750000	0.008503
1284.500000	0.008493
1284.250000	0.008485
1284.000000	0.008477
1283.750000	0.008470
1283.500000	0.008466
1283.250000	0.008466
1283.000000	0.008470

1282.750000	0.008480
1282.500000	0.008496
1282.250000	0.008519
1282.000000	0.008548
1281.750000	0.008583
1281.500000	0.008623
1281.250000	0.008665
1281.000000	0.008709
1280.750000	0.008752
1280.500000	0.008795
1280.250000	0.008838
1280.000000	0.008880
1279.750000	0.008924
1279.500000	0.008971
1279.250000	0.009020
1279.000000	0.009073
1278.750000	0.009129
1278.500000	0.009188
1278.250000	0.009248
1278.000000	0.009309
1277.750000	0.009372
1277.500000	0.009436
1277.250000	0.009501
1277.000000	0.009568
1276.750000	0.009637
1276.500000	0.009708

1276.250000	0.009780
1276.000000	0.009853
1275.750000	0.009927
1275.500000	0.010002
1275.250000	0.010077
1275.000000	0.010150
1274.750000	0.010223
1274.500000	0.010295
1274.250000	0.010367
1274.000000	0.010440
1273.750000	0.010514
1273.500000	0.010590
1273.250000	0.010666
1273.000000	0.010742
1272.750000	0.010817
1272.500000	0.010888
1272.250000	0.010957
1272.000000	0.011023
1271.750000	0.011087
1271.500000	0.011150
1271.250000	0.011213
1271.000000	0.011276
1270.750000	0.011341
1270.500000	0.011406
1270.250000	0.011472
1270.000000	0.011539

1269.750000	0.011606
1269.500000	0.011673
1269.250000	0.011739
1269.000000	0.011803
1268.750000	0.011866
1268.500000	0.011926
1268.250000	0.011986
1268.000000	0.012046
1267.750000	0.012108
1267.500000	0.012172
1267.250000	0.012238
1267.000000	0.012305
1266.750000	0.012372
1266.500000	0.012438
1266.250000	0.012503
1266.000000	0.012568
1265.750000	0.012633
1265.500000	0.012700
1265.250000	0.012769
1265.000000	0.012841
1264.750000	0.012914
1264.500000	0.012988
1264.250000	0.013063
1264.000000	0.013138
1263.750000	0.013213
1263.500000	0.013288

1263.250000	0.013362
1263.000000	0.013437
1262.750000	0.013513
1262.500000	0.013591
1262.250000	0.013671
1262.000000	0.013755
1261.750000	0.013842
1261.500000	0.013933
1261.250000	0.014027
1261.000000	0.014122
1260.750000	0.014218
1260.500000	0.014311
1260.250000	0.014402
1260.000000	0.014491
1259.750000	0.014577
1259.500000	0.014660
1259.250000	0.014743
1259.000000	0.014825
1258.750000	0.014907
1258.500000	0.014989
1258.250000	0.015073
1258.000000	0.015160
1257.750000	0.015249
1257.500000	0.015340
1257.250000	0.015433
1257.000000	0.015526

1256.750000	0.015619
1256.500000	0.015709
1256.250000	0.015798
1256.000000	0.015885
1255.750000	0.015971
1255.500000	0.016056
1255.250000	0.016139
1255.000000	0.016223
1254.750000	0.016305
1254.500000	0.016389
1254.250000	0.016472
1254.000000	0.016557
1253.750000	0.016643
1253.500000	0.016729
1253.250000	0.016815
1253.000000	0.016897
1252.750000	0.016976
1252.500000	0.017050
1252.250000	0.017118
1252.000000	0.017183
1251.750000	0.017244
1251.500000	0.017301
1251.250000	0.017356
1251.000000	0.017409
1250.750000	0.017459
1250.500000	0.017509

1250.250000	0.017558
1250.000000	0.017607
1249.750000	0.017655
1249.500000	0.017703
1249.250000	0.017751
1249.000000	0.017798
1248.750000	0.017848
1248.500000	0.017904
1248.250000	0.017968
1248.000000	0.018042
1247.750000	0.018124
1247.500000	0.018208
1247.250000	0.018283
1247.000000	0.018339
1246.750000	0.018367
1246.500000	0.018363
1246.250000	0.018330
1246.000000	0.018277
1245.750000	0.018215
1245.500000	0.018158
1245.250000	0.018115
1245.000000	0.018090
1244.750000	0.018081
1244.500000	0.018085
1244.250000	0.018095
1244.000000	0.018105

1243.750000	0.018113
1243.500000	0.018116
1243.250000	0.018116
1243.000000	0.018113
1242.750000	0.018109
1242.500000	0.018106
1242.250000	0.018104
1242.000000	0.018106
1241.750000	0.018111
1241.500000	0.018122
1241.250000	0.018137
1241.000000	0.018155
1240.750000	0.018174
1240.500000	0.018191
1240.250000	0.018205
1240.000000	0.018214
1239.750000	0.018220
1239.500000	0.018222
1239.250000	0.018222
1239.000000	0.018222
1238.750000	0.018222
1238.500000	0.018221
1238.250000	0.018221
1238.000000	0.018221
1237.750000	0.018223
1237.500000	0.018228

1237.250000	0.018234
1237.000000	0.018243
1236.750000	0.018252
1236.500000	0.018258
1236.250000	0.018261
1236.000000	0.018258
1235.750000	0.018249
1235.500000	0.018235
1235.250000	0.018218
1235.000000	0.018200
1234.750000	0.018182
1234.500000	0.018164
1234.250000	0.018148
1234.000000	0.018133
1233.750000	0.018117
1233.500000	0.018100
1233.250000	0.018080
1233.000000	0.018054
1232.750000	0.018022
1232.500000	0.017984
1232.250000	0.017940
1232.000000	0.017892
1231.750000	0.017840
1231.500000	0.017786
1231.250000	0.017729
1231.000000	0.017670

1230.750000	0.017606
1230.500000	0.017539
1230.250000	0.017467
1230.000000	0.017393
1229.750000	0.017317
1229.500000	0.017242
1229.250000	0.017168
1229.000000	0.017093
1228.750000	0.017015
1228.500000	0.016932
1228.250000	0.016841
1228.000000	0.016743
1227.750000	0.016638
1227.500000	0.016528
1227.250000	0.016418
1227.000000	0.016307
1226.750000	0.016198
1226.500000	0.016090
1226.250000	0.015982
1226.000000	0.015873
1225.750000	0.015762
1225.500000	0.015650
1225.250000	0.015536
1225.000000	0.015423
1224.750000	0.015310
1224.500000	0.015200

1224.250000	0.015092
1224.000000	0.014986
1223.750000	0.014881
1223.500000	0.014777
1223.250000	0.014672
1223.000000	0.014567
1222.750000	0.014462
1222.500000	0.014358
1222.250000	0.014257
1222.000000	0.014161
1221.750000	0.014075
1221.500000	0.014002
1221.250000	0.013945
1221.000000	0.013906
1220.750000	0.013884
1220.500000	0.013877
1220.250000	0.013884
1220.000000	0.013899
1219.750000	0.013919
1219.500000	0.013942
1219.250000	0.013965
1219.000000	0.013986
1218.750000	0.014005
1218.500000	0.014018
1218.250000	0.014025
1218.000000	0.014022

1217.750000	0.014009
1217.500000	0.013982
1217.250000	0.013942
1217.000000	0.013889
1216.750000	0.013823
1216.500000	0.013748
1216.250000	0.013666
1216.000000	0.013580
1215.750000	0.013494
1215.500000	0.013407
1215.250000	0.013323
1215.000000	0.013241
1214.750000	0.013161
1214.500000	0.013086
1214.250000	0.013015
1214.000000	0.012949
1213.750000	0.012890
1213.500000	0.012839
1213.250000	0.012796
1213.000000	0.012759
1212.750000	0.012728
1212.500000	0.012700
1212.250000	0.012674
1212.000000	0.012649
1211.750000	0.012625
1211.500000	0.012601

1211.250000	0.012578
1211.000000	0.012558
1210.750000	0.012542
1210.500000	0.012530
1210.250000	0.012523
1210.000000	0.012519
1209.750000	0.012519
1209.500000	0.012521
1209.250000	0.012524
1209.000000	0.012528
1208.750000	0.012534
1208.500000	0.012542
1208.250000	0.012554
1208.000000	0.012572
1207.750000	0.012596
1207.500000	0.012627
1207.250000	0.012663
1207.000000	0.012701
1206.750000	0.012738
1206.500000	0.012772
1206.250000	0.012800
1206.000000	0.012822
1205.750000	0.012840
1205.500000	0.012855
1205.250000	0.012871
1205.000000	0.012889

1204.750000	0.012912
1204.500000	0.012940
1204.250000	0.012972
1204.000000	0.013009
1203.750000	0.013047
1203.500000	0.013086
1203.250000	0.013123
1203.000000	0.013157
1202.750000	0.013188
1202.500000	0.013215
1202.250000	0.013238
1202.000000	0.013259
1201.750000	0.013279
1201.500000	0.013301
1201.250000	0.013326
1201.000000	0.013356
1200.750000	0.013391
1200.500000	0.013431
1200.250000	0.013475
1200.000000	0.013521
1199.750000	0.013567
1199.500000	0.013611
1199.250000	0.013651
1199.000000	0.013687
1198.750000	0.013716
1198.500000	0.013740

1198.250000	0.013760
1198.000000	0.013778
1197.750000	0.013797
1197.500000	0.013820
1197.250000	0.013849
1197.000000	0.013886
1196.750000	0.013930
1196.500000	0.013979
1196.250000	0.014032
1196.000000	0.014085
1195.750000	0.014135
1195.500000	0.014181
1195.250000	0.014222
1195.000000	0.014260
1194.750000	0.014298
1194.500000	0.014337
1194.250000	0.014380
1194.000000	0.014428
1193.750000	0.014481
1193.500000	0.014536
1193.250000	0.014593
1193.000000	0.014650
1192.750000	0.014706
1192.500000	0.014761
1192.250000	0.014816
1192.000000	0.014873

1191.750000	0.014933
1191.500000	0.014998
1191.250000	0.015066
1191.000000	0.015139
1190.750000	0.015215
1190.500000	0.015293
1190.250000	0.015374
1190.000000	0.015456
1189.750000	0.015538
1189.500000	0.015621
1189.250000	0.015705
1189.000000	0.015787
1188.750000	0.015868
1188.500000	0.015947
1188.250000	0.016025
1188.000000	0.016102
1187.750000	0.016179
1187.500000	0.016256
1187.250000	0.016335
1187.000000	0.016416
1186.750000	0.016500
1186.500000	0.016586
1186.250000	0.016675
1186.000000	0.016766
1185.750000	0.016859
1185.500000	0.016952

1185.250000	0.017046
1185.000000	0.017138
1184.750000	0.017230
1184.500000	0.017321
1184.250000	0.017411
1184.000000	0.017502
1183.750000	0.017593
1183.500000	0.017682
1183.250000	0.017770
1183.000000	0.017853
1182.750000	0.017931
1182.500000	0.018005
1182.250000	0.018076
1182.000000	0.018146
1181.750000	0.018218
1181.500000	0.018292
1181.250000	0.018369
1181.000000	0.018449
1180.750000	0.018528
1180.500000	0.018606
1180.250000	0.018681
1180.000000	0.018752
1179.750000	0.018820
1179.500000	0.018887
1179.250000	0.018954
1179.000000	0.019022

1178.750000	0.019091
1178.500000	0.019161
1178.250000	0.019232
1178.000000	0.019302
1177.750000	0.019371
1177.500000	0.019439
1177.250000	0.019507
1177.000000	0.019575
1176.750000	0.019645
1176.500000	0.019719
1176.250000	0.019798
1176.000000	0.019884
1175.750000	0.019977
1175.500000	0.020075
1175.250000	0.020176
1175.000000	0.020277
1174.750000	0.020377
1174.500000	0.020473
1174.250000	0.020566
1174.000000	0.020658
1173.750000	0.020752
1173.500000	0.020848
1173.250000	0.020949
1173.000000	0.021054
1172.750000	0.021162
1172.500000	0.021271

1172.250000	0.021380
1172.000000	0.021489
1171.750000	0.021596
1171.500000	0.021702
1171.250000	0.021808
1171.000000	0.021914
1170.750000	0.022021
1170.500000	0.022128
1170.250000	0.022237
1170.000000	0.022348
1169.750000	0.022459
1169.500000	0.022570
1169.250000	0.022680
1169.000000	0.022788
1168.750000	0.022892
1168.500000	0.022992
1168.250000	0.023089
1168.000000	0.023182
1167.750000	0.023272
1167.500000	0.023358
1167.250000	0.023441
1167.000000	0.023520
1166.750000	0.023593
1166.500000	0.023662
1166.250000	0.023725
1166.000000	0.023784

1165.750000	0.023840
1165.500000	0.023890
1165.250000	0.023936
1165.000000	0.023975
1164.750000	0.024006
1164.500000	0.024030
1164.250000	0.024045
1164.000000	0.024054
1163.750000	0.024057
1163.500000	0.024055
1163.250000	0.024049
1163.000000	0.024041
1162.750000	0.024031
1162.500000	0.024021
1162.250000	0.024013
1162.000000	0.024011
1161.750000	0.024015
1161.500000	0.024025
1161.250000	0.024039
1161.000000	0.024054
1160.750000	0.024066
1160.500000	0.024074
1160.250000	0.024076
1160.000000	0.024075
1159.750000	0.024073
1159.500000	0.024074

1159.250000	0.024080
1159.000000	0.024092
1158.750000	0.024110
1158.500000	0.024131
1158.250000	0.024154
1158.000000	0.024175
1157.750000	0.024194
1157.500000	0.024211
1157.250000	0.024227
1157.000000	0.024242
1156.750000	0.024261
1156.500000	0.024284
1156.250000	0.024314
1156.000000	0.024350
1155.750000	0.024392
1155.500000	0.024439
1155.250000	0.024486
1155.000000	0.024532
1154.750000	0.024575
1154.500000	0.024613
1154.250000	0.024648
1154.000000	0.024682
1153.750000	0.024716
1153.500000	0.024753
1153.250000	0.024792
1153.000000	0.024835

1152.750000	0.024878
1152.500000	0.024921
1152.250000	0.024962
1152.000000	0.025001
1151.750000	0.025037
1151.500000	0.025070
1151.250000	0.025102
1151.000000	0.025133
1150.750000	0.025163
1150.500000	0.025191
1150.250000	0.025218
1150.000000	0.025244
1149.750000	0.025269
1149.500000	0.025293
1149.250000	0.025315
1149.000000	0.025335
1148.750000	0.025353
1148.500000	0.025369
1148.250000	0.025385
1148.000000	0.025400
1147.750000	0.025414
1147.500000	0.025426
1147.250000	0.025435
1147.000000	0.025441
1146.750000	0.025444
1146.500000	0.025444

1146.250000	0.025445
1146.000000	0.025447
1145.750000	0.025454
1145.500000	0.025466
1145.250000	0.025481
1145.000000	0.025497
1144.750000	0.025512
1144.500000	0.025524
1144.250000	0.025531
1144.000000	0.025534
1143.750000	0.025532
1143.500000	0.025527
1143.250000	0.025519
1143.000000	0.025508
1142.750000	0.025494
1142.500000	0.025479
1142.250000	0.025463
1142.000000	0.025449
1141.750000	0.025439
1141.500000	0.025433
1141.250000	0.025430
1141.000000	0.025430
1140.750000	0.025428
1140.500000	0.025423
1140.250000	0.025414
1140.000000	0.025399

1139.750000	0.025381
1139.500000	0.025360
1139.250000	0.025341
1139.000000	0.025324
1138.750000	0.025311
1138.500000	0.025303
1138.250000	0.025302
1138.000000	0.025304
1137.750000	0.025310
1137.500000	0.025317
1137.250000	0.025321
1137.000000	0.025319
1136.750000	0.025312
1136.500000	0.025299
1136.250000	0.025283
1136.000000	0.025264
1135.750000	0.025247
1135.500000	0.025233
1135.250000	0.025222
1135.000000	0.025214
1134.750000	0.025206
1134.500000	0.025200
1134.250000	0.025194
1134.000000	0.025189
1133.750000	0.025184
1133.500000	0.025181

1133.250000	0.025179
1133.000000	0.025176
1132.750000	0.025171
1132.500000	0.025166
1132.250000	0.025161
1132.000000	0.025159
1131.750000	0.025160
1131.500000	0.025167
1131.250000	0.025177
1131.000000	0.025190
1130.750000	0.025204
1130.500000	0.025214
1130.250000	0.025221
1130.000000	0.025223
1129.750000	0.025222
1129.500000	0.025218
1129.250000	0.025214
1129.000000	0.025210
1128.750000	0.025208
1128.500000	0.025210
1128.250000	0.025215
1128.000000	0.025223
1127.750000	0.025235
1127.500000	0.025249
1127.250000	0.025263
1127.000000	0.025277

1126.750000	0.025288
1126.500000	0.025298
1126.250000	0.025309
1126.000000	0.025322
1125.750000	0.025339
1125.500000	0.025359
1125.250000	0.025382
1125.000000	0.025409
1124.750000	0.025437
1124.500000	0.025468
1124.250000	0.025500
1124.000000	0.025534
1123.750000	0.025563
1123.500000	0.025581
1123.250000	0.025581
1123.000000	0.025555
1122.750000	0.025502
1122.500000	0.025427
1122.250000	0.025344
1122.000000	0.025271
1121.750000	0.025226
1121.500000	0.025222
1121.250000	0.025264
1121.000000	0.025345
1120.750000	0.025452
1120.500000	0.025568

1120.250000	0.025678
1120.000000	0.025771
1119.750000	0.025843
1119.500000	0.025896
1119.250000	0.025936
1119.000000	0.025969
1118.750000	0.025998
1118.500000	0.026026
1118.250000	0.026050
1118.000000	0.026071
1117.750000	0.026089
1117.500000	0.026104
1117.250000	0.026118
1117.000000	0.026132
1116.750000	0.026147
1116.500000	0.026163
1116.250000	0.026179
1116.000000	0.026195
1115.750000	0.026210
1115.500000	0.026226
1115.250000	0.026243
1115.000000	0.026263
1114.750000	0.026284
1114.500000	0.026306
1114.250000	0.026328
1114.000000	0.026347

1113.750000	0.026363
1113.500000	0.026376
1113.250000	0.026385
1113.000000	0.026391
1112.750000	0.026393
1112.500000	0.026394
1112.250000	0.026394
1112.000000	0.026393
1111.750000	0.026394
1111.500000	0.026397
1111.250000	0.026402
1111.000000	0.026410
1110.750000	0.026418
1110.500000	0.026427
1110.250000	0.026433
1110.000000	0.026437
1109.750000	0.026438
1109.500000	0.026437
1109.250000	0.026434
1109.000000	0.026428
1108.750000	0.026420
1108.500000	0.026412
1108.250000	0.026404
1108.000000	0.026397
1107.750000	0.026393
1107.500000	0.026392

1107.250000	0.026394
1107.000000	0.026397
1106.750000	0.026401
1106.500000	0.026402
1106.250000	0.026401
1106.000000	0.026396
1105.750000	0.026388
1105.500000	0.026379
1105.250000	0.026371
1105.000000	0.026363
1104.750000	0.026358
1104.500000	0.026353
1104.250000	0.026349
1104.000000	0.026346
1103.750000	0.026344
1103.500000	0.026341
1103.250000	0.026338
1103.000000	0.026334
1102.750000	0.026327
1102.500000	0.026318
1102.250000	0.026307
1102.000000	0.026294
1101.750000	0.026281
1101.500000	0.026272
1101.250000	0.026266
1101.000000	0.026266

1100.750000	0.026270
1100.500000	0.026277
1100.250000	0.026285
1100.000000	0.026292
1099.750000	0.026296
1099.500000	0.026298
1099.250000	0.026295
1099.000000	0.026290
1098.750000	0.026282
1098.500000	0.026272
1098.250000	0.026262
1098.000000	0.026252
1097.750000	0.026242
1097.500000	0.026232
1097.250000	0.026224
1097.000000	0.026215
1096.750000	0.026208
1096.500000	0.026201
1096.250000	0.026195
1096.000000	0.026192
1095.750000	0.026192
1095.500000	0.026194
1095.250000	0.026198
1095.000000	0.026203
1094.750000	0.026208
1094.500000	0.026213

1094.250000	0.026218
1094.000000	0.026223
1093.750000	0.026230
1093.500000	0.026238
1093.250000	0.026247
1093.000000	0.026256
1092.750000	0.026262
1092.500000	0.026266
1092.250000	0.026269
1092.000000	0.026272
1091.750000	0.026278
1091.500000	0.026291
1091.250000	0.026309
1091.000000	0.026332
1090.750000	0.026356
1090.500000	0.026376
1090.250000	0.026390
1090.000000	0.026396
1089.750000	0.026394
1089.500000	0.026386
1089.250000	0.026376
1089.000000	0.026368
1088.750000	0.026364
1088.500000	0.026368
1088.250000	0.026379
1088.000000	0.026398

1087.750000	0.026423
1087.500000	0.026453
1087.250000	0.026485
1087.000000	0.026518
1086.750000	0.026548
1086.500000	0.026574
1086.250000	0.026596
1086.000000	0.026615
1085.750000	0.026632
1085.500000	0.026649
1085.250000	0.026666
1085.000000	0.026686
1084.750000	0.026708
1084.500000	0.026732
1084.250000	0.026756
1084.000000	0.026780
1083.750000	0.026804
1083.500000	0.026826
1083.250000	0.026847
1083.000000	0.026865
1082.750000	0.026880
1082.500000	0.026892
1082.250000	0.026901
1082.000000	0.026909
1081.750000	0.026916
1081.500000	0.026924

1081.250000	0.026935
1081.000000	0.026948
1080.750000	0.026962
1080.500000	0.026979
1080.250000	0.026996
1080.000000	0.027013
1079.750000	0.027032
1079.500000	0.027051
1079.250000	0.027071
1079.000000	0.027089
1078.750000	0.027106
1078.500000	0.027119
1078.250000	0.027129
1078.000000	0.027135
1077.750000	0.027140
1077.500000	0.027145
1077.250000	0.027152
1077.000000	0.027162
1076.750000	0.027176
1076.500000	0.027193
1076.250000	0.027213
1076.000000	0.027235
1075.750000	0.027259
1075.500000	0.027284
1075.250000	0.027308
1075.000000	0.027331

1074.750000	0.027350
1074.500000	0.027365
1074.250000	0.027376
1074.000000	0.027383
1073.750000	0.027387
1073.500000	0.027388
1073.250000	0.027389
1073.000000	0.027389
1072.750000	0.027388
1072.500000	0.027388
1072.250000	0.027389
1072.000000	0.027393
1071.750000	0.027399
1071.500000	0.027409
1071.250000	0.027423
1071.000000	0.027440
1070.750000	0.027460
1070.500000	0.027479
1070.250000	0.027498
1070.000000	0.027515
1069.750000	0.027529
1069.500000	0.027541
1069.250000	0.027550
1069.000000	0.027555
1068.750000	0.027558
1068.500000	0.027558

1068.250000	0.027557
1068.000000	0.027557
1067.750000	0.027559
1067.500000	0.027565
1067.250000	0.027573
1067.000000	0.027583
1066.750000	0.027593
1066.500000	0.027601
1066.250000	0.027605
1066.000000	0.027605
1065.750000	0.027603
1065.500000	0.027599
1065.250000	0.027596
1065.000000	0.027595
1064.750000	0.027595
1064.500000	0.027597
1064.250000	0.027600
1064.000000	0.027603
1063.750000	0.027603
1063.500000	0.027602
1063.250000	0.027597
1063.000000	0.027590
1062.750000	0.027581
1062.500000	0.027571
1062.250000	0.027563
1062.000000	0.027556

1061.750000	0.027552
1061.500000	0.027551
1061.250000	0.027550
1061.000000	0.027549
1060.750000	0.027546
1060.500000	0.027539
1060.250000	0.027528
1060.000000	0.027515
1059.750000	0.027500
1059.500000	0.027486
1059.250000	0.027475
1059.000000	0.027466
1058.750000	0.027460
1058.500000	0.027458
1058.250000	0.027459
1058.000000	0.027462
1057.750000	0.027468
1057.500000	0.027477
1057.250000	0.027487
1057.000000	0.027499
1056.750000	0.027509
1056.500000	0.027518
1056.250000	0.027522
1056.000000	0.027521
1055.750000	0.027513
1055.500000	0.027498

1055.250000	0.027476
1055.000000	0.027449
1054.750000	0.027420
1054.500000	0.027393
1054.250000	0.027369
1054.000000	0.027352
1053.750000	0.027344
1053.500000	0.027343
1053.250000	0.027348
1053.000000	0.027355
1052.750000	0.027361
1052.500000	0.027364
1052.250000	0.027363
1052.000000	0.027358
1051.750000	0.027351
1051.500000	0.027342
1051.250000	0.027334
1051.000000	0.027325
1050.750000	0.027316
1050.500000	0.027305
1050.250000	0.027292
1050.000000	0.027277
1049.750000	0.027263
1049.500000	0.027250
1049.250000	0.027241
1049.000000	0.027234

1048.750000	0.027232
1048.500000	0.027231
1048.250000	0.027231
1048.000000	0.027230
1047.750000	0.027227
1047.500000	0.027221
1047.250000	0.027210
1047.000000	0.027196
1046.750000	0.027181
1046.500000	0.027166
1046.250000	0.027155
1046.000000	0.027148
1045.750000	0.027148
1045.500000	0.027153
1045.250000	0.027161
1045.000000	0.027169
1044.750000	0.027174
1044.500000	0.027175
1044.250000	0.027172
1044.000000	0.027167
1043.750000	0.027162
1043.500000	0.027160
1043.250000	0.027160
1043.000000	0.027163
1042.750000	0.027169
1042.500000	0.027174

1042.250000	0.027178
1042.000000	0.027181
1041.750000	0.027183
1041.500000	0.027186
1041.250000	0.027190
1041.000000	0.027195
1040.750000	0.027199
1040.500000	0.027203
1040.250000	0.027204
1040.000000	0.027201
1039.750000	0.027195
1039.500000	0.027185
1039.250000	0.027173
1039.000000	0.027161
1038.750000	0.027149
1038.500000	0.027138
1038.250000	0.027131
1038.000000	0.027126
1037.750000	0.027125
1037.500000	0.027128
1037.250000	0.027132
1037.000000	0.027136
1036.750000	0.027140
1036.500000	0.027141
1036.250000	0.027141
1036.000000	0.027139

1035.750000	0.027137
1035.500000	0.027136
1035.250000	0.027135
1035.000000	0.027136
1034.750000	0.027136
1034.500000	0.027135
1034.250000	0.027133
1034.000000	0.027128
1033.750000	0.027123
1033.500000	0.027117
1033.250000	0.027112
1033.000000	0.027109
1032.750000	0.027107
1032.500000	0.027106
1032.250000	0.027104
1032.000000	0.027101
1031.750000	0.027094
1031.500000	0.027082
1031.250000	0.027064
1031.000000	0.027040
1030.750000	0.027011
1030.500000	0.026981
1030.250000	0.026951
1030.000000	0.026927
1029.750000	0.026910
1029.500000	0.026902

1029.250000	0.026902
1029.000000	0.026908
1028.750000	0.026916
1028.500000	0.026922
1028.250000	0.026922
1028.000000	0.026915
1027.750000	0.026902
1027.500000	0.026883
1027.250000	0.026860
1027.000000	0.026836
1026.750000	0.026813
1026.500000	0.026791
1026.250000	0.026771
1026.000000	0.026755
1025.750000	0.026740
1025.500000	0.026728
1025.250000	0.026717
1025.000000	0.026705
1024.750000	0.026692
1024.500000	0.026676
1024.250000	0.026657
1024.000000	0.026634
1023.750000	0.026609
1023.500000	0.026582
1023.250000	0.026554
1023.000000	0.026527

1022.750000	0.026501
1022.500000	0.026478
1022.250000	0.026458
1022.000000	0.026442
1021.750000	0.026430
1021.500000	0.026420
1021.250000	0.026411
1021.000000	0.026401
1020.750000	0.026389
1020.500000	0.026376
1020.250000	0.026361
1020.000000	0.026348
1019.750000	0.026337
1019.500000	0.026329
1019.250000	0.026325
1019.000000	0.026323
1018.750000	0.026321
1018.500000	0.026317
1018.250000	0.026309
1018.000000	0.026297
1017.750000	0.026280
1017.500000	0.026260
1017.250000	0.026239
1017.000000	0.026219
1016.750000	0.026203
1016.500000	0.026191

1016.250000	0.026186
1016.000000	0.026188
1015.750000	0.026195
1015.500000	0.026205
1015.250000	0.026215
1015.000000	0.026221
1014.750000	0.026220
1014.500000	0.026208
1014.250000	0.026188
1014.000000	0.026162
1013.750000	0.026133
1013.500000	0.026106
1013.250000	0.026086
1013.000000	0.026073
1012.750000	0.026069
1012.500000	0.026072
1012.250000	0.026079
1012.000000	0.026086
1011.750000	0.026089
1011.500000	0.026086
1011.250000	0.026076
1011.000000	0.026059
1010.750000	0.026035
1010.500000	0.026009
1010.250000	0.025981
1010.000000	0.025954

1009.750000	0.025929
1009.500000	0.025907
1009.250000	0.025885
1009.000000	0.025862
1008.750000	0.025837
1008.500000	0.025808
1008.250000	0.025775
1008.000000	0.025740
1007.750000	0.025704
1007.500000	0.025667
1007.250000	0.025631
1007.000000	0.025597
1006.750000	0.025565
1006.500000	0.025538
1006.250000	0.025514
1006.000000	0.025495
1005.750000	0.025477
1005.500000	0.025459
1005.250000	0.025437
1005.000000	0.025410
1004.750000	0.025376
1004.500000	0.025336
1004.250000	0.025293
1004.000000	0.025252
1003.750000	0.025215
1003.500000	0.025184

1003.250000	0.025159
1003.000000	0.025136
1002.750000	0.025112
1002.500000	0.025082
1002.250000	0.025045
1002.000000	0.024999
1001.750000	0.024948
1001.500000	0.024892
1001.250000	0.024837
1001.000000	0.024784
1000.750000	0.024736
1000.500000	0.024694
1000.250000	0.024657
1000.000000	0.024627
999.750000	0.024600
999.500000	0.024575
999.250000	0.024549
999.000000	0.024517
998.750000	0.024478
998.500000	0.024430
998.250000	0.024376
998.000000	0.024321
997.750000	0.024271
997.500000	0.024231
997.250000	0.024205
997.000000	0.024193

996.750000	0.024192
996.500000	0.024197
996.250000	0.024200
996.000000	0.024196
995.750000	0.024181
995.500000	0.024156
995.250000	0.024120
995.000000	0.024078
994.750000	0.024032
994.500000	0.023987
994.250000	0.023946
994.000000	0.023912
993.750000	0.023885
993.500000	0.023866
993.250000	0.023852
993.000000	0.023841
992.750000	0.023830
992.500000	0.023813
992.250000	0.023791
992.000000	0.023761
991.750000	0.023727
991.500000	0.023691
991.250000	0.023656
991.000000	0.023626
990.750000	0.023604
990.500000	0.023590

990.250000	0.023583
990.000000	0.023582
989.750000	0.023585
989.500000	0.023590
989.250000	0.023593
989.000000	0.023593
988.750000	0.023588
988.500000	0.023577
988.250000	0.023560
988.000000	0.023540
987.750000	0.023520
987.500000	0.023503
987.250000	0.023491
987.000000	0.023487
986.750000	0.023491
986.500000	0.023500
986.250000	0.023511
986.000000	0.023522
985.750000	0.023528
985.500000	0.023527
985.250000	0.023519
985.000000	0.023504
984.750000	0.023483
984.500000	0.023457
984.250000	0.023428
984.000000	0.023397

983.750000	0.023367
983.500000	0.023337
983.250000	0.023310
983.000000	0.023284
982.750000	0.023259
982.500000	0.023234
982.250000	0.023207
982.000000	0.023178
981.750000	0.023144
981.500000	0.023106
981.250000	0.023065
981.000000	0.023022
980.750000	0.022981
980.500000	0.022943
980.250000	0.022909
980.000000	0.022880
979.750000	0.022854
979.500000	0.022829
979.250000	0.022803
979.000000	0.022774
978.750000	0.022740
978.500000	0.022703
978.250000	0.022662
978.000000	0.022620
977.750000	0.022578
977.500000	0.022538

977.250000	0.022500
977.000000	0.022465
976.750000	0.022433
976.500000	0.022403
976.250000	0.022375
976.000000	0.022347
975.750000	0.022319
975.500000	0.022289
975.250000	0.022254
975.000000	0.022215
974.750000	0.022170
974.500000	0.022121
974.250000	0.022071
974.000000	0.022024
973.750000	0.021983
973.500000	0.021950
973.250000	0.021926
973.000000	0.021910
972.750000	0.021899
972.500000	0.021889
972.250000	0.021877
972.000000	0.021861
971.750000	0.021839
971.500000	0.021811
971.250000	0.021779
971.000000	0.021744

970.750000	0.021708
970.500000	0.021674
970.250000	0.021643
970.000000	0.021616
969.750000	0.021594
969.500000	0.021575
969.250000	0.021559
969.000000	0.021543
968.750000	0.021526
968.500000	0.021506
968.250000	0.021483
968.000000	0.021457
967.750000	0.021428
967.500000	0.021399
967.250000	0.021370
967.000000	0.021343
966.750000	0.021319
966.500000	0.021297
966.250000	0.021277
966.000000	0.021257
965.750000	0.021237
965.500000	0.021214
965.250000	0.021187
965.000000	0.021158
964.750000	0.021126
964.500000	0.021095

964.250000	0.021067
964.000000	0.021043
963.750000	0.021026
963.500000	0.021014
963.250000	0.021007
963.000000	0.021000
962.750000	0.020991
962.500000	0.020978
962.250000	0.020959
962.000000	0.020934
961.750000	0.020903
961.500000	0.020869
961.250000	0.020832
961.000000	0.020795
960.750000	0.020760
960.500000	0.020730
960.250000	0.020705
960.000000	0.020686
959.750000	0.020672
959.500000	0.020661
959.250000	0.020651
959.000000	0.020638
958.750000	0.020620
958.500000	0.020597
958.250000	0.020570
958.000000	0.020539

957.750000	0.020507
957.500000	0.020477
957.250000	0.020449
957.000000	0.020423
956.750000	0.020397
956.500000	0.020372
956.250000	0.020346
956.000000	0.020320
955.750000	0.020294
955.500000	0.020269
955.250000	0.020244
955.000000	0.020220
954.750000	0.020198
954.500000	0.020176
954.250000	0.020156
954.000000	0.020137
953.750000	0.020121
953.500000	0.020106
953.250000	0.020092
953.000000	0.020077
952.750000	0.020060
952.500000	0.020040
952.250000	0.020017
952.000000	0.019992
951.750000	0.019966
951.500000	0.019941

951.250000	0.019916
951.000000	0.019892
950.750000	0.019868
950.500000	0.019844
950.250000	0.019820
950.000000	0.019797
949.750000	0.019776
949.500000	0.019758
949.250000	0.019743
949.000000	0.019730
948.750000	0.019717
948.500000	0.019705
948.250000	0.019691
948.000000	0.019678
947.750000	0.019664
947.500000	0.019651
947.250000	0.019639
947.000000	0.019627
946.750000	0.019613
946.500000	0.019596
946.250000	0.019576
946.000000	0.019552
945.750000	0.019525
945.500000	0.019498
945.250000	0.019471
945.000000	0.019448

944.750000	0.019428
944.500000	0.019412
944.250000	0.019398
944.000000	0.019387
943.750000	0.019377
943.500000	0.019367
943.250000	0.019358
943.000000	0.019347
942.750000	0.019333
942.500000	0.019314
942.250000	0.019289
942.000000	0.019259
941.750000	0.019223
941.500000	0.019182
941.250000	0.019140
941.000000	0.019099
940.750000	0.019061
940.500000	0.019029
940.250000	0.019002
940.000000	0.018980
939.750000	0.018962
939.500000	0.018946
939.250000	0.018927
939.000000	0.018905
938.750000	0.018878
938.500000	0.018845

938.250000	0.018807
938.000000	0.018768
937.750000	0.018730
937.500000	0.018694
937.250000	0.018662
937.000000	0.018633
936.750000	0.018607
936.500000	0.018581
936.250000	0.018553
936.000000	0.018522
935.750000	0.018488
935.500000	0.018449
935.250000	0.018408
935.000000	0.018365
934.750000	0.018321
934.500000	0.018278
934.250000	0.018238
934.000000	0.018202
933.750000	0.018168
933.500000	0.018136
933.250000	0.018103
933.000000	0.018068
932.750000	0.018030
932.500000	0.017987
932.250000	0.017941
932.000000	0.017894

931.750000	0.017848
931.500000	0.017806
931.250000	0.017767
931.000000	0.017731
930.750000	0.017698
930.500000	0.017665
930.250000	0.017633
930.000000	0.017602
929.750000	0.017571
929.500000	0.017543
929.250000	0.017516
929.000000	0.017490
928.750000	0.017464
928.500000	0.017436
928.250000	0.017406
928.000000	0.017374
927.750000	0.017340
927.500000	0.017305
927.250000	0.017270
927.000000	0.017235
926.750000	0.017200
926.500000	0.017164
926.250000	0.017128
926.000000	0.017093
925.750000	0.017061
925.500000	0.017032

925.250000	0.017008
925.000000	0.016990
924.750000	0.016977
924.500000	0.016968
924.250000	0.016958
924.000000	0.016947
923.750000	0.016931
923.500000	0.016908
923.250000	0.016876
923.000000	0.016836
922.750000	0.016788
922.500000	0.016734
922.250000	0.016676
922.000000	0.016619
921.750000	0.016565
921.500000	0.016516
921.250000	0.016475
921.000000	0.016439
920.750000	0.016407
920.500000	0.016377
920.250000	0.016346
920.000000	0.016313
919.750000	0.016276
919.500000	0.016237
919.250000	0.016194
919.000000	0.016150

918.750000	0.016106
918.500000	0.016062
918.250000	0.016019
918.000000	0.015979
917.750000	0.015942
917.500000	0.015906
917.250000	0.015870
917.000000	0.015831
916.750000	0.015785
916.500000	0.015733
916.250000	0.015674
916.000000	0.015609
915.750000	0.015543
915.500000	0.015478
915.250000	0.015417
915.000000	0.015361
914.750000	0.015309
914.500000	0.015258
914.250000	0.015206
914.000000	0.015151
913.750000	0.015092
913.500000	0.015031
913.250000	0.014969
913.000000	0.014909
912.750000	0.014853
912.500000	0.014800

912.250000	0.014751
912.000000	0.014702
911.750000	0.014653
911.500000	0.014600
911.250000	0.014543
911.000000	0.014480
910.750000	0.014413
910.500000	0.014342
910.250000	0.014269
910.000000	0.014196
909.750000	0.014124
909.500000	0.014056
909.250000	0.013993
909.000000	0.013934
908.750000	0.013878
908.500000	0.013823
908.250000	0.013768
908.000000	0.013708
907.750000	0.013645
907.500000	0.013578
907.250000	0.013509
907.000000	0.013440
906.750000	0.013375
906.500000	0.013316
906.250000	0.013262
906.000000	0.013214

905.750000	0.013168
905.500000	0.013124
905.250000	0.013078
905.000000	0.013029
904.750000	0.012975
904.500000	0.012918
904.250000	0.012857
904.000000	0.012795
903.750000	0.012734
903.500000	0.012674
903.250000	0.012617
903.000000	0.012563
902.750000	0.012512
902.500000	0.012465
902.250000	0.012421
902.000000	0.012378
901.750000	0.012334
901.500000	0.012287
901.250000	0.012236
901.000000	0.012181
900.750000	0.012120
900.500000	0.012058
900.250000	0.011996
900.000000	0.011936
899.750000	0.011882
899.500000	0.011833

899.250000	0.011791
899.000000	0.011754
898.750000	0.011718
898.500000	0.011683
898.250000	0.011646
898.000000	0.011606
897.750000	0.011561
897.500000	0.011513
897.250000	0.011461
897.000000	0.011407
896.750000	0.011353
896.500000	0.011300
896.250000	0.011249
896.000000	0.011202
895.750000	0.011157
895.500000	0.011115
895.250000	0.011073
895.000000	0.011030
894.750000	0.010985
894.500000	0.010937
894.250000	0.010888
894.000000	0.010839
893.750000	0.010790
893.500000	0.010744
893.250000	0.010700
893.000000	0.010658

892.750000	0.010617
892.500000	0.010574
892.250000	0.010531
892.000000	0.010487
891.750000	0.010443
891.500000	0.010400
891.250000	0.010361
891.000000	0.010325
890.750000	0.010294
890.500000	0.010267
890.250000	0.010241
890.000000	0.010217
889.750000	0.010193
889.500000	0.010167
889.250000	0.010139
889.000000	0.010108
888.750000	0.010076
888.500000	0.010043
888.250000	0.010008
888.000000	0.009971
887.750000	0.009932
887.500000	0.009890
887.250000	0.009845
887.000000	0.009798
886.750000	0.009750
886.500000	0.009705

886.250000	0.009664
886.000000	0.009629
885.750000	0.009598
885.500000	0.009570
885.250000	0.009543
885.000000	0.009513
884.750000	0.009479
884.500000	0.009442
884.250000	0.009405
884.000000	0.009371
883.750000	0.009342
883.500000	0.009319
883.250000	0.009301
883.000000	0.009284
882.750000	0.009266
882.500000	0.009243
882.250000	0.009214
882.000000	0.009180
881.750000	0.009143
881.500000	0.009103
881.250000	0.009064
881.000000	0.009024
880.750000	0.008983
880.500000	0.008940
880.250000	0.008896
880.000000	0.008850

879.750000	0.008805
879.500000	0.008764
879.250000	0.008728
879.000000	0.008697
878.750000	0.008671
878.500000	0.008647
878.250000	0.008624
878.000000	0.008599
877.750000	0.008573
877.500000	0.008546
877.250000	0.008519
877.000000	0.008493
876.750000	0.008467
876.500000	0.008439
876.250000	0.008407
876.000000	0.008368
875.750000	0.008323
875.500000	0.008271
875.250000	0.008216
875.000000	0.008162
874.750000	0.008114
874.500000	0.008074
874.250000	0.008043
874.000000	0.008022
873.750000	0.008007
873.500000	0.007995

873.250000	0.007982
873.000000	0.007963
872.750000	0.007937
872.500000	0.007903
872.250000	0.007864
872.000000	0.007820
871.750000	0.007777
871.500000	0.007735
871.250000	0.007699
871.000000	0.007668
870.750000	0.007645
870.500000	0.007627
870.250000	0.007613
870.000000	0.007602
869.750000	0.007590
869.500000	0.007576
869.250000	0.007556
869.000000	0.007531
868.750000	0.007499
868.500000	0.007463
868.250000	0.007427
868.000000	0.007391
867.750000	0.007360
867.500000	0.007334
867.250000	0.007311
867.000000	0.007292

866.750000	0.007273
866.500000	0.007253
866.250000	0.007232
866.000000	0.007210
865.750000	0.007190
865.500000	0.007172
865.250000	0.007158
865.000000	0.007148
864.750000	0.007139
864.500000	0.007128
864.250000	0.007114
864.000000	0.007095
863.750000	0.007071
863.500000	0.007045
863.250000	0.007018
863.000000	0.006992
862.750000	0.006971
862.500000	0.006952
862.250000	0.006937
862.000000	0.006924
861.750000	0.006912
861.500000	0.006900
861.250000	0.006888
861.000000	0.006875
860.750000	0.006861
860.500000	0.006847

860.250000	0.006832
860.000000	0.006816
859.750000	0.006799
859.500000	0.006780
859.250000	0.006759
859.000000	0.006738
858.750000	0.006718
858.500000	0.006698
858.250000	0.006681
858.000000	0.006667
857.750000	0.006655
857.500000	0.006646
857.250000	0.006639
857.000000	0.006634
856.750000	0.006630
856.500000	0.006626
856.250000	0.006621
856.000000	0.006615
855.750000	0.006606
855.500000	0.006594
855.250000	0.006578
855.000000	0.006558
854.750000	0.006533
854.500000	0.006507
854.250000	0.006480
854.000000	0.006455

853.750000	0.006434
853.500000	0.006419
853.250000	0.006408
853.000000	0.006402
852.750000	0.006399
852.500000	0.006397
852.250000	0.006397
852.000000	0.006397
851.750000	0.006399
851.500000	0.006401
851.250000	0.006404
851.000000	0.006407
850.750000	0.006409
850.500000	0.006408
850.250000	0.006404
850.000000	0.006398
849.750000	0.006390
849.500000	0.006382
849.250000	0.006374
849.000000	0.006368
848.750000	0.006362
848.500000	0.006357
848.250000	0.006354
848.000000	0.006353
847.750000	0.006355
847.500000	0.006362

847.250000	0.006374
847.000000	0.006390
846.750000	0.006410
846.500000	0.006432
846.250000	0.006456
846.000000	0.006480
845.750000	0.006503
845.500000	0.006525
845.250000	0.006547
845.000000	0.006568
844.750000	0.006589
844.500000	0.006610
844.250000	0.006633
844.000000	0.006656
843.750000	0.006682
843.500000	0.006709
843.250000	0.006738
843.000000	0.006766
842.750000	0.006793
842.500000	0.006818
842.250000	0.006841
842.000000	0.006862
841.750000	0.006882
841.500000	0.006904
841.250000	0.006928
841.000000	0.006954

840.750000	0.006981
840.500000	0.007008
840.250000	0.007033
840.000000	0.007056
839.750000	0.007075
839.500000	0.007091
839.250000	0.007104
839.000000	0.007115
838.750000	0.007123
838.500000	0.007128
838.250000	0.007132
838.000000	0.007135
837.750000	0.007138
837.500000	0.007145
837.250000	0.007156
837.000000	0.007170
836.750000	0.007188
836.500000	0.007207
836.250000	0.007224
836.000000	0.007237
835.750000	0.007243
835.500000	0.007243
835.250000	0.007237
835.000000	0.007227
834.750000	0.007216
834.500000	0.007206

834.250000	0.007199
834.000000	0.007194
833.750000	0.007192
833.500000	0.007192
833.250000	0.007193
833.000000	0.007194
832.750000	0.007195
832.500000	0.007194
832.250000	0.007192
832.000000	0.007189
831.750000	0.007184
831.500000	0.007177
831.250000	0.007167
831.000000	0.007155
830.750000	0.007139
830.500000	0.007119
830.250000	0.007094
830.000000	0.007066
829.750000	0.007034
829.500000	0.007001
829.250000	0.006969
829.000000	0.006939
828.750000	0.006911
828.500000	0.006885
828.250000	0.006861
828.000000	0.006837

827.750000	0.006811
827.500000	0.006781
827.250000	0.006745
827.000000	0.006702
826.750000	0.006652
826.500000	0.006596
826.250000	0.006537
826.000000	0.006478
825.750000	0.006421
825.500000	0.006371
825.250000	0.006328
825.000000	0.006294
824.750000	0.006267
824.500000	0.006245
824.250000	0.006227
824.000000	0.006210
823.750000	0.006193
823.500000	0.006176
823.250000	0.006161
823.000000	0.006147
822.750000	0.006138
822.500000	0.006134
822.250000	0.006137
822.000000	0.006147
821.750000	0.006164
821.500000	0.006186

821.250000	0.006211
821.000000	0.006238
820.750000	0.006262
820.500000	0.006283
820.250000	0.006298
820.000000	0.006310
819.750000	0.006318
819.500000	0.006325
819.250000	0.006335
819.000000	0.006349
818.750000	0.006368
818.500000	0.006393
818.250000	0.006423
818.000000	0.006458
817.750000	0.006498
817.500000	0.006542
817.250000	0.006590
817.000000	0.006642
816.750000	0.006697
816.500000	0.006753
816.250000	0.006808
816.000000	0.006859
815.750000	0.006903
815.500000	0.006938
815.250000	0.006962
815.000000	0.006977

814.750000	0.006982
814.500000	0.006982
814.250000	0.006977
814.000000	0.006973
813.750000	0.006969
813.500000	0.006968
813.250000	0.006971
813.000000	0.006975
812.750000	0.006978
812.500000	0.006979
812.250000	0.006975
812.000000	0.006965
811.750000	0.006947
811.500000	0.006922
811.250000	0.006891
811.000000	0.006857
810.750000	0.006822
810.500000	0.006786
810.250000	0.006751
810.000000	0.006716
809.750000	0.006680
809.500000	0.006643
809.250000	0.006603
809.000000	0.006560
808.750000	0.006515
808.500000	0.006469

808.250000	0.006424
808.000000	0.006381
807.750000	0.006342
807.500000	0.006308
807.250000	0.006277
807.000000	0.006248
806.750000	0.006220
806.500000	0.006192
806.250000	0.006161
806.000000	0.006128
805.750000	0.006093
805.500000	0.006055
805.250000	0.006017
805.000000	0.005978
804.750000	0.005940
804.500000	0.005903
804.250000	0.005868
804.000000	0.005838
803.750000	0.005812
803.500000	0.005792
803.250000	0.005778
803.000000	0.005768
802.750000	0.005762
802.500000	0.005757
802.250000	0.005750
802.000000	0.005737

801.750000	0.005717
801.500000	0.005690
801.250000	0.005656
801.000000	0.005617
800.750000	0.005577
800.500000	0.005540
800.250000	0.005509
800.000000	0.005485
799.750000	0.005468
799.500000	0.005456
799.250000	0.005446
799.000000	0.005434
798.750000	0.005417
798.500000	0.005395
798.250000	0.005367
798.000000	0.005338
797.750000	0.005308
797.500000	0.005282
797.250000	0.005260
797.000000	0.005241
796.750000	0.005225
796.500000	0.005209
796.250000	0.005191
796.000000	0.005170
795.750000	0.005145
795.500000	0.005118

795.250000	0.005090
795.000000	0.005062
794.750000	0.005036
794.500000	0.005011
794.250000	0.004989
794.000000	0.004969
793.750000	0.004950
793.500000	0.004931
793.250000	0.004911
793.000000	0.004888
792.750000	0.004864
792.500000	0.004837
792.250000	0.004808
792.000000	0.004779
791.750000	0.004753
791.500000	0.004731
791.250000	0.004714
791.000000	0.004702
790.750000	0.004695
790.500000	0.004690
790.250000	0.004687
790.000000	0.004683
789.750000	0.004678
789.500000	0.004672
789.250000	0.004666
789.000000	0.004660

788.750000	0.004653
788.500000	0.004646
788.250000	0.004637
788.000000	0.004627
787.750000	0.004616
787.500000	0.004606
787.250000	0.004598
787.000000	0.004593
786.750000	0.004592
786.500000	0.004594
786.250000	0.004599
786.000000	0.004606
785.750000	0.004613
785.500000	0.004621
785.250000	0.004632
785.000000	0.004648
784.750000	0.004670
784.500000	0.004700
784.250000	0.004739
784.000000	0.004784
783.750000	0.004835
783.500000	0.004887
783.250000	0.004939
783.000000	0.004987
782.750000	0.005031
782.500000	0.005070

782.250000	0.005107
782.000000	0.005144
781.750000	0.005182
781.500000	0.005225
781.250000	0.005272
781.000000	0.005326
780.750000	0.005384
780.500000	0.005446
780.250000	0.005509
780.000000	0.005571
779.750000	0.005630
779.500000	0.005684
779.250000	0.005733
779.000000	0.005775
778.750000	0.005811
778.500000	0.005842
778.250000	0.005870
778.000000	0.005896
777.750000	0.005923
777.500000	0.005951
777.250000	0.005981
777.000000	0.006012
776.750000	0.006040
776.500000	0.006065
776.250000	0.006085
776.000000	0.006097

775.750000	0.006103
775.500000	0.006104
775.250000	0.006102
775.000000	0.006097
774.750000	0.006091
774.500000	0.006085
774.250000	0.006076
774.000000	0.006065
773.750000	0.006051
773.500000	0.006032
773.250000	0.006010
773.000000	0.005984
772.750000	0.005954
772.500000	0.005919
772.250000	0.005879
772.000000	0.005831
771.750000	0.005776
771.500000	0.005714
771.250000	0.005646
771.000000	0.005574
770.750000	0.005500
770.500000	0.005428
770.250000	0.005359
770.000000	0.005294
769.750000	0.005235
769.500000	0.005180

769.250000	0.005130
769.000000	0.005085
768.750000	0.005044
768.500000	0.005007
768.250000	0.004976
768.000000	0.004949
767.750000	0.004927
767.500000	0.004910
767.250000	0.004897
767.000000	0.004888
766.750000	0.004880
766.500000	0.004874
766.250000	0.004869
766.000000	0.004863
765.750000	0.004858
765.500000	0.004854
765.250000	0.004850
765.000000	0.004848
764.750000	0.004849
764.500000	0.004852
764.250000	0.004857
764.000000	0.004864
763.750000	0.004872
763.500000	0.004878
763.250000	0.004884
763.000000	0.004889

762.750000	0.004892
762.500000	0.004895
762.250000	0.004895
762.000000	0.004892
761.750000	0.004885
761.500000	0.004871
761.250000	0.004849
761.000000	0.004821
760.750000	0.004786
760.500000	0.004747
760.250000	0.004706
760.000000	0.004664
759.750000	0.004622
759.500000	0.004578
759.250000	0.004534
759.000000	0.004487
758.750000	0.004438
758.500000	0.004389
758.250000	0.004342
758.000000	0.004299
757.750000	0.004262
757.500000	0.004231
757.250000	0.004206
757.000000	0.004183
756.750000	0.004162
756.500000	0.004141

756.250000	0.004120
756.000000	0.004098
755.750000	0.004078
755.500000	0.004060
755.250000	0.004044
755.000000	0.004030
754.750000	0.004019
754.500000	0.004008
754.250000	0.003998
754.000000	0.003987
753.750000	0.003977
753.500000	0.003968
753.250000	0.003960
753.000000	0.003955
752.750000	0.003954
752.500000	0.003957
752.250000	0.003963
752.000000	0.003972
751.750000	0.003983
751.500000	0.003994
751.250000	0.004003
751.000000	0.004008
750.750000	0.004011
750.500000	0.004010
750.250000	0.004008
750.000000	0.004007

749.750000	0.004008
749.500000	0.004014
749.250000	0.004023
749.000000	0.004037
748.750000	0.004052
748.500000	0.004068
748.250000	0.004081
748.000000	0.004089
747.750000	0.004093
747.500000	0.004090
747.250000	0.004081
747.000000	0.004067
746.750000	0.004049
746.500000	0.004029
746.250000	0.004008
746.000000	0.003986
745.750000	0.003964
745.500000	0.003942
745.250000	0.003916
745.000000	0.003886
744.750000	0.003850
744.500000	0.003808
744.250000	0.003760
744.000000	0.003710
743.750000	0.003659
743.500000	0.003611

743.250000	0.003565
743.000000	0.003523
742.750000	0.003485
742.500000	0.003450
742.250000	0.003419
742.000000	0.003392
741.750000	0.003371
741.500000	0.003356
741.250000	0.003345
741.000000	0.003335
740.750000	0.003324
740.500000	0.003308
740.250000	0.003285
740.000000	0.003256
739.750000	0.003223
739.500000	0.003190
739.250000	0.003162
739.000000	0.003141
738.750000	0.003130
738.500000	0.003127
738.250000	0.003131
738.000000	0.003138
737.750000	0.003148
737.500000	0.003157
737.250000	0.003164
737.000000	0.003170

736.750000	0.003173
736.500000	0.003173
736.250000	0.003170
736.000000	0.003163
735.750000	0.003154
735.500000	0.003142
735.250000	0.003131
735.000000	0.003121
734.750000	0.003113
734.500000	0.003109
734.250000	0.003109
734.000000	0.003110
733.750000	0.003111
733.500000	0.003112
733.250000	0.003112
733.000000	0.003110
732.750000	0.003106
732.500000	0.003101
732.250000	0.003096
732.000000	0.003090
731.750000	0.003084
731.500000	0.003079
731.250000	0.003074
731.000000	0.003069
730.750000	0.003064
730.500000	0.003058

730.250000	0.003053
730.000000	0.003049
729.750000	0.003047
729.500000	0.003049
729.250000	0.003053
729.000000	0.003060
728.750000	0.003067
728.500000	0.003073
728.250000	0.003075
728.000000	0.003072
727.750000	0.003064
727.500000	0.003051
727.250000	0.003037
727.000000	0.003022
726.750000	0.003009
726.500000	0.002999
726.250000	0.002991
726.000000	0.002985
725.750000	0.002979
725.500000	0.002972
725.250000	0.002965
725.000000	0.002956
724.750000	0.002948
724.500000	0.002940
724.250000	0.002934
724.000000	0.002929

723.750000	0.002924
723.500000	0.002918
723.250000	0.002911
723.000000	0.002901
722.750000	0.002887
722.500000	0.002869
722.250000	0.002848
722.000000	0.002826
721.750000	0.002804
721.500000	0.002784
721.250000	0.002765
721.000000	0.002749
720.750000	0.002735
720.500000	0.002723
720.250000	0.002712
720.000000	0.002700
719.750000	0.002685
719.500000	0.002667
719.250000	0.002646
719.000000	0.002622
718.750000	0.002596
718.500000	0.002570
718.250000	0.002547
718.000000	0.002528
717.750000	0.002514
717.500000	0.002507

717.250000	0.002503
717.000000	0.002502
716.750000	0.002499
716.500000	0.002493
716.250000	0.002482
716.000000	0.002467
715.750000	0.002449
715.500000	0.002432
715.250000	0.002419
715.000000	0.002412
714.750000	0.002411
714.500000	0.002417
714.250000	0.002427
714.000000	0.002439
713.750000	0.002449
713.500000	0.002456
713.250000	0.002457
713.000000	0.002452
712.750000	0.002441
712.500000	0.002427
712.250000	0.002412
712.000000	0.002399
711.750000	0.002388
711.500000	0.002381
711.250000	0.002377
711.000000	0.002375

710.750000	0.002372
710.500000	0.002366
710.250000	0.002355
710.000000	0.002340
709.750000	0.002322
709.500000	0.002305
709.250000	0.002291
709.000000	0.002284
708.750000	0.002284
708.500000	0.002292
708.250000	0.002307
708.000000	0.002325
707.750000	0.002343
707.500000	0.002358
707.250000	0.002369
707.000000	0.002373
706.750000	0.002369
706.500000	0.002359
706.250000	0.002342
706.000000	0.002322
705.750000	0.002301
705.500000	0.002283
705.250000	0.002270
705.000000	0.002264
704.750000	0.002266
704.500000	0.002274

704.250000	0.002286
704.000000	0.002299
703.750000	0.002310
703.500000	0.002316
703.250000	0.002316
703.000000	0.002313
702.750000	0.002307
702.500000	0.002301
702.250000	0.002299
702.000000	0.002301
701.750000	0.002307
701.500000	0.002316
701.250000	0.002326
701.000000	0.002334
700.750000	0.002340
700.500000	0.002342
700.250000	0.002341
700.000000	0.002339
699.750000	0.002337
699.500000	0.002337
699.250000	0.002339
699.000000	0.002342
698.750000	0.002347
698.500000	0.002352
698.250000	0.002355
698.000000	0.002356

697.750000	0.002355
697.500000	0.002353
697.250000	0.002348
697.000000	0.002343
696.750000	0.002336
696.500000	0.002329
696.250000	0.002322
696.000000	0.002315
695.750000	0.002310
695.500000	0.002308
695.250000	0.002309
695.000000	0.002312
694.750000	0.002317
694.500000	0.002321
694.250000	0.002323
694.000000	0.002321
693.750000	0.002316
693.500000	0.002306
693.250000	0.002295
693.000000	0.002283
692.750000	0.002271
692.500000	0.002261
692.250000	0.002252
692.000000	0.002246
691.750000	0.002241
691.500000	0.002237

691.250000	0.002234
691.000000	0.002231
690.750000	0.002227
690.500000	0.002220
690.250000	0.002210
690.000000	0.002196
689.750000	0.002178
689.500000	0.002159
689.250000	0.002140
689.000000	0.002125
688.750000	0.002116
688.500000	0.002113
688.250000	0.002115
688.000000	0.002120
687.750000	0.002125
687.500000	0.002126
687.250000	0.002122
687.000000	0.002114
686.750000	0.002101
686.500000	0.002087
686.250000	0.002074
686.000000	0.002064
685.750000	0.002058
685.500000	0.002054
685.250000	0.002051
685.000000	0.002047

684.750000	0.002041
684.500000	0.002032
684.250000	0.002022
684.000000	0.002012
683.750000	0.002003
683.500000	0.001998
683.250000	0.001994
683.000000	0.001991
682.750000	0.001989
682.500000	0.001985
682.250000	0.001979
682.000000	0.001973
681.750000	0.001968
681.500000	0.001965
681.250000	0.001964
681.000000	0.001966
680.750000	0.001968
680.500000	0.001968
680.250000	0.001967
680.000000	0.001963
679.750000	0.001958
679.500000	0.001951
679.250000	0.001943
679.000000	0.001935
678.750000	0.001927
678.500000	0.001920

678.250000	0.001912
678.000000	0.001905
677.750000	0.001900
677.500000	0.001898
677.250000	0.001899
677.000000	0.001904
676.750000	0.001912
676.500000	0.001922
676.250000	0.001934
676.000000	0.001946
675.750000	0.001958
675.500000	0.001970
675.250000	0.001980
675.000000	0.001990
674.750000	0.001997
674.500000	0.002001
674.250000	0.002001
674.000000	0.001997
673.750000	0.001990
673.500000	0.001982
673.250000	0.001975
673.000000	0.001972
672.750000	0.001972
672.500000	0.001974
672.250000	0.001978
672.000000	0.001982

671.750000	0.001982
671.500000	0.001978
671.250000	0.001970
671.000000	0.001959
670.750000	0.001945
670.500000	0.001930
670.250000	0.001918
670.000000	0.001911
669.750000	0.001912
669.500000	0.001921
669.250000	0.001941
669.000000	0.001976
668.750000	0.002013
668.500000	0.002025
668.250000	0.001985
668.000000	0.001896
667.750000	0.001796
667.500000	0.001733
667.250000	0.001725
667.000000	0.001753
666.750000	0.001787
666.500000	0.001815
666.250000	0.001839
666.000000	0.001864
665.750000	0.001886
665.500000	0.001901

665.250000	0.001909
665.000000	0.001914
664.750000	0.001917
664.500000	0.001920
664.250000	0.001924
664.000000	0.001932
663.750000	0.001941
663.500000	0.001951
663.250000	0.001958
663.000000	0.001961
662.750000	0.001959
662.500000	0.001953
662.250000	0.001942
662.000000	0.001929
661.750000	0.001915
661.500000	0.001903
661.250000	0.001895
661.000000	0.001891
660.750000	0.001893
660.500000	0.001899
660.250000	0.001906
660.000000	0.001912
659.750000	0.001913
659.500000	0.001909
659.250000	0.001900
659.000000	0.001887

658.750000	0.001872
658.500000	0.001857
658.250000	0.001842
658.000000	0.001829
657.750000	0.001817
657.500000	0.001808
657.250000	0.001801
657.000000	0.001798
656.750000	0.001798
656.500000	0.001800
656.250000	0.001804
656.000000	0.001808
655.750000	0.001810
655.500000	0.001809
655.250000	0.001806
655.000000	0.001804
654.750000	0.001803
654.500000	0.001806
654.250000	0.001814
654.000000	0.001826
653.750000	0.001840
653.500000	0.001854
653.250000	0.001864
653.000000	0.001870
652.750000	0.001869
652.500000	0.001859

652.250000	0.001842
652.000000	0.001816
651.750000	0.001782
651.500000	0.001742
651.250000	0.001700
651.000000	0.001660
650.750000	0.001624
650.500000	0.001596
650.250000	0.001579
650.000000	0.001572
649.750000	0.001575
649.500000	0.001584
649.250000	0.001597
649.000000	0.001609
648.750000	0.001619
648.500000	0.001623
648.250000	0.001621
648.000000	0.001611
647.750000	0.001595
647.500000	0.001573
647.250000	0.001547
647.000000	0.001519
646.750000	0.001492
646.500000	0.001468
646.250000	0.001448
646.000000	0.001434

645.750000	0.001425
645.500000	0.001419
645.250000	0.001415
645.000000	0.001410
644.750000	0.001404
644.500000	0.001396
644.250000	0.001386
644.000000	0.001375
643.750000	0.001364
643.500000	0.001354
643.250000	0.001344
643.000000	0.001334
642.750000	0.001323
642.500000	0.001310
642.250000	0.001294
642.000000	0.001277
641.750000	0.001260
641.500000	0.001243
641.250000	0.001228
641.000000	0.001218
640.750000	0.001212
640.500000	0.001213
640.250000	0.001219
640.000000	0.001233
639.750000	0.001254
639.500000	0.001281

639.250000	0.001311
639.000000	0.001342
638.750000	0.001369
638.500000	0.001385
638.250000	0.001389
638.000000	0.001378
637.750000	0.001353
637.500000	0.001318
637.250000	0.001279
637.000000	0.001242
636.750000	0.001212
636.500000	0.001192
636.250000	0.001184
636.000000	0.001186
635.750000	0.001196
635.500000	0.001211
635.250000	0.001227
635.000000	0.001242
634.750000	0.001252
634.500000	0.001258
634.250000	0.001257
634.000000	0.001249
633.750000	0.001236
633.500000	0.001220
633.250000	0.001201
633.000000	0.001184

632.750000	0.001171
632.500000	0.001164
632.250000	0.001162
632.000000	0.001165
631.750000	0.001168
631.500000	0.001168
631.250000	0.001163
631.000000	0.001151
630.750000	0.001130
630.500000	0.001102
630.250000	0.001070
630.000000	0.001039
629.750000	0.001012
629.500000	0.000994
629.250000	0.000987
629.000000	0.000994
628.750000	0.001011
628.500000	0.001036
628.250000	0.001063
628.000000	0.001085
627.750000	0.001099
627.500000	0.001101
627.250000	0.001092
627.000000	0.001073
626.750000	0.001050
626.500000	0.001025

626.250000	0.001004
626.000000	0.000989
625.750000	0.000981
625.500000	0.000980
625.250000	0.000983
625.000000	0.000990
624.750000	0.000997
624.500000	0.001005
624.250000	0.001011
624.000000	0.001017
623.750000	0.001024
623.500000	0.001033
623.250000	0.001044
623.000000	0.001058
622.750000	0.001074
622.500000	0.001091
622.250000	0.001105
622.000000	0.001114
621.750000	0.001117
621.500000	0.001112
621.250000	0.001101
621.000000	0.001088
620.750000	0.001076
620.500000	0.001068
620.250000	0.001070
620.000000	0.001081

619.750000	0.001101
619.500000	0.001129
619.250000	0.001160
619.000000	0.001190
618.750000	0.001218
618.500000	0.001239
618.250000	0.001251
618.000000	0.001253
617.750000	0.001246
617.500000	0.001231
617.250000	0.001211
617.000000	0.001188
616.750000	0.001167
616.500000	0.001150
616.250000	0.001139
616.000000	0.001134
615.750000	0.001135
615.500000	0.001140
615.250000	0.001148
615.000000	0.001156
614.750000	0.001165
614.500000	0.001173
614.250000	0.001180
614.000000	0.001185
613.750000	0.001190
613.500000	0.001192

613.250000	0.001192
613.000000	0.001190
612.750000	0.001186
612.500000	0.001180
612.250000	0.001173
612.000000	0.001165
611.750000	0.001159
611.500000	0.001152
611.250000	0.001147
611.000000	0.001140
610.750000	0.001132
610.500000	0.001121
610.250000	0.001105
610.000000	0.001086
609.750000	0.001063
609.500000	0.001040
609.250000	0.001018
609.000000	0.001000
608.750000	0.000989
608.500000	0.000985
608.250000	0.000989
608.000000	0.000998
607.750000	0.001012
607.500000	0.001029
607.250000	0.001047
607.000000	0.001067

606.750000	0.001088
606.500000	0.001111
606.250000	0.001133
606.000000	0.001153
605.750000	0.001168
605.500000	0.001174
605.250000	0.001169
605.000000	0.001153
604.750000	0.001127
604.500000	0.001096
604.250000	0.001063
604.000000	0.001033
603.750000	0.001011
603.500000	0.000997
603.250000	0.000994
603.000000	0.001002
602.750000	0.001017
602.500000	0.001039
602.250000	0.001063
602.000000	0.001087
601.750000	0.001109
601.500000	0.001125
601.250000	0.001138
601.000000	0.001147
600.750000	0.001155
600.500000	0.001164

600.250000	0.001174
600.000000	0.001184
599.750000	0.001192
599.500000	0.001194
599.250000	0.001188
599.000000	0.001173
598.750000	0.001151
598.500000	0.001126
598.250000	0.001106
598.000000	0.001095
597.750000	0.001096
597.500000	0.001110
597.250000	0.001132
597.000000	0.001160
596.750000	0.001186
596.500000	0.001208
596.250000	0.001222
596.000000	0.001229
595.750000	0.001229
595.500000	0.001224
595.250000	0.001214
595.000000	0.001201
594.750000	0.001186
594.500000	0.001168
594.250000	0.001152
594.000000	0.001139

593.750000	0.001133
593.500000	0.001137
593.250000	0.001152
593.000000	0.001177
592.750000	0.001210
592.500000	0.001246
592.250000	0.001279
592.000000	0.001300
591.750000	0.001307
591.500000	0.001297
591.250000	0.001274
591.000000	0.001243
590.750000	0.001210
590.500000	0.001181
590.250000	0.001160
590.000000	0.001148
589.750000	0.001146
589.500000	0.001150
589.250000	0.001158
589.000000	0.001166
588.750000	0.001172
588.500000	0.001175
588.250000	0.001173
588.000000	0.001169
587.750000	0.001164
587.500000	0.001158

587.250000	0.001152
587.000000	0.001145
586.750000	0.001136
586.500000	0.001124
586.250000	0.001109
586.000000	0.001091
585.750000	0.001073
585.500000	0.001055
585.250000	0.001042
585.000000	0.001035
584.750000	0.001035
584.500000	0.001042
584.250000	0.001056
584.000000	0.001075
583.750000	0.001096
583.500000	0.001118
583.250000	0.001139
583.000000	0.001155
582.750000	0.001164
582.500000	0.001165
582.250000	0.001157
582.000000	0.001140
581.750000	0.001117
581.500000	0.001091
581.250000	0.001064
581.000000	0.001039

580.750000	0.001020
580.500000	0.001008
580.250000	0.001002
580.000000	0.001003
579.750000	0.001008
579.500000	0.001017
579.250000	0.001029
579.000000	0.001043
578.750000	0.001058
578.500000	0.001072
578.250000	0.001083
578.000000	0.001090
577.750000	0.001092
577.500000	0.001087
577.250000	0.001077
577.000000	0.001062
576.750000	0.001044
576.500000	0.001027
576.250000	0.001013
576.000000	0.001006
575.750000	0.001008
575.500000	0.001020
575.250000	0.001039
575.000000	0.001063
574.750000	0.001087
574.500000	0.001108

574.250000	0.001120
574.000000	0.001121
573.750000	0.001111
573.500000	0.001092
573.250000	0.001067
573.000000	0.001043
572.750000	0.001023
572.500000	0.001011
572.250000	0.001010
572.000000	0.001017
571.750000	0.001031
571.500000	0.001047
571.250000	0.001061
571.000000	0.001069
570.750000	0.001069
570.500000	0.001060
570.250000	0.001041
570.000000	0.001015
569.750000	0.000987
569.500000	0.000960
569.250000	0.000940
569.000000	0.000932
568.750000	0.000937
568.500000	0.000954
568.250000	0.000979
568.000000	0.001006

567.750000	0.001028
567.500000	0.001041
567.250000	0.001041
567.000000	0.001031
566.750000	0.001013
566.500000	0.000992
566.250000	0.000973
566.000000	0.000959
565.750000	0.000951
565.500000	0.000950
565.250000	0.000955
565.000000	0.000964
564.750000	0.000977
564.500000	0.000992
564.250000	0.001007
564.000000	0.001019
563.750000	0.001026
563.500000	0.001026
563.250000	0.001016
563.000000	0.000999
562.750000	0.000976
562.500000	0.000953
562.250000	0.000934
562.000000	0.000921
561.750000	0.000916
561.500000	0.000920

561.250000	0.000931
561.000000	0.000949
560.750000	0.000972
560.500000	0.000999
560.250000	0.001029
560.000000	0.001059
559.750000	0.001085
559.500000	0.001104
559.250000	0.001113
559.000000	0.001108
558.750000	0.001090
558.500000	0.001062
558.250000	0.001030
558.000000	0.001001
557.750000	0.000980
557.500000	0.000972
557.250000	0.000979
557.000000	0.000999
556.750000	0.001028
556.500000	0.001061
556.250000	0.001093
556.000000	0.001116
555.750000	0.001128
555.500000	0.001126
555.250000	0.001111
555.000000	0.001085

554.750000	0.001053
554.500000	0.001021
554.250000	0.000996
554.000000	0.000979
553.750000	0.000974
553.500000	0.000977
553.250000	0.000986
553.000000	0.000997
552.750000	0.001008
552.500000	0.001017
552.250000	0.001024
552.000000	0.001030
551.750000	0.001036
551.500000	0.001040
551.250000	0.001041
551.000000	0.001038
550.750000	0.001029
550.500000	0.001013
550.250000	0.000992
550.000000	0.000968
549.750000	0.000946
549.500000	0.000926
549.250000	0.000910
549.000000	0.000898
548.750000	0.000889
548.500000	0.000882

548.250000	0.000877
548.000000	0.000873
547.750000	0.000873
547.500000	0.000875
547.250000	0.000881
547.000000	0.000889
546.750000	0.000899
546.500000	0.000910
546.250000	0.000920
546.000000	0.000930
545.750000	0.000936
545.500000	0.000937
545.250000	0.000930
545.000000	0.000912
544.750000	0.000883
544.500000	0.000846
544.250000	0.000805
544.000000	0.000767
543.750000	0.000740
543.500000	0.000728
543.250000	0.000734
543.000000	0.000754
542.750000	0.000783
542.500000	0.000812
542.250000	0.000835
542.000000	0.000844

541.750000	0.000838
541.500000	0.000819
541.250000	0.000790
541.000000	0.000760
540.750000	0.000735
540.500000	0.000719
540.250000	0.000716
540.000000	0.000724
539.750000	0.000739
539.500000	0.000754
539.250000	0.000763
539.000000	0.000759
538.750000	0.000738
538.500000	0.000700
538.250000	0.000648
538.000000	0.000590
537.750000	0.000535
537.500000	0.000491
537.250000	0.000464
537.000000	0.000458
536.750000	0.000472
536.500000	0.000502
536.250000	0.000541
536.000000	0.000584
535.750000	0.000622
535.500000	0.000652

535.250000	0.000670
535.000000	0.000676
534.750000	0.000670
534.500000	0.000654
534.250000	0.000631
534.000000	0.000604
533.750000	0.000578
533.500000	0.000554
533.250000	0.000536
533.000000	0.000524
532.750000	0.000517
532.500000	0.000514
532.250000	0.000514
532.000000	0.000513
531.750000	0.000513
531.500000	0.000511
531.250000	0.000510
531.000000	0.000512
530.750000	0.000517
530.500000	0.000527
530.250000	0.000542
530.000000	0.000561
529.750000	0.000584
529.500000	0.000606
529.250000	0.000625
529.000000	0.000639

528.750000	0.000646
528.500000	0.000648
528.250000	0.000645
528.000000	0.000639
527.750000	0.000631
527.500000	0.000624
527.250000	0.000616
527.000000	0.000606
526.750000	0.000595
526.500000	0.000581
526.250000	0.000565
526.000000	0.000551
525.750000	0.000542
525.500000	0.000540
525.250000	0.000544
525.000000	0.000551
524.750000	0.000559
524.500000	0.000564
524.250000	0.000564
524.000000	0.000557
523.750000	0.000546
523.500000	0.000533
523.250000	0.000519
523.000000	0.000508
522.750000	0.000500
522.500000	0.000498

522.250000	0.000501
522.000000	0.000510
521.750000	0.000524
521.500000	0.000543
521.250000	0.000564
521.000000	0.000583
520.750000	0.000596
520.500000	0.000602
520.250000	0.000599
520.000000	0.000589
519.750000	0.000577
519.500000	0.000569
519.250000	0.000570
519.000000	0.000584
518.750000	0.000611
518.500000	0.000647
518.250000	0.000687
518.000000	0.000725
517.750000	0.000754
517.500000	0.000769
517.250000	0.000766
517.000000	0.000744
516.750000	0.000705
516.500000	0.000655
516.250000	0.000604
516.000000	0.000557

515.750000	0.000519
515.500000	0.000493
515.250000	0.000481
515.000000	0.000482
514.750000	0.000493
514.500000	0.000513
514.250000	0.000537
514.000000	0.000564
513.750000	0.000593
513.500000	0.000621
513.250000	0.000649
513.000000	0.000674
512.750000	0.000693
512.500000	0.000704
512.250000	0.000703
512.000000	0.000692
511.750000	0.000671
511.500000	0.000646
511.250000	0.000623
511.000000	0.000606
510.750000	0.000598
510.500000	0.000599
510.250000	0.000605
510.000000	0.000610
509.750000	0.000610
509.500000	0.000602

509.250000	0.000583
509.000000	0.000558
508.750000	0.000530
508.500000	0.000502
508.250000	0.000481
508.000000	0.000471
507.750000	0.000477
507.500000	0.000500
507.250000	0.000542
507.000000	0.000599
506.750000	0.000664
506.500000	0.000726
506.250000	0.000775
506.000000	0.000802
505.750000	0.000805
505.500000	0.000784
505.250000	0.000744
505.000000	0.000693
504.750000	0.000640
504.500000	0.000595
504.250000	0.000567
504.000000	0.000558
503.750000	0.000571
503.500000	0.000602
503.250000	0.000647
503.000000	0.000698

502.750000	0.000748
502.500000	0.000789
502.250000	0.000814
502.000000	0.000825
501.750000	0.000827
501.500000	0.000830
501.250000	0.000841
501.000000	0.000863
500.750000	0.000895
500.500000	0.000931
500.250000	0.000968
500.000000	0.000999
499.750000	0.001022
499.500000	0.001037
499.250000	0.001047
499.000000	0.001055
498.750000	0.001065
498.500000	0.001078
498.250000	0.001092
498.000000	0.001101
497.750000	0.001102
497.500000	0.001095
497.250000	0.001080
497.000000	0.001067
496.750000	0.001063
496.500000	0.001076

496.250000	0.001108
496.000000	0.001156
495.750000	0.001213
495.500000	0.001268
495.250000	0.001313
495.000000	0.001344
494.750000	0.001361
494.500000	0.001372
494.250000	0.001386
494.000000	0.001410
493.750000	0.001449
493.500000	0.001501
493.250000	0.001564
493.000000	0.001627
492.750000	0.001683
492.500000	0.001724
492.250000	0.001745
492.000000	0.001749
491.750000	0.001739
491.500000	0.001725
491.250000	0.001716
491.000000	0.001717
490.750000	0.001732
490.500000	0.001759
490.250000	0.001797
490.000000	0.001839

489.750000	0.001882
489.500000	0.001922
489.250000	0.001957
489.000000	0.001988
488.750000	0.002016
488.500000	0.002046
488.250000	0.002082
488.000000	0.002124
487.750000	0.002173
487.500000	0.002225
487.250000	0.002274
487.000000	0.002314
486.750000	0.002341
486.500000	0.002352
486.250000	0.002352
486.000000	0.002348
485.750000	0.002348
485.500000	0.002362
485.250000	0.002395
485.000000	0.002446
484.750000	0.002511
484.500000	0.002578
484.250000	0.002635
484.000000	0.002669
483.750000	0.002677
483.500000	0.002662

483.250000	0.002636
483.000000	0.002612
482.750000	0.002603
482.500000	0.002611
482.250000	0.002637
482.000000	0.002676
481.750000	0.002722
481.500000	0.002768
481.250000	0.002811
481.000000	0.002847
480.750000	0.002876
480.500000	0.002899
480.250000	0.002916
480.000000	0.002928
479.750000	0.002935
479.500000	0.002938
479.250000	0.002940
479.000000	0.002947
478.750000	0.002964
478.500000	0.002995
478.250000	0.003042
478.000000	0.003100
477.750000	0.003164
477.500000	0.003227
477.250000	0.003282
477.000000	0.003323

476.750000	0.003351
476.500000	0.003367
476.250000	0.003374
476.000000	0.003377
475.750000	0.003378
475.500000	0.003379
475.250000	0.003381
475.000000	0.003387
474.750000	0.003398
474.500000	0.003418
474.250000	0.003447
474.000000	0.003483
473.750000	0.003522
473.500000	0.003559
473.250000	0.003586
473.000000	0.003597
472.750000	0.003594
472.500000	0.003589
472.250000	0.003598
472.000000	0.003633
471.750000	0.003695
471.500000	0.003773
471.250000	0.003851
471.000000	0.003914
470.750000	0.003955
470.500000	0.003971

470.250000	0.003967
470.000000	0.003949
469.750000	0.003928
469.500000	0.003911
469.250000	0.003904
469.000000	0.003907
468.750000	0.003918
468.500000	0.003932
468.250000	0.003948
468.000000	0.003964
467.750000	0.003983
467.500000	0.004004
467.250000	0.004028
467.000000	0.004051
466.750000	0.004071
466.500000	0.004085
466.250000	0.004092
466.000000	0.004095
465.750000	0.004096
465.500000	0.004098
465.250000	0.004103
465.000000	0.004109
464.750000	0.004115
464.500000	0.004117
464.250000	0.004114
464.000000	0.004108

463.750000	0.004099
463.500000	0.004091
463.250000	0.004085
463.000000	0.004082
462.750000	0.004079
462.500000	0.004076
462.250000	0.004076
462.000000	0.004083
461.750000	0.004103
461.500000	0.004140
461.250000	0.004194
461.000000	0.004259
460.750000	0.004321
460.500000	0.004368
460.250000	0.004390
460.000000	0.004384
459.750000	0.004351
459.500000	0.004300
459.250000	0.004238
459.000000	0.004173
458.750000	0.004113
458.500000	0.004063
458.250000	0.004023
458.000000	0.003995
457.750000	0.003975
457.500000	0.003966

457.250000	0.003970
457.000000	0.003989
456.750000	0.004022
456.500000	0.004063
456.250000	0.004105
456.000000	0.004142
455.750000	0.004170
455.500000	0.004184
455.250000	0.004186
455.000000	0.004179
454.750000	0.004166
454.500000	0.004153
454.250000	0.004143
454.000000	0.004136
453.750000	0.004131
453.500000	0.004125
453.250000	0.004115
453.000000	0.004101
452.750000	0.004089
452.500000	0.004082
452.250000	0.004087
452.000000	0.004107
451.750000	0.004140
451.500000	0.004180
451.250000	0.004221
451.000000	0.004255

450.750000	0.004276
450.500000	0.004279
450.250000	0.004261
450.000000	0.004223
449.750000	0.004169
449.500000	0.004102
449.250000	0.004029
449.000000	0.003954
448.750000	0.003883
448.500000	0.003822
448.250000	0.003774
448.000000	0.003744
447.750000	0.003732
447.500000	0.003737
447.250000	0.003755
447.000000	0.003780
446.750000	0.003806
446.500000	0.003831
446.250000	0.003857
446.000000	0.003887
445.750000	0.003927
445.500000	0.003976
445.250000	0.004031
445.000000	0.004088
444.750000	0.004136
444.500000	0.004166

444.250000	0.004171
444.000000	0.004150
443.750000	0.004107
443.500000	0.004050
443.250000	0.003986
443.000000	0.003919
442.750000	0.003845
442.500000	0.003758
442.250000	0.003655
442.000000	0.003540
441.750000	0.003425
441.500000	0.003329
441.250000	0.003269
441.000000	0.003253
440.750000	0.003278
440.500000	0.003335
440.250000	0.003407
440.000000	0.003480
439.750000	0.003542
439.500000	0.003586
439.250000	0.003609
439.000000	0.003616
438.750000	0.003612
438.500000	0.003602
438.250000	0.003585
438.000000	0.003559

437.750000	0.003520
437.500000	0.003464
437.250000	0.003393
437.000000	0.003312
436.750000	0.003235
436.500000	0.003177
436.250000	0.003150
436.000000	0.003161
435.750000	0.003203
435.500000	0.003262
435.250000	0.003318
435.000000	0.003353
434.750000	0.003354
434.500000	0.003315
434.250000	0.003236
434.000000	0.003129
433.750000	0.003009
433.500000	0.002894
433.250000	0.002800
433.000000	0.002738
432.750000	0.002711
432.500000	0.002718
432.250000	0.002753
432.000000	0.002807
431.750000	0.002870
431.500000	0.002929

431.250000	0.002974
431.000000	0.002993
430.750000	0.002982
430.500000	0.002941
430.250000	0.002877
430.000000	0.002797
429.750000	0.002716
429.500000	0.002644
429.250000	0.002589
429.000000	0.002555
428.750000	0.002541
428.500000	0.002538
428.250000	0.002539
428.000000	0.002538
427.750000	0.002531
427.500000	0.002520
427.250000	0.002510
427.000000	0.002507
426.750000	0.002515
426.500000	0.002534
426.250000	0.002556
426.000000	0.002572
425.750000	0.002571
425.500000	0.002545
425.250000	0.002496
425.000000	0.002432

424.750000	0.002366
424.500000	0.002310
424.250000	0.002270
424.000000	0.002251
423.750000	0.002252
423.500000	0.002270
423.250000	0.002298
423.000000	0.002324
422.750000	0.002339
422.500000	0.002335
422.250000	0.002316
422.000000	0.002287
421.750000	0.002253
421.500000	0.002219
421.250000	0.002186
421.000000	0.002153
420.750000	0.002120
420.500000	0.002083
420.250000	0.002041
420.000000	0.001991
419.750000	0.001931
419.500000	0.001861
419.250000	0.001794
419.000000	0.001758
418.750000	0.001774
418.500000	0.001841

418.250000	0.001938
418.000000	0.002033
417.750000	0.002106
417.500000	0.002145
417.250000	0.002149
417.000000	0.002123
416.750000	0.002071
416.500000	0.002006
416.250000	0.001937
416.000000	0.001877
415.750000	0.001832
415.500000	0.001802
415.250000	0.001778
415.000000	0.001748
414.750000	0.001698
414.500000	0.001621
414.250000	0.001522
414.000000	0.001419
413.750000	0.001334
413.500000	0.001288
413.250000	0.001295
413.000000	0.001357
412.750000	0.001464
412.500000	0.001597
412.250000	0.001735
412.000000	0.001858

411.750000	0.001952
411.500000	0.002008
411.250000	0.002020
411.000000	0.001985
410.750000	0.001904
410.500000	0.001784
410.250000	0.001634
410.000000	0.001470
409.750000	0.001307
409.500000	0.001160
409.250000	0.001034
409.000000	0.000928
408.750000	0.000833
408.500000	0.000742
408.250000	0.000649
408.000000	0.000560
407.750000	0.000485
407.500000	0.000440
407.250000	0.000433
407.000000	0.000463
406.750000	0.000518
406.500000	0.000576
406.250000	0.000620
406.000000	0.000638
405.750000	0.000631
405.500000	0.000609

405.250000	0.000584
405.000000	0.000567
404.750000	0.000557
404.500000	0.000550
404.250000	0.000536
404.000000	0.000512
403.750000	0.000481
403.500000	0.000456
403.250000	0.000452
403.000000	0.000483
402.750000	0.000547
402.500000	0.000631
402.250000	0.000716
402.000000	0.000790
401.750000	0.000841
401.500000	0.000858
401.250000	0.000838
401.000000	0.000789
400.750000	0.000718
400.500000	0.000632
400.250000	0.000534
400.000000	0.000429

A1_2mm_cdown

PE IR SUBTECH SPECTRUM ASCII PEDS 4.00

-1

A1_2mm_c_s_1_1_1.ASC

21/07/23

16:18:35.00

21/12/06

15:42:15.00

ir

Sample 020 By ir Date venerd, luglio 23 2021

400.000000

#HDR

-1

-1

#GR

cm-1

A

1.0

0.0

4000.000000

-0.250000

14401

8

0.073493

0.001658

#DATA

4000.000000 0.001958

3999.750000	0.001956
3999.500000	0.001953
3999.250000	0.001950
3999.000000	0.001947
3998.750000	0.001943
3998.500000	0.001939
3998.250000	0.001933
3998.000000	0.001926
3997.750000	0.001919
3997.500000	0.001915
3997.250000	0.001914
3997.000000	0.001918
3996.750000	0.001928
3996.500000	0.001944
3996.250000	0.001965
3996.000000	0.001989
3995.750000	0.002014
3995.500000	0.002036
3995.250000	0.002052
3995.000000	0.002061
3994.750000	0.002062
3994.500000	0.002057
3994.250000	0.002048
3994.000000	0.002039
3993.750000	0.002031
3993.500000	0.002026

3993.250000	0.002023
3993.000000	0.002019
3992.750000	0.002015
3992.500000	0.002007
3992.250000	0.001995
3992.000000	0.001982
3991.750000	0.001971
3991.500000	0.001964
3991.250000	0.001966
3991.000000	0.001976
3990.750000	0.001994
3990.500000	0.002017
3990.250000	0.002041
3990.000000	0.002061
3989.750000	0.002074
3989.500000	0.002079
3989.250000	0.002078
3989.000000	0.002072
3988.750000	0.002065
3988.500000	0.002060
3988.250000	0.002059
3988.000000	0.002062
3987.750000	0.002070
3987.500000	0.002080
3987.250000	0.002093
3987.000000	0.002105

3986.750000	0.002116
3986.500000	0.002123
3986.250000	0.002126
3986.000000	0.002124
3985.750000	0.002116
3985.500000	0.002103
3985.250000	0.002088
3985.000000	0.002072
3984.750000	0.002060
3984.500000	0.002051
3984.250000	0.002049
3984.000000	0.002052
3983.750000	0.002059
3983.500000	0.002066
3983.250000	0.002073
3983.000000	0.002078
3982.750000	0.002080
3982.500000	0.002081
3982.250000	0.002083
3982.000000	0.002086
3981.750000	0.002093
3981.500000	0.002103
3981.250000	0.002116
3981.000000	0.002131
3980.750000	0.002144
3980.500000	0.002156

3980.250000	0.002164
3980.000000	0.002169
3979.750000	0.002169
3979.500000	0.002166
3979.250000	0.002160
3979.000000	0.002152
3978.750000	0.002144
3978.500000	0.002139
3978.250000	0.002138
3978.000000	0.002142
3977.750000	0.002152
3977.500000	0.002166
3977.250000	0.002183
3977.000000	0.002198
3976.750000	0.002210
3976.500000	0.002216
3976.250000	0.002217
3976.000000	0.002212
3975.750000	0.002206
3975.500000	0.002199
3975.250000	0.002195
3975.000000	0.002194
3974.750000	0.002196
3974.500000	0.002201
3974.250000	0.002208
3974.000000	0.002215

3973.750000	0.002223
3973.500000	0.002230
3973.250000	0.002238
3973.000000	0.002246
3972.750000	0.002254
3972.500000	0.002261
3972.250000	0.002266
3972.000000	0.002268
3971.750000	0.002268
3971.500000	0.002265
3971.250000	0.002261
3971.000000	0.002255
3970.750000	0.002250
3970.500000	0.002245
3970.250000	0.002240
3970.000000	0.002235
3969.750000	0.002231
3969.500000	0.002227
3969.250000	0.002224
3969.000000	0.002222
3968.750000	0.002221
3968.500000	0.002220
3968.250000	0.002217
3968.000000	0.002210
3967.750000	0.002199
3967.500000	0.002184

3967.250000	0.002165
3967.000000	0.002147
3966.750000	0.002132
3966.500000	0.002123
3966.250000	0.002122
3966.000000	0.002129
3965.750000	0.002144
3965.500000	0.002163
3965.250000	0.002184
3965.000000	0.002204
3964.750000	0.002223
3964.500000	0.002238
3964.250000	0.002250
3964.000000	0.002259
3963.750000	0.002267
3963.500000	0.002273
3963.250000	0.002278
3963.000000	0.002282
3962.750000	0.002285
3962.500000	0.002288
3962.250000	0.002290
3962.000000	0.002292
3961.750000	0.002291
3961.500000	0.002289
3961.250000	0.002284
3961.000000	0.002279

3960.750000	0.002273
3960.500000	0.002268
3960.250000	0.002267
3960.000000	0.002270
3959.750000	0.002277
3959.500000	0.002287
3959.250000	0.002298
3959.000000	0.002306
3958.750000	0.002308
3958.500000	0.002305
3958.250000	0.002296
3958.000000	0.002284
3957.750000	0.002272
3957.500000	0.002264
3957.250000	0.002262
3957.000000	0.002264
3956.750000	0.002271
3956.500000	0.002277
3956.250000	0.002281
3956.000000	0.002280
3955.750000	0.002274
3955.500000	0.002264
3955.250000	0.002254
3955.000000	0.002248
3954.750000	0.002248
3954.500000	0.002256

3954.250000	0.002271
3954.000000	0.002289
3953.750000	0.002305
3953.500000	0.002315
3953.250000	0.002317
3953.000000	0.002309
3952.750000	0.002293
3952.500000	0.002275
3952.250000	0.002260
3952.000000	0.002252
3951.750000	0.002258
3951.500000	0.002277
3951.250000	0.002310
3951.000000	0.002352
3950.750000	0.002396
3950.500000	0.002437
3950.250000	0.002468
3950.000000	0.002487
3949.750000	0.002493
3949.500000	0.002489
3949.250000	0.002479
3949.000000	0.002468
3948.750000	0.002460
3948.500000	0.002457
3948.250000	0.002457
3948.000000	0.002460

3947.750000	0.002461
3947.500000	0.002459
3947.250000	0.002451
3947.000000	0.002440
3946.750000	0.002427
3946.500000	0.002416
3946.250000	0.002409
3946.000000	0.002407
3945.750000	0.002410
3945.500000	0.002415
3945.250000	0.002421
3945.000000	0.002426
3944.750000	0.002427
3944.500000	0.002424
3944.250000	0.002420
3944.000000	0.002415
3943.750000	0.002413
3943.500000	0.002415
3943.250000	0.002420
3943.000000	0.002428
3942.750000	0.002434
3942.500000	0.002439
3942.250000	0.002440
3942.000000	0.002440
3941.750000	0.002439
3941.500000	0.002439

3941.250000	0.002441
3941.000000	0.002446
3940.750000	0.002455
3940.500000	0.002467
3940.250000	0.002481
3940.000000	0.002495
3939.750000	0.002507
3939.500000	0.002515
3939.250000	0.002519
3939.000000	0.002517
3938.750000	0.002511
3938.500000	0.002501
3938.250000	0.002489
3938.000000	0.002478
3937.750000	0.002469
3937.500000	0.002461
3937.250000	0.002456
3937.000000	0.002452
3936.750000	0.002450
3936.500000	0.002449
3936.250000	0.002448
3936.000000	0.002449
3935.750000	0.002449
3935.500000	0.002450
3935.250000	0.002452
3935.000000	0.002454

3934.750000	0.002457
3934.500000	0.002462
3934.250000	0.002467
3934.000000	0.002474
3933.750000	0.002484
3933.500000	0.002498
3933.250000	0.002516
3933.000000	0.002537
3932.750000	0.002557
3932.500000	0.002574
3932.250000	0.002583
3932.000000	0.002583
3931.750000	0.002576
3931.500000	0.002565
3931.250000	0.002552
3931.000000	0.002540
3930.750000	0.002534
3930.500000	0.002535
3930.250000	0.002541
3930.000000	0.002554
3929.750000	0.002570
3929.500000	0.002587
3929.250000	0.002603
3929.000000	0.002617
3928.750000	0.002628
3928.500000	0.002635

3928.250000	0.002638
3928.000000	0.002636
3927.750000	0.002630
3927.500000	0.002619
3927.250000	0.002605
3927.000000	0.002590
3926.750000	0.002575
3926.500000	0.002564
3926.250000	0.002560
3926.000000	0.002565
3925.750000	0.002579
3925.500000	0.002601
3925.250000	0.002628
3925.000000	0.002654
3924.750000	0.002676
3924.500000	0.002691
3924.250000	0.002696
3924.000000	0.002693
3923.750000	0.002681
3923.500000	0.002664
3923.250000	0.002644
3923.000000	0.002625
3922.750000	0.002607
3922.500000	0.002594
3922.250000	0.002586
3922.000000	0.002583

3921.750000	0.002586
3921.500000	0.002596
3921.250000	0.002610
3921.000000	0.002627
3920.750000	0.002643
3920.500000	0.002651
3920.250000	0.002648
3920.000000	0.002631
3919.750000	0.002601
3919.500000	0.002564
3919.250000	0.002525
3919.000000	0.002491
3918.750000	0.002468
3918.500000	0.002459
3918.250000	0.002469
3918.000000	0.002497
3917.750000	0.002539
3917.500000	0.002584
3917.250000	0.002624
3917.000000	0.002648
3916.750000	0.002654
3916.500000	0.002643
3916.250000	0.002620
3916.000000	0.002595
3915.750000	0.002572
3915.500000	0.002558

3915.250000	0.002554
3915.000000	0.002559
3914.750000	0.002574
3914.500000	0.002593
3914.250000	0.002615
3914.000000	0.002635
3913.750000	0.002651
3913.500000	0.002663
3913.250000	0.002669
3913.000000	0.002671
3912.750000	0.002669
3912.500000	0.002664
3912.250000	0.002657
3912.000000	0.002649
3911.750000	0.002643
3911.500000	0.002638
3911.250000	0.002635
3911.000000	0.002634
3910.750000	0.002635
3910.500000	0.002636
3910.250000	0.002638
3910.000000	0.002640
3909.750000	0.002641
3909.500000	0.002641
3909.250000	0.002639
3909.000000	0.002635

3908.750000	0.002629
3908.500000	0.002622
3908.250000	0.002616
3908.000000	0.002613
3907.750000	0.002614
3907.500000	0.002620
3907.250000	0.002630
3907.000000	0.002641
3906.750000	0.002650
3906.500000	0.002654
3906.250000	0.002650
3906.000000	0.002637
3905.750000	0.002617
3905.500000	0.002593
3905.250000	0.002570
3905.000000	0.002551
3904.750000	0.002536
3904.500000	0.002525
3904.250000	0.002514
3904.000000	0.002505
3903.750000	0.002501
3903.500000	0.002506
3903.250000	0.002521
3903.000000	0.002547
3902.750000	0.002581
3902.500000	0.002620

3902.250000	0.002654
3902.000000	0.002676
3901.750000	0.002681
3901.500000	0.002669
3901.250000	0.002643
3901.000000	0.002611
3900.750000	0.002579
3900.500000	0.002557
3900.250000	0.002555
3900.000000	0.002578
3899.750000	0.002621
3899.500000	0.002673
3899.250000	0.002715
3899.000000	0.002735
3898.750000	0.002730
3898.500000	0.002707
3898.250000	0.002676
3898.000000	0.002650
3897.750000	0.002637
3897.500000	0.002641
3897.250000	0.002663
3897.000000	0.002697
3896.750000	0.002738
3896.500000	0.002776
3896.250000	0.002805
3896.000000	0.002824

3895.750000	0.002833
3895.500000	0.002835
3895.250000	0.002835
3895.000000	0.002835
3894.750000	0.002836
3894.500000	0.002834
3894.250000	0.002827
3894.000000	0.002813
3893.750000	0.002790
3893.500000	0.002761
3893.250000	0.002729
3893.000000	0.002697
3892.750000	0.002671
3892.500000	0.002657
3892.250000	0.002660
3892.000000	0.002677
3891.750000	0.002699
3891.500000	0.002716
3891.250000	0.002716
3891.000000	0.002697
3890.750000	0.002667
3890.500000	0.002637
3890.250000	0.002615
3890.000000	0.002611
3889.750000	0.002625
3889.500000	0.002658

3889.250000	0.002701
3889.000000	0.002748
3888.750000	0.002786
3888.500000	0.002808
3888.250000	0.002807
3888.000000	0.002779
3887.750000	0.002726
3887.500000	0.002654
3887.250000	0.002573
3887.000000	0.002499
3886.750000	0.002448
3886.500000	0.002432
3886.250000	0.002448
3886.000000	0.002482
3885.750000	0.002516
3885.500000	0.002539
3885.250000	0.002552
3885.000000	0.002564
3884.750000	0.002583
3884.500000	0.002615
3884.250000	0.002662
3884.000000	0.002721
3883.750000	0.002788
3883.500000	0.002854
3883.250000	0.002912
3883.000000	0.002951

3882.750000	0.002968
3882.500000	0.002962
3882.250000	0.002938
3882.000000	0.002902
3881.750000	0.002868
3881.500000	0.002846
3881.250000	0.002842
3881.000000	0.002855
3880.750000	0.002871
3880.500000	0.002874
3880.250000	0.002852
3880.000000	0.002805
3879.750000	0.002743
3879.500000	0.002681
3879.250000	0.002630
3879.000000	0.002599
3878.750000	0.002592
3878.500000	0.002613
3878.250000	0.002656
3878.000000	0.002716
3877.750000	0.002782
3877.500000	0.002844
3877.250000	0.002894
3877.000000	0.002926
3876.750000	0.002941
3876.500000	0.002939

3876.250000	0.002923
3876.000000	0.002898
3875.750000	0.002869
3875.500000	0.002842
3875.250000	0.002824
3875.000000	0.002821
3874.750000	0.002830
3874.500000	0.002846
3874.250000	0.002857
3874.000000	0.002858
3873.750000	0.002848
3873.500000	0.002834
3873.250000	0.002821
3873.000000	0.002816
3872.750000	0.002820
3872.500000	0.002833
3872.250000	0.002852
3872.000000	0.002870
3871.750000	0.002883
3871.500000	0.002886
3871.250000	0.002882
3871.000000	0.002881
3870.750000	0.002893
3870.500000	0.002927
3870.250000	0.002972
3870.000000	0.003009

3869.750000	0.003017
3869.500000	0.002988
3869.250000	0.002935
3869.000000	0.002876
3868.750000	0.002829
3868.500000	0.002805
3868.250000	0.002808
3868.000000	0.002838
3867.750000	0.002888
3867.500000	0.002949
3867.250000	0.003007
3867.000000	0.003052
3866.750000	0.003075
3866.500000	0.003076
3866.250000	0.003059
3866.000000	0.003034
3865.750000	0.003009
3865.500000	0.002987
3865.250000	0.002965
3865.000000	0.002935
3864.750000	0.002897
3864.500000	0.002854
3864.250000	0.002817
3864.000000	0.002796
3863.750000	0.002795
3863.500000	0.002817

3863.250000	0.002859
3863.000000	0.002921
3862.750000	0.002998
3862.500000	0.003080
3862.250000	0.003154
3862.000000	0.003206
3861.750000	0.003227
3861.500000	0.003216
3861.250000	0.003180
3861.000000	0.003129
3860.750000	0.003075
3860.500000	0.003026
3860.250000	0.002988
3860.000000	0.002966
3859.750000	0.002958
3859.500000	0.002962
3859.250000	0.002974
3859.000000	0.002991
3858.750000	0.003010
3858.500000	0.003030
3858.250000	0.003052
3858.000000	0.003074
3857.750000	0.003094
3857.500000	0.003105
3857.250000	0.003100
3857.000000	0.003075

3856.750000	0.003025
3856.500000	0.002946
3856.250000	0.002835
3856.000000	0.002689
3855.750000	0.002510
3855.500000	0.002306
3855.250000	0.002101
3855.000000	0.001926
3854.750000	0.001816
3854.500000	0.001783
3854.250000	0.001816
3854.000000	0.001880
3853.750000	0.001948
3853.500000	0.002010
3853.250000	0.002073
3853.000000	0.002145
3852.750000	0.002229
3852.500000	0.002323
3852.250000	0.002428
3852.000000	0.002544
3851.750000	0.002666
3851.500000	0.002790
3851.250000	0.002906
3851.000000	0.003008
3850.750000	0.003092
3850.500000	0.003158

3850.250000	0.003209
3850.000000	0.003249
3849.750000	0.003279
3849.500000	0.003300
3849.250000	0.003310
3849.000000	0.003306
3848.750000	0.003289
3848.500000	0.003260
3848.250000	0.003222
3848.000000	0.003180
3847.750000	0.003139
3847.500000	0.003101
3847.250000	0.003067
3847.000000	0.003035
3846.750000	0.003006
3846.500000	0.002977
3846.250000	0.002949
3846.000000	0.002923
3845.750000	0.002899
3845.500000	0.002882
3845.250000	0.002874
3845.000000	0.002879
3844.750000	0.002900
3844.500000	0.002935
3844.250000	0.002977
3844.000000	0.003016

3843.750000	0.003041
3843.500000	0.003048
3843.250000	0.003038
3843.000000	0.003015
3842.750000	0.002986
3842.500000	0.002957
3842.250000	0.002934
3842.000000	0.002924
3841.750000	0.002932
3841.500000	0.002959
3841.250000	0.003001
3841.000000	0.003046
3840.750000	0.003085
3840.500000	0.003109
3840.250000	0.003113
3840.000000	0.003100
3839.750000	0.003070
3839.500000	0.003026
3839.250000	0.002975
3839.000000	0.002928
3838.750000	0.002895
3838.500000	0.002885
3838.250000	0.002890
3838.000000	0.002895
3837.750000	0.002887
3837.500000	0.002866

3837.250000	0.002844
3837.000000	0.002832
3836.750000	0.002836
3836.500000	0.002853
3836.250000	0.002880
3836.000000	0.002917
3835.750000	0.002960
3835.500000	0.003002
3835.250000	0.003032
3835.000000	0.003040
3834.750000	0.003022
3834.500000	0.002985
3834.250000	0.002940
3834.000000	0.002898
3833.750000	0.002869
3833.500000	0.002856
3833.250000	0.002862
3833.000000	0.002885
3832.750000	0.002921
3832.500000	0.002966
3832.250000	0.003010
3832.000000	0.003039
3831.750000	0.003043
3831.500000	0.003020
3831.250000	0.002973
3831.000000	0.002916

3830.750000	0.002861
3830.500000	0.002819
3830.250000	0.002798
3830.000000	0.002801
3829.750000	0.002825
3829.500000	0.002867
3829.250000	0.002919
3829.000000	0.002973
3828.750000	0.003022
3828.500000	0.003062
3828.250000	0.003090
3828.000000	0.003108
3827.750000	0.003120
3827.500000	0.003128
3827.250000	0.003131
3827.000000	0.003130
3826.750000	0.003122
3826.500000	0.003108
3826.250000	0.003089
3826.000000	0.003070
3825.750000	0.003054
3825.500000	0.003045
3825.250000	0.003043
3825.000000	0.003048
3824.750000	0.003057
3824.500000	0.003063

3824.250000	0.003061
3824.000000	0.003044
3823.750000	0.003008
3823.500000	0.002948
3823.250000	0.002865
3823.000000	0.002764
3822.750000	0.002655
3822.500000	0.002555
3822.250000	0.002486
3822.000000	0.002468
3821.750000	0.002509
3821.500000	0.002594
3821.250000	0.002694
3821.000000	0.002779
3820.750000	0.002833
3820.500000	0.002863
3820.250000	0.002883
3820.000000	0.002904
3819.750000	0.002934
3819.500000	0.002973
3819.250000	0.003019
3819.000000	0.003066
3818.750000	0.003104
3818.500000	0.003124
3818.250000	0.003117
3818.000000	0.003081

3817.750000	0.003018
3817.500000	0.002939
3817.250000	0.002862
3817.000000	0.002811
3816.750000	0.002797
3816.500000	0.002815
3816.250000	0.002844
3816.000000	0.002857
3815.750000	0.002847
3815.500000	0.002820
3815.250000	0.002794
3815.000000	0.002782
3814.750000	0.002788
3814.500000	0.002813
3814.250000	0.002854
3814.000000	0.002907
3813.750000	0.002966
3813.500000	0.003024
3813.250000	0.003070
3813.000000	0.003101
3812.750000	0.003114
3812.500000	0.003110
3812.250000	0.003095
3812.000000	0.003073
3811.750000	0.003048
3811.500000	0.003026

3811.250000	0.003006
3811.000000	0.002990
3810.750000	0.002976
3810.500000	0.002963
3810.250000	0.002949
3810.000000	0.002929
3809.750000	0.002902
3809.500000	0.002864
3809.250000	0.002815
3809.000000	0.002756
3808.750000	0.002687
3808.500000	0.002618
3808.250000	0.002558
3808.000000	0.002522
3807.750000	0.002520
3807.500000	0.002549
3807.250000	0.002595
3807.000000	0.002635
3806.750000	0.002657
3806.500000	0.002660
3806.250000	0.002655
3806.000000	0.002654
3805.750000	0.002666
3805.500000	0.002696
3805.250000	0.002742
3805.000000	0.002800

3804.750000	0.002864
3804.500000	0.002924
3804.250000	0.002968
3804.000000	0.002986
3803.750000	0.002971
3803.500000	0.002923
3803.250000	0.002844
3803.000000	0.002745
3802.750000	0.002643
3802.500000	0.002562
3802.250000	0.002524
3802.000000	0.002541
3801.750000	0.002602
3801.500000	0.002678
3801.250000	0.002742
3801.000000	0.002782
3800.750000	0.002803
3800.500000	0.002818
3800.250000	0.002836
3800.000000	0.002860
3799.750000	0.002892
3799.500000	0.002929
3799.250000	0.002969
3799.000000	0.003009
3798.750000	0.003041
3798.500000	0.003061

3798.250000	0.003066
3798.000000	0.003057
3797.750000	0.003039
3797.500000	0.003020
3797.250000	0.003003
3797.000000	0.002990
3796.750000	0.002977
3796.500000	0.002960
3796.250000	0.002936
3796.000000	0.002905
3795.750000	0.002872
3795.500000	0.002842
3795.250000	0.002819
3795.000000	0.002806
3794.750000	0.002805
3794.500000	0.002816
3794.250000	0.002836
3794.000000	0.002862
3793.750000	0.002888
3793.500000	0.002910
3793.250000	0.002924
3793.000000	0.002927
3792.750000	0.002921
3792.500000	0.002908
3792.250000	0.002890
3792.000000	0.002872

3791.750000	0.002854
3791.500000	0.002838
3791.250000	0.002825
3791.000000	0.002813
3790.750000	0.002805
3790.500000	0.002802
3790.250000	0.002803
3790.000000	0.002810
3789.750000	0.002820
3789.500000	0.002832
3789.250000	0.002843
3789.000000	0.002849
3788.750000	0.002850
3788.500000	0.002844
3788.250000	0.002833
3788.000000	0.002818
3787.750000	0.002803
3787.500000	0.002789
3787.250000	0.002780
3787.000000	0.002777
3786.750000	0.002782
3786.500000	0.002795
3786.250000	0.002818
3786.000000	0.002849
3785.750000	0.002885
3785.500000	0.002925

3785.250000	0.002963
3785.000000	0.002994
3784.750000	0.003014
3784.500000	0.003020
3784.250000	0.003012
3784.000000	0.002993
3783.750000	0.002967
3783.500000	0.002937
3783.250000	0.002909
3783.000000	0.002884
3782.750000	0.002864
3782.500000	0.002848
3782.250000	0.002835
3782.000000	0.002822
3781.750000	0.002808
3781.500000	0.002790
3781.250000	0.002770
3781.000000	0.002750
3780.750000	0.002734
3780.500000	0.002726
3780.250000	0.002729
3780.000000	0.002741
3779.750000	0.002755
3779.500000	0.002764
3779.250000	0.002765
3779.000000	0.002756

3778.750000	0.002744
3778.500000	0.002733
3778.250000	0.002728
3778.000000	0.002734
3777.750000	0.002750
3777.500000	0.002775
3777.250000	0.002803
3777.000000	0.002828
3776.750000	0.002846
3776.500000	0.002852
3776.250000	0.002846
3776.000000	0.002831
3775.750000	0.002812
3775.500000	0.002793
3775.250000	0.002778
3775.000000	0.002769
3774.750000	0.002767
3774.500000	0.002770
3774.250000	0.002776
3774.000000	0.002785
3773.750000	0.002792
3773.500000	0.002796
3773.250000	0.002795
3773.000000	0.002787
3772.750000	0.002772
3772.500000	0.002749

3772.250000	0.002720
3772.000000	0.002686
3771.750000	0.002649
3771.500000	0.002614
3771.250000	0.002588
3771.000000	0.002577
3770.750000	0.002588
3770.500000	0.002620
3770.250000	0.002664
3770.000000	0.002705
3769.750000	0.002730
3769.500000	0.002732
3769.250000	0.002715
3769.000000	0.002687
3768.750000	0.002658
3768.500000	0.002635
3768.250000	0.002625
3768.000000	0.002632
3767.750000	0.002653
3767.500000	0.002689
3767.250000	0.002732
3767.000000	0.002780
3766.750000	0.002827
3766.500000	0.002871
3766.250000	0.002905
3766.000000	0.002926

3765.750000	0.002932
3765.500000	0.002921
3765.250000	0.002897
3765.000000	0.002867
3764.750000	0.002837
3764.500000	0.002811
3764.250000	0.002793
3764.000000	0.002785
3763.750000	0.002786
3763.500000	0.002794
3763.250000	0.002804
3763.000000	0.002810
3762.750000	0.002811
3762.500000	0.002802
3762.250000	0.002783
3762.000000	0.002755
3761.750000	0.002720
3761.500000	0.002682
3761.250000	0.002646
3761.000000	0.002621
3760.750000	0.002615
3760.500000	0.002635
3760.250000	0.002679
3760.000000	0.002735
3759.750000	0.002787
3759.500000	0.002819

3759.250000	0.002827
3759.000000	0.002815
3758.750000	0.002791
3758.500000	0.002767
3758.250000	0.002752
3758.000000	0.002756
3757.750000	0.002783
3757.500000	0.002833
3757.250000	0.002896
3757.000000	0.002959
3756.750000	0.003006
3756.500000	0.003030
3756.250000	0.003029
3756.000000	0.003010
3755.750000	0.002980
3755.500000	0.002946
3755.250000	0.002914
3755.000000	0.002883
3754.750000	0.002852
3754.500000	0.002813
3754.250000	0.002762
3754.000000	0.002694
3753.750000	0.002608
3753.500000	0.002515
3753.250000	0.002432
3753.000000	0.002379

3752.750000	0.002367
3752.500000	0.002387
3752.250000	0.002417
3752.000000	0.002439
3751.750000	0.002445
3751.500000	0.002440
3751.250000	0.002430
3751.000000	0.002416
3750.750000	0.002405
3750.500000	0.002415
3750.250000	0.002468
3750.000000	0.002571
3749.750000	0.002705
3749.500000	0.002830
3749.250000	0.002912
3749.000000	0.002945
3748.750000	0.002949
3748.500000	0.002946
3748.250000	0.002952
3748.000000	0.002971
3747.750000	0.002998
3747.500000	0.003027
3747.250000	0.003042
3747.000000	0.003029
3746.750000	0.002974
3746.500000	0.002867

3746.250000	0.002702
3746.000000	0.002487
3745.750000	0.002254
3745.500000	0.002056
3745.250000	0.001952
3745.000000	0.001954
3744.750000	0.002013
3744.500000	0.002040
3744.250000	0.001989
3744.000000	0.001886
3743.750000	0.001801
3743.500000	0.001786
3743.250000	0.001853
3743.000000	0.001986
3742.750000	0.002170
3742.500000	0.002385
3742.250000	0.002609
3742.000000	0.002822
3741.750000	0.003002
3741.500000	0.003133
3741.250000	0.003211
3741.000000	0.003240
3740.750000	0.003230
3740.500000	0.003197
3740.250000	0.003149
3740.000000	0.003095

3739.750000	0.003040
3739.500000	0.002990
3739.250000	0.002946
3739.000000	0.002911
3738.750000	0.002882
3738.500000	0.002857
3738.250000	0.002833
3738.000000	0.002807
3737.750000	0.002782
3737.500000	0.002762
3737.250000	0.002751
3737.000000	0.002752
3736.750000	0.002769
3736.500000	0.002802
3736.250000	0.002853
3736.000000	0.002917
3735.750000	0.002986
3735.500000	0.003044
3735.250000	0.003075
3735.000000	0.003072
3734.750000	0.003039
3734.500000	0.002988
3734.250000	0.002929
3734.000000	0.002873
3733.750000	0.002823
3733.500000	0.002785

3733.250000	0.002766
3733.000000	0.002769
3732.750000	0.002793
3732.500000	0.002824
3732.250000	0.002848
3732.000000	0.002852
3731.750000	0.002834
3731.500000	0.002803
3731.250000	0.002770
3731.000000	0.002747
3730.750000	0.002740
3730.500000	0.002750
3730.250000	0.002774
3730.000000	0.002806
3729.750000	0.002837
3729.500000	0.002859
3729.250000	0.002864
3729.000000	0.002850
3728.750000	0.002817
3728.500000	0.002770
3728.250000	0.002714
3728.000000	0.002660
3727.750000	0.002614
3727.500000	0.002583
3727.250000	0.002568
3727.000000	0.002570

3726.750000	0.002585
3726.500000	0.002607
3726.250000	0.002632
3726.000000	0.002654
3725.750000	0.002673
3725.500000	0.002686
3725.250000	0.002695
3725.000000	0.002702
3724.750000	0.002710
3724.500000	0.002722
3724.250000	0.002737
3724.000000	0.002757
3723.750000	0.002780
3723.500000	0.002806
3723.250000	0.002835
3723.000000	0.002865
3722.750000	0.002891
3722.500000	0.002903
3722.250000	0.002897
3722.000000	0.002871
3721.750000	0.002828
3721.500000	0.002777
3721.250000	0.002727
3721.000000	0.002688
3720.750000	0.002666
3720.500000	0.002665

3720.250000	0.002688
3720.000000	0.002731
3719.750000	0.002784
3719.500000	0.002838
3719.250000	0.002880
3719.000000	0.002904
3718.750000	0.002907
3718.500000	0.002893
3718.250000	0.002868
3718.000000	0.002839
3717.750000	0.002812
3717.500000	0.002791
3717.250000	0.002777
3717.000000	0.002770
3716.750000	0.002766
3716.500000	0.002765
3716.250000	0.002765
3716.000000	0.002765
3715.750000	0.002765
3715.500000	0.002762
3715.250000	0.002756
3715.000000	0.002744
3714.750000	0.002723
3714.500000	0.002691
3714.250000	0.002647
3714.000000	0.002594

3713.750000	0.002536
3713.500000	0.002484
3713.250000	0.002453
3713.000000	0.002458
3712.750000	0.002500
3712.500000	0.002566
3712.250000	0.002631
3712.000000	0.002672
3711.750000	0.002685
3711.500000	0.002678
3711.250000	0.002666
3711.000000	0.002659
3710.750000	0.002666
3710.500000	0.002691
3710.250000	0.002734
3710.000000	0.002791
3709.750000	0.002846
3709.500000	0.002884
3709.250000	0.002893
3709.000000	0.002875
3708.750000	0.002838
3708.500000	0.002796
3708.250000	0.002761
3708.000000	0.002742
3707.750000	0.002741
3707.500000	0.002757

3707.250000	0.002786
3707.000000	0.002823
3706.750000	0.002859
3706.500000	0.002890
3706.250000	0.002911
3706.000000	0.002919
3705.750000	0.002914
3705.500000	0.002897
3705.250000	0.002870
3705.000000	0.002836
3704.750000	0.002797
3704.500000	0.002757
3704.250000	0.002717
3704.000000	0.002679
3703.750000	0.002645
3703.500000	0.002617
3703.250000	0.002601
3703.000000	0.002602
3702.750000	0.002624
3702.500000	0.002665
3702.250000	0.002717
3702.000000	0.002765
3701.750000	0.002795
3701.500000	0.002801
3701.250000	0.002786
3701.000000	0.002759

3700.750000	0.002732
3700.500000	0.002715
3700.250000	0.002716
3700.000000	0.002736
3699.750000	0.002772
3699.500000	0.002817
3699.250000	0.002861
3699.000000	0.002894
3698.750000	0.002912
3698.500000	0.002913
3698.250000	0.002903
3698.000000	0.002889
3697.750000	0.002877
3697.500000	0.002874
3697.250000	0.002881
3697.000000	0.002896
3696.750000	0.002916
3696.500000	0.002936
3696.250000	0.002951
3696.000000	0.002958
3695.750000	0.002957
3695.500000	0.002949
3695.250000	0.002938
3695.000000	0.002926
3694.750000	0.002917
3694.500000	0.002911

3694.250000	0.002907
3694.000000	0.002904
3693.750000	0.002899
3693.500000	0.002889
3693.250000	0.002874
3693.000000	0.002853
3692.750000	0.002832
3692.500000	0.002815
3692.250000	0.002810
3692.000000	0.002823
3691.750000	0.002851
3691.500000	0.002884
3691.250000	0.002908
3691.000000	0.002910
3690.750000	0.002889
3690.500000	0.002845
3690.250000	0.002785
3690.000000	0.002719
3689.750000	0.002663
3689.500000	0.002639
3689.250000	0.002668
3689.000000	0.002751
3688.750000	0.002866
3688.500000	0.002973
3688.250000	0.003041
3688.000000	0.003067

3687.750000	0.003065
3687.500000	0.003060
3687.250000	0.003065
3687.000000	0.003088
3686.750000	0.003130
3686.500000	0.003185
3686.250000	0.003248
3686.000000	0.003307
3685.750000	0.003354
3685.500000	0.003383
3685.250000	0.003390
3685.000000	0.003379
3684.750000	0.003352
3684.500000	0.003316
3684.250000	0.003278
3684.000000	0.003242
3683.750000	0.003213
3683.500000	0.003192
3683.250000	0.003181
3683.000000	0.003179
3682.750000	0.003184
3682.500000	0.003194
3682.250000	0.003209
3682.000000	0.003227
3681.750000	0.003246
3681.500000	0.003267

3681.250000	0.003287
3681.000000	0.003307
3680.750000	0.003326
3680.500000	0.003345
3680.250000	0.003364
3680.000000	0.003384
3679.750000	0.003404
3679.500000	0.003421
3679.250000	0.003434
3679.000000	0.003441
3678.750000	0.003441
3678.500000	0.003433
3678.250000	0.003416
3678.000000	0.003386
3677.750000	0.003341
3677.500000	0.003280
3677.250000	0.003203
3677.000000	0.003118
3676.750000	0.003041
3676.500000	0.002996
3676.250000	0.003016
3676.000000	0.003114
3675.750000	0.003270
3675.500000	0.003431
3675.250000	0.003545
3675.000000	0.003595

3674.750000	0.003600
3674.500000	0.003588
3674.250000	0.003580
3674.000000	0.003587
3673.750000	0.003611
3673.500000	0.003650
3673.250000	0.003693
3673.000000	0.003728
3672.750000	0.003741
3672.500000	0.003726
3672.250000	0.003685
3672.000000	0.003631
3671.750000	0.003582
3671.500000	0.003554
3671.250000	0.003553
3671.000000	0.003572
3670.750000	0.003595
3670.500000	0.003613
3670.250000	0.003625
3670.000000	0.003640
3669.750000	0.003671
3669.500000	0.003722
3669.250000	0.003792
3669.000000	0.003872
3668.750000	0.003948
3668.500000	0.004010

3668.250000	0.004049
3668.000000	0.004065
3667.750000	0.004061
3667.500000	0.004045
3667.250000	0.004026
3667.000000	0.004009
3666.750000	0.004000
3666.500000	0.003998
3666.250000	0.004001
3666.000000	0.004003
3665.750000	0.004002
3665.500000	0.003999
3665.250000	0.003993
3665.000000	0.003988
3664.750000	0.003986
3664.500000	0.003987
3664.250000	0.003991
3664.000000	0.003997
3663.750000	0.004004
3663.500000	0.004012
3663.250000	0.004022
3663.000000	0.004034
3662.750000	0.004047
3662.500000	0.004063
3662.250000	0.004079
3662.000000	0.004094

3661.750000	0.004106
3661.500000	0.004115
3661.250000	0.004122
3661.000000	0.004128
3660.750000	0.004133
3660.500000	0.004138
3660.250000	0.004142
3660.000000	0.004145
3659.750000	0.004144
3659.500000	0.004140
3659.250000	0.004132
3659.000000	0.004121
3658.750000	0.004109
3658.500000	0.004096
3658.250000	0.004082
3658.000000	0.004069
3657.750000	0.004060
3657.500000	0.004060
3657.250000	0.004079
3657.000000	0.004121
3656.750000	0.004180
3656.500000	0.004243
3656.250000	0.004294
3656.000000	0.004323
3655.750000	0.004334
3655.500000	0.004337

3655.250000	0.004345
3655.000000	0.004365
3654.750000	0.004399
3654.500000	0.004444
3654.250000	0.004491
3654.000000	0.004530
3653.750000	0.004554
3653.500000	0.004556
3653.250000	0.004536
3653.000000	0.004500
3652.750000	0.004458
3652.500000	0.004421
3652.250000	0.004399
3652.000000	0.004400
3651.750000	0.004422
3651.500000	0.004463
3651.250000	0.004512
3651.000000	0.004563
3650.750000	0.004607
3650.500000	0.004644
3650.250000	0.004671
3650.000000	0.004689
3649.750000	0.004701
3649.500000	0.004712
3649.250000	0.004727
3649.000000	0.004754

3648.750000	0.004803
3648.500000	0.004879
3648.250000	0.004983
3648.000000	0.005110
3647.750000	0.005243
3647.500000	0.005360
3647.250000	0.005437
3647.000000	0.005464
3646.750000	0.005444
3646.500000	0.005390
3646.250000	0.005319
3646.000000	0.005247
3645.750000	0.005189
3645.500000	0.005150
3645.250000	0.005135
3645.000000	0.005139
3644.750000	0.005158
3644.500000	0.005183
3644.250000	0.005209
3644.000000	0.005234
3643.750000	0.005258
3643.500000	0.005281
3643.250000	0.005306
3643.000000	0.005332
3642.750000	0.005356
3642.500000	0.005377

3642.250000	0.005389
3642.000000	0.005393
3641.750000	0.005388
3641.500000	0.005376
3641.250000	0.005359
3641.000000	0.005340
3640.750000	0.005322
3640.500000	0.005308
3640.250000	0.005299
3640.000000	0.005298
3639.750000	0.005306
3639.500000	0.005324
3639.250000	0.005352
3639.000000	0.005391
3638.750000	0.005437
3638.500000	0.005485
3638.250000	0.005529
3638.000000	0.005566
3637.750000	0.005590
3637.500000	0.005601
3637.250000	0.005600
3637.000000	0.005591
3636.750000	0.005578
3636.500000	0.005564
3636.250000	0.005553
3636.000000	0.005548

3635.750000	0.005550
3635.500000	0.005563
3635.250000	0.005589
3635.000000	0.005629
3634.750000	0.005683
3634.500000	0.005745
3634.250000	0.005808
3634.000000	0.005859
3633.750000	0.005892
3633.500000	0.005901
3633.250000	0.005888
3633.000000	0.005861
3632.750000	0.005826
3632.500000	0.005789
3632.250000	0.005754
3632.000000	0.005721
3631.750000	0.005687
3631.500000	0.005648
3631.250000	0.005599
3631.000000	0.005541
3630.750000	0.005480
3630.500000	0.005429
3630.250000	0.005407
3630.000000	0.005435
3629.750000	0.005521
3629.500000	0.005649

3629.250000	0.005781
3629.000000	0.005870
3628.750000	0.005891
3628.500000	0.005854
3628.250000	0.005793
3628.000000	0.005742
3627.750000	0.005728
3627.500000	0.005761
3627.250000	0.005842
3627.000000	0.005960
3626.750000	0.006095
3626.500000	0.006226
3626.250000	0.006336
3626.000000	0.006414
3625.750000	0.006457
3625.500000	0.006469
3625.250000	0.006461
3625.000000	0.006441
3624.750000	0.006418
3624.500000	0.006398
3624.250000	0.006384
3624.000000	0.006377
3623.750000	0.006375
3623.500000	0.006374
3623.250000	0.006372
3623.000000	0.006366

3622.750000	0.006357
3622.500000	0.006345
3622.250000	0.006333
3622.000000	0.006324
3621.750000	0.006319
3621.500000	0.006319
3621.250000	0.006327
3621.000000	0.006344
3620.750000	0.006374
3620.500000	0.006417
3620.250000	0.006470
3620.000000	0.006524
3619.750000	0.006568
3619.500000	0.006598
3619.250000	0.006616
3619.000000	0.006631
3618.750000	0.006655
3618.500000	0.006695
3618.250000	0.006750
3618.000000	0.006810
3617.750000	0.006860
3617.500000	0.006888
3617.250000	0.006888
3617.000000	0.006864
3616.750000	0.006830
3616.500000	0.006800

3616.250000	0.006790
3616.000000	0.006808
3615.750000	0.006852
3615.500000	0.006908
3615.250000	0.006955
3615.000000	0.006975
3614.750000	0.006959
3614.500000	0.006911
3614.250000	0.006848
3614.000000	0.006795
3613.750000	0.006778
3613.500000	0.006813
3613.250000	0.006899
3613.000000	0.007014
3612.750000	0.007123
3612.500000	0.007193
3612.250000	0.007206
3612.000000	0.007162
3611.750000	0.007076
3611.500000	0.006971
3611.250000	0.006870
3611.000000	0.006794
3610.750000	0.006759
3610.500000	0.006775
3610.250000	0.006842
3610.000000	0.006946

3609.750000	0.007063
3609.500000	0.007163
3609.250000	0.007226
3609.000000	0.007247
3608.750000	0.007235
3608.500000	0.007211
3608.250000	0.007196
3608.000000	0.007207
3607.750000	0.007251
3607.500000	0.007318
3607.250000	0.007393
3607.000000	0.007454
3606.750000	0.007485
3606.500000	0.007480
3606.250000	0.007447
3606.000000	0.007400
3605.750000	0.007353
3605.500000	0.007319
3605.250000	0.007305
3605.000000	0.007312
3604.750000	0.007336
3604.500000	0.007370
3604.250000	0.007406
3604.000000	0.007441
3603.750000	0.007468
3603.500000	0.007485

3603.250000	0.007492
3603.000000	0.007491
3602.750000	0.007484
3602.500000	0.007476
3602.250000	0.007474
3602.000000	0.007482
3601.750000	0.007504
3601.500000	0.007538
3601.250000	0.007580
3601.000000	0.007624
3600.750000	0.007664
3600.500000	0.007696
3600.250000	0.007721
3600.000000	0.007742
3599.750000	0.007762
3599.500000	0.007784
3599.250000	0.007807
3599.000000	0.007827
3598.750000	0.007839
3598.500000	0.007838
3598.250000	0.007822
3598.000000	0.007791
3597.750000	0.007753
3597.500000	0.007716
3597.250000	0.007690
3597.000000	0.007682

3596.750000	0.007697
3596.500000	0.007736
3596.250000	0.007791
3596.000000	0.007849
3595.750000	0.007896
3595.500000	0.007920
3595.250000	0.007918
3595.000000	0.007894
3594.750000	0.007862
3594.500000	0.007838
3594.250000	0.007837
3594.000000	0.007867
3593.750000	0.007924
3593.500000	0.007995
3593.250000	0.008061
3593.000000	0.008104
3592.750000	0.008114
3592.500000	0.008091
3592.250000	0.008042
3592.000000	0.007981
3591.750000	0.007919
3591.500000	0.007865
3591.250000	0.007823
3591.000000	0.007792
3590.750000	0.007772
3590.500000	0.007761

3590.250000	0.007763
3590.000000	0.007784
3589.750000	0.007828
3589.500000	0.007896
3589.250000	0.007979
3589.000000	0.008064
3588.750000	0.008131
3588.500000	0.008171
3588.250000	0.008183
3588.000000	0.008179
3587.750000	0.008176
3587.500000	0.008188
3587.250000	0.008219
3587.000000	0.008259
3586.750000	0.008291
3586.500000	0.008299
3586.250000	0.008275
3586.000000	0.008224
3585.750000	0.008159
3585.500000	0.008098
3585.250000	0.008056
3585.000000	0.008043
3584.750000	0.008061
3584.500000	0.008105
3584.250000	0.008164
3584.000000	0.008226

3583.750000	0.008280
3583.500000	0.008321
3583.250000	0.008344
3583.000000	0.008349
3582.750000	0.008340
3582.500000	0.008320
3582.250000	0.008295
3582.000000	0.008270
3581.750000	0.008252
3581.500000	0.008247
3581.250000	0.008256
3581.000000	0.008279
3580.750000	0.008312
3580.500000	0.008348
3580.250000	0.008378
3580.000000	0.008399
3579.750000	0.008405
3579.500000	0.008398
3579.250000	0.008384
3579.000000	0.008367
3578.750000	0.008354
3578.500000	0.008351
3578.250000	0.008361
3578.000000	0.008383
3577.750000	0.008415
3577.500000	0.008452

3577.250000	0.008488
3577.000000	0.008519
3576.750000	0.008542
3576.500000	0.008557
3576.250000	0.008568
3576.000000	0.008578
3575.750000	0.008590
3575.500000	0.008607
3575.250000	0.008628
3575.000000	0.008652
3574.750000	0.008675
3574.500000	0.008692
3574.250000	0.008700
3574.000000	0.008698
3573.750000	0.008686
3573.500000	0.008669
3573.250000	0.008648
3573.000000	0.008629
3572.750000	0.008615
3572.500000	0.008609
3572.250000	0.008613
3572.000000	0.008626
3571.750000	0.008646
3571.500000	0.008669
3571.250000	0.008689
3571.000000	0.008701

3570.750000	0.008702
3570.500000	0.008692
3570.250000	0.008675
3570.000000	0.008660
3569.750000	0.008658
3569.500000	0.008678
3569.250000	0.008727
3569.000000	0.008805
3568.750000	0.008903
3568.500000	0.009004
3568.250000	0.009090
3568.000000	0.009151
3567.750000	0.009187
3567.500000	0.009208
3567.250000	0.009228
3567.000000	0.009259
3566.750000	0.009300
3566.500000	0.009341
3566.250000	0.009369
3566.000000	0.009371
3565.750000	0.009344
3565.500000	0.009292
3565.250000	0.009230
3565.000000	0.009172
3564.750000	0.009134
3564.500000	0.009122

3564.250000	0.009136
3564.000000	0.009169
3563.750000	0.009207
3563.500000	0.009237
3563.250000	0.009250
3563.000000	0.009244
3562.750000	0.009220
3562.500000	0.009184
3562.250000	0.009145
3562.000000	0.009111
3561.750000	0.009089
3561.500000	0.009083
3561.250000	0.009095
3561.000000	0.009123
3560.750000	0.009159
3560.500000	0.009194
3560.250000	0.009218
3560.000000	0.009225
3559.750000	0.009212
3559.500000	0.009184
3559.250000	0.009149
3559.000000	0.009116
3558.750000	0.009094
3558.500000	0.009089
3558.250000	0.009103
3558.000000	0.009132

3557.750000	0.009170
3557.500000	0.009209
3557.250000	0.009243
3557.000000	0.009268
3556.750000	0.009283
3556.500000	0.009289
3556.250000	0.009287
3556.000000	0.009282
3555.750000	0.009276
3555.500000	0.009273
3555.250000	0.009274
3555.000000	0.009283
3554.750000	0.009299
3554.500000	0.009322
3554.250000	0.009350
3554.000000	0.009380
3553.750000	0.009411
3553.500000	0.009443
3553.250000	0.009476
3553.000000	0.009511
3552.750000	0.009548
3552.500000	0.009584
3552.250000	0.009611
3552.000000	0.009626
3551.750000	0.009622
3551.500000	0.009601

3551.250000	0.009568
3551.000000	0.009529
3550.750000	0.009494
3550.500000	0.009471
3550.250000	0.009462
3550.000000	0.009466
3549.750000	0.009479
3549.500000	0.009497
3549.250000	0.009515
3549.000000	0.009536
3548.750000	0.009560
3548.500000	0.009593
3548.250000	0.009637
3548.000000	0.009690
3547.750000	0.009746
3547.500000	0.009797
3547.250000	0.009836
3547.000000	0.009859
3546.750000	0.009868
3546.500000	0.009868
3546.250000	0.009868
3546.000000	0.009872
3545.750000	0.009881
3545.500000	0.009893
3545.250000	0.009903
3545.000000	0.009903

3544.750000	0.009894
3544.500000	0.009875
3544.250000	0.009852
3544.000000	0.009831
3543.750000	0.009815
3543.500000	0.009806
3543.250000	0.009802
3543.000000	0.009800
3542.750000	0.009798
3542.500000	0.009794
3542.250000	0.009791
3542.000000	0.009790
3541.750000	0.009794
3541.500000	0.009805
3541.250000	0.009821
3541.000000	0.009842
3540.750000	0.009864
3540.500000	0.009886
3540.250000	0.009907
3540.000000	0.009925
3539.750000	0.009942
3539.500000	0.009958
3539.250000	0.009971
3539.000000	0.009982
3538.750000	0.009990
3538.500000	0.009995

3538.250000	0.010000
3538.000000	0.010007
3537.750000	0.010018
3537.500000	0.010034
3537.250000	0.010055
3537.000000	0.010078
3536.750000	0.010098
3536.500000	0.010113
3536.250000	0.010118
3536.000000	0.010115
3535.750000	0.010106
3535.500000	0.010095
3535.250000	0.010087
3535.000000	0.010085
3534.750000	0.010091
3534.500000	0.010104
3534.250000	0.010120
3534.000000	0.010134
3533.750000	0.010144
3533.500000	0.010148
3533.250000	0.010147
3533.000000	0.010145
3532.750000	0.010147
3532.500000	0.010157
3532.250000	0.010177
3532.000000	0.010206

3531.750000	0.010240
3531.500000	0.010272
3531.250000	0.010296
3531.000000	0.010310
3530.750000	0.010312
3530.500000	0.010306
3530.250000	0.010297
3530.000000	0.010293
3529.750000	0.010298
3529.500000	0.010315
3529.250000	0.010341
3529.000000	0.010372
3528.750000	0.010404
3528.500000	0.010432
3528.250000	0.010452
3528.000000	0.010465
3527.750000	0.010472
3527.500000	0.010476
3527.250000	0.010479
3527.000000	0.010483
3526.750000	0.010488
3526.500000	0.010496
3526.250000	0.010504
3526.000000	0.010513
3525.750000	0.010520
3525.500000	0.010527

3525.250000	0.010534
3525.000000	0.010543
3524.750000	0.010556
3524.500000	0.010574
3524.250000	0.010598
3524.000000	0.010628
3523.750000	0.010661
3523.500000	0.010692
3523.250000	0.010718
3523.000000	0.010732
3522.750000	0.010734
3522.500000	0.010722
3522.250000	0.010702
3522.000000	0.010675
3521.750000	0.010649
3521.500000	0.010627
3521.250000	0.010611
3521.000000	0.010603
3520.750000	0.010601
3520.500000	0.010604
3520.250000	0.010609
3520.000000	0.010616
3519.750000	0.010624
3519.500000	0.010632
3519.250000	0.010639
3519.000000	0.010646

3518.750000	0.010652
3518.500000	0.010657
3518.250000	0.010661
3518.000000	0.010664
3517.750000	0.010667
3517.500000	0.010670
3517.250000	0.010674
3517.000000	0.010678
3516.750000	0.010682
3516.500000	0.010686
3516.250000	0.010691
3516.000000	0.010697
3515.750000	0.010704
3515.500000	0.010711
3515.250000	0.010719
3515.000000	0.010728
3514.750000	0.010735
3514.500000	0.010741
3514.250000	0.010746
3514.000000	0.010750
3513.750000	0.010755
3513.500000	0.010763
3513.250000	0.010777
3513.000000	0.010796
3512.750000	0.010819
3512.500000	0.010844

3512.250000	0.010866
3512.000000	0.010882
3511.750000	0.010891
3511.500000	0.010895
3511.250000	0.010897
3511.000000	0.010904
3510.750000	0.010921
3510.500000	0.010952
3510.250000	0.010999
3510.000000	0.011054
3509.750000	0.011109
3509.500000	0.011152
3509.250000	0.011174
3509.000000	0.011171
3508.750000	0.011146
3508.500000	0.011104
3508.250000	0.011056
3508.000000	0.011011
3507.750000	0.010977
3507.500000	0.010957
3507.250000	0.010953
3507.000000	0.010961
3506.750000	0.010977
3506.500000	0.010999
3506.250000	0.011024
3506.000000	0.011051

3505.750000	0.011078
3505.500000	0.011105
3505.250000	0.011132
3505.000000	0.011158
3504.750000	0.011182
3504.500000	0.011205
3504.250000	0.011228
3504.000000	0.011252
3503.750000	0.011276
3503.500000	0.011301
3503.250000	0.011323
3503.000000	0.011340
3502.750000	0.011351
3502.500000	0.011353
3502.250000	0.011346
3502.000000	0.011331
3501.750000	0.011308
3501.500000	0.011280
3501.250000	0.011250
3501.000000	0.011220
3500.750000	0.011193
3500.500000	0.011171
3500.250000	0.011158
3500.000000	0.011153
3499.750000	0.011156
3499.500000	0.011166

3499.250000	0.011178
3499.000000	0.011191
3498.750000	0.011201
3498.500000	0.011210
3498.250000	0.011219
3498.000000	0.011231
3497.750000	0.011247
3497.500000	0.011268
3497.250000	0.011293
3497.000000	0.011317
3496.750000	0.011337
3496.500000	0.011348
3496.250000	0.011349
3496.000000	0.011342
3495.750000	0.011329
3495.500000	0.011314
3495.250000	0.011302
3495.000000	0.011294
3494.750000	0.011293
3494.500000	0.011296
3494.250000	0.011304
3494.000000	0.011314
3493.750000	0.011325
3493.500000	0.011336
3493.250000	0.011348
3493.000000	0.011359

3492.750000	0.011369
3492.500000	0.011377
3492.250000	0.011384
3492.000000	0.011389
3491.750000	0.011391
3491.500000	0.011392
3491.250000	0.011392
3491.000000	0.011392
3490.750000	0.011395
3490.500000	0.011403
3490.250000	0.011418
3490.000000	0.011441
3489.750000	0.011472
3489.500000	0.011509
3489.250000	0.011548
3489.000000	0.011585
3488.750000	0.011615
3488.500000	0.011637
3488.250000	0.011648
3488.000000	0.011650
3487.750000	0.011646
3487.500000	0.011637
3487.250000	0.011625
3487.000000	0.011614
3486.750000	0.011602
3486.500000	0.011593

3486.250000	0.011585
3486.000000	0.011581
3485.750000	0.011582
3485.500000	0.011586
3485.250000	0.011595
3485.000000	0.011607
3484.750000	0.011620
3484.500000	0.011634
3484.250000	0.011649
3484.000000	0.011667
3483.750000	0.011688
3483.500000	0.011714
3483.250000	0.011744
3483.000000	0.011775
3482.750000	0.011804
3482.500000	0.011825
3482.250000	0.011836
3482.000000	0.011834
3481.750000	0.011820
3481.500000	0.011799
3481.250000	0.011775
3481.000000	0.011752
3480.750000	0.011734
3480.500000	0.011722
3480.250000	0.011719
3480.000000	0.011723

3479.750000	0.011735
3479.500000	0.011752
3479.250000	0.011773
3479.000000	0.011795
3478.750000	0.011817
3478.500000	0.011835
3478.250000	0.011850
3478.000000	0.011861
3477.750000	0.011869
3477.500000	0.011876
3477.250000	0.011884
3477.000000	0.011897
3476.750000	0.011914
3476.500000	0.011938
3476.250000	0.011965
3476.000000	0.011996
3475.750000	0.012027
3475.500000	0.012055
3475.250000	0.012079
3475.000000	0.012096
3474.750000	0.012105
3474.500000	0.012107
3474.250000	0.012104
3474.000000	0.012099
3473.750000	0.012094
3473.500000	0.012093

3473.250000	0.012096
3473.000000	0.012104
3472.750000	0.012113
3472.500000	0.012122
3472.250000	0.012128
3472.000000	0.012131
3471.750000	0.012132
3471.500000	0.012132
3471.250000	0.012135
3471.000000	0.012143
3470.750000	0.012156
3470.500000	0.012174
3470.250000	0.012194
3470.000000	0.012214
3469.750000	0.012232
3469.500000	0.012247
3469.250000	0.012260
3469.000000	0.012273
3468.750000	0.012286
3468.500000	0.012301
3468.250000	0.012317
3468.000000	0.012336
3467.750000	0.012357
3467.500000	0.012379
3467.250000	0.012404
3467.000000	0.012429

3466.750000	0.012456
3466.500000	0.012484
3466.250000	0.012510
3466.000000	0.012534
3465.750000	0.012555
3465.500000	0.012573
3465.250000	0.012589
3465.000000	0.012605
3464.750000	0.012621
3464.500000	0.012640
3464.250000	0.012663
3464.000000	0.012689
3463.750000	0.012719
3463.500000	0.012750
3463.250000	0.012781
3463.000000	0.012813
3462.750000	0.012842
3462.500000	0.012868
3462.250000	0.012889
3462.000000	0.012904
3461.750000	0.012913
3461.500000	0.012917
3461.250000	0.012917
3461.000000	0.012916
3460.750000	0.012917
3460.500000	0.012923

3460.250000	0.012934
3460.000000	0.012952
3459.750000	0.012975
3459.500000	0.013001
3459.250000	0.013028
3459.000000	0.013054
3458.750000	0.013078
3458.500000	0.013101
3458.250000	0.013122
3458.000000	0.013143
3457.750000	0.013163
3457.500000	0.013182
3457.250000	0.013199
3457.000000	0.013215
3456.750000	0.013229
3456.500000	0.013242
3456.250000	0.013254
3456.000000	0.013267
3455.750000	0.013282
3455.500000	0.013298
3455.250000	0.013317
3455.000000	0.013338
3454.750000	0.013360
3454.500000	0.013382
3454.250000	0.013402
3454.000000	0.013419

3453.750000	0.013434
3453.500000	0.013446
3453.250000	0.013458
3453.000000	0.013471
3452.750000	0.013490
3452.500000	0.013514
3452.250000	0.013543
3452.000000	0.013574
3451.750000	0.013606
3451.500000	0.013633
3451.250000	0.013654
3451.000000	0.013667
3450.750000	0.013673
3450.500000	0.013673
3450.250000	0.013671
3450.000000	0.013668
3449.750000	0.013666
3449.500000	0.013668
3449.250000	0.013675
3449.000000	0.013692
3448.750000	0.013721
3448.500000	0.013766
3448.250000	0.013828
3448.000000	0.013904
3447.750000	0.013985
3447.500000	0.014062

3447.250000	0.014122
3447.000000	0.014159
3446.750000	0.014170
3446.500000	0.014162
3446.250000	0.014143
3446.000000	0.014125
3445.750000	0.014116
3445.500000	0.014121
3445.250000	0.014139
3445.000000	0.014164
3444.750000	0.014190
3444.500000	0.014213
3444.250000	0.014231
3444.000000	0.014246
3443.750000	0.014263
3443.500000	0.014286
3443.250000	0.014317
3443.000000	0.014353
3442.750000	0.014390
3442.500000	0.014423
3442.250000	0.014447
3442.000000	0.014459
3441.750000	0.014462
3441.500000	0.014459
3441.250000	0.014453
3441.000000	0.014450

3440.750000	0.014453
3440.500000	0.014462
3440.250000	0.014478
3440.000000	0.014499
3439.750000	0.014523
3439.500000	0.014551
3439.250000	0.014580
3439.000000	0.014610
3438.750000	0.014639
3438.500000	0.014667
3438.250000	0.014691
3438.000000	0.014712
3437.750000	0.014728
3437.500000	0.014740
3437.250000	0.014748
3437.000000	0.014751
3436.750000	0.014751
3436.500000	0.014749
3436.250000	0.014746
3436.000000	0.014744
3435.750000	0.014748
3435.500000	0.014758
3435.250000	0.014779
3435.000000	0.014811
3434.750000	0.014855
3434.500000	0.014909

3434.250000	0.014969
3434.000000	0.015032
3433.750000	0.015093
3433.500000	0.015148
3433.250000	0.015193
3433.000000	0.015227
3432.750000	0.015252
3432.500000	0.015269
3432.250000	0.015282
3432.000000	0.015295
3431.750000	0.015311
3431.500000	0.015332
3431.250000	0.015358
3431.000000	0.015387
3430.750000	0.015416
3430.500000	0.015445
3430.250000	0.015473
3430.000000	0.015500
3429.750000	0.015527
3429.500000	0.015557
3429.250000	0.015590
3429.000000	0.015626
3428.750000	0.015666
3428.500000	0.015709
3428.250000	0.015754
3428.000000	0.015801

3427.750000	0.015851
3427.500000	0.015901
3427.250000	0.015951
3427.000000	0.016000
3426.750000	0.016044
3426.500000	0.016083
3426.250000	0.016117
3426.000000	0.016147
3425.750000	0.016175
3425.500000	0.016204
3425.250000	0.016236
3425.000000	0.016272
3424.750000	0.016310
3424.500000	0.016350
3424.250000	0.016388
3424.000000	0.016423
3423.750000	0.016453
3423.500000	0.016477
3423.250000	0.016496
3423.000000	0.016511
3422.750000	0.016524
3422.500000	0.016539
3422.250000	0.016558
3422.000000	0.016583
3421.750000	0.016618
3421.500000	0.016663

3421.250000	0.016718
3421.000000	0.016779
3420.750000	0.016843
3420.500000	0.016905
3420.250000	0.016963
3420.000000	0.017012
3419.750000	0.017054
3419.500000	0.017087
3419.250000	0.017115
3419.000000	0.017139
3418.750000	0.017160
3418.500000	0.017181
3418.250000	0.017202
3418.000000	0.017225
3417.750000	0.017251
3417.500000	0.017280
3417.250000	0.017313
3417.000000	0.017349
3416.750000	0.017388
3416.500000	0.017428
3416.250000	0.017469
3416.000000	0.017508
3415.750000	0.017546
3415.500000	0.017582
3415.250000	0.017615
3415.000000	0.017645

3414.750000	0.017672
3414.500000	0.017696
3414.250000	0.017717
3414.000000	0.017739
3413.750000	0.017763
3413.500000	0.017793
3413.250000	0.017832
3413.000000	0.017880
3412.750000	0.017936
3412.500000	0.017997
3412.250000	0.018060
3412.000000	0.018119
3411.750000	0.018170
3411.500000	0.018212
3411.250000	0.018245
3411.000000	0.018269
3410.750000	0.018290
3410.500000	0.018310
3410.250000	0.018335
3410.000000	0.018369
3409.750000	0.018413
3409.500000	0.018467
3409.250000	0.018531
3409.000000	0.018601
3408.750000	0.018673
3408.500000	0.018743

3408.250000	0.018808
3408.000000	0.018865
3407.750000	0.018915
3407.500000	0.018960
3407.250000	0.019001
3407.000000	0.019041
3406.750000	0.019080
3406.500000	0.019119
3406.250000	0.019158
3406.000000	0.019197
3405.750000	0.019234
3405.500000	0.019271
3405.250000	0.019306
3405.000000	0.019341
3404.750000	0.019374
3404.500000	0.019407
3404.250000	0.019438
3404.000000	0.019470
3403.750000	0.019501
3403.500000	0.019534
3403.250000	0.019571
3403.000000	0.019611
3402.750000	0.019655
3402.500000	0.019703
3402.250000	0.019753
3402.000000	0.019803

3401.750000	0.019851
3401.500000	0.019898
3401.250000	0.019942
3401.000000	0.019984
3400.750000	0.020024
3400.500000	0.020064
3400.250000	0.020105
3400.000000	0.020150
3399.750000	0.020199
3399.500000	0.020256
3399.250000	0.020322
3399.000000	0.020397
3398.750000	0.020480
3398.500000	0.020570
3398.250000	0.020661
3398.000000	0.020748
3397.750000	0.020827
3397.500000	0.020892
3397.250000	0.020940
3397.000000	0.020970
3396.750000	0.020985
3396.500000	0.020987
3396.250000	0.020981
3396.000000	0.020971
3395.750000	0.020962
3395.500000	0.020957

3395.250000	0.020960
3395.000000	0.020972
3394.750000	0.020996
3394.500000	0.021034
3394.250000	0.021085
3394.000000	0.021148
3393.750000	0.021217
3393.500000	0.021286
3393.250000	0.021348
3393.000000	0.021395
3392.750000	0.021423
3392.500000	0.021434
3392.250000	0.021432
3392.000000	0.021425
3391.750000	0.021422
3391.500000	0.021429
3391.250000	0.021449
3391.000000	0.021481
3390.750000	0.021521
3390.500000	0.021561
3390.250000	0.021596
3390.000000	0.021623
3389.750000	0.021641
3389.500000	0.021654
3389.250000	0.021665
3389.000000	0.021680

3388.750000	0.021700
3388.500000	0.021728
3388.250000	0.021760
3388.000000	0.021794
3387.750000	0.021827
3387.500000	0.021855
3387.250000	0.021877
3387.000000	0.021894
3386.750000	0.021906
3386.500000	0.021915
3386.250000	0.021924
3386.000000	0.021936
3385.750000	0.021950
3385.500000	0.021968
3385.250000	0.021988
3385.000000	0.022009
3384.750000	0.022028
3384.500000	0.022043
3384.250000	0.022053
3384.000000	0.022058
3383.750000	0.022056
3383.500000	0.022048
3383.250000	0.022036
3383.000000	0.022021
3382.750000	0.022006
3382.500000	0.021992

3382.250000	0.021982
3382.000000	0.021978
3381.750000	0.021981
3381.500000	0.021989
3381.250000	0.022002
3381.000000	0.022017
3380.750000	0.022029
3380.500000	0.022036
3380.250000	0.022037
3380.000000	0.022032
3379.750000	0.022025
3379.500000	0.022016
3379.250000	0.022008
3379.000000	0.022003
3378.750000	0.021999
3378.500000	0.021996
3378.250000	0.021993
3378.000000	0.021988
3377.750000	0.021982
3377.500000	0.021976
3377.250000	0.021972
3377.000000	0.021970
3376.750000	0.021969
3376.500000	0.021969
3376.250000	0.021968
3376.000000	0.021968

3375.750000	0.021969
3375.500000	0.021975
3375.250000	0.021987
3375.000000	0.022006
3374.750000	0.022031
3374.500000	0.022058
3374.250000	0.022082
3374.000000	0.022098
3373.750000	0.022102
3373.500000	0.022092
3373.250000	0.022071
3373.000000	0.022042
3372.750000	0.022008
3372.500000	0.021973
3372.250000	0.021938
3372.000000	0.021905
3371.750000	0.021873
3371.500000	0.021843
3371.250000	0.021814
3371.000000	0.021788
3370.750000	0.021766
3370.500000	0.021747
3370.250000	0.021733
3370.000000	0.021722
3369.750000	0.021713
3369.500000	0.021705

3369.250000	0.021696
3369.000000	0.021686
3368.750000	0.021676
3368.500000	0.021663
3368.250000	0.021649
3368.000000	0.021632
3367.750000	0.021613
3367.500000	0.021592
3367.250000	0.021572
3367.000000	0.021556
3366.750000	0.021548
3366.500000	0.021549
3366.250000	0.021559
3366.000000	0.021577
3365.750000	0.021597
3365.500000	0.021613
3365.250000	0.021621
3365.000000	0.021618
3364.750000	0.021604
3364.500000	0.021581
3364.250000	0.021553
3364.000000	0.021524
3363.750000	0.021499
3363.500000	0.021480
3363.250000	0.021466
3363.000000	0.021457

3362.750000	0.021453
3362.500000	0.021450
3362.250000	0.021447
3362.000000	0.021444
3361.750000	0.021437
3361.500000	0.021427
3361.250000	0.021414
3361.000000	0.021397
3360.750000	0.021377
3360.500000	0.021355
3360.250000	0.021333
3360.000000	0.021311
3359.750000	0.021291
3359.500000	0.021273
3359.250000	0.021255
3359.000000	0.021239
3358.750000	0.021225
3358.500000	0.021213
3358.250000	0.021204
3358.000000	0.021198
3357.750000	0.021195
3357.500000	0.021194
3357.250000	0.021192
3357.000000	0.021186
3356.750000	0.021174
3356.500000	0.021156

3356.250000	0.021133
3356.000000	0.021106
3355.750000	0.021078
3355.500000	0.021050
3355.250000	0.021024
3355.000000	0.020999
3354.750000	0.020973
3354.500000	0.020944
3354.250000	0.020910
3354.000000	0.020872
3353.750000	0.020831
3353.500000	0.020790
3353.250000	0.020751
3353.000000	0.020718
3352.750000	0.020692
3352.500000	0.020673
3352.250000	0.020662
3352.000000	0.020655
3351.750000	0.020652
3351.500000	0.020650
3351.250000	0.020646
3351.000000	0.020640
3350.750000	0.020630
3350.500000	0.020615
3350.250000	0.020598
3350.000000	0.020579

3349.750000	0.020560
3349.500000	0.020544
3349.250000	0.020532
3349.000000	0.020524
3348.750000	0.020520
3348.500000	0.020517
3348.250000	0.020514
3348.000000	0.020509
3347.750000	0.020501
3347.500000	0.020489
3347.250000	0.020475
3347.000000	0.020459
3346.750000	0.020442
3346.500000	0.020426
3346.250000	0.020409
3346.000000	0.020392
3345.750000	0.020374
3345.500000	0.020355
3345.250000	0.020335
3345.000000	0.020315
3344.750000	0.020295
3344.500000	0.020276
3344.250000	0.020258
3344.000000	0.020243
3343.750000	0.020228
3343.500000	0.020213

3343.250000	0.020196
3343.000000	0.020178
3342.750000	0.020158
3342.500000	0.020137
3342.250000	0.020115
3342.000000	0.020094
3341.750000	0.020075
3341.500000	0.020057
3341.250000	0.020041
3341.000000	0.020026
3340.750000	0.020011
3340.500000	0.019995
3340.250000	0.019980
3340.000000	0.019964
3339.750000	0.019947
3339.500000	0.019930
3339.250000	0.019910
3339.000000	0.019889
3338.750000	0.019865
3338.500000	0.019839
3338.250000	0.019814
3338.000000	0.019790
3337.750000	0.019768
3337.500000	0.019749
3337.250000	0.019732
3337.000000	0.019716

3336.750000	0.019697
3336.500000	0.019676
3336.250000	0.019651
3336.000000	0.019625
3335.750000	0.019598
3335.500000	0.019574
3335.250000	0.019552
3335.000000	0.019532
3334.750000	0.019511
3334.500000	0.019489
3334.250000	0.019462
3334.000000	0.019431
3333.750000	0.019397
3333.500000	0.019361
3333.250000	0.019327
3333.000000	0.019295
3332.750000	0.019266
3332.500000	0.019239
3332.250000	0.019214
3332.000000	0.019189
3331.750000	0.019164
3331.500000	0.019138
3331.250000	0.019112
3331.000000	0.019086
3330.750000	0.019060
3330.500000	0.019034

3330.250000	0.019007
3330.000000	0.018980
3329.750000	0.018952
3329.500000	0.018924
3329.250000	0.018898
3329.000000	0.018872
3328.750000	0.018848
3328.500000	0.018825
3328.250000	0.018801
3328.000000	0.018777
3327.750000	0.018750
3327.500000	0.018721
3327.250000	0.018690
3327.000000	0.018656
3326.750000	0.018620
3326.500000	0.018581
3326.250000	0.018540
3326.000000	0.018500
3325.750000	0.018462
3325.500000	0.018427
3325.250000	0.018399
3325.000000	0.018376
3324.750000	0.018360
3324.500000	0.018346
3324.250000	0.018334
3324.000000	0.018319

3323.750000	0.018301
3323.500000	0.018279
3323.250000	0.018252
3323.000000	0.018221
3322.750000	0.018187
3322.500000	0.018150
3322.250000	0.018111
3322.000000	0.018070
3321.750000	0.018028
3321.500000	0.017985
3321.250000	0.017945
3321.000000	0.017908
3320.750000	0.017876
3320.500000	0.017848
3320.250000	0.017823
3320.000000	0.017799
3319.750000	0.017773
3319.500000	0.017744
3319.250000	0.017711
3319.000000	0.017675
3318.750000	0.017638
3318.500000	0.017601
3318.250000	0.017566
3318.000000	0.017533
3317.750000	0.017503
3317.500000	0.017475

3317.250000	0.017449
3317.000000	0.017422
3316.750000	0.017395
3316.500000	0.017368
3316.250000	0.017339
3316.000000	0.017310
3315.750000	0.017279
3315.500000	0.017247
3315.250000	0.017214
3315.000000	0.017181
3314.750000	0.017148
3314.500000	0.017118
3314.250000	0.017091
3314.000000	0.017067
3313.750000	0.017046
3313.500000	0.017027
3313.250000	0.017005
3313.000000	0.016980
3312.750000	0.016949
3312.500000	0.016911
3312.250000	0.016866
3312.000000	0.016817
3311.750000	0.016765
3311.500000	0.016713
3311.250000	0.016663
3311.000000	0.016618

3310.750000	0.016577
3310.500000	0.016541
3310.250000	0.016508
3310.000000	0.016479
3309.750000	0.016451
3309.500000	0.016423
3309.250000	0.016394
3309.000000	0.016362
3308.750000	0.016326
3308.500000	0.016287
3308.250000	0.016246
3308.000000	0.016202
3307.750000	0.016158
3307.500000	0.016115
3307.250000	0.016074
3307.000000	0.016035
3306.750000	0.015998
3306.500000	0.015962
3306.250000	0.015927
3306.000000	0.015894
3305.750000	0.015863
3305.500000	0.015834
3305.250000	0.015807
3305.000000	0.015780
3304.750000	0.015753
3304.500000	0.015722

3304.250000	0.015688
3304.000000	0.015649
3303.750000	0.015608
3303.500000	0.015566
3303.250000	0.015525
3303.000000	0.015488
3302.750000	0.015456
3302.500000	0.015427
3302.250000	0.015403
3302.000000	0.015380
3301.750000	0.015357
3301.500000	0.015334
3301.250000	0.015310
3301.000000	0.015284
3300.750000	0.015255
3300.500000	0.015223
3300.250000	0.015187
3300.000000	0.015147
3299.750000	0.015106
3299.500000	0.015064
3299.250000	0.015023
3299.000000	0.014987
3298.750000	0.014954
3298.500000	0.014927
3298.250000	0.014902
3298.000000	0.014878

3297.750000	0.014852
3297.500000	0.014824
3297.250000	0.014792
3297.000000	0.014756
3296.750000	0.014717
3296.500000	0.014677
3296.250000	0.014635
3296.000000	0.014594
3295.750000	0.014554
3295.500000	0.014516
3295.250000	0.014481
3295.000000	0.014451
3294.750000	0.014425
3294.500000	0.014402
3294.250000	0.014381
3294.000000	0.014360
3293.750000	0.014335
3293.500000	0.014304
3293.250000	0.014266
3293.000000	0.014222
3292.750000	0.014174
3292.500000	0.014127
3292.250000	0.014085
3292.000000	0.014050
3291.750000	0.014026
3291.500000	0.014009

3291.250000	0.013998
3291.000000	0.013986
3290.750000	0.013969
3290.500000	0.013943
3290.250000	0.013908
3290.000000	0.013864
3289.750000	0.013816
3289.500000	0.013767
3289.250000	0.013722
3289.000000	0.013683
3288.750000	0.013651
3288.500000	0.013624
3288.250000	0.013601
3288.000000	0.013581
3287.750000	0.013561
3287.500000	0.013543
3287.250000	0.013524
3287.000000	0.013506
3286.750000	0.013488
3286.500000	0.013468
3286.250000	0.013446
3286.000000	0.013422
3285.750000	0.013395
3285.500000	0.013365
3285.250000	0.013332
3285.000000	0.013298

3284.750000	0.013263
3284.500000	0.013226
3284.250000	0.013188
3284.000000	0.013150
3283.750000	0.013114
3283.500000	0.013081
3283.250000	0.013052
3283.000000	0.013028
3282.750000	0.013007
3282.500000	0.012988
3282.250000	0.012969
3282.000000	0.012947
3281.750000	0.012921
3281.500000	0.012893
3281.250000	0.012864
3281.000000	0.012836
3280.750000	0.012810
3280.500000	0.012787
3280.250000	0.012766
3280.000000	0.012746
3279.750000	0.012726
3279.500000	0.012704
3279.250000	0.012680
3279.000000	0.012654
3278.750000	0.012629
3278.500000	0.012603

3278.250000	0.012579
3278.000000	0.012555
3277.750000	0.012532
3277.500000	0.012509
3277.250000	0.012485
3277.000000	0.012457
3276.750000	0.012425
3276.500000	0.012389
3276.250000	0.012349
3276.000000	0.012307
3275.750000	0.012264
3275.500000	0.012224
3275.250000	0.012190
3275.000000	0.012162
3274.750000	0.012142
3274.500000	0.012128
3274.250000	0.012116
3274.000000	0.012103
3273.750000	0.012086
3273.500000	0.012063
3273.250000	0.012035
3273.000000	0.012003
3272.750000	0.011968
3272.500000	0.011933
3272.250000	0.011898
3272.000000	0.011864

3271.750000	0.011830
3271.500000	0.011798
3271.250000	0.011769
3271.000000	0.011744
3270.750000	0.011726
3270.500000	0.011714
3270.250000	0.011709
3270.000000	0.011709
3269.750000	0.011712
3269.500000	0.011715
3269.250000	0.011716
3269.000000	0.011711
3268.750000	0.011701
3268.500000	0.011684
3268.250000	0.011662
3268.000000	0.011636
3267.750000	0.011606
3267.500000	0.011575
3267.250000	0.011544
3267.000000	0.011517
3266.750000	0.011494
3266.500000	0.011476
3266.250000	0.011462
3266.000000	0.011449
3265.750000	0.011435
3265.500000	0.011416

3265.250000	0.011390
3265.000000	0.011359
3264.750000	0.011324
3264.500000	0.011289
3264.250000	0.011256
3264.000000	0.011229
3263.750000	0.011208
3263.500000	0.011192
3263.250000	0.011179
3263.000000	0.011166
3262.750000	0.011151
3262.500000	0.011133
3262.250000	0.011112
3262.000000	0.011090
3261.750000	0.011071
3261.500000	0.011055
3261.250000	0.011044
3261.000000	0.011040
3260.750000	0.011041
3260.500000	0.011046
3260.250000	0.011050
3260.000000	0.011050
3259.750000	0.011044
3259.500000	0.011028
3259.250000	0.011002
3259.000000	0.010967

3258.750000	0.010926
3258.500000	0.010882
3258.250000	0.010841
3258.000000	0.010805
3257.750000	0.010777
3257.500000	0.010757
3257.250000	0.010743
3257.000000	0.010731
3256.750000	0.010720
3256.500000	0.010707
3256.250000	0.010693
3256.000000	0.010678
3255.750000	0.010666
3255.500000	0.010657
3255.250000	0.010652
3255.000000	0.010648
3254.750000	0.010642
3254.500000	0.010631
3254.250000	0.010612
3254.000000	0.010585
3253.750000	0.010550
3253.500000	0.010513
3253.250000	0.010477
3253.000000	0.010447
3252.750000	0.010426
3252.500000	0.010414

3252.250000	0.010410
3252.000000	0.010412
3251.750000	0.010417
3251.500000	0.010422
3251.250000	0.010425
3251.000000	0.010423
3250.750000	0.010418
3250.500000	0.010408
3250.250000	0.010394
3250.000000	0.010377
3249.750000	0.010358
3249.500000	0.010335
3249.250000	0.010308
3249.000000	0.010278
3248.750000	0.010242
3248.500000	0.010202
3248.250000	0.010158
3248.000000	0.010113
3247.750000	0.010071
3247.500000	0.010035
3247.250000	0.010009
3247.000000	0.009996
3246.750000	0.009997
3246.500000	0.010009
3246.250000	0.010031
3246.000000	0.010058

3245.750000	0.010085
3245.500000	0.010110
3245.250000	0.010127
3245.000000	0.010134
3244.750000	0.010130
3244.500000	0.010115
3244.250000	0.010089
3244.000000	0.010056
3243.750000	0.010020
3243.500000	0.009985
3243.250000	0.009956
3243.000000	0.009935
3242.750000	0.009923
3242.500000	0.009920
3242.250000	0.009922
3242.000000	0.009925
3241.750000	0.009927
3241.500000	0.009923
3241.250000	0.009914
3241.000000	0.009900
3240.750000	0.009882
3240.500000	0.009863
3240.250000	0.009842
3240.000000	0.009822
3239.750000	0.009801
3239.500000	0.009779

3239.250000	0.009756
3239.000000	0.009732
3238.750000	0.009708
3238.500000	0.009684
3238.250000	0.009663
3238.000000	0.009644
3237.750000	0.009628
3237.500000	0.009614
3237.250000	0.009602
3237.000000	0.009590
3236.750000	0.009576
3236.500000	0.009560
3236.250000	0.009541
3236.000000	0.009522
3235.750000	0.009502
3235.500000	0.009484
3235.250000	0.009469
3235.000000	0.009457
3234.750000	0.009449
3234.500000	0.009443
3234.250000	0.009440
3234.000000	0.009440
3233.750000	0.009443
3233.500000	0.009449
3233.250000	0.009459
3233.000000	0.009472

3232.750000	0.009487
3232.500000	0.009501
3232.250000	0.009510
3232.000000	0.009513
3231.750000	0.009506
3231.500000	0.009491
3231.250000	0.009469
3231.000000	0.009442
3230.750000	0.009413
3230.500000	0.009386
3230.250000	0.009361
3230.000000	0.009340
3229.750000	0.009322
3229.500000	0.009308
3229.250000	0.009295
3229.000000	0.009283
3228.750000	0.009272
3228.500000	0.009260
3228.250000	0.009248
3228.000000	0.009235
3227.750000	0.009222
3227.500000	0.009209
3227.250000	0.009198
3227.000000	0.009188
3226.750000	0.009180
3226.500000	0.009174

3226.250000	0.009170
3226.000000	0.009166
3225.750000	0.009163
3225.500000	0.009159
3225.250000	0.009154
3225.000000	0.009147
3224.750000	0.009139
3224.500000	0.009128
3224.250000	0.009116
3224.000000	0.009102
3223.750000	0.009087
3223.500000	0.009074
3223.250000	0.009062
3223.000000	0.009052
3222.750000	0.009044
3222.500000	0.009037
3222.250000	0.009029
3222.000000	0.009018
3221.750000	0.009002
3221.500000	0.008983
3221.250000	0.008962
3221.000000	0.008942
3220.750000	0.008925
3220.500000	0.008916
3220.250000	0.008914
3220.000000	0.008918

3219.750000	0.008927
3219.500000	0.008937
3219.250000	0.008944
3219.000000	0.008946
3218.750000	0.008941
3218.500000	0.008929
3218.250000	0.008912
3218.000000	0.008890
3217.750000	0.008866
3217.500000	0.008841
3217.250000	0.008816
3217.000000	0.008793
3216.750000	0.008772
3216.500000	0.008754
3216.250000	0.008741
3216.000000	0.008735
3215.750000	0.008735
3215.500000	0.008741
3215.250000	0.008753
3215.000000	0.008767
3214.750000	0.008780
3214.500000	0.008788
3214.250000	0.008790
3214.000000	0.008783
3213.750000	0.008769
3213.500000	0.008752

3213.250000	0.008734
3213.000000	0.008720
3212.750000	0.008709
3212.500000	0.008703
3212.250000	0.008698
3212.000000	0.008692
3211.750000	0.008683
3211.500000	0.008671
3211.250000	0.008655
3211.000000	0.008638
3210.750000	0.008624
3210.500000	0.008614
3210.250000	0.008608
3210.000000	0.008607
3209.750000	0.008610
3209.500000	0.008614
3209.250000	0.008618
3209.000000	0.008620
3208.750000	0.008621
3208.500000	0.008619
3208.250000	0.008613
3208.000000	0.008602
3207.750000	0.008586
3207.500000	0.008566
3207.250000	0.008543
3207.000000	0.008521

3206.750000	0.008502
3206.500000	0.008489
3206.250000	0.008483
3206.000000	0.008483
3205.750000	0.008486
3205.500000	0.008492
3205.250000	0.008496
3205.000000	0.008499
3204.750000	0.008500
3204.500000	0.008500
3204.250000	0.008498
3204.000000	0.008495
3203.750000	0.008490
3203.500000	0.008484
3203.250000	0.008476
3203.000000	0.008467
3202.750000	0.008458
3202.500000	0.008451
3202.250000	0.008445
3202.000000	0.008441
3201.750000	0.008440
3201.500000	0.008439
3201.250000	0.008439
3201.000000	0.008438
3200.750000	0.008438
3200.500000	0.008437

3200.250000	0.008435
3200.000000	0.008433
3199.750000	0.008429
3199.500000	0.008423
3199.250000	0.008414
3199.000000	0.008402
3198.750000	0.008388
3198.500000	0.008374
3198.250000	0.008359
3198.000000	0.008345
3197.750000	0.008332
3197.500000	0.008321
3197.250000	0.008311
3197.000000	0.008304
3196.750000	0.008298
3196.500000	0.008296
3196.250000	0.008296
3196.000000	0.008299
3195.750000	0.008303
3195.500000	0.008307
3195.250000	0.008310
3195.000000	0.008310
3194.750000	0.008308
3194.500000	0.008304
3194.250000	0.008297
3194.000000	0.008288

3193.750000	0.008276
3193.500000	0.008262
3193.250000	0.008246
3193.000000	0.008227
3192.750000	0.008207
3192.500000	0.008186
3192.250000	0.008167
3192.000000	0.008150
3191.750000	0.008135
3191.500000	0.008122
3191.250000	0.008113
3191.000000	0.008107
3190.750000	0.008105
3190.500000	0.008106
3190.250000	0.008109
3190.000000	0.008113
3189.750000	0.008116
3189.500000	0.008116
3189.250000	0.008110
3189.000000	0.008098
3188.750000	0.008081
3188.500000	0.008062
3188.250000	0.008044
3188.000000	0.008030
3187.750000	0.008022
3187.500000	0.008020

3187.250000	0.008023
3187.000000	0.008028
3186.750000	0.008034
3186.500000	0.008038
3186.250000	0.008039
3186.000000	0.008037
3185.750000	0.008031
3185.500000	0.008023
3185.250000	0.008012
3185.000000	0.008000
3184.750000	0.007985
3184.500000	0.007969
3184.250000	0.007951
3184.000000	0.007932
3183.750000	0.007911
3183.500000	0.007890
3183.250000	0.007870
3183.000000	0.007852
3182.750000	0.007839
3182.500000	0.007832
3182.250000	0.007832
3182.000000	0.007839
3181.750000	0.007853
3181.500000	0.007870
3181.250000	0.007888
3181.000000	0.007903

3180.750000	0.007913
3180.500000	0.007916
3180.250000	0.007911
3180.000000	0.007898
3179.750000	0.007881
3179.500000	0.007861
3179.250000	0.007843
3179.000000	0.007829
3178.750000	0.007822
3178.500000	0.007821
3178.250000	0.007827
3178.000000	0.007835
3177.750000	0.007844
3177.500000	0.007848
3177.250000	0.007845
3177.000000	0.007834
3176.750000	0.007816
3176.500000	0.007793
3176.250000	0.007767
3176.000000	0.007741
3175.750000	0.007718
3175.500000	0.007699
3175.250000	0.007685
3175.000000	0.007675
3174.750000	0.007670
3174.500000	0.007669

3174.250000	0.007671
3174.000000	0.007674
3173.750000	0.007676
3173.500000	0.007674
3173.250000	0.007668
3173.000000	0.007655
3172.750000	0.007636
3172.500000	0.007613
3172.250000	0.007590
3172.000000	0.007569
3171.750000	0.007552
3171.500000	0.007540
3171.250000	0.007532
3171.000000	0.007525
3170.750000	0.007515
3170.500000	0.007501
3170.250000	0.007482
3170.000000	0.007459
3169.750000	0.007434
3169.500000	0.007410
3169.250000	0.007389
3169.000000	0.007372
3168.750000	0.007359
3168.500000	0.007350
3168.250000	0.007345
3168.000000	0.007340

3167.750000	0.007337
3167.500000	0.007335
3167.250000	0.007333
3167.000000	0.007333
3166.750000	0.007334
3166.500000	0.007335
3166.250000	0.007337
3166.000000	0.007338
3165.750000	0.007338
3165.500000	0.007335
3165.250000	0.007330
3165.000000	0.007322
3164.750000	0.007312
3164.500000	0.007301
3164.250000	0.007290
3164.000000	0.007280
3163.750000	0.007272
3163.500000	0.007266
3163.250000	0.007261
3163.000000	0.007257
3162.750000	0.007253
3162.500000	0.007248
3162.250000	0.007241
3162.000000	0.007233
3161.750000	0.007225
3161.500000	0.007216

3161.250000	0.007208
3161.000000	0.007201
3160.750000	0.007193
3160.500000	0.007186
3160.250000	0.007178
3160.000000	0.007170
3159.750000	0.007162
3159.500000	0.007154
3159.250000	0.007146
3159.000000	0.007138
3158.750000	0.007130
3158.500000	0.007120
3158.250000	0.007109
3158.000000	0.007096
3157.750000	0.007081
3157.500000	0.007066
3157.250000	0.007051
3157.000000	0.007038
3156.750000	0.007027
3156.500000	0.007018
3156.250000	0.007012
3156.000000	0.007005
3155.750000	0.006999
3155.500000	0.006992
3155.250000	0.006984
3155.000000	0.006976

3154.750000	0.006967
3154.500000	0.006958
3154.250000	0.006950
3154.000000	0.006942
3153.750000	0.006936
3153.500000	0.006932
3153.250000	0.006928
3153.000000	0.006926
3152.750000	0.006925
3152.500000	0.006925
3152.250000	0.006925
3152.000000	0.006924
3151.750000	0.006923
3151.500000	0.006920
3151.250000	0.006915
3151.000000	0.006909
3150.750000	0.006901
3150.500000	0.006893
3150.250000	0.006887
3150.000000	0.006884
3149.750000	0.006885
3149.500000	0.006889
3149.250000	0.006896
3149.000000	0.006903
3148.750000	0.006908
3148.500000	0.006908

3148.250000	0.006903
3148.000000	0.006893
3147.750000	0.006878
3147.500000	0.006861
3147.250000	0.006843
3147.000000	0.006826
3146.750000	0.006812
3146.500000	0.006800
3146.250000	0.006791
3146.000000	0.006784
3145.750000	0.006777
3145.500000	0.006771
3145.250000	0.006766
3145.000000	0.006760
3144.750000	0.006754
3144.500000	0.006748
3144.250000	0.006741
3144.000000	0.006733
3143.750000	0.006724
3143.500000	0.006713
3143.250000	0.006701
3143.000000	0.006689
3142.750000	0.006677
3142.500000	0.006668
3142.250000	0.006662
3142.000000	0.006659

3141.750000	0.006661
3141.500000	0.006666
3141.250000	0.006672
3141.000000	0.006679
3140.750000	0.006684
3140.500000	0.006686
3140.250000	0.006685
3140.000000	0.006680
3139.750000	0.006671
3139.500000	0.006659
3139.250000	0.006646
3139.000000	0.006631
3138.750000	0.006616
3138.500000	0.006602
3138.250000	0.006589
3138.000000	0.006577
3137.750000	0.006567
3137.500000	0.006560
3137.250000	0.006554
3137.000000	0.006550
3136.750000	0.006550
3136.500000	0.006552
3136.250000	0.006559
3136.000000	0.006570
3135.750000	0.006584
3135.500000	0.006602

3135.250000	0.006620
3135.000000	0.006635
3134.750000	0.006646
3134.500000	0.006650
3134.250000	0.006646
3134.000000	0.006636
3133.750000	0.006620
3133.500000	0.006601
3133.250000	0.006583
3133.000000	0.006566
3132.750000	0.006553
3132.500000	0.006542
3132.250000	0.006532
3132.000000	0.006523
3131.750000	0.006513
3131.500000	0.006502
3131.250000	0.006490
3131.000000	0.006478
3130.750000	0.006467
3130.500000	0.006457
3130.250000	0.006450
3130.000000	0.006447
3129.750000	0.006448
3129.500000	0.006453
3129.250000	0.006460
3129.000000	0.006469

3128.750000	0.006477
3128.500000	0.006483
3128.250000	0.006484
3128.000000	0.006482
3127.750000	0.006475
3127.500000	0.006467
3127.250000	0.006458
3127.000000	0.006450
3126.750000	0.006445
3126.500000	0.006444
3126.250000	0.006447
3126.000000	0.006452
3125.750000	0.006460
3125.500000	0.006468
3125.250000	0.006475
3125.000000	0.006480
3124.750000	0.006482
3124.500000	0.006481
3124.250000	0.006475
3124.000000	0.006466
3123.750000	0.006455
3123.500000	0.006443
3123.250000	0.006433
3123.000000	0.006426
3122.750000	0.006423
3122.500000	0.006422

3122.250000	0.006424
3122.000000	0.006425
3121.750000	0.006424
3121.500000	0.006421
3121.250000	0.006415
3121.000000	0.006407
3120.750000	0.006399
3120.500000	0.006391
3120.250000	0.006385
3120.000000	0.006381
3119.750000	0.006378
3119.500000	0.006377
3119.250000	0.006377
3119.000000	0.006376
3118.750000	0.006374
3118.500000	0.006370
3118.250000	0.006363
3118.000000	0.006355
3117.750000	0.006345
3117.500000	0.006337
3117.250000	0.006331
3117.000000	0.006330
3116.750000	0.006336
3116.500000	0.006347
3116.250000	0.006364
3116.000000	0.006384

3115.750000	0.006404
3115.500000	0.006421
3115.250000	0.006434
3115.000000	0.006441
3114.750000	0.006443
3114.500000	0.006442
3114.250000	0.006440
3114.000000	0.006438
3113.750000	0.006438
3113.500000	0.006440
3113.250000	0.006444
3113.000000	0.006450
3112.750000	0.006456
3112.500000	0.006464
3112.250000	0.006473
3112.000000	0.006485
3111.750000	0.006500
3111.500000	0.006518
3111.250000	0.006538
3111.000000	0.006558
3110.750000	0.006575
3110.500000	0.006586
3110.250000	0.006590
3110.000000	0.006585
3109.750000	0.006575
3109.500000	0.006560

3109.250000	0.006544
3109.000000	0.006532
3108.750000	0.006526
3108.500000	0.006527
3108.250000	0.006534
3108.000000	0.006547
3107.750000	0.006563
3107.500000	0.006580
3107.250000	0.006596
3107.000000	0.006608
3106.750000	0.006618
3106.500000	0.006624
3106.250000	0.006629
3106.000000	0.006632
3105.750000	0.006637
3105.500000	0.006643
3105.250000	0.006650
3105.000000	0.006658
3104.750000	0.006666
3104.500000	0.006671
3104.250000	0.006673
3104.000000	0.006671
3103.750000	0.006667
3103.500000	0.006660
3103.250000	0.006655
3103.000000	0.006652

3102.750000	0.006652
3102.500000	0.006656
3102.250000	0.006663
3102.000000	0.006670
3101.750000	0.006675
3101.500000	0.006677
3101.250000	0.006676
3101.000000	0.006673
3100.750000	0.006670
3100.500000	0.006666
3100.250000	0.006665
3100.000000	0.006665
3099.750000	0.006667
3099.500000	0.006669
3099.250000	0.006670
3099.000000	0.006670
3098.750000	0.006669
3098.500000	0.006666
3098.250000	0.006662
3098.000000	0.006656
3097.750000	0.006650
3097.500000	0.006643
3097.250000	0.006638
3097.000000	0.006635
3096.750000	0.006635
3096.500000	0.006639

3096.250000	0.006645
3096.000000	0.006653
3095.750000	0.006661
3095.500000	0.006666
3095.250000	0.006669
3095.000000	0.006670
3094.750000	0.006669
3094.500000	0.006669
3094.250000	0.006670
3094.000000	0.006673
3093.750000	0.006678
3093.500000	0.006684
3093.250000	0.006689
3093.000000	0.006692
3092.750000	0.006690
3092.500000	0.006685
3092.250000	0.006675
3092.000000	0.006662
3091.750000	0.006648
3091.500000	0.006634
3091.250000	0.006621
3091.000000	0.006611
3090.750000	0.006605
3090.500000	0.006603
3090.250000	0.006606
3090.000000	0.006614

3089.750000	0.006626
3089.500000	0.006641
3089.250000	0.006659
3089.000000	0.006679
3088.750000	0.006698
3088.500000	0.006715
3088.250000	0.006728
3088.000000	0.006736
3087.750000	0.006736
3087.500000	0.006730
3087.250000	0.006717
3087.000000	0.006700
3086.750000	0.006683
3086.500000	0.006667
3086.250000	0.006656
3086.000000	0.006651
3085.750000	0.006653
3085.500000	0.006660
3085.250000	0.006673
3085.000000	0.006689
3084.750000	0.006707
3084.500000	0.006726
3084.250000	0.006745
3084.000000	0.006764
3083.750000	0.006782
3083.500000	0.006798

3083.250000	0.006813
3083.000000	0.006826
3082.750000	0.006837
3082.500000	0.006846
3082.250000	0.006853
3082.000000	0.006856
3081.750000	0.006857
3081.500000	0.006854
3081.250000	0.006847
3081.000000	0.006837
3080.750000	0.006825
3080.500000	0.006815
3080.250000	0.006807
3080.000000	0.006805
3079.750000	0.006809
3079.500000	0.006818
3079.250000	0.006832
3079.000000	0.006848
3078.750000	0.006864
3078.500000	0.006877
3078.250000	0.006887
3078.000000	0.006893
3077.750000	0.006895
3077.500000	0.006896
3077.250000	0.006897
3077.000000	0.006901

3076.750000	0.006909
3076.500000	0.006923
3076.250000	0.006942
3076.000000	0.006965
3075.750000	0.006990
3075.500000	0.007013
3075.250000	0.007032
3075.000000	0.007045
3074.750000	0.007052
3074.500000	0.007056
3074.250000	0.007058
3074.000000	0.007061
3073.750000	0.007067
3073.500000	0.007077
3073.250000	0.007089
3073.000000	0.007100
3072.750000	0.007110
3072.500000	0.007115
3072.250000	0.007116
3072.000000	0.007113
3071.750000	0.007108
3071.500000	0.007101
3071.250000	0.007095
3071.000000	0.007090
3070.750000	0.007086
3070.500000	0.007083

3070.250000	0.007080
3070.000000	0.007077
3069.750000	0.007073
3069.500000	0.007068
3069.250000	0.007063
3069.000000	0.007057
3068.750000	0.007053
3068.500000	0.007051
3068.250000	0.007052
3068.000000	0.007055
3067.750000	0.007062
3067.500000	0.007069
3067.250000	0.007077
3067.000000	0.007082
3066.750000	0.007086
3066.500000	0.007087
3066.250000	0.007088
3066.000000	0.007090
3065.750000	0.007095
3065.500000	0.007104
3065.250000	0.007116
3065.000000	0.007129
3064.750000	0.007141
3064.500000	0.007150
3064.250000	0.007155
3064.000000	0.007157

3063.750000	0.007158
3063.500000	0.007160
3063.250000	0.007165
3063.000000	0.007174
3062.750000	0.007185
3062.500000	0.007197
3062.250000	0.007208
3062.000000	0.007215
3061.750000	0.007218
3061.500000	0.007217
3061.250000	0.007213
3061.000000	0.007210
3060.750000	0.007208
3060.500000	0.007209
3060.250000	0.007213
3060.000000	0.007219
3059.750000	0.007224
3059.500000	0.007227
3059.250000	0.007226
3059.000000	0.007220
3058.750000	0.007211
3058.500000	0.007201
3058.250000	0.007190
3058.000000	0.007182
3057.750000	0.007177
3057.500000	0.007174

3057.250000	0.007173
3057.000000	0.007172
3056.750000	0.007168
3056.500000	0.007160
3056.250000	0.007149
3056.000000	0.007134
3055.750000	0.007120
3055.500000	0.007107
3055.250000	0.007098
3055.000000	0.007094
3054.750000	0.007097
3054.500000	0.007105
3054.250000	0.007116
3054.000000	0.007127
3053.750000	0.007135
3053.500000	0.007139
3053.250000	0.007135
3053.000000	0.007125
3052.750000	0.007110
3052.500000	0.007092
3052.250000	0.007074
3052.000000	0.007060
3051.750000	0.007052
3051.500000	0.007050
3051.250000	0.007055
3051.000000	0.007063

3050.750000	0.007075
3050.500000	0.007089
3050.250000	0.007103
3050.000000	0.007118
3049.750000	0.007133
3049.500000	0.007146
3049.250000	0.007157
3049.000000	0.007165
3048.750000	0.007167
3048.500000	0.007163
3048.250000	0.007155
3048.000000	0.007144
3047.750000	0.007132
3047.500000	0.007122
3047.250000	0.007115
3047.000000	0.007111
3046.750000	0.007110
3046.500000	0.007112
3046.250000	0.007114
3046.000000	0.007116
3045.750000	0.007120
3045.500000	0.007123
3045.250000	0.007127
3045.000000	0.007130
3044.750000	0.007132
3044.500000	0.007131

3044.250000	0.007125
3044.000000	0.007117
3043.750000	0.007107
3043.500000	0.007098
3043.250000	0.007093
3043.000000	0.007093
3042.750000	0.007099
3042.500000	0.007109
3042.250000	0.007123
3042.000000	0.007136
3041.750000	0.007148
3041.500000	0.007156
3041.250000	0.007162
3041.000000	0.007167
3040.750000	0.007172
3040.500000	0.007179
3040.250000	0.007191
3040.000000	0.007206
3039.750000	0.007225
3039.500000	0.007245
3039.250000	0.007263
3039.000000	0.007279
3038.750000	0.007290
3038.500000	0.007295
3038.250000	0.007296
3038.000000	0.007294

3037.750000	0.007292
3037.500000	0.007290
3037.250000	0.007290
3037.000000	0.007292
3036.750000	0.007297
3036.500000	0.007303
3036.250000	0.007309
3036.000000	0.007317
3035.750000	0.007326
3035.500000	0.007338
3035.250000	0.007353
3035.000000	0.007370
3034.750000	0.007389
3034.500000	0.007409
3034.250000	0.007426
3034.000000	0.007440
3033.750000	0.007451
3033.500000	0.007460
3033.250000	0.007468
3033.000000	0.007478
3032.750000	0.007490
3032.500000	0.007506
3032.250000	0.007524
3032.000000	0.007541
3031.750000	0.007555
3031.500000	0.007564

3031.250000	0.007568
3031.000000	0.007567
3030.750000	0.007563
3030.500000	0.007560
3030.250000	0.007558
3030.000000	0.007561
3029.750000	0.007569
3029.500000	0.007581
3029.250000	0.007598
3029.000000	0.007619
3028.750000	0.007642
3028.500000	0.007666
3028.250000	0.007689
3028.000000	0.007711
3027.750000	0.007728
3027.500000	0.007739
3027.250000	0.007744
3027.000000	0.007743
3026.750000	0.007737
3026.500000	0.007730
3026.250000	0.007724
3026.000000	0.007725
3025.750000	0.007732
3025.500000	0.007748
3025.250000	0.007771
3025.000000	0.007797

3024.750000	0.007823
3024.500000	0.007845
3024.250000	0.007863
3024.000000	0.007875
3023.750000	0.007884
3023.500000	0.007893
3023.250000	0.007906
3023.000000	0.007925
3022.750000	0.007952
3022.500000	0.007987
3022.250000	0.008027
3022.000000	0.008069
3021.750000	0.008111
3021.500000	0.008147
3021.250000	0.008178
3021.000000	0.008201
3020.750000	0.008219
3020.500000	0.008233
3020.250000	0.008246
3020.000000	0.008261
3019.750000	0.008280
3019.500000	0.008304
3019.250000	0.008334
3019.000000	0.008368
3018.750000	0.008407
3018.500000	0.008449

3018.250000	0.008494
3018.000000	0.008540
3017.750000	0.008589
3017.500000	0.008640
3017.250000	0.008692
3017.000000	0.008745
3016.750000	0.008797
3016.500000	0.008845
3016.250000	0.008888
3016.000000	0.008924
3015.750000	0.008952
3015.500000	0.008974
3015.250000	0.008991
3015.000000	0.009008
3014.750000	0.009028
3014.500000	0.009055
3014.250000	0.009090
3014.000000	0.009135
3013.750000	0.009187
3013.500000	0.009244
3013.250000	0.009302
3013.000000	0.009358
3012.750000	0.009410
3012.500000	0.009457
3012.250000	0.009499
3012.000000	0.009539

3011.750000	0.009580
3011.500000	0.009623
3011.250000	0.009671
3011.000000	0.009725
3010.750000	0.009784
3010.500000	0.009848
3010.250000	0.009916
3010.000000	0.009986
3009.750000	0.010055
3009.500000	0.010121
3009.250000	0.010183
3009.000000	0.010241
3008.750000	0.010295
3008.500000	0.010346
3008.250000	0.010397
3008.000000	0.010449
3007.750000	0.010503
3007.500000	0.010561
3007.250000	0.010623
3007.000000	0.010687
3006.750000	0.010752
3006.500000	0.010819
3006.250000	0.010887
3006.000000	0.010955
3005.750000	0.011023
3005.500000	0.011092

3005.250000	0.011159
3005.000000	0.011223
3004.750000	0.011284
3004.500000	0.011339
3004.250000	0.011390
3004.000000	0.011436
3003.750000	0.011480
3003.500000	0.011523
3003.250000	0.011567
3003.000000	0.011611
3002.750000	0.011658
3002.500000	0.011708
3002.250000	0.011762
3002.000000	0.011822
3001.750000	0.011888
3001.500000	0.011960
3001.250000	0.012038
3001.000000	0.012118
3000.750000	0.012198
3000.500000	0.012276
3000.250000	0.012351
3000.000000	0.012423
2999.750000	0.012495
2999.500000	0.012568
2999.250000	0.012643
2999.000000	0.012722

2998.750000	0.012803
2998.500000	0.012882
2998.250000	0.012959
2998.000000	0.013031
2997.750000	0.013101
2997.500000	0.013170
2997.250000	0.013242
2997.000000	0.013319
2996.750000	0.013403
2996.500000	0.013494
2996.250000	0.013589
2996.000000	0.013685
2995.750000	0.013780
2995.500000	0.013871
2995.250000	0.013956
2995.000000	0.014034
2994.750000	0.014105
2994.500000	0.014168
2994.250000	0.014225
2994.000000	0.014278
2993.750000	0.014328
2993.500000	0.014379
2993.250000	0.014434
2993.000000	0.014496
2992.750000	0.014567
2992.500000	0.014647

2992.250000	0.014735
2992.000000	0.014829
2991.750000	0.014925
2991.500000	0.015020
2991.250000	0.015113
2991.000000	0.015201
2990.750000	0.015286
2990.500000	0.015366
2990.250000	0.015444
2990.000000	0.015520
2989.750000	0.015597
2989.500000	0.015674
2989.250000	0.015752
2989.000000	0.015832
2988.750000	0.015913
2988.500000	0.015992
2988.250000	0.016068
2988.000000	0.016140
2987.750000	0.016206
2987.500000	0.016268
2987.250000	0.016326
2987.000000	0.016382
2986.750000	0.016440
2986.500000	0.016500
2986.250000	0.016563
2986.000000	0.016629

2985.750000	0.016696
2985.500000	0.016761
2985.250000	0.016823
2985.000000	0.016880
2984.750000	0.016930
2984.500000	0.016975
2984.250000	0.017015
2984.000000	0.017053
2983.750000	0.017089
2983.500000	0.017126
2983.250000	0.017163
2983.000000	0.017203
2982.750000	0.017244
2982.500000	0.017288
2982.250000	0.017334
2982.000000	0.017382
2981.750000	0.017431
2981.500000	0.017479
2981.250000	0.017526
2981.000000	0.017569
2980.750000	0.017607
2980.500000	0.017641
2980.250000	0.017670
2980.000000	0.017699
2979.750000	0.017729
2979.500000	0.017762

2979.250000	0.017800
2979.000000	0.017841
2978.750000	0.017883
2978.500000	0.017923
2978.250000	0.017958
2978.000000	0.017987
2977.750000	0.018010
2977.500000	0.018030
2977.250000	0.018050
2977.000000	0.018072
2976.750000	0.018099
2976.500000	0.018132
2976.250000	0.018172
2976.000000	0.018220
2975.750000	0.018274
2975.500000	0.018335
2975.250000	0.018403
2975.000000	0.018477
2974.750000	0.018555
2974.500000	0.018636
2974.250000	0.018719
2974.000000	0.018801
2973.750000	0.018882
2973.500000	0.018962
2973.250000	0.019043
2973.000000	0.019128

2972.750000	0.019220
2972.500000	0.019321
2972.250000	0.019434
2972.000000	0.019559
2971.750000	0.019692
2971.500000	0.019830
2971.250000	0.019968
2971.000000	0.020101
2970.750000	0.020222
2970.500000	0.020328
2970.250000	0.020417
2970.000000	0.020490
2969.750000	0.020549
2969.500000	0.020597
2969.250000	0.020639
2969.000000	0.020679
2968.750000	0.020719
2968.500000	0.020761
2968.250000	0.020807
2968.000000	0.020858
2967.750000	0.020913
2967.500000	0.020973
2967.250000	0.021037
2967.000000	0.021104
2966.750000	0.021173
2966.500000	0.021243

2966.250000	0.021313
2966.000000	0.021384
2965.750000	0.021458
2965.500000	0.021535
2965.250000	0.021616
2965.000000	0.021701
2964.750000	0.021789
2964.500000	0.021879
2964.250000	0.021969
2964.000000	0.022059
2963.750000	0.022148
2963.500000	0.022239
2963.250000	0.022331
2963.000000	0.022426
2962.750000	0.022522
2962.500000	0.022617
2962.250000	0.022710
2962.000000	0.022798
2961.750000	0.022881
2961.500000	0.022958
2961.250000	0.023030
2961.000000	0.023100
2960.750000	0.023170
2960.500000	0.023241
2960.250000	0.023313
2960.000000	0.023389

2959.750000	0.023468
2959.500000	0.023550
2959.250000	0.023636
2959.000000	0.023723
2958.750000	0.023811
2958.500000	0.023896
2958.250000	0.023976
2958.000000	0.024050
2957.750000	0.024117
2957.500000	0.024178
2957.250000	0.024236
2957.000000	0.024295
2956.750000	0.024357
2956.500000	0.024423
2956.250000	0.024494
2956.000000	0.024567
2955.750000	0.024641
2955.500000	0.024714
2955.250000	0.024785
2955.000000	0.024853
2954.750000	0.024918
2954.500000	0.024980
2954.250000	0.025039
2954.000000	0.025093
2953.750000	0.025144
2953.500000	0.025192

2953.250000	0.025239
2953.000000	0.025286
2952.750000	0.025336
2952.500000	0.025389
2952.250000	0.025447
2952.000000	0.025511
2951.750000	0.025579
2951.500000	0.025651
2951.250000	0.025726
2951.000000	0.025802
2950.750000	0.025878
2950.500000	0.025953
2950.250000	0.026026
2950.000000	0.026098
2949.750000	0.026171
2949.500000	0.026246
2949.250000	0.026326
2949.000000	0.026411
2948.750000	0.026503
2948.500000	0.026601
2948.250000	0.026706
2948.000000	0.026815
2947.750000	0.026929
2947.500000	0.027048
2947.250000	0.027170
2947.000000	0.027296

2946.750000	0.027425
2946.500000	0.027555
2946.250000	0.027685
2946.000000	0.027814
2945.750000	0.027942
2945.500000	0.028069
2945.250000	0.028198
2945.000000	0.028330
2944.750000	0.028467
2944.500000	0.028608
2944.250000	0.028754
2944.000000	0.028902
2943.750000	0.029052
2943.500000	0.029202
2943.250000	0.029351
2943.000000	0.029501
2942.750000	0.029651
2942.500000	0.029804
2942.250000	0.029960
2942.000000	0.030118
2941.750000	0.030278
2941.500000	0.030440
2941.250000	0.030603
2941.000000	0.030764
2940.750000	0.030924
2940.500000	0.031081

2940.250000	0.031236
2940.000000	0.031387
2939.750000	0.031537
2939.500000	0.031685
2939.250000	0.031832
2939.000000	0.031979
2938.750000	0.032127
2938.500000	0.032273
2938.250000	0.032420
2938.000000	0.032565
2937.750000	0.032709
2937.500000	0.032852
2937.250000	0.032994
2937.000000	0.033134
2936.750000	0.033273
2936.500000	0.033410
2936.250000	0.033543
2936.000000	0.033674
2935.750000	0.033801
2935.500000	0.033927
2935.250000	0.034053
2935.000000	0.034181
2934.750000	0.034311
2934.500000	0.034443
2934.250000	0.034574
2934.000000	0.034702

2933.750000	0.034824
2933.500000	0.034939
2933.250000	0.035045
2933.000000	0.035142
2932.750000	0.035233
2932.500000	0.035318
2932.250000	0.035399
2932.000000	0.035478
2931.750000	0.035557
2931.500000	0.035637
2931.250000	0.035717
2931.000000	0.035798
2930.750000	0.035879
2930.500000	0.035958
2930.250000	0.036035
2930.000000	0.036107
2929.750000	0.036174
2929.500000	0.036237
2929.250000	0.036297
2929.000000	0.036354
2928.750000	0.036409
2928.500000	0.036460
2928.250000	0.036507
2928.000000	0.036547
2927.750000	0.036578
2927.500000	0.036601

2927.250000	0.036613
2927.000000	0.036617
2926.750000	0.036615
2926.500000	0.036606
2926.250000	0.036592
2926.000000	0.036574
2925.750000	0.036550
2925.500000	0.036521
2925.250000	0.036486
2925.000000	0.036446
2924.750000	0.036400
2924.500000	0.036349
2924.250000	0.036292
2924.000000	0.036229
2923.750000	0.036160
2923.500000	0.036082
2923.250000	0.035996
2923.000000	0.035899
2922.750000	0.035790
2922.500000	0.035670
2922.250000	0.035538
2922.000000	0.035395
2921.750000	0.035241
2921.500000	0.035078
2921.250000	0.034906
2921.000000	0.034726

2920.750000	0.034538
2920.500000	0.034342
2920.250000	0.034137
2920.000000	0.033922
2919.750000	0.033698
2919.500000	0.033464
2919.250000	0.033222
2919.000000	0.032974
2918.750000	0.032721
2918.500000	0.032464
2918.250000	0.032203
2918.000000	0.031939
2917.750000	0.031672
2917.500000	0.031401
2917.250000	0.031127
2917.000000	0.030851
2916.750000	0.030571
2916.500000	0.030289
2916.250000	0.030005
2916.000000	0.029719
2915.750000	0.029432
2915.500000	0.029146
2915.250000	0.028861
2915.000000	0.028581
2914.750000	0.028306
2914.500000	0.028037

2914.250000	0.027775
2914.000000	0.027517
2913.750000	0.027264
2913.500000	0.027014
2913.250000	0.026765
2913.000000	0.026518
2912.750000	0.026273
2912.500000	0.026032
2912.250000	0.025795
2912.000000	0.025563
2911.750000	0.025339
2911.500000	0.025121
2911.250000	0.024911
2911.000000	0.024708
2910.750000	0.024512
2910.500000	0.024322
2910.250000	0.024137
2910.000000	0.023957
2909.750000	0.023783
2909.500000	0.023613
2909.250000	0.023450
2909.000000	0.023293
2908.750000	0.023141
2908.500000	0.022995
2908.250000	0.022852
2908.000000	0.022711

2907.750000	0.022571
2907.500000	0.022429
2907.250000	0.022286
2907.000000	0.022144
2906.750000	0.022003
2906.500000	0.021866
2906.250000	0.021736
2906.000000	0.021613
2905.750000	0.021497
2905.500000	0.021386
2905.250000	0.021279
2905.000000	0.021174
2904.750000	0.021069
2904.500000	0.020963
2904.250000	0.020859
2904.000000	0.020756
2903.750000	0.020657
2903.500000	0.020564
2903.250000	0.020477
2903.000000	0.020396
2902.750000	0.020319
2902.500000	0.020243
2902.250000	0.020167
2902.000000	0.020087
2901.750000	0.020004
2901.500000	0.019915

2901.250000	0.019823
2901.000000	0.019728
2900.750000	0.019633
2900.500000	0.019542
2900.250000	0.019455
2900.000000	0.019374
2899.750000	0.019299
2899.500000	0.019231
2899.250000	0.019166
2899.000000	0.019104
2898.750000	0.019041
2898.500000	0.018977
2898.250000	0.018911
2898.000000	0.018842
2897.750000	0.018772
2897.500000	0.018702
2897.250000	0.018632
2897.000000	0.018564
2896.750000	0.018498
2896.500000	0.018435
2896.250000	0.018374
2896.000000	0.018314
2895.750000	0.018255
2895.500000	0.018198
2895.250000	0.018144
2895.000000	0.018094

2894.750000	0.018048
2894.500000	0.018006
2894.250000	0.017969
2894.000000	0.017934
2893.750000	0.017899
2893.500000	0.017864
2893.250000	0.017825
2893.000000	0.017780
2892.750000	0.017730
2892.500000	0.017676
2892.250000	0.017619
2892.000000	0.017561
2891.750000	0.017506
2891.500000	0.017455
2891.250000	0.017411
2891.000000	0.017373
2890.750000	0.017339
2890.500000	0.017308
2890.250000	0.017277
2890.000000	0.017244
2889.750000	0.017208
2889.500000	0.017168
2889.250000	0.017126
2889.000000	0.017084
2888.750000	0.017042
2888.500000	0.017003

2888.250000	0.016967
2888.000000	0.016933
2887.750000	0.016901
2887.500000	0.016870
2887.250000	0.016839
2887.000000	0.016808
2886.750000	0.016776
2886.500000	0.016745
2886.250000	0.016716
2886.000000	0.016690
2885.750000	0.016668
2885.500000	0.016651
2885.250000	0.016640
2885.000000	0.016633
2884.750000	0.016629
2884.500000	0.016625
2884.250000	0.016620
2884.000000	0.016610
2883.750000	0.016596
2883.500000	0.016576
2883.250000	0.016551
2883.000000	0.016525
2882.750000	0.016499
2882.500000	0.016477
2882.250000	0.016459
2882.000000	0.016448

2881.750000	0.016444
2881.500000	0.016445
2881.250000	0.016451
2881.000000	0.016460
2880.750000	0.016471
2880.500000	0.016484
2880.250000	0.016497
2880.000000	0.016511
2879.750000	0.016527
2879.500000	0.016543
2879.250000	0.016561
2879.000000	0.016578
2878.750000	0.016595
2878.500000	0.016611
2878.250000	0.016627
2878.000000	0.016642
2877.750000	0.016656
2877.500000	0.016670
2877.250000	0.016684
2877.000000	0.016697
2876.750000	0.016710
2876.500000	0.016723
2876.250000	0.016736
2876.000000	0.016753
2875.750000	0.016773
2875.500000	0.016798

2875.250000	0.016828
2875.000000	0.016861
2874.750000	0.016894
2874.500000	0.016927
2874.250000	0.016957
2874.000000	0.016984
2873.750000	0.017009
2873.500000	0.017034
2873.250000	0.017062
2873.000000	0.017094
2872.750000	0.017130
2872.500000	0.017169
2872.250000	0.017208
2872.000000	0.017245
2871.750000	0.017278
2871.500000	0.017304
2871.250000	0.017324
2871.000000	0.017338
2870.750000	0.017348
2870.500000	0.017353
2870.250000	0.017355
2870.000000	0.017355
2869.750000	0.017352
2869.500000	0.017350
2869.250000	0.017352
2869.000000	0.017360

2868.750000	0.017377
2868.500000	0.017405
2868.250000	0.017445
2868.000000	0.017493
2867.750000	0.017545
2867.500000	0.017597
2867.250000	0.017645
2867.000000	0.017686
2866.750000	0.017719
2866.500000	0.017745
2866.250000	0.017769
2866.000000	0.017792
2865.750000	0.017819
2865.500000	0.017851
2865.250000	0.017890
2865.000000	0.017934
2864.750000	0.017983
2864.500000	0.018036
2864.250000	0.018091
2864.000000	0.018149
2863.750000	0.018211
2863.500000	0.018278
2863.250000	0.018349
2863.000000	0.018425
2862.750000	0.018505
2862.500000	0.018586

2862.250000	0.018668
2862.000000	0.018749
2861.750000	0.018830
2861.500000	0.018911
2861.250000	0.018991
2861.000000	0.019072
2860.750000	0.019152
2860.500000	0.019232
2860.250000	0.019309
2860.000000	0.019385
2859.750000	0.019459
2859.500000	0.019533
2859.250000	0.019607
2859.000000	0.019683
2858.750000	0.019758
2858.500000	0.019833
2858.250000	0.019905
2858.000000	0.019972
2857.750000	0.020033
2857.500000	0.020086
2857.250000	0.020132
2857.000000	0.020173
2856.750000	0.020210
2856.500000	0.020242
2856.250000	0.020268
2856.000000	0.020288

2855.750000	0.020298
2855.500000	0.020297
2855.250000	0.020284
2855.000000	0.020259
2854.750000	0.020223
2854.500000	0.020178
2854.250000	0.020126
2854.000000	0.020069
2853.750000	0.020007
2853.500000	0.019941
2853.250000	0.019871
2853.000000	0.019798
2852.750000	0.019720
2852.500000	0.019638
2852.250000	0.019549
2852.000000	0.019453
2851.750000	0.019349
2851.500000	0.019234
2851.250000	0.019110
2851.000000	0.018974
2850.750000	0.018826
2850.500000	0.018668
2850.250000	0.018499
2850.000000	0.018318
2849.750000	0.018126
2849.500000	0.017921

2849.250000	0.017704
2849.000000	0.017475
2848.750000	0.017233
2848.500000	0.016981
2848.250000	0.016720
2848.000000	0.016453
2847.750000	0.016181
2847.500000	0.015905
2847.250000	0.015626
2847.000000	0.015342
2846.750000	0.015054
2846.500000	0.014763
2846.250000	0.014468
2846.000000	0.014172
2845.750000	0.013879
2845.500000	0.013591
2845.250000	0.013312
2845.000000	0.013044
2844.750000	0.012789
2844.500000	0.012545
2844.250000	0.012313
2844.000000	0.012090
2843.750000	0.011875
2843.500000	0.011667
2843.250000	0.011465
2843.000000	0.011268

2842.750000	0.011079
2842.500000	0.010897
2842.250000	0.010722
2842.000000	0.010556
2841.750000	0.010398
2841.500000	0.010248
2841.250000	0.010104
2841.000000	0.009965
2840.750000	0.009829
2840.500000	0.009696
2840.250000	0.009564
2840.000000	0.009433
2839.750000	0.009304
2839.500000	0.009180
2839.250000	0.009061
2839.000000	0.008949
2838.750000	0.008846
2838.500000	0.008752
2838.250000	0.008666
2838.000000	0.008587
2837.750000	0.008515
2837.500000	0.008446
2837.250000	0.008380
2837.000000	0.008316
2836.750000	0.008254
2836.500000	0.008194

2836.250000	0.008136
2836.000000	0.008080
2835.750000	0.008027
2835.500000	0.007975
2835.250000	0.007925
2835.000000	0.007874
2834.750000	0.007822
2834.500000	0.007768
2834.250000	0.007714
2834.000000	0.007659
2833.750000	0.007606
2833.500000	0.007556
2833.250000	0.007509
2833.000000	0.007468
2832.750000	0.007432
2832.500000	0.007400
2832.250000	0.007373
2832.000000	0.007349
2831.750000	0.007326
2831.500000	0.007303
2831.250000	0.007281
2831.000000	0.007258
2830.750000	0.007234
2830.500000	0.007209
2830.250000	0.007184
2830.000000	0.007158

2829.750000	0.007132
2829.500000	0.007104
2829.250000	0.007076
2829.000000	0.007046
2828.750000	0.007015
2828.500000	0.006982
2828.250000	0.006949
2828.000000	0.006916
2827.750000	0.006884
2827.500000	0.006852
2827.250000	0.006822
2827.000000	0.006793
2826.750000	0.006766
2826.500000	0.006742
2826.250000	0.006719
2826.000000	0.006699
2825.750000	0.006681
2825.500000	0.006663
2825.250000	0.006646
2825.000000	0.006627
2824.750000	0.006606
2824.500000	0.006583
2824.250000	0.006558
2824.000000	0.006531
2823.750000	0.006505
2823.500000	0.006480

2823.250000	0.006458
2823.000000	0.006438
2822.750000	0.006421
2822.500000	0.006405
2822.250000	0.006390
2822.000000	0.006375
2821.750000	0.006358
2821.500000	0.006340
2821.250000	0.006319
2821.000000	0.006295
2820.750000	0.006267
2820.500000	0.006236
2820.250000	0.006204
2820.000000	0.006173
2819.750000	0.006146
2819.500000	0.006124
2819.250000	0.006110
2819.000000	0.006101
2818.750000	0.006098
2818.500000	0.006097
2818.250000	0.006096
2818.000000	0.006093
2817.750000	0.006086
2817.500000	0.006075
2817.250000	0.006061
2817.000000	0.006045

2816.750000	0.006027
2816.500000	0.006007
2816.250000	0.005987
2816.000000	0.005967
2815.750000	0.005948
2815.500000	0.005930
2815.250000	0.005915
2815.000000	0.005902
2814.750000	0.005889
2814.500000	0.005877
2814.250000	0.005862
2814.000000	0.005845
2813.750000	0.005826
2813.500000	0.005805
2813.250000	0.005785
2813.000000	0.005766
2812.750000	0.005750
2812.500000	0.005736
2812.250000	0.005722
2812.000000	0.005708
2811.750000	0.005692
2811.500000	0.005675
2811.250000	0.005657
2811.000000	0.005639
2810.750000	0.005624
2810.500000	0.005612

2810.250000	0.005603
2810.000000	0.005596
2809.750000	0.005591
2809.500000	0.005585
2809.250000	0.005578
2809.000000	0.005571
2808.750000	0.005561
2808.500000	0.005551
2808.250000	0.005539
2808.000000	0.005526
2807.750000	0.005511
2807.500000	0.005496
2807.250000	0.005482
2807.000000	0.005469
2806.750000	0.005460
2806.500000	0.005455
2806.250000	0.005452
2806.000000	0.005452
2805.750000	0.005452
2805.500000	0.005449
2805.250000	0.005444
2805.000000	0.005434
2804.750000	0.005423
2804.500000	0.005409
2804.250000	0.005397
2804.000000	0.005385

2803.750000	0.005377
2803.500000	0.005370
2803.250000	0.005364
2803.000000	0.005359
2802.750000	0.005354
2802.500000	0.005347
2802.250000	0.005339
2802.000000	0.005330
2801.750000	0.005320
2801.500000	0.005309
2801.250000	0.005297
2801.000000	0.005283
2800.750000	0.005268
2800.500000	0.005252
2800.250000	0.005234
2800.000000	0.005216
2799.750000	0.005198
2799.500000	0.005182
2799.250000	0.005168
2799.000000	0.005157
2798.750000	0.005149
2798.500000	0.005145
2798.250000	0.005144
2798.000000	0.005145
2797.750000	0.005148
2797.500000	0.005151

2797.250000	0.005153
2797.000000	0.005152
2796.750000	0.005148
2796.500000	0.005140
2796.250000	0.005129
2796.000000	0.005116
2795.750000	0.005103
2795.500000	0.005091
2795.250000	0.005082
2795.000000	0.005076
2794.750000	0.005072
2794.500000	0.005069
2794.250000	0.005066
2794.000000	0.005061
2793.750000	0.005054
2793.500000	0.005045
2793.250000	0.005036
2793.000000	0.005028
2792.750000	0.005022
2792.500000	0.005017
2792.250000	0.005014
2792.000000	0.005013
2791.750000	0.005011
2791.500000	0.005010
2791.250000	0.005006
2791.000000	0.005001

2790.750000	0.004994
2790.500000	0.004985
2790.250000	0.004975
2790.000000	0.004964
2789.750000	0.004955
2789.500000	0.004947
2789.250000	0.004941
2789.000000	0.004937
2788.750000	0.004935
2788.500000	0.004934
2788.250000	0.004933
2788.000000	0.004931
2787.750000	0.004927
2787.500000	0.004922
2787.250000	0.004917
2787.000000	0.004911
2786.750000	0.004904
2786.500000	0.004898
2786.250000	0.004891
2786.000000	0.004884
2785.750000	0.004878
2785.500000	0.004872
2785.250000	0.004866
2785.000000	0.004861
2784.750000	0.004857
2784.500000	0.004853

2784.250000	0.004847
2784.000000	0.004840
2783.750000	0.004832
2783.500000	0.004823
2783.250000	0.004815
2783.000000	0.004809
2782.750000	0.004804
2782.500000	0.004802
2782.250000	0.004802
2782.000000	0.004802
2781.750000	0.004801
2781.500000	0.004800
2781.250000	0.004797
2781.000000	0.004794
2780.750000	0.004790
2780.500000	0.004788
2780.250000	0.004785
2780.000000	0.004783
2779.750000	0.004781
2779.500000	0.004779
2779.250000	0.004777
2779.000000	0.004774
2778.750000	0.004770
2778.500000	0.004764
2778.250000	0.004756
2778.000000	0.004744

2777.750000	0.004729
2777.500000	0.004711
2777.250000	0.004690
2777.000000	0.004669
2776.750000	0.004651
2776.500000	0.004636
2776.250000	0.004628
2776.000000	0.004625
2775.750000	0.004627
2775.500000	0.004634
2775.250000	0.004642
2775.000000	0.004649
2774.750000	0.004656
2774.500000	0.004659
2774.250000	0.004660
2774.000000	0.004658
2773.750000	0.004655
2773.500000	0.004651
2773.250000	0.004646
2773.000000	0.004640
2772.750000	0.004634
2772.500000	0.004628
2772.250000	0.004621
2772.000000	0.004615
2771.750000	0.004611
2771.500000	0.004612

2771.250000	0.004616
2771.000000	0.004625
2770.750000	0.004637
2770.500000	0.004650
2770.250000	0.004660
2770.000000	0.004666
2769.750000	0.004666
2769.500000	0.004660
2769.250000	0.004649
2769.000000	0.004635
2768.750000	0.004622
2768.500000	0.004610
2768.250000	0.004600
2768.000000	0.004592
2767.750000	0.004588
2767.500000	0.004585
2767.250000	0.004585
2767.000000	0.004586
2766.750000	0.004589
2766.500000	0.004594
2766.250000	0.004600
2766.000000	0.004605
2765.750000	0.004609
2765.500000	0.004611
2765.250000	0.004612
2765.000000	0.004611

2764.750000	0.004610
2764.500000	0.004609
2764.250000	0.004608
2764.000000	0.004606
2763.750000	0.004605
2763.500000	0.004603
2763.250000	0.004600
2763.000000	0.004597
2762.750000	0.004594
2762.500000	0.004593
2762.250000	0.004593
2762.000000	0.004594
2761.750000	0.004595
2761.500000	0.004594
2761.250000	0.004592
2761.000000	0.004587
2760.750000	0.004580
2760.500000	0.004573
2760.250000	0.004566
2760.000000	0.004560
2759.750000	0.004556
2759.500000	0.004554
2759.250000	0.004554
2759.000000	0.004554
2758.750000	0.004552
2758.500000	0.004549

2758.250000	0.004543
2758.000000	0.004534
2757.750000	0.004523
2757.500000	0.004512
2757.250000	0.004502
2757.000000	0.004494
2756.750000	0.004490
2756.500000	0.004490
2756.250000	0.004492
2756.000000	0.004495
2755.750000	0.004497
2755.500000	0.004496
2755.250000	0.004494
2755.000000	0.004489
2754.750000	0.004483
2754.500000	0.004477
2754.250000	0.004473
2754.000000	0.004470
2753.750000	0.004468
2753.500000	0.004467
2753.250000	0.004464
2753.000000	0.004459
2752.750000	0.004451
2752.500000	0.004439
2752.250000	0.004425
2752.000000	0.004410

2751.750000	0.004396
2751.500000	0.004384
2751.250000	0.004377
2751.000000	0.004374
2750.750000	0.004374
2750.500000	0.004377
2750.250000	0.004380
2750.000000	0.004382
2749.750000	0.004381
2749.500000	0.004378
2749.250000	0.004374
2749.000000	0.004370
2748.750000	0.004370
2748.500000	0.004372
2748.250000	0.004378
2748.000000	0.004386
2747.750000	0.004395
2747.500000	0.004402
2747.250000	0.004408
2747.000000	0.004410
2746.750000	0.004412
2746.500000	0.004413
2746.250000	0.004416
2746.000000	0.004420
2745.750000	0.004426
2745.500000	0.004434

2745.250000	0.004442
2745.000000	0.004451
2744.750000	0.004461
2744.500000	0.004469
2744.250000	0.004477
2744.000000	0.004481
2743.750000	0.004482
2743.500000	0.004477
2743.250000	0.004466
2743.000000	0.004448
2742.750000	0.004425
2742.500000	0.004401
2742.250000	0.004378
2742.000000	0.004360
2741.750000	0.004349
2741.500000	0.004346
2741.250000	0.004348
2741.000000	0.004353
2740.750000	0.004357
2740.500000	0.004359
2740.250000	0.004358
2740.000000	0.004352
2739.750000	0.004345
2739.500000	0.004337
2739.250000	0.004330
2739.000000	0.004326

2738.750000	0.004323
2738.500000	0.004321
2738.250000	0.004320
2738.000000	0.004318
2737.750000	0.004316
2737.500000	0.004315
2737.250000	0.004317
2737.000000	0.004322
2736.750000	0.004329
2736.500000	0.004337
2736.250000	0.004344
2736.000000	0.004349
2735.750000	0.004349
2735.500000	0.004346
2735.250000	0.004339
2735.000000	0.004331
2734.750000	0.004322
2734.500000	0.004313
2734.250000	0.004303
2734.000000	0.004294
2733.750000	0.004283
2733.500000	0.004271
2733.250000	0.004259
2733.000000	0.004248
2732.750000	0.004238
2732.500000	0.004232

2732.250000	0.004230
2732.000000	0.004231
2731.750000	0.004234
2731.500000	0.004237
2731.250000	0.004238
2731.000000	0.004237
2730.750000	0.004232
2730.500000	0.004223
2730.250000	0.004212
2730.000000	0.004201
2729.750000	0.004191
2729.500000	0.004183
2729.250000	0.004179
2729.000000	0.004177
2728.750000	0.004177
2728.500000	0.004176
2728.250000	0.004174
2728.000000	0.004168
2727.750000	0.004159
2727.500000	0.004146
2727.250000	0.004132
2727.000000	0.004119
2726.750000	0.004109
2726.500000	0.004104
2726.250000	0.004104
2726.000000	0.004107

2725.750000	0.004112
2725.500000	0.004118
2725.250000	0.004121
2725.000000	0.004122
2724.750000	0.004120
2724.500000	0.004115
2724.250000	0.004109
2724.000000	0.004100
2723.750000	0.004091
2723.500000	0.004081
2723.250000	0.004070
2723.000000	0.004060
2722.750000	0.004052
2722.500000	0.004046
2722.250000	0.004043
2722.000000	0.004043
2721.750000	0.004045
2721.500000	0.004047
2721.250000	0.004047
2721.000000	0.004046
2720.750000	0.004042
2720.500000	0.004037
2720.250000	0.004031
2720.000000	0.004025
2719.750000	0.004021
2719.500000	0.004020

2719.250000	0.004020
2719.000000	0.004022
2718.750000	0.004024
2718.500000	0.004025
2718.250000	0.004025
2718.000000	0.004024
2717.750000	0.004022
2717.500000	0.004019
2717.250000	0.004017
2717.000000	0.004015
2716.750000	0.004014
2716.500000	0.004012
2716.250000	0.004010
2716.000000	0.004006
2715.750000	0.004001
2715.500000	0.003995
2715.250000	0.003988
2715.000000	0.003980
2714.750000	0.003972
2714.500000	0.003964
2714.250000	0.003956
2714.000000	0.003949
2713.750000	0.003944
2713.500000	0.003940
2713.250000	0.003939
2713.000000	0.003941

2712.750000	0.003944
2712.500000	0.003947
2712.250000	0.003949
2712.000000	0.003949
2711.750000	0.003944
2711.500000	0.003935
2711.250000	0.003924
2711.000000	0.003912
2710.750000	0.003901
2710.500000	0.003892
2710.250000	0.003888
2710.000000	0.003887
2709.750000	0.003889
2709.500000	0.003892
2709.250000	0.003897
2709.000000	0.003901
2708.750000	0.003903
2708.500000	0.003904
2708.250000	0.003903
2708.000000	0.003899
2707.750000	0.003892
2707.500000	0.003883
2707.250000	0.003872
2707.000000	0.003860
2706.750000	0.003849
2706.500000	0.003840

2706.250000	0.003833
2706.000000	0.003829
2705.750000	0.003828
2705.500000	0.003827
2705.250000	0.003828
2705.000000	0.003830
2704.750000	0.003832
2704.500000	0.003835
2704.250000	0.003839
2704.000000	0.003843
2703.750000	0.003846
2703.500000	0.003848
2703.250000	0.003847
2703.000000	0.003843
2702.750000	0.003836
2702.500000	0.003827
2702.250000	0.003816
2702.000000	0.003806
2701.750000	0.003797
2701.500000	0.003791
2701.250000	0.003788
2701.000000	0.003788
2700.750000	0.003789
2700.500000	0.003793
2700.250000	0.003797
2700.000000	0.003801

2699.750000	0.003806
2699.500000	0.003809
2699.250000	0.003813
2699.000000	0.003815
2698.750000	0.003816
2698.500000	0.003817
2698.250000	0.003816
2698.000000	0.003814
2697.750000	0.003811
2697.500000	0.003807
2697.250000	0.003803
2697.000000	0.003798
2696.750000	0.003793
2696.500000	0.003788
2696.250000	0.003784
2696.000000	0.003780
2695.750000	0.003776
2695.500000	0.003771
2695.250000	0.003767
2695.000000	0.003762
2694.750000	0.003758
2694.500000	0.003755
2694.250000	0.003753
2694.000000	0.003753
2693.750000	0.003754
2693.500000	0.003756

2693.250000	0.003759
2693.000000	0.003761
2692.750000	0.003762
2692.500000	0.003761
2692.250000	0.003758
2692.000000	0.003753
2691.750000	0.003747
2691.500000	0.003740
2691.250000	0.003734
2691.000000	0.003729
2690.750000	0.003727
2690.500000	0.003727
2690.250000	0.003729
2690.000000	0.003733
2689.750000	0.003737
2689.500000	0.003742
2689.250000	0.003746
2689.000000	0.003749
2688.750000	0.003749
2688.500000	0.003747
2688.250000	0.003743
2688.000000	0.003736
2687.750000	0.003727
2687.500000	0.003719
2687.250000	0.003711
2687.000000	0.003706

2686.750000	0.003704
2686.500000	0.003705
2686.250000	0.003709
2686.000000	0.003713
2685.750000	0.003716
2685.500000	0.003715
2685.250000	0.003711
2685.000000	0.003704
2684.750000	0.003693
2684.500000	0.003682
2684.250000	0.003671
2684.000000	0.003662
2683.750000	0.003656
2683.500000	0.003651
2683.250000	0.003647
2683.000000	0.003643
2682.750000	0.003639
2682.500000	0.003632
2682.250000	0.003626
2682.000000	0.003619
2681.750000	0.003613
2681.500000	0.003610
2681.250000	0.003608
2681.000000	0.003608
2680.750000	0.003609
2680.500000	0.003611

2680.250000	0.003611
2680.000000	0.003610
2679.750000	0.003607
2679.500000	0.003603
2679.250000	0.003598
2679.000000	0.003592
2678.750000	0.003587
2678.500000	0.003583
2678.250000	0.003580
2678.000000	0.003578
2677.750000	0.003578
2677.500000	0.003580
2677.250000	0.003583
2677.000000	0.003587
2676.750000	0.003591
2676.500000	0.003594
2676.250000	0.003595
2676.000000	0.003593
2675.750000	0.003588
2675.500000	0.003580
2675.250000	0.003570
2675.000000	0.003558
2674.750000	0.003547
2674.500000	0.003538
2674.250000	0.003530
2674.000000	0.003525

2673.750000	0.003522
2673.500000	0.003521
2673.250000	0.003521
2673.000000	0.003522
2672.750000	0.003523
2672.500000	0.003525
2672.250000	0.003526
2672.000000	0.003529
2671.750000	0.003531
2671.500000	0.003534
2671.250000	0.003536
2671.000000	0.003538
2670.750000	0.003537
2670.500000	0.003534
2670.250000	0.003528
2670.000000	0.003522
2669.750000	0.003515
2669.500000	0.003511
2669.250000	0.003510
2669.000000	0.003513
2668.750000	0.003520
2668.500000	0.003530
2668.250000	0.003541
2668.000000	0.003552
2667.750000	0.003560
2667.500000	0.003566

2667.250000	0.003568
2667.000000	0.003568
2666.750000	0.003564
2666.500000	0.003559
2666.250000	0.003552
2666.000000	0.003543
2665.750000	0.003534
2665.500000	0.003526
2665.250000	0.003518
2665.000000	0.003514
2664.750000	0.003512
2664.500000	0.003513
2664.250000	0.003516
2664.000000	0.003519
2663.750000	0.003522
2663.500000	0.003525
2663.250000	0.003526
2663.000000	0.003528
2662.750000	0.003532
2662.500000	0.003537
2662.250000	0.003544
2662.000000	0.003552
2661.750000	0.003558
2661.500000	0.003561
2661.250000	0.003561
2661.000000	0.003559

2660.750000	0.003556
2660.500000	0.003553
2660.250000	0.003553
2660.000000	0.003555
2659.750000	0.003559
2659.500000	0.003563
2659.250000	0.003564
2659.000000	0.003563
2658.750000	0.003557
2658.500000	0.003549
2658.250000	0.003538
2658.000000	0.003528
2657.750000	0.003518
2657.500000	0.003510
2657.250000	0.003505
2657.000000	0.003501
2656.750000	0.003499
2656.500000	0.003496
2656.250000	0.003494
2656.000000	0.003492
2655.750000	0.003489
2655.500000	0.003486
2655.250000	0.003483
2655.000000	0.003481
2654.750000	0.003479
2654.500000	0.003478

2654.250000	0.003477
2654.000000	0.003477
2653.750000	0.003478
2653.500000	0.003478
2653.250000	0.003479
2653.000000	0.003480
2652.750000	0.003482
2652.500000	0.003485
2652.250000	0.003488
2652.000000	0.003490
2651.750000	0.003493
2651.500000	0.003495
2651.250000	0.003496
2651.000000	0.003497
2650.750000	0.003498
2650.500000	0.003498
2650.250000	0.003497
2650.000000	0.003494
2649.750000	0.003489
2649.500000	0.003481
2649.250000	0.003471
2649.000000	0.003458
2648.750000	0.003444
2648.500000	0.003431
2648.250000	0.003420
2648.000000	0.003410

2647.750000	0.003403
2647.500000	0.003398
2647.250000	0.003395
2647.000000	0.003394
2646.750000	0.003393
2646.500000	0.003392
2646.250000	0.003391
2646.000000	0.003388
2645.750000	0.003383
2645.500000	0.003375
2645.250000	0.003365
2645.000000	0.003355
2644.750000	0.003345
2644.500000	0.003338
2644.250000	0.003336
2644.000000	0.003339
2643.750000	0.003344
2643.500000	0.003351
2643.250000	0.003357
2643.000000	0.003359
2642.750000	0.003356
2642.500000	0.003349
2642.250000	0.003337
2642.000000	0.003323
2641.750000	0.003309
2641.500000	0.003298

2641.250000	0.003290
2641.000000	0.003286
2640.750000	0.003286
2640.500000	0.003289
2640.250000	0.003294
2640.000000	0.003296
2639.750000	0.003296
2639.500000	0.003290
2639.250000	0.003279
2639.000000	0.003264
2638.750000	0.003245
2638.500000	0.003228
2638.250000	0.003213
2638.000000	0.003204
2637.750000	0.003201
2637.500000	0.003204
2637.250000	0.003211
2637.000000	0.003221
2636.750000	0.003232
2636.500000	0.003242
2636.250000	0.003251
2636.000000	0.003258
2635.750000	0.003263
2635.500000	0.003266
2635.250000	0.003267
2635.000000	0.003266

2634.750000	0.003262
2634.500000	0.003256
2634.250000	0.003250
2634.000000	0.003245
2633.750000	0.003241
2633.500000	0.003240
2633.250000	0.003242
2633.000000	0.003245
2632.750000	0.003250
2632.500000	0.003255
2632.250000	0.003257
2632.000000	0.003258
2631.750000	0.003254
2631.500000	0.003247
2631.250000	0.003237
2631.000000	0.003224
2630.750000	0.003211
2630.500000	0.003198
2630.250000	0.003187
2630.000000	0.003181
2629.750000	0.003179
2629.500000	0.003181
2629.250000	0.003186
2629.000000	0.003191
2628.750000	0.003195
2628.500000	0.003195

2628.250000	0.003192
2628.000000	0.003187
2627.750000	0.003180
2627.500000	0.003173
2627.250000	0.003167
2627.000000	0.003163
2626.750000	0.003159
2626.500000	0.003154
2626.250000	0.003148
2626.000000	0.003139
2625.750000	0.003129
2625.500000	0.003120
2625.250000	0.003114
2625.000000	0.003112
2624.750000	0.003114
2624.500000	0.003118
2624.250000	0.003122
2624.000000	0.003123
2623.750000	0.003121
2623.500000	0.003115
2623.250000	0.003108
2623.000000	0.003102
2622.750000	0.003101
2622.500000	0.003104
2622.250000	0.003111
2622.000000	0.003119

2621.750000	0.003124
2621.500000	0.003124
2621.250000	0.003117
2621.000000	0.003104
2620.750000	0.003086
2620.500000	0.003068
2620.250000	0.003051
2620.000000	0.003037
2619.750000	0.003026
2619.500000	0.003019
2619.250000	0.003015
2619.000000	0.003014
2618.750000	0.003018
2618.500000	0.003026
2618.250000	0.003039
2618.000000	0.003055
2617.750000	0.003072
2617.500000	0.003085
2617.250000	0.003093
2617.000000	0.003093
2616.750000	0.003088
2616.500000	0.003079
2616.250000	0.003069
2616.000000	0.003062
2615.750000	0.003060
2615.500000	0.003063

2615.250000	0.003069
2615.000000	0.003077
2614.750000	0.003084
2614.500000	0.003088
2614.250000	0.003088
2614.000000	0.003084
2613.750000	0.003077
2613.500000	0.003070
2613.250000	0.003063
2613.000000	0.003057
2612.750000	0.003055
2612.500000	0.003057
2612.250000	0.003062
2612.000000	0.003070
2611.750000	0.003079
2611.500000	0.003088
2611.250000	0.003096
2611.000000	0.003101
2610.750000	0.003104
2610.500000	0.003105
2610.250000	0.003103
2610.000000	0.003098
2609.750000	0.003090
2609.500000	0.003079
2609.250000	0.003065
2609.000000	0.003049

2608.750000	0.003033
2608.500000	0.003018
2608.250000	0.003008
2608.000000	0.003003
2607.750000	0.003002
2607.500000	0.003005
2607.250000	0.003009
2607.000000	0.003010
2606.750000	0.003007
2606.500000	0.002999
2606.250000	0.002986
2606.000000	0.002971
2605.750000	0.002955
2605.500000	0.002942
2605.250000	0.002934
2605.000000	0.002930
2604.750000	0.002931
2604.500000	0.002936
2604.250000	0.002943
2604.000000	0.002951
2603.750000	0.002959
2603.500000	0.002965
2603.250000	0.002968
2603.000000	0.002968
2602.750000	0.002965
2602.500000	0.002960

2602.250000	0.002954
2602.000000	0.002947
2601.750000	0.002941
2601.500000	0.002936
2601.250000	0.002934
2601.000000	0.002933
2600.750000	0.002934
2600.500000	0.002937
2600.250000	0.002939
2600.000000	0.002941
2599.750000	0.002941
2599.500000	0.002940
2599.250000	0.002937
2599.000000	0.002933
2598.750000	0.002927
2598.500000	0.002922
2598.250000	0.002917
2598.000000	0.002914
2597.750000	0.002913
2597.500000	0.002913
2597.250000	0.002915
2597.000000	0.002919
2596.750000	0.002923
2596.500000	0.002928
2596.250000	0.002935
2596.000000	0.002944

2595.750000	0.002955
2595.500000	0.002968
2595.250000	0.002980
2595.000000	0.002991
2594.750000	0.002998
2594.500000	0.003002
2594.250000	0.003001
2594.000000	0.002997
2593.750000	0.002989
2593.500000	0.002980
2593.250000	0.002969
2593.000000	0.002959
2592.750000	0.002947
2592.500000	0.002936
2592.250000	0.002924
2592.000000	0.002914
2591.750000	0.002907
2591.500000	0.002905
2591.250000	0.002910
2591.000000	0.002922
2590.750000	0.002941
2590.500000	0.002964
2590.250000	0.002988
2590.000000	0.003008
2589.750000	0.003021
2589.500000	0.003024

2589.250000	0.003018
2589.000000	0.003004
2588.750000	0.002985
2588.500000	0.002967
2588.250000	0.002953
2588.000000	0.002945
2587.750000	0.002945
2587.500000	0.002951
2587.250000	0.002962
2587.000000	0.002973
2586.750000	0.002984
2586.500000	0.002992
2586.250000	0.002996
2586.000000	0.002996
2585.750000	0.002994
2585.500000	0.002991
2585.250000	0.002987
2585.000000	0.002983
2584.750000	0.002980
2584.500000	0.002980
2584.250000	0.002983
2584.000000	0.002988
2583.750000	0.002995
2583.500000	0.003003
2583.250000	0.003008
2583.000000	0.003009

2582.750000	0.003005
2582.500000	0.002995
2582.250000	0.002982
2582.000000	0.002967
2581.750000	0.002953
2581.500000	0.002943
2581.250000	0.002936
2581.000000	0.002934
2580.750000	0.002934
2580.500000	0.002935
2580.250000	0.002936
2580.000000	0.002934
2579.750000	0.002931
2579.500000	0.002927
2579.250000	0.002922
2579.000000	0.002918
2578.750000	0.002916
2578.500000	0.002917
2578.250000	0.002921
2578.000000	0.002928
2577.750000	0.002934
2577.500000	0.002939
2577.250000	0.002939
2577.000000	0.002932
2576.750000	0.002918
2576.500000	0.002899

2576.250000	0.002879
2576.000000	0.002861
2575.750000	0.002850
2575.500000	0.002848
2575.250000	0.002856
2575.000000	0.002871
2574.750000	0.002887
2574.500000	0.002901
2574.250000	0.002909
2574.000000	0.002911
2573.750000	0.002907
2573.500000	0.002901
2573.250000	0.002895
2573.000000	0.002892
2572.750000	0.002894
2572.500000	0.002898
2572.250000	0.002904
2572.000000	0.002909
2571.750000	0.002911
2571.500000	0.002911
2571.250000	0.002907
2571.000000	0.002900
2570.750000	0.002892
2570.500000	0.002883
2570.250000	0.002873
2570.000000	0.002865

2569.750000	0.002859
2569.500000	0.002857
2569.250000	0.002861
2569.000000	0.002869
2568.750000	0.002881
2568.500000	0.002894
2568.250000	0.002904
2568.000000	0.002909
2567.750000	0.002907
2567.500000	0.002898
2567.250000	0.002883
2567.000000	0.002865
2566.750000	0.002847
2566.500000	0.002830
2566.250000	0.002816
2566.000000	0.002803
2565.750000	0.002793
2565.500000	0.002785
2565.250000	0.002779
2565.000000	0.002777
2564.750000	0.002779
2564.500000	0.002786
2564.250000	0.002798
2564.000000	0.002813
2563.750000	0.002830
2563.500000	0.002848

2563.250000	0.002864
2563.000000	0.002877
2562.750000	0.002888
2562.500000	0.002896
2562.250000	0.002900
2562.000000	0.002903
2561.750000	0.002904
2561.500000	0.002903
2561.250000	0.002901
2561.000000	0.002898
2560.750000	0.002894
2560.500000	0.002890
2560.250000	0.002884
2560.000000	0.002876
2559.750000	0.002867
2559.500000	0.002857
2559.250000	0.002846
2559.000000	0.002835
2558.750000	0.002827
2558.500000	0.002823
2558.250000	0.002823
2558.000000	0.002829
2557.750000	0.002838
2557.500000	0.002851
2557.250000	0.002864
2557.000000	0.002876

2556.750000	0.002886
2556.500000	0.002891
2556.250000	0.002891
2556.000000	0.002888
2555.750000	0.002882
2555.500000	0.002874
2555.250000	0.002865
2555.000000	0.002856
2554.750000	0.002846
2554.500000	0.002836
2554.250000	0.002824
2554.000000	0.002810
2553.750000	0.002794
2553.500000	0.002778
2553.250000	0.002762
2553.000000	0.002748
2552.750000	0.002738
2552.500000	0.002734
2552.250000	0.002736
2552.000000	0.002744
2551.750000	0.002757
2551.500000	0.002773
2551.250000	0.002788
2551.000000	0.002800
2550.750000	0.002808
2550.500000	0.002811

2550.250000	0.002811
2550.000000	0.002810
2549.750000	0.002811
2549.500000	0.002816
2549.250000	0.002825
2549.000000	0.002837
2548.750000	0.002849
2548.500000	0.002858
2548.250000	0.002862
2548.000000	0.002862
2547.750000	0.002856
2547.500000	0.002847
2547.250000	0.002837
2547.000000	0.002828
2546.750000	0.002820
2546.500000	0.002811
2546.250000	0.002803
2546.000000	0.002794
2545.750000	0.002784
2545.500000	0.002775
2545.250000	0.002769
2545.000000	0.002766
2544.750000	0.002767
2544.500000	0.002770
2544.250000	0.002774
2544.000000	0.002777

2543.750000	0.002777
2543.500000	0.002773
2543.250000	0.002767
2543.000000	0.002757
2542.750000	0.002745
2542.500000	0.002732
2542.250000	0.002717
2542.000000	0.002702
2541.750000	0.002687
2541.500000	0.002675
2541.250000	0.002667
2541.000000	0.002665
2540.750000	0.002669
2540.500000	0.002679
2540.250000	0.002690
2540.000000	0.002702
2539.750000	0.002711
2539.500000	0.002715
2539.250000	0.002715
2539.000000	0.002711
2538.750000	0.002705
2538.500000	0.002699
2538.250000	0.002694
2538.000000	0.002692
2537.750000	0.002694
2537.500000	0.002702

2537.250000	0.002714
2537.000000	0.002731
2536.750000	0.002752
2536.500000	0.002773
2536.250000	0.002792
2536.000000	0.002805
2535.750000	0.002811
2535.500000	0.002809
2535.250000	0.002803
2535.000000	0.002794
2534.750000	0.002785
2534.500000	0.002779
2534.250000	0.002775
2534.000000	0.002771
2533.750000	0.002766
2533.500000	0.002759
2533.250000	0.002750
2533.000000	0.002741
2532.750000	0.002737
2532.500000	0.002738
2532.250000	0.002747
2532.000000	0.002761
2531.750000	0.002777
2531.500000	0.002792
2531.250000	0.002801
2531.000000	0.002803

2530.750000	0.002800
2530.500000	0.002792
2530.250000	0.002784
2530.000000	0.002778
2529.750000	0.002777
2529.500000	0.002783
2529.250000	0.002793
2529.000000	0.002807
2528.750000	0.002822
2528.500000	0.002836
2528.250000	0.002846
2528.000000	0.002849
2527.750000	0.002845
2527.500000	0.002832
2527.250000	0.002813
2527.000000	0.002791
2526.750000	0.002768
2526.500000	0.002749
2526.250000	0.002736
2526.000000	0.002730
2525.750000	0.002731
2525.500000	0.002737
2525.250000	0.002743
2525.000000	0.002747
2524.750000	0.002746
2524.500000	0.002741

2524.250000	0.002732
2524.000000	0.002722
2523.750000	0.002712
2523.500000	0.002705
2523.250000	0.002704
2523.000000	0.002709
2522.750000	0.002719
2522.500000	0.002732
2522.250000	0.002744
2522.000000	0.002752
2521.750000	0.002756
2521.500000	0.002754
2521.250000	0.002749
2521.000000	0.002743
2520.750000	0.002741
2520.500000	0.002744
2520.250000	0.002752
2520.000000	0.002763
2519.750000	0.002772
2519.500000	0.002775
2519.250000	0.002767
2519.000000	0.002748
2518.750000	0.002720
2518.500000	0.002687
2518.250000	0.002657
2518.000000	0.002635

2517.750000	0.002624
2517.500000	0.002626
2517.250000	0.002638
2517.000000	0.002656
2516.750000	0.002677
2516.500000	0.002697
2516.250000	0.002712
2516.000000	0.002723
2515.750000	0.002728
2515.500000	0.002728
2515.250000	0.002723
2515.000000	0.002714
2514.750000	0.002702
2514.500000	0.002687
2514.250000	0.002672
2514.000000	0.002658
2513.750000	0.002649
2513.500000	0.002644
2513.250000	0.002645
2513.000000	0.002651
2512.750000	0.002660
2512.500000	0.002670
2512.250000	0.002679
2512.000000	0.002686
2511.750000	0.002689
2511.500000	0.002686

2511.250000	0.002679
2511.000000	0.002668
2510.750000	0.002654
2510.500000	0.002640
2510.250000	0.002629
2510.000000	0.002625
2509.750000	0.002631
2509.500000	0.002648
2509.250000	0.002673
2509.000000	0.002705
2508.750000	0.002736
2508.500000	0.002762
2508.250000	0.002777
2508.000000	0.002780
2507.750000	0.002771
2507.500000	0.002754
2507.250000	0.002734
2507.000000	0.002717
2506.750000	0.002707
2506.500000	0.002706
2506.250000	0.002713
2506.000000	0.002726
2505.750000	0.002741
2505.500000	0.002754
2505.250000	0.002763
2505.000000	0.002768

2504.750000	0.002767
2504.500000	0.002763
2504.250000	0.002758
2504.000000	0.002752
2503.750000	0.002748
2503.500000	0.002745
2503.250000	0.002745
2503.000000	0.002746
2502.750000	0.002747
2502.500000	0.002748
2502.250000	0.002748
2502.000000	0.002746
2501.750000	0.002743
2501.500000	0.002739
2501.250000	0.002734
2501.000000	0.002729
2500.750000	0.002725
2500.500000	0.002722
2500.250000	0.002721
2500.000000	0.002723
2499.750000	0.002726
2499.500000	0.002731
2499.250000	0.002737
2499.000000	0.002742
2498.750000	0.002744
2498.500000	0.002742

2498.250000	0.002735
2498.000000	0.002724
2497.750000	0.002711
2497.500000	0.002699
2497.250000	0.002691
2497.000000	0.002690
2496.750000	0.002696
2496.500000	0.002708
2496.250000	0.002721
2496.000000	0.002733
2495.750000	0.002739
2495.500000	0.002739
2495.250000	0.002734
2495.000000	0.002725
2494.750000	0.002716
2494.500000	0.002710
2494.250000	0.002707
2494.000000	0.002706
2493.750000	0.002706
2493.500000	0.002704
2493.250000	0.002700
2493.000000	0.002693
2492.750000	0.002685
2492.500000	0.002678
2492.250000	0.002674
2492.000000	0.002673

2491.750000	0.002673
2491.500000	0.002672
2491.250000	0.002669
2491.000000	0.002661
2490.750000	0.002651
2490.500000	0.002640
2490.250000	0.002631
2490.000000	0.002629
2489.750000	0.002634
2489.500000	0.002646
2489.250000	0.002664
2489.000000	0.002683
2488.750000	0.002700
2488.500000	0.002711
2488.250000	0.002714
2488.000000	0.002707
2487.750000	0.002692
2487.500000	0.002670
2487.250000	0.002643
2487.000000	0.002614
2486.750000	0.002587
2486.500000	0.002564
2486.250000	0.002547
2486.000000	0.002539
2485.750000	0.002541
2485.500000	0.002554

2485.250000	0.002577
2485.000000	0.002607
2484.750000	0.002643
2484.500000	0.002681
2484.250000	0.002716
2484.000000	0.002746
2483.750000	0.002768
2483.500000	0.002782
2483.250000	0.002785
2483.000000	0.002780
2482.750000	0.002768
2482.500000	0.002751
2482.250000	0.002732
2482.000000	0.002713
2481.750000	0.002694
2481.500000	0.002676
2481.250000	0.002660
2481.000000	0.002645
2480.750000	0.002633
2480.500000	0.002623
2480.250000	0.002616
2480.000000	0.002613
2479.750000	0.002613
2479.500000	0.002616
2479.250000	0.002622
2479.000000	0.002630

2478.750000	0.002638
2478.500000	0.002646
2478.250000	0.002654
2478.000000	0.002663
2477.750000	0.002673
2477.500000	0.002685
2477.250000	0.002699
2477.000000	0.002714
2476.750000	0.002728
2476.500000	0.002738
2476.250000	0.002741
2476.000000	0.002737
2475.750000	0.002725
2475.500000	0.002707
2475.250000	0.002686
2475.000000	0.002665
2474.750000	0.002647
2474.500000	0.002635
2474.250000	0.002627
2474.000000	0.002622
2473.750000	0.002618
2473.500000	0.002613
2473.250000	0.002604
2473.000000	0.002592
2472.750000	0.002577
2472.500000	0.002561

2472.250000	0.002548
2472.000000	0.002539
2471.750000	0.002536
2471.500000	0.002540
2471.250000	0.002552
2471.000000	0.002569
2470.750000	0.002590
2470.500000	0.002614
2470.250000	0.002637
2470.000000	0.002656
2469.750000	0.002669
2469.500000	0.002673
2469.250000	0.002666
2469.000000	0.002650
2468.750000	0.002626
2468.500000	0.002600
2468.250000	0.002576
2468.000000	0.002560
2467.750000	0.002555
2467.500000	0.002563
2467.250000	0.002582
2467.000000	0.002609
2466.750000	0.002639
2466.500000	0.002666
2466.250000	0.002686
2466.000000	0.002696

2465.750000	0.002695
2465.500000	0.002683
2465.250000	0.002664
2465.000000	0.002641
2464.750000	0.002617
2464.500000	0.002595
2464.250000	0.002580
2464.000000	0.002573
2463.750000	0.002574
2463.500000	0.002583
2463.250000	0.002598
2463.000000	0.002616
2462.750000	0.002632
2462.500000	0.002645
2462.250000	0.002651
2462.000000	0.002652
2461.750000	0.002648
2461.500000	0.002642
2461.250000	0.002637
2461.000000	0.002634
2460.750000	0.002633
2460.500000	0.002633
2460.250000	0.002632
2460.000000	0.002630
2459.750000	0.002624
2459.500000	0.002616

2459.250000	0.002607
2459.000000	0.002598
2458.750000	0.002592
2458.500000	0.002589
2458.250000	0.002589
2458.000000	0.002591
2457.750000	0.002597
2457.500000	0.002606
2457.250000	0.002618
2457.000000	0.002633
2456.750000	0.002652
2456.500000	0.002671
2456.250000	0.002687
2456.000000	0.002699
2455.750000	0.002702
2455.500000	0.002698
2455.250000	0.002685
2455.000000	0.002668
2454.750000	0.002650
2454.500000	0.002634
2454.250000	0.002623
2454.000000	0.002617
2453.750000	0.002616
2453.500000	0.002619
2453.250000	0.002623
2453.000000	0.002627

2452.750000	0.002630
2452.500000	0.002629
2452.250000	0.002626
2452.000000	0.002619
2451.750000	0.002610
2451.500000	0.002597
2451.250000	0.002584
2451.000000	0.002570
2450.750000	0.002558
2450.500000	0.002549
2450.250000	0.002542
2450.000000	0.002539
2449.750000	0.002538
2449.500000	0.002540
2449.250000	0.002544
2449.000000	0.002550
2448.750000	0.002559
2448.500000	0.002571
2448.250000	0.002586
2448.000000	0.002603
2447.750000	0.002619
2447.500000	0.002633
2447.250000	0.002641
2447.000000	0.002642
2446.750000	0.002634
2446.500000	0.002619

2446.250000	0.002598
2446.000000	0.002572
2445.750000	0.002547
2445.500000	0.002522
2445.250000	0.002501
2445.000000	0.002484
2444.750000	0.002471
2444.500000	0.002462
2444.250000	0.002456
2444.000000	0.002453
2443.750000	0.002451
2443.500000	0.002452
2443.250000	0.002453
2443.000000	0.002455
2442.750000	0.002455
2442.500000	0.002455
2442.250000	0.002453
2442.000000	0.002452
2441.750000	0.002452
2441.500000	0.002456
2441.250000	0.002464
2441.000000	0.002478
2440.750000	0.002496
2440.500000	0.002517
2440.250000	0.002536
2440.000000	0.002551

2439.750000	0.002560
2439.500000	0.002564
2439.250000	0.002562
2439.000000	0.002557
2438.750000	0.002551
2438.500000	0.002546
2438.250000	0.002543
2438.000000	0.002543
2437.750000	0.002546
2437.500000	0.002552
2437.250000	0.002559
2437.000000	0.002567
2436.750000	0.002575
2436.500000	0.002581
2436.250000	0.002584
2436.000000	0.002584
2435.750000	0.002580
2435.500000	0.002573
2435.250000	0.002565
2435.000000	0.002557
2434.750000	0.002550
2434.500000	0.002545
2434.250000	0.002542
2434.000000	0.002538
2433.750000	0.002534
2433.500000	0.002528

2433.250000	0.002519
2433.000000	0.002509
2432.750000	0.002498
2432.500000	0.002487
2432.250000	0.002479
2432.000000	0.002473
2431.750000	0.002472
2431.500000	0.002476
2431.250000	0.002485
2431.000000	0.002498
2430.750000	0.002515
2430.500000	0.002534
2430.250000	0.002552
2430.000000	0.002568
2429.750000	0.002580
2429.500000	0.002587
2429.250000	0.002588
2429.000000	0.002585
2428.750000	0.002577
2428.500000	0.002565
2428.250000	0.002552
2428.000000	0.002537
2427.750000	0.002523
2427.500000	0.002511
2427.250000	0.002504
2427.000000	0.002502

2426.750000	0.002502
2426.500000	0.002504
2426.250000	0.002503
2426.000000	0.002497
2425.750000	0.002486
2425.500000	0.002470
2425.250000	0.002452
2425.000000	0.002437
2424.750000	0.002429
2424.500000	0.002428
2424.250000	0.002434
2424.000000	0.002444
2423.750000	0.002453
2423.500000	0.002458
2423.250000	0.002455
2423.000000	0.002446
2422.750000	0.002432
2422.500000	0.002416
2422.250000	0.002401
2422.000000	0.002391
2421.750000	0.002386
2421.500000	0.002387
2421.250000	0.002392
2421.000000	0.002400
2420.750000	0.002410
2420.500000	0.002419

2420.250000	0.002425
2420.000000	0.002427
2419.750000	0.002423
2419.500000	0.002416
2419.250000	0.002407
2419.000000	0.002399
2418.750000	0.002395
2418.500000	0.002397
2418.250000	0.002404
2418.000000	0.002416
2417.750000	0.002428
2417.500000	0.002440
2417.250000	0.002448
2417.000000	0.002452
2416.750000	0.002455
2416.500000	0.002458
2416.250000	0.002462
2416.000000	0.002468
2415.750000	0.002476
2415.500000	0.002484
2415.250000	0.002491
2415.000000	0.002495
2414.750000	0.002496
2414.500000	0.002495
2414.250000	0.002493
2414.000000	0.002490

2413.750000	0.002486
2413.500000	0.002482
2413.250000	0.002476
2413.000000	0.002470
2412.750000	0.002464
2412.500000	0.002458
2412.250000	0.002453
2412.000000	0.002448
2411.750000	0.002445
2411.500000	0.002441
2411.250000	0.002437
2411.000000	0.002434
2410.750000	0.002431
2410.500000	0.002428
2410.250000	0.002428
2410.000000	0.002428
2409.750000	0.002430
2409.500000	0.002433
2409.250000	0.002436
2409.000000	0.002439
2408.750000	0.002441
2408.500000	0.002442
2408.250000	0.002443
2408.000000	0.002441
2407.750000	0.002436
2407.500000	0.002426

2407.250000	0.002413
2407.000000	0.002398
2406.750000	0.002384
2406.500000	0.002374
2406.250000	0.002369
2406.000000	0.002372
2405.750000	0.002380
2405.500000	0.002393
2405.250000	0.002406
2405.000000	0.002416
2404.750000	0.002421
2404.500000	0.002420
2404.250000	0.002414
2404.000000	0.002405
2403.750000	0.002395
2403.500000	0.002386
2403.250000	0.002380
2403.000000	0.002378
2402.750000	0.002380
2402.500000	0.002385
2402.250000	0.002392
2402.000000	0.002397
2401.750000	0.002400
2401.500000	0.002397
2401.250000	0.002389
2401.000000	0.002378

2400.750000	0.002364
2400.500000	0.002353
2400.250000	0.002346
2400.000000	0.002346
2399.750000	0.002354
2399.500000	0.002367
2399.250000	0.002384
2399.000000	0.002401
2398.750000	0.002416
2398.500000	0.002427
2398.250000	0.002433
2398.000000	0.002434
2397.750000	0.002429
2397.500000	0.002419
2397.250000	0.002405
2397.000000	0.002388
2396.750000	0.002369
2396.500000	0.002353
2396.250000	0.002341
2396.000000	0.002337
2395.750000	0.002342
2395.500000	0.002357
2395.250000	0.002380
2395.000000	0.002407
2394.750000	0.002435
2394.500000	0.002458

2394.250000	0.002475
2394.000000	0.002484
2393.750000	0.002487
2393.500000	0.002485
2393.250000	0.002481
2393.000000	0.002477
2392.750000	0.002474
2392.500000	0.002470
2392.250000	0.002466
2392.000000	0.002458
2391.750000	0.002447
2391.500000	0.002432
2391.250000	0.002415
2391.000000	0.002399
2390.750000	0.002387
2390.500000	0.002379
2390.250000	0.002377
2390.000000	0.002379
2389.750000	0.002386
2389.500000	0.002395
2389.250000	0.002404
2389.000000	0.002413
2388.750000	0.002422
2388.500000	0.002429
2388.250000	0.002436
2388.000000	0.002440

2387.750000	0.002442
2387.500000	0.002440
2387.250000	0.002435
2387.000000	0.002426
2386.750000	0.002417
2386.500000	0.002408
2386.250000	0.002400
2386.000000	0.002397
2385.750000	0.002397
2385.500000	0.002402
2385.250000	0.002412
2385.000000	0.002425
2384.750000	0.002439
2384.500000	0.002453
2384.250000	0.002465
2384.000000	0.002473
2383.750000	0.002477
2383.500000	0.002475
2383.250000	0.002469
2383.000000	0.002462
2382.750000	0.002454
2382.500000	0.002448
2382.250000	0.002445
2382.000000	0.002444
2381.750000	0.002443
2381.500000	0.002441

2381.250000	0.002436
2381.000000	0.002428
2380.750000	0.002418
2380.500000	0.002407
2380.250000	0.002399
2380.000000	0.002395
2379.750000	0.002395
2379.500000	0.002400
2379.250000	0.002408
2379.000000	0.002416
2378.750000	0.002422
2378.500000	0.002424
2378.250000	0.002422
2378.000000	0.002417
2377.750000	0.002410
2377.500000	0.002405
2377.250000	0.002403
2377.000000	0.002406
2376.750000	0.002412
2376.500000	0.002419
2376.250000	0.002424
2376.000000	0.002426
2375.750000	0.002422
2375.500000	0.002412
2375.250000	0.002400
2375.000000	0.002390

2374.750000	0.002386
2374.500000	0.002388
2374.250000	0.002397
2374.000000	0.002408
2373.750000	0.002419
2373.500000	0.002424
2373.250000	0.002420
2373.000000	0.002410
2372.750000	0.002398
2372.500000	0.002388
2372.250000	0.002385
2372.000000	0.002392
2371.750000	0.002408
2371.500000	0.002432
2371.250000	0.002462
2371.000000	0.002494
2370.750000	0.002526
2370.500000	0.002556
2370.250000	0.002580
2370.000000	0.002596
2369.750000	0.002596
2369.500000	0.002577
2369.250000	0.002541
2369.000000	0.002491
2368.750000	0.002437
2368.500000	0.002388

2368.250000	0.002355
2368.000000	0.002342
2367.750000	0.002347
2367.500000	0.002365
2367.250000	0.002386
2367.000000	0.002398
2366.750000	0.002395
2366.500000	0.002379
2366.250000	0.002356
2366.000000	0.002334
2365.750000	0.002319
2365.500000	0.002315
2365.250000	0.002322
2365.000000	0.002338
2364.750000	0.002357
2364.500000	0.002372
2364.250000	0.002379
2364.000000	0.002378
2363.750000	0.002372
2363.500000	0.002363
2363.250000	0.002354
2363.000000	0.002350
2362.750000	0.002354
2362.500000	0.002371
2362.250000	0.002404
2362.000000	0.002450

2361.750000	0.002507
2361.500000	0.002567
2361.250000	0.002624
2361.000000	0.002669
2360.750000	0.002698
2360.500000	0.002707
2360.250000	0.002698
2360.000000	0.002673
2359.750000	0.002637
2359.500000	0.002596
2359.250000	0.002552
2359.000000	0.002509
2358.750000	0.002464
2358.500000	0.002418
2358.250000	0.002373
2358.000000	0.002334
2357.750000	0.002307
2357.500000	0.002296
2357.250000	0.002301
2357.000000	0.002321
2356.750000	0.002355
2356.500000	0.002399
2356.250000	0.002447
2356.000000	0.002490
2355.750000	0.002522
2355.500000	0.002537

2355.250000	0.002537
2355.000000	0.002526
2354.750000	0.002508
2354.500000	0.002486
2354.250000	0.002464
2354.000000	0.002445
2353.750000	0.002432
2353.500000	0.002429
2353.250000	0.002434
2353.000000	0.002445
2352.750000	0.002458
2352.500000	0.002473
2352.250000	0.002485
2352.000000	0.002494
2351.750000	0.002500
2351.500000	0.002504
2351.250000	0.002508
2351.000000	0.002514
2350.750000	0.002519
2350.500000	0.002521
2350.250000	0.002518
2350.000000	0.002510
2349.750000	0.002501
2349.500000	0.002493
2349.250000	0.002490
2349.000000	0.002491

2348.750000	0.002494
2348.500000	0.002500
2348.250000	0.002508
2348.000000	0.002518
2347.750000	0.002530
2347.500000	0.002542
2347.250000	0.002552
2347.000000	0.002560
2346.750000	0.002566
2346.500000	0.002571
2346.250000	0.002573
2346.000000	0.002573
2345.750000	0.002568
2345.500000	0.002556
2345.250000	0.002539
2345.000000	0.002523
2344.750000	0.002511
2344.500000	0.002507
2344.250000	0.002510
2344.000000	0.002518
2343.750000	0.002526
2343.500000	0.002533
2343.250000	0.002538
2343.000000	0.002544
2342.750000	0.002552
2342.500000	0.002563

2342.250000	0.002573
2342.000000	0.002579
2341.750000	0.002578
2341.500000	0.002573
2341.250000	0.002566
2341.000000	0.002563
2340.750000	0.002566
2340.500000	0.002574
2340.250000	0.002585
2340.000000	0.002595
2339.750000	0.002603
2339.500000	0.002606
2339.250000	0.002605
2339.000000	0.002598
2338.750000	0.002589
2338.500000	0.002580
2338.250000	0.002577
2338.000000	0.002586
2337.750000	0.002607
2337.500000	0.002637
2337.250000	0.002667
2337.000000	0.002687
2336.750000	0.002689
2336.500000	0.002674
2336.250000	0.002644
2336.000000	0.002611

2335.750000	0.002583
2335.500000	0.002564
2335.250000	0.002557
2335.000000	0.002560
2334.750000	0.002572
2334.500000	0.002590
2334.250000	0.002615
2334.000000	0.002645
2333.750000	0.002676
2333.500000	0.002702
2333.250000	0.002719
2333.000000	0.002724
2332.750000	0.002717
2332.500000	0.002701
2332.250000	0.002682
2332.000000	0.002665
2331.750000	0.002656
2331.500000	0.002654
2331.250000	0.002658
2331.000000	0.002666
2330.750000	0.002672
2330.500000	0.002675
2330.250000	0.002676
2330.000000	0.002678
2329.750000	0.002684
2329.500000	0.002695

2329.250000	0.002711
2329.000000	0.002729
2328.750000	0.002747
2328.500000	0.002764
2328.250000	0.002780
2328.000000	0.002796
2327.750000	0.002811
2327.500000	0.002827
2327.250000	0.002843
2327.000000	0.002861
2326.750000	0.002887
2326.500000	0.002922
2326.250000	0.002971
2326.000000	0.003031
2325.750000	0.003097
2325.500000	0.003161
2325.250000	0.003214
2325.000000	0.003254
2324.750000	0.003281
2324.500000	0.003298
2324.250000	0.003310
2324.000000	0.003319
2323.750000	0.003322
2323.500000	0.003316
2323.250000	0.003302
2323.000000	0.003279

2322.750000	0.003252
2322.500000	0.003226
2322.250000	0.003202
2322.000000	0.003180
2321.750000	0.003157
2321.500000	0.003128
2321.250000	0.003094
2321.000000	0.003058
2320.750000	0.003027
2320.500000	0.003006
2320.250000	0.002995
2320.000000	0.002993
2319.750000	0.002993
2319.500000	0.002992
2319.250000	0.002985
2319.000000	0.002976
2318.750000	0.002968
2318.500000	0.002965
2318.250000	0.002969
2318.000000	0.002977
2317.750000	0.002984
2317.500000	0.002988
2317.250000	0.002988
2317.000000	0.002988
2316.750000	0.002991
2316.500000	0.003001

2316.250000	0.003016
2316.000000	0.003029
2315.750000	0.003032
2315.500000	0.003020
2315.250000	0.002993
2315.000000	0.002957
2314.750000	0.002923
2314.500000	0.002899
2314.250000	0.002891
2314.000000	0.002896
2313.750000	0.002910
2313.500000	0.002925
2313.250000	0.002937
2313.000000	0.002944
2312.750000	0.002947
2312.500000	0.002950
2312.250000	0.002951
2312.000000	0.002950
2311.750000	0.002942
2311.500000	0.002923
2311.250000	0.002896
2311.000000	0.002863
2310.750000	0.002832
2310.500000	0.002811
2310.250000	0.002801
2310.000000	0.002801

2309.750000	0.002806
2309.500000	0.002808
2309.250000	0.002804
2309.000000	0.002794
2308.750000	0.002782
2308.500000	0.002773
2308.250000	0.002771
2308.000000	0.002779
2307.750000	0.002793
2307.500000	0.002807
2307.250000	0.002819
2307.000000	0.002826
2306.750000	0.002828
2306.500000	0.002829
2306.250000	0.002832
2306.000000	0.002839
2305.750000	0.002847
2305.500000	0.002853
2305.250000	0.002852
2305.000000	0.002843
2304.750000	0.002828
2304.500000	0.002811
2304.250000	0.002797
2304.000000	0.002792
2303.750000	0.002795
2303.500000	0.002805

2303.250000	0.002816
2303.000000	0.002823
2302.750000	0.002822
2302.500000	0.002812
2302.250000	0.002796
2302.000000	0.002778
2301.750000	0.002762
2301.500000	0.002751
2301.250000	0.002745
2301.000000	0.002743
2300.750000	0.002743
2300.500000	0.002744
2300.250000	0.002746
2300.000000	0.002749
2299.750000	0.002754
2299.500000	0.002761
2299.250000	0.002769
2299.000000	0.002775
2298.750000	0.002776
2298.500000	0.002772
2298.250000	0.002763
2298.000000	0.002753
2297.750000	0.002746
2297.500000	0.002746
2297.250000	0.002754
2297.000000	0.002769

2296.750000	0.002788
2296.500000	0.002804
2296.250000	0.002815
2296.000000	0.002817
2295.750000	0.002811
2295.500000	0.002802
2295.250000	0.002791
2295.000000	0.002783
2294.750000	0.002779
2294.500000	0.002779
2294.250000	0.002782
2294.000000	0.002788
2293.750000	0.002796
2293.500000	0.002808
2293.250000	0.002823
2293.000000	0.002841
2292.750000	0.002859
2292.500000	0.002874
2292.250000	0.002882
2292.000000	0.002882
2291.750000	0.002874
2291.500000	0.002863
2291.250000	0.002851
2291.000000	0.002844
2290.750000	0.002843
2290.500000	0.002849

2290.250000	0.002856
2290.000000	0.002862
2289.750000	0.002862
2289.500000	0.002856
2289.250000	0.002847
2289.000000	0.002839
2288.750000	0.002834
2288.500000	0.002836
2288.250000	0.002844
2288.000000	0.002858
2287.750000	0.002872
2287.500000	0.002885
2287.250000	0.002893
2287.000000	0.002898
2286.750000	0.002899
2286.500000	0.002898
2286.250000	0.002896
2286.000000	0.002894
2285.750000	0.002890
2285.500000	0.002887
2285.250000	0.002884
2285.000000	0.002883
2284.750000	0.002885
2284.500000	0.002891
2284.250000	0.002898
2284.000000	0.002906

2283.750000	0.002910
2283.500000	0.002909
2283.250000	0.002900
2283.000000	0.002885
2282.750000	0.002867
2282.500000	0.002850
2282.250000	0.002835
2282.000000	0.002824
2281.750000	0.002817
2281.500000	0.002813
2281.250000	0.002809
2281.000000	0.002807
2280.750000	0.002806
2280.500000	0.002808
2280.250000	0.002812
2280.000000	0.002820
2279.750000	0.002827
2279.500000	0.002833
2279.250000	0.002834
2279.000000	0.002830
2278.750000	0.002820
2278.500000	0.002808
2278.250000	0.002799
2278.000000	0.002796
2277.750000	0.002801
2277.500000	0.002816

2277.250000	0.002838
2277.000000	0.002863
2276.750000	0.002885
2276.500000	0.002901
2276.250000	0.002907
2276.000000	0.002900
2275.750000	0.002882
2275.500000	0.002855
2275.250000	0.002822
2275.000000	0.002788
2274.750000	0.002756
2274.500000	0.002729
2274.250000	0.002709
2274.000000	0.002696
2273.750000	0.002688
2273.500000	0.002682
2273.250000	0.002678
2273.000000	0.002673
2272.750000	0.002669
2272.500000	0.002668
2272.250000	0.002673
2272.000000	0.002686
2271.750000	0.002707
2271.500000	0.002735
2271.250000	0.002763
2271.000000	0.002787

2270.750000	0.002801
2270.500000	0.002802
2270.250000	0.002791
2270.000000	0.002772
2269.750000	0.002749
2269.500000	0.002728
2269.250000	0.002713
2269.000000	0.002706
2268.750000	0.002705
2268.500000	0.002708
2268.250000	0.002713
2268.000000	0.002716
2267.750000	0.002718
2267.500000	0.002717
2267.250000	0.002718
2267.000000	0.002720
2266.750000	0.002727
2266.500000	0.002738
2266.250000	0.002752
2266.000000	0.002769
2265.750000	0.002786
2265.500000	0.002802
2265.250000	0.002814
2265.000000	0.002820
2264.750000	0.002818
2264.500000	0.002808

2264.250000	0.002790
2264.000000	0.002766
2263.750000	0.002739
2263.500000	0.002712
2263.250000	0.002688
2263.000000	0.002670
2262.750000	0.002658
2262.500000	0.002650
2262.250000	0.002644
2262.000000	0.002638
2261.750000	0.002632
2261.500000	0.002626
2261.250000	0.002624
2261.000000	0.002628
2260.750000	0.002641
2260.500000	0.002664
2260.250000	0.002695
2260.000000	0.002729
2259.750000	0.002763
2259.500000	0.002792
2259.250000	0.002814
2259.000000	0.002829
2258.750000	0.002836
2258.500000	0.002839
2258.250000	0.002838
2258.000000	0.002833

2257.750000	0.002827
2257.500000	0.002821
2257.250000	0.002815
2257.000000	0.002813
2256.750000	0.002815
2256.500000	0.002821
2256.250000	0.002830
2256.000000	0.002837
2255.750000	0.002839
2255.500000	0.002832
2255.250000	0.002813
2255.000000	0.002786
2254.750000	0.002752
2254.500000	0.002717
2254.250000	0.002686
2254.000000	0.002663
2253.750000	0.002648
2253.500000	0.002644
2253.250000	0.002646
2253.000000	0.002654
2252.750000	0.002665
2252.500000	0.002678
2252.250000	0.002691
2252.000000	0.002704
2251.750000	0.002716
2251.500000	0.002727

2251.250000	0.002738
2251.000000	0.002748
2250.750000	0.002759
2250.500000	0.002768
2250.250000	0.002775
2250.000000	0.002778
2249.750000	0.002776
2249.500000	0.002769
2249.250000	0.002756
2249.000000	0.002740
2248.750000	0.002725
2248.500000	0.002712
2248.250000	0.002706
2248.000000	0.002706
2247.750000	0.002711
2247.500000	0.002721
2247.250000	0.002731
2247.000000	0.002741
2246.750000	0.002750
2246.500000	0.002760
2246.250000	0.002773
2246.000000	0.002790
2245.750000	0.002815
2245.500000	0.002846
2245.250000	0.002881
2245.000000	0.002918

2244.750000	0.002952
2244.500000	0.002978
2244.250000	0.002993
2244.000000	0.002997
2243.750000	0.002987
2243.500000	0.002965
2243.250000	0.002933
2243.000000	0.002893
2242.750000	0.002851
2242.500000	0.002810
2242.250000	0.002775
2242.000000	0.002750
2241.750000	0.002738
2241.500000	0.002740
2241.250000	0.002754
2241.000000	0.002779
2240.750000	0.002811
2240.500000	0.002846
2240.250000	0.002881
2240.000000	0.002914
2239.750000	0.002941
2239.500000	0.002960
2239.250000	0.002969
2239.000000	0.002965
2238.750000	0.002947
2238.500000	0.002913

2238.250000	0.002865
2238.000000	0.002808
2237.750000	0.002745
2237.500000	0.002684
2237.250000	0.002632
2237.000000	0.002595
2236.750000	0.002577
2236.500000	0.002579
2236.250000	0.002600
2236.000000	0.002635
2235.750000	0.002678
2235.500000	0.002722
2235.250000	0.002759
2235.000000	0.002787
2234.750000	0.002802
2234.500000	0.002807
2234.250000	0.002805
2234.000000	0.002801
2233.750000	0.002798
2233.500000	0.002800
2233.250000	0.002807
2233.000000	0.002816
2232.750000	0.002826
2232.500000	0.002831
2232.250000	0.002832
2232.000000	0.002826

2231.750000	0.002815
2231.500000	0.002801
2231.250000	0.002787
2231.000000	0.002775
2230.750000	0.002766
2230.500000	0.002762
2230.250000	0.002763
2230.000000	0.002769
2229.750000	0.002783
2229.500000	0.002803
2229.250000	0.002828
2229.000000	0.002855
2228.750000	0.002880
2228.500000	0.002896
2228.250000	0.002898
2228.000000	0.002884
2227.750000	0.002855
2227.500000	0.002815
2227.250000	0.002773
2227.000000	0.002737
2226.750000	0.002714
2226.500000	0.002708
2226.250000	0.002719
2226.000000	0.002741
2225.750000	0.002767
2225.500000	0.002790

2225.250000	0.002803
2225.000000	0.002806
2224.750000	0.002800
2224.500000	0.002788
2224.250000	0.002777
2224.000000	0.002771
2223.750000	0.002773
2223.500000	0.002783
2223.250000	0.002798
2223.000000	0.002812
2222.750000	0.002822
2222.500000	0.002825
2222.250000	0.002819
2222.000000	0.002805
2221.750000	0.002786
2221.500000	0.002765
2221.250000	0.002745
2221.000000	0.002730
2220.750000	0.002721
2220.500000	0.002721
2220.250000	0.002732
2220.000000	0.002754
2219.750000	0.002787
2219.500000	0.002827
2219.250000	0.002869
2219.000000	0.002907

2218.750000	0.002933
2218.500000	0.002943
2218.250000	0.002934
2218.000000	0.002908
2217.750000	0.002872
2217.500000	0.002833
2217.250000	0.002802
2217.000000	0.002784
2216.750000	0.002781
2216.500000	0.002790
2216.250000	0.002806
2216.000000	0.002819
2215.750000	0.002822
2215.500000	0.002810
2215.250000	0.002783
2215.000000	0.002745
2214.750000	0.002706
2214.500000	0.002673
2214.250000	0.002657
2214.000000	0.002660
2213.750000	0.002686
2213.500000	0.002731
2213.250000	0.002788
2213.000000	0.002849
2212.750000	0.002904
2212.500000	0.002945

2212.250000	0.002967
2212.000000	0.002969
2211.750000	0.002953
2211.500000	0.002925
2211.250000	0.002892
2211.000000	0.002861
2210.750000	0.002839
2210.500000	0.002829
2210.250000	0.002828
2210.000000	0.002831
2209.750000	0.002832
2209.500000	0.002823
2209.250000	0.002800
2209.000000	0.002762
2208.750000	0.002713
2208.500000	0.002663
2208.250000	0.002622
2208.000000	0.002599
2207.750000	0.002603
2207.500000	0.002634
2207.250000	0.002686
2207.000000	0.002750
2206.750000	0.002813
2206.500000	0.002863
2206.250000	0.002891
2206.000000	0.002895

2205.750000	0.002876
2205.500000	0.002843
2205.250000	0.002804
2205.000000	0.002770
2204.750000	0.002747
2204.500000	0.002742
2204.250000	0.002757
2204.000000	0.002791
2203.750000	0.002839
2203.500000	0.002896
2203.250000	0.002954
2203.000000	0.003005
2202.750000	0.003042
2202.500000	0.003057
2202.250000	0.003048
2202.000000	0.003018
2201.750000	0.002970
2201.500000	0.002912
2201.250000	0.002853
2201.000000	0.002798
2200.750000	0.002753
2200.500000	0.002719
2200.250000	0.002696
2200.000000	0.002684
2199.750000	0.002682
2199.500000	0.002691

2199.250000	0.002711
2199.000000	0.002739
2198.750000	0.002773
2198.500000	0.002807
2198.250000	0.002834
2198.000000	0.002851
2197.750000	0.002853
2197.500000	0.002841
2197.250000	0.002820
2197.000000	0.002794
2196.750000	0.002769
2196.500000	0.002749
2196.250000	0.002737
2196.000000	0.002735
2195.750000	0.002743
2195.500000	0.002762
2195.250000	0.002791
2195.000000	0.002827
2194.750000	0.002868
2194.500000	0.002905
2194.250000	0.002932
2194.000000	0.002943
2193.750000	0.002938
2193.500000	0.002918
2193.250000	0.002892
2193.000000	0.002869

2192.750000	0.002859
2192.500000	0.002866
2192.250000	0.002891
2192.000000	0.002927
2191.750000	0.002967
2191.500000	0.003002
2191.250000	0.003026
2191.000000	0.003038
2190.750000	0.003041
2190.500000	0.003040
2190.250000	0.003039
2190.000000	0.003041
2189.750000	0.003046
2189.500000	0.003051
2189.250000	0.003052
2189.000000	0.003048
2188.750000	0.003038
2188.500000	0.003023
2188.250000	0.003010
2188.000000	0.003004
2187.750000	0.003008
2187.500000	0.003027
2187.250000	0.003059
2187.000000	0.003102
2186.750000	0.003147
2186.500000	0.003187

2186.250000	0.003214
2186.000000	0.003221
2185.750000	0.003205
2185.500000	0.003168
2185.250000	0.003115
2185.000000	0.003053
2184.750000	0.002994
2184.500000	0.002946
2184.250000	0.002916
2184.000000	0.002908
2183.750000	0.002922
2183.500000	0.002954
2183.250000	0.002998
2183.000000	0.003047
2182.750000	0.003090
2182.500000	0.003120
2182.250000	0.003132
2182.000000	0.003119
2181.750000	0.003084
2181.500000	0.003030
2181.250000	0.002965
2181.000000	0.002902
2180.750000	0.002852
2180.500000	0.002827
2180.250000	0.002834
2180.000000	0.002875

2179.750000	0.002944
2179.500000	0.003031
2179.250000	0.003119
2179.000000	0.003195
2178.750000	0.003244
2178.500000	0.003260
2178.250000	0.003243
2178.000000	0.003200
2177.750000	0.003145
2177.500000	0.003091
2177.250000	0.003052
2177.000000	0.003034
2176.750000	0.003038
2176.500000	0.003057
2176.250000	0.003080
2176.000000	0.003097
2175.750000	0.003101
2175.500000	0.003087
2175.250000	0.003058
2175.000000	0.003018
2174.750000	0.002972
2174.500000	0.002928
2174.250000	0.002891
2174.000000	0.002862
2173.750000	0.002847
2173.500000	0.002847

2173.250000	0.002864
2173.000000	0.002901
2172.750000	0.002953
2172.500000	0.003017
2172.250000	0.003085
2172.000000	0.003145
2171.750000	0.003191
2171.500000	0.003214
2171.250000	0.003215
2171.000000	0.003195
2170.750000	0.003164
2170.500000	0.003129
2170.250000	0.003101
2170.000000	0.003087
2169.750000	0.003093
2169.500000	0.003120
2169.250000	0.003167
2169.000000	0.003229
2168.750000	0.003299
2168.500000	0.003367
2168.250000	0.003425
2168.000000	0.003464
2167.750000	0.003482
2167.500000	0.003476
2167.250000	0.003452
2167.000000	0.003415

2166.750000	0.003371
2166.500000	0.003328
2166.250000	0.003288
2166.000000	0.003256
2165.750000	0.003234
2165.500000	0.003222
2165.250000	0.003223
2165.000000	0.003240
2164.750000	0.003272
2164.500000	0.003322
2164.250000	0.003387
2164.000000	0.003465
2163.750000	0.003551
2163.500000	0.003641
2163.250000	0.003732
2163.000000	0.003819
2162.750000	0.003900
2162.500000	0.003969
2162.250000	0.004022
2162.000000	0.004054
2161.750000	0.004064
2161.500000	0.004053
2161.250000	0.004022
2161.000000	0.003974
2160.750000	0.003911
2160.500000	0.003829

2160.250000	0.003723
2160.000000	0.003585
2159.750000	0.003412
2159.500000	0.003204
2159.250000	0.002974
2159.000000	0.002738
2158.750000	0.002519
2158.500000	0.002334
2158.250000	0.002193
2158.000000	0.002096
2157.750000	0.002032
2157.500000	0.001990
2157.250000	0.001957
2157.000000	0.001929
2156.750000	0.001908
2156.500000	0.001902
2156.250000	0.001918
2156.000000	0.001961
2155.750000	0.002032
2155.500000	0.002124
2155.250000	0.002228
2155.000000	0.002333
2154.750000	0.002427
2154.500000	0.002503
2154.250000	0.002556
2154.000000	0.002586

2153.750000	0.002593
2153.500000	0.002580
2153.250000	0.002548
2153.000000	0.002501
2152.750000	0.002443
2152.500000	0.002379
2152.250000	0.002316
2152.000000	0.002263
2151.750000	0.002226
2151.500000	0.002211
2151.250000	0.002219
2151.000000	0.002248
2150.750000	0.002291
2150.500000	0.002340
2150.250000	0.002384
2150.000000	0.002420
2149.750000	0.002443
2149.500000	0.002455
2149.250000	0.002461
2149.000000	0.002465
2148.750000	0.002469
2148.500000	0.002471
2148.250000	0.002470
2148.000000	0.002461
2147.750000	0.002442
2147.500000	0.002415

2147.250000	0.002384
2147.000000	0.002354
2146.750000	0.002333
2146.500000	0.002322
2146.250000	0.002322
2146.000000	0.002328
2145.750000	0.002334
2145.500000	0.002336
2145.250000	0.002331
2145.000000	0.002319
2144.750000	0.002303
2144.500000	0.002285
2144.250000	0.002268
2144.000000	0.002254
2143.750000	0.002240
2143.500000	0.002228
2143.250000	0.002218
2143.000000	0.002210
2142.750000	0.002208
2142.500000	0.002213
2142.250000	0.002225
2142.000000	0.002241
2141.750000	0.002255
2141.500000	0.002264
2141.250000	0.002262
2141.000000	0.002251

2140.750000	0.002231
2140.500000	0.002208
2140.250000	0.002188
2140.000000	0.002174
2139.750000	0.002167
2139.500000	0.002166
2139.250000	0.002168
2139.000000	0.002171
2138.750000	0.002173
2138.500000	0.002176
2138.250000	0.002182
2138.000000	0.002194
2137.750000	0.002215
2137.500000	0.002242
2137.250000	0.002273
2137.000000	0.002301
2136.750000	0.002320
2136.500000	0.002324
2136.250000	0.002312
2136.000000	0.002283
2135.750000	0.002243
2135.500000	0.002197
2135.250000	0.002150
2135.000000	0.002106
2134.750000	0.002065
2134.500000	0.002026

2134.250000	0.001989
2134.000000	0.001953
2133.750000	0.001922
2133.500000	0.001899
2133.250000	0.001890
2133.000000	0.001899
2132.750000	0.001925
2132.500000	0.001966
2132.250000	0.002014
2132.000000	0.002061
2131.750000	0.002100
2131.500000	0.002125
2131.250000	0.002134
2131.000000	0.002128
2130.750000	0.002112
2130.500000	0.002089
2130.250000	0.002064
2130.000000	0.002042
2129.750000	0.002027
2129.500000	0.002022
2129.250000	0.002029
2129.000000	0.002051
2128.750000	0.002088
2128.500000	0.002137
2128.250000	0.002194
2128.000000	0.002252

2127.750000	0.002303
2127.500000	0.002341
2127.250000	0.002361
2127.000000	0.002361
2126.750000	0.002341
2126.500000	0.002305
2126.250000	0.002258
2126.000000	0.002206
2125.750000	0.002156
2125.500000	0.002115
2125.250000	0.002087
2125.000000	0.002078
2124.750000	0.002088
2124.500000	0.002117
2124.250000	0.002161
2124.000000	0.002215
2123.750000	0.002275
2123.500000	0.002334
2123.250000	0.002390
2123.000000	0.002439
2122.750000	0.002478
2122.500000	0.002502
2122.250000	0.002510
2122.000000	0.002499
2121.750000	0.002466
2121.500000	0.002416

2121.250000	0.002354
2121.000000	0.002288
2120.750000	0.002227
2120.500000	0.002179
2120.250000	0.002148
2120.000000	0.002137
2119.750000	0.002141
2119.500000	0.002158
2119.250000	0.002183
2119.000000	0.002213
2118.750000	0.002246
2118.500000	0.002283
2118.250000	0.002326
2118.000000	0.002373
2117.750000	0.002424
2117.500000	0.002474
2117.250000	0.002518
2117.000000	0.002552
2116.750000	0.002570
2116.500000	0.002573
2116.250000	0.002561
2116.000000	0.002540
2115.750000	0.002515
2115.500000	0.002495
2115.250000	0.002483
2115.000000	0.002484

2114.750000	0.002496
2114.500000	0.002518
2114.250000	0.002545
2114.000000	0.002572
2113.750000	0.002597
2113.500000	0.002617
2113.250000	0.002632
2113.000000	0.002643
2112.750000	0.002649
2112.500000	0.002651
2112.250000	0.002646
2112.000000	0.002636
2111.750000	0.002619
2111.500000	0.002600
2111.250000	0.002580
2111.000000	0.002566
2110.750000	0.002560
2110.500000	0.002564
2110.250000	0.002577
2110.000000	0.002597
2109.750000	0.002619
2109.500000	0.002639
2109.250000	0.002654
2109.000000	0.002663
2108.750000	0.002663
2108.500000	0.002654

2108.250000	0.002635
2108.000000	0.002605
2107.750000	0.002566
2107.500000	0.002521
2107.250000	0.002475
2107.000000	0.002435
2106.750000	0.002408
2106.500000	0.002399
2106.250000	0.002408
2106.000000	0.002432
2105.750000	0.002465
2105.500000	0.002497
2105.250000	0.002524
2105.000000	0.002541
2104.750000	0.002548
2104.500000	0.002548
2104.250000	0.002545
2104.000000	0.002541
2103.750000	0.002538
2103.500000	0.002535
2103.250000	0.002532
2103.000000	0.002526
2102.750000	0.002518
2102.500000	0.002508
2102.250000	0.002498
2102.000000	0.002489

2101.750000	0.002483
2101.500000	0.002481
2101.250000	0.002480
2101.000000	0.002480
2100.750000	0.002479
2100.500000	0.002478
2100.250000	0.002477
2100.000000	0.002479
2099.750000	0.002487
2099.500000	0.002502
2099.250000	0.002523
2099.000000	0.002547
2098.750000	0.002569
2098.500000	0.002582
2098.250000	0.002583
2098.000000	0.002569
2097.750000	0.002540
2097.500000	0.002501
2097.250000	0.002459
2097.000000	0.002420
2096.750000	0.002389
2096.500000	0.002368
2096.250000	0.002356
2096.000000	0.002349
2095.750000	0.002344
2095.500000	0.002337

2095.250000	0.002328
2095.000000	0.002317
2094.750000	0.002305
2094.500000	0.002295
2094.250000	0.002287
2094.000000	0.002282
2093.750000	0.002281
2093.500000	0.002284
2093.250000	0.002290
2093.000000	0.002298
2092.750000	0.002308
2092.500000	0.002317
2092.250000	0.002322
2092.000000	0.002323
2091.750000	0.002319
2091.500000	0.002311
2091.250000	0.002305
2091.000000	0.002305
2090.750000	0.002316
2090.500000	0.002339
2090.250000	0.002372
2090.000000	0.002410
2089.750000	0.002446
2089.500000	0.002475
2089.250000	0.002493
2089.000000	0.002499

2088.750000	0.002493
2088.500000	0.002476
2088.250000	0.002451
2088.000000	0.002419
2087.750000	0.002382
2087.500000	0.002345
2087.250000	0.002314
2087.000000	0.002296
2086.750000	0.002297
2086.500000	0.002320
2086.250000	0.002363
2086.000000	0.002417
2085.750000	0.002473
2085.500000	0.002519
2085.250000	0.002547
2085.000000	0.002554
2084.750000	0.002544
2084.500000	0.002526
2084.250000	0.002509
2084.000000	0.002501
2083.750000	0.002505
2083.500000	0.002520
2083.250000	0.002541
2083.000000	0.002561
2082.750000	0.002575
2082.500000	0.002579

2082.250000	0.002572
2082.000000	0.002556
2081.750000	0.002533
2081.500000	0.002508
2081.250000	0.002484
2081.000000	0.002464
2080.750000	0.002453
2080.500000	0.002454
2080.250000	0.002468
2080.000000	0.002494
2079.750000	0.002531
2079.500000	0.002571
2079.250000	0.002609
2079.000000	0.002637
2078.750000	0.002650
2078.500000	0.002647
2078.250000	0.002630
2078.000000	0.002603
2077.750000	0.002575
2077.500000	0.002552
2077.250000	0.002537
2077.000000	0.002533
2076.750000	0.002536
2076.500000	0.002542
2076.250000	0.002546
2076.000000	0.002547

2075.750000	0.002543
2075.500000	0.002537
2075.250000	0.002533
2075.000000	0.002535
2074.750000	0.002545
2074.500000	0.002562
2074.250000	0.002582
2074.000000	0.002599
2073.750000	0.002611
2073.500000	0.002617
2073.250000	0.002618
2073.000000	0.002618
2072.750000	0.002621
2072.500000	0.002628
2072.250000	0.002637
2072.000000	0.002644
2071.750000	0.002642
2071.500000	0.002627
2071.250000	0.002599
2071.000000	0.002561
2070.750000	0.002521
2070.500000	0.002485
2070.250000	0.002461
2070.000000	0.002453
2069.750000	0.002459
2069.500000	0.002477

2069.250000	0.002501
2069.000000	0.002528
2068.750000	0.002551
2068.500000	0.002570
2068.250000	0.002582
2068.000000	0.002586
2067.750000	0.002583
2067.500000	0.002573
2067.250000	0.002561
2067.000000	0.002549
2066.750000	0.002541
2066.500000	0.002540
2066.250000	0.002546
2066.000000	0.002559
2065.750000	0.002575
2065.500000	0.002590
2065.250000	0.002603
2065.000000	0.002612
2064.750000	0.002619
2064.500000	0.002625
2064.250000	0.002629
2064.000000	0.002632
2063.750000	0.002630
2063.500000	0.002622
2063.250000	0.002608
2063.000000	0.002590

2062.750000	0.002572
2062.500000	0.002559
2062.250000	0.002552
2062.000000	0.002551
2061.750000	0.002551
2061.500000	0.002550
2061.250000	0.002544
2061.000000	0.002532
2060.750000	0.002519
2060.500000	0.002510
2060.250000	0.002511
2060.000000	0.002526
2059.750000	0.002552
2059.500000	0.002585
2059.250000	0.002616
2059.000000	0.002639
2058.750000	0.002651
2058.500000	0.002650
2058.250000	0.002641
2058.000000	0.002628
2057.750000	0.002616
2057.500000	0.002607
2057.250000	0.002601
2057.000000	0.002598
2056.750000	0.002597
2056.500000	0.002598

2056.250000	0.002602
2056.000000	0.002609
2055.750000	0.002619
2055.500000	0.002632
2055.250000	0.002643
2055.000000	0.002648
2054.750000	0.002643
2054.500000	0.002626
2054.250000	0.002599
2054.000000	0.002566
2053.750000	0.002534
2053.500000	0.002512
2053.250000	0.002509
2053.000000	0.002531
2052.750000	0.002581
2052.500000	0.002657
2052.250000	0.002753
2052.000000	0.002859
2051.750000	0.002962
2051.500000	0.003052
2051.250000	0.003117
2051.000000	0.003151
2050.750000	0.003152
2050.500000	0.003124
2050.250000	0.003072
2050.000000	0.003004

2049.750000	0.002928
2049.500000	0.002850
2049.250000	0.002771
2049.000000	0.002694
2048.750000	0.002618
2048.500000	0.002545
2048.250000	0.002477
2048.000000	0.002418
2047.750000	0.002373
2047.500000	0.002345
2047.250000	0.002337
2047.000000	0.002348
2046.750000	0.002376
2046.500000	0.002417
2046.250000	0.002464
2046.000000	0.002512
2045.750000	0.002555
2045.500000	0.002592
2045.250000	0.002619
2045.000000	0.002636
2044.750000	0.002645
2044.500000	0.002647
2044.250000	0.002641
2044.000000	0.002630
2043.750000	0.002614
2043.500000	0.002594

2043.250000	0.002571
2043.000000	0.002546
2042.750000	0.002523
2042.500000	0.002503
2042.250000	0.002491
2042.000000	0.002489
2041.750000	0.002500
2041.500000	0.002523
2041.250000	0.002556
2041.000000	0.002594
2040.750000	0.002632
2040.500000	0.002665
2040.250000	0.002689
2040.000000	0.002702
2039.750000	0.002708
2039.500000	0.002714
2039.250000	0.002724
2039.000000	0.002745
2038.750000	0.002778
2038.500000	0.002820
2038.250000	0.002865
2038.000000	0.002905
2037.750000	0.002931
2037.500000	0.002942
2037.250000	0.002937
2037.000000	0.002923

2036.750000	0.002906
2036.500000	0.002893
2036.250000	0.002885
2036.000000	0.002879
2035.750000	0.002871
2035.500000	0.002852
2035.250000	0.002817
2035.000000	0.002763
2034.750000	0.002693
2034.500000	0.002612
2034.250000	0.002530
2034.000000	0.002458
2033.750000	0.002408
2033.500000	0.002390
2033.250000	0.002407
2033.000000	0.002462
2032.750000	0.002548
2032.500000	0.002653
2032.250000	0.002759
2032.000000	0.002849
2031.750000	0.002906
2031.500000	0.002922
2031.250000	0.002897
2031.000000	0.002839
2030.750000	0.002764
2030.500000	0.002688

2030.250000	0.002624
2030.000000	0.002579
2029.750000	0.002554
2029.500000	0.002544
2029.250000	0.002543
2029.000000	0.002544
2028.750000	0.002543
2028.500000	0.002536
2028.250000	0.002522
2028.000000	0.002501
2027.750000	0.002473
2027.500000	0.002442
2027.250000	0.002414
2027.000000	0.002397
2026.750000	0.002399
2026.500000	0.002425
2026.250000	0.002475
2026.000000	0.002542
2025.750000	0.002613
2025.500000	0.002673
2025.250000	0.002709
2025.000000	0.002712
2024.750000	0.002681
2024.500000	0.002622
2024.250000	0.002546
2024.000000	0.002468

2023.750000	0.002400
2023.500000	0.002353
2023.250000	0.002332
2023.000000	0.002340
2022.750000	0.002374
2022.500000	0.002427
2022.250000	0.002491
2022.000000	0.002557
2021.750000	0.002613
2021.500000	0.002653
2021.250000	0.002671
2021.000000	0.002665
2020.750000	0.002636
2020.500000	0.002587
2020.250000	0.002520
2020.000000	0.002439
2019.750000	0.002348
2019.500000	0.002257
2019.250000	0.002176
2019.000000	0.002119
2018.750000	0.002098
2018.500000	0.002122
2018.250000	0.002192
2018.000000	0.002299
2017.750000	0.002427
2017.500000	0.002552

2017.250000	0.002651
2017.000000	0.002709
2016.750000	0.002718
2016.500000	0.002680
2016.250000	0.002608
2016.000000	0.002518
2015.750000	0.002423
2015.500000	0.002337
2015.250000	0.002266
2015.000000	0.002217
2014.750000	0.002194
2014.500000	0.002200
2014.250000	0.002237
2014.000000	0.002302
2013.750000	0.002388
2013.500000	0.002485
2013.250000	0.002582
2013.000000	0.002667
2012.750000	0.002734
2012.500000	0.002778
2012.250000	0.002800
2012.000000	0.002804
2011.750000	0.002792
2011.500000	0.002772
2011.250000	0.002747
2011.000000	0.002721

2010.750000	0.002698
2010.500000	0.002683
2010.250000	0.002678
2010.000000	0.002684
2009.750000	0.002701
2009.500000	0.002726
2009.250000	0.002753
2009.000000	0.002774
2008.750000	0.002782
2008.500000	0.002770
2008.250000	0.002734
2008.000000	0.002673
2007.750000	0.002591
2007.500000	0.002496
2007.250000	0.002399
2007.000000	0.002313
2006.750000	0.002247
2006.500000	0.002209
2006.250000	0.002197
2006.000000	0.002209
2005.750000	0.002235
2005.500000	0.002263
2005.250000	0.002284
2005.000000	0.002291
2004.750000	0.002279
2004.500000	0.002249

2004.250000	0.002206
2004.000000	0.002157
2003.750000	0.002107
2003.500000	0.002063
2003.250000	0.002030
2003.000000	0.002010
2002.750000	0.002001
2002.500000	0.002002
2002.250000	0.002007
2002.000000	0.002015
2001.750000	0.002023
2001.500000	0.002034
2001.250000	0.002053
2001.000000	0.002083
2000.750000	0.002130
2000.500000	0.002190
2000.250000	0.002259
2000.000000	0.002325
1999.750000	0.002375
1999.500000	0.002398
1999.250000	0.002390
1999.000000	0.002353
1998.750000	0.002300
1998.500000	0.002247
1998.250000	0.002208
1998.000000	0.002197

1997.750000	0.002217
1997.500000	0.002266
1997.250000	0.002332
1997.000000	0.002404
1996.750000	0.002470
1996.500000	0.002523
1996.250000	0.002561
1996.000000	0.002589
1995.750000	0.002613
1995.500000	0.002640
1995.250000	0.002671
1995.000000	0.002705
1994.750000	0.002734
1994.500000	0.002748
1994.250000	0.002738
1994.000000	0.002698
1993.750000	0.002628
1993.500000	0.002536
1993.250000	0.002433
1993.000000	0.002335
1992.750000	0.002257
1992.500000	0.002210
1992.250000	0.002198
1992.000000	0.002216
1991.750000	0.002254
1991.500000	0.002302

1991.250000	0.002353
1991.000000	0.002402
1990.750000	0.002450
1990.500000	0.002493
1990.250000	0.002530
1990.000000	0.002555
1989.750000	0.002564
1989.500000	0.002553
1989.250000	0.002523
1989.000000	0.002479
1988.750000	0.002430
1988.500000	0.002386
1988.250000	0.002354
1988.000000	0.002336
1987.750000	0.002331
1987.500000	0.002332
1987.250000	0.002336
1987.000000	0.002343
1986.750000	0.002357
1986.500000	0.002382
1986.250000	0.002425
1986.000000	0.002486
1985.750000	0.002559
1985.500000	0.002634
1985.250000	0.002697
1985.000000	0.002738

1984.750000	0.002752
1984.500000	0.002739
1984.250000	0.002710
1984.000000	0.002677
1983.750000	0.002652
1983.500000	0.002644
1983.250000	0.002659
1983.000000	0.002694
1982.750000	0.002745
1982.500000	0.002804
1982.250000	0.002863
1982.000000	0.002915
1981.750000	0.002957
1981.500000	0.002990
1981.250000	0.003017
1981.000000	0.003045
1980.750000	0.003080
1980.500000	0.003127
1980.250000	0.003184
1980.000000	0.003242
1979.750000	0.003288
1979.500000	0.003308
1979.250000	0.003288
1979.000000	0.003223
1978.750000	0.003115
1978.500000	0.002977

1978.250000	0.002826
1978.000000	0.002681
1977.750000	0.002558
1977.500000	0.002464
1977.250000	0.002402
1977.000000	0.002370
1976.750000	0.002364
1976.500000	0.002379
1976.250000	0.002412
1976.000000	0.002457
1975.750000	0.002505
1975.500000	0.002546
1975.250000	0.002570
1975.000000	0.002570
1974.750000	0.002543
1974.500000	0.002493
1974.250000	0.002430
1974.000000	0.002367
1973.750000	0.002317
1973.500000	0.002288
1973.250000	0.002283
1973.000000	0.002297
1972.750000	0.002319
1972.500000	0.002339
1972.250000	0.002348
1972.000000	0.002338

1971.750000	0.002309
1971.500000	0.002266
1971.250000	0.002214
1971.000000	0.002162
1970.750000	0.002118
1970.500000	0.002089
1970.250000	0.002077
1970.000000	0.002083
1969.750000	0.002102
1969.500000	0.002131
1969.250000	0.002162
1969.000000	0.002190
1968.750000	0.002210
1968.500000	0.002218
1968.250000	0.002216
1968.000000	0.002204
1967.750000	0.002187
1967.500000	0.002171
1967.250000	0.002164
1967.000000	0.002169
1966.750000	0.002189
1966.500000	0.002223
1966.250000	0.002267
1966.000000	0.002315
1965.750000	0.002358
1965.500000	0.002392

1965.250000	0.002413
1965.000000	0.002420
1964.750000	0.002416
1964.500000	0.002404
1964.250000	0.002388
1964.000000	0.002372
1963.750000	0.002359
1963.500000	0.002350
1963.250000	0.002345
1963.000000	0.002343
1962.750000	0.002342
1962.500000	0.002342
1962.250000	0.002338
1962.000000	0.002330
1961.750000	0.002316
1961.500000	0.002295
1961.250000	0.002270
1961.000000	0.002240
1960.750000	0.002210
1960.500000	0.002182
1960.250000	0.002158
1960.000000	0.002138
1959.750000	0.002122
1959.500000	0.002108
1959.250000	0.002095
1959.000000	0.002082

1958.750000	0.002069
1958.500000	0.002058
1958.250000	0.002052
1958.000000	0.002054
1957.750000	0.002067
1957.500000	0.002092
1957.250000	0.002125
1957.000000	0.002163
1956.750000	0.002201
1956.500000	0.002233
1956.250000	0.002256
1956.000000	0.002270
1955.750000	0.002276
1955.500000	0.002279
1955.250000	0.002280
1955.000000	0.002283
1954.750000	0.002284
1954.500000	0.002280
1954.250000	0.002268
1954.000000	0.002245
1953.750000	0.002214
1953.500000	0.002180
1953.250000	0.002154
1953.000000	0.002144
1952.750000	0.002155
1952.500000	0.002185

1952.250000	0.002228
1952.000000	0.002272
1951.750000	0.002303
1951.500000	0.002315
1951.250000	0.002305
1951.000000	0.002279
1950.750000	0.002246
1950.500000	0.002218
1950.250000	0.002201
1950.000000	0.002197
1949.750000	0.002202
1949.500000	0.002212
1949.250000	0.002219
1949.000000	0.002221
1948.750000	0.002215
1948.500000	0.002205
1948.250000	0.002191
1948.000000	0.002176
1947.750000	0.002159
1947.500000	0.002139
1947.250000	0.002116
1947.000000	0.002092
1946.750000	0.002070
1946.500000	0.002054
1946.250000	0.002049
1946.000000	0.002059

1945.750000	0.002082
1945.500000	0.002115
1945.250000	0.002155
1945.000000	0.002195
1944.750000	0.002228
1944.500000	0.002248
1944.250000	0.002251
1944.000000	0.002231
1943.750000	0.002188
1943.500000	0.002125
1943.250000	0.002051
1943.000000	0.001977
1942.750000	0.001918
1942.500000	0.001882
1942.250000	0.001874
1942.000000	0.001890
1941.750000	0.001922
1941.500000	0.001959
1941.250000	0.001995
1941.000000	0.002024
1940.750000	0.002049
1940.500000	0.002073
1940.250000	0.002103
1940.000000	0.002142
1939.750000	0.002187
1939.500000	0.002235

1939.250000	0.002279
1939.000000	0.002314
1938.750000	0.002337
1938.500000	0.002347
1938.250000	0.002345
1938.000000	0.002332
1937.750000	0.002311
1937.500000	0.002281
1937.250000	0.002246
1937.000000	0.002209
1936.750000	0.002174
1936.500000	0.002147
1936.250000	0.002131
1936.000000	0.002126
1935.750000	0.002128
1935.500000	0.002131
1935.250000	0.002129
1935.000000	0.002119
1934.750000	0.002100
1934.500000	0.002075
1934.250000	0.002052
1934.000000	0.002034
1933.750000	0.002028
1933.500000	0.002032
1933.250000	0.002046
1933.000000	0.002067

1932.750000	0.002092
1932.500000	0.002117
1932.250000	0.002143
1932.000000	0.002167
1931.750000	0.002188
1931.500000	0.002205
1931.250000	0.002217
1931.000000	0.002221
1930.750000	0.002220
1930.500000	0.002216
1930.250000	0.002212
1930.000000	0.002213
1929.750000	0.002220
1929.500000	0.002233
1929.250000	0.002249
1929.000000	0.002263
1928.750000	0.002269
1928.500000	0.002262
1928.250000	0.002240
1928.000000	0.002206
1927.750000	0.002164
1927.500000	0.002122
1927.250000	0.002089
1927.000000	0.002070
1926.750000	0.002072
1926.500000	0.002095

1926.250000	0.002136
1926.000000	0.002191
1925.750000	0.002252
1925.500000	0.002311
1925.250000	0.002361
1925.000000	0.002396
1924.750000	0.002411
1924.500000	0.002403
1924.250000	0.002373
1924.000000	0.002323
1923.750000	0.002260
1923.500000	0.002192
1923.250000	0.002131
1923.000000	0.002088
1922.750000	0.002068
1922.500000	0.002075
1922.250000	0.002103
1922.000000	0.002145
1921.750000	0.002191
1921.500000	0.002231
1921.250000	0.002260
1921.000000	0.002276
1920.750000	0.002281
1920.500000	0.002277
1920.250000	0.002268
1920.000000	0.002253

1919.750000	0.002231
1919.500000	0.002201
1919.250000	0.002163
1919.000000	0.002121
1918.750000	0.002083
1918.500000	0.002058
1918.250000	0.002054
1918.000000	0.002073
1917.750000	0.002114
1917.500000	0.002169
1917.250000	0.002229
1917.000000	0.002284
1916.750000	0.002326
1916.500000	0.002350
1916.250000	0.002355
1916.000000	0.002345
1915.750000	0.002326
1915.500000	0.002303
1915.250000	0.002278
1915.000000	0.002254
1914.750000	0.002230
1914.500000	0.002208
1914.250000	0.002188
1914.000000	0.002172
1913.750000	0.002162
1913.500000	0.002157

1913.250000	0.002159
1913.000000	0.002164
1912.750000	0.002171
1912.500000	0.002179
1912.250000	0.002184
1912.000000	0.002188
1911.750000	0.002188
1911.500000	0.002184
1911.250000	0.002175
1911.000000	0.002162
1910.750000	0.002147
1910.500000	0.002134
1910.250000	0.002128
1910.000000	0.002132
1909.750000	0.002146
1909.500000	0.002169
1909.250000	0.002195
1909.000000	0.002218
1908.750000	0.002234
1908.500000	0.002239
1908.250000	0.002231
1908.000000	0.002215
1907.750000	0.002196
1907.500000	0.002179
1907.250000	0.002171
1907.000000	0.002174

1906.750000	0.002189
1906.500000	0.002212
1906.250000	0.002239
1906.000000	0.002264
1905.750000	0.002282
1905.500000	0.002289
1905.250000	0.002284
1905.000000	0.002270
1904.750000	0.002248
1904.500000	0.002225
1904.250000	0.002205
1904.000000	0.002192
1903.750000	0.002188
1903.500000	0.002194
1903.250000	0.002207
1903.000000	0.002227
1902.750000	0.002250
1902.500000	0.002273
1902.250000	0.002295
1902.000000	0.002313
1901.750000	0.002326
1901.500000	0.002333
1901.250000	0.002333
1901.000000	0.002329
1900.750000	0.002322
1900.500000	0.002317

1900.250000	0.002315
1900.000000	0.002317
1899.750000	0.002321
1899.500000	0.002327
1899.250000	0.002331
1899.000000	0.002331
1898.750000	0.002328
1898.500000	0.002322
1898.250000	0.002317
1898.000000	0.002314
1897.750000	0.002316
1897.500000	0.002324
1897.250000	0.002335
1897.000000	0.002348
1896.750000	0.002359
1896.500000	0.002366
1896.250000	0.002368
1896.000000	0.002364
1895.750000	0.002357
1895.500000	0.002348
1895.250000	0.002342
1895.000000	0.002340
1894.750000	0.002344
1894.500000	0.002354
1894.250000	0.002367
1894.000000	0.002381

1893.750000	0.002392
1893.500000	0.002396
1893.250000	0.002393
1893.000000	0.002386
1892.750000	0.002376
1892.500000	0.002370
1892.250000	0.002372
1892.000000	0.002383
1891.750000	0.002401
1891.500000	0.002421
1891.250000	0.002435
1891.000000	0.002433
1890.750000	0.002409
1890.500000	0.002360
1890.250000	0.002289
1890.000000	0.002207
1889.750000	0.002132
1889.500000	0.002078
1889.250000	0.002059
1889.000000	0.002076
1888.750000	0.002122
1888.500000	0.002180
1888.250000	0.002234
1888.000000	0.002272
1887.750000	0.002289
1887.500000	0.002287

1887.250000	0.002274
1887.000000	0.002259
1886.750000	0.002250
1886.500000	0.002251
1886.250000	0.002263
1886.000000	0.002281
1885.750000	0.002300
1885.500000	0.002318
1885.250000	0.002332
1885.000000	0.002341
1884.750000	0.002350
1884.500000	0.002357
1884.250000	0.002364
1884.000000	0.002370
1883.750000	0.002372
1883.500000	0.002370
1883.250000	0.002363
1883.000000	0.002353
1882.750000	0.002342
1882.500000	0.002334
1882.250000	0.002332
1882.000000	0.002337
1881.750000	0.002348
1881.500000	0.002362
1881.250000	0.002375
1881.000000	0.002382

1880.750000	0.002381
1880.500000	0.002370
1880.250000	0.002350
1880.000000	0.002325
1879.750000	0.002298
1879.500000	0.002271
1879.250000	0.002248
1879.000000	0.002230
1878.750000	0.002218
1878.500000	0.002212
1878.250000	0.002211
1878.000000	0.002215
1877.750000	0.002222
1877.500000	0.002233
1877.250000	0.002247
1877.000000	0.002263
1876.750000	0.002281
1876.500000	0.002299
1876.250000	0.002314
1876.000000	0.002324
1875.750000	0.002327
1875.500000	0.002323
1875.250000	0.002314
1875.000000	0.002303
1874.750000	0.002294
1874.500000	0.002290

1874.250000	0.002294
1874.000000	0.002305
1873.750000	0.002324
1873.500000	0.002347
1873.250000	0.002372
1873.000000	0.002397
1872.750000	0.002419
1872.500000	0.002438
1872.250000	0.002454
1872.000000	0.002465
1871.750000	0.002471
1871.500000	0.002469
1871.250000	0.002454
1871.000000	0.002423
1870.750000	0.002376
1870.500000	0.002314
1870.250000	0.002245
1870.000000	0.002179
1869.750000	0.002131
1869.500000	0.002111
1869.250000	0.002124
1869.000000	0.002166
1868.750000	0.002227
1868.500000	0.002292
1868.250000	0.002349
1868.000000	0.002391

1867.750000	0.002415
1867.500000	0.002424
1867.250000	0.002425
1867.000000	0.002422
1866.750000	0.002419
1866.500000	0.002418
1866.250000	0.002417
1866.000000	0.002413
1865.750000	0.002406
1865.500000	0.002395
1865.250000	0.002381
1865.000000	0.002366
1864.750000	0.002352
1864.500000	0.002340
1864.250000	0.002331
1864.000000	0.002321
1863.750000	0.002312
1863.500000	0.002300
1863.250000	0.002288
1863.000000	0.002275
1862.750000	0.002263
1862.500000	0.002252
1862.250000	0.002243
1862.000000	0.002237
1861.750000	0.002233
1861.500000	0.002229

1861.250000	0.002226
1861.000000	0.002221
1860.750000	0.002214
1860.500000	0.002206
1860.250000	0.002197
1860.000000	0.002190
1859.750000	0.002186
1859.500000	0.002185
1859.250000	0.002189
1859.000000	0.002197
1858.750000	0.002209
1858.500000	0.002224
1858.250000	0.002239
1858.000000	0.002251
1857.750000	0.002259
1857.500000	0.002260
1857.250000	0.002256
1857.000000	0.002247
1856.750000	0.002237
1856.500000	0.002227
1856.250000	0.002221
1856.000000	0.002220
1855.750000	0.002224
1855.500000	0.002232
1855.250000	0.002241
1855.000000	0.002249

1854.750000	0.002256
1854.500000	0.002260
1854.250000	0.002261
1854.000000	0.002259
1853.750000	0.002256
1853.500000	0.002252
1853.250000	0.002248
1853.000000	0.002246
1852.750000	0.002246
1852.500000	0.002249
1852.250000	0.002254
1852.000000	0.002261
1851.750000	0.002267
1851.500000	0.002269
1851.250000	0.002267
1851.000000	0.002260
1850.750000	0.002248
1850.500000	0.002236
1850.250000	0.002225
1850.000000	0.002220
1849.750000	0.002221
1849.500000	0.002229
1849.250000	0.002241
1849.000000	0.002254
1848.750000	0.002264
1848.500000	0.002269

1848.250000	0.002267
1848.000000	0.002259
1847.750000	0.002247
1847.500000	0.002237
1847.250000	0.002230
1847.000000	0.002228
1846.750000	0.002230
1846.500000	0.002229
1846.250000	0.002218
1846.000000	0.002188
1845.750000	0.002136
1845.500000	0.002062
1845.250000	0.001976
1845.000000	0.001892
1844.750000	0.001830
1844.500000	0.001806
1844.250000	0.001830
1844.000000	0.001901
1843.750000	0.002009
1843.500000	0.002138
1843.250000	0.002261
1843.000000	0.002358
1842.750000	0.002411
1842.500000	0.002422
1842.250000	0.002402
1842.000000	0.002366

1841.750000	0.002328
1841.500000	0.002298
1841.250000	0.002279
1841.000000	0.002270
1840.750000	0.002266
1840.500000	0.002264
1840.250000	0.002262
1840.000000	0.002258
1839.750000	0.002254
1839.500000	0.002252
1839.250000	0.002251
1839.000000	0.002251
1838.750000	0.002250
1838.500000	0.002245
1838.250000	0.002235
1838.000000	0.002217
1837.750000	0.002193
1837.500000	0.002166
1837.250000	0.002139
1837.000000	0.002118
1836.750000	0.002107
1836.500000	0.002110
1836.250000	0.002123
1836.000000	0.002145
1835.750000	0.002169
1835.500000	0.002190

1835.250000	0.002205
1835.000000	0.002212
1834.750000	0.002211
1834.500000	0.002205
1834.250000	0.002195
1834.000000	0.002184
1833.750000	0.002173
1833.500000	0.002162
1833.250000	0.002155
1833.000000	0.002151
1832.750000	0.002153
1832.500000	0.002160
1832.250000	0.002167
1832.000000	0.002170
1831.750000	0.002160
1831.500000	0.002134
1831.250000	0.002088
1831.000000	0.002027
1830.750000	0.001961
1830.500000	0.001904
1830.250000	0.001869
1830.000000	0.001862
1829.750000	0.001884
1829.500000	0.001928
1829.250000	0.001986
1829.000000	0.002049

1828.750000	0.002109
1828.500000	0.002159
1828.250000	0.002194
1828.000000	0.002213
1827.750000	0.002217
1827.500000	0.002211
1827.250000	0.002194
1827.000000	0.002167
1826.750000	0.002130
1826.500000	0.002085
1826.250000	0.002034
1826.000000	0.001984
1825.750000	0.001943
1825.500000	0.001916
1825.250000	0.001906
1825.000000	0.001917
1824.750000	0.001949
1824.500000	0.001999
1824.250000	0.002061
1824.000000	0.002123
1823.750000	0.002172
1823.500000	0.002202
1823.250000	0.002211
1823.000000	0.002206
1822.750000	0.002192
1822.500000	0.002176

1822.250000	0.002163
1822.000000	0.002153
1821.750000	0.002146
1821.500000	0.002140
1821.250000	0.002133
1821.000000	0.002125
1820.750000	0.002117
1820.500000	0.002110
1820.250000	0.002105
1820.000000	0.002102
1819.750000	0.002100
1819.500000	0.002097
1819.250000	0.002092
1819.000000	0.002084
1818.750000	0.002074
1818.500000	0.002062
1818.250000	0.002049
1818.000000	0.002034
1817.750000	0.002021
1817.500000	0.002011
1817.250000	0.002004
1817.000000	0.002004
1816.750000	0.002010
1816.500000	0.002022
1816.250000	0.002040
1816.000000	0.002061

1815.750000	0.002079
1815.500000	0.002093
1815.250000	0.002100
1815.000000	0.002100
1814.750000	0.002094
1814.500000	0.002087
1814.250000	0.002079
1814.000000	0.002073
1813.750000	0.002069
1813.500000	0.002068
1813.250000	0.002069
1813.000000	0.002070
1812.750000	0.002072
1812.500000	0.002075
1812.250000	0.002076
1812.000000	0.002076
1811.750000	0.002074
1811.500000	0.002070
1811.250000	0.002066
1811.000000	0.002064
1810.750000	0.002065
1810.500000	0.002073
1810.250000	0.002089
1810.000000	0.002115
1809.750000	0.002147
1809.500000	0.002179

1809.250000	0.002206
1809.000000	0.002222
1808.750000	0.002227
1808.500000	0.002221
1808.250000	0.002208
1808.000000	0.002194
1807.750000	0.002183
1807.500000	0.002176
1807.250000	0.002176
1807.000000	0.002181
1806.750000	0.002189
1806.500000	0.002200
1806.250000	0.002211
1806.000000	0.002221
1805.750000	0.002228
1805.500000	0.002233
1805.250000	0.002235
1805.000000	0.002236
1804.750000	0.002237
1804.500000	0.002237
1804.250000	0.002237
1804.000000	0.002235
1803.750000	0.002229
1803.500000	0.002218
1803.250000	0.002201
1803.000000	0.002179

1802.750000	0.002154
1802.500000	0.002131
1802.250000	0.002113
1802.000000	0.002106
1801.750000	0.002109
1801.500000	0.002122
1801.250000	0.002141
1801.000000	0.002159
1800.750000	0.002173
1800.500000	0.002180
1800.250000	0.002181
1800.000000	0.002180
1799.750000	0.002182
1799.500000	0.002191
1799.250000	0.002209
1799.000000	0.002237
1798.750000	0.002273
1798.500000	0.002311
1798.250000	0.002344
1798.000000	0.002366
1797.750000	0.002375
1797.500000	0.002370
1797.250000	0.002357
1797.000000	0.002342
1796.750000	0.002329
1796.500000	0.002325

1796.250000	0.002330
1796.000000	0.002343
1795.750000	0.002360
1795.500000	0.002379
1795.250000	0.002397
1795.000000	0.002412
1794.750000	0.002421
1794.500000	0.002418
1794.250000	0.002397
1794.000000	0.002357
1793.750000	0.002302
1793.500000	0.002240
1793.250000	0.002183
1793.000000	0.002143
1792.750000	0.002129
1792.500000	0.002144
1792.250000	0.002185
1792.000000	0.002244
1791.750000	0.002307
1791.500000	0.002359
1791.250000	0.002393
1791.000000	0.002411
1790.750000	0.002419
1790.500000	0.002428
1790.250000	0.002447
1790.000000	0.002477

1789.750000	0.002513
1789.500000	0.002550
1789.250000	0.002580
1789.000000	0.002600
1788.750000	0.002611
1788.500000	0.002616
1788.250000	0.002621
1788.000000	0.002627
1787.750000	0.002637
1787.500000	0.002648
1787.250000	0.002657
1787.000000	0.002662
1786.750000	0.002660
1786.500000	0.002650
1786.250000	0.002634
1786.000000	0.002611
1785.750000	0.002586
1785.500000	0.002563
1785.250000	0.002547
1785.000000	0.002543
1784.750000	0.002555
1784.500000	0.002585
1784.250000	0.002628
1784.000000	0.002679
1783.750000	0.002728
1783.500000	0.002769

1783.250000	0.002795
1783.000000	0.002807
1782.750000	0.002806
1782.500000	0.002795
1782.250000	0.002777
1782.000000	0.002756
1781.750000	0.002735
1781.500000	0.002717
1781.250000	0.002706
1781.000000	0.002707
1780.750000	0.002723
1780.500000	0.002753
1780.250000	0.002796
1780.000000	0.002849
1779.750000	0.002904
1779.500000	0.002956
1779.250000	0.002998
1779.000000	0.003027
1778.750000	0.003044
1778.500000	0.003051
1778.250000	0.003054
1778.000000	0.003056
1777.750000	0.003059
1777.500000	0.003064
1777.250000	0.003067
1777.000000	0.003069

1776.750000	0.003068
1776.500000	0.003068
1776.250000	0.003069
1776.000000	0.003077
1775.750000	0.003092
1775.500000	0.003117
1775.250000	0.003151
1775.000000	0.003191
1774.750000	0.003229
1774.500000	0.003258
1774.250000	0.003271
1774.000000	0.003264
1773.750000	0.003240
1773.500000	0.003211
1773.250000	0.003191
1773.000000	0.003196
1772.750000	0.003239
1772.500000	0.003321
1772.250000	0.003431
1772.000000	0.003546
1771.750000	0.003649
1771.500000	0.003727
1771.250000	0.003782
1771.000000	0.003822
1770.750000	0.003857
1770.500000	0.003891

1770.250000	0.003925
1770.000000	0.003952
1769.750000	0.003963
1769.500000	0.003955
1769.250000	0.003930
1769.000000	0.003898
1768.750000	0.003874
1768.500000	0.003874
1768.250000	0.003907
1768.000000	0.003973
1767.750000	0.004069
1767.500000	0.004184
1767.250000	0.004306
1767.000000	0.004422
1766.750000	0.004519
1766.500000	0.004595
1766.250000	0.004651
1766.000000	0.004694
1765.750000	0.004732
1765.500000	0.004769
1765.250000	0.004809
1765.000000	0.004853
1764.750000	0.004900
1764.500000	0.004947
1764.250000	0.004993
1764.000000	0.005032

1763.750000	0.005064
1763.500000	0.005086
1763.250000	0.005098
1763.000000	0.005106
1762.750000	0.005119
1762.500000	0.005156
1762.250000	0.005230
1762.000000	0.005344
1761.750000	0.005488
1761.500000	0.005648
1761.250000	0.005808
1761.000000	0.005962
1760.750000	0.006105
1760.500000	0.006232
1760.250000	0.006338
1760.000000	0.006422
1759.750000	0.006489
1759.500000	0.006546
1759.250000	0.006598
1759.000000	0.006647
1758.750000	0.006694
1758.500000	0.006740
1758.250000	0.006787
1758.000000	0.006839
1757.750000	0.006903
1757.500000	0.006987

1757.250000	0.007098
1757.000000	0.007238
1756.750000	0.007401
1756.500000	0.007579
1756.250000	0.007766
1756.000000	0.007955
1755.750000	0.008138
1755.500000	0.008312
1755.250000	0.008473
1755.000000	0.008621
1754.750000	0.008760
1754.500000	0.008896
1754.250000	0.009031
1754.000000	0.009166
1753.750000	0.009297
1753.500000	0.009417
1753.250000	0.009522
1753.000000	0.009611
1752.750000	0.009689
1752.500000	0.009769
1752.250000	0.009874
1752.000000	0.010030
1751.750000	0.010257
1751.500000	0.010550
1751.250000	0.010880
1751.000000	0.011207

1750.750000	0.011500
1750.500000	0.011752
1750.250000	0.011969
1750.000000	0.012160
1749.750000	0.012336
1749.500000	0.012511
1749.250000	0.012703
1749.000000	0.012922
1748.750000	0.013169
1748.500000	0.013440
1748.250000	0.013728
1748.000000	0.014026
1747.750000	0.014334
1747.500000	0.014651
1747.250000	0.014976
1747.000000	0.015305
1746.750000	0.015634
1746.500000	0.015959
1746.250000	0.016276
1746.000000	0.016585
1745.750000	0.016887
1745.500000	0.017186
1745.250000	0.017487
1745.000000	0.017793
1744.750000	0.018113
1744.500000	0.018456

1744.250000	0.018834
1744.000000	0.019257
1743.750000	0.019729
1743.500000	0.020241
1743.250000	0.020769
1743.000000	0.021285
1742.750000	0.021765
1742.500000	0.022198
1742.250000	0.022584
1742.000000	0.022932
1741.750000	0.023252
1741.500000	0.023562
1741.250000	0.023884
1741.000000	0.024253
1740.750000	0.024715
1740.500000	0.025318
1740.250000	0.026079
1740.000000	0.026960
1739.750000	0.027868
1739.500000	0.028705
1739.250000	0.029415
1739.000000	0.030009
1738.750000	0.030533
1738.500000	0.031026
1738.250000	0.031514
1738.000000	0.032007

1737.750000	0.032507
1737.500000	0.033007
1737.250000	0.033490
1737.000000	0.033939
1736.750000	0.034341
1736.500000	0.034693
1736.250000	0.035001
1736.000000	0.035282
1735.750000	0.035570
1735.500000	0.035911
1735.250000	0.036353
1735.000000	0.036969
1734.750000	0.037872
1734.500000	0.039139
1734.250000	0.040659
1734.000000	0.042133
1733.750000	0.043279
1733.500000	0.044035
1733.250000	0.044534
1733.000000	0.044925
1732.750000	0.045272
1732.500000	0.045586
1732.250000	0.045877
1732.000000	0.046172
1731.750000	0.046501
1731.500000	0.046898

1731.250000	0.047392
1731.000000	0.048009
1730.750000	0.048758
1730.500000	0.049615
1730.250000	0.050515
1730.000000	0.051368
1729.750000	0.052108
1729.500000	0.052716
1729.250000	0.053227
1729.000000	0.053696
1728.750000	0.054172
1728.500000	0.054681
1728.250000	0.055234
1728.000000	0.055824
1727.750000	0.056440
1727.500000	0.057066
1727.250000	0.057688
1727.000000	0.058291
1726.750000	0.058870
1726.500000	0.059421
1726.250000	0.059948
1726.000000	0.060453
1725.750000	0.060942
1725.500000	0.061420
1725.250000	0.061896
1725.000000	0.062378

1724.750000	0.062877
1724.500000	0.063401
1724.250000	0.063951
1724.000000	0.064516
1723.750000	0.065070
1723.500000	0.065585
1723.250000	0.066037
1723.000000	0.066421
1722.750000	0.066753
1722.500000	0.067057
1722.250000	0.067360
1722.000000	0.067675
1721.750000	0.067998
1721.500000	0.068311
1721.250000	0.068592
1721.000000	0.068826
1720.750000	0.069008
1720.500000	0.069148
1720.250000	0.069267
1720.000000	0.069392
1719.750000	0.069560
1719.500000	0.069805
1719.250000	0.070160
1719.000000	0.070654
1718.750000	0.071317
1718.500000	0.072123

1718.250000	0.072915
1718.000000	0.073433
1717.750000	0.073493
1717.500000	0.073134
1717.250000	0.072572
1717.000000	0.072034
1716.750000	0.071635
1716.500000	0.071391
1716.250000	0.071289
1716.000000	0.071333
1715.750000	0.071535
1715.500000	0.071876
1715.250000	0.072267
1715.000000	0.072572
1714.750000	0.072675
1714.500000	0.072538
1714.250000	0.072226
1714.000000	0.071858
1713.750000	0.071545
1713.500000	0.071336
1713.250000	0.071220
1713.000000	0.071161
1712.750000	0.071119
1712.500000	0.071069
1712.250000	0.071003
1712.000000	0.070930

1711.750000	0.070872
1711.500000	0.070846
1711.250000	0.070856
1711.000000	0.070881
1710.750000	0.070884
1710.500000	0.070823
1710.250000	0.070674
1710.000000	0.070440
1709.750000	0.070148
1709.500000	0.069839
1709.250000	0.069543
1709.000000	0.069277
1708.750000	0.069042
1708.500000	0.068839
1708.250000	0.068672
1708.000000	0.068556
1707.750000	0.068510
1707.500000	0.068547
1707.250000	0.068654
1707.000000	0.068780
1706.750000	0.068836
1706.500000	0.068732
1706.250000	0.068435
1706.000000	0.067989
1705.750000	0.067502
1705.500000	0.067083

1705.250000	0.066798
1705.000000	0.066641
1704.750000	0.066552
1704.500000	0.066444
1704.250000	0.066239
1704.000000	0.065912
1703.750000	0.065498
1703.500000	0.065064
1703.250000	0.064674
1703.000000	0.064361
1702.750000	0.064098
1702.500000	0.063848
1702.250000	0.063639
1702.000000	0.063513
1701.750000	0.063415
1701.500000	0.063318
1701.250000	0.063466
1701.000000	0.064190
1700.750000	0.065296
1700.500000	0.065895
1700.250000	0.065185
1700.000000	0.063300
1699.750000	0.061173
1699.500000	0.059653
1699.250000	0.058928
1699.000000	0.058671

1698.750000	0.058515
1698.500000	0.058324
1698.250000	0.058128
1698.000000	0.057944
1697.750000	0.057742
1697.500000	0.057523
1697.250000	0.057332
1697.000000	0.057222
1696.750000	0.057242
1696.500000	0.057373
1696.250000	0.057392
1696.000000	0.056932
1695.750000	0.055817
1695.500000	0.054280
1695.250000	0.052771
1695.000000	0.051592
1694.750000	0.050761
1694.500000	0.050151
1694.250000	0.049646
1694.000000	0.049191
1693.750000	0.048763
1693.500000	0.048340
1693.250000	0.047893
1693.000000	0.047407
1692.750000	0.046877
1692.500000	0.046311

1692.250000	0.045719
1692.000000	0.045113
1691.750000	0.044510
1691.500000	0.043918
1691.250000	0.043331
1691.000000	0.042739
1690.750000	0.042128
1690.500000	0.041483
1690.250000	0.040801
1690.000000	0.040087
1689.750000	0.039360
1689.500000	0.038641
1689.250000	0.037947
1689.000000	0.037282
1688.750000	0.036641
1688.500000	0.036011
1688.250000	0.035379
1688.000000	0.034741
1687.750000	0.034108
1687.500000	0.033500
1687.250000	0.032938
1687.000000	0.032432
1686.750000	0.031978
1686.500000	0.031574
1686.250000	0.031225
1686.000000	0.030944

1685.750000	0.030718
1685.500000	0.030537
1685.250000	0.030430
1685.000000	0.030409
1684.750000	0.030335
1684.500000	0.029945
1684.250000	0.029080
1684.000000	0.027878
1683.750000	0.026680
1683.500000	0.025757
1683.250000	0.025169
1683.000000	0.024821
1682.750000	0.024595
1682.500000	0.024429
1682.250000	0.024300
1682.000000	0.024194
1681.750000	0.024089
1681.500000	0.023973
1681.250000	0.023850
1681.000000	0.023718
1680.750000	0.023564
1680.500000	0.023368
1680.250000	0.023118
1680.000000	0.022817
1679.750000	0.022486
1679.500000	0.022160

1679.250000	0.021866
1679.000000	0.021620
1678.750000	0.021416
1678.500000	0.021241
1678.250000	0.021079
1678.000000	0.020913
1677.750000	0.020735
1677.500000	0.020540
1677.250000	0.020333
1677.000000	0.020121
1676.750000	0.019914
1676.500000	0.019721
1676.250000	0.019561
1676.000000	0.019436
1675.750000	0.019307
1675.500000	0.019102
1675.250000	0.018781
1675.000000	0.018382
1674.750000	0.017998
1674.500000	0.017706
1674.250000	0.017517
1674.000000	0.017391
1673.750000	0.017280
1673.500000	0.017161
1673.250000	0.017034
1673.000000	0.016903

1672.750000	0.016770
1672.500000	0.016633
1672.250000	0.016489
1672.000000	0.016328
1671.750000	0.016139
1671.500000	0.015916
1671.250000	0.015670
1671.000000	0.015420
1670.750000	0.015183
1670.500000	0.014973
1670.250000	0.014797
1670.000000	0.014659
1669.750000	0.014540
1669.500000	0.014405
1669.250000	0.014216
1669.000000	0.013961
1668.750000	0.013669
1668.500000	0.013395
1668.250000	0.013177
1668.000000	0.013020
1667.750000	0.012902
1667.500000	0.012800
1667.250000	0.012703
1667.000000	0.012606
1666.750000	0.012506
1666.500000	0.012401

1666.250000	0.012291
1666.000000	0.012176
1665.750000	0.012056
1665.500000	0.011932
1665.250000	0.011803
1665.000000	0.011670
1664.750000	0.011533
1664.500000	0.011395
1664.250000	0.011258
1664.000000	0.011127
1663.750000	0.011007
1663.500000	0.010898
1663.250000	0.010790
1663.000000	0.010663
1662.750000	0.010508
1662.500000	0.010333
1662.250000	0.010165
1662.000000	0.010030
1661.750000	0.009937
1661.500000	0.009877
1661.250000	0.009832
1661.000000	0.009785
1660.750000	0.009730
1660.500000	0.009666
1660.250000	0.009595
1660.000000	0.009519

1659.750000	0.009445
1659.500000	0.009374
1659.250000	0.009308
1659.000000	0.009244
1658.750000	0.009181
1658.500000	0.009118
1658.250000	0.009055
1658.000000	0.008995
1657.750000	0.008938
1657.500000	0.008886
1657.250000	0.008838
1657.000000	0.008793
1656.750000	0.008747
1656.500000	0.008697
1656.250000	0.008639
1656.000000	0.008575
1655.750000	0.008503
1655.500000	0.008420
1655.250000	0.008331
1655.000000	0.008241
1654.750000	0.008154
1654.500000	0.008072
1654.250000	0.008005
1654.000000	0.007979
1653.750000	0.008021
1653.500000	0.008103

1653.250000	0.008117
1653.000000	0.007965
1652.750000	0.007705
1652.500000	0.007543
1652.250000	0.007626
1652.000000	0.007889
1651.750000	0.008147
1651.500000	0.008294
1651.250000	0.008341
1651.000000	0.008337
1650.750000	0.008304
1650.500000	0.008256
1650.250000	0.008211
1650.000000	0.008180
1649.750000	0.008160
1649.500000	0.008148
1649.250000	0.008143
1649.000000	0.008146
1648.750000	0.008164
1648.500000	0.008195
1648.250000	0.008233
1648.000000	0.008267
1647.750000	0.008292
1647.500000	0.008310
1647.250000	0.008334
1647.000000	0.008381

1646.750000	0.008460
1646.500000	0.008564
1646.250000	0.008675
1646.000000	0.008775
1645.750000	0.008864
1645.500000	0.008950
1645.250000	0.009040
1645.000000	0.009130
1644.750000	0.009209
1644.500000	0.009268
1644.250000	0.009312
1644.000000	0.009350
1643.750000	0.009390
1643.500000	0.009440
1643.250000	0.009502
1643.000000	0.009578
1642.750000	0.009664
1642.500000	0.009757
1642.250000	0.009855
1642.000000	0.009954
1641.750000	0.010054
1641.500000	0.010157
1641.250000	0.010262
1641.000000	0.010368
1640.750000	0.010474
1640.500000	0.010575

1640.250000	0.010667
1640.000000	0.010746
1639.750000	0.010814
1639.500000	0.010872
1639.250000	0.010923
1639.000000	0.010970
1638.750000	0.011016
1638.500000	0.011062
1638.250000	0.011104
1638.000000	0.011131
1637.750000	0.011136
1637.500000	0.011113
1637.250000	0.011069
1637.000000	0.011013
1636.750000	0.010960
1636.500000	0.010924
1636.250000	0.010925
1636.000000	0.010956
1635.750000	0.010955
1635.500000	0.010836
1635.250000	0.010584
1635.000000	0.010293
1634.750000	0.010084
1634.500000	0.010000
1634.250000	0.009999
1634.000000	0.010018

1633.750000	0.010024
1633.500000	0.010011
1633.250000	0.009983
1633.000000	0.009944
1632.750000	0.009895
1632.500000	0.009840
1632.250000	0.009779
1632.000000	0.009713
1631.750000	0.009645
1631.500000	0.009574
1631.250000	0.009503
1631.000000	0.009431
1630.750000	0.009356
1630.500000	0.009279
1630.250000	0.009199
1630.000000	0.009118
1629.750000	0.009038
1629.500000	0.008959
1629.250000	0.008882
1629.000000	0.008807
1628.750000	0.008732
1628.500000	0.008653
1628.250000	0.008562
1628.000000	0.008452
1627.750000	0.008325
1627.500000	0.008192

1627.250000	0.008070
1627.000000	0.007973
1626.750000	0.007905
1626.500000	0.007859
1626.250000	0.007824
1626.000000	0.007789
1625.750000	0.007747
1625.500000	0.007694
1625.250000	0.007630
1625.000000	0.007557
1624.750000	0.007479
1624.500000	0.007403
1624.250000	0.007338
1624.000000	0.007282
1623.750000	0.007226
1623.500000	0.007151
1623.250000	0.007053
1623.000000	0.006946
1622.750000	0.006855
1622.500000	0.006795
1622.250000	0.006766
1622.000000	0.006755
1621.750000	0.006752
1621.500000	0.006748
1621.250000	0.006740
1621.000000	0.006725

1620.750000	0.006703
1620.500000	0.006675
1620.250000	0.006644
1620.000000	0.006614
1619.750000	0.006588
1619.500000	0.006567
1619.250000	0.006548
1619.000000	0.006527
1618.750000	0.006501
1618.500000	0.006469
1618.250000	0.006434
1618.000000	0.006403
1617.750000	0.006396
1617.500000	0.006435
1617.250000	0.006526
1617.000000	0.006648
1616.750000	0.006762
1616.500000	0.006836
1616.250000	0.006874
1616.000000	0.006904
1615.750000	0.006947
1615.500000	0.006993
1615.250000	0.007025
1615.000000	0.007038
1614.750000	0.007047
1614.500000	0.007067

1614.250000	0.007104
1614.000000	0.007159
1613.750000	0.007231
1613.500000	0.007315
1613.250000	0.007407
1613.000000	0.007501
1612.750000	0.007595
1612.500000	0.007689
1612.250000	0.007784
1612.000000	0.007880
1611.750000	0.007978
1611.500000	0.008078
1611.250000	0.008181
1611.000000	0.008287
1610.750000	0.008396
1610.500000	0.008510
1610.250000	0.008628
1610.000000	0.008750
1609.750000	0.008875
1609.500000	0.009001
1609.250000	0.009129
1609.000000	0.009260
1608.750000	0.009397
1608.500000	0.009543
1608.250000	0.009701
1608.000000	0.009873

1607.750000	0.010059
1607.500000	0.010257
1607.250000	0.010464
1607.000000	0.010675
1606.750000	0.010888
1606.500000	0.011103
1606.250000	0.011321
1606.000000	0.011547
1605.750000	0.011782
1605.500000	0.012027
1605.250000	0.012280
1605.000000	0.012539
1604.750000	0.012801
1604.500000	0.013065
1604.250000	0.013329
1604.000000	0.013590
1603.750000	0.013846
1603.500000	0.014089
1603.250000	0.014313
1603.000000	0.014509
1602.750000	0.014675
1602.500000	0.014811
1602.250000	0.014919
1602.000000	0.015004
1601.750000	0.015068
1601.500000	0.015108

1601.250000	0.015121
1601.000000	0.015099
1600.750000	0.015039
1600.500000	0.014941
1600.250000	0.014808
1600.000000	0.014648
1599.750000	0.014463
1599.500000	0.014256
1599.250000	0.014028
1599.000000	0.013777
1598.750000	0.013502
1598.500000	0.013206
1598.250000	0.012892
1598.000000	0.012566
1597.750000	0.012232
1597.500000	0.011894
1597.250000	0.011553
1597.000000	0.011211
1596.750000	0.010868
1596.500000	0.010528
1596.250000	0.010194
1596.000000	0.009870
1595.750000	0.009558
1595.500000	0.009259
1595.250000	0.008969
1595.000000	0.008687

1594.750000	0.008409
1594.500000	0.008138
1594.250000	0.007874
1594.000000	0.007623
1593.750000	0.007390
1593.500000	0.007176
1593.250000	0.006982
1593.000000	0.006806
1592.750000	0.006643
1592.500000	0.006490
1592.250000	0.006346
1592.000000	0.006211
1591.750000	0.006085
1591.500000	0.005967
1591.250000	0.005860
1591.000000	0.005761
1590.750000	0.005669
1590.500000	0.005583
1590.250000	0.005501
1590.000000	0.005423
1589.750000	0.005348
1589.500000	0.005277
1589.250000	0.005212
1589.000000	0.005154
1588.750000	0.005103
1588.500000	0.005059

1588.250000	0.005023
1588.000000	0.004995
1587.750000	0.004973
1587.500000	0.004957
1587.250000	0.004945
1587.000000	0.004938
1586.750000	0.004935
1586.500000	0.004935
1586.250000	0.004939
1586.000000	0.004946
1585.750000	0.004958
1585.500000	0.004975
1585.250000	0.004995
1585.000000	0.005017
1584.750000	0.005041
1584.500000	0.005064
1584.250000	0.005086
1584.000000	0.005108
1583.750000	0.005128
1583.500000	0.005149
1583.250000	0.005170
1583.000000	0.005192
1582.750000	0.005214
1582.500000	0.005237
1582.250000	0.005261
1582.000000	0.005287

1581.750000	0.005315
1581.500000	0.005346
1581.250000	0.005379
1581.000000	0.005414
1580.750000	0.005449
1580.500000	0.005483
1580.250000	0.005516
1580.000000	0.005547
1579.750000	0.005576
1579.500000	0.005604
1579.250000	0.005631
1579.000000	0.005655
1578.750000	0.005673
1578.500000	0.005680
1578.250000	0.005676
1578.000000	0.005661
1577.750000	0.005640
1577.500000	0.005619
1577.250000	0.005617
1577.000000	0.005652
1576.750000	0.005725
1576.500000	0.005810
1576.250000	0.005870
1576.000000	0.005884
1575.750000	0.005867
1575.500000	0.005852

1575.250000	0.005854
1575.000000	0.005857
1574.750000	0.005837
1574.500000	0.005790
1574.250000	0.005731
1574.000000	0.005678
1573.750000	0.005635
1573.500000	0.005604
1573.250000	0.005584
1573.000000	0.005570
1572.750000	0.005560
1572.500000	0.005551
1572.250000	0.005542
1572.000000	0.005531
1571.750000	0.005517
1571.500000	0.005497
1571.250000	0.005472
1571.000000	0.005441
1570.750000	0.005412
1570.500000	0.005393
1570.250000	0.005396
1570.000000	0.005417
1569.750000	0.005439
1569.500000	0.005440
1569.250000	0.005415
1569.000000	0.005389

1568.750000	0.005391
1568.500000	0.005428
1568.250000	0.005488
1568.000000	0.005554
1567.750000	0.005616
1567.500000	0.005670
1567.250000	0.005716
1567.000000	0.005755
1566.750000	0.005787
1566.500000	0.005815
1566.250000	0.005842
1566.000000	0.005873
1565.750000	0.005912
1565.500000	0.005960
1565.250000	0.006017
1565.000000	0.006075
1564.750000	0.006129
1564.500000	0.006179
1564.250000	0.006229
1564.000000	0.006286
1563.750000	0.006353
1563.500000	0.006428
1563.250000	0.006504
1563.000000	0.006575
1562.750000	0.006638
1562.500000	0.006689

1562.250000	0.006725
1562.000000	0.006746
1561.750000	0.006756
1561.500000	0.006761
1561.250000	0.006767
1561.000000	0.006780
1560.750000	0.006806
1560.500000	0.006857
1560.250000	0.006949
1560.000000	0.007097
1559.750000	0.007299
1559.500000	0.007537
1559.250000	0.007782
1559.000000	0.008003
1558.750000	0.008166
1558.500000	0.008247
1558.250000	0.008259
1558.000000	0.008254
1557.750000	0.008295
1557.500000	0.008410
1557.250000	0.008581
1557.000000	0.008765
1556.750000	0.008928
1556.500000	0.009053
1556.250000	0.009144
1556.000000	0.009213

1555.750000	0.009277
1555.500000	0.009353
1555.250000	0.009467
1555.000000	0.009635
1554.750000	0.009853
1554.500000	0.010090
1554.250000	0.010307
1554.000000	0.010482
1553.750000	0.010620
1553.500000	0.010743
1553.250000	0.010866
1553.000000	0.010991
1552.750000	0.011111
1552.500000	0.011222
1552.250000	0.011326
1552.000000	0.011427
1551.750000	0.011526
1551.500000	0.011628
1551.250000	0.011739
1551.000000	0.011872
1550.750000	0.012036
1550.500000	0.012234
1550.250000	0.012448
1550.000000	0.012648
1549.750000	0.012810
1549.500000	0.012936

1549.250000	0.013047
1549.000000	0.013169
1548.750000	0.013315
1548.500000	0.013481
1548.250000	0.013656
1548.000000	0.013830
1547.750000	0.013992
1547.500000	0.014139
1547.250000	0.014270
1547.000000	0.014390
1546.750000	0.014506
1546.500000	0.014632
1546.250000	0.014781
1546.000000	0.014966
1545.750000	0.015190
1545.500000	0.015438
1545.250000	0.015678
1545.000000	0.015879
1544.750000	0.016029
1544.500000	0.016138
1544.250000	0.016231
1544.000000	0.016322
1543.750000	0.016420
1543.500000	0.016532
1543.250000	0.016665
1543.000000	0.016828

1542.750000	0.017024
1542.500000	0.017245
1542.250000	0.017466
1542.000000	0.017657
1541.750000	0.017802
1541.500000	0.017906
1541.250000	0.017994
1541.000000	0.018091
1540.750000	0.018217
1540.500000	0.018403
1540.250000	0.018704
1540.000000	0.019143
1539.750000	0.019636
1539.500000	0.020021
1539.250000	0.020191
1539.000000	0.020194
1538.750000	0.020170
1538.500000	0.020212
1538.250000	0.020316
1538.000000	0.020436
1537.750000	0.020545
1537.500000	0.020642
1537.250000	0.020741
1537.000000	0.020848
1536.750000	0.020966
1536.500000	0.021093

1536.250000	0.021223
1536.000000	0.021344
1535.750000	0.021444
1535.500000	0.021522
1535.250000	0.021587
1535.000000	0.021653
1534.750000	0.021745
1534.500000	0.021888
1534.250000	0.022093
1534.000000	0.022329
1533.750000	0.022529
1533.500000	0.022628
1533.250000	0.022618
1533.000000	0.022556
1532.750000	0.022522
1532.500000	0.022554
1532.250000	0.022642
1532.000000	0.022757
1531.750000	0.022872
1531.500000	0.022972
1531.250000	0.023052
1531.000000	0.023113
1530.750000	0.023164
1530.500000	0.023215
1530.250000	0.023273
1530.000000	0.023340

1529.750000	0.023413
1529.500000	0.023488
1529.250000	0.023564
1529.000000	0.023637
1528.750000	0.023704
1528.500000	0.023761
1528.250000	0.023800
1528.000000	0.023814
1527.750000	0.023795
1527.500000	0.023746
1527.250000	0.023681
1527.000000	0.023618
1526.750000	0.023576
1526.500000	0.023575
1526.250000	0.023622
1526.000000	0.023710
1525.750000	0.023805
1525.500000	0.023859
1525.250000	0.023841
1525.000000	0.023752
1524.750000	0.023630
1524.500000	0.023515
1524.250000	0.023435
1524.000000	0.023396
1523.750000	0.023393
1523.500000	0.023416

1523.250000	0.023455
1523.000000	0.023502
1522.750000	0.023546
1522.500000	0.023580
1522.250000	0.023607
1522.000000	0.023641
1521.750000	0.023691
1521.500000	0.023744
1521.250000	0.023757
1521.000000	0.023686
1520.750000	0.023525
1520.500000	0.023322
1520.250000	0.023141
1520.000000	0.023020
1519.750000	0.022955
1519.500000	0.022930
1519.250000	0.022929
1519.000000	0.022947
1518.750000	0.022983
1518.500000	0.023041
1518.250000	0.023133
1518.000000	0.023280
1517.750000	0.023491
1517.500000	0.023739
1517.250000	0.023957
1517.000000	0.024079

1516.750000	0.024090
1516.500000	0.024051
1516.250000	0.024042
1516.000000	0.024112
1515.750000	0.024260
1515.500000	0.024456
1515.250000	0.024673
1515.000000	0.024888
1514.750000	0.025085
1514.500000	0.025259
1514.250000	0.025414
1514.000000	0.025567
1513.750000	0.025732
1513.500000	0.025926
1513.250000	0.026158
1513.000000	0.026430
1512.750000	0.026725
1512.500000	0.027005
1512.250000	0.027231
1512.000000	0.027382
1511.750000	0.027467
1511.500000	0.027515
1511.250000	0.027556
1511.000000	0.027607
1510.750000	0.027671
1510.500000	0.027740

1510.250000	0.027803
1510.000000	0.027843
1509.750000	0.027843
1509.500000	0.027792
1509.250000	0.027688
1509.000000	0.027540
1508.750000	0.027370
1508.500000	0.027201
1508.250000	0.027045
1508.000000	0.026904
1507.750000	0.026780
1507.500000	0.026661
1507.250000	0.026484
1507.000000	0.026119
1506.750000	0.025436
1506.500000	0.024418
1506.250000	0.023220
1506.000000	0.022097
1505.750000	0.021235
1505.500000	0.020658
1505.250000	0.020272
1505.000000	0.019974
1504.750000	0.019710
1504.500000	0.019454
1504.250000	0.019189
1504.000000	0.018906

1503.750000	0.018608
1503.500000	0.018300
1503.250000	0.017985
1503.000000	0.017661
1502.750000	0.017322
1502.500000	0.016965
1502.250000	0.016592
1502.000000	0.016205
1501.750000	0.015811
1501.500000	0.015419
1501.250000	0.015042
1501.000000	0.014692
1500.750000	0.014371
1500.500000	0.014081
1500.250000	0.013817
1500.000000	0.013573
1499.750000	0.013340
1499.500000	0.013106
1499.250000	0.012850
1499.000000	0.012558
1498.750000	0.012234
1498.500000	0.011904
1498.250000	0.011605
1498.000000	0.011359
1497.750000	0.011171
1497.500000	0.011029

1497.250000	0.010924
1497.000000	0.010845
1496.750000	0.010772
1496.500000	0.010671
1496.250000	0.010519
1496.000000	0.010323
1495.750000	0.010121
1495.500000	0.009954
1495.250000	0.009840
1495.000000	0.009771
1494.750000	0.009723
1494.500000	0.009682
1494.250000	0.009644
1494.000000	0.009610
1493.750000	0.009578
1493.500000	0.009549
1493.250000	0.009524
1493.000000	0.009503
1492.750000	0.009486
1492.500000	0.009470
1492.250000	0.009454
1492.000000	0.009437
1491.750000	0.009420
1491.500000	0.009410
1491.250000	0.009414
1491.000000	0.009439

1490.750000	0.009486
1490.500000	0.009540
1490.250000	0.009579
1490.000000	0.009583
1489.750000	0.009554
1489.500000	0.009514
1489.250000	0.009488
1489.000000	0.009488
1488.750000	0.009513
1488.500000	0.009555
1488.250000	0.009609
1488.000000	0.009672
1487.750000	0.009739
1487.500000	0.009802
1487.250000	0.009853
1487.000000	0.009886
1486.750000	0.009906
1486.500000	0.009918
1486.250000	0.009931
1486.000000	0.009949
1485.750000	0.009972
1485.500000	0.010001
1485.250000	0.010034
1485.000000	0.010071
1484.750000	0.010110
1484.500000	0.010150

1484.250000	0.010187
1484.000000	0.010220
1483.750000	0.010249
1483.500000	0.010276
1483.250000	0.010301
1483.000000	0.010326
1482.750000	0.010352
1482.500000	0.010379
1482.250000	0.010408
1482.000000	0.010438
1481.750000	0.010469
1481.500000	0.010502
1481.250000	0.010536
1481.000000	0.010569
1480.750000	0.010604
1480.500000	0.010642
1480.250000	0.010685
1480.000000	0.010735
1479.750000	0.010791
1479.500000	0.010851
1479.250000	0.010914
1479.000000	0.010974
1478.750000	0.011029
1478.500000	0.011077
1478.250000	0.011117
1478.000000	0.011150

1477.750000	0.011182
1477.500000	0.011219
1477.250000	0.011271
1477.000000	0.011344
1476.750000	0.011434
1476.500000	0.011525
1476.250000	0.011599
1476.000000	0.011648
1475.750000	0.011683
1475.500000	0.011724
1475.250000	0.011782
1475.000000	0.011857
1474.750000	0.011944
1474.500000	0.012039
1474.250000	0.012144
1474.000000	0.012263
1473.750000	0.012395
1473.500000	0.012542
1473.250000	0.012704
1473.000000	0.012880
1472.750000	0.013067
1472.500000	0.013255
1472.250000	0.013429
1472.000000	0.013579
1471.750000	0.013708
1471.500000	0.013830

1471.250000	0.013959
1471.000000	0.014100
1470.750000	0.014253
1470.500000	0.014412
1470.250000	0.014575
1470.000000	0.014741
1469.750000	0.014908
1469.500000	0.015073
1469.250000	0.015233
1469.000000	0.015385
1468.750000	0.015532
1468.500000	0.015672
1468.250000	0.015808
1468.000000	0.015939
1467.750000	0.016063
1467.500000	0.016175
1467.250000	0.016270
1467.000000	0.016347
1466.750000	0.016409
1466.500000	0.016464
1466.250000	0.016521
1466.000000	0.016597
1465.750000	0.016706
1465.500000	0.016853
1465.250000	0.017023
1465.000000	0.017182

1464.750000	0.017301
1464.500000	0.017378
1464.250000	0.017434
1464.000000	0.017495
1463.750000	0.017575
1463.500000	0.017671
1463.250000	0.017773
1463.000000	0.017875
1462.750000	0.017975
1462.500000	0.018073
1462.250000	0.018166
1462.000000	0.018251
1461.750000	0.018328
1461.500000	0.018395
1461.250000	0.018452
1461.000000	0.018500
1460.750000	0.018543
1460.500000	0.018583
1460.250000	0.018625
1460.000000	0.018673
1459.750000	0.018731
1459.500000	0.018799
1459.250000	0.018871
1459.000000	0.018940
1458.750000	0.018998
1458.500000	0.019039

1458.250000	0.019067
1458.000000	0.019098
1457.750000	0.019160
1457.500000	0.019276
1457.250000	0.019440
1457.000000	0.019614
1456.750000	0.019757
1456.500000	0.019857
1456.250000	0.019930
1456.000000	0.019996
1455.750000	0.020059
1455.500000	0.020113
1455.250000	0.020146
1455.000000	0.020161
1454.750000	0.020165
1454.500000	0.020163
1454.250000	0.020161
1454.000000	0.020161
1453.750000	0.020166
1453.500000	0.020179
1453.250000	0.020197
1453.000000	0.020218
1452.750000	0.020235
1452.500000	0.020238
1452.250000	0.020220
1452.000000	0.020177

1451.750000	0.020113
1451.500000	0.020039
1451.250000	0.019964
1451.000000	0.019897
1450.750000	0.019838
1450.500000	0.019784
1450.250000	0.019730
1450.000000	0.019675
1449.750000	0.019618
1449.500000	0.019563
1449.250000	0.019514
1449.000000	0.019475
1448.750000	0.019442
1448.500000	0.019403
1448.250000	0.019338
1448.000000	0.019230
1447.750000	0.019078
1447.500000	0.018906
1447.250000	0.018743
1447.000000	0.018614
1446.750000	0.018523
1446.500000	0.018463
1446.250000	0.018419
1446.000000	0.018381
1445.750000	0.018340
1445.500000	0.018290

1445.250000	0.018231
1445.000000	0.018163
1444.750000	0.018091
1444.500000	0.018018
1444.250000	0.017946
1444.000000	0.017878
1443.750000	0.017811
1443.500000	0.017746
1443.250000	0.017681
1443.000000	0.017615
1442.750000	0.017547
1442.500000	0.017477
1442.250000	0.017406
1442.000000	0.017335
1441.750000	0.017262
1441.500000	0.017188
1441.250000	0.017114
1441.000000	0.017040
1440.750000	0.016968
1440.500000	0.016895
1440.250000	0.016822
1440.000000	0.016747
1439.750000	0.016671
1439.500000	0.016592
1439.250000	0.016511
1439.000000	0.016425

1438.750000	0.016337
1438.500000	0.016248
1438.250000	0.016158
1438.000000	0.016070
1437.750000	0.015995
1437.500000	0.015942
1437.250000	0.015894
1437.000000	0.015801
1436.750000	0.015618
1436.500000	0.015348
1436.250000	0.015053
1436.000000	0.014815
1435.750000	0.014674
1435.500000	0.014611
1435.250000	0.014579
1435.000000	0.014544
1434.750000	0.014499
1434.500000	0.014442
1434.250000	0.014370
1434.000000	0.014278
1433.750000	0.014167
1433.500000	0.014040
1433.250000	0.013899
1433.000000	0.013750
1432.750000	0.013596
1432.500000	0.013444

1432.250000	0.013298
1432.000000	0.013162
1431.750000	0.013036
1431.500000	0.012919
1431.250000	0.012812
1431.000000	0.012712
1430.750000	0.012614
1430.500000	0.012510
1430.250000	0.012390
1430.000000	0.012250
1429.750000	0.012096
1429.500000	0.011944
1429.250000	0.011811
1429.000000	0.011709
1428.750000	0.011638
1428.500000	0.011590
1428.250000	0.011556
1428.000000	0.011525
1427.750000	0.011492
1427.500000	0.011456
1427.250000	0.011418
1427.000000	0.011380
1426.750000	0.011344
1426.500000	0.011313
1426.250000	0.011286
1426.000000	0.011263

1425.750000	0.011246
1425.500000	0.011236
1425.250000	0.011235
1425.000000	0.011247
1424.750000	0.011273
1424.500000	0.011308
1424.250000	0.011344
1424.000000	0.011367
1423.750000	0.011370
1423.500000	0.011359
1423.250000	0.011347
1423.000000	0.011349
1422.750000	0.011371
1422.500000	0.011414
1422.250000	0.011468
1422.000000	0.011524
1421.750000	0.011571
1421.500000	0.011607
1421.250000	0.011633
1421.000000	0.011653
1420.750000	0.011673
1420.500000	0.011705
1420.250000	0.011765
1420.000000	0.011861
1419.750000	0.011974
1419.500000	0.012061

1419.250000	0.012088
1419.000000	0.012058
1418.750000	0.012009
1418.500000	0.011983
1418.250000	0.011998
1418.000000	0.012042
1417.750000	0.012091
1417.500000	0.012134
1417.250000	0.012166
1417.000000	0.012189
1416.750000	0.012206
1416.500000	0.012222
1416.250000	0.012240
1416.000000	0.012266
1415.750000	0.012297
1415.500000	0.012330
1415.250000	0.012364
1415.000000	0.012398
1414.750000	0.012431
1414.500000	0.012466
1414.250000	0.012502
1414.000000	0.012540
1413.750000	0.012580
1413.500000	0.012619
1413.250000	0.012658
1413.000000	0.012695

1412.750000	0.012731
1412.500000	0.012767
1412.250000	0.012806
1412.000000	0.012847
1411.750000	0.012891
1411.500000	0.012937
1411.250000	0.012983
1411.000000	0.013030
1410.750000	0.013079
1410.500000	0.013131
1410.250000	0.013188
1410.000000	0.013248
1409.750000	0.013309
1409.500000	0.013369
1409.250000	0.013425
1409.000000	0.013478
1408.750000	0.013528
1408.500000	0.013577
1408.250000	0.013627
1408.000000	0.013678
1407.750000	0.013729
1407.500000	0.013778
1407.250000	0.013825
1407.000000	0.013870
1406.750000	0.013916
1406.500000	0.013964

1406.250000	0.014017
1406.000000	0.014076
1405.750000	0.014138
1405.500000	0.014194
1405.250000	0.014233
1405.000000	0.014245
1404.750000	0.014233
1404.500000	0.014209
1404.250000	0.014189
1404.000000	0.014186
1403.750000	0.014201
1403.500000	0.014230
1403.250000	0.014265
1403.000000	0.014297
1402.750000	0.014321
1402.500000	0.014332
1402.250000	0.014331
1402.000000	0.014318
1401.750000	0.014298
1401.500000	0.014272
1401.250000	0.014245
1401.000000	0.014217
1400.750000	0.014193
1400.500000	0.014174
1400.250000	0.014161
1400.000000	0.014155

1399.750000	0.014152
1399.500000	0.014142
1399.250000	0.014111
1399.000000	0.014055
1398.750000	0.013978
1398.500000	0.013897
1398.250000	0.013826
1398.000000	0.013775
1397.750000	0.013744
1397.500000	0.013727
1397.250000	0.013717
1397.000000	0.013711
1396.750000	0.013706
1396.500000	0.013701
1396.250000	0.013699
1396.000000	0.013702
1395.750000	0.013713
1395.500000	0.013733
1395.250000	0.013758
1395.000000	0.013780
1394.750000	0.013790
1394.500000	0.013790
1394.250000	0.013788
1394.000000	0.013796
1393.750000	0.013824
1393.500000	0.013873

1393.250000	0.013936
1393.000000	0.014007
1392.750000	0.014079
1392.500000	0.014151
1392.250000	0.014222
1392.000000	0.014292
1391.750000	0.014361
1391.500000	0.014430
1391.250000	0.014496
1391.000000	0.014561
1390.750000	0.014623
1390.500000	0.014683
1390.250000	0.014741
1390.000000	0.014798
1389.750000	0.014853
1389.500000	0.014907
1389.250000	0.014959
1389.000000	0.015014
1388.750000	0.015074
1388.500000	0.015147
1388.250000	0.015237
1388.000000	0.015343
1387.750000	0.015458
1387.500000	0.015568
1387.250000	0.015661
1387.000000	0.015736

1386.750000	0.015802
1386.500000	0.015869
1386.250000	0.015946
1386.000000	0.016035
1385.750000	0.016131
1385.500000	0.016233
1385.250000	0.016337
1385.000000	0.016442
1384.750000	0.016547
1384.500000	0.016651
1384.250000	0.016755
1384.000000	0.016857
1383.750000	0.016955
1383.500000	0.017048
1383.250000	0.017133
1383.000000	0.017210
1382.750000	0.017279
1382.500000	0.017341
1382.250000	0.017398
1382.000000	0.017451
1381.750000	0.017498
1381.500000	0.017539
1381.250000	0.017572
1381.000000	0.017596
1380.750000	0.017612
1380.500000	0.017620

1380.250000	0.017623
1380.000000	0.017618
1379.750000	0.017606
1379.500000	0.017585
1379.250000	0.017553
1379.000000	0.017510
1378.750000	0.017456
1378.500000	0.017390
1378.250000	0.017315
1378.000000	0.017232
1377.750000	0.017143
1377.500000	0.017048
1377.250000	0.016952
1377.000000	0.016856
1376.750000	0.016763
1376.500000	0.016673
1376.250000	0.016586
1376.000000	0.016501
1375.750000	0.016413
1375.500000	0.016318
1375.250000	0.016211
1375.000000	0.016089
1374.750000	0.015951
1374.500000	0.015796
1374.250000	0.015628
1374.000000	0.015452

1373.750000	0.015277
1373.500000	0.015114
1373.250000	0.014970
1373.000000	0.014851
1372.750000	0.014758
1372.500000	0.014689
1372.250000	0.014637
1372.000000	0.014595
1371.750000	0.014557
1371.500000	0.014517
1371.250000	0.014472
1371.000000	0.014424
1370.750000	0.014375
1370.500000	0.014328
1370.250000	0.014287
1370.000000	0.014252
1369.750000	0.014222
1369.500000	0.014195
1369.250000	0.014165
1369.000000	0.014129
1368.750000	0.014087
1368.500000	0.014040
1368.250000	0.013994
1368.000000	0.013956
1367.750000	0.013930
1367.500000	0.013920

1367.250000	0.013929
1367.000000	0.013956
1366.750000	0.013998
1366.500000	0.014052
1366.250000	0.014111
1366.000000	0.014164
1365.750000	0.014204
1365.500000	0.014221
1365.250000	0.014213
1365.000000	0.014182
1364.750000	0.014131
1364.500000	0.014069
1364.250000	0.014001
1364.000000	0.013932
1363.750000	0.013862
1363.500000	0.013784
1363.250000	0.013693
1363.000000	0.013586
1362.750000	0.013473
1362.500000	0.013366
1362.250000	0.013276
1362.000000	0.013206
1361.750000	0.013149
1361.500000	0.013091
1361.250000	0.013022
1361.000000	0.012935

1360.750000	0.012831
1360.500000	0.012717
1360.250000	0.012600
1360.000000	0.012490
1359.750000	0.012389
1359.500000	0.012296
1359.250000	0.012207
1359.000000	0.012121
1358.750000	0.012035
1358.500000	0.011950
1358.250000	0.011869
1358.000000	0.011794
1357.750000	0.011727
1357.500000	0.011670
1357.250000	0.011621
1357.000000	0.011580
1356.750000	0.011546
1356.500000	0.011517
1356.250000	0.011494
1356.000000	0.011475
1355.750000	0.011461
1355.500000	0.011448
1355.250000	0.011434
1355.000000	0.011415
1354.750000	0.011392
1354.500000	0.011363

1354.250000	0.011332
1354.000000	0.011302
1353.750000	0.011275
1353.500000	0.011252
1353.250000	0.011232
1353.000000	0.011215
1352.750000	0.011198
1352.500000	0.011181
1352.250000	0.011162
1352.000000	0.011142
1351.750000	0.011121
1351.500000	0.011097
1351.250000	0.011071
1351.000000	0.011040
1350.750000	0.011004
1350.500000	0.010965
1350.250000	0.010924
1350.000000	0.010882
1349.750000	0.010839
1349.500000	0.010796
1349.250000	0.010751
1349.000000	0.010703
1348.750000	0.010652
1348.500000	0.010598
1348.250000	0.010544
1348.000000	0.010492

1347.750000	0.010443
1347.500000	0.010397
1347.250000	0.010352
1347.000000	0.010308
1346.750000	0.010262
1346.500000	0.010213
1346.250000	0.010163
1346.000000	0.010113
1345.750000	0.010064
1345.500000	0.010015
1345.250000	0.009969
1345.000000	0.009923
1344.750000	0.009878
1344.500000	0.009835
1344.250000	0.009794
1344.000000	0.009755
1343.750000	0.009717
1343.500000	0.009680
1343.250000	0.009644
1343.000000	0.009608
1342.750000	0.009574
1342.500000	0.009543
1342.250000	0.009518
1342.000000	0.009499
1341.750000	0.009487
1341.500000	0.009480

1341.250000	0.009478
1341.000000	0.009476
1340.750000	0.009471
1340.500000	0.009461
1340.250000	0.009444
1340.000000	0.009422
1339.750000	0.009397
1339.500000	0.009372
1339.250000	0.009349
1339.000000	0.009331
1338.750000	0.009318
1338.500000	0.009312
1338.250000	0.009315
1338.000000	0.009327
1337.750000	0.009349
1337.500000	0.009379
1337.250000	0.009415
1337.000000	0.009453
1336.750000	0.009490
1336.500000	0.009524
1336.250000	0.009557
1336.000000	0.009590
1335.750000	0.009626
1335.500000	0.009667
1335.250000	0.009713
1335.000000	0.009765

1334.750000	0.009819
1334.500000	0.009874
1334.250000	0.009930
1334.000000	0.009986
1333.750000	0.010045
1333.500000	0.010107
1333.250000	0.010174
1333.000000	0.010243
1332.750000	0.010314
1332.500000	0.010384
1332.250000	0.010452
1332.000000	0.010516
1331.750000	0.010578
1331.500000	0.010640
1331.250000	0.010705
1331.000000	0.010774
1330.750000	0.010847
1330.500000	0.010925
1330.250000	0.011005
1330.000000	0.011085
1329.750000	0.011165
1329.500000	0.011243
1329.250000	0.011319
1329.000000	0.011393
1328.750000	0.011466
1328.500000	0.011536

1328.250000	0.011605
1328.000000	0.011674
1327.750000	0.011743
1327.500000	0.011812
1327.250000	0.011883
1327.000000	0.011956
1326.750000	0.012030
1326.500000	0.012103
1326.250000	0.012175
1326.000000	0.012246
1325.750000	0.012316
1325.500000	0.012384
1325.250000	0.012453
1325.000000	0.012522
1324.750000	0.012592
1324.500000	0.012662
1324.250000	0.012733
1324.000000	0.012804
1323.750000	0.012874
1323.500000	0.012943
1323.250000	0.013008
1323.000000	0.013070
1322.750000	0.013128
1322.500000	0.013182
1322.250000	0.013231
1322.000000	0.013275

1321.750000	0.013316
1321.500000	0.013353
1321.250000	0.013387
1321.000000	0.013418
1320.750000	0.013446
1320.500000	0.013472
1320.250000	0.013495
1320.000000	0.013515
1319.750000	0.013533
1319.500000	0.013547
1319.250000	0.013556
1319.000000	0.013561
1318.750000	0.013561
1318.500000	0.013559
1318.250000	0.013555
1318.000000	0.013552
1317.750000	0.013549
1317.500000	0.013546
1317.250000	0.013541
1317.000000	0.013532
1316.750000	0.013521
1316.500000	0.013506
1316.250000	0.013492
1316.000000	0.013479
1315.750000	0.013471
1315.500000	0.013469

1315.250000	0.013471
1315.000000	0.013477
1314.750000	0.013483
1314.500000	0.013489
1314.250000	0.013494
1314.000000	0.013496
1313.750000	0.013496
1313.500000	0.013494
1313.250000	0.013489
1313.000000	0.013478
1312.750000	0.013462
1312.500000	0.013440
1312.250000	0.013415
1312.000000	0.013389
1311.750000	0.013366
1311.500000	0.013349
1311.250000	0.013340
1311.000000	0.013337
1310.750000	0.013339
1310.500000	0.013342
1310.250000	0.013346
1310.000000	0.013350
1309.750000	0.013356
1309.500000	0.013364
1309.250000	0.013377
1309.000000	0.013396

1308.750000	0.013420
1308.500000	0.013449
1308.250000	0.013482
1308.000000	0.013518
1307.750000	0.013558
1307.500000	0.013603
1307.250000	0.013653
1307.000000	0.013709
1306.750000	0.013772
1306.500000	0.013841
1306.250000	0.013914
1306.000000	0.013992
1305.750000	0.014074
1305.500000	0.014160
1305.250000	0.014251
1305.000000	0.014347
1304.750000	0.014446
1304.500000	0.014549
1304.250000	0.014655
1304.000000	0.014764
1303.750000	0.014876
1303.500000	0.014992
1303.250000	0.015113
1303.000000	0.015237
1302.750000	0.015364
1302.500000	0.015494

1302.250000	0.015625
1302.000000	0.015756
1301.750000	0.015887
1301.500000	0.016020
1301.250000	0.016153
1301.000000	0.016287
1300.750000	0.016422
1300.500000	0.016558
1300.250000	0.016694
1300.000000	0.016830
1299.750000	0.016967
1299.500000	0.017104
1299.250000	0.017241
1299.000000	0.017376
1298.750000	0.017509
1298.500000	0.017638
1298.250000	0.017760
1298.000000	0.017877
1297.750000	0.017986
1297.500000	0.018088
1297.250000	0.018182
1297.000000	0.018267
1296.750000	0.018343
1296.500000	0.018408
1296.250000	0.018462
1296.000000	0.018506

1295.750000	0.018540
1295.500000	0.018564
1295.250000	0.018578
1295.000000	0.018583
1294.750000	0.018576
1294.500000	0.018557
1294.250000	0.018525
1294.000000	0.018482
1293.750000	0.018428
1293.500000	0.018363
1293.250000	0.018290
1293.000000	0.018209
1292.750000	0.018119
1292.500000	0.018023
1292.250000	0.017919
1292.000000	0.017809
1291.750000	0.017695
1291.500000	0.017576
1291.250000	0.017455
1291.000000	0.017331
1290.750000	0.017205
1290.500000	0.017078
1290.250000	0.016952
1290.000000	0.016828
1289.750000	0.016707
1289.500000	0.016589

1289.250000	0.016475
1289.000000	0.016364
1288.750000	0.016254
1288.500000	0.016146
1288.250000	0.016039
1288.000000	0.015935
1287.750000	0.015835
1287.500000	0.015742
1287.250000	0.015657
1287.000000	0.015579
1286.750000	0.015509
1286.500000	0.015445
1286.250000	0.015386
1286.000000	0.015332
1285.750000	0.015282
1285.500000	0.015239
1285.250000	0.015202
1285.000000	0.015173
1284.750000	0.015151
1284.500000	0.015137
1284.250000	0.015131
1284.000000	0.015132
1283.750000	0.015140
1283.500000	0.015156
1283.250000	0.015179
1283.000000	0.015208

1282.750000	0.015244
1282.500000	0.015285
1282.250000	0.015331
1282.000000	0.015383
1281.750000	0.015440
1281.500000	0.015503
1281.250000	0.015572
1281.000000	0.015648
1280.750000	0.015729
1280.500000	0.015816
1280.250000	0.015906
1280.000000	0.016001
1279.750000	0.016100
1279.500000	0.016204
1279.250000	0.016312
1279.000000	0.016427
1278.750000	0.016547
1278.500000	0.016672
1278.250000	0.016802
1278.000000	0.016936
1277.750000	0.017073
1277.500000	0.017214
1277.250000	0.017358
1277.000000	0.017506
1276.750000	0.017656
1276.500000	0.017810

1276.250000	0.017966
1276.000000	0.018124
1275.750000	0.018283
1275.500000	0.018443
1275.250000	0.018603
1275.000000	0.018760
1274.750000	0.018916
1274.500000	0.019069
1274.250000	0.019220
1274.000000	0.019371
1273.750000	0.019524
1273.500000	0.019679
1273.250000	0.019837
1273.000000	0.019997
1272.750000	0.020156
1272.500000	0.020312
1272.250000	0.020463
1272.000000	0.020608
1271.750000	0.020747
1271.500000	0.020884
1271.250000	0.021019
1271.000000	0.021155
1270.750000	0.021293
1270.500000	0.021434
1270.250000	0.021575
1270.000000	0.021716

1269.750000	0.021856
1269.500000	0.021994
1269.250000	0.022129
1269.000000	0.022260
1268.750000	0.022389
1268.500000	0.022516
1268.250000	0.022641
1268.000000	0.022767
1267.750000	0.022894
1267.500000	0.023026
1267.250000	0.023161
1267.000000	0.023303
1266.750000	0.023450
1266.500000	0.023601
1266.250000	0.023755
1266.000000	0.023910
1265.750000	0.024065
1265.500000	0.024218
1265.250000	0.024369
1265.000000	0.024519
1264.750000	0.024669
1264.500000	0.024821
1264.250000	0.024976
1264.000000	0.025134
1263.750000	0.025296
1263.500000	0.025462

1263.250000	0.025631
1263.000000	0.025801
1262.750000	0.025972
1262.500000	0.026144
1262.250000	0.026319
1262.000000	0.026496
1261.750000	0.026678
1261.500000	0.026866
1261.250000	0.027057
1261.000000	0.027253
1260.750000	0.027449
1260.500000	0.027646
1260.250000	0.027841
1260.000000	0.028035
1259.750000	0.028228
1259.500000	0.028422
1259.250000	0.028615
1259.000000	0.028810
1258.750000	0.029004
1258.500000	0.029199
1258.250000	0.029393
1258.000000	0.029587
1257.750000	0.029783
1257.500000	0.029981
1257.250000	0.030182
1257.000000	0.030388

1256.750000	0.030597
1256.500000	0.030809
1256.250000	0.031022
1256.000000	0.031234
1255.750000	0.031444
1255.500000	0.031650
1255.250000	0.031851
1255.000000	0.032047
1254.750000	0.032238
1254.500000	0.032424
1254.250000	0.032607
1254.000000	0.032786
1253.750000	0.032962
1253.500000	0.033134
1253.250000	0.033303
1253.000000	0.033468
1252.750000	0.033629
1252.500000	0.033788
1252.250000	0.033945
1252.000000	0.034099
1251.750000	0.034251
1251.500000	0.034398
1251.250000	0.034538
1251.000000	0.034671
1250.750000	0.034794
1250.500000	0.034909

1250.250000	0.035018
1250.000000	0.035123
1249.750000	0.035224
1249.500000	0.035323
1249.250000	0.035416
1249.000000	0.035502
1248.750000	0.035579
1248.500000	0.035645
1248.250000	0.035703
1248.000000	0.035754
1247.750000	0.035801
1247.500000	0.035846
1247.250000	0.035889
1247.000000	0.035931
1246.750000	0.035969
1246.500000	0.036002
1246.250000	0.036032
1246.000000	0.036056
1245.750000	0.036077
1245.500000	0.036095
1245.250000	0.036108
1245.000000	0.036116
1244.750000	0.036118
1244.500000	0.036115
1244.250000	0.036108
1244.000000	0.036098

1243.750000	0.036089
1243.500000	0.036081
1243.250000	0.036076
1243.000000	0.036073
1242.750000	0.036069
1242.500000	0.036063
1242.250000	0.036054
1242.000000	0.036042
1241.750000	0.036027
1241.500000	0.036010
1241.250000	0.035992
1241.000000	0.035974
1240.750000	0.035956
1240.500000	0.035939
1240.250000	0.035923
1240.000000	0.035910
1239.750000	0.035898
1239.500000	0.035887
1239.250000	0.035876
1239.000000	0.035861
1238.750000	0.035841
1238.500000	0.035817
1238.250000	0.035788
1238.000000	0.035757
1237.750000	0.035726
1237.500000	0.035695

1237.250000	0.035666
1237.000000	0.035637
1236.750000	0.035605
1236.500000	0.035570
1236.250000	0.035530
1236.000000	0.035485
1235.750000	0.035434
1235.500000	0.035379
1235.250000	0.035319
1235.000000	0.035255
1234.750000	0.035185
1234.500000	0.035112
1234.250000	0.035034
1234.000000	0.034953
1233.750000	0.034868
1233.500000	0.034780
1233.250000	0.034687
1233.000000	0.034588
1232.750000	0.034480
1232.500000	0.034364
1232.250000	0.034239
1232.000000	0.034108
1231.750000	0.033970
1231.500000	0.033829
1231.250000	0.033683
1231.000000	0.033533

1230.750000	0.033378
1230.500000	0.033218
1230.250000	0.033051
1230.000000	0.032879
1229.750000	0.032701
1229.500000	0.032518
1229.250000	0.032328
1229.000000	0.032131
1228.750000	0.031928
1228.500000	0.031719
1228.250000	0.031505
1228.000000	0.031289
1227.750000	0.031071
1227.500000	0.030853
1227.250000	0.030634
1227.000000	0.030413
1226.750000	0.030190
1226.500000	0.029962
1226.250000	0.029731
1226.000000	0.029497
1225.750000	0.029263
1225.500000	0.029030
1225.250000	0.028800
1225.000000	0.028573
1224.750000	0.028352
1224.500000	0.028135

1224.250000	0.027923
1224.000000	0.027715
1223.750000	0.027510
1223.500000	0.027309
1223.250000	0.027108
1223.000000	0.026908
1222.750000	0.026708
1222.500000	0.026509
1222.250000	0.026315
1222.000000	0.026128
1221.750000	0.025952
1221.500000	0.025790
1221.250000	0.025645
1221.000000	0.025518
1220.750000	0.025409
1220.500000	0.025316
1220.250000	0.025239
1220.000000	0.025176
1219.750000	0.025125
1219.500000	0.025082
1219.250000	0.025045
1219.000000	0.025011
1218.750000	0.024976
1218.500000	0.024937
1218.250000	0.024891
1218.000000	0.024835

1217.750000	0.024768
1217.500000	0.024688
1217.250000	0.024594
1217.000000	0.024486
1216.750000	0.024365
1216.500000	0.024233
1216.250000	0.024093
1216.000000	0.023950
1215.750000	0.023807
1215.500000	0.023666
1215.250000	0.023530
1215.000000	0.023400
1214.750000	0.023277
1214.500000	0.023161
1214.250000	0.023053
1214.000000	0.022954
1213.750000	0.022864
1213.500000	0.022782
1213.250000	0.022708
1213.000000	0.022641
1212.750000	0.022580
1212.500000	0.022526
1212.250000	0.022477
1212.000000	0.022435
1211.750000	0.022398
1211.500000	0.022366

1211.250000	0.022337
1211.000000	0.022310
1210.750000	0.022285
1210.500000	0.022263
1210.250000	0.022244
1210.000000	0.022232
1209.750000	0.022227
1209.500000	0.022230
1209.250000	0.022240
1209.000000	0.022256
1208.750000	0.022276
1208.500000	0.022299
1208.250000	0.022325
1208.000000	0.022354
1207.750000	0.022387
1207.500000	0.022424
1207.250000	0.022466
1207.000000	0.022512
1206.750000	0.022560
1206.500000	0.022609
1206.250000	0.022659
1206.000000	0.022711
1205.750000	0.022765
1205.500000	0.022822
1205.250000	0.022883
1205.000000	0.022946

1204.750000	0.023013
1204.500000	0.023082
1204.250000	0.023154
1204.000000	0.023230
1203.750000	0.023309
1203.500000	0.023393
1203.250000	0.023481
1203.000000	0.023570
1202.750000	0.023661
1202.500000	0.023751
1202.250000	0.023841
1202.000000	0.023932
1201.750000	0.024024
1201.500000	0.024119
1201.250000	0.024218
1201.000000	0.024318
1200.750000	0.024420
1200.500000	0.024521
1200.250000	0.024620
1200.000000	0.024716
1199.750000	0.024809
1199.500000	0.024900
1199.250000	0.024990
1199.000000	0.025078
1198.750000	0.025167
1198.500000	0.025257

1198.250000	0.025348
1198.000000	0.025443
1197.750000	0.025540
1197.500000	0.025641
1197.250000	0.025746
1197.000000	0.025854
1196.750000	0.025965
1196.500000	0.026078
1196.250000	0.026193
1196.000000	0.026311
1195.750000	0.026431
1195.500000	0.026553
1195.250000	0.026675
1195.000000	0.026796
1194.750000	0.026917
1194.500000	0.027037
1194.250000	0.027156
1194.000000	0.027277
1193.750000	0.027401
1193.500000	0.027529
1193.250000	0.027662
1193.000000	0.027802
1192.750000	0.027947
1192.500000	0.028099
1192.250000	0.028257
1192.000000	0.028422

1191.750000	0.028593
1191.500000	0.028770
1191.250000	0.028952
1191.000000	0.029136
1190.750000	0.029321
1190.500000	0.029506
1190.250000	0.029690
1190.000000	0.029876
1189.750000	0.030062
1189.500000	0.030251
1189.250000	0.030443
1189.000000	0.030639
1188.750000	0.030838
1188.500000	0.031041
1188.250000	0.031250
1188.000000	0.031464
1187.750000	0.031684
1187.500000	0.031908
1187.250000	0.032134
1187.000000	0.032361
1186.750000	0.032586
1186.500000	0.032808
1186.250000	0.033029
1186.000000	0.033249
1185.750000	0.033472
1185.500000	0.033698

1185.250000	0.033927
1185.000000	0.034159
1184.750000	0.034390
1184.500000	0.034620
1184.250000	0.034847
1184.000000	0.035073
1183.750000	0.035296
1183.500000	0.035517
1183.250000	0.035736
1183.000000	0.035951
1182.750000	0.036161
1182.500000	0.036365
1182.250000	0.036563
1182.000000	0.036755
1181.750000	0.036942
1181.500000	0.037125
1181.250000	0.037304
1181.000000	0.037478
1180.750000	0.037646
1180.500000	0.037808
1180.250000	0.037965
1180.000000	0.038120
1179.750000	0.038275
1179.500000	0.038433
1179.250000	0.038596
1179.000000	0.038763

1178.750000	0.038935
1178.500000	0.039111
1178.250000	0.039290
1178.000000	0.039472
1177.750000	0.039657
1177.500000	0.039843
1177.250000	0.040030
1177.000000	0.040217
1176.750000	0.040403
1176.500000	0.040588
1176.250000	0.040775
1176.000000	0.040964
1175.750000	0.041161
1175.500000	0.041365
1175.250000	0.041579
1175.000000	0.041801
1174.750000	0.042031
1174.500000	0.042268
1174.250000	0.042511
1174.000000	0.042761
1173.750000	0.043018
1173.500000	0.043281
1173.250000	0.043549
1173.000000	0.043819
1172.750000	0.044090
1172.500000	0.044359

1172.250000	0.044628
1172.000000	0.044897
1171.750000	0.045168
1171.500000	0.045442
1171.250000	0.045721
1171.000000	0.046002
1170.750000	0.046286
1170.500000	0.046570
1170.250000	0.046855
1170.000000	0.047139
1169.750000	0.047424
1169.500000	0.047708
1169.250000	0.047990
1169.000000	0.048266
1168.750000	0.048534
1168.500000	0.048792
1168.250000	0.049037
1168.000000	0.049270
1167.750000	0.049491
1167.500000	0.049702
1167.250000	0.049903
1167.000000	0.050094
1166.750000	0.050273
1166.500000	0.050442
1166.250000	0.050600
1166.000000	0.050749

1165.750000	0.050887
1165.500000	0.051016
1165.250000	0.051132
1165.000000	0.051234
1164.750000	0.051318
1164.500000	0.051385
1164.250000	0.051436
1164.000000	0.051472
1163.750000	0.051498
1163.500000	0.051516
1163.250000	0.051526
1163.000000	0.051529
1162.750000	0.051525
1162.500000	0.051514
1162.250000	0.051499
1162.000000	0.051483
1161.750000	0.051470
1161.500000	0.051462
1161.250000	0.051460
1161.000000	0.051464
1160.750000	0.051472
1160.500000	0.051484
1160.250000	0.051500
1160.000000	0.051521
1159.750000	0.051549
1159.500000	0.051582

1159.250000	0.051619
1159.000000	0.051656
1158.750000	0.051690
1158.500000	0.051721
1158.250000	0.051749
1158.000000	0.051778
1157.750000	0.051816
1157.500000	0.051865
1157.250000	0.051928
1157.000000	0.052005
1156.750000	0.052093
1156.500000	0.052188
1156.250000	0.052287
1156.000000	0.052388
1155.750000	0.052491
1155.500000	0.052596
1155.250000	0.052705
1155.000000	0.052816
1154.750000	0.052929
1154.500000	0.053044
1154.250000	0.053161
1154.000000	0.053278
1153.750000	0.053396
1153.500000	0.053515
1153.250000	0.053634
1153.000000	0.053749

1152.750000	0.053861
1152.500000	0.053967
1152.250000	0.054067
1152.000000	0.054164
1151.750000	0.054258
1151.500000	0.054353
1151.250000	0.054449
1151.000000	0.054545
1150.750000	0.054639
1150.500000	0.054732
1150.250000	0.054820
1150.000000	0.054903
1149.750000	0.054979
1149.500000	0.055049
1149.250000	0.055110
1149.000000	0.055162
1148.750000	0.055205
1148.500000	0.055241
1148.250000	0.055273
1148.000000	0.055303
1147.750000	0.055336
1147.500000	0.055372
1147.250000	0.055410
1147.000000	0.055448
1146.750000	0.055482
1146.500000	0.055513

1146.250000	0.055538
1146.000000	0.055560
1145.750000	0.055580
1145.500000	0.055595
1145.250000	0.055605
1145.000000	0.055607
1144.750000	0.055598
1144.500000	0.055578
1144.250000	0.055549
1144.000000	0.055516
1143.750000	0.055485
1143.500000	0.055461
1143.250000	0.055443
1143.000000	0.055433
1142.750000	0.055426
1142.500000	0.055421
1142.250000	0.055415
1142.000000	0.055409
1141.750000	0.055404
1141.500000	0.055401
1141.250000	0.055399
1141.000000	0.055397
1140.750000	0.055392
1140.500000	0.055381
1140.250000	0.055365
1140.000000	0.055343

1139.750000	0.055317
1139.500000	0.055289
1139.250000	0.055259
1139.000000	0.055228
1138.750000	0.055196
1138.500000	0.055161
1138.250000	0.055127
1138.000000	0.055094
1137.750000	0.055065
1137.500000	0.055040
1137.250000	0.055022
1137.000000	0.055006
1136.750000	0.054992
1136.500000	0.054978
1136.250000	0.054961
1136.000000	0.054942
1135.750000	0.054921
1135.500000	0.054898
1135.250000	0.054874
1135.000000	0.054848
1134.750000	0.054821
1134.500000	0.054794
1134.250000	0.054769
1134.000000	0.054749
1133.750000	0.054736
1133.500000	0.054730

1133.250000	0.054729
1133.000000	0.054728
1132.750000	0.054724
1132.500000	0.054716
1132.250000	0.054702
1132.000000	0.054687
1131.750000	0.054673
1131.500000	0.054662
1131.250000	0.054655
1131.000000	0.054650
1130.750000	0.054643
1130.500000	0.054632
1130.250000	0.054617
1130.000000	0.054599
1129.750000	0.054582
1129.500000	0.054571
1129.250000	0.054567
1129.000000	0.054571
1128.750000	0.054582
1128.500000	0.054597
1128.250000	0.054615
1128.000000	0.054635
1127.750000	0.054656
1127.500000	0.054678
1127.250000	0.054701
1127.000000	0.054724

1126.750000	0.054746
1126.500000	0.054768
1126.250000	0.054790
1126.000000	0.054815
1125.750000	0.054845
1125.500000	0.054881
1125.250000	0.054922
1125.000000	0.054968
1124.750000	0.055017
1124.500000	0.055068
1124.250000	0.055123
1124.000000	0.055185
1123.750000	0.055260
1123.500000	0.055349
1123.250000	0.055452
1123.000000	0.055564
1122.750000	0.055678
1122.500000	0.055785
1122.250000	0.055878
1122.000000	0.055952
1121.750000	0.056007
1121.500000	0.056046
1121.250000	0.056075
1121.000000	0.056100
1120.750000	0.056126
1120.500000	0.056156

1120.250000	0.056191
1120.000000	0.056231
1119.750000	0.056277
1119.500000	0.056326
1119.250000	0.056379
1119.000000	0.056433
1118.750000	0.056488
1118.500000	0.056545
1118.250000	0.056603
1118.000000	0.056663
1117.750000	0.056725
1117.500000	0.056788
1117.250000	0.056852
1117.000000	0.056915
1116.750000	0.056975
1116.500000	0.057033
1116.250000	0.057087
1116.000000	0.057138
1115.750000	0.057186
1115.500000	0.057230
1115.250000	0.057272
1115.000000	0.057310
1114.750000	0.057348
1114.500000	0.057385
1114.250000	0.057423
1114.000000	0.057465

1113.750000	0.057510
1113.500000	0.057556
1113.250000	0.057601
1113.000000	0.057640
1112.750000	0.057672
1112.500000	0.057694
1112.250000	0.057709
1112.000000	0.057719
1111.750000	0.057727
1111.500000	0.057735
1111.250000	0.057745
1111.000000	0.057755
1110.750000	0.057765
1110.500000	0.057775
1110.250000	0.057784
1110.000000	0.057796
1109.750000	0.057809
1109.500000	0.057825
1109.250000	0.057841
1109.000000	0.057856
1108.750000	0.057867
1108.500000	0.057874
1108.250000	0.057875
1108.000000	0.057872
1107.750000	0.057866
1107.500000	0.057859

1107.250000	0.057851
1107.000000	0.057842
1106.750000	0.057833
1106.500000	0.057824
1106.250000	0.057815
1106.000000	0.057809
1105.750000	0.057804
1105.500000	0.057801
1105.250000	0.057795
1105.000000	0.057784
1104.750000	0.057767
1104.500000	0.057743
1104.250000	0.057715
1104.000000	0.057687
1103.750000	0.057661
1103.500000	0.057639
1103.250000	0.057621
1103.000000	0.057603
1102.750000	0.057582
1102.500000	0.057557
1102.250000	0.057528
1102.000000	0.057497
1101.750000	0.057467
1101.500000	0.057443
1101.250000	0.057425
1101.000000	0.057412

1100.750000	0.057404
1100.500000	0.057399
1100.250000	0.057394
1100.000000	0.057391
1099.750000	0.057388
1099.500000	0.057385
1099.250000	0.057382
1099.000000	0.057377
1098.750000	0.057370
1098.500000	0.057359
1098.250000	0.057347
1098.000000	0.057335
1097.750000	0.057325
1097.500000	0.057318
1097.250000	0.057315
1097.000000	0.057313
1096.750000	0.057313
1096.500000	0.057313
1096.250000	0.057314
1096.000000	0.057316
1095.750000	0.057321
1095.500000	0.057327
1095.250000	0.057333
1095.000000	0.057336
1094.750000	0.057337
1094.500000	0.057335

1094.250000	0.057332
1094.000000	0.057331
1093.750000	0.057336
1093.500000	0.057347
1093.250000	0.057363
1093.000000	0.057380
1092.750000	0.057397
1092.500000	0.057412
1092.250000	0.057426
1092.000000	0.057443
1091.750000	0.057464
1091.500000	0.057494
1091.250000	0.057532
1091.000000	0.057575
1090.750000	0.057621
1090.500000	0.057668
1090.250000	0.057713
1090.000000	0.057757
1089.750000	0.057802
1089.500000	0.057848
1089.250000	0.057895
1089.000000	0.057941
1088.750000	0.057985
1088.500000	0.058027
1088.250000	0.058069
1088.000000	0.058113

1087.750000	0.058164
1087.500000	0.058222
1087.250000	0.058288
1087.000000	0.058357
1086.750000	0.058427
1086.500000	0.058493
1086.250000	0.058553
1086.000000	0.058610
1085.750000	0.058665
1085.500000	0.058720
1085.250000	0.058776
1085.000000	0.058832
1084.750000	0.058886
1084.500000	0.058938
1084.250000	0.058987
1084.000000	0.059034
1083.750000	0.059081
1083.500000	0.059133
1083.250000	0.059189
1083.000000	0.059249
1082.750000	0.059312
1082.500000	0.059376
1082.250000	0.059440
1082.000000	0.059504
1081.750000	0.059567
1081.500000	0.059630

1081.250000	0.059690
1081.000000	0.059747
1080.750000	0.059800
1080.500000	0.059850
1080.250000	0.059899
1080.000000	0.059949
1079.750000	0.060002
1079.500000	0.060060
1079.250000	0.060119
1079.000000	0.060178
1078.750000	0.060233
1078.500000	0.060282
1078.250000	0.060325
1078.000000	0.060366
1077.750000	0.060407
1077.500000	0.060451
1077.250000	0.060499
1077.000000	0.060549
1076.750000	0.060598
1076.500000	0.060645
1076.250000	0.060688
1076.000000	0.060731
1075.750000	0.060773
1075.500000	0.060816
1075.250000	0.060860
1075.000000	0.060902

1074.750000	0.060939
1074.500000	0.060969
1074.250000	0.060992
1074.000000	0.061012
1073.750000	0.061031
1073.500000	0.061053
1073.250000	0.061079
1073.000000	0.061109
1072.750000	0.061141
1072.500000	0.061171
1072.250000	0.061201
1072.000000	0.061229
1071.750000	0.061260
1071.500000	0.061293
1071.250000	0.061331
1071.000000	0.061372
1070.750000	0.061414
1070.500000	0.061456
1070.250000	0.061495
1070.000000	0.061532
1069.750000	0.061567
1069.500000	0.061601
1069.250000	0.061634
1069.000000	0.061666
1068.750000	0.061695
1068.500000	0.061724

1068.250000	0.061751
1068.000000	0.061778
1067.750000	0.061807
1067.500000	0.061837
1067.250000	0.061867
1067.000000	0.061893
1066.750000	0.061911
1066.500000	0.061922
1066.250000	0.061924
1066.000000	0.061922
1065.750000	0.061919
1065.500000	0.061918
1065.250000	0.061923
1065.000000	0.061933
1064.750000	0.061946
1064.500000	0.061962
1064.250000	0.061980
1064.000000	0.062000
1063.750000	0.062024
1063.500000	0.062050
1063.250000	0.062075
1063.000000	0.062096
1062.750000	0.062110
1062.500000	0.062113
1062.250000	0.062105
1062.000000	0.062090

1061.750000	0.062070
1061.500000	0.062052
1061.250000	0.062037
1061.000000	0.062027
1060.750000	0.062021
1060.500000	0.062019
1060.250000	0.062018
1060.000000	0.062017
1059.750000	0.062016
1059.500000	0.062015
1059.250000	0.062012
1059.000000	0.062008
1058.750000	0.062001
1058.500000	0.061992
1058.250000	0.061983
1058.000000	0.061975
1057.750000	0.061970
1057.500000	0.061968
1057.250000	0.061967
1057.000000	0.061964
1056.750000	0.061958
1056.500000	0.061945
1056.250000	0.061929
1056.000000	0.061910
1055.750000	0.061891
1055.500000	0.061875

1055.250000	0.061860
1055.000000	0.061843
1054.750000	0.061822
1054.500000	0.061794
1054.250000	0.061760
1054.000000	0.061724
1053.750000	0.061691
1053.500000	0.061665
1053.250000	0.061648
1053.000000	0.061639
1052.750000	0.061634
1052.500000	0.061630
1052.250000	0.061623
1052.000000	0.061611
1051.750000	0.061596
1051.500000	0.061578
1051.250000	0.061561
1051.000000	0.061544
1050.750000	0.061529
1050.500000	0.061516
1050.250000	0.061505
1050.000000	0.061498
1049.750000	0.061495
1049.500000	0.061495
1049.250000	0.061496
1049.000000	0.061497

1048.750000	0.061494
1048.500000	0.061485
1048.250000	0.061471
1048.000000	0.061455
1047.750000	0.061438
1047.500000	0.061423
1047.250000	0.061413
1047.000000	0.061408
1046.750000	0.061406
1046.500000	0.061408
1046.250000	0.061414
1046.000000	0.061423
1045.750000	0.061436
1045.500000	0.061451
1045.250000	0.061465
1045.000000	0.061477
1044.750000	0.061483
1044.500000	0.061482
1044.250000	0.061475
1044.000000	0.061466
1043.750000	0.061457
1043.500000	0.061451
1043.250000	0.061448
1043.000000	0.061449
1042.750000	0.061451
1042.500000	0.061453

1042.250000	0.061456
1042.000000	0.061461
1041.750000	0.061469
1041.500000	0.061481
1041.250000	0.061495
1041.000000	0.061508
1040.750000	0.061519
1040.500000	0.061524
1040.250000	0.061525
1040.000000	0.061523
1039.750000	0.061523
1039.500000	0.061528
1039.250000	0.061538
1039.000000	0.061554
1038.750000	0.061573
1038.500000	0.061590
1038.250000	0.061605
1038.000000	0.061614
1037.750000	0.061619
1037.500000	0.061619
1037.250000	0.061615
1037.000000	0.061608
1036.750000	0.061598
1036.500000	0.061587
1036.250000	0.061576
1036.000000	0.061567

1035.750000	0.061564
1035.500000	0.061566
1035.250000	0.061571
1035.000000	0.061578
1034.750000	0.061583
1034.500000	0.061583
1034.250000	0.061578
1034.000000	0.061569
1033.750000	0.061559
1033.500000	0.061549
1033.250000	0.061538
1033.000000	0.061527
1032.750000	0.061512
1032.500000	0.061492
1032.250000	0.061466
1032.000000	0.061438
1031.750000	0.061408
1031.500000	0.061379
1031.250000	0.061353
1031.000000	0.061330
1030.750000	0.061309
1030.500000	0.061290
1030.250000	0.061272
1030.000000	0.061258
1029.750000	0.061246
1029.500000	0.061238

1029.250000	0.061229
1029.000000	0.061217
1028.750000	0.061198
1028.500000	0.061170
1028.250000	0.061134
1028.000000	0.061093
1027.750000	0.061052
1027.500000	0.061014
1027.250000	0.060981
1027.000000	0.060953
1026.750000	0.060927
1026.500000	0.060898
1026.250000	0.060864
1026.000000	0.060822
1025.750000	0.060772
1025.500000	0.060716
1025.250000	0.060656
1025.000000	0.060593
1024.750000	0.060532
1024.500000	0.060473
1024.250000	0.060422
1024.000000	0.060380
1023.750000	0.060349
1023.500000	0.060328
1023.250000	0.060315
1023.000000	0.060303

1022.750000	0.060289
1022.500000	0.060268
1022.250000	0.060240
1022.000000	0.060205
1021.750000	0.060166
1021.500000	0.060125
1021.250000	0.060083
1021.000000	0.060041
1020.750000	0.059999
1020.500000	0.059955
1020.250000	0.059911
1020.000000	0.059868
1019.750000	0.059830
1019.500000	0.059798
1019.250000	0.059773
1019.000000	0.059753
1018.750000	0.059737
1018.500000	0.059722
1018.250000	0.059704
1018.000000	0.059684
1017.750000	0.059661
1017.500000	0.059637
1017.250000	0.059613
1017.000000	0.059589
1016.750000	0.059567
1016.500000	0.059548

1016.250000	0.059535
1016.000000	0.059530
1015.750000	0.059535
1015.500000	0.059547
1015.250000	0.059566
1015.000000	0.059586
1014.750000	0.059603
1014.500000	0.059613
1014.250000	0.059614
1014.000000	0.059606
1013.750000	0.059593
1013.500000	0.059577
1013.250000	0.059563
1013.000000	0.059553
1012.750000	0.059548
1012.500000	0.059551
1012.250000	0.059560
1012.000000	0.059576
1011.750000	0.059595
1011.500000	0.059616
1011.250000	0.059631
1011.000000	0.059636
1010.750000	0.059628
1010.500000	0.059604
1010.250000	0.059566
1010.000000	0.059519

1009.750000	0.059468
1009.500000	0.059416
1009.250000	0.059364
1009.000000	0.059312
1008.750000	0.059257
1008.500000	0.059194
1008.250000	0.059123
1008.000000	0.059042
1007.750000	0.058952
1007.500000	0.058857
1007.250000	0.058756
1007.000000	0.058653
1006.750000	0.058550
1006.500000	0.058447
1006.250000	0.058349
1006.000000	0.058258
1005.750000	0.058174
1005.500000	0.058098
1005.250000	0.058026
1005.000000	0.057955
1004.750000	0.057879
1004.500000	0.057797
1004.250000	0.057709
1004.000000	0.057617
1003.750000	0.057524
1003.500000	0.057434

1003.250000	0.057347
1003.000000	0.057263
1002.750000	0.057178
1002.500000	0.057090
1002.250000	0.056998
1002.000000	0.056903
1001.750000	0.056809
1001.500000	0.056717
1001.250000	0.056629
1001.000000	0.056545
1000.750000	0.056463
1000.500000	0.056381
1000.250000	0.056297
1000.000000	0.056213
999.750000	0.056130
999.500000	0.056051
999.250000	0.055975
999.000000	0.055901
998.750000	0.055827
998.500000	0.055749
998.250000	0.055665
998.000000	0.055574
997.750000	0.055479
997.500000	0.055382
997.250000	0.055288
997.000000	0.055200

996.750000	0.055121
996.500000	0.055051
996.250000	0.054988
996.000000	0.054933
995.750000	0.054882
995.500000	0.054835
995.250000	0.054790
995.000000	0.054744
994.750000	0.054696
994.500000	0.054645
994.250000	0.054591
994.000000	0.054534
993.750000	0.054474
993.500000	0.054412
993.250000	0.054350
993.000000	0.054286
992.750000	0.054221
992.500000	0.054156
992.250000	0.054094
992.000000	0.054035
991.750000	0.053983
991.500000	0.053939
991.250000	0.053904
991.000000	0.053878
990.750000	0.053861
990.500000	0.053852

990.250000	0.053851
990.000000	0.053856
989.750000	0.053867
989.500000	0.053882
989.250000	0.053897
989.000000	0.053910
988.750000	0.053917
988.500000	0.053916
988.250000	0.053909
988.000000	0.053897
987.750000	0.053884
987.500000	0.053870
987.250000	0.053859
987.000000	0.053850
986.750000	0.053844
986.500000	0.053840
986.250000	0.053840
986.000000	0.053847
985.750000	0.053859
985.500000	0.053876
985.250000	0.053893
985.000000	0.053903
984.750000	0.053898
984.500000	0.053871
984.250000	0.053818
984.000000	0.053741

983.750000	0.053645
983.500000	0.053538
983.250000	0.053428
983.000000	0.053324
982.750000	0.053229
982.500000	0.053146
982.250000	0.053073
982.000000	0.053009
981.750000	0.052950
981.500000	0.052890
981.250000	0.052826
981.000000	0.052755
980.750000	0.052676
980.500000	0.052590
980.250000	0.052498
980.000000	0.052404
979.750000	0.052310
979.500000	0.052219
979.250000	0.052128
979.000000	0.052037
978.750000	0.051942
978.500000	0.051844
978.250000	0.051742
978.000000	0.051640
977.750000	0.051542
977.500000	0.051451

977.250000	0.051368
977.000000	0.051290
976.750000	0.051214
976.500000	0.051133
976.250000	0.051042
976.000000	0.050941
975.750000	0.050829
975.500000	0.050710
975.250000	0.050588
975.000000	0.050470
974.750000	0.050360
974.500000	0.050262
974.250000	0.050176
974.000000	0.050103
973.750000	0.050040
973.500000	0.049983
973.250000	0.049928
973.000000	0.049869
972.750000	0.049802
972.500000	0.049725
972.250000	0.049639
972.000000	0.049547
971.750000	0.049453
971.500000	0.049364
971.250000	0.049283
971.000000	0.049213

970.750000	0.049154
970.500000	0.049104
970.250000	0.049061
970.000000	0.049021
969.750000	0.048982
969.500000	0.048941
969.250000	0.048897
969.000000	0.048850
968.750000	0.048800
968.500000	0.048750
968.250000	0.048700
968.000000	0.048655
967.750000	0.048616
967.500000	0.048584
967.250000	0.048557
967.000000	0.048532
966.750000	0.048506
966.500000	0.048476
966.250000	0.048438
966.000000	0.048393
965.750000	0.048340
965.500000	0.048282
965.250000	0.048220
965.000000	0.048157
964.750000	0.048096
964.500000	0.048037

964.250000	0.047985
964.000000	0.047939
963.750000	0.047899
963.500000	0.047864
963.250000	0.047832
963.000000	0.047798
962.750000	0.047761
962.500000	0.047718
962.250000	0.047670
962.000000	0.047620
961.750000	0.047567
961.500000	0.047514
961.250000	0.047460
961.000000	0.047404
960.750000	0.047344
960.500000	0.047278
960.250000	0.047209
960.000000	0.047139
959.750000	0.047071
959.500000	0.047008
959.250000	0.046951
959.000000	0.046902
958.750000	0.046856
958.500000	0.046812
958.250000	0.046766
958.000000	0.046716

957.750000	0.046662
957.500000	0.046604
957.250000	0.046542
957.000000	0.046475
956.750000	0.046405
956.500000	0.046333
956.250000	0.046260
956.000000	0.046189
955.750000	0.046121
955.500000	0.046059
955.250000	0.046002
955.000000	0.045951
954.750000	0.045904
954.500000	0.045858
954.250000	0.045812
954.000000	0.045765
953.750000	0.045718
953.500000	0.045670
953.250000	0.045621
953.000000	0.045571
952.750000	0.045520
952.500000	0.045468
952.250000	0.045414
952.000000	0.045359
951.750000	0.045305
951.500000	0.045252

951.250000	0.045200
951.000000	0.045150
950.750000	0.045102
950.500000	0.045056
950.250000	0.045012
950.000000	0.044971
949.750000	0.044934
949.500000	0.044899
949.250000	0.044868
949.000000	0.044836
948.750000	0.044805
948.500000	0.044771
948.250000	0.044736
948.000000	0.044700
947.750000	0.044664
947.500000	0.044629
947.250000	0.044594
947.000000	0.044558
946.750000	0.044521
946.500000	0.044482
946.250000	0.044442
946.000000	0.044403
945.750000	0.044366
945.500000	0.044332
945.250000	0.044301
945.000000	0.044272

944.750000	0.044243
944.500000	0.044212
944.250000	0.044178
944.000000	0.044142
943.750000	0.044103
943.500000	0.044063
943.250000	0.044021
943.000000	0.043977
942.750000	0.043928
942.500000	0.043872
942.250000	0.043810
942.000000	0.043743
941.750000	0.043673
941.500000	0.043603
941.250000	0.043536
941.000000	0.043474
940.750000	0.043415
940.500000	0.043360
940.250000	0.043307
940.000000	0.043254
939.750000	0.043198
939.500000	0.043137
939.250000	0.043070
939.000000	0.042995
938.750000	0.042912
938.500000	0.042824

938.250000	0.042732
938.000000	0.042642
937.750000	0.042557
937.500000	0.042477
937.250000	0.042403
937.000000	0.042332
936.750000	0.042259
936.500000	0.042179
936.250000	0.042091
936.000000	0.041992
935.750000	0.041882
935.500000	0.041765
935.250000	0.041644
935.000000	0.041523
934.750000	0.041404
934.500000	0.041290
934.250000	0.041183
934.000000	0.041085
933.750000	0.040995
933.500000	0.040909
933.250000	0.040826
933.000000	0.040742
932.750000	0.040653
932.500000	0.040558
932.250000	0.040458
932.000000	0.040355

931.750000	0.040254
931.500000	0.040157
931.250000	0.040066
931.000000	0.039982
930.750000	0.039902
930.500000	0.039824
930.250000	0.039745
930.000000	0.039664
929.750000	0.039579
929.500000	0.039491
929.250000	0.039400
929.000000	0.039307
928.750000	0.039214
928.500000	0.039121
928.250000	0.039032
928.000000	0.038946
927.750000	0.038866
927.500000	0.038790
927.250000	0.038718
927.000000	0.038647
926.750000	0.038577
926.500000	0.038506
926.250000	0.038434
926.000000	0.038362
925.750000	0.038290
925.500000	0.038220

925.250000	0.038150
925.000000	0.038079
924.750000	0.038005
924.500000	0.037928
924.250000	0.037850
924.000000	0.037771
923.750000	0.037696
923.500000	0.037625
923.250000	0.037559
923.000000	0.037496
922.750000	0.037432
922.500000	0.037364
922.250000	0.037289
922.000000	0.037204
921.750000	0.037111
921.500000	0.037011
921.250000	0.036909
921.000000	0.036806
920.750000	0.036703
920.500000	0.036603
920.250000	0.036504
920.000000	0.036408
919.750000	0.036312
919.500000	0.036216
919.250000	0.036119
919.000000	0.036021

918.750000	0.035920
918.500000	0.035818
918.250000	0.035713
918.000000	0.035605
917.750000	0.035494
917.500000	0.035380
917.250000	0.035262
917.000000	0.035139
916.750000	0.035011
916.500000	0.034880
916.250000	0.034747
916.000000	0.034613
915.750000	0.034478
915.500000	0.034343
915.250000	0.034206
915.000000	0.034066
914.750000	0.033920
914.500000	0.033770
914.250000	0.033616
914.000000	0.033463
913.750000	0.033312
913.500000	0.033167
913.250000	0.033027
913.000000	0.032890
912.750000	0.032755
912.500000	0.032618

912.250000	0.032477
912.000000	0.032332
911.750000	0.032183
911.500000	0.032031
911.250000	0.031877
911.000000	0.031723
910.750000	0.031569
910.500000	0.031416
910.250000	0.031263
910.000000	0.031112
909.750000	0.030963
909.500000	0.030814
909.250000	0.030667
909.000000	0.030519
908.750000	0.030369
908.500000	0.030217
908.250000	0.030063
908.000000	0.029909
907.750000	0.029754
907.500000	0.029602
907.250000	0.029452
907.000000	0.029308
906.750000	0.029168
906.500000	0.029032
906.250000	0.028901
906.000000	0.028772

905.750000	0.028643
905.500000	0.028513
905.250000	0.028379
905.000000	0.028240
904.750000	0.028095
904.500000	0.027945
904.250000	0.027793
904.000000	0.027642
903.750000	0.027496
903.500000	0.027355
903.250000	0.027221
903.000000	0.027093
902.750000	0.026970
902.500000	0.026849
902.250000	0.026730
902.000000	0.026610
901.750000	0.026488
901.500000	0.026363
901.250000	0.026236
901.000000	0.026104
900.750000	0.025970
900.500000	0.025834
900.250000	0.025701
900.000000	0.025574
899.750000	0.025454
899.500000	0.025343

899.250000	0.025239
899.000000	0.025138
898.750000	0.025039
898.500000	0.024936
898.250000	0.024830
898.000000	0.024720
897.750000	0.024607
897.500000	0.024494
897.250000	0.024380
897.000000	0.024267
896.750000	0.024155
896.500000	0.024045
896.250000	0.023936
896.000000	0.023830
895.750000	0.023727
895.500000	0.023629
895.250000	0.023534
895.000000	0.023440
894.750000	0.023346
894.500000	0.023250
894.250000	0.023149
894.000000	0.023044
893.750000	0.022936
893.500000	0.022826
893.250000	0.022717
893.000000	0.022612

892.750000	0.022511
892.500000	0.022415
892.250000	0.022324
892.000000	0.022236
891.750000	0.022150
891.500000	0.022064
891.250000	0.021977
891.000000	0.021887
890.750000	0.021795
890.500000	0.021702
890.250000	0.021610
890.000000	0.021519
889.750000	0.021431
889.500000	0.021345
889.250000	0.021261
889.000000	0.021178
888.750000	0.021096
888.500000	0.021015
888.250000	0.020936
888.000000	0.020860
887.750000	0.020787
887.500000	0.020716
887.250000	0.020643
887.000000	0.020568
886.750000	0.020488
886.500000	0.020405

886.250000	0.020320
886.000000	0.020238
885.750000	0.020162
885.500000	0.020092
885.250000	0.020029
885.000000	0.019967
884.750000	0.019903
884.500000	0.019832
884.250000	0.019754
884.000000	0.019668
883.750000	0.019578
883.500000	0.019487
883.250000	0.019398
883.000000	0.019313
882.750000	0.019233
882.500000	0.019157
882.250000	0.019084
882.000000	0.019013
881.750000	0.018943
881.500000	0.018875
881.250000	0.018807
881.000000	0.018738
880.750000	0.018667
880.500000	0.018594
880.250000	0.018521
880.000000	0.018448

879.750000	0.018378
879.500000	0.018311
879.250000	0.018249
879.000000	0.018188
878.750000	0.018127
878.500000	0.018062
878.250000	0.017992
878.000000	0.017914
877.750000	0.017831
877.500000	0.017744
877.250000	0.017654
877.000000	0.017564
876.750000	0.017475
876.500000	0.017388
876.250000	0.017304
876.000000	0.017224
875.750000	0.017146
875.500000	0.017071
875.250000	0.016996
875.000000	0.016918
874.750000	0.016836
874.500000	0.016749
874.250000	0.016659
874.000000	0.016568
873.750000	0.016481
873.500000	0.016404

873.250000	0.016338
873.000000	0.016283
872.750000	0.016238
872.500000	0.016198
872.250000	0.016158
872.000000	0.016115
871.750000	0.016065
871.500000	0.016008
871.250000	0.015946
871.000000	0.015880
870.750000	0.015813
870.500000	0.015746
870.250000	0.015682
870.000000	0.015621
869.750000	0.015562
869.500000	0.015507
869.250000	0.015454
869.000000	0.015403
868.750000	0.015352
868.500000	0.015300
868.250000	0.015247
868.000000	0.015193
867.750000	0.015139
867.500000	0.015086
867.250000	0.015035
867.000000	0.014985

866.750000	0.014937
866.500000	0.014891
866.250000	0.014845
866.000000	0.014800
865.750000	0.014755
865.500000	0.014710
865.250000	0.014665
865.000000	0.014619
864.750000	0.014570
864.500000	0.014520
864.250000	0.014467
864.000000	0.014414
863.750000	0.014364
863.500000	0.014318
863.250000	0.014278
863.000000	0.014245
862.750000	0.014219
862.500000	0.014198
862.250000	0.014178
862.000000	0.014158
861.750000	0.014134
861.500000	0.014105
861.250000	0.014068
861.000000	0.014024
860.750000	0.013973
860.500000	0.013917

860.250000	0.013857
860.000000	0.013798
859.750000	0.013740
859.500000	0.013686
859.250000	0.013637
859.000000	0.013594
858.750000	0.013557
858.500000	0.013525
858.250000	0.013498
858.000000	0.013474
857.750000	0.013452
857.500000	0.013430
857.250000	0.013408
857.000000	0.013382
856.750000	0.013354
856.500000	0.013323
856.250000	0.013290
856.000000	0.013257
855.750000	0.013227
855.500000	0.013201
855.250000	0.013179
855.000000	0.013161
854.750000	0.013144
854.500000	0.013126
854.250000	0.013106
854.000000	0.013081

853.750000	0.013052
853.500000	0.013020
853.250000	0.012987
853.000000	0.012955
852.750000	0.012926
852.500000	0.012901
852.250000	0.012882
852.000000	0.012869
851.750000	0.012860
851.500000	0.012854
851.250000	0.012849
851.000000	0.012844
850.750000	0.012838
850.500000	0.012832
850.250000	0.012826
850.000000	0.012822
849.750000	0.012822
849.500000	0.012827
849.250000	0.012835
849.000000	0.012845
848.750000	0.012856
848.500000	0.012866
848.250000	0.012877
848.000000	0.012889
847.750000	0.012904
847.500000	0.012924

847.250000	0.012951
847.000000	0.012983
846.750000	0.013021
846.500000	0.013063
846.250000	0.013109
846.000000	0.013159
845.750000	0.013213
845.500000	0.013273
845.250000	0.013337
845.000000	0.013405
844.750000	0.013476
844.500000	0.013547
844.250000	0.013616
844.000000	0.013684
843.750000	0.013750
843.500000	0.013814
843.250000	0.013879
843.000000	0.013944
842.750000	0.014009
842.500000	0.014073
842.250000	0.014138
842.000000	0.014201
841.750000	0.014263
841.500000	0.014325
841.250000	0.014384
841.000000	0.014440

840.750000	0.014492
840.500000	0.014539
840.250000	0.014579
840.000000	0.014615
839.750000	0.014647
839.500000	0.014676
839.250000	0.014705
839.000000	0.014734
838.750000	0.014762
838.500000	0.014789
838.250000	0.014815
838.000000	0.014839
837.750000	0.014864
837.500000	0.014892
837.250000	0.014922
837.000000	0.014955
836.750000	0.014990
836.500000	0.015025
836.250000	0.015057
836.000000	0.015083
835.750000	0.015104
835.500000	0.015119
835.250000	0.015130
835.000000	0.015139
834.750000	0.015145
834.500000	0.015152

834.250000	0.015158
834.000000	0.015163
833.750000	0.015168
833.500000	0.015172
833.250000	0.015172
833.000000	0.015168
832.750000	0.015159
832.500000	0.015142
832.250000	0.015118
832.000000	0.015088
831.750000	0.015054
831.500000	0.015018
831.250000	0.014983
831.000000	0.014949
830.750000	0.014916
830.500000	0.014882
830.250000	0.014845
830.000000	0.014803
829.750000	0.014753
829.500000	0.014695
829.250000	0.014628
829.000000	0.014553
828.750000	0.014470
828.500000	0.014379
828.250000	0.014281
828.000000	0.014177

827.750000	0.014069
827.500000	0.013957
827.250000	0.013843
827.000000	0.013728
826.750000	0.013613
826.500000	0.013498
826.250000	0.013385
826.000000	0.013274
825.750000	0.013167
825.500000	0.013066
825.250000	0.012971
825.000000	0.012884
824.750000	0.012804
824.500000	0.012730
824.250000	0.012662
824.000000	0.012600
823.750000	0.012544
823.500000	0.012496
823.250000	0.012456
823.000000	0.012426
822.750000	0.012405
822.500000	0.012393
822.250000	0.012389
822.000000	0.012393
821.750000	0.012405
821.500000	0.012427

821.250000	0.012457
821.000000	0.012495
820.750000	0.012541
820.500000	0.012592
820.250000	0.012648
820.000000	0.012706
819.750000	0.012767
819.500000	0.012831
819.250000	0.012901
819.000000	0.012977
818.750000	0.013061
818.500000	0.013150
818.250000	0.013245
818.000000	0.013342
817.750000	0.013440
817.500000	0.013537
817.250000	0.013634
817.000000	0.013730
816.750000	0.013826
816.500000	0.013922
816.250000	0.014016
816.000000	0.014109
815.750000	0.014198
815.500000	0.014282
815.250000	0.014359
815.000000	0.014427

814.750000	0.014484
814.500000	0.014530
814.250000	0.014564
814.000000	0.014586
813.750000	0.014597
813.500000	0.014598
813.250000	0.014589
813.000000	0.014572
812.750000	0.014546
812.500000	0.014513
812.250000	0.014471
812.000000	0.014420
811.750000	0.014362
811.500000	0.014295
811.250000	0.014221
811.000000	0.014142
810.750000	0.014059
810.500000	0.013971
810.250000	0.013882
810.000000	0.013790
809.750000	0.013696
809.500000	0.013599
809.250000	0.013500
809.000000	0.013399
808.750000	0.013295
808.500000	0.013191

808.250000	0.013088
808.000000	0.012989
807.750000	0.012896
807.500000	0.012809
807.250000	0.012729
807.000000	0.012652
806.750000	0.012577
806.500000	0.012500
806.250000	0.012419
806.000000	0.012333
805.750000	0.012243
805.500000	0.012154
805.250000	0.012067
805.000000	0.011987
804.750000	0.011914
804.500000	0.011849
804.250000	0.011791
804.000000	0.011738
803.750000	0.011686
803.500000	0.011636
803.250000	0.011587
803.000000	0.011538
802.750000	0.011491
802.500000	0.011445
802.250000	0.011400
802.000000	0.011354

801.750000	0.011307
801.500000	0.011259
801.250000	0.011208
801.000000	0.011156
800.750000	0.011103
800.500000	0.011050
800.250000	0.010997
800.000000	0.010944
799.750000	0.010891
799.500000	0.010837
799.250000	0.010782
799.000000	0.010727
798.750000	0.010672
798.500000	0.010619
798.250000	0.010566
798.000000	0.010514
797.750000	0.010460
797.500000	0.010404
797.250000	0.010344
797.000000	0.010279
796.750000	0.010212
796.500000	0.010143
796.250000	0.010077
796.000000	0.010015
795.750000	0.009960
795.500000	0.009912

795.250000	0.009871
795.000000	0.009834
794.750000	0.009799
794.500000	0.009765
794.250000	0.009731
794.000000	0.009696
793.750000	0.009660
793.500000	0.009623
793.250000	0.009585
793.000000	0.009546
792.750000	0.009507
792.500000	0.009466
792.250000	0.009424
792.000000	0.009383
791.750000	0.009343
791.500000	0.009304
791.250000	0.009267
791.000000	0.009231
790.750000	0.009196
790.500000	0.009162
790.250000	0.009127
790.000000	0.009094
789.750000	0.009063
789.500000	0.009035
789.250000	0.009012
789.000000	0.008996

788.750000	0.008986
788.500000	0.008982
788.250000	0.008982
788.000000	0.008985
787.750000	0.008991
787.500000	0.008998
787.250000	0.009006
787.000000	0.009017
786.750000	0.009029
786.500000	0.009044
786.250000	0.009063
786.000000	0.009088
785.750000	0.009119
785.500000	0.009158
785.250000	0.009206
785.000000	0.009264
784.750000	0.009332
784.500000	0.009410
784.250000	0.009497
784.000000	0.009591
783.750000	0.009690
783.500000	0.009794
783.250000	0.009900
783.000000	0.010008
782.750000	0.010116
782.500000	0.010224

782.250000	0.010333
782.000000	0.010443
781.750000	0.010554
781.500000	0.010669
781.250000	0.010787
781.000000	0.010909
780.750000	0.011035
780.500000	0.011163
780.250000	0.011292
780.000000	0.011421
779.750000	0.011549
779.500000	0.011675
779.250000	0.011798
779.000000	0.011917
778.750000	0.012033
778.500000	0.012145
778.250000	0.012251
778.000000	0.012350
777.750000	0.012441
777.500000	0.012524
777.250000	0.012597
777.000000	0.012662
776.750000	0.012717
776.500000	0.012765
776.250000	0.012804
776.000000	0.012836

775.750000	0.012859
775.500000	0.012873
775.250000	0.012878
775.000000	0.012871
774.750000	0.012853
774.500000	0.012824
774.250000	0.012782
774.000000	0.012729
773.750000	0.012665
773.500000	0.012592
773.250000	0.012509
773.000000	0.012417
772.750000	0.012317
772.500000	0.012209
772.250000	0.012092
772.000000	0.011970
771.750000	0.011842
771.500000	0.011710
771.250000	0.011578
771.000000	0.011445
770.750000	0.011314
770.500000	0.011185
770.250000	0.011059
770.000000	0.010937
769.750000	0.010820
769.500000	0.010707

769.250000	0.010600
769.000000	0.010496
768.750000	0.010397
768.500000	0.010301
768.250000	0.010208
768.000000	0.010117
767.750000	0.010028
767.500000	0.009945
767.250000	0.009870
767.000000	0.009805
766.750000	0.009753
766.500000	0.009717
766.250000	0.009695
766.000000	0.009685
765.750000	0.009687
765.500000	0.009696
765.250000	0.009712
765.000000	0.009732
764.750000	0.009758
764.500000	0.009788
764.250000	0.009823
764.000000	0.009862
763.750000	0.009901
763.500000	0.009939
763.250000	0.009971
763.000000	0.009994

762.750000	0.010006
762.500000	0.010007
762.250000	0.009995
762.000000	0.009972
761.750000	0.009937
761.500000	0.009890
761.250000	0.009834
761.000000	0.009767
760.750000	0.009692
760.500000	0.009609
760.250000	0.009520
760.000000	0.009425
759.750000	0.009325
759.500000	0.009222
759.250000	0.009116
759.000000	0.009009
758.750000	0.008904
758.500000	0.008803
758.250000	0.008707
758.000000	0.008618
757.750000	0.008535
757.500000	0.008454
757.250000	0.008376
757.000000	0.008298
756.750000	0.008221
756.500000	0.008148

756.250000	0.008082
756.000000	0.008027
755.750000	0.007983
755.500000	0.007952
755.250000	0.007930
755.000000	0.007914
754.750000	0.007900
754.500000	0.007886
754.250000	0.007873
754.000000	0.007860
753.750000	0.007852
753.500000	0.007848
753.250000	0.007849
753.000000	0.007855
752.750000	0.007863
752.500000	0.007872
752.250000	0.007881
752.000000	0.007891
751.750000	0.007904
751.500000	0.007923
751.250000	0.007949
751.000000	0.007981
750.750000	0.008018
750.500000	0.008056
750.250000	0.008093
750.000000	0.008125

749.750000	0.008154
749.500000	0.008178
749.250000	0.008200
749.000000	0.008219
748.750000	0.008237
748.500000	0.008251
748.250000	0.008259
748.000000	0.008260
747.750000	0.008252
747.500000	0.008235
747.250000	0.008209
747.000000	0.008177
746.750000	0.008139
746.500000	0.008099
746.250000	0.008054
746.000000	0.008006
745.750000	0.007951
745.500000	0.007889
745.250000	0.007820
745.000000	0.007744
744.750000	0.007662
744.500000	0.007575
744.250000	0.007486
744.000000	0.007396
743.750000	0.007304
743.500000	0.007211

743.250000	0.007116
743.000000	0.007022
742.750000	0.006928
742.500000	0.006836
742.250000	0.006749
742.000000	0.006668
741.750000	0.006594
741.500000	0.006526
741.250000	0.006464
741.000000	0.006407
740.750000	0.006355
740.500000	0.006307
740.250000	0.006264
740.000000	0.006227
739.750000	0.006196
739.500000	0.006173
739.250000	0.006155
739.000000	0.006142
738.750000	0.006131
738.500000	0.006122
738.250000	0.006113
738.000000	0.006103
737.750000	0.006092
737.500000	0.006081
737.250000	0.006071
737.000000	0.006063

736.750000	0.006058
736.500000	0.006057
736.250000	0.006059
736.000000	0.006061
735.750000	0.006062
735.500000	0.006059
735.250000	0.006050
735.000000	0.006036
734.750000	0.006017
734.500000	0.005997
734.250000	0.005978
734.000000	0.005962
733.750000	0.005949
733.500000	0.005939
733.250000	0.005930
733.000000	0.005922
732.750000	0.005914
732.500000	0.005905
732.250000	0.005898
732.000000	0.005893
731.750000	0.005892
731.500000	0.005895
731.250000	0.005900
731.000000	0.005907
730.750000	0.005913
730.500000	0.005916

730.250000	0.005916
730.000000	0.005914
729.750000	0.005910
729.500000	0.005905
729.250000	0.005900
729.000000	0.005893
728.750000	0.005885
728.500000	0.005873
728.250000	0.005857
728.000000	0.005838
727.750000	0.005815
727.500000	0.005790
727.250000	0.005763
727.000000	0.005736
726.750000	0.005710
726.500000	0.005684
726.250000	0.005659
726.000000	0.005635
725.750000	0.005612
725.500000	0.005589
725.250000	0.005567
725.000000	0.005543
724.750000	0.005519
724.500000	0.005493
724.250000	0.005465
724.000000	0.005436

723.750000	0.005405
723.500000	0.005374
723.250000	0.005341
723.000000	0.005309
722.750000	0.005277
722.500000	0.005246
722.250000	0.005217
722.000000	0.005188
721.750000	0.005160
721.500000	0.005132
721.250000	0.005103
721.000000	0.005071
720.750000	0.005037
720.500000	0.005001
720.250000	0.004964
720.000000	0.004929
719.750000	0.004897
719.500000	0.004867
719.250000	0.004840
719.000000	0.004815
718.750000	0.004792
718.500000	0.004769
718.250000	0.004747
718.000000	0.004725
717.750000	0.004703
717.500000	0.004681

717.250000	0.004656
717.000000	0.004629
716.750000	0.004599
716.500000	0.004568
716.250000	0.004538
716.000000	0.004512
715.750000	0.004493
715.500000	0.004482
715.250000	0.004480
715.000000	0.004484
714.750000	0.004492
714.500000	0.004499
714.250000	0.004502
714.000000	0.004500
713.750000	0.004491
713.500000	0.004476
713.250000	0.004457
713.000000	0.004436
712.750000	0.004415
712.500000	0.004397
712.250000	0.004383
712.000000	0.004375
711.750000	0.004374
711.500000	0.004378
711.250000	0.004387
711.000000	0.004397

710.750000	0.004406
710.500000	0.004412
710.250000	0.004412
710.000000	0.004405
709.750000	0.004392
709.500000	0.004375
709.250000	0.004355
709.000000	0.004336
708.750000	0.004319
708.500000	0.004306
708.250000	0.004298
708.000000	0.004296
707.750000	0.004299
707.500000	0.004307
707.250000	0.004318
707.000000	0.004331
706.750000	0.004344
706.500000	0.004354
706.250000	0.004359
706.000000	0.004358
705.750000	0.004350
705.500000	0.004337
705.250000	0.004320
705.000000	0.004304
704.750000	0.004292
704.500000	0.004286

704.250000	0.004288
704.000000	0.004299
703.750000	0.004316
703.500000	0.004336
703.250000	0.004356
703.000000	0.004372
702.750000	0.004382
702.500000	0.004387
702.250000	0.004386
702.000000	0.004383
701.750000	0.004378
701.500000	0.004376
701.250000	0.004376
701.000000	0.004380
700.750000	0.004386
700.500000	0.004395
700.250000	0.004406
700.000000	0.004417
699.750000	0.004428
699.500000	0.004439
699.250000	0.004449
699.000000	0.004457
698.750000	0.004462
698.500000	0.004466
698.250000	0.004469
698.000000	0.004471

697.750000	0.004473
697.500000	0.004476
697.250000	0.004480
697.000000	0.004485
696.750000	0.004490
696.500000	0.004493
696.250000	0.004493
696.000000	0.004491
695.750000	0.004487
695.500000	0.004482
695.250000	0.004476
695.000000	0.004469
694.750000	0.004463
694.500000	0.004456
694.250000	0.004450
694.000000	0.004444
693.750000	0.004438
693.500000	0.004433
693.250000	0.004426
693.000000	0.004418
692.750000	0.004406
692.500000	0.004390
692.250000	0.004369
692.000000	0.004343
691.750000	0.004312
691.500000	0.004279

691.250000	0.004247
691.000000	0.004216
690.750000	0.004189
690.500000	0.004167
690.250000	0.004148
690.000000	0.004131
689.750000	0.004116
689.500000	0.004100
689.250000	0.004084
689.000000	0.004066
688.750000	0.004046
688.500000	0.004026
688.250000	0.004006
688.000000	0.003986
687.750000	0.003968
687.500000	0.003952
687.250000	0.003937
687.000000	0.003926
686.750000	0.003917
686.500000	0.003911
686.250000	0.003907
686.000000	0.003905
685.750000	0.003902
685.500000	0.003897
685.250000	0.003891
685.000000	0.003883

684.750000	0.003872
684.500000	0.003861
684.250000	0.003849
684.000000	0.003837
683.750000	0.003826
683.500000	0.003814
683.250000	0.003804
683.000000	0.003793
682.750000	0.003784
682.500000	0.003777
682.250000	0.003773
682.000000	0.003772
681.750000	0.003774
681.500000	0.003778
681.250000	0.003782
681.000000	0.003787
680.750000	0.003790
680.500000	0.003790
680.250000	0.003788
680.000000	0.003783
679.750000	0.003775
679.500000	0.003765
679.250000	0.003752
679.000000	0.003737
678.750000	0.003719
678.500000	0.003702

678.250000	0.003685
678.000000	0.003671
677.750000	0.003662
677.500000	0.003658
677.250000	0.003660
677.000000	0.003666
676.750000	0.003676
676.500000	0.003689
676.250000	0.003702
676.000000	0.003715
675.750000	0.003727
675.500000	0.003737
675.250000	0.003745
675.000000	0.003751
674.750000	0.003754
674.500000	0.003755
674.250000	0.003753
674.000000	0.003747
673.750000	0.003740
673.500000	0.003730
673.250000	0.003720
673.000000	0.003712
672.750000	0.003708
672.500000	0.003708
672.250000	0.003713
672.000000	0.003721

671.750000	0.003730
671.500000	0.003737
671.250000	0.003740
671.000000	0.003739
670.750000	0.003734
670.500000	0.003724
670.250000	0.003714
670.000000	0.003707
669.750000	0.003707
669.500000	0.003715
669.250000	0.003733
669.000000	0.003764
668.750000	0.003806
668.500000	0.003846
668.250000	0.003864
668.000000	0.003847
667.750000	0.003803
667.500000	0.003754
667.250000	0.003719
667.000000	0.003697
666.750000	0.003679
666.500000	0.003662
666.250000	0.003649
666.000000	0.003643
665.750000	0.003645
665.500000	0.003649

665.250000	0.003654
665.000000	0.003658
664.750000	0.003663
664.500000	0.003667
664.250000	0.003672
664.000000	0.003677
663.750000	0.003686
663.500000	0.003699
663.250000	0.003714
663.000000	0.003729
662.750000	0.003741
662.500000	0.003749
662.250000	0.003750
662.000000	0.003744
661.750000	0.003731
661.500000	0.003712
661.250000	0.003690
661.000000	0.003669
660.750000	0.003651
660.500000	0.003638
660.250000	0.003631
660.000000	0.003631
659.750000	0.003634
659.500000	0.003639
659.250000	0.003643
659.000000	0.003645

658.750000	0.003642
658.500000	0.003635
658.250000	0.003624
658.000000	0.003611
657.750000	0.003597
657.500000	0.003585
657.250000	0.003575
657.000000	0.003569
656.750000	0.003568
656.500000	0.003571
656.250000	0.003578
656.000000	0.003587
655.750000	0.003597
655.500000	0.003604
655.250000	0.003607
655.000000	0.003604
654.750000	0.003595
654.500000	0.003581
654.250000	0.003563
654.000000	0.003545
653.750000	0.003527
653.500000	0.003512
653.250000	0.003501
653.000000	0.003492
652.750000	0.003484
652.500000	0.003475

652.250000	0.003463
652.000000	0.003448
651.750000	0.003427
651.500000	0.003404
651.250000	0.003380
651.000000	0.003357
650.750000	0.003338
650.500000	0.003323
650.250000	0.003313
650.000000	0.003306
649.750000	0.003300
649.500000	0.003293
649.250000	0.003284
649.000000	0.003271
648.750000	0.003256
648.500000	0.003238
648.250000	0.003219
648.000000	0.003200
647.750000	0.003179
647.500000	0.003156
647.250000	0.003132
647.000000	0.003106
646.750000	0.003079
646.500000	0.003054
646.250000	0.003031
646.000000	0.003010

645.750000	0.002991
645.500000	0.002974
645.250000	0.002955
645.000000	0.002933
644.750000	0.002909
644.500000	0.002883
644.250000	0.002857
644.000000	0.002833
643.750000	0.002812
643.500000	0.002794
643.250000	0.002779
643.000000	0.002765
642.750000	0.002749
642.500000	0.002732
642.250000	0.002711
642.000000	0.002686
641.750000	0.002658
641.500000	0.002626
641.250000	0.002592
641.000000	0.002559
640.750000	0.002530
640.500000	0.002507
640.250000	0.002494
640.000000	0.002493
639.750000	0.002505
639.500000	0.002528

639.250000	0.002558
639.000000	0.002591
638.750000	0.002623
638.500000	0.002649
638.250000	0.002670
638.000000	0.002685
637.750000	0.002693
637.500000	0.002695
637.250000	0.002692
637.000000	0.002683
636.750000	0.002667
636.500000	0.002645
636.250000	0.002618
636.000000	0.002587
635.750000	0.002557
635.500000	0.002530
635.250000	0.002509
635.000000	0.002496
634.750000	0.002492
634.500000	0.002494
634.250000	0.002500
634.000000	0.002506
633.750000	0.002509
633.500000	0.002505
633.250000	0.002495
633.000000	0.002479

632.750000	0.002460
632.500000	0.002443
632.250000	0.002430
632.000000	0.002425
631.750000	0.002424
631.500000	0.002423
631.250000	0.002417
631.000000	0.002402
630.750000	0.002374
630.500000	0.002335
630.250000	0.002288
630.000000	0.002241
629.750000	0.002199
629.500000	0.002167
629.250000	0.002147
629.000000	0.002138
628.750000	0.002136
628.500000	0.002139
628.250000	0.002143
628.000000	0.002148
627.750000	0.002153
627.500000	0.002158
627.250000	0.002162
627.000000	0.002166
626.750000	0.002170
626.500000	0.002172

626.250000	0.002175
626.000000	0.002178
625.750000	0.002183
625.500000	0.002189
625.250000	0.002195
625.000000	0.002200
624.750000	0.002201
624.500000	0.002201
624.250000	0.002201
624.000000	0.002204
623.750000	0.002214
623.500000	0.002234
623.250000	0.002262
623.000000	0.002297
622.750000	0.002335
622.500000	0.002370
622.250000	0.002399
622.000000	0.002420
621.750000	0.002433
621.500000	0.002439
621.250000	0.002441
621.000000	0.002439
620.750000	0.002434
620.500000	0.002426
620.250000	0.002417
620.000000	0.002408

619.750000	0.002402
619.500000	0.002400
619.250000	0.002404
619.000000	0.002414
618.750000	0.002429
618.500000	0.002445
618.250000	0.002459
618.000000	0.002470
617.750000	0.002474
617.500000	0.002473
617.250000	0.002466
617.000000	0.002456
616.750000	0.002444
616.500000	0.002432
616.250000	0.002421
616.000000	0.002413
615.750000	0.002410
615.500000	0.002412
615.250000	0.002420
615.000000	0.002432
614.750000	0.002446
614.500000	0.002458
614.250000	0.002468
614.000000	0.002473
613.750000	0.002473
613.500000	0.002468

613.250000	0.002461
613.000000	0.002452
612.750000	0.002442
612.500000	0.002433
612.250000	0.002422
612.000000	0.002412
611.750000	0.002401
611.500000	0.002391
611.250000	0.002380
611.000000	0.002371
610.750000	0.002362
610.500000	0.002355
610.250000	0.002351
610.000000	0.002351
609.750000	0.002358
609.500000	0.002372
609.250000	0.002393
609.000000	0.002423
608.750000	0.002457
608.500000	0.002493
608.250000	0.002526
608.000000	0.002552
607.750000	0.002568
607.500000	0.002573
607.250000	0.002568
607.000000	0.002555

606.750000	0.002539
606.500000	0.002524
606.250000	0.002514
606.000000	0.002511
605.750000	0.002517
605.500000	0.002530
605.250000	0.002548
605.000000	0.002566
604.750000	0.002581
604.500000	0.002591
604.250000	0.002595
604.000000	0.002593
603.750000	0.002589
603.500000	0.002585
603.250000	0.002584
603.000000	0.002586
602.750000	0.002592
602.500000	0.002601
602.250000	0.002609
602.000000	0.002616
601.750000	0.002622
601.500000	0.002628
601.250000	0.002635
601.000000	0.002646
600.750000	0.002662
600.500000	0.002685

600.250000	0.002712
600.000000	0.002742
599.750000	0.002771
599.500000	0.002795
599.250000	0.002814
599.000000	0.002824
598.750000	0.002829
598.500000	0.002828
598.250000	0.002825
598.000000	0.002822
597.750000	0.002821
597.500000	0.002823
597.250000	0.002830
597.000000	0.002841
596.750000	0.002855
596.500000	0.002871
596.250000	0.002887
596.000000	0.002903
595.750000	0.002917
595.500000	0.002928
595.250000	0.002936
595.000000	0.002940
594.750000	0.002941
594.500000	0.002938
594.250000	0.002933
594.000000	0.002927

593.750000	0.002919
593.500000	0.002912
593.250000	0.002907
593.000000	0.002905
592.750000	0.002905
592.500000	0.002908
592.250000	0.002912
592.000000	0.002915
591.750000	0.002913
591.500000	0.002905
591.250000	0.002892
591.000000	0.002876
590.750000	0.002861
590.500000	0.002850
590.250000	0.002847
590.000000	0.002851
589.750000	0.002863
589.500000	0.002880
589.250000	0.002897
589.000000	0.002912
588.750000	0.002921
588.500000	0.002923
588.250000	0.002917
588.000000	0.002905
587.750000	0.002889
587.500000	0.002870

587.250000	0.002850
587.000000	0.002829
586.750000	0.002808
586.500000	0.002787
586.250000	0.002767
586.000000	0.002749
585.750000	0.002736
585.500000	0.002729
585.250000	0.002729
585.000000	0.002738
584.750000	0.002754
584.500000	0.002775
584.250000	0.002798
584.000000	0.002819
583.750000	0.002835
583.500000	0.002843
583.250000	0.002845
583.000000	0.002840
582.750000	0.002833
582.500000	0.002827
582.250000	0.002826
582.000000	0.002831
581.750000	0.002842
581.500000	0.002855
581.250000	0.002867
581.000000	0.002874

580.750000	0.002874
580.500000	0.002867
580.250000	0.002855
580.000000	0.002844
579.750000	0.002835
579.500000	0.002832
579.250000	0.002835
579.000000	0.002843
578.750000	0.002855
578.500000	0.002870
578.250000	0.002888
578.000000	0.002908
577.750000	0.002928
577.500000	0.002949
577.250000	0.002969
577.000000	0.002986
576.750000	0.002999
576.500000	0.003011
576.250000	0.003021
576.000000	0.003033
575.750000	0.003047
575.500000	0.003063
575.250000	0.003080
575.000000	0.003094
574.750000	0.003103
574.500000	0.003105

574.250000	0.003102
574.000000	0.003096
573.750000	0.003090
573.500000	0.003088
573.250000	0.003092
573.000000	0.003101
572.750000	0.003113
572.500000	0.003125
572.250000	0.003134
572.000000	0.003137
571.750000	0.003131
571.500000	0.003116
571.250000	0.003093
571.000000	0.003063
570.750000	0.003028
570.500000	0.002990
570.250000	0.002954
570.000000	0.002923
569.750000	0.002901
569.500000	0.002889
569.250000	0.002888
569.000000	0.002898
568.750000	0.002917
568.500000	0.002943
568.250000	0.002973
568.000000	0.003006

567.750000	0.003040
567.500000	0.003070
567.250000	0.003094
567.000000	0.003109
566.750000	0.003113
566.500000	0.003105
566.250000	0.003089
566.000000	0.003068
565.750000	0.003046
565.500000	0.003027
565.250000	0.003012
565.000000	0.003001
564.750000	0.002991
564.500000	0.002982
564.250000	0.002973
564.000000	0.002964
563.750000	0.002957
563.500000	0.002957
563.250000	0.002963
563.000000	0.002976
562.750000	0.002994
562.500000	0.003014
562.250000	0.003031
562.000000	0.003046
561.750000	0.003058
561.500000	0.003069

561.250000	0.003082
561.000000	0.003100
560.750000	0.003123
560.500000	0.003151
560.250000	0.003182
560.000000	0.003213
559.750000	0.003243
559.500000	0.003268
559.250000	0.003287
559.000000	0.003297
558.750000	0.003297
558.500000	0.003286
558.250000	0.003265
558.000000	0.003236
557.750000	0.003203
557.500000	0.003170
557.250000	0.003144
557.000000	0.003127
556.750000	0.003123
556.500000	0.003130
556.250000	0.003146
556.000000	0.003167
555.750000	0.003188
555.500000	0.003207
555.250000	0.003220
555.000000	0.003226

554.750000	0.003227
554.500000	0.003223
554.250000	0.003215
554.000000	0.003203
553.750000	0.003187
553.500000	0.003164
553.250000	0.003135
553.000000	0.003100
552.750000	0.003060
552.500000	0.003022
552.250000	0.002988
552.000000	0.002964
551.750000	0.002950
551.500000	0.002945
551.250000	0.002944
551.000000	0.002939
550.750000	0.002925
550.500000	0.002898
550.250000	0.002858
550.000000	0.002809
549.750000	0.002759
549.500000	0.002715
549.250000	0.002684
549.000000	0.002666
548.750000	0.002660
548.500000	0.002661

548.250000	0.002662
548.000000	0.002659
547.750000	0.002648
547.500000	0.002628
547.250000	0.002599
547.000000	0.002565
546.750000	0.002527
546.500000	0.002488
546.250000	0.002451
546.000000	0.002418
545.750000	0.002391
545.500000	0.002373
545.250000	0.002365
545.000000	0.002368
544.750000	0.002381
544.500000	0.002401
544.250000	0.002423
544.000000	0.002441
543.750000	0.002452
543.500000	0.002450
543.250000	0.002434
543.000000	0.002405
542.750000	0.002367
542.500000	0.002325
542.250000	0.002286
542.000000	0.002256

541.750000	0.002237
541.500000	0.002229
541.250000	0.002229
541.000000	0.002232
540.750000	0.002234
540.500000	0.002230
540.250000	0.002219
540.000000	0.002201
539.750000	0.002181
539.500000	0.002160
539.250000	0.002140
539.000000	0.002123
538.750000	0.002107
538.500000	0.002092
538.250000	0.002075
538.000000	0.002058
537.750000	0.002041
537.500000	0.002028
537.250000	0.002020
537.000000	0.002018
536.750000	0.002021
536.500000	0.002026
536.250000	0.002030
536.000000	0.002030
535.750000	0.002025
535.500000	0.002016

535.250000	0.002004
535.000000	0.001992
534.750000	0.001981
534.500000	0.001972
534.250000	0.001964
534.000000	0.001957
533.750000	0.001950
533.500000	0.001943
533.250000	0.001937
533.000000	0.001933
532.750000	0.001932
532.500000	0.001934
532.250000	0.001937
532.000000	0.001939
531.750000	0.001940
531.500000	0.001938
531.250000	0.001934
531.000000	0.001932
530.750000	0.001932
530.500000	0.001937
530.250000	0.001949
530.000000	0.001967
529.750000	0.001989
529.500000	0.002013
529.250000	0.002037
529.000000	0.002059

528.750000	0.002080
528.500000	0.002098
528.250000	0.002113
528.000000	0.002121
527.750000	0.002121
527.500000	0.002110
527.250000	0.002085
527.000000	0.002048
526.750000	0.002002
526.500000	0.001953
526.250000	0.001911
526.000000	0.001885
525.750000	0.001878
525.500000	0.001887
525.250000	0.001908
525.000000	0.001931
524.750000	0.001952
524.500000	0.001968
524.250000	0.001983
524.000000	0.001999
523.750000	0.002017
523.500000	0.002040
523.250000	0.002063
523.000000	0.002082
522.750000	0.002090
522.500000	0.002083

522.250000	0.002060
522.000000	0.002026
521.750000	0.001987
521.500000	0.001951
521.250000	0.001925
521.000000	0.001912
520.750000	0.001911
520.500000	0.001920
520.250000	0.001933
520.000000	0.001944
519.750000	0.001948
519.500000	0.001944
519.250000	0.001936
519.000000	0.001928
518.750000	0.001926
518.500000	0.001934
518.250000	0.001954
518.000000	0.001983
517.750000	0.002016
517.500000	0.002046
517.250000	0.002065
517.000000	0.002068
516.750000	0.002052
516.500000	0.002023
516.250000	0.001987
516.000000	0.001953

515.750000	0.001926
515.500000	0.001910
515.250000	0.001905
515.000000	0.001911
514.750000	0.001924
514.500000	0.001943
514.250000	0.001964
514.000000	0.001984
513.750000	0.002001
513.500000	0.002016
513.250000	0.002028
513.000000	0.002039
512.750000	0.002050
512.500000	0.002061
512.250000	0.002072
512.000000	0.002082
511.750000	0.002090
511.500000	0.002095
511.250000	0.002095
511.000000	0.002092
510.750000	0.002085
510.500000	0.002078
510.250000	0.002074
510.000000	0.002072
509.750000	0.002076
509.500000	0.002083

509.250000	0.002092
509.000000	0.002101
508.750000	0.002109
508.500000	0.002114
508.250000	0.002119
508.000000	0.002126
507.750000	0.002137
507.500000	0.002156
507.250000	0.002182
507.000000	0.002213
506.750000	0.002244
506.500000	0.002270
506.250000	0.002286
506.000000	0.002292
505.750000	0.002289
505.500000	0.002282
505.250000	0.002278
505.000000	0.002283
504.750000	0.002300
504.500000	0.002331
504.250000	0.002372
504.000000	0.002416
503.750000	0.002455
503.500000	0.002484
503.250000	0.002500
503.000000	0.002503

502.750000	0.002499
502.500000	0.002497
502.250000	0.002506
502.000000	0.002534
501.750000	0.002583
501.500000	0.002649
501.250000	0.002726
501.000000	0.002808
500.750000	0.002890
500.500000	0.002970
500.250000	0.003043
500.000000	0.003109
499.750000	0.003162
499.500000	0.003202
499.250000	0.003229
499.000000	0.003244
498.750000	0.003253
498.500000	0.003264
498.250000	0.003282
498.000000	0.003313
497.750000	0.003358
497.500000	0.003414
497.250000	0.003479
497.000000	0.003546
496.750000	0.003612
496.500000	0.003675

496.250000	0.003734
496.000000	0.003792
495.750000	0.003851
495.500000	0.003913
495.250000	0.003982
495.000000	0.004058
494.750000	0.004143
494.500000	0.004236
494.250000	0.004335
494.000000	0.004438
493.750000	0.004540
493.500000	0.004638
493.250000	0.004727
493.000000	0.004804
492.750000	0.004870
492.500000	0.004925
492.250000	0.004972
492.000000	0.005015
491.750000	0.005056
491.500000	0.005099
491.250000	0.005144
491.000000	0.005192
490.750000	0.005246
490.500000	0.005307
490.250000	0.005376
490.000000	0.005455

489.750000	0.005545
489.500000	0.005642
489.250000	0.005745
489.000000	0.005849
488.750000	0.005954
488.500000	0.006057
488.250000	0.006161
488.000000	0.006264
487.750000	0.006365
487.500000	0.006462
487.250000	0.006548
487.000000	0.006617
486.750000	0.006666
486.500000	0.006695
486.250000	0.006711
486.000000	0.006723
485.750000	0.006742
485.500000	0.006777
485.250000	0.006830
485.000000	0.006900
484.750000	0.006983
484.500000	0.007071
484.250000	0.007160
484.000000	0.007248
483.750000	0.007335
483.500000	0.007425

483.250000	0.007523
483.000000	0.007628
482.750000	0.007742
482.500000	0.007859
482.250000	0.007978
482.000000	0.008094
481.750000	0.008206
481.500000	0.008312
481.250000	0.008410
481.000000	0.008501
480.750000	0.008584
480.500000	0.008660
480.250000	0.008729
480.000000	0.008796
479.750000	0.008865
479.500000	0.008940
479.250000	0.009026
479.000000	0.009123
478.750000	0.009229
478.500000	0.009338
478.250000	0.009443
478.000000	0.009539
477.750000	0.009623
477.500000	0.009696
477.250000	0.009764
477.000000	0.009833

476.750000	0.009910
476.500000	0.009997
476.250000	0.010093
476.000000	0.010193
475.750000	0.010289
475.500000	0.010377
475.250000	0.010454
475.000000	0.010519
474.750000	0.010576
474.500000	0.010628
474.250000	0.010678
474.000000	0.010731
473.750000	0.010790
473.500000	0.010860
473.250000	0.010947
473.000000	0.011054
472.750000	0.011178
472.500000	0.011306
472.250000	0.011425
472.000000	0.011524
471.750000	0.011603
471.500000	0.011667
471.250000	0.011727
471.000000	0.011789
470.750000	0.011859
470.500000	0.011938

470.250000	0.012026
470.000000	0.012119
469.750000	0.012212
469.500000	0.012302
469.250000	0.012383
469.000000	0.012457
468.750000	0.012525
468.500000	0.012591
468.250000	0.012658
468.000000	0.012729
467.750000	0.012807
467.500000	0.012892
467.250000	0.012981
467.000000	0.013070
466.750000	0.013155
466.500000	0.013231
466.250000	0.013291
466.000000	0.013335
465.750000	0.013366
465.500000	0.013390
465.250000	0.013417
465.000000	0.013454
464.750000	0.013505
464.500000	0.013569
464.250000	0.013636
464.000000	0.013692

463.750000	0.013727
463.500000	0.013734
463.250000	0.013713
463.000000	0.013672
462.750000	0.013623
462.500000	0.013578
462.250000	0.013550
462.000000	0.013544
461.750000	0.013564
461.500000	0.013606
461.250000	0.013665
461.000000	0.013736
460.750000	0.013810
460.500000	0.013882
460.250000	0.013944
460.000000	0.013990
459.750000	0.014016
459.500000	0.014021
459.250000	0.014004
459.000000	0.013971
458.750000	0.013932
458.500000	0.013895
458.250000	0.013865
458.000000	0.013839
457.750000	0.013803
457.500000	0.013746

457.250000	0.013670
457.000000	0.013587
456.750000	0.013514
456.500000	0.013462
456.250000	0.013434
456.000000	0.013427
455.750000	0.013438
455.500000	0.013461
455.250000	0.013489
455.000000	0.013514
454.750000	0.013533
454.500000	0.013550
454.250000	0.013570
454.000000	0.013600
453.750000	0.013647
453.500000	0.013710
453.250000	0.013782
453.000000	0.013851
452.750000	0.013906
452.500000	0.013941
452.250000	0.013958
452.000000	0.013967
451.750000	0.013976
451.500000	0.013991
451.250000	0.014010
451.000000	0.014027

450.750000	0.014034
450.500000	0.014023
450.250000	0.013987
450.000000	0.013925
449.750000	0.013838
449.500000	0.013733
449.250000	0.013617
449.000000	0.013496
448.750000	0.013378
448.500000	0.013269
448.250000	0.013175
448.000000	0.013102
447.750000	0.013057
447.500000	0.013046
447.250000	0.013071
447.000000	0.013128
446.750000	0.013205
446.500000	0.013284
446.250000	0.013347
446.000000	0.013379
445.750000	0.013372
445.500000	0.013328
445.250000	0.013253
445.000000	0.013158
444.750000	0.013058
444.500000	0.012969

444.250000	0.012908
444.000000	0.012886
443.750000	0.012906
443.500000	0.012958
443.250000	0.013020
443.000000	0.013070
442.750000	0.013087
442.500000	0.013062
442.250000	0.012996
442.000000	0.012903
441.750000	0.012798
441.500000	0.012697
441.250000	0.012609
441.000000	0.012539
440.750000	0.012484
440.500000	0.012437
440.250000	0.012395
440.000000	0.012355
439.750000	0.012315
439.500000	0.012275
439.250000	0.012236
439.000000	0.012199
438.750000	0.012165
438.500000	0.012133
438.250000	0.012102
438.000000	0.012070

437.750000	0.012035
437.500000	0.011995
437.250000	0.011947
437.000000	0.011895
436.750000	0.011843
436.500000	0.011800
436.250000	0.011776
436.000000	0.011772
435.750000	0.011784
435.500000	0.011798
435.250000	0.011793
435.000000	0.011753
434.750000	0.011664
434.500000	0.011527
434.250000	0.011353
434.000000	0.011165
433.750000	0.010991
433.500000	0.010855
433.250000	0.010774
433.000000	0.010752
432.750000	0.010784
432.500000	0.010854
432.250000	0.010942
432.000000	0.011029
431.750000	0.011094
431.500000	0.011118

431.250000	0.011091
431.000000	0.011011
430.750000	0.010887
430.500000	0.010735
430.250000	0.010573
430.000000	0.010422
429.750000	0.010294
429.500000	0.010196
429.250000	0.010129
429.000000	0.010088
428.750000	0.010064
428.500000	0.010050
428.250000	0.010043
428.000000	0.010044
427.750000	0.010056
427.500000	0.010081
427.250000	0.010113
427.000000	0.010141
426.750000	0.010148
426.500000	0.010111
426.250000	0.010013
426.000000	0.009848
425.750000	0.009627
425.500000	0.009379
425.250000	0.009143
425.000000	0.008953

424.750000	0.008824
424.500000	0.008756
424.250000	0.008735
424.000000	0.008744
423.750000	0.008765
423.500000	0.008786
423.250000	0.008800
423.000000	0.008805
422.750000	0.008804
422.500000	0.008805
422.250000	0.008810
422.000000	0.008821
421.750000	0.008832
421.500000	0.008835
421.250000	0.008824
421.000000	0.008796
420.750000	0.008752
420.500000	0.008695
420.250000	0.008629
420.000000	0.008566
419.750000	0.008519
419.500000	0.008491
419.250000	0.008452
419.000000	0.008353
418.750000	0.008168
418.500000	0.007916

418.250000	0.007653
418.000000	0.007422
417.750000	0.007235
417.500000	0.007083
417.250000	0.006948
417.000000	0.006820
416.750000	0.006692
416.500000	0.006564
416.250000	0.006440
416.000000	0.006330
415.750000	0.006246
415.500000	0.006197
415.250000	0.006187
415.000000	0.006211
414.750000	0.006257
414.500000	0.006305
414.250000	0.006337
414.000000	0.006335
413.750000	0.006293
413.500000	0.006210
413.250000	0.006098
413.000000	0.005972
412.750000	0.005850
412.500000	0.005750
412.250000	0.005683
412.000000	0.005651

411.750000	0.005645
411.500000	0.005646
411.250000	0.005631
411.000000	0.005575
410.750000	0.005465
410.500000	0.005299
410.250000	0.005089
410.000000	0.004860
409.750000	0.004637
409.500000	0.004439
409.250000	0.004273
409.000000	0.004131
408.750000	0.003993
408.500000	0.003838
408.250000	0.003655
408.000000	0.003444
407.750000	0.003228
407.500000	0.003042
407.250000	0.002927
407.000000	0.002913
406.750000	0.003011
406.500000	0.003209
406.250000	0.003470
406.000000	0.003745
405.750000	0.003980
405.500000	0.004134

405.250000	0.004182
405.000000	0.004123
404.750000	0.003977
404.500000	0.003771
404.250000	0.003531
404.000000	0.003274
403.750000	0.003005
403.500000	0.002729
403.250000	0.002450
403.000000	0.002181
402.750000	0.001946
402.500000	0.001769
402.250000	0.001670
402.000000	0.001658
401.750000	0.001723
401.500000	0.001846
401.250000	0.001999
401.000000	0.002143
400.750000	0.002228
400.500000	0.002211
400.250000	0.002096
400.000000	0.001928

A1_2mm_d

PE IR SUBTECH SPECTRUM ASCII PEDS 4.00

-1

A1_2mm_d_1_1_1.ASC

21/07/23

16:26:33.00

21/12/06

15:42:15.00

ir

Sample 023 By ir Date venerd, luglio 23 2021

400.000000

#HDR

-1

-1

#GR

cm-1

A

1.0

0.0

4000.000000

-0.250000

14401

8

0.030425

0.000595

#DATA

4000.000000 0.000853

3999.750000	0.000858
3999.500000	0.000864
3999.250000	0.000870
3999.000000	0.000875
3998.750000	0.000881
3998.500000	0.000887
3998.250000	0.000894
3998.000000	0.000901
3997.750000	0.000908
3997.500000	0.000914
3997.250000	0.000918
3997.000000	0.000920
3996.750000	0.000921
3996.500000	0.000921
3996.250000	0.000920
3996.000000	0.000921
3995.750000	0.000922
3995.500000	0.000925
3995.250000	0.000930
3995.000000	0.000934
3994.750000	0.000939
3994.500000	0.000943
3994.250000	0.000947
3994.000000	0.000951
3993.750000	0.000956
3993.500000	0.000963

3993.250000	0.000970
3993.000000	0.000979
3992.750000	0.000988
3992.500000	0.000995
3992.250000	0.001001
3992.000000	0.001004
3991.750000	0.001003
3991.500000	0.000998
3991.250000	0.000991
3991.000000	0.000982
3990.750000	0.000971
3990.500000	0.000961
3990.250000	0.000951
3990.000000	0.000942
3989.750000	0.000936
3989.500000	0.000934
3989.250000	0.000934
3989.000000	0.000938
3988.750000	0.000943
3988.500000	0.000951
3988.250000	0.000959
3988.000000	0.000966
3987.750000	0.000973
3987.500000	0.000979
3987.250000	0.000983
3987.000000	0.000987

3986.750000	0.000989
3986.500000	0.000989
3986.250000	0.000987
3986.000000	0.000981
3985.750000	0.000973
3985.500000	0.000962
3985.250000	0.000949
3985.000000	0.000936
3984.750000	0.000925
3984.500000	0.000917
3984.250000	0.000912
3984.000000	0.000912
3983.750000	0.000916
3983.500000	0.000925
3983.250000	0.000936
3983.000000	0.000949
3982.750000	0.000963
3982.500000	0.000976
3982.250000	0.000986
3982.000000	0.000992
3981.750000	0.000994
3981.500000	0.000992
3981.250000	0.000987
3981.000000	0.000980
3980.750000	0.000972
3980.500000	0.000965

3980.250000	0.000959
3980.000000	0.000953
3979.750000	0.000947
3979.500000	0.000940
3979.250000	0.000933
3979.000000	0.000926
3978.750000	0.000922
3978.500000	0.000923
3978.250000	0.000929
3978.000000	0.000941
3977.750000	0.000958
3977.500000	0.000978
3977.250000	0.000996
3977.000000	0.001009
3976.750000	0.001015
3976.500000	0.001014
3976.250000	0.001006
3976.000000	0.000994
3975.750000	0.000981
3975.500000	0.000971
3975.250000	0.000966
3975.000000	0.000968
3974.750000	0.000976
3974.500000	0.000990
3974.250000	0.001009
3974.000000	0.001031

3973.750000	0.001053
3973.500000	0.001073
3973.250000	0.001090
3973.000000	0.001101
3972.750000	0.001105
3972.500000	0.001103
3972.250000	0.001096
3972.000000	0.001086
3971.750000	0.001075
3971.500000	0.001068
3971.250000	0.001064
3971.000000	0.001066
3970.750000	0.001072
3970.500000	0.001079
3970.250000	0.001086
3970.000000	0.001090
3969.750000	0.001088
3969.500000	0.001080
3969.250000	0.001066
3969.000000	0.001048
3968.750000	0.001028
3968.500000	0.001008
3968.250000	0.000991
3968.000000	0.000978
3967.750000	0.000969
3967.500000	0.000963

3967.250000	0.000960
3967.000000	0.000959
3966.750000	0.000957
3966.500000	0.000956
3966.250000	0.000955
3966.000000	0.000954
3965.750000	0.000955
3965.500000	0.000960
3965.250000	0.000969
3965.000000	0.000983
3964.750000	0.001002
3964.500000	0.001026
3964.250000	0.001053
3964.000000	0.001080
3963.750000	0.001106
3963.500000	0.001127
3963.250000	0.001143
3963.000000	0.001152
3962.750000	0.001152
3962.500000	0.001144
3962.250000	0.001130
3962.000000	0.001111
3961.750000	0.001090
3961.500000	0.001070
3961.250000	0.001054
3961.000000	0.001045

3960.750000	0.001043
3960.500000	0.001048
3960.250000	0.001060
3960.000000	0.001076
3959.750000	0.001093
3959.500000	0.001110
3959.250000	0.001125
3959.000000	0.001136
3958.750000	0.001145
3958.500000	0.001150
3958.250000	0.001153
3958.000000	0.001154
3957.750000	0.001154
3957.500000	0.001152
3957.250000	0.001148
3957.000000	0.001142
3956.750000	0.001132
3956.500000	0.001119
3956.250000	0.001101
3956.000000	0.001080
3955.750000	0.001058
3955.500000	0.001036
3955.250000	0.001017
3955.000000	0.001003
3954.750000	0.000997
3954.500000	0.001000

3954.250000	0.001012
3954.000000	0.001029
3953.750000	0.001050
3953.500000	0.001068
3953.250000	0.001080
3953.000000	0.001083
3952.750000	0.001077
3952.500000	0.001066
3952.250000	0.001054
3952.000000	0.001047
3951.750000	0.001050
3951.500000	0.001066
3951.250000	0.001095
3951.000000	0.001134
3950.750000	0.001176
3950.500000	0.001216
3950.250000	0.001246
3950.000000	0.001265
3949.750000	0.001272
3949.500000	0.001268
3949.250000	0.001258
3949.000000	0.001246
3948.750000	0.001234
3948.500000	0.001223
3948.250000	0.001213
3948.000000	0.001203

3947.750000	0.001190
3947.500000	0.001177
3947.250000	0.001162
3947.000000	0.001148
3946.750000	0.001138
3946.500000	0.001134
3946.250000	0.001134
3946.000000	0.001140
3945.750000	0.001149
3945.500000	0.001160
3945.250000	0.001170
3945.000000	0.001180
3944.750000	0.001188
3944.500000	0.001195
3944.250000	0.001201
3944.000000	0.001207
3943.750000	0.001211
3943.500000	0.001213
3943.250000	0.001210
3943.000000	0.001201
3942.750000	0.001184
3942.500000	0.001161
3942.250000	0.001136
3942.000000	0.001112
3941.750000	0.001093
3941.500000	0.001083

3941.250000	0.001082
3941.000000	0.001090
3940.750000	0.001105
3940.500000	0.001124
3940.250000	0.001145
3940.000000	0.001165
3939.750000	0.001181
3939.500000	0.001191
3939.250000	0.001193
3939.000000	0.001188
3938.750000	0.001177
3938.500000	0.001161
3938.250000	0.001145
3938.000000	0.001130
3937.750000	0.001118
3937.500000	0.001111
3937.250000	0.001110
3937.000000	0.001114
3936.750000	0.001121
3936.500000	0.001129
3936.250000	0.001139
3936.000000	0.001147
3935.750000	0.001153
3935.500000	0.001158
3935.250000	0.001162
3935.000000	0.001169

3934.750000	0.001178
3934.500000	0.001192
3934.250000	0.001212
3934.000000	0.001236
3933.750000	0.001265
3933.500000	0.001297
3933.250000	0.001329
3933.000000	0.001357
3932.750000	0.001375
3932.500000	0.001380
3932.250000	0.001370
3932.000000	0.001347
3931.750000	0.001315
3931.500000	0.001281
3931.250000	0.001251
3931.000000	0.001232
3930.750000	0.001225
3930.500000	0.001233
3930.250000	0.001252
3930.000000	0.001277
3929.750000	0.001302
3929.500000	0.001323
3929.250000	0.001335
3929.000000	0.001337
3928.750000	0.001330
3928.500000	0.001317

3928.250000	0.001302
3928.000000	0.001288
3927.750000	0.001279
3927.500000	0.001276
3927.250000	0.001280
3927.000000	0.001290
3926.750000	0.001305
3926.500000	0.001322
3926.250000	0.001340
3926.000000	0.001358
3925.750000	0.001372
3925.500000	0.001383
3925.250000	0.001388
3925.000000	0.001387
3924.750000	0.001379
3924.500000	0.001367
3924.250000	0.001352
3924.000000	0.001335
3923.750000	0.001320
3923.500000	0.001306
3923.250000	0.001296
3923.000000	0.001290
3922.750000	0.001287
3922.500000	0.001289
3922.250000	0.001294
3922.000000	0.001302

3921.750000	0.001313
3921.500000	0.001325
3921.250000	0.001338
3921.000000	0.001348
3920.750000	0.001350
3920.500000	0.001340
3920.250000	0.001315
3920.000000	0.001276
3919.750000	0.001228
3919.500000	0.001180
3919.250000	0.001139
3919.000000	0.001114
3918.750000	0.001108
3918.500000	0.001123
3918.250000	0.001157
3918.000000	0.001205
3917.750000	0.001256
3917.500000	0.001298
3917.250000	0.001320
3917.000000	0.001315
3916.750000	0.001283
3916.500000	0.001232
3916.250000	0.001173
3916.000000	0.001116
3915.750000	0.001072
3915.500000	0.001046

3915.250000	0.001040
3915.000000	0.001054
3914.750000	0.001082
3914.500000	0.001119
3914.250000	0.001159
3914.000000	0.001195
3913.750000	0.001223
3913.500000	0.001239
3913.250000	0.001243
3913.000000	0.001236
3912.750000	0.001220
3912.500000	0.001199
3912.250000	0.001176
3912.000000	0.001156
3911.750000	0.001141
3911.500000	0.001132
3911.250000	0.001129
3911.000000	0.001130
3910.750000	0.001133
3910.500000	0.001137
3910.250000	0.001142
3910.000000	0.001147
3909.750000	0.001152
3909.500000	0.001158
3909.250000	0.001164
3909.000000	0.001172

3908.750000	0.001180
3908.500000	0.001190
3908.250000	0.001203
3908.000000	0.001220
3907.750000	0.001242
3907.500000	0.001269
3907.250000	0.001301
3907.000000	0.001334
3906.750000	0.001365
3906.500000	0.001391
3906.250000	0.001407
3906.000000	0.001410
3905.750000	0.001402
3905.500000	0.001383
3905.250000	0.001357
3905.000000	0.001330
3904.750000	0.001306
3904.500000	0.001287
3904.250000	0.001276
3904.000000	0.001276
3903.750000	0.001288
3903.500000	0.001308
3903.250000	0.001334
3903.000000	0.001361
3902.750000	0.001382
3902.500000	0.001394

3902.250000	0.001395
3902.000000	0.001384
3901.750000	0.001363
3901.500000	0.001338
3901.250000	0.001313
3901.000000	0.001294
3900.750000	0.001284
3900.500000	0.001288
3900.250000	0.001309
3900.000000	0.001347
3899.750000	0.001394
3899.500000	0.001435
3899.250000	0.001456
3899.000000	0.001446
3898.750000	0.001405
3898.500000	0.001344
3898.250000	0.001275
3898.000000	0.001214
3897.750000	0.001170
3897.500000	0.001150
3897.250000	0.001154
3897.000000	0.001179
3896.750000	0.001215
3896.500000	0.001255
3896.250000	0.001292
3896.000000	0.001322

3895.750000	0.001343
3895.500000	0.001357
3895.250000	0.001367
3895.000000	0.001374
3894.750000	0.001380
3894.500000	0.001383
3894.250000	0.001383
3894.000000	0.001380
3893.750000	0.001374
3893.500000	0.001365
3893.250000	0.001355
3893.000000	0.001345
3892.750000	0.001337
3892.500000	0.001333
3892.250000	0.001333
3892.000000	0.001337
3891.750000	0.001336
3891.500000	0.001325
3891.250000	0.001298
3891.000000	0.001258
3890.750000	0.001214
3890.500000	0.001176
3890.250000	0.001151
3890.000000	0.001146
3889.750000	0.001160
3889.500000	0.001190

3889.250000	0.001230
3889.000000	0.001272
3888.750000	0.001310
3888.500000	0.001336
3888.250000	0.001346
3888.000000	0.001337
3887.750000	0.001310
3887.500000	0.001269
3887.250000	0.001223
3887.000000	0.001182
3886.750000	0.001159
3886.500000	0.001159
3886.250000	0.001179
3886.000000	0.001206
3885.750000	0.001228
3885.500000	0.001240
3885.250000	0.001245
3885.000000	0.001251
3884.750000	0.001266
3884.500000	0.001291
3884.250000	0.001325
3884.000000	0.001365
3883.750000	0.001407
3883.500000	0.001448
3883.250000	0.001483
3883.000000	0.001510

3882.750000	0.001528
3882.500000	0.001538
3882.250000	0.001543
3882.000000	0.001545
3881.750000	0.001550
3881.500000	0.001559
3881.250000	0.001570
3881.000000	0.001574
3880.750000	0.001559
3880.500000	0.001515
3880.250000	0.001442
3880.000000	0.001352
3879.750000	0.001262
3879.500000	0.001190
3879.250000	0.001145
3879.000000	0.001132
3878.750000	0.001147
3878.500000	0.001181
3878.250000	0.001228
3878.000000	0.001277
3877.750000	0.001324
3877.500000	0.001363
3877.250000	0.001392
3877.000000	0.001413
3876.750000	0.001426
3876.500000	0.001434

3876.250000	0.001438
3876.000000	0.001441
3875.750000	0.001446
3875.500000	0.001456
3875.250000	0.001472
3875.000000	0.001495
3874.750000	0.001519
3874.500000	0.001533
3874.250000	0.001530
3874.000000	0.001507
3873.750000	0.001470
3873.500000	0.001432
3873.250000	0.001404
3873.000000	0.001394
3872.750000	0.001406
3872.500000	0.001435
3872.250000	0.001473
3872.000000	0.001512
3871.750000	0.001541
3871.500000	0.001557
3871.250000	0.001562
3871.000000	0.001567
3870.750000	0.001581
3870.500000	0.001608
3870.250000	0.001637
3870.000000	0.001649

3869.750000	0.001623
3869.500000	0.001561
3869.250000	0.001478
3869.000000	0.001396
3868.750000	0.001334
3868.500000	0.001305
3868.250000	0.001309
3868.000000	0.001343
3867.750000	0.001397
3867.500000	0.001462
3867.250000	0.001523
3867.000000	0.001571
3866.750000	0.001601
3866.500000	0.001613
3866.250000	0.001615
3866.000000	0.001615
3865.750000	0.001620
3865.500000	0.001628
3865.250000	0.001632
3865.000000	0.001621
3864.750000	0.001593
3864.500000	0.001552
3864.250000	0.001510
3864.000000	0.001478
3863.750000	0.001463
3863.500000	0.001468

3863.250000	0.001493
3863.000000	0.001534
3862.750000	0.001583
3862.500000	0.001631
3862.250000	0.001665
3862.000000	0.001676
3861.750000	0.001660
3861.500000	0.001620
3861.250000	0.001567
3861.000000	0.001511
3860.750000	0.001464
3860.500000	0.001433
3860.250000	0.001421
3860.000000	0.001427
3859.750000	0.001450
3859.500000	0.001485
3859.250000	0.001527
3859.000000	0.001570
3858.750000	0.001610
3858.500000	0.001645
3858.250000	0.001672
3858.000000	0.001690
3857.750000	0.001697
3857.500000	0.001691
3857.250000	0.001670
3857.000000	0.001634

3856.750000	0.001582
3856.500000	0.001513
3856.250000	0.001424
3856.000000	0.001312
3855.750000	0.001176
3855.500000	0.001021
3855.250000	0.000867
3855.000000	0.000741
3854.750000	0.000675
3854.500000	0.000683
3854.250000	0.000761
3854.000000	0.000880
3853.750000	0.001008
3853.500000	0.001119
3853.250000	0.001202
3853.000000	0.001252
3852.750000	0.001278
3852.500000	0.001293
3852.250000	0.001315
3852.000000	0.001355
3851.750000	0.001416
3851.500000	0.001491
3851.250000	0.001572
3851.000000	0.001645
3850.750000	0.001703
3850.500000	0.001743

3850.250000	0.001766
3850.000000	0.001777
3849.750000	0.001778
3849.500000	0.001770
3849.250000	0.001751
3849.000000	0.001722
3848.750000	0.001682
3848.500000	0.001635
3848.250000	0.001586
3848.000000	0.001541
3847.750000	0.001503
3847.500000	0.001473
3847.250000	0.001452
3847.000000	0.001435
3846.750000	0.001421
3846.500000	0.001408
3846.250000	0.001395
3846.000000	0.001384
3845.750000	0.001376
3845.500000	0.001373
3845.250000	0.001377
3845.000000	0.001391
3844.750000	0.001415
3844.500000	0.001445
3844.250000	0.001477
3844.000000	0.001501

3843.750000	0.001514
3843.500000	0.001514
3843.250000	0.001505
3843.000000	0.001493
3842.750000	0.001485
3842.500000	0.001486
3842.250000	0.001499
3842.000000	0.001529
3841.750000	0.001576
3841.500000	0.001637
3841.250000	0.001704
3841.000000	0.001765
3840.750000	0.001811
3840.500000	0.001834
3840.250000	0.001833
3840.000000	0.001811
3839.750000	0.001772
3839.500000	0.001724
3839.250000	0.001676
3839.000000	0.001637
3838.750000	0.001615
3838.500000	0.001611
3838.250000	0.001617
3838.000000	0.001619
3837.750000	0.001610
3837.500000	0.001590

3837.250000	0.001569
3837.000000	0.001554
3836.750000	0.001545
3836.500000	0.001542
3836.250000	0.001546
3836.000000	0.001559
3835.750000	0.001581
3835.500000	0.001610
3835.250000	0.001634
3835.000000	0.001645
3834.750000	0.001637
3834.500000	0.001614
3834.250000	0.001582
3834.000000	0.001551
3833.750000	0.001527
3833.500000	0.001512
3833.250000	0.001511
3833.000000	0.001522
3832.750000	0.001545
3832.500000	0.001576
3832.250000	0.001606
3832.000000	0.001625
3831.750000	0.001622
3831.500000	0.001595
3831.250000	0.001548
3831.000000	0.001492

3830.750000	0.001437
3830.500000	0.001391
3830.250000	0.001361
3830.000000	0.001348
3829.750000	0.001353
3829.500000	0.001375
3829.250000	0.001412
3829.000000	0.001458
3828.750000	0.001510
3828.500000	0.001562
3828.250000	0.001612
3828.000000	0.001655
[truncated: 364,413 more chars]
